# A role for small RNA in regulating innate immunity during plant growth

**DOI:** 10.1371/journal.ppat.1006756

**Published:** 2018-01-02

**Authors:** Yingtian Deng, Jubin Wang, Jeffrey Tung, Dan Liu, Yingjia Zhou, Shuang He, Yunlian Du, Barbara Baker, Feng Li

**Affiliations:** 1 Key Laboratory of Horticultural Plant Biology, Ministry of Education, College of Horticulture and Forestry Sciences, Huazhong Agricultural University, Wuhan, China; 2 Department of Plant and Microbial Biology, University of California, Berkeley, Berkeley, CA, United States of America; 3 Plant Gene expression Center, ARS-USDA, Albany, CA, United States of America; University of California, Davis Genome Center, UNITED STATES

## Abstract

Plant genomes encode large numbers of nucleotide-binding (NB) leucine-rich repeat (LRR) immune receptors (*NLR*) that mediate effector triggered immunity (ETI) and play key roles in protecting crops from diseases caused by devastating pathogens. Fitness costs are associated with plant *NLR* genes and regulation of *NLR* genes by micro(mi)RNAs and phased small interfering RNAs (phasiRNA) is proposed as a mechanism for reducing these fitness costs. However, whether *NLR* expression and *NLR*-mediated immunity are regulated during plant growth is unclear. We conducted genome-wide transcriptome analysis and showed that *NLR* expression gradually increased while expression of their regulatory small RNAs (sRNA) gradually decreased as plants matured, indicating that sRNAs could play a role in regulating *NLR* expression during plant growth. We further tested the role of miRNA in the growth regulation of *NLRs* using the tobacco mosaic virus (TMV) resistance gene *N*, which was targeted by miR6019 and miR6020. We showed that *N*-mediated resistance to TMV effectively restricted this virus to the infected leaves of 6-week old plants, whereas TMV infection was lethal in 1- and 3-week old seedlings due to virus-induced systemic necrosis. We further found that *N* transcript levels gradually increased while miR6019 levels gradually decreased during seedling maturation that occurs in the weeks after germination. Analyses of reporter genes in transgenic plants showed that growth regulation of *N* expression was post-transcriptionally mediated by *MIR6019/6020* whereas *MIR6019/6020* was regulated at the transcriptional level during plant growth. TMV infection of *MIR6019/6020* transgenic plants indicated a key role for miR6019-triggered phasiRNA production for regulation of *N*-mediated immunity. Together our results demonstrate a mechanistic role for miRNAs in regulating innate immunity during plant growth.

## Author summary

In plants, nucleotide-binding (NB) leucine-rich repeat (LRR) receptors (NLR) mediate pathogen-specific effector triggered immunity and are widely used in breeding to generate pathogen-resistant crops. However, dysregulation of *NLR* expression can inhibit plant growth and how *NLR* expression and function are regulated in different stages of plant growth is poorly understood. Using a high-throughput sequencing and bioinformatics approach, we found an overall increase in *NLR* expression, but expression of *NLR*-targeting sRNA during plant growth was decreased. We also used resistance to tobacco mosaic virus (TMV) mediated by the resistance gene *N* as a model system to study the biological significance of growth regulation of *NLR* by miRNAs. We found that *N*-mediated TMV immunity strengthened and *N* transcript levels increased during plant maturation. Using genetic analysis, we showed that up-regulation of *N* was due to transcriptional down-regulation of the *N-*targeting miR6019/6020 cluster during plant growth. We also showed that sRNA-mediated growth regulation of *N* expression and function was conserved between tobacco and tomato plants. This study therefore reveals a role for miRNAs in regulating innate immunity during plant growth.

## Introduction

Plant nucleotide-binding leucine-rich repeat receptors (NLR) recognize specific pathogen effectors and trigger effective defenses against invading pathogens that are usually accompanied by a hypersensitive response (HR) in infected tissues [1]. Plant genomes usually encode hundreds of NLRs that are divided into TIR-NB-LRR (TNL) and CC-NB-LRR (CNL) groups, which contain an N-terminal Toll-like and Interlukin-1 receptor domain (TIR) and coiled-coil domain, respectively [2]. In humans, Toll-like receptors (TLRs) are innate immune receptors that recognize a variety of ligands from viruses, bacteria, fungi and other types of pathogens [3]. Extensive medical research and clinical observations suggested that TLR-mediated immunity and *TLR* expression are regulated in a growth-stage specific manner [4]. However, whether plant NLR-mediated immunity responses to infection differ during plant growth is unknown.

Micro(mi)RNAs are 20- to 24-nucleotide (nt) long short RNAs that are processed from hairpin RNA precursors by Dicer-like (DCL) enzymes [5, 6]. They form an RNA induced silencing complex (RISC) with the endoribonuclease Argonaute (AGO) protein and guide AGO to cleave target mRNAs based on sequence complementarity [7, 8]. miRNAs play general roles in plant and animal development. For example, the conserved plant miR156 and animal miRNA let-7 control developmental phase changes in plants and animals, respectively [9–11]. Both plant and animal miRNAs can trigger mRNA degradation and translational inhibition in their targets [12], whereas some plant miRNAs have unique functions in triggering phased siRNA (phasiRNA) generation from the cleavage products of their targets [13]. These miRNA precursors usually have an asymmetric bulge in their hairpin structures and produce 22-nt mature miRNAs instead of the more typical 21-nt mature miRNAs [14, 15], which confer unique functionality to AGO1 to feed the 3' cleavage product into the RNA dependent RNA Polymerase 6 (RDR6)/DCL4 pathway for phasiRNA production. Plant *NLR*s are frequent targets of plant miRNAs, many of which are 22-nt in length and can trigger phasiRNA synthesis from *NLR* target transcripts [16–18]. The tobacco mosaic virus (TMV) resistance gene *N* is regulated by the miRNA cluster miR6019/6020 in tobacco plants and the 22-nt miR6019 cleaves the *N* transcript at its TIR coding region and triggers phasiRNA production in an *RDR6/DCL4*-dependent manner [17]. Viral and fungal pathogen infections have been reported to inhibit miRNA function and induce *NLR* expression, suggesting that miRNA-mediated *NLR* regulation can be modulated by pathogen infection [18, 19]. However, whether miRNA-mediated *NLR* regulation is modulated during plant growth is unclear. Here we showed that during growth plant *NLR* expression gradually increased, and this increase was accompanied by decreased accumulation of *NLR* regulatory sRNAs. Using an *N*-miR6019/6020*-*TMV trilateral system, we showed that *N*-mediated immunity strengthened as plants matured, which correlated well with increasing accumulation of *N* transcripts. Further analysis showed that transcriptional regulation of miR6019/6020 was involved in growth-regulated *N* expression and function. As *NLR*s represent rich natural resources for disease resistance, enhanced understanding of *NLR* regulation mechanisms will facilitate better uses of *NLR* in breeding programs. Our studies described here provide a mechanism for miRNAs in regulation of plant innate immunity during plant growth.

## Results

### Transcriptome analysis reveals that a majority of the *NLR* genes is regulated by sRNAs during plant growth

To test whether sRNAs play a role in the growth regulation of *NLRs*, we conducted genome wide mRNA and sRNA expression profiling using high-throughput sequencing of RNA samples prepared from tomato and tobacco plants at 1, 3 and 6 weeks after germination (WAG) (S1 Fig).

**Fig 1:**
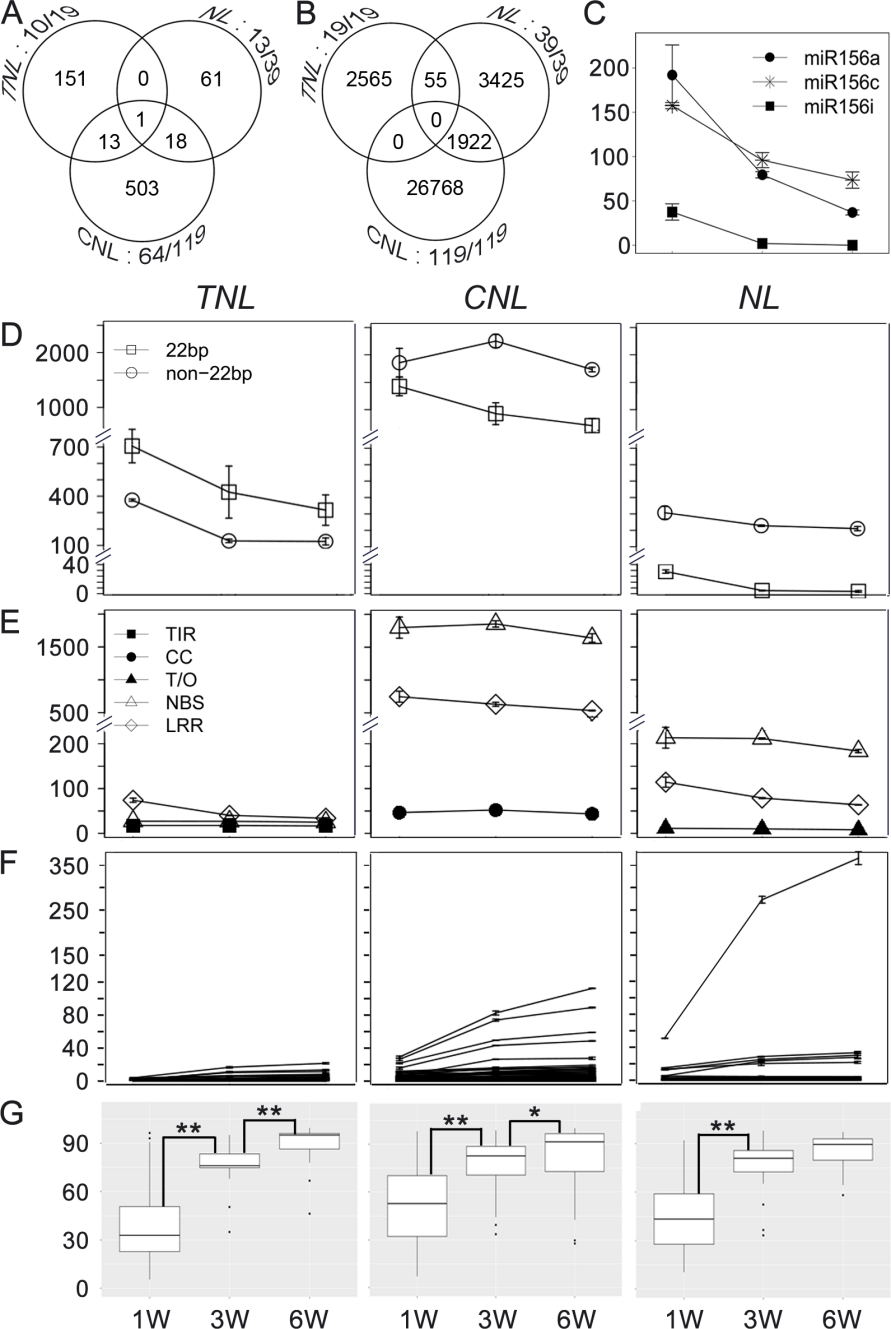
Regulation of the majority of *NLRs* by sRNAs in D51 tomato plants during growth. (A) Venn diagram for numbers of *NLR* silencers targeting different classes of *NLRs* in tomato. Three circles represent the number of silencers targeting *TNL*, *CNL* and *NL*, respectively, as indicated in each circle. Numbers next to each circle indicate the number of silencer targeted *NLR* genes out of the total numbers in each class. (B) Venn diagram for numbers of secondary siRNAs derived from different class of *NLRs* in tomato. Three circles represent secondary siRNAs derived from *TNL*, *CNL* and *NL*, respectively, as indicated in each circle. Numbers next to each circle indicate the number of *NLR* genes with secondary siRNAs out of the total number in each class. (C) Expression profile of the conserved miR156 members. (D) TNL (left), CNL (middle) and NL (right) silencer expression profile at 1-, 3- and 6 WAG stages. Open square, 22-nt silencer; open circle, non-22-nt silencer. Each line represents an individual silencer. (E) *TNL* (left), *CNL* (middle) and *NL* (right) secondary siRNA expression profile at 1, 3 and 6 WAG stages. Filled square, TIR of *TNL*; filled circle, CC of *CNL*; filled triangle, N-terminal region of *NL*; open triangle, NBS region of all *NLR*; open diamond, LRR region of all *NLR*. Each line represents an individual gene. (F) *TNL* (left), *CNL* (middle) and *NL* (right) gene expression profile at 1-, 3- and 6 WAG stages. Each line represents an individual gene. (G) The box plot of data in E. Asterisks indicate statistically significant differences between expression levels of *NLRs* at two time-points (*, 0.01<P<0.05; **, P<0.01). The statistical analysis was conducted using the R t.test method and plotted using the R ggplot2 package. Y axes are all in TPM units.

Before quantifying expression of *NLR* and its sRNA regulators (referred to as *NLR* silencer hereafter), a complete set of 624 and 177 *NLR* genes was extracted from tobacco and tomato genomes, respectively, and divided into *TNL*, *CNL* and *NL* (without N terminal TIR or CC domain) classes based on the N-terminal structure (S1 Data and S1 Table). Using these *NLR* cDNAs, small RNA databases, and degradome RNA databases, we identified 210 and 747 *NLR* silencers from tobacco and tomato genomes, respectively, which were predicted to cleave *NLR* genes. The predicted cleavages were supported by degradome RNAs mapped to the *NLR* transcripts (S2 Data and S2 Table). Among the 747 *NLR* silencers in tomato, 151, 503 and 61 targeted *TNL*, *CNL* and *NL*, respectively. Furthermore, 13 targeted both *TNL* and *CNL* whereas 18 targeted both *CNL* and *NL*. One *NLR* silencer targeted all three classes (Fig 1A). Ten out of 20 *TNL*, 64 out of 119 *CNL*, and 13 out of 39 *NL* were directly targeted by *NLR* silencers (Fig 1A). A similar situation was observed for tobacco, but fewer *NLR* silencers were identified and smaller portion of *NLRs* were directly targeted (S2A Fig). These data suggest that *NLR* silencers are specific to *TNL* and *CNL* classes and have a broad impact on *NLR* regulation. Since secondary siRNAs are processed from *NLR* transcripts and play an important role in *NLR* gene regulation [20], we also mapped sRNAs to different classes of *NLR* transcripts to assess *NLR* secondary siRNAs (*NLR* siRNAs). In tomato, over 20,000 20- to 22-nt siRNAs mapped to *CNL* transcripts, about 2,000 siRNAs mapped to *TNL* and about 5,000 siRNAs mapped to *NL* transcripts with 100% identity (Fig 1B). In tobacco, over 10,000 siRNAs perfectly matched with *TNL* transcripts, nearly 10,000 siRNAs matched with *NL* transcripts, and about 6,000 siRNAs matched with *CNL* transcripts (S2B Fig). As for the *NLR* silencers, secondary siRNAs were also specific to *TNL* and *CNL* classes and about 98% of tobacco *NLR* genes and 96% of tomato *NLR* genes spawned secondary siRNAs (Fig 1B and S2B Fig). These data indicate that secondary siRNAs have an even broader impact on *NLR* regulation than miRNAs.

The expression levels of each *NLR* silencer were determined in sRNA databases derived from different growth stages in both tobacco and tomato plants (S2 Table). For quality control, the expression profile for the conserved miR156 family members was determined and showed clearly decreasing accumulation in maturing tomato and tobacco plants (Fig 1C and S2C Fig), which was consistent with a previous report for *Arabidopsis* [21]. The overall expression trend of *NLR* silencers targeting each class of *NLR* genes was calculated by combining the total transcripts per million (TPM) of all *NLR* silencers. In tomato, the 22-nt and non-22-nt *TNL* silencer levels were around 700 and 400 TPM, respectively, at 1 WAG and gradually decreased by the 3 and 6 WAG stages (Fig 1D, left). The *CNL* and *NL* silencer levels ranged from 200 to 2,200 and 10 to 300 TPM, respectively, and showed a decreasing accumulation pattern during plant growth, except that non-22-nt *CNL* silencers accumulated to the highest level at 3 WAG (Fig 1D, middle and right). In tobacco, there were around 500 and 50 TPM for 22-nt and non-22-nt *TNL* silencers, respectively, in 1 WAG plants, and these silencers showed a general decreasing pattern, except for a slight increase in 22-nt silencers at 3 WAG (S2D Fig, left). The *CNL* and *NL* silencer counts ranged from 1,000 to over 10,000 and their levels first increased before then decreased (S2D Fig, middle and right).

The *NLR* siRNAs mapping to different regions of *NLR* transcripts were also quantified. In tomato, *CNL* genes spawned more abundant siRNAs than *TNL* and *NL* genes (Fig 1E, middle). The *CNL* and *NL* siRNAs were enriched in the NBS coding region whereas *TNL* siRNAs were enriched in the LRR region (Fig 1E). All siRNA levels showed a decreasing pattern during plant growth (Fig 1E). In tobacco, *TNL* genes spawned more abundant siRNAs compared to *CNL* and *NL* genes (S2E Fig). Among the *TNL* siRNAs, those derived from the TIR coding region represented around 80% of the total and showed a decreasing accumulation pattern during plant growth (S2E Fig, left). In contrast, *CNL* and *NL* siRNAs were distributed among different regions at similar levels (S2E Fig, middle and right).

We further determined *NLR* gene expression levels using mRNA sequencing data. Tomato *TNL* genes were expressed with a dynamic range from 0 to 20 TPM. The dynamic range of tomato *CNL* transcripts was between 0 and 100 TPM. Most tomato *NL* transcripts were expressed at low levels ranging from 0 to about 50 TPM, except the level of one *RPW8-*like transcript ranged from 50 to 350 TPM, which was the highest level among all tomato *NLRs* (Fig 1F). The majority of the tomato *NLR* transcript levels showed a gradually increasing pattern during plant growth (Fig 1F and 1G). In tobacco, the *TNL*, *CNL* and most *NL* transcript levels were below 10 TPM and showed no trends (S2F Fig). Interestingly, six *RPW8-*like transcripts accumulated above 16 TPM at 6 WAG and showed a clear upregulation during growth, which was similar to that seen from tomato (S2F and S2G Fig, right panels).

Overall, high-throughput sequencing of sRNA and mRNA samples from different plant growth stages revealed that most *NLRs* in tomato and tobacco were regulated by sRNAs. Levels of *NLR* silencers generally decreased and their *NLR* target levels generally increased as plants matured. Our results thus indicate that sRNAs play an important role in regulating *NLR* innate immune receptors during plant growth.

### *N*-mediated immunity against TMV is regulated during plant growth

**Fig 2:**
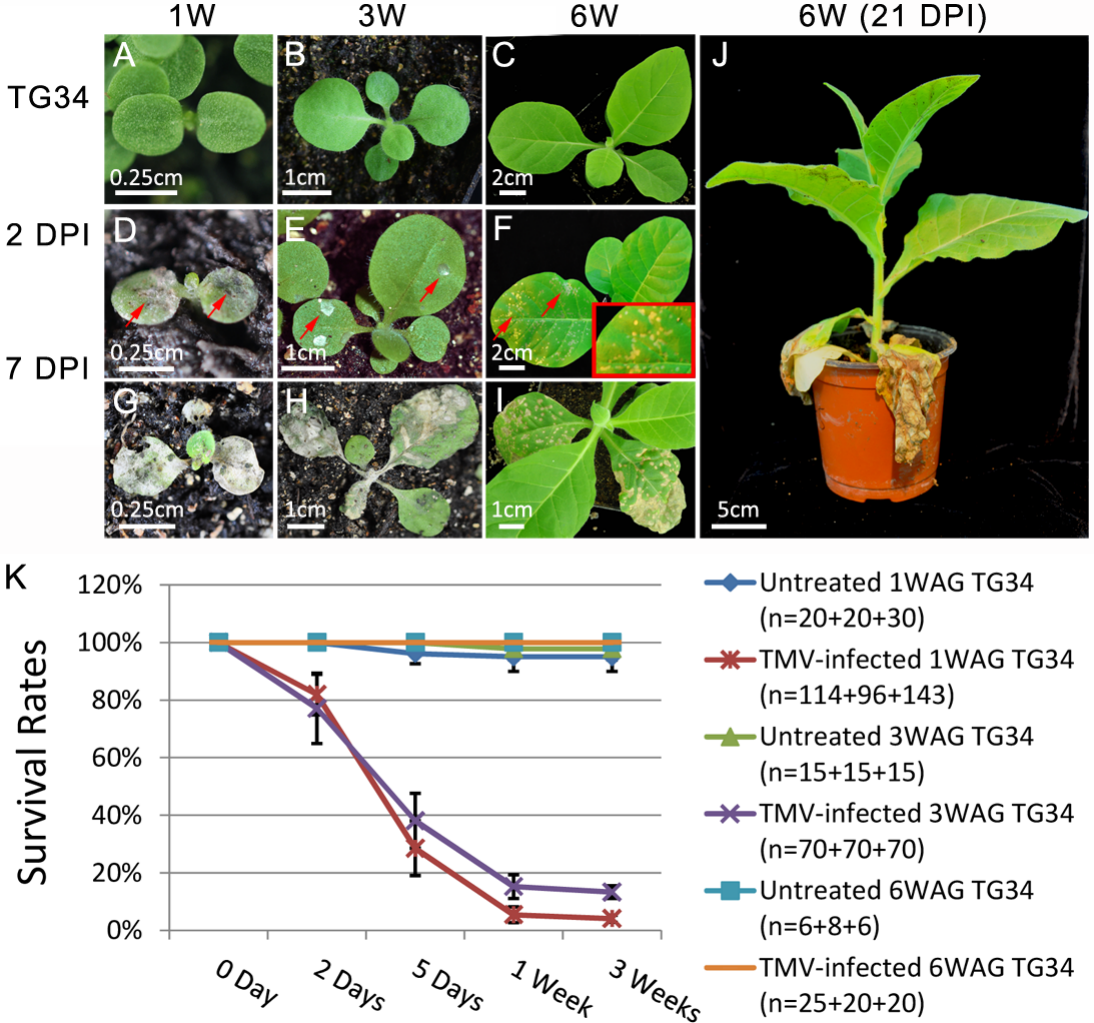
*N*-mediated resistance to TMV is regulated during TG34 seedling growth. (A-C) Untreated TG34 seedlings at 1, 3 and 6 WAG, which are labeled 1W, 3W and 6W, respectively in all of the figures. (D-F) TMV-infected 1-, 3- and 6 WAG seedlings at 2 DPI. Red arrows show HR on the infected leaves. The boxed area shows an enlargement of the HR area. (G-I) TMV-infected 1-, 3- and 6 WAG seedlings at 7 DPI. (J) TMV-infected 6 WAG plant at 21 DPI. The length of bars is indicated in each photo. (K) The average percentage rate of surviving TG34 plants at different times post TMV infection with the survival rates of untreated plants as controls. The plants were infected at 1, 3 and 6 WAG as indicated. Three independent experiments were performed, and the number (n) of test plants in each experiment are shown on the graph. The Y axes are in percentage units.

The *N*-TMV interaction has served as a classical model system for the study of plant immune responses to pathogens [22]. Since the *N* gene is regulated by tobacco miR6019/6020 [17], we chose the *N-*TMV-miR6019/6020 system to investigate the potential biological significance of *NLR* regulation during plant growth. For this, we challenged our well-characterized *N* transgenic TG34 tobacco plants at different growth stages with TMV [22]. We germinated TG34 seeds at consecutive time points, so that plants at 1, 3 and 6 WAG were inoculated with the TMV U1 strain at the same time (Fig 2A–2C). The results showed that hypersensitive response (HR) lesions appeared on inoculated leaves of all plants at 2 days post inoculation (DPI) (Fig 2D–2F, red arrows). However, at 7 DPI, plants that were inoculated at 1- and 3 WAG died due to systemic HR (Fig 2G and 2H), whereas plants inoculated at 6 WAG survived (Fig 2I). Furthermore, these plants showed no viral symptoms even at 21 DPI (Fig 2J). The experiment was repeated three times with similar results and average survival rates of TMV-infected and untreated plants clearly showed that TMV U1 infection was lethal to plants infected at 1 and 3 WAG, while plants infected at 6 WAG were fully resistant to TMV (Fig 2K). To rule out the possibility that the death of young TG34 seedlings was due to hypervirulence or rubbing damage, SR1 control plants without the *N* transgene were also inoculated with the TMV U1 strain at 1-, 3- and 6 WAG (S3 Fig, upper panel). Subsequent symptom observation showed that all SR1 tobacco plants survived at 21 DPI, although plants infected at 1- and 3 WAG showed more severe deformation in terms of leaf morphology (S3 Fig, lower panel). These results indicate that *N*-mediated resistance against TMV in tobacco is regulated during growth and the resistance response strengthens as plants mature.

### *N* gene expression increases as plants mature

**Fig 3:**
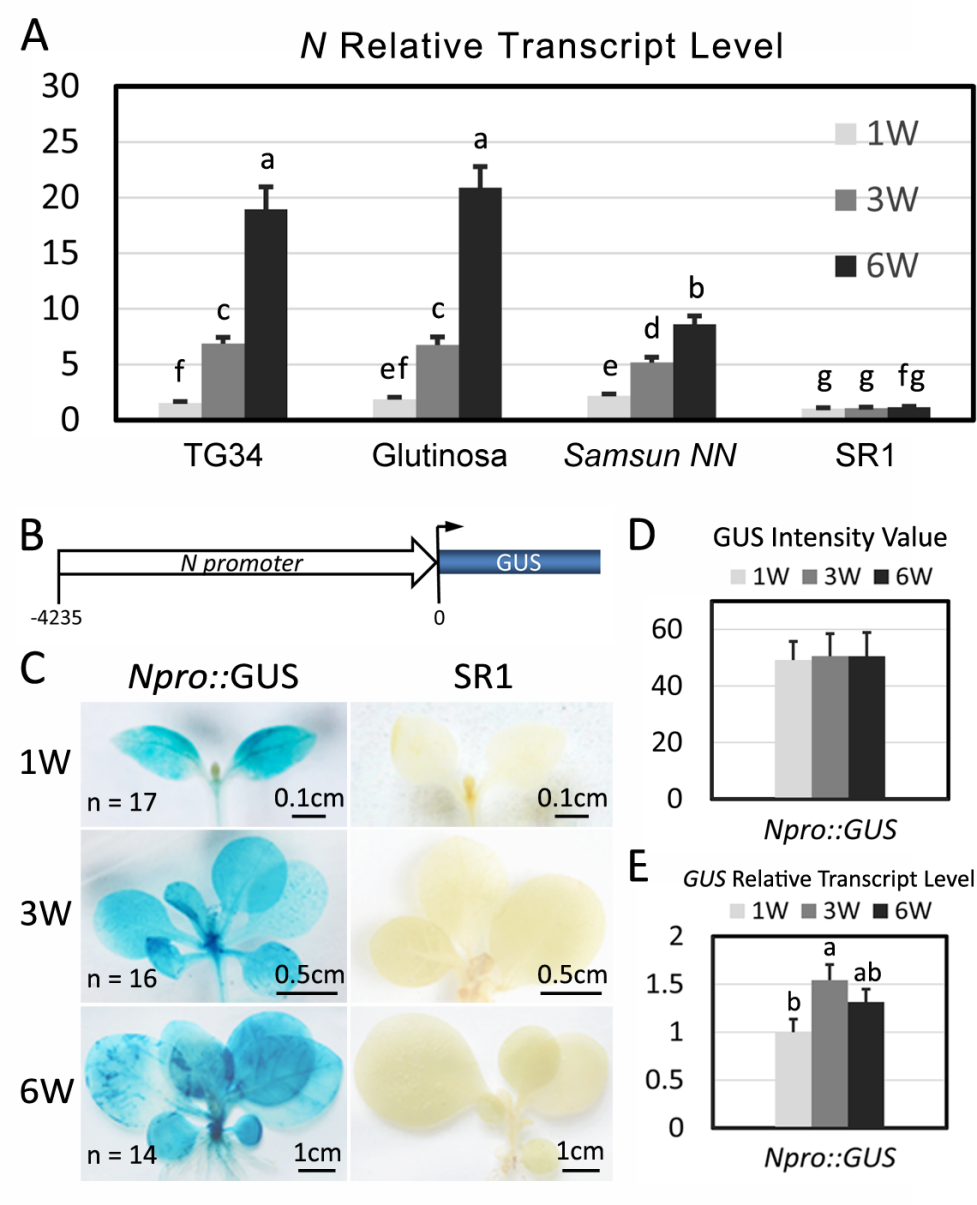
*N* expression increases as seedlings mature. (A) Relative *N* transcript levels at 1-, 3- and 6 WAG were determined by quantitative real-time reverse transcription-PCR (RT-qPCR) in *Nicotiana glutinosa* (Glutinosa), *Samsun NN*, TG34 and SR1 plants. The *GAPDH* gene was used as the reference gene. (B) Map of the *N* promoter *Npro*::*GUS* construct showing the *N* gene promoter and GUS CDS. (C) GUS staining of *Npro*::*GUS* transgenic plants and SR1 controls at 1-, 3- and 6 WAG. The number (n) of stained plants and the length of each bar are indicated on the image. (D) The intensity of GUS staining in transgenic *Npro*::*GUS* seedlings at 1-, 3- and 6 WAG was measured by image analysis and the relative values of GUS staining intensity are plotted. (E) The relative levels of GUS mRNAs determined by qRT-PCR in *Npro*::*GUS* seedlings at 1-, 3-, and 6 WAG. The data are the means of three replicates with SD (standard deviation). Different letters indicate significant differences between the treatments according to One-Way ANOVA (analysis of variance) test (P < 0.05). Y axes all indicate relative fold differences.

To investigate the molecular mechanisms underlying regulation of *N*-mediated immunity during growth, we determined the expression of *N* by real-time reverse transcription-PCR (qRT-PCR) using *N-*specific primers (S3 Table). We performed this experiment in three different *N-*expressing *Nicotiana* species (*N*. *glutinosa*, *N*. *tabacum* TG34 and *N*. *tabacum* cultivar *Samsun NN*), as well as in the cultivar Petite Havana SR1 (SR1) tobacco lacking the *N* gene. *N*. *glutinosa* is a wild tobacco species from which *N* has been introgressed into cultivated tobacco [23]. *Samsun NN* expresses *N* from a large genomic region introgressed from *N*. *glutinosa*. TG34 is a transgenic line that expresses *N* genomic DNA isolated from *Samsun NN* [22]. The qRT-PCR experiment detected a similar pattern of *N* expression in all three *N-*expressing *Nicotiana* species, starting at low levels for 1 WAG and gradually increasing for 3 to 6 WAG (Fig 3A). As expected, the *N* transcript was largely undetectable in SR1 at all three time points (Fig 3A). These results show that *N* expression is subject to developmental regulation and its expression level increases as plants mature.

To further investigate mechanisms of *N* regulation during plant growth, we constructed a GUS reporter gene under the control of a 4 kilobase (kb) *N* promoter (*Npro*::GUS, Fig 3B) and transformed the construct into SR1 plants. The 4 kb *N* promoter was previously shown to be sufficient to direct *N* expression that conferred complete resistance to TMV in tobacco [22]. Reporter gene expression levels in *Npro*::GUS plants at 1-, 3- and 6 WAG were determined by GUS staining and qRT-PCR. GUS staining showed that the intensity of blue color resulting from GUS activity was similar among plants from the three stages as determined by visual observation (Fig 3C, left panel) and quantification of the average gray value (Fig 3D). In contrast, wild type control plants showed no staining (Fig 3C, right panel). The qRT-PCR results showed that the GUS mRNA levels increased by 0.5- and 0.2-fold in the 3- and 6 WAG stages respectively, relative to the 1 WAG stage, whereas *N* mRNA levels increased around 5- and 17-fold in TG34 tobacco plants at the corresponding stages (Fig 3E and 3A). These data suggest that *N* gene transcription does not increase significantly as plants mature and thus the increased level of *N* mRNA in older plant leaves could be due to a relief from miR6019-mediated post-transcriptional gene silencing.

### Expression of miR6019 decreases during plant growth

**Fig 4:**
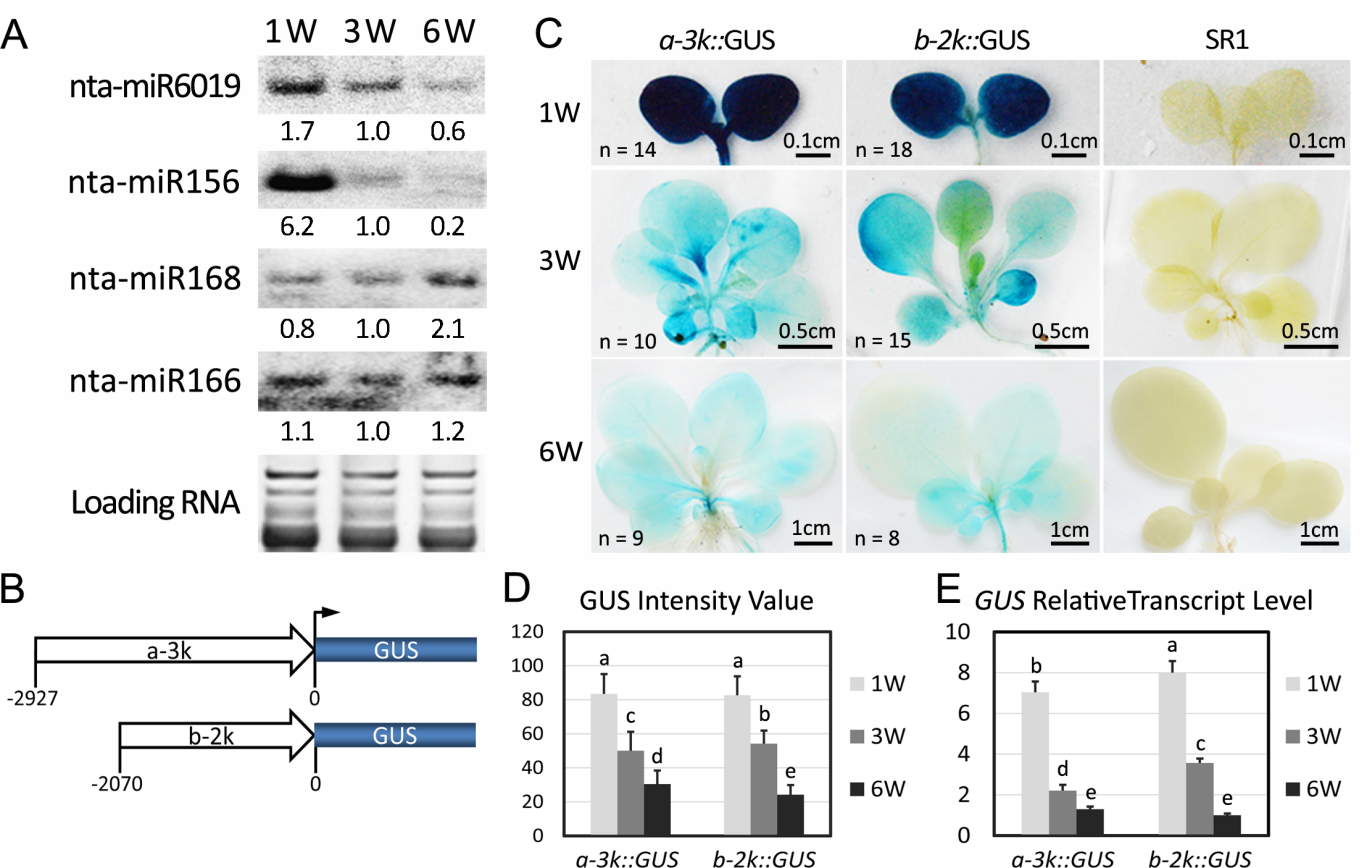
Expression of nta-miR6019/6020 decreases during tobacco seedling growth. (A) Northern blot hybridization of sRNAs isolated from TG34 plants at 1-, 3- and 6 WAG. Hybridization probes, miR6019, miR156, miR168 and miR166 are indicated to the left and probe sequences are listed in S3 Table. (B) Maps of the *nta-MIR6019/6020a* promoter reporter *a-3kpro*::*GUS* and *nta-MIR6019/6020b* promoter reporter *b-2kpro*::*GUS* constructs. Open arrows represent the a-3k or b-2k promoter, and blue boxes represent GUS CDS. (C) GUS staining of *a-3kpro*::*GUS* and *b-2kpro*::*GUS* transgenic plants and SR1 wild-type plants at 1-, 3- and 6 WAG. The number (n) of stained plants and the length of each bar are indicated on the image. (D) Intensity of GUS staining in *a-3kpro*::*GUS* and *b-2kpro*::*GUS* transgenic seedlings at 1, 3 and 6WAG. GUS activity was quantified using the average gray values that are plotted on the Y-axis. (E) Relative GUS mRNA levels determined by qRT-PCR in *a-3kpro*::*GUS* and *b-2kpro*::*GUS* transgenic seedlings at 1-, 3- and 6WAG. The data are the means of three replicates with SD. Different letters indicate significant differences between the treatments according to a one-way ANOVA test (P < 0.05). Y axes all indicate relative fold.

Our previous work showed that *N* and its *TNL* homologs were regulated by the miR6019/miR6020 cluster in tobacco plants [17]. Therefore, we tested whether miR6019/miR6020 was involved in regulation of *N* expression during growth by examining miR6019 levels in TG34 plant leaves at 1-, 3- and 6 WAG by northern blot analysis. The results showed that the relative accumulation levels of nta-miR6019 were 1.7, 1.0 and 0.6 at 1-, 3- and 6 WAG, respectively (Fig 4A). As expected, the nta-miR156 control showed decreasing relative expression levels of 6.2, 1.0 and 0.2 (Fig 4A), which was consistent with our sequencing results (S2C Fig) and the previous findings in *Arabidopsis* [21]. Meanwhile, the nta-miR168 levels were 0.8, 1.0 and 2.1 and nat-miR166 levels were 1.1, 1.0 and 1.2 (Fig 4A). These results indicate that miR6019/miR6020 expression is regulated and its expression decreases as plants mature.

To determine the mechanism by which the MIR6019/6020 cluster is regulated, we next attempted to identify the promoter region of *nta-MIR6019/6020a* and *nta-MIR6019/6020b* in tobacco [17]. A panel of *nta-MIR6019/6020* genomic clones containing exon 1 and different lengths of upstream sequences were cloned (S4A Fig) and transiently expressed in *N*. *benthamiana*. Northern blot analysis showed that miR6019 driven by the 3 kb *nta-MIR6019/6020a* (*a-3k*) and the 2 kb *nta-MIR6019/6020b* (*b-2k*) promoter showed higher expression levels (S4B Fig). Hence, we generated transgenic SR1 tobacco expressing GUS driven by these promoters (Fig 4B). GUS staining assays performed on 1-, 3- and 6 WAG seedlings showed that the GUS expression was very high at 1 WAG and the expression gradually decreased at 3- and 6 WAG as determined by visual observation (Fig 4C, left and middle panels) and quantification of the average gray value (Fig 4D). In agreement with the decreased GUS activity, qRT-PCR analysis showed decreased GUS mRNA levels in both GUS reporter transgenic plants (Fig 4E). These results indicate that *MIR6019/6020* transcription decreases as plants mature. The observed opposite expression pattern between miR6019 and the *N* gene suggest that miR6019/miR6020 could play a role in regulating *N* expression and *N*-mediated innate immunity during plant growth.

### Regulation of *N* during plant growth is controlled by nta-miR6019/6020

**Fig 5:**
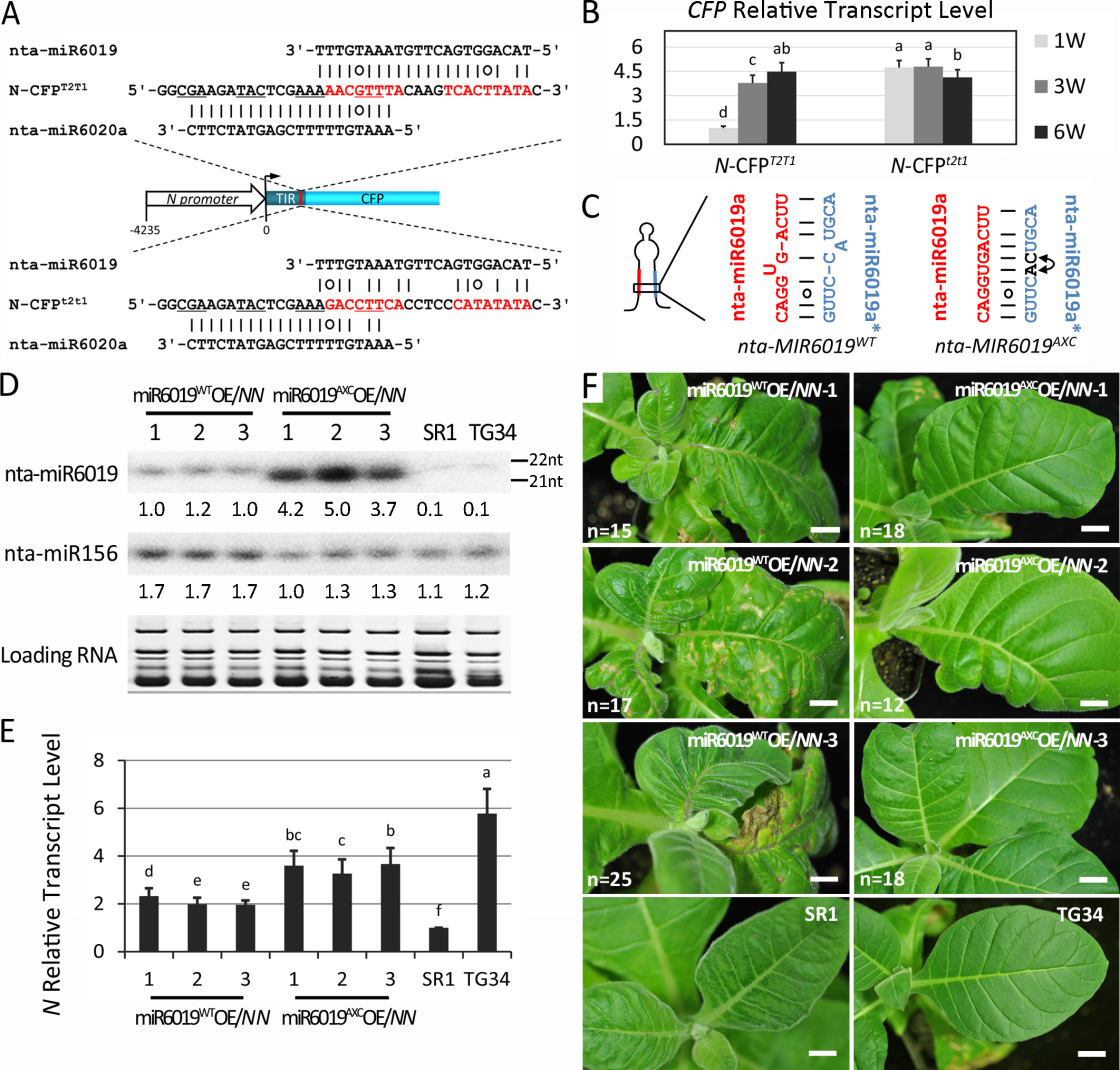
nta-miR6019/6020 regulates *N* gene expression and function during seedling growth. (A) Maps of *N-CFP*^*T2T1*^ and *N-CFP*^*t2t1*^ constructs. The sequences of wild type and mutated miR6019/miR6020 binding sites are shown above and below the map. (B) Relative CFP transcript levels measured by RT-qPCR at 1-, 3- and 6 WAG in *N-CFP*^*T2T1*^ and *N-CFP*^*t2t1*^ transgenic plants. The *GAPDH* gene was used as the reference gene. CFP levels in 1 WAG *N-CFP*^*T2T1*^ is considered 1. (C) Secondary structures of wild type and AXC mutants of nta-MIR6019. Mature miRNA and miRNA star sequences are highlighted in red. (D) Northern blot hybridization of sRNAs isolated from *nta-MIR6019*^*WT*^*/NN* and *nta-MIR6019*^*AXC*^*/NN* transgenic plants and both TG34 and SR1 plants at 6 WAG. Small RNA sizes are indicated to the right. (E) Relative *N* transcript levels in *nta-MIR6019*^*WT*^*/NN* and *nta-MIR6019*^*AXC*^*/NN* plants, and both TG34 and SR1 seedlings at 6 WAG. The *GAPDH* gene was used as the reference gene. The lowest *N* transcript level in *N*-containing plants (*nta-MIR6019*^*WT*^*/NN-3*) is considered as 1. (F) *nta-MIR6019*^*WT*^*/NN* and *nta-MIR6019*^*AXC*^*/NN* transgenic plants and both TG34 and SR1 plants inoculated with TMV at 6 WAG. The total number (n) of test plants is indicated on the image. Bars = 1cm. The data are the means of three replicates with SD. Different letters indicate significant differences between the treatments according to one-way ANOVA test (P < 0.05). Y axes indicate relative fold change.

To determine if the upregulation of *N* gene expression during plant growth is due to downregulation of nta-miR6019/6020 expression, we analyzed the two transgenic reporter lines *N-CFP*^*T2T1*^ and *N-CFP*^*t2t1*^ that we described previously [17]. Both lines express Cyan Fluorescent Protein (CFP) under the control of the same *N* gene promoter and 3' regulatory sequences. However, *N-CFP*^*T2T1*^ has the wild type binding sites for miR6019/6020 whereas *N-CFP*^*t2t1*^ has mutated binding sites (Fig 5A). RNA samples were prepared from 1-, 3- and 6 WAG plants from *N-CFP*^*T2T1*^ and *N-CFP*^*t2t1*^ and the *CFP* transcript levels were determined by qRT-PCR. The relative *CFP* levels of 1, 3.5 and 4.5 in *N-CFP*^*T2T1*^ plants at 1-, 3- and 6 WAG, respectively (Fig 5B, left), demonstrated a gradual increase in expression during growth. This result is similar to the pattern of *N* gene expression (Fig 3A). In contrast, the relative *CFP* transcripts levels in *N-CFP*^*t2t1*^ plants did not change significantly (Fig 5B, right). However, miR6019 accumulation gradually decreased in both *N-CFP*^*T2T1*^ and *N-CFP*^*t2t1*^ plants (S4C and S4D Fig, top panel). These results further support that miR6019/6020 regulate *N* expression during plant growth.

To further investigate whether the decreased miR6019 levels seen during plant growth contribute to an increased level of *N*-mediated immunity, we generated transgenic SR1 plants that overexpressed wild type and the AXC mutant of the *MIR6019/6020* cluster, which produce wild type 22-nt and mutated 21-nt miR6019, respectively, with wild type miR6020 (Fig 5C) [17], designated *miR6019*^*WT*^*OE* and *miR6019*^*AXC*^*OE*. The selected lines were crossed to TG34 expressing *N* and F2 progeny with homozygous *N* loci were selected (miR6019^WT^OE/*NN* and miR6019^AXC^OE/*NN*). The sRNA northern blot analyses confirmed that the 22- and 21-nt miR6019 accumulated to 10-fold greater levels in miR6019^WT^OE/*NN* and to around 40-fold greater levels in miR6019^AXC^OE/*NN* plants compared to the miR6019 level in SR1 and TG34 plants (Fig 5D). Notably, miR6019^WT^ or miR6019^AXC^ overexpression did not affect the growth phenotype of tobacco plants (S5 Fig). We next analyzed the *N* transcript level in different miR6019^WT^OE/*NN* and miR6019^AXC^OE/*NN* lines at 6 WAG. As expected, the *N* transcript level in miR6019^WT^OE/*NN* was reduced to about 36% of the *N* transcript level in TG34 (Fig 5E), while miR6019^AXC^OE/*NN* was reduced to about 61% of its level in TG34 plants (Fig 5E). It is interesting to note that even though the level of 21-nt miR6019 in miR6019^AXC^OE is much higher than that of the 22-nt miR6019 in miR6019^WT^OE, downregulation of *N* expression by miR6019^AXC^ was not as efficient as that by miR6019^WT^. Since the 21-nt miR6019^AXC^ cannot trigger phasiRNA production as miR6019^WT^ does, these results suggest that nta-miR6019-triggered phasiRNA production significantly potentiate miR6019-mediated repression of *N* expression.

To determine if nta-miR6019 overexpression affects *N*-mediated resistance to TMV, we infected miR6019^WT^OE/*NN* and miR6019^AXC^OE/*NN* TG34 and SR1 plants at 6 WAG with TMV U1. The *miR6019*^*WT*^*OE/NN* plants displayed strong systemic HR symptoms, whereas the *miR6019*^*AXC*^*OE/NN* plants showed only mild wrinkling symptoms in systemic leaves (Fig 5F). These results provide further evidence to support a role for both miR6019 and miR6019-triggered *N* phasiRNAs in the negative regulation of *N-*mediated resistance to TMV.

### Growth regulation of *N*-mediated TMV immunity is conserved in tomato

**Fig 6:**
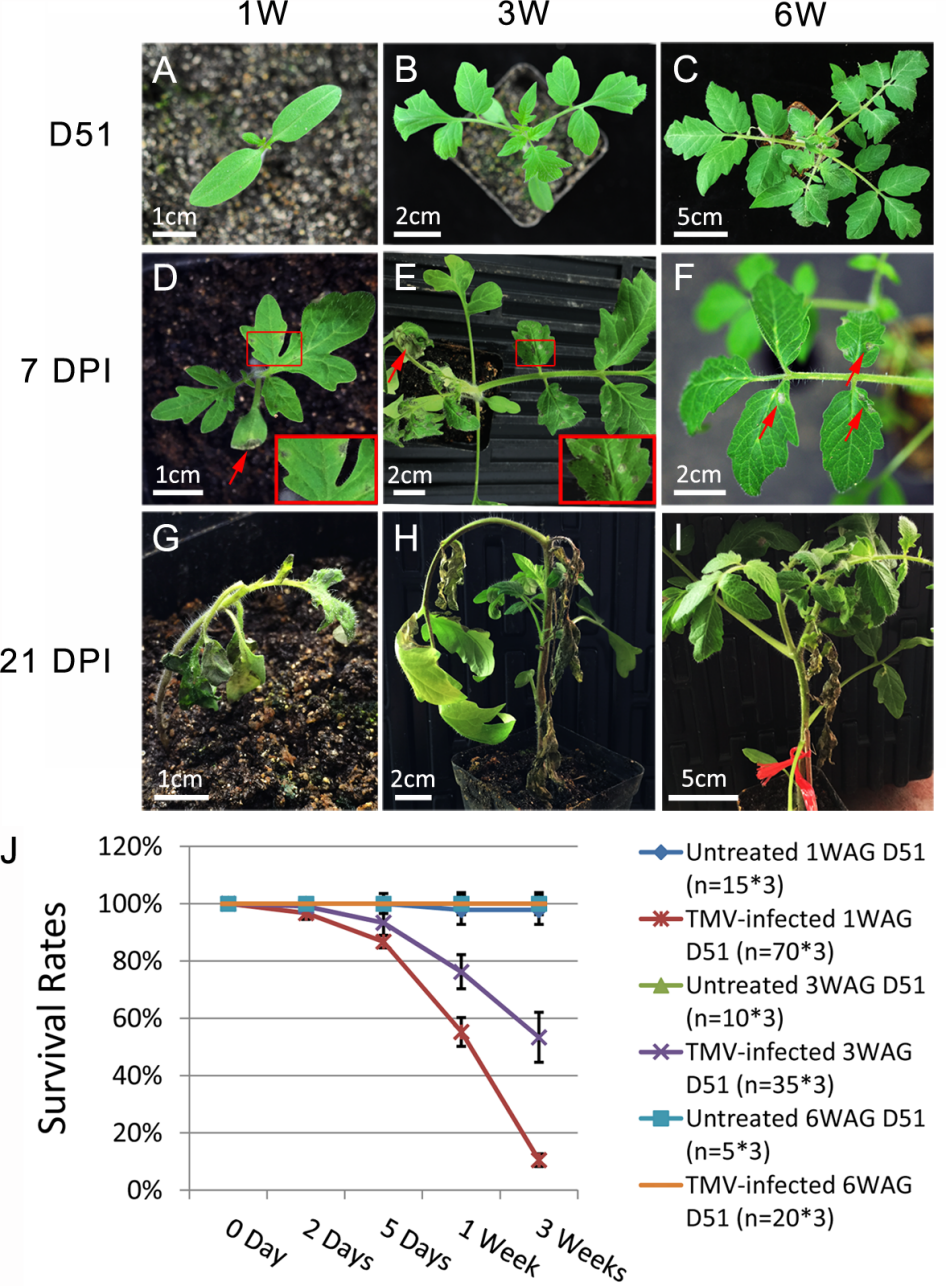
Regulation of *N*-gene-mediated resistance to TMV during D51 tomato growth. (A-C) Untreated plants at 1-, 3- and 6 WAG stages. (D-F) Plants inoculated with TMV at 1-, 3- and 6 WAG stages and photographed at 7 DPI. Arrows show the HR on the inoculated leaves. The boxed area represents enlargement of the SHR area in the systemic leaves. (G-I) Plants inoculated with TMV at 1-, 3- and 6 WAG and photographed at 21 DPI. The length of bars is labeled on each photo. (J) The average percentage rate of surviving D51 plants at different time post TMV infection with the survival rates of untreated plants as controls. The plants were infected at 1-, 3- and 6 WAG as indicated. Three independent replicates were performed. The total number (n) of plants is shown on the line chart. Y axes are in percentage units.

We extended our study on growth regulation of *N-*mediated TMV immunity to include our *N-* expressing transgenic D51 and control VF36 tomato lines [24]. D51 tomato plants at 1-, 3- and 6 WAG were infected with TMV and HR was observed on inoculated leaves of all plants at 7 DPI (Fig 6A–6F). At 21 DPI, strong systemic HR was observed for plants infected at 1- and 3 WAG while complete resistance was observed for plants infected at 6 WAG (Fig 6G–6I). The tomato infection experiment was also repeated three times and the average survival rates for infected and control plants were calculated. The survival rates were about 10%, 50% and 100% at 21 DPI for plants infected with TMV at 1-, 3- and 6 WAG, respectively, which is consistent with increased immunity as the plant matured (Fig 6J). For VF36 plants without *N*, all plants survived at 21 DPI, although they showed TMV symptoms (S6 Fig). These results indicate that *N-*mediated immunity is also regulated during tomato growth.

**Fig 7:**
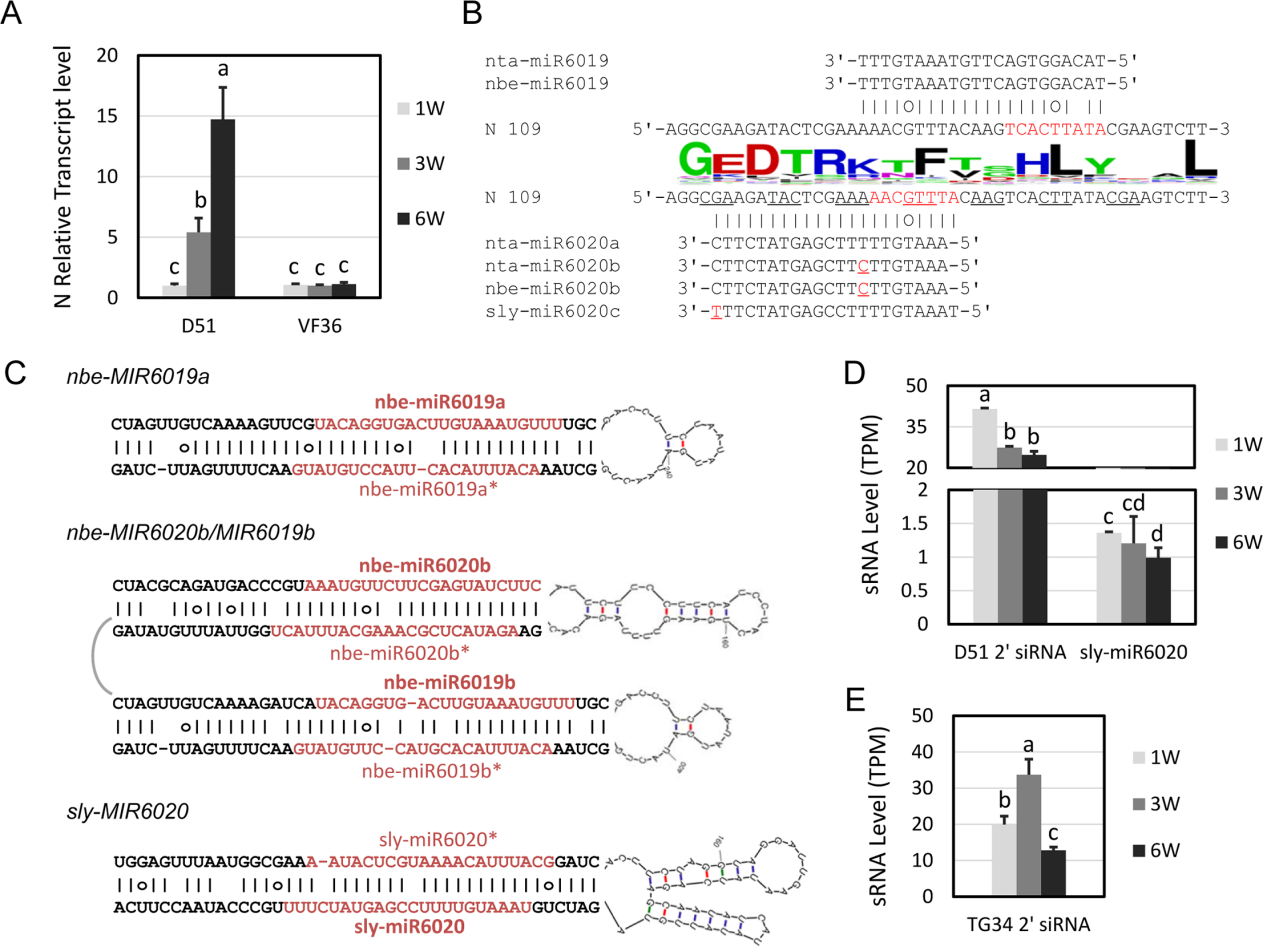
miR6019/6020 family is conserved in the Solanaceae plant family. (A) Relative *N* transcript levels in tomato D51 (blue) and VF36 (red) plants at 1-, 3- and 6 WAG stages as determined by qRT-PCR. (B) Mature miR6019 and miR6020 sequences from different *Solanaceae* plants are paired to the TIR coding sequences of the *N* gene. The sequence logo of the TIR amino acid sequences encoded by the miR6019/6020 target sequence is shown. *N* sequences highlighted in red represent the binding sites for the miR6019 and miR6020 seed sequences. Polymorphic nucleotides in mature miR6020 are highlighted in red. (C) Secondary structures of the *N*. *benthamiana* and *S*. *lycopersicum* miR6019/6020 precursors. Mature miRNA and miR star are highlighted in red. (D) Levels of tomato miR6020 and secondary siRNAs derived from the coding region of *N* at 1-, 3- and 6 WAG stages. (E) Levels of tobacco secondary siRNAs derived from the coding region of *N* at 1-, 3- and 6 WAG stages. The data are the means of three replicates with SD. Different letters indicate significant differences between the treatments according to one-way ANOVA test (P < 0.05). The Y axes in A indicate relative fold. The Y axes in D and E are in TPM units.

We further investigated the mechanism of *N* regulation in tomato. The qRT-PCR analysis determined that the relative *N-*transcript levels were 1, 5 and 15 in D51 tomato plants at 1-, 3-, and 6 WAG, respectively, while the levels remained low in all VF36 tomato plants (Fig 7A). These results show that *N-*mediated immunity and *N-*expression are also subject to developmental regulation in tomato. Then slicer detector analysis was performed. Using *N* transcript sequences and the solanaceae small RNA databases [25], we identified conserved miR6019/miR6020 in *N*. *benthamiana* and miR6020 in *S*. *lycopersicum* (Fig 7B and 7C). Unlike its counterpart in tobacco, the sly-miR6020 is 22-nt, which may trigger phasiRNA synthesis similar to that with miR6019. Small RNA sequencing analysis showed that sly-miR6020 accumulated at around 1 TPM at 1-, 3- and 6 WAG with a slight decreasing pattern (Fig 7D). However, the secondary siRNAs mapped to *N* transcript sequences accumulated at levels comparable to those in TG34 tobacco plants and showed a clear decreasing pattern (Fig 7D and 7E).

**Fig 8:**
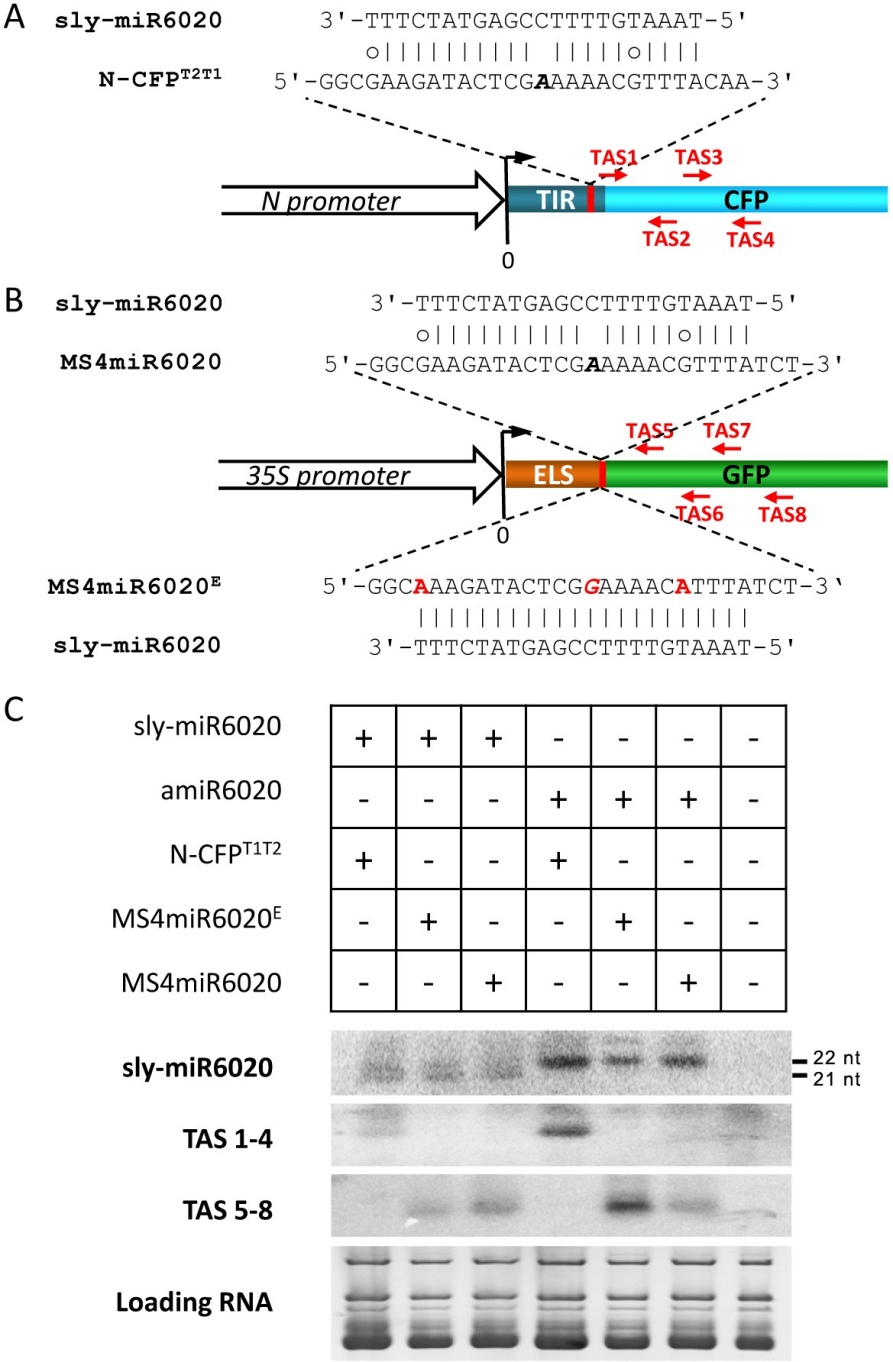
Sly-MIR6020 generates 22-nt miRNA and triggers phasiRNA production. (A) Map of sly-miR6020::*N-CFP*^*T2T1*^ sequence alignment and four CFP-phasiRNA (red arrows) down-stream of the miR6020 binding site. The sequence of sly-miR6020 binding site (red area) is shown above the *N-CFP*^*T2T1*^ construct map. Bases in italics indicate the cleavage site. (B) A map of sly-miR6020::*MS4miR6020* sequence alignment and four GFP-phasiRNA (red arrows) down-stream of the miR6020 binding site. The sequences of sly-miR6020 binding site (red area) in *MS4miR6020* and *MS4miR6020*^*E*^ are shown above and below the construct map respectively. Bases in italics indicate the cleavage site. The mutated bases are indicated in red. ELS, Endoplasmic reticulum (ER) localization signal. (C) Northern blot detection of phasiRNAs triggered by sly-miR6020. Probes are indicated to the left. EB staining of tRNA and rRNA serves as a loading control.

To test whether *sly-MIR6020* can produce 22-nt miR6020 and trigger phasiRNA production from *N* transcripts, we cloned the *sly-MIR6020* foldback structure with flanking sequence into a binary vector driven by a *35S* promoter (S3 Data). As a positive control, we cloned an artificial miRNA using *MIR171* as a backbone to express sly-miR6020 (*AMIR6020*, S3 Data). We used the *N-CFP*^*T2T1*^ and two miRNA sensor constructs, *MS4miR6020* and *MS4miR6020*^*E*^ (S3 Data, Fig 8A and 8B), as reporters for *N* transcripts. *N-CFP*^*T2T1*^ and *MS4miR6020* have wild type binding sites for sly-miR6020, which has a mismatch and G-U wobble pairs that may interfere with target cleavage and triggering phasiRNA production (Fig 8A and 8B). Meanwhile, *MS4miR6020*^*E*^ has a mutated binding site that completely matches the sly-miR6020 and served as positive control for sly-miR6020 triggered phasiRNA production (Fig 8C). The sly-miR6020 and reporters were co-expressed in *N*. *benthamiana* using Agrobacterium-mediated infiltration (Fig 8C). We successfully detected sly-miR6020 expressed from both *sly-MIR6020* and *AMIR6020* constructs (Fig 8C) and confirmed that *sly-MIR6020* generated both 22- and 21-nt miR6020, whereas *AMIR6020* generated only 22-nt miR6020 (Fig 8C, top panel lane1-3 vs. 4–6). Predicted phasiRNAs were detected in leaves co-expressing miR6020 and *MS4miR6020*^*E*^ as expected (Fig 8C, TAS5-8, lane 2 and 5). Both the *N-CFP*^*T2T1*^ and *MS4miR6020* transcript targeted by sly-miR6020 or amiR6020 also produced predicted phasiRNAs (Fig 8C, TAS1-4, lane1 and 4; TAS5-8, lane 3 and 6). These results indicate that tomato sly-miR6020 may cleave the *N* transcript and trigger phasiRNA synthesis and thus was functionally similar to nta-miR6019 in tobacco.

## Discussion

In plants, *NLR* genes function as double-edged swords. Although they can recognize pathogen effectors and mediate effective immune responses to protect plants from disease caused by pathogens, *NLR* expression in the absence of pathogen pressure is accompanied by a fitness cost [26] and causes intraspecific genetic incompatibility [27]. miRNA-mediated repression of *NLR* gene expression provides a simple and general mechanism for plants to solve this problem yet maintain a large repertoire of *NLR* receptors because *NLR-*targeting miRNA genes can be generated during *NLR* gene duplication and diversification processes [17, 28]. Consistent with this idea, many miRNAs were reported to be involved in *NLR* regulation [16–18]. Using bioinformatic analysis, we showed that about half and a quarter of tomato and tobacco *NLR* genes, respectively, were directly targeted by *NLR* silencers (Fig 1A and S2A Fig). Considering that we used strict criteria to select only *NLR* silencers that could cleave *NLR* transcripts and had dRNA reads supporting the predicted cleavage site, and that *NLR* silencers could also direct translational repression of *NLR* without *NLR* transcript cleavage, a larger portion of *NLR* genes are expected to be under direct regulation by *NLR* silencers. Indeed, our analysis showed that over 98% of tobacco *NLR* genes and over 96% of tomato *NLR* genes spawned secondary siRNA synthesis (Fig 1B and S2B Fig). These results support a broad impact for sRNA-mediated *NLR* regulation.

In our previous study, we showed that miR6019 could specifically cleave *N* and its homologous transcripts, triggered secondary siRNA synthesis from cleaved *N* transcripts and attenuated *N-*mediated resistance against TMV in transient expression assays [17]. In this study, we found that *N*-mediated resistance to TMV in transgenic tobacco and tomato was incomplete during the young seedling stage and reached full resistance as the plant matured (Fig 2 and Fig 6). This process is accompanied by an increased level of *N* gene transcripts (Fig 3 and Fig 7). These findings are in agreement with the dose-dependence of *CNL-*mediated resistance to TMV among independent transgenic lines [29]. It also suggests that *N-*mediated resistance can be modulated at the *N* transcript level during plant growth. GUS staining and qRT-PCR analysis of two reporter genes driven by the *N* gene promoter revealed only moderate changes in *N* transcription during plant growth. In contrast, northern blot analysis of miR6019 and analysis of MIR6019 promoter reporter activity in transgenic plants showed a decreasing pattern of MIR6019 transcription and miR6019 accumulation as plants matured, which prompted us to hypothesize that miR6019/6020 could play a role in regulating *N* expression during plant growth. Comparing the expression pattern of the *N* reporter gene with or without the miR6019/6020 binding sites provided further evidence that dynamic changes in miR6019 levels could be associated with growth-dependent regulation of *N* expression. We also showed that overexpression of the 22-nt but not the 21-nt miR6019 significantly impaired *N-*mediated resistance. Together with the previous finding that the 22-nt but not the 21-nt miR6019 triggers phasiRNA synthesis [17], these results point to a key role for phasiRNA in regulating *N* transcript levels and *N-*mediated innate immunity. Results from our *N-*TMV interaction experiments in D51 tomato are in agreement with this conclusion despite the possibility that phasiRNA could be triggered by the 22-nt miR6020 or derived from homologous *N-*like transcripts cleaved by other 22-nt miRNAs.

Although pathogen infection has been reported to interfere with miRNA-mediated regulation of *NLR* [18, 30, 31], whether this regulation is modulated during plant growth is unknown. Our results showed that *N* targeting miR6019/6020 is transcriptionally regulated during plant growth. We first demonstrated by high-throughput sequencing that accumulation of *NLR* silencers and *NLR* secondary siRNAs is downregulated during plant growth and accompanied by upregulation of their *NLR* targets (Fig 1 and S2 Fig). Our viral infection experiments on susceptible plants showed that younger plants are more sensitive to developmental interferences. As *NLR* gene overexpression promotes abnormal development [32, 33], high levels of sRNAs that target *NLR* expression during early developmental stages can maintain low levels of *NLR* expression and in turn minimize the possibility of developmental defects caused by auto-activation of *NLR-*mediated immunity.

Activation of immune responses often results in growth inhibition [32–35] and mechanisms underlying the trade-off between defense and growth are being actively investigated. Resource allocation theory was used to explain the growth-defense tradeoff in an ecological study of plant-pest interaction [36, 37]. Studies of hormone cross-talk in the context of pathogen-triggered defense and growth inhibition showed that many important plant hormones, including auxin, jasmonate (JA), gibberellins (GA), salicylic acid (SA), brassinosteroids (BR) and cytokinin (CK), were all involved in balancing growth and defense during pathogen attack [34, 38–40]. These studies were mostly done using a fixed time point during plant growth and thus could address how growth and defense were balanced at a certain time point. Our study on miR6019 regulation of *N-*mediated immunity during growth of tobacco and tomato plants provides a new angle to examine the trade-off between growth and defense and suggests that miRNAs can play a role in balancing growth and defense during plant growth by fine-tuning *NLR* expression. As reported previously, *NLR* genes are usually colocalized with various transposons in the plant genome and may be subjected to transcriptional silencing by transposon-derived siRNAs [41]. Moreover, NLR proteins are subject to negative regulation by SKP1-CULLIN1-F-box (SCF) complex-mediated stability control [42]. Thus, transcriptional and post-translational regulatory mechanisms may also contribute to regulation of growth and defense during plant maturation.

## Materials and methods

### Plant materials and growth conditions

Tomato line D51 (*Solanum lycopersicum*) and tobacco lines SR1 (*Nicotiana tabacum*), TG34 (*Nicotiana tabacum*), Samsun NN (*Nicotiana tabacum*), *Nicotiana glutinosa*, *Nicotiana benthamiana* were described previously [22, 24]. *N-CFP*^*T2T1*^ and *N-CFP*^*t2t1*^ transgenic plants were described recently [17]. The other transgenic plant materials were generated in this study. All plants were grown in a growth chamber at 22 ± 2°C with a 16-h-light/8-h-dark photoperiod.

### High-throughput sequencing of mRNAs, sRNAs and degradome RNAs from tobacco and tomato samples

Total RNA samples were prepared using the TRIzol reagent (Invitrogen) from the aerial parts of TG34 and D51 plants at 1- and 3 WAG and fully expanded leaves from plants at 6 WAG that were grown in soil. Paired-end mRNA libraries were prepared from total RNA samples using NEBNext Ultra RNA Library Prep Kit for Illumina (NEB, USA) according to the manufacture’s instruction and was sequenced on an Illumina HiSeqX TEN platform using the PE150 sequencing mode. For data analysis, tomato genome annotation ITAG2.4 and tobacco genome BX, K326 and TN90 were used [43, 44]. Fastq data were mapped to the genome using bowtie2 and the length distribution of the library insert was analyzed by Picard (CollectInsertSizeMetrics). mRNA expression values were determined using a perl script in Trinity, which calls bowtie and RSEM to do the mapping and calculation. mRNA expression was further normalized based on the TMM model using Trinity and its R module (edgeR). Reproducibility between biological replicates was estimated by the R program with plot methods (S1 Fig). sRNA sequencing library was prepared using NEBNext Multiplex Small RNA Library Prep Set for Illumina (NEB, USA) and was sequenced on an Illumina HiSeq2500 platform using SE50 sequencing mode. Fastq data was processed with an in-house perl script to remove adapter sequences, retrieve sRNA read sequences and read number. Reproducibility between biological replicates was also estimated by the R program with plot methods (S1 Fig). The degradome RNA library was prepared as described earlier [25, 45] and sequenced on an Illumina Genome Analyzer (QB3, UC Berkley) using SE50 mode.

### *NLR* gene and *NLR* slicer identification in tobacco and tomato

*NLR* gene was extracted from annotated tobacco and tomato genomes and combined with results from HMM search using NBS, LRR, TIR and CC domain consensus sequences. The number of tomato *NLR* genes identified is consistent with a previous report on the total number of *TNL-*, *CNL-* and *NL-type NLRs* and their full-length *NLR* genes [46]. To identify the *NLR* silencers, sRNAs of 20- to 24-nt and TPM greater than 1 were extracted from each tobacco and tomato sRNA databases (S4 Table). *NLR-*sRNA pairs were identified using the SlicerDetector Perl program. The degradome RNAs were mapped to the *NLR* transcripts using the dRNAmapper perl program. The *NLR* silencer-*NLR* pair with dRNA read support was obtained using the SmartCompare Perl program [25]. For *NLR* secondary siRNA detection, all small RNAs of 20- to 22-nt were aligned to *NLR* transcripts using bowtie with 0 mismatches and counted with an in-house perl program. Subsequent data integration and statistics were carried out using in-house perl and R scripts.

### Constructs and plant transformation

All constructs used in this study were described in S3 Data. To investigate their transcriptional regulation, the promoters of *MIR6019/6020a*, *MIR6019/6020b*, and *N* gene were amplified and inserted into the *pCambia1381Xb* vector (Cambia) upstream of the GUS reporter gene. After sequencing, all constructs were introduced into tobacco cv. SR1 by Agrobacterium-mediated leaf disc transformation. After screening on MS medium with 10 mg/L hygromycin, positive transformants were used for subsequent analysis. To analyze the promoter activity of three *nta-MIR6019/6020a* (a-1k, a-3k, a-5k) and two *nta-MIR6019/6020b* (b-2k, b-4k) fragments, each sequence fragment fused with Exon1 of *nta-MIR6019/6020a* or *b* were amplified and inserted into the empty pH7Lic14.0 vector. After sequencing, all constructs were tested in tobacco *N*. *benthamiana* by Agrobacterium-mediated infiltration. Primers used in the experiments are listed in S3 Table.

### TMV strains and inoculation procedures

TMV U1 strain was propagated in the TMV susceptible SR1 tobacco. Viral inoculations were performed as described [47]. For plants at the 1 WAG stage, the seedlings were very small. We generated wounds using needles on the cotyledons or leaves, and then covered them with wet gauze immersed in the viral sap. Inoculated plants were placed in the incubator at 22°C.

### RNA isolation, sRNA northern blot analysis and quantitative real-time PCR analysis

Total RNA was extracted from leaf tissue using the TRIzol reagent (Invitrogen) according to the manufacture’s protocol. sRNA northern blot analysis was performed as previously described [41]. For nta-miR6019 northern blot, we used the locked nucleic acid (LNA) modified oligonucleotide probe as previously described [48]. Probe sequences are listed in S3 Table. Quantitative real-time PCR was carried out using SYBR Green fluorescence and a Light Cycle 96 machine (Roche). The threshold cycle (Ct) value was automatically calculated by the Roche Light Cycle 96 1.1 system software and the ΔΔCt method was used to calculate the relative expression levels [49]. *GAPDH* was used to normalize the expression of genes in various RNA samples. Three independent biological replicates and three technical replicates of each sample were used for quantitative PCR analysis. Primers used in the experiments are listed in S3 Table.

### GUS staining and image analysis

All plants were sown on the 1/2MS medium. The histochemical GUS staining was performed as described [50]. After staining, samples were photographed using a NIKON D3300 digital camera. The value of GUS intensity was quantified using the PIL (Python Imaging Library) based on the average RGB values of each pixel in each image. Then the average grey value of each image was obtained using the "Color turn Gray" formula: Gray = R*0.299 + G*0.587 + B*0.114. For this method, the gray value is smaller for the darker color and the gray value of white color is assigned the maximum value of 255. The GUS intensity value was obtained with the formula: Intensity = 255—Gray value.

### Accession numbers

Raw reads of Illumina RNA-seq libraries generated in this study are available from the Sequence Read Archive (SRA) at NCBI (http://www.ncbi.nlm.nih.gov/sra/) under the accession number SRP125463. These data have also been deposited in the genome sequence archive in the BIG Data Center [51] under accession number CRA000618 that are publicly accessible at http://bigd.big.ac.cn/gsa.

## Supporting information

S1 Fig Correlation analysis between biological reduplicates of sRNA and mRNA high-throughput sequencing data sets. Correlation between sRNA-seq (A) and mRNA-seq (B) data sets from TG34 tobacco plants at 1, 3 and 6 WAG and sRNA-seq (C) and mRNA-seq (D) data sets from D51 tomato plants at 1, 3 and 6 WAG. Red points represent sRNA or mRNA expression levels and black diagonal lines represent the regression lines.
(TIF)

S2 Fig Majority of NLRs are regulated by sRNAs in TG34 tobacco plants during growth. (A) Venn diagram of the numbers of NLR silencers targeting different classes of NLRs in tobacco. The three circles represent the number of silencers targeting TNL, CNL and NL as indicated inside each circle. The numbers outside of each circle indicate the number of silencer targeted NLR genes out of the total number in each class. (B) Venn diagram of the numbers of secondary siRNAs derived from different class of NLRs in tobacco. Three circles represent numbers of secondary siRNAs derived from TNL, CNL and NL as indicated inside each circle. Numbers outside of each circle indicate the number of NLR genes with secondary siRNAs out of the total number in each class. (C) Expression profile of the conserved miR156 members. (D) TNL (left), CNL (middle) and NL (right) silencer expression profile at 1, 3 and 6 WAG stages. Open square, 22-bp; open circle, non-22-bp silencer. Each line represents an individual silencer. (E) TNL (left), CNL (middle) and NL (right) secondary siRNA expression profile at 1-, 3- and 6 WAG stages. Filled square, TIR of TNL; filled circle, CC of CNL; filled triangle, N-terminal region of NL; open triangle, NB region of all NLR; open diamond, LRR region of all NLR. Each line represents an individual gene. (F) TNL (left), CNL (middle) and NL (right) gene expression profile at 1-, 3- and 6 WAG stages. Each line represents an individual gene. The six NL gene expressed at high levels are RPW8-like genes. (G) The box plot of data in E. Asterisks indicate statistically significant differences between the expression levels of NLR genes in two time-points (*, 0.01<P<0.05; **, P<0.01). The statistical analysis was conducted using the R t.test method and plotted using the R ggplot2 package. Y axes are in TPM units.
(TIF)

S3 Fig TMV induced symptoms during SR1 plant growth. Untreated SR1 plants at 1-, 3- and 6 WAG are shown in the upper panel. The lower panel shows TMV-induced symptoms at 21 DPI on plants that are infected at 1, 3 and 6 WAG, respectively. The length of bars is labeled in each photo.
(TIF)

S4 Fig Characterization of a miR6019 promoter in a transient assay. (A) Maps of *nta-MIR6019/6020a* and *nta-MIR6019/6020b* promoter regions with different lengths of promoters used for optimal miR6019 expression in B. The red lines in Exon1 indicate the position of nta-miR6019/6020 pre-miRNA. (B) Northern blot hybridization of sRNAs isolated from *N*. *benthamiana* leaves infiltrated with indicated vectors shown in A. Probes are indicated to the left, EB staining of tRNA and rRNA serves as loading control. (C and D) Northern blot detection of sRNAs isolated from N-CFPT2T1 (C) and N-CFPt2t1 (D) transgenic plants at 1-, 3- and 6 WAG. Probes are indicated to the left, EB staining of tRNA and rRNA serves as loading control.
(TIF)

S5 Fig Photos of nta-miR6019 over-expressing transgenic plants during growth. The untreated miR6019^WT^OE/NN plants at 1-, 3- and 6 WAG are shown in the middle panel. The untreated miR6019^AXC^OE/NN plants at 1-, 3- and 6 WAG are shown in the lower panel. The length of the bars is labeled in each photo. The top panels of TG34 plants are the same in Fig 2A–2C. They are placed here to provide a direct comparison of the growth phenotype with miR6019OE transgenic plants.
(TIF)

S6 Fig Phenotype of TMV symptoms during growth of VF36. Untreated VF36 plants at 1-, 3- and 6 WAG are shown in the upper panel. The lower panel shows TMV-induced symptoms at 21 DPI on plants that are infected at 1, 3 and 6 WAG, respectively. The length of bars is indicated on each photo.
(TIF)

S1 Table Annotation and expression profile of tomato and tobacco *NLRs*. (XLSX)

S2 Table Annotation and expression profile of tomato and tobacco *NLR* silencers. (XLSX)

S3 Table Oligonucleotides used in this study. (DOCX)

S4 Table List of sequencing databases used in this study. (XLSX)

S1 Data Tomato and tobacco *NLR* cDNA sequences in fasta format. (DOCX)

S2 Data Alignments between tomato and tobacco *NLRs* and their sRNA silencers. (DOCX)

S3 Data Information on vectors used in this study. (DOCX)
