# Supplementary figures and images for: A role for small RNA in regulating innate immunity during plant growth
Source: PLoS Pathog. 2018 Jan 2;14(1):e1006756. doi: 10.1371/journal.ppat.1006756 (PMC5766230; doi:10.1371/journal.ppat.1006756)

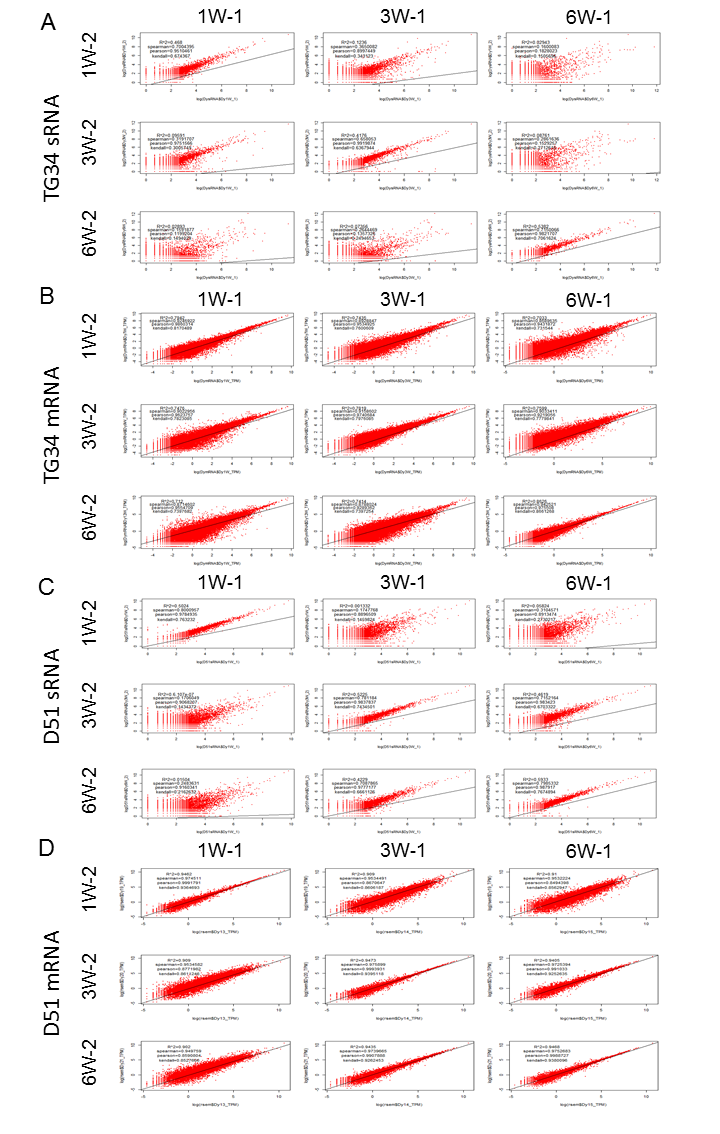

Supplement: S1 Fig — Correlation between sRNA-seq (A) and mRNA-seq (B) data sets from TG34 tobacco plants at 1, 3 and 6 WAG and sRNA-seq (C) and mRNA-seq (D) data sets from D51 tomato plants at 1, 3 and 6 WAG. Red points represent sRNA or mRNA expression levels and black diagonal lines represent the regression lines. (TIF) [file ppat.1006756.s001.tif]

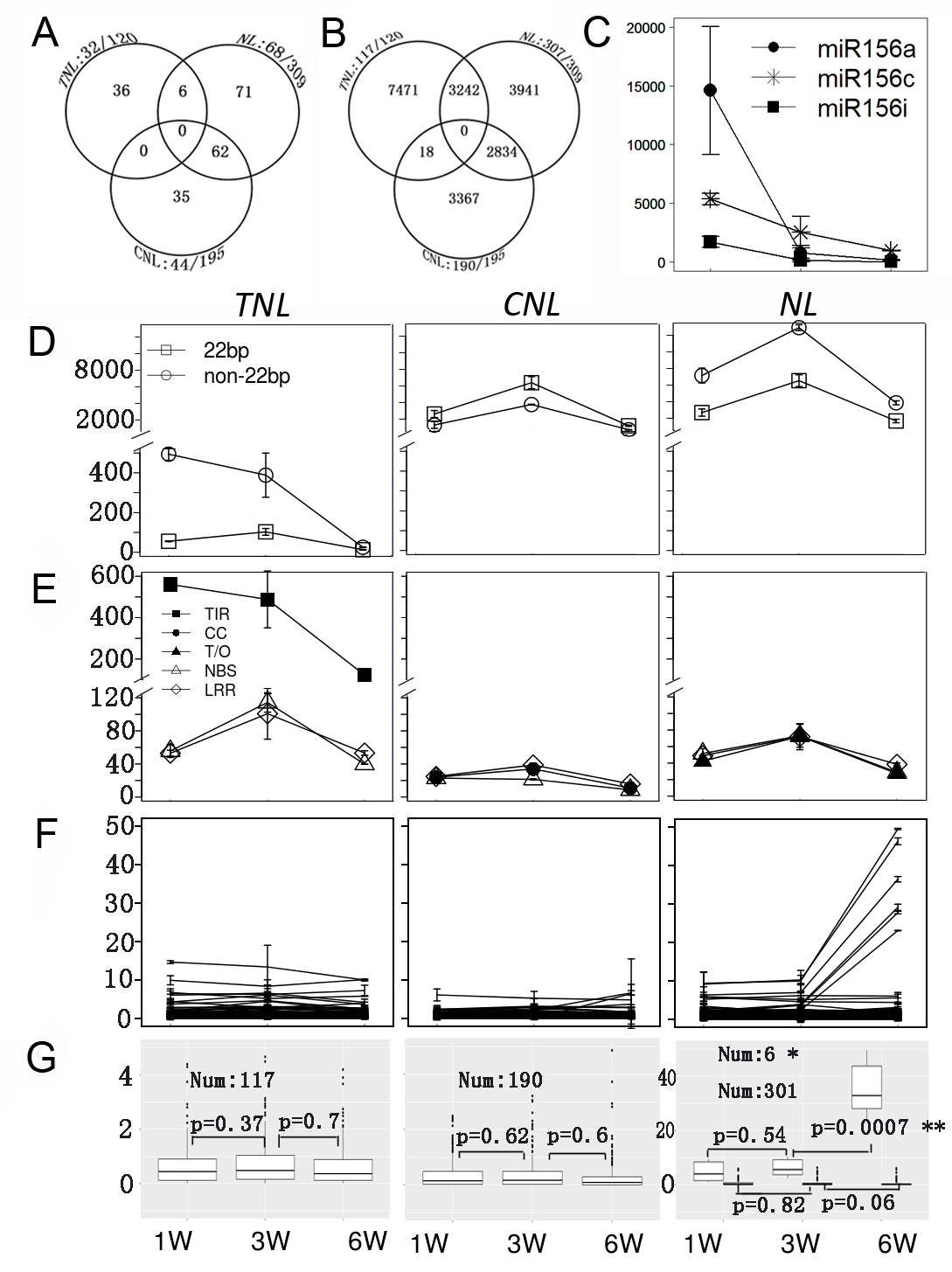

Supplement: S2 Fig — (A) Venn diagram of the numbers of NLR silencers targeting different classes of NLRs in tobacco. The three circles represent the number of silencers targeting TNL, CNL and NL as indicated inside each circle. The numbers outside of each circle indicate the number of silencer targeted NLR genes out of the total number in each class. (B) Venn diagram of the numbers of secondary siRNAs derived from different class of NLRs in tobacco. Three circles represent numbers of secondary siRNAs derived from TNL, CNL and NL as indicated inside each circle. Numbers outside of each circle indicate the number of NLR genes with secondary siRNAs out of the total number in each class. (C) Expression profile of the conserved miR156 members. (D) TNL (left), CNL (middle) and NL (right) silencer expression profile at 1, 3 and 6 WAG stages. Open square, 22-bp; open circle, non-22-bp silencer. Each line represents an individual silencer. (E) TNL (left), CNL (middle) and NL (right) secondary siRNA expression profile at 1-, 3- and 6 WAG stages. Filled square, TIR of TNL; filled circle, CC of CNL; filled triangle, N-terminal region of NL; open triangle, NB region of all NLR; open diamond, LRR region of all NLR. Each line represents an individual gene. (F) TNL (left), CNL (middle) and NL (right) gene expression profile at 1-, 3- and 6 WAG stages. Each line represents an individual gene. The six NL gene expressed at high levels are RPW8-like genes. (G) The box plot of data in E. Asterisks indicate statistically significant differences between the expression levels of NLR genes in two time-points (*, 0.01<P<0.05; **, P<0.01). The statistical analysis was conducted using the R t.test method and plotted using the R ggplot2 package. Y axes are in TPM units. (TIF) [file ppat.1006756.s002.tif]

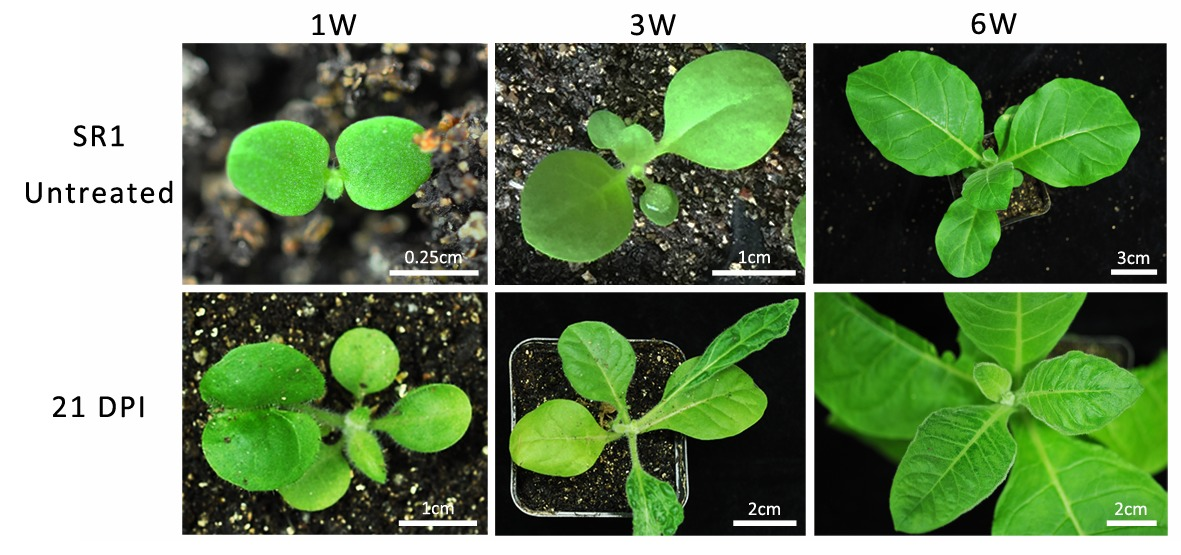

Supplement: S3 Fig — Untreated SR1 plants at 1-, 3- and 6 WAG are shown in the upper panel. The lower panel shows TMV-induced symptoms at 21 DPI on plants that are infected at 1, 3 and 6 WAG, respectively. The length of bars is labeled in each photo. (TIF) [file ppat.1006756.s003.tif]

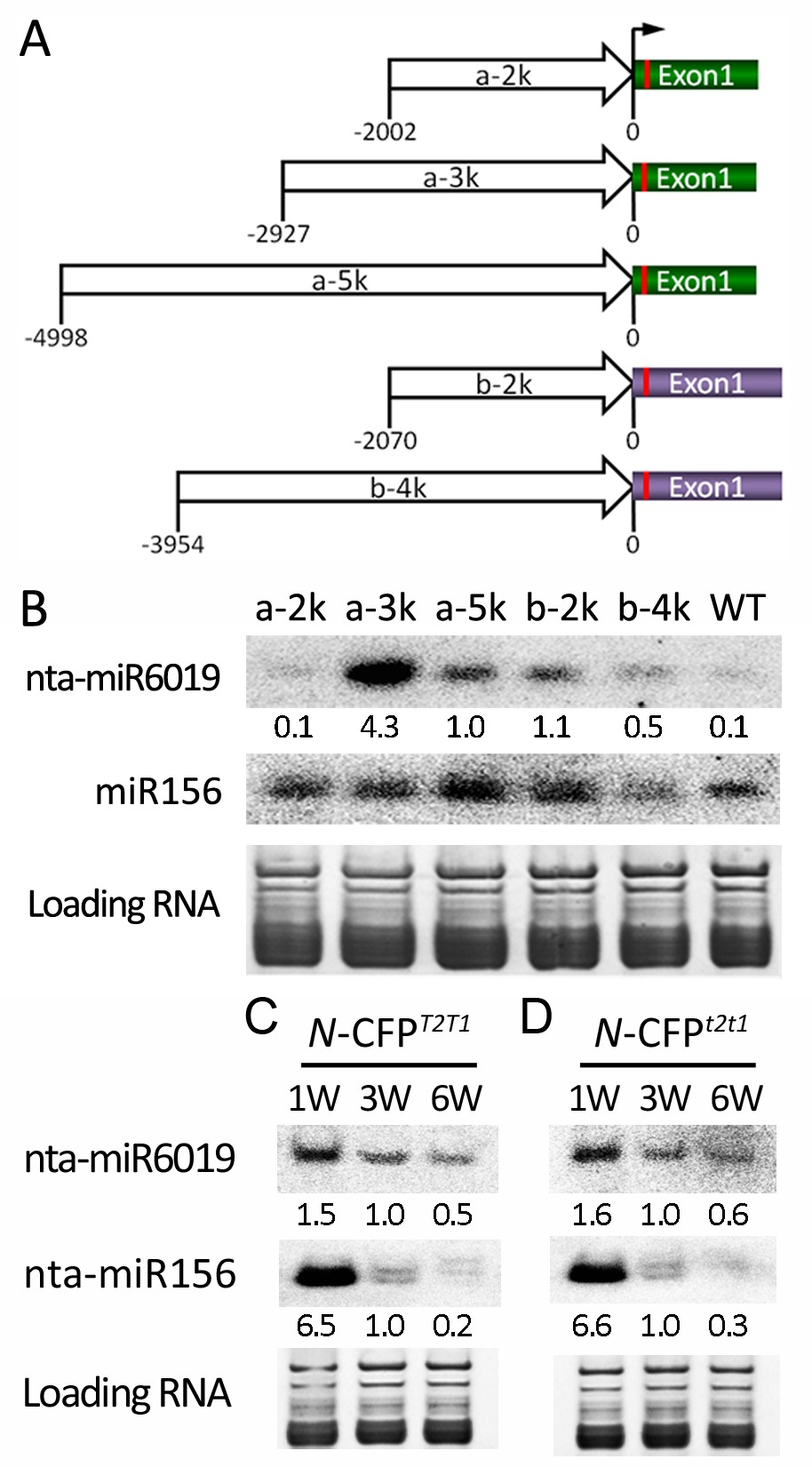

Supplement: S4 Fig — (A) Maps of nta-MIR6019/6020a and nta-MIR6019/6020b promoter regions with different lengths of promoters used for optimal miR6019 expression in B. The red lines in Exon1 indicate the position of nta-miR6019/6020 pre-miRNA. (B) Northern blot hybridization of sRNAs isolated from N. benthamiana leaves infiltrated with indicated vectors shown in A. Probes are indicated to the left, EB staining of tRNA and rRNA serves as loading control. (C and D) Northern blot detection of sRNAs isolated from N-CFPT2T1 (C) and N-CFPt2t1 (D) transgenic plants at 1-, 3- and 6 WAG. Probes are indicated to the left, EB staining of tRNA and rRNA serves as loading control. (TIF) [file ppat.1006756.s004.tif]

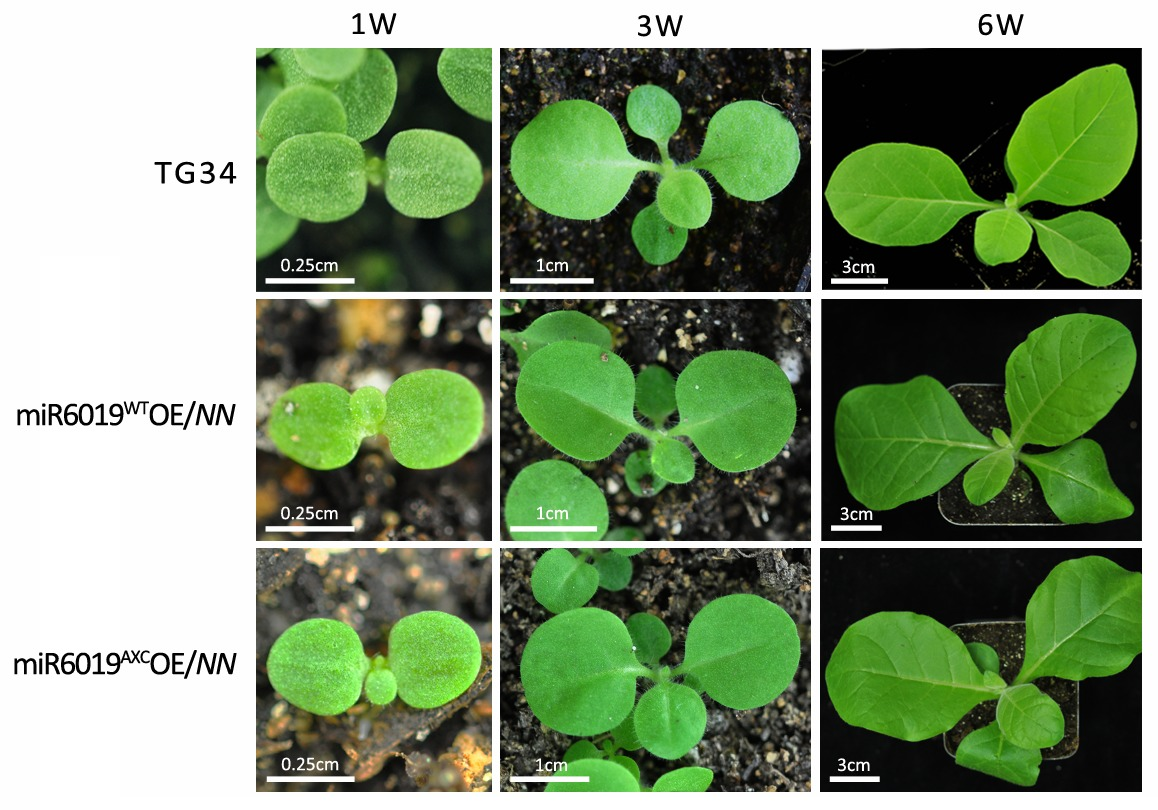

Supplement: S5 Fig — The untreated miR6019WTOE/NN plants at 1-, 3- and 6 WAG are shown in the middle panel. The untreated miR6019AXCOE/NN plants at 1-, 3- and 6 WAG are shown in the lower panel. The length of the bars is labeled in each photo. The top panels of TG34 plants are the same in Fig 2A–2C. They are placed here to provide a direct comparison of the growth phenotype with miR6019OE transgenic plants. (TIF) [file ppat.1006756.s005.tif]

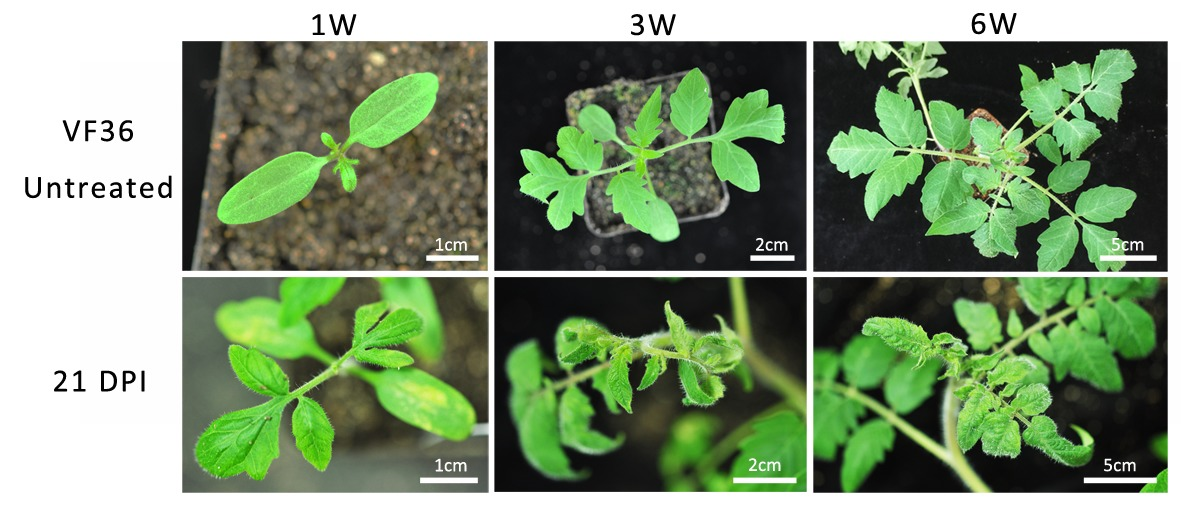

Supplement: S6 Fig — Untreated VF36 plants at 1-, 3- and 6 WAG are shown in the upper panel. The lower panel shows TMV-induced symptoms at 21 DPI on plants that are infected at 1, 3 and 6 WAG, respectively. The length of bars is indicated on each photo. (TIF) [file ppat.1006756.s006.tif]
