# Supplementary material for: A role for small RNA in regulating innate immunity during plant growth
Source: PLoS Pathog. 2018 Jan 2;14(1):e1006756. doi: 10.1371/journal.ppat.1006756 (PMC5766230; doi:10.1371/journal.ppat.1006756)
Supplement: S3 Table — (DOCX) [file ppat.1006756.s009.docx]

**Supplemenal Table 3 Oligonucleotides used in this work**

| **Primer ID** | **Sequence** | **Experiment** | **Target** | **Description** | **Notes** |
| --- | --- | --- | --- | --- | --- |
| DP44 | CCCATTCTGAATAGTTGGATGATT | RT-PCR | N gene | N gene specific RT primer during cDNA synthesis | Designed by Barbara Baker Lab (UC Berkely, US) |
| DP41 | GCGCGACATCTTGTCATCACATACGG |  |  | N gene RT-PCR complementary primer |  |
| DP43 | TATTTACCGGTCAACCGTATCCTGAA |  |  | N gene RT-PCR direct primer |  |
| ZHP055 | GCCTTGGCACCCGAGAATTCCATTTTTTTTTTTTTTTTTTVN |  |  | General gene OligodT during cDNA synthesis |  |
| DYTP226 | GGCCGCCCCGTTGTATCA |  | GAPDH gene | GAPDH gene RT-PCR direct primer |  |
| DYTP227 | TGCGACAAGCTCAACATCATCTC |  |  | GAPDH gene RT-PCR complementary primer |  |
| DYTP222 | GGCGGGGGGAGACGTACAC |  | BASTA gene | BASTA gene RT-PCR direct primer |  |
| DYTP223 | CCGCAGGAACCGCAGGAGT |  |  | BASTA gene RT-PCR complementary primer |  |
| DYTP232 | CGGCGCGGGTCTTGTAGTT |  | CFP gene | CFP gene RT-PCR direct primer |  |
| DYTP233 | CCCGCTACCCCGACCACAT |  |  | CFP gene RT-PCR complementary primer |  |
| DYTP280 | GCGGGCAACGTCTGGTATCA |  | GUS gene | GUS gene RT-PCR direct primer |  |
| DYTP281 | CGGCAATAACATACGGCGTGAC |  |  | GUS gene RT-PCR complementary primer |  |
| DYTP282 | AAACATTTACAAGTCACCTGTA | Northern blot | nta-miR6019 |  | Modification: LNA~DNA(10,12,15,17), marked with a underline |
| DYTP283 | AAAGATACTCGGAAAACATTTA |  | sly-miR6020 |  |  |
| ZQLP100 | GTGCTCACTCTCTTCTGTCA |  | nta-miR156a |  |  |
| DYTP326 | TTCCCGACCTGCACCAAGCGA |  | nta-miR168 |  |  |
| ZQLP102 | GGGGAATGAAGCCTGGTCCGA |  | nta-miR166a |  |  |
| WJBP1062 | CTCGCCCTTGCTCACCATGTA |  | TAS1 |  |  |
| WJBP1063 | ACGGCGACGTAAACGGCCACA |  | TAS2 |  |  |
| WJBP1064 | GCCCTCGCCGGACACGCTGAA |  | TAS3 |  |  |
| WJBP1065 | GCAAGCTGACCCTGAAGTTCA |  | TAS4 |  |  |
| DYTP343 | GGAGAGGGTGAAGGTGATGCA |  | TAS5 |  |  |
| DYTP344 | AAATTTATTTGCACTACTGGA |  | TAS6 |  |  |
| DYTP345 | AAACTACCTGTTCCATGGCCA |  | TAS7 |  |  |
| DYTP346 | TATGGTGTTCAATGCTTTTCA |  | TAS8 |  |  |
| DYTP102 | CAGAGAATTAACGTTGAGatgGGATTACGTCCTGTAGAA | Vector construction | GUS gene | GUS gene synthesis direct primer | Vector backbone:  pHN9M6H |
| DYTP101 | GGAGATTAGCTTTTGTTCCACGTGGTGGTGGTGGTG |  |  | GUS gene synthesis complementary primer |  |
| DYTP083 | ccagggcgccctctTTGATTAACCGGGGAATG |  | nta-MIR6019, 6020a promoter | nta-MIR6019, 6020a-5k promoter synthesis direct primer | Vector backbone:  pH7Lic14.0 |
| DYTP084 | ccagggcgccctctTAACCAACTCTACTTGCT |  |  | nta-MIR6019, 6020a-3k promoter synthesis direct primer |  |
| DYTP085 | ccagggcgccctctTTGTTTTGGTCAATTTTA |  |  | nta-MIR6019, 6020a-1k promoter synthesis direct primer |  |
| DYTP086 | cgacccgacgccctgaTCACCGACTGTGAATATC |  |  | nta-MIR6019, 6020a promoter synthesis complementary primer |  |
| DYTP087 | ccagggcgccctctTGAAGCCAGGACGGGAAA |  | nta-MIR6019, 6020b promoter | nta-MIR6019, 6020b-4k promoter synthesis direct primer |  |
| DYTP088 | ccagggcgccctctTGTTGAATCTTTTGAAAG |  |  | nta-MIR6019, 6020b-2k promoter synthesis direct primer |  |
| DYTP089 | cgacccgacgccctgaTACAAAACAAAAACAATA |  |  | nta-MIR6019, 6020b promoter synthesis complementary primer |  |
| DYTP099 | atGTCGACTAACCAACTCTACTTGCT |  | nta-MIR6019, 6020a promoter | nta-MIR6019, 6020a-3k promoter synthesis direct primer | Vector backbone:  pCambia1381xb |
| DYTP057 | atAGATCTgccatACCATTGATTTTGCTAGGGT |  |  | nta-MIR6019, 6020a-3k promoter synthesis complementary primer |  |
| DYTP105 | atGAATTCTGTTGAATCTTTTGAAAG |  | nta-MIR6019, 6020b promoter | nta-MIR6019, 6020b-2k promoter synthesis direct primer |  |
| DYTP106 | atCTGCAGgccatACCATTGATTTAGCTAGGGT |  |  | nta-MIR6019, 6020b-2k promoter synthesis complementary primer |  |
| WJBP1030 | CCAATTTCAAATAAGGCCCAA |  | sly-MIR6020 | sly-MIR6020pre direct primer | Vector backbone:  pH7Lic2.0 |
| WJBP1031 | CTGACAATTTGTAGATAAACCT |  |  | sly-MIR6020pre complementary primer |  |
| WJBP1056 | GTGTGTGGTCAGGTAAGAAAAAGATACTCGGAAAACATTTAGAACAGGGACTCTCTCA |  | AMIR6020 | artificial_slymiRNA6020_primer2 | Vector backbone:  pH7Lic1.0 |
| WJBP1057 | TTATTCTCTCATGATTAAAAAGATATCGGAAAACACTTAGATCAGTACTCTCTCGTCAA |  |  | artificial_slymiRNA6020_primer4 |  |
| WCHF017 | ccagggcgccctCACCAAACAAAACCTCACAATGAGAGAGTCCCTGT |  |  | artificial_slymiRNA6020_primer1 |  |
| WCHF019 | AACCAACAAACACGAAATCCGTCACATTTGCTTATT |  |  | artificial_slymiRNA6020_primer3 |  |
| WCHF021 | cgacccgacgccctAGTTCTGTTGGGTAATGGTATTGGGGTGAAGTGCTCAT |  |  | artificial_slymiRNA6020_primer5 |  |
| WJBP1058 | TCGAGGCGAAGATACTCGAAAAACGTTTAT |  | MS4miR6020 |  |  |
| WJBP1059 | CTAGATAAACGTTTTTCGAGTATCTTCGCC |  |  |  |  |
| WJBP1060 | TCGAGGCAAAGATACTCGGAAAACATTTAT |  | MS4miR6020^E^ |  |  |
| WJBP1061 | CTAGATAAATGTTTTCCGAGTATCTTTGCC |  |  |  |  |
