# Supplementary material for: A role for small RNA in regulating innate immunity during plant growth
Source: PLoS Pathog. 2018 Jan 2;14(1):e1006756. doi: 10.1371/journal.ppat.1006756 (PMC5766230; doi:10.1371/journal.ppat.1006756)
Supplement: S1 Data — (DOCX) [file ppat.1006756.s011.docx]

**Supplemental data 1 Tobacco and tomato NLR CDS in fasta format**

Supplemental data 1 (a) tobacco NLR CDS sequences in fasta format.

>NTTN90_mRNA_20690_cds mRNA_20690 gene_11583|id=AT5G36930.2:evalue=2e-161:annot='Disease resistance protein (TIR-NBS-LRR class) family';id=Solyc01g113620.1.1:evalue=0.0:annot='NBS-LRR resistance protein (Fragment)'

ATGGCCACGGAGCAAGATTATGAGCAATCAATTTCTAACACATATAGAGGTTCATATGAT

GTGTTTTTGAGTTTCAAAAGTGAAGATACAGGTAAAAATTTCACGGATCACCTCTCCACA

GCTTTAAACCAAGCCGGATTTCGCACGTTCAAAGGTGGTGATGATGAATCCAGAAGTACC

GAAGAAGAAGACAGCAGTTCAGATTTTGCTAAAGCAATTCAAGAATCAAAGATTTGTACC

ATTGTATTCTCACAAAATTATGCGTCTTCTAGTTGGTGCCTAGATCAACTTGTCACAATT

CTTGAACGTAAAATGAAGTTTGCATGTACAACAATATTGCCTATATTCTACCATGTTGAT

CCTTCCAATCTCAGGAAGCACAAGGGAAGTTTTGGGGAAGCATTAAACAGACATGAAGAA

AAATTCAAGTCTGAATTAAGACATGGAAACGAAGAAGAAGAGCGGGAGGAGAAACTAAAG

AAATGGAAGGATGCACTTAGTCAAGCTGCAGATTTGGCCGGAATGGTCCTTGAAAATCAG

CATGAATCCAGATTCATCAAGAAGATTATTAATGTTATCAGTACTAGACTAAGTCGGCCA

GCCTTGTATATTTCTTCTTGCACAATTGGAATACATCGTCGTGCTAGACCCATTAATTCC

TGGGTTCAACATGAATCTAATACTGACATTGGAATACTCCTGGTTTCTGGTATTGGTGGA

ATTGGAAAGACAACTCTTGCCAAATTTGTCTACAACTTAAACTTTGGATATTTTGAATGC

AGTTGCTTTTTGGCAAACATTAGAGAAACTTCAAAACTCCCTAATGGGGTCATCATATTA

CAAAAACAACTTCTTTCTACCCTTCTCAAACATGATAAAGTAAAAATATCAAGTGTTGAT

GAAGGAATTATTAAGATAAGGAATGCTTTATGTTATAGAAAAATTCTTCTCATTCTTGAT

GATGTTGATGAGCCTGATTTAGTTGAAGCAATATTTGACATTAAAGATTGGTTTGGTTTT

GGAAGTAAAATTATTGTGACAACTAGACACAAGAATTTATTAAGACCTCAAATAGGTCAT

GAGGTACATGAAGTTGAAATTTTGTACACAACTGAAGCAACTGAGCTATTTAATTGGCAT

GCATTTGGGGAAAACAATACAAGTGAAGGTTATTACAAAGAGTATTCAGAGGAAGTGATT

GAATGGTGTAGAGGACTTCCATTAGCTATTCAAGTTATCGGTTCTTCGTTGGCTGGAAAA

TCGAAGGATGTGTGGAGAAGTGCAATAGAAAAGTTGAGAGATATTCCCACCAATAAAATT

GTTGACAAATTGAGATTGAGTTATGAATTGTTGGATGATGATCATGATCAGAATTTGTTC

CTTCATCTTTGTTGTTTCTTTGAGGGGATGAAAAAAGATTTTGTGGTTAGGATACTAGAT

AAATGTGACTTTTACACATTGGTGGGGATTCAGAATCTCATCGATAGAAGTTTAGTGACA

ATCGGATATGTAAATGAAATAACAATGCATCAATTGGTACGAGATATGGGAAGAGACATT

GTTCGTCGAGAAGCACCTGTGGAGCCTGGAAAGCGTAGTAGACTCTGGCAACACACAGAT

TCTTACAATATCCTAAGAGGAAAAACAGGAACAGAGACAGTGCAAGGGATGGTACTTGAC

ATGCGTATGATTAAGAAAGGAAGGTACTCTAGCCCCTTAATACCAGTGACACAAAATGCA

ATTGCCTTTTTCACTTGGAGTACAAAAGGCAATTCTAGTTTACAGGATCTTAGAACTGAT

GCCTTTGAGAAAATGCACAAGCTAAGATTTCTCCAATTCAACAAAGTTCAAGTGGATGGA

AGTTTCAAGAATTTCCCTAAGGGATTGAGATGGCTGTGTTGGAGTGGATTCCCAGAGGAG

TGTATACCAAATGAGTTTCCAATGGGAACTGTTGATTCCATTGACATGCAATACAGCAGC

TTGAAACAACTTTGGAAAGGATTCAAGCTTCTCCCATTCCTGGAGATACTGGATCTAGGC

CACTCCTATGAGCTCATCACCACACCGGATTTCTCGGGCCTTTCCAATCTTGAAAGGTTG

ATACTTGAACATTGTACCAAGTTAATCTATGTTCATGAGACCATAGGAAGCTTGCAAAAA

CTCGTGATTTTGAACCTTAAAGATTGTCAAAAACTAAAGAGCCTTCCTGACAGCATTTGT

GAGTTAAAATCTCTTGAGACACTTAATATATCAGGCTGCTCAAATATTGAATATCTGCCA

ACAGAACTGGATAAATTGACTTCGCTAAAGGAGCTTTATGCTGATGGGATCTCTATGAAT

AGGGTGGCAATATCTACTGAATTGGGAGCCCATACATGGTATTCATCTTTGTATTCTTGG

GCCTTGAAAGGAAGGGTAATATCCCCTAAAAAAATTCAAGAAATCCATTTTCCTAGGTCC

TTGCATGTGTTAAACATTGCAAAATGCAATTTATGTCCAGATGCATTTAGCAATGTTGAT

TTAGGCATTCTGAGCCTGTTAGATTGGCTAGATTTGGGAGGAAATCCAATCTGCAATCTA

CCAGATAGCATCAAGAATCTTACAAGACTTAAAACTCTTAACATCGCGTACTGTACAAAG

ATCAAGTATCTTGACGGATTACCATCAAATATCAGTGACTTAAATGCTGATGGATGCATG

TCTTTAGAGAAAGTAGCAGCTTCTTGTGCAAAAGGACATCCAGTAGAAGGTTATTTAAAT

TGCATCAATCTTGTTGAAGCTGAGGGTGTTTTCAAGTTAGAGCCACTGGAAAATGCAGAT

GCTCAAGTTCTTGCCAATATGGGAATTTCAACTTTGGAACCTATAAAAAGTAGTATTAAG

GTTTCTCTGGTATTTGGTCGCGCGTACAATGCTGGAAGAGTTAGAAATGAATACCCAAGT

TGTGTCCAAGATGATATAGCAAGTTTATTTTCACTTGAGCCTAAGAAGTTCCCTCCACAG

ATATTGTACCACCGCGGCATTTTCTCCACTTTTTTACCTGGTGAAAGCGTACCTAATTGG

TTCAGCTACAAATTCGCTGATGCAGCAGATGTATATTGCACCCTACCAAATGTTGATAGC

CATGGAGTAATAAGTGGTTTGAGCATTTGCTTTGTGTACGCATGTCCTGAAGCATACACC

AATGTTGGATTGTACGACGGACCAGCCATTTGGGTAAGAAATCGGACAAAATACTTAAAC

TGGGCTCTTTATCCTGCTTGGTTTGGCCTTCCAGAGGATGAAATTAGTGGGATGATGTGG

TTAAGTTATTGGAAAGTTGAGACTTTGTTTCAACAAGGAGATGTGATTGAGGTTATGGGG

TCTCCTCAGTTTGCTGCATTTAAGGAGCTTGGTGTTAAAATCTTTTACCTCGACGAGCAA

ACAGAGAATATTTCTGATCATTCTAATGTGTGTAAAAGTAGCAACCCGTTTGAAGATATT

CTAAAGAACCACTTTAGTGATGAAGAACGTACTTATTACATTTGCACTTGA

>NTBX_mRNA_13089_cds NTBX_mRNA_13089 gene_7477|id=AT1G27170.1

ATGGCGGAAGAAGAAGAAGAGACGTGGTCCTTGACCTCAGGGCATAGGTTCCATTGGGAC

ATATTCCTCAGCTTCAGAGGAGAAGACACACGCCACGGATTTACCAACAAACTCTACAAT

GAACTCGTACGGAATGGCGTACGGACATTCATCGACGACGAAGGTCTGGATCGCGGCGAG

GAGATCGCTCCGAATCTCTCTGCCGCGATCGAAGACTCGGCGGCTTCGATCGCCGTGATT

TCACAGAACTACGCCTCTTCGAAGTGGTGCCTCGAAGAACTGGTGAAGATCTCGGAATGC

AAAAGACTATTGCTGCCTGTTTTCTACGGAGTTGACCCGTCGGACGTCCGAAGACAGACG

GGTCCGTTTGAGGAGCATTTTAGGAAGCACGAAATAATGGTGGAAGCAGAGAAAGTTTGT

CGGTGGAGAGAAGCTATGAAAAAAGCTGGTAATATCTCCGGTTGGGATTCCAAGCTCTGG

GAAGAATCGGAGTTAATCCATTCTCTAGTCAAAGAAGTTCTGTCGAAACTCAATAACACA

CCTTTAGGCGTGGCAAAATATCCAGTAGGTCTTCATTCTCGTCTTAATGAGTTATGCAGG

AAATTGGACGTGAAAGGAAATGGTGTGAAAGTATTAGGACTATATGGAATGGGGGGAGTT

GGGAAGACCACTCTTGCCAAGGCTTTGTACAATCAGTTTGTTGTTTATTTCAAGAAACGT

AGCTTTATTTCAGATGTTAAAGAAATTGCAAGGCGCCAAAATGGTATGGCCACTCTTCAA

AGCAAACTTATTGGTGATCTTAACTCAGGTGCTTCGCCAATCATAGACGATACTGCTAAA

GGAATCCGATCAATCAAGGAGGCTATGAATAATGAGCCAGTTGCTGTTTTCCTAGATGAT

GTGGATAATGCAGACCAACTTCGTGTGTTGGTTGGTAGGAGAGACTGGTTTTGCCAAGGA

AGCAGAGTCGTCGTCACCACTAGAGATCAAAATGTTTTACTCCCAAGTATTGTAAACGAA

ACTTTTGAAGTGAATGAGCTTTCTTTGTCCGAGTCATTTACGTTATTTAGTTATCATGCA

TTTGGAAGAGAGCATCCTCCTAAGAACTTTTCTGATCTTGCTGAAGAAGTTGTAAAACTC

AGTGGGGGATTACCTCTGGCTCTGGAAGTTTTCGGATCTTTATTGTTCTACAAGAAAAGA

TTAAAGGAGTGGGAAGATTTAGTGCAAAAGCTGAGACAGATTCGCCCGGGTGATCTTCAA

CAAGTCTTGGAAATAAGTTTTGGAGCTCTAGATGAACAAGAAAAGTGCATCTTTCTTGAT

TTAGCATGTCTTCTTCTTAATACAAGGCTTGAAAGGGAAGATGCAATTGCGATATTTGAA

GGTTGTAGCTTTGGCGCTGAAAGTGCAATCACAGAGCTCACAGCAAAATCGCTTCTTAAA

ATCGTTGATGGGAATATTTTATGGATGCATGATCAGCTCAAAGACATGGGAAGGCAAATT

GTACAACATGAGAATTTTGCAGATGCTGGTAAACGTAGTAGACTGTGGAATCATGACGAT

ATTCTGACTGTCCTAAAGAACCACATGGGGACAAGAACAATTGAGGGCATTGTGCTTGAC

TTTGAGAAGAAGCATGATCTAAATCCTAAGGAAGTAAAGTGGAGTTTGAAGAAAGTATTT

AGAAAGTATATTGGTCAAGGTAGAAAGGAAAATGGTGTAACATTTTACACTAGAGCTTTT

CAGCGTATGGTAAAACTGAGACTTCTTCAAATCAATCATGCCAAATTGGTTGGAAATTTC

AAGCTATTACCTGCTGAACTGAGGTGGCTGCAGTGGAAAGGTTGCCCTCTGGAAGTTATT

CCTCCAGAATTACTGTCCCGAAAGATTGCAGTTCTTGATTTGTCGGAGAGCAAGATTACA

CAGCTCTGGAATAAGAAAAAGTGGAATTGCTACCAGAACAAGATGGCAAAGCAGCTGAAA

GTTATTAATCTACGTAGTTGTCGCCAGCTTAAGGAAATCCCTGATTTATCTGGAATTCAA

TTGGAGAAGCTGATTCTTGAGCAATGCAACGAACTAGTTCTGATCCATCCGTCAATTGGA

GACTTGACTATGTTGACCTATTTGAACATGAAGGACTGTAAGAACATTTTGGCATTTCCA

AATGACGTGTCTGGATTGAAACGTCTACAAATACTAATCCTATCTGGTTGCTCAAGTCTA

AGAGAATTACCAGAGGACTTGAGTGGCTGGAAATCTTTGCGAGAGCTTCTTCTAGATGGT

ACAGCAATAAGAAAGCTACCTAACTCTATCTTTCACCTGAAGAGTCTTCAGATCTTGAAT

TTAAATCATTGCCGATCTTTGGAGTTACTGCCTAGAGCTATTGGGAATCTAAGTTCGCTG

AGAGAGCTTTCTTTTAACGGATCTGCTTTAAAGGAAATGCCCGATTCCATTGGAAATTTG

AAAAATCTTGAGGAATTAGGCTTGAGAATGTGCAAGGGGCTCATCTCACTTCCTGATTCC

CTTGGCGATCTTAAATCTTTAATAGGACTTTATCTTGATCAGAGCTCCATAGAAGAATTG

CCACCTTCTGTTGGTTTATTATCTCATTTGAAGTTCTTTACGGTCAGCAATTGCAAGTCC

TTAACCGAATTGCCAAATTCCACGAGTAACTTATCGTCATTGGTTTGGCTTTGTCTACAA

GGGACCTCAGTTAGTGAACTAAATTTCCATTTAGGAAACTTCAAGTCCCTTGAGAAGCTT

GAGATGAGGAACTGCATTTCAATCAGATGTTTACCTGACTCAATTGGAAATATGTTATGT

TTAACTACTTTGGCCCTATGCAATACATCGATTATTGAGTTGCCAGACTCTATAGGCTTA

TTAGAAAGACTTTGGATGTTGGACCTGAGCAACTGTTTGAATCTCCAACGTCTTCCGGCT

TCAATTGGAAGACTTAAGAGTTTGTGTTACCTCTATATGGATGAAACTGCTGTCTCAGAA

TTACCCAATGAAATTGGAAAGCTTTCAAGCTTAAAACTACTGAAGATGAGAAAGAAACCA

CAGCCTAGAGAGGACGGAAATGAGGATGATTTACATGTAGGAGAAAGCTCAAAACGCGTT

ACTCTTCCAGAATCATTTTCAAATCTATCATCCTTGGAATTCCTAGATGCTCATGCATGG

AAAATATCCGGAAAGATTTCTGATGATTTTCAAAAGTTGTCTGCTTTGGAAAAGCTCGAC

CTTGGACACAACGATTTTTGTAGTCTCCCTTGTAGCATGAAAGGACTTTGCGTTCTCAAG

CGTTTGCTTCTCCCAAACTGCAGAAAGCTCAAGTTTCTACCTCAACTTCCTTCAAGTTTG

GAATGGTTAAATGCTGCAAACTGCTCCGCGTTGGAACATATAGCTAGCATATCAGATTTG

GAATATTTGGAAGAACTCAACTTCAGTAATTGCAAGAAAATAATAGATATTCCTGGCCTT

GAAAGCCTGAAATCTTTGAGAAGGTTGTATACTATAGGTTGCAACGCGTGCTTTGCTTCT

ATAAAAAGGAGGATTTCCAAGGATTGTCTAAGGCATATGAAGTATCTCTGTGTTCCAGGG

GATGATCTTCCAGATTGGTTTATTAAGGAAGTACCTATTAGCTTCTCAACTCGCAAGAAT

CGTGATATCAAGGGGGTGATCATCGGTATAGTTCTCTCGCTAGACCAACAAGTGGAGGAT

AATTTCAGACACGAAGTCCCTTCGATCGTAGATATACAAGCAACGATTACCAGACAAGGT

GATGTTGAACCTAAACTTAAGAAAACTTTGTACTTGATGGGGGTTCCTGATACAGATGAC

GATCAGCTCTATTTATGTCGATTTCAAGAATACAGTGATTTTACGCTCATGTTGGAAGAC

GGAGACAGAGTGCAGGTTGGAATTAGAGAGCGTCCACGTTTTAATGGCCTTAAACTGAAG

AAACATGGGATGTACTTAGTTTTCGAAAATGAGGATGATTTTGATGATAATGATGAGGAT

TTGTTTGACGAATCTCAGCAGTCTGTGTCAAAGAAACTTGCTGACTTCTTTCATTCATTA

TGA

>NTTN90_mRNA_88824_cds mRNA_88824 gene_50216|id=AT5G36930.1:evalue=0.0:annot='Disease resistance protein (TIR-NBS-LRR class) family';id=Solyc09g092410.2.1:evalue=0.0:annot='Tir-nbs-lrr, resistance protein'

ATGGCTGATCAGAAAGGCAAAGATCAATCGTCTAATTCTCAGCCTTCGCTTGCACTTCGC

CCTTGGAAATATGATGTCTTCTTGAGTTGTAGAGGTGGTGATACTTCAAAATACTTTGCA

GATCAACTATACTTAACACTCTGCCAAGTTGGGGTCAATACATTCAGAACTGATGATGAG

GGGGGACATGTTTCCTCTGAAGTAGTCATGAATGCAATTGAAGGATCAATCATTTTTATT

ATTGTTCTTTCCAAAAACTATGCCTCATCTAGAAGGTGTCTTAATGAGCTTCTACATATC

CTTGAGCTCAAAAAGAATTCCAAACGGTTAGTTCTTCCTATATTCTATGATATTGACCCT

TCTGATGTGCGCAAACAAACAGGAATTTTCGCTGAAGCCTTTGAAAGGCATGGAATATGT

TCTCAATCAGAGCAAAACATTCAACTATGGAGAGCTGCACTCAGTAGAGTTGGTAATTTA

TCAGGATGGGATCTCAAGCATGTTGCTGAAGGGTTTGAATCCAAATTTATCCATATTATT

ATTGAGGAGGTCTTACAGGAAGTCAAATCTCGAACACCCCTCTATGTTACCAAGTACCCT

GTGGCACTTTTTCCCCGTGTTAATCAAATAGAGAAGTTATTGTTCAAAGGAGGTTGTGAT

GATGTTCGTGTGATTGGGATTCACGGCATGGGTGGAATTGGCAAAACAACTCTTGCAAAA

GCTGTGTTTAACCAACTTCTTCAGCATTTTGAGGCAAGTTGCTTTCTTGAAAATGTGAAA

TCAGGGGCTTCTGAAAGACATAATGCATTAGTTCATTTACAAGAGCAACTTCTTCGAACG

ATTCTTAGGAGAAAGATCAAAGTGCACAATGTGGATGAGGGTATTACATTGATCAAAGAA

GGGATTTGGCAGAAAAAGGTTTTCATAGTCCTTGATGATTTGGACGATCAATGCCAGTTA

AATGCATTACTTGGAGAACGTGATTGGCTTCGCCCGGGTAGTAGAGTTGTTATAACAACC

CGAGACAAGCATTTGCTCAAAGAACTACAATTGAATGAGCAATATGAAGCCATGAAATTG

GATCACGAAAGCTCTTTACAACTCTTCACTTTACACGCCTTTAGAAATGCACCACCGGCT

GAAGACTATAGTATGCTTGTGGAAGGTATTGTAACTTACTGTGCAGGAGTTCCATTAGCT

CTTCAAGTTTTGGGCGCTTATTTGTCTGATAAAAAGATCGAAGAATGGAAAAATGCCTTG

GAGAAATTAAAGACGATTCCTTCTAACGATATTCATGAAAAACTCAGAATAAGCTTTGAT

GGACTTCCTGATGATTTTACAAAAGCTGTTTTCCTTGATATTGCTTGTTTCTGCTTCAAA

GTCCAGAAGAGTGAGGTCGTAGGTATATTCACAGCATGCGGTTTTTACCCTGAGGTTGAA

ATTTGCGAATTGATTGACAAATCTTTGTTAGCAATCGATGAAAATAAGAATTTGAATGTG

CATAATTTGATCCGGGATATGGGACGAGAAATTGTTCATAGGGAATCACCCGATAACCCG

GGGAAGCGTAGCAGATTATGGTGCCCTAAAGACATTTCTGATGTATTAATAGGACACAAG

GGCACAAAAGCAGTTGAAGGAATAGTCCTTGAATCTTCAGCATTGAAGGATGTACCTTTT

AGTACAAAAGCATTTGAAAAAATGGCCAAGTTAAGACTCCTACGCATCAATCATTTGCAG

TTATATGGAAGTTTTCAGTATCTACCAAAGTCACTAAAATATTTGCATTGGCACTATTGC

CCTTTGAAATGCTTGCCATCTGACTTTTGTCTGGAGAATCTTGTCATTCTCAACATGAGT

TTTGGCAATTTCAAGGAATCCCAAGCGCCATTAAAGTATTTCAAGTGTTTGAAGATGTTG

GTATTCTACAGTTGTGAGAATCTCAAGAAATCCCCAGAGTTCGTTGGTTTACATAGTCTT

GAGAAGTTATCATTTGGTTATTGCTCAAATTTGATGGGATTGGATTCAACAATTGGAGAA

TTGAAGAGACTTCGTATGTTAGACGTAGCTAATTGTATGAACCTACGAGAACTCCCACGA

AGAATCTGTGAGTTAAAATCACTTGAAATCTTATATCTCTATGGCTGCTCAAAACTAGAA

GAATTGCCTGACGATTTGGGAAAGTTGGAACGTCTGAAAGAATTGAGTGCAGTTGCAACA

GCTATCACAAGATTACCTGGTTCTGTTGGACATTTAAAGAACTTGGAGATGCTATTGCTA

TCACAGGACTTCCTATTGAAAAGACAATCCAAATTTTCAGACATATTCTCAACTTGGTTG

CAACCAAAAAGAAGCCTTAGTAGAGTGGGATATTTACCTTCTTCATTTTCAAATTTAAGT

GCTTTAAAAGTTTTACAAATTGAGAACTGGAATATGACTGAAGATGATATTCCTTTTTCT

CTTGCGAGCTTATCATCTTTACAGAATCTATGTTTTAGCAAAAATAAGTTTCGTGCTATA

CCTTTCAACCTTTGTGACCTTTCCAGTCTAAAGTATCTGAATTTAAGCGAATGTCCGAAT

CTTAAAAGTATTCCTGAAATTCCTCCCACTCTTCAGAATATCAGAGCTTATAAGTGCAAG

TCATTAGAGAGACTTCCAAATTTGTCAGGCTTGAAAAGGTTGGAGGAACTGGACTTGTGT

CGTTGTGAAATGTTGATGGAGATTCAAGGCTTGGAGAACCTTGATTCTGTCAGACGACTA

AGCTTATGGAGCTGCAAGAGTTTTGGAAGACTACTCGATGTATCTAACTTGAGTAAATTG

AAGAACTTGGAACTTAGTCATTGCGAAAGATTGATAGAGATTCTGGGCTTGGACAACCTT

CATTCCATACGCTACATCAACTTATTCAATTGCAAGGCTCTCAAAAATCCTTTCACTGAA

AACTTCTTCAAAGCCCATTATGAACATGGTAGTGAGCTCCAACTAGGGCTTTGCAACAGC

AATGTTCCGAATTGGTTTAGCTACAAAGTAGATGGATGTTCAATGTGCTTCAATATGCCT

CTACAGGGGGAGAGTACATTCTTGGGCATGTTCCTTTGGGTTGTTTATGGAACAGTGGAT

GAAACTAAAAATGTTTATCCTAAAGCCACCATTGTCAATCAAACAAATGGCGTTGAGTTT

AACCATCGTCTGTGGACGACCATATCCTTTGCAGAAAATTCGTCCATCCATTACATACCA

CCAAATTACTTCAAATGTCCAGTTAAAGGCAGAGAAATGATGAGCATTCTTATTGAATGC

TATGACTTTCCAACTGAAGATTTTGTTAAGAAATGTGGAGTTCATCTGTTGTACAAAGAC

AAGAATGGCCAGGTTCATTCTTTGTCTGTGAGTTCTCCTGGTTTTCTTTAG

>NTBX_mRNA_49496_cds NTBX_mRNA_49496 gene_28941|id=AT5G36930.1

ATGGATACTCAATTAGTTAGAGGAGAATTATCTACGTCTTCTCACTTCTCTTATGAAGTA

TTCCTGAGTTTTAGAGGTGAAGACACCCGGAAAACATTCACTGGTCATCTTTATTCCAAA

TTGGATAATGTTGGAGTCAAAACCTTCATTGACGATGAGGAATTGAGAAAGGGTGATGTG

ATTTCAAGAGAATTAGAGAAAGCAATTGAAGGGTCAAGAATTTCCATTGTAGTATTGTCG

AGAAATTATGCTTCCTCTAGTTGGTGTCTAAATGAACTAGTTAAAATTCTTGAATGCAAA

GAGAAATTAAAGCAGACGGTTTTGCCTATTTTCTATGATGTTCATCCTTCTGAGGTACGA

AAGCAAATTGGGTTATTTGGTGAAGCTTTGGCTAAACACAAGGAACGACCATTTGGAGCT

CAAATGGTGGAGAAATGGAGAGCTGCACTTACTGAAGCTGCAAATTTATCTGGATGGGAT

TTGCAAAATGTTGCTGACGGGCATGAATCAAAGTTTATTGAAAACATTATACAGCAAGTC

CTACAAGAGGTCAACCAGACACCTCTAGATGTTGCTTGGCACCCAGTTGGAGTAGATTCT

CGTGTCAAAGATATAGAGTTGTTATTGCAAAATGAATGTGAAGATGAAGTTCGCATGATT

GGTATTCACGGAGTTGGTGGCATAGGGAAAACAACTCTGGCAAAAGCTATCTACAATCGA

ATGTTTCGACTCTTCGATAGTAGTTGCTTCCTTTCAGATGTTAGATCAGAAGCTGAAGAA

TTTGGTCTTGTCAAGCTACAAGAGAAACTTCTTCAACAAGTTCTCAAAAATAAGGACATC

AAAGTTGGCAGTGTTGCTCAAGGCATCAATCTAATCAAAGCAAGACTCGAGTCAAAGAAG

GTTCTAATTGTTCTTGATGACGTGGACCACAAAAACCAATTAGTATCCCTAACAAGAGAA

AGAAGTTGGTTTGGTTCGGGTAGTTTTATAATCATTACCACCCGAGACAAGCGATTGCTA

TGTCGGCTTGGAGAAAAAGAGAGATATGAGGCCAAACTATTAAATGACAAGCGATTGCTA

TGTCGGCTTGGAGAAAAAGAGAGATATGAGGCCAAACTATTAAATGGCAATGAAGCTATG

TTACTTTTTTGTTGGCATGCTTTTGACAGTGATTTTCCACCACAAGATTATGTTAATTTG

GCACACGACATAATCGAATATTCAGGCAGGCTACCATTAGCTCTTGTGACATTGGGGTCA

CATTTACAAGGAAGTTCTGTAGAAGAATGGGGAAATGAATTAGAAAAACTAAGAGCAATT

CCTCATTGTGATATCCAAACGATTCTCAAGAAAAGCTTTGATGGACTTGATGATGAAACA

CAGGCAGTTTTTCTTGATATTGCGTGTGCCTTCCAGGGGTTATTTGAGTATGAAATTACC

GAAATATTAAATACATGTGGCTTTCATGCTAAAATTGCAATTGCAACTTTAGTCCAAAAA

CACTTGCTCCAAAGATCTTTGCATGGTTTGGTGATGCATGATCTAGTGCGAGATATGGGA

AGAGAAATTGTTCGCATGGAATCAGCTCGAGATCCTGAAAAATGGAGTAGATTGTTCATC

CCGCAAGAAGTCTGTGATGTTCTACAAGGAAATAAAGGTTCCAAAAAGGTAGAAGTACTG

AAGGTAGATCGACGAGCATTTGAGGGACTAAACTTGAGCACCAAAGCATTTAAGAAAATG

AAAAACCTTAGGGTTCTTATAATGAATGAGTTACATATTAGTGGAGATTTTGGGTTGTTG

TCCAAGAAGCTCAGATGGTTGTCTTGGAAAAAATGTCCTTTAAAATGTATATCATCAAAT

TTTCCAGCTGAGAATCTTGTAGTTCTAGATATGGAGGAGAGTGATATCCAAGAATTTCGA

TTGAATTTGCAGTGTTGTAGAAGTTTGAAGAAGCTGAATCTCTCTTATTGCAAGCAACTC

AGAAGCACTCCAAACTTCAATGGTTCCCTGAGTCTTGAGATTTTGAGTCTCTATGGTTGC

TCAAGTCTGACAGAGATCCATCCATCAATAGGAAATTTGGACAGACTAATTAAACTATAT

ATGTCTCGTTGCGAAAAACTTAGGGATCTTCCAAGCAGCATATGCCAGCTAATATCCCTT

GAAGAATTGTTCATTCATCACTGCTCCTCTATAAAAACACTGCCAGATAACCTTGGAGAT

ATGAAAAGTCTAACATTTCTTTATGCATCTTGTACGGGTATAAAACAATTGCCTAGATCT

GTTGAAATGCTAAGAAATCTTGTAGCTTTGAGAGTGGGAGGTCAAGTGTTAGAGGCCAAA

ATGAGTATTTCTGGAAGAGGAGTCCATCGGATACAATATTCCTTGCTAACTTTTGTAACC

GAATTGAGCCTTAGATACTGGAATTTGTCCGATGCTGATATTCCTAGGGATATTGGGAGC

TTATCCTCCTTAAAGTTTTTAGATTTGAGTGGCAATAGTTTCCATTATCTACCCTTCGAT

TTTTCTAAGTTACGATTATTGGAGAAGTTGTGTTTGAATGACTGTGAGAATCTTCAAACA

CTCCCGCCAGTATCAAATTTAGAGAATCTAGCAATTATTGAACTTGAGAATTGCCAAAAA

TTGGTCAAGATTACACAGTTGGACAACCTCCCTTCTATACGGTTGATCAACACGAATAAT

TGTAGTTCTCTGCAGAATCCATTCAATGAAGGCTTCTTTAGTGCACCTGCTCTATCATTT

CTATCTAGAAGAGATCCAGATTTGGATGTTTTAGATTTTTATCTCGAATGCAATGAAATT

CCAGAATGGTGCAGCTATCAAGTAACAGCTTCATCTATGTGTTTGACTATGCCGACACAT

AATAATGAGTATAACTTCTTGGGAATGGTTCTCTGGTTTGTTTCCGACTATTTCCATGCA

GCCCCTGTTTTAAAATTCTGGATTAGTATTGCCCATAAAGAGAATTTAGTTTTTCCTCCG

TGGAGTACACCTGATGGACATAGAGAAGTATCATGTGTATATTACATATCTTACTTACAT

AGAGCTTTTGATGGCCAGATGATCAAAGGTGGGGAAACGATAGAAGTGGGGTCAGAAGAC

ATTACAATAAAGAAGATAGGGATCCATCTGTTATATTTAGGCCAACATGGTAACGTTATA

TCTTTACCGGGAGACGTGGATCATTCTTATTCTAAGTACCCAAAAAGTTCAACAGGCATA

TCAACTCCTTATAAAAGAGCAAAGTTCTGA

>NTK326_mRNA_114984_cds NTK326_mRNA_114984 gene_67960|id=AT5G17680.1

ATGGCATCATCTTCTGCTTCTACGAGTACTTTACAGTTTCCTCGGTGGAACTACAAAGTC

TTTCTAAGTTTTAGAGGTGAAGATACTCGAAAAATATTTACAGGTCACCTCTTCAAAGGC

TTGGAAAACAGTGGAATATTCACGTTTCAAGATGATAAAAGGCTAGAGCATGGCGCATCA

ATACCAAATGAACTCTTGAAAGCTATCGAGCAGTCTCAAGTTGCTCTCGTCATTTTCTCA

AAGAATTATGCCACATCTAGGTGGTGCTTAGATGAGTTAGTGAAGATCATGGAATGTAAG

GATCAATGCGGACAAACTGTCATACCAGTCTTCTATGATGTGGATCCATCACATGTTCGG

AAACAGAGGGAGAGCTTTGCTGAAGCCTTTGACAAACATGAAACAAGCTATAAGGATGAT

GATGAAGGAATGCTGAAGCTCCAAAGATGGAGGAATGCTCTAACTGCTGCCGCAAATCTA

AAAGGATATGATGTCCGTGACGGGATTGAAGCAGAGAATATTCAGCACATTATCGACCAA

ATTTCTAAATTGTGCAATAGTGCTACTTTATCTTCTTTGCGAGATATTGTGGGAATAGAT

ACTCACTTGGAGAAATTAAAGTCCCTACTTAAGGTAGGAATTTATGATGTTCGGATCATA

TTGGGGATCTGGGGCATGGGCGGTCTAGGGAAGACGACAATAGCAAGAGCCATTTTTGAC

ACTTTATCTCATAAATTTGAAGCTGCTTGTTTCCTTATGGATATTAAAGAAAATGAAAAA

AGACATCAACTGTATTCTTTGCAAAACACCCTTCTCTCTGAATTGTTAAGAAGAAAAGAT

GATTATGTCAATAATAAGGATGATGGGAAGCGGATGATTCCGGACAAACTTTGCTCTAAG

AAGGTGCTAATTGTGCTTGATGATATAGATCATAAAGATCATTTAGAGTATTTAGCTGGT

AATCTTGATTGGTTTGGTGATGGCAGTAGAATTATTGTAACAACTAGAGACAAGCATTTC

ATAGAGAAGAATGATGTAATATATGAAGTGACTGCACTACCTGAGAAGGAATCCATGCAA

TTGTTCAATCAACATGCTTTCGGAAAAGAATTTCCAAATGAGCATTTTAAGGAGCTTTCA

TTGGAGGTTGTAAATTATGCTAAAGGCCTTCCTTTAGCCCTCAAAGTGTGGGGTTCTTTG

CTGCATAACCTAGGCTTAACTGAATGGAAAAGTGCGATAGAGCACATGAAAATTAATTCT

AATTCGGAAATTGTTGAAAAGCTCAAAATCAGTTATGATGGATTGGAGCCCATCCAAAAA

GAGATGTTTCTAGATATAGCATGCTTATTGCGAGGGCAAGAAAAAGATTACGCCATGCAA

GTTCTTGAGAGTTGTCATATTGGAGCTGAATACACATTGCGTATTTTAATTGACAAATCT

CTTGTGTTCATCTCTGAAAATGATGAGATTCAAATGCACGACTTTATAGAAGATATGGGT

AAATATATAGTGAACTTGCAAAAGAATCCGGGAGAACGCAGCAGATTATGGCTCGCCGAG

GATTTTGAAGAAATCATGACCAATAATGCAGGGACCACAATGGAAGCAATCTTTCTTGCT

AATTTCAATAATGGTAGATTACGCTTTAGCAAAAAGGCCATGAAAAATATGAAAAGGCTT

AGGATATTTAACATTAATGTCGACATATTTGAGGAGAGGTTGTCGCCCCGTTATGCCATT

GAGTATCTGCCCAACAACTTGCGTTGGTTTGTGTGGAAAAACTATCCTTGGAAGTCATTA

CCATCTACATTTGAACCCAAAATGCTTGTTCACCTTGCACTCTCGGATAGTTCACTGCGT

TATTTATGGATGAAAACAAAGCATTTGCCGTCTCTACGGAGGATAGATCTCAGCGGGTCT

AGAAGACTGATGCGAACACCGGATTTCACGGGGATGCCAAATTTGGAGTATTTGAATCTG

TTTTGTTGTGATAATCTTGAAGAGGTTCATCATTCCCTGGGATGTTGCAACAAACTCATT

CGGTTAGTTTTGTTTCATTGTCGTCGCCTTAAGAGGTTTCCATGTGTTAACATGGAATCT

CTTGAATATCTGGATTTAATATATTGCTCTAGTTTAGAGAAATTTCCAGAAATACACGGG

AGAATGAAGCCGGAGATACAGATTCACATGCGAAGCTCTGGGATAAGGGAACTACCATCC

TCTATTTTTCAATACCAAACTCATATTACCGAGCTAAATTTGAGTTATATGAAAAACCTT

TTAGCTCCTCCAAGCAGCATCTGTAGGATGAAAAATTTGGTTATTTTAGATGTGTCGGAG

TGCTCAAAACTTGAAAGCCTGCCAGAAGAGATAGGGGATTTAGAAAACTTGGAAAAGCTT

GATGCCAGCTGGACTCTAATTTCACGACCTCCATCTTCCATCGCACGCTTGAACAAACTT

AAAGTATTGGATTTTGGATTCGTACGGGATAGAGTGCACTTTGAGTTCCCTCCAGTGGCT

GAAGGATTACGCTCATTGGAAATTCTGAATCTCAGGTACTGCAATCTAATAGATGGAGGA

CTTCCGGAAGACATTGGATCCTTATCCTCTTTGAAAGAGTTGAATCTCCATGGAAATAAT

TTTGAGCATTTGCCTCGAAGCATAGCCCAACTTGGTGCTCTTCGATCCTTAGACTTATCA

TATTGCCAGAGGCTTACACAGCTACCAGAACTTCCCCCTAAATTAAATATATTGCATGTT

GATTGTCATATGGCTCTGAAATTTATCCATGATTTAGTAACAAAGAGGAAGAAACAACAG

AGGGTGATATTCAAACCACTGTATTATAAGGATGATGCACACAACGATGCTATATATAAT

TTGTTTGCACATGCTCTGTTTCAGAATATCTCTTCCTTGAGGCATGACATCTCTGCTTCA

GATTCATTGTCCGAAAGTGTGTTTACCATTGTGCATATTGAGAGGAAGATCCCAAGTTGG

TTCTTCTATCAGGGAACGGATAGTAGTGTATCAGTTAATTTGCCTGGAAATTGGTATATA

CCTGATAAATTCTTGGGATTTGCTGTATGTTACTCTGGCAGATTAGTTTACACTGCAACT

CAATTGATTCCCGTAGGTAATGACACAATGTCGTGGATGATCCAGGAACTATACTTATCC

AACCCTTCAGAATCTGATTCAGAATATTATATACGTTTTTTCTTTGTACCTTTTGCTGTC

TTATGGGATACATCTAAGGCAAATGGAAAAACACCAAATGACTATGGGATTATTAGGCTA

TCTTTTTCTGGAGTAATGAAGAAGTATGGACTTCGTTTGTTGTATAAAGATGAACACCAT

GACACCCTGACCGATGCAGCCAGTTCCTGTCGCATACTGTAA

>NTK326_mRNA_99997_cds NTK326_mRNA_99997 gene_59218|id=AT5G36930.1

ATGGATACTCAATTAGTTCGAGGAAAATCATCTACCTCTTCTCCCTTCTCTTATGAAGTA

TTCCTGAGTTTTAGAGGGGAAGACACCCGAAAAACATTCACCGGTCATCTTTATTCCAAA

TTGTGTGATGTTGGAATTAATACCTTCATTGACGATGAGGAATTGAGAAAGGGTGATGTG

ATTTCAAGTAAACTAGAGAAAGCAATTGAAGTGTCGAGGATTTCCATTGTAGTTTTCTCG

AGAAATTATGCTTCCTCTAGTTGGTGTCTAAATGAGCTAGTTAAAATTCTCGAATGCAAA

GAGAAATTAAAGCAGATGGTTTTGCCTATTTTCTACGATGTTGATCCTTCTGAAGTGCGA

AAAAAAACTGGGTTATTTGGGGAAGCTTTGGCTAAGCACAAGGAGCGACCATTTGGAGCT

CAAAGGGTGGAGAAATGGAGAGCTGCACTTACTGAAGCTGCAAATTTATCTGGATGGGAT

TTGCAAAATGTTGCTGACGGGCATGAATCAAAGTTTATTGAAAAAATTATACAAGTAGTC

CTACAAGAGGTCAACCAGACACCTCTAGATGTTGCTTGGCACCCAGTTGGTGTAGATTAT

CGTGTCAAAGATATAGAGTTGTTATTGCAAAATGAATGTGAAGATGAAGTTCGCATGATT

GGTATTCATGGAGTTGGTGGCATAGGGAAAACAACTCTGGCAAAAGCTATCTACAATCGA

ATGTTTCGACTCTTCGATAGTAGTTGCTTCCTTTCAGATGTTAGATCAGAAGCTGAAGAA

TTTGGTCTTGTCAAGCTACAAGAGAAACTTCTTCAACAAGTTCTCAAAACCGAGGACATC

AAAGTTGGGAGTATTGCTCAAGGCGTTAATCTAATCAAAGCAAGACTTGGGTCAAAGAAG

GTTCTAATTGTTCTTGATGATGTTGACCATAAAAGACAGTTAGAAGCCTTGACAAGAGAA

AGACATTGGTTTGGTTCAGGTAGTTTAATAATCATTACCACCCGAGACGAGAGATTGCTA

TGTCAGCTTGGAGAAAAAGAGAGATATGAGGCTGAACTATTAAATGGCAATGAAGCTATG

TTACTTTTTTGTTGGCATGCTTTTGACAGTCATTTTCCACCACAAGATTATGTTAATTTG

GCACACGACGTAATTGAATATTCAGGTAGGCTACCATTAGCTCTTGTGACATTGGGGTCA

CATTTACAAGGAAGTTCTGTTGAAGAATGGGGATATGAATTAGAAAAACTAAGAGCAATT

CCTCATTGTGATATCCAAAAGATTCTCAAGATAAGCTTTGATGGACTTGATGATGAAACA

CAGGCAGTTTTTCTTGATATTGCGTGTGCCTTCCAGGGGTTATTTGAGTATGAAATTACC

GAAATATTAAATGCATGTGGCTTTCATGCTGAAATTTCAATTGCAACTTTAGTCCAAAAA

CACTTGCTCCAAAGGGATGATCTCTATTTGGTGATGCATGATCTAGTGCGAGATATGGGA

AGAGAAATTGTTCGCATGGAGTCAGCTCGAGACCCTGGAAAACGGAGTAGATTGTTCATC

CCTCAAGAAGTCTGTGATGTTCTACAAGGAAATAAAGGTTCAAAAAAGGTAGAAGTACTG

AAAATAGATCGACGAGCATTTGAGAGACAGAACTTGAGCACCAAAGCATTTAAGAAAATG

AAAAACCTTAGGGTTCTTATAATGAATGAGTTACATATTAGTGGAGGTTTTGAGCTGTTA

TCCAAGGAGCTCAGATGTTTGTCTTGGAAAAGATGTCCCTTAAAATGTGTACCATCGAAT

TTTCCAGCTGAGAATCTTGTAGTTCTAGATATGCATGAGAGTGCTATCCAAGAATTTCAA

TTGAATTTGCAGTGTTGTAGAAGTTTGAAGAAGTTGGATCTCTCTTATTGCAAGCAACTC

AGAAGCACTCCAGACTTCACTGGTTCACGAAGTCTTGAGAATTTGCTGCTTGGTGGTTGC

TCAAGTCTGATGGAGATCCATCCATCAATAGGAAATCTGAACAGACTAATTAAACTATAT

ATGCATGATTGCGAAAAACTTAGGGATCTTCCAAGCAGCATATGCCAGCTAATATCCGTT

GATTACTTGGACATTAATTCCTGCTCATCTATAAAAACTCTGCCAGATAACATTGGAGAT

ATGAAAAGTCTAAGACATCTTGATGCATGTCGTACGGGTATAAAACAATTGCCTAAATCT

GTTGAAATGCTAAAAAATCTTGTAGCTTTGATTGTGGGAGGTCGAAAGTTTGAGGCCAAA

AGGAGTATTTCTGGAAGAGGAGTCCATCAGATACAATATTCCCTGTCAACTTTTGTATCC

AGATTGAGCCTTACATATTTGTCTGAGGCTGATATTCCTAGGAATATTGGGAGCTTATCC

TCCTTGGTGATTTTAGATTTGAGTGGCAACAGTTTCTATTGTCTACCCTTTGATCTTTCT

ATGTTACGATTATTGGAGGAGTTGTATTTGAATGACTGTGAGAATCTTCAAACACTCCCA

TCAGTATCAAATTTAGAGAAGCTTGAAAGAATTGAGCTTAAAAATTGCCAAAAATTGGTC

AAGATTACGGAGTTGGACAACCTCCCTTCTATACGGTGGATCAACATGATGAATTGTAGT

TCTATGCAGAATCCATTCAATGAAGGCTTATTTAGTGCACCTGCTCTATATGCATCTAGA

AATGATCGAAATATGGTTAGTCTCTCTCTCTCTCTCTCTCACACACACACACACTCATGT

TAA

>NTK326_mRNA_16499_cds NTK326_mRNA_16499 gene_9365|id=AT5G17680.1

ATGATGCAGAAGAGCTCTTCTTCTTTCTCCTCTGTTCAGACGTTTCGGTGGAGTTATGAT

GTTTTCTTAAGTTTTAGAGGTGAAGATGTACGCAAAACATTTGTTGACCATCTCTACGTT

GCTCTGCAACAAAGGGGTATTCATACCTTCAATGATGATGAGAAGTTAGAGAGAGGCAAG

TCCATTTCACCTGATCTTATAAGAGCAATTGAAGAAGCGCGCATAGCTTTGATCATCTTC

TCCAAAAACTATGCTGATTCGAAATGGTGTTTAGATGAATTAATGAAGATCATGGAATGC

AACAAACAAAAAGGACAAATTGTCCTTCCGGTCTTCTACGACGTAGATCCATCAACAGTG

AGGAAACAAAAGTCCAGCTTTGGAGAAGCATTTAGCAATCACGAAATCAGCTGTTTCAAG

GATAACAAGGTTGAAAAATGGAGGGCAGCACTGGAGGAAGCAGCTAATTTATCTGGCTGG

GATTTGCCAAATACTGCCAATGTGCATGAAGCTAAAGTCATAAAGCAAATTGTGGAAGAT

ATAATGGCTAAATTGGGTGGTCGGAGACAAGCCAGCAATGCTGAAAATCTTGTTGGAATG

GAGTCACACATGCAAAAAGTATATAAAATGCTTCAAGTCGGGTATGACGGAGTTCACTTC

GTTGGAATATTGGGAATGAGTGGAGTGGGGAAGACAACTCTAGCAAGAGTTATTTATGAT

AATATTCGGATTCAATTTGAGGGTGCATGTTTTCTGCATGAAGTTGGAGACCGTTCAGCA

AAACAAGGCTTAGAGCGATTGCAAGAGATACTTCTTTCTGAGATCCTTGTCATAAAAGAT

GTAAGGATCAGTGATTCATTTGAAGGAGCTAATATGCAAAAACAAAGACTACCGCGCAAA

AAGGTTCTTCTTGTTCTTGATGATGTAGATCGCAGAGATCAGTTAGATGTTTTAGCTGGG

GAACGTGAATGGTTTGGTCCTGGAAGTAGAATCATCGTAACAACTAAAGACAAACACTTA

CTTGTTAAGCATAGGGTGGAAAAGATATACAGAATGAGAACATTAAGTGAATATGAAAGT

TTACAGCTCTTTAAACGACATGCTTTTAAGAAGAGCTACCCAACTAAGGAATTTGACCAT

CTTTCAGTTCAAGTGATAAAGCATACCGCAGGACTCCCCTTGGCTCTGAAAGTCCTGGGC

AGTTTCCTGTATGGAAGAGATTTGGTTGAATGGACAAGTGAAGTGGAATGGTTGAACACA

ATCCCAGGAAATGAAATTTTGAAGAAACTCGAACGAAGTTTCACTGGACTCAACAGTATC

GAGCAAAAGATATTCTTAGACATTGCGTGTTTCTTTACAGGGAAGAAGAAAGATTCAGTG

ACCAGAATACTCGAGAGTTTTAATTTTAGACCTGCCATTGGCATAAAAGTTCTCATGGAG

AAATCTTTGATTACCATTTCAAAAGGTCGAATTTTAATGCACCAATTGGTACAAGAAATG

GGCTGGCACATTGTTCGTCGAGAAGCTTATGATGATCCAAGAATATATAGTAGGTTGTGG

AAGCGCGAAGATATTTCTCCAGTACTTGAAAGAAATTTCGGCACTGAAAAGATCGAAGGC

ATATCGTTGAACTTGACTAGTGAAGAAGAAGTGAATGTTAGTCACAAGGCCTTCACGCAG

ATGACAAGACTAAGGTTTCTCAAATTTCGGAATGTATATGTTTGCCAGGGTCCTGATTTT

CTTTCTGATGAGTTGAGGTGGCTCGATTGGCACGAATATCCTTCAAAAAGTCTGCCAATT

AGCTTTCAGGGAGAACAACTTGTTGCTTTGAACTTGAAAAATAGTTGCATCATACAACTT

TGGAATACCTCTAAGGTTCTAGATAAATTGAAGTACATAAACCTTAGCCATTCACAGAAG

CTAATAAGGACCCCAGATTTTTCGGGTACCCCTAATCTTGAAAGGTTGGTTCTTGAGGAG

TGCACGAGTTTGGTAGAAATCAATTTTTCTGTTAGCGATCTTGGAAAGCTAGTCTTGCTG

AATCTGAAGAACTGCAGAAATTTAAAGACCCTACCAAAGAGTATTCGATTGGAAAATCTT

GAGGTTCTCATTCTTTCAGGCTGCTCCAAGCTAAAAGTATTCCCAGAAATAGAAGAGACA

ATGAATCATTTAGCAAAACTGTGTTTGGAGGCGACTGCTTTGAGTGAACTGCCCGCATCA

GTTGAGAAACTATCAGGAGTTACTGTAATAAATCTAAGTTACTGCAAGCATCTTGAGAGT

CTTCCTAGCAGTATTTTTAGGTTGAAATGTCTGAAAACACTTGATGTGTCTGGTTGCTCA

AAACTAAAAAATTTACCAGAGGACTTGGGACTTTTAGTTGGCAGCCATCCGTGA

>NTK326_mRNA_28250_cds NTK326_mRNA_28250 gene_16297|id=AT5G17680.1

ATGGAATGCAAGGATCAATGCGGACAAACTGTCATGCCAGTCTTCTATGATGTGGATCCA

TCACATGTTCGGAACCAGAGGGAGAGCTTTGCTGAAGCCTTTAACAAACATGAAACAAAC

TATAAGGATGATGATGAAGGAATGGAGAAGCTCCAAAGATGGAGGAACGCTCTAACTGCT

GCCGCAGATCTAAAAGGATATGATATTCGTGACGGGATTGAAGCAGAGAATATTCAGCAG

ATTGTCGACCAAATTTCCAAATTGTGTAATAGTGCTAATTTGTCTTCTTTGCGAGAAGTT

GTGGGAATAGATACTCATCTGGAGAAATTAAAGTCTCTACTTAAGGTAGGAATCAATGAT

GTTCGAATCATATTGGGGATCTGGGGCATGGGCGGTCTAGGGAAGACGACAATAGCAAGA

GCCATTTTTGGCATTTTATCTCATCAATTTGAAGCTGCTTGTTTCCTTGCGGATATTAAA

GAAAATGAAAAACTACATTCTTTGCAAAACACCCTTCTCTCTGAATTGTTAAGAAAAAAA

GATGATTATGTCAATAATAAGCATGATGGGAAGCAAATGATTCCAGACAGACTTCGCTTT

AAGAAGGTGCTAATTGTGCTCGATGATATAGATCATAAAGACCATTTAGATTATTTAGCG

GGTGATCTTGGTTGGTTTGGTAATGGCAGTAGGGTTGTTGTAACAACTAGAGACAAGCAT

TTGATAGGGAAGGATGATATAATATACGAAGTGACTGCACTACCTGATCATGAATCCATT

CGATTGTTCTATCAGCATGCTTTCAAAAAAGAGGTTCCAGATGAGTGTTTTAAGGAGCTT

TCATTGAAGGTAGTAAATCATGCTAAAGGCCTTCCTTTAGCCCTCAAAGTATGGGGTTCA

ATGCTGCATAACCTACGAATAACTGAATGGAAAAGTGCTATAGAGCACATGAAAAATAAT

TCTAATTCTGGAATTGTTGATAAGCTCAAAATTAGTTATGATGGATTAGAGCTCATCCAA

CAAGAGATGTTTCTAGATATAGCATGCTTCTTGAGAGGGAAATATAAAGATTACGTCATG

CAAATTCTTGAGAGTTGTCATTCTGGAGTTGAATACGGATTGCGTGTCTTAATTGACAAA

TCTCTTATATTTATCTCTGAAAAATGTCAGATTCAAATGCACGACTTAATACAAGAAATG

GGTAAATATATAGTGAACTTGCCAAAGAATCCGGGAGAGCGCAGCAGACTATGGCTCGTC

AAGGATTTCAAAGAAGTGATGAGCAACAGTACAGGGACCATGGCAATGGAAGCAATCTTT

CTTCCTTATCTCAATTCTGGTACATCACGCTTTAGCAAAAATATGAAAAGGCTTAGGATA

TTTAACATAGAGAGGTCGTTGAACTGTGATGGTTCCATTAAGTATCTGCCCAACAGCTTG

CGTTGGTTTGTGTGGGAGAACTTTCCTTGGAAGTCATTGCCATCTACATTTGAACCCAAA

ATGCTTGTTCACCTTGAACTCTGGGGTAGTTCACTGCATTATTTATGGATGGAAACAAAG

CATTTGCCATCTCTACGGAGGATAGATCTCAGCTCCTCTAGAAGACTGAGGCGAACACCA

GATTTCACGGGGATGCCAAATTTGGAGTATTTGAATATGTTATATTGCAGAAATCTTGAA

GAGGTTCACCATTCCCTGAGATGTTGCAGCAAACTCATTCGGTTAAATTTGAATAATTGT

AAAAGCCTTAAGAGGTTTCCATGTGTTAACGTGGAATCTCTTGAATATCTGAGTTTAGAA

TATTGCTCAAGTTTAGAGAAATTTCCAGAAATCCACGGGAGAATGAAGCCGGAGATACAG

ATTCACATGCAAGGCTCTGGGATAAGGGAACTACCATCATCTATTACTCAGTACCAAACT

CATATTACCAAGCTAGATTTGAGAGGTATGGAAAAACTTGTAGCTCTTCCAAGCAGCATC

TGTAGGTTGAAAAGTTTGGTTAGTCTGAGTGTGTCGGGTTGCTTCAAACTTGAAAGCTTG

CCAGAAGAGGTAGGGGATTTAGAAAACTTGGAGGAGCTTGATGCCAGTTGTACTCTAATT

TCACGACCTCCGTCTTCCATCGTTCGCTTGAGCAAACTTAAAATCTTTGATTTTGGAAGC

TCCAAAGATAGAGTGCACTTTGAGCTCCCTCCGGTGGCAGAAGGATTTCGCTCATTGGAA

ACTTTGAGTCTTAGAAACTGCAATCTAATAGATGGAGGACTTCCGGAAGATATGGGATCC

CTATCCTCTTTGAAAAAGTTGTATCTTAGTGGAAATAATTTTGAGCATTTGCCTCGAAGC

ATAGCCCAACTTGGTGCCCTTCGAATCTTGGAATTAAGAAATTGCAAGAGGCTTACACAG

CTGCCAGAATTTACGGGGATGCTAAATTTGGAGTATTTGGATCTGGAGGGATGTAGTTAT

CTTGAAGAGGTTCACCATTCCCTGGGGTGTTGCAAAAAACTCATTCGGTTAAATTTGAGT

TTTTGTAGTAGCCTTATAAGGTTTCCATGTGTTAACATGGAATCTCTTAAATATCTGAGT

GTAGGAGAGTGCTCTCGCTTAGAGAAATTCCCAGATATCCATGGGAGAATGAAGCCGGAG

ATACAGATTCACATGAAACGCTCTGGGATAAGGGAACTACCATCATCTATTTCTCAGTAC

CAAACTCATATTACCGAGCTAGATTTGAGAAGGATGGATAACCTAGATTCCAGAGGCTAC

TAG

>NTTN90_mRNA_69999_cds mRNA_69999 gene_39449|id=AT5G17680.1:evalue=0.0:annot='disease resistance protein (TIR-NBS-LRR class), putative';id=Solyc01g008800.1.1:evalue=0.0:annot='Tir-nbs-lrr, resistance protein'

ATGGCATCATCTTCTGCTTCTGGTACTTCACAGTTTCCTCGATGGAACTACGATGTCTTT

CTAAGTTTTAGAGGTGAAGATACTCGGAAAACATTTACGAGTCACCTGTACGAAATCTTG

GATATCAGGGGAATAAAAACCTTTCAAGATGATAAAAGGCTAGAGCATGGCGCATCCATT

TCGGATGAACTCTGTAAAGCTATCGAAGAGTCTCAATGTGCAGTCATCATTTTCTCAAAG

AATTATGCAACATCGAGGTGGTGCTTGAATGAACTAGTGAAGATCATGGATTGCAAGACT

CAATTTGGACAAACTGTCATACCGGTCTTCTATGATGTGGATCCATCACATGTTCGGAAC

CAGAGGGAGAGCTTTGCCGAAGCATTTTCCAAACATGAAACAAAGTATAAGGATGATATC

GAAGGAATGCAAAGATGGAGGATTGCTTTAACTGCAGCGGCCAATCTCAAAGGCTGTGAT

ATTCGTGACAAGACTGAATCAGACTGTATTCGGCAGATTATTGACCAAATCTCGTCCAAA

TTATGCAAGATTTCTTTATCTTATTTGCAAAACCTTGTTGGAATAGATACTTATTTAAAG

GAAATAGAATCCTTACTAGGGATAGGAATCAATGATGTTCGGATTATGGGGATCTGGGGC

ATGGGAGGAGTCGGTAAGACGACAATAGCAAGAGCTATGTTTGATACTCTCTTAGTGAGA

AGGGATAGTTCCTATCAATTTGATGGTGCTTGTTTCCTTGAGAATATTAAAGAATACAAA

GGTAGAATACATTCTTTGCAAAATACCCTTCTCGCTAAACTGTTAAGGGAAAAAGCTGAG

TACAATAATAAGGAGGACGGAAAGCACCAAATGGCCAGTAGACTTCGTTCTAAGAAGGTC

CTAATTGTGCTTGATGACATAGATGATAAAGATCATTATTTGGAGTATTTAGCAGATCTT

GATTGGTTTGATAATGGAAGTAGAATTATTGTAACAACTAGAGACAAGCATTTGATAGAG

AAGAATAATGTAATATATGAAGTGAATGCACTACCTGATCATGAATCTATTCAATTGTTC

AATCAGTATGCTTTCAATAAAGAGGTTCCAAATGAGTATTTTAAGAAGCTTTCATTGGAG

GTTGTAAATCATGCTAAAGGCCTTCCTTTAGCCCTAAGAGTGTTGGGTTCTTCCTTACGT

AATAGGGGTATAACTGTGTGGAGAAGTGCTATAGAGCAAATGAAAAATAATCCTAATTCA

AAAATTGTTGCAAATCTCAAAATTAGTTATGATGGATTAGAGCCCATACAACAAAAGATG

TTCCTAGATATATCATGCTTCTTCCGAGGGTACGGAAAAGGTGCCATCATGCAAGTTCTT

AAGAGTTGTGATTGTGGAGCTGAATACGGATTGGATGTCCTAATTGAAAGATCTCTTGTG

TTCATCACTGAATACGGTAAAATTCAAATGCATGACTTAATACAAGAAATGGGTAGATAT

ATAGTGAACTTGCAAAAGGATTTGGGAGAATGCAGCAGACTATGGCTCGCCAAGGATTTT

GAAGAAGTGATGATCAACAATACGGGGACCATGGCAGTGGAAGCAATTTGGCTTCATTCT

AATGCACTACGCTTTAACAATGAGGCCATGAAAAATATGAAAAGGCTTAGGATATTACAC

ATCGAGAGTTGGCCCTATGATGGTTCCAATATTAGCCATGATGGCTCCATTGAGTATCTG

CCCAACAACTTGCGTTGGTTTTTCTGGGATTACTATCCTTGTGAATCATTGCCATCTACA

TTTGAACCCAAAATGCTTGTTCACCTTAAACTCTCTGGCAATTCACTGCGTTATTTCTGG

ATGGAAACAAAGCATTTGCCGTCTCTACGGAGGATAGATCTCAGCGGCTCTGAAAGCCTG

ATGCGAACACCCGATTTCACGGGGATGTCAAATTTGGAGTATTTGAGCTTGGCATGTTGT

AGTAATCTTGAAGAGGTTCACCATTCCCTGCGATGTTGCAGCAAACTCATTCGGTTAGAT

TTGAGTCGTTGTGGTCGCCTTAAGATGTTTCCATGTGTTAATGTGGAATCTCTTGAATAT

CTGAGTTTAGAAAAGTGCTCTCGGTTAGAGAAATTTTCAGAAATCCAAGGGAGAATGAAG

CCAGAGGCACAGATTCACATGCAAGGCTCTGGGTTAAGGGAACTACCATCCTCATATTTT

CAGTACCAAACTCATATTACCGAGCTAGATTTGAGTTATATGAAAAACCTTGTAGCTCTT

CCAAGCAGCATCTGTAACTTGCCAAATTTGGAGTATTTGCATTTAAATAATTGTAGTAAT

CTTGAAGAGGTTCACCATTCCCTGAGATGTTGCAGCAAACTCATTCAGTTAAATTTGTAT

TATTGTAAAAGCCTTAAGAGGTTTCCATGTGTTAATGTGGAATCTCTTGAATATCTGGAA

TTAGAACATTGCTCAAGTTTAGAGAAATTTCCAGAAATCCACGGGAGAATGAAGCCGGAG

ATACAAATTCACATGAAATACTCTGGGTTAAGGGAACTACCATCCTCTTATTTTCAGTAC

AAAACTCATATTACCAAGCTAGATTTGAGCGATATGGAAAACCTTGTAGCTCTTCCAAGC

AGCATCTGTAGGTTGAAAAGTTTGGTTAGTCTGAGTGTGTCATGTTGCTTAAAACTGGAA

AGCTTGCCAGAAGAAATAGGGGATTTAGACAACTTGGAGGAGCTTGATGCCAGCTATACT

CTAATTTCACGACCTCCGTCTTCCATCGTACGCTTGAACAAACTTAAAATCTTGAGCTTT

AGCTGCTTCAGAGACGATGGAGTGCACTTTGAGTTTCCTCCTGTGGCGGAAGGATTACGC

TCATTGGAAACTTTGAGTCTCATTTACTGCAATCTAATAGATGGAGGACTTCCGGAAGAT

ATTGGATCCTTATCCTCTTTGAAAGAGTTGGATCTCAGTGGAAATAATTTTGAGCATTTG

CCTCGAAGCATAGCCCAACTAGGTGCTCTTCGAATCTTGGACTTAAGAAATGGCAAGAGG

CTTACACAGCTGCCAGAACTTCCACCAGAATTAAATGAATTGCGTGTAGATTGTCATATG

GCTCTGAAATTTATCCATGATTTAGTAACAAAGAGAAAGAAACTACAGAGGGTGATATTC

ACACCACTGTATGGTAAGGATGATGCACACAATGATTCTATATATAATTTGTTTACCCAT

GCCCTGTTTCAGAATATCTCTTCCTTGAGGCATGACATATCTGCTTCAGATTCCTTGTCC

GAAAATGTGTTTACCATTGTGCATCCTTGGAAGAAGATCCCAAGTTGGTTCCACCATAAG

GGAAGGGATAGAAGTGTATCAGTCAATTTGCCTGGAAATTGGTATATACCTGATAAATTC

TTGGGATTTGCTGTATGTTACAAAGGCATCTTAACTGACACCACAGCTCAATTGATTCCC

GTATGTGATGATGGGATGTCGTGGATGACCCAGAAACTTGCCTTATCCAACCATTCAGAA

TCTGATTCGGTATATTATATACATTTTTTCTTTGTACCTTTTGCTGTCTTATGGGATACA

TCTAAGGCAAATGGAAAAACACCAAATGACTATGGGCATGTCAGGCTATCTTTTTCCGGA

GAAATGAAGAAGTTTGGATTTCGTTTGTTGTATAAAGATGAACCAACAGAACATCGCGTT

GGGATAAGGAGCAGATATGACAATGGTGAACAACATGACACCGTGACTGATGAAGCCAGC

TGCTGTCGCATACTCTAA

>NTBX_mRNA_108285_cds NTBX_mRNA_108285 gene_64864|id=AT4G16890.1

ATGGCATCATCTTGTGCTTCTGAGAGTACTTCACAGTTTCCTCGGTGGAACTACAAAGTC

TTTCTAAGTTTTAGAGGTGAAGATACTCGAAGAACATTTACAGGTCACCTCTTCAAAGGC

TTGGAAAACAGTGGAATATTTACGTTTCAAGATGATAAAAGGCTAGAGCATGGCGCATCA

ATATCAGATGAACTCTTGAAAGCTATCGAACAGTCTCAAGTTGCCCTCGTCGTTTTCTCA

AAGAATTATGCAACATCGAGGTGGTGCTTAGATGAGCTAGTGAAGATCATGGAATGCAAG

GATCAATGTGGACAGACTGTCATACCAGTCTTCTATGGTGTGGATCCATCACATGTTCGG

AAACAGAGGGAGAGCTTTGCTGAAGCCTTTGACAGACATGAAACAAGCTATAAGGATGAT

GATGAAGGAATGGAAAAGCTCCAAAGATGGAGGAATGCTCTAACTGCTGCCGCAAATCTA

AAAGGATATGATGTCCGTGACGGGATTGAAGCAGAGAATATTCAGCAGATTGTCGACCAA

ATTTCCAAATTGTGCAATAGTGCTACTTTGTCTTCTTTGAGCGATGTTGTGGGAATAGAT

ACTCATCTGGAGAAATTAAAGTCCCTACTTAAGGTAGGAATCAATGATGTTAGGATCATA

TTGGGGATTTGGGGCATGGGCGGTCTAGGCAAGATGACAATAGCAAGAGCCATTTTTGAC

ACTTTATCTCATCAATTTGAAGCTGCTTGTTTCCTTGCGGATATTAAAGAAAATGAAAAA

CTGCATTCCTTGCAAAACACCCTTCTCTCTGAATTGCTAAGAAAGAAAGATGATTACGTC

AATAATAAGCTTGATGGGAAGCAGATGATTCCGGACAAACTTTGCTCTAAGAAGGTGCTA

ATTGTGCTTGATGATATAGATCATAAAGATCATTTAGATTATTTAGCAGGTGATATTGGT

TGGTTTGGTAATGGCAGTAGGATTGTTGTAACAACTAGAGACAAGCATTTGATAGGGAAG

GATGATGCAATATATGAAGTGAGTGCACTACCTGATCATGAATCCATTCAATTGTTCTAT

CAGCATGCTTTCAAAAGAGAGGTTCCAGATGAGTGCTTTAAAGAGCTTTCATTGAAGATT

CAAATGCACGACTTAATACAAGAAATGGGTAAATATATAGTGAACTTGCAAAAGAATCCG

GGAGAACGCAGCAGACTATGGCTCGTCAAGGATTTCAAAGAAGTGATCAACAACAATACA

GGAACCATGGCAATGGAAGCAATCTTTCTTCCTTATTTCAATTCGGGTATATTACGTTTT

AGCAAAAAGGCCATGAAAAATATGAAAAGGCTTAGGATATTAAACATAGAGAGGTCGTTG

ACCTATGATGGTTCCATTGAGTATCTGCCCAACAACTTGCGTTGGTTTGTCTTGTATGGC

CATCCTTGTGAGTCACTGCCATCTACATTTGAACCCAAAATGCTTGTTCACCTTGAACTC

TGGGGTAGTTCACTGCATTATTTATGGATGGAAACAAAGCATTTGCCGTCTCTACGGAGG

ATAGATCTCAGCTCCTCTAGAAGCCTGATGCGAACACCAGATTTCACGGGGATGCCAAAT

TTGGAGTATTTGAATATGTTATATTGTAGTAATCTTGAAGAGGTTCACCATTCCCTGAGA

TGTTGCAACAAACTCATTCGGTTAAATTTGAATTATTGTAAAAGCCTTAAGAGGTTTCCA

TGTGTTAACGTGGAATCTCTTGAATATCTGAGTTTAGAATATTGCTCTAGGTTAGAGAAA

TTTCCAGAAATCCACGGGAGAATGAAGCCGGAGATACAGATTCACATGAAACACTCTGGG

ATAAGGGAACTACCATCATCTATTACTCAGTACCAAACTCATATTACCAAGCTAGATTTG

AGCGGTATGGAAAAACTTGTAGCTCTTCCAAGCAGCATCTGTAGGTTGAAAAGTTTGGTT

AGTCTGAGTGTGTCGGGTTGCGTCAAACTTGAAAGCTTGCCAGAAGAGATAGGGGATTTA

GAAAACTTGGAGGAGCTTGATGCCAGCTGTACCCTAATTTCACGACCTCCATCTTCCATC

GTACGCTTAAGCAAACTTAAAATCTTTGATTTTGGAAGCTCCAAAGATAGAGTGCTCTTT

GAGTTCCCTCCGGTGGCAGAAGGATTTCGCTCATTGGAAACTTTGAGTCTCAGAAACTGC

AATCTAATAGATGGAGGACTTCCGGAAGAGATTGCATCCTTATCCTCTTTGAAAAAGTTG

TATCTCAGTGGAAATAATTTTGAGCATTTGCCTCGAGGCATAGCCCAACTTGGTGCTCTT

CGAATCTTGGACTTAAGAAATTGCAAGAGGCTTACACAATTGCCAGAATTCACGGGGATG

CCAAATTTGGAGTATTTGGATCTGGAGGGATGTAGTTATCTTGAAGAGGTTCACCATTCC

CTGGGGTGTTGCAAAAAACTCATTCGGTTAAATTTGAGTTTTTGTAGTCGCCTTATAAGG

TTTCCATGTGTTAATGTGGAATCTCTTAAATATCTGAATGTAGGAGAGTGCTCTCGGTTA

GGGAAATTTCCAGAAATCCACGGGAGATTGAAGCCGGAGATACAGATTCACATGAAACGC

TCTGGGATAAGGGAACTACCATCATCTATTTCTCGGTACCAAACTCATATTACCGAGCTA

GATTTGAGAAGTATGGATAACCTTTTAGATCTTCCAAGCAGATCGGTAGGTTGA

>NTK326_mRNA_109105_cds NTK326_mRNA_109105 gene_64484|id=AT5G17680.1

ATGGCATCATCTTTTGCTTCTGTGAGTACTTCACAGTTTTCTCGGTGGAACTACAAAGTC

TTTCTAAGTTTTAGAGGTGAAGATACTCGAAGAACATTTACAGGTCACCTCTTCAAAGGC

TTGGAAAACAGTGGAATATTCACGTTTCATGATGATAAAAGGCTAGAGCATGGGGCATCA

ATAACAGATGAACTCTTGAAAGCTATCGAACAGTCTCAAGTCGCCCTCGTCGTTTTCTCA

AAGAATTATGCAACATCGAGGTACTGTTTAGATGAGCTAGTAAAGATCATGGAATGCAAG

GATCAATGTGGACAGATTGTCATACCAGTCTTCTATGATGTGGATCCATCACATGTTCGG

AAACAGAAGGAGAGCTTTGCTGAAGCCTTTGACAAACATGAAACAAGCTATAAGGATGAT

GATGAAGGAATGCAGAATCTCCAAAGATGGAGGAATGCTCTAACTGCTGCCGCAAATCTA

AAAGGATACGATGTCCGTGACGGGATTGAAGCAGAGAATATTCAGCAAATTGTCGATCAA

ATTTCCAAATTGTGCAATAATGCTACTTTGTCTTCTTTGCAAGATGTTGTGGGAATAGAT

ACTCATCTCGAGAAATTAAAGTCCCTACTTAAGGTAGGAATCAATGATGTTCGAATCATA

TTGGGGATCTGGGGCATGGGCGGTTTAGGGAAGACGACAATAGCAAGAGCCATTTTTGAC

ACTATATCTCATCAATTTGAAGTTGCTTGTTTCCTTGCAGATATTAAAGAAAATGAAAAT

TTACATTCTTTGCAAAACACCCTTCTCTCTGAATTGTTAAGAAGAAAAGATGATTACGTC

AATAATAAGCTTGATGGGAAGCGGATGATTCCAAATAGACTTTGCTCTAAGAAGGTGCTA

ATTGTGCTTGATGATATAGATCACAAAGATCATTTAGAGTATTTAGCAGGTGATATTGGT

TGGTTTGGTAATGGCAGTAGGGTTGTTATAACAACTAGAGATAAGCATTTGATAGGGAAG

GATGATGCAATATATGAAGTGACTGCACTACCTGATCATGAATCAATTCAATTGTTCTAT

CAACATGCTTTCAGAAAAAAAGATCCAGATGAGTGTTTTAAGGAGCTCTCATTGGAGGTA

GTAAATTATGCTAAAGGCCTTCCTTTAGCGCTCAAAGTGTGGGGTTCTTTGCTGCATAAC

CTAGACTTAACTGAATGGAAAAGTGCTATAGAGCACATGAAAATTAATTCTAATTCAGAA

ATTGTTGAAAAGCTCAAAATCAGTTATGATGGATTGGAGCCCATCCATCAAGAGATGTTT

CTAGATATAACATGCTTTTTACGAGGAAAATATAAAGATTACGCCATGCAAATTCTTAAG

AGTTGTCATGCTGGAGCTGAATATGGATTGCGTGTCTTAATTGACAAATCTCTTGTGTCC

ATCTCTGAAAATGATCATATTCAAATGCATGACCTAATGCAAGATATGGGTAAATATATA

GTGCACTTGCAAAAGAATCCGGGAGAACGCAGCAGACTATGGCTCGCCGAGGATTTCGAA

GAAGTGATGACCAATAATGCAGGGACCATGGCAGTGGAAGCAATCTGGGTTACTTATTTT

GGTAGACTAGGCTTTAGCAAAGAGGCCATGAAAAATATGAAAAGACTTCGAATATTAAAC

ATAGGGATGTGGCAGACGTGTGATGGTTCCCTTGAGTGTCTGCCCAATAACTTGCGTTGG

TTTAAGTGGTTTTGCTATCCTTGTGAGTCATTGCCATCAACATTTGAACCCAAAATGCTT

GTTCACCTTCAACTCAAACACAATTCACTGCGTCATTTATGGACTGAAACAAAGCATTTG

CCGTTTCTACGGACACTAGATCTGAGCCACTCTAAAAGCCTAATGCGAACACCAGATTTC

ACGGGGATGCCAAATTTGAAGTATTTGGATCTGTCTTGTTGTCATAATCTTGAAGAGGTT

CACCATTCCCTGGGATGTTGCAGCAAACTCATTCAGTTACATTTGTATTATTGTAAAAGC

CTTAAGAGGTTTCCATGTGTTAACGTGGAATCTCTTGAATATCTGGGTTTAGAAGATTGC

TCTAGTTTAGAGAAATTTCCAGAAATCCACGGGATAAGGGAACTACCATTTTCTATCTTT

CAGTACCAAACTCATATTAGCGAGCTAGATTTGTTCGCTATGGAAAACCTTGTAGCTCTT

CCAAGCAGCATCTGTAGGTTGAAAAGTTTGGTTAGTCTAAATGTGTCGGAGTGCTCAAAA

CTGGAAAGCTTGCCAGAAGAGATAGGGGATTTAGACAACTTGGAGGAGCTTGATGCTAGA

TGGACTCTAATTTCACGACCTCCGTCTTCCATCGTACGCTTGAAAAAACTCAAAATCTTG

ATGTTTGGACTCTTCAAGCATGGAGGAGTGTACTTTGAGTTCCCTCCGGTGGCTGAAGGA

TTACGCTCATTGGAACATCTGGATCTCAGTTACTGCAATCTAATAGATGGAGGACTTCCG

GAAGACATTGGATCCTTATCCTCTTTGAAAAAGTTGGATCTCAGTAGAAATAATTTTGAG

CATTTGCCTCGAAGCATCGCCCAACTTGGTGCTCTTCAATCCTTAGACTTAATAGAGTGC

AAGAGTCTTACACAGCTGCCAGGACTTCCCCCTGAATTAAATGATTTGCGTGTAGATTGT

TATATGGCTCTGAAAAGTATCCATGATTTAGTAACAAAGAGAAAGAAACTACAGTGGGTA

ATATTCCACCGTGATATGCCACTTGATGAGGATGATACATACAATGATCCTATATATAAT

TTGTTTGCACATGCCCTGTTTCAGAATATCTCTTCCTTGAGGCATGATATCTCTGCTTCA

AATTCCTTGTCCGAAAATGTATTTACCTTTCTGCATCCTACGAAGAAGATCCCAAGTTGG

TTCTACCATTCGGATAGTGGTGTATCAGTCAATTTGCTCGAAAATTGGTATATACCTGAT

AAATTCTTGGGATTTGCTGTATGTTACTCTGGCAGATTAGTAGACACCACAGTCCACTTG

ATTCCCGTATGTGATGACGGGATGTCGTCCATGACCCAGAAACTTGCATTATTCAACCAT

TCAGAATGGGCTACAGAATCTAACATTCCATTTTCTACTCTACATTTTTTCTTTGTACCT

TTTGCTGTGTTATGGGATACATCTAAGTCAAATGGAAAAACACCAAATGACTATGGTATT

ATTAGGCTATCTTTTTCTGGAGAAGTGGAGGAGTATGGACTTCGTTTGTTGTATAAAGAA

GAAGCTGAGGTTGAGGCCTTGTCACAAATGAGGGAAAATAACAATGAACATTCCACTGGG

ATAAGGACGACCCGATATAACAATAGTGAACACCACTTCATGATCAATGAAGCCAGTTGC

TCCTCGGTTCAAATGGGAACATTGCATATGAAGGATTGGAAGTCCCTTCAAGATCAAATG

ATGGAGAAGGTCAAAGAGGTTTTATTTCTGTACTTAAATGAATAA

>NTTN90_mRNA_34378_cds mRNA_34378 gene_19271|id=AT5G17680.1:evalue=9e-177:annot='disease resistance protein (TIR-NBS-LRR class), putative';id=Solyc05g007850.1.1:evalue=0.0:annot='Tir-nbs-lrr, resistance protein'

ATGGCATCTTCTTCTTCTGCTAGATGGAGCTATGATGTTTTCCTAAGTTTTAGAGGTGAA

GATACTCGGAAAACATTTACAAGTCACTTATACGAAGTCTTGAATGATAGGGGAATAAAA

ACCTTTCAAGATGATAAAAGGCTAGAGTACGGTGCGACCATCTCAGAAGAACTCTGTAAA

GCTATAGAAGAGTCTCAATTTTCCATCGTCATTTTCTCAAAGAATTATACAACATCGAGG

TGGTGTATGAATGAACTAGTGAAGATCATGGAATGCAAGACTCAATTTGGACAAATTGTT

ATACCGATATTCTATGATGTGGATCCATCACATGTTCGGAACCAAAAGGAGAGCTTTGCA

AAAGCCTTTGAAGAACATGTAACAAAGTATAAGGATGATGTTGAGGGAATACAAAGATGG

AGGATTGCTTTAACTGCAGCGGCCAATCTCAAAGGCTCATGTGATAATCGTGACAAGACT

GATGCAGAATGTATTCGGCATATTGTTGGCCAAATCTCATCCAAATTATGCAAGATTTCT

TTATCTTATTTGCAAAACATTGTTGGAATAGATACTCATTTAGAGAAAATAGAATCCTTA

CTAGAGATAGGAATCAATGATGTTCGGATTATGGGGATGTGGGGAATGGGGGGAGTCGGT

AAAACGACAATAGCAAGAGCTATGTTTGATACTCTTTTAGGAAGAAGGGATAGTTCCTAT

CAATTTGATGGTGCTTGTTTCCTTAAAGATATTAAAGAAAACAAACATAGAATGCATTCT

CTGCAAAATATCCTTCTCTCTAATCTTTTAAGGGAAAAAGCTAATTACAAAAATGAGGAG

GACGGAAAGCACCAAATGGCTAGTAGACTGCGTTCTAAGAAGGTCCTAATTGTGCTTGAT

GACATAGATGATAAAGATCATTATTTGGAGTATTTAGCAGGTGATCTTGATTGGTTTGGT

AATGGCAGTAGAATTATTGTAACAACTAGAGACAAGCATTTGATAGGGAAGAATGATGTA

ATATATGAAGTGACTGCACTACCTGATCATGAATCCATTCAATTGTTCTATCAGCATGCT

TTCAAAAAAGAAGATCCAGATGAGTGTTTTAAGGAGCTCTCATTGGAGGTAGTAAATTAT

ACTAAAGGCCTTCCTTTAGCCCTCGGAGTGTTAGGTTCTTCCTTATATAATAGGGATATA

ACTGTGTGGAAAAGTGCTATAGAGCAAATGAAAAATAATCCTAATTCAAAAATTGTTGAA

AAGCTCAAAATCAGTTATGATGGATTAGAGTCCACGCAACAAGAGATTTTTCTGGATATA

GCATGCTTCTTCCGAGGGAAAAAAAAAGATGATATCATGCAAGTTCTCAAGAGTTGTCAT

TTTGGAGCCGAATATGGATTGGATGTCCTAATCGAAAAATCTCTTGTGTTCATCACAGAA

GACGGTGAAATTGAAATGCATGATTTAATACAAGAAATGGGTAGATATATAGTGAACTTG

CAAAAGGATCTGGGAAAATGCAGCAGACTATGGCTCGCCAAGGATTTTGAAGAAGTGATG

ATCAACAATACGGGGACCATGGCAATGGAAGCAATATTTCTTCCTTATTTCGATTTTGAT

ACATTACGCTTTAGCAAAAAGGCCATGGAAAATATGAAAAGGCTTAGGATATTTAACATA

GGGAGGTCGTTGGCCCATGATGGTTTCATTGAGTATTTGTCCAATAGCTTGCGTTGGTTT

GTGTGGTATTACTATCCTTTTGAGTCATTGCCATCTACATTTGAACCCAAAATGCTTGTT

CATCTTCAACTCGTATGCAGTTCGCTACATCATTTATGGACGGAAACAAAGCATTTGCTG

TCTCTACGGAGGATAAATCTCAGCTCCTCTAAAAGCCTGATGCGAACACCGGATTTCACG

GGGATACCAAATTTGGAGTATTTGAATCTGCATGGATGTACTAGTCTTGAAGAGGTTCAC

CATTCTCTGGGGCGTTGTAGAAAACTCATTCAGTTAGATTTGTATTATTGTAAAAGCCTT

AAGAGGTTTCCATGTGTTAACGTGGAATCTCTTGAATATCTGTGTTTAGAAAATTGCTCA

AGTTTAGAGAAATTTCCAGAAAGTCACGGGAGAATGAAGCCGGAGATACAGATTGACATG

GAAGGCTGTAGGATAAGCGAACTACCATCATCTATTACTCAGTACCAAACTCATATTACC

AAGCTAAATTTGAGCGGTATGGAAAAACTTGTAGCTCTTCCAAGCAGCATCTCTAGGTTG

AAAAGTTTGGTTAGTCTGAGTGTGTCGGATTGCTCAAAACTGGAAAGCTTGCCAGAAGAG

ATAGGGGATTTGGACAACTTGGAGGAGCTTGATGCCAGAGATACTCTAATTTCACGACCT

CCGTCTACCATCGTACGCTTGAACAAACTTAAAATCTTGGATTTTAGAAAGTTAAACTCC

ATCTCTGAGCACGCCATCTCTGAGCCCTCCATCATGCTCAGAAATGGAGTGCACTTTGAG

TTCCCTCCGGTGGCTGAAGGATTACGCTCATTGGAAATTCTGGATCTCGGGTACTGCAAT

CTAATAGATGGAGGACTTCCGGAAGATATTGGATCCTTATCCTCTTTGAAAGAATTGAAT

CTCCATGGAAATAATTTTGAGCATTTGCCTCGAAGCATAGCCCAACTTGGTGCTCTTCGA

TCCTTAGACTTATCATATTGCCAGAGGCTTACACAGCTGCCAGAACTTCCACCAGAATTA

GATACAATATATGTAGATTGGAGCAATGATTTCATCTGTAATTTGTTGTTTCAGAATATC

TCGTCATTGCAGCATGACATCTCTGCTTCAGATTCCTTGTCACTAAGAGTATTTACCAGT

ATGCATTATATCTGTGTGCTTATTGAGGTGAAGATCCCAAGTTGGTTCCACCATCAGGGA

ACGGATAGTAGTGTATCAGTCAATTTGCCTGAAAATTGGTATATACCTCATAAATTCTTG

GGATTTGCTCTATGTTACTCTTGCAGATTAGTTGACGCCACAGCTCAATTGATTCTATGT

GATGACGGGATGTCGTGGAAGACCCGTAAACTTGCCTTATCCAACCATTCAGAATTAGGT

TGTTTCTGTGGAGACTATGATATACATTTATTCTTTGTACCTTTTGCTGGCTTATGGGAT

ACATCTAAGGCAAATGGAAAAACACCAAATGACTATGGGATTATTAGGCTATCTTTTTCT

GGAAAAGTGAAGAAGTATGGACTTCGTTTGTTGTATAAAGAAGAACCTGAGGTTGAGGCC

TTGTTACAAATGAGGGAAAATAACAATGAACCAACAGAACATTCCATTGGGATAAGGAGG

AGCAGATCTGACAATAGTGAACACCATGACTCCGTGACCGATGGAGCCAGTTGCTGTCGC

ATACTGTAA

>NTTN90_mRNA_37001_cds mRNA_37001 gene_20691|id=AT1G27170.1:evalue=0.0:annot='transmembrane receptors';id=Solyc05g006630.2.1:evalue=0.0:annot='Tir-nbs-lrr, resistance protein'

ATGGCGGAAGAAGAAGAAGAGACGTGGTCCTTGACCTCAGGGCATAGGTTCAATTGGGAC

ATATTCCTCAGCTTCAGAGGAGAAGACACGCGCCACGGATTTACCAACAAACTCTACAAT

GAACTCGTACGGAATGGTGTACGGACATTCATCGACGACGAAGGCCTGGATCGCGGCGAA

GAGATCGCTCCGAATCTCTCGGCCGCGATCGAAGACTCGGCAGCTTCGATCGCCGTGATT

TCACAGAACTACGCCTCTTCGAAGTGGTGCCTCGAAGAACTGGTGAAGATCTCGGAATGC

AAAAGGCTATTACTGCCTGTTTTCTACGGAGTTGACCCGTCGGACGTGCGAAGACAGAAG

GGTCCGTTTGAGGAGCACTTTAGGAAGCACGAAATTGTGGTGGAAGCGGAGAAGGTTTGT

CGCTGGAGAGAAGCTATGAAAAAAGCTGGTAATATCTCCGGTTGGGATTCCATGATCTGG

GAAGAATCAGAGTTAATCCTGTCTCTAGTCAAGAAAGTTCTGGAAAAACTCGATAATACA

CCGTTAGGGGTGGCAAAATATCCAGTAGGACTTCATTCCCGTCTTAATGAGTTACGCAGG

AAATTGGACGTGAAAGGAAATGGTGTGAAAGTATTAGGACTATATGGAATGGGGGGAGTT

GGCAAGACCACTCTTGCCAAGGCTTTGTACAATCAGTTTGTTGTTTATTTCAAGAAACGT

AGCTTTATTTCAGATGTTAAAGAAATTGCAAGGCGCCAAAATGGTATGGCCACTCTTCAA

AGCAAACTCATTGGTGATCTTAAGTCAGGTGCTTCGCCAATCATAGACGATACTGCTAAA

GGTATCCGATCAATCAAGGAGTCTATGAATAATGAGCCAGTTGCTATTTTCCTAGATGAT

GTGGATAACGCAGACCAACTCCGTGTGTTGGTTGGCAGGAGAGACTGGTTTTGCCAAGGA

AGCAGAGTCGTTGTCACCACTAGAGATCAAAATGTTTTACTCCCAAGTGTTGTAAACGAA

ACTTTTGAGGTAAAGGAGCTTTCTTTGTCGGAGTCACTTACGCTATTTAGTTATCATGCA

TTCGGAAGAGAGCATCCTCCTAAGAACTTTTCGGTTCTTGCTGAAGAAGTTGTAAAACTC

AGTGGGGGATTACCTCTGGCTCTGGAAGTCTTCGGATCTTTGTTGTTCTACAAGAAAAGA

TTGAAGGAGTGGGAAGATCTCGTGCAAAAGCTGAGACAGATTCGCCCGGGTGATCTTCAA

CATGTCTTGGAAATAAGTTTTGGAGCTCTAGATGAACAAGAAAAGAGCATCTTTCTTGAT

TTAGCATGTCTTCTTCTTAATACAAGGCTTGACAGGGAAGATGCAATTGCGATATTTGAA

GGTTGTGGCTTTGGCGCTGAAAGTGCAATCACAGAGCTCACAGCAAAATCGCTTCTCAAA

ATCGTTGATGGGAATATTTTATGGATGCATGATCAGCTCAAAGACATGGGAAGGCAAATC

GTACTACGTGAGAATTTTGGAGATGCTGGTAAACGCAGTAGACTGTGGAATCATGGCGAT

ATTTTGACTGTCCTTAAGAACCACATGGGGACAAGAACAATTGAGGGCATTGTGCTCGAC

TCTGAGAAGAAGAATGATCTAAAACCTAAGGAAGTAAAGTGGATCTATTTGAAGAAAGTA

TTTAGAAAATATATTGGTCAAGGTATAAAGGAAAATGGTGTAACATTTTACACTAGAGCT

TTTCAGCGCATGGTCAATCTGAGACTTCTTCAAATCAATCATGTCAAATTGGTCGGAAAT

TTCAAGCTATTACCTGCTGAACTGAGGTGGCTGCAGTGGAAAGGTTGCCCTCTGGAAGTT

ATTCCTCCAGAATTACTGTCCCGAAAGATTGCGGTTCTTGATTTGTCGGAGAGCAAGATT

ACACAGCTCTGGAATAAGAAAAAGTGGAATTGCTACCAGAACAAGATGGCAAAGCAGCTG

AAAGTCATGAATCTACATAGTTGTCGCCAACTTAAGGAAATCCCTGATTTATCTGGAATT

CAATTGGAGAGGCTGATTCTTGAACAATGCAATGAACTAGTTATGATCCATCCGTCAATT

GGAGACTTGACTAAGTTGAACTATTTGAACATGAAGGACTGTAAGAGCATTTTGGCATTT

CCAAATGACGTGTCTGGATTGAAACATCTACAAATACTTATCCTATCTGGTTGCTCAAGT

CTAAGTGAATTACCAGAGGACTTGAGTGGCTGGAAATCTTTGCAAGAGCTTCTTCTAGAT

GGCACGGCAATAAGAAAGCTACCTAACTCTATCTTTCACCTGAAGAATCTTCAAATCTTG

AACTTAAATGATTGCCGATCTTTGGAGTTGCTTCCTAGAGCCATTGGGAATCTAAGTTCG

CTGAGAGAGCTTTCTTTTAACGGATCCGCTTTAAAGGAAATGCCTGATTCCATTGGAAAT

TTGAAAAATCTTGAGGAATTAGGCTTGAGAATGTGCAGGGGACTCATCTCACTTCCTGAT

TCCCTTGGCGATCTTAAATCTTTAGTAGGACTTTATCTTGATCACAGCTCCATAAAAGAA

TTGCCATCTTCTGTTGGTTTATTATCTCATTTGAAGTTCTTTACGGCCAGCAATTGCAAG

TCCTTGAGTGAATTGCCCAATTCCATGAGTAACTTATCGTCATTGGTTTGGCTTTGTCTA

CAAGGGACCTCAGTTAGTGAACTAAATTTCCATTTAGGAAATTTCAAGTCCCTTGAGAAG

CTTGAGATGAGGAACTGCATTTCAATCAGTTGTTTACCTGACTCAATTGGAAATATGTTA

TGTTTAACTACTTTGGCCCTATGCAATACATCGATTACTGAGTTACCAGAATCTATAGGC

TTATTGGAACGACTTTGGATGTTGGAGCTGAATAACTGTTTGAATCTCCAACGTCTTCCG

GCTTCAATTGTAAGCCTTAAGAGTTTGTGTTACCTATATATGGATGAAACTGCTGTCTCC

GAATTACCCGATGAAATTGGAAAGCTTTCAAGCTTAAAACTACTGAAGATGAGAAAGAAA

CCACAGCCTAGAGTGGACGAAAATGAGGATGGTTTACATGTAGGAGAAAGCTCTAAGCGC

GTTACTCTTCCAGAATCATTTTCAAATCTATCATCCTTGGAATTCCTAGATGCTCATGCA

TGGAAAATATCTGGAAAGATTTCTGATGATTTTCAGAAGTTGTCTGCTTTGGAAAAGCTC

GACCTTGGACACAATGATTTTTGCAGTCTCCCTTGTAGCATGAAAGGACTTTGTGTTCTC

AAGCGTTTGCTTCTCCCTAACTGCAGAAAGCTCAAGTTTCTCCCTCAACTTCCCTCAAGT

TTGGAATGGTTAAATGCTGCAAACTGCTCCGCGTTGGAACATATAGCTAGCATATCAAGT

TTGGAATATTTGGAAGAACTCAACTTCAGTAATTGCAAGAGGATAATGGATATTCCTGGC

CTTGAAAGCTTGAAATCTTTGAAGAGGTTGTATACTCTCGGTTGCAACGCGTGCTTTTCT

TCTATAAAAAGGAGGATTTCCAAGGATTGTCTAAGGCATATGAAGTATCTCTGTGTTCCA

GGGGATGAACTTCCAGATTGGTTTATTGAGGATGTACCTATTAGCTTCTCAACTCGCAAG

AACCGCGATATCAAGGGGGTGATCATCGGTATAGTTGTGTCTCTGAACCAACAAGTAGAG

GATAATTTCAGATACGAAGTCCCAGCGATCGTGGATATACAGGCAACGATTACCAGACAA

GGTGATGTTGAACCTAAACTTAAGAAAACTTTGTACTTGTTGGGAGTTCCTGATACAGAC

GAAGATCAGCTCTATTTATGTCGATTTCAAGAATACAGTGATTTTACCCTTATGTTGGAA

GACGGAGACACAGTGCAGGTTGGAATTAAAGAGCGTCCACGTTTTAATGGCCTCGAACTG

AAGAAACATGGGATGTACTTAGTTTTCGAAAATGAGGATGATTTTGATGATAATGATGAG

GATTTGTTTGATGAATCTCAGCAGTCTGTGTCAAAGAAACTTACTAACTTCTTTCATTCA

TTATGA

>NTK326_mRNA_15738_cds NTK326_mRNA_15738 gene_8943|id=AT5G36930.1

ATGGCCACTGAACTGAAGTCTCAAGTGTACTTGAGTTTCAAAGCGAAAGACACCGGCAAA

ATTTTTGCAGATCACCTCTATGAAGCTCTGGTGGGAGCAGGTTTTGTAACATTAAGAAGC

TGTGGTGATGAAAATGAGGGAGGTGAAGATATCAAGTTCAATTTGCAAAAGGGTATTAAA

GAATCTGGGGTTTCAGTTATAATCTTCTCAAATGATTACGTGTCCTCAAGTTGGTGTCTT

GATGAGTTGGTAATGATCTTGGATTGTAAAAAGATAGCAAAACGTGCAGTTCTGCCCATA

TTTTACCACGTGGATCCTTCTGATGTTAGGAAACAGAAGGGAAGAATTGGAGAAGCATTT

GATATGGACAAAGAACTGGGAGGGAATCAAGGTGAAAATGAGAGGGTCAGAAAATGGAGG

GAAGCACTCAAAGAAGTTGCAGACTTGGGAGGAATGGTCTTACAAAACCAAGCTGATGGA

CACGAGTCCAAATTCATCCAGAAGATTCTTAAAGTGGTTGAGAATAAACTGAGCAGGCCA

GTCCTGTATATTTGCCCTCATCTGATTGGAATAGAACGGCGTGTTGAAAAGATCAACTTG

TGGCTAGAGGATGGATCTATTGATGTTGACACTCTTGTTATTTGTGGCATCGGTGGAATA

GGCAAGACAACAATGGCAAAGTTTGTGTATAATTTGAACTTCAGTAAGTTTGATGGTAGC

AGCTTTTTGTCCAACATTAGAGAAAATTCAACACACCGTAAAGGTTTAGTTACTCTTCAA

AGGCAATTTCTTTCTGATATTTGCAAAAGAAAGAAGAAAGCTATGTTTTCTGTGGATGAG

GGAATGACTGAGATGAGAGAGGCTGTACAGTGTAAAAGAATCCTTCTTGTTCTTGATGAT

GTAGATAACCGTGATCAAGTGGATGCTCTACTGGGAATGAAGGACTTGTTATATCCTGGT

AGTAAAGTCATTGTGACAACTAGGAACAAGAGATTGCTTAGGCCTTTTGATGTGCATAAG

ATTTATGAGTTTGAAGCATTGAATAGAGATGAATCGGTTGAGCTCTTAAGTTGGCATGCA

TTTGGTCAAGATTGTCCTATTAAAGGTTTTGAAATGTGTTCAGAACAAGTAGCAATCCAT

TGTGGAGGACTTCCATTAGCACTTGAAGTTCTTGGTGCTACTTTGGCAGGAAGAAACATA

GACATTTGGAAAAGTACAATACAGAAATTGGAAACAATTCCGAATCATCAAATTCTCAGG

AAATTAACAATAAGTTACGAATCTCTTGAGGATGATCATGATAAGAATTTATTTCTCCAC

CTAGCTTGCTTTTTCATTGGGAAGGACAGAGATCTAGCAGTAACTATTCTCAATAGGTGC

AACTTTTACACTGTAATTGGAATTGAGAATCTCATTGACAGAAATTTTATAAAAGTTGGT

AAGTCTAACAGCTTGATTATGCATCAAATGATTCGAGATATGGGAAGAGACATTGTTCGC

CAAGAATCACCACTGGAGCCTGGGAAACGCTCTAGACTATGGCGTTCAAAGGATTCCTTT

AACGTCTTAATCCAGAACCGTGCCACTCAAACAATTCAAGGCATTATTCTTGACATGGAT

ATGCTCAAGGAAAGTGACATAGTTAGCTCAAGCTTTTTCGCCAAGGATTTCAAGAAACAC

AAAATAAAAAACTTTCTCAACTATCCTAATCCTCAGAGAGTTCAATTCAAACAGAAAAGG

TTTGTTTTTTTCCCATGGCATTTGTCAGATGCCAAAGAAGCCACAAATGAGCTGGTTCTG

GGAACTGATGTATTTGCAAATATGCAAAAGTTAAAACTGCTCCAATTCGATCACGTTGAG

CTTCAAGGATCTTTTGATGTTTTTCCTAAGAGATTAAGATGGTTGCGCTGGTCTGAGCTG

CAACTTGAGTGCATGCCAATTGATTTTCCTCTGGAGAGCCTTGTAGTGATTGAATTACAC

CGTAGCAGCTTGAGGAGGATTTGGCATGGAGTCAAGTTCCTTAAAGATCTGAAGATTTTC

GATCTCAGCCATTCCTACGAGCTTCTAAGAACACCTGATTTTTCAGGACTCCCCAATCTT

GAAAAGTTGATCCTTCGATATTGTACAAGCTTGATTGAGCTTCATGAGACCATCGGGTGT

CTAGAATCACTTATTCTTTTGAATCTCAAAAATTGCAAAAATCTCCAGAGACTTCCAGAT

AGCATTTGCATGCTAAAATGTCTGGTGACACTAAATATCTCTGGTTGCTTGAATCTTGAA

TATGTGCCGATGGATCTAGATAAAATGGATTCACTGAGAGAGCTTTATGCTGATGAAATT

GCAGTTCACCAAATGATTTCTACTCCAGAAGAGGTCCAACCGTGGTATGGATTTCTGCGG

TCCTGGATGCTGAAGGGGAAAATATGTCCTAAAGTTTCACATATTAGTTTACCTAATTCC

TTGGTTACTCTGAGTCTTGCTAACTGTAATCTATCCAATGATGCTTTTCCAGTTGCTTTC

AGTAGCCTCTCCTTATTGCAAAACTTAGATTTGAGCGAAAATCCAATTTGCTGCCTACCA

AAGGGCATAATTTATCTCACCGGTCTTCAGAAGCTTGAAGTGGAAGGCTGTGAAAAGCTC

AGATCGCTCGTAGGGCTTCCCAATGTAGAACATCTCAATGTTACTAATTGCTGGTCGTTA

GAGAAAATATCATATCAATCAAGATCATCTAGACTGAAGGATTTACTTGTGTCGAATTGT

GCTAAATTAGTTGAAATAGATGGAAATTTCAAGTTAGAGCCCTTAAGAAATACTGAGGCA

GAGATGCTTTGCAAGTTGGGCTTGTCGAACTTAGCTTCTATGGATAATGTCATGATCAAT

CTTACATCTAATATCCTGAGTTACTACCGAATACATGGTAAAGGATGGACTCCAACAAGG

AAGACAAAGAAAGTTGTTCTTCAGGTATGTCTTGCTCGTTCTTTCATTGTTTCAGTTTTG

TGA

>NTTN90_mRNA_114425_cds mRNA_114425 gene_64714|id=AT5G36930.1:evalue=0.0:annot='Disease resistance protein (TIR-NBS-LRR class) family';id=Solyc09g092410.2.1:evalue=0.0:annot='Tir-nbs-lrr, resistance protein'

ATGGATACTCAATTAGTTCGAGGAGAATCATCTACATCTTCTCACTTCTCTTATGATGTA

TTCCTCAGCTTTCGAGGCGAAGACACCCGAAAAACATTCACTGGTCATCTTTATTCCAAA

TTGTGTGATGTTGGAATTAATACCTTTATTGACGATGAGGAATTGAGAAAGGGTGACGTG

ATTTCAAGTAAACTAGAGAAAGCAATTGAAGGGTCAAGAATTTCCATTATTGTTTTCTCA

AGAAATTATGCTTCCTCTAGTTGGTGTCTAAATGAACTAGTTAAAATTCTTGAATGCAAA

GAGAAACTAAAGCAGATGGTTTTGCCTATTTTCTATGATGTTGATCCGTCTGAGGTACGA

AAGCAAACTGGGTTATTTGGTGAAGCTTTGGCAAAACATAAGGAACGACCATTTGGAGCT

CAAAGGGCGGAGAAATGGAGAGCTGCACTTACTGAAGCTGCGAATTTATCTGGATGGGAT

TTGCAAAATGTTGCTGACGGGCATGAATCAAAGTTTATTGAAAAAATTATACAGCAAGTC

CTACAAGAGGTTAACCAGACACCTCTAGATGTTGCTTGGCACCCAGTTGGAGTAGATTCT

CGTGTCAAAGATATAGAGTTGTTATTACAAAATGAATGTGAAGATGGAGTTCGCATGATT

GGTATTCACGGAGTTGGAGGCATAGGGAAAACAACTCTAGCAAAAGCTATCTACAATCAA

ATGTTTCGACTCTTCGACAGTAGTTGCTTCCTTTCGGATGTTAGATCAGAAGCTGAAGAA

TTTGGTCTTGTCAAGCTACAAGAGAAACTTCTTCAACAAGTACTCAAAAATAAGGACATC

AAAGTTGGCAATGTCGCTCAAGGCATCAATTTAATCAAAGCAAGACTCGAGTCAAAGAAG

GTTCTAATTATTCTTGATGACGTGGACCACAGAAACCAATTAGAATCCTTAACAAGAGAA

AGAAGTTGGTTTGGTTCGGGTAGTTTAATAATCACTACCACCCGAGACAAGCGATTGCTA

TGTCGGTTTGGAGAAAAAGAGCGATATGAGGCCAAACTATTAAATGACGATGAAGCTATG

TTACTTTTTTGTTGGCATGCTTTTGATAGTCATTTTCCACCAGAAGATTATGTTAATTTG

GCACGAGACATAATCAGATATTCAGGTAGGCTACCATTAGCTCTTGTGACATTGGGGTCA

CATTTACAAGGAAGTTCTATAGAAGAATGGGGATATGAATTCAAAAAACTAAAATCAATT

CCTCATTGTGATATCCAAAAGATTCTCAAGATAAGCTTTGATGGACTTGATGATGAAACA

CAAACTGTTTTCCTCGATATTGCATGCGCCTTCCATGGGTTTTATGAGCAAGAAGTTACT

GAAATGGTAAATGCATGTGGCTTTCATGCTAAAAGTGCAATTGCAACTTTAGTCCAAAAA

CACTTACTCCAAAGATCTTGGAATATTTTGGGGATGCACGATTTAGTGCGAGATATGGGA

AGAGAAGTCGTTCGCATGGAATCAGCTCGAGACCCTGGAAAACGGAGTAGATTATTCATC

CCTCAAGAAGTCCGTGATGTTCTACAAGGAAATAAAGGTTCCAAAAAGGTAGAAGTACTG

AAGGTAGATCAACTAGCATTTGAGGGACTGCACTTGAGCACCAAAGCATTTAAGAAAATG

AAAAACCTTAGGGTTCTTATGATGGATGAGTTACATATTAGTGGAGATTTTGAGCTGTTA

TCCAAGGAGCTCAGATGGTTGTCTTGGAAAAGATGTCCTTTAAAATGTATACCATCAAAT

TTTCCGGCTGTGAATCTTGTAGTTCTAGATATGCGGGGGAGTGATATCCAAGAATTTCAA

TTGAATTTGCAGTGTTGTAGAAGTTTGAAGAAACTGGATCTCTCTCATTGCAAGCAACTC

AGAAGCACTCCAAACTTCAATGGTTCGATGAGTCTTGAGGCTTTGTATCTCTATGGATGT

TCAGGTCTGACGGAGATCCATCCATCAATAGGAAATTTGTCCAGACTAATTGAACTATAT

ATATCTGGTTGCGAAAAACTTACGGATCTTCCAAGCAGCATATGCCAGCTAATATCCGTT

AATTACTTGAGCATTACTGACTGCTCATCAATAAAAACACTGCCAGATAACCTTGGAGAT

ATGAAAAGTCTAAGATTTCTTTCTGCAGTTGATACGGGTATAAAACAATTGCCTAGATCT

GTTGAAATGCTAAGAAATCTTGAATCATTGGGAGTGGGAGGTCGAAAGTTAGAGGCCAAA

AGGAGTATTTCTGGAAGAGGAGTCCATCAGATACAATATTCCTTGTCAAATTTTGTGACC

ATATTGAGGCTTGTATACTGTAATTTGTCCGAGGCTGATATTCCTAGGAATATTGGGAGC

TTATCCTCCTTAAAATATTTAGATTTGAGTGGCAACAGTTTCTATTGTCTACCCTTTGAT

TTTTCTAAGTTACAATTATTGGAAGAGTTATGTTTGAATGACTGTCCGAATCTTCAAACA

CTCCCATCAATATCAAATTTAGAGAATCTTCGATATCTTGAACTTTATAATTGCCAAAAA

TTGGTCAAGATTACAGAGTTGGACAACCTCCCCTCTGTAGAGCGATTTAATATGATTAAT

TGTAGTTCTCTGCAGAATCCATTCAATGAAGGCTTCTTTAATGCACCTGCTCGAGCATTT

CAATCTAAACAACAACAAGATCAGCTTGATGTAGAAATTTTTCTCGAATGCAAGGAGATT

CCAGAATGGTGCAGGAATCAAGTAACAGCTTCATCTATGTGTTTGACTATTCCGACACAT

AATAATGATGAGTATAACTTCTTAGGAATGGTTCTCTGGTTTGTTATCGACTCTTTGGAT

GCAGCCCCTTATCCATGCCACAGGATTAGTATTGCCCATAAAGAGACTGTAATTGTTCCG

TGGAAATATAGCTTACATGAGGCTTTTGATGGACATAGAGATATTGCTTACATAAGGCTT

TTGATGGCCTTAAATGAACGTTTTGATGGCGAGATGATTAAAGGTGGGGAAAGGATAGAA

GCATGGTCCGAAGAAGTTACAGTAAAGAAGATAGGGATCCATCTGTTATATTTAGACCAA

CATGGTAATGTTATATCTTTACCTGGAGACGTTATACAATAA

>NTTN90_mRNA_72762_cds mRNA_72762 gene_41128|id=AT5G36930.2:evalue=0.0:annot='Disease resistance protein (TIR-NBS-LRR class) family';id=Solyc09g092410.2.1:evalue=0.0:annot='Tir-nbs-lrr, resistance protein'

ATGGATAGTCAATTAGTTAGAGGAGAATCATGTACATCTTCTCACTTCTCTTATGAAGTA

TTCCTCAGTTTTAGAGGTGAAGACACCCGAAAAACATTCACTGGTCATCTTTTTTCCAAA

TTGTCTGATGTTGGAGTTAATACCTTCATTGATGATGAGGAATTGAGAAAGGGTGATATA

ATTTCAAGTAAACTAGAGAAAGCAATTGAAGAGTCAAGAATTTCCATTATTGTTTTCTCG

AGAAATTATGCTTCCTCTAGTTGGTGTCTAAATGAACTAGTTAAAATTCTTGAATGCAAA

GAGAAATTAAAGCAGATGGTTTTGCCTATTTTCTATGATGTTGATCCTTCTGAGGTACGA

AAGCAAACTGGGTTATTTGGGGAAGCTTTGGCTAAACACAAGGAACGACCATTTGGAGAT

GAAATGGTGGAGAAATGGAGAGATGCACTTACTCAAGCTGCAAATTTATCTGGATGGGAT

TTGCAAAATGTTGCTGACGGGCATGAATCAAAGTTTATTGAAAAAATTATACAACAAGTC

CTACAAGAGGTCAACCAGACACCTCTAGATGTTGCTTGGCACCCAGTTGGTGTAGACTAT

CGTGTTAAAGATATAGAGTTGTTATTGCAAAATGAATGTGAAGATAAAGTTCGCATGATT

GGTATTCACGGAGTTGGTGGCATAGGGAAAACAACTCTGGCAAAAGCTATCTACAATCGA

ATGTTTCGACTCTTCGATAGTAGTTGCTTCCTTTCAGATGTTAGATCAGAAGTTGAAGAA

TTTGGTCTTGTCAAGCTACAAGAGAAACTTCTTCGACAAATTCTCAAAACTGAGGACATC

AAAGTTGGCAGTGTTGCTCAAGGCATCAATCTAATCAAAGCAAGACTCGGGTCAAAGAAG

GTTCTAGTTGTTCTTGATGATGTGGACCATAAAAGACAGTTAGAAGCCTTAACAAGAGAA

AGAATTTGGTTTGGTTCGGGTAGTTTAATAATCATCACCACCCGAGACGAGCGATTGCTA

TGTCGGCTTGGAGAAAAAGAGAGATATGAAGCCAAACTATTAAATGGCAATGAAGCTATG

TTACTTTTTTGTTGGCATGCTTTTGACAGTCATTTTCCACCACAAGATTATGTTAATTTG

GCACACGGCATAATCGAATATTCAGGCAGGCTACCATTAGCTCTTGTGACATTGGGGTCA

CATTTACAAGGAAGTTCTATAGAAGAATGGGGATATGAAGTCGAAAAACTAAGAGCAATT

CCTCATAGTGATATCCAAAAGATTCTCAAGATAAGCTTTGATGGACTTGATGATGAAACA

CAAACTGTTTTCCTTGATATTGCGTGCGCCTTCCATGGGTTTCTTGAGCATGAAGTTACT

GAAATATTAAATGCATGTGGCTTTCATGCTAAAAGTACAATTGCAACTTTAGTCCAAAAA

CACTTGCTCCAAAGATCTCCGTATCATTTGCAGATGCATGATCTAGTGCGAGATATGGGA

AGAGAAATTGTTCGCACGGAATCGGCTCGAGACCCTGGAAAACGGAGTAGATTGTTTATC

CCTCAAGAAGTCTGTGGTGTTCTACAAGGAAATAAAGGTTCCGAAAATATAGAAGTATTG

AAGGTAGATCGAGGGACATTAAAGGGAGTGAAGTTGAGCACCAAAGCATTTGAACAAATG

GAAAACTTGAGGGTTCTTATAATGGATGAGTTACATATTAGTGGAGATTTTGGGTTGTTG

TCCAAGAAGCTCAGATGGTTGTCTTGGAAAAGATGTCCTTTAAAATGTATACCATCAAAT

TTTCCAGCTGAGAATCTTGTTGTTCTAGATATGCGGGAAAGTGCTATTCTAGAATTTCAA

CTGAATTTGCAGTGTTCTAGAAGTTTGAAGGAGTTGAATCTCTCTAATTGCAAGCAACTT

AGAAGCACTCCAAACTTCAATGGTTCACCGAGTCTTGAGATTTTGAGTCTCTTTGGTTGC

TCAAGTCTGATGGAGATCCATCCATCAATAGGGAATTTGTCCAGACTAATTAAACTATAT

ATGCGTGGTTGCGAAAAACTTACGGATCTTCCTAGCAGCATATGCCAGCTAAAATCCCTT

GATTACTTGGACATTGATGAATGCTCATCAATAAAAACACTGCCAGATAACCTTGGAGAT

ATGAAAAGTCTAAGACATCTTTATGCATCTGATACAGGTATAAAACAATTGCCTAGATCG

GTTGAAATGCTAAGAAATCTTGAAACTTTGAGAGTGGAAGGTGAAAAGTTAGAGGCCAAA

AGGAGTATTTCTGGAAGAGAAGTCCATCAGATACAATATTCCTTGTCAACTTTTGTATCC

GATTTGAGACTTACATACTGTAATTTGTCCGAGGCTGATATTCCTAGGGATATTGGGAGC

TTATCCTCCTTAGAGCTTTTAGATTTGAGTGGCAACAGTTTCTATCGTCTACCCATTGAT

TTTTCTAAGTTACGATTATTGGTGGAGTTGTATTTGAATGACTGTGAGAATCTTCAAACA

ATCCCGTCAATATCAAATTTAGAGAATCTTGAAATTCTTGAACTTGAAAATTGCCAAAAA

TTGGTCAAGATTAGAGAGTTGGACAACCTCCCTTGTATAGAGCGGATCAATATGATGAAT

TGTAGTTCTCTGCAGAATCCATTCAATGCAGGCTTCTTTAGTGCACCTGCTCTATCATTT

CTATCTGGAGAAGATTCAGATTATGGTTATTTAGAAATTTATCTGGAATGCAATGAGATT

CCAGAATGGTGCAGGAATCAAGTAATAGCTTCATCTATTTGTTTGACTATGCCTACACAT

AATAAGGAGTATAACTTCTTAGGAATGGTTCTCTGGTTTGTTATTAGCGACTTTTTGGAT

GCAGCCCCTTATCCAAGCTTCTTGATTAGTACTGTCCATAAAGAGACTGCCGTTGGTGTC

TGGTTTTTAATCGACCCTTCGGATCCAAGCTTCAGGATAAGTATTGCTCATAAAGAGAAT

TTAATTTCTCCGTGGGGTATACCTCATGGACAGAGAGAAGTTTCATGTGTATATTACATC

TCTTCCTTACATAAAGCTTGTGATTGCGAGATGATCATCGAAGGCGGGGAAAGGATAAAA

GTGTGGTCTGAGCACATTACAATAAAGAAGATTGGGATCCATTTGTTATATTTAGACCAA

TATGGTAATGTTATATCTTTACCGGGAGACGTGGATCGTTCTTATACTAGGCCAAAAGAT

TTCAGGAATTGGTTGGAAAGAGATTTTTTTGACGGGAAAATGTATAGTTGA

>NTTN90_mRNA_35268_cds mRNA_35268 gene_19764|id=AT5G17680.1:evalue=2e-36:annot='disease resistance protein (TIR-NBS-LRR class), putative';id=Solyc01g008800.1.1:evalue=0.0:annot='Tir-nbs-lrr, resistance protein'

ATGGCATCATCTTCTACTTTTGCGAGTACTTCACAGTTTCCTCGATGGAACTATGATGTC

TTTCTAAGCTTTAGAGGTGAAGATACTCGGAAAACATTTACGAGTCACCTGTACGAAATC

TTGGATATCAGGGGAATAAAAACCTTTCAAGATGATAAAAGGCTAGAGCATGGCGCATCC

ATTTCAGATGAACTCTGTAAAGCTATCGAAGAGTCTCAATGTGCTGTCATCATTTTCTCA

AAGAATTATGCAACATCGAGGTGGTGCTTGAATGAACTAGTGAAGATCATGAATTGCAAG

ACTCAATTTGGACAAACTGTAATACCAGTCTTCTATGATGTGGATCCATCATATGTTCGG

AACCAGAGGGAGAGCTTTGCTGAAGCATTTGCCAAACATGAAACAAAGTATATGGATGAT

GTCGAAGGAATACAAAGTTGGAGGATTGCTTTAACTGCAGCGGCCAATCTCAAAGGCTGT

GATATTCGTGACAAGACTGAATCAGACTGTATTCGACAGATTGTTGACCGAATCTCGTCC

AAATTATGCAAGATTTCTTTATCTTATTTGCAAAACATTGTTGGAATAGATACTCATTTA

AAGGAAATAGAATCCTTACTAGGGATAGGAATCAATGATGTTCGGATTGTGGGGATTTGG

GGCATGGGGGGAGTCGGTAAAACGACAATAGCTAGAGCTATGTTTGATACTCTCTTAGTA

AAAAGGGATAGTTCTTATCAATTTGATGGTGCTTGTTTCCTTGCGAATATTAAAGAAAAC

AAACGTGGAATGCATTCTCTGCAAAATATCCTTCTCTCTGAACTTTTAAAGGAAAAAGCT

AATAACAATAGTGAGGAGGACGGAAAGCACCAAATGGCTAGTAGGTTTCGTTCTAAGAAG

GTCCTAATTGTGCTTGATGACATAGATGATAAAGATCATTATTTGGAGTATTTAGCAGGT

CATCTTGATTGGTTTGGTAATGGCAGTAGAATTATTGTAACAACTAGAGACAAGCATTTG

ATAGGGAAGAATGATGTAATATATGAAGTGACTGCACTACCTGACCATGAATCCATTCAA

TTGTTCTATCAGCATGCTTTCAAAAAAGAGGTTCCAAATGAGCATTTTAAGGAGCTTTCA

TTGGAGGTAGTAAATTATGCTAAAGGCCTTCCTTTAGCCCTCAGAGTGTGGGGTTCTTTG

CTACATAACCTAGGCCTAACTGAATGGAAAAGTGCTATAGAGCACATGAAAAATTACTCT

AATTCTGGAATTGTTGATAAGCTCAAAATTAGTTATGATGGATTAGAGCCCAAACAACAA

GAGATGTTTCTAGATATAGCATGCTTCTTCCGAGGGGCAAAAAAAGAGTATGCCATGCAA

ATTCTTGAGAGTTGTCATTGTGCAGCTGAATACGGATTGCGTGTCTTAATTGACAAATCT

CTTGTGTCCATCATTGAAAATGATCGTATTCAAATGCATGACTTAATGCAAGATATGGGT

AAATATATAGTGAACTTGCAAAAGGATTCGGGGGAATGCAGCAGGCTATGGCTCGACGAG

GATTTTGAAGAAGTGATGATCAACAATAAGGGGACCACGAAAATGGAAGCAATCTGGTTT

CCTTATTACCATTGTGGTACATTACGCTTTAGCAAAGAGGCCATGAAAAATATGAAAAGG

CTTAGGATATTAAACATAGGGAAGTGGCAGACCGGTGATGGTTCCATTGATTATCTGCCC

AACAACTTGCGTTGTTTTGTCTGGACTAAGTATCCTTGGGAGTCATTGCCATCTACATTT

GAACCCAAAATGCTTGTTCACCTTGAACTCCAGAGCAGTTCACTGTGTTCTTTATGGATG

GAAACAAAGCATTTGCCGTCTCTACGGACGATAGATCTCAGCTACTCTAAAAGCTTGATA

CGAACACCAGATTTCAAGGGAATGCCAAATTTGGAGTATTTGAATTTGGAGGAATGTAGT

AATCTTGATGAGGTTCACCATTCGCTGGGGTGTTGTAGAAAACTCATTCGGTTAGATTTG

AGTTATTGTGAAAGCCTTGAGAGGTTTCCATGTGTTAACGTGGAATCTCTTGAATATCTG

ACTGTAAATGATTGCTATAGTTTAGAGACATTTCCAGAAATTCACGGGAGAATGAAGCCG

AAGATAGAGATTCACATGCTAGGCTCTGGGATAAGGGAACTACCATCCTCTTTTTTTCAG

TACCAAACTCATATTACCGAGCTAGATTTGAGCGGTATGGAAAACCTTGTAGCTCTTCCA

AGCAGCATCTCTAGGTTGAAAAGTTTGGTTATTTTATGTGTGTTGGGTTGCTCAAAACTT

GAAAGCTTGCCAGAAGAGATCGGGGATTTAGAAAACTTGGAGAGGCTTGATGCCACCTTT

ACCCTAATATCACGACCTCCGTCTTCCATCGTACGCTTGAACAAACTTAGAGTCTTGGAT

TTTGGATTCTGCAAGGATGGAATGTACTTTGAGTTCCCTCCAGTGGCTGAAGGATTATGC

TCGTTGGAATATCTGAATCTCAGTAACTGCAATCTAACAGATGGAGGACTTCCGGAAGAT

ATTGGATCCTTATCCTCTTTGAAATATTTGGATCTCACAGGAAATAATTTTGAGCATTTG

CCTCAAAGCATAGCCCAACTTGGTGCTCTTCGAATCTTGGACTTAACAGAGTGCGAGAGG

CTTACACAACTACCAGAACTTCCACCAGAATTAAATGAATTGTATGTAGATTGTCATATG

GCTCTGAAAAGTATCCATGATTTAGTATCAAAGAGAAAGAAACTACAGAGGGTGAAATTC

TTGCCTCTGTATGGTAAGGATGATGCACATAATGATTCTATATATAATTTGTTTGCACAT

GCCCTGTTTCAGAATATCTCTGCTTCATATTCCTTGTCCGAAAATGTACTTACCATTTGG

CATCCTCACAAGAAGATCCCAAGTTGGTTCCTCTATCAGGGAACGGATCGTAGAGTATCA

GTGAATTTGCCTGAAAATTGGTATATACCTGATAAATTCTTGGGATTTGCTGGATGTTAC

AATGGCAGCTTAATTGACACCACAGCTCAATTGATTCCCAAATGTGATGACGGGATGTCG

TGCATGACCCAGAAACTTGCTCAAATTTGCATGACCCATTCAAATCTTGATAAAGAATAT

AATATACATTTTTTCTTTGTACCTTTTGCTGGCTTATGGGATACGTCTAAGGCAGATGGA

CAAACACCAAATGACTATGGGCTTATTAGGCTATCTTTTTCTGGAGAAATGAAGAAGTAT

GGACTTCATTTGTTGTATAAAGAAGAACCTGAGGTTGAGGCCTTGTTACAAATTAGGGAA

AATAACAATGAACCAACAGAATATCGCATTGAGATAAGGAGTAGCAGATCTGACAATAGT

GAACACCATGACTCCGTGACAGATGAAGCCAGTTGCTGTCGCATACTGTAA

>NTTN90_mRNA_42253_cds mRNA_42253 gene_23647|id=AT5G36930.1:evalue=0.50:annot='Disease resistance protein (TIR-NBS-LRR class) family';id=Solyc09g092410.2.1:evalue=0.0:annot='Tir-nbs-lrr, resistance protein'

ATGGATACTCAATTAGTTAGAGGAGAATTATCTACGTCTTCTCACTTCTCTTATGAAGTA

TTCCTGAGTTTTAGAGGTGAAGACACCCGGAAAACATTCACTGGTCATCTTTATTCCAAA

TTGGATAATGTTGGAGTCAAAACCTTCATTGACGATGAGGAATTGAGAAAGGGTGATGTG

ATTTCAAGAGAATTAGAGAAAGCAATTGAAGGGTCAAGAATTTCCATTGTAGTATTGTCG

AGAAATTATGCTTCCTCTAGTTGGTGTCTAAATGAACTAGTTAAAATTCTTGAATGCAAA

GAGAAATTAAAGCAGACGGTTTTGCCTATTTTCTATGATGTTCATCCTTCTGAGGTACGA

AAGCAAATTGGGTTATTTGGTGAAGCTTTGGCTAAACACAAGGAACGACCATTTGGAGCT

CAAATGGTGGAGAAATGGAGAGCTGCACTTACTGAAGCTGCAAATTTATCTGGATGGGAT

TTGCAAAATGTTGCTGACGGGCATGAATCAAAGTTTATTGAAAAAATTATACAGCAAGTC

CTACAAGAGGTCAACCAGACACCTCTAGATGTTGCTTGGCACCCAGTTGGAATAGATTCT

CGTGTCAAAGATGTAGAGTTGTTATTGCAAAATGAATGTGTAGATAAAGTTCGCATGATT

GGTATTCACGGAGTTGGTGGCATAGGGAAAACAACTCTGGCAAAAGCTATCTACAATCGA

ATGTTTCGACTCTTCGATAGTAGTTGCTTCCTTTCAGATGTTAGATCAGAAGCTGAAGAA

TTTGGTCTTGTCAAGCTACAAGAGAAACTTCTTCGACAAATTCTCAAAATTGAGGACATC

AAAGTTGGCAGTGTTGCTCAAGGCATCAATCTAATCAAAGCAAGACTCGAGTCAAAGAAG

GTTCTAATTGTTCTTGATGATATGGACCACAAAAATCAATTAGAATCCTTAACAAGAGAA

AGAAGTTGGTTTGGTTCGGGTAGCTTAATAATCATTACCACCCGGGACAAGCGATTGCTA

TGTCGGCTTGGAGAAAAAGAGAGATATGAGGCCAAACTATTAAATGACAATGAAGCGATG

TTAGTTTTTTGTTGGCATGCTTTTGACAGTCATTTTCCACCAGAAGATTATGTTAATTTG

GCACGAGACATAATCAAATATTCAGGTAGGCTACCATTAGCTCTTGTGACATTGGGGTCA

CATTTACAAGGAAGTTCTGTAGACGAATGGGGACATGAATTCGAAAAACTAATAGCGATT

CCTCATTCTGATATCCAAAAGATTCTCAAGATAAGCTTTGATGGGCTTGATGGTGAAACA

CAGACTGTTTTCCTCGATATCGCATGCGCCTTCCATGGGTTTGATGAGCATGAAGTTACT

GAAATATTAAATGCATGTGGCTTTCATGCTAAAAGTGCAATTGCAACTTTAGTCCAAAAA

CACTTGCTCCAAAGAATTGGGTATCATTTGGTGATGCATGATCTAGTGCGAGATATGGGA

AGAGAAATCGTTCGTATGGAATCATCTCGAGACCCTGGAAAACGGAGTAGATTGTTCATC

CCTCAAGAAGTTCGTGATGTTCTACAAGGAAATGAAGGTTCCGAAAATGTAGAAGTGCTG

AAGGTAGATCGAGGGACATTAAAGGGAGTGAACTTGAGCACCAAAGCATTTGAGCAAATG

AAAAACCTTAGGGTTCTTATAATGGATGAGTTACATATTAGTGGAGATTTTGGGTTGTTG

TCCAAGAAGCTCAGATGGTTGTCTTGGAAAAAATGTCCTTTAAAATATATACCATCAAAT

TTTCCAGCTGACAAACTTGTAGTTCTAGATATGCGGGAGAGTGATATCCAAGAGTTTGGT

TTGAATACGCAGTGTTGTAGAAGTTTGAAGGAGCTGAATCTCTCCCATTGCAAGCAACTC

AGAAGCACTCCAAACTTCAATGGTTCACTGAGTCTTGAGACTTTGTATCTCCATGGTTGC

TCAAGTCTGACTGAGATCCATCCATCAATAGGAAATTTGTCCAGACTAATTGAACTATCT

ATGTCTGGTTGCGAAAAACTTACGGATCTTCCAAGCAGCATATGCCAGCTAATATCCGTT

AATTACTTGAGCATTAGTAACTGCTCATTTATAAAAACACTGCCAGATGACCTTGGAGAT

ATGAAAAGTCTAAGATCTCTTTATGCATCTCGTACGGGTATAAAACAATTTCCTAGATCT

GTTGAAATGCTAAGAAATCTTGTAACTTTGATAGTGGGAGGTCAAAAGTTAGAGGCCAAA

AGGAGTATTTCTGGAAGAGGAGTCCATCAGATACAATATTCCTTGCCAACTTTTGTATGC

GATTTGAGCCTTACATACTGTAATTTGTCCGAGGCTGATATTCCTAGGGATATTGGGAGC

TTGTCCTCCTTAAAATATTTGGATTTGAGTGGCAACAGTTTCTATTGTCTACCCTTTGAT

TTTTCTAAGTTACGATTGTTGGAGAAGTTGTGTTTGAATGACTGTGAGAATCTTCAAACA

CTCCCGTCAGTATCAAATTTAGAGAATCTTTATAAAATTAACCTTTATAATTGCCAAAAA

TTGGTCAAGATTACAGAGTTGGACAACCTCCCTTCTATAGAGCGGATTAATATGATTAAT

TGTAGTTCTCTGCAGAATCCATTCAATGAAGGCTTCTTTAGTGCACCTGCTCTATTTGCA

TTTAGTGCACCTGCTCGATATATGGTTAGTCTCTCTCCCTCTCTGTCTCTCTCTTTCCTA

TCATATTAG

>NTK326_mRNA_24132_cds NTK326_mRNA_24132 gene_13820|id=AT5G17680.1

ATGCCATCTACCTCTTATAGTTCATTACCACCTTCCAAACAGTGGGATTATGATGTGTTC

TTGAGTTTTAGAGGAGAAGACACGCGTAAGACCTTTGTAGCCCACCTTTACAGAGAATTA

CGTCGGGCTGGAATCAATACCTTCAAAGATGATGAAACACTAGAACGAGGTGCATCGATT

TCACCTCAACTTGTCAATGCCATTAAACGGTCAAGGTTTGCCATTATCATCTTCTCAAAG

AACTATGCATCATCCAAATGGTGCTTGGATGAGCTAGTGAAGATCATGGAATGTCGAAAT

GAAATTGGGCAGGCAGTTGTACCAATTTTCTATGACATAAATCCATCAGAAGTACGTTCC

CAGAGAAATAGTTTTGCCGAAGCCTTTTCCAAATATGAGGAGGAATTCAAGGGTGACACC

AATAAGGTGCATAGTTGGAGGAAAGCCTTAAATAAAGCAGCCAATTTGGCAGGCCATGAT

CTGCATAGTACTACTTACAATGGAAATGAGTCTTGGTGCATTCAACGCATTGTGAAAGAG

ATAGCTAATGAGTTGTGCCAAAAGTCGATGATCAGTGGCACTCTAGTCGGAGCAGAATCA

CAAATTCAGGCTGTAAGCTCGATGTTGATGATGGAATGTGAAGATGTTCGATTTATCGGT

ATTTCAGGGATGGGTGGTATTGGCAAGACCACTATTGCAAGAGCCATTTTTGACAGGTTT

GCTCATCGATTTGAAGGTGCTTGCTTTGTTGCAAATATTAAAGAAAATCAAGATAAACAA

GGATTGCTGTCTTTGCAAAAGACTGTGCTTTCTAAGGTGTTGACAATTGAATCTGTGAAT

CTTGCTGATGAATATGGTGGAATTGATATAATAAGAAAAAGGCTTGGCTTTAAGAAGGTT

CTAGTAGTTCTTGATGATGTGGATCACCAAAACCAGTTGGATGGGCTAGCTGGAGCCCAT

GATTGGTTTGGTAAATACAGTAGAATTATTATAACAGCGAGGGACGAGCACTTGCTTTTG

AACTGTGATGACACTTATAGAGTTAGTTTACTTGCTATAGCTGAAGCTACTCGGCTATTT

AGTTGGCATGCTTTTAGAAAGACATCCCCGGTCAATGGTTTTGAGCTGTTTTCTCACCGA

GTTGTACAGTACGCTGGTGGCCTTCCCCTGGCGCTTAAAGTCTTGGGTTCCTTCCTTCGT

GGACGGAACATAAAGCAGTGGAGGAGTGCACTGGATACACTGCAAGATATTCCTAATGAT

GAAATCATTTCAAAGCTTAAGATAAGTTATGATGGGCTAGGGGATAAAGTGAAGCAAGTT

TTCCTAGACTTAACATGTTCATTTGTAACATCAGATGCTGCTACTCTGAAAAAACTGTTC

ACTGAAATAATTATTGATGTCCTGGTTGAGAAGTCCCTCCTTTTTGTATCATCATTTGGT

AGGATTGGGATACATGATTTGATTCGAGAAATGGGCCGACGTATTGCAGTGCAAGAATAC

CCAAGGCGCCGGATATGGCTTCATGAGGATATTGCTGATATTTTAAGTGAACATACGGGC

GGAGAAGCAATAGAAGTCATATTAATCCCGTTGATGTCCAGCTCAGAGGAGAATACCATT

CACCTGAGCAATGAAGTCTTCAGACATATGAAGCGACTAAGAATATTTGTATCATCATCT

CACAAGAACGTCATGTATTTTTGCTCTCATGACCCCATTAAGTTTCTTCCTAACAGCTTA

TGCTGGATTAATTGGTCATATTATCCTTCACCATCATTGCCAGAAAATTTTGAACCACCA

AAGCTGGTGGGGCTTATTATGCATTGCAGTTACGTGGTTAACCTCTGGAAGGGGTCAAAG

CATTTAAACAGGTTGAGTATTCTTGATTTGAGTGATTCTCGAAAATTAATCCAAATTTCA

GACCTTTCCGGGTCTCCAAATTTGGAGAGGCTAATCCTATGTCACTGTGTTCGGCTGGTA

GAGGTCCACCCATCTGTTGGAGCCCTCAAAAAGCTTACTATTTTAGATGTGGAAGGTTGT

GAAAAACTTGAAAGGCTTCCCTCTAAGTTTCAATCCGGGTCTCTTGAAGTTCTAAATTTT

TCTGGTTGTCGGAGTTTGAGAAAAGTTCCAGACATTCAGCAAAATGTGAATCGTTTAACA

GAATTCAAGAAGCCAAACTTTGGGGTACTTGAATTAACATCATCATTCCTGAGCTATCTA

GATTTGAGTGGCTGCAGTAACATTGAAACGCTCCCTAGCAGCATGTGCAGGTTGAAAAAT

TTGAAGTATCTTTACCTCAATCGCTGTACCAAACTAAAGAACTTGCCAGAAGATATTTGT

GAACTAGTGAACTTGGAGGGGCTTGATGCGAGTGAAACATCAGTTTGGTGCACCCCAAAT

TCCATCACATGCTTGAGGAAACTGAAGTACCTATCTTTTCGGAAAGTACCCAAGGCTTTT

CATTTGAGCGATTTATGCAGTTGGGGATGTTTTGCCCAACTTGATTTGAAGTTTCAGCTG

CCCAGCACTTTATCAGTCTTTTGCACACTTATAAGACTGGATCTCAGTGCTTGCAATTTG

TTTGATGGCAGTATTCCTGAGGATCTTGGATGCTTGGCCTCACTACTAGAGTTAAACCTG

AGCAGAAATAACTTCACCTTTTTACCTAAAAGCATTGTGCTACTTAACCGCCTTCGACAC

CTTGACATAACCTATTGTGAAAAGCTAACAGAGCTGCCAGAGCTTCCTCCACGTATAATG

AAACTGTTTCTAGACGATCGCTTTGCTTTGGAAAGCATTCCAACACTACCAACCATGTAC

AAAGAGTTGTACTTGGTCTCATTCGCTAATCAGAAGTTGCAAGAGATGTGGTGTGCTTCA

CGCAGGGAAAGTTCTGGTGCAAAGATGAGAAACTGTATAAGGAAGAACATGACGGATATG

CTAGAACAGATTCTCCTTCCATTTTTGTCAATAGTGCAGCTTAAGTTGGTTTTTGACGAC

CAGCGCAGAGGGGGGAGATTTGGTATTGTCTTCCCTAGAAGTGATCCAAGTGCTAAGGTT

CCTAGGTGGTTCAAGTATCGTAAGACATGTTCAACGAGGATTTCCTTCAACCTGAAGAGA

CACTGGTATAATAATAAGTTTATGGGATTTGCTATCTATTGTCAGCTTCCTTTCTTAAAT

GACGAATCACCGAAAAACCGTAATAGGCAGTTTGCATTCAGTTTGCTATGGGGTGCAACA

ATCACTACAAAATTGGTGCCAGGACGTACTGCTATGGATCAACAAACTCCTGCAAAAATA

GTTCATTTGCAAGTGTCAAATGTAGTAGCATATGGCAGTCATGACTGCTTTATTTTCTTA

CAATTGGACCTAAGAAAGTTACATTTTAATGGTAAAGGTAAGGGCACAATGTTGATAGAT

AATCCAAATGACTATTGTAGATTTGAGGCATCTCTAGATTGTCGTATGTCATCAAATTGG

GGAGTTCGTCTGGTATATGCTGATGATATTGAGGTGATGAGGTTGGAATGGGCTTGGCAG

TCTAACAGATTTGAGCTTGAGCATTTGCATGAAGAGGATCTCGAGTTGTGTACTGGGAGG

TTGAAAGAATTGTCAGTCCCAGGGGAAATAAGATATTTTTATGCAACACTTATTTTCTAT

GAATTGCCAGTTCTATCGACTTGCCCGAGATGCTTATCCTTTGCTGAAGACTTGACCCAC

GTATTCTTGAACTGTAAATACTCTCAGCTAGTCTGGGAAGGATCAAGATTGGGCCTAAAC

TTTAAAGTTGGGACCCCAGTTGAATTTCAAGAGTGGTTACTTAAGTGGACTTTGTCAGCT

CCTGAAAATGAAGATTTCAGTTTTTCGTTAGCTGTCCTATGGGCGATCTGGAAGCACAGA

AACAAGTTAGTGCATGAAAGAGCTGTTTTTGTTCCCTCAGAAGTCATTTCCACTGCCATT

AAAGAGCACTCAAGGTTTCCGTATTCTGTTGGCATAGCAATAAGAGAGCTCTATGAGGTG

CATAGAGGAACAATTGTCGATGTCAATGCTTTCAACGTTGATTCTGATGTACAGCTGGAC

GACAACGTACTAGTAATGGAGGTTAAGGGAGCTTGGAAAGAGGGACAAGAATGGGCTGGA

ATGGCATGGGTAGCTTATCGGTGGAACTCTGGGGTTAAAGTGGCTGAGGCTAGAAGATCG

TTGAAGGTCAGGAGCAAACTACATGCAGAAGTACATGCCTTTGAGCATGCAGTGAAATGG

TCCCTCGTCATAGATTCTCCAATCGTAATTTTTTCTGATAACCAGATCTTGATTAAGGAA

CTCCATAATTCAGATTGCTGTTCAGATCAAGACATTGTCTGTATGTTGCATGAATGTCTG

GAATTCATGGAAGCAAATGAAATATCCTGGAAAGTTGTCAAAGTTGCAAGTTATGCAGTA

TCAGCTGCAAAGAGAGCAGCAAAAATGGCCATGAAGACTCACATGGATGTATTGATCTGG

GATCCTATTGACATTTAA

>NTK326_mRNA_79633_cds NTK326_mRNA_79633 gene_46232|id=AT5G17680.1

ATGGCATCATCTTCTGCTTCTGCGAGTACTTCACAGTATTTTTGGTGGAACTACAAAGTC

TTTCTAAATTTTAGAGGTGAAGATACTCGAAGGACATTTACAGATCACCTCTTCAGAGGC

TTGGAAAACAATGGAATATTCACGTTTCAAGATGATAAAAGGCTAGAGCATGGCGCATCA

ATATCATATGAACTCTTGAAAGCTATCGAACAGTCTCAAGTTGCCCTTGTCATTTTCTCA

AAGAATTATGCAACATCGAGGTGGTGCTTAGATGAGTTAGTAAAGATCATGGAATGCAAG

GATCAATACGGACAAACTGTCATACCAGTCTTCTATGATGTGGATCCATCACATGTTCGG

AAACAAAGGGAGAGCTTTGCTGAAGCCTTTGACAGACATGAAACAAGCTATAAGGATGAT

GATGAAGGAATGCAGAAGCTCCAAAGATGGAGGAATGCTCTAACTGTTGCCGCAAATCTA

AAAAGATATGATGTCCGTGACGGGATTGAAGCAGAGAATATTCAGCAGATTGTCGACCAA

ATTTCCAAATTGTGCAATAGTGCTACTTTGTCTTCTTTGCGAGATGTTGTGGGAATAGAT

ACTCAATTGGAGAAATTAAAGTCCCTACTTAAGGTAGGAATCAATGATGTTCGGATCATA

TTGGGGATCTGGGGCATGGGCGGTCTAGGGAAGACGACAATAGCAAGAGCCATTTTTGAC

ACTTTATCTCATCAATTTGAAGCTGCTTGTTTCCTTATGGATATTAAAGAAAATGAAAAA

AGACATCAACTGCATTCTTTGCAAAACACCCTTCTCTCTGAATTGTTAAGAAAAAAAGAT

GATTACGTCAATAATAAGCATGATGGGAAGCGGATGATTCCGGACAAACTTCGCTCTAAG

AAGGTATTAATTGTGCTTGATGATATAGATCATAAAGATCATTTAGAGTATTTAGCAGGT

GATCTTGTTTGGTTTGGTAATGGCAGTAGAATTATTGTAACAACTAGAGACAAGCATTTG

ATAGAGAAGAATGATGTAATATATGAAGTGACTGCACTACCTAATCATGAATCCATGCAA

TTGTTCAATCAGTATGCTTTCCGAAAAGAATTTCCAGATGAGCATTTTAAGGAGCTTTCA

TTGAAAGTAGTAAATTATGCTAAAGGCCTTCCTTTAGCCCTCAAAGTGTGGGGTTCTTTG

CTGCATAACCTAGGCTTAACTGAATGGAAAAGTGCAATAGAGCACATGAAAATTAATTCT

AATTCGAAAATTGTTGAAAAGCTCAAAATCAGTTATGATGGATTGGAGCCCATCCAACAA

GAGATGTTTCTAGATATAGCATGCATCTTGCGAGGGGAAGAAAAAGATTATGCCATGCAA

GTTCTTGAGAGTTGTCATATTGGAGCTGAATACGGATTGCGTATTTTAATTGACAAATCA

CTTGTGTTCATCTCTGAAAATGATGAGATTCAAATGCATGACTTTATAGAAGATATGGGT

AAATATATAGTGAACTTGCAAAAGAATCTGGGAGAACGCAGCAGATTATGGCTCCCCAGT

GATTTCGAAGCAGTGATGACCAACAATGCACATTTGCCGTCTCTACGGAGGATAGATCTC

AGCGGGTCTAAAAGACTGATGCGAACACCAGATTTCACGGGGATGCCGAATTTGGAGTAT

TTGAATCTGTTTTGTTGTTATAATCTTGAAGAAGTTCACCATTCCCTGGGATGTTGCAGC

AAACTCATTCAGTTAAATTTGAGTTTTTGTGATAGCCTTAAGAGGTTTCCATGTGTTAAC

GTGGAATCTCTTGAATATCTGGGTTTAAAATATTGCTATAGGTTAGAGAAATTTCCAGAA

ATCCACGGGAGGATGAAGCCGGAGATACAGATTGACATGCGAAGCTCTGGGATAAGGGAA

CTACCATCCTCTATTTTTCAGTACCAAACTCATATTACCGAGCTAGATTTGAGGCGTATG

AATAACCTTGTAGCTCTTCCAAGCAACATCTGTAGGTTGAAAAGTTTGGTTATTTTAGAT

GTGTCGGAGTGCTCAAAACTGGAAAGCTTGCCAGAAGAGATAGGGGATTTAGACAACTTG

GAGAAGCTTGATGCCAGTTGTACCCTAATTTCACGACCTCCGTCTTCCATCGCACGCTTG

AACAAACTTAAAGTCTTGGATTTTGGATTCGTACGGGATAGAGTGCACTTTGAGTTCCCT

CCAGTGGCTGAAGGATTACGCTCATTGGAAATTCTGAATCTCAGGTACTGCAATCTAATA

GATGGAGGACTTCCGGAAGACATTGGATCCTTATCCTCTTTGAAAGAATTGAATCTCCAT

GGAAATAATTTTGAGAATTTGCCTCGAAGCATAGCCCAACTTGGTGCTCTTCGATCCTTA

GACTTATCATATTGTCAGAGGCTTACACAGCTACCAGAACTTCCCCCTGAATTAAATATA

TTGCATGTAGATTATCATATGGCTCTGAAATTTATCCATGATTTAGTAACAAAGAGGAAG

AAACAACAGAGGGTGATATTCAAACCATTGTATTATAAGGATGATGCACACAATGATACT

ATATATAATTTGTTTGCACATGCCCTGTTTCAGAATATCTTTTCCTTGAGGCATGACATC

TCTGCTTCAGATTCCTTGTCCGAAAGTGTGTTTACCATTGTGCATATTGAGAAGAAGATC

CCAAGTTGGTTCCTCTATCAGGGAACGGATAGTAGTGTATCAGTCAATTTGCCTGGAAAT

TGGTATATACCTGATAAATTCTTGGGATTTGCTGTATGTTACTCTGGCAGATTAGTTTAC

ACCAAAACTCAATTGATTCCCGTATGTAATGACAGGATGTCGTGGATGACCCAGGAACTT

GGCTTATCCAACCCTTCAGAATCTGATTCAAAATATTATATACATTTTTTCTTTGTACCT

TTTGCTGTCTTATGGGATACATCTAAGGCAAAAGGAAAAACACCAAATGACTATGGGATT

ATTAGGCTATCTTTTTCTGGAGTAATAAAGAAGTATGGACTTCGTTTGTTGTATAAAGAT

GAACACCATGACACCGTGACCGATGCAGCTAGTTCCTGTCGCATACTCTAA

>NTTN90_mRNA_18119_cds mRNA_18119 gene_10151|id=AT5G36930.1:evalue=1e-164:annot='Disease resistance protein (TIR-NBS-LRR class) family';id=Solyc01g113620.1.1:evalue=0.0:annot='NBS-LRR resistance protein (Fragment)'

ATGGCCACTGAACTGAAGTCTCAAGTGTACTTGAGTTTCAAAGCGAAAGACACCGGCAAA

ATTTTTGCAGATCACCTCTATGAAGCTCTGGTGGGAGCAGGTTTTGTAACATTAAGAAGC

TGTGGTGATGAAAATGAGGGAGGTGAAGATATCAAGTTCAATTTGCAAAAGGGTATTAAA

GAATCTGGGGTTTCAGTTATAATCTTCTCAAATGATTACGTGTCCTCAAGTTGGTGTCTT

GATGAGTTGGTAATGATCTTGGATTGTAAAAAGATAGCAAAACGTGCAGTTCTGCCCATA

TTTTACCACGTGGATCCTTCTGATGTTAGGAAACAGAAGGGAAGAATTGGAGAAGCATTT

GATATGGACAAAGAACTGGGAGGGAATCAAGGTGAAAATGAGAGGGTCAGAAAATGGAGG

GAAGCACTCAAAGAAGTTGCAGACTTGGGAGGAATGGTCTTACAAAACCAAGCTGATGGA

CACGAGTCCAAATTCATCCAGAAGATTCTTAAAGTGGTTGAGAATAAACTGAGCAGGCCA

GTCCTGTATATTTGCCCTCATCTGATTGGAATAGAACGGCGTGTTGAAAAGATCAACTTG

TGGCTAGAGGATGGATCTATTGATGTTGACACTCTTGTTATTTGTGGCATCGGTGGAATA

GGCAAGACAACAATGGCAAAGTTTGTGTATAATTTGAACTTCAGTAAGTTTGATGGTAGC

AGCTTTTTGTCCAACATTAGAGAAAATTCAACACACCGTAAAGGTTTAGTTACTCTTCAA

AGGCAATTTCTTTCTGATATTTGCAAAAGAAAGAAGAAAGCTATGTTTTCCGTGGACGAG

GGAATGACTGAGATGAGAGAGGCTGTACAGTGTAAAAGAATCCTTCTTGTTCTTGATGAT

GTAGATAATCGTGATCAAGTGGATGCTCTACTGGGAATGAAGGACTTGTTATATCCTGGT

AGTAAAGTCATTGTGACAACTAGGAACAAGAGATTGCTTAGGCCTTTTGATGTGCATAAG

ATTTATGAGTTTGAAGCATTGAATAGAGATGAATCGGTTGAGCTCTTAAGTTGGCATGCA

TTTGGTCAAGATTGTCCTATTAAAGGTTTTGAAATGTGTTCAGAACAAGTAGCAATCCAT

TGTGGAGGACTTCCATTAGCACTTGAAGTTCTTGGTGCTACTTTGGCAGGAAGAAACATA

GACATTTGGAAAAGTACAATACAGAAATTGGAAACAATTCCGAATCATCAAATTCTCAGG

AAATTAACAATAAGTTACGAATCTCTTGAGGATGATCATGATAAGAATTTATTTCTCCAC

CTAGCTTGCTTTTTCATTGGGAAGGACAGAGATCTAGCAGTAGCTATTCTCAATAGGTGC

AACTTTTACACTGTAATTGGAATTGAGAATCTCATTGACAGAAATTTTATAAAAGTTGGT

AAGTCTAACAGCTTGATTATGCATCAAATGATTCGAGATATGGGAAGAGACATTGTTCGC

CAAGAATCACCACTGGAGCCTGGGAAACGCTCTAGACTATGGCGTTCAAAGGATTCCTTT

AACGTCTTAATCCAGAACCGTGCCACTCAAACAATTCAAGGCATTATTCTTGACATGGAT

ATGCTCAAGGAAAGTGACATAGTTAGCTCAAGCTTTTTCGCCAAGGATTTCAAGAAACAC

AAAATAAAAAACTTTCTCAACTATCCTAATCCTCAGAGAGTTCAATTCAAACAGAAAAGG

TTTGTTTTTTTCCCATGGCATTTGTCAGATGCCAAAGAAGCCACAAATGAGCTGGTTCTG

GGAACTGATGTATTTGCAAATATGCAAAAGTTAAAACTGCTCCAATTCGATCACGTTGAG

CTTCAAGGATCTTTTGATGTTTTTCCTAAGAGATTAAGATGGTTGCGCTGGTCTGAGCTG

CAACTTGAGTGCATGCCAATTGATTTTCCTCTGGAGAGCCTTGTAGTGATTGAATTACAC

CGTAGCAGCTTGAGGAGGATTTGGCATGGAGTCAAGTTCCTTAAAGATCTGAAGATTTTC

GATCTCAGCCATTCCTACGAGCTTCTAAGAACACCTGATTTTTCAGGACTCCCCAATCTT

GAAAAGTTGATCCTTCGATATTGTACAAGCTTGATTGAGCTTCATGAGACCATCGGGTGT

CTAGAATCACTTATTCTTTTGAATCTCAAAAATTGCAAAAATCTCCAGAGACTTCCAGAT

AGCATTTGCATGCTAAAATGTCTGGTGACACTAAATATCTCTGGTTGCTTGAATCTTGAA

TATGTGCCGATGGATCTAGATAAAATGGATTCACTGAGAGAGCTTTATGCTGATGAAATT

GCAGTTCACCAAATGATTTCTACTCCAGAAGAGGTCCAACCGTGGTATGGATTTCTGCGG

TCCTGGATGCTGAAGGGGAAAATATGTCCTAAAGTTTCACATATTAGTTTACCTAATTCC

TTGGTTACTCTGAGTCTTGCTAACTGTAATCTATCCAATGATGCTTTTCCAGTTGCTTTC

AGTAGCCTCTCCTTATTGCAAAACTTAGATTTGAGCGAAAATCCAATTTGCTGCCTACCA

AAGGGCATAATTTATCTCACCGGTCTTCAGAAGCTTGAAGTGGAAGGCTGTGAAAAGCTC

AGATCGCTCGTAGGGCTTCCCAATGTAGAACATCTCAATGTTACTAATTGCTGGTCGTTA

GAGAAAATATCATATCAATCAAGATCATCTAGACTGAAGGATTTACTTGTGTCGAATTGT

GCTAAATTAGTTGAAATAGATGGAAATTTCAAGTTAGAGCCCTTAAGAAATACTGAGGCA

GAGATGCTTTGCAAGTTGGGCTTGTCGAACTTAGCTTCTATGGATAATGTCATGATCAAT

CTTACATCTAATATCCTGAGTTACTACCGAATACATGGTAAAGGATGGACTCCAACAAGG

AAGACAAAGAAAGTTGTTCTTCAGGTATGTCTTGCTCGTTCTTTCATTGTTTCAGTTTTG

TGA

>NTK326_mRNA_93052_cds NTK326_mRNA_93052 gene_54896|id=AT1G27170.1

ATGGAGGAAATTGAGCAGGCCACAAAAACTTCACTCCCATCGCTAAGGCTAAATTACGAC

GTGTTCTTGAGTTTCAGAGGCGAAGATACTCGCGAAAACATCACTAAAAACTTATACGAT

GCCTTATACTCAAAAGGCGTCCGAGTTTTTCGTGACACAAACGGGTTAACTCAGGGCGAC

GAGATCGCTCCAGGTCTTATGGACGCAATCAACGATTCAGCTGCAGCTATTGCTATTATT

TCACCCAATTATGCTTCGTCGAGATGGTGTCTCGAGGAATTAGCTACGATTTGTGAGTTG

AGTAAACTCGTTCTGCCCGTTTTCTACCGGGTTGACCCGTCGGATGTGAGAAGGCAGAGA

GGACCATTTCTACGTGATTTTGCGAGTTTAGAAGGAAGGTTTGGAGTGGAAAAGGTGGTG

AGATGGAGAAACGCTATGGAAAGAGTTGGTGGAATCTCCGGCTGGGTTTATTATAATCGT

GAAGAGTCACAGTTGATACAGAATTTGGTGAAAAGAGTTTTACAAGAATTGAGCAATTCC

CCAATATTTGTAGCTCCATTTGTTGTTGGAATTGACTACCGCCTGGAAGAACTCATAAGA

CAGTTAGATGTGAAGCACAATGGTGTCAAGATTATTGGGTTGCATGGAATCGGAGGGGTT

GGTAAAACAACTCTTTCTAAGGCTGTTTATAATAAACTTGCTTCTCATTTCACACACAGG

ACTTTTATCTTGAATGTTAAGGAAATAGCTGCTCAACAAGGCATTGTGTCCCTTCAGAAG

AAAATAATACAAGGTCTTTTCCCGAGCAAGGTCTTCTCCTTCTCCCCCAGTAATGCACAT

GAAGGAAGAGTAAAATTCGGACGATTTCTTCAAGAAAAGCGCGTCCTGCTTGTCTTAGAT

GATGTAGATTATGTAAATGATGATGTTAACATACTGAAGGCACTAATTGGAGGGAAAAAC

TGGTTCTTTGAAGGGAGCAGGGTTGTTATTAGTACTAGAAACAGAGGAATTTTGCTAAAT

GACATCGTTAACGAGACAATTGAGGTGAGAGAATTGGGTGATACTGACTCACTAAAACTA

CTCAGTTATCATGCATTTAGAAGACATGAGCCATTTCCAGCTTTTGTGAATATTTCCAAG

CAAATTGTCTCAATTACTGGAGGGCTACCGTTGGCTCTTGAAGTTTTTGGGTCTTTCTTG

TTTGATAAAAGAAGCGAGGAGGAATGGATAGATGCTCTAGAAAAGCTAAAACAAATTCGC

TCACCGCGTCTTCAGGACATCTTGAAAATAAGTTATGATGGTCTTGATGACGAAGAGAAG

TGTATATTCCTGGATGTTGCATGTTTATTTCTTGATCAATTAGAAAAGAAAGCTGAAGAT

ATAATTGATGTGATGAAAGGATGTGGTTTCAGAGCCAGGGTTGCATTTGACACTTTAACT

GCTAGATCATTGATTAAGGTAATTGATGGTGGGGATTTGTGGATGCATGACCAGATAAGA

GATATGGGAAGACAAATTGTTATACAACAAGGCATTTCAGATCCCGGAAAGCGCAGCAGA

CTTTGGGATGTTGCTGATGTTTTGAGTGTGTTACAAGGAAGGAAGGGGACACAGAACATC

CAAGGGATCATCCTGGATCTGTATCAGAATTCATCATCAAAGATTAAAAGCGCAAAAGCA

ATTACTAGAGAGCATTTTCAACAAGTTCCCACTTTTACTTCTGCATTAGCTTACATTAAA

GAGTTGTGCAAAGAACAATTTCAAAATGATGCAAAAGAAACTAATGAGTTGGTATTGAAC

AGTGAAGTATTTGATCCAATAGTTAATCTGCGACTACTCCAATTCGATAATGTGAAACTA

GAGGGAAATTTGGGGAAGTTACCTTCTTCACTAAAATGGCTCCACTGGAAAAGATGCACT

CTTTCAAGTTTTTATTCTAATGATTATCCAAGTGAACTTACCATACTTGATCTCTCAGAG

AGCCAAATAGAGAGGGTTGGAAGCCGGGAATGGACGTGGAATTGCAAAAAGGTGGCGAAC

AAGTTGATAGTTATGAATCTCTCTGATTGTCATAAAATAACAGTTATTCCTGATTTATCC

ACGCATAAAGCATTGGAGAAGTTGATAGCTGAGCGTTGCAGTGCATTGCAAAGGATTCAC

AGAACAATTGGGAATCTGAATACATTACGTCATTTAAATCTAAGAGATTGCCGCAACCTT

GTTGAATTTCCAGGTGAGGTCTCCGGGCTGAAGAATCTTGAAAAGCTGATACTCTCGGGT

TGCTCGAGATTGAAACAGCTACCTGAAGATATAGGTAAGATGAAGTCTTTACAAGAACTT

CTATTAGATGGAACTGCTATTGAGAAGTTGCCTGAAAGTATCTTCCGCTTAACAAAACTT

GAGAAGTTAAGCTTAATCCAGTGCCACTCATTGAAACAACTTCCCCGGTTCATAGGAAAG

CTAAGTGCTTTGAAGGAACTCTCTCTTAATGGTTCTGCTGTGGAAGAAATACCTGATTCT

ATTGAACATTTGCAGAACCTTCATACATTAAACTTAATTAGGTGTGAGTCACTTTCTGCT

ATTCCCAGTTGTGTTGGCAACCTCAAATCTTTAGTAAATCTCTGGCTTTATGGCAGTGCA

ATAAAAATGATGCCAGAATCTATTGGGTCTCTGTATTATCTTAGGTCCTTATCGCTCGGA

AATAGTCAGCATTTGAATGCATTGCCTGTTTCAATTAAAGGATTGTCTTCTTTGGTTGAG

CTTCAAATAGAAAAGGTTCCAATTATTAGTCTTCCAGATCATGTTTTTGGTGGACTTAAA

TCACTGAAGAATCTTGAGATAAGGAACTGTGAGCGCCTTGGCTCGCTTCCCCACTCCATT

GGAGAATTGTTAGCTCTTAGAACAATGACTCTTACCAGAAATGATGCTATTAAGGAGCTG

CCAGAATCAGTTGGGAATTTGCAGAATCTTGTCATACTGAGATTGACCAGATGTAAGCGA

CTTTGCAAATTGCCAGCTTCAATTGGGAAACTAAAGAACTTAGTACACCTGCTAATGGAG

GAGACTTCAGTAACAAAATTACCTGAAACATTCGGGATGCTATCGAGCTTAATGATTCTG

AAGATGGGAAAGAAGCCTTTCTGCCAGGTATCACAAAGTACTGAAACCACAAAACCAGCT

ACCTACACAGAAAGGGAAACAGCACCTGTTGTGCTTCCTTCGTATTTCTCAGAGCTATCC

ATGTTACAAGAACTTGATGCCCGTGCATGGAGAATAGTTGGGAAAATACCGGATGATTTT

GAGAAACTATCATCTTTGGAGATCATCAATCTTGGTTTCAATGATTTTTCCCATCTCCCG

TCTAGTCTGAAAGGACTACCTTTCTTGAAAGAGCTCCTTATTCCCCACTACAAACAGTTG

AAAGCTATTCCTCCTCTTCCCTCAAGTTTGCTCAAGATAAATGCTGCAAACTGTGGAGCC

CTAGAGAGTATGTACGATATCTCAAGATTAGAGTTCTTGCGCGAGCTAAACCTTGCAAAT

TGCATGAGTTTGGTAGACATCCAAGGTATCGAATGCTTGAAATCCTTAAGAATGCTACAT

ATGGCTGGTTGCAATGTCTCCTGTGCCTCTATTGTTAGAAGCAAACTTGATAAGGTTGCT

GTGAAAAACTTGTATAATTTTAGCATTCCAGGCAGTGAAATCCCAAGTTGGTTAACTCCA

AGCGAGGTGCATTTCTCAAGGCACAGAAACAATGAAATTAAAGCAGTGGTTATTGCCATA

GTTGTGTCAGTGAACTGTGCTAAACTAGATGATTTAAGGGATGAATTGCCTGTAATAGCT

AACATCCATGCAAAAATCGTTAGAGCAAATCGAGCAGTATATACTACTGGTATGTACTTG

GTAGGAGTCCCAACTACGCCCGAGGATCAAGTTTACTTGTGCAGGTATCGAGATTATCAT

CCATTAGTATCTATACTCGAGGATGGTGATATAATACAGGTGGGATTGGGCAACTTTCCC

ATTACAGGGATTGAACTAAAGAAGTGTGGAATACATTTAGTTTACGAAAGTGATGATGAT

TATGAAGGTAATGAAGAATCATTGGATGAAAGCCAACAATCTGTATCAGAAAGATTAACA

AGGTTTATTGGAGCTTCTAATAGAGAGAGCAATGTCTTCAGCTCGAACTCAGCTCAAGAA

GATGGAGAAGAGGAAAGAAGGCATAACTGTTTTAGTTTCGTTAAGGAGATTTTCCGTGCT

TTAAAGTACCTTCTCTTTAGGCGATTTGCATCCTTCCTTCTAGCATGA

>NTK326_mRNA_15737_cds NTK326_mRNA_15737 gene_8943|id=AT5G36930.1

ATGGCCACTGAACTGAAGTCTCAAGTGTACTTGAGTTTCAAAGCGAAAGACACCGGCAAA

ATTTTTGCAGATCACCTCTATGAAGCTCTGGTGGGAGCAGGTTTTGTAACATTAAGAAGC

TGTGGTGATGAAAATGAGGGAGGTGAAGATATCAAGTTCAATTTGCAAAAGGGTATTAAA

GAATCTGGGGTTTCAGTTATAATCTTCTCAAATGATTACGTGTCCTCAAGTTGGTGTCTT

GATGAGTTGGTAATGATCTTGGATTGTAAAAAGATAGCAAAACGTGCAGTTCTGCCCATA

TTTTACCACGTGGATCCTTCTGATGTTAGGAAACAGAAGGGAAGAATTGGAGAAGCATTT

GATATGGACAAAGAACTGGGAGGGAATCAAGGTGAAAATGAGAGGGTCAGAAAATGGAGG

GAAGCACTCAAAGAAGTTGCAGACTTGGGAGGAATGGTCTTACAAAACCAAGCTGATGGA

CACGAGTCCAAATTCATCCAGAAGATTCTTAAAGTGGTTGAGAATAAACTGAGCAGGCCA

GTCCTGTATATTTGCCCTCATCTGATTGGAATAGAACGGCGTGTTGAAAAGATCAACTTG

TGGCTAGAGGATGGATCTATTGATGTTGACACTCTTGTTATTTGTGGCATCGGTGGAATA

GGCAAGACAACAATGGCAAAGTTTGTGTATAATTTGAACTTCAGTAAGTTTGATGGTAGC

AGCTTTTTGTCCAACATTAGAGAAAATTCAACACACCGTAAAGGTTTAGTTACTCTTCAA

AGGCAATTTCTTTCTGATATTTGCAAAAGAAAGAAGAAAGCTATGTTTTCTGTGGATGAG

GGAATGACTGAGATGAGAGAGGCTGTACAGTGTAAAAGAATCCTTCTTGTTCTTGATGAT

GTAGATAACCGTGATCAAGTGGATGCTCTACTGGGAATGAAGGACTTGTTATATCCTGGT

AGTAAAGTCATTGTGACAACTAGGAACAAGAGATTGCTTAGGCCTTTTGATGTGCATAAG

ATTTATGAGTTTGAAGCATTGAATAGAGATGAATCGGTTGAGCTCTTAAGTTGGCATGCA

TTTGGTCAAGATTGTCCTATTAAAGGTTTTGAAATGTGTTCAGAACAAGTAGCAATCCAT

TGTGGAGGACTTCCATTAGCACTTGAAGTTCTTGGTGCTACTTTGGCAGGAAGAAACATA

GACATTTGGAAAAGTACAATACAGAAATTGGAAACAATTCCGAATCATCAAATTCTCAGG

AAATTAACAATAAGTTACGAATCTCTTGAGGATGATCATGATAAGAATTTATTTCTCCAC

CTAGCTTGCTTTTTCATTGGGAAGGACAGAGATCTAGCAGTAACTATTCTCAATAGGTGC

AACTTTTACACTGTAATTGGAATTGAGAATCTCATTGACAGAAATTTTATAAAAGTTGGT

AAGTCTAACAGCTTGATTATGCATCAAATGATTCGAGATATGGGAAGAGACATTGTTCGC

CAAGAATCACCACTGGAGCCTGGGAAACGCTCTAGACTATGGCGTTCAAAGGATTCCTTT

AACGTCTTAATCCAGAACCGTGCCACTCAAACAATTCAAGGCATTATTCTTGACATGGAT

ATGCTCAAGGAAAGTGACATAGTTAGCTCAAGCTTTTTCGCCAAGGATTTCAAGAAACAC

AAAATAAAAAACTTTCTCAACTATCCTAATCCTCAGAGAGTTCAATTCAAACAGAAAAGG

TTTGTTTTTTTCCCATGGCATTTGTCAGATGCCAAAGAAGCCACAAATGAGCTGGTTCTG

GGAACTGATGTATTTGCAAATATGCAAAAGTTAAAACTGCTCCAATTCGATCACGTTGAG

CTTCAAGGATCTTTTGATGTTTTTCCTAAGAGATTAAGATGGTTGCGCTGGTCTGAGCTG

CAACTTGAGTGCATGCCAATTGATTTTCCTCTGGAGAGCCTTGTAGTGATTGAATTACAC

CGTAGCAGCTTGAGGAGGATTTGGCATGGAGTCAAGTTCCTTAAAGATCTGAAGATTTTC

GATCTCAGCCATTCCTACGAGCTTCTAAGAACACCTGATTTTTCAGGACTCCCCAATCTT

GAAAAGTTGATCCTTCGATATTGTACAAGCTTGATTGAGCTTCATGAGACCATCGGGTGT

CTAGAATCACTTATTCTTTTGAATCTCAAAAATTGCAAAAATCTCCAGAGACTTCCAGAT

AGCATTTGCATGCTAAAATGTCTGGTGACACTAAATATCTCTGGTTGCTTGAATCTTGAA

TATGTGCCGATGGATCTAGATAAAATGGATTCACTGAGAGAGCTTTATGCTGATGAAATT

GCAGTTCACCAAATGATTTCTACTCCAGAAGAGGTCCAACCGTGGTATGGATTTCTGCGG

TCCTGGATGCTGAAGGGGAAAATATGTCCTAAAGTTTCACATATTAGTTTACCTAATTCC

TTGGTTACTCTGAGTCTTGCTAACTGTAATCTATCCAATGATGCTTTTCCAGTTGCTTTC

AGTAGCCTCTCCTTATTGCAAAACTTAGATTTGAGCGAAAATCCAATTTGCTGCCTACCA

AAGGGCATAATTTATCTCACCGGTCTTCAGAAGCTTGAAGTGGAAGGCTGTGAAAAGCTC

AGATCGCTCGTAGGGCTTCCCAATGTAGAACATCTCAATGTTACTAATTGCTGGTCGTTA

GAGAAAATATCATATCAATCAAGATCATCTAGACTGAAGGATTTACTTGTGTCGAATTGT

GCTAAATTAGTTGAAATAGATGGAAATTTCAAGTTAGAGCCCTTAAGAAATACTGAGGCA

GAGATGCTTTGCAAGTTGGGCTTGTCGAACTTAGCTTCTATGGATAATGTCATGATCAAT

CTTACATCTAATATCCTGAGTTACTACCGAATACATGGTAAAGGATGGACTCCAACAAGG

AAGACAAAGAAAGTTGTTCTTCAGGTACTGTACCAACCAGGTGTCTTTAGCACTTTTCTG

CCAGGTGAACATGTACCTTCTTGGTTCAGCTCAAAATACACAAAAGAATCACATACATCC

TTCAAAGTGCCTACTTGTACTTCCACGATTGAAGGCTTGAGTTTTTGCATTGTGTACAAG

CGTTCCGTATTTGGTCTAAGTGCTCATCGTCCCCCGCGCCTAACTCCGCCTTCAAGAATA

GCTCCTCTTGCCATGCACAAAGCTCAAAGAGGACCCATTCGGTATCGGCCAGTGGAAAAT

AAACCATATGAATCAACCTTTGACTGCCCGTGCATTACTGTTAATAACTTAACTCGGAGT

GTGAAATGGTCTTACCAGCCCTTGTTCTATGGAGTTCCGGAAGGGAAAGAAGGAATGATG

TGGTTAAGCCATTGGAAACTTGAGAATCAGTTGGGCAGTGATGATATACTGGAGATCACA

GTTACCTCAGGAGATGGAATCAGAATTGTGGAGTTTGGGCTCAAAATTCTGCATGTTGAA

GGGCCAAATGTGCAAATAGGAGAACCAAGTTGTGAAGATGCAAGGGGAGAGAAAGATATT

GTCAATCCATTTTGGGATGTTGTTTTAAAAGATGCTAGTTCAAAGAATACTTGTTCTGTT

CGGCTTCCTCCTACATATCGTCCCCTACGTGTTGCTCGTGAGCCATTTCTGGAGAAGGCG

CTGAAAAGAAATATGTCAGACTATAACTAG

>NTTN90_mRNA_13877_cds mRNA_13877 gene_7792|id=AT1G27170.1:evalue=3e-09:annot='transmembrane receptors';id=Solyc05g006620.2.1:evalue=9e-15:annot='Tir-nbs-lrr, resistance protein'

ATGGAGGAAATTGAGCAGGCCACAAAAACTTCACTCCCATCGCTAAGGCTAAATTACGAC

GTGTTCTTGAGTTTCAGAGGCGAAGATACTCGCGAAAACATCACTAAAAACTTATACGAT

GCCTTATACTCAAAAGGCGTCCGAGTTTTTCGTGACACAAACGGGTTAACTCAGGGCGAC

GAGATCGCTCCAGGTCTTATGGACGCAATCAACGATTCAGCTGCAGCTATTGCTATTATT

TCACCCAATTATGCTTCGTCGAGATGGTGTCTCGAGGAATTAGCTACGATTTGTGAGTTG

AGTAAACTCGTTCTGCCCGTTTTCTACCGGGTTGACCCGTCGGATGTGAGAAGGCAGAGA

GGACCATTTCTACGTGATTTTGCGAGTTTAGAAGGAAGGTTTGGAGTGGAAAAGGTGGTG

AGATGGAGAAACGCTATGGAAAGAGTTGGTGGAATCTCCGGCTGGGTTTATTATAATCGT

GAAGAGTCACAGTTGATACAGAATTTGGTGAAAAGAGTTTTACAAGAATTGAGCAATTCC

CCAATATTTGTAGCTCCATTTGTTGTTGGAATTGACTACCGCCTGGAAGAACTCATAAGA

CAGTTAGATGTGAAGCACAATGGTGTCAAGATTATTGGGTTGCATGGAATCGGAGGGGTT

GGTAAAACAACTCTTTCTAAGGCTGTTTATAATAAACTTGCTTCTCATTTCACACACAGG

ACTTTTATCTTGAATGTTAAGGAAATAGCTGCTCAACAAGGCATTGTGTCCCTTCAGAAG

AAAATAATACAAGGTCTTTTCCCGAGCAAGGTCTTCTCCTTCTCCCCCAGTAATGCACAT

GAAGGAAGAGTAAAATTCGGACGATTTCTTCAAGAAAAGCGCGTCCTGCTTGTCTTAGAT

GATGTAGATTATGTAAATGATGATGTTAACATACTGAAGGCACTAATTGGAGGGAAAAAC

TGGTTCTTTGAAGGGAGCAGGGTTGTTATTAGTACTAGAAACAGAGGAATTTTGCTAAAT

GACATCGTTAACGAGACAATTGAGGTGAGAGAATTGGGTGATACTGACTCACTAAAACTA

CTCAGTTATCATGCATTTAGAAGACATGAGCCATTTCCAGCTTTTGTGAATATTTCCAAG

CAAATTGTCTCAATTACTGGAGGGCTACCGTTGGCTCTTGAAGTTTTTGGGTCTTTCTTG

TTTGATAAAAGAAGCGAGGAGGAATGGATAGATGCTCTAGAAAAGCTAAAACAAATTCGC

TCACCGCGTCTTCAGGACATCTTGAAAATAAGTTATGATGGTCTTGATGACGAAGAGAAG

TGTATATTCCTGGATGTTGCATGTTTATTTCTTGATCAATTAGAAAAGAAAGCTGAAGAT

ATAATTGATGTGATGAAAGGATGTGGTTTCAGAGCCAGGGTTGCATTTGACACTTTAACT

GCTAGATCATTGATTAAGGTAATTGATGGTGGGGATTTGTGGATGCATGACCAGATAAGA

GATATGGGAAGACAAATTGTTATACAACAAGGCATTTCAGATCCCGGAAATCGCAGCAGA

CTTTGGGATGTTGCTGATGTTTTGAGTGTGTTACAAGGAAGGAAGGGGACACAGAACATC

CAAGGGATCATCCTGGATCTGTATCAGAATTCATCATCAAAGATTAAAAGCGCAAAAGCA

ATTACTAGAGAGCATTTTCAACAAGTTCCCACTTTTACTTCTGCATTAGCTTACATTAAA

GAGTTGTGCAAAGAACAATTTCAAAATGATGCAAAAGAAACTAATGAGTTGGTATTGAAC

AGTGAAGTATTTGATCCAATAGTTAATCTGCGACTACTCCAATTCGATAATGTGAAACTA

GAGGGAAATTTGGGGAAGTTACCTTCTTCACTAAAATGGCTCCACTGGAAAAGATGCACT

CTTTCAAGTTTTTATTCTAATGATTATCCAAGTGAACTTACCATACTTGATCTCTCAGAG

AGCCAAATAGAGAGGGTTGGAAGCCGGGAATGGACGTGGAATTGCAAAAAGGTGGCGAAC

AAGTTGATAGTTATGAATCTCTCTGATTGTCATAAAATAACAGTTATTCCTGATTTATCC

ACGCATAAAGCATTGGAGAAGTTGATAGCTGAGCGTTGCAGTGCATTGCAAAGGATTCAC

AGAACAATTGGGAATCTGAATACATTACGTCATTTAAATCTAAGAGATTGCCGCAACCTT

GTTGAATTTCCAGGTGAGGTCTCCGGGCTGAAGAATCTTGAAAAGCTGATACTCTCGGGT

TGCTCGAGATTGAAACAGCTACCTGAAGATATAGGTAAGATGAAGTCTTTACAAGAACTT

CTATTAGATGGAACTGCTATTGAGAAGTTGCCTGAAAGTATCTTCCGCTTAACAAAACTT

GAGAAGTTAAGCTTAATCCAGTGCCACTCATTGAAACAACTTCCCCGGTTCATAGGAAAG

CTAAGTGCTTTGAAGGAACTCTCTCTTAATGGTTCTGCTGTGGAAGAAATACCTGATTCT

ATTGAACATTTGCAGAACCTTCATACATTAAACTTAATTAGGTGTGAGTCACTTTCTGCT

ATTCCCAGTTGTGTTGGCAACCTCAAATCTTTAGTAAATCTCTGGCTTTATGGCAGTGCA

ATAAAAATGATGCCAGAATCTATTGGGTCTCTGTATTATCTTAGGTCCTTATCGCTCGGA

AATAGTCAGCATTTGAATGCATTGCCTGTTTCAATTAAAGGATTGTCTTCTTTGGTTGAG

CTTCAAATAGAAAAGGTTCCAATTATTAGTCTTCCAGATCATGTTTTTGGTGGACTTAAA

TCACTGAAGAATCTTGAGATAAGGAACTGTGAGCGCCTTGGCTCGCTTCCCCACTCCATT

GGAGAATTGTTAGCTCTTAGAACAATGACTCTTACCAGAAATGAT

>NTTN90_mRNA_81516_cds mRNA_81516 gene_46122|id=AT5G17680.1:evalue=2e-05:annot='disease resistance protein (TIR-NBS-LRR class), putative';id=Solyc07g055380.1.1:evalue=0.0:annot='Nbs-lrr, resistance protein'

ATGAGAGCAATTGAAGAGTCGCGCATAGCTTTGATTATATTCTCCAGAAACTATGCTAAT

TCGATATGGTGCTTAGATGAATTAGTGAAGATCATGGAATGCAAGAACTTGAATGGACAA

ATTGTGTTTCCGGTCTTCTACGATGTAGATCCATCAACAGTGAGGAAACAAAAGTCAAGC

TTTGGAGAAGCATTTAGCAGTCATGAAGCCCATGGCTGTTTCAAGTTGCAAAAATGGAGG

GCGGCATTGGAGGAAGCTGCTAATTTATCTGGCTGCGATTTGCCAAATACTGCTAATGCG

CATGAAGCTAAAGTCATAAAGCAAATTGTGGAAGATATACTGGCTAAATTGGGTGGTCAG

AGGCATGCAATCAATGCTGAAAATCTTGTTGGAATGGAGTCACAAATGCAGAAAGTGTAT

AAAATGCTTGGCATCGGTTTTGGAGGAGTTCACTTCGTTGGAATATTTGGAATGAGCGGA

GTGGGAAAGACAACTTTAGCGAGAGTCATTTATGATAACATTTCAAGTCAATTTGAGGGT

GCTTGTTTTCTTCATGAGGTTAGAGACCGTTCAGAAAAACAAGGCCTAGCGCGATTGCAA

GAGATACTTCTTTCCAAGATCCTTGTCATAAAAGATCTAAGGATCAACAATTTATTTGAA

GGAGTTAATATGCATAGACATAGATTACGGTACAAAAAGGTTCTTCTTGTTCTTGATGAT

GTTGATCACATAGATCAGTTAGAGGTTTTAGCTCAGAAGCGTGAATGGTTTGGTTCTGGA

AGTAGAATCATCATAACAACTAAAGACAAACACTTGCTTGTTAAGCATGATGTGGAAAAG

ATATACAAAATGAGAACATTAAGTGACGATGAAAGTCTAGAACTATTTAAACAATATGCT

TTCAAGAAGAACCATCCTACCAAGAAATTTGAGGATCTCTCAGCTCAAGTGATAAAGTAT

ACTGCTGGACTCCCCTTGGCTCTGAAGGTCCTGGGCAGTTTCTTGTATGGAAGAGATTTG

GCTGAATGGAGAAGTGAAGTGGAACGATTGAAACAAATCCCGGAAGATGAAATTTTGAGG

AAACTCGAACCAAGTTTCACTGGACTCAAAAAAGGTAGGATTTTAATGCACCAATTGATA

CAAGAAATGGGATGGCACATTGTTCGTCGAGAAGCTTTCGATTATCCAAGAAGATATAGT

AGGTTATGGAAGTCTGAAGATATTTCTCATGTACTTGCAAGAAATATGGGCACAGAAAAG

ATCGAAGGCATATCTCTGAACTTGAGAAAGATGCTCACAGATATTTCTCATGCACTTGAA

AGAAATTTGGGCACAGAGAAGATCAAAGGGATACCATTAAACTTGACCAATGTCAAAGAA

GTGAATGTTAGTGCAACAGCCTTCATGCCGATGACCAGACTGAGGTTTCTCAAAATCAAG

AATGCATATGTTTCTCAGAGTCCTGATATTCTTCCTAGTGAGTTGAGCTGGCTTTCTTGG

CACGGATATCCTTCAAAAAGTCTTCCAATTAGCTTTCAGGGAGAACGACTCGTTAGTTTG

AAGTTAAAAAATAGTCGCATCATACAACTTTGGAAAGGCTCCAAGGTTCTAGGACAACTG

AAGTACATCAACCTTAGCCATTCACATAAGCTAATAAGGACTCCAGATTTTTCGGGTACC

CCTAATCTTGAAAGGTTGGTTCTTGAAGAGTGCACAAGTTTGGTAGAAATCAATTTTTCT

GTTGGAGATCTCAAAAAGCTAGTCTTGCTCAAGTTGAAGAACTGCATCAATTTAAAGACC

CTGCCAAAGAGTATTCAATTGGAAAATCTTGACGTTCTTATTCTATCAGGCTGCTCAAAG

CTAAAAGTATTCCCAGAAATAGAAGAGGAAATGAATCGTTTATCAGAACTATATTTGGAA

GCGACTGCTTTTAGTGAACTACCCGCATCAGTTGAGAAACTATCAGGAGTTAAAGTGATA

AATCTAAGCTCATGCAAGAATCTTGAGAGTCTTCCAAATAGTATTGTTAGGTTGAAATAT

CTTAAAGAACTTAATGTGTCCAAATGCTCAAAACTTAAAAGTTTACCAGATGACTTGGGT

TCTTTAGTCAGTTTGGAGGGGCTCCATTGTGATGACACACCGATCCAAATGATACCCTCC

ACCATTTCCCTTCTAAAGAACCTTAAGCACTTATCTCTCCGTCAATGTAATGCTTTAGGT

TTGCACGTAAGGAGTTCAATCTCAAGAGAATCTATGGGACTAGTTTTCTCTAATTTATCA

GGTCTTTGTTCATTGACAATGCTGGATATAGGTGGCTGCAGCATTTCAGATGGAGGCATC

CTATGTAATCTTGGGTTCTTACCATCTTTGGCGGAATTGAATCTTGGTGGTAACACATTT

ACTAATATCTCAGCTTCAAGCATCAGTGGTCTGACTCGACTAAAGGTTCTTCAATTGGTT

GGCTGTAGTAGGCTTGAACATTTCCCAGAGCTTCCTCGAGCTATAGAAGAGGTGCATGCT

GATGAGTGTATATCTTTGAAGAGTATCCATCAATTAGCAAAATATCCAACATTGCGCCGA

CTTTCACTTAGCCAATGTCATCAGCTTCATGATACTGACATGGTTGATGCATTATGGAGC

AACATGCTCAAGGGACTATACGTGCTACGAAATGATCTCAGCATTTGCATCCCTGGATCG

CAGATTCCTATGTGGTTTACATACAAGAACTTTGGGGAAAATGTTACACTGACTCTTGCC

AATAATTGGTACACTGATAACCTCTGGGGTTTTGCTTTCTGTATTGTTTTTGAACGTATG

GAATGGTGCGGTCTATATGATGGTTACCTACAACCATCACTTGGATTTCCAGTTAACCTT

AAATTCAAAACATATGATGGTAAGGAAGGCGATATACGTAGCATTATTGGCATAAAAGGA

GGTGATATGTCAATTCGGAACTCAGAGCACACTCTCCTTGCCTACGTACCATCTCGTCGT

TTTCTGCAACCTTACAATAACGAGGTTTACAGTCCCAACGACTGGATAGAAATTGTGGCT

TATTCTACAGTACAATTCGACAGCAAAGCTTGGGGAACGCGTCTTGTGTACTTGGACGAT

ATTATTGAAGCATGA

>NTTN90_mRNA_17803_cds mRNA_17803 gene_9991|id=AT5G17680.1:evalue=1e-10:annot='disease resistance protein (TIR-NBS-LRR class), putative';id=Solyc11g011090.1.1:evalue=0.021:annot='Tir-nbs-lrr, resistance protein'

ATGGCATCATCTTCTGCTTCTGCGAGTACTTCACAATTTCCTTGGTGGAACTACAAAGTC

TTTCTAAGTTTTAAAGGTGAAGATACTCGAAAAACATTTATAGGTCACCTCTTCAAAGGC

TTGGAAAACAGTGGAATATTCACATTTCAAGATGATAAAAGGCTAGAGCATGGCACTTCA

ATATCAGATGAACTCTTGAAAGCTATCGAACAGTCTCAAGTTGCCCTCGTCGTTTTCTCA

AAAAATTATGCGACATCGAGGTGGTGCTTAGATGAGCTAGTGAAGATCATGGAATGCAAG

GATCAATGCGGACAAACTGTCATGCCAGTCTTCTATGATGTGGATCCATCACATGTTCGG

AACCAGAGGGAGAGCTTTGCTGAAGCCTTTAACAAACATGAAACAAACTATAAGGATGAT

GATGAAGGAATGGAGAAGCTCCAAAGATGGAGGAACGCTCTAACTGCTGCCGCAGATCTA

AAAGGATATGATATTCGTGACGGGATTGAAGCAGAGAATATTCAGCAGATTGTCGACCAA

ATTTCCAAATTGTGTAATAGTGCTAATTTGTCTTCTTTGCGAGAAGTTGTGGGAATAGAT

ACTCATCTGGAGAAATTAAAGTCTCTACTTAAGGTAGGAATCAATGATGTTCGAATCATA

TTGGGGATCTGGGGCATGGGCGGTCTAGGGAAGACGACAATAGCAAGAGCCATTTTTGGC

ATTTTATCTCATCAATTTGAAGCTGCTTGTTTCCTTGCGGATATTAAAGAAAATGAAAAA

CTACATTCTTTGCAAAACACCCTTCTCTCTGAATTGTTAAGAAAAAAAGATGATTATGTC

AATAATAAGCATGATGGGAAGCAAATGATTCCAGACAGACTTCGCTTTAAGAAGGTGCTA

ATTGTGCTCGATGATATAGATCATAAAGACCATTTAGATTATTTAGCGGGTGATCTTGGT

TGGTTTGGTAATGGCAGTAGGGTTGTTGTAACAACTAGAGACAAGCATTTGATAGGGAAG

GATGATATAATATACGAAGTGACTGCACTACCTGATCATGAATCCATTCGATTGTTCTAT

CAGCATGCTTTCAAAAAAGAGGTTCCAGATGAGTGTTTTAAGGAGCTTTCATTGAAGGTA

GTAAATCATGCTAAAGGCCTTCCTTTAGCCCTCAAAGTATGGGGTTCAATGCTGCATAAC

CTACGAATAACTGAATGGAAAAGTGCTATAGAGCACATGAAAAATAATTCTAATTCTGGA

ATTGTTGATAAGCTCAAAATTAGTTATGATGGATTAGAGCTCATCCAACAAGAGATGTTT

CTAGATATAGCATGCTTCTTGAGAGGGAAATATAAAGATTACGTCATGCAAATTCTTGAG

AGTTGTCATTCTGGAGTTGAATACGGATTGCGTGTCTTAATTGACAAATCTCTTATATTT

ATCTCTGAAAAATGTCAGATTCAAATGCACGACTTAATACAAGAAATGGGTAAATATATA

GTGAACTTGCCAAAGAATCCGGGAGAGCGCAGCAGACTATGGCTCGTCAAGGATTTCAAA

GAAGTGATGAGCAACAGTACACAGGGGACCATGGCAATGGAAGCAATCTTTCTTCCTTAT

CTCAATTCTGGTACATCACGCTTTAGCAAAAATATGAAAAGGCTTAGGATATTTAACATA

GAGAGGTCGTTGAACTGTGATGGTTCCATTAAGTATCTGCCCAACAGCTTGCGTTGGTTT

GTGTGGGAGAACTTTCCTTGGAAGTCATTGCCATCTACATTTGAACCCAAAATGCTTGTT

CACCTTGAACTCTGGGGTAGTTCACTGCATTATTTATGGATGGAAACAAAGCATTTGCCA

TCTCTACGGAGGATAGATCTCAGCTCCTCTAGAAGACTGAGGCGAACACCAGATTTCACG

GGGATGCCAAATTTGGAGTATTTGAATATGTTATATTGCAGAAATCTTGAAGAGGTTCAC

CATTCCCTGAGATGTTGCAGCAAACTCATTCGGTTAAATTTGAATAATTGTAAAAGCCTT

AAGAGGTTTCCATGTGTTAACGTGGAATCTCTTGAATATCTGAGTTTAGAATATTGCTCA

AGTTTAGAGAAATTTCCAGAAATCCACGGGAGAATGAAGCCGGAGATACAGATTCACATG

CAAGGCTCTGGGATAAGGGAACTACCATCATCTATTACTCAGTACCAAACTCATATTACC

AAGCTAGATTTGAGAGGTATGGAAAAACTTGTAGCTCTTCCAAGCAGCATCTGTAGGTTG

AAAAGTTTGGTTAGTCTGAGTGTGTCGGGTTGCTTCAAACTTGAAAGCTTGCCAGAAGAG

GTAGGGGATTTAGAAAACTTGGAGGAGCTTGATGCCAGTTGTACTCTAATTTCACGACCT

CCGTCTTCCATCGTTCGCTTGAGCAAACTTAAAATCTTTGATTTTGGAAGCTCCAAAGAT

AGAGTGCACTTTGAGCTCCCTCCGGTGGCAGAAGGATTTCGCTCATTGGAAACTTTGAGT

CTTAGAAACTGCAATCTAATAGATGGAGGACTTCCGGAAGATATGGGATCCCTATCCTCT

TTGAAAAAGTTGTATCTTAGTGGAAATAATTTTGAGCATTTGCCTCGAAGCATAGCCCAA

CTTGGTGCCCTTCGAATCTTGGAATTAAGAAATTGCAAGAGGCTTACACAGCTGCCAGAA

TTTACGGGGATGCTAAATTTGGAGTATTTGGATCTGGAGGGATGTAGTTATCTTGAAGAG

GTTCACCATTCCCTGGGGTGTTGCAAAAAACTCATTCGGTTAAATTTGAGTTTTTGTAGT

AGCCTTATAAGGTTTCCATGTGTTAACATGGAATCTCTTAAATATCTGAGTGTAGGAGAG

TGCTCTCGCTTAGAGAAATTCCCAGATATCCATGGGAGAATGAAGCCGGAGATACAGATT

CACATGAAACGCTCTGGGATAAGGGAACTACCATCATCTATTTCTCAGTACCAAACTCAT

ATTACCGAGCTAGATTTGAGAAGGATGGATAACCTAGATTCCAGAGGCTACTAG

>NTK326_mRNA_16508_cds NTK326_mRNA_16508 gene_9366|id=AT5G17680.1

ATGATGCAGAAGAGCTGTTTGCTTCCTTCTTCTTCTCTTTCCGTTGGCCATACCTTTCGG

TGGAGTTACGATGTTTTCTTAAGTTTTAGAGGTGAAGATGTACGCAAAACATTTGTTGAC

CATCTCTACGTTGCTCTGCAACAAAAGGGTATTCATACCTTCAAAGATGATGAGAAGTTA

GAGAGAGATGAATTAATGAAGATCATGGAATGCAACAAACAAAAAGGACAAATTGTCCTT

CCGGTCTTCTACGATGTAGATCCATCAACAGTGAGGAAACAAAAGTCCAGCTTTGGAGAA

GCATTTAGCAATCACGAAATCAGCTGTTTCAAGGATAACAAGGTACAAAAATGGAGGGCA

GCACTGGAGGAAGCTGCTAATTTATCTGGCTGGGATTTGCCAAATACTGCCAATGCGCAT

GAAGCTAAAGTCATAAAGCAAATTGTGGAAGATATGATGGCTAAATTAGGTGGTCAGAGG

CATGCAATCAATGCTGAAAATCTTGTTGGAATGGAGTCGCAAATGCAGAAAGTGTATAAA

ATGCTTGGCATCGGGTCTGGTGGAGTTCACTTTGTTGGAATATTTGGAATGAGCGGAGTG

GGAAAGACAACTTTAGCGAGAGTCATTTATGATAACATTTCGAGTCAATTTGAGGGTGCT

TGTTTTCTTCATGAGGTTAGAGACCGTTCAGAAAAACAAGGCCTAGCGCGATTACAAGAG

ATACTTCTTTCCAAGATCCTTGTCATAAAAGACCTAAGGATCAACAATTTATTTGAAGGA

CTTAATATGCAAAGACAGAGACTACGGTTCAAAAAGGTTCTTCTTGTTCTTGATGATGTT

GATCACATAGATCAGTTAGATGTTTTAGCTCAGAAGCGCGAATGGTTTGGTTCTGGAAGT

AGAATCATCATAACAACTAAAGACAAACACTTGCTTGTTAAGCATGATGTGGAAAAGATA

TACAAAATGAGAACATTAAGTGAAGATGAAAGTCTACAACTATTTAAACAGTATGCTTTC

AAGAAGAACCATCCAACCAAGAAATTTGAGGATCTCTCAGCTCAAGTGATAAAGTATAGT

GCTGGACTCCCCTTGGCTCTGAAAGTCCTGGGCAGTTTCTTGTATGGAAGAGATTTGGCT

GAATGGAGAAGTGAAGTAGAAAGATTGAAACAAATCCCGGAAGATGAAATTTTGAGGAAA

CTCGAACCAAGTTTCACTGGACTCAAAAGTATCGATCAAATGATATTCTTAGACATTGCG

TGTTTCTTTACAGGGAAGAAGAAAGATTCAGTGACTAGAATACTTAAGAGTTTTAATTTT

AGCCCTGTTATTGGCTTAAAAGTTCTCATGGAGAAATCTTTGATTACTATTTCAGAAGGT

AGGATTTTAATGCACCAATTGATACAAGAAATGGGCTGGCACATTGTTCGTCGAGAAGCT

TTCGATTATCCCAGAAAATATAGTAGGTTATGGAAGTCTGAAGATATTTCTCATGTACTT

GCAAGAAATATGGGCACAGAAAAGATCGAAGGCATATCTTTGAACTTGACTAAGACGCTC

ACAGATATTTCTCATGCACTTGAAAGAAATTTGGGCACAGAAAAGATCAAAGGGATATCA

TTGAACTTGACTATCGTCAAAGAAGTGAATGTTAGTGCAACAGCCTTTATGCAGATGACC

AGACTGAGGTTTCTCAAAATCAAGAATGCATATGTTTCTCAGGGTCCGGACATTCTTCCT

AGTGAGTTGAGCTGGCTTTCTTGGCACGGATATCCTTCAAAAAGTCTGCCAATTAGCTTT

CAGGGAGAACGACTCGTTAGTTTGAAGTTGAAAAATAGTCGCATCATACAACTTTGGAAA

GGCTCCAAGGTTCTAGGACAACTGAAGTACATCAACCTTAGCCATTCACATAAGCTAATA

AGGACTCCAGATTTTTCGGGTACCCCTAATCTTGAAAGGTTGGTTCTTGAAGAGTGCACG

AGTTTGGTAGAAATCAATTTTTCTGTTGGAGATCTCAAAAAGCTAGTCTTACTCAAGTTG

AAGAATTGCATCAATTTAAAGACCCTGCCAAAGAGTATTCAATTGGAAAATCTTGAGGTT

CTTATTCTATCAGGCTGCTCAAAGCTAAAACTATTCCCAGAAATAGAAGATGGAATGAAT

CGTTTATCAGAACTATATTTGGAAGCGACTTCTTTGAGTGAACTACCCGCATCAGTTGAG

AAACTATCAAGAGTTAAAGTGATAAATCTAAGCTCATGCAAGCATCTTGAGAGTCTTCCA

AATAGTATTGTTAGGTTGAAATGTCTTAAAGAACTTAATGTGTCGAGGTGCTCAAAACTT

AAAAGTTTACCAGATGACTTGGGTTCTTTAGTCGGATTGGAGGGGCTCCATTGTGATGAC

ACACCGATCCAAATGATACCATCCACCATTTCCCTTCTAAAGAACCTTAAACACTTATCT

CTCCGTCGATGTAATGCTTTGGGTTTGCAAGTAAGGAGTTCAATCTCAAGAGAATCTATG

GGACTAGTTTTCTCTAATTTATCGGGTCTTTGTTCATTGACAATGCTGGATATAGGTGGC

TGCAGCATTTCAGATGGAGGCATCCTATGTAATCTTGGGTTCCTACCATCTTTGGCGGAA

TTGAATCTTGGTGGTAACACGTTTACCAATATCTCAGCTTCAAGCATCAGTGGCCTCACT

CGACTAAAGGTTCTTCAATTGGTTGGCTGTAGTAGGCTTGAGCATTTCCCAGAACTTCCT

CAAGCTATAGAAGAGGTGCATGCCGATGAATGTATATCTTTGAAGAGTATCGATCAATTA

GCAAAATATCCAACATTACGCCGACTTTCACTTAGCCAATGTCATCAGCTTCATGATACT

GACATGGTTGATGCATTATGGAGCAACATGCTCAAGGGACTATACGTGCTACGAAATGAT

CTCAGCATTTGCATCCCTGGATCGCAAATTCCTATGTGGTTTACATATAAGAACTTTGGG

GAAAATGTTACACTGACTCTTGCCAATAATTGGTACACTGATAACCTCTGGGGTTTTGCT

TTCTGTATTGTTTTTGAACGTATGGAATGGTGCGGTCTATATGATGGTTACCTACAACCA

TCACTTGGATTTCCAGTTAACCTTAAATTCAAAACATATGATGGTAAGGAAGGCGATATA

CGCAGCATTATTGGCATCAAAGGAGGTGATATGTCAATTCGGAACTCAGAGCACACTCTC

CTTTCTTATGTACCATCTCGTCGTTTTCTGCAACCTTACAATAACGAGGTTTACTGTCCC

AACGACTGGATCGAAATTGTGGCATATTCGACTGTACAATTCGACAGTAAAGCTTGGGGG

ACGCGTCTTGTGTATTTGGACGATATTATTGAAGCATGA

>NTTN90_mRNA_46477_cds mRNA_46477 gene_26069|id=AT5G36930.1:evalue=3e-09:annot='Disease resistance protein (TIR-NBS-LRR class) family';id=Solyc09g092410.2.1:evalue=0.16:annot='Tir-nbs-lrr, resistance protein'

ATGGATACTCAATTAGTTAGAGGAGAATCATGTACATCTTCTCACATCTCTTATGAAGTA

TTCCTCAGTTTTAGAGGTGAAGACACCCGAAAAACATTCACCGGTCATCTTTATTCCAAA

TTGTCTGATGTTGGAGTTAATACCTTCATTGATGATGAGGAATTGAGAAAGGGTGATGTG

ATTTCAAGTAAACTAGAGAAAGCAATTGAAGAGTCAAGAATTTCCATTATTGTTTTCTCA

AGAAATTATGCTTCCTCTAGTTGGTGTCTAAATGAGCTAGTTAAAATTCTTGAGTGCAAA

GAGAAATTAAAGCAGATGGTTTTGCCTATTTTCTACGATGTTGATCCTTCTGAGGTACGA

AAGCAAACAGGGTTATTTGATGAAGCTTTGGCTAAACATAAGGAACGACCATTTGGAGGT

CAAATGGTGGAGAAATGGAGAGCTGCACTTACTGAAGCAGCAAATTTATCTGGATGGCAT

TTGCAAAATGTTGCTGGCGGGCATGAATCAAAGTTTATTGAAACAATTATACAGCAAGTC

CTACAAGAGGTCAACCAGACACCTCTAGATGTTGCTTGGCACCCAGTTGCAGTAGATTCT

AGTGTCAAAGATATAGAGTTGTTATTGCAAATTGAATGTGAAGATGAAGTTCGCATGATT

GGTATTCACGGACTTGGTGGCATAGGGAAAACAACTCTGGCAAAAGCTATGTACAATCGA

ATGTTTCGACTCTTCGATAGTGGTTGCTTCCTTTCAGATGTTAGATCAGAAGATGAAGAA

TTTGGTCTTGTCAAGCTACAAGAGAAACTTCTTCAACAAGTTCTCAAAACCAAGGACATC

AAAGTTGGGAGTGTTGCTCAAGGCGTTAATCTAATCAAAGCAAGACTTGGGTCAAAGAAG

GTTCTAATTGTTCTTGATGATGTGGACCATAAAAGACAGTTAGAAGCCTTAACAAGAGAA

AGAAGTTGGTTTGGTTCGGGTAGTTTAATAATCATTACCACCCGGGACAAGCGATTGCTA

CGTCGGCTTGGAGAAAAGGAGAGATATGAGGCCAAACTATTAACTGACAATGAAGCTATG

TCACTTTTTTGTTGGCATGCTTTTGATAATCATTTTCCACCAGAAGATTATGTTAAATTG

GTACACGGCATAATCGAATATTCAGGTAGGCTACCATTAGCTCTTGTGACATTGGGGTCA

CATTTACAAGGAAGTTTGGCAGAAGAATGGGGATATGAATTTGAAAAACTAAGAGCAATT

CCTCATAGTGATATCCAAAAGATTCTCAAGATAAGCTTTGATGGACTTGATGATGAAGCT

CAATCTGTTTTTCTCGATATTGCTTGCACCTTCCATGGGTTTGATGAGCATCAAGTTAAT

GAGATATTAAATGCGTGTGGCTTTCATACTAGAAGTGCAATTGCAACTTTAGTCCAAAAA

CACTTGCTCCGAAGATCTTGGAATCATTTGTTGGTGATGCATGATCTAGTGCGAGATATG

GGAAGAGAAATCGTTCGCATGGAATCACCTCGAGACCCTGGAAAACGGAGTAGATTGTTC

ATCCCTCAAGAAGTTTGTGATGTTCTACAAGGAAATAAAGGTTCCGAAAATGTAGAAGTA

CTGAAGGTAGATCGAGAGACGTTAAAGGGAGTGCACTTGAGCACCAAAGCATTTGAGCAA

ATGAAGAACCTTAGGGTGCTTATAACCAATGAGTTACATATTAGTGGAGATTTTGGGATG

TTGTCCAAGAAGCTCAAATGGTTGTCTTGGCGAAAATGTCCTTTAAAATGTATACCATCA

AATTTTCCAGCTGAGAATCTTGTAGTTCTAGATATGCGGAAGAGTGATATCCAAGAATTT

CAATTGAATTTACAGTGTTGTAGAAGTTTAAAGAAGCTGAATCTCTCTTATTGCGAGCAA

CTAAGAATGACTCCAAACTTCAACGATTCACGAAGTCTTGAGACTTTGCTGCTTGGTGGT

TGCTCAAGTCTGACAGAGATCCATCCATCAATAGGAAATTTGGACAGACTAATTAAACTA

GATATGTCTCGTTGCGAAAAACTTAGGGATCTTCCAAGCAGCGTATGCCAGCTAAAATCC

CTTGAAGAATTGTTCATTAATGACTGCTCATCAATAAAAATACTGCCAGATAACCTTGGA

GATGTGAAAAGTCTAAGATCTCTTAAAGCATATGGTACGGGTATAAAACAATTGCCTAGA

TCCGTTGAAATGCTAAGAAATCTTGAAACTTTGAGCGCGGGAGGTCAAGAGTTAGAGGCT

AAAAGGAATATTCCTGGAAGAGGTGTCCATCGGATACAATATTCCTTGCCAACTTTTGTA

ACCGAATTGAGCCTTATATACTGGAATTTGTCCTATGCTGATATTCCTAGGGATATTGGG

AGCTTATCCTCCTTAAAGTTTTTAGATTTGAGTGGCAACAGTTTCCATTCTCTACCCTTC

GATTTTTCTAAGTTACGAGTATTGAAGAAGTTGTATTTGAATGACTGTGAGAATCTTCAC

ACACTCCCGTCAGTATCAAATTTAGAGAATCTTGGACATCTTGAACTTCAAAATTGCAAA

AAATTGGTCAAGATTACACAGTTGGACAACCTCCCTTCTATACGGTTGATTAACACGAGT

AATTGTAGTTCTCTGCAGAATCCATTCAATGAAGGCTTCGCTGCTCTATTAATTCCATTT

AGAGGACATATGGGTTATTTAGAAACTTATCTGGAATGCAATGAGTTTCCAGAATGGTGC

AGCAATCAAGTAACAGCTTCATCTATCTGTTTCACTATGCCGACACATAATAATGAGTAC

AACTTCTTAGGAATGGTTCTCTGGTTTGTTGTCGACGCTTTGGATGTAGCCCTTAGCATT

GCCCATAAAGTGCCTTCAGGTATTCCGTGGAGTCATATTGGAATACTTAATGGACACAGA

GAACTGACATGTGTATATTACATATCTATCTTAAATGAACTTTTCTATGGCCGGATGATT

AAAGGCAGGGAAAGGATAGACGTGTGGTCTGAAGACATTACCCTAAAGAAGATAGGGATA

GATCTGTTATATTTAGACCAAAATGGTAAAGTTATATCTTTGCCGGGAGACGTGGATCAT

TCTTATTCTAGGGCGATAGATGTCAGGAATTGGCAGGTGGAGATTTTTGATGGCCAGACG

ATAGATATGAGTATTACATACTTTAATTTGTCCGAGGATGATATTCCTAAGAATATTGGG

AGCTTATCCTCCTTAGAATATTTAGATTTGAGTGGCAGCAGTTTCTATTGTCTACCCTTT

GATATTTCTAAGTTACGATTGCTGAAGGTGTTGTGTTTGAATGACTGTGAGAATCTTCAA

ACGCTCCCGTCAGTTTCAAATTTAGAGTATCTTGAAAGAATTGGACTTTATAATTGCCAG

AAATTGGTCAAGATTTCAGAGTTGGACAACCTCCCTTCTATATGGTCGATCAACATGATT

AATTGTAGTTCTCTGCAGAATCCATTCAATGAAGGCTTCTTTAGTGCACCTGCACTATAT

GCATCTAGAAAAGATCCTGAGTTGGATCCTTTAGAAATATTTCTGGAATGCAAGGAGATT

CCAGAATGGTGCAGGAATCAAGTAACAGCTTCATCTATGTGTTTGACTATGCCGACACAT

AATAATGATGAGTATAACTTCTTAGGAATGGTTCTCTGGTCTGTTTTCGACTCTTGGCAT

GAATCCTTTTTCTTGATTAGTATAGCAGGTAGAAAGACTTTAATTTCTCCGCGGAGTATA

CCTGATGGAGGACATAGAGAACTGTCATGTGTATATTACATATCTTACTTAAATGAAGCT

TTTGATGGCCAGATGATTAAAGGCGGGGAAATGGTAGAAGTGTGGGCTCCAGATTTTACA

GTAAAGAAGATAGGGATAGATCTGTTATATGTAGACCAAAATGGTAAAGTTATATCTTTA

CCGGGAGACATGGATCATTCTTATTCTAGGGCGAAAGATGTCAGGATAGAGGTCAGGATC

AGGAATTGGCGGGAAGAGTTGCTTAGTTTGGCAGACACAATAAGAGAAATTTGA

>NTBX_mRNA_42648_cds NTBX_mRNA_42648 gene_24961|id=AT5G17680.1

ATGGAATGCAAGGATCAATGTGGACAGACTGTCATACCAGTCTTCTATGATGTGGATCCA

TTACATGTTCGAAACCAGAGGGAGAGCTTTGCTGAAGCCTTTGACAAACACGAAACAAGA

TATAAGGATGATGATGAAGGAATGCAGAAGCTCCAAAGATGGAGGAGTGCTCTAACTGCT

GCCGCAAATCTAAAAGGATATGATGTCCGTGACGGGATTGAAGCAGAAAATATTCAGAAG

ATTATCGACCAAATTTCCAAATTGTGCAATAGTGCTACTTTGTCTTCTTTGCGAGATGTT

GTGGGAATAGAAACTCATTTGGAGAAATTAAAGTCCCTACTTAAGGTAGGAATCAATGAT

GTTCGGATCATATTGGGGATGTGGGGCATGGGCGGTGTAGGGAAGACGACAATAGCAAGA

GCTATTTTTGACACTTTATCTCATCAATTTGAAGCTGCTTGTTTCCTTGCGGATATTAAA

GAAAATGAAAAATTACATTCGTTGCAAAATACCCTTCTCTCTAAATTGTTAAGAAGAAAA

GATGATTACGTCAATAATAAGCATGATGGGAAGCGGATGATTTCGGATAGACTTTGCTCT

AAGAAGGTGCTAATTGTGCTTGATGATATAGGTCATAAAGATCATTTAGAGTATTTAGCA

GGTGATATTGGTTGGTTTGGTAATGGCAGTAGGGTTATTGTAACAACTAGAGACAAGCAT

TTGATAGGGAAGGATGATTCTATATATGAAGTGACTGCACTACCTGATCATGAATCCATT

CAATTGTTCCATCAACATGCTTTCAAAAAAGAGGTTCCAGATGAGTGTTTTAAGGAGCTT

TCATTGAAGGTAGTAAATTATGCTAAAGGCCTTCCTTTAGCCCTCAAAGTGTGGGGTTCT

TTGTTACATAAGAGGGATATAACTGTTTGGAGAAGTGCTATAGAGCAAATGAAAATTAAT

CCTTACTCGGAAATTGTTGAAAAGCTCAAAATCAGTTATGACGGATTGGAGCCCATCCAC

CAAGAGATGTTTCTAGATATAGCATGCTTCTTGCGAGGGGAAGAAAAAGATTACGCCATA

CAAGTTCTTGAGAGTTGTCATTCTGGAGTTGAATATGGATTGCATGTCTTAATTGACAAA

TCTCTTGTATTTATCTCTGAAAAAGATGAGTTCCAAATGCACGACTTAATACAAGAAATG

GGTAAATATATAGTGAATTTGCAAAAGAATCCGGGAGAACGCAACAGACTATGGCTCGTC

AAGAATTTCAAAGAAGTGATGACAAATAATACAGGGACCATGGCAATGGAAGCAATTTGG

CTTCATGATAATTTTGGTACACTACGCTTTAGCAATGAGGCCATGAAAAATATGAAAAGG

CTTAGGATATTATACATAGAGAGGTGGTCCTGTTATGGTTCCATTGAGTATCTGTCCAAC

AACTTGCATTGGCTTGTCTTGGATGGCTATCCTTGTGAGTCACTGCCATCTACATTTGAA

CCCAAAATGCTTGTTCACGTTCAACTCAAACACAATTCACTGCATTATTTATGGATGGAA

ACAAAGCATTTGCCGTCACTGAAAAAGCTAATTCTCAGCGGCTCTGGAAACCTGATGCGA

ACACCAGATTTCAAGGGGATGCCAAATTTGGAGTATTTGGATTTGAGTTTTTGCAGTAAT

TTTGAAGAGCTTCACGACACCCTGGGATGTTGCAGAAAACTCGTCGAGTTAAATTTGACT

TGGTGTGAACGCCTTAAGAGGTTTCCATGTGTTAACGTGGAATCTCTTGAATATCTGAGT

TTAGAAAATTGCTCAAGTTTAGAGAAATTTCCAGAAATCCACGGGAGAATGAAGCCGAAG

ATACAGATTCACATGCTTGGCTCTGGGATAAGGGAACTTCCATCCTCATATTTTCAGTAC

CAAACTCATATTACCGAGCTAGATTTGAGCTTTATGAGAAACCTTGTAGCTCTTCCAAGC

AGCATCTCTAGGTTGAAAAGTTTGGTTAGTCTGAGTGTGTTGGATTGCTCAAAACTGGAA

AGCTTGCCAGAAGACATTGGATCCTTATCCTCTTTGAAAGAATTGTATCTCAATGGAAAT

AATTTTGAACATTTGCCTCGAAGCATAGCCCAACTTGGTGCTCTTCGATCCTTAGACTTA

TCATATTGCCAGAGGCTTACACAGCTGCCAGAACTTCCCCCTGAATTAAATGAATTACAT

GTAGATTGTCATATGGCTCTGAAATTTATCCATGATTTAGTAACAAAGAGAAAAAAACTA

CAGAGGGTGATATTCCCTGATGATGAGGATGATGCACTCGATGATCCTATATATAATTTG

TTTGCACATGCCCTGTTTCAGAATATCTCTTCCTTGAGGCATCACATCTCTGCTTCAGAT

TCCTTGTCCGAAAGTGTGTTTACCATTCTGCATCCTTGGAAGAAGATCCCAAGTTAG

>NTTN90_mRNA_51079_cds mRNA_51079 gene_28678|id=AT5G36930.2:evalue=0.021:annot='Disease resistance protein (TIR-NBS-LRR class) family';id=Solyc09g092410.2.1:evalue=0.0:annot='Tir-nbs-lrr, resistance protein'

ATGACTGGGCTAGTTGTTGGGCTTCATCAAATGGATACTCACTTCTCTTATGAAGTATTC

CTGAGTTTTAGAGGTGAAGACACCCGAAAAACATTCACTGGTCATCTTTATTCCAAATTG

GATAATGTTGGAGTTAAAACCTTCATTGACGATGAGGAATTGAGAAAGGGTGATGTGATT

TCAAGTAAACTAGACAAAGCAATTGAAGAGTCAAGAATTTCCATTATTATTTTCTCGAGA

AATTATGCTTCCTCTAGTTGGTGTCTAAATGAGCTAGTTAAAATTCTTGAATGCAAAGAG

AAATTAAAGCAGATGGTTTTGCCTATTTTCTATGATGTTGATCCTTTTGAGGTACGAAAG

CAAACTGGATTATTTGGTGAAGCTTTGGCAAAACATAAGGAACGACCATTTGGAGCTCAA

AGGGTGGAGAAATGGAGAGCTGCACTTACTGAAGCTGCGAATTTATCTGGATGGGATTTG

CAAAATGTTGTTGACGGGCATGAATCAAAGTTTATCGAAAAAATTATACAACAAGTCCTA

CAAGAGGTCAACCAGACACCTCTAGATGTTGCTTGGCACCCAGTTGGAGTAGATTCTCGT

GTCAAAGATATAGAATTGTTATTGCAAAAGGAATGTGAAGATGAAGTTCGCATGATTGGT

ATTCACGGAGTTGGTGGTATAGGAAAAACAACTCTGGCAAAAGCTATATACAATACAGAA

TTTCGGCGCTTCAATAGTAGTTGCTTCCTTTCAGATGTTAGATCAGAAGCTGAAGAATTT

GGTCTTGTCAAGCTACAAGAGAAACTTCTTCAACAAGTACTCAAAACTAAGGACGTCAAA

GTTGATAGTGTTGCTCAAGGCATCAATTTAATCAAAGCAAGACTTGGGTCAAAGAAGGTT

CTAATTCTTCTTGATGATGTGGATCACAAAAAACAATTAGAATCCTTAACACGAGAAAGA

AGTTGGTTTGGTTCGGGTAGCTTAATAATTATTACAACCCAAGACAAGGATTTGCTACGT

GGGCTTAGAGAAAAAGAGAGATATGAGGTCAAACTGTTAAATGACAATGAAGCTATGTTA

CTTTTTAGTTGGCATGCTTTTGACAGTCATTTTCCACCAAAAGACTATGTTAATTTTGCA

CAAGACATAATCAAATATTCAGGCAGGCTATCATTAGCTCTTGTAACATTGGGGTCACAT

TTACAAGGAAGTTCTGTAGAAGAATGGGGATACGAATTCGAAAAACTAAGAGCAATACCT

CATTGTGATATCCAAAAGATTCTCAAGATAAGCTTTGATGGACTTGATGGTGATACACAA

ACTGTGTTCCTTGATATTGCGTGCGCCTTCCATGGTTTTTATGAGCATGAAGTTACAGAA

ATATTGAATGCATGTGGCTTTCATGCTAAAAGTGCAATTGCAACGTTAGTCCAAAAACAC

TTGCTCCAAAGGGATGATCTTTATTTGGTGATGCATGATCTAGTGCGAGATATGGGAAGA

GAAATTGTTCGCTTGGAATCAAGTCGATACCCAGGAAAACGGAGCAGATTGTTCATCCCT

CAAGAAGTCTGTGATGTTCTACAAGGAAATAAAGGTTCCAAAAAGGTAGAAGTACTGAAG

GTAGATCGACGAGCATTTAAGGGAGTGAACTTGAGCACCAAAGCATTTAAGAAAATGGAA

AACCTTAGGGTTCTTATAATGGATGAGTTACATATTAGTGGAGATTTTGGGATGTTGTCC

AAGGAGCTCAAATGGTTGTCTTGGAAAAAATGTCCTTTAAATTGTATACCATCAAATTTT

CCAGCTGAGAATCTAGTAGTTCTAGATATGCAGGAGAGTGATATCCAAGAATTTCGATTG

AATTTGCAGTGTTGTAGAAGTTTGAAGAAGTTGGATCTTTCTTATTGCAAGCAACTCAGA

AGCACTCCAGACTTCAATGGTTCAGTGAGTCTTGAGACTTTGTATCTCTATGGTTGCTCA

AGTCTAACGGAGATCCATCCATCAATAGGAAATTTGGACAGGCTAATTAAACTATATATG

CGTGGTTGCGAAAAACTTACGGATCTTCCAAGCAGCATATGCCAGCTAATATCCCTTGAT

TCCTTGGACATTGATGACTGCTCATCAATAAAAATACTGCCAGATAACCTTGGAGATATG

AAAAGTCTAAGATCTCTTTATGCATCTCGTACGGGTATAAAACAATTGCCTAGATCTGTT

GAAATGCTACGAAATCTTCGAACTTTGTCAGCAGGAGGTCAAAAGTTAGAGGCCAAAAGG

AGTATTTCGGGAAGAGGAGTCCATCGGATACAATATTCCTTGCCAACTTTTGTATCCTAT

TTGAGCCTTACATATTGTAATTTGTCCGATGCTGATATTCCTAGGGATATTGGGAACTTG

TCCTCCTTAGAGCTTTTAGATTTGAGTGGCAACAGTTTCCATTGTCTACCCTTTGATTTT

TGTAAGTTAGGATTCTTGAAGTGGCTGTATTTGAATGACTGTGAGAATCTTCAAACACTC

CCGTCAGTATCAAATTTAGAGAATCTTGAAATTCTTGAACTTCAAAATTGCCAAAAATTG

GTCAAGATTAGAGAGTTGGACAACCTCCCTTCTATATGGTCGATCAATATGATTAATTGT

ATTTCTCTGCAGAATCCATTCAATGAAGGCTTCTTTAGTGCACCTGCTCTATCATTTCCA

TCTAGAAAAGATCGATATATGGATCATTTAGAAATTTATCTCACATGCAATGAGATTCCA

AAAAGGTGCAACAATCAGATGTTAGATCAGAAGCTGGAGAATTTGATCTTGTCAAGCTAC

AAGAGAAACTTCTTCGAGCCCGTTTGGATTGGCTTGTTTTAA

>NTTN90_mRNA_68293_cds mRNA_68293 gene_38440|id=AT5G17680.1:evalue=2.6:annot='disease resistance protein (TIR-NBS-LRR class), putative';id=Solyc05g007850.1.1:evalue=0.0:annot='Tir-nbs-lrr, resistance protein'

ATGGCATCATCTTCTGCTTCTGGTACTTCACAGTTTCCTCGATGGAACTACGATGTCTTC

CTAAGTTTTAGAGGTGAAGATACTCGGAAAACATTTACGAGTCACCTGTACGAAATCTTG

GATATCAGGGGAATAAAAACCTTTCAAGATGATAAAAGGCTAGAGCATGGCGCATCCATT

TCGGATGAACTATGTAAAGCTATCGAAGAGTCTCAATGTGCAGTCATCATTTTCTCAAAA

AATTATGCAACATCGAGGTGGTGCTTGAATGAACTAGTGAAGATCATGGATGTCAAGACT

CAATTTGGACAAACTGTCATACCGGTCTTCTATGATGTGGATCCATCACATGTTCGGAAC

CAGAGGGAGAGCTTTGCTGAAGCATTTTCCAAACATGAAACAAAGTATAAGGATGATGTC

GAAGGAATGCAAAGATGGAGGATTGCTTTAACTGCAGCGGCCAATCTCAAAGGTTGTGAT

ATTCGTGACAAGACTGAATCAGACTGTATTCGACAGATTGTTGATCAAATCTCGTCCAAA

TTATGCAAGATTTCTTTATCTTATTTGCAAAACATTGTTGGAATAGATACTCATTTAGAG

AAAATAGAATCCTTACTAGGGATAGGAATCAATGGTGTTCGGATTGTGGGGATTTGGNGG

ATAGGAATCAATGATGTTCGGATTGTGGGGATTTGGGGCATGGGGGGAGTCGGTAAAACG

ACAATAGCTAGAGCTATGTTTGATATTCTCTTAGTAAGAAGGGATAGTTCCTATCAATTT

GATGGTGCTTGTTTCCTTGCGAATATTAAAGAAAACAAACGTGGAATGCATTCTCTGCAA

AATATTATTTTCTCTGAACTTTTAAAGGAAAAAGCTGATTACAACAATAAGGAGGACGGA

AAGCACCAAATGGCTAGTAGGCTTCGTTCAAAGAAGGTCCTAATTGTGCTTGATGACATA

GATGATAAAGATCATTATTTGGAGTATTTAGCAGGTGATCTTGATTGGTTTGGTAATGGC

AGTAGAATTATTATAACAACTAGAGACAAGCATTTGATGGGGAAGAATGGTGTAATATAT

GAAGTGACTGCACTACCTAATCATGAATCCATTCAATTGTTCTATCAGCATGCTTTCAAA

AAAGAGGTTCCAAATGAGCATTTTAAGAAGCTTTCATTGGAAGTCGTAAATTATGCTAAA

GGCCTTCCTTTAGCCCTCAGAGTGTGGGGTTCTTTGCTGCATAACCTAGGACTAACTGAA

TGGAAAAGTGCTATAGAGCACATGAAAAATAACTCTAATTCTGAAATTGTTAAAAAGCTC

AAAATTAGTTATGATGGATTAATAGAGCCCATACAAGAGATTTTTCTGGATATAGCATGC

TTCTTCCGAGGGACAAAAAAAGAGTACGCCATGCAAATTCTTGAGAGCTGTCATTGTGCA

GTTGAATACGGATTGCGTGTCTTAATTGACAAATCTCTTGTGTCCATCTCTGAAAATGAT

CAGATTCAAATGCATGACTTGATGCAAGATATGGGTAAATATATAGTGAACTTGCAAAAG

AATCCGGGAGAACGCAGCAGATTATGGCTCGACAAGGATTTCGAAGAAGTGATGATGAAC

AATACAGGGACCACGAAAATGGAAGCAATCTGGTTTCCTTATTACCATTATGTTACATTA

CGCTTTGGCAAAGAGGCCATGAAAAATATGAAAAAGCTTAGGATATTAAACATAGAGATG

TCGTGGCCTTGTGATGGTTCCATTGAGTATCTGCCCAACAGCTTGCGTTGGTTTGTCTGG

ACTGACTATCCTTGGGAGTCGTTGCCAGCTGAATTTGAACCCAAAAAGCTTGTTCATCTT

GCACTCAAATCCAGTTCACTGTGTTATTTATGGACGGAAGCAAAGCAATTGTCGTCTCTA

CGGACGCTAGATCTCAGATACTCTGAAAGCCTAGTGCGAACACCAGATTTCACAGGGATG

CCAAATTTGGAGTATTTGAATCTGGAGGAATGTCGTGATCTTGAAGAGGTGCACCATTCC

CTGGGATTTTGCAGAAAACTCATTCGATTAAATTTGGAGTCTTGTGGACGCCTTAATTGG

TTTCCATGTGTTAACGTGGAATCTCTTGAATATCTGGATCTAGATTTTTGCTCTAGTTTA

GAGAAATTTCCAGAAATCCATGGGAGAATGAAGCGGGAGATACAGATTCACATGAAACGC

TCTGGGATAAGGGAACTACCATCATCTATTATTCAGTACCAAACTCATATTACCTTCCTA

GATTTGAGCGCTATGAAAAACCTTGTAGCTCTTCCAAGCAGCATCTGTAGGTTGAAAAGT

TTGGTTAGTCTAAATGTGTCGGGCTGCACAAAACTTGAAAGCTTGCCAGAAGAGATAGGG

GATTTAGAAAACTTGGAGGAGTTTTATGCCAGGTATGCTCTAATTTCACGACCTCCGTCT

ACCATCGTACGCTTGAACAAACTTAAAATCTTGAAGTTTGGAGGCCTCCATGATGGAGTG

CACTTTTTGTTCCCTCCAGTGGCTGAAGGATTACGGTCATTGGAACATCTGGATCTCACT

TGTTGCAATCTAATAGATGAAGGACTTCCGGAAGACATTGGATGCCTATCTTCTTTGAAA

GAACTGTATCTCAGTGGTAATAATTTTGAGCATTTGCCTCGAAGTATAGCCCAACTTGGT

GCTCTTCGAATCTTGGACTTGAGAAATTGCAAGAGGCTTACACAGCTGCCAGAACTTCCA

CCAGAATCAGATACAATATATGCAGATTGGAGCAATGATTTGATCTGTAATTCGTTGTTT

CAGAATATCTCGTCAGTGCAGCATGACATCTCTGCTTCAGATTCCTTGTCACTAAGAGCA

TTTACCAGTGTGCATCTTGGGAAGAAGATCCCAAGTTGGTTCCTCTATCAGGGAATGGAT

AGTGGTGTATCAGTCAATTTGCCTGGAAATTGGTATATACCTGATAAATTCTTGGGATTT

GCTGTATATTACTCAGGCAGCTTAATTGACACCACAACTCAATTGATTCCCGTATGTGAT

GATGGGATGTTGTGGATGACCCAGAAACTTGCCTTATCCAACCATTCAGAATGTGATACA

AAATATAATATTAATTTTTTCTTGGTACCTCTTGCTGGCTTATGGGATACATCTAAGGCA

AATGGAAAAACACCAAACGACTATGGGCTTATTAGGCTATCTTTTTCTGGAGTAATGAAG

GATTATGGACTTCGTTTGTTGTATAAAGAAGAACCTGAGCTTGAGGCCTTGTTACAGAAT

ATTGCATTGGAGGAGCAGATATGA

>NTK326_mRNA_85438_cds NTK326_mRNA_85438 gene_49971|id=AT5G17680.1

ATGGCATCATCTTCTGCTTATGCGAGTACTTCACAGTTTCCTCGGTGGAACTACAAAGTC

TTTCTAAGTTTTAGAGGCGATGATACTCGAAGAACATTTATAGGTCACCTCTTCAAAGGC

TTGGAAAATAGTGGAATATTCACGTTTCATGATGATAAAAGGCTAGAGCATGGTGCATCA

ATATCGGATGAACTCTTGAAAGCTATCGAACAGTCTCAAGTTGCCCTCGTCGTTTTCTCA

AAGAATTATGCAACATCGAGGTGGTGCTTAGATGAGTTAGTGAAGATCATGGAATGCAAG

GATCAATGTGGACAGACTGTCATACCAGTCTTCTATGATGTGGATCCATCACATGTTCGG

AAACAGACGGAAAGCTTTGCTGAAGCCTTTGACAAACATGAAACAAGCTATAAGGATGAT

GATGAAGGAATGCAGAAGCTCCAAAGATGGAGGAATGCTCTAACTGCTGCCGCAAATCTA

AAAGGATATGATGTCCGTGACGGGATTGAAGCGGAGAATATTCAGCAGATTGTCGACCAA

ATTTCCAAATTGTGCAATAGTGCTACTTTGTCTTCTTTGCGAGATATTGTGGGAATAGAT

ACTCATATGGAGAAATTAAAGTCCCTACTTAAGGTAGGAACCAATGATGTTCGGATCATA

TTGGGGATCTGGGGCATGGGCGGTCTAGGGAAGACGACAATAGCAAGAGCCATTTTTGAC

ACTTTGTCTCATCAATTTGAAGCTGCTTGTTTCCTTGCTGATATTAAAGAAAATGAAAAA

CTGCATTCTTTGCAAAACACTCTTCTTTCTGAATTGTTAAGAAGAAAAGATGATTACGTC

AATAATAAGCTTGATGGGAAGCGGATGATTCCAGACAGACTTTGCTCTAAGAAGGTGCTA

ATTGTGCTTGATGATATAGGTCATAAAGATCATTTAGAGTATTTAGCAGGTGATATTGGT

TGGTTTGGTAATGGCAGCAGGGTTGTTGTAACAACTAGAGACAAGCATTTGATGGGGAAG

GATGATGNTGTAACAACTAGAGACAAGCATCTGATGGGGAAGGATGATGCAATATATGAA

GTGACTGCATTACCTGATCATGAATCCATTCAATTATTTTATCAACATGCTTTTAGAAAA

GAAGATCCAGATGAGTGTTTTAAGGAACTTTCATTGGAGGTAGTAAATCATGCTAAAGGC

CTTCCTTTAGCCCTCAAAGTGTGGGGTTCTTTGATGCATAACCTAGGCTTAACTGAATGG

AAAAGTGCTATAGAGCACATGAAAGTTAATTCTAATTCGGAAATTGTTGAAAAGCTCAAA

ATCAGTTATGATGGATTGGAGCCCATCCAACAAGAGATGTTTCTAGATATAGCATGCTTC

TTGCGAGGGGAAGAAAAAAATTACGCCATGCATGTTCTTGAGAGTTGTCATTCTGGAGTT

GAATATGGATTGCGTGTCTTAATTAACAAATCTCTTGTGTCTATCTCTGAAAATGATCAA

ATTCAAATGCATGACTTAATACAGGATATGGGTAAATATATAGTGAATTTTAGAAAAGAT

CCTGAAGAACGTAGCAGGCTATGGCTCGCCGAGGAAGTCGAAGAAGTGATGAGCAACAAT

GGAGGGACCATGGCAGTGGAAACAATTTGGCTTCATTGTAATTTTAGGACACTAAGCTTT

AACAATGAGGCCATGAAACATATGAAAAGGCTTAAGATATTAAACATAAAGAGTTGGGCC

TATCATGGCTCCTTTGAGTATCTGTCCAACAACTTGCGTTGGTTAGTCTTGGATGGCTAT

CCTTGTGAAACATTGCCATCTACATTTGATCCCAAAATTCTTGTTAGCCTTAACCTCCGG

GGCAGTTCACTGCATTATTTATGGATGGAAACAAAGCAATTGTCGTCTCTACGGACGCTA

GATCTCAGATACTCTAAAAGCTTGGTGCAAACACCAGATTTCACGGGGATGCCAAATTTG

GAGTATTTGAATCTGTCTTCGTGTTCTAATCTTGAAGAGGTTCACTATTCCCTGGGATGT

TGCAACAAACTCATTCGGTTAAATTTGTGTTTTTGTAAAATACTTAAGAGGTTTCCATGT

GTTAACGTGGAATCTCTTGAATATTTGGGTTTAGAATTTTGCTATAGGTTAGAGAAATTT

CCAGAAATTCACGGGAGAATGAAGCCTGGGATACAGATTCACATGTCATCCTCTGGGTTA

AGGGAACTGCCATCCTCTGTTTTTCAGTACCAAACTCATATTACCAAGCTAGATTTGAGT

AATATGGAAAACCTTGTAGCTCTTCCAAGCAGCATCTGTAGGTTGAAAAGTTTGGTTAGT

CTGAGTGTGAATTGCTCAAAACTGGAAAGCTTGCCAGAAGAGATAGGGGATTTAGACAAC

TTGGAGGAGCTTGATGCCAGTGCTACTCTAATTTCACGACTTCCGTCTACCATTGTACGC

TTGAACAAACTTAAAGTCTTGAAGTTAGCTGGATGCTTCAGATACAATAGAGTGCACTTT

GAGTTCCTTCTGGCTGAAGGATTACGCTCATTGGAACATCTGGATCTCAGTCGCTGCAAC

TTAACAGATGGAGAACTTCCGGAAGATATTGGATCCTTATCCTCTTTGAAAGAGTTGGAG

CTCAGAGGAAATAATTTTGAGCATATTCCTCGAAGCATAGCCCAACTTGGTGCTCTTCGA

TCCTTAGACTTATCATATTGCAAGAGGCTTACACAACTACCAGAATTTCCCCCTGAATTA

AATAAATTGCGTGTAGATTATCTTATGGCTCTGAAATTTATCCATGATTTAGTAACAAAG

AGGAAGAAACTACAGATGGTGATATTCGCACCACTGTATGATAAGGATGATGCAGACAAT

AATTCTATATATAATTTGTTTGCACATGCCCTGTTTCAGAAAATCTCTTCCTTGAGGCAT

GACATCTCTTCTTCAGATTCCTTGTCCGAAAATGTGTTTACCATTTGGCATCATGAGAAG

AAGATCCCAAGTTGGTTCCACCATCAGGGAAGGGATAGTAGTCTATCAGTCAATTTGCCC

GGAAATTGGTATATACTTGATAAATTCTTGGGATTTGCTGTATGTTACTGTGGTAGCTTA

ATTGACACCACAGCTCAATTGATTCCCGTATGTGATGACGGGGTGTCGTGCATGACCCAG

AAACTTGCCTTATCAGAATGTGATACAGAATCACACAGCTATCGAGAACGGTATACACCA

ATTCATTTTTTCTTGGTACCTCTTGCTGTCTTATGGGATACATCTAAGGCAAATGGAAAA

ACACCAAATGACTATGGACTTATTAGGCTATCTTTTTCTGGAAAACAGAAGAAGTATGGA

CTTCGTTTGTTGTATAAAGAAGAACCTGAGGTTGAGGCCTTGTCACAAATGAGGGAAAAT

AACAATGAACCAACAGAACATTCCAATGTGATAAGGAGGAGCAGATCTGACATTAGTGAA

CACCATGACTCCGTGACGGATGAATCCAGTTGTTGTTGTTGTCGCATACTGTAA

>NTBX_mRNA_47640_cds NTBX_mRNA_47640 gene_27857|id=AT5G17680.1

ATGGCTTCTACTTCTTCACCCACTCAAAATTGGAAGAATGATGTTTTCTTGAGTTTTAGA

GGTAAAGATACTCGTAAAACTTTTGTGGGTCATCTCTACTATGCTCTAAAACACAAAGGG

GTTCACACTTTCAAAGATGATGTAAGGTTAGAGAGAGGAAAGTCCATTTCACCTGAACTT

GTGAAAGCTATTGAACAATCAAGATTTGCTATTGTTGTATTTTCTAAGAACTATGCATCC

TCCACTTGGTGCTTGGATGAACTTGTAAAGATCATGAAATGCAAGAAAGAATTAGGACAA

ACTGTGATACCCATATTCTATGACGTAGATCCATCGGATGTGAGTAAGCAAAGTGGAACT

TTTGCTGAATCATTTGCTAGACATGAGGAAAATTTTAGAGATGATTTGGAGAAGGTGCAA

TCTTGGAGGGATGCATTTGGTGAGGCAGGCAAAACAGCAGGATATGATTTACCAAATGGC

TACGACGGGTATGAATCGAATTGCATCCAGCATGTTGTTGAAGACATACTGGGTAAATTG

TGTCAAGTTACTTCAACCATTGATAATGATTTAGTGGGGATGGAGTCTCGAGTGCGTGAA

GTAAGTTCATTACTAAGGATGGAAACACCTGATGTTCGTTTTATTGGAATTTGGGGGATG

GGCGGCATTGGTAAGACAACAATTGCAAGCGCTGTGTTTGGCAAATATTCTGGCCTATTT

GAAGGTGTTTGTTTTCTTGATAATGTTGCAGAAATGCAAAGGACATATGGACTGCAATAT

TTGCAAGGTGTTCTCCTCTCAAAAATCCTAAAGTTAAGCTTAACTATTACAAGTGTATAT

GAAGGCATGGAAATCATAAAGAAGAGGTTGCGCTCAATGAAGGTTTTTATCATTCTTGAT

GATGTAAATCAAAAAGACCAATTAGAAATGTTAGTTGGACGGCATGATTGGTTTGGTAGT

GGTAGTAGAATTTTGATTACAACAAGAGATAAAAATTTGTTAGATAATCATATGGTGGAT

GAAGTGTATTCTGTGAACTTGATGACTCTTAATGAAGCTATTGAGCTATTTAACCTACAT

GCCTTTAAGCAAAGAATTCCTAAGAAAGACTTTGAGGAGCTTTCAAATCAAGTTGTACAT

TGTGCCGGTTTGCTCCCTTTAGCTCTGAAAGTTTTAGGTTCGTTTCTCTATGGATTAGAC

AGGAGGCATTGGAGATCAACTTGGAAAAGGCTGAAGGATCTGCCAAATGATGAAATTCTT

GCTAAGCTTAAGATAAGCTTTGAAGGACTGGGGCATGTTGATCAGAGACTCTTTCTAGAT

ATTGCATGCTTTTATAGAGGAAAATTGAGGAGTTATGTAGAGGAAATACTTGAGAGCTGT

GATATCGGATCTACAATAAGAATAAAAGTCTTAATTGAAAAGTCTCTTTTATTTATCTCA

CCATATGACACAATTGAAATGCATGATTTGATACAAGAAATGGCCTGGCACATCGTGAGT

CAAGATGACTCACGAAGGAGTAGAATATGGCTTCCTGAGGACATTGAGGATTTGTTTACT

GGAAATTTGGAAGCAGAATCTGTGGAGGGACTATGGATACCAAGGAATTACATTACAAAA

CAGGATATATCATATTACAACATCAGTGAAGCATTTAGGAGAATGAAAAGATTAAGGGTA

CTTGTAGTTAGAGCAACAGATTTCTGCTCTATTGACCCGATTACTCATCTTCCTAGCAGC

CTAAGGTGGCTTGATTGGGAAGGTTGCCCTTTAAATTCATTGCCACAGAGTTTTGAACCA

TCAAAGCTTCTTCGCCTTGATATACTCGAATGTACTACACTTCATAAACTCTGGTTAATT

CCGAAGGGTTTGGACAAACTAAAAACTTTGTACCTCAGCTATTGCGAACACTTGGAAGAA

GTTCCAAGCTTTGAGATGATGCCAAATTTAGAGAGAGTAAAGCTAGAGGGATGTAAGAGT

TTGAGAGAAGTGAGCCCATCCTTTGGAGTTCTCATGAAGCTCATTTCACTGGAGCTAATT

GATTGTCAGAGCCTTGAGAAGCTTCCAAGTTATATTCAGATGGAATCCCTTAAGAGTCTC

AAACTTTCTTGTCTTCCAAAGTTGAGGGAATTACCAGAAACCAAGGGGTTGCACCGTTTA

TTGACATTGGAGATAACTGATTGTCAGAGTCTTGAGATGCTTCCAAGTTGTAATCAGATG

GAATCTCTTGCAACTCTCAAACTTTCTTGTCTTCCAAAAATAATGGCCTTGCCGGCAACA

GAAGAGATGCACCATTTATTGGAACTTGTTATAGAATATACTCCAATAGTAGAGCTTCCG

GTGTCAATTGGAAATCTTGGTTCCCTCAAACAACTACGGTTAAGTCATTGTAAAGATCTA

GTAAGCATTCCGAACAGCTTTTCTTGTCTGAAGAATCTAAGAGTTCTTGTGATCTACAAC

TGCAAAAGACTTGCAGATTTGCCAGAGAAGATGGGTGAGTTGAAGCTGTTAGAAAAGCTA

GTATTATCTGGTACTGCAATTTCCCAAATACCTCCTTCAGTTGCAGACCTTCATGAACTA

AGCTTTTTATCATTCTCTCCCTGGTTTGGATACAGAGAAGATGCAACTTTTCTGTTACCC

TCTGCATCAGGTTCATCGTCATTTAGGGTGTTAAAGCTTAATAAGCACACACTATGTAGT

GGAGAACATTATCAGGATCTTGGATGCTTATCTTCTTTGGCTCACTTGGATTTGACTAGA

AATGATTTTACTAGTTTCAATGAAAGCAACAATCAGCACTTTCATTACCTAGATATAACA

TTTTGTGAGAAGCTTGTAATGCCCAGACTTCCATCATGCATAAAGGAGTTATATGCATAT

GATCCTTTAGTCTTGAAAAGCATCCCTGATTTCCCCACCAAATATTCAGAGCTGTATTCA

GTGTCATTCACACAGCATATTGAGAATAGAGGTGAACTGACTGATATCTTGCACTTTGTC

CTCCACTTAATTAGTGTGGCATCTCAGTCTGAGAAAAGGCTACCATTTAGCATTTTTTTC

CCTGGAGATATAAGATGGAGCGGGTTCAATTATTATCGAAAAGAGCATACGAAAAGATTC

TCCACTCCACTTGATCCAAGTTGGCATGAGAGTAAATTCAAAGGATTTGTTATATGCTTT

CGTGTACCATTGGATACTGTTCAGAACCAGAAACCTTTGGATTCTAAATCACGAAGAGGA

AGTCACTGGTTCGGTTGCACTAAGGTTACAGCTAAGTTAGTGCAAAGATATGACAGGCAA

GAACAAGATGTACTCCAGAAAAAATGTTTGATTGTTGCTCGCCAAGCAATTTGCTCTCAT

AGTAGTAAATATGCCATTTGCTTTAGCTACATACCTTTTCTAGCACTATGGCATACTTCT

GATAGTGAAAAGGGGAAGAAGCCAAATGATTATTGCTTCTTTGAGGCGTCTATAGACCCA

GGCATTGCAACAAAATGGGGACTTCTTCTGGTGTACGAGAATAAAATTAAACAGATAGAT

CAATCAACCATCTCGGTCCAACGTGATGTGGAGTCTCCAAGTTCTGACCTGTTGAGAGAA

TCTAATGATGACCAAGTCCAGAAAACGGAGGATGCTTCTGTTAAGAGAAGACGGGTTGAT

TTTTGTCAAAGAGATAATATGGTTTCATTTGAAGCTGGCTGCTCTATGAAATTTCAAGCG

ATGAAGGACTCATGCTCTCCCAGTGACTTTCAGACTCTCCAAATAATTCCTGATCAACAA

TTGGAAACACCATGCTCTTCTGCAGCTCAAAGCTTCCGACACAGGGAAGAGTCATGCTCT

TCTGGACAGCCACAAACTTTACAGCTCCCTCCAGCTGATCGACAAGTTGATGAAGCGATA

AATGAGGCTACCTCCTGCATGGTATTTGAACTGGAAGCGCCAAGCTCTTCGGAGCAGCCT

GAATCTTTTGAAGTCTCTCCAGATGAGCGCAAAGATAATTCAGTGACAAATGGGTCAAGC

AGCTCTGAGGTATTCCAAGAATTGGAGGCACCATGCTCTTCTGGACAACCTCAAATTCTC

CAGCTCTTTCCCTAG

>NTTN90_mRNA_128708_cds mRNA_128708 gene_72643|id=AT5G17680.1:evalue=1e-05:annot='disease resistance protein (TIR-NBS-LRR class), putative';id=Solyc05g007850.1.1:evalue=0.0:annot='Tir-nbs-lrr, resistance protein'

ATGGCATCATCTTCTGCTTATGCGAGTACTTCACAGTTTCCTCGGTGGAACTACAAAGTC

TTTCTAAGTTTTAGAGGCGATGATACTCGAAGAACATTTATAGGTCACCTCTTCAAAGGC

TTGGAAAATAGTGGAATATTCACGTTTCATGATGATAAAAGGCTAGAGCATGGTGCATCA

ATATCGGATGAACTCTTGAAAGCTATCGAACAGTCTCAAGTTGCCCTCGTCGTTTTCTCA

AAGAATTATGCAACATCGAGGTGGTGCTTAGATGAGTTAGTGAAGATCATGGAATGCAAG

GATCAATGTGGACAGACTGTCATACCAGTCTTCTATGATGTGGATCCATCACATGTTCGG

AAACAGACGGAAAGCTTTGCTGAAGCCTTTGACAAACATGAAACAAGCTATAAGGATGAT

GATGAAGGAATGCAGAAGCTCCAAAGATGGAGGAATGCTCTAACTGCTGCCGCAAATCTA

AAAGGATATGATGTCCGTGACGGGATTGAAGCGGAGAATATTCAGCAGATTGTCGACCAA

ATTTCCAAATTGTGCAATAGTGCTACTTTGTCTTCTTTGCGAGATATTGTGGGAATAGAT

ACTCATATGGAGAAATTAAAGTCCCTACTTAAGGTAGGAACCAATGATGTTCGGATCATA

TTGGGGATCTGGGGCATGGGCGGTCTAGGGAAGACGACAATAGCAAGAGCCATTTTTGAC

ACTTTGTCTCATCAATTTGAAGCTGCTTGTTTCCTTGCTGATATTAAAGAAAATGAAAAA

CTGCATTCTTTGCAAAACACTCTTCTTTCTGAATTGTTAAGAAGAAAAGATGATTACGTC

AATAATAAGCTTGATGGGAAGCGGATGATTCCAGACAGACTTTGCTCTAAGAAGGTGCTA

ATTGTGCTTGATGATATAGGTCATAAAGATCATTTAGAGTATTTAGCAGGTGATATTGGT

TGGTTTGGTAATGGCAGCAGGGTTGTTGTAACAACTAGAGACAAGCATCTGATGGGGAAG

GATGATGCAATATATGAAGTGACTGCATTACCTGATCATGAATCCATTCAATTATTTTAT

CAACATGCTTTTAGAAAAGAAGATCCAGATGAGTGTTTTAAGGAACTTTCATTGGAGGTA

GTAAATCATGCTAAAGGCCTTCCTTTAGCCCTCAAAGTGTGGGGTTCTTTGATGCATAAC

CTAGGCTTAACTGAATGGAAAAGTGCTATAGAGCACATGAAAGTTAATTCTAATTCGGAA

ATTGTTGAAAAGCTCAAAATCAGTTATGATGGATTGGAGCCCATCCAACAAGAGATGTTT

CTAGATATAGCATGCTTCTTGCGAGGGGAAGAAAAAAATTACGCCATGCATGTTCTTGAG

AGTTGTCATTCTGGAGTTGAATATGGATTGCGTGTCTTAATTAACAAATCTCTTGTGTCT

ATCTCTGAAAATGATCAAATTCAAATGCATGACTTAATACAGGATATGGGTAAATATATA

GTGAATTTTAGAAAAGATCCTGAAGAACGTAGCAGGCTATGGCTCGCCGAGGAAGTCGAA

GAAGTGATGAGCAACAATGGAGGGACCATGGCAGTGGAAACAATTTGGCTTCATTGTAAT

TTTAGGACACTAAGCTTTAACAATGAGGCCATGAAACATATGAAAAGGCTTAAGATATTA

AACATAAAGAGTTGGGCCTATCATGGCTCCTTTGAGTATCTGTCCAACAACTTGCGTTGG

TTAGTCTTGGATGGCTATCCTTGTGAAACATTGCCATCTACATTTGATCCCAAAATTCTT

GTTAGCCTTAACCTCCGGGGCAGTTCACTGCATTATTTATGGATGGAAACAAAGCAATTG

TCGTCTCTACGGACGCTAGATCTCAGATACTCTAAAAGCCTGGTGCAAACACCAGATTTC

ACGGGGATGCCAAATTTGGAGTATTTGAATCTGTCTTCGTGTTCTAATCTTGAAGAGGTT

CACTATTCCCTGGGATGTTGCAACAAACTCATTCGGTTAAATTTGTGTTTTTGTAAAATA

CTTAAGAGGTTTCCATGTGTTAACGTGGAATCTCTTGAATATTTGGGTTTAGAATTTTGC

TATAGGTTAGAGAAATTTCCAGAAATTCACGGGAGAATGAAGCCTGGGATACAGATTCAC

ATGTCATCCTCTGGGTTAAGGGAACTGCCATCCTCTGTTTTTCAGTACCAAACTCATATT

ACCAAGCTAGATTTGAGTAATATGGAAAACCTTGTAGCTCTTCCAAGCAGCATCTGTAGG

TTGAAAAGTTTGGTTAGTCTGAGTGTGAATTGCTCAAAACTGGAAAGCTTGCCAGAAGAG

ATAGGGGATTTAGACAACTTGGAGGAGCTTGATGCCAGTGCTACTCTAATTTCACGACTT

CCGTCTACCATTGTACGCTTGAACAAACTTAAAGTCTTGAAGTTAGCTGGATGCTTCAGA

TACAATAGAGTGCACTTTGAGTTCCTTCTGGCTGAAGGATTACGCTCATTGGAACATCTG

GATCTCAGTCGCTGCAACTTAACAGATGGAGAACTTCCGGAAGATATTGGATCCTTATCC

TCTTTGAAAGAGTTGGAGCTCAGAGGAAATAATTTTGAGCATATTCCTCGAAGCATAGCC

CAACTTGGTGCTCTTCGATCCTTAGACTTATCATATTGCAAGAGGCTTACACAACTACCA

GAATTTCCCCCTGAATTAAATAAATTGCGTGTAGATTATCTTATGGCTCTGAAATTTATC

CATGATTTAGTAACAAAGAGGAAGAAACTACAGATGGTGATATTCGCACCACTGTATGAT

AAGGATGATGCAGACAATAATTCTATATATAATTTGTTTGCACATGCCCTGTTTCAGAAA

ATCTCTTCCTTGAGGCATGACATCTCTTCTTCAGATTCCTTGTCCGAAAATGTGTTTACC

ATTTGGCATCATGAGAAGAAGATCCCAAGTTGGTTCCACCATCAGGGAAGGGATAGTAGT

CTATCAGTCAATTTGCCCGGAAATTGGTATATACTTGATAAATTCTTGGGATTTGCTGTA

TGTTACTGTGGTAGCTTAATTGACACCACAGCTCAATTGATTCCCGTATGTGATGACGGG

GTGTCGTGCATGACCCAGAAACTTGCCTTATCAGAATGTGATACAGAATCACACAGCTAT

CGAGAACGGTATACACCAATTCATTTTTTCTTGGTACCTCTTGCTGTCTTATGGGATACA

TCTAAGGCAAATGGAAAAACACCAAATGACTATGGACTTATTAGGCTATCTTTTTCTGGA

AAACAGAAGAAGTATGGACTTCGTTTGTTGTATAAAGAAGAACCTGAGGTTGAGGCCTTG

TCACAAATGAGGGAAAATAACAATGAACCAACAGAACATTCCAATGTGATAAGGAGGAGC

AGATCTGACATTAGTGAACACCATGACTCCGTGACGGATGAATCCAGTTGTTGTTGTTGT

CGCATACTGTAA

>NTBX_mRNA_32477_cds NTBX_mRNA_32477 gene_18953|id=AT1G27170.1

ATGGAGGAAATTCAGACCACAACTTCACTCCCATCGCTAAGGCTGAATTACGACGTGTTC

TTGAGTTTTAGAGGCGAAGATACTCGCGAAAACATCACTAAAAACTTATACGATGCCTTA

TACTCAAAAGGCGTCCGAGTGTTTCGAGACACAAACGGGTTAACTCAGGGCGACGAGATC

GCACCAGGTCTTATGGACGCAATCAACGATTCAGCTGCAGCTATTGCTATTATTTCACCC

AATTATGCTTCGTCGAGATGGTGTCTAGAGGAATTAGCAACGATTTGTGAGTTGGGTAAA

CTCGTTCTGCCCGTGTTCTACCGGGTTGACCCGTCGGATGTTCGAAGGCAGAGAGGACCG

TTTCTACATGATTTTGAGAGTTTGGAAGGAAGATTTGGAGTGGAAAAGGTGGTGAGATGG

AGAAATGCTATGGAAAGAGTTGGGGGAATCTCCGGCTGGGTTTATTATAATAGTGAAGAG

TCACAGTTGATACAGACTTTGGTGAAAAGAGTTTTACAAGAATTGAGCAATTCCCCAATA

TTTGTAGCTCCATTTGTTGTTGGAATTGACTACCGTCTGGAAGAACTCATAAGACAGTTA

GATGTGAAGCGCAGTGGTGTCAAGATCATTGGGTTGCATGGAATAGGAGGAGTTGGTAAA

ACAACTCTTTCTAAGGCTCTTTATAATAAACTTGCTTCTCATTTTACACACAGGGCTTTT

ATCTTGAATGTTAAGGAAATAGCTGCTCAACAAGGCATTGTGTCCGTTCAGAAGAAAATA

ATACAAGGTCTTTTCCCGAGCAAGGTCTTCTCCTTCTCCCCTGGTAATGCACATGAAAGA

AGAGTAAAATTCGGACGATTTCTTCAAGAAAAGCGTGTCCTGCTCGTCTTAGATGATGTA

GATTATGTAAATGATGATGTAAGCATATTGAAGGCACTAATTGGAGGGAAAAACTGGTTC

TTTGAAGGGAGCAGGGTTGTTATTAGTACTAGAAACAGAGGAATTTTGCTAGAAGACATC

GTTAACGAGACATTTGAGGTGAGAGAATTGGGTGGTCCTGACTCACTAAAACTATTCAGT

TACCATGCATTTAGAAGACAGGAGCCATTTCCAGCTTTTGTGAATATGTCCAAGCAAATT

GTCTCAATCACTGGAGGGCTACCCTTGGCTCTTGAAGTTTTTGGTTCTTTCTTGTTTGAT

AAAAGAAGCGAGGAGGAATGGCTAGATGCTCTAGAAAAGCTAAAACAAATTCGCTCTCCA

CATCTTCAGGAAATCTTGAAAATAAGTTATGATGGTCTTGATGATGAAGAGAAGTGTATA

TTCCTGGATGTTGCATGTTTATTTCTTGATCAATTAGAAAAGAAAGCTGAAGATGTAATT

GATGTGATGAAAGGATGTGGTTTTAGAGCCAGCATTGCATTTGACACTTTAACTGCTAGA

TCATTGATTAAGGTAATTGATGGTGGGGATTTGTGGATGCATGACCAGATAAGAGATATG

GGAAGACAAATTGTTATACAACAAGGCATTTCAGATCCCGGAAAGCGCAGCAGACTTTGG

GATGTTGCTGATGTTTTGAGTGTGTTACAAGGAAGGAAGGGGACACAGAACATCCAAGGG

ATCATCCTGGATCAGTATCAGAAGCCATCATCAAAGATTAAAAGCACGAAAGCAATTACT

AGAGAGCATTTTCAACAAGTTCCCACTTTTACTTCTGCATTAGCTTACATTAAAGAGTTG

TGCAAAGAACAATTTCAAAATGATGCAAAAGAAACCAATGATTTGGTATTGAACACTGAA

GCATTTGATCCAATAGTTAATCTGCGGCTACTCCAATTCGATAATGTGAAACTAGAGGGA

AATTTGGGGAAGTTACCTTCTTCACTAAAATGGCTCCAATGGAAAAGGTGCACACTTTCA

AGCTTTTATTCTGATTATTATCCAAGTGAACTTACCATGCTTGATCTCTCAGAGAGCCAA

ATAGAGAAGTTTGGAAGCCGGGAATGGACTTGGACTCGCAAAAAGGTGGAAAACAAGTTG

ATAGTTATGAATCTCTCTGGTTGTCATAAAATAACAGCTATTCCTGATTTATCCACGCAT

AAAGCATTGGAAAAGTTGATAGCTGAACGTTGCAGTGCATTGCAAAGGATTCACAGAACA

ATTGGGAATCTGAAAACTTTACGTCATTTAAATTTAAGAGATTGCCGCAACCTTGTTGAA

TTTCCAGGTGAAGTCTCCGGGCTGAAAAATCTTCAAAAGCTGATACTCTCGGGCTGCTCG

AGATTGAAACAGTTACCTGAAGATATAGGCAAGATGAAGTCCTTACAAGAACTTCTATTA

GATGGGACTGCTATAGAGAAGTTGCCTGAAAGTATATTTCGCTTAACAAAACTTGAGAAG

TTAAGCTTAAGCCAGTGCCACTCACTGAAACAACTTTCCCGGTTCATAGGAAAGCTAAGT

TCTTTGAAGGAACTCTCTCTTAATGGTTCTGCTTTGGAAGAAATACCTGATTCTATTGAA

CATTTGCAGAACCTTCATACATTAAACTTAATTAGGTGTGAGTCACTTGCTGCTATTCCC

AATTCTTTTGGCAACCTCAAATCTTTAGCAAATCTCTGGCTTTATGGCAGTGCAATAAAA

ATGATGCCAGAATCTATTGGTTCTCTGTATTATCTTAGGTCCTTATCGCTCGGAAACAGT

CAGCATTTAAATGCATTGCCTGTTTCAATTAAAGGATTGTCTTCTTTGGTTGAGCTTCAA

ATAGACAAGGTTCCAATTATTAGTCTTCCAGATCATGTTTTTGGTGGACTTAAATCACTG

AAGAATCTTGAGATAAGGAACTGTGAGCGCCTTGGCTCGCTTCCCCACTCCATTGGAGAA

TTGTTAGCTCTTAGAACAATGACTCTTACCAGAAATGATGCTATTACGGAGCTGCCAGAA

TCAGTTGGGAATTTGCAGAATCTTGTCATATTGAGATTGACCAGATGTAAGCGACTTTGC

AAATTGCCAGCTTCAATTGGGGAACTAAAGAACTTAGTACACCTGCTAATGGAGGAGACT

TCAGTAACAAAATTACCTGAAACATTTGGGATGCTATCGAGCTTAATAATTCTGAAGATG

GGAAAGAAGCCTTTCTGCCAGGTATCACAAAGTACTGAAAACACAGAAGCAGCTACCTAC

ACAGAAAGGGAAACATCACCTGTTGTGCTTCCTTCATCTTTCTCAGAGCTATCCATGTTA

GAAGAACTTGATGCCCGCGCGTGGGGAATAGTTGGGAAAATACCGGATGATTTTGAGAAA

CTATCATCTTTGGAGATCATCAATCTTGGTTTCAATGATTTTTCCTATCTCCCGTCTAGT

CTGAAAGGACTACTTTTCTTGAAAGAGCTCCTTGTTCCCCACTGCAAACAGTTGAAAGCT

ATTCCTCCTCTTCCCTCAAGTTTGCTCAAGATAAATGCTGCAAACTGTGGAGCACTCGAG

AGCATACACGATATCTCAAAATTAGAGTTCTTGCACGAGCTAAACCTTGCAAATTGCATG

AGTTTGGTAGATATCCAAGGTATCGAATGCTTGAAATCCTTAAGAATGCTACATATGGCT

GGATGCAATGTCTCCTGTGCCTTTATGGTTAGAAGCAAACTTGATAAGGTGAGGTGCATT

TCTCAAAACACAGAAACAATGAAATTAAAGCAGTGGTTATTGCCATAG

>NTBX_mRNA_95914_cds NTBX_mRNA_95914 gene_57325|id=AT5G36930.1

ATGGATACTCAATTAGTCAGAGGAGAATCATCTCACTTCTCTTATGAAGTATTCCTGAGT

TTTAGAGGTGAAGACACCCGAAAAACATTCACTGGTCATCTTTATTCCAAATTGTCTGAT

GTTGGAGTTAATACCTTCATTGACGATGAGGAATTGAGAAAGGGTGACGTGATTTCAAGA

GAATTAGAGAAAGCAATTGAAGAGTCAAGAATTTCCATTATTGTTTTCTCAAGAAATTAT

GCTTCCTCTAGTTGGTGTCTAAATGAACTAGTTAAAATTCTTGAATGCAAAGATAAACTA

AAGCAGATGGTTTTGCCTATTTTCTATGATGTTGATCCTTCTGAGGTACGAAAGCAAACT

GGGTTATTTGGTGAATATTTGGCTAAACACAAGGAACGACCATTTGGAGCTCAAAGGGTG

GAGAAGTGGATAGCTGCACTTACTGAAGCTGCAAATTTATCTGGATGGGATTTGCAAAAT

GTTGCTGACGGGCATGAATCAAAGTTTATTGAAAAAATTATACAGCAAGTCCTACAAGAG

GTCAACCAGACACCTCTAGATGTTGCTTGGCACCCAGTTGGAGTAGATTCTCGTGTCAAA

GATATAGAATTGTTATTGCAAAATGAATGTGAAGATGAAGTTCGCATGATTGGTATTCAC

GGAGTTGGTGGCATAGGGAAAACAACTCTGGCAAAAGCTATCTACAATCGAATGTTTCGA

CTCTTCGATAGTAGTTGCTTCCTTTCAGATGTTAGATCAGAAGCTGAAGAAGTTGGTCTT

GTCAAGCTACAAGAGAAACTTCTTCAACAAGTTCTCAAAACTGAGGACATCAAAGTTGGA

AGTGTTGCTCAAGGCATCAATCTAATCAAAGCAAGGCTTGGGTCAAAGAAGGTTCTAATT

GTTCTTGATGACGTGGACCACAAAAAACAATTAGAATCATTAACGCGAGAAAGAAGTTGG

TTTGGTTTGGGTAGTTTAATAATCATTACCACCCGAGACGAACGATTGCTATGTCGGCTT

GGAGAAAAAGAGAGATATGAGGCCAAACTATTAAATGACAATGAAGCTATGTTACTTTTT

TGTTGGCATGCTTTTGACCGTCATTTTCCACCAGAAGATTATGTTAATTTGGCACGAGAC

ATAATCAAATATTCAGGTAGGTTACCATTAGCTCTTGTGACATTGGGGTCACATTTACAT

GGAAGTTCTGTAGAAGAATGGGGCCATGAATTTGAAAAACTAAGAGCGATTCCTCATTGT

GATATCCAAAAGATTCTCAAGATAAGCTTTGATGGACTTGATGATGAAACACAGACTGTT

TTCCTCGATATTGCATGTGCCTTCCATGGGTTTGATGAGCATGAAGTTACTGAAATATTA

AATGCATGTGGCTTTCATGCTAAAATTGCAATTGCAACTTTAGTCCAAAAACACTTGCTC

CAAAAATCTTGGAATATTTTGGAGATGCATGATCTAGTGCGAGATATGGGAAGAGAAGTC

GTTCGCATGGAATCAGCTCGGGATCCTGGAAAACGGAGTAGATTGTTCATCCCGCAAGAA

GTCTGTGATGTTCTACAAGGAAATAAAGGTTCCAAAAAGGTAGAAGTACTGAAGGTAGAT

CGACGAGCATTTGAGGGAGTGAACTTGAGCACCAAAGCATTTAAGAAAATGAAAAACCTT

AGGGTTCTTATAATGGATGAGTTACATATTAGTGGAGATTTTGAGCTGTTGTCCAAGGAG

CTCAGATGGTTGTCTTGGAAAAAATGTCCTTTAAAATGTATACCATCAAATTTTCCAGCT

GAGAATCTTGTAGTTCTAGATATGCGGGAGAGTGATATCCAAGAATTTCAATTGAATTTG

CAGTGTTGCAGAAGTTTGAAGAAGTTGGATCTCTCTTATTGCAAGCAACTCAGAAGCACT

CCAAACTTCACTGGTTCAATGAGTCTTGAGAATTTGTCCCTTGGTAGTTGCTCAAGTCTG

GCAGAGATACATCCATCAATAGGAAATTTGGACAGACTAATTAAACTAGATATGTCTAAT

TGCGGAAAAATTATGGATCTTCCAAGCAGCATATGCCAGCTAAAATCCCTTGAAGACTTG

GACATTGATGGCTGCTCATCTATAAAAGCACTGCCAGATAACCTTGGAGATTTGAAAAGT

CTAAGATCTCTTGATGCATATGATACGGGTATAAAACAAGTGCCTAGATCTGTTGAAATG

CTAAGAAATCTTGAAACTTTGAGAGTGGGAGGTCGAAAGCTAGAGGCCAAAAGGAGTATT

TCTGGAAGAGGAGTCCATCGGATACAATATTCCTTGTCAACTTTTGTATCCGATTTGAGC

CTTACATACTGTAATTTGTCCGAGGCTGATATTCCTAGGAATATTGGGAGCTTATCCTCC

TTAGAATATTTAGATTTGAGTGGCAACAGTTTCCATTGTCTACCCATTGATTTTTCTAAG

TTACGATTATTGGTGGAGTTGTGTTTGAATGACTGTGAGAATCTTCAAACACTCCTGTCA

GTATCAAATTTAGAGAATCTTGCAATTATTGAACTTGAGAATTGCCAAAAATTGGTCAAG

ATTACAGAGTTGGACAACCTCCCTTCTATATGGTCGATCAACATGATAAATTGTAGTTCT

CTGCAGAATCCATTCAATGAAGGCTTCTTTAGTGCACCTGCTCTATCATTTCTATCTAGA

AAAGATCCTGATTTGCGTGATTTAGAAGTTTATCTCCAATGCAATGAGATTCCAGAATGG

TGCAGGAATCAAGTAACAGCTTCATCTATGCGTTTGACTATGCCGATACATAATAATAAG

GAGTATAACTTCTTAGGAATGGTTCTCTGGTTTGTTTTCTGCTTTTTCGATGAAGCCCCT

TTTCCAAGCTTCTCAATTAGTATTGCCCATAAAAAGACTTTAATTGAGCCGTTGAATATA

CCTGATGAACACAGAGAACTGACATTTGTGTGTTACATATCTTACTTAGATGAACCTTTT

GATGGCCAGATAATCAAAGGTGGGAAAAGGATAAAAGTGTGGTCTGACGACTTTACAGTA

AAGAAGATAGGGATCCATCTGTTATATTTAGACCAACATGGTAATGTTATATCTTTACCG

GGAGACGTGGATCATTCTTATACTAGGGCGAAAGATGTCAGGAATTGGTGGAAATAG

>NTTN90_mRNA_88234_cds mRNA_88234 gene_49867|id=AT5G17680.1:evalue=7e-177:annot='disease resistance protein (TIR-NBS-LRR class), putative';id=Solyc01g008800.1.1:evalue=0.0:annot='Tir-nbs-lrr, resistance protein'

ATGGCATCTTCTTCTTCTTTCGCGAGTAATTCACAGCACTGTCCTCGATGGAAGTACGAT

GTTTTCCTAAGTTTTAGAGGTGAAGATACTCGCAAAACGTTTACAGGGCACTTATATGAA

GGCTTGAGAAATGGGGGAATATTTACCTTTCAAGACGACAAAAGGCTAGAGCATGGCGCA

TCCATCTCAGAAGAACTTTGTAAAGCTATCGAAGAGTCTCAAGTTGCCGTCATCATTTTC

TCAAAGAATTATGCTACATCGAGGTGGTGCTTGGATGAACTAGTGAAGATTATGGAATGC

AAGACTCAATTTGGACAAACTGTCATACCGGTCTTCTATGATGTGGATCCATCACATGTT

CGGAACCAAAGGGAGAGTTTTGCAGAAGCATTTTCCAAACATGAATCAAAGTTTAAGGAT

GATGTTGAGGGAATGCAGAAGGTACAAAGATGGAGGAGTGCTTTAACTGAAGCGGCAAAT

CTCAAAGGTTGTGATATTCGTGCCAGGATTGAATCAGATTGTGTTCAGCAAATCGTTAAC

CAAATTTCCAAGTTATGCAAGTTTTCTTTGTCTTACTTGCAAGATATTGTGGGAATAAAT

CCACATTTAGAGGAAGTAAAATCCCTACTACAGATAGAAATCAATGATGTTCGGATTGTG

GGGATCTGGGGCATGGGAGGAGTTGGTAAAACGACAATAGCAAGGGCTATTTTTGATACT

CTCTCATATCAATTTGAAGGTACTTGTTTCCTTGCGAATGTTAAAGAAAACAAATGTGGA

ATGCATTCTTTGCAAAATATCCTTCTCTCAGAACTGTCAAGGGAAAACGCTAATTACGTG

AATAATAAGGAGGACGGAAAGCAGCTGATGGCTCGTAGACTTCGTTCTAAGAAGGTTTTA

GTTGTGCTTGATGACATAGATCACAGAGACCATTTGGAGTACCTAGCAGGGGATCTTGGT

TGGTTCGGCAATGGCAGTAGAATTATTGCAACAACAAGAGACAAGCATTTGATTGGGAAG

AAGGACGCATTATATGAAATGACTACACTAGCTGACCATGAAGCTATTCAATTGTTCAAT

CGATACGCTTTTAAGGAAGATGTTCCAGATGAGTTCTTTGAGAAGCTAACGCTGGAGGTA

GTAAGTCATGCTAAAGGCCTTCCTTTAGCGCTGAAAGTGTGGGGTTCTCTCTTTCATAAG

AGAAATATAACTGAGTGGAGAAGTGCTATACTGCAAATGAAAAAACACTCTAATTCAGAA

ATTGTTGACAAGCTCAAAATTAGTTATGATGGATTAGAGCCCGTGGAACAGGCGATATTT

TTAGATATAGCATGCTTCTTACGAGGGAGAGAAAAGAATGAGATCATACAGATTCTTGAG

AGCTGTGAATTTGGAGCTGATATCGGATTGCGTGTCCTAATTGACAAATCTCTTGTGTTC

ATCTCCGAAAAAGATACGATTGAAATGCATGACTTAATACAAGATATGGGTAAACATGTA

GTGAACATACAAAAGGATCCGGGAGAACGTAGCAGACTATGGCTCGCTGAAGATTTCGAA

GAAGTGATGATCAACAATACGGGGACAAAGGCAATGGAAGCAATTTGGTTTCTTTATTTA

AAAGGACTATCTTTTACCAGAGAGGGCATGAAAAATATGAAAAGGCTTAGGATATTATAT

ATACGTGATGGGTCACAGAATATCTGGGCTGACTCCATTTGCCATAATGGCTCCATGGAG

TATTTGCCCAACAACTTGCGTTGGTTTGTCTGGTTTTGCTATCCTTGGGAGTCATTGCCA

TCTACATTTGAACCCAAAAAGCTCGTTCATCTTGAACTCCAGTCCAGTTCACTGCGTTAT

TTATGGACGGAAATAACGCATTTGCCGTCTCTAAGAAAGCTAAGTCTCAGTTGCTGTGTA

AGCCTAATGCAAACACCAGATTTCACGGGAATGCCAAATTTGGAGTATTTGGATTTGAGT

CTTTGCAGTAATTTTGAAGAGCTTCACTACTCCCTGGGATGTTGCAGAAAACTTGTCGAG

TTAAATTTGATTTGGTGTGATGGCCTTAAGAGGTTTCCATGTGTTAACGTGGAATCTCTT

GAACATCTGGAACTGCATGGTTGCTCCAGTTTACAGAAATTTCCAGAAATGAAAAACCTA

TCATCTATTCAGTACCAAACTCATATTACCAATCTAGACTTGAGCTTCTTAACAGACCTT

GCAACTCTTCCAAGCAGCATCTGTAGGTTGAAAAATTTGGTTAGTCTAGATGTGTCATTT

TGTTACAGACTTGAAAGCTTGCCCGAAGAGATAGGAGATTTAGAAAACTTGGAGAATCTT

GATGCTAGCAGTACCCTAATATCACGGCCTCCCACTTCCATCGTCCGTTTGAACAAACTT

AACTCCTTGGCTTTTGCAAAAGGAAAATCAGATAATGGTCAAGTTGCAGTTTACTTTGTG

TTCCCTCCCGTGGCTGAAGGGTTACGGTCATTGGAATTTTTGAATCTCAATTACTGCAAT

CTAATAGATGGAGGACTTCCAGAAGACATTGGCTGCTTATATTCTTTGAAAGAGTTGTAT

CTCAGGGGAAACTATTTTGAGCATTTGCCTCAAAGCATGGCTCAACTCGGTGCTCTTCGA

TCCTTGGACTTATCATATTGTTATAGGCTTAAAGAGTTGCCAGATTTCATGGGAATGCCA

AATTTGGAAACTTTGAATCCATTATATTGTATGAATCTTGAAGAGGTTCCTCATTCCATA

GGAGTTTTTGAAAAGCTCGCTGAATTGACTTTGACTGATTGTGAACGCCTTAAGAGGGTT

CCAACTCTGTGGATCGATTCCCTTAAATGTCTGCAGCTAAAAAAATGCTCTAGTTTAGAA

TATTTTCCTGATATCCGCGGAAGCATGAAATTGGAGTTAGAGATTCACATGCTAGGCAGC

GTGATAAGGGATCTTAATTCGTTCTATAATTCGTTTCAACTTACCTTGTATCAGAATGAC

ATCTCTATTTCAGATTCCTTGTCACAAAGAGTGTTTACCATTTTGCATAAAGGGAAGAGG

ATTCCAAGTTGGTTCCGCATTAAGGGAATGGATAGTAGTGTATCAGTCAATTTGCTTGAA

AATTGGTATGTACGCGATAACTTCTTGGGATTTGCTGTATGTTACTCTGGCTGCTTAATT

GACACCACAGCTCACTTGATTCCCTTTTGTAATGATGGGATGTTGGCGATGACCTTGGAA

CTAGGTTTTTTCTACCATTTAGAATGTGATGAGGAATCTACTATTCATCCTTTTAAAAAA

AAATGGGATGGAGAATCTACTATTCATTTTTTCTTGGTACCTTTTGCTAGTTTATGGGAT

GCATCTAAGGCAAATGGCAAAACACCAAATGACTATGGGATTATTAGGCTATGGTTTTCT

GGAGCAATGAAGGAGTTTGGATTTCGTTTGTTGTATAAAGATGAACTTGAGATCGAGGCC

TTGTTACAAATGAGGGAAAATAATGATGAGTCAGCAGAACGTTGCACTGGGATAAGGAGG

AGCAGATATGACAATGAAGCCAGTTGCTCCTCTGGTAAGAAACAAAGGTCACATTCTAAT

ATTCAGGGCAGCTCTGTCTTTGAGAATTTGCAGCAACAAGTAGAGCCGCCAGTCTCTTCA

GAAATTTTGAGGCTCAATCGTTCATTCCCAGAAATTTTTCCTAGTTGGCGGTGA

>NTTN90_mRNA_125457_cds mRNA_125457 gene_70838|id=AT5G17680.1:evalue=6e-31:annot='disease resistance protein (TIR-NBS-LRR class), putative';id=Solyc11g011350.1.1:evalue=0.0:annot='Tir-nbs-lrr, resistance protein'

ATGGCATCATCTTTTGCTTCTGCGAGTACTTCACAGTTTCCTCGATGGAACTACAAAGTC

TTTCTAAGTTTTAGAGGTGAAGATACTCGAAAAACATTTACAGGTCACCTCTTCAAAGGG

TTGGAAAACAATGGAATATTTACGTTTCAAGATGATAAAAGGCTAGAGCATGGCGCATCA

ATATCAGATGAACTCTTGAAAGCTATCGAACAGTCTCAAGTTGCCCTCGTCGTTTTCTCA

AAGAATTATGCAACATCGAGGTGGTGCTTAGATGAGTTAGTAAAGATCATGGAATGCAAG

GATCAATGTGGACAGACTGTCATACCAGTCTTCTATGATGTGGATCCATCACATGTTCGA

AACCAGAGAGAGAGCTTTGCCGAAGCCTTTGACAAACACGAACCAAGATATAGGGATGAT

GATGAAGGAAGGCAGAAGCTCCAAAGATGGAGGAATGCTCTAACTGCTGCCGCAAATCTA

AAAGGATATGATGTCCGTGACGGGATTGAAGCAGAGAATATTCAGCAGATTGTCGACCAA

ATTTCCAAATTGTGCAATAGTGCTACTTTGTCTTCTTTGCGAGATGTTGTAGGAATAGAT

ACTCATTTGGATAAATTAAAGTCCCTACTTAAGGTAGGAATCAATGATGTTCGGATCATA

TTGGGGATCTGGGGCATGGGTGGACTAGGGAAGACGACGATAGCAAGAGTCATTTTTGAC

ATTTTATCTCATCAATTTGAAGCTGCTTGTTTCCTTGCGGATATAAAAGAAAATGAAAAA

AGACATCAACTGCATTCTTTGCAAAACACCCTTCTCTCTGAATTGTCAAGAAGAAAAGAT

GATTACGTCAATAATAAGCATGATGGGAAGCGGATGATTCCAGACAGACTTTTCTCTAAG

AAGGTGCTAATTGTGCTTGATGATATAGATCATAAAGATCATTTAGAGTATTTAGCAGGT

GATATTGGTTGGTTTGGTAATGGTAGTAGAGTTGTTGTAACAACTAGAAACAAACATTTG

ATAGAAAAGAATGATGTCATTTATGAAATGACTGCACTATCTGATCATGAATCCATTCAA

TTGTTCTGTCAACATGCTTTCAGAAAAGAAGATCCAGATGAGCATTTTAAGAAGCTTTCA

TTGGAGGTAGTAAAATATGCCAATGGCCTTCCTTTAGCCCTCAAAGTGTGGGGTTCTCTG

CTGCATAACCTAGGCTTAACTGAATGGAAAAGTGCAATAGAGCAAATGAAAATTAATTCT

AATTCGGAAATTGTTGATAAGCTCAAAATCAGTTATGATGGATTAGAGCCCATACAACAG

GAGATGTTTCTAGATATAGCATGCTTCTTACGAGGGGAACAAAAAGCTTACATCCTACAA

ATTCTTGAGAGCTGTCATATTGGAGCTGAATATGGATTGCGTATTTTAATTGACAAATCT

CTTGTGTTCATCACTGAAGATTATCAGATTATTCAAATGCATGACTTAATTCAAGATATG

GGTAAATATATCGTGAACTTGCAAAAGAATCCGGGAGAACGCAGCAGACTATGGCTCAAC

GAGGATTTCGAAGAAGTGATGACCAACAATGCAGGGACCGTGGCAGTGGAAGCAATTTGG

GTTCATGATTTGGATACACTACGCTTTAACAATGAGGCCATGAAAAATATGAAAAAGCTT

AGGATATTATACATAGACAGAGAGGTCTATGATTTCAATATTAGCGATGAACCCATTGAG

TATCTATCCAACAACTTGCGTTGGTTTAACGTGGATGGCTATCCTTGTGAGTCATTGCCA

TCTACATTTGAACCCAAAATGCTTGTTCACCTTGAACTCTCATTTAGTTCACTGCGTTAT

TTATGGATGGAAACAAAGCATTTGCCGTCTCTACGGACGATAAATCTCACGGGCTCTGAA

AGCCTGATGCGAACACCAGATTTCACGGGGATGCCAAATTTGGAGTATTTGGATATGTCT

TTCTGTTTTAATCTTGAAGAGGTTCACCATTCCTTGGGATGTTGCAGCAAACTCATTGGG

TTAGATTTGACCGATTGTAAAAGCCTTAAGAGGTTTCCATGTGTTAACGTGGAATCTCTT

GAATATCTGGATTTACCAGGTTGCTCAAGTTTAGAGAAATTTCCAGAAATCCGCGGGAGA

ATGAAGCTGGAGATACAGATTCACATGAGATCTGGGATAAGGGAACTACCATCATCTAGT

TTTCACTACCAGACTCGTATTACCTGGCTAGATTTGAGCGATATGGAAAACCTTGTAGTT

TTTCCAAGCAGCATCTGTCGGTTGATAAGTTTGGTTCAATTATTTGTGTCTGGTTGCTCA

AAACTGGAAAGCTTGCCAGAAGAGATAGGGGATTTAGACAACTTGGAGGTGCTTTATGCC

AGTGATACTCTAATTTCACGACCTCCATCTTCCATCGTACGCTTGAACAAACTTAACAGC

TTGAGCTTTAGGTGCTCCGGAGACAATGGAGTGCACTTTGAGTTCCCTCCAGTGGCTGAA

GGATTACTGTCATTGAAAAATCTAGATCTCAGTTATTGCAATCTAATAGATGGAGGACTT

CCGGAAGACATTGGATCCTTATCCTCTTTGAAAGAATTGGATCTCAGAGGAAATAATTTT

GAGCATTTGCCTCGAAGCATAGCCCAACTTGGTGCTCTTCGATCCTTAGGCTTATCATTT

TGCCAGACGCTTATACAACTGCCAGAACTTTCCCATGAATTAAATGAATTGCATGTAGAT

TGTCATATGGCTCTGAAATTTATCAATGATTTAGTAACAAAGAGGAAGAAACTACAGAGG

GTGGTATTCCCGCCACTGTATGATGATGCACACAATGATTCTATATATAATTTATTTGCA

CATGCCCTGTTTCAGAATATCTCTTCCTTGAGGCATGACATCTCTGTTTCAGATTCCTTG

TTCGAAAATGTGTTTACCATTTGGCATTATTGGAAGAAGATCCCAAGTTGGTTCCACCAT

AAGGGAACTGATAGTAGTGTATCAGTCGATTTGCCTGAAAATTGGTATATACCTGACAAA

TTCTTGGGTTTTGCTGTATGTTACGATGACATTTTAATTGACACCACAGCTCAATTGATT

CCCGTATGTGATGATGGGATGTCGTGCATGACCCAGAAACTTGCCTTATCAGAATGTGAT

ACAGAATCATCCGATGATTCAGAACGGTATACACCAATTCATTTTTTCTTTGTACCTCTT

GCTGTCTTATGGGATACATCTAAGGCAAATGGAAAAACACCAAATGACTATGGGATTATT

AGGCTATCTTTTTCTGGAGAAATGAAGAAGTATGGACTTCGTTTGTTGTATAAAGAAGAA

GCTGAGGTTGAGGCCTTGTTACAAATGAGGGAAAATAACAATGAACCAATAGAACATTCC

AATGTGATAAGGAGGAGCAGATCTGACAATAGTGAACACCATGACTCCGTGACCGATGAA

TCCAGTTTATGTTGCTGTCGCATACTGTAA

>NTK326_mRNA_65174_cds NTK326_mRNA_65174 gene_37742|id=AT5G17680.1

ATGTTGACTTGTTCAAGTAGCTCTTCATCTTCCAATTATGGACGCAGTTATGAGGTTTTC

CTAAGCTTTAGGGGAGAAGACACTAGAAAAACATTTGTGGGTCATCTTTTTAATGCTCTT

ATTGAAAAGGGTATTCACACATTCATGGATGATAAGGAGTTAAAGAGAGGGAGGTCAATC

TCATCTGAACTCATGAAAGCCATTGGAGAATCAAAATTTGCTGTTGTTGTCTTTTCCAAG

AACTATGCTTCCTCTACATGGTGCTTGGAAGAACTGGTAAAGATTCTTGAAATCCATGAA

AAATTTGAGCTAATTGTTGTCCCAGTTTTCTATGATGTGGATCCATCAACTGTGAGGAAA

CAAAATGGGGAGTATGCTGTGTTTTTTACTAAATTTGAGGCTGAATTGGTTGATGACAAG

GACAAAGTGATAAGGTGGAGGGAGGCACTTACTAAGGTAGCTAACATATCAGGGCATGAT

TTGCGCAACACTTACAATGGGGATGAATCAAAATGCATACAACAGATTGTGAGAGACATA

TTTGATAAATTTTGTTTCTCCATCTCAATAACTAACAGAGATTTAGTTGGAATAGAATCT

CAAATTAAGAAACTAAATTCATTGTTGAGGATGGACCTGAAAGGAGTTCGTCTTGTTGGG

ATTTGGGGGATGGGTGGTGTAGGAAAGACAACTGCTGCAAGAGCATTGTTCAACAGATAC

TATCAGAATTTTGAAGGTGCTTGTTTACTCGAAGATGTTAAAGAATATCTACAACACCAT

ACCTTGTTGTATTTGCAGAAGACTCTCCTTTCCAAACTGTTGAAGGTAGAATTTGTAGAT

TGTACTGATACTGAAGAAATGTGTGCAATATTAAAGAGGAGACTTTGTTCTAAGAAAGTT

CTGGTTGTTCTTGATGATGTTAATCATAGCAACCAGCTGGATAAGCTGGTTGGAGCTGAA

GATTGGTTTGGTAGTGGTAGTAGAATTGTTATCACAACTAGGGATATGAAACTGTTGAAA

AACCATGATGTGCATGAAACTTACGAGGTAAAGGTTTTGGAAAGAGATGAAGCAATTGAG

CTTTTCAATTTGCACGCATTCAAGAGAAAGTCACCTGAAAAGGAGTTTGAGGAGCTCTTA

AATCTTGTGGTAGATTATACTGGAGGCCTTCCTCTAGCTCTTAAGGTGCTTGGTTCTCTG

CTGTACAAGGAAGATCTTGATGTATGGATAAGTACAATTGATAGACTAAAAGATACCCCT

GAGGGTGAAATTATGGCTACACTTAAAATAAGTTTTGATGGATTAAGATATTACGAAAAA

AGTATATTTTTAGATATTGCATGTTTCTTCAGGGGTTACAACCAAAGAGATATGACAGCA

TTATTTCACGCTTCTGGTTTCCACCCAGTTCTAGGAGTAAAGACCCTTGTTGAAAAATCT

CTCATTTTTATTTTAGAGGACAAAATCCAAATGCATGATTTGATGCAAGAGATGGGTAGA

CAAATCGCAGTTCAAGAATTACCTATGAGAAGAATATATCGTCCAGAGGATGTTAAGGAT

GCATGCATAGGAGATATGGGGAAAGAAGCAATTGAAGGCTTGTTACTCACAGAGCCTGAA

CAGTTTGAAGAGAGTGAGTTGGAATATATGTACAGCGCTGAAGCTCTCAAAAAGATGAGG

AGGCTGAGGATACTTGTGAAACAATATTACAACAGGGGGTTTGATGAGCCTGTTGCCTAT

CTTCCTAACAGTCTGCTTTGGCTTGAATGGCGTGACTATTCTGCAGACTCATTGCCATCG

AATTTTGAACCATCAAAGCTCATATATCTTACTATGAAGAGTAGTTCTATCATCGAACTC

TGGAATGGAGCAAAGAGGTTAGCCCTTTTGACAACTCTTGATCTCAGTTATTGCTACAAA

TTGATACAAACCCCAGATTTTAAGATGATCGCAAAATTGGAAAGGTTGATCCTGAGCTTT

TGTGATGCATTGGTGGAAGTCCATCCATCTGTTGGGTTTCTCAAGAAGCTTATTTTGTTA

AATATGGATCATTGCACATCACTTGAGAGACTTCCGGAAATTATTCAATCAGAATGTCTT

GAAGTTCTTGATCTCAATTATTGCTTTAATTTGAAAAGGTTCCCGGAGGTGGAAAGGAAC

ATAAAGCACTTGAAGAAACTCGACCTCAGTTCAACTGGGATAAGAGAACTGCCGGCATCA

ATTGAGCATCTCAGTTCCCTAGAAAACCTACAATTGCATTCCTGCAACCAACTTGTACGT

CTCCCAAGTAGTATTTGGAGATTCAGAAATCTAAAGATTAGTGAATGTGAGAAACTGGGG

AGTTTACCAGAAATTCACAGGAATAGCAATTGTACTCGTGAAATCATCTTAAAATTAGTT

TCTATTAAGGAGCTTCCCACCTCTATAGGAAACCTCACCTCACTAAATTTCCTTGAAATC

TGTAATTGCAAAACTATTTCGAGTCTCTCGAGCAGCATATGGAGATTGACAAGTCTTACA

AATCTAAAGCTGTTAGACTGCAGAAAACTTAAAAACTTGCCCGAAATTCCAAATGCCATA

AATCATTTGGCAGGACATGGACTTCAGCTCCTTCTGACATTGGAGCAGCCCACAATTTAT

GAACATCTTGACTTGCTTAGGATTATTGATATGAGTTGGTGTAGTTCTATTAGCAGTCTC

CCCCACAACATTTGGATGTTGAAAAGTTTAAGGATTCTGCGCATCTCTTACTGCTCGGGA

CTGGAGTATTTACCAGAAAATTTGGGTCACTTGGAACATTTGGAGGAATTACTTGCAGAT

GGTACTGGTATTTTAAGACTACCATCTTCGGTTGCACGTTTAAATAAACTTGAGGTCTTA

TCATTCAGGAAAAAGTTTGTGATTGGACCAAAAGTCCAATATTCATCATCAATGCTCAAT

TTACCTGATGATGTTTTTGGAAGTCTTGGAAGCTTAGGCTCTGTGGTGAAGTTAAATCTT

AGTGGAAATGGTTTTTGTAATTTGCCTGAAACGATGAATCAACTTTTTTGCCTTGAATAC

CTTGACATAACATTTTGCCAGAGGCTTGAAGCATTGCCGGAGCTTCCCCCAAGCATCAAG

GAGCTATATGTAGATGAACACTTGGCCTTGAGAAGCATGGAA

>NTK326_mRNA_109110_cds NTK326_mRNA_109110 gene_64485|id=AT5G17680.1

ATGGCATCATCTTGTGCTTCTGAGAGTACTTCACAGTTTCCTCGGTGGAACTACAAAGTC

TTTCTAAGTTTTAGAGGTGAAGATACTCGAAGAACATTTACAGGTCACCTCTTCAAAGGC

TTGGAAAACAGTGGAATATTTACGTTTCAAGATGATAAAAGGCTAGAGCATGGCGCATCA

ATATCAGATGAACTCTTGAAAGCTATCGAACAGTCTCAAGTTGCCCTCGTCGTTTTCTCA

AAGAATTATGCAACATCGAGGTGGTGCTTAGATGAGCTAGTGAAGATCATGGAATGCAAG

GATCAATGTGGACAGACTGTCATACCAGTCTTCTATGGTGTGGATCCATCACATGTTCGG

AAACAGAGGGAGAGCTTTGCTGAAGCCTTTGACAGACATGAAACAAGCTATAAGGATGAT

GATGAAGGAATGGAAAAGCTCCAAAGATGGAGGAATGCTCTAACTGCTGCCGCAAATCTA

AAAGGATATGATGTCCGTGACGGGATTGAAGCAGAGAATATTCAGCAGATTGTCGACCAA

ATTTCCAAATTGTGCAATAGTGCTACTTTGTCTTCTTTGAGCGATGTTGTGGGAATAGAT

ACTCATCTGGAGAAATTAAAGTCCCTACTTAAGGTAGGAATCAATGATGTTAGGATCATA

TTGGGGATTTGGGGCATGGGCGGTCTAGGCAAGACGACAATAGCAAGAGCCATTTTTGAC

ACTTTATCTCATCAATTTGAAGCTGCTTGTTTCCTTGCGGATATTAAAGAAAATGAAAAA

CTGCATTCCTTGCAAAACACCCTTCTCTCTGAATTGCTAAGAAAGAAAGATGATTACGTC

AATAATAAGCTTGATGGGAAGCATATGATTCCGGACAAACTTTGCTCTAAGAAGGTGCTA

ATTGTGCTTGATGATATAGATCATAAAGATCATTTAGATTATTTAGCAGGTGATATTGGT

TGGTTTGGTAATGGCAGTAGGATTGTTGTAACAACTAGAGACAAGCATTTGATAGGGAAG

GATGATGCAATATATGAAGTGAGTGCACTACCTGATCATGAATCCATTCAATTGTTCTAT

CAGCATGCTTTCAAAAGAGAGGTTCCAGATGAGTGCTTTAAAGAGCTTTCATTGAAGGTA

GTAAATTATGCTAAAGGCCTTCCTTTAGCCCTCAAAGTGTGGGGTTCGTTGCTGCATAAT

TTACGACTAACTGAATGGAAAAGTGCTATAGAGCACATGAAAAGTAACTCTAATTCTGGA

ATTGTTGATAAGCTCAAAATTAGTTATGATGGATTAGAGCCCAAACAACAAGAGATGTTT

CTAGATATAGCATGCTTTTTGCGAGGGAAATATAAAGATTACGCCATGCAAATTCTTGAG

AGTTGTCATTCTGGAGTTGAATATGGATTGCGTGTCTTAATTGACAAATCTCTTGTATTT

TTTTCTGAAAAATGTCAGATTCAAATGCACGACTTAATACAAGAAATGGGTAAATATATA

GTGAACTTGCAAAAGAATCCGGGAGAACGCAGCAGACTATGGCTCGTCAAGGATTTCAAA

GAAGTGATCAACAACAATACAGGAACCATGGCAATGGAAGCAATCTTTCTTCCTTATTTC

AATTCGGGTATATTACGTTTTAGCAAAAAGGCCATGAAAAATATGAAAAGGCTTAGGATA

TTAAACATAGAGAGGTCGTTGACCTATGATGGTTCCATTGAGTATCTGCCCAACAACTTG

CGTTGGTTTGTCTTGTATGGCCATCCTTGTGAGTCACTGCCATCTACATTTGAACCCAAA

ATGCTTGTTCACCTTGAACTCTGGGGTAGTTCACTGCATTATTTATGGATGGAAACAAAG

CATTTGCCGTCTCTACGGAGGATAGATCTCAGCTCCTCTAGAAGCCTGATGCGAACACCA

GATTTCACGGGGATGCCAAATTTGGAGTATTTGAATATGTTATATTGTAGTAATCTTGAA

GAGGTTCACCATTCCCTGAGATGTTGCAACAAACTCATTCGGTTAAATTTGAATTATTGT

AAAAGCCTTAAGAGGTTTCCATGTGTTAACGTGGAATCTCTTGAATATCTGAGTTTAGAA

TATTGCTCTAGGTTAGAGAAATTTCCAGAAATCCACGGGAGAATGAAGCCGGAGATACAG

ATTCACATGAAACACTCTGGGATAAGGGAACTACCATCATCTATTACTCAGTACCAAACT

CATATTACCAAGCTAGATTTGAGCGGTATGGAAAAACTTGTAGCTCTTCCAAGCAGCATC

TGTAGGTTGAAAAGTTTGGTTAGTCTGAGTGTGTCGGGTTGCGTCAAACTTGAAAGCTTG

CCAGAAGAGATAGGGGATTTAGAAAACTTGGAGGAGCTTGATGCCAGCTGTACCCTAATT

TCACGACCTCCATCTTCCATCGTACGCTTAAGCAAACTTAAAATCTTTGATTTTGGAAGC

TCCAAAGATAGAGTGCTCTTTGAGTTCCCTCCGGTGGCAGAAGGATTTCGCTCATTGGAA

ACTTTGAGTCTCAGAAACTGCAATCTAATAGATGGAGGACTTCCGGAAGAGATTGCATCC

TTATCCTCTTTGAAAAAGTTGTATCTCAGTGGAAATAATTTTGAGCATTTGCCTCGAGGC

ATAGCCCAACTTGGTGCTCTTCGAATCTTGGACTTAAGAAATTGCAAGAGGCTTACACAA

TTGCCAGAATTCACGGGGATGCCAAATTTGGAGTATTTGGATCTGGAGGGATGTAGTTAT

CTTGAAGAGGTTCACCATTCCCTGGGGTGTTGCAAAAAACTCATTCGGTTAAATTTGAGT

TTTTGTAGTCGCCTTATAAGGTTTCCATGTGTTAATGTGGAATCTCTTAAATATCTGAAT

GTAGGAGAGTGCTCTCGGTTAGGGAAATTTCCAGAAATCCACGGGAGATTGAAGCCGGAG

ATACAGATTCACATGAAACGCTCTGGGATAAGGGAACTACCATCATCTATTTCTCGGTAC

CAAACTCATATTACCGAGCTAGATTTGAGAAGTATGGATAACCTTTTAGATCTTCCAAGC

AGATCGGTAGGTTGA

>NTTN90_mRNA_40198_cds mRNA_40198 gene_22483|id=AT5G17680.1:evalue=2e-171:annot='disease resistance protein (TIR-NBS-LRR class), putative';id=Solyc07g052770.2.1:evalue=0.0:annot='Tir-nbs-lrr, resistance protein'

ATGTATCTTGTAAAGCAGCAACATAACGACCAGTTATCGTCAAAAATGTCTTGCGTTTCT

TCCAGTATGTCTTCATCTTCCAAAGTCTGGAAGTATGATGTCTTTTTGAGTTTTAGAGGT

GAAGATACGCGTAAAAACTTTGTGAGTCATCTCTATAATGCTCTAGAACAGAGAGGAATC

CATGCTTTCAAAGACGATGAGCGTTTGGAAACGGGAAAATCAATTTCTGTTGAACTTTTG

AAAGCCATAGAAGAGTCCAGATTTGCTGTCGTGATATTTTCCAAGAGATATGCATCCTCA

AAATGGTGCTTAGAGGAGCTTGCCCACATCATAAAGTGCCGAAATGAATTGGACCAAATT

GTGCTTCCAATCTTTTATGATGTGAGCCCATCTGTTGTACGCCATCAAAATCCCCCGTTC

ACTAAATCATTTTCCAAGCACGAGGAAACATACAAAGATGATAAGGAGAAGGTTCAAAGA

TGGAGGGATGCATTTGCAGAGGCTGGAAAATTATCAGGTCATGATCTAAAAAATTATAAG

GATGAGGTTGAGTGCATCAATAAGGTAGTTGATTACATATTACCAAAGTCGCTTCAAGTT

ATCCCACTGTCTTCCGGAAGCTTAGTGGGTATGGAACCTCAAATTGGGAAAATAATCTCA

TTATTAGATACGGAATCAAATGATGTTCGTTCCATTGGATTATGGGGGATGAGCGGAATT

GGCAAGACAGAAATTGCAAGTGTTATATATGAGAGATATCGTCATCAATTTGAAGCTGAT

TGTTTTCTTGGCGATGTTGGAGAAATGTACCTGAAAAAGGGACTTACATGGTTACAACAA

GCTCTCATCCATAAGCTATTGGGGAAAAATATACCTATAACTAGTGAACGTGAAGGTGCC

ATAATTATAAAGAATGGGCTTCGCTGGAAGAAAGTTTTGGTCATTCTTGATGATGTAAGC

CATCTAAGTCAACTAGAGCTTATAGTTGGAGGGACAGAGTGGTTTGGTAGGGGTAGTAGA

ATTTTGATTACCACGAGGGACAAGCACATCATAATCGCTCATGTCAAGGAGGATAAAGTG

TATGAAGTCCAACTGTTATCTGAGAATGATGCACTTGAACTGTTCTATGTGCATGCTTTC

AATAGAAATTCTCCAGAGAGAGATTTTGAGGAACTTTCAAGGGAAGTGGTGAAGTATGCT

GACGGGCTCCCTTTAGCTCTTAAAGTTTTGGGTCCTTCTTTTTGTGGACGAAACAAAGAG

CAATGGAGAGATATAATTGATAGACTGAAGAAAATCCCTAATGATGACATTTTAGGAAAA

CTTAAGATTGGTCTTGATGGATTGAACAGGGATGAGATGAGGATATTTCTAGATATCGCA

AGCTTGTACAATTATAAATCAATGGATCATGTGGCACTAATACTTAAGAGTTGCGGTATT

CATCAGTCGATAGGAATAAGCCGCCTCATCGAAAAATCTCTCTTATCCTTCAGTAGATAT

GACTACACATTTAGGATGCATAGTTTGATAAGAAAAATGGGTGAAAATATGTTAAGGGAA

GAGTATGCAAACAGCAGAATATGGCTTCATGAAGAGGTTAATGACCTTTTTGCTGGAAAG

TTGAAAACAAAAAAGGTGGAAAGCCTATGGATTCCAAAAGGTTTTGATTTTGAAGATGAT

CGTGTCAATCATAGCAAGGTATTCAAGAGGATGAAAAGCTTACAGGTGTTGATACTTGGT

GAAACTGTTTGGTCAGATTTTCTTTTTCTTGTCATGGCTGAAACTATTTGCTCGCGTAGC

ATTATCACTTGTCTTCCTTCTAGCCTTCGGTGGATTGAATGGCCAAATTATCCTTCAAGA

TTATTGCCGGAGAGGTTTGAACCATCACACCTCGTTGGCCTTTGTTTAAAGGGAAGTCGG

CTTGTCGAACTTTGGCCAATATCAAAGAGATTGAGCAACTTGAAGCATTTGGATCTAAGC

AAGAGCCTTGGGTTAAGAAAAACACCTAGTTTTGGTGACATGCCAAACTTGGAGAGACTA

ATATTAGAGGGGTGTAAGAATTTGGAAGAGGTCCATTCCTCTCTTGGACATTGCAGAATG

CTTACTTCTTTGAATTTGAGGGGTTGTAGCAAACTTAAGAAGCTTCCAAAATTTGTCTCC

ATGGAATCTCTTGAGACTCTCAACCTCCGTGAATGCACAAGTTTAAGAAAATTTCCAAAA

ATCTGTGGAAATATGCAGCGCTTATCAGAACTCTATGTGGAATCCCCCTGGATAAGAAGC

TTACCCCTCATGTCTCTTAGCGGCCTGAGCAAGTTACATTTGTACTATTGTGAAGATCTT

GAAAGTATTCCAGACACTATTATTCAAAATCTTAGATATCTGGACATTTTGGGTTGCAAT

AAACTTGCAACGCTGCCAAACAGCCTCTCTGAATCAGAGCAATTGGAGCAACTTAGTATA

CACCGCTGTTCTAGATTGGTAGAGCTCCCCATATCTCTTAGAGTTCAAAGAAAGCTTGTC

CGGTTAGCCTTAGACAGATGTGAGAACTTAAAGAAGCTCCCGAAATCCATTCAGATGGAA

TCCCTTGGTTATCTCGGTATATATAATTGTCCAAGATTAGATACATTTCCAGAAATCAAT

GGAGATATGCGTTGCTTGAAACATTTGACTGTGAATTCTACAGAGATAAGAGAACTACCT

TCATCCATTGGGAATCTGAGCGGCCTCAATACTCTCAATCTGGAAGGTTGTGAAGATCTT

GCAAGTCTACCAAACAGCCTCTGTAATTTGACGAATCTTCAAAGTCTTATTCTCTACGGC

TGCAAAAAGCTAGAGAATCTTCCGGAAAACATTGGTGATCTGCAACAGTTAAGGTTACTT

GATGCAAGAGGAACTGCAATCTCCCAACCACCTCCCTCCATCATCAAGCTTGGCAAACTG

TGGAGTTTTAGATTCTCACATGTTGTACAACTTCAACATTCATCAAGTTTTGTTTTGCAT

CAACTTTCAGCTTTATCCTCCTTGACACATCTTTATCTTAATAATCTCAATATGTTGGAT

GGACTTCCTGAGGATCTTGGATCTTTGCACTTTCTGGAATATTTGAATGTAAGTGGAAGC

AATATTTCTTGTTTACCTAACAGCATCAACGAACTCTTATGCCTTGAGTGTCTGAACGTA

CAATTCTGTAAGGGTCTTACTGAACTGCCAGGAGAGCTACCCCCGAATTTACGGGTGCTA

TGTGCAGACTATCATTTAGCCTTGAAGAGCATCAGAGATCTGGTATTCAAGTGTGTTAAC

TTGGAAGAGATCTCAATATCATGGTGTGGTCATGAAATAACCGAATGCAGAACTGTCACA

AGTAACCAAGTTAATGTGTTCAAGTTCCTACATCATTTTCTTAGGAGATCTATCCAGAAT

GACTTCTTCCGCCAAAGGTGGATAAGATTTAGCATTTCATTTCCCCAAGGCAAAATTCCC

GAGTTTTTCACTTATCAGTTTATAAATCAAAACAGAATCTCAGTTAATCTGAACCCATCT

TGGTATACTGATAAATTCATGGGTTTTTCGGTATGCTACCAAGTTCATGGAGGGGAAAAA

GATTCAAAAGTTACTCCTACATTGGTCTGCAGATTATCTGGCCTAGAAACATTACTTGGC

TCGGAAGATCCCCTTTGCTTATATGAGTCGCCAAATGATAATCCTGCTCCCGGCATGCTT

TTTATCTACATACCATTTCAAATATTTCGGGACCATTTTAAGCCTTTAGGCACCAAAGCG

AAGAACCCAAATGATTATTGCCTATTTGAGGTTTCTGTAATGTCAGGGAAAGAAGGATGC

TGGGGAATTCGCTTGGAGTATGAGAATAAAGTTAGGAGATGGAGAAGGAAGCAACGTGTG

ACACAAAGTCCCAAACTCCATCCAGTTCCGCAAAAAGATAATGCAGTGACAACTGAAATT

GGTTGTTCAATGGCATTTAAACAACAAGAGCACTCATCATGCACTTCTAGTTCGCAGGCG

TTTGTTGAAAATAACATCGCAACAGAGAGGGGACTACATTTGTACTATGAGAATAAAGAT

CTGGAGGTGGATCAAGCAGCAACGGTAGTACAAAAGGAACATGAATCTCAGAATGTTGAG

CTGATTGGAGTGTGTGATAAGCCATTAAGGAAGATGGACGACGCTCCGAGAAAGAGAAAG

AAAAAGAGAAAGATGGCAGGAGAAAATGAGAACCAACGAGCTTGCATGACTTTGATTGGA

TCATGA

>NTBX_mRNA_95749_cds NTBX_mRNA_95749 gene_57210|id=AT5G36930.2

ATGGATACTCAATTAGTCAGAGGAGAATCATCTACATCTTCTCACTTCTCTTATGAAGTA

TTCCTGAGTTTTAGAGGTGAAGACACCCGAAAAACATTCACTGGTCATCTTTATTCCAAA

TTAGATAATGTTGGAGTTAAAACCTTCATCGACGATGAGGAATTGAGAAAGGGTGACGTG

ATTTCCAGAGAATTAGAGAAAGCAATTGAAGAGTCAAGAATTTCCATTATTGTTTTCTCA

AGAAACTATGCTTCCTCTAGTTGGTGTCTAAATGAACTAGTTAAAATTCTTGAATGCAAA

GAGAAATTAAAGCAGATGGTTTTGCCTATTTTCTATGATGTTGATCCTTCTGAAGTGCGA

AAGCAAACTGGGTTATTTGGTGAAGCTTTGGTTAAACATAAGGAACGATCCATTGGGGCT

CAAATGGTGGAGAAATGGAGAGCTGCACTTACTGAAGCTGCAAATTTATCTGGATGGCAT

TTGCAAAATGTTGCTGACGGGCATGAATCAAAGTTTATTGAAAAAATTATACAGCAAGTC

CTACAAGAGGTCAACCAGACACCTCTAGATGTTGCTTGGCACCCAGTTGGTGTAGATTAT

CGTGTCAAAGATGTAGAGTTGTTATTGCAAAATGAATGTGAAGATGAAGTTCGCATGATT

GGTATTCACGGAGTTGGTGGCATAGGGAAAACAACTCTGGCAAAAGCTATCTACAACCGA

ATGTTACGACTCTTCGATAATAGTTGCTTCCTTTCAGATGTTAGATCAGAAGCTGAAGAA

TTTGGTCTTGTCAAGCTACAAGAGAAACTTCTTCGACAAATTCTCAAAACTGAGGACATC

AAAGTTGGCAATGTTGCTCAAGGCATCAATCTAATCAAAGCAAGATTCGGGTCAAAGAAG

GTTTTAATTGTTCTTGATGATGTGGACCATAAAAGACAGTTAGAAGCCTTAACAAGAGAA

AGAAGTTGGTTTGGTTCGGGTAGTTTAATAATCATTACCACCCGAGACGAGCGATTACTA

TGTCGGCTTGGAGAAAAAGAGAGATATGAGGCCAAACTATTAAATGGCAATGAAGCTATG

TTACTTTTTTGTTGGCATGCTTTTGACAGTCATTTTCCACCACAAGATTATGTTAATTTG

GCACACGACATAATCGAATATTCAGGTAGGCTACCATTAGCTCTTGTGACATTGGGGTCA

CATTTACAAGGAAGTTCTATAGAAGAATGGGGATATGAATTCGAAAAACTAAGAGCAATT

CCTCATAGTGATATCCAAAAGATTCTCAAGATAAGCTTTGATGGACTTGATGATGAAACA

CAATCTGTTTTCCTCGATATTGCTTGCACCTTCCATGGGTTTGATGAGCATAAAGTTAAT

GAAATATTAAATGCATGTGGCTTTCATACTAGAAGTGCAATTGCAACTTTAGTCCAAAAA

CACTTGCTCCGAAGATCTTGGAATCATATGTTGGTGATGCATGATCTAGTGCGAGATATG

GGAAGAGAAATCGTTCGCATGGAATCACCCCCATACCCTGGCAAACGGAGTAGATTGTTC

ATCCCTCAAGAAGTCTGTGATGTTCTACAACGAAATAAAGGTTCCGAAAATGTAGAAGTA

CTGAAGGTAGATCGAGGGACATTAAAGGTAGTGAACTTGAGCACCAAAGCATTTGAACAA

ATGGAAAACCTGAGGGTTCTTATAATGGATGAGTTACATATTAGTGGAGATTTTGGGTTG

TTGTCCAAGAAGCTCAGATGGTTGTCTTGGAAAAGATGTCCTTTAAAATGTATACCATCA

AATTTTCCAGCTGAGAATCTTGCAGTTCTAGATATGCGGGAGAGTGATATCCAAGAATTT

CAATTGAATTTGCAGTGTTGTACAAGTTTGAAGAAGCTGGATCTCTCTTATTGCGAGCAA

CTCAGAAGGACTCCAAACTTCAATGGTTCACGAAGTCTTGAGACTTTGCTACTTGTTGGT

TGCTCAAGTCTGACTGTAATCCATCCATCAATAGGAAATTTGTCCAGACTAATTAAATTA

TCTATGTCCGGTTGCGAAAAACTTAGGGATCTTCCAAGCAGCATATGCCAGCTAATATCC

CTTGAAGAATTGTTCATTCATCACTGCTCCTCTATAAAAACACTGCCAAATAACCTTGGA

GATATGAAAAGTTTAAGATATTTTTCTGCATCTTCTACGGGTATAAAACAAATGCCTAGA

TCTCTTGAAATGCTAAGAAATCTTGTAACTTTGAGAGTGGGAGGTCAAGAGCTAGAGGCC

AAAAGGAGTATTTTTGGAAGAGGTGTCCATCAGATACAATATTCCTTGCTAACTTTTGTA

ACCGAATTGAGCCTTAGATACTGGAATTTGTCCGATTCTGATATTCCTAGGGATATTGGG

AGCTTATCCTCCTTAAAGTTTTTAGATTTGAGTGGCAACAGTTTCCATTGTCTACCCTTC

GATTTTTCTAAGTTACGAGTATTGAAGAAGTTGTGTTTGAATGACTGTGAGAATCTTCAA

ACACTCCCGTCAGTATCAAATTTAGAGAATCTTGGGCATCTTGAACTTCAAAATTGCCAA

AAATTGGTCAAGATTACACAGTTGGACAACCTCCCTTCTATACGGTTGATTAACATGAGT

AATTGTAGTTCTCTGCAGAATCCATTCAATGAAGGCTTCTTTAGTGCACCTGCTCTATCA

TTTCCATTTAGAGAAGATCGAGATATGGGTTATTTAGAAATTTATCTGGAATGCAGTGAG

ATTCCAGAATGGTGCAGCAATCAAGTAAGAGCTTCATCTATCTGTTTGACTGTGCAGACA

CATAATAATGAGGAGTATAACTTCTTAGGAATGGTTCTCTGGTTTGTTGTCGACGCTTTG

GATGTAGCCCTTTTTCCAACCTTCAGGATTAGTATTGCCCATAAAGTGCCTTCAGGTATT

CCGTGGAGTAATATTGGAATACTTGATGGACACAGAGAACTGACATGTGTATATTACATA

TCTATCTTAAATGAACTTTATTATGGCCAGATGATTAAAGGCGGGGAAAGGATAGAAGTG

TGGTCTGAAGACATTACCCTAAAGAAGATAGGGATGGATCTGTTATATTTAGACCAAAAT

GGTAAAGTTATATCTTTGCCGGGAGACGTGGATCATTCTTATTCTAGGGCGAAAGATGTC

AGGAATTGGCGGGAAGAGATTCTTAGTTGGTCAGATAATTTTAATTGA

>NTTN90_mRNA_18117_cds mRNA_18117 gene_10151|id=AT5G36930.2:evalue=2e-150:annot='Disease resistance protein (TIR-NBS-LRR class) family';id=Solyc01g113620.1.1:evalue=0.0:annot='NBS-LRR resistance protein (Fragment)'

ATGGCCACTGAACTGAAGTCTCAAGTGTACTTGAGTTTCAAAGCGAAAGACACCGGCAAA

ATTTTTGCAGATCACCTCTATGAAGCTCTGGTGGGAGCAGGTTTTGTAACATTAAGAAGC

TGTGGTGATGAAAATGAGGGAGGTGAAGATATCAAGTTCAATTTGCAAAAGGGTATTAAA

GAATCTGGGGTTTCAGTTATAATCTTCTCAAATGATTACGTGTCCTCAAGTTGGTGTCTT

GATGAGTTGGTAATGATCTTGGATTGTAAAAAGATAGCAAAACGTGCAGTTCTGCCCATA

TTTTACCACGTGGATCCTTCTGATGTTAGGAAACAGAAGGGAAGAATTGGAGAAGCATTT

GATATGGACAAAGAACTGGGAGGGAATCAAGGTGAAAATGAGAGGGTCAGAAAATGGAGG

GAAGCACTCAAAGAAGTTGCAGACTTGGGAGGAATGGTCTTACAAAACCAAGCTGATGGA

CACGAGTCCAAATTCATCCAGAAGATTCTTAAAGTGGTTGAGAATAAACTGAGCAGGCCA

GTCCTGTATATTTGCCCTCATCTGATTGGAATAGAACGGCGTGTTGAAAAGATCAACTTG

TGGCTAGAGGATGGATCTATTGATGTTGACACTCTTGTTATTTGTGGCATCGGTGGAATA

GGCAAGACAACAATGGCAAAGTTTGTGTATAATTTGAACTTCAGTAAGTTTGATGGTAGC

AGCTTTTTGTCCAACATTAGAGAAAATTCAACACACCGTAAAGGTTTAGTTACTCTTCAA

AGGCAATTTCTTTCTGATATTTGCAAAAGAAAGAAGAAAGCTATGTTTTCCGTGGACGAG

GGAATGACTGAGATGAGAGAGGCTGTACAGTGTAAAAGAATCCTTCTTGTTCTTGATGAT

GTAGATAATCGTGATCAAGTGGATGCTCTACTGGGAATGAAGGACTTGTTATATCCTGGT

AGTAAAGTCATTGTGACAACTAGGAACAAGAGATTGCTTAGGCCTTTTGATGTGCATAAG

ATTTATGAGTTTGAAGCATTGAATAGAGATGAATCGGTTGAGCTCTTAAGTTGGCATGCA

TTTGGTCAAGATTGTCCTATTAAAGGTTTTGAAATGTGTTCAGAACAAGTAGCAATCCAT

TGTGGAGGACTTCCATTAGCACTTGAAGTTCTTGGTGCTACTTTGGCAGGAAGAAACATA

GACATTTGGAAAAGTACAATACAGAAATTGGAAACAATTCCGAATCATCAAATTCTCAGG

AAATTAACAATAAGTTACGAATCTCTTGAGGATGATCATGATAAGAATTTATTTCTCCAC

CTAGCTTGCTTTTTCATTGGGAAGGACAGAGATCTAGCAGTAGCTATTCTCAATAGGTGC

AACTTTTACACTGTAATTGGAATTGAGAATCTCATTGACAGAAATTTTATAAAAGTTGGT

AAGTCTAACAGCTTGATTATGCATCAAATGATTCGAGATATGGGAAGAGACATTGTTCGC

CAAGAATCACCACTGGAGCCTGGGAAACGCTCTAGACTATGGCGTTCAAAGGATTCCTTT

AACGTCTTAATCCAGAACCGTGCCACTCAAACAATTCAAGGCATTATTCTTGACATGGAT

ATGCTCAAGGAAAGTGACATAGTTAGCTCAAGCTTTTTCGCCAAGGATTTCAAGAAACAC

AAAATAAAAAACTTTCTCAACTATCCTAATCCTCAGAGAGTTCAATTCAAACAGAAAAGG

TTTGTTTTTTTCCCATGGCATTTGTCAGATGCCAAAGAAGCCACAAATGAGCTGGTTCTG

GGAACTGATGTATTTGCAAATATGCAAAAGTTAAAACTGCTCCAATTCGATCACGTTGAG

CTTCAAGGATCTTTTGATGTTTTTCCTAAGAGATTAAGATGGTTGCGCTGGTCTGAGCTG

CAACTTGAGTGCATGCCAATTGATTTTCCTCTGGAGAGCCTTGTAGTGATTGAATTACAC

CGTAGCAGCTTGAGGAGGATTTGGCATGGAGTCAAGTTCCTTAAAGATCTGAAGATTTTC

GATCTCAGCCATTCCTACGAGCTTCTAAGAACACCTGATTTTTCAGGACTCCCCAATCTT

GAAAAGTTGATCCTTCGATATTGTACAAGCTTGATTGAGCTTCATGAGACCATCGGGTGT

CTAGAATCACTTATTCTTTTGAATCTCAAAAATTGCAAAAATCTCCAGAGACTTCCAGAT

AGCATTTGCATGCTAAAATTTCACCAAATGATTTCTACTCCAGAAGAGGTCCAACCGTGG

TATGGATTTCTGCGGTCCTGGATGCTGAAGGGGAAAATATGTCCTAAAGTTTCACATATT

AGTTTACCTAATTCCTTGGTTACTCTGAGTCTTGCTAACTGTAATCTATCCAATGATGCT

TTTCCAGTTGCTTTCAGTAGCCTCTCCTTATTGCAAAACTTAGATTTGAGCGAAAATCCA

ATTTGCTGCCTACCAAAGGGCATAATTTATCTCACCGGTCTTCAGAAGCTTGAAGTGGAA

GGCTGTGAAAAGCTCAGATCGCTCGTAGGGCTTCCCAATGTAGAACATCTCAATGTTACT

AATTGCTGGTCGTTAGAGAAAATATCATATCAATCAAGATCATCTAGACTGAAGGATTTA

CTTGTGTCGAATTGTGCTAAATTAGTTGAAATAGATGGAAATTTCAAGTTAGAGCCCTTA

AGAAATACTGAGGCAGAGATGCTTTGCAAGTTGGGCTTGTCGAACTTAGCTTCTATGGAT

AATGTCATGATCAATCTTACATCTAATATCCTGAGTTACTACCGAATACATGGTAAAGGA

TGGACTCCAACAAGGAAGACAAAGAAAGTTGTTCTTCAGGTACTGTACCAACCAGGTGTC

TTTAGCACTTTTCTGCCAGGTGAACATGTACCTTCTTGGTTCAGCTCAAAATACACAAAA

GAATCACATACATCCTTCAAAGTGCCTACTTGTACTTCCACGATTGAAGGCTTGAGTTTT

TGCATTGTGTACAAGCGTTCCGTATTTGGTCTAAGTGCTCATCGTCCCCCGCGCCTAACT

CCGCCTTCAAGAATAGCTCCTCTTGCCATGCACAAAGCTCAAAGAGGACCCATTCGGTAT

CGGCCAGTGGAAAATAAACCATATGAATCAACCTTTGACTGCCCGTGCATTACTGTTAAT

AACTTAACTCGGAGTGTGAAATGGTCTTACCAGCCCTTGTTCTATGGAGTTCCGGAAGGG

AAAGAAGGAATGATGTGGTTAAGCCATTGGAAACTTGAGAATCAGTTGGGCAGTGATGAT

ATACTGGAGATCACAGTTACCTCAGGAGATGGAATCAGAATTGTGGAGTTTGGGCTCAAA

ATTCTGCATGTTGAAGGGCCAAATGTGCAAATAGGAGAACCAAGTTGTGAAGATGCAAGG

GGAGAGAAAGATATTGTCAATCCATTTTGGGATGTTGTTTTAAAAGATGCTAGTTCAAAG

AATACTTGTTCTGTTCGGCTTCCTCCTACATATCGTCCCCTACGTGTTGCTCGTGAGCCA

TTTCTGGAGAAGGCGCTGAAAAGAAATATGTCAGACTATAACTAG

>NTBX_mRNA_99245_cds NTBX_mRNA_99245 gene_59605|id=AT5G17680.1

ATGGCATCTTCTTCTTCTTCTTCTGCTAGATGGAGCTATGATGTTTTCCTAAGTTTTAGA

GGTGAAGATACTCGGAAAACGTTTACAAGTCACTTATACGAAGTCTTGAAGGATAGGGGA

ATAAAAACCTTTCAAGATGAAAAAAGGCTAGAGTACGGTGCAACCATCCCAGAAGAACTC

TGTAAAGCTATAGAAGAGTCTCAATTTGCCATCGTCGTTTTCTCGGAGAATTATGCGACA

TCGAGGTGGTGTTTGAATGAACTAGTGAAGATCATGGAATGCAAGACTCAATTTAGACAA

ACTATTATACCGATATTCTATGATGTGGATCCATCACATGTTCGGAACCAAAAGGAGAGC

TTTGCAAAAGCCTTTGAAGAACATGAAACAAAGTATAAGGATGATGTTGAGGGAATACAA

AGATGGAGGACTGCTTTAAATGCCGCAGCCAATCTCAAAGGCTCATGTGAAAATCGTGAC

AAGACTGATGCAGACTGTATTCGGCAAATTGTTGACCAAATCTCGTCCAAATTATCCAAG

ATTTCTTTATCTTATTTGCAAAACATTGTTGGAATAGATACTCATTTAGAGGAAATAGAA

TCCTTACTAGGGATAGGAATCAATGATGTTCGGATTGTGGGGATCTGGGGCATGGGGGGA

GTGGGTAAAACGACAATAGCTAGAGCTATGTTCGATACTCTTTTAGGAAGAAGGGATAGT

TCCTATCAATTTGATGGTGCTTGTTTCCTTAAGGATATTAAAGAAAACAAACGTGGAATG

CATTCTCTTCAAAATACCCTTCTCTTTGAACTTTTAAGGGAAAATGCTAATTACAATAAT

GAGGACGATGGAAAGCACCAAATGGCTAGTAGACTTCGTTCTAAGAAGGTCCTAATTGTG

CTTGATGACATAGATGATAAAGATCATTATTTGGAGTATTTAGCAGGTGATCTTGATTGG

TTTGGTAATGGCAGTAGAATTATTGTAACAACTAGAGACAAGCATTTGATTGGGAAGAAT

GATATAATATATGAAGTGACTGCACTACCTGATCATGAAGCCATTCAATTGTTCTATCAA

CATGCTTTCAAAAAAGAGGTTCCAGATGAGTGTTTTAAGGAGCTTTCATTGGAGGTAGTA

AATCATGCTAAAGGCCTTCCTTTAGCCCTCAAAGTGTGGGGTTCTTCCTTACATAAGAGG

GATATAACTGTGTGGAAAAGTGCTATAGAGCAAATGAAAATTAATCCTAATTCAAAAATT

GTTGAAAAGCTGAAAATTAGTTATGATGGATTAGAGTCCATGCAACAAGAGATGTTCCTA

GATATAGCATGCTTCTTCCGAGGGAGACAAAAAGATTACATCATGCAAGTTCTCAAGAGT

TGTCATTTTGGAGCTGAATATGGATTGGATGTCTTAATTGAAAAATCTCTTGTGTTCATC

TCTGAATATAATCAGGTTGAAATGCATGACTTAATACAGGATATGGGTAAATATATAGTG

AATTTTAAAAAAGATCCTGGAGAACGTAGCAGACTATGGCTCGCCGAGGATGTCGAAGAA

GTGATGAACAACAATGCAGGGACCATGTCAGTGGAAGTAATTTGGGTTCATTATGATTTT

GGCCTATACTTTAGCAATGATGCCATGAAAAATATGAAAAGGCTTAGGATATTACACATA

AAGAGGTATCTGTCCAGTACTAGCCATGATGGTTCCATTGAGTATCTGCCCAACAACTTG

CGTTGGTTTGTCTTGGATGACTATCCTTGGGAGTCATTGCCATCTACATTTGATCTCAAA

ATGCTTGTTCACCTTGAACTCTCGCGTAGTTCACTGCATTATTTATGGATGGAAACAAAG

CATTTGCCGTCTCTACGGACGATAAATCTCACGGGCTCTGAAAGCCTGATGCGAACACCA

GATTTCACGGGGATGCCAAATTTGGAGTATTTGGATCTGTCTTTTTGTTTTAATCTTGAA

GAGGTTCACCATTCCTTGGGATGTTGCAGCAAACTCATTCGGTTAGATTTGAGGGGGTGT

CAAAGCCTTGAGAGGTTTCCATGTGTTAACGTGGAATCTCTTCAGCATTTGGATTTACCA

GGTTGCTCAAGTTTAGAGAAATTTCCAGAATTCCGCGGGAGAATGAAGCTGGAGACACCG

ATTCACATGAGATCTGGGATAAGTGAACTACCATCATCTAGTTTTCACTACCAGACTCGT

ATTACCTGCCTAGATTTGAGCGATATGGAAAACCTTGTAGTTCTTCCAAGCAGCATCTGT

AGGCTGAAAAGTTTGGTTCAATTATATGTGTCTGATTGCTCAAGCCTGGAAAGTTTGCCA

GAAGAGATAGGGGATTTAGACAACTTGGAGGTGCTTTATGCCAGTGATACTCTAATTTCA

CGACCTCCGTCTTCCATCGTACGCTTGAACAAACTTAACAGCTTGAGCTTTAGGTGCTCC

GGAGACATCGAAGTGCACTTTGAGTTCCCTCCAGTGGCTGAAGGATTATGGTCATTGAAA

GATCTGGATCTCAGTTACTGCAATCTAATAGATGGAGGACTTCCGGAAGACATTGGATCC

TTATCCTCTTTAAAATATTTGAATCTCAATGGAAATAATTTTGAGCATTTGCCTCGAAGC

ATAGCCCAACTTGGTGCTCTTCGATCCTTAGACTTATCATATTGCGAGAGGCTTACACAG

CTACCAGAAATTCCCCCTGAATTAAATGAATTGCATGTAGATTGTCATATGGCTATGAAA

TTTATCCATGATTTAGTAACAAAGAGAAAGAAACTACAGAGGGTGAAACTTGATGATGCA

CGCAATGATTCTATATATAATTTGTTTACTCATGTCCTGTTTCAGAATATCTCTTCCTTA

AGGCATGACATCTCTGCTTCAGATTCCTTGTCCGAAAGTGTGTTTACCATTGTGCATCCT

TATAATAAGATCCCAAATTGGTTCCTCCAACAAGGAAGGGATAGAAGTGTATCAGTCAAT

TTGCCTGAAAATTGGTACATACCTGATAAATTCTTGGGATTTGCTCTATGTTACGATGGC

ATCTTAATTGACACCATAGCTCAATTGATTCCCGTATGTGATGACGGGATGTCGTGGATG

ACCCGGAAACTTGCTTTATCCAACCATTCAGAATGTGATACAGAATCATCCGACAATTCA

GTAAAGGATATACATTTCTTCGCTACATTTTTTGATATTCATTTTTTGTTGGTACCTCTT

GCTGTCTTATGGGATACATCTAAGGCAAATGGAAAAACACCAAATGACTATGGAGTTATT

AGGCTATCTTTTTCTGGAGAAATGAAGGAGTATGGACTTCGTTTGTTGTATAAAGAAGAA

GCTGAGGCTGAGGCCTTGTCACAAATGAGGGAAAATAACAATGAACCAACAGATCATTCC

ACTGGCATAAGGAGGACCCGATATAACAATAGTGAACATCATTACTCGGTGACCAATGAA

GCCAGTTCCTCCTCCTCTTCTAAGAAACAACGGTCACATTTCTAA

>NTTN90_mRNA_37128_cds mRNA_37128 gene_20767|id=AT1G27170.1:evalue=0.0:annot='transmembrane receptors';id=Solyc05g006620.2.1:evalue=0.0:annot='Tir-nbs-lrr, resistance protein'

ATGGAGGAAATTCAGACCACAACTTCACTCCCATCGCTAAGGCTGAATTACGACGTGTTC

TTGAGTTTTAGAGGCGAAGATACTCGCGAAAACATCACTAAAAACTTATACGATGCCTTA

TACTCAAAAGGCGTCCGAGTGTTTCGAGACACAAACGGGTTAACTCAGGGCGACGAGATC

GCACCAGGTCTTATGGACGCAATCAACGATTCAGCTGCAGCTATTGCTATTATTTCACCC

AATTATGCTTCGTCGAGATGGTGTCTAGAGGAATTAGCAACGATTTGTGAGTTGGGTAAA

CTCGTTCTGCCCGTGTTCTACCGGGTTGACCCGTCGGATGTTCGAAGGCAGAGAGGACCG

TTTCTACATGATTTTGAGAGTTTGGAAGGAAGATTTGGAGTGGAAAAGGTGGTGAGATGG

AGAAATGCTATGGAAAGAGTTGGGGGAATCTCCGGCTGGGTTTATTATAATAGTGAAGAG

TCACAGTTGATACAGACTTTGGTGAAAAGAGTTTTACAAGAATTGAGCAATTCCCCAATA

TTTGTAGCTCCATTTGTTGTTGGAATTGACTACCGTCTGGAAGAACTCATAAGACAGTTA

GATGTGAAGCGCAGTGGTGTCAAGATCATTGGGTTGCATGGAATAGGAGGAGTTGGTAAA

ACAACTCTTTCTAAGGCTCTTTATAATAAACTTGCTTCTCATTTTACACACAGGGCTTTT

ATCTTGAATGTTAAGGAAATAGCTGCTCAACAAGGCATTGTGTCCGTTCAGAAGAAAATA

ATACAAGGTCTTTTCCCGAGCAAGGTCTTCTCCTTCTCCCCTGGTAATGCACATGAAAGA

AGAGTAAAATTCGGACGATTTCTTCAAGAAAAGCGTGTCCTGCTCGTCTTAGATGATGTA

GATTATGTAAATGATGATGTAAGCATATTGAAGGCACTAATTGGAGGGAAAAACTGGTTC

TTTGAAGGGAGCAGGGTTGTTATTAGTACTAGAAACAGAGGAATTTTGCTAGAAGACATC

GTTAACGAGACATTTGAGGTGAGAGAATTGGGTGGTCCTGACTCACTAAAACTATTCAGT

TACCATGCATTTAGAAGACAGGAGCCATTTCCAGCTTTTGTGAATATGTCCAAGCAAATT

GTCTCAATCACTGGAGGGCTACCCTTGGCTCTTGAAGTTTTTGGTTCTTTCTTGTTTGAT

AAAAGAAGCGAGGAGGAATGGCTAGATGCTCTAGAAAAGCTAAAACAAATTCGCTCTCCA

CATCTTCAGGAAATCTTGAAAATAAGTTATGATGGTCTTGATGATGAAGAGAAGTGTATA

TTCCTGGATGTTGCATGTTTATTTCTTGATCAATTAGAAAAGAAAGCTGAAGATGTAATT

GATGTGATGAAAGGATGTGGTTTTAGAGCCAGCATTGCATTTGACACTTTAACTGCTAGA

TCATTGATTAAGGTAATTGATGGTGGGGATTTGTGGATGCATGACCAGATAAGAGATATG

GGAAGACAAATTGTTATACAACAAGGCATTTCAGATCCCGGAAAGCGCAGCAGACTTTGG

GATGTTGCTGATGTTTTGAGTGTGTTACAAGGAAGGAAGGGGACACAGAACATCCAAGGG

ATCATCCTGGATCAGTATCAGAAGCCATCATCAAAGATTAAAAGCACGAAAGCAATTACT

AGAGAGCATTTTCAACAAGTTCCCACTTTTACTTCTGCATTAGCTTACATTAAAGAGTTG

TGCAAAGAACAATTTCAAAATGATGCAAAAGAAACCAATGATTTGGTATTGAACACTGAA

GCATTTGATCCAATAGTTAATCTGCGGCTACTCCAATTCGATAATGTGAAACTAGAGGGA

AATTTGGGGAAGTTACCTTCTTCACTAAAATGGCTCCAATGGAAAAGGTGCACACTTTCA

AGCTTTTATTCTGATTATTATCCAAGTGAACTTACCATGCTTGATCTCTCAGAGAGCCAA

ATAGAGAAGTTTGGAAGCCGGGAATGGACTTGGACTCGCAAAAAGGTGGAAAACAAGTTG

ATAGTTATGAATCTCTCTGGTTGTCATAAAATAACAGCTATTCCTGATTTATCCACGCAT

AAAGCATTGGAAAAGTTGATAGCTGAACGTTGCAGTGCATTGCAAAGGATTCACAGAACA

ATTGGGAATCTGAAAACTTTACGTCATTTAAATTTAAGAGATTGCCGCAACCTTGTTGAA

TTTCCAGGTGAAGTCTCCGGGCTGAAAAATCTTCAAAAGCTGATACTCTCGGGCTGCTCG

AGATTGAAACAGTTACCTGAAGATATAGGCAAGATGAAGTCCTTACAAGAACTTCTATTA

GATGGGACTGCTATAGAGAAGTTGCCTGAAAGTATATTTCGCTTAACAAAACTTGAGAAG

TTAAGCTTAAGCCAGTGCCACTCACTGAAACAACTTTCCCGGTTCATAGGAAAGCTAAGT

TCTTTGAAGGAACTCTCTCTTAATGGTTCTGCTTTGGAAGAAATACCTGATTCTATTGAA

CATTTGCAGAACCTTCATACATTAAACTTAATTAGGTGTGAGTCACTTGCTGCTATTCCC

AATTCTTTTGGCAACCTCAAATCTTTAGCAAATCTCTGGCTTTATGGCAGTGCAATAAAA

ATGATGCCAGAATCTATTGGTTCTCTGTATTATCTTAGGTCCTTATCGCTCGGAAACAGT

CAGCATTTAAATGCATTGCCTGTTTCAATTAAAGGATTGTCTTCTTTGGTTGAGCTTCAA

ATAGACAAGGTTCCAATTATTAGTCTTCCAGATCATGTTTTTGGTGGACTTAAATCACTG

AAGAATCTTGAGATAAGGAACTGTGAGCGCCTTGGCTCGCTTCCCCACTCCATTGGAGAA

TTGTTAGCTCTTAGAACAATGACTCTTACCAGAAATGATGCTATTACGGAGCTGCCAGAA

TCAGTTGGGAATTTGCAGAATCTTGTCATATTGAGATTGACCAGATGTAAGCGACTTTGC

AAATTGCCAGCTTCAATTGGGGAACTAAAGAACTTAGTACACCTGCTAATGGAGGAGACT

TCAGTAACAAAATTACCTGAAACATTTGGGATGCTATCGAGCTTAATAATTCTGAAGATG

GGAAAGAAGCCTTTCTGCCAGGTATCACAAAGTACTGAAAACACAGAAGCAGCTACCTAC

ACAGAAAGGGAAACATCACCTGTTGTGCTTCCTTCATCTTTCTCAGAGCTATCCATGTTA

GAAGAACTTGATGCCCGCGCGTGGGGAATAGTTGGGAAAATACCGGATGATTTTGAGAAA

CTATCATCTTTGGAGATCATCAATCTTGGTTTCAATGATTTTTCCTATCTCCCGTCTAGT

CTGAAAGGACTACTTTTCTTGAAAGAGCTCCTTGTTCCCCACTGCAAACAGTTGAAAGCT

ATTCCTCCTCTTCCCTCAAGTTTGCTCAAGATAAATGCTGCAAACTGTGGAGCACTCGAG

AGCATACACGATATCTCAAAATTAGAGTTCTTGCACGAGCTAAACCTTGCAAATTGCATG

AGTTTGGTAGATATCCAAGGTATCGAATGCTTGAAATCCTTAAGAATGCTACATATGGCT

GGATGCAATGTCTCCTGTGCCTTTATGGTTAGAAGCAAACTTGATAAGGTACTGTTTTCT

CCTATTTAA

>NTTN90_mRNA_93148_cds mRNA_93148 gene_52671|id=AT5G36930.1:evalue=7e-156:annot='Disease resistance protein (TIR-NBS-LRR class) family';id=Solyc01g113620.1.1:evalue=0.0:annot='NBS-LRR resistance protein (Fragment)'

ATGGAGAAACCTCTGGATCCAACACCTTCGTCTTCATCTTATGGGTGTTCCTCAAATCAA

GTATTCTTGAGTTTTAGAGCAGAAGACTCTTGTTGCATGTCTTTCACTGATCACCTTTAC

ACTGCCTTAGTTCAAGCTGGGTTTCAAACATTCAAACAGGGCACTGACACAAGAAAAGAA

CAACTCCACAACGCAATCCGAGAGTCGAAAGTTTCGCTGATTGTCCTGTCCGAAGGCTAT

GCCTTTTCTCAATCATGTCTTGATCAGCTCGATGTGATTCTGACATGTAAGGAGAAATTG

GATCGGGCAATTCTACCCGTCTTCTACTATGTGGATCCTTCTGATGTTAGGAACAAGAAG

GGAAGGATTGGGGAAGCATTAGCATTGCATGAACAAGAATTGAAATGGGAAAGCAGTGGA

AGGGAAAGGGTAGAAAGATGGAGGCAGGCACTTGCTAAAGTTGCTGACTTGGGGGGAATG

GTCTTACACAATCAAGCTCGTGGGCATGAGTCGAAATTTATTCGAAAGATTGTTAATGTG

GTCACAAATAGACTAAGTCGGACAGCTTTGTATGTTGCACCTTACCTAATCGGCATAGAT

CGCCGGGCTAAGCATATTAGCTTTTGGCTGCAAAATGGATCGGCTGACGTTGGCATATTG

ATTGTTTGTGGCATGGGCGGTATAGGGAAGACTACTCTGGCCAAGTTCATCTACAATTCA

AACTTTCATGCTTTTGAAGGTAGCAGCTTTGTGCTTAACATAAGAGAAATTTCAAAGCAA

CCTAACGGTTTAGTTAAATTGCAAAAGCAAATACTTTCTGACATTCTAAAAAGGACAAAG

GAACGAGTATCATGTGTTGATGAAGGAATTGTTAAGATTTCAGATGCCTTAAGTGGCAAA

AGAGTCCTACTTGTTCTTGATGATGTGGATGATTCTGATCAACTAAATGCAGTATTGGGG

ATGAAAAGTTTATTTTACCCAGGAAGTAAAATCATCATAACAACCCGACATGAGCGAATT

CTACATCCTCACCTAGTTGATAAGGTGTATACCGTTGAGACACTGAGCACGGACGAATCC

TTAGAGCTCTTCAGTTGGCATGCCTTTGGAAAACCACATCCTGCGGAAGGTTTTCTTGTG

GGTTCAAATGAAGTAGTGAAACGGTGTGGAGGAATTCCACTTGCACTAAGAGTTTTGGGT

TCTTCCCTGGCAGGACAAAATTTAGATGTATGGCAAAGTACAATAAAGAAGTTGCAAGTT

GTTCCTAATAATCGGATTATTGAACTGCTCAAAATCAGTTATGAATCTCTAGAAGATGAT

GATAGGAGTTTATTCCTTCATATTGCATGTTTCTTTCTCTGGGAGGATAAAGATTTTGCT

GTCAAAATACTGGATAAATGTGAACTTTTCACTATAGTGGGGATCCAAAACCTCATTGAT

AGAGATCTCTTGTCAATACTAAATGGTAGGCTCTTCATGCATCAACTGATTCAAGATCTG

GGGAGAGAAATTGTTCGTCAAGAATCTGTTAAGGAGCCAGGGAGGCGTAGTAGACTGTGG

CGTCATGAAGAATCTTTGTACGTATTGAGAAACAAAACGGGAACTGAAGCAATTGAAGGC

ATCATTCTCGACGGAAATATGTGTAAGGGGCATGGATCAACCAGGATAACATCTAATGAA

AATTATGGCAAAAAAAGTAAAGTGGAAGAATTTATGAATAATTCTCAGGAAGATCGACCA

AAGCAGAATTGGATGTCCATTTTCTCTCGTCATATCATGGGCACAAGGGAAGTTCCAAAT

GAGGATTTGGAAACTGACTCATTCACAAACATGCTCAAGTTGAAATTTCTGTTGCTTAGC

AATATACAACTTTCTGGATGTTACAGGAAATTTCCCAAGAAATTAAGATGGTTGTTTTGG

CGTTATCTCCAGTTAGAATCCCTACCAAGCGACTTTCCGATGGGAAAACTTGTTGCTATA

GACCTTTGTTACAGCAGCTTGAAACAACTTTGGACAGCACCAAAGTTACTCAGATGGTTA

AAGTTTCTCAATCTCAGCCACTCTTATCAGCTTAGTAGAACTCCTGATTTTTCATTACTT

CCCAACCTTGAACAATTAATCCTCGAATATTGTACAAGTCTAACTGAGGTGGATGACACT

ATTGGATATCTGGAAGGACTCACTGTTTTAAGCCTTAATGGTTGTATAAACTTGAGGAGC

ATTTCAGAAAGTATTTGCATGTTAACACATCTTGAGACCCTTGATATTTCTGGTTGCTCA

AATCTTGAATATGTCGCCCTGAAGCTTGAAAAGTCGGATTTTCCAAGTGAGCTTTCAGAT

GAAAGTGGAAGAAACCAAATAGATAATACCAAGCTAGTAAGACCATGGCATACAATCTTG

TGGTCTTTGCTGAGGAAGGAGAAAGTATGTCATAGAGTTTCACCAATCAGTTTTCCGACT

TCTCTAGTTACTCTAAGACTTTCTGACTGCAATCTGGGTGATAATGCATTTCTCCATGTT

GATTTTAGTAAGCTCAATTTGCTGAAAGAATTGAGTTTGAGCCGAAATCCACTTTGCCAT

CCCCCAGAGAGCATTAGATATCTTAGCAGGCTCGAAAACCTTTCACTAAATTCATGTACA

AGGCTAAAATCAGTACTCGAGCTGCCAAATGGTGTTGAGATTGTTGATGCAACTGACTGT

ATATCCTTGGAGAAAGTATCAGGCGCACCTAGCTCATGCAGCATTCTTTACATAAATTGT

GCTAATCTGGTTGAGATGAACGCTAATTTCAAGTTAGAACATCTTGAAAATGTCAATGCA

GAAACTCTCGGCTATTTGGGTCTGTCAAACTTGGAGTTGATTAGAAATGTTACTTTTAGA

TTAAGATTTGACATTCAGAAATTGCATGCTGATGAAATGGAATTTCCGGAATTTGTCCAA

AATGACATGGCAAAATTACAATCTCTCCCACCTAAGAAGCTTCCCGCTCAGGGATATTAC

TGCAATGGCGTCTTCTCCACATTCCTATCGGGTGAACACGTGCCAAGTTGCTTTGACACG

AAGTTGAGTGAGCCTTTCTGTTCATCGTTCATCGTGCCTACTCCTGATAATCATAGAATT

CGAGGCTTGAGTTTTTGCTTGGTGTATACATGCTTGGAGAGTGAAGAAATGGTAAGTGAG

GGCCACTGCATGTCCATTACAATTAATAATTTGTCGCAAAGGATCAAGTGGAAGCAAGAC

CCTATGTTTCTAGCTATTCCTGAAGTTGAAGAGCGGATGATGTGGTTAAGTTATTGGGAA

ATTGGCAACTCGTTGCAAACAGGTGATGTTGTAGAGATTTCAGCCAGTGTTGTAGATAAA

CAACGTTTCAGTATCAACGAGGTTGGAATGAGGATTCTGTTCCTGGAAGAACAACAAGAC

CAGGACAAAGAATCTAATTGTGAAGTTGAAGAGTTTTTTTCTAGTCCATGTCATCAGAAT

TTGCTCCTTGTCAGAGTCTTCCCGAAGTGA

>NTTN90_mRNA_93149_cds mRNA_93149 gene_52671|id=AT5G36930.2:evalue=3e-123:annot='Disease resistance protein (TIR-NBS-LRR class) family';id=Solyc01g113620.1.1:evalue=0.0:annot='NBS-LRR resistance protein (Fragment)'

ATGGAGAAACCTCTGGATCCAACACCTTCGTCTTCATCTTATGGGTGTTCCTCAAATCAA

GTATTCTTGAGTTTTAGAGCAGAAGACTCTTGTTGCATGTCTTTCACTGATCACCTTTAC

ACTGCCTTAGTTCAAGCTGGGTTTCAAACATTCAAACAGGGCACTGACACAAGAAAAGAA

CAACTCCACAACGCAATCCGAGAGTCGAAAGTTTCGCTGATTGTCCTGTCCGAAGGCTAT

GCCTTTTCTCAATCATGTCTTGATCAGCTCGATGTGATTCTGACATGTAAGGAGAAATTG

GATCGGGCAATTCTACCCGTCTTCTACTATGTGGATCCTTCTGATGTTAGGAACAAGAAG

GGAAGGATTGGGGAAGCATTAGCATTGCATGAACAAGAATTGAAATGGGAAAGCAGTGGA

AGGGAAAGGGTAGAAAGATGGAGGCAGGCACTTGCTAAAGTTGCTGACTTGGGGGGAATG

GTCTTACACAATCAAGCTCGTGGGCATGAGTCGAAATTTATTCGAAAGATTGTTAATGTG

GTCACAAATAGACTAAGTCGGACAGCTTTGTATGTTGCACCTTACCTAATCGGCATAGAT

CGCCGGGCTAAGCATATTAGCTTTTGGCTGCAAAATGGATCGGCTGACGTTGGCATATTG

ATTGTTTGTGGCATGGGCGGTATAGGGAAGACTACTCTGGCCAAGTTCATCTACAATTCA

AACTTTCATGCTTTTGAAGGTAGCAGCTTTGTGCTTAACATAAGAGAAATTTCAAAGCAA

CCTAACGGTTTAGTTAAATTGCAAAAGCAAATACTTTCTGACATTCTAAAAAGGACAAAG

GAACGAGTATCATGTGTTGATGAAGGAATTGTTAAGATTTCAGATGCCTTAAGTGGCAAA

AGAGTCCTACTTGTTCTTGATGATGTGGATGATTCTGATCAACTAAATGCAGTATTGGGG

ATGAAAAGTTTATTTTACCCAGGAAGTAAAATCATCATAACAACCCGACATGAGCGAATT

CTACATCCTCACCTAGTTGATAAGGTGTATACCGTTGAGACACTGAGCACGGACGAATCC

TTAGAGCTCTTCAGTTGGCATGCCTTTGGAAAACCACATCCTGCGGAAGGTTTTCTTGTG

GGTTCAAATGAAGTAGTGAAACGGTGTGGAGGAATTCCACTTGCACTAAGAGTTTTGGGT

TCTTCCCTGGCAGGACAAAATTTAGATGTATGGCAAAGTACAATAAAGAAGTTGCAAGTT

GTTCCTAATAATCGGATTATTGAACTGCTCAAAATCAGTTATGAATCTCTAGAAGATGAT

GATAGGAGTTTATTCCTTCATATTGCATGTTTCTTTCTCTGGGAGGATAAAGATTTTGCT

GTCAAAATACTGGATAAATGTGAACTTTTCACTATAGTGGGGATCCAAAACCTCATTGAT

AGAGATCTCTTGTCAATACTAAATGGTAGGCTCTTCATGCATCAACTGATTCAAGATCTG

GGGAGAGAAATTGTTCGTCAAGAATCTGTTAAGGAGCCAGGGAGGCGTAGTAGACTGTGG

CGTCATGAAGAATCTTTGTACGTATTGAGAAACAAAACGGGAACTGAAGCAATTGAAGGC

ATCATTCTCGACGGAAATATGTGTAAGGGGCATGGATCAACCAGGATAACATCTAATGAA

AATTATGGCAAAAAAAGTAAAGTGGAAGAATTTATGAATAATTCTCAGGAAGATCGACCA

AAGCAGAATTGGATGTCCATTTTCTCTCGTCATATCATGGGCACAAGGGAAGTTCCAAAT

GAGGATTTGGAAACTGACTCATTCACAAACATGCTCAAGTTGAAATTTCTGTTGCTTAGC

AATATACAACTTTCTGGATGTTACAGGAAATTTCCCAAGAAATTAAGATGGTTGTTTTGG

CGTTATCTCCAGTTAGAATCCCTACCAAGCGACTTTCCGATGGGAAAACTTGTTGCTATA

GACCTTTGTTACAGCAGCTTGAAACAACTTTGGACAGCACCAAAGGTACTTTGTCCATGC

CCTTTGTCATTAACTGCTTTTACTCAGATGGTTAAAGTTTCTCAATCTCAGCCACTCTTA

TCAGCTTAG

>NTK326_mRNA_72081_cds NTK326_mRNA_72081 gene_41683|id=AT5G17680.1

ATGGCATCATCTTTTGCTTCTGCGAGTACTTCACAGTTTCCTCGATGGAACTACAAAGTC

TTTCTAAGTTTTAGAGGTGAAGATACTCGAAAAACATTTACAGGTCACCTCTTCAAAGGG

TTGGAAAACAATGGAATATTTACGTTTCAAGATGATAAAAGGCTAGAGCATGGCGCATCA

ATATCAGATGAACTCTTGAAAGCTATCGAACAGTCTCAAGTTGCCCTCGTCGTTTTCTCA

AAGAATTATGCAACATCGAGGTGGTGCTTAGATGAGTTAGTAAAGATCATGGAATGCAAG

GATCAATGTGGACAGACTGTCATACCAGTCTTCTATGATGTGGATCCATCACATGTTCGA

AACCAGAGAGAGAGCTTTGCCGAAGCCTTTGACAAACACGAACCAAGATATAGGGATGAT

GATGAAGGAAGGCAGAAGCTCCAAAGATGGAGGAATGCTCTAACTGCTGCCGCAAATCTA

AAAGGATATGATGTCCGTGACGGGATTGAAGCAGAGAATATTCAGCAGATTGTCGACCAA

ATTTCCAAATTGTGCAATAGTGCTACTTTGTCTTCTTTGCGAGATGTTGTGGGAATAGAT

ACTCATTTGGATAAATTAAAGTCCCTACTTAAGGTAGGAATCAATGATGTTCGGATCATA

TTGGGGATCTGGGGCATGGGTGGACTAGGGAAGACGACGATAGCAAGAGTCATTTTTGAC

ATTTTATCTCATCAATTTGAAGCTGCTTGTTTCCTTGCGGATATAAAAGAAAATGAAAAA

AGACATCAACTGCATTCTTTGCAAAACACCCTTCTCTCTGAATTGTCAAGAAGAAAAGAT

GATTACGTCAATAATAAGCATGATGGGAAGCGGATGATTCCAGACAGACTTTTCTCTAAG

AAGGTGCTAATTGTGCTTGATGATATAGATCATAAAGATCATTTAGAGTATTTAGCAGGT

GATATTGGTTGGTTTGGTAATGGTAGTAGAGTTGTTGTAACAACTAGAAACAAACATTTG

ATAGAAAAGAATGATGTCATTTATGAAATGACTGCACTATCTGATCATGAATCCATTCAA

TTGTTCTGTCAACATGCTTTCAGAAAAGAAGATCCAGATGAGCATTTTAAGAAGCTTTCA

TTGGAGGTAGTAAAATATGCCAATGGCCTTCCTTTAGCCCTCAAAGTGTGGGGTTCTCTG

CTGCATAACCTAGGCTTAACTGAATGGAAAAGTGCAATAGAGCAAATGAAAATTAATTCT

AATTCGGAAATTGTTGATAAGCTCAAAATCAGTTATGATGGATTAGAGCCCATACAACAG

GAGATGTTTCTAGATATAGCATGCTTCTTACGAGGGGAACAAAAAGCTTACATCCTACAA

ATTCTTGAGAGCTGTCATATTGGAGCTGAATATGGATTGCGTATTTTAATTGACAAATCT

CTTGTGTTCATCACTGAAGATTATCAGATTATTCAAATGCATGACTTAATTCAAGATATG

GGTAAATATATCGTGAACTTGCAAAAGAATCCGGGAGAACGCAGCAGACTATGGCTCAAC

GAGGATTTCGAAGAAGTGATGACCAACAATGCAGGGACCGTGGCAGTGGAAGCAATTTGG

GTTCATGATTTGGATACACTACGCTTTAACAATGAGGCCATGAAAAATATGAAAAAGCTT

AGGATATTATACATAGACAGAGAGGTCTATGATTTCAATATTAGCGATGAACCCATTGAG

TATCTATCCAACAACTTGCGTTGGTTTAACGTGGATGGCTATCCTTGTGAGTCATTGCCA

TCTACATTTGAACCCAAAATGCTTGTTCACCTTGAACTCTCATTTAGTTCACTGCGTTAT

TTATGGATGGAAACAAAGCATTTGCCGTCTCTACGGACGATAAATCTCACGGGCTCTGAA

AGCCTGATGCGAACACCAGATTTCACGGGGATGCCAAATTTGGAGTATTTGGATATGTCT

TTCTGTTTTAATCTTGAAGAGGTTCACCATTCCTTGGGATGTTGCAGCAAACTCATTGGG

TTAGATTTGACCGATTGTAAAAGCCTTAAGAGGTTTCCATGTGTTAACGTGGAATCTCTT

GAATATCTGGATTTACCAGGTTGCTCAAGTTTAGAGAAATTTCCAGAAATCCGCGGGAGA

ATGAAGCTGGAGATACAGATTCACATGAGATCTGGGATAAGGGAACTACCATCATCTAGT

TTTCACTACCAGACTCGTATTACCTGGCTAGATTTGAGCGATATGGAAAACCTTGTAGTT

TTTCCAAGCAGCATCTGTCGGTTGATAAGTTTGGTTCAATTATTTGTGTCTGGTTGCTCA

AAACTGGAAAGCTTGCCAGAAGAGATAGGGGATTTAGACAACTTGGAGGTGCTTTATGCC

AGTGATACTCTAATTTCACGACCTCCATCTTCCATCGTACGCTTGAACAAACTTAACAGC

TTGAGCTTTAGGTGCTCCGGAGACAATGGAGTGCACTTTGAGTTCCCTCCAGTGGCTGAA

GGATTACTGTCATTGAAAAATCTAGATCTCAGTTATTGCAATCTAATAGATGGAGGACTT

CCGGAAGACATTGGATCCTTATCCTCTTTGAAAGAATTGGATCTCAGAGGAAATAATTTT

GAGCATTTGCCTCGAAGCATAGCCCAACTTGGTGCTCTTCGATCCTTAGGCTTATCATTT

TGCCAGACGCTTATACAACTGCCAGAACTTTCCCATGAATTAAATGAATTGCATGTAGAT

TGTCATATGGCTCTGAAATTTATCAATGATTTAGTAACAAAGAGGAAGAAACTACAGAGG

GTGGTATTCCCGCCACTGTATGATGATGCACACAATGATTCTATATATAATTTATTTGCA

CATGCCCTGTTTCAGAATATCTCTTCCTTGAGGCATGACATCTCTGTTTCAGATTCCTTG

TTCGAAAATGTGTTTACCATTTGGCATTATTGGAAGAAGATCCCAAGTTGGTTCCACCAT

AAGGGAACTGATAGTAGTGTATCAGTCGATTTGCCTGAAAATTGGTATATACCTGACAAA

TTCTTGGGTTTTGCTGTATGTTACGATGACATTTTAATTGACACCACAGCTCAATTGATT

CCCGTATGTGATGATGGGATGTCGTGCATGACCCAGAAACTTGCCTTATCAGAATGTGAT

ACAGAATCATCCGATGATTCAGAACGGTATACACCAATTCATTTTTTCTTTGTACCTCTT

GCTGTCTTATGGGATACATCTAAGGCAAATGGAAAAACACCAAATGACTATGGGATTATT

AGGCTATCTTTTTCTGGAGAAATGAAGAAGTATGGACTTCGTTTGTTGTATAAAGAAGAA

GCTGAGGTTGAGGCCTTGTTACAAATGAGGGAAAATAACAATGAACCAATAGAACATTCC

AATGTGATAAGGAGGAGCAGATCTGACAATAGTGAACACCATGACTCCGTGACCGATGAA

TCCAGTTTATGTTGCTGTCGCATACTGTAA

>NTTN90_mRNA_125459_cds mRNA_125459 gene_70838|id=AT5G17680.1:evalue=5e-31:annot='disease resistance protein (TIR-NBS-LRR class), putative';id=Solyc11g011350.1.1:evalue=0.0:annot='Tir-nbs-lrr, resistance protein'

ATGGCATCATCTTTTGCTTCTGCGAGTACTTCACAGTTTCCTCGATGGAACTACAAAGTC

TTTCTAAGTTTTAGAGGTGAAGATACTCGAAAAACATTTACAGGTCACCTCTTCAAAGGG

TTGGAAAACAATGGAATATTTACGTTTCAAGATGATAAAAGGCTAGAGCATGGCGCATCA

ATATCAGATGAACTCTTGAAAGCTATCGAACAGTCTCAAGTTGCCCTCGTCGTTTTCTCA

AAGAATTATGCAACATCGAGGTGGTGCTTAGATGAGTTAGTAAAGATCATGGAATGCAAG

GATCAATGTGGACAGACTGTCATACCAGTCTTCTATGATGTGGATCCATCACATGTTCGA

AACCAGAGAGAGAGCTTTGCCGAAGCCTTTGACAAACACGAACCAAGATATAGGGATGAT

GATGAAGGAAGGCAGAAGCTCCAAAGATGGAGGAATGCTCTAACTGCTGCCGCAAATCTA

AAAGGATATGATGTCCGTGACGGGATTGAAGCAGAGAATATTCAGCAGATTGTCGACCAA

ATTTCCAAATTGTGCAATAGTGCTACTTTGTCTTCTTTGCGAGATGTTGTAGGAATAGAT

ACTCATTTGGATAAATTAAAGTCCCTACTTAAGGTAGGAATCAATGATGTTCGGATCATA

TTGGGGATCTGGGGCATGGGTGGACTAGGGAAGACGACGATAGCAAGAGTCATTTTTGAC

ATTTTATCTCATCAATTTGAAGCTGCTTGTTTCCTTGCGGATATAAAAGAAAATGAAAAA

AGACATCAACTGCATTCTTTGCAAAACACCCTTCTCTCTGAATTGTCAAGAAGAAAAGAT

GATTACGTCAATAATAAGCATGATGGGAAGCGGATGATTCCAGACAGACTTTTCTCTAAG

AAGGTGCTAATTGTGCTTGATGATATAGATCATAAAGATCATTTAGAGTATTTAGCAGGT

GATATTGGTTGGTTTGGTAATGGTAGTAGAGTTGTTGTAACAACTAGAAACAAACATTTG

ATAGAAAAGAATGATGTCATTTATGAAATGACTGCACTATCTGATCATGAATCCATTCAA

TTGTTCTGTCAACATGCTTTCAGAAAAGAAGATCCAGATGAGCATTTTAAGAAGCTTTCA

TTGGAGGTAGTAAAATATGCCAATGGCCTTCCTTTAGCCCTCAAAGTGTGGGGTTCTCTG

CTGCATAACCTAGGCTTAACTGAATGGAAAAGTGCAATAGAGCAAATGAAAATTAATTCT

AATTCGGAAATTGTTGATAAGCTCAAAATCAGTTATGATGGATTAGAGCCCATACAACAG

GAGATGTTTCTAGATATAGCATGCTTCTTACGAGGGGAACAAAAAGCTTACATCCTACAA

ATTCTTGAGAGCTGTCATATTGGAGCTGAATATGGATTGCGTATTTTAATTGACAAATCT

CTTGTGTTCATCACTGAAGATTATCAGATTATTCAAATGCATGACTTAATTCAAGATATG

GGTAAATATATCGTGAACTTGCAAAAGAATCCGGGAGAACGCAGCAGACTATGGCTCAAC

GAGGATTTCGAAGAAGTGATGACCAACAATGCACATTTGCCGTCTCTACGGACGATAAAT

CTCACGGGCTCTGAAAGCCTGATGCGAACACCAGATTTCACGGGGATGCCAAATTTGGAG

TATTTGGATATGTCTTTCTGTTTTAATCTTGAAGAGGTTCACCATTCCTTGGGATGTTGC

AGCAAACTCATTGGGTTAGATTTGACCGATTGTAAAAGCCTTAAGAGGTTTCCATGTGTT

AACGTGGAATCTCTTGAATATCTGGATTTACCAGGTTGCTCAAGTTTAGAGAAATTTCCA

GAAATCCGCGGGAGAATGAAGCTGGAGATACAGATTCACATGAGATCTGGGATAAGGGAA

CTACCATCATCTAGTTTTCACTACCAGACTCGTATTACCTGGCTAGATTTGAGCGATATG

GAAAACCTTGTAGTTTTTCCAAGCAGCATCTGTCGGTTGATAAGTTTGGTTCAATTATTT

GTGTCTGGTTGCTCAAAACTGGAAAGCTTGCCAGAAGAGATAGGGGATTTAGACAACTTG

GAGGTGCTTTATGCCAGTGATACTCTAATTTCACGACCTCCATCTTCCATCGTACGCTTG

AACAAACTTAACAGCTTGAGCTTTAGGTGCTCCGGAGACAATGGAGTGCACTTTGAGTTC

CCTCCAGTGGCTGAAGGATTACTGTCATTGAAAAATCTAGATCTCAGTTATTGCAATCTA

ATAGATGGAGGACTTCCGGAAGACATTGGATCCTTATCCTCTTTGAAAGAATTGGATCTC

AGAGGAAATAATTTTGAGCATTTGCCTCGAAGCATAGCCCAACTTGGTGCTCTTCGATCC

TTAGGCTTATCATTTTGCCAGACGCTTATACAACTGCCAGAACTTTCCCATGAATTAAAT

GAATTGCATGTAGATTGTCATATGGCTCTGAAATTTATCAATGATTTAGTAACAAAGAGG

AAGAAACTACAGAGGGTGGTATTCCCGCCACTGTATGATGATGCACACAATGATTCTATA

TATAATTTATTTGCACATGCCCTGTTTCAGAATATCTCTTCCTTGAGGCATGACATCTCT

GTTTCAGATTCCTTGTTCGAAAATGTGTTTACCATTTGGCATTATTGGAAGAAGATCCCA

AGTTGGTTCCACCATAAGGGAACTGATAGTAGTGTATCAGTCGATTTGCCTGAAAATTGG

TATATACCTGACAAATTCTTGGGTTTTGCTGTATGTTACGATGACATTTTAATTGACACC

ACAGCTCAATTGATTCCCGTATGTGATGATGGGATGTCGTGCATGACCCAGAAACTTGCC

TTATCAGAATGTGATACAGAATCATCCGATGATTCAGAACGGTATACACCAATTCATTTT

TTCTTTGTACCTCTTGCTGTCTTATGGGATACATCTAAGGCAAATGGAAAAACACCAAAT

GACTATGGGATTATTAGGCTATCTTTTTCTGGAGAAATGAAGAAGTATGGACTTCGTTTG

TTGTATAAAGAAGAAGCTGAGGTTGAGGCCTTGTTACAAATGAGGGAAAATAACAATGAA

CCAATAGAACATTCCAATGTGATAAGGAGGAGCAGATCTGACAATAGTGAACACCATGAC

TCCGTGACCGATGAATCCAGTTTATGTTGCTGTCGCATACTGTAA

>NTTN90_mRNA_41259_cds mRNA_41259 gene_23091|id=AT5G17680.1:evalue=1e-170:annot='disease resistance protein (TIR-NBS-LRR class), putative';id=Solyc01g014840.2.1:evalue=0.0:annot='Tir-nbs-lrr, resistance protein'

ATGGCTTCTACTTCTTCACCCACTCAAAATTGGAAGAATGATGTTTTCTTGAGTTTTAGA

GGTGAAGATACTCGTAAAACTTTTGTGGGTCATCTCTACTATGCTCTAAAACATAAAGGG

ATTCATACTTTCAAAGATGATGTAAGGCTGGAGAGAGGAAAGTCCATTTCACCTGAACTT

GTGAAAGCTATTGAACAATCAAGATTTGCTATTGTTGTATTTTCTAAGAACTATGCATCC

TCCACTTGGTGCTTGGATGAACTTGTAAAGATCATGAAATGCAAGAAAGAATTAGGACTA

ACTGTGATGCCCATATTCTATGACGTAGATCCATCGGATGTGAGTAAGCAAAGTGGAACT

TTTGCTGAATCATTTTCTAGACATGAGGAAAATTTGAGAGATGATTTGGAGAAGGTGCAA

TGTTGGAGGGATGCATTTGGTGAGGCAGGCAAAACAGCAGGATATGATTTACCAAATGGC

TACGACGGGTATGAATCTAATTGCATCCAGCATGTTGTTGAAGACATACTGGGTAAATTG

TGTCAAGTTACTTCAACCATTGATAATGATTTAGTGGGGATGGAATCTCGAGTGCGTGAA

GTAAGTTCATTACTAAGGATGGAAACACATGATGTTCGTTTTATTGGAATTTGGGGGATG

GGCGGCATTGGTAAGACAACAATTGCAAGCGCTGTGTTTGGCAAATATTCTGGCCTATTT

GAAGGTGTTTGTTTTCTTGATAATGTTGCAGAAATGCAAAGGACATATGGACTGCAATAT

TTGCAAGGTGTTCTCCTCTCAAAAATCCTAAAGGTAAGCTTAACTATTACAAGTGTATAT

GAAGGCATGGAAATCATAAAGAAGAGGTTGCGCACAATGAAGGTTTTGATCATTCTTGAT

GATGTAAATCAAAAAGACCAATTAAAAATGTTAGTTGGATGGCATGATTGGTTTGGTAGT

GGTAGTAGAATTTTGATTACAACAAGAGATAAACATTTGTTAGATAATCATATTGTGGAT

GAAGTGTATTCTGTGAACTTGATGACTCTTAATGAAGCTATTGAGCTATTTAGCCTACAT

GCCTTTAAGCAAAGAATTCCTAAGAAAGACTTTGAGGAGCTTTCAAATCAAGTTGTACAT

TGTGCCGCTTTGCTCCCTTTAGCTTTGAAAGTTTTAGGTTCGTTTCTCTATGGATTAGAC

AGGAGCCAGTGGAGATCCGCTTGGGAAAGCCTGAAGGATCTGCCAAATGATGAAATTCTT

GCTAAGCTTAAGATAAGTTTTGAAGGACTGGGGCATGTTGATCAGAGACTCTTTCTAGAT

ATTGCATGCTTTTATAGAGGAAAATTGAGGAGTTATGTAGAGGAAATACTTGAGAGCTGC

GATATCGGATCTACAATAAGAATAAAAGTCTTAATTGAAAAGTCTCTCTTATTTATCTCA

CCATATGACACAATTGAAATGCATGATTTGATACAAGAAATGGCCTGGCACATCGTGAGT

CAAGATGACTCGCGAAGGAGTAGAATATGGCTTCCCGAGGACATCGAGGATTTGTTTACT

GGGAATTTGGAAGCAGAATCTGTGGAGGGACTATGGATACCAAGGAATTACATTCCAAAA

CAGGATATATCATATTACAACATCAGTGAAGCATTTAGGAGAATGAAAAGATTAAGGGTA

CTTGTAGTTAGAGCAACAAATTTCTGCTCTATTGACCCGATTACTCATCTTCCTAGCAGC

CTAAGGTGGCTTGATTGGGAAGCTTGCCCTTTAAATTCATTGCCACAGAGTTTTGAACCA

TCAAAGCTTCTTCGCCTTGATATACTCGAATGTAGTACACTTCAGAAACTCTGGTTAATT

CCGAAGGGTTTGGACAAATTAAAAACTTTGTACCTCAGCTATTGCGAACACTTGGAAGAA

GTTCCAAGCTTTGAGTTGATGCCAAATTTAGAGAGAGTAAAGCTAGAGGGATGTAAGAGT

TTGAGAGAAGTGAGCCCATCGTTTGGAGTTCTCATGAAGCTCACTTCACTGGAGCTAATT

GATTGTCAGAGCCTTGAGAAGCTTCCAAGTTATATTCAGATGGAATCCCTTAAGAGTCTC

AAACTTTCTTGTCTTCCAAAGTTGAGGGAATTACCAGAAACCAAGGGGTTGCACCGTTTA

TTGACATTGGAGCTAACTGATTGTCAGAGTCTTGAGATGCTTCCAAGTTGTAATCAGATG

GAATCTCTTGTGATTCTCAAACTTTCTTGTCTTCCAAAAATAATGGCTTTGCCGGCAACA

GAAGGGATGCACCATTTATTGGAACTTGTTATAGAATATACTCCAATAGTAGAGCTTCCG

GTGTCAATTGGAAATCTTGGTTCCCTCAAACAACTATGGTTAAGTCATTGTAAAGATCTG

GTAAGCATTCCGAACAGCTTTTCTTGTCTGAAGAATCTAAGAGTTCTTGTGATCTACAAC

TGCAAAAGATTTGCAGATTTGCCAGAGAAGATGGGGGACTTGAAGCTTTTAGAAAAGCTA

GTAATATCTGGTACTGCAATTTCCCGTATACCCCCTTCAGTTGCAGACCTTGGTGAACTA

AGCTTTTTATCATTCTCTCGCTGGTTTGGATACAGAGAAGATGCAACTTTTCTGTTACCC

TCTGCATCAGGTTCATCGTCGTTTAGGGTGTTAAAGCTTAAGAAGCACACACTATGCAGT

GGAGAACATTTTCAGGATCTTGGATGCTTATCTTCTTTGGCTCACTTGGATTTCACTAGA

AATGATTTTACGAGTTTCAATGAAAGCAACAATCAGCCCTTTCATTACCTAGATATAACA

TTTTGTGAGAAGCTTGTATTGCCCAGACTTCCAGCATGCATAAAGGAGTTATATGCATAT

GATCCTTTAGTCTTGAAAAGCATTCCTGATTTCCCCACAAAATATTCAGAGCTGTATTCA

GTGTCATTCGCACAGCATATTGAGAACAGAGGGGAACTGACTGATATCTTGCACTTTGTC

CTTCGTTTAATTAGTGCGGCATCTCAGTGTGAGAAAGTGCTACCTTTTAGCATTTTTTCC

CCTGGAGATATAAGATGGAGCGGGTTCAATTATTATCGAAAAGAACATACAAAAAGATTC

TCCACTCCACTTGATCCATGTTGGTATGAGAGTAAATTCAAGGGATTTGTTATATGCTTT

CGTGTACCATTGGATACTGTTCAGAACCAGAAACCTTTGGATGCTAAATCACGAAGAGGA

AGTCACTGGTTCGGTTGCACTAAGGTTACAGTTAAGTTAGTGCAAAGATATGACAGGCAA

GAACAAGATGTACTCCAGAAAAAATGTTTGATTGTTGCTCGCCAAGCATTTTGCTCTCAT

AGTAGTAAATATGCCATTTGCTTTAGCTACATACCTTTTGTAGCACTATGGCATACTTCT

GATAGTGAAAAGGGGAAGAAGCCAAATGACTATTGCTTCTTTGAGGCGTCTATAGACCCA

GGCACTGCAACAAAATGGGGACTTCTTCTGGTGTACGAGAATAAAATTCAACAGATAGAT

CAATCAACCATCGTGGTCCAACGTGATGTTGAGTCTCCAAGTTCTGACCTGTTGAGAGAA

TCTAATGATGACCAAGGCCAGAAAACGGAGGATGCTTCTGTTAAGAGAAGACGGCTTGAT

ATTTGTCAAAGAGATAATATGGTTTCATTTGAAGCTGGCTGCTCTATGAAATTTCAAGCG

ATAAAGGACTCATGCTCTCCCAGTGAGTTTCAAACTTTCCAAATAATTCCTGATCAACAA

TTGGAAACACCATGCTCTTCTGCAGCTCAAAGCTTCCGACACAGGGAAGAGTCATGCTCT

TCTGGACAGCCACAAACTTTACAGCTCCCTCTAGCTGATCCACAAGTTGATGAAGTGATA

AATGAGGCTACTTCCGGCATGGTATTTGAACAACTGGAAGCGCCAAGCTCTTCCGAGCAG

CCTGAATCTTTTGAAGTCTCTCCAGATGAGCACAAAGATAATTCAGTGACAAATGGGTCT

AGCAGCTCTGAGGTATTCCAAGAATTGGAGGCACCATGCTCTTCTGGACAACCTCAAATT

CTCCAGCTCTTTCCCGAGCATTCATGA

>NTTN90_mRNA_40745_cds mRNA_40745 gene_22802|id=AT5G17680.1:evalue=8e-174:annot='disease resistance protein (TIR-NBS-LRR class), putative';id=Solyc01g014840.2.1:evalue=6.0:annot='Tir-nbs-lrr, resistance protein'

ATGGCTTCTACTTCTTCACCCACTCAAAATTGGAAGAATGATGTTTTCTTGAGTTTTAGA

GGTAAAGATACTCGTAAAACTTTTGTGGGTCATCTCTACTATGCTCTAAAACACAAAGGG

GTTCACACTTTCAAAGATGATGTAAGGTTAGAGAGAGGAAAGTCCATTTCACCTGAACTT

GTGAAAGCTATTGAACAATCAAGATTTGCTATTGTTGTATTTTCTAAGAACTATGCATCC

TCCACTTGGTGCTTGGATGAACTTGTAAAGATCATGAAATGCAAGAAAGAATTAGGACAA

ACTGTGATACCCATATTCTATGACGTAGATCCATCGGATGTGAGTAAGCAAAGTGGAACT

TTTGCTGAATCATTTGCTAGACATGAGGAAAATTTTAGAGATGATTTGGAGAAGGTGCAA

TCTTGGAGGGATGCATTTGGTGAGGCAGGCAAAACAGCAGGATATGATTTACCAAATGGC

TACGACGGGTATGAATCGAATTGCATCCAGCATGTTGTTGAAGACATACTGGGTAAATTG

TGTCAAGTTACTTCAACCATTGATAATGATTTAGTGGGGATGGAGTCTCGAGTGCGTGAA

GTAAGTTCATTACTAAGGATGGAAACACCTGATGTTCGTTTTATTGGAATTTGGGGGATG

GGCGGCATTGGTAAGACAACAATTGCAAGCGCTGTGTTTGGCAAATATTCTGGCCTATTT

GAAGGTGTTTGTTTTCTTGATAATGTTGCAGAAATGCAAAGGACATATGGACTGCAATAT

TTGCAAGGTGTTCTCCTCTCAAAAATCCTAAAGGTAAGCTTAACTATTACAAGTGTATAT

GAAGGCATGGAAATCATAAAGAAGAGGTTGCGCTCAATGAAGGTTTTTATCATTCTTGAT

GATGTAAATCAAAAAGACCAATTAGAAATGTTAGTTGGACGGCATGATTGGTTTGGTAGT

GGTAGTAGAATTTTGATTACAACAAGAGATAAAAATTTGTTAGATAATCATATGGTGGAT

GAAGTGTATTCTGTGAACTTGATGACTCTTAATGAAGCTATTGAGCTATTTAACCTACAT

GCCTTTAAGCAAAGAATTCCTAAGAAAGACTTTGAGGAGCTTTCAAATCAAGTTGTACAT

TGTGCCGGTTTGCTCCCTTTAGCTCTGAAAGTTTTAGGTTCGTTTCTCTATGGATTAGAC

AGGAGGCATTGGAGATCAACTTGGAAAAGGCTGAAGGATCTGCCAAATGATGAAATTCTT

GCTAAGCTTAAGATAAGCTTTGAAGGACTGGGGCATGTTGATCAGAGACTCTTTCTAGAT

ATTGCATGCTTTTATAGAGGAAAATTGAGGAGTTATGTAGAGGAAATACTTGAGAGCTGT

GATATCGGATCTACAATAAGAATAAAAGTCTTAATTGAAAAGTCTCTTTTATTTATCTCA

CCATATGACACAATTGAAATGCATGATTTGATACAAGAAATGGCCTGGCACATCGTGAGT

CAAGATGACTCACGAAGGAGTAGAATATGGCTTCCTGAGGACATTGAGGATTTGTTTACT

GGAAATTTGGAAGCAGAATCTGTGGAGGGACTATGGATACCAAGGAATTACATTACAAAA

CAGGATATATCATATTACAACATCAGTGAAGCATTTAGGAGAATGAAAAGATTAAGGGTA

CTTGTAGTTAGAGCAACAGATTTCTGCTCTATTGACCCGATTACTCATCTTCCTAGCAGC

CTAAGGTGGCTTGATTGGGAAGGTTGCCCTTTAAATTCATTGCCACAGAGTTTTGAACCA

TCAAAGCTTCTTCGCCTTGATATACTCGAATGTACTACACTTCATAAACTCTGGTTAATT

CCGAAGGGTTTGGACAAACTAAAAACTTTGTACCTCAGCTATTGCGAACACTTGGAAGAA

GTTCCAAGCTTTGAGATGATGCCAAATTTAGAGAGAGTAAAGCTAGAGGGATGTAAGAGT

TTGAGAGAAGTGAGCCCATCCTTTGGAGTTCTCATGAAGCTCATTTCACTGGAGCTAATT

GATTGTCAGAGCCTTGAGAAGCTTCCAAGTTATATTCAGATGGAATCCCTTAAGAGTCTC

AAACTTTCTTGTCTTCCAAAGTTGAGGGAATTACCAGAAACCAAGGGGTTGCACCGTTTA

TTGACATTGGAGATAACTGATTGTCAGAGTCTTGAGATGCTTCCAAGTTGTAATCAGATG

GAATCTCTTGCAACTCTCAAACTTTCTTGTCTTCCAAAAATAATGGCCTTGCCGGCAACA

GAAGAGATGCACCATTTATTGGAACTTGTTATAGAATATACTCCAATAGTAGAGCTTCCG

GTGTCAATTGGAAATCTTGGTTCCCTCAAACAACTACGGTTAAGTCATTGTAAAGATCTA

GTAAGCATTCCGAACAGCTTTTCTTGTCTGAAGAATCTAAGAGTTCTTGTGATCTACAAC

TGCAAAAGACTTGCAGATTTGCCAGAGAAGATGGGTGAGTTGAAGCTGTTAGAAAAGCTA

GTATTATCTGGTACTGCAATTTCCCAAATACCTCCTTCAGTTGCAGACCTTCATGAACTA

AGCTTTTTATCATTCTCTCCCTGGTTTGGATACAGAGAAGATGCAACTTTTCTGTTACCC

TCTGCATCAGGTTCATCGTCATTTAGGGTGTTAAAGCTTAATAAGCACACACTATGTAGT

GGAGAACATTATCAGGATCTTGGATGCTTATCTTCTTTGGCTCACTTGGATTTGACTAGA

AATGATTTTACTAGTTTCAATGAAAGCAACAATCAGCACTTTCATTACCTAGATATAACA

TTTTGTGAGAAGCTTGTAATGCCCAGACTTCCATCATGCATAAAGGAGTTATATGCATAT

GATCCTTTAGTCTTGAAAAGCATCCCTGATTTCCCCACCAAATATTCAGAGCTGTATTCA

GTGTCATTCACACAGCATATTGAGAATAGAGGTGAACTGACTGATATCTTGCACTTTGTC

CTCCACTTAATTAGTGTGGCATCTCAGTCTGAGAAAAGGCTACCATTTAGCATTTTTTTC

CCTGGAGATATAAGATGGAGCGGGTTCAATTATTATCGAAAAGAGCATACGAAAAGATTC

TCCACTCCACTTGATCCAAGTTGGCATGAGAGTAAATTCAAAGGATTTGTTATATGCTTT

CGTGTACCATTGGATACTGTTCAGAACCAGAAACCTTTGGATTCTAAATCACGAAGAGGA

AGTCACTGGTTCGGTTGCACTAAGGTTACAGCTAAGTTAGTGCAAAGATATGACAGGCAA

GAACAAGATGTACTCCAGAAAAAATGTTTGATTGTTGCTCGCCAAGCAATTTGCTCTCAT

AGTAGTAAATATGCCATTTGCTTTAGCTACATACCTTTTCTAGCACTATGGCATACTTCT

GATAGTGAAAAGGGGAAGAAGCCAAATGATTATTGCTTCTTTGAGGCGTCTATAGACCCA

GGCATTGCAACAAAATGGGGACTTCTTCTGGTGTACGAGAATAAAATTAAACAGATAGAT

CAATCAACCATCTCGGTCCAACGTGATGTGGAGTCTCCAAGTTCTGACCTGTTGAGAGAA

TCTAATGATGACCAAGTCCAGAAAACGGAGGATGCTTCTGTTAAGAGAAGACGGGTTGAT

TTTTGTCAAAGAGATAATATGGTTTCATTTGAAGCTGGCTGCTCTATGAAATTTCAAGCG

ATGAAGGACTCATGCTCTCCCAGTGACTTTCAGACTCTCCAAATAATTCCTGATCAACAA

TTGGAAACACCATGCTCTTCTGCAGCTCAAAGCTTCCGACACAGGGAAGAGTCATGCTCT

TCTGGACAGCCACAAACTTTACAGCTCCCTCCAGCTGATCGACAAGTTGATGAAGCGATA

AATGAGGCTACCTCCTGCATGGTATTTGAACTGGAAGCGCCAAGCTCTTCGGAGCAGCCT

GAATCTTTTGAAGTCTCTCCAGATGAGCGCAAAGATAATTCAGTGACAAATGGGTCAAGC

AGCTCTGAGGTATTCCAAGAATTGGAGGCACCATGCTCTTCTGGACAACCTCAAATTCTC

CAGCTCTTTCCCTAG

>NTK326_mRNA_90605_cds NTK326_mRNA_90605 gene_53249|id=AT5G17680.1

ATGGCATCTCCTTCTCCTTCTTCTTCTTCTTCGGCTAGATGGAGCTATGATGTTTTCCTA

AGTTTTAGAGGTGAAGATACTCGGAAAACGTTTACAAGTCACTTATACGAAGTCTTGAAG

GATAGGGGAATAAAAACCTTTCAAGATGAAAAAAGGCTAGAGTACGGTGCAACCATCCCA

GAGGAACTCTCTAAAGCTATAGAAGAGTCTCAATTTGCCATCGTCGTTTTCTCGAAGAAT

TATGCGACATCGAGGTGGTGTTTGAATGAACTAGTGAAGATCATGGAATGCAAGACTCAA

TTTAGACAAACTGTTATACCGATATTCTATGATGTGGATCCATCACATGTTCGGAACCAA

AAGGAAAGCTTTGCAAAAGCCTTTGAAGAACATGAAACAAAGTATAAAGATGATGCCGAG

GGAATACAAAGATGGAGGATTGCTTTAAATGCAGCGGCCAATCTCAAAGGCTCATGTGAT

AATCGTGACAAGAGTGATGCAGATTGTATTCGGCAGATTGTTGGCCAAATCTCATCCAAA

TTATGCAAGATTTCTTTATCTTATCTGCAAAACATTGTTGGAATAGATACTCATTTAAAG

AAAATAGAATCCTTACTCGAGATAGGAATCAATGATGTTCGGATTGTGGGAATCTGCGGA

ATGGGTGGAGTCGGTAAAACGACAATAGCAAGAGCTATGTTTGATACTCTCTTAGTAAGA

AGGGATAGTTCCTATCAATTTGATGGTGCTTGTTTCCTTGAGGATATTAAAGAAAACAAA

GGTAGAATAAATTCTCTGCAAAATACCCTTCTCTCTAAACTGTTAAGGGAAAAAGCTGAG

TACAATAATAAGGAGGACGGAAAGCACCAAATGGCTAGTAGACTACGTTCTAAGAAGGTC

CTAATTGTGCTTGATGACATAGATGATAAAGATCATTATTTGGAGTATTTAGCAGGTGAC

CTTGATTGGTTTGGTAATGGCAGTAGAATTATTGTAACAACTAGAGACAAGCATTTGATA

GAGAAGTTTGGTATACATCTAGTGACTGCTCTAACTGGTCATGAAGCTATTCAATTGTTC

AATCAATATGCTTTTGGGAAAGAAGTTTCAGATGAGCATTTTAAGAAGCTTTCATTGGAG

GTAGTAAAATATGCTAAAGGCCTTCCTTTAGCCCTAAGAGTGTTGGGTTCTTCCTTACGT

AATAGGGGTATAACTGTGTGGAAAAGTGCTATAGAGCAAATGAAAAATAATCCTAATTCA

AAAATTGTTGAAAATCTCAAAATTAGTTATGATGGATTAGAGCCCATACAACAAGAGATG

TTCCTAGATATAGCATGCTTCTTCCGAGGGAAAGAAAAAGGTGCCATCATGCAAGTTCTT

AAGAGTTGCGATTGTGGAGCTGAATACGGATTGGATGTCCTAATTGAAAGATCTCTTGTG

TTCATCACTAAATACAGTAAAATTGAAATGCATGACTTAATACAAGAAATGGGTAGATAT

ATAGTGAACTTGCAAAAGAATCTGGGAGAATGCAGCAGACTATGGCTCACCAAGGATTTT

GAAGAAATGATGATCAACAATACGGGTACCATGGCAATGGAAGCAATCTGGGTATCGACC

TATAGTACACTACGCATTAGCAATGAGGCCATGAAAAATATGAAAAGGCTTAGGATATTA

TACATAGACAATTGGACGTGGTCCTCTGATGGTTCCTATATTACCCATGATGGCTCAATT

GAGTATCTGTCCAACAACTTGCGTTGGTTTGTTTTGCCTGGCTATCCTCGTGAGTCATTG

CCATCTACATTTGAACCCAAAATGCTTGTCCATCTTAAACTCTCTGGTAATTCATTGCGT

TATTTATGGATGGAAACAAAGCATTTGCCGTCTCTGCGGAGGATAGATCTCAGTCGGTCT

AAAAGACTAATGCGAACACCAGATTTCACGGGGATGCCAAATTTGGAGTACTTGGATCTG

ACTTGGTGTTCTAATCTTGAAGAGGTTCACCATTCCCTGGGGTGTTGCAGAAAACTCATT

CGGTTAGATTTGTATAATTGTAAAAGCCTTATGAGGTTTCCATGTGTTAACGTGGAATCT

CTTGAATATCTGGGTTTAGAATATTGCGATAGTTTAGAGAAATTTCCAGAAATCCACAGG

AGAATGAAGCCGGAGATACAGATTCACATGGGAGACTCTGGGATAAGGGAACTGCCATCC

TCTTATTTTCAGTACCAAACTCATATTACCAAGCTAGATTTGAGCGGTATAAGAAACCTT

GTAGCTCTTCCAAGCAGCATCTGTAGGTTGAAAAGTTTGGTTCGTCTAAATGTGTGGGGT

TGCCCAAAACTGGAAAGCTTGCCAGAAGAGATAGGGGATTTAGACAACTTGGAGGAGCTT

GATGCCAAATGTACTCTAATTTCACGACCTCCGTCTTCCATCGTACGCTTGAACAAACTT

AAAATCTTGAGCTTTAGCAGTTTCGGATATGATGGAGTGCACTTTGAGTTCCCTCCGGTG

GCTGAAGGATTACACTCTTTGGAACATCTGGATCTCAGTTACTGCAATCTAATAGATGGA

GGACTTCCGGAAGATATTGGATCCCTATCCTCTTTGAAAGAATTGTGTCTCGATGGAAAT

AATTTTGAGCATTTGCCTCGAAGCATAGCCCAACTTGGCGCTCTTCAAATCTTGGACTTA

TCAGATTGCAAGAGGCTTACACAGTTGCCAGAACTCCACCCTGGATTAAATGTATTGCAT

GTAGATTGTCATATGGCTCTAAAATTTTTTCGTGATTTGGTAACAAAGAGAAAGAAACTA

CAGAGGGTGGGACTTGATGATGCACACAATGATTCTATATATAATTTATTTGCACATGCC

CTGTTTCAGAATATCTCTTCCTTGAGGCATGACATCTTTGCTTCAGATTCCTTGTCCGAA

AGTGTGTTTTCCATTGTGCATCCTTGGAAGAAGATCCCAAGTTGGTTCCACCATCAGGGA

AGGGATAGTAGTGTATCAGCCAATTTGCCTAAAAATTGGTATATACCTGATAAATTCTTG

GGATTTGCTGTATGTTACTCTGGCAGATTAATTGACTCCACAGCTGAATTGATTTCCGTA

TGTGATGACGTGATATCGTGGATGACCCAGAAACTTGCCTTATCAAACCATTCAGAATGG

GATACAGAATCTAACATTCATTTTTTCTTGGTACCTCTTGCTGTCTTATGGGATACATCT

AAGGCAAATGGAAAAACACCAAATGACTATGGACTTATTAGGTTATTTTTTTCTGGAGAA

GTGAAGAAGTATGGACTTCGTTTGTTGTATAAAGAAGATCCTGAGGTTGAGGCCTTGTTA

CAAATGAGGAAAAATAACAATGAACCAATAGAACATTCCACTAGGATAAGGAGGATCCGA

TATAACAATAGTGAACACGACTTCATGATCAATGAAGCCAGTTGCTCCTCGGGTAAGAAA

CAAAAGTCACATTTCTAA

>NTTN90_mRNA_98067_cds mRNA_98067 gene_55408|id=AT5G17680.1:evalue=0.0:annot='disease resistance protein (TIR-NBS-LRR class), putative';id=Solyc02g082050.2.1:evalue=0.0:annot='Tir-nbs-lrr, resistance protein'

ATGAGCACAGAAGACAGTAATGTGTCTTCTTCTCAGACGACGACAGGTACAGAAACTAGT

TGCCGTCAATGGTCATACGACGTTTTCTTGAGTTTTAGAGGTGAAGATACTCGAAAGAGT

TTTGTTGATCACCTCTACACCACTTTGCATGAAAAAGGGATTCATGCATTTCGAGACGAC

ATAGAGTTACGGAGGGGGAAATTCATTTCCCCTGAACTCCGTAACGCTATTGAAAAGTCT

AGGTTTGCAGTTGTCATTTTCTCACAAAACTATGCCAATTCCTCCTGGTGTTTGGAGGAA

TTGACGAAGATTGTTGATTGCACTAAGCATAGAGGGCAAACGCTGATGCCTGTCTTCTAC

AGCGTGGATCCTTCAGTAGTGAGAAAACAGAAGGGGAGTTATCAAGAAGCTTTTGCAGAA

CATGAAAAGAATTTCGAGGAAAAGAAGATCAAAGAATGGAGGGATGCTCTGAAGGAAGCA

GCTAACATCTCTGGATATGACGTCCAACACATGGAAGATGGGCATGAGTCAAGATGCATT

AGACAAATTGCAGTGGCAGTTTTGAACAAGTTGGGCCATGTCCGACCTAAAATTGCAGAA

AATCTAGTCGGAATCGAGCCCCAAGTACAAAACTTAATATCTCTGCTGAATACAAATTCT

GAGACTGATGTTCGCATAATTGGGATATGGGGTATGGGAGGCATCGGCAAATCAACCATT

GCACGAGCTGTTTTTGATCAACTTCAAGAAATGTTTGAAGGTGGCTGCTTTCTTGATAAT

GTTAGAGAAGCTGCATCCAAATTTGGACTTCAAGCTTTGGCAGAAAAATTACTTTCAGAA

ACATTAAAAGAGACCAAAGACAATCTTTACACCAACACCAACTTACTGATGAACAGATTG

AGCTATAAAAAAGTGATGATCGTTCTCGACGACGTGGATCACGATGAGCAGATAGAAAAC

TTGATAGCTGGAGGACACAAAGGGTTCGGGCCTGGTAGTAGAATAATTATCACTACAAGA

AACAAGCAATTGCTAGCGGCTTGTGGAGTGGACCAAGTGTATGAAGTTAGCCTATTAGGA

ACTAATGAGGCTTTGATGCTCTTCAACAGGTTCGCCTTCAAGGAAGCTCAACCGCAGGAT

CATTTCATGGAACTAGCGTTACGAGTGGTGAAATGTGCTTGGGGACTCCCGTTGGCTCTC

AAGGTTTTGGGATGTTTTTTGCACAAAAGAGAAAAAGAAGAATGGGAAAGCGAGTTGACG

AGGTTGGAAGGTATTCCTCATGATGATGTGATAGGGAAACTTAAATTGAGCATTGATGCA

TTGAATGATTTAGACAAGCAAATATTGCTTGATATTGCTTGTTTCTTTAAGGGGAAACGA

AGAGAACCTGTGATCAAGAAATTCCATGCATTCGGTTTCAAACCTGAAATTGGAATACCA

GTGCTTGTTCAAAGATCTTTGTTATCTATATCTGATGATGACAGATTTCAGATGCATGAT

TTAGTTCAAGAAACCGCTTGGTACATGGTTCGCCAGGGACAAACTAAAGAGAAATACAGC

AGGTTGTGGATTCCTGACGACATATGTGATGTTATGTCAAAGAAATCGGGTACAGAAGCG

ATTCAGTCAATAATATTGACTTACCCACAAAAAGAGAAATTGAACTTATCATCACGGGCA

TTAAAAGGTATGGAAAATCTACGTCTGCTCAAAATCCGCAATGCCTACTTCAATAGAGGT

CCAAGTTATCTTCCGAATGAGTTACAATGGCTCAATTGGCACAAGTTCCCCTCAACCTCT

CTTCCACAAGACTTTGACGGAGAAAAGCTTGTCGGACTCAAGTTAAGTCGTGGCCAAATT

TCACAACTATGGCCAGAACCTAAGAACCTTGAGCAGTTAAAGTATTTGAATCTCAGCTAC

TCCAATGGGCTAACTACCTCTCCAGATTTCAGCATGATGCCAAATCTTGAAAAGTTGAAT

CTTAGCAACTGTAAATGCTTGGTAGTAGTTCATGAGTCAATTGGAACACTTGAAAGGCTT

AAATACTTGAATATGTCTCACTGCTCGAAGCTGAGTCGTCTTCCGAATACCATTCACCTT

GAATCTTTGGAAACTTTTCTTCTGTGGGACTGCACCAATCTTCAAAATTTTCCTCAAGTT

ATTGGCTTAATGCCAAATCTTTCAGAACTTCACTTGGAAGGGACTGCTATTAAAGAGCTA

CCCGACTCCCTCATAAATATCAGTGGCCTTGTGTCCATAAACCTCAGCAATTGCAAAAGC

CTGGAAAATATAACCTACAGCATCTGCGGTTTGAGATGTCTCAGGAGTCTTAATTTCTCT

GGCTGTTCAAAACTCGAGTCATTGCCCAAAACTCTTGGCCAATTGGAAACTTTGGAAGAA

GTCCTTGTGGATGGAACTGCAATTACCAAGCTACCATCAACTATCTCCAAAATGGGAAAC

TTGAAAATCCTTTCTTTCAGTGGATGCAAGAATATTAAGGAAAATGATACGTCATTTGGA

GTGGCAAGTTTAAGCATGTTCACTTCAATGACAAATATCAGGAATCTTATGAACCGATCA

GATGCTGAGAGAAAGAAGCCACAAGCAGCACGGCCATCCTTATCTGGTTTGCGTTACTTG

AAGAAATTAGACCTCAGTGACTCTGATTTGGTAGATGAAGTTGCTGCTGATATTTGGCAC

TTGGCCTCATTAGAGGAGTTAAATTTGAGCCGAAATAATTTTGTGCAATTTCCTTCAAGA

ATATCTGGACTCCCAGGATTCAAAGTCCTGAAATTGGAAGAATGCAAGAACCTTGAAGTA

CTACCTGATCTTCCATTGAGTATTACTGTAATAGAGGCAAACGAGTGCCCGGCTCTGCAC

ACTCTTGGAAATCTGTCAACCCAACATGCATTCTTAAGGAAGGTTTCCTTTTCCAATTGT

CACAAATTGCACGAACAAAGCAAGAAAACTGGCATTTGTGCTGCGGATTTGTTGTTGGAA

CTGCTGCTTCAGGGGCACTCCATCATATATGGTCGCTTCAGTATACTGATTGCTGGAGGA

AAAATTCCCGTGTGGTTTGATCATCAGAAAATAGGCGGTTCTATCTCAGTCCAGCTACCT

TCAGATTGGCAAGTTAACATTGTGGGAATTGCAGTGTGTTTCGTTTTGGACAGCTTTATT

CCAAAGTCAAGACTAGGTGTTACTTTCAAGTTGGTTAGCCCAGACCACAGAGAATACACT

TTTCAAAATGCACCTTCTGCTGCTTCAAAGATGGGAGAAGTGTACGATTCTGATCACGTG

TGGATAACTTATATCTCTTTCAATCTATTTCGGCTCCTCTTCCCTGATTTCACAACTGAA

GATTGGACTAAAGTTTGCGGTAATCTTTCAATCAGAATAAGGCAGGATCCGTGGACAAAG

GTAAGGAGGTGTGGAATTCAGCTTGTTTACAAACAAGATTTGAGTACATTGGCAGCTGAA

CGCGCGGGGGTTAATAAAGATTCAGTTGGGAGCAAAGAATTAGTGGTATATGAAGGAGGA

GGTAAAGAAAGTAAAGAAGAAGCTGCGATCAAAGAGGATATTGCTGCACTTATGGCTGGG

GTTACTGAATTGAATTGGGATGTTGACCCCATTGAGCAAGATACCACTCAGCTCATGAAT

TTGAGGAAATCAATTGCCTACAAAATTCAAAAGACTCTCTCTTTTGAATGCTAG

>NTBX_mRNA_61045_cds NTBX_mRNA_61045 gene_35651|id=AT5G36930.1

ATGGCCACTGAACTGAAGTCTCAAGTGTACTTGAGTTTCAAAGCGAAAGACACCGGCAAA

ATTTTTGCAGATCACCTCTATGAAGCTCTGGTGGGAGCAGGTTTTGTAACATTAAGAAGC

TGTGGTGATGAAAATGAGGGAGGTGAAGATATCAAGTTCAATTTGCAAAAGGGTATTAAA

GAATCTGGGGTTTCAGTTATAATCTTCTCAAATGATTACGTGTCCTCAAGTTGGTGTCTT

GATGAGTTGGTAATGATCTTGGATTGTAAAAAGATAGCAAAACGTGCAGTTCTGCCCATA

TTTTACCACGTGGATCCTTCTGATGTTAGGAAACAGAAGGGAAGAATTGGAGAAGCATTT

GATATGGACAAAGAACTGGGAGGGAATCAAGGTGAAAATGAGAGGGTCAGAAAATGGAGG

GAAGCACTCAAAGAAGTTGCAGACTTGGGAGGAATGGTCTTACAAAACCAAGCTGATGGA

CACGAGTCCAAATTCATCCAGAAGATTCTTAAAGTGGTTGAGAATAAACTGAGCAGGCCA

GTCCTGTATATTTGCCCTCATCTGATTGGAATAGAACGGCGTGTTGAAAAGATCAACTTG

TGGCTAGAGGATGGATCTATTGATGTTGACACTCTTGTTATTTGTGGCATCGGTGGAATA

GGCAAGACAACAATGGCAAAGTTTGTGTATAATTTGAACTTCAGTAAGTTTGATGGTAGC

AGCTTTTTGTCCAACATTAGAGAAAATTCAACACACCGTAAAGGTTTAGTTACTCTTCAA

AGGCAATTTCTTTCTGATATTTGCAAAAGAAAGAAGAAAGCTATGTTTTCCGTGGACGAG

GGAATGACTGAGATGAGAGAGGCTGTACAGTGTAAAAGAATCCTTCTTGTTCTTGATGAT

GTAGATAATCGTGATCAAGTGGATGCTCTACTGGGAATGAAGGACTTGTTATATCCTGGT

AGTAAAGTCATTGTGACAACTAGGAACAAGAGATTGCTTAGGCCTTTTGATGTGCATAAG

ATTTATGAGTTTGAAGCATTGAATAGAGATGAATCGGTTGAGCTCTTAAGTTGGCATGCA

TTTGGTCAAGATTGTCCTATTAAAGGTTTTGAAATGTGTTCAGAACAAGTAGCAATCCAT

TGTGGAGGACTTCCATTAGCACTTGAAGTTCTTGGTGCTACTTTGGCAGGAAGAAACATA

GACATTTGGAAAAGTACAATACAGAAATTGGAAACAATTCCGAATCATCAAATTCTCAGG

AAATTAACAATAAGTTACGAATCTCTTGAGGATGATCATGATAAGAATTTATTTCTCCAC

CTAGCTTGCTTTTTCATTGGGAAGGACAGAGATCTAGCAGTAACTATTCTCAATAGGTGC

AACTTTTACACTGTAATTGGAATTGAGAATCTCATTGACAGAAATTTTATAAAAGTTGGT

AAGTCTAACAGCTTGATTATGCATCAAATGATTCGAGATATGGGAAGAGACATTGTTCGC

CAAGAATCACCACTGGAGCCTGGGAAACGCTCTAGACTATGGCGTTCAAAGGATTCCTTT

AACGTCTTAATCCAGAACCGTGCCACTCAAACAATTCAAGGCATTATTCTTGACATGGAT

ATGCTCAAGGAAAGTGACATAGTTAGCTCAAGCTTTTTCGCCAAGGATTTCAAGAAACAC

AAAATAAAAAACTTTCTCAACTATCCTAATCCTCAGAGAGTTCAATTCAAACAGAAAAGG

TTTGTTTTTTTCCCATGGCATTTGTCAGATGCCAAAGAAGCCACAAATGAGCTGGTTCTG

GGAACTGATGTATTTGCAAATATGCAAAAGTTAAAACTGCTCCAATTCGATCACGTTGAG

CTTCAAGGATCTTTTGATGTTTTTCCTAAGAGATTAAGATGGTTGCGCTGGTCTGAGCTG

CAACTTGAGTGCATGCCAATTGATTTTCCTCTGGAGAGCCTTGTAGTGATTGAATTACAC

CGTAGCAGCTTGAGGAGGATTTGGCATGGAGTCAAGTTCCTTAAAGATCTGAAGATTTTC

GATCTCAGCCATTCCTACGAGCTTCTAAGAACACCTGATTTTTCAGGACTCCCCAATCTT

GAAAAGTTGATCCTTCGATATTGTACAAGCTTGATTGAGCTTCATGAGACCATCGGGTGT

CTAGAATCACTTATTCTTTTGAATCTCAAAAATTGCAAAAATCTCCAGAGACTTCCAGAT

AGCATTTGCATGCTAAAATGTCTGGTGACACTAAATATCTCTGGTTGCTTGAATCTTGAA

TATGTGCCGATGGATCTAGATAAAATGGATTCACTGAGAGAGCTTTATGCTGATGAAATT

GCAGTTCACCAAATGATTTCTACTCCAGAAGAGGTCCAACCGTGGTATGGATTTCTGCGG

TCCTGGATGCTGAAGGGGAAAATATGTCCTAAAGTTTCACATATTAGTTTACCTAATTCC

TTGGTTACTCTGAGTCTTGCTAACTGTAATCTATCCAATGATGCTTTTCCAGTTGCTTTC

AGTAGCCTCTCCTTATTGCAAAACTTAGATTTGAGCGAAAATCCAATTTGCTGCCTACCA

AAGGGCATAATTTATCTCACCGGTCTTCAGAAGCTTGAAGTGGAAGGCTGTGAAAAGCTC

AGATCGCTCGTAGGGCTTCCCAATGTAGAACATCTCAATGTTACTAATTGCTGGTCGTTA

GAGAAAATATCATATCAATCAAGATCATCTAGACTGAAGGATTTACTTGTGTCGAATTGT

GCTAAATTAGTTGAAATAGATGGAAATTTCAAGTTAGAGCCCTTAAGAAATACTGAGGCA

GAGATGCTTTGCAAGTTGGGCTTGTCGAACTTAGCTTCTATGGATAATGTCATGATCAAT

CTTACATCTAATATCCTGAGTTACTACCGAATACATGGTAAAGGATGGACTCCAACAAGG

AAGACAAAGAAAGTTGTTCTTCAGGTATGTCTTGCTCGTTCTTTCATTGTTTCAGTTTTG

TGA

>NTTN90_mRNA_105967_cds mRNA_105967 gene_59970|id=AT5G36930.1:evalue=9e-04:annot='Disease resistance protein (TIR-NBS-LRR class) family';id=Solyc09g092410.2.1:evalue=0.0:annot='Tir-nbs-lrr, resistance protein'

ATGGAAGAGTCAAGAATTTCCATTATTGTTTTCTCAAGAAATTATGCTTCCTCTAGTTGG

TGTCTAAATGAACTAGTTAAAATTCTCGAATGCAAAGAGAAATTAAAGCAGATGGTTTTG

CCTATTTTCTATGATGTTGATCCTTCTGAGGTACGAAAGCAAACTGGGTTATTTGGGAAA

GCTTTGGCTAAACATAAGGAACGATCATTTGGAGCTCAAAGGGTGGAGAAATGGAGAGCT

GCACTTACTGAAGCTGCAAATTTATCTGGATGGGATTTGCAAAATGTTGCTGACGGAGTT

GGTGGCATAAGGAAAACAACTCTGGCAGAAGCTATCTATAATCGAATGTTTCGACTCTTC

GATAGCAGTTGCTTCCTTTCAGATGTTAGATCAGAAGTTGAAGAATTTGGTCTTGTCAAG

CTACAAGAGAAACTTCTTCAACAAGTTCTCAAAATCGAGGACATCAAAGTTGGCAGTGTT

GCTCAAGGCGTTAATCTAATCAAAGCAAGACTTGGGTCAAAGAAGGTTCTAATTGTTCTT

GATGATGTGGACCATAAAAGACAGTTAGAAGCCTTAACAAGAGAAAGAAGTTGGTTTGGT

TCAGGTAGTTTAATAATCATTACCACCCGTGACGAGCGATTGCTATGTCGGATTGCAGAA

AAAGAGAGATATGAGGCCAAACTATTAAATGGCAATGAAGCTATGTTACTTTTTTGTTGG

CATGCTTTTGACAGTGATTTTCCACCACAAGATTATGTTAATTTGGCACACGACATAATC

GAATATTCAGGTAGGCTGCCTTTAGCTCTTGTGACATTGGGGTCACATTTACAAGGAAGT

TCTGTAGAAGAATGGGGATATGAATTAGAAAAACTAAGAGCAATTCCTCATTGTGATATC

CAAAAGATTCTCAAGATAAGCTTTGATGGGCTTGATGGAGAAACACAGATTGTTTTCCTC

GATATTGCATGCGCCTTCCATGGGTTTGATGAGCATGAAGTTACTGAAATATTAAATGCA

TGTGGTTTTCATTCTAAAATTGCAATTGCAACTTTAGTCCAAAAACACTTGCTCCAAAGA

ACTCCGTATCATTTGGTGATGCATGATCTAGTGCGAGATATGGGAAGAGAAATCGTTCGC

TTGGAATCACCTCGAGACCCCGGAAAACGAAGTAGATTGTTCATCCCTCGAGAAGTTCGT

GATGTTCTACAAGGAAATGAAGGTTCCGAAAATGTAGAAGTACTGAAGGTAGATCGAGGG

ACATTAAACGGAGTGAACTTGAGCACCAAAGCATTTGAACAAATGAAAAACCTTAGGGTT

CTTATAATGGATGAGTTACATATTAGTGGAGATTTTGGGTTGTTGTCCAAGAAGCTCAGA

TGGTTGTCTTGGAAAAAATGTCCTTTAAAATGTATACCATCAAATCTTCCAGCTGAGAAT

CTTGTAGTTCTATATATGCGGGAGAGTGATATCCAAGAATTTCAATTGAATTTGCAGTGT

TGTAAAAGTTTGAAGGAGCTGAATCTCTCTCATTGCAAGCAACTGAGAAGCACTCCAAAC

TTCAATGGTTCACTGAGTCTTGAGACTTTGCATCTCTATGGTTGCTCAAGTCTGACGGAG

ATCCATCCATCAATAGGAAATTTGTTCAGACTAAGTTATCTATATATAGACGGTTGCAAA

AGACTTACGGATCTTCCAAGCAGCATATGCCAGCTAATATCCGTTGATTACTTGAGCATT

AGTAACTGCTCATCAATAAAAACACTGCCAGATAACGTTGGAGATATGAAAAGTCTAAGA

TGTCTTGATGCATCTGATACTGGTATAAAACAATTGCCTAGATCCGTTGAAATGCTAAGA

AATCTTGAAAGATTGGATGTGGGAGGTCGAAATTTAGAGGCCAAAAGGAGTATTTATAGA

AGAGGAGTCCATCGGATACAATACTCCTTGCCAACTTTTGTATACGATTTGAGCCTTACA

TACTGTAATTTGTCCGAGGCTGATATTCCTAGGAATATTGGGAGCTTATCCTCCTTAAAA

TATATAGATTTGAGCGGCAAGAGTTTCCATTGTCTACCCATTGATTTTTCTAAGTTACGA

TTGTTGGAGAAGTTAAGTTTGAAGGACTGTGAGAATCTCCAAACACTCCTGTCAGTATCA

AATTTAGAGAATCTTTATACAATTAAACTTGAGAATTGCCGAAAATTGGTCAAGATTATA

GAGTTGGACAACCTCCCTTCTATAGAGCAGATTAATATGATTAATTGTAATTCTCTGCAG

AATCCATTCAATGAAGGCTTCTTTAGTGCACCTGCTCTATATGCATCTAGAAATGATCGA

TATATGGTTAGTCTCTCTCTTTCTCTCTCTCCCCCCCATCATATTAGTGATCTTGAGTCA

TTGTCTGATGCAGATGCAGGCTACTGTTAG

>NTTN90_mRNA_42249_cds mRNA_42249 gene_23647|id=AT5G36930.2:evalue=1e-176:annot='Disease resistance protein (TIR-NBS-LRR class) family';id=Solyc09g092410.2.1:evalue=0.0:annot='Tir-nbs-lrr, resistance protein'

ATGAATACTCAATTAGTTCGAGGAGAATCATCTCCATCTTCTCACTTCTCTTATGAAGTA

TTCCTGAGCTTCAGAGGTGAAGACACCCGAAAAACATTCACTGGTCATCTTTATTCCAAA

TTGTCTGATGTTGGAATTAATATCTTCATTGACGATGAGGAATTGAGAAAGGGTGACGTG

ATTTCAAGAGAACTAGAGAAAGCAATTGAAGGATCAAGAATTTCCATTGTAGTTTTCTCA

AGAAATTATGCTTCCTCCAGTTGGTGTCTAAATGAACTAGTTAAGATTCTTGAATGCAAA

GAGAAATTAAAGCAGATGGTTTTGCCTATTTTCTATGATGTTGATCCTTCTCAAGTGCGA

AAGCAAACTGGGTTATTTGGGGAAGCTTTGGCAAAACATAAGGAACGATCAATTGGGGCT

CAAATGGTGCAGAAATGGACAGCTGCACTTACTGAAGCTGCAAATTTATCTGGATGGGAT

TTGCAAAATGTTGCTGACGGGCATGAATCAAAGTTTATTGAAAAAATTATACAGCAAGTC

CTACAAGAGGTCAACCAGACACCTCTAGATGTTGCTTGGCACCCAGTTGGAATAGATTCT

CGTGTCAAAGATGTAGAGTTGTTATTGCAAAATGAATGTGTAGATAAAGTTCGCATGATT

GGTATTCACGGAGTTGGTGGCATAGGGAAAACAACTCTGGCAAAAGCTATCTACAATCGA

ATGTTTCGACTCTTCGATAGTAGTTGCTTCCTTTCAGATGTTAGATCAGAAGCTGAAGAA

TTTGGTCTTGTCAAGCTACAAGAGAAACTTCTTCGACAAATTCTCAAAATTGAGGACATC

AAAGTTGGCAGTGTTGCTCAAGGCATCAATCTAATCAAAGCAAGACTCGAGTCAAAGAAG

GTTCTAATTGTTCTTGATGATATGGACCACAAAAATCAATTAGAATCCTTAACAAGAGAA

AGAAGTTGGTTTGGTTCGGGTAGCTTAATAATCATTACCACCCGGGACAAGCGATTGCTA

TGTCGGCTTGGAGAAAAAGAGAGATATGAGGCCAAACTATTAAATGACAATGAAGCGATG

TTAGTTTTTTGTTGGCATGCTTTTGACAGTCATTTTCCACCAGAAGATTATGTTAATTTG

GCACGAGACATAATCAAATATTCAGGTAGGCTACCATTAGCTCTTGTGACATTGGGGTCA

CATTTACAAGGAAGTTCTGTAGACGAATGGGGACATGAATTCGAAAAACTAATAGCGATT

CCTCATTCTGATATCCAAAAGATTCTCAAGATAAGCTTTGATGGGCTTGATGGTGAAACA

CAGACTGTTTTCCTCGATATCGCATGCGCCTTCCATGGGTTTGATGAGCATGAAGTTACT

GAAATATTAAATGCATGTGGCTTTCATGCTAAAAGTGCAATTGCAACTTTAGTCCAAAAA

CACTTGCTCCAAAGAATTGGGTATCATTTGGTGATGCATGATCTAGTGCGAGATATGGGA

AGAGAAATCGTTCGTATGGAATCATCTCGAGACCCTGGAAAACGGAGTAGATTGTTCATC

CCTCAAGAAGTTCGTGATGTTCTACAAGGAAATGAAGGTTCCGAAAATGTAGAAGTGCTG

AAGGTAGATCGAGGGACATTAAAGGGAGTGAACTTGAGCACCAAAGCATTTGAGCAAATG

AAAAACCTTAGGGTTCTTATAATGGATGAGTTACATATTAGTGGAGATTTTGGGTTGTTG

TCCAAGAAGCTCAGATGGTTGTCTTGGAAAAAATGTCCTTTAAAATATATACCATCAAAT

TTTCCAGCTGACAAACTTGTAGTTCTAGATATGCGGGAGAGTGATATCCAAGAGTTTGGT

TTGAATACGCAGTGTTGTAGAAGTTTGAAGGAGCTGAATCTCTCCCATTGCAAGCAACTC

AGAAGCACTCCAAACTTCAATGGTTCACTGAGTCTTGAGACTTTGTATCTCCATGGTTGC

TCAAGTCTGACTGAGATCCATCCATCAATAGGAAATTTGTCCAGACTAATTGAACTATCT

ATGTCTGGTTGCGAAAAACTTACGGATCTTCCAAGCAGCATATGCCAGCTAATATCCGTT

AATTACTTGAGCATTAGTAACTGCTCATTTATAAAAACACTGCCAGATGACCTTGGAGAT

ATGAAAAGTCTAAGATCTCTTTATGCATCTCGTACGGGTATAAAACAATTTCCTAGATCT

GTTGAAATGCTAAGAAATCTTGTAACTTTGATAGTGGGAGGTCAAAAGTTAGAGGCCAAA

AGGAGTATTTCTGGAAGAGGAGTCCATCAGATACAATATTCCTTGCCAACTTTTGTATGC

GATTTGAGCCTTACATACTGTAATTTGTCCGAGGCTGATATTCCTAGGGATATTGGGAGC

TTGTCCTCCTTAAAATATTTGGATTTGAGTGGCAACAGTTTCTATTGTCTACCCTTTGAT

TTTTCTAAGTTACGATTGTTGGAGAAGTTGTGTTTGAATGACTGTGAGAATCTTCAAACA

CTCCCGTCAGTATCAAATTTAGAGAATCTTTATAAAATTAACCTTTATAATTGCCAAAAA

TTGGTCAAGATTACAGAGTTGGACAACCTCCCTTCTATAGAGCGGATTAATATGATTAAT

TGTAGTTCTCTGCAGAATCCATTCAATGAAGGCTTCTTTAGTGCACCTGCTCTATTTGCA

TTTAGTGCACCTGCTCGATATATGATGCAGGATACTATTAGAATTTATCTCGAATGTAAT

GAGATTCCAGAATGGTGCAGGAATCAAGTAACAGCTTCATCTATGTGTTTGACTATGCCA

ACACATAATAACTTCTTAGGAATGGTTCTCTGGTTTGTTTCCGACTTGTTCGATGTAGTC

CCGCATAATCCATGCTTCAGGATTAGTATTGCACATGGAAAGCCTTCAAATATTCGGTGG

AGAAAAGGGAAAAAACCTCTGTCATGTGTATATTACATATCTTACTTACATAAAGCTTTA

GATGGCCAGATGATCAAAGGCGGGGAAATGATAGAAGTGTGGGCTGACAACATTACAATA

AAGAAGATAGGGATCCATCTGTTATATTTAGACCAACATGGTAAAGTTGTATCTTTGCTG

GGAGACGTGGATCATTCTTATTCTAAGTACCCAAAAAGAGTTTCAACTCCTCCTTGTAAA

AGAGCAACGTTCTGA

>NTTN90_mRNA_26498_cds mRNA_26498 gene_14861|id=AT4G12010.1:evalue=0.0:annot='Disease resistance protein (TIR-NBS-LRR class) family';id=Solyc04g056570.2.1:evalue=0.0:annot='Tir-nbs-lrr, resistance protein'

ATGGATTCCCAAAAGGAATTGAACAAAAAATGGGAATATGATGCCTTCTTGAGCTTTAGA

GGTGAAGATACCCGCAACAACTTTGTGGCCCATCTCCACAAACGTTTGGAAGAGAGAGGA

GTCAACGTATTCAAAGACGATGAGAAACTTGAAAGAGGAAGACCCATTTCAGCTGAACTA

TTGAAAGCCATTGAAGAATCAAGAGTTGCCATAATCATATTCTCCAAAAATTATGCTTCA

TCGGTATGGTGTTTGGAGGAACTCACCAAACTTATGGAGTGTGTTGAAAAGAAGGGACAG

GAAGCTATCCCTATATTCTACAATGTTGACCCATCAGATGTACGCATGCAAAGATCCAAA

AGTAGTTTCGCAAAAGCGATGGCCAAACACAAGGCTAATTTCGAGGGTAATGATTTGGAG

AAGGTGCAGAGGTGGACTGACGCTCTCCAAAAAGCAGCCAATATAGCAGGATGGGATCTT

GGTAAATGTGCTAACGGGAATGAAGCAGAATGCATTGATCGGATTGTACACGAGAAATTT

CAAAATGTGCACCACACAGTTTCAGCAACTGAGAAGTATTTAGTGGGAGTCGAATCTCGT

ACTGGTGGAGTGGAATCAATGTTGAAAGTTGGATCAGGAGGTGTTTATTTTGTGGGAATA

TGGGGAATGGGTGGCGTGGGGAAGACAACGGTCGCAAGAAAAATCTTTGACAATATTTCT

AATCAGTTTCAAGGGTCTTGTTTTCTTGCAAATGTTAGAGAAGAATCAAAGAAGCATGGG

ATAAAACACTTGCAGAAGACACTTCTTTCAAGAATCTTAAATGAAAAATCTTTGAAGGTA

GCAAGTTTTTATGAAGGAGCTGACATGTTAAAAGGAAAGTTTTGTCTCAGGAAGGTTTTA

ATTGTTTTTGATGATGTGGATGACAACCACCAGTTGGAGTATTTAGTTGGAAAGCATGAT

TGGTTTGGTGATGGCAGTAGAATTGTTACAACAACCCGAAATGCAGATTTACTTCGTTGC

CACGACGAGTTATATTCTGTCCCTGAATTGGCGAAATGCGAGGCTCTTGAACTTTTTAGT

TGGCATGCCTTTCAGAAGAGGACTCCAGATAAAGAGTTTTTAAAACTCTCTAAGTCTGTA

GTAGATTATGCTAAAGGTTTACCCTTAGCTCTTACGGTATTGGGTTCTTTTCTCTACAAA

CGAGGCATAACTGAGTGGGGAAGCGCATTGGATAGACTCAAAGATACTGGATATGAAGAA

ATTGTTAAGCAGCTCAGCTTAAGTGTAGATGGATTGTCCTATGAAGACAAGAATATATTT

CTTGATATTGCATGCTTCTTTAGAGGAAAAAGGAGAGATTTTGTGATAACAATACTAAAT

AGTTTTGGCTTCAAATCAGAGATTGGAATAGATGTCCTTACAAAGAAATCACTTCTTTAT

ATTTCAGAAGGAATGGTTGAGATGCATGATTTGATTGAACAAATGGGTCAGCAATTGGCA

CGTGATGTTGATCAGGACAAACCGTGGAACCATAGTAGAATATGGCATGAAAACGATATA

GAAACTGTTTTTTCTGCAAATCGGTGGACAGAGTCAGTAAAAGGCATAATGGTACCAATT

GGCTCAGACCGACATATATGCAAGTGGAGCAAAGCTTTCAGAAATATGCCTTGTCTTAGG

TTACTCATGGTCAAAGGGGAGGAGGTCCGACATTATGAACAAGTTTCTGACACTATTGAA

TATCTCCCCAGTAGCTTGAAATGGCTTGATTGGTCTTACTACAGTTTTGAATCTTTACCA

ACAAATTTTCAACCAAGAAACCTGGTTGGGCTCAACATGACTTTTAGTTCTCTTGTTGAA

ATTTGCAAGGAACCAAAGGCATTTGTCAAGTTGACGATTCTCAATTTAAGTTTTTCAGAG

AATTTACTCCGAACACCCAATTTTTCTGAGATTCCAAACCTGCAGAGGATAATACTGAAA

AGTTGTTTAAGCTTGAAAGATGTTCATCCATCCATTGGCAATCTCAAAAAGCTTGTTTCT

CTGAACATGAAGAACTGTAAAAATCTCAAGTTTTTACCAAGCAGTATTCAAATGGAATCT

CTTGAATGCCTCAACCTCTCTGGTTGTGAGAAATTAGATACTCTTCCAGAAATTCGGGGG

AATATGGAATTGCTATCAGAGCTTCTTTTGGGACGTACTGCAATTCGGGAACTACCCTCA

TCAATAGGACGGCTCTTTGGTATTAGTTTGCTTGATTTGCATTCATGTGAAAATCTTGTA

AGACTCCCAGCCAGTGTTAGCGAGATGAGAAAACTGAAAGTTTTAATTCTTAAAGGCTGC

TTAAAACTGGCAACTTTTCCAGAAAGCCTGGGTGATCTAGAAGAATTGGAGGAGCTCTAT

GCTGGAAACACTGCCGTTTGGCGACTACCAGATTCTATGGAAAACTTAAGCAAACTTAAA

ATCCTATCATTAAAAGGTAGACGAGAGATGAAGTGTCAATCTGCTACAGGTTTGATATTC

CCTTGTGCATTTCATGGTTTGAGGGCATTGAAAAGTTTGGATCTCAGTGGATGCAATTTA

TCTGGTGACGAGATTAATGGTCTTACGTGCTTGACTTCTCTATTGGAACTAAACCTTAGC

AGAAACAAGTTTATTTCTCTACCTGATGGCATCAGTCAACTTTATCAACTTCGATATCTC

AACATAACACACTGTCATGAACTTAAGAAACTTCCCAAACTTCCTCCAAGTATAAAGGAA

TTGTATTCAGAAGATTTTCTGGCCAAACAAATTATTCTAGCTATGTCGGTATACCGAGGG

TTGTGTTTGGCCTCATTCACCAACTATAGTTTTGATCAACAACCATACACAGAGAAGAGC

GATGATAACTCAGTGTTGGATGAGATTCTGAGCTTGTTTCTTTCAGACAGCATGGATGAT

AAGATGACTTATTGTATTATCTTTCCTGAACGTGCTATCCCCACATGGTTTAAACATCAG

AGTACTGAGGAAAAGATCTTGCTTAAACTGCCTGAGGATTGGTATGATGATAGATTTGAG

GGCTTTGCTATATGCTGTGTCACTTGCATGGGGGCAGGTGTCCACGATCCTGATTCAGGG

CTATCAGGGAAGTACGACTATACTTTCATCAAAGCCAAATTGATATGCAATAATCATATG

GAAGAGCTTGAAGTGCTGGAGAAAGAGTGTAAAGTCAGTACAACGTCTAGAACTTATAGC

TGGTGTGTTTGCTTTGCCTACATACCATTGTATTCTTTGCTGCAGACTTCTGGCACACAA

GTTCTAAACTTTAACCAGTATGGCCTATTCGAGGCATCTATCCAAAGGCACATTACGAGA

CAATGGGGAGTTCATTTGATTTACAAGTCTGAAAGACAATTTTTCGAGAGCAGAACTGAG

AAAGGGTTGCTTGCCCAGAAGTTGAGTCTAGTCAAGCCAATTCGAGTCAAAACGAGAATT

GTTCGATTAGAATTACCTAAATAG

>NTK326_mRNA_109102_cds NTK326_mRNA_109102 gene_64483|id=AT5G17680.1

ATGGCATCTTCTTCTGCTTCGGCGAGTATTTCACAGTTTTCTCGGTGGAACTACAAAGTC

TTTCTAAGTTTTAGAGGTGAAGATACTCGGAGAACATTTACAGGTCACCTCTTCAAAAGC

TTAGAAAATAGTGGAATATTCACGTTTCAAGATGATAAAAGGCTAGATCATGGCACATCA

ATATCACATGAACTCTTGAAAGCTATCGAACAGTCTCAAGTTGCCCTCGTCATTTTCTCA

AAGTATTATGCAACATCGAGGTGGTGCTTAGATGAGCTAGTGAAGATCATGGAATGCAAG

GATCAATGTGGACAGACTGTCATACCAGTCTTCTATGGTGTGGATCCATCACATGTTAGG

AACCAGAGGGACAACTTTGCTGAAGCCTTTGACAAACATGAAACAAGCTATAAGGATGAT

GATGAAGGAATGCAAAAGCTCCAAAGATGGAGGAATGCACTAACTGCTGCCGCAGATCTA

AAAGGATATGATATCCGTGACGGGATTGAAGCAGAGAATATTCAGCAGATTGTCGACCAA

ATTTCCAAATTGTGCAATAGTGCTACTTTGTCTTCTTTGCGAGATGTTGTGGGAATAGAT

ACTCATTTGGAGAAATTAAAGTCCCTACTTAAGGTAGGAATCAATGATGTTCGGATCATA

TTGGGGATCTGGGGCATGGGTGGTGTAGGGAAGACGACAATAGCAAGAGCCATTTTTGAC

ACTTTATCTCATCAATTTGAAGCTGCTTGTTTCCTTACTGATATTAAAGAAAATGAAAAA

AGACATCAACTGCATTCTTTGCAAAACACCCTTCTCTCTGAATTGTTAAGAAGAAAAGAT

GATTACGTCAATAATAAGCATGATGGGAAGCGAATGATTCCAGACAAACTTCGCTCTAAG

AAGGTGCTAATTGTGCTTGATGATTTAGATCATAAAGATCATTTAGAATATTTAGCAGAT

GATATTGGTTGGTTTGGTAATGGCAGTAGAGTTGTTGTAACAACTAGAAACAAGCATTTG

ATAGGGAAGAATGATGTCATTTATGAAGTGACTGGACTAGCTCACTGTGAAGCTATGCAA

TTGTTCAGTCAACATGCTTTTAGAAAAGAAGATCCAGATGAGTGTTTTAAGGAGCTCTCA

TTGGAGGTAGTAAATTATGCTAAAGGCCTTCCTTTAGCCCTCAAAGTGTGGGGTTCTTTG

CTGCATAACCTAGGCTTAACTGAATGGAAAAGTGCAATAGAGCAAATGAAAATTAATTCT

AATTCGGAAATTGTTGAAAAGCTCAAAATCAGTTATGATGGATTGGAGCCCATCCAACAA

GAGATGTTTCTAGATATAGCATGCTTCTTTCGAGGGAAAGAAAAAGATTACGCCATGAAA

ATTCTTGAGAGTTGTCATTCTGGAGTTGAATACGGATTGCGTGTCTTAATTAACAAATCT

CTTGTGTCCATCTCTGAAAATGATGAGATTCAAATGCATGACCTTATAGAAGATATGGGT

AAATATATAGTGAATTTTCAAAAATATCCAGGAGAATGTAGCAGATTATGGCTCGTCGAG

GAAGTTGAAGAAGTGATGAACAACAATACAGGGACCACGAAAATGGAAGTAATCTGGTTT

CCTTATTACCATTCTGGTACATTACGCTTTAGCAAAAAGGCTATGAAAAATATGAAAAGG

CTTAGGATATTTAACATAGGGAGTTCATCGACCCGTGATGCCATTGAGTATTTGCCCAAC

AGCTTGCGTTGTTTTGTGTGTCATGACTATTCTTGGAAGTCATTGCCAGAAAATTTTGAA

CCCAAAATGCTTGTTCACCTTCAACTCTCGTACAGTTCACTGCATTATTTATGGATGGAA

ACAAAGCATTTGCGGTCTCTACGGAGGATAGATCTCAGCTACTCTAGAAGACTGATGCGA

ACACCAGATTTCACGGGGATGCCAAATTTGGAGTATTTGGATCTGACTTGGTGTTCTAAT

CTTAAAGAGGTTCACCATTCCATGGGATGTTGCAGCAAACTCATTCGGTTAAATTTGAGG

TATTGTGTAAGCCTTAAGAGGTTTTCATGTGTTAACGTGGAATCTCTTGAATATCTGAAT

CTAAATAAGTGCTATAGTTTAGAGAAATTCCCAGAAATCCACGGGAGAATGAAGCCGGAG

ATACAAATTCACATGCAACGCTCTGGGATAAGGGAACTACCATCATCTATTTTTCAGTAT

CAAACTCATATTACCAAGCTAGATTTGAGCTGTATGAATAACCTTGTAGCTCTTCCAAGC

AGCATTTGTAGGTTGAAAAGTTTGGTTAGTCTAAATGTGTTGGGGTGCTTCAATATGGAA

AGCTTGCCAGAAGAGATAGGGGATTTAGACAACTTGGAGGAGCTTGATGCCGGTGATAGT

ACTCTAATTTCACGACCTCCATCTTCCATCGTACGCTTGAACAAACTTAAAATCTTGAAC

TTTAGCGGCTTTAGATACAATGGAGTGCACTTTGAGTTCCCTCCGGTGGCTGAAGGATTA

CGGTCATTGGAACATCTGAATCTTAGTTACTGCAATCTAACAGATGGAGGACTTCCGGAA

GATATTGGATCCTTATCCTCTTTGAAAGAATTGTATCTCGGTGGAAATAATTTTAAGCAT

TTGCCTCGAAGCATAGCCCAATTTGGTGCTCTTCGAATCTTGGACTTAAGAAATTGCAAG

AGGCTTACACAGCTGCCACAACTT

>NTTN90_mRNA_17802_cds mRNA_17802 gene_9991|id=AT5G17680.1:evalue=1e-10:annot='disease resistance protein (TIR-NBS-LRR class), putative';id=Solyc11g011090.1.1:evalue=0.021:annot='Tir-nbs-lrr, resistance protein'

ATGGCATCATCTTCTGCTTCTGCGAGTACTTCACAATTTCCTTGGTGGAACTACAAAGTC

TTTCTAAGTTTTAAAGGTGAAGATACTCGAAAAACATTTATAGGTCACCTCTTCAAAGGC

TTGGAAAACAGTGGAATATTCACATTTCAAGATGATAAAAGGCTAGAGCATGGCACTTCA

ATATCAGATGAACTCTTGAAAGCTATCGAACAGTCTCAAGTTGCCCTCGTCGTTTTCTCA

AAAAATTATGCGACATCGAGGTGGTGCTTAGATGAGCTAGTGAAGATCATGGAATGCAAG

GATCAATGCGGACAAACTGTCATGCCAGTCTTCTATGATGTGGATCCATCACATGTTCGG

AACCAGAGGGAGAGCTTTGCTGAAGCCTTTAACAAACATGAAACAAACTATAAGGATGAT

GATGAAGGAATGGAGAAGCTCCAAAGATGGAGGAACGCTCTAACTGCTGCCGCAGATCTA

AAAGGATATGATATTCGTGACGGGATTGAAGCAGAGAATATTCAGCAGATTGTCGACCAA

ATTTCCAAATTGTGTAATAGTGCTAATTTGTCTTCTTTGCGAGAAGTTGTGGGAATAGAT

ACTCATCTGGAGAAATTAAAGTCTCTACTTAAGGTAGGAATCAATGATGTTCGAATCATA

TTGGGGATCTGGGGCATGGGCGGTCTAGGGAAGACGACAATAGCAAGAGCCATTTTTGGC

ATTTTATCTCATCAATTTGAAGCTGCTTGTTTCCTTGCGGATATTAAAGAAAATGAAAAA

CTACATTCTTTGCAAAACACCCTTCTCTCTGAATTGTTAAGAAAAAAAGATGATTATGTC

AATAATAAGCATGATGGGAAGCAAATGATTCCAGACAGACTTCGCTTTAAGAAGGTGCTA

ATTGTGCTCGATGATATAGATCATAAAGACCATTTAGATTATTTAGCGGGTGATCTTGGT

TGGTTTGGTAATGGCAGTAGGGTTGTTGTAACAACTAGAGACAAGCATTTGATAGGGAAG

GATGATATAATATACGAAGTGACTGCACTACCTGATCATGAATCCATTCGATTGTTCTAT

CAGCATGCTTTCAAAAAAGAGGTTCCAGATGAGTGTTTTAAGGAGCTTTCATTGAAGGTA

GTAAATCATGCTAAAGGCCTTCCTTTAGCCCTCAAAGTATGGGGTTCAATGCTGCATAAC

CTACGAATAACTGAATGGAAAAGTGCTATAGAGCACATGAAAAATAATTCTAATTCTGGA

ATTGTTGATAAGCTCAAAATTAGTTATGATGGATTAGAGCTCATCCAACAAGAGATGTTT

CTAGATATAGCATGCTTCTTGAGAGGGAAATATAAAGATTACGTCATGCAAATTCTTGAG

AGTTGTCATTCTGGAGTTGAATACGGATTGCGTGTCTTAATTGACAAATCTCTTATATTT

ATCTCTGAAAAATGTCAGATTCAAATGCACGACTTAATACAAGAAATGGGTAAATATATA

GTGAACTTGCCAAAGAATCCGGGAGAGCGCAGCAGACTATGGCTCGTCAAGGATTTCAAA

GAAGTGATGAGCAACAGTACAGGGACCATGGCAATGGAAGCAATCTTTCTTCCTTATCTC

AATTCTGGTACATCACGCTTTAGCAAAAATATGAAAAGGCTTAGGATATTTAACATAGAG

AGGTCGTTGAACTGTGATGGTTCCATTAAGTATCTGCCCAACAGCTTGCGTTGGTTTGTG

TGGGAGAACTTTCCTTGGAAGTCATTGCCATCTACATTTGAACCCAAAATGCTTGTTCAC

CTTGAACTCTGGGGTAGTTCACTGCATTATTTATGGATGGAAACAAAGCATTTGCCATCT

CTACGGAGGATAGATCTCAGCTCCTCTAGAAGACTGAGGCGAACACCAGATTTCACGGGG

ATGCCAAATTTGGAGTATTTGAATATGTTATATTGCAGAAATCTTGAAGAGGTTCACCAT

TCCCTGAGATGTTGCAGCAAACTCATTCGGTTAAATTTGAATAATTGTAAAAGCCTTAAG

AGGTTTCCATGTGTTAACGTGGAATCTCTTGAATATCTGAGTTTAGAATATTGCTCAAGT

TTAGAGAAATTTCCAGAAATCCACGGGAGAATGAAGCCGGAGATACAGATTCACATGCAA

GGCTCTGGGATAAGGGAACTACCATCATCTATTACTCAGTACCAAACTCATATTACCAAG

CTAGATTTGAGAGGTATGGAAAAACTTGTAGCTCTTCCAAGCAGCATCTGTAGGTTGAAA

AGTTTGGTTAGTCTGAGTGTGTCGGGTTGCTTCAAACTTGAAAGCTTGCCAGAAGAGGTA

GGGGATTTAGAAAACTTGGAGGAGCTTGATGCCAGTTGTACTCTAATTTCACGACCTCCG

TCTTCCATCGTTCGCTTGAGCAAACTTAAAATCTTTGATTTTGGAAGCTCCAAAGATAGA

GTGCACTTTGAGCTCCCTCCGGTGGCAGAAGGATTTCGCTCATTGGAAACTTTGAGTCTT

AGAAACTGCAATCTAATAGATGGAGGACTTCCGGAAGATATGGGATCCCTATCCTCTTTG

AAAAAGTTGTATCTTAGTGGAAATAATTTTGAGCATTTGCCTCGAAGCATAGCCCAACTT

GGTGCCCTTCGAATCTTGGAATTAAGAAATTGCAAGAGGCTTACACAGCTGCCAGAATTT

ACGGGGATGCTAAATTTGGAGTATTTGGATCTGGAGGGATGTAGTTATCTTGAAGAGGTT

CACCATTCCCTGGGGTGTTGCAAAAAACTCATTCGGTTAAATTTGAGTTTTTGTAGTAGC

CTTATAAGGTTTCCATGTGTTAACATGGAATCTCTTAAATATCTGAGTGTAGGAGAGTGC

TCTCGCTTAGAGAAATTCCCAGATATCCATGGGAGAATGAAGCCGGAGATACAGATTCAC

ATGAAACGCTCTGGGATAAGGGAACTACCATCATCTATTTCTCAGTACCAAACTCATATT

ACCGAGCTAGATTTGAGAAGGATGGATAACCTAGATTCCAGAGGCTACTAG

>NTK326_mRNA_107125_cds NTK326_mRNA_107125 gene_63314|id=AT5G36930.2

ATGACTCTGATCTCCAGCTGTTGGGACTTGTCACTTGTTGTGTCTCAGGTGTGCACTGCG

AAACAATTTTGCAGAGATTCTTACAATTATATTTACGCAAGCTCCTTTACCTTTATCTCC

ATTAGTACGTCCATGGAGAAACCTCTGGATCCAACACCTTCGTCTTCATCTTATGGGTGT

TCCTCAAATCAAGTATTCTTGAGTTTTAGAGCAGAAGACTCTTGTTGCATGTCTTTCACT

GATCACCTTTACACTGCCTTAGTTCAAGCTGGGTTTCAAACATTCAAACAGGGCACTGAC

ACAAGAAAAGAACAACTCCACAACGCAATCCGAGAGTCGAAAGTTTCGCTGATTGTCCTG

TCCGAAGGCTATGCCTTTTCTCAATCATGTCTTGATCAGCTCGATGTGATTCTGACATGT

AAGGAGAAATTGGATCGGGCAATTCTACCCGTCTTCTACTATGTGGATCCTTCTGATGTT

AGGAACAAGAAGGGAAGGATTGGGGAAGCATTAGCATTGCATGAACAAGAATTGAAATGG

GAAAGCAGTGGAAGGGAAAGGGTAGAAAGATGGAGGCAGGCACTTGCTAAAGTTGCTGAC

TTGGGGGGAATGGTCTTACACAATCAAGCTCGTGGGCATGAGTCGAAATTTATTCGAAAG

ATTGTTAATGTGGTCACAAATAGACTAAGTCGGACAGCTTTGTATGTTGCACCTTACCTA

ATCGGCATAGATCGCCGGGCTAAGCATATTAGCTTTTGGCTGCAAAATGGATCGGCTGAC

GTTGGCATATTGATTGTTTGTGGCATGGGCGGTATAGGGAAGACTACTCTGGCCAAGTTC

ATCTACAATTCAAACTTTCATGCTTTTGAAGGTAGCAGCTTTGTGCTTAACATAAGAGAA

ATTTCAAAGCAACCTAACGGTTTAGTTAAATTGCAAAAGCAAATACTTTCTGACATTCTA

AAAAGGACAAAGGAACGAGTATCATGTGTTGATGAAGGAATTGTTAAGATTTCAGATGCC

TTAAGTGGCAAAAGAGTCCTACTTGTTCTTGATGATGTGGATGATTCTGATCAACTAAAT

GCAGTATTGGGGATGAAAAGTTTATTTTTCCCAGGAAGTAAAATCATCATAACAACCCGA

CATGAGCGAATTCTACATCCTCACCTAGTTGATAAGGTGTATACCGTTGAGACACTGAGC

ACGGACGAATCCTTAGAGCTCTTCAGTTGGCATGCCTTTGGAAAACCACATCCTGCGGAA

GGTTTTCTTGTGGGTTCAAATGAAGTAGTGAAACGGTGTGGAGGAATTCCACTTGCACTA

AGAGTTTTGGGTTCTTCCCTGGCAGGACAAAATTTAGATGTATGGCAAAGTACAATAAAG

AAGTTGCAAGTTGTTCCTAATAATCGGATTATTGAACTGCTCAAAATCAGTTATGAATCT

CTAGAAGATGATGATAGGAGTTTATTCCTTCATATTGCATGTTTCTTTCTCTGGGAGGAT

AAAGATTTTGCTGTCAAAATACTGGATAAATGTGAACTTTTCACTATAGTGGGGATCCAA

AACCTCATTGATAGAGATCTCTTGTCAATACTAAATGGTAGGCTCTTCATGCATCAACTG

ATTCAAGATCTGGGGAGAGAAATTGTTCGTCAAGAATCTGTTAAGGAGCCAGGGAGGCGT

AGTAGACTGTGGCGTCATGAAGAATCTTTGTACGTATTGAGAAACAAAACGGGAACTGAA

GCAATTGAAGGCATCATTCTCGACGGAAATATGTGTAAGGGGCATGGATCAACCAGGATA

ACATCTAATGAAAATTATGGCAAAAAAAGTAAAGTGGAAGAATTTATGAATAATTCTCAG

GAAGATCGACCAAAGCAGAATTGGATGTCCATTTTCTCTCGTCATATCATGGGCACAAGG

GAAGTTCCAAATGAGGATTTGGAAACTGACTCATTCACAAACATGCTCAAGTTGAAATTT

CTGTTGCTTAGCAATATACAACTTTCTGGATGTTACAGGAAATTTCCCAAGAAATTAAGA

TGGTTGTTTTGGCGTTATCTCCAGTTAGAATCCCTACCAAGCGACTTTCCGATGGGAAAA

CTTGTTGCTATAGACCTTTGTTACAGCAGCTTGAAACAACTTTGGACAGCACCAAAGTTA

CTCAGATGGTTAAAGTTTCTCAATCTCAGCCACTCTTATCAGCTTAGTAGAACTCCTGAT

TTTTCATTACTTCCCAACCTTGAACAATTAATCCTCGAATATTGTACAAGTCTAACTGAG

GTGGATGACACTATTGGATATCTGGAAGGACTCACTGTTTTAAGCCTTAATGGTTGTATA

AACTTGAGGAGCATTTCAGAAAGTATTTGCATGTTAACACATCTTGAGACCCTTGATATT

TCTGGTTGCTCAAATCTTGAATATGTCGCCCTGAAGCTTGAAAAGTCGGATTTTCCAAGT

GAGCTTTCAGATGAAAGTGGAAGAAACCAAATAGATAATACCAAGCTAGTAAGACCATGG

CATACAATCTTGTGGTCTTTGCTGAGGAAGGAGAAAGTATGTCATAGAGTTTCACCAATC

AGTTTTCCGACTTCTCTAGTTACTCTAAGACTTTCTGACTGCAATCTGGGTGATAATGCA

TTTCTCCATGTTGATTTTAGTAAGCTCAATTTGCTGAAAGAATTGAGTTTGAGCCGAAAT

CCACTTTGCCATCCCCCAGAGAGCATTAGATATCTTAGCAGGCTCGAAAACCTTTCACTA

AATTCATGTACAAGGCTAAAATCAGTACTCGAGCTGCCAAATGGTGTTGAGATTGTTGAT

GCAACTGACTGTATATCCTTGGAGAAAGTATCAGGCGCACCTAGCTCATGCAGCATTCTT

TACATAAATTGTGCTAATCTGGTTGAGATGAACGCTAATTTCAAGTTAGAACATCTTGAA

AATGTCAATGCAGAAACTCTCGGCTATTTGGGTCTGTCAAACTTGGAGTTGATTAGAAAT

GTTACTTTTAGATTAAGATTTGACATTCAGAAATTGCATGCTGATGAAATGGAATTTCCG

GAATTTGTCCAAAATGACATGGCAAAATTACAATCTCTCCCACCTAAGAAGCTTCCCGCT

CAGGGATATTACTGCAATGGCGTCTTCTCCACATTCCTATCGGGTGAACACGTGCCAAGT

TGCTTTGACACGAAGTTGAGTGAGCCTTTCTGTTCATCGTTCATCGTGCCTACTCCTGAT

AATCATAGAATTCGAGGCTTGAGTTTTTGCTTGGTGTATACATGCTTGGAGAGTGAAGAA

ATGGTAAGTGAGGGCCACTGCATGTCCATTACAATTAATAATTTGTCGCAAAGGATCAAG

TGGAAGCAAGACCCTATGTTTCTAGCTATTCCTGAAGTTGAAGAGCGGATGATGTGGTTA

AGTTATTGGGAAATTGGCAACTCGTTGCAAACAGGTGATGTTGTAGAGATTTCAGCCAGT

GTTGTAGATAAACAACGTTTCAGTATCAACGAGGTTGGAATGAGGATTCTGTTCCTGGAA

GAACAACAAGACCAGGACAAAGAATCTAATTGTGAAGTTGAAGAGTTTTTTTCTAGTCCA

TGTCATCAGAATTTGCTCCTTGTCAGAGTCTTCCCGAAGTGA

>NTBX_mRNA_78055_cds NTBX_mRNA_78055 gene_45674|id=AT5G17680.1

ATGGCATCATCTTTTGCTTCTGCGAGTACTTCACAGTTTCCTCGATGGAACTACAAAGTC

TTTCTAAGTTTTAGAGGTGAAGATACTCGAAAAACATTTACAGGTCACCTCTTCAAAGGG

TTGGAAAACAATGGAATATTTACGTTTCAAGATGATAAAAGGCTAGAGCATGGCGCATCA

ATATCAGATGAACTCTTGAAAGCTATCGAACAGTCTCCAGTTGCCCTCGTCGTTTTCTCA

AAGAATTATGCAACATCGAGGTGGTGCTTAGATGAGTTAGTAAAGATCATGGAATGCAAG

GATCAATGTGGACAGACTGTCATACCAGTCTTCTATGATGTGGATCCATCACATGTTCGA

AACCAGAGAGAGAGCTTTGCCGAAGCCTTTGACAAACACGAACCAAGATATAGGGATGAT

GATGAAGGAAGGCAGAAGCTCCAAAGATGGAGGAATGCTCTAACTGCTGCCGCAAATCTA

AAAGGATATGATGTCCGTGACGGGATTGAAGCAGAGAATATTCAGCAGATTGTCGACCAA

ATTTCCAAATTGTGCAATAGTGCTACTTTGTCTTCTTTGCGAGATGTTGTAGGAATAGAT

ACTCATTTGGATAAATTAAAGTCCCTACTTAAGGTAGGAATCAATGATGTTCGGATCATA

TTGGGGATCTGGGGCATGGGTGGACTAGGGAAGACGACGATAGCAAGAGTCATTTTTGAC

ATTTTATCTCATCAATTTGAAGCTGCTTGTTTCCTTGCGGATATAAAAGAAAATGAAAAA

AGACATCAACTGCATTCTTTGCAAAACACCCTTCTCTCTGAATTGTCAAGAAGAAAAGAT

GATTACGTCAATAATAAGCATGATGGGAAGCGGATGATTCCAGACAGACTTTTCTCTAAG

AAGGTGCTAATTGTGCTTGATGATATAGATCATAAAGATCATTTAGAGTATTTAGCAGGT

GATATTGGTTGGTTTGGTAATGGTAGTAGAGTTGTTGTAACAACTAGAAACAAACATTTG

ATAGAAAAGAATGATGTCATTTATGAAATGACTGCACTATCTGATCATGAATCCATTCAA

TTGTTCTGTCAACATGCTTTCAGAAAAGAAGATCCAGATGAGCATTTTAAGAAGCTTTCA

TTGGAGGTAGTAAAATATGCCAATGGCCTTCCTTTAGCCCTCAAAGTGTGGGGTTCTCTG

CTGCATAACCTAGGCTTAACTGAATGGAAAAGTGCAATAGAGCAAATGAAAATTAATTCT

AATTCGGAAATTGTTGATAAGCTCAAAATCAGTTATGATGGATTAGAGCCCATACAACAG

GAGATGTTTCTAGATATAGCATGCTTCTTACGAGGGGAACAAAAAGCTTACATCCTACAA

ATTCTTGAGAGCTGTCATATTGGAGCTGAATATGGATTGCGTATTTTAATTGACAAATCT

CTTGTGTTCATCACTGAAGATTATCAGATTATTCAAATGCATGACTTAATTCAAGATATG

GGTAAATATATCGTGAACTTGCAAAAGAATCCGGGAGAACGCAGCAGACTATGGCTCAAC

GAGGATTTCGAAGAAGTGATGACCAACAATGCAGGGACCGTGGCAGTGGAAGCAATTTGG

GTTCATGATTTGGATACACTACGCTTTAACAATGAGGCCATGAAAAATATGAAAAAGCTT

AGGATATTATACATAGACAGAGAGGTCTATGATTTCAATATTAGCGATGAACCCATTGAG

TATCTATCCAACAACTTGCGTTGGTTTAACGTGGATGGCTATCCTTGTGAGTCATTGCCA

TCTACATTTGAACCCAAAATGCTTGTTCACCTTGAACTCTCATTTAGTTCACTGCGTTAT

TTATGGATGGAAACAAAGCATTTGCCGTCTCTACGGACGATAAATCTCACGGGCTCTGAA

AGCCTGATGCGAACACCAGATTTCACGGGGATGCCAAATTTGGAGTATTTGGATATGTCT

TTCTGTTTTAATCTTGAAGAGGTTCACCATTCCTTGGGATGTTGCAGCAAACTCATTGGG

TTAGATTTGACCGATTGTAAAAGCCTTAAGAGGTTTCCATGTGTTAACGTGGAATCTCTT

GAATATCTGGATTTACCAGGTTGCTCAAGTTTAGAGAAATTTCCAGAAATCCGCGGGAGA

ATGAAGCTGGAGATACAGATTCACATGAGATCTGGGATAAGGGAACTACCATCATCTAGT

TTTCACTACCAGACTCGTATTACCTGGCTAGATTTGAGCGATATGGAAAACCTTGTAGTT

TTTCCAAGCAGCATCTGTCGGTTGATAAGTTTGGTTCAATTATTTGTGTCTGGTTGCTCA

AAACTGGAAAGCTTGCCAGAAGAGATAGGGGATTTAGACAACTTGGAGGTGCTTTATGCC

AGTGATACTCTAATTTCACGACCTCCATCTTCCATCGTACGCTTGAACAAACTTAACAGC

TTGAGCTTTAGGTGCTCCGGAGACAATGGAGTGCACTTTGAGTTCCCTCCAGTGGCTGAA

GGATTACTGTCATTGAAAAATCTAGATCTCAGTTATTGCAATCTAATAGATGGAGGACTT

CCGGAAGACATTGGATCCTTATCCTCTTTGAAAGAATTGGATCTCAGAGGAAATAATTTT

GAGCATTTGCCTCGAAGCATAGCCCAACTTGGTGCTCTTCGATCCTTAGGCTTATCATTT

TGCCAGACGCTTATACAACTGCCAGAACTTTCCCATGAATTAAATGAATTGCATGTAGAT

TGTCATATGGCTCTGAAATTTATCAATGATTTAGTAACAAAGAGGAAGAAACTACAGAGG

GTGGTATTCCCGCCACTGTATGATGATGCACACAATGATTCTATATATAATTTATTTGCA

CATGCCCTGTTTCAGAATATCTCTTCCTTGAGGCATGACATCTCTGTTTCAGATTCCTTG

TTCGAAAATGTGTTTACCATTTGGCATTATTGGAAGAAGATCCCAAGTTGGTTCCACCAT

AAGGGAACTGATAGTAGTGTATCAGTCGATTTGCCTGAAAATTGGTATATACCTGACAAA

TTCTTGGGTTTTGCTGTATGTTACGATGACATTTTAATTGACACCACAGCTCAATTGATT

CCCGTATGTGATGATGGGATGTCGTGCATGACCCAGAAACTTGCCTTATCAGAATGTGAT

ACAGAATCATCCGATGATTCAGAACGGTATACACCAATTCATTTTTTCTTTGTACCTCTT

GCTGTCTTATGGGATACATCTAAGGCAAATGGAAAAACACCAAATGACTATGGGATTATT

AGGCTATCTTTTTCTGGAGAAATGAAGAAGTATGGACTTCGTTTGTTGTATAAAGAAGAA

GCTGAGGTTGAGGCCTTGTTACAAATGAGGGAAAATAACAATGAACCAATAGAACATTCC

AATGTGATAAGGAGGAGCAGATCTGACAATAGTGAACACCATGACTCCGTGACCGATGAA

TCCAGTTTATGTTGCTGTCGCATACTGTAA

>NTTN90_mRNA_81517_cds mRNA_81517 gene_46122|id=AT5G17680.1:evalue=2e-05:annot='disease resistance protein (TIR-NBS-LRR class), putative';id=Solyc04g007320.1.1:evalue=4e-36:annot='Tir-nbs-lrr, resistance protein'

ATGATGCAGAAGAGTTCTTCTTGTTTCTCCTCTGCTCAGACGTTTCGGTGGAGTTATGAT

GTTTTCTTAAGTTTTAGAGGTGAAGATGTACGTAAAACTTTTGTTGACCATCTCTACGTT

GCTCTGCAGCAAAAGGGTATTCATACATTCAAAGATGATGAAAAACTGGAGAGAGGCAAG

TCCATTTCACCTGATCTTACGAGAGCAATTGAAGAGTCGCGTATAGCTTTGATCATCTTT

TCCAAAAACTATGCTAATTCAACATGGTGTTTAGATGAACTAATGAAGATCATGGAATGC

AACAAACAAAAAGGACAAATTGTCCTTCCGGTCTTCTACGATGTAGATCCATCAACAGTG

AGGAAACAAAAGTCAAGCTTTGGAGAAGCATTTAGCAGTCATGAAGCCCGTGGTTGTTTC

AAGTTGCAAAAATGGAGGGCAGCATTGGTGGAAGCTGCTAATTTATCTGGCTGGGATTTG

CCAAATACTGCCAATGCGCATGAAGCTAAAGTCATAAAGCAAATTGTGGAAGATATACTG

GCTAAATTGGGTGGTCAGAGGCATGCAATCAATGCTGAAAATCTTGTTGGAATGGAGTCA

CAAATGCAGAAAGTGTATAAAATGCTTGGCATCGGTTTTGGAGGAGTTCACTTCGTTGGA

ATATTTGGAATGAGCGGAGTGGGAAAGACAACTTTAGCGAGAGTCATTTATGATAACATT

TCAAGTCAATTTGAGGGTGCTTGTTTTCTTCATGAGGTTAGAGACCGTTCAGAAAAACAA

GGCCTAGCGCGATTGCAAGAGATACTTCTTTCCAAGATCCTTGTCATAAAAGATCTAAGG

ATCAACAATTTATTTGAAGGAGTTAATATGCATAGACATAGATTACGGTACAAAAAGGTT

CTTCTTGTTCTTGATGATGTTGATCACATAGATCAGTTAGAGGTTTTAGCTCAGAAGCGT

GAATGGTTTGGTTCTGGAAGTAGAATCATCATAACAACTAAAGACAAACACTTGCTTGTT

AAGCATGATGTGGAAAAGATATACAAAATGAGAACATTAAGTGACGATGAAAGTCTAGAA

CTATTTAAACAATATGCTTTCAAGAAGAACCATCCTACCAAGAAATTTGAGGATCTCTCA

GCTCAAGTGATAAAGTATACTGCTGGACTCCCCTTGGCTCTGAAGGTCCTGGGCAGTTTC

TTGTATGGAAGAGATTTGGCTGAATGGAGAAGTGAAGTGGAACGATTGAAACAAATCCCG

GAAGATGAAATTTTGAGGAAACTCGAACCAAGTTTCACTGGACTCAAAAGTATCGATCAA

AAGATATTCTTAGACATTGCGTGTTTCTTTACAGGGAAAAAGAAAGATTCAGTGACTAGA

GTTCTTGAGAGTTTTAATTTTAGCCCTATTATTGGCATAAAAGTTCTTATGGAGAAATCT

TTGATTACTATTTCAGAAGGTAGGATTTTAATGCACCAATTGATACAAGAAATGGGATGG

CACATTGTTCGTCGAGAAGCTTTCGATTATCCAAGAAGATATAGTAGGTTATGGAAGTCT

GAAGATATTTCTCATGTACTTGCAAGAAATATGGGCACAGAAAAGATCGAAGGCATATCT

CTGAACTTGAGAAAGATGCTCACAGATATTTCTCATGCACTTGAAAGAAATTTGGGCACA

GAGAAGATCAAAGGGATACCATTAAACTTGACCAATGTCAAAGAAGTGAATGTTAGTGCA

ACAGCCTTCATGCCGATGACCAGACTGAGGTTTCTCAAAATCAAGAATGCATATGTTTCT

CAGAGTCCTGATATTCTTCCTAGTGAGTTGAGCTGGCTTTCTTGGCACGGATATCCTTCA

AAAAGTCTTCCAATTAGCTTTCAGGGAGAACGACTCGTTAGTTTGAAGTTAAAAAATAGT

CGCATCATACAACTTTGGAAAGGCTCCAAGGTTCTAGGACAACTGAAGTACATCAACCTT

AGCCATTCACATAAGCTAATAAGGACTCCAGATTTTTCGGGTACCCCTAATCTTGAAAGG

TTGGTTCTTGAAGAGTGCACAAGTTTGGTAGAAATCAATTTTTCTGTTGGAGATCTCAAA

AAGCTAGTCTTGCTCAAGTTGAAGAACTGCATCAATTTAAAGACCCTGCCAAAGAGTATT

CAATTGGAAAATCTTGACGTTCTTATTCTATCAGGCTGCTCAAAGCTAAAAGTATTCCCA

GAAATAGAAGAGGAAATGAATCGTTTATCAGAACTATATTTGGAAGCGACTGCTTTTAGT

GAACTACCCGCATCAGTTGAGAAACTATCAGGAGTTAAAGTGATAAATCTAAGCTCATGC

AAGAATCTTGAGAGTCTTCCAAATAGTATTGTTAGGTTGAAATATCTTAAAGAACTTAAT

GTGTCCAAATGCTCAAAACTTAAAAGTTTACCAGATGACTTGGGTTCTTTAGTCAGTTTG

GAGGGGCTCCATTGTGATGACACACCGATCCAAATGATACCCTCCACCATTTCCCTTCTA

AAGAACCTTAAGCACTTATCTCTCCGTCAATGTAATGCTTTAGGTTTGCACGTAAGGAGT

TCAATCTCAAGAGAATCTATGGGACTAGTTTTCTCTAATTTATCAGGTCTTTGTTCATTG

ACAATGCTGGATATAGGTGGCTGCAGCATTTCAGATGGAGGCATCCTATGTAATCTTGGG

TTCTTACCATCTTTGGCGGAATTGAATCTTGGTGGTAACACATTTACTAATATCTCAGCT

TCAAGCATCAGTGGTCTGACTCGACTAAAGGTTCTTCAATTGGTTGGCTGTAGTAGGCTT

GAACATTTCCCAGAGCTTCCTCGAGCTATAGAAGAGGTGCATGCTGATGAGTGTATATCT

TTGAAGAGTATCCATCAATTAGCAAAATATCCAACATTGCGCCGACTTTCACTTAGCCAA

TGTCATCAGCTTCATGATACTGACATGGTTGATGCATTATGGAGCAACATGCTCAAGGGA

CTATACGTGCTACGAAATGATCTCAGCATTTGCATCCCTGGATCGCAGATTCCTATGTGG

TTTACATACAAGAACTTTGGGGAAAATGTTACACTGACTCTTGCCAATAATTGGTACACT

GATAACCTCTGGGGTTTTGCTTTCTGTATTGTTTTTGAACGTATGGAATGGTGCGGTCTA

TATGATGGTTACCTACAACCATCACTTGGATTTCCAGTTAACCTTAAATTCAAAACATAT

GATGGTAAGGAAGGCGATATACGTAGCATTATTGGCATAAAAGGAGGTGATATGTCAATT

CGGAACTCAGAGCACACTCTCCTTGCCTACGTACCATCTCGTCGTTTTCTGCAACCTTAC

AATAACGAGGTTTACAGTCCCAACGACTGGATAGAAATTGTGGCTTATTCTACAGTACAA

TTCGACAGCAAAGCTTGGGGAACGCGTCTTGTGTACTTGGACGATATTATTGAAGCATGA

>NTTN90_mRNA_35271_cds mRNA_35271 gene_19764|id=AT5G17680.1:evalue=7.9:annot='disease resistance protein (TIR-NBS-LRR class), putative';id=Solyc01g008800.1.1:evalue=0.025:annot='Tir-nbs-lrr, resistance protein'

ATGGCATCATCTTCTACTTTTGCGAGTACTTCACAGTTTCCTCGATGGAACTATGATGTC

TTTCTAAGCTTTAGAGGTGAAGATACTCGGAAAACATTTACGAGTCACCTGTACGAAATC

TTGGATATCAGGGGAATAAAAACCTTTCAAGATGATAAAAGGCTAGAGCATGGCGCATCC

ATTTCAGATGAACTCTGTAAAGCTATCGAAGAGTCTCAATGTGCTGTCATCATTTTCTCA

AAGAATTATGCAACATCGAGGTGGTGCTTGAATGAACTAGTGAAGATCATGAATTGCAAG

ACTCAATTTGGACAAACTGTAATACCAGTCTTCTATGATGTGGATCCATCATATGTTCGG

AACCAGAGGGAGAGCTTTGCTGAAGCATTTGCCAAACATGAAACAAAGTATATGGATGAT

GTCGAAGGAATACAAAGTTGGAGGATTGCTTTAACTGCAGCGGCCAATCTCAAAGGCTGT

GATATTCGTGACAAGACTGAATCAGACTGTATTCGACAGATTGTTGACCGAATCTCGTCC

AAATTATGCAAGATTTCTTTATCTTATTTGCAAAACATTGTTGGAATAGATACTCATTTA

AAGGAAATAGAATCCTTACTAGGGATAGGAATCAATGATGTTCGGATTGTGGGGATTTGG

GGCATGGGGGGAGTCGGTAAAACGACAATAGCTAGAGCTATGTTTGATACTCTCTTAGTA

AAAAGGGATAGTTCTTATCAATTTGATGGTGCTTGTTTCCTTGCGAATATTAAAGAAAAC

AAACGTGGAATGCATTCTCTGCAAAATATCCTTCTCTCTGAACTTTTAAAGGAAAAAGCT

AATAACAATAGTGAGGAGGACGGAAAGCACCAAATGGCTAGTAGGTTTCGTTCTAAGAAG

GTCCTAATTGTGCTTGATGACATAGATGATAAAGATCATTATTTGGAGTATTTAGCAGGT

CATCTTGATTGGTTTGGTAATGGCAGTAGAATTATTGTAACAACTAGAGACAAGCATTTG

ATAGGGAAGAATGATGTAATATATGAAGTGACTGCACTACCTGACCATGAATCCATTCAA

TTGTTCTATCAGCATGCTTTCAAAAAAGAGGTTCCAAATGAGCATTTTAAGGAGCTTTCA

TTGGAGGTAGTAAATTATGCTAAAGGCCTTCCTTTAGCCCTCAGAGTGTGGGGTTCTTTG

CTACATAACCTAGGCCTAACTGAATGGAAAAGTGCTATAGAGCACATGAAAAATTACTCT

AATTCTGGAATTGTTGATAAGCTCAAAATTAGTTATGATGGATTAGAGCCCAAACAACAA

GAGATGTTTCTAGATATAGCATGCTTCTTCCGAGGGGCAAAAAAAGAGTATGCCATGCAA

ATTCTTGAGAGTTGTCATTGTGCAGCTGAATACGGATTGCGTGTCTTAATTGACAAATCT

CTTGTGTCCATCATTGAAAATGATCGTATTCAAATGCATGACTTAATGCAAGATATGGGT

AAATATATAGTGAACTTGCAAAAGGATTCGGGGGAATGCAGCAGGCTATGGCTCGACGAG

GATTTTGAAGAAGTGATGATCAACAATAAGGGGACCACGAAAATGGAAGCAATCTGGTTT

CCTTATTACCATTGTGGTACATTACGCTTTAGCAAAGAGGCCATGAAAAATATGAAAAAG

CTTAGGATATTAAACATAGAGAGGTCGTGTACCTGTGATGGTTCTATTGAGTATCTGCCC

AACAGCTTGCGTTGGTTTGTGTGGAAATGCTATCCTTGGGAGTCATTGCCAGCTGAATTT

GAACCCAAAAAGCTTGTTCATCTTGCAGTCAAATCCAGTTCACTATGTTATTTATGGACG

GGAACAAAGCAATTGTCGTCTCTACGGACGCTAGATCTCAGATACTCTGAAAGCCTGGTG

CGAACGCCAGATTTCACGGGGATGCCAAATTTGGAGTATTTGAATCTGGAGGAATGTTGT

GATCTTGAAGAGGTTCACCATTCCTTGGGATTTTGCAGAAAACTCATTCGGTTAAATTTG

GAGTCTTGTGGACGCCTTAATTGGTTTCCATGTGTTAACGTGGAATCTCTTGAATATCTG

GATCTAGATTTTTGCTGTAGTTTAGAGAAATTTCCAGAAATCCATGGGAGAATGAAGCCG

GAGATACAGATTCACATGAAACGCTCTGGGATAAGGGAACTACCATCATCTATTATTCAG

TACCAAACTCATATTACCTTCCTAGATTTGAGCGCTATGAAAAACCTTGTAGCTCTTCCA

AGCAGCATCTGTAGGTTGAAAAGTTTGGTTAGTCTAAATGTGTCGGGCTGCACAAAACTT

GAAAGCTTGCCAGAAGAGATAGGGGATTTAGAAAACTTGGAGGAGTTTTATGCCANCTTT

ATGCAAGGTATGCTCTAA

>NTBX_mRNA_108280_cds NTBX_mRNA_108280 gene_64864|id=AT5G17680.1

ATGGCATCTTCTTCTGCTTCGGCGAGTATTTCACAGTTTTCTCGGTGGAACTACAAAGTC

TTTCTAAGTTTTAGAGGTGAAGATACTCGGAGAACATTTACAGGTCACCTCTTCAAAAGC

TTAGAAAATAGTGGAATATTCACGTTTCAAGATGATAAAAGGCTAGATCATGGCACATCA

ATATCACATGAACTCTTGAAAGCTATCGAACAGTCTCAAGTTGCCCTCGTCATTTTCTCA

AAGTATTATGCAACATCGAGGTGGTGCTTAGATGAGCTAGTGAAGATCATGGAATGCAAG

GATCAATGTGGACAGACTGTCATACCAGTCTTCTATGGTGTGGATCCATCACATGTTAGG

AACCAGAGGGACAACTTTGCTGAAGCCTTTGACAAACATGAAACAAGCTATAAGGATGAT

GATGAAGGAATGCAAAAGCTCCAAAGATGGAGGAATGCACTAACTGCTGCCGCAGATCTA

AAAGGATATGATATCCGTGACGGGATTGAAGCAGAGAATATTCAGCAGATTGTCGACCAA

ATTTCCAAATTGTGCAATAGTGCTACTTTGTCTTCTTTGCGAGATGTTGTGGGAATAGAT

ACTCATTTGGAGAAATTAAAGTCCCTACTTAAGGTAGGAATCAATGATGTTCGGATCATA

TTGGGGATCTGGGGCATGGGTGGTGTAGGGAAGACGACAATAGCAAGAGCCATTTTTGAC

ACTTTATCTCATCAATTTGAAGCTGCTTGTTTCCTTACTGATATTAAAGAAAATGAAAAA

AGACATCAACTGCATTCTTTGCAAAACACCCTTCTCTCTGAATTGTTAAGAAGAAAAGAT

GATTACGTCAATAATAAGCATGATGGGAAGCGAATGATTCCAGACAAACTTCGCTCTAAG

AAGGTGCTAATTGTGCTTGATGATTTAGATCATAAAGATCATTTAGAATATTTAGCAGAT

GATATTGGTTGGTTTGGTAATGGCAGTAGAGTTGTTGTAACAACTAGAAACAAGCATTTG

ATAGGGAAGAATGATGTCATTTATGAAGTGACTGGACTAGCTCACTGTGAAGCTATGCAA

TTGTTCAGTCAACATGCTTTTAGAAAAGAAGATCCAGATGAGTGTTTTAAGGAGCTCTCA

TTGGAGGTAGTAAATTATGCTAAAGGCCTTCCTTTAGCCCTCAAAGTGTGGGGTTCTTTG

CTGCATAACCTAGGCTTAACTGAATGGAAAAGTGCAATAGAGCAAATGAAAATTAATTCT

AATTCGGAAATTGTTGAAAAGCTCAAAATCAGTTATGATGGATTGGAGCCCATCCAACAA

GAGATGTTTCTAGATATAGCATGCTTCTTTCGAGGGAAAGAAAAAGATTACGCCATGAAA

ATTCTTGAGAGTTGTCATTCTGGAGTTGAATACGGATTGCGTGTCTTAATTAACAAATCT

CTTGTGTCCATCTCTGAAAATGATGAGATTCAAATGCATGACCTTATAGAAGATATGGGT

AAATATATAGTGAATTTTCAAAAATATCCAGGAGAATGTAGCAGATTATGGCTCGTCGAG

GAAGTTGAAGAAGTGATGAACAACAATACAGGGACCACGAAAATGGAAGTAATCTGGTTT

CCTTATTACCATTCTGGTACATTACGCTTTAGCAAAAAGGCTATGAAAAATATGAAAAGG

CTTAGGATATTTAACATAGGGAGTTCATCGACCCGTGATGCCATTGAGTATTTGCCCAAC

AGCTTGCGTTGTTTTGTGTGTCATGACTATTCTTGGAAGTCATTGCCAGAAAATTTTGAA

CCCAAAATGCTTGTTCACCTTCAACTCTCGTACAGTTCACTGCATTATTTATGGATGGAA

ACAAAGCATTTGCCGTCTCTACGGAGGATAGATCTCAGCTCCTCTAGAAGCCTGATGCGA

ACACCAGATTTCACGGGGATGCCAAATTTGGAGTATTTGAATATGTTATATTGTAGTAAT

CTTGAAGAGGTTCACCATTCCCTGAGATGTTGCAACAAACTCATTCGGTTAAATTTGAAT

TATTGTAAAAGCCTTAAGAGGTTTCCATGTGTTAACGTGGAATCTCTTGAATATCTGAGT

TTAGAATATTGCTCTAGGTTAGAGAAATTTCCAGAAATCCACGGGAGAATGAAGCCGGAG

ATACAGATTCACATGAAACACTCTGGGATAAGGGAACTACCATCATCTATTACTCAGTAC

CAAACTCATATTACCAAGCTAGATTTGAGCGGTATGGAAAAACTTGTAGCTCTTCCAAGC

AGCATCTGTAGGTTGAAAAGTTTGGTTAGTCTGAGTGTGTCGGGTTGCGTCAAACTTGAA

AGCTTGCCAGAAGAGATAGGGGATTTAGAAAACTTGGAGGAGCTTGATGCCAGCTGTACC

CTAATTTCACGACCTCCATCTTCCATCGTACGCTTAAGCAAACTTAAAATCTTTGATTTT

GGAAGCTCCAAAGATAGAGTGCTCTTTGAGTTCCCTCCGGTGGCAGAAGGATTTCGCTCA

TTGGAAACTTTGAGTCTCAGAAACTGCAATCTAATAGATGGAGGACTTCCGGAAGAGATT

GCATCCTTATCCTCTTTGAAAAAGTTGTATCTCAGTGGAAATAATTTTGAGCATTTGCCT

CGAGGCATAGCCCAACTTGGTGCTCTTCGAATCTTGGACTTAAGAAATTGCAAGAGGCTT

ACACAATTGCCAGAATTCACGGGGATGCCAAATTTGGAGTATTTGGATCTGGAGGGATGT

AGTTATCTTGAAGAGGTTCACCATTCCCTGGGGTGTTGCAAAAAACTCATTCGGTTAAAT

TTGAGTTTTTGTAGTCGCCTTATAAGGTTTCCATGTGTTAATGTGGAATCTCTTAAATAT

CTGAATGTAGGAGAGTGCTCTCGGTTAGGGAAATTTCCAGAAATCCACGGGAGATTGAAG

CCGGAGATACAGATTCACATGAAACGCTCTGGGATAAGGGAACTACCATCATCTATTTCT

CGGTACCAAACTCATATTACCGAGCTAGATTTGAGAAGTATGGATAACCTTTTAGATCTT

CCAAGCAGATCGGTAGGTTGA

>NTBX_mRNA_107641_cds NTBX_mRNA_107641 gene_64511|id=AT5G36930.2

ATGACTCTGATCTCCAGCTGTTGGGACTTGTCACTTGTTGTGTCTCAGGTGTGCACTGCG

AAACAATTTTGCAGAGATTCTTACAATTATATTTACGCAAGCTCCTTTACCTTTATCTCC

ATTAGTACGTCCATGGAGAAACCTCTGGATCCAACACCTTCGTCTTCATCTTATGGGTGT

TCCTCAAATCAAGTATTCTTGAGTTTTAGAGCAGAAGACTCTTGTTGCATGTCTTTCACT

GATCACCTTTACACTGCCTTAGTTCAAGCTGGGTTTCAAACATTCAAACAGGGCACTGAC

ACAAGAAAAGAACAACTCCACAACGCAATCCGAGAGTCGAAAGTTTCGCTGATTGTCCTG

TCCGAAGGCTATGCCTTTTCTCAATCATGTCTTGATCAGCTCGATGTGATTCTGACATGT

AAGGAGAAATTGGATCGGGCAATTCTACCCGTCTTCTACTATGTGGATCCTTCTGATGTT

AGGAACAAGAAGGGAAGGATTGGGGAAGCATTAGCATTGCATGAACAAGAATTGAAATGG

GAAAGCAGTGGAAGGGAAAGGGTAGAAAGATGGAGGCAGGCACTTGCTAAAGTTGCTGAC

TTGGGGGGAATGGTCTTACACAATCAAGCTCGTGGGCATGAGTCGAAATTTATTCGAAAG

ATTGTTAATGTGGTCACAAATAGACTAAGTCGGACAGCTTTGTATGTTGCACCTTACCTA

ATCGGCATAGATCGCCGGGCTAAGCATATTAGCTTTTGGCTGCAAAATGGATCGGCTGAC

GTTGGCATATTGATTGTTTGTGGCATGGGCGGTATAGGGAAGACTACTCTGGCCAAGTTC

ATCTACAATTCAAACTTTCATGCTTTTGAAGGTAGCAGCTTTGTGCTTAACATAAGAGAA

ATTTCAAAGCAACCTAACGGTTTAGTTAAATTGCAAAAGCAAATACTTTCTGACATTCTA

AAAAGGACAAAGGAACGAGTATCATGTGTTGATGAAGGAATTGTTAAGATTTCAGATGCC

TTAAGTGGCAAAAGAGTCCTACTTGTTCTTGATGATGTGGATGATTCTGATCAACTAAAT

GCAGTATTGGGGATGAAAAGTTTATTTTACCCAGGAAGTAAAATCATCATAACAACCCGA

CATGAGCGAATTCTACATCCTCACCTAGTTGATAAGGTGTATACCGTTGAGACACTGAGC

ACGGACGAATCCTTAGAGCTCTTCAGTTGGCATGCCTTTGGAAAACCACATCCTGCGGAA

GGTTTTCTTGTGGGTTCAAATGAAGTAGTGAAACGGTGTGGAGGAATTCCACTTGCACTA

AGAGTTTTGGGTTCTTCCCTGGCAGGACAAAATTTAGATGTATGGCAAAGTACAATAAAG

AAGTTGCAAGTTGTTCCTAATAATCGGATTATTGAACTGCTCAAAATCAGTTATGAATCT

CTAGAAGATGATGATAGGAGTTTATTCCTTCATATTGCATGTTTCTTTCTCTGGGAGGAT

AAAGATTTTGCTGTCAAAATACTGGATAAATGTGAACTTTTCACTATAGTGGGGATCCAA

AACCTCATTGATAGAGATCTCTTGTCAATACTAAATGGTAGGCTCTTCATGCATCAACTG

ATTCAAGATCTGGGGAGAGAAATTGTTCGTCAAGAATCTGTTAAGGAGCCAGGGAGGCGT

AGTAGACTGTGGCGTCATGAAGAATCTTTGTACGTATTGAGAAACAAAACGGGAACTGAA

GCAATTGAAGGCATCATTCTCGACGGAAATATGTGTAAGGGGCATGGATCAACCAGGATA

ACATCTAATGAAAATTATGGCAAAAAAAGTAAAGTGGAAGAATTTATGAATAATTCTCAG

GAAGATCGACCAAAGCAGAATTGGATGTCCATTTTCTCTCGTCATATCATGGGCACAAGG

GAAGTTCCAAATGAGGATTTGGAAACTGACTCATTCACAAACATGCTCAAGTTGAAATTT

CTGTTGCTTAGCAATATACAACTTTCTGGATGTTACAGGAAATTTCCCAAGAAATTAAGA

TGGTTGTTTTGGCGTTATCTCCAGTTAGAATCCCTACCAAGCGACTTTCCGATGGGAAAA

CTTGTTGCTATAGACCTTTGTTACAGCAGCTTGAAACAACTTTGGACAGCACCAAAGTTA

CTCAGATGGTTAAAGTTTCTCAATCTCAGCCACTCTTATCAGCTTAGTAGAACTCCTGAT

TTTTCATTACTTCCCAACCTTGAACAATTAATCCTCGAATATTGTACAAGTCTAACTGAG

GTGGATGACACTATTGGATATCTGGAAGGACTCACTGTTTTAAGCCTTAATGGTTGTATA

AACTTGAGGAGCATTTCAGAAAGTATTTGCATGTTAACACATCTTGAGACCCTTGATATT

TCTGGTTGCTCAAATCTTGAATATGTCGCCCTGAAGCTTGAAAAGTCGGATTTTCCAAGT

GAGCTTTCAGATGAAAGTGGAAGAAACCAAATAGATAATACCAAGCTAGTAAGACCATGG

CATACAATCTTGTGGTCTTTGCTGAGGAAGGAGAAAGTATGTCATAGAGTTTCACCAATC

AGTTTTCCGACTTCTCTAGTTACTCTAAGACTTTCTGACTGCAATCTGGGTGATAATGCA

TTTCTCCATGTTGATTTTAGTAAGCTCAATTTGCTGAAAGAATTGAGTTTGAGCCGAAAT

CCACTTTGCCATCCCCCAGAGAGCATTAGATATCTTAGCAGGCTCGAAAACCTTTCACTA

AATTCATGTACAAGGCTAAAATCAGTACTCGAGCTGCCAAATGGTGTTGAGATTGTTGAT

GCAACTGACTGTATATCCTTGGAGAAAGTATCAGGCGCACCTAGCTCATGCAGCATTCTT

TACATAAATTGTGCTAATCTGGTTGAGATGAACGCTAATTTCAAGTTAGAACATCTTGAA

AATGTCAATGCAGAAACTCTCGGCTATTTGGGTCTGTCAAACTTGGAGTTGATTAGAAAT

GTTACTTTTAGATTAAGATTTGACATTCAGAAATTGCATGCTGATGAAATGGAATTTCCG

GAATTTGTCCAAAATGACATGGCAAAATTACAATCTCTCCCACCTAAGAAGCTTCCCGCT

CAGGGATATTACTGCAATGGCGTCTTCTCCACATTCCTATCGGGTGAACACGTGCCAAGT

TGCTTTGACACGAAGTTGAGTGAGCCTTTCTGTTCATCGTTCATCGTGCCTACTCCTGAT

AATCATAGAATTCGAGGCTTGAGTTTTTGCTTGGTGTATACATGCTTGGAGAGTGAAGAA

ATGGTAAGTGAGGGCCACTGCATGTCCATTACAATTAATAATTTGTCGCAAAGGATCAAG

TGGAAGCAAGACCCTATGTTTCTAGCTATTCCTGAAGTTGAAGAGCGGATGATGTGGTTA

AGTTATTGGGAAATTGGCAACTCGTTGCAAACAGGTGATGTTGTAGAGATTTCAGCCAGT

GTTGTAGATAAACAACGTTTCAGTATCAACGAGGTTGGAATGAGGATTCTGTTCCTGGAA

GAACAACAAGACCAGGACAAAGAATCTAATTGTGAAGTTGAAGAGTTTTTTTCTAGTCCA

TGTCATCAGAATTTGCTCCTTGTCAGAGTCTTCCCGAAGTGA

>NTTN90_mRNA_43938_cds mRNA_43938 gene_24582|id=AT5G17680.1:evalue=2e-172:annot='disease resistance protein (TIR-NBS-LRR class), putative';id=Solyc08g005510.1.1:evalue=0.0:annot='Tir-nbs-lrr, resistance protein'

ATGGCATCTTCTTCTTCTTCAAGATGGAGCTATGATGTTTTCCTAAGTTTTAGAGGTGAA

GATACTCGGAAAACATTTACAAGTCACTTATACGAAGTCTTGAATGATAGGGGAATAAAA

ACCTTTCAAGATGATAAAAGGCTAGAGTACGGTGCAACCATCCCAGAAGAACTCTGTAAA

GCTATAGAAGAGTCTCAATTTGCTATCGTCATTTTCTCAAAGAATTATGCAACATCGAGG

TGGTGTTTGAATGAACTAGTGGAGATCATGGAATGCAAGACTCAATTTAGACAAACTGTT

ATACCGATATTCTATGATGTGGATCCATCACATATTCGGAACCAAAAGGAGAGCTTTGCA

AAAGCCTTTGAAGAACATGAAACAAAGTATAAGGATGATGTTGAGGGAATAAAAAGATGG

AGGATTGCTTTAACTGCAGCGGCCAATCTCAAAGGCTCATGTGATAATCGTGACAAGACT

GATGCAGACTGTATTCGGCAAATTGTTGACCAAATCTCATCCAAATTATGCAAGATTTCT

TTATCTTATCTGCAAAACATTGTTGGAATAAATACTCATTTAGAGGAAATAGAATCCTTA

CTAGAGATAGGAATCAATGATGTTCGGATTGTGGGAATCTGGGGAATGGGTGGAGTCGGT

AAAACGACAATAGCAAGAGCGATGTTTGATACTCTCTTAGTAAGAAGGGATAGTTCCTAT

CAATTTGATGGTGCTTGTTTCCTTGAGGATATTAAAGAAAACAAAGGTAGAATACATTCT

CTGCAAAATACCCTTCTCTCTAAACTGTTAAGGAAAAAAGCTGAGTACAATAATAAGGAG

GACGGAAAGCACCAAATGGCTAGTAGACTATGTTCTAAGAAGGTCCTAATTGTGCTTGAT

GACATAGATGATAAAGATCATTATTTGGAGTATTTAGCAGGTGATCTTGGTTGGTTTGGT

AAGGGTAGTAGGATTGTTGTAACAACTAGAAACAAGCATTTGATAGGGAAGAATGATGTA

ATTTATGAAGTGACTAGACTAGCTGACCAGGAAGCTATGCAATTGTTCAGTCAACATGCT

TTCAGAAAAGAAGATCTAGATGAGTGTTTTAAGGAGCTCTCATTGGAGGTCGTAAATTAT

GCTAAAGGCCTTCCTTTAGCCCTCAAAGTGTGGGGTTCTTTGTTGCATAATCTAGGCTTA

ACTGAATGGAAAAGTGCTATAGAGCACATGAAAATTAATTCTAATTCGAAAATTGTTGAA

AAGCTCAAAATAAGTTACGATGGATTGGAGCCCATCCAACAAAAGATGTTTCTAGATATA

GCATGCTTCTTTCGAGGGAGATCTAAAGGTTACGCCATGCAAATTCTTGAGAGTTGTCAT

TCTGGAATTGAATACGGATTGCGTGTCTTAATTGACAAATCTCTTGTGTCCATCTCTGAG

AAAGGTGAGATTCAAATGCATGACTTTATAGAAGATATGGGTAAATATATAGTAAATTTT

CAAAAAGATCCCGGAGAACGCAGCAGACTATGGCTCGTCGAGGATTTCGAAGAAGTGATG

ATCAACAATACAGGGACCATGGCAATGGAAGCAATCTGGGTTTCTTCTTATTCTTGTACA

GTACGCTTTAGCAATGAGGCCATGAAAAATATGAAAAGGCTGAGGATATTTAACATAGAG

AGGTCGTCGACCCGTGATGCCATTGAGTATTTGCCCAATAGCTTGCGTTGTTTTGTCTGG

TCTTACTATCCTTGGAAGTCACTGCCAGAAAATTTTGAACCCAAAATGCTTGTTCACCTT

GAACTCTGGAGCAGTTCACTGCGTTATTTATGGATGGAAACAAAGCATTTCCGGTCTCTA

CGGAGGATAGATCTCAGCTTCTCCGAAAGCCTGATGCGAACACCAAATTTCACGGGGATG

CCAAATTTGGAATATTTGAATCTGAAAAAATGTAGTAATCTTGAAGAGGTTCACCATTCC

CTGGGATGTTGCAGCAAACTCATTCGGTTAAATTTGATGTATTGTGAAAACCTTAAGAGA

TTTCCATGTATTAACATGGAATCTCTTAAATATCTGAGTGTAAGAGAGTGCTCTCGGTTA

GAGAAATTTCCAGAATTCCTCGGAAGAATAAAGCCGGAGTTGGAGATTGGCATAAGGGAA

CTACCATCCTCTTATTTTCAGTACCAAACTCATATTACCGAGCTAGATTTGAGCTATATG

AAAAATCTTGTAGCTCTTCCAAGCAGCATCGGTAGGTTGAAAAGTTTGGTTAATCTAAAT

GTGTCTCATTGCTCAAAACTTGAAAGCTTGCCAGAAGAGATAGGGGGTTTAGAAAACTTG

GAGGAGCTTGATGCTAGAGGTACTCTAATTTCACGACCTCCGTCTACCATTGTATGCTTG

AACAAACTTAAAATCTTGAAGTTTGGAGAGAGAGATGGAGTGCACTTTGAGTTCCCTCCA

GTAGTGGCTGAAGGATTACAGTCATTGGTACATCTGAATCTCAGTGACTGCAATCTAATA

GATGGAGGACTTCCGGAAGACATTGGATCCTTATCGTCTTTGAAAGAATTGTATCTCAAT

GGAAATAATTTTGAGCATTTGCCTCGAAGCATAGCCCAACTTGGTGCTCTTCGAATCTTG

CACTTAAGAGATTGCAAGAGGCTTACACAGCTGCCAGAACTTCCACCAAAATTAAATGAA

TTGCATGTAGATTGTCATACGGCTATGAAAAGTATCAATGATTTTGTAACAAAGAGAAAG

AAACTACAGTGCGTGATATTCGAGTCATTTGATTATGAGGATGATGCACACAATGATTAT

ATATATAATTTGTTTGCGCATGCCCTGTTTCAGAAAATCTCTTCCTTGAGGCATGACATC

TCTGCTTCAGATTCCTTGTCCGAAAATGTGTTTACCATTGTGCATCCAAAGAAGAAGATC

CCGAGTTGGTTCCACCATCAGGGTTGGGATAGTAGTGTATCAGTCAATTTGCCTGAAAAT

TGGTATATACCTGATAAATTCTTGGGATTTGCTGTATGTTACAATGGCAGCTTAATTGAC

ACCACAGCTCAATTGATTCCCGTATGTGATGACGGGATGACGTGCATCACCCAGAAACTT

GCCTTATCCAACCATTCAGAATGTGATACAGAATCATCCGACAGTTCAAAAAGGGATACA

GAATTTGCTATTCATTTTTTGTTGGTACCTCTTACTGTCTTATGGGATACATCTGAGGCA

AATGGAAAAACACCAAATGACTATGGGATTATTAGGCTATCTTTTACTGGAGAAATGAAT

AAGTATGGACTTCGTTTGTTGTATAAAGAAGAACCTGAGGATGAGGCCTTGTTACAAATG

TATGAACCAACAGAACATTCCACTGGGATAAGGAGGACCCGATATAACAATAGTGAACAC

CGTGACTCTGTGACCGATGAATCCAGTTGCTGCTGTCGCATACTCTAG

>NTTN90_mRNA_90508_cds mRNA_90508 gene_51165|id=AT5G36930.1:evalue=2e-163:annot='Disease resistance protein (TIR-NBS-LRR class) family';id=Solyc01g102880.1.1:evalue=3e-38:annot='Tir-nbs-lrr, resistance protein'

ATGTTCATGATGAGAGCTGAAAAGGCATCGTTCTCCAGTCCTAGATGCCCTTATCATGTA

TACTTGAGTTATAGAGGCCAAGAGATCAGTATGAACTTCATCAATCTTCTGCACACGGAA

TTGACACGTGTAGGTCTTCGAACATTCAAGAAACATGGTGAAGCTAGGAAAGGAAAGATT

GTTGGATCAGAATTGCAAAAAGCATTCAAGGAAGCGAGAATTTCAATAATTGTATTCTCG

GAAGATTATGCATACTCTAGAAGGTGCCTAGATGAACTTGTGAACATCCTGGAAAGGAAG

TTCAGTGCCGGTCATATGATTCTACCTGTGTTCTATCATATGGATCCATCCCATGTGAGA

AAGCAGAAAGGATCTTATGCAAAAGCGCTTCATAATTATGAAGAGCAGATCATGGGGGGG

AAGGGTGAAAGAAGGAATGAGCAGATAGCAAAGGTGAAGATATGGAGAGCATCTCTCAAG

GAGGTTGCAAATATGGCAGGGATAGTTCTAGAAGACGGGGATGAGTTAACGTTTATTCAA

GAAATTGTTGAGGACATCTGGGGTAAGATGAGTCGCAAAGTTTTGAGTATTGCCCAATAT

CCAGTTGGAATATATCACCGCGTGAAAGAGATCAGTTTCTGGTTACAAGATAGCTTGACT

GGTGCTCATACATTGATGATTTATGGAGAACCTGGAATAGGGAAAACAACCATTGCCAGA

GCTCTTTTTAACCTACATTGTGACAGATTTCAGTACAGCAGTTTTCTTGCAAATATTCGA

GAAATTGCAAAAGAAAGCAGCAGTCTAATTAGCCTACAGAAAAATCTGCTTTCAGATCTT

TTGAAAGAGGATAGGATTGATCTATATGATATTGATGGGGGAGCTTCTAAGATCAAAGAA

TTTTTGGTTCACAGAAGGTTTCTTTTGGTTCTTGATGATGTTGATGATTTGGACCAACTG

AAAGCATTACTTGATTCAAGAGATTGGATTCCTCCTGGGAGTAAAGTTATTATAACAACT

ACAAATAAAAGCTTGGTAAACCCTCACGATGCTTGTGTGATGTATGAAGCCAAGAGGTTT

GACATTCCTGAGGCGCTTCAGCTCTTCAGCTTGCATACTTTTGGTCAAGACCATCCCGCC

AAGGAGTACATGATGCAATCCAAACAGATAGTTAAACATTGTCGAGGGGTTCCTTTAGCT

CTTCAAGTTCTAGCCTCTTCGCTACGTGGTGGAAGTATAGATTTATGGGAAGCTGCAATA

AATAAATTAGAAAGATATTCTGAATTCCATAAACATAAAATTCTTGAACTAAGCTATGAA

GCTTTGCCTGATGACCATGAGAAAAATGTATTTCTTGATATTGCTTGCTTCTTTGTTGGA

AAGGACAAAGATTATACAATCAAAATTCTTGATGAATGTGGTTTCCATGCTACTGCTGAA

ATACAGAATCTCATCGATAGATGTCTTCTGACAGTGACTCCTGAAAACAAGCTAATGATG

CACCAGTTACTACAAGAAATGGGTAAAGAGGTCATCTGCCGAGAATCGCCCACAGAACCT

GGTAAACGCAGCAGAATCTGGCATCACAAGGATGCCCTCAGTATACTGCAGGAAGAAACT

GTTACAGAATCTATTGAGGGGCTCACTCTTAAAATGCGTGGATCAGATGAAAGCAAATCA

GACAAACATGAAAATATGTCAAAAAGACCTCACTGTGATGATTCACCGAATACTTCAACA

TTGATTCTCAAAAACTCATCAAAGAGGCTTTGCCTACGCTTTTTCTCTTGGCGTCAAGTA

AATTCTGTTTCGGCAAGATCACAGACTGCACCAAATGAAGCTGGAATAAGTTCTAAAGCA

TTTTTTAAAATGCAAGAACTGAGATTTCTTGAGCTTGATAATGTGCAGCTTTCTGGCACC

TATGAAGGATTTCCAAAGAAATTAAGATGGATGTGCTGGTATCAATTCCAATTAACTTCC

TTTCCAAGTGGCTTCCCTCTAGAAAATCTTGTAGTTCTTGAAATGAGCAATAGCAACTTG

CACCAAACATGGGAGGGAGCAAAGTCTCTCCGATCATTGAAGATACTTGATCTTAGTCAC

TCACGCCATCTCATGAAGACCCCTGACTTCTCTGGACTGCCGAGTTTAGAAAGAGTTATT

CTTGAACATTGCATAGGTTTGGTTAAGGTCCATGAGTCCATTGGAAGACTTCATAAACTT

CTTGTTTTAAATCTGAAGGGATGCGAGAGCCTTAGGAAGCTTCCAAGGAAGATGAGGGAA

ATAAAATCGTTAGAAGAACTAACACTCTGTGGGTGCTCGAAGCTGGAGTTTTCCACGACA

ATGCGAAATCAATTTTTTTTGCAAGCACTTCTGCAGAATGTGACTAACAGAAATCAACTT

GCCTCTAAGGGTGAAAAGCCAATATGTAGTGACTCTCTGGCTGCTAAATCTGTCTATTCA

ATTTTCTGGTCTTGGATGTCACCGTGGCCAAAGTCAGCACGTTCAATGTCAGAACTCTTT

CAGATCACCTTACAAAGTTTGGATATTTCACATTGCAATCTGACTGGCACTTTTATTCCC

TATGATCTTTCTGTCTTGTCCTCCTTAAAATATCTGAATTTAAGAGGAAATCCCATTACT

ACCCTGCCAGAGAGCCTGAAGAGCCTAACTGTGCTGCAGTCCCTTCAGTTAGCTGACTGC

ACAAAGCTCCAGTGGATCCCGGAACTTCCATTGAGTTTACAATTATTGAATGCACGTAAC

TGCATATCACTGAATAGAGTAACAAATCTACCCAACTTCATGAGGTCACTTGACTTGCAC

CTAGAGAATTGTGGAAATCTGGTTGAGGTTCAGGGAGTATTCAAGCTAGATCCTATTGAT

GATATTGTTGTTGTATCTTGCCTAGATAATTTGGAGGTCCGAGCAGTAGATGTGGAACTT

TGCAACTATCTGACCTCAACGAAAAGCAAAGGTCCCGTTCAGGGACTCTATGAATTTGGT

ATACACAGCATTTTCATTCCTGGAGGCAAGGTTCCTACCGAGTTCAATAATATAAGCACA

GGGAGCTCATTAGCTTTCTCTGTGCCTCAACTTCCTAACATCAAGACACAAGGATTAGAG

ATATGCATTGCTTATGTAGAATGTTATGACGAATGTTTCAGCCAAAGTCACTTCATAAAA

GTGAGCAATAAAACCAAGAGGATCAAATGGAATTATGGTCCAACGGTTTTTGGGATTGCA

ACTCCTGGTAATCCAATGCTATGGTTAAGTCGTTGGAAGTTTGGAGATCAGCTAGAAAAA

GGAGATCAAGTTGTTGTCTCCTTAAGCATGAGTTGCTTGGTTAGGGAATTTGGTGCCCAT

CTTGTATGCAGTGAACAAAGGGAGGAAGACACCCTATCTGAGAATACAAAGGAGGGATGT

CGTCGTCCCTGCTATCCACTTCATCATGCAATTGGAGGAGATTTGTCTCCTTATGAGCTA

AGCTCAGGCGTCTATCACCTCTCCATTTATTCTTGA

>NTTN90_mRNA_56562_cds mRNA_56562 gene_31705|id=AT5G17680.1:evalue=3.8:annot='disease resistance protein (TIR-NBS-LRR class), putative';id=Solyc11g011090.1.1:evalue=2e-88:annot='Tir-nbs-lrr, resistance protein'

ATGGAATCATCTTCTGCTTCTGCGAGTACTTCACAGTTTTTTCGATGGAACTACGATGTT

TTTCTAAGTTTTAGAGGTGTAGATACACGGAAATCATTTACGAGTCACCTGGGCGAAAAC

TTGAATATGAGGGGAATAAAAACCTTTCAAGATGATAAAAGTCTAGAGCATGGCGCATCC

ATCCCAGATGAACTCTGTAAAGCTATCGAGGAGTCTCAATGTGCACTCATCATTTTCTCA

AAGAATTATGCAACATCGAGGTGGTGCTTGAATGAACTAGTGAAAATCATGGATTGCAAG

TCCCAGTTTGGACAAACTGTCATACCGGTCTTCTATGATGTGGATCCATCACATGTTCGG

AATCAGAGGGAGAGCTTTGCTGAAGCATTTGCCAAACATGAAACAAAGTATAAGGATAAT

GTCGAGGGAATGCAAAGATGGAGGATTGCTTTAACTGCAGCGGCCAATCTCAAAGGCTGT

GATATTCGTGACAAGACTGAAGCAGACTGTATTCGACAGATTGTTGACCAAATCTCCTCC

AAATTATGCAAGATTTCTTTATCTTATTTGCATAACATTGTTGGAATAAATACCCATTTA

GAGGAAATAGAATCCCTACTAAGGATAGGAATCAATGATGTTCGGATTGTGGGGATCTGG

GGCATGGGGGGAGTGGGTAAAACGACAATAGCTAGAGCTATGTTTGATACTCTTTTAGGA

AGAAAGGATAGTTCCTATCAATTTGATGGTGCTTGTTTCCTTAAGGATATTAAAGAAAAC

AAACGTGGAATGCATTCTCTTCAAAATACCCTTCTCTCTCAACTTTTAAGGGAAAATGCT

AATTACAATAATGAGGATGACGGAAAGCACCAAATGGCTAGTAGACTTCGTTCTAAGAAA

GTCCTAATTGTGCTTGATGACATAGATGATAAAGATCATTATTTAGAGCATTTAGCAGGT

GATCTTGATTGGTTTGGTAATGGCAGTAGAATTATTGTAACAACTAGAGACAAGCATTTG

ATTGGGAAGAATGATGTAATATATGAAGTGACTGCACTACCTGATCATGAATCAATTCAA

TTGTTCTACCAGTATGCTTTCAAAAAAGAGGTTCCAGATGAGTATTTTAAGAAGCTTTCA

TTGGAGGTCATAAATCATGCTAAAGGCCTTCCTTTAGCCCTCAAAGTGTGGGGTTCTTCC

TTACATAAGATGGATATAACTGTGTGGAAAAGTGCTATAGAGCAAATGAAGAATAATCCT

AATTCAAAAATTGTTCAAAAACTCAAAATTAGTTATGATGGATTAGAGTCCATGCAACAA

GAGATTTTTCTGGATATAGCATGCTTCTTACGCGGGGAAGAAAAAGATTACATCATGCAA

GTTCTCAAGAGTTGTTATTTTGGAGTTGAATATGGATTGGATGTCCTAATCAGAAAATCT

CTTGTGTTCATCACTGAAGATGGTAAAATTGATATGCATGACCTAATACAAGAAATGGGT

AGATATATAGTGAACTTTTCAAAGGATCCGGGAGAACGCAACAGACTATGGCTCGCCAAG

GATTTTGAAGACGTGATGATCAATAATACGGGTACCATGGCAGTGGAAGCAATTTGGGTT

TGTGAGGCCATGAATTGGGATACAATTCGTACACTACACTTAGGCAATGAGGCTATGAAA

AATATGAAAAGGCTTAGGATATTAACCATAATGAGGTTGTGGTCCTTTGATGGCTCCATT

GAGTATCTGCCCAACAGCTTGCGTTGGTTTGTCTTTGGTGGCTATCCTTGGGAGTCATTC

CCAGCTGAATTTGAACCCAAAAAGCTTGTTCACCTTGCACTCACATCCAGTTCACTGTGT

TATTTATGGACGGGAACAAAGGATTTGCCGTCTCTACGGAGGATAGATCTCATGCGGTCT

AAAAGACTGATGCAAACACCAGATTTCACGGGGATGCCAAATTTGGAGTATGTGAATTTT

TATAAATGTAGTAATCTTGAAGAGGTTCACCATTCCATGGGATGTTGCAGAAAACTCATT

CGGTTAAGTTTGGGTTATTGTAAAAGCCTTAAGAGGTTTCCATGTGTTAACGTGGAATCT

CTTGAATCTCTCAGTTTAGGATATTGCTCAAGTTTAGAGAAATTTCCAGAAATCCACGGG

AGAATGAAGCCGGAGATACAGATTCACATGCTAGGCTCTGGGATAAGGGAACTACCATCC

TCATATTTTCAGTACCAAACTCATATTACCGACCTACATTTGAGCTTTATGAAAAACCTT

GTTGCTCTTCCAAGCAGCATCTGTAGGTTGAAAAGTTTGGTTAGTCTAAATGTGTGGGGT

TGCCAAAAACTGGAAAGTTTGCCAGAAGAGATAGGGGATTTAGACAACTTGGAGGAGCTT

TATGCCCGCTGTACTCTAATTTCACGACCTCCGTCTTCCATCGTACACTTGAACAAACTT

AAAATCTTGAAGTTTGGAGGCTTCAAAGATGGAGTGTACTTTGAGTTCCCTGCGGTGGCT

GCAGGATTACGATCATTGGAAGATTTGGATCTCAGTGAGTGCAATCTAATAGATGGAGGA

CTTCCGGAAGATGTTGGATCCTTATCCTCTTTGAAAGAGTTGGATCTCAGTAGAAATAAT

TTTGAGCATTTGCCTCGAAGCATAGCCCAACTTGGTGCTCTTCAATCCTTAAACTTATCA

AATTGCGAGAGGCTTACACAGCTACCAGAACTTCCCCCTGAATTAAATTTATTGCATGTA

GATTGTCATATGGCTCTGAAATTTATCAATTATTTAGTAACAAAGAGAAAGAAACTACAG

AGAGTGATATTCCCTTATGATGAGGATGATGAGGATGATGCACACAATGATACTAAATAT

AATTTGTTTGCACATGCCCTGTTTCATAATATCTCTTCCTTGAGGCATGACATCTCTGCT

TCAGATACCTTGTCCGAAAATGTGTTTACCATTTGGAATCCTAAGAAGAAGATCCCAAGT

TGGTTCCTCCTTCAGGGAACGAATAGTAGTGTATCGGTCAATTTGCCTGAAAATTGGTAT

ATACCTGATAAATTTTTGGGATTTGCTGTATGTTACTGTGGCAGCTTAATTGACACCACA

GCTCAATTGATTCCCGAATGTGATGATGGGATGTCGTGCATGACCCAGAAACTTGCCTTA

TCAGAATGTGATACAGAATCATCCGACGACTCAGAACGGTATACACCAATTCATTTTTTC

TTGGTACCTCTTGCAGTCTTATGGGATACATCTAAGGCAAATGGCAAAACACCAAATGAC

TATGAGATTATTAGGATATCTTTTTCTGGAGAAATGAAGGACTATGGACTTCGTTTGTTG

TATAAAGAAGAAGCTGATGATGAGGCCTTGTCACAAATGAGGGAAAATAACAATGAAACA

ACAGAACATTCCACTGGGATAAGGAGTAGCAGATCTGACCATAGTGAACACCATGACTCC

GTGACCGATGAATCCAGTTGCTGCTGTCACATACTGTAA

>NTTN90_mRNA_73131_cds mRNA_73131 gene_41359|id=AT5G36930.1:evalue=3e-141:annot='Disease resistance protein (TIR-NBS-LRR class) family';id=Solyc01g113620.1.1:evalue=0.0:annot='NBS-LRR resistance protein (Fragment)'

ATGGAGCAAGGTATACCTTCGTCGCCTCCTACTTCTCAATGTTCATATCATGTGTTCTTG

AGTTTCAGAGCAAAAGACACTGGCAAGACTTTCACTGATCATCTCCACAGAAATTTGGTG

CGAGCTGGGTTTCATGTATTCAAATGTGATAACGATGATTATGACGGTGAAAAAGAGGAC

TTGAAGTCAAAATTGCAAAAGGGAATAGAGCAATCGAAGATGTCAGTGATTGTATTGTCC

CAAAATTATGCATCATCTGAAAAGTGTCTTGATGAGCTGGTCGTGATTTTGGAGCAAAAG

AGGAATTTTGGGCACATAGTTTTGCCTGTCTTTTTTAACGTGGATCCTTCTGATGTTAGG

AAGCTAAAGGGAAGTTTTGGTCAGCCTTTTTCTGCAAATGGAGAGAGTCAAAAGTTAAGA

GATTGGAGAAATGCTCTCAAACAAGTTGCAGATTTGGGAGGGATGCCCTTGCAAAATCAA

GCTGATGGATATGAGGCAAAGTTCATTGAGAATATAGTTGAAGTAATTGCAAGCAAGCTA

CGTCCCAGAGCCTTAAACAATGCTCCTTACCTCATCGAAATCAGTTATCGGGCTGAAGAT

ATTATCTTGTGGCTACAAGATAGATCAACTAATGTAGGGCTATATGTGATCTGCGGGATT

GGTGGAATTGGGAAGACAACCCTTGCCAAATTTGCCTATAACTCAAGTGCAAGATCATTT

GAAGGAAGCAGCTTTCTTGCAAACATCAATGAAACTGCAAAACAATGTAATGGTCTAGTT

CGTCTGCAGAAGCAAGTTTTGTATGATATAGTTGGAAAAAAAGAGAGGATATCCAATGCT

GATGAAGGAATTATGATGATTGAAGATGCCCTACGCTATGAACAAATTCTTCTTGTTCTT

GATGATGTTGATGAAGTTGACCAGATAGATAAAATTTTAGGAATGAGAGATTGGCTTAAT

CCTGCTAGTAAAATCATTATAACAACGAGGCATGAGTCCTTGCTAAAGCCTTTTGTACCT

CACAAGGTGCTTAAAGTAGAAGCCTTGAATAAAATGGATTCCCTAAAGCTGTTCAGCTGG

CATGCCTTCGGAGAAGACCATCCTTTGGAAGGTTATGTCGAGCTCTCAAAACGGGTGGTT

CTTCAATGTGCAGGACTTCCTTTAGCTCTTCGTGTTTTAGGTTCAGCTCTGTCGGGTAGA

AGACCAGAAATATGGGGAAGTGCATTAGAAAAGCTGGAAACAATCCCTGATGGTCATGTC

ATTGAAAAACTTAAAGTGAGTTTTTACTCTCTAGAAGATGACCATGACAAAGATATATTC

CTTCATATAGCTCTTTTCTTTCTTGGGATGGACAGAGACGACTCTGTCAGAATACTGAAT

GGATGTGGTTTTTACACAATAATTGGGATGCAAAACCTCATTGATAGAAGTCTTTTGACA

ATCAATGACTTAAATAAGTTGGAGATGCATCAGTTGCTTCGAGACCTTGGGAGAGATATC

GATCGTAGGGAATCACAGGATCCTGGAAAACGCAGTAGACTTTGGAATAACAAGGATTCT

TTCAGAGTGTTGAACGATAAAACTGGCACTGAAAGGATTGAAGGGATCAGCTTTGACATG

CCTATGTTGATGGAGGATAAATCAGCCAAACAATTTTTCACTGGAAATAGCTCGAAAAGG

CGCTTCCGAAAAGATCATGTAGAGAACTGTGCAGATCATAATCTTTCGCTGCAACAGCCT

TCTTCTGTCTTTTATTCATGGAATTCAGGAGACACTTCATCACGAAACTCAAACTACAGC

ATAGAAACTGATGCATTTACGATAATGCGAAACCTAAGGGTACTCAAACTAAATGATGTA

AACCTCATTGGATGCTATAAAGAATTTCCCAAGAGATTAAAATTGTTGTCTTGGCGTAAA

TGCCCTTTAAAATCCTTACCTAGTGACCTTTCCTGGGAAAACCTTGTTTCTATTGACATG

CGGTACAGCAATTTACAACAAACATGGAGTGAAACTGAGTATTTCAGATTTCTCAGGATT

CTCAACCTTAGTCATTCTTGGGAACTTACCAGAACACCTAGCTTCGCTGGAATGGCCCGA

CTTGAGAAACTAATTCTTAAAGATTGTATTAAACTGGTTGATATCGATGAAACTATCGGC

TGCCTCCAAGAAATCACCCTGCTAAATCTGAAAGACTGCAAGAGCATCAGAAAGCTGCCA

AGAAATATCGGTGAACTTATAACTCTTAAGATACTTGATATATCCTTCTGCTCAAGCCTG

GAGTGGCTGCCAATGGAGCTTAACATGATAGATTCTTTGAAAGTTCTAAGAGCTGATGGA

ATTGATCTAAACCAAATACTCTGTACCACCCACGAGCGGAAATCATTGCAGGCATTATTT

TCATCTTGGGTATCAAAGCCAAGAAACACTCCTGAAATATCGTGGGCCTTTTTACCAAGC

TCTTTGGTGAGCTTGAGTCTTGTGAGTTGCAGGCTGTCTGATGAATATATTCCCCAGAAG

TTTATCAATCTTCCGCTGCTCCAAGAACTGGATCTAAGTGAAAATTCAATTAGCTGCCTC

CCGGAGTGGGTCAAGAGTCTGCCTCAGCTCCAAAGCCTCAGTGTCAAGTCATGGGAAATT

TAA

>NTK326_mRNA_39598_cds NTK326_mRNA_39598 gene_22838|id=AT5G17680.1

ATGGCATCATCTTCTGCTTCTGGTACTTCACAGTTTCCTCGATGGAACTACGATGTCTTC

CTAAGTTTTAGAGGTGAAGATACTCGGAAAACATTTACGAGTCACCTGTACGAAATCTTG

GATATCAGGGGAATAAAAACCTTTCAAGATGATAAAAGGCTAGAGCATGGCGCATCCATT

TCGGATGAACTATGTAAAGCTATCGAAGAGTCTCAATGTGCAGTCATCATTTTCTCAAAA

AATTATGCAACATCGAGGTGGTGCTTGAATGAACTAGTGAAGATCATGGATGTCAAGACT

CAATTTGGACAAACTGTCATACCGGTCTTCTATGATGTGGATCCATCACATGTTCGGAAC

CAGAGGGAGAGCTTTGCTGAAGCATTTTCCAAACATGAAACAAAGTATAAGGATGATGTC

GAAGGAATGCAAAGATGGAGGATTGCTTTAACTGCAGCGGCCAATCTCAAAGGTTGTGAT

ATTCGTGACAAGACTGAATCAGACTGTATTCGACAGATTGTTGATCAAATCTCGTCCAAA

TTATGCAAGATTTCTTTATCTTATTTGCAAAACATTGTTGGAATAGATACTCATTTAGAG

AAAATAGAATCCTTACTAGGGATAGGAATCAATGATGTTCGGATTGTGGGGATTTGGGGC

ATGGGGGGAGTCGGTAAAACGACAATAGCTAGAGCTATGTTTGATATTCTCTTAGTAAGA

AGGGATAGTTCCTATCAATTTGATGGTGCTTGTTTCCTTGCGAATATTAAAGAAAACAAA

CGTGGAATGCATTCTCTGCAAAATATTATTTTCTCTGAACTTTTAAAGGAAAAAGCTGAT

TACAACAATAAGGAGGACGGAAAGCACCAAATGGCTAGTAGGCTTCGTTCAAAGAAGGTC

CTAATTGTGCTTGATGACATAGATGATAAAGATCATTATTTGGAGTATTTAGCAGGTGAT

CTTGATTGGTTTGGTAATGGCAGTAGAATTATTATAACAACTAGAGACAAGCATTTGATG

GGGAAGAATGGTGTAATATATGAAGTGACTGCACTACCTAATCATGAATCCATTCAATTG

TTCTATCAGCATGCTTTCAAAAAAGAGGTTCCAAATGAGCATTTTAAGAAGCTTTCATTG

GAAGTCGTAAATTATGCTAAAGGCCTTCCTTTAGCCCTCAGAGTGTGGGGTTCTTTGCTG

CATAACCTAGGACTAACTGAATGGAAAAGTGCTATAGAGCACATGAAAAATAACTCTAAT

TCTGAAATTGTTAAAAAGCTCAAAATTAGTTATGATGGATTAATAGAGCCCATACAAGAG

ATTTTTCTGGATATAGCATGCTTCTTCCGAGGGACAAAAAAAGAGTACGCCATGCAAATT

CTTGAGAGCTGTCATTGTGCAGTTGAATACGGATTGCGTGTCTTAATTGACAAATCTCTT

GTGTCCATCTCTGAAAATGATCAGATTCAAATGCATGACTTGATGCAAGATATGGGTAAA

TATATAGTGAACTTGCAAAAGAATCCGGGAGAACGCAGCAGATTATGGCTCGACAAGGAT

TTCGAAGAAGTGATGATGAACAATACAGGGACCACGAAAATGGAAGCAATCTGGTTTCCT

TATTACCATTATGTTACATTACGCTTTGGCAAAGAGGCCATGAAAAATATGAAAAAGCTT

AGGATATTAAACATAGAGATGTCGTGGCCTTGTGATGGTTCCATTGAGTATCTGCCCAAC

AGCTTGCGTTGGTTTGTCTGGACTGACTATCCTTGGGAGTCGTTGCCAGCTGAATTTGAA

CCCAAAAAGCTTGTTCATCTTGCACTCAAATCCAGTTCACTGTGTTATTTATGGACGGAA

GCAAAGCAATTGTCGTCTCTACGGACGCTAGATCTCAGATACTCTGAAAGCCTAGTGCGA

ACACCAGATTTCACAGGGATGCCAAATTTGGAGTATTTGAATCTGGAGGAATGTCGTGAT

CTTGAAGAGGTGCACCATTCCCTGGGATTTTGCAGAAAACTCATTCGATTAAATTTGGAG

TCTTGTGGACGCCTTAATTGGTTTCCATGTGTTAACGTGGAATCTCTTGAATATCTGGAT

CTAGATTTTTGCTCTAGTTTAGAGAAATTTCCAGAAATCCATGGGAGAATGAAGCGGGAG

ATACAGATTCACATGAAACGCTCTGGGATAAGGGAACTACCATCATCTATTATTCAGTAC

CAAACTCATATTACCTTCCTAGATTTGAGCGCTATGAAAAACCTTGTAGCTCTTCCAAGC

AGCATCTGTAGGTTGAAAAGTTTGGTTAGTCTAAATGTGTCGGGCTGCACAAAACTTGAA

AGCTTGCCAGAAGAGATAGGGGATTTAGAAAACTTGGAGGAGCTTTATGCAAGTGGCTGA

>NTTN90_mRNA_3372_cds mRNA_3372 gene_1859|id=AT5G17680.1:evalue=0.68:annot='disease resistance protein (TIR-NBS-LRR class), putative';id=Solyc02g082050.2.1:evalue=0.0:annot='Tir-nbs-lrr, resistance protein'

ATGAGCACAGAAGACAGTAATGTGTCTTCTTCTTATCAGACGACGACTGATACTGAAACA

AGTAGTCGTCGTCAATGGTCATATGACGTTTTCTTGAGTTTTAGGGGTGAAGATACTCGA

AAGAGTTTTGTTGATCACCTCTACACCACTTTGCATGAAAAGGGGATTCATGCATTTCGA

GACGACATAGAGTTACGGAGGGGAAAATTCATTTCCCCTGAACTCCTTAACGCAATTGAA

AAGTCTAGGTTTGCAGTTGTCATTTTCTCAAAAAACTATGCCAATTCCTCCTGGTGTTTG

GAGGAATTGAGGAAGATTGTTGATTGCACAAAGCATAGAGGGCAAACGCTGATGCCTGTC

TTCTACGGAGTAGATCCTTCGGTAGTAAGAAAACAGAAGGGGAGTTATTTAGAAGCCTTT

GCAGAACATGAAAAGAATTTCGAGGAAAAGAAGATCAAAGAATGGAGGGATGCTTTGAAG

GAGGCAGCTAACATCTCTGGTTATGACGTCCAACACATGGAAGATGGGCATGAGTCGAGG

TGCATGAGACAAATTGCAGTTGCAATTTTGAACAAGTTGGGCCATGTACGACCTAAAATT

GCAGACAATCTAGTCGGGATCGAGCCCCAAGTACAAAACTTAATATCTCTGCTGAATACA

AATTCTGAGACTGATGTTCGCATAATTGGGATATGGGGTATGGGCGGCATCGGCAAATCA

ACCATAGCACGAGCTGTTTTTGATCAACTTCAAGAAATGTTTGAAGGTGGCTGCTTTCTT

GATAACGTTAGAGAAGCTGCATCAAAATTTGGACTGCAAGCTTTGGCTGAAAAATTGCTT

TCCGAGACATTAAAAGAGACCAAAGACAATCTTTACAGCAGCACCAACTTACTGATGAAC

AGGTTGAGCTATAAAAAAGTGATGATCGTTCTGGACGACGTGGATCAAGATGAACAGATA

GAAAACCTGATAGCTGGAGGGCACAAAGGGTTCGGAGCTGGTAGTAGAATAATTATCACA

ACAAGAAACAAGCAATTGCTATCTTCTTGTGGAGTGGACCAAGTTTATGAAGTTAGCCTA

TTAGGAACTAATGAGGCTTTGATGCTCTTCAACAGGTTCGCCTTCAAGGAAGCTCAACCG

CATGATCATTTCATGGAACTAGCGTTACGAGTGGTGAAATGTGCTTGGGGACTCCCCTTG

GCCCTCAAAGTTTTGGGATGTTTTTTGCACAAAAGAGAAAAAGAAGAATGGGAGAGCGAG

TTGACGAGGTTGGAAGGTATTCCTCATGATGATGTGATAGGGAAACTTAAGTTAAGCATT

GATGCATTGAATGATTTAGACAAGCAGATATTGCTTGATATTGCTTGTTTCTTTAAGGGG

AAACGAAGAGAACCTGTGATCAAGAAATTCCATGCTTTCGGTTTCAAACCTGAAATTGGA

ATACCAGTGCTTGTTCAAAGATCTTTGTTATCTATATCTGATGATGACAGATTTCAGATG

CATGATTTAGTTCAAGAAACCGCTTGGTACATGGTTCGCCAGGGACAAATTAAAGAGAAA

TACAGCAGGTTGTGGATTCCTGACGATATATGTGATGTTATGTCAAAGAAATCGGGTACA

GAAGCGATTCAGGCAATAATATTGACTTACCCACAAAAAGAGAAATTGAACTTATCATCA

CAGGCATTAAAAGGTATGGAAAATCTACGTCTGCTCAAAATCCGCAATGCCTACTTCAAT

AGAGGTCCAAGTTATCTTCCGAATGGGTTACAATGGCTCAATTGGCACAAGTTTCCCTCA

ACCTCTCTTCCACAAGACTTTGAGGGAGAAAAGCTTGTCGGACTTAAGCTAAGTCGTGGC

CAAATTTTGCAACTATGGCCAGAACCTAAGAACCTTGAGCAGTTGAAGTATTTGAACCTC

AGCTACTCCAATGGGCTAATTAACAGTCCAGATTTCAGCATGATGCCAAATCTTGAAAAG

TTGAATCTTAGCAACTGTAAAAACTTGGTAGTAGTTCATGAGTCAATTGGAACACTTCAA

AGGCTTAAATACTTGAATATGTCTCACTGCTCAAAGCTCAGTCGTCTTCCGAATACCATT

CACCTTGAATCTTTGGAAATTTTTCTTCTTTGGGACTGCACCAAACTTGAAAATTTTCCT

CAAGTTATTGGCTTAATGCCAAATCTTTCAGAACTTCACTTGGAAGGGACTGCTATTAAA

GAGCTACCCGACTCCCTGATAAATATCAGTGGCCTTGTGTCCATAAACCTCAGCAATTGC

AAATGCCTGGAAAATATAACCTACAGCATCTGCGGTTTGAGATGTCTCCGTAGTCTTAAT

CTCTCTGGCTGTTCAAAACTCGAGACATTGCCCGAAACTCTTGGCCAATTGGAAACTTTG

GAAGAAGTCCTTGTGGATGGAACTGCAATTACCAAGCTACCATCAACTATCTCCAAAATG

GGGAACTTGAAAATCCTTTCTTTCAGTGGATGCAAGAATATTAAAAAGGATACGTCATTT

GGAGTGGCAAGTTTAAGCAAGTTCACTTCAATGACAAATGTCAAGAATCTTATGAAACGA

TCAGATGCTGAGAGAAAGAAGCCACAAACAGCACGGCCATCCTTATCTGGTTTGCGTTAC

TTGAAGAAATTAGACCTCAGTGACTCTGATTTGGTAGATGAAGTTGCTACTGATGTTTGG

CACTTGGCCTCATTGGAGGAGTTAAATTTGAGCCGAAATAATTTTGTGCAATGTCCTTCA

AAAATATCTGGACTCCCGGGATTCAAAGTCCTGAAATTGGAAGAATGCAAGAGACTTGAA

GTACTGCCTGATCTTCCATTGAGTATTGCTGTGATAGAGGCAAACGAGTGCCCGGCCCTA

CACACTCTTGGAAATCTTTCAACCCGACATGCATTCTTAAGGAAGGTTTCCTTTTCCAAT

TGTCACAAATTGCATGAACAAAGCAAGAAAACTGGCATTTTTGCTGTGGATTTGTTGTTG

GAACTGCTACTTCAGGGGCACTCCATCATATATGGTCGATTCAGTATACTGGTTGCTGGA

GGAAAAATCCCCGTGTGGTTTGATCATCAGAAAGTAGGCGGTTCTATCTCAGTGCAGCTA

CCTTCAGATTGGCAAGATAACATTGTGGGAATTGCAGTGTGTTTCGTTTTGGACAGTTTT

ATTCCGAAGTCAAAACTAGGTGTTACTTTCAAGTTGGTTAGCCCAGACCACAGAGAATAC

ACTTCTGAAAATGCACCTTCTGCTGCTTCAAAGATGGGAGAAGTGTACGATTCTGATCAC

GTGTGGATAACTTATATCTCTTTCAATCTTTTTCGTCTCCTCTTCCCTGATTTCACAACT

GAAGATTGGTCTAAAGTTTGTGGTAGTCTTTCAATTAGAATAAGGCAGGATCCGTGGACA

AAGGTAAGGAGGTGTGGAATTCAGCTTGTTTACAAACAAGATTTGAGCACATTGGCAGCT

GAACGTGCAGGGGTTAATAAAGATTCAGCTGGGAGCAAAGAATTAGTGGTGTATGAAGGA

GGAGGTAAAGAAAGTAAAGAAGAAGCTGTGATCAAAGAGGATATTGCTGCACTTATGGCT

GGTGTTACTGAATTGAATTGGGATGTTGACCCCATTGAGCAAGATACCACTCAGCTCATG

AATTTGAGGAAATCAATTGCCTATAAAATTCAAAAGACTCTCTCTTTTGAATGCTAG

>NTTN90_mRNA_79954_cds mRNA_79954 gene_45214|id=AT5G17680.1:evalue=1e-32:annot='disease resistance protein (TIR-NBS-LRR class), putative';id=Solyc11g011090.1.1:evalue=3e-110:annot='Tir-nbs-lrr, resistance protein'

ATGGAATGCAAGACTCAATTTGGACAAACTGTCATACCGGTCTTCTATGATGTGGATCCA

TCACATGTTCGGAACCAAAGGGAGAGTTTTGCAGAAGCATTTTCCAAACATGAATCAAAG

TTTAAGGATGATGTTGAGGGAATGCAGAAGGTACAAAGATGGAGGAGTGCTTTAACTGAA

GCGGCAAATCTCAAAGGTTGTGATATTCGTAACAGGATTGAATCAGACTGTGTTCAGCAG

ATCGTTGACCAAATTTCCAAGTTATGCAAGTTTTCTTTATCTTATTTGCAAGATATTGTA

GGAATAAATCCACATTTAGAGGAAGTAAAATCCCTACTACAAATAGAAATCAATGATGTT

CGGATTGTGGGGATCTGGGGCATGGGAGGAGTTGGTAAAACGACAATAGCAAGAGCCATT

TTTGATACACTCTCGTATCAATTTGAAGTTACTTGCTTCCTGGCGGATGTTAAAGAAAAC

AAATGTGGAATGCATTCTTTGCAAAATATCCTTCTCTCAGAACTGTTAAGGGAAAACGCT

AATTACGTGAATAATAAGGAGGACGGAAAGCACCTGATGGCTCGTAGACTTCGCTCTAAG

AAGGTTTTAGTTGTGCTTGATGACATAGATCACAGAGACCATTTGGAGTACCTAGCAGGG

GATCTTGGTTGGTTCGGCAATGGCAGTAGAATTATTGCAACAACAAGAGACAAGCATTTG

ATTGGGAAGAAGGATGCATTATATGAAGTGACTACACTAGCTGACCATGAAGCTATTCGA

TTGTTCAATCGATACGCTTTTAAGGAAGATGTTCCAGATGAGGTTTTTGAGAAGCTAACG

CTGGAGGTAGTAAGTCATGCGAAAGGCCTTCCTTTAGCGCTGAAAGTGTGGGGTTCTTTC

TTTCATAAGAGGAATATAACTGAGTGGAGAAGTGCTATACCGCAAATGAAAAAACACTCT

AATTCAGAAATTGTTGACAAGCTCAAAATTAGTTATGATGGATTAGAGCCCGTGGAACAG

GCGATATTTTTAGATATAGCATGCTTCTTACGAGGGAGAGAAAAGGATGAGATCATACAG

ATTCTTGAGAGCTGTGAATTTGGAGCTGATATCGGATTGCGTGTCCTAATTGATAAATCT

CTTGTGTTCATCTCCGAAAAAGATACGATTGAAATGCATGATTTAATACAAGATATGGGT

AAATATGTCGTGAACATGCAGAAGGATCCTGGAGAACGTAGCAGACTATGGCTCGCTGAA

GATTTCGAAGAAGTGATGACCAACAATACGGGGACCAAGGCAATGGAAGCAATCTGGTTT

CGTTATTCTCAACGACTATACTTTAGCAAAGAGGCCATGAAAAATATGAAAAGGCTTAGG

ATATTCTACATACGTGCTCAGTACTTGAATTCGTGGATCCGTGATGACTTCAATTGCCAT

GATGGCCCCAATGAGTACCTGTCTAACAACTTGCGTTGGTTTGTCTGGGATCACTATCCT

TGGGATTCATTGTCAACTAATTTTGAACCCAAAAGGCTTGTTCATCTTCAACTCTGGCGC

AGTTCAGTGCATCATTTATGGACAGGGATAAAGCATTTGCCGTATCTGCGAAAGCTAGAT

CTCAGGGAATCTAAAAGCCTGATGCGAACACCAGATTTTACGGGGATGCCAAATTTGGAG

TATTTGGATCTGGAAAAATGCTCTAATCTTGAAGAGGTTCATCATTCCCTGGGATGTTCC

AGAAGACTCATTGTCTTAAATTTGTATGAGTGTGGACGCCTTAAGAGGTTTCCATGTGTT

AACATGGAATCTCTTGAATATCTGGGACTATATTGTTGCTATAGTTTAGAGAAATTTCCA

GAAATCCACGGAAGAATGAAGCTGGAGTTAAAGCTTTACATGCAATACTCTGGGATAAGA

GAAGTACCATCATCTATTACTCAGTGCCAAACTCACATTACCAAGCTAAATTTGAGCAAG

TTAAGAGACATTGCAACTCTTCCAAGTAGCATTCGCATGTTGAAAAGCTTAGTGGAGCTA

GATGTGTCGGATTGCTCAAAACTTGAAATCTTGCCAGAAGAGATAGGGGATTTAGAAAAC

TTGGAGAAGTTTGATGCTGCACGTACTTTAATTTCACGGCCTCCACCTTCCATTGTATGC

TTGAACAAACTTAAATTGTTGTCTTTTGATCAAAGAGAATCAAAAGAAGGCCAAGTCGTG

TATTTTGTGTTCCCTCCTGTGGCTGAAGGCTTACACTCATTGGAAATTTTGCATCTCAGT

GACTCCAATCTAATAGATGGAGGACTTCCAGAAGACATCGGATGCTTATCCTCTTTGAAA

AAGTTGTATCTTGGTTACAATAATTTTGAGCATTTGCCTCAAAGCATAGCCCAACTTGGT

GCTCTTCAATTCTTGTACTTATCAGGTTGCAGTTTGCTTAAAGAGTTGCCAGATTTTATG

GGGATGCCAAATTTGGAGAAGTTGGAGCTGTCATATTGTGAGAATCTTGAAGAGGTTCAT

CATTCCCTAGGATTTTTTAAAAAGCTCCGTAAATTATCATTGGATACTTGTGAACGGCTT

AAGAGGTTTCCAGGTCTATGCATTGATTCTCTTGAATTTCTGTGGATACGGGGTTGCTCT

AGTTTAGAAAAATTTCCAGAAATCCACGGAAGCATAAACTCTGAGTTAGAGATTCACATG

CTAGACAATGTGATAAGAGATCTAGATTTGAGAGGTCTAGAAAACCTTGTAACACTTCCG

AGCTGCATTTGTAAGTTTAAAAGCTTGGTGAAGCTAGATGTGTCAGATTGCTCAAAACTT

GAAATCTTGCCAGAAGAGATAGGGGATTTAGAAAACTTGGAGTGGCTTGATGCCAGAGAT

ACTATAATCTCACAACCTCCGCCTTCCATTATCCGATTGAACAAGCTTAAATTCTTGAGT

TTTGCAAAACAAAAATCACAACTAGGCGTAGAAGATGAAGTGTACTTTGTGTTCCCTCCG

GTGGCTCAAGGATTACGCTCATTGGAAATTCTGAATCTCAGTTACTGCAATCTAATAGAT

GGAGGACTTCCGGAAGACATAGGATGCTTATCCGCTTTGAAAGAGTTGAATCTCAGTGGA

AATAATTTTGAGCATTTGCCTCGAAGCATAGCTGAATTTGGTGCTCTTCGATCCTTGGAC

TTAACAGAGTGCAAGAGTCTTACACAGCTTCCAGAACTTCCACCAGAATTAGATGCATTG

CGTGCAGATTGTCATATGGCTCTGAAAAGTATTCATAATATAGCAACCAAGAAGAAGAAA

TTGCAGCAGGTGACATTCAAACCACTGTATGATAGCGATGATACGTACAATGATTCAATC

TGTAATTTGTTTACCATTGTACATTCTGAGAAGAAGGTCCCAAGTTGGTTCCATTATCAG

GGAACGGATAGAATTGTATCAGTCAATTTGCCTGAAAATTGGTACGTATGTGATAACTTC

TTGGGATTTGTTGTATGTTACTTTGGCAGCGTAGTTGAAACCATAGCTCAATTGATTCTC

TTGTGTGATGATGGGATGTTGTCGATGACCCAGAAACTTGCCTCACACGACTATTCAAGA

TCTGAAATGAAATCGATGATTCATTTTTTCTTTGTACCTCTTGCTGGCTTATGGGATACA

TCTAAGGCAAATGGAAAAACACCAAATGACTTTGGGCTCATTAGGCTATCTTTTTCTGGA

GTAATGAAGGAGTATGGATTCCGCTTGTTGTATAAAGATGAACCTGAGCTTGAGGCCTTG

TTACAAATGAGGGAAAATAACAATGAACCAACAGAACAATGCATTGGGATAAGGAGGAGC

AGATATGACAATAGTGAACACCATGACTCTGTGACCAATAAAGCCAGTTCCTCCTCCTCT

TCTAAGAAACAAAGGTCACATTTCTAA

>NTBX_mRNA_108292_cds NTBX_mRNA_108292 gene_64864|id=AT5G17680.1

ATGGCATCATCTTGTGCTTCTGAGAGTACTTCACAGTTTCCTCGGTGGAACTACAAAGTC

TTTCTAAGTTTTAGAGGTGAAGATACTCGAAGAACATTTACAGGTCACCTCTTCAAAGGC

TTGGAAAACAGTGGAATATTTACGTTTCAAGATGATAAAAGGCTAGAGCATGGCGCATCA

ATATCAGATGAACTCTTGAAAGCTATCGAACAGTCTCAAGTTGCCCTCGTCGTTTTCTCA

AAGAATTATGCAACATCGAGGTGGTGCTTAGATGAGCTAGTGAAGATCATGGAATGCAAG

GATCAATGTGGACAGACTGTCATACCAGTCTTCTATGGTGTGGATCCATCACATGTTCGG

AAACAGAGGGAGAGCTTTGCTGAAGCCTTTGACAGACATGAAACAAGCTATAAGGATGAT

GATGAAGGAATGGAAAAGCTCCAAAGATGGAGGAATGCTCTAACTGCTGCCGCAAATCTA

AAAGGATATGATGTCCGTGACGGGATTGAAGCAGAGAATATTCAGCAGATTGTCGACCAA

ATTTCCAAATTGTGCAATAGTGCTACTTTGTCTTCTTTGAGCGATGTTGTGGGAATAGAT

ACTCATCTGGAGAAATTAAAGTCCCTACTTAAGGTAGGAATCAATGATGTTAGGATCATA

TTGGGGATTTGGGGCATGGGCGGTCTAGGCAAGATGACAATAGCAAGAGCCATTTTTGAC

ACTTTATCTCATCAATTTGAAGCTGCTTGTTTCCTTGCGGATATTAAAGAAAATGAAAAA

CTGCATTCCTTGCAAAACACCCTTCTCTCTGAATTGCTAAGAAAGAAAGATGATTACGTC

AATAATAAGCTTGATGGGAAGCAGATGATTCCGGACAAACTTTGCTCTAAGAAGGTGCTA

ATTGTGCTTGATGATATAGATCATAAAGATCATTTAGATTATTTAGCAGGTGATATTGGT

TGGTTTGGTAATGGCAGTAGGATTGTTGTAACAACTAGAGACAAGCATTTGATAGGGAAG

GATGATGCAATATATGAAGTGAGTGCACTACCTGATCATGAATCCATTCAATTGTTCTAT

CAGCATGCTTTCAAAAGAGAGGTTCCAGATGAGTGCTTTAAAGAGCTTTCATTGAAGGTA

GTAAATTATGCTAAAGGCCTTCCTTTAGCCCTCAAAGTGTGGGGTTCGTTGCTGCATAAT

TTACGACTAACTGAATGGAAAAGTGCTATAGAGCACATGAAAAGTAACTCTAATTCTGGA

ATTGTTGATAAGCTCAAAATTAGTTATGATGGATTAGAGCCCAAACAACAAGAGATGTTT

CTAGATATAGCATGCTTTTTGCGAGGGAAATATAAAGATTACGCCATGCAAATTCTTGAG

AGTTGTCATTCTGGAGTTGAATATGGATTGCGTGTCTTAATTGACAAATCTCTTGTATTT

TTTTCTGAAAAATGTCAGATTCAAATGCACGACTTAATACAAGAAATGGGTAAATATATA

GTGAACTTGCAAAAGAATCCGGGAGAACGCAGCAGACTATGGCTCGTCAAGGATTTCAAA

GAAGTGATCAACAACAATACAGGAACCATGGCAATGGAAGCAATCTTTCTTCCTTATTTC

AATTCGGGTATATTACGTTTTAGCAAAAAGGCCATGAAAAATATGAAAAGGCTTAGGATA

TTAAACATAGAGAGGTCGTTGACCTATGATGGTTCCATTGAGTATCTGCCCAACAACTTG

CGTTGGTTTGTCTTGTATGGCCATCCTTGTGAGTCACTGCCATCTACATTTGAACCCAAA

ATGCTTGTTCACCTTGAACTCTGGGGTAGTTCACTGCATTATTTATGGATGGAAACAAAG

CATTTGCCGTCTCTACGGAGGATAGATCTCAGCTCCTCTAGAAGCCTGATGCGAACACCA

GATTTCACGGGGATGCCAAATTTGGAGTATTTGAATATGTTATATTGTAGTAATCTTGAA

GAGGTTCACCATTCCCTGAGATGTTGCAACAAACTCATTCGGTTAAATTTGAATTATTGT

AAAAGCCTTAAGAGGTTTCCATGTGTTAACGTGGAATCTCTTGAATATCTGAGTTTAGAA

TATTGCTCTAGGTTAGAGAAATTTCCAGAAATCCACGGGAGAATGAAGCCGGAGATACAG

ATTCACATGAAACACTCTGGGATAAGGGAACTACCATCATCTATTACTCAGTACCAAACT

CATATTACCAAGCTAGATTTGAGCGGTATGGAAAAACTTGTAGCTCTTCCAAGCAGCATC

TGTAGGTTGAAAAGTTTGGTTAGTCTGAGTGTGTCGGGTTGCGTCAAACTTGAAAGCTTG

CCAGAAGAGATAGGGGATTTAGAAAACTTGGAGGAGCTTGATGCCAGCTGTACCCTAATT

TCACGACCTCCATCTTCCATCGTACGCTTAAGCAAACTTAAAATCTTTGATTTTGGAAGC

TCCAAAGATAGAGTGCTCTTTGAGTTCCCTCCGGTGGCAGAAGGATTTCGCTCATTGGAA

ACTTTGAGTCTCAGAAACTGCAATCTAATAGATGGAGGACTTCCGGAAGAGATTGCATCC

TTATCCTCTTTGAAAAAGTTGTATCTCAGTGGAAATAATTTTGAGCATTTGCCTCGAGGC

ATAGCCCAACTTGGTGCTCTTCGAATCTTGGACTTAAGAAATTGCAAGAGGCTTACACAA

TTGCCAGAATTCACGGGGATGCCAAATTTGGAGTATTTGGATCTGGAGGGATGTAGTTAT

CTTGAAGAGGTTCACCATTCCCTGGGGTGTTGCAAAAAACTCATTCGGTTAAATTTGAGT

TTTTGTAGTCGCCTTATAAGGTTTCCATGTGTTAATGTGGAATCTCTTAAATATCTGAAT

GTAGGAGAGTGCTCTCGGTTAGGGAAATTTCCAGAAATCCACGGGAGATTGAAGCCGGAG

ATACAGATTCACATGAAACGCTCTGGGATAAGGGAACTACCATCATCTATTTCTCGGTAC

CAAACTCATATTACCGAGCTAGATTTGAGAAGTATGGATAACCTTTTAGATCTTCCAAGC

AGATCGGTAGGTTGA

>NTTN90_mRNA_72592_cds mRNA_72592 gene_41020|id=AT5G17680.1:evalue=5.5:annot='disease resistance protein (TIR-NBS-LRR class), putative';id=Solyc08g005510.1.1:evalue=0.0:annot='Tir-nbs-lrr, resistance protein'

ATGGCATCATCTTCTGCTTCTACGAGTACTTTACAGTTTCCTCGGTGGAACTACAAAGTC

TTTCTAAGTTTTAGAGGTGAAGATACTCGAAAAATATTTACAGGTCACCTCTTCAAAGGC

TTGGAAAACAGTGGAATATTCACGTTTCAAGATGATAAAAGGCTAGAGCATGGCGCATCA

ATACCAAATGAACTCTTGAAAGCTATCGAGCAGTCTCAAGTTGCTCTCGTCATTTTCTCA

AAGAATTATGCCACATCTAGGTGGTGCTTAGATGAGTTAGTGAAGATCATGGAATGTAAG

GATCAATGCGGACAAACTGTCATACCAGTCTTCTATGATGTGGATCCATCACATGTTCGG

AAACAGAGGGAGAGCTTTGCTGAAGCCTTTGACAAACATGAAACAAGCTATAAGGATGAT

GATGAAGGAATGCTGAAGCTCCAAAGATGGAGGAATGCTCTAACTGCTGCCGCAAATCTA

AAAGGATATGATGTCCGTGACGGGATTGAAGCAGAGAATATTCAGCACATTATCGACCAA

ATTTCTAAATTGTGCAATAGTGCTACTTTATCTTCTTTGCGAGATATTGTGGGAATAGAT

ACTCACTTGGAGAAATTAAAGTCCCTACTTAAGGTAGGAATTTATGATGTTCGGATCATA

TTGGGGATCTGGGGCATGGGCGGTCTAGGGAAGACGACAATAGCAAGAGCCATTTTTGAC

ACTTTATCTCATAAATTTGAAGCTGCTTGTTTCCTTATGGATATTAAAGAAAATGAAAAA

AGACATCAACTGTATTCTTTGCAAAACACCCTTCTCTCTGAATTGTTAAGAAGAAAAGAT

GATTATGTCAATAATAAGGATGATGGGAAGCGGATGATTCCGGACAAACTTTGCTCTAAG

AAGGTGCTAATTGTGCTTGATGATATAGATCATAAAGATCATTTAGAGTATTTAGCTGGT

AATCTTGATTGGTTTGGTGATGGCAGTAGAATTATTGTAACAACTAGAGACAAGCATTTC

ATAGAGAAGAATGATGTAATATATGAAGTGACTGCACTACCTGAGAAGGAATCCATGCAA

TTGTTCAATCAACATGCTTTCGGAAAAGAATTTCCAAATGAGCATTTTAAGGAGCTTTCA

TTGGAGGTTGTAAATTATGCTAAAGGCCTTCCTTTAGCCCTCAAAGTGTGGGGTTCTTTG

CTGCATAACCTAGGCTTAACTGAATGGAAAAGTGCGATAGAGCACATGAAAATTAATTCT

AATTCGGAAATTGTTGAAAAGCTCAAAATCAGTTATGATGGATTGGAGCCCATCCAAAAA

GAGATGTTTCTAGATATAGCATGCTTATTGCGAGGGCAAGAAAAAGATTACGCCATGCAA

GTTCTTGAGAGTTGTCATATTGGAGCTGAATACACATTGCGTATTTTAATTGACAAATCT

CTTGTGTTCATCTCTGAAAATGATGAGATTCAAATGCACGACTTTATAGAAGATATGGGT

AAATATATAGTGAACTTGCAAAAGAATCCGGGAGAACGCAGCAGATTATGGCTCGCCGAG

GATTTTGAAGAAATCATGACCAATAATGCAGGGACCACAATGGAAGCAATCTTTCTTGCT

AATTTCAATAATGGTAGATTACGCTTTAGCAAAAAGGCCATGAAAAATATGAAAAGGCTT

AGGATATTTAACATTAATGTCGACATATTTGAGGAGAGGTTGTCGCCCCGTTATGCCATT

GAGTATCTGCCCAACAACTTGCGTTGGTTTGTGTGGAAAAACTATCCTTGGAAGTCATTA

CCATCTACATTTGAACCCAAAATGCTTGTTCACCTTGCACTCTCGGATAGTTCACTGCGT

TATTTATGGATGAAAACAAAGCATTTGCCGTCTCTACGGAGGATAGATCTCAGCGGGTCT

AGAAGACTGATGCGAACACCGGATTTCACGGGGATGCCAAATTTGGAGTATTTGAATCTG

TTTTGTTGTGATAATCTTGAAGAGGTTCATCATTCCCTGGGATGTTGCAACAAACTCATT

CGGTTAGTTTTGTTTCATTGTCGTCGCCTTAAGAGGTTTCCATGTGTTAACATGGAATCT

CTTGAATATCTGGATTTAATATATTGCTCTAGTTTAGAGAAATTTCCAGAAATACACGGG

AGAATGAAGCCGGAGATACAGATTCACATGCGAAGCTCTGGGATAAGGGAACTACCATCC

TCTATTTTTCAATACCAAACTCATATTACCGAGCTAAATTTGAGTTATATGAAAAACCTT

TTAGCTCCTCCAAGCAGCATCTGTAGGATGAAAAATTTGGTTATTTTAGATGTGTCGGAG

TGCTCAAAACTTGAAAGCCTGCCAGAAGAGATAGGGGATTTAGAAAACTTGGAAAAGCTT

GATGCCAGCTGGACTCTAATTTCACGACCTCCATCTTCCATCGCACGCTTGAACAAACTT

AAAGTATTGGATTTTGGATTCGTACGGGATAGAGTGCACTTTGAGTTCCCTCCAGTGGCT

GAAGGATTACGCTCATTGGAAATTCTGAATCTCAGGTACTGCAATCTAATAGATGGAGGA

CTTCCGGAAGACATTGGATCCTTATCCTCTTTGAAAGAGTTGAATCTCCATGGAAATAAT

TTTGAGCATTTGCCTCGAAGCATAGCCCAACTTGGTGCTCTTCGATCCTTAGACTTATCA

TATTGCCAGAGGCTTACACGGCTACCAGAACTTCCCCCTAAATTAAATATATTGCATGTT

GATTGTCATATGGCTCTGAAATTTATCCATGATTTAGTAACAAAGAGGAAGAAACAACAG

AGGGTGATATTCAAACCACTGTATTATAAGGATGATGCACACAACGATGCTATATATAAT

TTGTTTGCACATGCTCTGTTTCAGAATATCTCTTCCTTGAGGCATGACATCTCTGCTTCA

GATTCATTGTCCGAAAGTGTGTTTACCATTGTGCATATTGAGAGGAAGATCCCAAGTTGG

TTCTTCTATCAGGGAACGGATAGTAGTGTATCAGTTAATTTGCCTGGAAATTGGTATATA

CCTGATAAATTCTTGGGATTTGCTGTATGTTACTCTGGCAGATTAGTTTACACTGCAACT

CAATTGATTCCCGTAGGTAATGACACAATGTCGTGGATGATCCAGGAACTATACTTATCC

AACCCTTCAGAATCTGATTCAGAATATTATATACGTTTTTTCTTTGTACCTTTTGCTGTC

TTATGGGATACATCTAAGGCAAATGGAAAAACACCAAATGACTATGGGATTATTAGGCTA

TCTTTTTCTGGAGTAATGAAGAAGTATGGACTTCGTTTGTTGTATAAAGATGAACACCAT

GACACCCTGACCGATGCAGCCAGTTCCTGTCGCATACTGTAA

>NTTN90_mRNA_93173_cds mRNA_93173 gene_52683|id=AT5G36930.1:evalue=1e-133:annot='Disease resistance protein (TIR-NBS-LRR class) family';id=Solyc01g102850.1.1:evalue=0.0:annot='Tir-nbs-lrr, resistance protein'

ATGCCTCTTGCCAAAAGTCAATCAGAAAAAATGTCTCAGTTTGTTCATCATGTATTCTTG

AGTTCCAGAAGCAAAGAAATAAGCAAGACATTTGGAGATCATCTTCAAACAGCTTTGCTA

AATGCTGGTATTCGCGCATTCAAGTTTGATGAAGAGCTTGAGGAAGAAGAGGAACACCAG

AAAAAATTGCAGAAAGTAATTCAGGAATCCAGAATTCTTATAGTTGTTTTTTCGAAAGAC

TATGCTTCTTCAGAGAGATGCCTTGATGAACTTGTGTATAGTCTTGAAAGCAAGAAACGT

TTTGGACATTTTGTTCTTCCTGTGTTTTATGATGTGGATCCATCAGAAGTCCGAAAGCAG

AAAGGCAGCTTTGAAGAAGCTTTCTTTAGATATGAAGAGAAATATAAAACAGAGAACAAC

GAAAGGAGAAAGAAATGGATGGAGAAGGTGGACAAATGGAGGGCTGCCTTTAGAGAAGTT

GCTGATTTGGGAGGAATGGTCTTACAGAATCAAGCTGATGGGTATGAATCAAGGTTTATT

CAAGAAATCGTCAAGGTGGTTGCAAGTAAAATGAACCGCACAATTTTAAGTGTTGCCCTC

CATCCAATTGGAATAGATTCTCGAGTCAAAGACCTTAACTTATGGTTACAAGAGGAATCA

ACCACTGTAGATATCCTGGCAATACATGGTATGGGAGGAATTGGGAAATCTACAATAGCG

AAAACAGCTTATAACCTGAACTTTGACAGATTTGAGGGAAGCAGCTTTCTTGCAGATGTG

AGAAAAGCTTTAGAAAAGTACGATGGTTTAGCTCGTCTACAAAGACAACTTCTCTCAAAT

ATTCTTGGGAAGAATGTTGAAAAGTTGTACAATGTCAATGAAGGGGCTGTCAAGATTCAA

GAAGCCATCAGCTGCAAAAGAGTTCTTGTTGTTCTCGATGATGTGGACGAGTTAGATCAG

CTAAATGCTGTACTTGGTATGCGGCAATTGTTTTATCCAGGTAGTAAAATTATCATAACA

ACAAGAAATGGGCACTTGCTAAATTCCAGTGAAGCCTGTAGATGTAAGATGTATAAGCTG

AACTCTTTGGATGCTAGAGAATCACTACAGCTGTTCAGTTGGCATGCATTTGGTGAAAAA

TACCCTCCCTTAGATTATATGAATCTTTCCAGAGACGTTATAGTTCATTGTCAGGGAATT

CCATTAGCTCTTAAAGTTTTGGGTTCCTCTCTTTGTGACAGAAGCACAGAGGTGTGGGAA

AGCGCATTAAGGAAATTGAAAGCGATTCCAGACAGTAAAACCCTGGAAAAACTAAGGATA

AGCTATGAATGTCTACCAGATGATAATGTCCAGAACCTATTCCTTGATATTGTCTGTTTC

TTTGTCGGGAAGGACAAAGATTATGCAGTAACAATACTTGACGGATGTGGGTTCTTTTCA

GTTGTTGGAATTCAGATTCTTGTTGATAGATGCTTATTAGCAATTACTGACAATAAAAAA

CTGATGGTGCATCAACTGCTTCAAGACATGGGAAGAGAAATTATTCGCAAAGAATCCCCT

CGAGAGCCTTGGAAAAGAAGCAGAATTTGGCTACATAAAGATGCCTTTAACATATTGCAG

GCAAACACTGGTACTGAAAATATCCAAGGCCTAGTGCTTGACATGCGACTGTTGAAGGAA

GTCAAATATGTCGGATCAAATCTAAGGGTAAATGACGCTAGACACAACCGCTCTAATGAT

CCTGCAGGCGAGAGATCGCTGGTGGTGAGTGACATCAACTCACAAAAGCGACGGAGGTTA

ACTGTTTTCAAGCTGTTCACAAATGTCTTTTCAGAAATTTCTAATGGTGTACAGTTTGAA

CTTGATGCATTTTCAAAGATGAAGAAACTGAGAATTCTACAGCTTACTGATACAAGGTTC

ACTGGCAGCTATAGTTGGTTTCCTAAGAATTTAAGATCACTTCATTGGCGTGGATTTTAT

TTGAAATCCATTCCGAAGGATTTTCCTTTGGAGAGTCTGGTAGCTCTTGATATGAGGCAC

AGCAGCTTGGAACAAGCCTGGATCGGAACCAGGGTGCTCAAATTATTAAAAGTTCTCAAT

CTCAGTCATTCTCATTTCCTAGGAAGGACTCCTGATTTTTCTGGGCTTCCTAATCTGGAA

AGACTCATCCTAAAGGACTGCATAAGATTGTTCAGTATTGATGAGTCGATAGGAGACGTT

AAGGCACTTGTTCTATTGAACTTGAGAGACTGTAAGAATCTGAGGAATCTGCCAAGAAGT

TTTTGTAAGCTCAAATCCTTGGAAACACTTAACATTTCTGGCTGCTCTAGACTTTCTATA

TCAACAATGGAATTAGGAAAGCTGGAATCCTTAACAACCCTCAACGCAGATGAAATAAGT

TACAATCAGGAGAAATCATGGACTGCGCTCTGGCAATCTTGGTCATCAAAATTAAGAAAA

TCCCCTGATTGTAACTTGTCTTCTTTATCAAGTTCCCTGGTTAACTTGAGTCTTGCAAAA

TGCAGGCTAACGGATGATTTTCTATCCGTTGGTCTTTGCAATCTTTCCTCATTAAGGCAT

TTGAATCTAAGCGAAAACCTGATTTGTAACCTGCCACAAAATATCACAAATCTAAGTATG

CTTCAGGAACTTTGGCTAGATGCATGCTCAAGTCTCCAATCGCTTCCCAAGCTACCGCCG

AGCCTCATCAAGCTGAAGGCTATAGACTGCACATCACTAGAAAGAGTTACAAATCTGCCA

AACCTATGGGAAACTCAAACTCTACTCTTGGATGTTACAGGCAGCGAGAAACTAACTGAG

ATTCCTGGACTTTTCAAGCTAGAGCCAGTCGGTAATTTTAAGGAGGAAATGTTGAACATT

TTAGGCCATCTCAACCTGGAAGATATAGAAAATGCAGAGGTAGAACTATTCAATAGGCTT

ACAGACACGAAGAGGAAATATCCTGTGCAGGGACTCCACGAGTTTGGAATCTTCAGCACT

TATTTTCTTGGAAGTGAGATTCCAAGTTGGTTCAGCAACAAAGATTTCAACGCTGGGGTG

AAAACCAACTTGGGAACTGGTATTCTTTTTTCATCAAACTGA

>NTK326_mRNA_16507_cds NTK326_mRNA_16507 gene_9366|id=AT5G17680.1

ATGAGAGCAATTGAAGAGTCGCGCATAGCTTTGATTATATTCTCCAAAAACTATGCTAAT

TCAAGATGGTGCTTAGATGAATTAGTGAAGATCATGGAATGCAAGAATTTGAAAGGACAA

ATTGTGTTTCCAGTGTTCTACGATGTTGATCCATCAACAGTGAAGGAACAAAAATTCAGC

TTTGGAGAAGCATTTCGTACTCATGAAGCCCATGGCTGTTTCAAGGTGCAAAAATGGAGA

GCAGCACTGGAGGAAGCAGCTAATTTATCTGGCTGCGATTTGCCAAATACTGCCAATGCG

CATGAAGCTAAAGTCATAAAGCAAATTGTGGAAGATATGATGGCTAAATTAGGTGGTCAG

AGGCATGCAATCAATGCTGAAAATCTTGTTGGAATGGAGTCGCAAATGCAGAAAGTGTAT

AAAATGCTTGGCATCGGGTCTGGTGGAGTTCACTTTGTTGGAATATTTGGAATGAGCGGA

GTGGGAAAGACAACTTTAGCGAGAGTCATTTATGATAACATTTCGAGTCAATTTGAGGGT

GCTTGTTTTCTTCATGAGGTTAGAGACCGTTCAGAAAAACAAGGCCTAGCGCGATTACAA

GAGATACTTCTTTCCAAGATCCTTGTCATAAAAGACCTAAGGATCAACAATTTATTTGAA

GGACTTAATATGCAAAGACAGAGACTACGGTTCAAAAAGGTTCTTCTTGTTCTTGATGAT

GTTGATCACATAGATCAGTTAGATGTTTTAGCTCAGAAGCGCGAATGGTTTGGTTCTGGA

AGTAGAATCATCATAACAACTAAAGACAAACACTTGCTTGTTAAGCATGATGTGGAAAAG

ATATACAAAATGAGAACATTAAGTGAAGATGAAAGTCTACAACTATTTAAACAGTATGCT

TTCAAGAAGAACCATCCAACCAAGAAATTTGAGGATCTCTCAGCTCAAGTGATAAAGTAT

AGTGCTGGACTCCCCTTGGCTCTGAAAGTCCTGGGCAGTTTCTTGTATGGAAGAGATTTG

GCTGAATGGAGAAGTGAAGTAGAAAGATTGAAACAAATCCCGGAAGATGAAATTTTGAGG

AAACTCGAACCAAGTTTCACTGGACTCAAAAGTATCGATCAAATGATATTCTTAGACATT

GCGTGTTTCTTTACAGGGAAGAAGAAAGATTCAGTGACTAGAATACTTAAGAGTTTTAAT

TTTAGCCCTGTTATTGGCTTAAAAGTTCTCATGGAGAAATCTTTGATTACTATTTCAGAA

GGTAGGATTTTAATGCACCAATTGATACAAGAAATGGGCTGGCACATTGTTCGTCGAGAA

GCTTTCGATTATCCCAGAAAATATAGTAGGTTATGGAAGTCTGAAGATATTTCTCATGTA

CTTGCAAGAAATATGGGCACAGAAAAGATCGAAGGCATATCTTTGAACTTGACTAAGACG

CTCACAGATATTTCTCATGCACTTGAAAGAAATTTGGGCACAGAAAAGATCAAAGGGATA

TCATTGAACTTGACTATCGTCAAAGAAGTGAATGTTAGTGCAACAGCCTTTATGCAGATG

ACCAGACTGAGGTTTCTCAAAATCAAGAATGCATATGTTTCTCAGGGTCCGGACATTCTT

CCTAGTGAGTTGAGCTGGCTTTCTTGGCACGGATATCCTTCAAAAAGTCTGCCAATTAGC

TTTCAGGGAGAACGACTCGTTAGTTTGAAGTTGAAAAATAGTCGCATCATACAACTTTGG

AAAGGCTCCAAGGTTCTAGGACAACTGAAGTACATCAACCTTAGCCATTCACATAAGCTA

ATAAGGACTCCAGATTTTTCGGGTACCCCTAATCTTGAAAGGTTGGTTCTTGAAGAGTGC

ACGAGTTTGGTAGAAATCAATTTTTCTGTTGGAGATCTCAAAAAGCTAGTCTTACTCAAG

TTGAAGAATTGCATCAATTTAAAGACCCTGCCAAAGAGTATTCAATTGGAAAATCTTGAG

GTTCTTATTCTATCAGGCTGCTCAAAGCTAAAACTATTCCCAGAAATAGAAGATGGAATG

AATCGTTTATCAGAACTATATTTGGAAGCGACTTCTTTGAGTGAACTACCCGCATCAGTT

GAGAAACTATCAAGAGTTAAAGTGATAAATCTAAGCTCATGCAAGCATCTTGAGAGTCTT

CCAAATAGTATTGTTAGGTTGAAATGTCTTAAAGAACTTAATGTGTCGAGGTGCTCAAAA

CTTAAAAGTTTACCAGATGACTTGGGTTCTTTAGTCGGATTGGAGGGGCTCCATTGTGAT

GACACACCGATCCAAATGATACCATCCACCATTTCCCTTCTAAAGAACCTTAAACACTTA

TCTCTCCGTCGATGTAATGCTTTGGGTTTGCAAGTAAGGAGTTCAATCTCAAGAGAATCT

ATGGGACTAGTTTTCTCTAATTTATCGGGTCTTTGTTCATTGACAATGCTGGATATAGGT

GGCTGCAGCATTTCAGATGGAGGCATCCTATGTAATCTTGGGTTCCTACCATCTTTGGCG

GAATTGAATCTTGGTGGTAACACGTTTACCAATATCTCAGCTTCAAGCATCAGTGGCCTC

ACTCGACTAAAGGTTCTTCAATTGGTTGGCTGTAGTAGGCTTGAGCATTTCCCAGAACTT

CCTCAAGCTATAGAAGAGGTGCATGCCGATGAATGTATATCTTTGAAGAGTATCGATCAA

TTAGCAAAATATCCAACATTACGCCGACTTTCACTTAGCCAATGTCATCAGCTTCATGAT

ACTGACATGGTTGATGCATTATGGAGCAACATGCTCAAGGGACTATACGTGCTACGAAAT

GATCTCAGCATTTGCATCCCTGGATCGCAAATTCCTATGTGGTTTACATATAAGAACTTT

GGGGAAAATGTTACACTGACTCTTGCCAATAATTGGTACACTGATAACCTCTGGGGTTTT

GCTTTCTGTATTGTTTTTGAACGTATGGAATGGTGCGGTCTATATGATGGTTACCTACAA

CCATCACTTGGATTTCCAGTTAACCTTAAATTCAAAACATATGATGGTAAGGAAGGCGAT

ATACGCAGCATTATTGGCATCAAAGGAGGTGATATGTCAATTCGGAACTCAGAGCACACT

CTCCTTTCTTATGTACCATCTCGTCGTTTTCTGCAACCTTACAATAACGAGGTTTACTGT

CCCAACGACTGGATCGAAATTGTGGCATATTCGACTGTACAATTCGACAGTAAAGCTTGG

GGGACGCGTCTTGTGTATTTGGACGATATTATTGAAGCATGA

>NTBX_mRNA_99861_cds NTBX_mRNA_99861 gene_60009|id=AT5G36930.2

ATGGATACTCAATTAGTTCGAGGAAAATCATCTACCTCTTCTCCCTTCTCTTATGAAGTA

TTCCTGAGTTTTAGAGGGGAAGACACCCGAAAAACATTCACCGGTCATCTTTATTCCAAA

TTGTGTGATGTTGGAATTAATACCTTCATTGACGATGAGGAATTGAGAAAGGGTGATGTG

ATTTCAAGTAAACTAGAGAAAGCAATTGAAGTGTCGAGGATTTCCATTGTAGTTTTCTCG

AGAAATTATGCTTCCTCTAGTTGGTGTCTAAATGAGCTAGTTAAAATTCTCGAATGCAAA

GAGAAATTAAAGCAGATGGTTTTGCCTATTTTCTACGATGTTGATCCTTCTGAAGTGCGA

AAAAAAACTGGGTTATTTGGGGAAGCTTTGGCTAAGCACAAGGAGCGACCATTTGGAGCT

CAAAGGGTGGAGAAATGGAGAGCTGCACTTACTGAAGCTGCAAATTTATCTGGATGGGAT

TTGCAAAATGTTGCTGACGGGCATGAATCAAAGTTTATTGAAAAAATTATACAAGTAGTC

CTACAAGAGGTCAACCAGACACCTCTAGATGTTGCTTGGCACCCAGTTGGTGTAGATTAT

CGTGTCAAAGATATAGAGTTGTTATTGCAAAATGAATGTGAAGATGAAGTTCGCATGATT

GGTATTCATGGAGTTGGTGGCATAGGGAAAACAACTCTGGCAAAAGCTATCTACAATCGA

ATGTTTCGACTCTTCGATAGTAGTTGCTTCCTTTCAGATGTTAGATCAGAAGCTGAAGAA

TTTGGTCTTGTCAAGCTACAAGAGAATCTTCTTCAACAAGTTCTCAAAACCGAGGACATC

AAAGTTGGGAGTATTGCTCAAGGCGTTAATCTAATCAAAGCAAGACTTGGGTCAAAGAAG

GTTCTAATTGTTCTTGATGATGTTGACCATAAAAGACAGTTAGAAGCCTTGACAAGAGAA

AGACATTGGTTTGGTTCAGGTAGTTTAATAATCATTACCACCCGAGACGAGAGATTGCTA

TGTCAGCTTGGAGAAAAAGAGAGATATGAGGCTGAACTATTAAATGGCAATGAAGCTATG

TTACTTTTTTGTTGGCATGCTTTTGACAGTCATTTTCCACCACAAGATTATGTTAATTTG

GCACACGACGTAATTGAATATTCAGGTAGGCTACCATTAGCTCTTGTGACATTGGGGTCA

CATTTACAAGGAAGTTCTGTTGAAGAATGGGGATATGAATTAGAAAAACTAAGAGCAATT

CCTCATTGTGATATCCAAAAGATTCTCAAGATAAGCTTTGATGGACTTGATGATGAAACA

CAGGCAGTTTTTCTTGATATTGCGTGTGCCTTCCAGGGGTTATTTGAGTATGAAATTACC

GAAATATTAAATGCATGTGGCTTTCATGCTGAAATTTCAATTGCAACTTTAGTCCAAAAA

CACTTGCTCCAAAGGGATGATCTCTATTTGGTGATGCATGATCTAGTGCGAGATATGGGA

AGAGAAATTGTTCGCATGGAGTCAGCTCGAGACCCTGGAAAACGGAGTAGATTGTTCATC

CCTCAAGAAGTCTGTGATGTTCTACAAGGAAATAAAGGTTCAAAAAAGGTAGAAGTACTG

AAAATAGATCGACGAGCATTTGAGAGACAGAACTTGAGCACCAAAGCATTTAAGAAAATG

AAAAACCTTAGGGTTCTTATAATGAATGAGTTACATATTAGTGGAGGTTTTGAGCTGTTA

TCCAAGGAGCTCAGATGTTTGTCTTGGAAAAGATGTCCCTTAAAATGTGTACCATCGAAT

TTTCCAGCTGAGAATCTTGTAGTTCTAGATATGCATGAGAGTGCTATCCAAGAATTTCAA

TTGAATTTGCAGTGTTGTAGAAGTTTGAAGAAGTTGGATCTCTCTTATTGCAAGCAACTC

AGAAGCACTCCAGACTTCACTGGTTCACGAAGTCTTGAGAATTTGCTGCTTGGTGGTTGC

TCAAGTCTGATGGAGATCCATCCATCAATAGGAAATCTGAACAGACTAATTAAACTATAT

ATGCATGATTGCGAAAAACTTAGGGATCTTCCAAGCAGCATATGCCAGCTAATATCCGTT

GATTACTTGGACATTAATTCCTGCTCATCTATAAAAACTCTGCCAGATAACATTGGAGAT

ATGAAAAGTCTAAGACATCTTGATGCATGTCGTACGGGTATAAAACAATTGCCTAAATCT

GTTGAAATGCTAAAAAATCTTGTAGCTTTGATTGTGGGAGGTCGAAAGTTTGAGGCCAAA

AGGAGTATTTCTGGAAGAGGAGTCCATCAGATACAATATTCCCTGTCAACTTTTGTATCC

AGATTGAGCCTTACATATTTGTCTGAGGCTGATATTCCTAGGAATATTGGGAGCTTATCC

TCCTTGGTGATTTTAGATTTGAGTGGCAACAGTTTCTATTGTCTACCCTTTGATCTTTCT

ATGTTACGATTATTGGAGGAGTTGTATTTGAATGACTGTGAGAATCTTCAAACACTCCCA

TCAGTATCAAATTTAGAGAAGCTTGAAAGAATTGAGCTTAAAAATTGCCAAAAATTGGTC

AAGATTACGGAGTTGGACAACCTCCCTTCTATACGGTGGATCAACATGATGAATTGTAGT

TCTATGCAGAATCCATTCAATGAAGGCTTATTTAGTGCACCTGCTCTATATGCATCTAGA

AATGATCGAAATATGGTTAGTCTCTCTCTCTCTCTCTCTCTCACACACACACACACTCAT

GTTAATGATCTGAGTCTTTATCCGATGCAGCGTGATTTAGAAATTTATCTCGAATGCAAT

GAGATTCCAGAATGGTGCAGGAATCAAGTAACAGCTTCGTCTATCTGTTTGACTATGCCA

ACACATAATAATGATGAGTGTAACTTCTTAGGAATGGTTCTCTGGTTTGTTATCGACTCT

TTGGATGCAGCCCTTTATCCAAGCTTCTGGATTAGTATTGGCCATAAAGAGACTTTAATT

GTTCCATACCCACCATTAGTATTGCTTCACGATGGACAGAGAGAAGTATCATGTGTATAT

TACATATCTTTCTTACATAAAGCTTTTGATGGCGGGGAAAGGATAGAAGTGGGGCCTAGA

GGAGTTACAGTAAAGAAGATAGGGATCCATCTGTTATATTTAGACCAAAATGGTAATGTT

ATATCTTTGCCGGGAGACGTGGATCATTCTTATGTCAGGAATTGGTGGAAATAG

>NTK326_mRNA_107151_cds NTK326_mRNA_107151 gene_63326|id=AT5G36930.2

ATGCCTCTTGCCAAAAGTCAATCAGAAAAAATGTCTCAGTTTGTTCATCATGTATTCTTG

AGTTCCAGAAGCAAAGAAATAAGCAAGACATTTGGAGATCATCTTCAAACAGCTTTGCTA

AATGCTGGTATTCGCGCATTCAAGTTTGATGAAGAGCTTGAGGAAGAAGAGGAACACCAG

AAAAAATTGCAGAAAGTAATTCAGGAATCCAGAATTCTTATAGTTGTTTTTTCGAAAGAC

TATGCTTCTTCAGAGAGATGCCTTGATGAACTTGTGTATAGTCTTGAAAGCAAGAAACGT

TTTGGACATTTTGTTCTTCCTGTGTTTTATGATGTGGATCCATCAGAAGTCCGAAAGCAG

AAAGGCAGCTTTGAAGAAGCTTTCTTTAGATATGAAGAGAAATATAAAACAGAGAACAAC

GAAAGGAGAAAGAAATGGATGGAGAAGGTGGACAAATGGAGGGCTGCCTTTAGAGAAGTT

GCTGATTTGGGAGGAATGGTCTTACAGAATCAAGCTGATGGGTATGAATCAAGGTTTATT

CAAGAAATCGTCAAGGTGGTTGCAAGTAAAATGAACCGCACAATTTTAAGTGTTGCCCTC

CATCCAATTGGAATAGATTCTCGAGTCAAAGACCTTAACTTATGGTTACAAGAGGAATCA

ACCACTGTAGATATCCTGGCAATACATGGTATGGGAGGAATTGGGAAATCTACAATAGCG

AAAACAGCTTATAACCTGAACTTTGACAGATTTGAGGGAAGCAGCTTTCTTGCAGATGTG

AGAAAAGCTTTAGAAAAGTACGATGGTTTAGCTCGTCTACAAAGACAACTTCTCTCAAAT

ATTCTTGGGAAGAATGTTGAAAAGTTGTACAATGTCAATGAAGGGGCTGTCAAGATTCAA

GAAGCCATCAGCTGCAAAAGAGTTCTTGTTGTTCTCGATGATGTGGACGAGTTAGATCAG

CTAAATGCTGTACTTGGTATGCGGCAATTGTTTTATCCAGGTAGTAAAATTATCATAACA

ACAAGAAATGGGCACTTGCTAAATTCCAGTGAAGCCTGTAGATGTAAGATGTATAAGCTG

AACTCTTTGGATGCTAGAGAATCACTACAGCTGTTCAGTTGGCATGCATTTGGTGAAAAA

TACCCTCCCTTAGATTATATGAATCTTTCCAGAGACGTTATAGTTCATTGTCAGGGAATT

CCATTAGCTCTTAAAGTTTTGGGTTCCTCTCTTTGTGACAGAAGCACAGAGGTGTGGGAA

AGCGCATTAAGGAAATTGAAAGCGATTCCAGACAGTAAAACCCTGGAAAAACTAAGGATA

AGCTATGAATGTCTACCAGATGATAATGTCCAGAACCTATTCCTTGATATTGTCTGTTTC

TTTGTCGGGAAGGACAAAGATTATGCAGTAACAATACTTGACGGATGTGGGTTCTTTTCA

GTTGTTGGAATTCAGATTCTTGTTGATAGATGCTTATTAGCAATTACTGACAATAAAAAA

CTGATGGTGCATCAACTGCTTCAAGACATGGGAAGAGAAATTATTCGCAAAGAATCCCCT

CGAGAGCCTTGGAAAAGAAGCAGAATTTGGCTACATAAAGATGCCTTTAACATATTGCAG

GCAAACACTGGTACTGAAAATATCCAAGGCCTAGTGCTTGACATGCGACTGTTGAAGGAA

GTCAAATATGTCGGATCAAATCTAAGGGTAAATGACGCTAGACACAACCGCTCTAATGAT

CCTGCAGGCGAGAGATCGCTGGTGGTGAGTGACATCAACTCACAAAAGCGACGGAGGTTA

ACTGTTTTCAAGCTGTTCACAAATGTCTTTTCAGAAATTTCTAATGGTGTACAGTTTGAA

CTTGATGCATTTTCAAAGATGAAGAAACTGAGAATTCTACAGCTTACTGATACAAGGTTC

ACTGGCAGCTATAGTTGGTTTCCTAAGAATTTAAGATCACTTCATTGGCGTGGATTTTAT

TTGAAATCCATTCCGAAGGATTTTCCTTTGGAGAGTCTGGTAGCTCTTGATATGAGGCAC

AGCAGCTTGGAACAAGCCTGGATCGGAACCAGGGTGCTCAAATTATTAAAAGTTCTCAAT

CTCAGTCATTCTCATTTCCTAGGAAGGACTCCTGATTTTTCTGGGCTTCCTAATCTGGAA

AGACTCATCCTAAAGGACTGCATAAGATTGTTCAGTATTGATGAGTCGATAGGAGACGTT

AAGGCACTTGTTCTATTGAACTTGAGAGACTGTAAGAATCTGAGGAATCTGCCAAGAAGT

TTTTGTAAGCTCAAATCCTTGGAAACACTTAACATTTCTGGCTGCTCTAGACTTTCTATA

TCAACAATGGAATTAGGAAAGCTGGAATCCTTAACAACCCTCAACGCAGATGAAATAAGT

TACAATCAGGAGAAATCATGGACTGCGCTCTGGCAATCTTGGTCATCAAAATTAAGAAAA

TCCCCTGATTGTAACTTGTCTTCTTTATCAAGTTCCCTGGTTAACTTGAGTCTTGCAAAA

TGCAGGCTAACGGATGATTTTCTATCCGTTGGTCTTTGCAATCTTTCCTCATTAAGGCAT

TTGAATCTAAGCGAAAACCTGATTTGTAACCTGCCACAAAATATCACAAATCTAAGTATG

CTTCAGGAACTTTGGCTAGATGCATGCTCAAGTCTCCAATCGCTTCCCAAGCTACCGCCG

AGCCTCATCAAGCTGAAGGCTATAGACTGCACATCACTAGAAAGAGTTACAAATCTGCCA

AACCTATGGGAAACTCAAACTCTACTCTTGGATGTTACAGGCAGCGAGAAACTAACTGAG

ATTCCTGGACTTTTCAAGCTAGAGCCAGTCGGTAATTTTAAGGAGGAAATGTTGAACATT

TTAGGCCATCTCAACCTGGAAGATATAGAAAATGCAGAGGTAGAACTATTCAATAGGCTT

ACAGACACGAAGAGGAAATATCCTGTGCAGGGACTCCACGAGTTTGGAATCTTCAGCACT

TATTTTCTTGGAAGTGAGATTCCAAGTTGGTTCAGCAACAAAGGTGAAGAAAGCATATTG

ACTCTGAAAGTGGATTCTCATACTAATACAAAGATAATAGGACTTAATATCTGCGTTGTA

TATTCACGTTCCACTCATCATAGATTTCAACGCTGGGGTGAAAACCAACTTGGGAACTGG

TATTCTTTTTTCATCAAACTGAGTAATGTGACCAGCGGTGACAAGTGGATTTATGCTCCA

ACATTCATAGGCATTCCAGGATCAAATGAAGATCTGACATTTTTGTGCCACTGGAAATTT

GGAAAGTATATACAAGCTGGTGACGAGATTAATGTCTCTGTACTTGGTTGGAGTTATACT

TTTCAAATGAAAGAGTTTGGAGTCAGCATTGAATGTGACAGACCAGAAACAGACCAGCAC

TTAACATCAACTAGTGAATCAAAAGAGCTAGCAATCCAACATGGGTATCCAAGTACTTCT

ATGCAAGAGCATTGTGTCATGGGAAGTTATATGCCTGTATATCAGGTTGCAGCTCGCCAT

TACTACTTTTCTCACCCAGACTACTTTTTGCTTAAATCCAATGAACATACAGCTGTGAGG

TCGATCTTGTACGAGAACTTATTTGAGGATTTTGTGCACAGTACTACAGGTACCAAAGAT

GCAGGAGCAGAAGATGATAGCGATTTTGATTTCGATGATGAGAACTACACTGAGGAGGCT

GCCTTGGCAGAACTAGAGGACAACTTAGAATGGCCTTGGTAA

>NTTN90_mRNA_86966_cds mRNA_86966 gene_49172|id=AT5G36930.1:evalue=0.0:annot='Disease resistance protein (TIR-NBS-LRR class) family';id=Solyc09g092410.2.1:evalue=0.0:annot='Tir-nbs-lrr, resistance protein'

ATGGATACTCAATTAGTCAGAGGAGAATCATCTCACTTCTCTTATGAAGTATTCCTGAGT

TTTAGAGGTGAAGACACCCGAAAAACATTCACTGGTCATCTTTATTCCAAATTGTCTGAT

GTTGGAGTTAATACCTTCATTGACGATGAGGAATTGAGAAAGGGTGACGTGATTTCAAGA

GAATTAGAGAAAGCAATTGAAGAGTCAAGAATTTCCATTATTGTTTTCTCAAGAAATTAT

GCTTCCTCTAGTTGGTGTCTAAATGAACTAGTTAAAATTCTTGAATGCAAAGATAAACTA

AAGCAGATGGTTTTGCCTATTTTCTATGATGTTGATCCTTCTGAGGTACGAAAGCAAACT

GGGTTATTTGGTGAATATTTGGCTAAACACAAGGAACGACCATTTGGAGCTCAAAGGGTG

GAGAAGTGGATAGCTGCACTTACTGAAGCTGCAAATTTATCTGGATGGGATTTGCAAAAT

GTTGCTGACGGGCATGAATCAAAGTTTATTGAAAAAATTATACAGCAAGTCCTACAAGAG

GTCAACCAGACACCTCTAGATGTTGCTTGGCACCCAGTTGGAGTAGATTCTCGTGTCAAA

GATATAGAATTGTTATTGCAAATTGAATGTGAAGATGAAGTTCGCATGATTGGTATTCAC

GGAGTTGGTGGCATAGGGAAAACAACTCTGGCAAAAGCTATCTACAATCGAATGTTTCGA

CTCTTCGATAGTAGTTGCTTCCTTTCAGATGTTAGATCAGAAGCTGAAGAAGTTGGTCTT

GTCAAGCTACAAGAGAAACTTCTTCAACAAGTTCTCAAAACTGAGGACATCAAAGTTGGA

AGTGTTGCTCAAGGCATCAATCTAATCAAAGCAAGGCTTGGGTCAAAGAAGGTTCTAATT

GTTCTTGATGACGTGGACCACAAAAAACAATTAGAATCATTAATGCGAGAAAGAAGTTGG

TTTGGTTTGGGTAGTTTAATAATCATTACCACCCGAGACGAACGATTGCTATGTCGGCTT

GGAGAAAAAGAGAGATATGAGGCCAAACTATTAAATGACAATGAAGCTATGTTACTTTTT

TGTTGGCATGCTTTTGACCGTCATTTTCCACCAGAAGATTATGTTAATTTGGCACGAGAC

ATAATCAAATATTCAGGTAGGTTACCATTAGCTCTTGTGACATTGGGGTCACATTTACAT

GGAAGTTCTGTAGAAGAATGGGGCCATGAATTTGAAAAACTAAGAGCGATTCCTCATTGT

GATATCCAAAAGATTCTCAAGATAAGCTTTGATGGACTTGATGATGAAACACAGACTGTT

TTCCTCGATATTGCATGTGCCTTCCATGGGTTTGATGAGCATGAAGTTACTGAAATATTA

AATGCATGTGGCTTTCATGCTAAAATTGCAATTGCAACTTTAGTCCAAAAACACTTGCTC

CAAAAATCTTGGAATATTTTGGAGATGCATGATCTAGTGCGAGATATGGGAAGAGAAGTC

GTTCGCATGGAATCAGCTCGGGATCCTGGAAAACGGAGTAGATTGTTCATCCCGCAAGAA

GTCTGTGATGTTCTACAAGGAAATAAAGGTTCCAAAAAGGTAGAAGTACTGAAGGTAGAT

CGACGAGCATTTGAGGGACTGAACTTGAGCACCAAAGCATTTAAGAAAATGAAAAACCTT

AGGGTTCTTATAATGGATGAGTTACATATTAGTGGAGATTTTGAGCTGTTGTCCAAGGAG

CTCAGATGGTTGTCTTGGAAAAAATGTCCTTTAAAATGTATACCATCAAATTTTCCAGCT

GAGAATCTTGTAGTTCTAGATATGCGGGAGAGTGATATCCAAGAATTTCAATTGAATTTG

CAGTGTTGCAGAAGTTTGAAGAAGTTGGATCTCTCTTATTGCAAGCAACTCAGAAGCACT

CCAAACTTCACTGGTTCAATGAGTCTTGAGAATTTGTCCCTTGGTAGTTGCTCAAGTCTG

GCAGAGATACATCCATCAATAGGAAATTTGGACAGACTAATTAAACTAGATATGTCTAAT

TGCGGAAAAATTATGGATCTTCCAAGCAGCATATGCCAGCTAAAATCCCTTGAAGACTTG

GACATTGATGGCTGCTCATCTATAAAAGCACTGCCAGATAACCTTGGAGATTTGAAAAGT

CTAAGATCTCTTGATGCATATGATACGGGTATAAAACAAGTGCCTAGATCTGTTGAAATG

CTAAGAAATCTTGAAACTTTGAGAGTGGGAGGTCGAAAGCTAGAGGCCAAAAGGAGTATT

TCTGGAAGAGGAGTCCATCGGATACAATATTCCTTGTCAACTTTTGTATCCGATTTGAGC

CTTACATACTGTAATTTGTCCGAGGCTGATATTCCTAGGAATATTGGGAGCTTATCCTCC

TTAGAATATTTAGATTTGAGTGGCAACAGTTTCCATTGTCTACCCATTGATTTTTCTAAG

TTACGATTATTGGTGGAGTTGTGTTTGAATGACTGTGAGAATCTTCAAACACTCCTGTCA

GTATCAAATTTAGAGAATCTTGCAATTATTGAACTTGAGAATTGCCAAAAATTGGTCAAG

ATTACAGAGTTGGACAACCTCCCTTCTATATGGTCGATCAACATGATAAATTGTAGTTCT

CTGCAGAATCCATTCAATGAAGGCTTCTTTAGTGCACCTGCTCTATCATTTCTATCTAGA

AAAGATCCTGATTTGCGTGATTTAGAAGTTTATCTCCAATGCAATGAGATTCCAGAATGG

TGCAGGAATCAAGTAACAGCTTCATCTATGCGTTTGACTATGCCGATACATAATAATAAG

GAGTATAACTTCTTAGGAATGGTTCTCTGGTTTGTTTTCTGCTTTTTCGATGAAGCCCCT

TTTCCAAGCTTCTCAATTAGTATTGCCCATAAAAAGACTTTAATTGAGCCGTTGAATATA

CCTGATGAACACAGAGAACTGACATTTGTGTGTTACATATCTTACTTAGATGAACCTTTT

GATGGCCAGATAATCAAAGGTGGGAAAAGGATAAAAGTGTGGTCTGACGACTTTACAGTA

AAGAAGATAGGGATCCATCTGTTATATTTAGACCAACATGGTAATGTTATATCTTTACCG

GGAGACGTGGATCATTCTTATACTAGGGCGAAAGATGTCAGGAATTGGTGGAAATAG

>NTBX_mRNA_32478_cds NTBX_mRNA_32478 gene_18953|id=AT1G27170.1

ATGGAGGAAATTCAGACCACAACTTCACTCCCATCGCTAAGGCTGAATTACGACGTGTTC

TTGAGTTTTAGAGGCGAAGATACTCGCGAAAACATCACTAAAAACTTATACGATGCCTTA

TACTCAAAAGGCGTCCGAGTGTTTCGAGACACAAACGGGTTAACTCAGGGCGACGAGATC

GCACCAGGTCTTATGGACGCAATCAACGATTCAGCTGCAGCTATTGCTATTATTTCACCC

AATTATGCTTCGTCGAGATGGTGTCTAGAGGAATTAGCAACGATTTGTGAGTTGGGTAAA

CTCGTTCTGCCCGTGTTCTACCGGGTTGACCCGTCGGATGTTCGAAGGCAGAGAGGACCG

TTTCTACATGATTTTGAGAGTTTGGAAGGAAGATTTGGAGTGGAAAAGGTGGTGAGATGG

AGAAATGCTATGGAAAGAGTTGGGGGAATCTCCGGCTGGGTTTATTATAATAGTGAAGAG

TCACAGTTGATACAGACTTTGGTGAAAAGAGTTTTACAAGAATTGAGCAATTCCCCAATA

TTTGTAGCTCCATTTGTTGTTGGAATTGACTACCGTCTGGAAGAACTCATAAGACAGTTA

GATGTGAAGCGCAGTGGTGTCAAGATCATTGGGTTGCATGGAATAGGAGGAGTTGGTAAA

ACAACTCTTTCTAAGGCTCTTTATAATAAACTTGCTTCTCATTTTACACACAGGGCTTTT

ATCTTGAATGTTAAGGAAATAGCTGCTCAACAAGGCATTGTGTCCGTTCAGAAGAAAATA

ATACAAGGTCTTTTCCCGAGCAAGGTCTTCTCCTTCTCCCCTGGTAATGCACATGAAAGA

AGAGTAAAATTCGGACGATTTCTTCAAGAAAAGCGTGTCCTGCTCGTCTTAGATGATGTA

GATTATGTAAATGATGATGTAAGCATATTGAAGGCACTAATTGGAGGGAAAAACTGGTTC

TTTGAAGGGAGCAGGGTTGTTATTAGTACTAGAAACAGAGGAATTTTGCTAGAAGACATC

GTTAACGAGACATTTGAGGTGAGAGAATTGGGTGGTCCTGACTCACTAAAACTATTCAGT

TACCATGCATTTAGAAGACAGGAGCCATTTCCAGCTTTTGTGAATATGTCCAAGCAAATT

GTCTCAATCACTGGAGGGCTACCCTTGGCTCTTGAAGTTTTTGGTTCTTTCTTGTTTGAT

AAAAGAAGCGAGGAGGAATGGCTAGATGCTCTAGAAAAGCTAAAACAAATTCGCTCTCCA

CATCTTCAGGAAATCTTGAAAATAAGTTATGATGGTCTTGATGATGAAGAGAAGTGTATA

TTCCTGGATGTTGCATGTTTATTTCTTGATCAATTAGAAAAGAAAGCTGAAGATGTAATT

GATGTGATGAAAGGATGTGGTTTTAGAGCCAGCATTGCATTTGACACTTTAACTGCTAGA

TCATTGATTAAGGTAATTGATGGTGGGGATTTGTGGATGCATGACCAGATAAGAGATATG

GGAAGACAAATTGTTATACAACAAGGCATTTCAGATCCCGGAAAGCGCAGCAGACTTTGG

GATGTTGCTGATGTTTTGAGTGTGTTACAAGGAAGGAAGCAACAGGGGACACAGAACATC

CAAGGGATCATCCTGGATCAGTATCAGAAGCCATCATCAAAGATTAAAAGCACGAAAGCA

ATTACTAGAGAGCATTTTCAACAAGTTCCCACTTTTACTTCTGCATTAGCTTACATTAAA

GAGTTGTGCAAAGAACAATTTCAAAATGATGCAAAAGAAACCAATGATTTGGTATTGAAC

ACTGAAGCATTTGATCCAATAGTTAATCTGCGGCTACTCCAATTCGATAATGTGAAACTA

GAGGGAAATTTGGGGAAGTTACCTTCTTCACTAAAATGGCTCCAATGGAAAAGGTGCACA

CTTTCAAGCTTTTATTCTGATTATTATCCAAGTGAACTTACCATGCTTGATCTCTCAGAG

AGCCAAATAGAGAAGTTTGGAAGCCGGGAATGGACTTGGACTCGCAAAAAGGTGGAAAAC

AAGTTGATAGTTATGAATCTCTCTGGTTGTCATAAAATAACAGCTATTCCTGATTTATCC

ACGCATAAAGCATTGGAAAAGTTGATAGCTGAACGTTGCAGTGCATTGCAAAGGATTCAC

AGAACAATTGGGAATCTGAAAACTTTACGTCATTTAAATTTAAGAGATTGCCGCAACCTT

GTTGAATTTCCAGGTGAAGTCTCCGGGCTGAAAAATCTTCAAAAGCTGATACTCTCGGGC

TGCTCGAGATTGAAACAGTTACCTGAAGATATAGGCAAGATGAAGTCCTTACAAGAACTT

CTATTAGATGGGACTGCTATAGAGAAGTTGCCTGAAAGTATATTTCGCTTAACAAAACTT

GAGAAGTTAAGCTTAAGCCAGTGCCACTCACTGAAACAACTTTCCCGGTTCATAGGAAAG

CTAAGTTCTTTGAAGGAACTCTCTCTTAATGGTTCTGCTTTGGAAGAAATACCTGATTCT

ATTGAACATTTGCAGAACCTTCATACATTAAACTTAATTAGGTGTGAGTCACTTGCTGCT

ATTCCCAATTCTTTTGGCAACCTCAAATCTTTAGCAAATCTCTGGCTTTATGGCAGTGCA

ATAAAAATGATGCCAGAATCTATTGGTTCTCTGTATTATCTTAGGTCCTTATCGCTCGGA

AACAGTCAGCATTTAAATGCATTGCCTGTTTCAATTAAAGGATTGTCTTCTTTGGTTGAG

CTTCAAATAGACAAGGTTCCAATTATTAGTCTTCCAGATCATGTTTTTGGTGGACTTAAA

TCACTGAAGAATCTTGAGATAAGGAACTGTGAGCGCCTTGGCTCGCTTCCCCACTCCATT

GGAGAATTGTTAGCTCTTAGAACAATGACTCTTACCAGAAATGATGCTATTACGGAGCTG

CCAGAATCAGTTGGGAATTTGCAGAATCTTGTCATATTGAGATTGACCAGATGTAAGCGA

CTTTGCAAATTGCCAGCTTCAATTGGGGAACTAAAGAACTTAGTACACCTGCTAATGGAG

GAGACTTCAGTAACAAAATTACCTGAAACATTTGGGATGCTATCGAGCTTAATAATTCTG

AAGATGGGAAAGAAGCCTTTCTGCCAGGTATCACAAAGTACTGAAAACACAGAAGCAGCT

ACCTACACAGAAAGGGAAACATCACCTGTTGTGCTTCCTTCATCTTTCTCAGAGCTATCC

ATGTTAGAAGAACTTGATGCCCGCGCGTGGGGAATAGTTGGGAAAATACCGGATGATTTT

GAGAAACTATCATCTTTGGAGATCATCAATCTTGGTTTCAATGATTTTTCCTATCTCCCG

TCTAGTCTGAAAGGACTACTTTTCTTGAAAGAGCTCCTTGTTCCCCACTGCAAACAGTTG

AAAGCTATTCCTCCTCTTCCCTCAAGTTTGCTCAAGATAAATGCTGCAAACTGTGGAGCA

CTCGAGAGCATACACGATATCTCAAAATTAGAGTTCTTGCACGAGCTAAACCTTGCAAAT

TGCATGAGTTTGGTAGATATCCAAGGTATCGAATGCTTGAAATCCTTAAGAATGCTACAT

ATGGCTGGATGCAATGTCTCCTGTGCCTTTATGGTTAGAAGCAAACTTGATAAGGTACTG

TTTTCTCCTATTTAA

>NTK326_mRNA_42452_cds NTK326_mRNA_42452 gene_24553|id=AT5G17680.1

ATGGCATCTTCTTCTTCTGCTAGATGGAGCTATGATGTTTTCCTAAGTTTTAGAGGTGAA

GATACTCGGAAAACATTTACAAGTCACTTATACGAAGTCTTGAATGATAGGGGAATAAAA

ACCTTTCAAGATGATAAAAGGCTAGAGTACGGTGCGACCATCTCAGAAGAACTCTGTAAA

GCTATAGAAGAGTCTCAATTTTCCATCGTCATTTTCTCAAAGAATTATACAACATCGAGG

TGGTGTATGAATGAACTAGTGAAGATCATGGAATGCAAGACTCAATTTGGACAAATTGTT

ATACCGATATTCTATGATGTGGATCCATCACATGTTCGGAACCAAAAGGAGAGCTTTGCA

AAAGCCTTTGAAGAACATGTAACAAAGTATAAGGATGATGTTGAGGGAATACAAAGATGG

AGGATTGCTTTAACTGCAGCGGCCAATCTCAAAGGCTCATGTGATAATCGTGACAAGACT

GATGCAGAATGTATTCGGCATATTGTTGGCCAAATCTCATCCAAATTATGCAAGATTTCT

TTATCTTATTTGCAAAACATTGTTGGAATAGATACTCATTTAGAGAAAATAGAATCCTTA

CTAGAGATAGGAATCAATGATGTTCGGATTATGGGGATGTGGGGAATGGGGGGAGTCGGT

AAAACGACAATAGCAAGAGCTATGTTTGATACTCTTTTAGGAAGAAGGGATAGTTCCTAT

CAATTTGATGGTGCTTGTTTCCTTAAAGATATTAAAGAAAACAAACATAGAATGCATTCT

CTGCAAAATATCCTTCTCTCTAATCTTTTAAGGGAAAAAGCTAATTACAAAAATGAGGAG

GACGGAAAGCACCAAATGGCTAGTAGACTGCGTTCTAAGAAGGTCCTAATTGTGCTTGAT

GACATAGATGATAAAGATCATTATTTGGAGTATTTAGCAGGTGATCTTGATTGGTTTGGT

AATGGCAGTAGAATTATTGTAACAACTAGAGACAAGCATTTGATAGGGAAGAATGATGTA

ATATATGAAGTGACTGCACTACCTGATCATGAATCCATTCAATTGTTCTATCAGCATGCT

TTCAAAAAAGAAGATCCAGATGAGTGTTTTAAGGAGCTCTCATTGGAGGTAGTAAATTAT

ACTAAAGGCCTTCCTTTAGCCCTCGGAGTGTTAGGTTCTTCCTTATATAATAGGGATATA

ACTGTGTGGAAAAGTGCTATAGAGCAAATGAAAAATAATCCTAATTCAAAAATTGTTGAA

AAGCTCAAAATCAGTTATGATGGATTAGAGTCCACGCAACAAGAGATTTTTCTGGATATA

GCATGCTTCTTCCGAGGGAAAAAAAAAGATGATATCATGCAAGTTCTCAAGAGTTGTCAT

TTTGGAGCCGAATATGGATTGGATGTCCTAATCGAAAAATCTCTTGTGTTCATCACAGAA

GACGGTGAAATTGAAATGCATGATTTAATACAAGAAATGGGTAGATATATAGTGAACTTG

CAAAAGGATCTGGGAAAATGCAGCAGACTATGGCTCGCCAAGGATTTTGAAGAAGTGATG

ATCAACAATACGGGGACCATGGCAATGGAAGCAATATTTCTTCCTTATTTCGATTTTGAT

ACATTACGCTTTAGCAAAAAGGCCATGGAAAATATGAAAAGGCTTAGGATATTTAACATA

GGGAGGTCGTTGGCCCATGATGGTTTCATTGAGTATTTGTCCAATAGCTTGCGTTGGTTT

GTGTGGTATTACTATCCTTTTGAGTCATTGCCATCTACATTTGAACCCAAAATGCTTGTT

CATCTTCAACTCGTATGCAGTTCGCTACATCATTTATGGACGGAAACAAAGCATTTGCTG

TCTCTACGGAGGATAAATCTCAGCTCCTCTAAAAGCCTGATGCGAACACCGGATTTCACG

GGGATACCAAATTTGGAGTATTTGAATCTGCATGGATGTACTAGTCTTGAAGAGGTTCAC

CATTCTCTGGGGCGTTGTAGAAAACTCATTCAGTTAGATTTGTATTATTGTAAAAGCCTT

AAGAGGTTTCCATGTGTTAACGTGGAATCTCTTGAATATCTGTGTTTAGAAAATTGCTCA

AGTTTAGAGAAATTTCCAGAAAGTCACGGGAGAATGAAGCCGGAGATACAGATTGACATG

GAAGGCTGTAGGATAAGCGAACTACCATCATCTATTACTCAGTACCAAACTCATATTACC

AAGCTAAATTTGAGCGGTATGGAAAAACTTGTAGCTCTTCCAAGCAGCATCTCTAGGTTG

AAAAGTTTGGTTAGTCTGAGTGTGTCGGATTGCTCAAAACTGGAAAGCTTGCCAGAAGAG

ATAGGGGATTTGGACAACTTGGAGGAGCTTGATGCCAGAGATACTCTAATTTCACGACCT

CCGTCTACCATCGTACGCTTGAACAAACTTAAAATCTTGGATTTTAGAAAGTTAAACTCC

ATCTCTGAGCACGCCATCTCTGAGCCCTCCATCATGCTCAGAAATGGAGTGCACTTTGAG

TTCCCTCCGGTGGCTGAAGGATTACGCTCATTGGAAATTCTGGATCTCGGGTACTGCAAT

CTAATAGATGGAGGACTTCCGGAAGATATTGGATCCTTATCCTCTTTGAAAGAATTGAAT

CTCCATGGAAATAATTTTGAGCATTTGCCTCGAAGCATAGCCCAACTTGGTGCTCTTCGA

TCCTTAGACTTATCATATTGCCAGAGGCTTACACAGCTGCCAGAACTTCCACCAGACTTA

GATACAATATATGTAGATTGGAGCAATGATTTCATCTGTAATTTGTTGTTTCAGAATATC

TCGTCATTGCAGCATGACATCTCTGCTTCAGATTCCTTGTCACTAAGAGTATTTACCAGT

ATGCATTATATCTGTGTGCTTATTGAGGTGAAGATCCCAAGTTGGTTCCACCATCAGGGA

ACGGATAGTAGTGTATCAGTCAATTTGCCTGAAAATTGGTATATACCTCATAAATTCTTG

GGATTTGCTCTATGTTACTCTTGCAGATTAGTTGACGCCACAGCTCAATTGATTCTATGT

GATGACGGGATGTCGTGGAAGACCCGTAAACTTGCCTTATCCAACCATTCAGAATTAGGT

TGTTTCTGTGGAGACTATGATATACATTTATTCTTTGTACCTTTTGCTGGCTTATGGGAT

ACATCTAAGGCAAATGGAAAAACACCAAATGACTATGGGATTATTAGGCTATCTTTTTCT

GGAAAAGTGAAGAAGTATGGACTTCGTTTGTTGTATAAAGAAGAACCTGAGGTTGAGGCC

TTGTTACAAATGAGGGAAAATAACAATGAACCAACAGAACATTCCATTGGGATAAGGAGG

AGCAGATCTGACAATAGTGAACACCATGACTCCGTGACCGATGGAGCCAGTTGCTGTCGC

ATACTGTAA

>NTBX_mRNA_21610_cds NTBX_mRNA_21610 gene_12528|id=AT4G12010.1

ATGGATTCCCAAAAGGAATTGAACAAAAAATGGGAATATGATGCCTTCTTGAGCTTTAGA

GGTGAAGATACCCGCAACAACTTTGTGGCCCATCTCCACAAACGTTTGGAAGAGAGAGGA

GTCAACGTATTCAAAGACGATGAGAAACTTGAAAGAGGAAGACCCATTTCAGCTGAACTA

TTGAAAGCCATTGAAGAATCAAGAGTTGCCATAATCATATTCTCCAAAAATTATGCTTCA

TCGGTATGGTGTTTGGAGGAACTCACCAAACTTATGGAGTGTGTTGAAAAGAAGGGACAG

GAAGCTATCCCTATATTCTACAATGTTGACCCATCAGATGTACGCATGCAAAGATCCAAA

AGTAGTTTCGCAAAAGCGATGGCCAAACACAAGGCTAATTTCGAGGGTAATGATTTGGAG

AAGGTGCAGAGGTGGACTGACGCTCTCCAAAAAGCAGCCAATATAGCAGGATGGGATCTT

GGTAAATGTGCTAACGGGAATGAGCCAGAATGCATTGATCGGATTGTACACGAGAAATTT

CAAAATGTGCACCACACAGTTTCAGCAACTGAGAAGTATTTAGTGGGAGTCGAATCTCGT

ACTGGTGGAGTGGAATCAATGTTGAAAGTTGGATCAGGAGGTGTTTATTTTGTGGGAATA

TGGGGAATGGGTGGCGTGGGGAAGACAACGGTCGCAAGAAAAATCTTTGACAATATTTCT

AATCAGTTTCAAGGGTCTTGTTTTCTTGCAAATGTTAGAGAAGAATCAAAGAAGCATGGG

ATAAAACACTTGCAGAAGACACTTCTTTCAAGAATCTTAAATGAAAAATCTTTGAAGGTA

GCAAGTTTTTATGAAGGAGCTGACATGTTAAAAGGAAAGTTTTGTCTCAGGAAGGTTTTA

ATTGTTTTTGATGATGTGGATGACAACCACCAGTTGGAGTATTTAGTTGGAAAGCATGAT

TGGTTTGGTGATGGCAGTAGAATTGTTACAACAACCCGAAATGCAGATTTACTTCGTTGC

CACGACGAGTTATATTCTGTCCCTGAATTGGCGAAATGCGAGGCTCTTGAACTTTTTAGT

TGGCATGCCTTTCAGAAGAGGACTCCAGATAAAGAGTTTTTAAAACTCTCTAAGTCTGTA

GTAGATTATGCTAAAGGTTTACCCTTAGCTCTTACGGTATTGGGTTCTTTTCTCTACAAA

CGAGGCATAACTGAGTGGGGAAGCGCATTGGATAGACTCAAAGATACTGGATATGAAGAA

ATTGTTAAGCAGCTCAGCTTAAGTGTAGATGGATTGTCCTATGAAGACAAGAATATATTT

CTTGATATTGCATGCTTCTTTAGAGGAAAAAGGAGAGATTTTGTGATAACAATACTAAAT

AGTTTTGGCTTCAAATCAGAGATTGGAATAGATGTCCTTACAAAGAAATCACTTCTTTAT

ATTTCAGAAGGAATGGTTGAGATGCATGATTTGATTGAACAAATGGGTCAGCAATTGGCA

CGTGATGTTGATCAGGACAAACCGTGGAACCATAGTAGAATATGGCATGAAAACGATATA

GAAACTGTTTTTTCTGCAAATCGGTGGACAGAGTCAGTAAAAGGCATAATGGTACCAATT

GGCTCAGACCGACATATATGCAAGTGGAGCAAAGCTTTCAGAAATATGCCTTGTCTTAGG

TTACTCATGGTCAAAGGGGAGGAGGTCCGACATTATGAACAAGTTTCTGACACTATTGAA

TATCTCCCCAGTAGCTTGAAATGGCTTGATTGGTCTTACTACAGTTTTGAATCTTTACCA

ACAAATTTTCAACCAAGAAACCTGGTTGGGCTCAACATGACTTTTAGTTCTCTTGTTGAA

ATTTGCAAGGAACCAAAGGCATTTGTCAAGTTGACGATTCTCAATTTAAGTTTTTCAGAG

AATTTACTCCGAACACCCAATTTTTCTGAGATTCCAAACCTGCAGAGGATAATACTGAAA

AGTTGTTTAAGCTTGAAAGATGTTCATCCATCCATTGGCAATCTCAAAAAGCTTGTTTCT

CTGAACATGAAGAACTGTAAAAATCTCAAGTTTTTACCAAGCAGTATTCAAATGGAATCT

CTTGAATGCCTCAACCTCTCTGGTTGTGAGAAATTAGATACTCTTCCAGAAATTCGGGGG

AATATGGAATTGCTATCAGAGCTTCTTTTGGGACGTACTGCAATTCGGGAACTACCCTCA

TCAATAGGACGGCTCTTTGGTATTAGTTTGCTTGATTTGCATTCATGTGAAAATCTTGTA

AGACTCCCAGCCAGTGTTAGCGAGATGAGAAAACTGAAAGTTTTAATTCTTAAAGGCTGC

TTAAAACTGGCAACTTTTCCAGAAAGCCTGGGTGATCTAGAAGAATTGGAGGAGCTCTAT

GCTGGAAACACTGCCGTTTGGCGACTACCAGATTCTATGGAAAACTTAAGCAAACTTAAA

ATCCTATCATTAAAAGGTAGACGAGAGATGAAGTGTCAATCTGCTACAGGTTTGATATTC

CCTTGTGCATTTCATGGTTTGAGGGCATTGAAAAGTTTGGATCTCAGTGGATGCAATTTA

TCTGGTGACGAGATTAATGGTCTTACGTGCTTGACTTCTCTATTGGAACTAAACCTTAGC

AGAAACAAGTTTATTTCTCTACCTGATGGCATCAGTCAACTTTATCAACTTCGATATCTC

AACATAACACACTGTCATGAACTTAAGAAACTTCCCAAACTTCCTCCAAGTATAAAGGAA

TTGTATGCAGAAGATTTTCTGGCCAAACAAATTATTCTAGCTATGTCGGTATACCGAGGG

TTGTGTTTGGCCTCATTCACCAACTATAGTTTTGATCAACAACCATACACAGAGAAGAGC

GATGATAACTCAGTGTTGGATGAGATTCTGAGCTTGTTTCTTTCAGACAGCATGGATGAT

AAGATGACTTATTGTATTATCTTTCCTGAACGTGCTATCCCCACATGGTTTAAACATCAG

AGTACTGAGGAAAAGATCTTGCTTAAACTGCCTGAGGATTGGTATGATGATAGATTTGAG

GGCTTTGCTATATGCTGTGTCACTTGCATGGGGGCAGGTGTCCACGATCCTGATTCAGGG

CTATCAGGGAAGTACGACTATACTTTCATCAAAGCCAAATTGATATGCAATAATCATATG

GAAGAGCTTGAAGTGCTGGAGAAAGAGTGTAAAGTCAGTACAACGTCTAGAACTTATAGC

TGGTGTGTTTGCTTTGCCTACATACCATTGTATTCTTTGCTGCAGACTTCTGGCACACAA

GTTCTAAACTTTAACCAGTATGGCCTATTCGAGGCATCTATCCAAAGGCACATTACGAGA

CAATGGGGAGTTCATTTGATTTACAAGTCTGAAAGACAATTTTTCGAGAGCAGAACTGAG

AAAGGGTTGCTTGCCCAGAAGTTGAGTCTAGTCAAGCCAATTCGAGTCAAAACGAGAATT

GTTCGATTAGAATTACCTAAATAG

>NTK326_mRNA_30477_cds NTK326_mRNA_30477 gene_17584|id=AT5G36930.1

ATGGCCACTGAACTGAAGTCTCAAGTGTTCTTGAGTTTCAAAGCGAAAGACACCGGCAAT

AATTTTGCAGATCATCTCTATGAAGCTCTGGTGGGAGCAGGTTTTGTAACATTAAGAAGC

TGTGGTGATGAAAATGAGGGAGGTGAAGATATCAAGTTAAATTTGCAAAAGGATATTAAA

GAGTCAGGGGTTTCAGTTATTGTCTTCTCAAATGATTATGTGTGTTCAAGTTGGTGTCTT

GATGAGTTGGTAATGATCTTGGATTGTAAAAGGATAGCAAGACGTGCAGTTCTGCCCATA

TTTTACCACGTGGATCCTTCTGATGTTAGGAAACAGATGGGGAAAATTGGAGAAGCATTT

GATAGGCATGAAAATCTGGGAGGGAATCAAAGTGGAAATGAGAGGGTCAGAAAATGGAGG

GAAGCACTCAAAGAAGTTGCAGACTTGGGTGGAATGGTCTTACAAAACCAAGCTGATGGA

CACGAGTCCAAATTCATCCAGAAGATTCTTAAAGTGGTTGAGAATAAACTGAGCAGGCCA

GTCCTGTATATTTGCCCTCATCTGATTGGAATAGAACGGCGTGTTGAAAAGATTAACACA

TGGCTAGAGGATGGATCTACTGAGGTTGACACTCTTGTTATTTGTGGTATCGGTGGAATA

GGCAAGACAACAATGGCAAAGTATGTGTATAATTTAAACTTCAGTAAGTTTGATGGTAGC

AGCTTTTTGTCCAACATTAGAGAAAATTCAACACACCGTAAAGGTTTAGTTACTCTTCAA

AGGCAATTTCTTTCTGATATCTGCAAAAGAAAGAAGAAAGCTATGTTTTCTGTGGATGAG

GGAATGACTGAGATGAGAGAGGCTGTCCGGTGTAAAAGAATCCTTCTTGTTCTTGATGAT

GTAGATAACCGTGATCAATTAGATGTTCTACTGGGAATGAAGGACTGGTTCTACCCTGGA

AGTAAAGTCATTGTGACAACTAGGAACAAGAGATTGCTTAGGCCTTTTGATGTGCATAAG

ATTTATGAGTTTGAAGCTTTGAACAGAGATGAATCGGTTGAGCTCTTAAGTTGGCATGCA

TTTGGTCAAGATTGTCCTATTAAAGGTTTTGAAATGTGTTCAGAACAAGTAGCAATCCAT

TGTGGAGGACTTCCATTAGCACTTGAAGTTCTTGGTGCTACTTTGGCAGGAAGAAACATA

GACATTTGGAAAAGTACAATACAGAAATTGGAAACAATTCCGAATCATCAAATTCTCAGG

AAATTAACAATAAGTTACGAATCTCTTGAGGATGATCATGATAAGAATTTATTTCTCCAC

CTAGCTTGCTTTTTCATTGGGAAGGACAGAGATCTAGCAGTAACTATTCTCAATAGGTGC

AACTTTTACACTGTAATTGGAATTGAGAATCTCATTGACAGAAATTTTATAAAAGTTGGT

AAGTCTAACATGTTGATTATGCATCAAATGATTCGAGATATGGGAAGAGACATTGTTCGC

CAAGAATCACCAGTGGATCCTGGGAAACGCTCTAGACTATGGCGTTCAAAGGATTCCTTT

AATGTGTTAATCCAGAACCGTGCCACTCAAACAATTCAAGGCATTATCCTTGACATGGAT

ATGCTCAAGGAAAGTGACATAGTTAGCTCAAGCTTTTCCGCCAATGATTTCAGGAAACAC

AAAACAAAAAACTTTCTCAACTATTCTAATCCTCAGAGAGTTCAATTCAAACAGAAAAGG

TTTGGTTTTTTTCCATGGCATTTGTCAGACACCAAAGAAGCCACAAATGAGCTGGTTCTA

GAAACTGATGTATTTGCAAATATGCAAAAGTTAAGACTGCTTCAATTCGATCACGTTGAG

CTTCAAGGATCTTTTGATGTTTTTCCTAAGAGATTAAGATGGTTGCGCTGGTCTGAGCTG

CAACTTGAATGCATGCCAATTGATTTTCCTCTGGAGAGTCTTGTAGTGATTGAATTACAC

CGCAGCAGCTTGAGGAAGATTTGGCATGGAGTCAAGTTCCTTAAATATCTGAAGATTTTC

GATCTCAGCCATTCCCACGAGCTTCTAAGAACACCTGATTTTTCAGGACTCCCCAATCTT

GAAAAGTTGATCCTTCGATATTGTACAAGCTTGATTGAGCTTCATGACACCATCGGATGT

CTAGAATCACTTATTCTTTTGAATCTCAAAAATTGCAAAAATCTGCAGAAACTTCCAGAT

AGCATTTGCATGCTAAAATGTCTGGTGACACTAAATATCTCTGGTTGCTCGAATCTTGAG

TATGTGCCGATGAATCTAGATAAAATGGATTCTCTGAGAGAGCTTTATGCTGATGAAATT

GCAGTTCACCAAATGATTTCTACTCCAGAAGAGGTCCAACAGTGGTATGGATTTCTGTGG

TCCTGGATGCTGAAAGGGAAAATTTGTCCTAAAGTTTCACATATTAGTTTACCTAATTCC

TTGGTTACTCTGAGTCTTGCTAACTGTAATCTATCCGATGATACTTTTCCAGTTGCTTTC

AGTAGCCTCTCCTTATTGCAAAACTTAGATTTGAGCGAAAATCCAATTTGCAGTCTACCA

AAGGGCATAATTTATCTCACCGGACTTCAGAAGCTAGAAGTGGAAGGCTGTGAAAAGCTC

AGATCGCTCATAGGGCTTCCCAATGTAGAACATCTCAATGTTACTAATTGCTGGTTGTTA

GAGAAAATATCATATAAATCAAGATCATCTAGACTGAAGGATTTGCTTGTGTCAAATTGT

GCTAAATTAGTTGAAATAGATGGAAATTTCAAGTTAGAACCCTTAAGAAATACTGAGGCC

CTTTGCAAGTTGGGCTTATCGAACTTGGCTCCTATGGATAATGTCATGATCAACCTTACA

TCTAATATCCTGAGTTACTACCGAATACATGGTAAAGGATGGACTCCAACAAGGAAGACA

AAGAAAGTTGTTCTTCAGGTACTATACCAACCAGGTGTCTTTAGCACTTTTCTGCCAGGT

GAACATGTACCTTCTTGGTTCAGCTCAAAATATACAAAAGAATCACGTACATCTCTCAGA

GTGCCTACTTGTAATTCCAGAATTGAAGGCTTGAGTTTTTGCATTGTGTACAAGCGTTCT

GCATTTGGTCTAAGTGCTCACCGTCCCCCACGCTTGGCTCCGCCTTCAAGAATAGCTCCT

CTTGCTATGCGCAAAGCTCAAAGAGGACCCATTCGGTATCGGCCAGTGGAAAATAAACCA

TATGAATCAACTTTTGACTGCCCGTGCATTACAGTTAATAACTTAACTCGGAGTTTGAAA

TGGTCTTACCAGCCCTTGTTCTATGGAGTTCCGGAAGGGAAAGAAGGAATGATGTGGTTA

AGCCATTGGAAACTTGAGAATCAGTTGAGCAGTGATGATATTCTGGAGATCACAGTTACC

TCAGGAGATGGAATCACAACTGTAGAGTTTGGGCTCAAAATTATGCATGTTGAAGGGCCA

AAACTGCAAATAGTAGAACCAAGTTGTGAAGATGCAAGGGCTGAGAAAGACATTGTCAAT

CCATTTTGGGATGTTGTTTTGGAAGATGCTAGTTCAAAGAATACTTGTTCTATTCGGCTT

CCTCCTACTTATCGTCCCCTATGTGTTGCTCGTGAGCCATTTCTGGAGAAGGCGCTCAAA

AGAAATATGTCAGAATATAACTAA

>NTTN90_mRNA_46475_cds mRNA_46475 gene_26069|id=AT5G36930.1:evalue=3e-09:annot='Disease resistance protein (TIR-NBS-LRR class) family';id=Solyc09g092410.2.1:evalue=0.15:annot='Tir-nbs-lrr, resistance protein'

ATGGATAGTCAATTAGTAAGAGGAGAATCATGTACATCTTCTCACTTCTCTTATGAAGTA

TTCCTCAGTTTTAGAGGTGAAGACACCCGAAAAACATTCACTGACCATCTTTATTCCAAA

TTGCGTGATGTTGGAGTCAATACCTTCATTGATGAGGAATTGAGAAAGGGTGATGTGATT

TCAAGTAAACTAGAGAAAGCAATTGAAGAGTCAAGAATTTCCATTATTGTTTTCTCAAGA

AATTATGCTTCCTCTAGTTGGTGTCTAAATGAACTAGTTAAAATTCTTGAATGCAAAGAG

AAATTAAAGCAGATGGTTTTGCCTATTTTCTACGATGTTGATCCTTCTGAGGTACGAAAG

CTAACTGGGTTATTTGGTGAACCATTTGGAGCTCAAAGGGTGGAGAAATGGAGAGCTGCA

CTTACTGAAGCTGCAAATTTATCTGGATGGCATTTGCAAAATGTTGCTGACGGGCATGAA

TCAAAGTTTATTGAAAAAATTATACAGCAAGTTCTACAAGAGGTCAACCAGACACCTCTA

GATGTTGCTTGGCACCCAGTTGCAGTAGATTCTAGTGTCAAAGATATAGAGTTGTTATTG

CAAATTGAATGTGAAGATGAAGTTCGCATGATTGGTATTCACGGACTTGGTGGCATAGGG

AAAACAACTCTGGCAAAAGCTATGTACAATCGAATGTTTCGACTCTTCGATAGTGGTTGC

TTCCTTTCAGATGTTAGATCAGAAGATGAAGAATTTGGTCTTGTCAAGCTACAAGAGAAA

CTTCTTCAACAAGTTCTCAAAACCAAGGACATCAAAGTTGGGAGTGTTGCTCAAGGCGTT

AATCTAATCAAAGCAAGACTTGGGTCAAAGAAGGTTCTAATTGTTCTTGATGATGTGGAC

CATAAAAGACAGTTAGAAGCCTTAACAAGAGAAAGAAGTTGGTTTGGTTCGGGTAGTTTA

ATAATCATTACCACCCGGGACAAGCGATTGCTACGTCGGCTTGGAGAAAAGGAGAGATAT

GAGGCCAAACTATTAACTGACAATGAAGCTATGTCACTTTTTTGTTGGCATGCTTTTGAT

AATCATTTTCCACCAGAAGATTATGTTAAATTGGTACACGGCATAATCGAATATTCAGGT

AGGCTACCATTAGCTCTTGTGACATTGGGGTCACATTTACAAGGAAGTTTGGCAGAAGAA

TGGGGATATGAATTTGAAAAACTAAGAGCAATTCCTCATAGTGATATCCAAAAGATTCTC

AAGATAAGCTTTGATGGACTTGATGATGAAGCTCAATCTGTTTTTCTCGATATTGCTTGC

ACCTTCCATGGGTTTGATGAGCATCAAGTTAATGAGATATTAAATGCGTGTGGCTTTCAT

ACTAGAAGTGCAATTGCAACTTTAGTCCAAAAACACTTGCTCCGAAGATCTTGGAATCAT

TTGTTGGTGATGCATGATCTAGTGCGAGATATGGGAAGAGAAATCGTTCGCATGGAATCA

CCTCGAGACCCTGGAAAACGGAGTAGATTGTTCATCCCTCAAGAAGTTTGTGATGTTCTA

CAAGGAAATAAAGGTTCCGAAAATGTAGAAGTACTGAAGGTAGATCGAGAGACGTTAAAG

GGAGTGCACTTGAGCACCAAAGCATTTGAGCAAATGAAGAACCTTAGGGTGCTTATAACC

AATGAGTTACATATTAGTGGAGATTTTGGGATGTTGTCCAAGAAGCTCAAATGGTTGTCT

TGGCGAAAATGTCCTTTAAAATGTATACCATCAAATTTTCCAGCTGAGAATCTTGTAGTT

CTAGATATGCGGAAGAGTGATATCCAAGAATTTCAATTGAATTTACAGTGTTGTAGAAGT

TTAAAGAAGCTGAATCTCTCTTATTGCGAGCAACTAAGAATGACTCCAAACTTCAACGAT

TCACGAAGTCTTGAGACTTTGCTGCTTGGTGGTTGCTCAAGTCTGACAGAGATCCATCCA

TCAATAGGAAATTTGGACAGACTAATTAAACTAGATATGTCTCGTTGCGAAAAACTTAGG

GATCTTCCAAGCAGCGTATGCCAGCTAAAATCCCTTGAAGAATTGTTCATTAATGACTGC

TCATCAATAAAAATACTGCCAGATAACCTTGGAGATGTGAAAAGTCTAAGATCTCTTAAA

GCATATGGTACGGGTATAAAACAATTGCCTAGATCCGTTGAAATGCTAAGAAATCTTGAA

ACTTTGAGCGCGGGAGGTCAAGAGTTAGAGGCTAAAAGGAATATTCCTGGAAGAGGTGTC

CATCGGATACAATATTCCTTGCCAACTTTTGTAACCGAATTGAGCCTTATATACTGGAAT

TTGTCCTATGCTGATATTCCTAGGGATATTGGGAGCTTATCCTCCTTAAAGTTTTTAGAT

TTGAGTGGCAACAGTTTCCATTCTCTACCCTTCGATTTTTCTAAGTTACGAGTATTGAAG

AAGTTGTATTTGAATGACTGTGAGAATCTTCACACACTCCCGTCAGTATCAAATTTAGAG

AATCTTGGACATCTTGAACTTCAAAATTGCAAAAAATTGGTCAAGATTACACAGTTGGAC

AACCTCCCTTCTATACGGTTGATTAACACGAGTAATTGTAGTTCTCTGCAGAATCCATTC

AATGAAGGCTTCGCTGCTCTATTAATTCCATTTAGAGGACATATGGGTTATTTAGAAACT

TATCTGGAATGCAATGAGTTTCCAGAATGGTGCAGCAATCAAGTAACAGCTTCATCTATC

TGTTTCACTATGCCGACACATAATAATGAGTACAACTTCTTAGGAATGGTTCTCTGGTTT

GTTGTCGACGCTTTGGATGTAGCCCTTAGCATTGCCCATAAAGTGCCTTCAGGTATTCCG

TGGAGTCATATTGGAATACTTAATGGACACAGAGAACTGACATGTGTATATTACATATCT

ATCTTAAATGAACTTTTCTATGGCCGGATGATTAAAGGCAGGGAAAGGATAGACGTGTGG

TCTGAAGACATTACCCTAAAGAAGATAGGGATAGATCTGTTATATTTAGACCAAAATGGT

AAAGTTATATCTTTGCCGGGAGACGTGGATCATTCTTATTCTAGGGCGATAGATGTCAGG

AATTGGCAGGTGGAGATTTTTGATGGCCAGACGATAGATATGAGTATTACATACTTTAAT

TTGTCCGAGGATGATATTCCTAAGAATATTGGGAGCTTATCCTCCTTAGAATATTTAGAT

TTGAGTGGCAGCAGTTTCTATTGTCTACCCTTTGATATTTCTAAGTTACGATTGCTGAAG

GTGTTGTGTTTGAATGACTGTGAGAATCTTCAAACGCTCCCGTCAGTTTCAAATTTAGAG

TATCTTGAAAGAATTGGACTTTATAATTGCCAGAAATTGGTCAAGATTTCAGAGTTGGAC

AACCTCCCTTCTATATGGTCGATCAACATGATTAATTGTAGTTCTCTGCAGAATCCATTC

AATGAAGGCTTCTTTAGTGCACCTGCACTATATGCATCTAGAAAAGATCCTGAGTTGGAT

CCTTTAGAAATATTTCTGGAATGCAAGGAGATTCCAGAATGGTGCAGGAATCAAGTAACA

GCTTCATCTATGTGTTTGACTATGCCGACACATAATAATGATGAGTATAACTTCTTAGGA

ATGGTTCTCTGGTCTGTTTTCGACTCTTGGCATGAATCCTTTTTCTTGATTAGTATAGCA

GGTAGAAAGACTTTAATTTCTCCGCGGAGTATACCTGATGGAGGACATAGAGAACTGTCA

TGTGTATATTACATATCTTACTTAAATGAAGCTTTTGATGGCCAGATGATTAAAGGCGGG

GAAATGGTAGAAGTGTGGGCTCCAGATTTTACAGTAAAGAAGATAGGGATAGATCTGTTA

TATGTAGACCAAAATGGTAAAGTTATATCTTTACCGGGAGACATGGATCATTCTTATTCT

AGGGCGAAAGATGTCAGGATAGAGGTCAGGATCAGGAATTGGCGGGAAGAGTTGCTTAGT

TTGGCAGACACAATAAGAGAAATTTGA

>NTTN90_mRNA_69387_cds mRNA_69387 gene_39083|id=AT5G36930.1:evalue=1e-172:annot='Disease resistance protein (TIR-NBS-LRR class) family';id=Solyc09g092410.2.1:evalue=0.0:annot='Tir-nbs-lrr, resistance protein'

ATGGATCCTCTAATTATCCAAGAAGAATCATCTACATCTTCCCGCTTCACTTATGAAGTA

TTCCTGAGTTTTAGAGGTGAAGACACCCGAAAAACATTCACTGGCCATCTTTATTCCAAA

TTGCGTGATGTTGGAGTCAAAACCTTCATTGACGATGAGGAATTGAGAAAGGGTGACGTG

ATTTCAAGAGAATTAGAGAAAACAATTGAAGAGACAAGAATTTCCATTATTGTTTTCTCA

AGAAATTATGCTTCCTCTAGTTGGTGTCTCAATGAACTAGTTAAAATTCTTGAATGCAAA

GAGAAACTAAAGCAGATGGTTTTGCCTATTTTCTATGATGTTGATCCTTCTGAGGTGCGA

AGACAAACTGGGTTATTTGGGGAAGCTTTGGCAAAACATAAGGAACGATCATTTGGAGCT

CAAAGGGTGGAGAGATGGAGAGTTGCACTTACTGAAGCTGCAAATTTATCTGGATGGAAT

TTGCAAAATATGGTTGATGGGCATGAATCAAAGTTTATTGAAAAAATTATACAACAAGTC

CTACAAGAGGTCAACCAGACACCTCTAGAAGTTGCTTGGCACCCAGTTGGAGTAGATTCT

CGTGTTAAAGATATAGAGTTGTTATTGCAAAATGAATGTGAAGATGAAGTTCGCATGATT

GGTATTCACGGAGTTGGTGGCATAGGGAAAACAACTCTGGCAAAAGCTATCTACAATCAA

ATGTTTCGATTTTTTGACAGTTGTTGCTTCCTTTCAGATGTTAGATCAGAAGCCGAAGAA

TTTGGTCTTGTCAAGCTACAAGAGAAACTTCTTCAACAAGTTCTCAAAACTGAGGACATC

AAAGTTGGCAGTGTTTCACTAGGTATCAATTTAATCAAAGCAAGACTCGGGTCAAAGAAA

GTTCTAATTGTTCTTGATGATGTGAACCACAAAAAACAATTAGAATCCTTAACAAGAGAA

AGATGTTGGTTTGGTTCGGGTAGTTTAATAATCATTACCACTCGAGACGAGAGATTGCTA

TGCCGGCTTCAAGAAAAAGAGAGATATGTGGCAAAACTATTACATGACAATGAAGCTATG

TTACTTTTTTGTTGTCATGCTTTTGACGGTCATTTTCCACCACAAGATTATGTTAATTTG

GCACGAGACATAATCGAATATTCAGGTAGGTTACCATTAGCTCTTGTGACATTGGGGTCA

CATTTACATGGATGTTCTGTAGAAGAATGGAGATATGAATTAGAAAAACTAAGAGCAATT

CCTCATCACGATATCCAGCAGATTCTCAAGATAAGCTTTGATGGACTTGATGATGAAACA

CAAACCGTTTTCCTCGATATTGCATGCGCTTTCCATGGGGTTAGTGAGCGTGAAGTTATT

GAAATATTTAATGCATGTGGCTTTCATGCTAAAAGTGCAATTGCAAGTTTAGTTCAAAAA

CACTTGCTCAAAAAATCTTGGGATGGTTTGGTGATGCATGATCTAGTGCGAGATATGGGA

AGATATATTGTTTGCATGGAATCAGCTCGAGACCCTGGAAAACGGAGTAGATTGTTCATC

CCTCAAGAAGTCTGTGATGTTCTACAAGGAAATAACGGTTCTGAAAATGTAGAAGTACTG

AAAGTAGATCCAGAGACATTAAAGGGAGTGAACTTGAGCACCAAGGCTTTTGAGCAAATG

AAGAATCTTAGGGTGCTTATAATCAATGAGTTACATATTAGTGGAGATTTTGGGCTGTTG

TCCAAGAAGCTCAAATGGTTGTCTTGGCAAAATTGTCCTTTAAAATATATACCATCAAAT

TTTCCAGCTGAGAATCTTGTAGTTATAGATATGCGGAAGAGTGATATCCAAGAATTTCAA

TTGAATTTGCAGTGTTGTAGAAGTTTGAAGGAGTTGAATCTCTCTTATTGCAAGCAACTC

AGAAGCACTCCAAACTTCAATGGTTCAGGGAATCTTGAGACTTTGCATCTCTATGGTTGC

TCAAGTCTGAAGAAGATCCATCCATCAATAGGAAATTTGTCTAGACTAACTAAACTATAT

CTGCATGATTGCGAAAAACTTACGGATCTTCCAAGCAGCATATGCCAGCTAATATCCGTT

GATCACTTGGACATTAATTCCTGCTCATCAATAAAAACACTGCCAGATAACCTTGGAGCT

ATGAAAAGTCTAAGATTTCTTTATGCATCTTATACAAGTATAAAACAATTGCCTAGATCT

GTTGAAATGCTAAGGAATCTTGAAAGATTGGAAGTGGGAGGTCAAAAGTCAGAGGCCAAA

AGGAGTACTTTTAGAAGAGGAGTCCGTCAGATACAATATTCGTTGCCAACTTTTGTATCC

TATTTGAGCCTTACATACTGTAATTTGTCCGAGGATGATATTCCTAAGGATATTGGGAGC

TTATCCTCCTTAGAATATTTAGATTTGAGTGGTAACAGTTTCTATTGTCTACCCTTTGAT

TTTTCTAAATTACGATTGTTGAAGGTGTTGTCTTTGAATGACTGTGAGAATCTTCAAAAG

CTCCCGTCAGTATCAAATTTAGAGTATCTTGAAAGAATTGGACTTTATAATTGCCAAAAA

TTGGTTAAGATTACAGAGTTGGACAACCTCCCTTCTATATGGTTGATCAACATGATGAAA

TGTAGTTCTCTGCAGAATCCATTCAATGAAGGCTTCTTTAGTGCACCTGCTCTATCATTT

>NTK326_mRNA_16512_cds NTK326_mRNA_16512 gene_9366|id=AT5G17680.1

ATGATGCAGAAGAGCCCTTCTTCTTCCCCTGATCATACTTTTCGTTGGAGTTACGATGTT

TTCTTAAGTTTTAGAGGTGAAGATGTACGTAAAAGATTTGTCGACCATCTCTATGTTGCC

CTGCAGCAAAAGGGTATTCATACATTCAAAGATGATGAGAAACTAGAGAGAGGCAAGTCC

ATTTCACCCGATCTTATGAGAGCAATTGAAGAGTCGCGCATAGCTTTGATTATATTCTCC

AAAAACTATGCTAATTCAAGATGGTGCTTAGATGAATTAGTGAAGATCATGGAATGCAAG

AATTTGAAAGGACAAATTGTGTTTCCAGTGTTCTACGATGTTGATCCATCAACAGTGAAG

GAACAAAAATTCAGCTTTGGAGAAGCATTTCGTACTCATGAAGCCCATGGCTGTTTCAAG

GTGCAAAAATGGAGAGCAGCACTGGAGGAAGCAGCTAATTTATCTGGCTGCGATTTGCCA

AATACTGCCAATGCGCATGAAGCTAAAGTCATAAAGCAAATTGTGGAAGATATGATGGCT

AAATTAGGTGGTCAGAGGCATGCAATCAATGCTGAAAATCTTGTTGGAATGGAGTCGCAA

ATGCAGAAAGTGTATAAAATGCTTGGCATCGGGTCTGGTGGAGTTCACTTTGTTGGAATA

TTTGGAATGAGCGGAGTGGGAAAGACAACTTTAGCGAGAGTCATTTATGATAACATTTCG

AGTCAATTTGAGGGTGCTTGTTTTCTTCATGAGGTTAGAGACCGTTCAGAAAAACAAGGC

CTAGCGCGATTACAAGAGATACTTCTTTCCAAGATCCTTGTCATAAAAGACCTAAGGATC

AACAATTTATTTGAAGGACTTAATATGCAAAGACAGAGACTACGGTTCAAAAAGGTTCTT

CTTGTTCTTGATGATGTTGATCACATAGATCAGTTAGATGTTTTAGCTCAGAAGCGCGAA

TGGTTTGGTTCTGGAAGTAGAATCATCATAACAACTAAAGACAAACACTTGCTTGTTAAG

CATGATGTGGAAAAGATATACAAAATGAGAACATTAAGTGAAGATGAAAGTCTACAACTA

TTTAAACAGTATGCTTTCAAGAAGAACCATCCAACCAAGAAATTTGAGGATCTCTCAGCT

CAAGTGATAAAGTATAGTGCTGGACTCCCCTTGGCTCTGAAAGTCCTGGGCAGTTTCTTG

TATGGAAGAGATTTGGCTGAATGGAGAAGTGAAGTAGAAAGATTGAAACAAATCCCGGAA

GATGAAATTTTGAGGAAACTCGAACCAAGTTTCACTGGACTCAAAAGTATCGATCAAATG

ATATTCTTAGACATTGCGTGTTTCTTTACAGGGAAGAAGAAAGATTCAGTGACTAGAATA

CTTAAGAGTTTTAATTTTAGCCCTGTTATTGGCTTAAAAGTTCTCATGGAGAAATCTTTG

ATTACTATTTCAGAAGGTAGGATTTTAATGCACCAATTGATACAAGAAATGGGCTGGCAC

ATTGTTCGTCGAGAAGCTTTCGATTATCCCAGAAAATATAGTAGGTTATGGAAGTCTGAA

GATATTTCTCATGTACTTGCAAGAAATATGGGCACAGAAAAGATCGAAGGCATATCTTTG

AACTTGACTAAGACGCTCACAGATATTTCTCATGCACTTGAAAGAAATTTGGGCACAGAA

AAGATCAAAGGGATATCATTGAACTTGACTATCGTCAAAGAAGTGAATGTTAGTGCAACA

GCCTTTATGCAGATGACCAGACTGAGGTTTCTCAAAATCAAGAATGCATATGTTTCTCAG

GGTCCGGACATTCTTCCTAGTGAGTTGAGCTGGCTTTCTTGGCACGGATATCCTTCAAAA

AGTCTGCCAATTAGCTTTCAGGGAGAACGACTCGTTAGTTTGAAGTTGAAAAATAGTCGC

ATCATACAACTTTGGAAAGGCTCCAAGGTTCTAGGACAACTGAAGTACATCAACCTTAGC

CATTCACATAAGCTAATAAGGACTCCAGATTTTTCGGGTACCCCTAATCTTGAAAGGTTG

GTTCTTGAAGAGTGCACGAGTTTGGTAGAAATCAATTTTTCTGTTGGAGATCTCAAAAAG

CTAGTCTTACTCAAGTTGAAGAATTGCATCAATTTAAAGACCCTGCCAAAGAGTATTCAA

TTGGAAAATCTTGAGGTTCTTATTCTATCAGGCTGCTCAAAGCTAAAACTATTCCCAGAA

ATAGAAGATGGAATGAATCGTTTATCAGAACTATATTTGGAAGCGACTTCTTTGAGTGAA

CTACCCGCATCAGTTGAGAAACTATCAAGAGTTAAAGTGATAAATCTAAGCTCATGCAAG

CATCTTGAGAGTCTTCCAAATAGTATTGTTAGGTTGAAATGTCTTAAAGAACTTAATGTG

TCGAGGTGCTCAAAACTTAAAAGTTTACCAGATGACTTGGGTTCTTTAGTCGGATTGGAG

GGGCTCCATTGTGATGACACACCGATCCAAATGATACCATCCACCATTTCCCTTCTAAAG

AACCTTAAACACTTATCTCTCCGTCGATGTAATGCTTTGGGTTTGCAAGTAAGGAGTTCA

ATCTCAAGAGAATCTATGGGACTAGTTTTCTCTAATTTATCGGGTCTTTGTTCATTGACA

ATGCTGGATATAGGTGGCTGCAGCATTTCAGATGGAGGCATCCTATGTAATCTTGGGTTC

CTACCATCTTTGGCGGAATTGAATCTTGGTGGTAACACGTTTACCAATATCTCAGCTTCA

AGCATCAGTGGCCTCACTCGACTAAAGGTTCTTCAATTGGTTGGCTGTAGTAGGCTTGAG

CATTTCCCAGAACTTCCTCAAGCTATAGAAGAGGTGCATGCCGATGAATGTATATCTTTG

AAGAGTATCGATCAATTAGCAAAATATCCAACATTACGCCGACTTTCACTTAGCCAATGT

CATCAGCTTCATGATACTGACATGGTTGATGCATTATGGAGCAACATGCTCAAGGGACTA

TACGTGCTACGAAATGATCTCAGCATTTGCATCCCTGGATCGCAAATTCCTATGTGGTTT

ACATATAAGAACTTTGGGGAAAATGTTACACTGACTCTTGCCAATAATTGGTACACTGAT

AACCTCTGGGGTTTTGCTTTCTGTATTGTTTTTGAACGTATGGAATGGTGCGGTCTATAT

GATGGTTACCTACAACCATCACTTGGATTTCCAGTTAACCTTAAATTCAAAACATATGAT

GGTAAGGAAGGCGATATACGCAGCATTATTGGCATCAAAGGAGGTGATATGTCAATTCGG

AACTCAGAGCACACTCTCCTTTCTTATGTACCATCTCGTCGTTTTCTGCAACCTTACAAT

AACGAGGTTTACTGTCCCAACGACTGGATCGAAATTGTGGCATATTCGACTGTACAATTC

GACAGTAAAGCTTGGGGGACGCGTCTTGTGTATTTGGACGATATTATTGAAGCATGA

>NTK326_mRNA_39595_cds NTK326_mRNA_39595 gene_22838|id=AT5G17680.1

ATGGCATCATCTTCTGCTTCTGGTACTTCACAGTTTCCTCGATGGAACTACGATGTCTTC

CTAAGTTTTAGAGGTGAAGATACTCGGAAAACATTTACGAGTCACCTGTACGAAATCTTG

GATATCAGGGGAATAAAAACCTTTCAAGATGATAAAAGGCTAGAGCATGGCGCATCCATT

TCGGATGAACTATGTAAAGCTATCGAAGAGTCTCAATGTGCAGTCATCATTTTCTCAAAA

AATTATGCAACATCGAGGTGGTGCTTGAATGAACTAGTGAAGATCATGGATGTCAAGACT

CAATTTGGACAAACTGTCATACCGGTCTTCTATGATGTGGATCCATCACATGTTCGGAAC

CAGAGGGAGAGCTTTGCTGAAGCATTTTCCAAACATGAAACAAAGTATAAGGATGATGTC

GAAGGAATGCAAAGATGGAGGATTGCTTTAACTGCAGCGGCCAATCTCAAAGGTTGTGAT

ATTCGTGACAAGACTGAATCAGACTGTATTCGACAGATTGTTGATCAAATCTCGTCCAAA

TTATGCAAGATTTCTTTATCTTATTTGCAAAACATTGTTGGAATAGATACTCATTTAGAG

AAAATAGAATCCTTACTAGGGATAGGAATCAATGATGTTCGGATTGTGGGGATTTGGGGC

ATGGGGGGAGTCGGTAAAACGACAATAGCTAGAGCTATGTTTGATATTCTCTTAGTAAGA

AGGGATAGTTCCTATCAATTTGATGGTGCTTGTTTCCTTGCGAATATTAAAGAAAACAAA

CGTGGAATGCATTCTCTGCAAAATATTATTTTCTCTGAACTTTTAAAGGAAAAAGCTGAT

TACAACAATAAGGAGGACGGAAAGCACCAAATGGCTAGTAGGCTTCGTTCAAAGAAGGTC

CTAATTGTGCTTGATGACATAGATGATAAAGATCATTATTTGGAGTATTTAGCAGGTGAT

CTTGATTGGTTTGGTAATGGCAGTAGAATTATTATAACAACTAGAGACAAGCATTTGATG

GGGAAGAATGGTGTAATATATGAAGTGACTGCACTACCTAATCATGAATCCATTCAATTG

TTCTATCAGCATGCTTTCAAAAAAGAGGTTCCAAATGAGCATTTTAAGAAGCTTTCATTG

GAAGTCGTAAATTATGCTAAAGGCCTTCCTTTAGCCCTCAGAGTGTGGGGTTCTTTGCTG

CATAACCTAGGACTAACTGAATGGAAAAGTGCTATAGAGCACATGAAAAATAACTCTAAT

TCTGAAATTGTTAAAAAGCTCAAAATTAGTTATGATGGATTAATAGAGCCCATACAAGAG

ATTTTTCTGGATATAGCATGCTTCTTCCGAGGGACAAAAAAAGAGTACGCCATGCAAATT

CTTGAGAGCTGTCATTGTGCAGTTGAATACGGATTGCGTGTCTTAATTGACAAATCTCTT

GTGTCCATCTCTGAAAATGATCAGATTCAAATGCATGACTTGATGCAAGATATGGGTAAA

TATATAGTGAACTTGCAAAAGAATCCGGGAGAACGCAGCAGATTATGGCTCGACAAGGAT

TTCGAAGAAGTGATGATGAACAATACAGGGACCACGAAAATGGAAGCAATCTGGTTTCCT

TATTACCATTATGTTACATTACGCTTTGGCAAAGAGGCCATGAAAAATATGAAAAAGCTT

AGGATATTAAACATAGAGATGTCGTGGCCTTGTGATGGTTCCATTGAGTATCTGCCCAAC

AGCTTGCGTTGGTTTGTCTGGACTGACTATCCTTGGGAGTCGTTGCCAGCTGAATTTGAA

CCCAAAAAGCTTGTTCATCTTGCACTCAAATCCAGTTCACTGTGTTATTTATGGACGGAA

GCAAAGCAATTGTCGTCTCTACGGACGCTAGATCTCAGATACTCTGAAAGCCTAGTGCGA

ACACCAGATTTCACAGGGATGCCAAATTTGGAGTATTTGAATCTGGAGGAATGTCGTGAT

CTTGAAGAGGTGCACCATTCCCTGGGATTTTGCAGAAAACTCATTCGATTAAATTTGGAG

TCTTGTGGACGCCTTAATTGGTTTCCATGTGTTAACGTGGAATCTCTTGAATATCTGGAT

CTAGATTTTTGCTCTAGTTTAGAGAAATTTCCAGAAATCCATGGGAGAATGAAGCGGGAG

ATACAGATTCACATGAAACGCTCTGGGATAAGGGAACTACCATCATCTATTATTCAGTAC

CAAACTCATATTACCTTCCTAGATTTGAGCGCTATGAAAAACCTTGTAGCTCTTCCAAGC

AGCATCTGTAGGTTGAAAAGTTTGGTTAGTCTAAATGTGTCGGGCTGCACAAAACTTGAA

AGCTTGCCAGAAGAGATAGGGGATTTAGAAAACTTGGAGGAGCTTTATGCAAGGTATGCT

CTAATTTCACGACCTCCGTCTACCATCGTACGCTTGAACAAACTTAAAATCTTGAAGTTT

GGAGGCCTCCATGATGGAGTGCACTTTTTGTTCCCTCCAGTGGCTGAAGGATTACGGTCA

TTGGAACATCTGGATCTCACTTGTTGCAATCTAATAGATGAAGGACTTCCGGAAGACATT

GGATGCCTATCTTCTTTGAAAGAACTGTATCTCAGTGGTAATAATTTTGAGCATTTGCCT

CGAAGTATAGCCCAACTTGGTGCTCTTCGAATCTTGGACTTGAGAAATTGCAAGAGGCTT

ACACAGCTGCCAGAACTTCCACCAGAATCAGATACAATATATGCAGATTGGAGCAATGAT

TTGATCTGTAATTCGTTGTTTCAGAATATCTCGTCAGTGCAGCATGACATCTCTGCTTCA

GATTCCTTGTCACTAAGAGCATTTACCAGTGTGCATCTTGGGAAGAAGATCCCAAGTTGG

TTCCTCTATCAGGGAATGGATAGTGGTGTATCAGTCAATTTGCCTGGAAATTGGTATATA

CCTGATAAATTCTTGGGATTTGCTGTATATTACTCAGGCAGCTTAATTGACACCACAACT

CAATTGATTCCCGTATGTGATGATGGGATGTTGTGGATGACCCAGAAACTTGCCTTATCC

AACCATTCAGAATGTGATACAAAATATAATATTAATTTTTTCTTGGTACCTCTTGCTGGC

TTATGGGATACATCTAAGGCAAATGGAAAAACACCAAACGACTATGGGCTTATTAGGCTA

TCTTTTTCTGGAGTAATGAAGGATTATGGACTTCGTTTGTTGTATAAAGAAGAACCTGAG

CTTGAGGCCTTGTTACAGAATATTGCATTGGAGGAGCAGATATGA

>NTTN90_mRNA_87883_cds mRNA_87883 gene_49683|id=AT5G17680.1:evalue=1e-172:annot='disease resistance protein (TIR-NBS-LRR class), putative';id=Solyc11g011350.1.1:evalue=0.0:annot='Tir-nbs-lrr, resistance protein'

ATGGCATCTTCTTCTTCTTCTTCTAGATGGAGCTATGATGTTTTCTTAAGTTTTAGAGGC

GAAGATACTCGAAAAACGTTTACAAGTCACTTATACGAAGTCTTGAATGATAAGGGAATA

AAAACCTTTCAAGATGATAAAAGGCTAGAGTACGGCGCAACCATCCCAGGTGAACTCTGT

AAAGCTATAGAAGAGTCTCAATTTGCCATTGTTGTTTTCTCAGAGAATTATGCAACATCA

AGGTGGTGTTTGAATGAACTAGTGAAGATCATGGAATGCAAAACTCGATTTAAGCAAACT

GTTATACCGATATTCTATGATGTGGATCCATCACATGTTCGGAACCAAAAGGAGAGCTTT

GCAAAAGCCTTTGAAGAACATGAAACAAAGTATAAGGATGATGTTGAGGGAATACAAAGA

TGGAGGATTGCTTTAAATGAAGCGGCCAATCTCAAAGGCTCATGTGATAATCGTGACAAG

ACTGATGCAGACTGTATTCGACAGATTGTTGACCAAATCTCATCCAAATTATGCAAGATT

TCTTTATCTTATTTGCAAAACATTGTTGGAATAGATACTCATTTAGAGAAAATAGAATCC

TTACTAGAGATAGGAATCAATGGTGTTCGGATTATGGGGATCTGGGGAATGGGGGGAGTC

GGTAAAACAACAATAGCAAGAGCTATATTTGATACTCTTTTAGGAAGAATGGATAGTTCC

TATCAATTTGATGGTGCTTGTTTCCTTAAGGATATTAAAGAAAACAAACGTGGAATGCAT

TCTTTGCAAAATGCCCTTCTCTCTGAACTTTTAAGGGAAAAAGCTAATTACAATAATGAG

GAGGATGGAAAGCACCAAATGGCTAGTAGACTTCGTTCGAAGAAGGTCCTAATTGTGCTT

GATGATATAGATAATAAAGATCATTATTTGGAGTATTTAGCAGGTGATCTTGATTGGTTT

GGTAATGGTAGTAGAATTATTATAACAACTAGAGACAAGCATTTGATAGAGAAGAATGAT

ATAATATATGAGGTGACTGCACTACCCGATCATGAATCCATTCAATTGTTCAAACAACAT

GCTTTCGGAAAAGAAGTTCCAAATGAGAATTTTGAGAAGCTTTCATTAGAGGTAGTAAAT

TATGCTAAAGGCCTTCCTTTAGCCCTCAAAGTGTGGGGTTCTTTGCTGCATAACCTACGA

TTAACTGAATGGAAAAGTGCTATAGAGCACATGAAAAATAACTCTTATTCTGGAATTATT

GATAAGCTCAAAATAAGTTATGATGGATTAGAGCCCAAACAACAAGAGATGTTTTTAGAT

ATAGCATGCTTCTTGCGAGGGGAAGAAAAAGATTACATCCTACAAATCCTTGAGAGTTGT

CATATTGGAGCTGAATACGGGTTACGTATTTTAATTGACAAATCTCTTGTGTTCATCTCT

GAATATAATCAGGTTCAAATGCATGACTTAATACAGGATATGGGTAAATATATAGTGAAT

TTTCAAAAAGATCCCGGAGAACGTAGCAGATTATGGCTCGCCAAGGAAGTCGAAGAAGTG

ATGAGCAACAACACAGGGACCATGGCAATGGAAGCAATTTGGGTTTCTTCTTATTCTAGT

ACTCTACGCTTTAGCAATCAGGCCGTGAAAAATATGAAAAGGCTTAGGGTATTTAACATG

GGGAGGTCGTCGACACATTATGCCATCGATTATCTGCCCAACAACTTGCGTTGTTTTGTT

TGCACTAACTATCCTTGGGAGTCATTTCCATCTACATTTGAACTCAAAATGCTTGTTCAC

CTCCAACTCCGACACAATTCTCTGCGTCATTTATGGACAGAAACAAAGCATTTGCCGTCT

CTACGGAGGATAGATCTCAGCTGGTCTAAAAGATTGACGCGAACACCAGATTTCACGGGG

ATGCCAAATTTGGAGTATGTGAATTTGTATCAATGTAGTAATCTTGAAGAAGTTCACCAT

TCCCTGGGATGTTGCAGCAAAGTCATTGGTTTATATTTGAATGATTGTAAAAGCCTTAAG

AGGTTTCCATGTGTTAACGTGGAATCTCTTGAATATCTGGGTCTAAGAAGTTGCGATAGT

TTAGAGAAATTGCCAGAAATCTACGGGAGAATGAAGCCGGAGATACAGATTCACATGCAA

GGCTCTGGGATAAGGGAACTACCATCATCTATTTTTCAGTACAAAACTCATGTTACCAAG

CTATTGTTGTGGAATATGAAAAACCTTGTAGCTCTTCCAAGCAGCATATGTAGGTTGAAA

AGTTTGGTTAGTCTGAGTGTGTCGGGTTGCTCAAAACTTGAAAGCTTGCCAGAAGAGATA

GGGGATTTAGACAACTTACGGGTGTTTGATGCCAGTGATACTCTAATTTTACGACCTCCG

TCTTCCATCATACGCTTGAACAAACTTATAATCTTGATGTTTCGAGGCTTCAAAGATGGA

GTGCACTTTGAGTTCCCTCCTGTGGCTGAAGGATTACACTCATTGGAATATCTGAATCTC

AGTTACTGCAATCTAATAGATGGAGGACTTCCGGAAGATATTGGATCCTTATCCTCTTTG

AAAAAGTTGGATCTCAGTAGAAATAATTTTGAGCATTTGCCTTCAAGTATAGCCCAACTT

GGTGCTCTTCAATCCTTAGACTTAAAAGATTGCCAGAGGCTTACACAGCTACCAGAACTT

CCCCCAGAATTAAATGAATTGCATGTAGATTGTCATATGGCTCTGAAATTTATCCATGAT

TTAGTAACAAAGAGAAAGAAACTACATAGAGTGAAACTTGATGATGCACACAATGATACT

ATGTACAATTTGTTTGCATATACCATGTTTCAGAATATCTCTTCCATGAGGCATGACATC

TCTGCTTCAGATTCCTTGTCACTAACAGTATTTACCGGTCAACCGTATCCTGAAAAGATC

CCGAGTTGGTTCCACCATCAGGGTTGGGATAGTAGTGTATCAGTCAATTTGCCTGAAAAT

TGGTATATACCTGATAAATTCTTGGGATTTGCTGTATGTTACTCTCGTAGCTTAATTGAC

ACAACAGCTCACTTGATTCCCGTATGTGATGACAAGATGTCGCGCATGACCCAGAAACTT

GCCTTATCAGAATGTGATACAGAATCATCCAACTATTCAGAATGGGATATACATTTTTTC

TTTGTACCTTTTGCTGGCTTATGGGATACATCTAAGGCAAATGGAAAAACACCAAATGAT

TATGGGATTATTAGGCTATCTTTTTCTGGAGAAGAGAAGATGTATGGACTTCGTTTGTTG

TATAAAGAAGGACCAGAGGTTAATGCCTTGTTACAAATGAGGGAAAATAGCAATGAACCA

ACAGAACATTCCACTGGGATAAGGAGGACTCAATATAACAACAGAACTTCCTTTTATGAG

CTCATCAATGGGTGA

>NTTN90_mRNA_46474_cds mRNA_46474 gene_26069|id=AT5G36930.1:evalue=8e-177:annot='Disease resistance protein (TIR-NBS-LRR class) family';id=Solyc09g092410.2.1:evalue=0.0:annot='Tir-nbs-lrr, resistance protein'

ATGGATAGTCAATTAGTAAGAGGAGAATCATGTACATCTTCTCACTTCTCTTATGAAGTA

TTCCTCAGTTTTAGAGGTGAAGACACCCGAAAAACATTCACTGACCATCTTTATTCCAAA

TTGCGTGATGTTGGAGTCAATACCTTCATTGATGAGGAATTGAGAAAGGGTGATGTGATT

TCAAGTAAACTAGAGAAAGCAATTGAAGAGTCAAGAATTTCCATTATTGTTTTCTCAAGA

AATTATGCTTCCTCTAGTTGGTGTCTAAATGAACTAGTTAAAATTCTTGAATGCAAAGAG

AAATTAAAGCAGATGGTTTTGCCTATTTTCTACGATGTTGATCCTTCTGAGGTACGAAAG

CTAACTGGGTTATTTGGTGAACCATTTGGAGCTCAAAGGGTGGAGAAATGGAGAGCTGCA

CTTACTGAAGCTGCAAATTTATCTGGATGGCATTTGCAAAATGTTGCTGACGGGCATGAA

TCAAAGTTTATTGAAAAAATTATACAGCAAGTTCTACAAGAGGTCAACCAGACACCTCTA

GATGTTGCTTGGCACCCAGTTGGAGTAGATTCTCGTGTTAAAGATATAGAGTTATTATTG

CAAAATGAATATAAAGATGAAGTTCTAATTATTGGTATTCATGGAGTTGGTGGCATAGGG

AAAACAACTCTGGCAAAAGCTATCTACAATCAAATGTTTCGACTCTTCGATAGGAGTTGC

TTCCTTTCAGATGTTAGATCAGAAGTTGAAGAATTTGGTCTTGTCAAGCTACAAGAGAAA

CTTCTTCAACAAGTTCTCAAAACCGAGGACATCAAAGTTGGGAGTGTTGCTCAAGGCGTT

AATCTAATCAAAGCAAGACTTGGGTCAAAGAAAGTTCTAATTGTTCTTGATGATGTGGAC

CATAAAAGACAGTTAGAAGTCTTAACAAGAGAGAGAAGTTGGTTTGGTTCAGGTAGTTTA

ATAATCATTACCACCCGAGACGAGCGATTGCTATGTCGGCTTGGAGAAAAAGAGAGATAT

GAGGCCAAACTATTAAGTGGCAATGAAGCTATGTTACTTTTTTGTTGGCATGCTTTTGAC

AGTGATTTTCCACCACAAGATTATGTTAATTTGGCACACGACATAATCGAATATTCAGGT

AGGCTGCCATTAGCTCTTGTGACATTGGGGTCACATTTACAAGGAAGTTCTGTAGAAGAA

TGGGGATATGAATTAGAAAAACTAAGAGCAATTCCTCATTGTGATATCCAAAAGATTCTC

AAGATAAGCTTTGATGGGCTTGATGGTGAAACACAGATTGTTTTCCTCGATATTGCATGT

GCCTTCCATGAGGTTGATGAGCATGAAGTTACTGAAATATTAAATGCATGTGGCTTTCAT

GCTAAAATTGCAATTGCAACTTTAGTCCAAAAACACTTGCTCCAAAGAACTCCTGATCAT

TTGGTGATGCATGATCTAGTGCGAGATATGGGAAGAGAAATCGTTCGCTTGGAATCACCT

CGAGACCCCGGAAAACGAAGTAGATTGTTCATCCCTCGAGAAGTTCGTGATGTTCTACAA

GGAAATGAAGGTTCCGAAAATGTAGAAGTACTAAAGGTAGATCGAGGGACATTAAACGGA

GTGAACTTGAGCACCAAAGCATTTGAACAAATGAAAAACCTTAGGGTTCTTATAATGGAT

GAGTTACATATTAGTGGAGATTTTGGGTTGTTGTCCAAGAAGCTCAGATGGTTGTCTTGG

AAAAAATGTCCTTTAAAATGTATACCGTCAAATTTTCCAGCCGAGAATCTTGTAGTTCTA

GATATGCGGGAGAGTGATATCCAAGAATTTCAATTGGATTTGCAGTGTTGTAAAAGTTTG

AAGGAGCTGAATCTCTCTCATTGCAAGCAACTCAGAAGCACTCCAAACTTCAATGGTTCA

CTGAGTCTTGAGAATTTGCATCTTTATGGTTGCTCAAGTCTGACAGAGATCCATCCATCA

ATCGGAAATTTGTCCAGACTATGCGAGCTATCTATGTCTGGTTGCAATAGACTTATGGAT

CTTCCAAACAGCATATGCCAGCTAATATCCGTTAATTACTTGAGCATTAGTAACTGCTCA

TCAATAAAAACACTGCCAGATAACCTTGGAGATATGAAAAGTCTAAGATCTCTTTATGCA

TCTGGTACGGGTATAAAACAATTGCCTAGATCTGTTGAAATGCTAAGAAATCTTGAAACT

TTGAGCGTGGAAGGTCGAAAGCTAGAGGCCAAAAGGAGTATTTCTGGAAGAGGAGTCCAT

CGGATACAATATTCCTTGTCAACATTTGTATCCAATTTGAGCCTTACATACTGTAATTTG

TCCGAGGCTGATATTCCAAGGAATATTGGGAGCTTATCCTCCTTAGAATATTTAGATTTG

AGTGGCAACAGTTTCCGTTGTCTACCCATTGATTTTTCTAAGTTACGATTGTTGGAGAAG

TTAAGTTTGAAGGACTGTGAGAATCTCCAAACACTCCCGTCAGTATCAAATTTAGAGAAT

CTTTATACAATTAAACTTGAGAATTGCCGAAAATTGGTGAAGATTACAGAGTTGGACAAC

CTCCCTTCTATAGAGCAGATTAATATGATTAATTGTAATTCTCTGCAGAATCCATTCAAT

GAAGGCTTCTTTAGTGCACCTGCTCTATATGCATCTAGAAATGATCGATATATGATGCAG

GCTACTGTTAGAATTTATCTCGAATGTGATGAGATTCCAAAATGGTGCAAGAATCAAGTA

ACAGCTTCATCTATGTGTTTGACTTTGCCAACACATAATAATGAGGAGTATAACTTCTTA

GGAATGGTTCTCTGGTTTGTTTCCGACTTGTTCGATGTAGTCCCGTATAATCCGTGCTTC

AAGATTAGTATTGCCCATGGAAAGCCTTCAATTATTCGGTGGAGTAAAGGGAAAAAACCT

CTGTCATGTGTATATTACGTATCTTACTTACATAAAGCTTTAGATGGCCAAATGATCAAA

GGCGGGGAAACGATAAAAGTGTGGGCTCAAAACATTACAGTAAAGAAGATAGGGATCCAT

CTGTTATATTTAGACCAACATGGTAATGTTATATCTTTACCGGGAGACATGGATCATCCT

TATTCTAGGGTGAAAGATGTCATGTTCAGGAATTGGCGGGAAGAGTTTCTTAGTTTGGCA

GACACAACAGGAGAAATTTGA

>NTBX_mRNA_42672_cds NTBX_mRNA_42672 gene_24966|id=AT5G17680.1

ATGGCATCATCTTCTACTTTTGCGAGTACTTCACAGTTTCCTCGATGGAACTATGATGTC

TTTCTAAGCTTTAGAGGTGAAGATACTCGGAAAACATTTACGAGTCACCTGTACGAAATC

TTGGATATCAGGGGAATAAAAACCTTTCAAGATGATAAAAGGCTAGAGCATGGCGCATCC

ATCTCAGATGAACTCTGTAAAGCTATCGAAGAGTCTCAATGTGCTGTCATCATTTTCTCA

AAGAATTATGCAACATCGAGGTGGTGCTTGAATGAACTAGTGAAGATCATGAATTGCAAG

ACTCAATTTGGACAAACTGTAATACCAGTCTTCTATGATGTGGATCCATCATATGTTCGG

AACCAGAGGGAGAGCTTTGCTGAAGCATTTGCCAAACATGAAACAAAGTATATGGATGAT

GTCGAAGGAATACAAAGTTGGAGGATTGCTTTAACTGCAGCGGCCAATCTCAAAGGCTGT

GATATTCGTGACAAGACTGAATCAGACTGTATTCGACAGATTGTTGACCGAATCTCGTCC

AAATTATGCAAGATTTCTTTATCTTATTTGCAAAACATTGTTGGAATAGATACTCATTTA

AAGGAAATAGAATCCTTACTAGGGATAGGAATCAATGATGTTCGGATTGTGGGGATTTGG

GGCATGGGGGGAGTCGGTAAAACGACAATAGCTAGAGCTATGTTTGATACTCTCTTAGTA

AAAAGGGATAGTTCTTATCAATTTGATGGTGCTTGTTTCCTTGCGAATATTAAAGAAAAC

AAACGTGGAATGCATTCTCTGCAAAATATCCTTCTCTCTGAACTTTTAAAGGAAAAAGCT

AATAACAATAGTGAGGAGGACGGAAAGCACCAAATGGCTAGTAGGTTTCGTTCTAAGAAG

GTCCTAATTGTGCTTGATGACATAGATGATAAAGATCATTATTTGGAGTATTTAGCAGGT

CATCTTGATTGGTTTGGTAATGGCAGTAGAATTATTGTAACAACTAGAGACAAGCATTTG

ATAGGGAAGAATGATGTAATATATGAAGTGACTGCACTACCTGACCATGAATCCATTCAA

TTGTTCTATCAGCATGCTTTCAAAAAAGAGGTTCCAAATGAGCATTTTAAGGAGCTTTCA

TTGGAGGTAGTAAATTATGCTAAAGGCCTTCCTTTAGCCCTCAGAGTGTGGGGTTCTTTG

CTACATAACCTAGGCCTAACTGAATGGAAAAGTGCTATAGAGCACATGAAAAATTACTCT

AATTCTGGAATTGTTGATAAGCTCAAAATTAGTTATGATGGATTAGAGCCCAAACAACAA

GAGATGTTTCTAGATATAGCATGCTTCTTCCGAGGGGCAAAAAAAGAGTATGCCATGCAA

ATTCTTGAGAGTTGTCATTGTGCAGCTGAATACGGATTGCGTGTCTTAATTGACAAATCT

CTTGTGTCCATCATTGAAAATGATCGTATTCAAATGCATGACTTAATGCAAGATATGGGT

AAATATATAGTGAACTTGCAAAAGGATTCGGGGGAATGCAGCAGGCTATGGCTCGACGAG

GATTTTGAAGAAGTGATGATCAACAATAAGGGGACCACGAAAATGGAAGCAATCTGGTTT

CCTTATTACCATTGTGGTACATTACGCTTTAGCAAAGAGGCCATGAAAAATATGAAAAAG

CTTAGGATATTAAACATAGAGAGGTCGTGTACCTGTGATGGTTCTATTGAGTATCTGCCC

AACAACTTGCGTTGGTTTGTATGGAAATGCTATCCTTGGGAGTCATTGCCAGCTGAATTT

GAACCCAAAAAGCTTGTTCATCTTGCAGTCAAATCCAGTTCACTATGTTATTTATGGACG

GGAACAAAGCAATTGTCGTCTCTACGGACGCTAGATCTCAGATACTCTGAAAGCCTGGTG

CGAACGCCAGATTTCACGGGGATGCCAAATTTGGAGTATTTGAATCTGGAGGAATGTTGT

GATCTTGAAGAGGTTCACCATTCCTTGGGATTTTGCAGAAAACTCATTCGGTTAAATTTG

GAGTCTTGTGGACGCCTTAATTGGTTTCCATGTGTTAACGTGGAATCTCTTGAATATCTG

GATCTAGATTTTTGCTGTAGTTTAGAGAAATTTCCAGAAATCCATGGGAGAATGAAGCCG

GAGATACAGATTCACATGAAACGCTCTGGGATAAGGGAACTACCATCATCTATTATTCAG

TACCAAACTCATATTACCTTCCTAGATTTGAGCGCTATGAAAAACCTTGTAGCTCTTCCA

AGCAGCATCTGTAGGTTGAAAAGTTTGGTTAGTCTAAATGTGTCGGGCTGCACAAAACTT

GAAAGCTTGCCAGAAGAGATAGGGGATTTAGAAAACTTGGAGGAGCTTTATGCAAGGTAT

GCTCTAATTTCACGACCTCCGTCTACCATCGTACGCTTGAACAAACTTAAAATCCTGAAG

TTTGGAGGCCTCCAAGATGGAGTGCACTTTGAGTTCCCTCCAGCGGCTGAAGGATTACGG

TCATTGGAACATCTGGATCTCACTTGTTGCAATCTAATAGATGGAGGACTTCCGGAAGAC

ATTGGATGCCTATCTTCTTTGAAAGAATTGTATCTCAGTGGAAATAATTTTGAGCATTTG

CCTCGAAGTATAGCCCAACTTGGTGCTCTTCGAATCTTGAACTTAAGAAATTGCAAGAGG

CTTACACAGCTGCCAGAACTTCCACCAGAATCAGATACAATATATGCAGATTGGAGCAAT

GATTTGATCTGTAATTCGTTGTTTCAGAATATCTCGTCAGTGCAGCATGACATCTCTGCT

TCAGATTCCTTGTCACTAAGAGTATTTACCAGTGTGCATCTTGGGAAGAAGCCAAGTTGG

TTCCTCTATCAGGGAACAGATAGTGGTGTATCAGTCAATACGGATAGTGGTGTGTCAGTC

AATTTGCCTGGAAATTGGTATATACCTGATAAATTCTTGGGATTTGCTGTATATTACTCT

GGCAGCTTAATTGGCACCACAGCTCAATTGATTCCCGTATGTGATGATGGGATGTTGTGG

ATGACCCAGAAACTTGCCTTATCCAACCATTCAGAATGTGATACAAAATATAATATTAAT

TTTTTCTTGGTACCTCTTGCTGGCTTATGGGATACATCTAAGGCAAATGGAAAAACACCA

AATGACTATGGGCTTATTAGGCTATCTTTTTCTGGAGAAGTGAAGAAGTGTGGACTTCGT

TTGTTGTATAAAGAAGAACCTGAGGTTGAGGCCTTGTTACAAATGAGGGAAAATAACCAT

GAACCAACAGAACATTCCACTTGGATAAGGAGGACCCGATATAACAATAGTGAACACGAC

TTCGTGATCAATGAAGCCAGCTGCTCCTCGGGTAAGAAACAAAAGAGTCACATTTCTAAT

ATTCAGGGGAGCTCTGTCTTTGAGAATCTGCAGCAACAAGTAGAGGGGCCTGTCTCTTCA

GAAACTTTGCAGCTCTTTCCTGCAAACCCAGGATTTTAG

>NTBX_mRNA_109159_cds NTBX_mRNA_109159 gene_65343|id=AT5G17680.1

ATGGCATTATCTTCTGCTTCTACAACTACTTCACAGTTTCGGTGGAACTACAAAGTCTTT

CTAAGCTTTAGAGGTAAAGATACTCGAAGCAAATTTACAAGTCACCTCTTCAAAGGCTTG

GAAAGCAGTGGAATATTCACGTTTCAAGATGATAAAAGGCTAGAGCATGGTGCATCAATA

TCAGATGAACTCCTGAAAGCTATCGAACAGTCTCAAGTTGCCCTTGTCGTTTTCTCACAG

AATTATGCAACATCGAGGTGGTGCTTAGATGAGTTAGTAAAGATCATGGAATGCAAGGCT

CAATGTGGACATACTGTCATACCAGTCTTCTATGATGTGGATCCAGCACATGTTCGATAC

CAGAGGAAGAGCTTTGCCAAAGCCTTTAAGAAACACGAAACAAGATATAAGGATGATGAT

GAAGGAATGCAGAAGGTCCAAAGATGGAGGAATGCTCTAACTGCTGCTGCAAATCTAAAA

GGATATGATATCCGTGCCGGGATTGAGGCAGAGAATATTCAGCACATTGTCGACCAAATT

TCCAAATTGTGCAATAGTGCTAGTTTATCTTCTTTGCGAGATGTTGTCGGGATAGATACT

CATCTGGAGAAATTAAAGTCCCTACTTAAGGTAGGAACCAATGATGTTCGGATCATATTG

GGGATCTGGGGCATGGGGGGTCTAGGGAAGACGACAATAGCAAGAGCCATTTTTGACACT

TTATCTCATCAATTTGAAGCTGTTTGTTTCCTTGCGGATATTAAAGAAAATGAAAAAAGA

CATCAACTGTATTCTTTGCAAAACACCCTTCTCTCTGAATTGTTAAGAAGAAAAGATGAT

TACGTCAATAATAAGCATGATGGGAAGCGGATGATTCCGGCTAGACTTTGCTCTAAGAAG

GTGCCAATTGTGCTTGATGATATAGATCATAAAGATCATTTAGAGTATTTAGCGGGTGAT

ATTGGTTGGTTTGATAATGGCAGTAGAGTTGTTGTCACAACTAGAAACAAGCATTTGATA

GAGAAGAATGATGTCATTTATGAAGTGACTGGACTAGCTGACCATGAAGCTATGCAGTTA

TTCAGTCAACATGCTTTCAGAAAAGAAGATCCAGATGAGTGTTTTAAGGAGCTCTCATTG

GAGGTAGTAAATTATGCTAAAGGTCTTCCTTTAGCCCTCAAAGTGTGGGGTTCTTTGCTG

CATAACTTAGGCTTAACTGAATGGAAAAGTGCTATAAAGCACATGAAAATTAATTCTAAT

TCGGAAATTGTTGAAAAGCTCAAAATCAGTTATGACGGATTGGAGCCCATCCACCAAGAG

ATGTTTCTAGATATAGCATGCTTCTTGCGAGGGGAAAAAAACGATTACGCCGTGCAAATT

CTTGAGAGTTGTCATTCTGGAGTTGAATACGGATTGCGTATTTTAATTGACAAATCCCTT

GTGTCTATCTCTGAAAATGATCGAATTCAAATGCATGACTTAATACATGATATGGGTAAA

TATATAGTGAATTTTAAAAAAGATCCTGGAGAACGTAGCAGGCTATGGCTCGCCGAGGAA

GTCGAAGAAGTGATGAGCAACAATGCAGGGACCATGGCAGTGGAAGCAATTTGGCTTCAT

GGTAATTTTAGTACACTACGCTTTAACAATGAGGCCATGAAAAATATGAAAAGGCTTAGG

ATATTATACATAGACAAAGAGTTCTATGATTTCAATATTTGGGATGATGGCTCCATTGAG

TATCTGTCCAACAACTTGCGTTGGTTAGTCTTGGATGGCTATCCTTGTGAAACATTGCCA

TCTACATTTGATCCCAAAATGCTTGTTAACCTTCAACTCCACCTTAGTTCACTGCGTTAT

TTATGGACGGAAACAAAGCAATTGTCGTCTCTACGGACGCTAGATCTCAGATACTCTAAA

AGCTTGGTGCGAACACCAGATTTCACGGGGATGCCAAATTTGGAGTATTTGGATCTGTTT

TGGTGTTATAATCTTGAAGAGGTTCACCATTCCCTGGGATGTTGCAGCAAACTCATTTGG

TTAGATTTGAGTTGGTGTCAAAGCCTTAAGAGATTTCCATGTGTTAGCGTGGATTCTCTT

GAATATCTGAGTTTAGATGGTTGCTTTAGGTTAGAGAAATTTCCAGAAATCCACGGGAGA

ATGAAGCCGGAGATACATATTCGCATGGGAGCCTCTGGGATAAGGGAAATACCATCATCT

ATTTTTCAGTACCAGACTCATATTACCAAGCTAGATTTCAGCGGTATGAGATGA

>NTK326_mRNA_26472_cds NTK326_mRNA_26472 gene_15235|id=AT4G12010.1

ATGGATTCCCAAAAGGAATTGAACAAAAAATGGGAATATGATGCCTTCTTGAGCTTTAGA

GGTGAAGATACCCGCAACAACTTTGTGGCCCATCTCCACAAACGTTTGGAAGAGAGAGGA

GTCAACGTATTCAAAGACGATGAGAAACTTGAAAGAGGAAGACCCATTTCAGCTGAACTA

TTGAAAGCCATTGAAGAATCAAGAGTTGCCATAATCATATTCTCCAAAAATTATGCTTCA

TCGGTATGGTGTTTGGAGGAACTCACCAAACTTATGGAGTGTGTTGAAAAGAAGGGACAG

GAAGCTATCCCTATATTCTACAATGTTGACCCATCAGATGTACGCATGCAAAGATCCAAA

AGTAGTTTCGCAAAAGCGATGGCCAAACACAAGGCTAATTTCGAGGGTAATGATTTGGAG

AAGGTGCAGAGGTGGACTGACGCTCTCCAAAAAGCAGCCAATATAGCAGGATGGGATCTT

GGTAAATGTGCTAACGGGAATGAAGCAGAATGCATTGATCGGATTGTACACGAGAAATTT

CAAAATGTGCACCACACAGTTTCAGCAACTGAGAAGTATTTAGTGGGAGTCGAATCTCGT

ACTGGTGGAGTGGAATCAATGTTGAAAGTTGGATCAGGAGGTGTTTATTTTGTGGGAATA

TGGGGAATGGGTGGCGTGGGGAAGACAACGGTCGCAAGAAAAATCTTTGACAATATTTCT

AATCAGTTTCAAGGGTCTTGTTTTCTTGCAAATGTTAGAGAAGAATCAAAGAAGCATGGG

ATAAAACACTTGCAGAAGACACTTCTTTCAAGAATCTTAAATGAAAAATCTTTGAAGGTA

GCAAGTTTTTATGAAGGAGCTGACATGTTAAAAGGAAAGTTTTGTCTCAGGAAGGTTTTA

ATTGTTTTTGATGATGTGGATGACAACCACCAGTTGGAGTATTTAGTTGGAAAGCATGAT

TGGTTTGGTGATGGCAGTAGAATTGTTACAACAACCCGAAATGCAGATTTACTTCGTTGC

CACGACGAGTTATATTCTGTCCCTGAATTGGCGAAATGGGGGGCTCTTGAACTTTTTAGT

TGGCATGCCTTTCAGAAGAGGACTCCAGATAAAGAGTTTTTAAAACTCTCTAAGTCTGTA

GTAGATTATGCTAAAGGTTTACCCTTAGCTCTTACGGTATTGGGTTCTTTTCTCTACAAA

CGAGGCATAACTGAGTGGGGAAGCGCATTGGATAGACTCAAAGATACTGGATATGAAGAA

ATTGTTAAGCAGCTCAGCTTAAGTGTAGATGGATTGTCCTATGAAGACAAGAATATATTT

CTTGATATTGCATGCTTCTTTAGAGGAAAAAGGAGAGATTTTGTGATAACAATACTAAAT

AGTTTTGGCTTCAAATCAGAGATTGGAATAGATGTCCTTACAAAGAAATCACTTCTTTAT

ATTTCAGAAGGAATGGTTGAGATGCATGATTTGATTGAACAAATGGGTCAGCAATTGGCA

CGTGATGTTGATCAGGACAAACCGTGGAACCATAGTAGAATATGGCATGAAAACGATATA

GAAACTGTTTTTTCTGCAAATCGGTGGACAGAGTCAGTAAAAGGCATAATGGTACCAATT

GGCTCAGACCGACATATATGCAAGTGGAGCAAAGCTTTCAGAAATATGCCTTGTCTTAGG

TTACTCATGGTCAAAGGGGAGGAGGTCCGACATTATGAACAAGTTTCTGACACTATTGAA

TATCTCCCCAGTAGCTTGAAATGGCTTGATTGGTCTTACTACAGTTTTGAATCTTTACCA

ACAAATTTTCAACCAAGAAACCTGGTTGGGCTCAACATGACTTTTAGTTCTCTTGTTGAA

ATTTGCAAGGAACCAAAGGCATTTGTCAAGTTGACGATTCTCAATTTAAGTTTTTCAGAG

AATTTACTCCGAACACCCAATTTTTCTGAGATTCCAAACCTGCAGAGGATAATACTGAAA

AGTTGTTTAAGCTTGAAAGATGTTCATCCATCCATTGGCAATCTCAAAAAGCTTGTTTCT

CTGAACATGAAGAACTGTAAAAATCTCAAGTTTTTACCAAGCAGTATTCAAATGGAATCT

CTTGAATGCCTCAACCTCTCTGGTTGTGAGAAATTAGATACTCTTCCAGAAATTCGGGGG

AATATGGAATTGCTATCAGAGCTTCTTTTGGGACGTACTGCAATTCGGGAACTACCCTCA

TCAATAGGACGGCTCTTTGGTATTAGTTTGCTTGATTTGCATTCATGTGAAAATCTTGTA

AGACTCCCAGCCAGTGTTAGCGAGATGAGAAAACTGAAAGTTTTAATTCTTAAAGGCTGC

TTAAAACTGGCAACTTTTCCAGAAAGCCTGGGTGATCTAGAAGAATTGGAGGAGCTCTAT

GCTGGAAACACTGCCGTTTGGCGACTACCAGATTCTATGGAAAACTTAAGCAAACTTAAA

ATCCTATCATTAAAAGGTAGACGAGAGATGAAGTGTCAATCTGCTACAGGTTTGATATTC

CCTTGTGCATTTCATGGTTTGAGGGCATTGAAAAGTTTGGATCTCAGTGGATGCAATTTA

TCTGGTGACGAGATTAATGGTCTTACGTGCTTGACTTCTCTATTGGAACTAAACCTTAGC

AGAAACAAGTTTATTTCTCTACCTGATGGCATCAGTCAACTTTATCAACTTCGATATCTC

AACATAACACACTGTCATGAACTTAAGAAACTTCCCAAACTTCCTCCAAGTATAAAGGAA

TTGTATGCAGAAGATTTTCTGGCCAAACAAATTATTCTAGCTATGTCGGTATACCGAGGG

TTGTGTTTGGCCTCATTCACCAACTATAGTTTTGATCAACAACCATACACAGAGAAGAGC

GATGATAACTCAGTGTTGGATGAGATTCTGAGCTTGTTTCTTTCAGACAGCATGGATGAT

AAGATGACTTATTGTATTATCTTTCCTGAACGTGCTATCCCCACATGGTTTAAACATCAG

AGTACTGAGGAAAAGATCTTGCTTAAACTGCCTGAGGATTGGTATGATGATAGATTTGAG

GGCTTTGCTATATGCTGTGTCACTTGCATGGGGGCAGGTGTCCACGATCCTGATTCAGGG

CTATCAGGGAAGTACGACTATACTTTCATCAAAGCCAAATTGATATGCAATAATCATATG

GAAGAGCTTGAAGTGCTGGAGAAAGAGTGTAAAGTCAGTACAACGTCTAGAACTTATAGC

TGGTGTGTTTGCTTTGCCTACATACCATTGTATTCTTTGCTGCAGACTTCTGGCACACAA

GTTCTAAACTTTAACCAGTATGGCCTATTCGAGGCATCTATCCAAAGGCACATTACGAGA

CAATGGGGAGTTCATTTGATTTACAAGTCTGAAAGACAATTTTTCGAGAGCAGAACTGAG

AAAGGGTTGCTTGCCCAGAAGTTGAGTCTAGTCAAGCCAATTCGAGTCAAAACGAGAATT

GTTCGATTAGAATTACCTAAATAG

>NTTN90_mRNA_72761_cds mRNA_72761 gene_41127|id=AT5G36930.1:evalue=5e-176:annot='Disease resistance protein (TIR-NBS-LRR class) family';id=Solyc09g092410.2.1:evalue=0.0:annot='Tir-nbs-lrr, resistance protein'

ATGGATCCTCTAATTATCCAAGAAGAATCATCTACATCTTCCCACTTCACTTATGAAGTA

TTCCTGAGTTTTAGAGGTGAAGACACCCGAAAAACATTCACTGGCCATCTTTATTCCAAA

TTGCGTGATGTTGGAGTCAAAACCTTCATTGACGATGAGGAATTGAGAAAGGGTGACGTG

ATTTCAAGAGAATTAGAGAAAGCAATTGAAGAGTCAAGAATTTCCATTATTGTTTTCTCA

AGAAATTATGCTTCCTCTAGTTGGTGTCTAAATGAACTAGTTAAAATTCTTGAATGCAAA

GAGAAACTAAAGCAGATGGTTTTGCCTATTTTCTATGATGTTGATCCTTCTGAGGTGCGA

AGACAAACTGGGTTATTTGGGGAAGCTTTTGCAAAACATAAGGAACGACCATTTGGAGCT

CAAAGGGTGGAGAAATGGAGAGCTGCACTTACTGAAGCTGCAAATTTATCTGGATGGGAT

TTGCAAAATGTTGCTGACGGGCATGAATCAAAGTTTATTGAAAAAATTATACAGCAAGTC

CTACAAGAGGTTAACCAAACACCTCTAGATGTTGCTTGGCACCCAGTTGGAGTAGATTCT

CATGTCAAAGATATAGAGTTGTTATTGCAAAATGAATGTGAAGATAGAGTTCACATGATT

GGTATTCATGGAGTTGGTGGCATAGGGAAAACAACTCTAGCAAAAGCTATCTACAATCAA

ATGTTTCGACTCTTCGACAGTAGTTGCTTCCTTTCAGATGTTAGATCAGAAGCTGAAGAA

TTTGGTCTTGTCAAGCTACAAGAGAAACTTCTTCAACAAGTACTCAAAAATAAGGACATC

AAAGTTGGCAGTGTCGCTCAAGGCATCTATTTAATCAAAGAAAGACTCGAGTCGAAGAAG

GTTCTAATTGTTCTTGATGACGTGGACCACAGAAACCAATTAGAATCCTTAACAAGAGAA

AGAAGTTGGTTTGGTTCGGGTAGTTTAATAATCACTACCACCCGAGACAAGCGATTGCTA

TGTCGGTTTGGAGAAAAAGAGAGATATGAGGCCAAACTATTAAATGACAATGAAGCTATG

GTACTTTTTTGTTGGCATGCTTTTGATAGTCATTTTTCACCAGAAGATTATGTTAATTTG

GCACGAGACATAATCAGATATTCAGGTAGGCTACCATTAGCTCTTGTGACATTGGGGTCA

CATTTACAAGGAAGTTCTATAGAAGAATGGGGATATGAATTCGAAAAACTAAAATCAATT

CCTCATTGTGATATCCAAAAGATTCTCAAGATAAGCTTTGATGGACTTGATGATGGAACA

CGGAGTGTTTTCCTCGATATTGCATGCGCCTTTCATGGGTTTGATGAGCATGAAGTTACT

GAAACATTAAATGCATGCGGCTTTCATGCTGAAAGTGCAATTGCAACTTTAGTCCAAAAA

CACTTGCTCCAAAGATCTTGGAATCATTTGTTGGTGATGCATGATCTAGTGCGAGATATG

GGAAGAGAAATCGTTCGCATGGAATCACCTCGAGACCCTGGAAAACGGAGTAGATTATTC

ATCCCTCAAGAAGTCCGTGATGTTCTACAAGGAAATAAAGGTTCCAAAAAGGTAGAAGTA

CTGAAGGTAGATCGACGAGAATTTAAGGGAGAGAATTTGAGCACCAAAGCATTTAAGAAA

ATGAAAAACCTTAGGGTTCTTATAATGGATGAGTTACATATTAATGGAGATTTTGAGTTG

TTATCCAAGGAGCTCAGATGGTTGTCTTGGAAAAGATGTCCTTTAAAATGTATACCATCA

AATTTTCCAGCTGAGAATCTTGTAGTTCTAGATATGCGGGAGAGTGATATCCTAGAATTT

CAATTGAATTTGCAGTGTTGTACAAGTTTGAAGAAGCTGGATCTCTCTTGTTGCAAGCAA

CTCAGAAGCACTCCAAACTTCAATGGTTCACGAAGTCTTGAGACTTTGCTACTTGGTGGT

TGCTCAAGTCTGACGGAGATCCATCCATCAATAGAAAATTTGTTCAGTCTAATTATACTA

TGTATGCCATATTGCAAGAAACTTAGGGATCTTCCAAGCAGCATATGCCAGCTAAAATCC

CTTGAAGACTTGGACATCGATGGCTGCTTATCTATAAAAACACTGCCAGATAACCTTGGA

GACTTGAAAAGTCTAAGATCTCTTCAAGCGAGTTATACGGGTATAAAACAATTGCCTAGA

TCCGTTGAAATGCTAAGAAATCTTAAAACTTTGAGAGTGGAAGGTCGAAAGCTAAAGGTC

GAAAGGAGTATTTCTGAAAGAGGATTCCATCGGGTACAATATTCCTTGCCAACCTTTGTA

TCCGATTTGATCCTTACATACTATTATTTGTCTGAGGCTGATATTCCTAGGAATATTGGG

AGCTTATCCTCCTTAGAATATTTAGATTTGAGTGGCAACAGTTTTCATTGTCTACCCTTT

GATTTTTCTAAGTTACGATTATTGAAGAGGTTGTGGTTGGTTGACTGTGAGAATCTTCAA

ACACTCCCGTCAATATCAAATTTAGAGAATCTTAGAGTACTTCGAATTAAATATTGCCAA

AAGTTGGTCAAGATTACAAAGTTGGATAACCTCCCTTCTATAAGGCTGATCCAAATAATA

GAGTGTAGTTCTCTGCAGAATCCATTCAATGAAGGCTTCTTTAGTGCACCTGCTCTATCA

TTTTTATCTGGAGAAAATTCAGATTATGATGATATTAAGATTTATCTCGAATGTGATGAG

ATTCCAGAATGGTGCAGGAATCAAGTAACAGCTTCATATATGCGTTTGACTATGCCGACA

CATATTAATGATGAGTATAACTTCTTAGGAATGGTTCTCTGGTTTGTTACCGACTGTTTT

GATGAACCCTTTGGTATTCCAACCTTCTTGATTAGTATTGCCGGTAGAAAGACTTTAATT

TCTCCGTTTAGTAGACCTGATGATGGACATAGAGAAGTATCATGTGTATATTACATACCT

TACATACATAAAGCTTTTGATGGCGAGATGATCAAAGGCGGGGAAAGGATAGAAGTGTGG

TCTGAATACATTACAGCAAAGAAGATAGGGATCCATCTGTTATATTTAGACCAACATGGT

AAAGTTATATCTTTGCCGGGAGACGTGGACTGA

>NTK326_mRNA_88540_cds NTK326_mRNA_88540 gene_51913|id=AT5G17680.1

ATGGCTTCTACTTCTTCACCCACTCAAAATTGGAAGAATGATGTTTTCTTGAGTTTTAGA

GGTAAAGATACTCGTAAAACTTTTGTGGGTCATCTCTACTATGCTCTAAAACACAAAGGG

GTTCACACTTTCAAAGATGATGTAAGGTTAGAGAGAGGAAAGTCCATTTCACCTGAACTT

GTGAAAGCTATTGAACAATCAAGATTTGCTATTGTTGTATTTTCTAAGAACTATGCATCC

TCCACTTGGTGCTTGGATGAACTTGTAAAGATCATGAAATGCAAGAAAGAATTAGGACAA

ACTGTGATACCCATATTCTATGACGTAGATCCATCGGATGTGAGTAAGCAAAGTGGAACT

TTTGCTGAATCATTTGCTAGACATGAGGAAAATTTTAGAGATGATTTGGAGAAGGTGCAA

TCTTGGAGGGATGCATTTGGTGAGGCAGGCAAAACAGCAGGATATGATTTACCAAATGGC

TACGACGGGTATGAATCGAATTGCATCCAGCATGTTGTTGAAGACATACTGGGTAAATTG

TGTCAAGTTACTTCAACCATTGATAATGATTTAGTGGGGATGGAGTCTCGAGTGCGTGAA

GTAAGTTCATTACTAAGGATGGAAACACATGATGTTCGTTTTATTGGAATTTGGGGGATG

GGCGGCATTGGTAAGACAACAATTGCAAGCGCTGTGTTTGGCAAATATTCTGGCCTATTT

GAAGGTGTTTGTTTTCTTGATAATGTTGCAGAAATGCAAAGGACATATGGACTGCAATAT

TTGCAAGGTGTTCTCCTCTCAAAAATCCTAAAGTTAAGCTTAACTATTACAAGTGTATAT

GAAGGCATGGAAATCATAAAGAAGAGGTTGCGCTCAATGAAGGTTTTTATCATTCTTGAT

GATGTAAATCAAAAAGACCAATTAGAAATGTTAGTTGGACGGCATGATTGGTTTGGTAGT

GGTAGTAGAATTTTGATTACAACAAGAGATAAAAATTTGTTAGATAATCATATGGTGGAT

GAAGTGTATTCTGTGAACTTGATGACTCTTAATGAAGCTATTGAGCTATTTAACCTACAT

GCCTTTAAGCAAAGAATTCCTAAGAAAGACTTTGAGGAGCTTTCAAATCAAGTTGTACAT

TGTGCCGGTTTGCTCCCTTTAGCTCTGAAAGTTTTAGGTTCGTTTCTCTATGGATTAGAC

AGGAGGCATTGGAGATCAACTTGGAAAAGGCTGAAGGATCTGCCAAATGATGAAATTCTT

GCTAAGCTTAAGATAAGCTTTGAAGGACTGGGGCATGTTGATCAGAGACTCTTTCTAGAT

ATTGCATGCTTTTATAGAGGAAAATTGAGGAGTTATGTAGAGGAAATACTTGAGAGCTGT

GATATCGGATCTACAATAAGAATAAAAGTCTTAATTGAAAAGTCTCTTTTATTTATCTCA

CCATATGACACAATTGAAATGCATGATTTGATACAAGAAATGGCCTGGCACATCGTGAGT

CAAGATGACTCACGAAGGAGTAGAATATGGCTTCCTGAGGACATTGAGGATTTGTTTACT

GGAAATTTGGAAGCAGAATCTGTGGAGGGACTATGGATACCAAGGAATTACATTACAAAA

CAGGATATATCATATTACAACATCAGTGAAGCATTTAGGAGAATGAAAAGATTAAGGGTA

CTTGTAGTTAGAGCAACAGATTTCTGCTCTATTGACCCGATTACTCATCTTCCTAGCAGC

CTAAGGTGGCTTGATTGGGAAGGTTGCCCTTTAAATTCATTGCCACAGAGTTTTGAACCA

TCAAAGCTTCTTCGCCTTGATATACTCGAATGTACTACACTTCATAAACTCTGGTTAATT

CCGAAGGGTTTGGACAAACTAAAAACTTTGTACCTCAGCTATTGCGAACACTTGGAAGAA

GTTCCAAGCTTTGAGATGATGCCAAATTTAGAGAGAGTAAAGCTAGAGGGATGTAAGAGT

TTGAGAGAAGTGAGCCCATCCTTTGGAGTTCTCATGAAGCTCATTTCACTGGAGCTAATT

GATTGTCAGAGCCTTGAGAAGCTTCCAAGTTATATTCAGATGGAATCCCTTAAGAGTCTC

AAACTTTCTTGTCTTCCAAAGTTGAGGGAATTACCAGAAACCAAGGGGTTGCACCGTTTA

TTGACATTGGAGATAACTGATTGTCAGAGTCTTGAGATGCTTCCAAGTTGTAATCAGATG

GAATCTCTTGCAACTCTCAAACTTTCTTGTCTTCCAAAAATAATGGCCTTGCCGGCAACA

GAAGAGATGCACCATTTATTGGAACTTGTTATAGAATATACTCCAATAGTAGAGCTTCCG

GTGTCAATTGGAAATCTTGGTTCCCTCAAACAACTACGGTTAAGTCATTGTAAAGATCTA

GTAAGCATTCCGAACAGCTTTTCTTGTCTGAAGAATCTAAGAGTTCTTGTGATCTACAAC

TGCAAAAGACTTGCAGATTTGCCAGAGAAGATGGGTGAGTTGAAGCTGTTAGAAAAGCTA

GTATTATCTGGTACTGCAATTTCCCAAATACCTCCTTCAGTTGCAGACCTTCATGAACTA

AGCTTTTTATCATTCTCTCCCTGGTTTGGATACAGAGAAGATGCAACTTTTCTGTTACCC

TCTGCATCAGGTTCATCGTCATTTAGGGTGTTAAAGCTTAATAAGCACACACTATGTAGT

GGAGAACATTATCAGGATCTTGGATGCTTATCTTCTTTGGCTCACTTGGATTTGACTAGA

AATGATTTTACTAGTTTCAATGAAAGCAACAATCAGCACTTTCATTACCTAGATATAACA

TTTTGTGAGAAGCTTGTAATGCCCAGACTTCCATCATGCATAAAGGAGTTATATGCATAT

GATCCTTTAGTCTTGAAAAGCATCCCTGATTTCCCCACCAAATATTCAGAGCTGTATTCA

GTGTCATTCACACAGCATATTGAGAATAGAGGTGAACTGACTGATATCTTGCACTTTGTC

CTCCACTTAATTAGTGTGGCATCTCAGTCTGAGAAAAGGCTACCATTTAGCATTTTTTTC

CCTGGAGATATAAGATGGAGCGGGTTCAATTATTATCGAAAAGAGCATACGAAAAGATTC

TCCACTCCACTTGATCCAAGTTGGCATGAGAGTAAATTCAAAGGATTTGTTATATGCTTT

CGTGTACCATTGGATACTGTTCAGAACCAGAAACCTTTGGATTCTAAATCACGAAGAGGA

AGTCACTGGTTCGGTTGCACTAAGGTTACAGCTAAGTTAGTGCAAAGATATGACAGGCAA

GAACAAGATGTACTCCAGAAAAAATGTTTGATTGTTGCTCGCCAAGCAATTTGCTCTCAT

AGTAGTAAATATGCCATTTGCTTTAGCTACATACCTTTTCTAGCACTATGGCATACTTCT

GATAGTGAAAAGGGGAAGAAGCCAAATGATTATTGCTTCTTTGAGGCGTCTATAGACCCA

GGCATTGCAACAAAATGGGGACTTCTTCTGGTGTACGAGAATAAAATTAAACAGATAGAT

CAATCAACCATCTCGGTCCAACGTGATGTGGAGTCTCCAAGTTCTGACCTGTTGAGAGAA

TCTAATGATGACCAAGTCCAGAAAACGGAGGATGCTTCTGTTAAGAGAAGACGGGTTGAT

TTTTGTCAAAGAGATAATATGGTTTCATTTGAAGCTGGCTGCTCTATGAAATTTCAAGCG

ATGAAGGACTCATGCTCTCCCAGTGACTTTCAGACTCTCCAAATAATTCCTGATCAACAA

TTGGAAACACCATGCTCTTCTGCAGCTCAAAGCTTCCGACACAGGGAAGAGTCATGCTCT

TCTGGACAGCCACAAACTTTACAGCTCCCTCCAGCTGATCGACAAGTTGATGAAGCGATA

AATGAGGCTACCTCCTGCATGGTATTTGAACTGGAAGCGCCAAGCTCTTCGGAGCAGCCT

GAATCTTTTGAAGTCTCTCCAGATGAGCGCAAAGATAATTCAGTGACAAATGGGTCAAGC

AGCTCTGAGGTATTCCAAGAATTGGAGGCACCATGCTCTTCTGGACAACCTCAAATTCTC

CAGCTCTTTCCCTAG

>NTBX_mRNA_61043_cds NTBX_mRNA_61043 gene_35651|id=AT5G36930.1

ATGGCCACTGAACTGAAGTCTCAAGTGTACTTGAGTTTCAAAGCGAAAGACACCGGCAAA

ATTTTTGCAGATCACCTCTATGAAGCTCTGGTGGGAGCAGGTTTTGTAACATTAAGAAGC

TGTGGTGATGAAAATGAGGGAGGTGAAGATATCAAGTTCAATTTGCAAAAGGGTATTAAA

GAATCTGGGGTTTCAGTTATAATCTTCTCAAATGATTACGTGTCCTCAAGTTGGTGTCTT

GATGAGTTGGTAATGATCTTGGATTGTAAAAAGATAGCAAAACGTGCAGTTCTGCCCATA

TTTTACCACGTGGATCCTTCTGATGTTAGGAAACAGAAGGGAAGAATTGGAGAAGCATTT

GATATGGACAAAGAACTGGGAGGGAATCAAGGTGAAAATGAGAGGGTCAGAAAATGGAGG

GAAGCACTCAAAGAAGTTGCAGACTTGGGAGGAATGGTCTTACAAAACCAAGCTGATGGA

CACGAGTCCAAATTCATCCAGAAGATTCTTAAAGTGGTTGAGAATAAACTGAGCAGGCCA

GTCCTGTATATTTGCCCTCATCTGATTGGAATAGAACGGCGTGTTGAAAAGATCAACTTG

TGGCTAGAGGATGGATCTATTGATGTTGACACTCTTGTTATTTGTGGCATCGGTGGAATA

GGCAAGACAACAATGGCAAAGTTTGTGTATAATTTGAACTTCAGTAAGTTTGATGGTAGC

AGCTTTTTGTCCAACATTAGAGAAAATTCAACACACCGTAAAGGTTTAGTTACTCTTCAA

AGGCAATTTCTTTCTGATATTTGCAAAAGAAAGAAGAAAGCTATGTTTTCCGTGGACGAG

GGAATGACTGAGATGAGAGAGGCTGTACAGTGTAAAAGAATCCTTCTTGTTCTTGATGAT

GTAGATAATCGTGATCAAGTGGATGCTCTACTGGGAATGAAGGACTTGTTATATCCTGGT

AGTAAAGTCATTGTGACAACTAGGAACAAGAGATTGCTTAGGCCTTTTGATGTGCATAAG

ATTTATGAGTTTGAAGCATTGAATAGAGATGAATCGGTTGAGCTCTTAAGTTGGCATGCA

TTTGGTCAAGATTGTCCTATTAAAGGTTTTGAAATGTGTTCAGAACAAGTAGCAATCCAT

TGTGGAGGACTTCCATTAGCACTTGAAGTTCTTGGTGCTACTTTGGCAGGAAGAAACATA

GACATTTGGAAAAGTACAATACAGAAATTGGAAACAATTCCGAATCATCAAATTCTCAGG

AAATTAACAATAAGTTACGAATCTCTTGAGGATGATCATGATAAGAATTTATTTCTCCAC

CTAGCTTGCTTTTTCATTGGGAAGGACAGAGATCTAGCAGTAACTATTCTCAATAGGTGC

AACTTTTACACTGTAATTGGAATTGAGAATCTCATTGACAGAAATTTTATAAAAGTTGGT

AAGTCTAACAGCTTGATTATGCATCAAATGATTCGAGATATGGGAAGAGACATTGTTCGC

CAAGAATCACCACTGGAGCCTGGGAAACGCTCTAGACTATGGCGTTCAAAGGATTCCTTT

AACGTCTTAATCCAGAACCGTGCCACTCAAACAATTCAAGGCATTATTCTTGACATGGAT

ATGCTCAAGGAAAGTGACATAGTTAGCTCAAGCTTTTTCGCCAAGGATTTCAAGAAACAC

AAAATAAAAAACTTTCTCAACTATCCTAATCCTCAGAGAGTTCAATTCAAACAGAAAAGG

TTTGTTTTTTTCCCATGGCATTTGTCAGATGCCAAAGAAGCCACAAATGAGCTGGTTCTG

GGAACTGATGTATTTGCAAATATGCAAAAGTTAAAACTGCTCCAATTCGATCACGTTGAG

CTTCAAGGATCTTTTGATGTTTTTCCTAAGAGATTAAGATGGTTGCGCTGGTCTGAGCTG

CAACTTGAGTGCATGCCAATTGATTTTCCTCTGGAGAGCCTTGTAGTGATTGAATTACAC

CGTAGCAGCTTGAGGAGGATTTGGCATGGAGTCAAGTTCCTTAAAGATCTGAAGATTTTC

GATCTCAGCCATTCCTACGAGCTTCTAAGAACACCTGATTTTTCAGGACTCCCCAATCTT

GAAAAGTTGATCCTTCGATATTGTACAAGCTTGATTGAGCTTCATGAGACCATCGGGTGT

CTAGAATCACTTATTCTTTTGAATCTCAAAAATTGCAAAAATCTCCAGAGACTTCCAGAT

AGCATTTGCATGCTAAAATGTCTGGTGACACTAAATATCTCTGGTTGCTTGAATCTTGAA

TATGTGCCGATGGATCTAGATAAAATGGATTCACTGAGAGAGCTTTATGCTGATGAAATT

GCAGTTCACCAAATGATTTCTACTCCAGAAGAGGTCCAACCGTGGTATGGATTTCTGCGG

TCCTGGATGCTGAAGGGGAAAATATGTCCTAAAGTTTCACATATTAGTTTACCTAATTCC

TTGGTTACTCTGAGTCTTGCTAACTGTAATCTATCCAATGATGCTTTTCCAGTTGCTTTC

AGTAGCCTCTCCTTATTGCAAAACTTAGATTTGAGCGAAAATCCAATTTGCTGCCTACCA

AAGGGCATAATTTATCTCACCGGTCTTCAGAAGCTTGAAGTGGAAGGCTGTGAAAAGCTC

AGATCGCTCGTAGGGCTTCCCAATGTAGAACATCTCAATGTTACTAATTGCTGGTCGTTA

GAGAAAATATCATATCAATCAAGATCATCTAGACTGAAGGATTTACTTGTGTCGAATTGT

GCTAAATTAGTTGAAATAGATGGAAATTTCAAGTTAGAGCCCTTAAGAAATACTGAGGCA

GAGATGCTTTGCAAGTTGGGCTTGTCGAACTTAGCTTCTATGGATAATGTCATGATCAAT

CTTACATCTAATATCCTGAGTTACTACCGAATACATGGTAAAGGATGGACTCCAACAAGG

AAGACAAAGAAAGTTGTTCTTCAGGTACTGTACCAACCAGGTGTCTTTAGCACTTTTCTG

CCAGGTGAACATGTACCTTCTTGGTTCAGCTCAAAATACACAAAAGAATCACATACATCC

TTCAAAGTGCCTACTTGTACTTCCACGATTGAAGGCTTGAGTTTTTGCATTGTGTACAAG

CGTTCCGTATTTGGTCTAAGTGCTCATCGTCCCCCGCGCCTAACTCCGCCTTCAAGAATA

GCTCCTCTTGCCATGCACAAAGCTCAAAGAGGACCCATTCGGTATCGGCCAGTGGAAAAT

AAACCATATGAATCAACCTTTGACTGCCCGTGCATTACTGTTAATAACTTAACTCGGAGT

GTGAAATGGTCTTACCAGCCCTTGTTCTATGGAGTTCCGGAAGGGAAAGAAGGAATGATG

TGGTTAAGCCATTGGAAACTTGAGAATCAGTTGGGCAGTGATGATATACTGGAGATCACA

GTTACCTCAGGAGATGGAATCAGAATTGTGGAGTTTGGGCTCAAAATTCTGCATGTTGAA

GGGCCAAATGTGCAAATAGGAGAACCAAGTTGTGAAGATGCAAGGGGAGAGAAAGATATT

GTCAATCCATTTTGGGATGTTGTTTTAAAAGATGCTAGTTCAAAGAATACTTGTTCTGTT

CGGCTTCCTCCTACATATCGTCCCCTACGTGTTGCTCGTGAGCCATTTCTGGAGAAGGCG

CTGAAAAGAAATATGTCAGACTATAACTAG

>NTBX_mRNA_95736_cds NTBX_mRNA_95736 gene_57205|id=AT5G36930.2

ATGGATACTCAATTAGTTCGAGGAGAATCATCTCACTTCTCTTATGAAGTATTCCTCAGT

TTTAGAGGTGAAGACACCCGAAAAACATTCACTGGTCATCTTTATTCCAAATTGTGTGAT

GTTGGAGTTAATACCTTCATTGATGATGAGGAATTGAGAAAGGGTGATGTGATTTCAAGA

GAACTAGAGAAAGCAATTGAAGAGTCAAGAATTTCCATTATTGTTTTCTCAAGAAATTAT

GCTTCCTCTAGTTGGTGTCTAAATGAACTGGTTAAAATTCTTGAATGCAAAGAGAAACTA

AAGCAGATGGTTTTGCCTATTTTCTATGATGTTGATCCTTCCGAGATACGAAAGCAAACT

GGGTTATTTGGGGAAGCTTTGGCTAAACACAAGGAACGACCATTTGGAGCTCAAAGGGTG

GAGAAATGGAGAGCTGCACTTACTGAAGCTGCAAATTTATCTGGATGGGATTTGCAAAAT

GTTGCTGAAGGGCATGAATCAAAGTTTATTGAAAAAATTATACAGCAAGTTCTACAAGAG

GTCAACCAGACACCTCTTGATGTTGCTTGGCACCCAGTTGGAGTAGATTCTCGTGTTAAA

GATATAGAGTTGTTATTGCAAAATGAATATGAAGATGAAGTTCGAATTATTGGTATTCAC

GGAGTTGGTGGCATAGGGAAAACAACTCTGGCAAAAGCTATCTACAATCGAATGTTTCGA

CTCTTCGATAGTAGTTGCTTCCTTTCAGATGTTAGATCAGAAGTTCAAGAATTTGGTCTT

GTCAAGCTACAAGAGAAACTTCTTCAACAAGTTCTCAAAACCGAGGACATCAAAGTTGGG

AGTGTTGCTCAAGGCGTTAATCTAATCAAAGCAAGACTTGGGTCAAAGAAGGTTCTAATT

GTTCTTGATGATGTGGATCATAAAAGACAGTTAGAAGCCTTAACAAGAGAAAGAAGTTGG

TTTGGTTCAGGTAGTTTAATAATCATTACCACCCGAGACGAGCGATTGCTATGTCGGCTT

GGAGAAAAAGAGAGATATGAGGCCAAACTATTAAATGGCACTGAAGCTATGTTACTTTTT

TGTTGGCATGCTTTTGACAGTCATTTTCCACCAAAAGATTATGTTAATTTGGCACACGAC

ATAATCGAATATTCAGATAGGCTGCCATTAGCTCTTGTGACATTGGGGTCACATTTACAA

GGAAGTTCTGTAGAAGAATGGGGATTTGAATTAGAAAAACTAAGAGCAATTCCTCATTGT

GATATCCAAAAGATTCTCAAGATAAGCTTTGATGGGCTTGATGGTGAAACACAGATTGTT

TTCCTCGATATTGCATGTGCCTTCCATGGGTTTGATGAGCATGAAGTTACTGAAATATTA

AATGCATGTGGCTTTCATGCTAAAATTGCAATTGCAACTTTAGTCCAAAAACACTTGCTC

CAAAGAACTCCGTATCATTTGGTGATGCATGATCTAGTGCGAGATATGGGAAGAGAAATC

GTTCGCTTGGAATCACCTCGAGACCCCGGAAAACGAAGTAGATTGTTCATCCCTCGAGAA

GTTCGTGATGTTCTACAAGGAAATGAAGGTTCCGAAAATGTTGAAGTACTGAAGTTAGAT

CGAGGGACATTAAACGGAGTGAACTTGAGCACCAAAGCATTTGAACAAATGAAAAACCTT

AGGGTTCTTATAATGGATGAGTTACATATTAGTGGAGATTTTGGGTTGTTGTCCAAGAAG

CTCAGATGGTTGTCTTGGAAAAAATGTCCTTTAAAATGTATACCATCAAATTTTCCAGCT

GAGAATCTTGTAGTTCTAGATATGCGGGAGAGTGATATCCAAGAATTTCAATTGAATTTG

CAGTGTTGTAAAAGTTTGAAGGAGCTGAATCTCTCTCATTGCAAGCAACTCAGAAGCACT

CCAAACTTCAATGGTTTTCTGAGTCTTGAGAATTTGCATCTCTATGGTTGCTCAAGTCTG

ATAGAGATCCATCCATCAATCGGAAATTTGTCCAGACTATGCGAGCTATCTATGTCTGGT

TGCAATAGACTTACGGATCTTCCAAACAGCATATGCCAGCTAATATCCGTTAATTACTTG

AGCATTAGTAACTGCTCATCAATAAAAACACTGCCTGATAACCTTGGAGATATGAAAAGT

CTAAGATCTCTCGATGCATATGATACGGGTATAAAACAATTGCCTAGATCCATTGAAATG

CTAAGAAATCTTAAAATATTGGAAGTGGGAGGTCGAAAGGTAGAGACCGAAAGGAGTATT

TCTGGAAGAGGAGTCCATCGGATACAATATTCCTTGCCAACTTTTGTATCCAATTTGAGC

CTTACATACTGTAATTTGTCCGAGGCTGATATTCCTAGGGATATTGGGAGCTTATCCTCC

TTAGAATATTTAGATTTGAGTGGCAACAGTTTCCGTTGTCTACCCATTGATTTTTCTAAG

TTACGATTGTTGGAGAAGTTAAGTTTGAAGGACTGTGAGAATCTCCAAACACTCCCGTCA

GTATCAAATTTAGAGAATCTTTATGCAATTAAACTTAATAATTGCCAAAAATTGGTCAAG

ATTACAGAGTTGGACAACCTCCCTTGTATAGAGCGGATTAATATGATTAATTGTAGTTCT

CTGCAGAATCCATTCAATGAAGGCTTCTTTAGTGCACCTGCTCTATTTGCATTTAGTGCA

CCTGCTCGATATATGATGCAGGATACTATTAGAATTTATCTCGAATGTAATGAGATTCCA

GAATGGTGCAGGAATCAAGTAACAGCTTCATCTATGTGTTTGACTATGCCAACACATAAT

AACTTCTTAGGAATGGTTCTCTGGTTTGTTTCCGACTTGTTCGATGTAGTCCCGCATAAT

CCATGCTTCAGGATTAGTATTGCACATGGAAAGCCTTCAAATATTCGGTGGAGAAAAGGG

AAAAAACCTNGAGTTTATCTCGAATGTAATGAGATTCCAGACTGGTGCAAGAATCAAGTA

ACAGCTTCATCTATGTGTTTGACTATGCCTACACATAATAACTTCTTAGGAATGGTCCTC

TGGTTTGTTACCGACTTGTTCGATGTAGTCCCGCAAAAACCATGCTTCAGGATTAGTATT

GCTCATGGAAAGCCTTCAAATATTCGGTGGAGAAGATGGAAAAAACCTCCGTCGATGTGT

GTATATTACATATCTTACTTACATAAAGCTTTTGATGGCCAAATGATCAAAGGCGGGGAA

AGGATAGAAGTGTGGTCAGAAGACATCGGGGAAAGGATAGAAGTGGGGTCAGAAGACATT

ACAATAAAGAAGATAGGGATCCATCTGTTATATTTAGACCAACATGGTAATGTTATATCT

TTGCCTGGAGACGTGGATCATTCTTATACTAGGGCGAAAAATGTCAGGAATTGA

>NTBX_mRNA_79430_cds NTBX_mRNA_79430 gene_46583|id=AT5G36930.1

ATGGAGCAAGGTATACCTTCGTCGCCTCCTACTTCTCAATGTTCATATCATGTGTTCTTG

AGTTTCAGAGCAAAAGACACTGGCAAGACTTTCACTGATCATCTCCACAGAAATTTGGTG

CGAGCTGGGTTTCATGTATTCAAATGTGATAACGATGATTATGACGGTGAAAAAGAGGAC

TTGAAGTCAAAATTGCAAAAGGGAATAGAGCAATCGAAGATGTCAGTGATTGTATTGTCC

CAAAATTATGCATCATCTGAAAAGTGTCTTGATGAGCTGGTCGTGATTTTGGAGCAAAAG

AGGAATTTTGGGCACATAGTTTTGCCTGTCTTTTTTAACGTGGATCCTTCTGATGTTAGG

AAGCTAAAGGGAAGTTTTGGTCAGCCTTTTTCTGCAAATGGAGAGAGTCAAAAGTTAAGA

GATTGGAGAAATGCTCTCAAACAAGTTGCAGATTTGGGAGGGATGCCCTTGCAAAATCAA

GCTGATGGATATGAGGCAAAGTTCATTGAGAATATAGTTGAAGTAATTGCAAGCAAGCTA

CGTCCCAGAGCCTTAAACAATGCTCCTTACCTCATCGAAATCAGTTATCGGGCTGAAGAT

ATTATCTTGTGGCTACAAGATAGATCAACTAATGTAGGGCTATATGTGATCTGCGGGATT

GGTGGAATTGGGAAGACAACCCTTGCCAAATTTGCCTATAACTCAAGTGCAAGATCATTT

GAAGGAAGCAGCTTTCTTGCAAACATCAATGAAACTGCAAAACAATGTAATGGTCTAGTT

TGTCTGCAGAAGCAAGTTTTGTATGATATAGTTGGAAAAAAAGAGAGGATATCCAATGCT

GATGAAGGAATTATGATGATTGAAGATGCCCTACGCTATGAACAAATTCTTCTTGTTCTT

GATGATGTTGATGAAGTTGACCAGATAGATAAAATTTTAGGAATGAGAGATTGGCTTAAT

CCTGCTAGTAAAATCATTATAACAACGAGGCATGAGTCCTTGCTAAAGCCTTTTGTACCT

CACAAGGTGCTTAAAGTAGAAGCCTTGAATAAAATGGATTCCCTAAAGCTGTTCAGCTGG

CATGCCTTCGGAGAAGACCATCCTTTGGAAGGTTATGTCGAGCTCTCAAAACGGGTGGTT

CTTCAATGTGCAGGACTTCCTTTAGCTCTTCGTGTTTTAGGTTCAGCTCTGTCGGGTAGA

AGACCAGAAATATGGGGAAGTGCATTAGAAAAGCTGGAAACAATCCCTGATGGTCATGTC

ATTGAAAAACTTAAAGTGAGTTTTTACTCTCTAGAAGATGACCATGACAAAGATATATTC

CTTCATATAGCTCTTTTCTTTCTTGGGATGGACAGAGACGACTCTGTCAGAATACTGAAT

GGATGTGGTTTTTACACAATAATTGGGATGCAAAACCTCATTGATAGAAGTCTTTTGACA

ATCAATGACTTAAATAAGTTGGAGATGCATCAGTTGCTTCGAGACCTTGGGAGAGATATC

GATCGTAGGGAATCACAGGATCCTGGAAAACGCAGTAGACTTTGGAATAACAAGGATTCT

TTCAGAGTGTTGAACGATAAAACTGGCACTGAAAGGATTGAAGGGATCAGCTTTGACATG

CCTATGTTGATGGAGGATAAATCAGCCAAACAATTTTTCACTGGAAATAGCTCGAAAAGG

CGCTTCCGAAAAGATCATGTAGAGAACTGTGCAGATCATAATCTTTCGCTGCAACAGCCT

TCTTCTGTCTTTTATTCATGGAATTCAGGAGACACTTCATCACGAAACTCAAACTACAGC

ATAGAAACTGATGCATTTACGATAATGCGAAACCTAAGGGTACTCAAACTAAATGATGTA

AACCTCATTGGATGCTATAAAGAATTTCCCAAGAGATTAAAATTGTTGTCTTGGCGTAAA

TGCCCTTTAAAATCCTTACCTAGTGACCTTTCCTGGGAAAACCTTGTTTCTATTGACATG

CGGTACAGCAATTTACAACAAACATGGAGTGAAACTGAGTATTTCAGATTTCTCAGGATT

CTCAACCTTAGTCATTCTTGGGAACTTACCAGAACACCTAGCTTCGCTGGAATGGCCCGA

CTTGAGAAACTAATTCTTAAAGATTGTATTAAACTGGTTGATATCGATGAAACTATCGGC

TGCCTCCAAGAAATCACCCTGCTAAATCTGAAAGACTGCAAGAGCATCAGAAAGCTGCCA

AGAAATATCGGTGAACTTATAACTCTTAAGATACTTGATATATCCTTCTGCTCAAGCCTG

GAGTGGCTGCCAATGGAGCTTAACATGATAGATTCTTTGAAAGTTCTAAGAGCTGATGGA

ATTGATCTAAACCAAATACTCTGTACCACCCACGAGCGGAAATCATTGCAGGCATTATTT

TCATCTTGGGTATCAAAGCCAAGAAACACTCCTGAAATATCGTGGGCCTTTTTACCAAGC

TCTTTGGTGAGCTTGAGTCTTGTGAGTTGCAGGCTGTCTGATGAATATATTCCCCAGAAG

TTTATCAATCTTCCGCTGCTCCAAGAACTGGATCTAAGTGAAAATTCAATTAGCTGCCTC

CCGGAGTGGGTCAAGAGTCTGCCTCAGCTCCAAAGCCTCAGTGTCAAGTCATGGGAAATT

TAA

>NTTN90_mRNA_21896_cds mRNA_21896 gene_12276|id=AT5G36930.1:evalue=5e-168:annot='Disease resistance protein (TIR-NBS-LRR class) family';id=Solyc01g113620.1.1:evalue=0.0:annot='NBS-LRR resistance protein (Fragment)'

ATGGCCACTGAACTGAAGTCTCAAGTGTTCTTGAGTTTCAAAGCGAAAGACACCGGCAAT

AATTTTGCAGATCATCTCTATGAAGCTCTGGTGGGAGCAGGTTTTGTAACATTAAGAAGC

TGTGGTGATGAAAATGAGGGAGGTGAAGATATCAAGTTAAATTTGCAAAAGGATATTAAA

GAGTCAGGGGTTTCAGTTATTGTCTTCTCAAATGATTATGTGTGTTCAAGTTGGTGTCTT

GATGAGTTGGTAATGATCTTGGATTGTAAAAGGATAGCAAGACGTGCAGTTCTGCCCATA

TTTTACCACGTGGATCCTTCTGATGTTAGGAAACAGATGGGGAAAATTGGAGAAGCATTT

GATAGGCATGAAAATCTGGGAGGGAATCAAAGTGGAAATGAGAGGGTCAGAAAATGGAGG

GAAGCACTCAAAGAAGTTGCAGACTTGGGTGGAATGGTCTTACAAAACCAAGCTGATGGA

CACGAGTCCAAATTCATCCAGAAGATTCTTAAAGTGGTTGAGAATAAACTGAGCAGGCCA

GTCCTGTATATTTGCCCTCATCTGATTGGAATAGAACGGCGTGTTGAAAAGATTAACACA

TGGCTAGAGGATGGATCTACTGAGGTTGACACTCTTGTTATTTGTGGTATCGGTGGAATA

GGCAAGACAACAATGGCAAAGTATGTGTATAATTTAAACTTCAGTAAGTTTGATGGTAGC

AGCTTTTTGTCCAACATTAGAGAAAATTCAACACACCGTAAAGGTTTAGTTACTCTTCAA

AGGCAATTTCTTTCTGATATCTGCAAAAGAAAGAAGAAAGCTATGTTTTCTGTGGATGAG

GGAATGACTGAGATGAGAGAGGCTGTCCGGTGTAAAAGAATCCTTCTTGTTCTTGATGAT

GTAGATAACCGTGATCAATTAGATGTTCTACTGGGAATGAAGGACTGGTTCTACCCTGGA

AGTAAAGTCATTGTGACAACTAGGAACAAGAGATTGCTTAGGCCTTTTGATGTGCATAAG

ATTTATGAGTTTGAAGCTTTGAACAGAGATGAATCGGTTGAGCTCTTAAGTTGGCATGCA

TTTGGTCAAGATTGTCCTATTAAAGGTTTTGAAATGTGTTCAGAACAAGTAGCAATCCAT

TGTGGAGGACTTCCATTAGCACTTGAAGTTCTTGGTGCTACTTTGGCAGGAAGAAACATA

GACATTTGGAAAAGTACAATACAGAAATTGGAAACAATTCCGAATCATCAAATTCTCAGG

AAATTAACAATAAGTTACGAATCTCTTGAGGATGATCATGATAAGAATTTATTTCTCCAC

CTAGCTTGCTTTTTCATTGGGAAGGACAGAGATCTAGCAGTAGCTATTCTCAATAGGTGC

AACTTTTACACTGTAATTGGAATTGAGAATCTCATTGACAGAAATTTTATAAAAGTTGGT

AAGTCTAACATGTTGATTATGCATCAAATGATTCGAGATATGGGAAGAGACATTGTTCGC

CAAGAATCACCAGTGGATCCTGGGAAACGCTCTAGACTATGGCGTTCAAAGGATTCCTTT

AATGTGTTAATCCAGAACCGTGCCACTCAAACAATTCAAGGCATTATCCTTGACATGGAT

ATGCTCAAGGAAAGTGACATAGTTAGCTCAAGCTTTTCCGCCAATGATTTCAGGAAACAC

AAAACAAAAAACTTTCTCAACTATTCTAATCCTCAGAGAGTTCAATTCAAACAGAAAAGG

TTTGGTTTTTTTCCATGGCATTTGTCAGACACCAAAGAAGCCACAAATGAGCTGGTTCTA

GAAACTGATGTATTTGCAAATATGCAAAAGTTAAGACTGCTTCAATTCGATCACGTTGAG

CTTCAAGGATCTTTTGATGTTTTTCCTAAGAGATTAAGATGGTTGCGCTGGTCTGAGCTG

CAACTTGAATGCATGCCAATTGATTTTCCTCTGGAGAGTCTTGTAGTGATTGAATTACAC

CGCAGCAGCTTGAGGAAGATTTGGCATGGAGTCAAGTTCCTTAAATATCTGAAGATTTTC

GATCTCAGCCATTCCCACGAGCTTCTAAGAACACCTGATTTTTCAGGACTCCCCAATCTT

GAAAAGTTGATCCTTCGATATTGTACAAGCTTGATTGAGCTTCATGACACCATCGGATGT

CTAGAATCACTTATTCTTTTGAATCTCAAAAATTGCAAAAATCTGCAGAAACTTCCAGAT

AGCATTTGCATGCTAAAATGTCTGGTGACACTAAATATCTCTGGTTGCTCGAATCTTGAG

TATGTGCCGATGAATCTAGATAAAATGGATTCTCTGAGAGAGCTTTATGCTGATGAAATT

GCAGTTCACCAAATGATTTCTACTCCAGAAGAGGTCCAACAGTGGTATGGATTTCTGTGG

TCCTGGATGCTGAAAGGGAAAATTTGTCCTAAAGTTTCACATATTAGTTTACCTAATTCC

TTGGTTACTCTGAGTCTTGCTAACTGTAATCTATCCGATGATACTTTTCCAGTTGCTTTC

AGTAGCCTCTCCTTATTGCAAAACTTAGATTTGAGCGAAAATCCAATTTGCAGTCTACCA

AAGGGCATAATTTATCTCACCGGACTTCAGAAGCTAGAAGTGGAAGGCTGTGAAAAGCTC

AGATCGCTCATAGGGCTTCCCAATGTAGAACATCTCAATGTTACTAATTGCTGGTTGTTA

GAGAAAATATCATATAAATCAAGATCATCTAGACTGAAGGATTTGCTTGTGTCAAATTGT

GCTAAATTAGTTGAAATAGATGGAAATTTCAAGTTAGAACCCTTAAGAAATACTGAGGCC

CTTTGCAAGTTGGGCTTATCGAACTTGGCTCCTATGGATAATGTCATGATCAACCTTACA

TCTAATATCCTGAGTTACTACCGAATACATGGTAAAGGATGGACTCCAACAAGGAAGACA

AAGAAAGTTGTTCTTCAGGTACTATACCAACCAGGTGTCTTTAGCACTTTTCTGCCAGGT

GAACATGTACCTTCTTGGTTCAGCTCAAAATATACAAAAGAATCACGTACATCTCTCAGA

GTGCCTACTTGTAATTCCAGAATTGAAGGCTTGAGTTTTTGCATTGTGTACAAGCGTTCT

GCATTTGGTCTAAGTGCTCACCGTCCCCCACGCTTGGCTCCGCCTTCAAGAATAGCTCCT

CTTGCTATGCGCAAAGCTCAAAGAGGACCCATTCGGTATCGGCCAGTGGAAAATAAACCA

TATGAATCAACTTTTGACTGCCCGTGCATTACAGTTAATAACTTAACTCGGAGTTTGAAA

TGGTCTTACCAGCCCTTGTTCTATGGAGTTCCGGAAGGGAAAGAAGGAATGATGTGGTTA

AGCCATTGGAAACTTGAGAATCAGTTGAGCAGTGATGATATTCTGGAGATCACAGTTACC

TCAGGAGATGGAATCACAACTGTAGAGTTTGGGCTCAAAATTATGCATGTTGAAGGGCCA

AAACTGCAAATAGTAGAACCAAGTTGTGAAGATGCAAGGGCTGAGAAAGACATTGTCAAT

CCATTTTGGGATGTTGTTTTGGAAGATGCTAGTTCAAAGAATACTTGTTCTATTCGGCTT

CCTCCTACTTATCGTCCCCTATGTGTTGCTCGTGAGCCATTTCTGGAGAAGGCGCTCAAA

AGAAATATGTCAGAATATAACTAA

>NTTN90_mRNA_122583_cds mRNA_122583 gene_69240|id=AT5G36930.2:evalue=1e-179:annot='Disease resistance protein (TIR-NBS-LRR class) family';id=Solyc09g092410.2.1:evalue=0.0:annot='Tir-nbs-lrr, resistance protein'

ATGGATACTCAATTAGTTCGAGGAGAATCGTCTACATCATCTCACTTCTCTTATGAAGTA

TTCTTGAGTTTTAGAGGTGAAGACACCCGAAAAACATTCATTGGTCATCTTTATTCCAAA

TTGCGTGATGTTGGAATTAATACTTTCATTGACGATGAGGAATTAAGAAAAGGTGACGTG

ATTTCAAGCAAATTAGAGAAAGCAATTGAAGAGTCGAGAATTTCCATTATTGTTTTCTCA

AGAAATTATGCTTCCTCTAGTTGGTGTCTAAATGAACTAGTCAAAATTCTTGAATGCAAA

GAGAAACTAAAACAGATGGTTTTGCCTATTTTCTATGATGTTGATCCTTCTGAGGTACGA

AAGCAAATTGTGTTATTTGGTGATGCTTTGGCTAAACACAAGGAACGACCATTTGGAGCT

CAAAGGGTTGAGAAATGGAGAGCTGCTCTTACTCAAGCTGCAAATTTATCTGGATGGGAT

TTGCAAAATGTTACTGACGGGCATGAATCAAAGTTTATTGAAAAAATTATACAACAAGTC

CTACAAGAGGTCAACCAGACACCACTAGATGTTGCTTGGCACCCCGTTGGTGTAGATTAT

CGTGTCAAAGATATAGAGTTGTTATTGCAAAATGAATGTGAAGATGATGTTCGCATGATT

GGTATTCACGGAGTTGGTGGCATAGGGAAAACAACTCTGGCAAAAGCTATCTATAATCGA

ATGTTTCGACTCTTCGATAGTAGTTACTTCCTTTCAGATGTTAGGTCAGAAGCTGAAGAA

TTTGGTCTTGTCAAGCTACAAGAGAAACTTCTGCGACAAACTCTCAAAACTGAGGGCATC

AAAGTTGGTAGTGTTGCTCAAGGCATCAATCTAATCAAAGCAAGACTCAGGGCAAAGAAG

GTTCTAATTGTTCTTGATGATGTGGACCATAAAAGACAGTTAGAAGCCTTAACAAGAGAA

AGAAGTTGGTTTGGTTTGGGTAGTTTAATAATCATTACCACCCGAGACGAGCGATTGCTA

TGTCGGCTTGGAGAAAAAGAGAGATATGAGGCCAAACTATTAAATGGCAATGAAGCTATG

TTACTTTTTTGTTGGCATGCTTTTGATAGTCATTTTCCGCCACAAGATTATGTTAATTTG

GCACATGACATAATCGATTATTCAGGTAGGCTACCATTAGCTCTTGTGACATTGGGTTCA

CATTTACAAGGAAGTTCAATAGAGGAATGGGGATATGAAGTTGAAAAACTAAGAGCAATT

CCTCATAGTGATATTCAAAAGATTCTCAAGATAAGCTTTGATGGACTTGATGATGAAACA

CAAACTGTTTTCCTTGATATTGCGTGCACCTTCCATGGGTTTCTTGAGTATGAAGTTACT

AAAATATTAAATGCATGTGGCTTTCATGCTAAATATACAATTGCAACTTTAGTCCAAAAA

CACTTGCTCCAAAGATCTCCATATTATTTACAGATGCATGATCTAGTGCGAGATATGGGA

AGAGAAATTGTTCGCACGGAATCAGCTCGAGACCCTGGAAAACGGAGTAGATTGTTCATC

CCTCAAGAAGTCTGTGATGTTCTACAAGGAAATAAAGGTTCCAAAAATGTTGAAGCACTG

AGGGTAGATCCAGGGACATTAAAGGGAGTGAACTTGAGCACCAAAGCATTTGAGAAAATG

AAGAATCTTAGGGTGCTTATAATCAATGAGTTACATATTAGTGGCGATTTTGGGTTGTTG

TCCAAGAATCTCAGATGGTTGTCTTGGAAAAAATGTCCTTTAAAGTGTATACCGTCAAAT

TTTCCAGCTGAGAATCTTGTAGTTCTAGATATGCGGGAGAGTGATATCCAAGAATTTCAA

TTGAATTTGCAGTGTTGTAGAAGTTTGAAGGAGTTGAATCTCTCTCGTTGCAAGCAACTC

AGAAGCACTCCAAACTTCAATGGTTCACTGAGTCTTGAGACTTTGTATCTCTATGGTTGC

TCAAGTTTGGCGGAGATCCATCCATCAATAGGAAATTTGTCCAGACTAATTAAACTATAT

ATGTCTGATTGCAAAAAACTTACGGATCTTCCAAGCAGCATATGCCAGCTAATATCCGTT

GATTACTTGGACATTGATGGCTGCTCATCTATAAAGACATTGCCAGATAACCTTGGAGAT

ATGAGAAGTCTAAGACATCTTTATGCATCTTACACGGGTATAAAACAATTGCCTAGATCT

GTTGAAATGCTAAGAAATCTTGTAACTTTGAGAGTGGAAGGTGAAAAGTTAGAGGCCAAA

TGGAGTATTTCTGGAAGAGGAGTCCATCGGATACAATATTCCTTGTCAACTTTTGTATCC

TTTTTGAGCCTTACATACTGTAATTTGTCCGAGGCTGATATTCCTAGGGATATTGGGAGC

TTATTCTCCTTAGAATATTTAGATTTGAGTGGCAACAATTTCTATTGTCTACCCATTGAT

TTTTCTAAGTTACGATTATTGGTGGAGTTGTATTTGAATGACTGTGAGAATCTTCAAACA

CTCCCGTCAGTATCAAATTTAGAGAATCTTAGGATACTTCAGATTAAGAATTGCCAAAAA

TTGGTCAAGATTACAGAGTTGGACAACCTCCCTTCTATAGAGCGGATTATCATGATGAAT

TGTAGTTCTCTGCAGAATCCATTCAATGAAGGCTTCTTTAGTGCACCTGCTCTATATGCA

TCTAGAAAAGAAGATCCAGATATGGATGGATTAGATATTTATTTCCAATGCAATGAGATT

CCAAAATGGTGCAGGAATCAAATAACAGCTTCATCTATGTGTTTGACTGTGCCGACACAT

AATAATAATGAGTATAACTTCTTAGGAATGGTTCTCTGGTTTGTTTCTGACATGTTCGAT

GTATTCCGGAATAATCCATGCTTCAGGATTAGTATTGCCCATAAAGAGACTTTTATTACG

ACTTCGACTGATGATATTGATGTAACTGATGATATTGATATAACTGATGGACACAATGAA

GTGTCATGTGTATATTACAGATCTGACTTGGATGAACTTTTTGATGGCCAGATGATAGAA

GTGTGGTCGGACAACGTTACTGTAAAGAAGATAGGGATTGATCTGTTATATGTAGGCCAA

AATGGTAAAGTTATATCTTTGCCAGGAGACGTGGATCATTCTTATTCTAAGTACCCAAAA

AGAGTTTCAACAGGCTTGTCAACTCCTCCTTATAAAAGATTATAA

>NTK326_mRNA_43111_cds NTK326_mRNA_43111 gene_24938|id=AT5G17680.1

ATGGCATCATCTTCTACTTTTGCGAGTACTTCACAGTTTCCTCGATGGAACTATGATGTC

TTTCTAAGCTTTAGAGGTGAAGATACTCGGAAAACATTTACGAGTCACCTGTACGAAATC

TTGGATATCAGGGGAATAAAAACCTTTCAAGATGATAAAAGGCTAGAGCATGGCGCATCC

ATTTCGGATGAACTCTGTAAAGCTATCGAAGAGTCTCAATGTGCTGTCATCATTTTCTCA

AAGAATTATGCAACATCGAGGTGGTGCTTGAATGAACTAGTGAAGATCATGAATTGCAAG

ACTCAATTTGGACAAACTGTAATACCAGTCTTCTATGATGTGGATCCATCATATGTTCGG

AACCAGAGGGAGAGCTTTGCTGAAGCATTTGCCAAACATGAAACAAAGTATATGGATGAT

GTCGAAGGAATACAAAGTTGGAGGATTGCTTTAACTGCAGCGGCCAATCTCAAAGGCTGT

GATATTCGTGACAAGACTGAATCAGACTGTATTCGACAGATTGTTGACCGAATCTCGTCC

AAATTATGCAAGATTTCTTTATCTTATTTGCAAAACATTGTTGGAATAGATACTCATTTA

GAGAAAATAGAATCCTTACTAGGGATAGGAATCAATGATGTTCGGATTGTGGGGATTTGG

GGCATGGGGGGAGTCGGTAAAACGACAATAGCTAGAGCTATGTTTGATACTCTCTTAGTA

AAAAGGGATAGTTCTTATCAATTTGATGGTGCTTGTTTCCTTGCGAATATTAAAGAAAAC

AAACGTGGAATGCATTCTCTGCAAAATATCCTTCTCTCTGAACTTTTAAAGGAAAAAGCT

AATAACAATAGTGAGGAGGACGGAAAGCACCAAATGGCTAGTAGGTTTCGTTCTAAGAAG

GTCCTAATTGTGCTTGATGACATAGATGATAAAGATCATTATTTGGAGTATTTAGCAGGT

CATCTTGATTGGTTTGGTAATGGCAGTAGAATTATTGTAACAACTAGAGACAAGCATTTG

ATAGGGAAGAATGATGTAATATATGAAGTGACTGCACTACCTGACCATGAATCCATTCAA

TTGTTCTATCAGCATGCTTTCAAAAAAGAGGTTCCAAATGAGCATTTTAAGGAGCTTTCA

TTGGAGGTAGTAAATTATGCTAAAGGCCTTCCTTTAGCCCTCAGAGTGTGGGGTTCTTTG

CTACATAACCTAGGCCTAACTGAATGGAAAAGTGCTATAGAGCACATGAAAAATTACTCT

AATTCTGGAATTGTTGATAAGCTCAAAATTAGTTATGATGGATTAGAGCCCAAACAACAA

GAGATGTTTCTAGATATAGCATGCTTCTTCCGAGGGGCAAAAAAAGAGTATGCCATGCAA

ATTCTTGAGAGTTGTCATTGTGCAGCTGAATACGGATTGCGTGTCTTAATTGACAAATCT

CTTGTGTCCATCATTGAAAATGATCGTATTCAAATGCATGACTTAATGCAAGATATGGGT

AAATATATAGTGAACTTGCAAAAGGATTCGGGGGAATGCAGCAGGCTATGGCTCGACGAG

GATTTTGAAGAAGTGATGATCAACAATAAGGGGACCACGAAAATGGAAGCAATCTGGTTT

CCTTATTACCATTGTGGTACATTACGCTTTAGCAAAGAGGCCATGAAAAATATGAAAAAG

CTTAGGATATTAAACATAGAGAGGTCGTGTACCTGTGATGGTTCTATTGAGTATCTGCCC

AACAACTTGCGTTGGTTTGTATGGAAATGCTATCCTTGGGAGTCATTGCCAGCTGAATTT

GAACCCAAAAAGCTTGTTCATCTTGCAGTCAAATCCAGTTCACTATGTTATTTATGGACG

GGAACAAAGCAATTGTCGTCTCTACGGACGCTAGATCTCAGATACTCTGAAAGCCTGGTG

CGAACGCCAGATTTCACGGGGATGCCAAATTTGGAGTATTTGAATCTGGAGGAATGTTGT

GATCTTGAAGAGGTTCACCATTCCTTGGGATTTTGCAGAAAACTCATTCGGTTAAATTTG

GAGTCTTGTGGACGCCTTAATTGGTTTCCATGTGTTAACGTGGAATCTCTTGAATATCTG

GATCTAGATTTTTGCTGTAGTTTAGAGAAATTTCCAGAAATCCATGGGAGAATGAAGCCG

GAGATACAGATTCACATGAAACGCTCTGGGATAAGGGAACTACCATCATCTATTATTCAG

TACCAAACTCATATTACCTTCCTAGATTTGAGCGCTATGAAAAACCTTGTAGCTCTTCCA

AGCAGCATCTGTAGGTTGAAAAGTTTGGTTAGTCTAAATGTGTCGGGCTGCACAAAACTT

GAAAGCTTGCCAGAAGAGATAGGGGATTTAGAAAACTTGGAGGAGCTTTATGCAAGGTAT

GCTCTAATTTCACGACCTCCGTCTACCATCGTACGCTTGAACAAACTTAAAATCCTGAAG

TTTGGAGGCCTCCAAGATGGAGTGCACTTTGAGTTCCCTCCAGCGGCTGAAGGATTACGG

TCATTGGAACATCTGGATCTCACTTGTTGCAATCTAATAGATGGAGGACTTCCGGAAGAC

ATTGGATGCCTATCTTCTTTGAAAGAATTGTATCTCAGTGGAAATAATTTTGAGCATTTG

CCTCGAAGTATAGCCCAACTTGGTGCTCTTCGAATCTTGAACTTAAGAAATTGCAAGAGG

CTTACACAGCTGCCAGAACTTCCACCAGAATCAGATACAATATATGCAGATTGGAGCAAT

GTTTTGATCTGTAATTCGTTGTTTCAGAATATCTCGTCAGTGCAGCATGACATCTCTGCT

TCAGATTCCTTGTCACTAAGAGTATTTACCAGTGTGCATCTTGGGAAGAAGCCAAGTTGG

TTCCTCTATCAGGGAACAGATAGTGGTGTATCAGTCAATACGGATAGTGGTGTGTCAGTC

AATTTGCCTGGAAATTGGTATATACCTGATAAATTCTTGGGATTTGCTGTATATTACTCT

GGCAGCTTAATTGGCACCACAGCTCAATTGATTCCCGTATGTGATGATGGGATGTTGTGG

ATGACCCAGAAACTTGCCTTATCCAACCATTCAGAATGTGATACAAAATATACTATTAAT

TTTTTCTTGGTACCTCTTGCTGGCTTATGGGATACATCTAAGGCAAATGGAAAAACACCA

AATGACTATGGGCTTATTAGGCTATCTTTTTCTGGAGAAGTGAAGAAGTGTGGACTTCGT

TTGTTGTATAAAGAAGAACCTGAGGTTGAGGCCTTGTTACAAATGAGGGAAAATAACCAT

GAACCAACAGAACATTCCACTTGGATAAGGAGGACCCGATATAACAATAGTGAACACGAC

TTCGTGATCAATGAAGCCAGCTGCTCCTCGGGTAAGAAACAAAAGAGTCACATTTCTAAT

ATTCAGGGGAGCTCTGTCTTTGAGAATCTGCAGCAACAAGTAGAGGGGCCTGTCTCTTCA

GAAACTTTGCAGCTCTTTCCTGCAAACCCAGGATTTTAG

>NTK326_mRNA_93162_cds NTK326_mRNA_93162 gene_54964|id=AT5G17680.1

ATGACGCAGAAGAGCTCTTCCTCTGCCCATACTTTTCGGTGGAGTTATGATGTTTTCTTA

AGTTTTAGAGGTGAGGACGTACGCAAAACATTTGTTGACCATCTCTATGTTGCTCTACAG

CAAAAGGGTATTCATACCTTCAAAGATGATGAGAATCTAGAGAAAGGCAAGTCCATTTCA

CCTGATCTTATGAGAGCAATTGAAGAGTCGCGCATAGCTTTGATTATATTCTCCAGAAAC

TATGCTAATTCGATATGGTGCTTAGATGAATTAGTGAAGATCATGGAATGCAAGAACTTG

AATGGACAAATTGTGTTTCCGGTCTTCTACGATGTAGATCCATCAACAGTGAGGAAACAA

AAGTCAAGCTTTGGAGAAGCATTTAGCAGTCATGAAGCCCATGGCTGTTTCAAGTTGCAA

AAATGGAGGGCGGCATTGGAGGAAGCTGCTAATTTATCTGGCTGCGATTTGCCAAATACT

GCTAATGCGCATGAAGCTAAAGTCATAAAGCAAATTGTGGAAGATATACTGGCTAAATTG

GGTGGTCAGAGGCATGCAATCAATGCTGAAAATCTTGTTGGAATGGAGTCACAAATGCAG

AAAGTGTATAAAATGCTTGGCATCGGTTTTGGAGGAGTTCACTTCGTTGGAATATTTGGA

ATGAGCGGAGTGGGAAAGACAACTTTAGCGAGAGTCATTTATGATAACATTTCAAGTCAA

TTTGAGGGTGCTTGTTTTCTTCATGAGGTTAGAGACCGTTCAGAAAAACAAGGCCTAGCG

CGATTGCAAGAGATACTTCTTTCCAAGATCCTTGTCATAAAAGATCTAAGGATCAACAAT

TTATTTGAAGGAGTTAATATGCATAGACATAGATTACGGTACAAAAAGGTTCTTCTTGTT

CTTGATGATGTTGATCACATAGATCAGTTAGAGGTTTTAGCTCAGAAGCGTGAATGGTTT

GGTTCTGGAAGTAGAATCATCATAACAACTAAAGACAAACACTTGCTTGTTAAGCATGAT

GTGGAAAAGATATACAAAATGAGAACATTAAGTGACGATGAAAGTCTAGAACTATTTAAA

CAATATGCTTTCAAGAAGAACCATCCTACCAAGAAATTTGAGGATCTCTCAGCTCAAGTG

ATAAAGTATACTGCTGGACTCCCCTTGGCTCTGAAGGTCCTGGGCAGTTTCTTGTATGGA

AGAGATTTGGCTGAATGGAGAAGTGAAGTGGAACGATTGAAACAAATCCCGGAAGATGAA

ATTTTGAGGAAACTCGAACCAAGTTTCACTGGACTCAAAAGTATCGATCAAAAGATATTC

TTAGACATTGCGTGTTTCTTTACAGGGAAAAAGAAAGATTCAGTGACTAGAGTTCTTGAG

AGTTTTAATTTTAGCCCTATTATTGGCATAAAAGTTCTTATGGAGAAATCTTTGATTACT

ATTTCAGAAGGTAGGATTTTAATGCACCAATTGATACAAGAAATGGGATGGCACATTGTT

CGTCGAGAAGCTTTCGATTATCCAAGAAGATATAGTAGGTTATGGAAGTCTGAAGATATT

TCTCATGTACTTGCAAGAAATATGGGCACAGAAAAGATCGAAGGCATATCTCTGAACTTG

AGAAAGATGCTCACAGATATTTCTCATGCACTTGAAAGAAATTTGGGCACAGAGAAGATC

AAAGGGATACCATTAAACTTGACCAATGTCAAAGAAGTGAATGTTAGTGCAACAGCCTTC

ATGCCGATGACCAGACTGAGGTTTCTCAAAATCAAGAATGCATATGTTTCTCAGAGTCCT

GATATTCTTCCTAGTGAGTTGAGCTGGCTTTCTTGGCACGGATATCCTTCAAAAAGTCTT

CCAATTAGCTTTCAGGGAGAACGACTCGTTAGTTTGAAGTTAAAAAATAGTCGCATCATA

CAACTTTGGAAAGGCTCCAAGGTTCTAGGACAACTGAAGTACATCAACCTTAGCCATTCA

CATAAGCTAATAAGGACTCCAGATTTTTCGGGTACCCCTAATCTTGAAAGGTTGGTTCTT

GAAGAGTGCACAAGTTTGGTAGAAATCAATTTTTCTGTTGGAGATCTCAAAAAGCTAGTC

TTGCTCAAGTTGAAGAACTGCATCAATTTAAAGACCCTGCCAAAGAGTATTCAATTGGAA

AATCTTGACGTTCTTATTCTATCAGGCTGCTCAAAGCTAAAAGTATTCCCAGAAATAGAA

GAGGAAATGAATCGTTTATCAGAACTATATTTGGAAGCGACTGCTTTTAGTGAACTACCC

GCATCAGTTGAGAAACTATCAGGAGTTAAAGTGATAAATCTAAGCTCATGCAAGAATCTT

GAGAGTCTTCCAAATAGTATTGTTAGGTTGAAATATCTTAAAGAACTTAATGTGTCCAAA

TGCTCAAAACTTAAAAGTTTACCAGATGACTTGGGTTCTTTAGTCAGTTTGGAGGGGCTC

CATTGTGATGACACACCGATCCAAATGATACCCTCCACCATTTCCCTTCTAAAGAACCTT

AAGCACTTATCTCTCCGTCAATGTAATGCTTTAGGTTTGCACGTAAGGAGTTCAATCTCA

AGAGAATCTATGGGACTAGTTTTCTCTAATTTATCAGGTCTTTGTTCATTGACAATGCTG

GATATAGGTGGCTGCAGCATTTCAGATGGAGGCATCCTATGTAATCTTGGGTTCTTACCA

TCTTTGGCGGAATTGAATCTTGGTGGTAACACATTTACTAATATCTCAGCTTCAAGCATC

AGTGGTCTGACTCGACTAAAGGTTCTTCAATTGGTTGGCTGTAGTAGGCTTGAACATTTC

CCAGAGCTTCCTCGAGCTATAGAAGAGGTGCATGCTGATGAGTGTATATCTTTGAAGAGT

ATCCATCAATTAGCAAAATATCCAACATTGCGCCGACTTTCACTTAGCCAATGTCATCAG

CTTCATGATACTGACATGGTTGATGCATTATGGAGCAACATGCTCAAGGGACTATACGTG

CTACGAAATGATCTCAGCATTTGCATCCCTGGATCGCAGATTCCTATGTGGTTTACATAC

AAGAACTTTGGGGAAAATGTTACACTGACTCTTGCCAATAATTGGTACACTGATAACCTC

TGGGGTTTTGCTTTCTGTATTGTTTTTGAACGTATGGAATGGTGCGGTCTATATGATGGT

TACCTACAACCATCACTTGGATTTCCAGTTAACCTTAAATTCAAAACATATGATGGTAAG

GAAGGCGATATACGTAGCATTATTGGCATCAAAGGAGGTGATATGTCAATTCGGAACTCA

GAGCACACTCTCCTTGCCTACGTACCATCTCGTCGTTTTCTGCAACCTTACAATAACGAG

GTTTACAGTCCCAACGACTGGATAGAAATTGTGGCTTATTCTACAGTACAATTCGACAGC

AAAGCTTGGGGAACGCGTCTTGTGTACTTGGACGATATTATTGAAGCATGA

>NTTN90_mRNA_49004_cds mRNA_49004 gene_27480|id=AT5G17680.1:evalue=2e-04:annot='disease resistance protein (TIR-NBS-LRR class), putative';id=Solyc05g007850.1.1:evalue=0.003:annot='Tir-nbs-lrr, resistance protein'

ATGGCATTATCTTCTGCTTCTACAACTACTTCACAGTTTCGGTGGAACTACAAAGTCTTT

CTAAGCTTTAGAGGTAAAGATACTCGAAGCAAATTTACAAGTCACCTCTTCAAAGGCTTG

GAAAGCAGTGGAATATTCACGTTTCAAGATGATAAAAGGCTAGAGCATGGTGCATCAATA

TCAGATGAACTCCTGAAAGCTATCGAACAGTCTCAAGTTGCCCTTGTCGTTTTCTCACAG

AATTATGCAACATCGAGGTGGTGCTTAGATGAGTTAGTAAAGATCATGGAATGCAAGGCT

CAATGTGGACATACTGTCATACCAGTCTTCTATGATGTGGATCCAGCACATGTTCGATAC

CAGAGGAAGAGCTTTGCCAAAGCCTTTAAGAAACACGAAACAAGATATAAGGATGATGAT

GAAGGAATGCAGAAGGTCCAAAGATGGAGGAATGCTCTAACTGCTGCTGCAAATCTAAAA

GGATATGATATCCGTGCCGGGATTGAGGCAGAGAATATTCAGCACATTGTCGACCAAATT

TCCAAATTGTGCAATAGTGCTAGTTTATCTTCTTTGCGAGATGTTGTCGGGATAGATACT

CATCTGGAGAAATTAAAGTCCCTACTTAAGGTAGGAACCAATGATGTTCGGATCATATTG

GGGATCTGGGGCATGGGGGGTCTAGGGAAGACGACAATAGCAAGAGCCATTTTTGACACT

TTATCTCATCAATTTGAAGCTGTTTGTTTCCTTGCGGATATTAAAGAAAATGAAAAAAGA

CATCAACTGTATTCTTTGCAAAACACCCTTCTCTCTGAATTGTTAAGAAGAAAAGATGAT

TACGTCAATAATAAGCATGATGGGAAGCGGATGATTCCGGATAGACTTTGCTCTAAGAAG

GTGCTAATTGTGCTTGATGATATAGATCATAAAGATCATTTAGAGTATTTAGCGGGTGAT

ATTGGTTGGTTTGATAATGGCAGTAGAGTTGTTGTCACAACTAGAAACAAGCATTTGATA

GAGAAGAATGATGTCATTTATGAAGTGACTGGACTAGCTGACCATGAAGCTATGCAGTTA

TTCAGTCAACATGCTTTCAGAAAAGAAGATCCAGATGAGTGTTTTAAGGAGCTCTCATTG

GAGGTAGTAAATTATGCTAAAGGTCTTCCTTTAGCCCTCAAAGTGTGGGGTTCTTTGCTG

CATAACTTAGGCTTAACTGAATGGAAAAGTGCTATAAAGCACATGAAAATTAATTCTAAT

TCGGAAATTGTTGAAAAGCTCAAAATCAGTTATGACGGATTGGAGCCCATCCACCAAGAG

ATGTTTCTAGATATAGCATGCTTCTTGCGAGGGGAAAAAAACGATTACGCCGTGCAAATT

CTTGAGAGTTGTCATTCTGGAGTTGAATACGGATTGCGTATTTTAATTGACAAATCCCTT

GTGTCTATCTCTGAAAATGATCGAATTCAAATGCATGACTTAATACATGATATGGGTAAA

TATATAGTGAATTTTAAAAAAGATCCTGGAGAACGTAGCAGGCTATGGCTCGCCGAGGAA

GTCGAAGAAGTGATGAGCAACAATGCAGGGACCATGGCAGTGGAAGCAATTTGGCTTCAT

GGTAATTTTAGTACACTACGCTTTAACAATGAGGCCATGAAAAATATGAAAAGGCTTAGG

ATATTATACATAGACAAAGAGTTCTATGATTTCAATATTTGGGATGATGGCTCCATTGAG

TATCTGTCCAACAACTTGCGTTGGTTAGTCTTGGATGGCTATCCTTGTGAAACATTGCCA

TCTACATTTGATCCCAAAATGCTTGTTAACCTTCAACTCCACCTTAGTTCACTGCGTTAT

TTATGGACGGAAACAAAGCAATTGTCGTCTCTACGGACGCTAGATCTCAGATACTCTAAA

AGCTTGGTGCGAACACCAGATTTCACGGGGATGCCAAATTTGGAGTATTTGGATCTGTTT

TGGTGTTATAATCTTGAAGAGGTTCACCATTCCCTGGGATGTTGCAGCAAACTCATTTGG

TTAGATTTGAGTTGGTGTCAAAGCCTTAAGAGATTTCCATGTGTTAGCGTGGATTCTCTT

GAATATCTGAGTTTAGATGGTTGCTTTAGGTTAGAGAAATTTCCAGAAATCCACGGGAGA

ATGAAGCCGGAGATACATATTCGCATGGGAGCCTCTGGGATAAGGGAAATACCATCATCT

ATTTTTCAGTACCAGACTCATATTACCAAGCTAGATTTCAGCGGTATGAGATGA

>NTTN90_mRNA_90522_cds mRNA_90522 gene_51170|id=AT5G36930.2:evalue=1e-166:annot='Disease resistance protein (TIR-NBS-LRR class) family';id=Solyc01g102850.1.1:evalue=0.0:annot='Tir-nbs-lrr, resistance protein'

ATGACTCAGTTTGCTTATCATGCATTTCTGAGTTTGGCAACCAAAATAGGCATGTCCTTT

GGAGATCATCTTCTTTCAGCTTTATCAAATGCTGGTATTCGCGCATTCAGAGTTGATGAA

CTTGAGATAGATGAAAAGGGATGCAAAGAACTGCAGAAAACAATTCAAGAATCAAGAATT

CTCATTGTTGTCTTCACTAAAGAGTATACCTCTTCAGAGAGGTGCCTTGATGAACTTGTG

TTTATACTTGAGAGTAAGAGAACTTTTGGACGTTTCGTTCTGCCTGTGTTTTATGATGTG

GATCCCTCAGAAGTCAGGAAACAAAAAGGGAGTTTTGAACAAGATTTTTTAATGTATGAA

GAGAGATTTAAATCCGGGGCCGAAGGAAGGAGACTGGAATGGTTGCAGAAGGTGAAGGAA

TGGAAGGCTGCTCTAACAGAAGTTGCTGATTTGGGAGGAATGGTCTTACAGAATCAATCT

GATGGGTGTGAGTCAAAGTTTATTGAAGAGATTGTGAAGGTGGTTGCAAGAAAACTAAAT

CGCACAGTTTTAAGTGTTGCTCTCCATCCAGTTGGAATAGATTCTCGAGTTAGAGACATC

AATCTCTGGTTGCAAGATGGATCGACTAGTGTTGATATTATGGCTATATATGGGATGGGA

GGTATAGGTAAAACTACACTAGCTAAGACTGCATACAATCTGAACTTTGACAAATTTGAT

GGCAGCAGCTTTCTTGCTGATGTAAATAAAACTTCAGAAAGACATGATGGTCTTGTAAGT

CTACAAAGACAACTTCTTTCAAATGTTCTAGGGAAGAAGGTTGAAAAGATATACAACGTC

GATGAAGGAGTCAGCAAGATTCAAGAAGCCATCCGTTGCAGAAGAATTCTTCTTGTTCTC

GATGATGTGGACGATAGAGATCAGTTAAATGTTGTACTTGGGATGAGAGGATGGTATTAT

CCGGGTAGTAAAATTATCATAACAACCAGAAATCAGCACCTATTTGATGCTAGTGAAGTC

TGCAGATGTAAGATGTATAAGGTCACACCATTGAATGCTCAAGAATCAATTCGACTCTTC

AGTTGGCACGCTTTTGGAAAAGAACAACCTTCAGAAGATCACAAGGACCTTTCAGAAAAT

GTGATACTTCATTGTAAAGGGATTCCTTTGGCTCTCAAAGTTTTAGGTTCTTCTCTTTGT

GACAGAAGTATAGAGGTGTGGGAAAGTGCATTGAGGAAATTAAAGGCAATCCCTGACAAT

AAGATCCTGGAAAAACTCAGAGTTAGCTATGATTTGCTGCCAGATGATGATGTACAGAAT

CTATTCCTCGATATTGTCTGTTTCTTTGTTGGAAAGGATAAAGACTATGCAGTTACTATA

CTTGATGGATGCGGCTTTTTCTCAGTAGTAGGACTTGAGATTCTTTCAGATAGATGTTTG

ATTGAAATGGACAATGATAAGCTGAAAGTGCATTCATTGATTCAAGATATGGGAAAAGAA

ATTATTCGTCTAGAATCCCCTTGGGAGCCTTGGAAGAGAAGCAGAGTTTGGCGATACAGA

GATTCCTTCAATATCTTGAATGAAAAAACTGGAACTGAAAAAATTGAATGCTTAGTTCTT

GACAAGGAAATGTCCACAAAATTGTCCAAACTACTCAAATCTGTCAGATCATATTTCTTC

AACGAAGATATTGGTCTGGTTGGTCATGGCAATTCAAAGAAACGCCGTAAGCATTTGGAG

CATTTTGGTGATGCCGATACAGAGGGTTCAAACAATATAGAATTTGAAGCTGATGCATTT

TCAAGAATGCAGAGGCTGAGGATTCTCCAGCTTAGTTATGTAAGATTCACTGAATTCTAT

AGTTTGTTTCCGAAAAGTCTGAGATTACTGTGTTGGTCTGGATTTCATATGAAGACCATT

CCTGAAGATCTCCCTCTAGAGAGTCTGGTTGCCCTTGAAATGAAAAAAAGTTGTTTGGAA

AAAACCTGGGATGGAACTAAGATTCTCAGATCATTGAAGATCCTTAACTTTAGTCATTCC

CATTTCCTAAAAAGAACTCCTGATTTTTGCGGACTTCCCAATCTAAAAACTTTGATATTG

AAAGACTGCATTAAGTTGGTCAAGATTCATGAATCAATTGGAGTCCTCGACCGACTGGTC

TATCTGAACTTAAGAGACTGCAAGAATCTAAGGAAGCTACCTGGAAGCTTTTGTAAGCTC

AAATCCTTAGAAAAACTTATTATCTCTGGATGTTCTAGACTAGTTACATCAGCAATAGAG

CTAGGCAAGCTAGAATCTTTGAAAACTCTACAGGCTAATGGCATGAATTTTGGTCAGCTA

GCGCCAGTTGGTGGTAATGAGAAATCATGGAGGTCCCTCTGGCAAACTTGGTCATCAAAG

CTAAGAAGATCCCCAGACTCTAACCAGTTTTCCTTCTCTTCTTTATCAAGTTCGCTCGTC

AGCTTAAGTCTTGCTAAATGCAACTTAACAGACGATGCTCTATCGTTTGGCCTCTGTAAC

CTTCCCTCACTATGCTTCTTGAATCTAAGCGAAAATCTGATTTATAACCTGCCTCAGAAT

ATAAAGAACCTTTGTTTGCTCCAAGACCTTTGGTTAGATGGATGCCCGAGCCTCCAATCT

CTGCCAGAGCTTCCGCCGAGCCTTGTTAAGCTGAAGGCAGTTAGATGTTCATCACTGGAA

ACAGTTACAAATCTACCCAACCTAATGTCAGCTCTCTTTTTAGATGTATTGGAAAGTGAG

AATTTAAGTGAGATTTCAGGACTTTTCAAGCTAAATCCCATTGATCATTTTGAAGTGGAA

ATATTGAATACTCTAAGACTTCTCAAGCTGGATAACAGACAAGATACAGTGGTGGAAATA

TTTAGTAGGTTCACTTGCACAAAAAGCACATATTCAGTACAGCAGGGACTCCATGAATTT

GGCATCTTCAGTACTTATTTTCCAGGAAATGAGGTTCCAAGCTGGTTCAGCAACAAGAGT

GAACAGAGGTTGTTGACCCTGAATGTCGATTCACTTCCTAATATCAAGATAACAGGACTG

CACATTTGCGTTGTCTATGCACGTTCTAGTCCTCGTAAATTTCGCTATTTTAGGGACGGG

CATACATTTTACATCAAGGTCCAAAACATTACAAAAGGTCTTAAGTGGATTTATGCCCCA

TCGTTCATTGGCATTCCAGGAGAGAACAACAGGTTGACATTTTTATGCCACTGGGAGTTT

GGGAAATATTTACAAACTGGTGATCAGATTAATGTTTCATTGCCTTGTTGGGGTAACACT

TTTAAGATGAAAGAGCTTGGAGCTACACTTGCATATGATAAGCAAGAATTGGACCAGTCC

TCAGCATCTACTAGTGACGCAAGAGAGCTTGCAATACGACATAATCCAATTAGTGATTAT

CAATCCGAAGAATGTGTCATGGAAGTCTTTATGCCTTCATATCAGATGGCGGCTCATCAT

TACTACCTTTCTCACCCTAATTACTTTGTGCTCCGCGACAATTCAGATTTAGCTGTGAGG

TCAATTTTGAATGAGAAATTGTTTGAGGATTCTTATGTGGAGACTGCATGTTCAGGAGCA

GAGGAAGAAGCTGACGATGATAGCATCTTTGATTACGATGAGGATGAAGATGAAGAGGCT

GCAATAGCAGAGTTAGAGGAGTTGCTGCTAAGATCTTGGTGA

>NTBX_mRNA_28330_cds NTBX_mRNA_28330 gene_16476|id=AT5G17680.1

ATGGCATCTTCTTCTTCTTCTTTCGCGAGTAATTCACAGTACTGTCCTCGATGGAAGTAC

GATGTTTTCCTAAGTTTTAGAGGTGAAGAAACTCGCAAAACGTTTACAGGGCACTTATAT

GAAGGCTTGAGAAACAGGGGAATATTCACGTTTCAAGATGATAAAAGGCTAGAGCATGGC

GCATCCATCTCAGAAGAACTTTGTAAAGCTATCGAAGAGTCTCAAATTGTCGTCATCATT

TTCTCAAAGAATTATGCTACATCGAGGTGGTGCTTGGATGAATTAGTGAAGATCATGGAA

TGCAAGACTCAATTTGGACAAACTGTCATACCGGTCTTCTATGATGTGGATCCATCACAT

GTTCGGAACCAAAGGGAGAGTTTTGCAGAAGCATTTTCCAAACATGAATCAAAGTTTAAG

GATGATGTTGAGGGAATGCAGAAGGTACAAAGATGGAGGAGTGCTTTAACTGAAGCGGCA

AATCTCAAAGGTTGTGATATTCGTAACAGGATTGAATCAGACTGTGTTCAGCAGATCGTT

GACCAAATTTCCAAGTTATGCAAGTTTTCTTTATCTTATTTGCAAGATATTGTAGGAATA

AATCCACATTTAGAGGAAGTAAAATCCCTACTACAAATAGAAATCAATGATGTTCGGATT

GTGGGGATCTGGGGCATGGGAGGAGTTGGTAAAACGACAATAGCAAGAGCCATTTTTGAT

ACACTCTCGTATCAATTTGAAGTTACTTGCTTCCTGGCGGATGTTAAAGAAAACAAATGT

GGAATGCATTCTTTGCAAAATATCCTTCTCTCAGAACTGTTAAGGGAAAACGCTAATTAC

GTGAATAATAAGGAGGACGGAAAGCACCTGATGGCTCGTAGACTTCGCTCTAAGAAGGTT

TTAGTTGTGCTTGATGACATAGATCACAGAGACCATTTGGAGTACCTAGCAGGGGATCTT

GGTTGGTTCGGCAATGGCAGTAGAATTATTGCAACAACAAGAGACAAGCATTTGATTGGG

AAGAAGGATGCATTATATGAAGTGACTACACTAGCTGACCATGAAGCTATTCGATTGTTC

AATCGATACGCTTTTAAGGAAGATGTTCCAGATGAGGTTTTTGAGAAGCTAACGCTGGAG

GTAGTAAGTCATGCGAAAGGCCTTCCTTTAGCGCTGAAAGTGTGGGGTTCTTTCTTTCAT

AAGAGGAATATAACTGAGTGGAGAAGTGCTATACCGCAAATGAAAAAACACTCTAATTCA

GAAATTGTTGACAAGCTCAAAATTAGTTATGATGGATTAGAGCCCGTGGAACAGGCGATA

TTTTTAGATATAGCATGCTTCTTACGAGGGAGAGAAAAGGATGAGATCATACAGATTCTT

GAGAGCTGTGAATTTGGAGCTGATATCGGATTGCGTGTCCTAATTGATAAATCTCTTGTG

TTCATCTCCGAAAAAGATACGATTGAAATGCATGATTTAATACAAGATATGGGTAAATAT

GTCGTGAACATGCAGAAGGATCCTGGAGAACGTAGCAGACTATGGCTCGCTGAAGATTTC

GAAGAAGTGATGACCAACAATACGGGGACCAAGGCAATGGAAGCAATCTGGTTTCGTTAT

TCTCAACGACTATACTTTAGCAAAGAGGCCATGAAAAATATGAAAAGGCTTAGGATATTC

TACATACGTGCTCAGTACTTGAATTCGTGGATCCGTGATGACTTCAATTGCCATGATGGC

CCCAATGAGTACCTGTCTAACAACTTGCGTTGGTTTGTCTGGGATCACTATCCTTGGGAT

TCATTGTCAACTAATTTTGAACCCAAAAGGCTTGTTCATCTTCAACTCTGGCGCAGTTCA

GTGCATCATTTATGGACAGGGATAAAGCATTTGCCGTATCTGCGAAAGCTAGATCTCAGG

GAATCTAAAAGCCTGATGCGAACACCAGATTTTACGGGGATGCCAAATTTGGAGTATTTG

GATCTGGAAAAATGCTCTAATCTTGAAGAGGTTCATCATTCCCTGGGATGTTCCAGAAGA

CTCATTGTCTTAAATTTGTATGAGTGTGGACGCCTTAAGAGGTTTCCATGTGTTAACATG

GAATCTCTTGAATATCTGGGACTATATTGTTGCTATAGTTTAGAGAAATTTCCAGAAATC

CACGGAAGAATGAAGCTGGAGTTAAAGCTTTACATGCAATACTCTGGGATAAGAGAAGTA

CCATCATCTATTACTCAGTGCCAAACTCACATTACCAAGCTAAATTTGAGCAAGTTAAGA

GACATTGCAACTCTTCCAAGTAGCATTCGCATGTTGAAAAGCTTAGTGGAGCTAGATGTG

TCGGATTGCTCAAAACTTGAAATCTTGCCAGAAGAGATAGGGGATTTAGAAAACTTGGAG

AAGTTTGATGCTGCACGTACTTTAATTTCACGGCCTCCACCTTCCATTGTATGCTTGAAC

AAACTTAAATTGTTGTCTTTTGATCAAAGAGAATCAAAAGAAGGCCAAGTCGTGTATTTT

GTGTTCCCTCCTGTGGCTGAAGGCTTACACTCATTGGAAATTTTGCATCTCAGTGACTCC

AATCTAATAGATGGAGGACTTCCAGAAGACATCGGATGCTTATCCTCTTTGAAAAAGTTG

TATCTTGGTTACAATAATTTTGAGCATTTGCCTCAAAGCATAGCCCAACTTGGTGCTCTT

CAATTCTTGTACTTATCAGGTTGCAGTTTGCTTAAAGAGTTGCCAGATTTTATGGGGATG

CCAAATTTGGAGAAGTTGGAGCTGTCATATTGTGAGAATCTTGAAGAGGTTCATCATTCC

CTAGGATTTTTTAAAAAGCTCCGTAAATTATCATTGGATACTTGTGAACGGCTTAAGAGG

TTTCCAGGTCTATGCATTGATTCTCTTGAATTTCTGTGGATACGGGGTTGCTCTAGTTTA

GAAAAATTTCCAGAAATCCACGGAAGCATAAACTCTGAGTTAGAGATTCACATGCTAGAC

AATGTGATAAGAGATCTAGATTTGAGAGGTCTAGAAAACCTTGTAACACTTCCGAGCTGC

ATTTGTAAGTTTAAAAGCTTGGTGAAGCTAGATGTGTCAGATTGCTCAAAACTTGAAATC

TTGCCAGAAGAGATAGGGGATTTAGAAAACTTGGAGTGGCTTGATGCCAGAGATACTATA

ATCTCACAACCTCCGCCTTCCATTATCCGATTGAACAAGCTTAAATTCTTGAGTTTTGCA

AAACAAAAATCACAACTAGGCGTAGAAGATGAAGTGTACTTTGTGTTCCCTCCGGTGGCT

CAAGGATTACGCTCATTGGAAATTCTGAATCTCAGTTACTGCAATCTAATAGATGGAGGA

CTTCCGGAAGACATAGGATGCTTATCCGCTTTGAAAGAGTTGAATCTCAGTGGAAATAAT

TTTGAGCATTTGCCTCGAAGCATAGCTGAATTTGGTGCTCTTCGATCCTTGGACTTAACA

GAGTGCAAGAGTCTTACACAGCTTCCAGAACTTCCACCAGAATTAGATGCATTGCGTGCA

GATTGTCATATGGCTCTGAAAAGTATTCATAATATAGCAACCAAGAAGAAGAAATTGCAG

CAGGTGACATTCAAACCACTGTATGATAGCGATGATACGTACAATGATTCAATCTGTAAT

TTGTTTACCATTGTACATTCTGAGAAGAAGGTCCCAAGTTGGTTCCATTATCAGGGAACG

GATAGAATTGTATCAGTCAATTTGCCTGAAAATTGGTACGTATGTGATAACTTCTTGGGA

TTTGTTGTATGTTACTTTGGCAGCGTAGTTGAAACCATAGCTCAATTGATTCTCTTGTGT

GATGATGGGATGTTGTCGATGACCCAGAAACTTGCCTCACACGACTATTCAAGATCTGAA

ATGAAATCGATGATTCATTTTTTCTTTGTACCTCTTGCTGGCTTATGGGATACATCTAAG

GCAAATGGAAAAACACCAAATGACTTTGGGCTCATTAGGCTATCTTTTTCTGGAGTAATG

AAGGAGTATGGATTCCGCTTGTTGTATAAAGATGAACCTGAGCTTGAGGCCTTGTTACAA

ATGAGGGAAAATAACAATGAACCAACAGAACAATGCATTGGGATAAGGAGGAGCAGATAT

GACAATAGTGAACACCATGACTCTGTGACCAATAAAGCCAGTTCCTCCTCCTCTTCTAAG

AAACAAAGGTCACATTTCTAA

>NTK326_mRNA_35288_cds NTK326_mRNA_35288 gene_20428|id=AT5G17680.1

ATGGAATGCAAGACTCAATTTGGACAAACTGTCATACCGGTCTTCTATGATGTGGATCCA

TCACATGTTCGGAACCAAAGGGAGAGTTTTGCAGAAGCATTTTCCAAACATGAATCAAAG

TTTAAGGGTGATGTTGAGGGAATGCAGAAGGTACAAAGATGGAGGAGTGCTTTAACTGAA

GCGGCAAATCTCAAAGGTTGTGATATTCGTAACAGGATTGAATCAGACTGTGTTCAGCAG

ATCGTTGACCAAATTTCCAAGTTATGCAAGTTTTCTTTATCTTATTTGCAAGATATTGTA

GGAATAAATCCACATTTAGAGGAAGTAAAATCCCTACTACAAATAGAAATCAATGATGTT

CGGATTGTGGGGATCTGGGGCATGGGAGGAGTTGGTAAAACGACAATAGCAAGAGCCATT

TTTGATACACTCTCGTATCAATTTGAAGTTACTTGCTTCCTGGCGGATGTTAAAGAAAAC

AAATGTGGAATGCATTCTTTGCAAAATATCCTTCTCTCAGAACTGTTAAGGGAAAACGCT

AATTACGTGAATAATAAGGAGGACGGAAAGCACCTGATGGCTCGTAGACTTCGCTCTAAG

AAGGTTTTAGTTGTGCTTGATGACATAGATCACAGAGACCATTTGGAGTACCTAGCAGGG

GATCTTGGTTGGTTCGGCAATGGCAGTAGAATTATTGCAACAACAAGAGACAAGCATTTG

ATTGGGAAGAAGGATGCATTATATGAAGTGACTACACTAGCTGACCATGAAGCTATTCGA

TTGTTCAATCGATACGCTTTTAAGGAAGATGTTCCAGATGAGGTTTTTGAGAAGCTAACG

CTGGAGGTAGTAAGTCATGCGAAAGGCCTTCCTTTAGCGCTGAAAGTGTGGGGTTCTTTC

TTTCATAAGAGGAATATAACTGAGTGGAGAAGTGCTATACCGCAAATGAAAAAACACTCT

AATTCAGAAATTGTTGACAAGCTCAAAATTAGTTATGATGGATTAGAGCCCGTGGAACAG

GCGATATTTTTAGATATAGCATGCTTCTTACGAGGGAGAGAAAAGGATGAGATCATACAG

ATTCTTGAGAGCTGTGAATTTGGAGCTGATATCGGATTGCGTGTCCTAATTGATAAATCT

CTTGTGTTCATCTCCGAAAAAGATACGATTGAAATGCATGATTTAATACAAGATATGGGT

AAATATGTCGTGAACATGCAGAAGGATCCTGGAGAACGTAGCAGACTATGGCTCGCTGAA

GATTTCGAAGAAGTGATGACCAACAATACGGGGACCAAGGCAATGGAAGCAATCTGGTTT

CGTTATTCTCAACGACTATACTTTAGCAAAGAGGCCATGAAAAATATGAAAAGGCTTAGG

ATATTCTACATACGTGCTCAGTACTTGAATTCGTGGATCCGTGATGACTTCAATTGCCAT

GATGGCCCCAATGAGTACCTGTCTAACAACTTGCGTTGGTTTGTCTGGGATCACTATCCT

TGGGATTCATTGTCAACTAATTTTGAACCCAAAAGGCTTGTTCATCTTCAACTCTGGCGC

AGTTCAGTGCATCATTTATGGACAGGGATAAAGCATTTGCCGTATCTGCGAAAGCTAGAT

CTCAGGGAATCTAAAAGCCTGATGCGAACACCAGATTTTACGGGGATGCCAAATTTGGAG

TATTTGGATCTGGAAAAATGCTCTAATCTTGAAGAGGTTCATCATTCCCTGGGATGTTCC

AGAAGACTCATTGTCTTAAATTTGTATGAGTGTGGACGCCTTAAGAGGTTTCCATGTGTT

AACATGGAATCTCTTGAATATCTGGGACTATATTGTTGCTATAGTTTAGAGAAATTTCCA

GAAATCCACGGAAGAATGAAGCTGGAGTTAAAGCTTTACATGCAATACTCTGGGATAAGA

GAAGTACCATCATCTATTACTCAGTGCCAAACTCACATTACCAAGCTAAATTTGAGCAAG

TTAAGAGACATTGCAACTCTTCCAAGTAGCATTCGCATGTTGAAAAGCTTAGTGGAGCTA

GATGTGTCGGATTGCTCAAAACTTGAAATCTTGCCAGAAGAGATAGGGGATTTAGAAAAC

TTGGAGAAGTTTGATGCTGCACGTACTTTAATTTCACGGCCTCCACCTTCCATTGTATGC

TTGAACAAACTTAAATTGTTGTCTTTTGATCAAAGAGAATCAAAAGAAGGCCAAGTCGTG

TATTTTGTGTTCCCTCCTGTGGCTGAAGGCTTACACTCATTGGAAATTTTGCATCTCAGT

GACTGCAATCTAATAGATGGAGGACTTCCAGAAGACATCGGATGCTTATCCTCTTTGAAA

AAGTTGTATCTCGGTTACAATGATTTTGAGCATTTGCCTCAAAGCATAGCCCAACTTGGT

GCTCTTCAATTCTTGTACTTATCAGGTTGCAGTTTGCTTAAAGAGTTGCCAGATTTTATG

GGGATGCCAAATTTGGAGAAGTTGGAGCTGTCATATTGTGAGAATCTTGAAGAGGTTCAT

CATTCCCTAGGATTTTTTAAAAAGCTCCGTAAATTATCATTGGATACTTGTGAACGGCTT

AAGAGGTTTCCAGGTCTATGCATTGATTCTCTTGAATTTCTGTGGATACGGGGTTGCTCT

AGTTTAGAAAAATTTCCAGAAATCCACGGAAGCATAAACTCTGAGTTAGAGATTCACATG

CTAGACAATGTGATAAGAGATCTAGATTTGAGAGGTCTAGAAAACCTTGTAACACTTCCG

AGCTGCATTTGTAAGTTTAAAAGCTTGGTGAAGCTAGATGTGTCAGATTGCTCAAAACTT

GAAATCTTGCCAGAAGAGATAGGGGATTTAGAAAACTTGGAGTGGCTTGATGCCAGAGAT

ACTATAATCTCACAACCTCCGCCTTCCATTATCCGATTGAACAAGCTTAAATTCTTGAGT

TTTGCAAAACAAAAATCACAACTAGGCGTAGAAGATGAAGTGTACTTTGTGTTCCCTCCG

GTGGCTCAAGGATTACGCTCATTGGAAATTCTGAATCTCAGTTACTGCAATCTAATAGAT

GGAGGACTTCCGGAAGACATAGGATGCTTATCCGCTTTGAAAGAGTTGAATCTCAGTGGA

AATAATTTTGAGCATTTGCCTCGAAGCATAGCTGAATTTGGTGCTCTTCGATCCTTGGAC

TTAACAGAGTGCAAGAGTCTTACACAGCTTCCAGAACTTCCACCAGAATTAGATGCATTG

CGTGCAGATTGTCATATGGCTCTGAAAAGTATTCATAATATAGCAACCAAGAAGAAGAAA

TTGCAGCAGGTGACATTCAAACCACTGTATGATAGCGATGATACGTACAATGATTCAATC

TGTAATTTGTTTACCATTGTACATTCTGAGAAGAAGGTCCCAAGTTGGTTCCATTATCAG

GGAACGGATAGAATTGTATCAGTCAATTTGCCTGAAAATTGA

>NTTN90_mRNA_35258_cds mRNA_35258 gene_19762|id=AT5G41540.1:evalue=0.63:annot='Disease resistance protein (TIR-NBS-LRR class) family';id=Solyc05g007850.1.1:evalue=1e-90:annot='Tir-nbs-lrr, resistance protein'

ATGGAATGCAAGGATCAATGTGGACAGACTGTCATACCAGTCTTCTATGATGTGGATCCA

TTACATGTTCGAAACCAGAGGGAGAGCTTTGCTGAAGCCTTTGACAAACACGAAACAAGA

TATAAGGATGATGATGAAGGAATGCAGAAGCTCCAAAGATGGAGGAGTGCTCTAACTGCT

GCCGCAAATCTAAAAGGATATGATGTCCGTGACGGGATTGAAGCAGAAAATATTCAGAAG

ATTATCGACCAAATTTCCAAATTGTGCAATAGTGCTACTTTGTCTTCTTTGCGAGATGTT

GTGGGAATAGAAACTCATTTGGAGAAATTAAAGTCCCTACTTAAGGTAGGAATCAATGAT

GTTCGGATCATATTGGGGATGTGGGGCATGGGCGGTGTAGGGAAGACGACAATAGCAAGA

GCTATTTTTGACACTTTATCTCATCAATTTGAAGCTGCTTGTTTCCTTGCGGATATTAAA

GAAAATGAAAAATTACATTCGTTGCAAAATACCCTTCTCTCTAAATTGTTAAGAAGAAAA

GATGATTACGTCAATAATAAGCATGATGGGAAGCGGATGATTTCGGATAGACTTTGCTCT

AAGAAGGTGCTAATTGTGCTTGATGATATAGGTCATAAAGATCATTTAGAGTATTTAGCA

GGTGATATTGGTTGGTTTGGTAATGGCAGTAGGGTTATTGTAACAACTAGAGACAAGCAT

TTGATAGGGAAGGATGATTCTATATATGAAGTGACTGCACTACCTGATCATGAATCCATT

CAATTGTTCCATCAACATGCTTTCAAAAAAGAGGTTCCAGATGAGTGTTTTAAGGAGCTT

TCATTGAAGGGGACCATGGCAATGGAAGCAATTTGGCTTCATGATAATTTTGGTACACTA

CGCTTTAGCAATGAGGCCATGAAAAATATGAAAAGGCTTAGGATATTATACATAGAGAGG

TGGTCCTGTTATGGTTCCATTGAGTATCTGTCCAACAACTTGCATTGGCTTGTCTTGGAT

GGCTATCCTTGTGAGTCACTGCCATCTACATTTGAACCCAAAATGCTTGTTCACGTTCAA

CTCAAACACAATTCACTGCATTATTTATGGATGGAAACAAAGCATTTGCCGTCACTGAAA

AAGCTAATTCTCAGCGGCTCTGGAAACCTGATGCGAACACCAGATTTCAAGGGGATGCCA

AATTTGGAGTATTTGGATTTGAGTTTTTGCAGTAATTTTGAAGAGCTTCACGACACCCTG

GGATGTTGCAGAAAACTCGTCGAGTTAAATTTGACTTGGTGTGAACGCCTTAAGAGGTTT

CCATGTGTTAACGTGGAATCTCTTGAATATCTGAGTTTAGAAAATTGCTCAAGTTTAGAG

AAATTTCCAGAAATCCACGGGAGAATGAAGCCGAAGATACAGATTCACATGCTTGGCTCT

GGGATAAGGGAACTTCCATCCTCATATTTTCAGTACCAAACTCATATTACCGAGCTAGAT

TTGAGCTTTATGAGAAACCTTGTAGCTCTTCCAAGCAGCATCTCTAGGTTGAAAAGTTTG

GTTAGTCTGAGTGTGTTGGATTGCTCAAAACTGGAAAGCTTGCCAGAAGACATTGGATCC

TTATCCTCTTTGAAAGAATTGTATCTCAATGGAAATAATTTTGAACATTTGCCTCGAAGC

ATAGCCCAACTTGGTGCTCTTCGATCCTTAGACTTATCATATTGCCAGAGGCTTACACAG

CTGCCAGAACTTCCCCCTGAATTAAATGAATTACATGTAGATTGTCATATGGCTCTGAAA

TTTATCCATGATTTAGTAACAAAGAGAAAAAAACTACAGAGGGTGATATTCCCTGATGAT

GAGGATGATGCACTCGATGATCCTATATATAATTTGTTTGCACATGCCCTGTTTCAGAAT

ATCTCTTCCTTGAGGCATCACATCTCTGCTTCAGATTCCTTGTCCGAAAGTGTGTTTACC

ATTCTGCATCCTTGGAAGAAGATCCCAAGTTAG

>NTK326_mRNA_97564_cds NTK326_mRNA_97564 gene_57784|id=AT5G36930.1

ATGGCTGATCAGAAAGGCAAAGATCAATCATCTAATATTCAGCCTTCGTTTGCACTTGGT

CCTTGGAAATATGATGTGTTCTTGAGTTGTATAGGTGGTGATGCTTCACAAAACTTTGTA

GATCAACTATACTTAAAACTTTGCCAAGTTAGGATAAACACATTCAAAACTGATGATGTT

TCAGCTGAAGTAGTGATGAATGCAATCGAAGGATCAATCATTTTTATTATTGTTCTTTCC

AAAAACTATGCCTCATCTAGAAGGTGTCTTAATGAGCTTCTACATATCCTTGAGCTCAAG

AAGAATTCCAAACGGTTACTTCTTCCTATATTCTATGATATCGACCCTTCTGATGTGCGC

AAGCAAACTGGAATTTTCGCTGAAGCCTTTGAAAGGCATAGAACATGTTCTCAATCAGAG

CAAACCATTCAATATTGGAAAACTGCCCTCAATAAAGTTGGTAATTTATCTGGATGGGAT

CTCAGGCATGCTGCTGAAGGGTTTGAATCAAAATTTATCCGTATTATCATTGAGGAAGTC

TTACACGAAGTCAAATCTCGAACACCCCTTTATGTTACCAAGCACCCCGTGGCTCTTTCT

CCCCGTGTTAATCAAATAGAGAAGTTATTGTTCAAAGGAGACGGTGATGATGTTCGTGTG

ATTGGGATTCACGGCATGGGTGGAATTGGCAAAACAACTCTTGCAAAAGCTGTGTTTAAC

CAAGTTTTTCAGCATTTCGAGGCAAGTTGCTTTCTTGAAAATGTGAAATCAGAGGCTTCT

GAAAGACATAATGGATTAGTTCATTTACAAGAGCAACTTCTTGGAACGATTCTTAGGAGA

AAGATCAAAGTGCACAATGTGGATGAGGGCATTACATTGATCAAAGAAGGGATTTGGCAG

AAAAAGGTTTTCATAGTCCTTGATGATGTGGATGATCAATGCCAGTTAAATGCATTACTT

GGAGAACGTGATTGGCTTCGCCCGGGTAGTAGAGTTGTTATAACAACCCGAGACAAGCAT

TTGCTCAAAGAACTACATTTGAATGAGCAATATGAAGCCATGAAATTGGATCACAAAAGC

TCTTTACAACTCTTCACTTTACATGCCTTTAGAAATGCACCACCGGCTGAAGACTATAGT

ATGCTTGTCGATGGCATTGTAACTTACTGTGCAGGAGTTCCACTGGCTCTTCAAGTTTTG

GGCGCTTATTTGTCTGATAAAAAGATTGAAGAATGGAAAAATGCAGTGGACAAATTAAAG

ATGATTCCTTCTAACGATATTCATACAAAACTCAGAATAAGCTTTGATGGACTTCCTGAT

GATTTTACTAAAGCTGCTTTCCTTGATATTGCTTATTTCTTCTTCAAAATCCAGAAGAGT

GAGGTCGTAGGTATATTCACAGCTTGCGGTTTCTACCCTGAGGTTGAAATTTGCGAATTG

ATTGACAAATCTTTGTTAACAATCGATGAAAATAAGCATTTGAATATGCATAATTTGATC

CGGGATATGGGACGAGAAATTGTTCATAGGGAATCACCCGATAACCCGGGGAAGCGTAGC

AGATTATGGTGCGCTAAAGACATTTCCGATGTACTCATAGGACACAAGGGCACAAAAGCA

GTTGAAGGAATAGTCCTTGAATCTTCAGCATTGAAGGAAGTACCTTTTAGTACAAAAGCA

TTTGAAAAAATGGCCAAGTTAAGACTCCTACGCATCAATCATTTGCAGTTATATGGAAGT

TTTCAGTATTTACCAAAGTCACTAAAATATTTGCATTGGCACTATTGTCCTTTGAAATGC

TTGCCATCTGACTTTTGTCTGGAGAATCTTGTCATTCTCAACATGAGTTTTGGCAAATTC

AAGGAATCCCAAGCGCCGTTAAAGTATTTCAAGTGTTTGAAGAGCTTGGTATTCTACAGT

TGTGAGTATCTCAAGAAATCCCCAGAGTTTGTTGGTTTACATAGTCTTGAGAAGTTATCA

TTTGGTTATTGCTCAAATTTAATGGGATTGGACTCAACAATTGGAGAATTGAAGAGACTT

CGTATTTTAAACGTAGCTGATTGTAAGAACCTACGAGAACTCCCACGAAGAATCTGTGAG

TTAAAATCACTTGAAATCTTATATCTCGATAGCTGCACAAAACTAGAAGAATTGCCTGAT

GATTTGGGAAAGTTGGAAGGTCTGAAAGAGTTGAATGCAGTTGCAACAGCTATTACAAGA

TTACCTGGTTCTGTTGGACATTTAAAGAACTTAGAGATGCTATTGCTATTACAGGACCTC

GTATTGAAAAGACAATCCAAATTTTCGGACATATTCTCAACTTGGTTGCAACCAAAAGGA

AGCCTTAGTAGAGTGGGATATTTACCTTCTTCAGTTTCAAGTTTAAGTGCTTTAAAAGTT

TTACAAATTGAGAACTGGAATATGACTGAAGATGATATTCCTTTTTCTCTTGCGAGTTTA

TCATCTTTACAGAATCTATGTTTTAGCAATAATAAGTTTCATGTTATACCTTTCAACCTT

TGTGACCTTTCTAGTCTCAAGCATCTGAATTTAAGCGAATGTCCGAATCTTAAAAGTATC

CCTGAAATTCCTCCCACTCTTCAGAATCTCAGAGCTTATAATTGCAAGTCGTTAGAGAGA

CTTCCAAATTTGTCAGGTTTGAAAAGGTTGAAGGAACTGGAATTGTTTCGTTGTGAAATG

TTGACGGAGATTCAAGGGTTGGAGAACCTTGATTCTGTTAGAGAAATAAGTTTATGGAGC

TGCAAGAGTTTTGGAAGACTACTTGACGTATCTAACTTGAGTAAATTGAAGAACTTGGAT

CTTAGTCACTGCGAAAGATTGATAGAGATTCGAGGCTTGGACAACCTTCGTTCCATACGC

TACATCAACTTATTCAATTGCAAGGGTCTCAAAAATCCTTTCACTGAAAACTTCTTCAAA

GTCCATTATGAACATGGTAGTGAGCTCCAACTAGGGCTTTGCAACAGCAATGTTCCGAAT

TGGTTTAGCTACAAAGTAGATGGATGTTCAATGTGCTTCAATATGCCTCTACAAGTGGAG

AGTACATTCTTGGGCATGTTTCTTTGGGTTGTTTATGGAACAGTGGATGAAACTAAAAAT

GTTTATCCTAAAGCCACCATTGTCGATGAAACAAATGGCGTTGAGTTTAACCATCGTCTG

TGGACAACCATATCCTTTGCAGAAAACTCGTCCATCCATTACATACCACGAAATTACTTC

AAATGTCCGGTTAAAGGCAGAGAAACGATGAGCATTCTTATTGAATGCTATGACTTTCCA

ACTGAAGATTTTGTTAAGAAATGTGGAGTTTATCTGTTGTACAAAGACAAGAATGGCCCG

GTTCATTCTTTGTCTCCTGGTTTTCTTTAG

>NTBX_mRNA_29733_cds NTBX_mRNA_29733 gene_17320|id=AT5G17680.1

ATGGCATCATCTTCTGCTTCTGGTACTTCACAGTTTCCTCGATGGAACTACGATGTCTTC

CTAAGTTTTAGAGGTGAAGATACTCGGAAAACATTTACGAGTCACCTGTACGAAATCTTG

GATATCAGGGGAATAAAAACCTTTCAAGATGATAAAAGGCTAGAGCATGGCGCATCCATT

TCGGATGAACTATGTAAAGCTATCGAAGAGTCTCAATGTGCAGTCATCATTTTCTCAAAA

AATTATGCAACATCGAGGTGGTGCTTGAATGAACTAGTGAAGATCATGGATGTCAAGACT

CAATTTGGACAAACTGTCATACCGGTCTTCTATGATGTGGATCCATCACATGTTCGGAAC

CAGAGGGAGAGCTTTGCTGAAGCATTTTCCAAACATGAAACAAAGTATAAGGATGATGTC

GAAGGAATGCAAAGATGGAGGATTGCTTTAACTGCAGCGGCCAATCTCAAAGGTTGTGAT

ATTCGTGACAAGACTGAATCAGACTGTATTCGACAGATTGTTGATCAAATCTCGTCCAAA

TTATGCAAGATTTCTTTATCTTATTTGCAAAACATTGTTGGAATAGATACTCATTTAGAG

AAAATAGAATCCTTACTAGGGATAGGAATCAATGATGTTCGGATTGTGGGGATTTGGGGC

ATGGGGGGAGTCGGTAAAACGACAATAGCTAGAGCTATGTTTGATATTCTCTTAGTAAGA

AGGGATAGTTCCTATCAATTTGATGGTGCTTGTTTCCTTGCGAATATTAAAGAAAACAAA

CGTGGAATGCATTCTCTGCAAAATATTATTTTCTCTGAACTTTTAAAGGAAAAAGCTGAT

TACAACAATAAGGAGGACGGAAAGCACCAAATGGCTAGTAGGCTTCGTTCAAAGAAGGTC

CTAATTGTGCTTGATGACATAGATGATAAAGATCATTATTTGGAGTATTTAGCAGGTGAT

CTTGATTGGTTTGGTAATGGCAGTAGAATTATTGTAACAACTAGAGACAAGCATTTGATG

GGGAAGAATGGTGTAATATATGAAGTGACTGCACTACCTAATCATGAATCCATTCAATTG

TTCTATCAGCATGCTTTCAAAAAAGAGGTTCCAAATGAGCATTTTAAGAAGCTTTCATTG

GAAGTCGTAAATTATGCTAAAGGCCTTCCTTTAGCCCTCAGAGTGTGGGGTTCTTTGCTG

CATAACCTAGGACTAACTGAATGGAAAAGTGCTATAGAGCACATGAAAAATAACTCTAAT

TCTGAAATTGTTAAAAAGCTCAAAATTAGTTATGATGGATTAATAGAGCCCATACAAGAG

ATTTTTCTGGATATAGCATGCTTCTTCCGAGGGACAAAAAAAGAGTACGCCATGCAAATT

CTTGAGAGCTGTCATTGTGCAGTTGAATACGGATTGCGTGTCTTAATTGACAAATCTCTT

GTGTCCATCTCTGAAAATGATCAGATTCAAATGCATGACTTGATGCAAGATATGGGTAAA

TATATAGTGAACTTGCAAAAGAATCCGGGAGAACGCAGCAGATTATGGCTCGACAAGGAT

TTCGAAGAAGTGATGATGAACAATACAGGGACCACGAAAATGGAAGCAATCTGGTTTCCT

TATTACCATTATGTTACATTACGCTTTGGCAAAGAGGCCATGAAAAATATGAAAAAGCTT

AGGATATTAAACATAGAGATGTCGTGGCCTTGTGATGGTTCCATTGAGTATCTGCCCAAC

AGCTTGCGTTGGTTTGTCTGGACTGACTATCCTTGGGAGTCGTTGCCAGCTGAATTTGAA

CCCAAAAAGCTTGTTCATCTTGCACTCAAATCCAGTTCACTGTGTTATTTATGGACGGAA

GCAAAGCAATTGTCGTCTCTACGGACGCTAGATCTCAGATACTCTGAAAGCCTAGTGCGA

ACACCAGATTTCACAGGGATGCCAAATTTGGAGTATTTGAATCTGGAGGAATGTCGTGAT

CTTGAAGAGGTGCACCATTCCCTGGGATTTTGCAGAAAACTCATTCGATTAAATTTGGAG

TCTTGTGGACGCCTTAATTGGTTTCCATGTGTTAACGTGGAATCTCTTGAA

>NTTN90_mRNA_13879_cds mRNA_13879 gene_7793|id=AT1G27170.1:evalue=0.0:annot='transmembrane receptors';id=Solyc05g006630.2.1:evalue=0.0:annot='Tir-nbs-lrr, resistance protein'

ATGGCGGAAGAAGAAGAAGAGACGTGGTCCTTGACCTCAGGGCATAGGTTCCATTGGGAC

ATATTCCTCAGCTTCAGAGGAGAAGACACACGCCACGGATTTACCAACAAACTCTACAAT

GAACTCGTACGGAATGGCGTACGGACATTCATCGACGACGAAGGTCTGGATCGCGGCGAG

GAGATCGCTCCGAATCTCTCTGCCGCGATCGAAGACTCGGCGGCTTCGATCGCCGTGATT

TCACAGAACTACGCCTCTTCGAAGTGGTGCCTCGAAGAACTGGTGAAGATCTCGGAATGC

AAAAGACTATTGCTGCCTGTTTTCTACCGGGTTGACCCGTCGGACGTCCGAAGACAGACG

GGTCCGTTTGAGGAGCATTTTAGGAAGCACGAAATAATGGTGGAAGCAGAGAAAGTTTGT

CGGTGGAGAGAAGCTATGAAAAAAGCTGGTAATATCTCCGGTTGGGATTCCAAGCTCTGG

GAAGAATCGGAGTTAATCCATTCTCTAGTCAAAGAAGTTCTGTCGAAACTCAATAACACA

CCTTTAGGCGTGGCAAAATATCCAGTAGGTCTTCATTCTCGTCTTAATGAGTTATGCAGG

AAATTGGACGTGAAAGGAAATGGTGTGAAAGTATTAGGACTATATGGAATGGGGGGAGTT

GGGAAGACCACTCTTGCCAAGGCTTTGTACAATCAGTTTGTTGTTTATTTCAAGAAACGT

AGCTTTATTTCAGATGTTAAAGAAATTGCAAGGCGCCAAAATGGTATGGCCACTCTTCAA

AGCAAACTTATTGGTGATCTTAACTCAGGTGCTTCGCCAATCATAGACGATACTGCTAAA

GGAATCCGATCAATCAAGGAGGCTATGAATAATGAGCCAGTTGCTGTTTTCCTAGATGAT

GTGGATAATGCAGACCAACTTCGTGTGTTGGTTGGTAGGAGAGACTGGTTTTGCCAAGGA

AGCAGAGTCGTCGTCACCACTAGAGATCAAAATGTTTTACTCCCAAGTATTGTAAACGAA

ACTTTTGAAGTGAATGAGCTTTCTTTGTCCGAGTCATTTACGTTATTTAGTTATCATGCA

TTTGGAAGAGAGCATCCTCCTAAGAACTTTTCTGATCTTGCTGAAGAAGTTGTAAAACTC

AGTGGGGGATTACCTCTGGCTCTGGAAGTTTTCGGATCTTTATTGTTCTACAAGAAAAGA

TTAAAGGAGTGGGAAGATTTAGTGCAAAAGCTGAGACAGATTCGCCCGGGTGATCTTCAA

CAAGTCTTGGAAATAAGTTTTGGAGCTCTAGATGAACAAGAAAAGTGCATCTTTCTTGAT

TTAGCATGTCTTCTTCTTAATACAAGGCTTGAAAGGGAAGATGCAATTGCGATATTTGAA

GGTTGTAGCTTTGGCGCTGAAAGTGCAATCACAGAGCTCACAGCAAAATCGCTTCTTAAA

ATCGTTGATGGGAATATTTTATGGATGCATGATCAGCTCAAAGACATGGGAAGGCAAATT

GTACAACATGAGAATTTTGCAGATGCTGGTAAACGTAGTAGACTGTGGAATCATGACGAT

ATTCTGACTGTCCTAAAGAACCACATGGGGACAAGAACAATTGAGGGCATTGTGCTTGAC

TTTGAGAAGAAGCATGATCTAAATCCTAAGGAAGTAAAGTGGAGTTTGAAGAAAGTATTT

AGAAAGTATATTGGTCAAGGTAGAAAGGAAAATGGTGTAACATTTTACACTAGAGCTTTT

CAGCGTATGGTAAAACTGAGACTTCTTCAAATCAATCATGCCAAATTGGTTGGAAATTTC

AAGCTATTACCTGCTGAACTGAGGTGGCTGCAGTGGAAAGGTTGCCCTCTGGAAGTTATT

CCTCCAGAATTACTGTCCCGAAAGATTGCAGTTCTTGATTTGTCGGAGAGCAAGATTACA

CAGCTCTGGAATAAGAAAAAGTGGAATTGCTACCAGAACAAGATGGCAAAGCAGCTGAAA

GTTATTAATCTACGTAGTTGTCGCCAGCTTAAGGAAATCCCTGATTTATCTGGAATTCAA

TTGGAGAAGCTGATTCTTGAGCAATGCAACGAACTAGTTCTGATCCATCCGTCAATTGGA

GACTTGACTATGTTGACCTATTTGAACATGAAGGACTGTAAGAACATTTTGGCATTTCCA

AATGACGTGTCTGGATTGAAACGTCTACAAATACTAATCCTATCTGGTTGCTCAAGTCTA

AGAGAATTACCAGAGGACTTGAGTGGCTGGAAATCTTTGCGAGAGCTTCTTCTAGATGGT

ACAGCAATAAGAAAGCTACCTAACTCTATCTTTCACCTGAAGAGTCTTCAGATCTTGAAT

TTAAATCATTGCCGATCTTTGGAGTTACTGCCTAGAGCTATTGGGAATCTAAGTTCGCTG

AGAGAGCTTTCTTTTAACGGATCTGCTTTAAAGGAAATGCCCGATTCCATTGGAAATTTG

AAAAATCTTGAGGAATTAGGCTTGAGAATGTGCAAGGGGCTCATCTCACTTCCTGATTCC

CTTGGCGATCTTAAATCTTTAATAGGACTTTATCTTGATCAGAGCTCCATAGAAGAATTG

CCACCTTCTGTTGGTTTATTATCTCATTTGAAGTTCTTTACGGTCAGCAATTGCAAGTCC

TTAACCGAATTGCCAAATTCCACGAGTAACTTATCGTCATTGGTTTGGCTTTGTCTACAA

GGGACCTCAGTTAGTGAACTAAATTTCCATTTAGGAAACTTCAAGTCCCTTGAGAAGCTT

GAGATGAGGAACTGCATTTCAATCAGATGTTTACCTGACTCAATTGGAAATATGTTATGT

TTAACTACTTTGGCCCTATGCAATACATCGATTATTGAGTTGCCAGACTCTATAGGCTTA

TTAGAAAGACTTTGGATGTTGGACCTGAGCAACTGTTTGAATCTCCAACGTCTTCCGGCT

TCAATTGGAAGACTTAAGAGTTTGTGTTACCTCTATATGGATGAAACTGCTGTCTCAGAA

TTACCCAATGAAATTGGAAAGCTTTCAAGCTTAAAACTACTGAAGATGAGAAAGAAACCA

CAGCCTAGAGAGGACGGAAATGAGGATGATTTACATGTAGGAGAAAGCTCAAAACGCGTT

ACTCTTCCAGAATCATTTTCAAATCTATCATCCTTGGAATTCCTAGATGCTCATGCATGG

AAAATATCCGGAAAGATTTCTGATGATTTTCAAAAGTTGTCTGCTTTGGAAAAGCTCGAC

CTTGGACACAACGATTTTTGTAGTCTCCCTTGTAGCATGAAAGGACTTTGCGTTCTCAAG

CGTTTGCTTCTCCCAAACTGCAGAAAGCTCAAGTTTCTACCTCAACTTCCTTCAAGTTTG

GAATGGTTAAATGCTGCAAACTGCTCCGCGTTGGAACATATAGCTAGCATATCAGATTTG

GAATATTTGGAAGAACTCAACTTCAGTAATTGCAAGAAAATAATAGATATTCCTGGCCTT

GAAAGCCTGAAATCTTTGAGAAGGTTGTATACTATAGGTTGCAACGCGTGCTTTGCTTCT

ATAAAAAGGAGGATTTCCAAGGATTGTCTAAGGCATATGAAGTATCTCTGTGTTCCAGGG

GATGATCTTCCAGATTGGTTTATTAAGGAAGTACCTATTAGCTTCTCAACTCGCAAGAAT

CGTGATATCAAGGGGGTGATCATCGGTATAGTTCTCTCGCTAGACCAACAAGTGGAGGAT

AATTTCAGACACGAAGTCCCTTCGATCGTAGATATACAAGCAACGATTACCAGACAAGGT

GATGTTGAACCTAAACTTAAGAAAACTTTGTACTTGATGGGGGTTCCTGATACAGATGAC

GATCAGCTCTATTTATGTCGATTTCAAGAATACAGTGATTTTACGCTCATGTTGGAAGAC

GGAGACAGAGTGCAGGTTGGAATTAGAGAGCGTCCACGTTTTAATGGCCTTAAACTGAAG

AAACATGGGATGTACTTAGTTTTCGAAAATGAGGATGATTTTGATGATAATGATGAGGAT

TTGTTTGACGAATCTCAGCAGTCTGTGTCAAAGAAACTTGCTGACTTCTTTCATTCATTA

TGA

>NTK326_mRNA_93159_cds NTK326_mRNA_93159 gene_54964|id=AT5G17680.1

ATGAGAGCAATTGAAGAGTCGCGCATAGCTTTGATTATATTCTCCAGAAACTATGCTAAT

TCGATATGGTGCTTAGATGAATTAGTGAAGATCATGGAATGCAAGAACTTGAATGGACAA

ATTGTGTTTCCGGTCTTCTACGATGTAGATCCATCAACAGTGAGGAAACAAAAGTCAAGC

TTTGGAGAAGCATTTAGCAGTCATGAAGCCCATGGCTGTTTCAAGTTGCAAAAATGGAGG

GCGGCATTGGAGGAAGCTGCTAATTTATCTGGCTGCGATTTGCCAAATACTGCTAATGCG

CATGAAGCTAAAGTCATAAAGCAAATTGTGGAAGATATACTGGCTAAATTGGGTGGTCAG

AGGCATGCAATCAATGCTGAAAATCTTGTTGGAATGGAGTCACAAATGCAGAAAGTGTAT

AAAATGCTTGGCATCGGTTTTGGAGGAGTTCACTTCGTTGGAATATTTGGAATGAGCGGA

GTGGGAAAGACAACTTTAGCGAGAGTCATTTATGATAACATTTCAAGTCAATTTGAGGGT

GCTTGTTTTCTTCATGAGGTTAGAGACCGTTCAGAAAAACAAGGCCTAGCGCGATTGCAA

GAGATACTTCTTTCCAAGATCCTTGTCATAAAAGATCTAAGGATCAACAATTTATTTGAA

GGAGTTAATATGCATAGACATAGATTACGGTACAAAAAGGTTCTTCTTGTTCTTGATGAT

GTTGATCACATAGATCAGTTAGAGGTTTTAGCTCAGAAGCGTGAATGGTTTGGTTCTGGA

AGTAGAATCATCATAACAACTAAAGACAAACACTTGCTTGTTAAGCATGATGTGGAAAAG

ATATACAAAATGAGAACATTAAGTGACGATGAAAGTCTAGAACTATTTAAACAATATGCT

TTCAAGAAGAACCATCCTACCAAGAAATTTGAGGATCTCTCAGCTCAAGTGATAAAGTAT

ACTGCTGGACTCCCCTTGGCTCTGAAGGTCCTGGGCAGTTTCTTGTATGGAAGAGATTTG

GCTGAATGGAGAAGTGAAGTGGAACGATTGAAACAAATCCCGGAAGATGAAATTTTGAGG

AAACTCGAACCAAGTTTCACTGGACTCAAAAGTATCGATCAAAAGATATTCTTAGACATT

GCGTGTTTCTTTACAGGGAAAAAGAAAGATTCAGTGACTAGAGTTCTTGAGAGTTTTAAT

TTTAGCCCTATTATTGGCATAAAAGTTCTTATGGAGAAATCTTTGATTACTATTTCAGAA

GGTAGGATTTTAATGCACCAATTGATACAAGAAATGGGATGGCACATTGTTCGTCGAGAA

GCTTTCGATTATCCAAGAAGATATAGTAGGTTATGGAAGTCTGAAGATATTTCTCATGTA

CTTGCAAGAAATATGGGCACAGAAAAGATCGAAGGCATATCTCTGAACTTGAGAAAGATG

CTCACAGATATTTCTCATGCACTTGAAAGAAATTTGGGCACAGAGAAGATCAAAGGGATA

CCATTAAACTTGACCAATGTCAAAGAAGTGAATGTTAGTGCAACAGCCTTCATGCCGATG

ACCAGACTGAGGTTTCTCAAAATCAAGAATGCATATGTTTCTCAGAGTCCTGATATTCTT

CCTAGTGAGTTGAGCTGGCTTTCTTGGCACGGATATCCTTCAAAAAGTCTTCCAATTAGC

TTTCAGGGAGAACGACTCGTTAGTTTGAAGTTAAAAAATAGTCGCATCATACAACTTTGG

AAAGGCTCCAAGGTTCTAGGACAACTGAAGTACATCAACCTTAGCCATTCACATAAGCTA

ATAAGGACTCCAGATTTTTCGGGTACCCCTAATCTTGAAAGGTTGGTTCTTGAAGAGTGC

ACAAGTTTGGTAGAAATCAATTTTTCTGTTGGAGATCTCAAAAAGCTAGTCTTGCTCAAG

TTGAAGAACTGCATCAATTTAAAGACCCTGCCAAAGAGTATTCAATTGGAAAATCTTGAC

GTTCTTATTCTATCAGGCTGCTCAAAGCTAAAAGTATTCCCAGAAATAGAAGAGGAAATG

AATCGTTTATCAGAACTATATTTGGAAGCGACTGCTTTTAGTGAACTACCCGCATCAGTT

GAGAAACTATCAGGAGTTAAAGTGATAAATCTAAGCTCATGCAAGAATCTTGAGAGTCTT

CCAAATAGTATTGTTAGGTTGAAATATCTTAAAGAACTTAATGTGTCCAAATGCTCAAAA

CTTAAAAGTTTACCAGATGACTTGGGTTCTTTAGTCAGTTTGGAGGGGCTCCATTGTGAT

GACACACCGATCCAAATGATACCCTCCACCATTTCCCTTCTAAAGAACCTTAAGCACTTA

TCTCTCCGTCAATGTAATGCTTTAGGTTTGCACGTAAGGAGTTCAATCTCAAGAGAATCT

ATGGGACTAGTTTTCTCTAATTTATCAGGTCTTTGTTCATTGACAATGCTGGATATAGGT

GGCTGCAGCATTTCAGATGGAGGCATCCTATGTAATCTTGGGTTCTTACCATCTTTGGCG

GAATTGAATCTTGGTGGTAACACATTTACTAATATCTCAGCTTCAAGCATCAGTGGTCTG

ACTCGACTAAAGGTTCTTCAATTGGTTGGCTGTAGTAGGCTTGAACATTTCCCAGAGCTT

CCTCGAGCTATAGAAGAGGTGCATGCTGATGAGTGTATATCTTTGAAGAGTATCCATCAA

TTAGCAAAATATCCAACATTGCGCCGACTTTCACTTAGCCAATGTCATCAGCTTCATGAT

ACTGACATGGTTGATGCATTATGGAGCAACATGCTCAAGGGACTATACGTGCTACGAAAT

GATCTCAGCATTTGCATCCCTGGATCGCAGATTCCTATGTGGTTTACATACAAGAACTTT

GGGGAAAATGTTACACTGACTCTTGCCAATAATTGGTACACTGATAACCTCTGGGGTTTT

GCTTTCTGTATTGTTTTTGAACGTATGGAATGGTGCGGTCTATATGATGGTTACCTACAA

CCATCACTTGGATTTCCAGTTAACCTTAAATTCAAAACATATGATGGTAAGGAAGGCGAT

ATACGTAGCATTATTGGCATCAAAGGAGGTGATATGTCAATTCGGAACTCAGAGCACACT

CTCCTTGCCTACGTACCATCTCGTCGTTTTCTGCAACCTTACAATAACGAGGTTTACAGT

CCCAACGACTGGATAGAAATTGTGGCTTATTCTACAGTACAATTCGACAGCAAAGCTTGG

GGAACGCGTCTTGTGTACTTGGACGATATTATTGAAGCATGA

>NTTN90_mRNA_54400_cds mRNA_54400 gene_30496|id=AT5G36930.2:evalue=0.0:annot='Disease resistance protein (TIR-NBS-LRR class) family';id=Solyc09g092410.2.1:evalue=0.0:annot='Tir-nbs-lrr, resistance protein'

ATGGATACTCAATTAGTCAGAGGAGAATCATCTACATCTTCTCACTTCTCTTATGAAGTA

TTCCTCAGTTTTAGAGGTGAAGACACCCGAAAAACATTCACTGGTCATCTTTATTCCAAA

TTGTCTGATGTTGGAGTTAATACCTTCATTGATGATGAGGAATTAAGAAAGGGTGATGTG

ATTTCAAGTAAACTAGAGAAAGCAATTGAAGAATCAAGAATTTCCATTATTGTTTTCTCA

AGAAATTATGCTTCCTCTAGTTGGTGTCTAAATGAGCTAGTTAAAATTCTTGAATGCAAA

GAGAAATTAAAGCAGATAGTTTTGCCTATTTTCTATGATGTTGATCCGTCTGAGGTACGA

AAGCAAACTGGGTTATTTGGTGAAGCTTTGGCAAAACATAAGGAACGACCATTTGGAGCT

CAAAGGGTGGAGAAATGGAGAGCTGCACTTACTGAAGCTGCGAATTTATCTGGATGGGAT

TTGCCAAATGTTGCTGATGGGCATGAATCAAAGTTTATTGAAAAAATTATACAACAAGTC

CTACAAGAGGTCAACCAGACACCTCTAGATGTTGCTTGGCACCCAGTTGGAGTAGATTCT

CGTGTCAAAGATATAGAGTTGTTATTGCAAAATGAATGTGAGGATGAAGTTCGCATGATT

GGTATTCACGGAGTTGGTGGCATAGGGAAAACAACTCTGGCAAAATCTATCTACAATCGA

ATGTTTCGACTCTTCGATAGTAGTTGCTTCCTTTCAGATGTTAGATCAGAAGCTGAAGAA

TTTGGTCTTGTCAAGTTACAAGAGAAACTTCTTCAACAAGTACTCAAAAATAAGGACATG

AAAGTTGGCAGTGTCGCTCAAGGCATCAATCTAATCAAAGCAAGACTCGAGTCAAAGAAG

GTTCTAATTGTTCTTGATGACGTGGACCACAAAAACCAATTAGAATCCTTAACAAGAGAA

AGAAGTTGGTTTGGTTCGGGTAGTTTAATAATCACTACCACCCGAGACAAGCGATTGCTA

TGCCGGTTTGGAGAAAAAGAGAGATATGAGGCCAAACTATTAAATGACAATGAAGCTATG

TTACTTTTTTGCTGGCATGCTTTTGACAGTCATTTTCCACCAGAAGATTATGTTAATTTG

GCACGAGACATAATTAGATATTCAGGTAGGCTACCATTAGCTCTTGTGACATTGGGGTCA

CATTTACAAGGAAGTTCTATAGAAGAATGGGGATATGAATTCGAAAAACTAAAATCAATT

CCTCATTGTGATATCCAAAAGATTCTCAAGATAAGCTTTGATGGACTTGATGATGAAACA

CAGACTGTTTTCCTCGATATTGCATGCGCCTTCCATGGGTTTTATGAGCATGAAGTTACT

GAAATGGTAAATGCATGTGGCTTTCATGCTAAAAGTGCAATTGCAACTTTAGTCCAAAGA

CACTTGCTTCAAAGATCTTTGAATATTTTGGAGATGCATGATTTAGTGCGAGATATGGGA

AGAGAAGTCGTTCGCATGGAATCAGCTCGAGATCCTGGAAAACGGAGTAGATTATTCAAC

CCGCAAGAAGTCCGTGATGTTCTACAAGGAAATAAAGGTTCCAAAAAGGTAGAACTACTG

AAGGTAGATCGACGGGAATTTAAGGGAGTGAACTTGAGCACCAAAGCATTTAAGAAAATG

AAAACCCTTAGGGTTCTTATAATGGATGAGTTACATATTAGTGGAGATTTTGAGCTGTTG

TCCAAGGAGCTCAGATGGTTATCTTGGAAAAAATGTCCTTTAAAATGTATACCATCAAAT

TTTCCAGCTGAGAATCTGGTAGTTCTAGATATGCGGGAGAGTGATATCCAAGAATTTCAA

TTGAATTTGCAGTGTTGTAGAAGTTTGAAGAAGTTGGATCTCTCTCATTGCAAGCAACTC

AGAAGCACTCCAAACTTCGATGGTTCTGTGAGTCTTGAGAATTTGTTCCTTGGTAATTGT

TCAAGTCTGACAGAGATCCATCCGTCAATAGGAAATTTGGACAGAATAATTAAACTAGAT

ATGTCTAATTGCGAAAAAATTACGGATCTTCCAAGCAGCATATGTCAGCTAAAATCCCTT

GAAGACTTGGACATCGATGGCTGCTCATCTATAAAAACACTGCCAGATAACCTTGGAGAT

ATGAAAAATCTAAGATCTCTTGAAGCATATGATACGGGTATAAAACAATTGCCTAGATCC

GTTGAAATGCTAAGAAATCTTGAAACTTTGAGAGTGGGAGGTCGAAAGCTAGAGGCCGAA

AGGAGTATTTCTGAAAGAGGAGTCCATCGGATACAATATTCCTTGTCAACCTTTGTATCC

GATTTGAGCCTTACATACTGTAATTTGTCCGAGGCTCATATTCCTAGGAATATTGGGAGT

TTATCCTCCTTAAAATATTTAGATTTGAGTGGCAACAGTTTTCATTGTCTACCCATTGAT

TTTTCCAAGTTACGATTATTGAAGGAGTTGTATTTGAATGACTGTGAGAATCTTCAAACA

CTCATGTCAGTGTGTAATTTAGAGAATCTTGCAATTATTGAACTTGAGAATTGCCAAAAA

TTGGTCAAGATTACAGTGTTGGACAACCTCCCTTCTATATGGTCGATCAACATGACAAGT

TGTAGTTCTCTGCAGAATCCATTCAATGAAGGCTTCTTCAGTGCACCTGCTCTATCATTT

CTATCTAGAAAAGATCCAAATGATCGTAAAATAGAAATTTATCTGGAATGTGATGAGATT

CCAGAATGGTGCAGGAATCAAGTAACAGCTTCATCTATGCGTTTGACTATGCCGACACAT

AATAAGGAGTGTAACTTCTTAGGAATGGTTCTCTGGTTTGTTTTCGGCTGTTCTGATGAA

GCCCCTTTTCCAAGCTTCTCAATTAGTATTGCCCGTAAAAAGACTTTAATTGAGCCGTTG

AATATACCTGAAGAACACAGAGAATTGACCTTTGTATGTTACATATCTTACTTAGATGAA

CCTTTTGATGGCCAGATGATCAAAGGCGGGAAAAAGATAAAAGTGTGGTCTGACGACTTT

ACCCTAAAGAAGATAGGGATCCATTTGTTATATTTAGACCAACATGGTAATGTTATATCA

TTGCCCGGAGACGTGGATCGTTCTTATACTAGGGCGAAAAAGGTTAGGGATTGGTGGGAA

TAG

>NTBX_mRNA_92336_cds NTBX_mRNA_92336 gene_54990|id=AT5G17680.1

ATGACGCAGAAGAGCTCTTCCTCTGCCCATACTTTTCGGTGGAGTTATGATGTTTTCTTA

AGTTTTAGAGGTGAGGACGTACGCAAAACATTTGTTGACCATCTCTATGTTGCTCTACAG

CAAAAGGGTATTCATACCTTCAAAGATGATGAGAATCTAGAGAAAGGCAAGTCCATTTCA

CCTGATCTTATGAGAGCAATTGAAGAGTCGCGCATAGCTTTGATTATATTCTCCAGAAAC

TATGCTAATTCGATATGGTGCTTAGATGAATTAGTGAAGATCATGGAATGCAAGAACTTG

AATGGACAAATTGTGTTTCCGGTCTTCTACGATGTAGATCCATCAACAGTGAGGAAACAA

AAGTCAAGCTTTGGAGAAGCATTTAGCAGTCATGAAGCCCATGGCTGTTTCAAGTTGCAA

AAATGGAGGGCGGCATTGGAGGAAGCTGCTAATTTATCTGGCTGCGATTTGCCAAATACT

GCTAATGCGCATGAAGCTAAAGTCATAAAGCAAATTGTGGAAGATATACTGGCTAAATTG

GGTGGTCAGAGGCATGCAATCAATGCTGAAAATCTTGTTGGAATGGAGTCACAAATGCAG

AAAGTGTATAAAATGCTTGGCATCGGTTTTGGAGGAGTTCACTTCGTTGGAATATTTGGA

ATGAGCGGAGTGGGAAAGACAACTTTAGCGAGAGTCATTTATGATAACATTTCAAGTCAA

TTTGAGGGTGCTTGTTTTCTTCATGAGGTTAGAGACCGTTCAGAAAAACAAGGCCTAGCG

CGATTGCAAGAGATACTTCTTTCCAAGATCCTTGTCATAAAAGATCTAAGGATCAACAAT

TTATTTGAAGGAGTTAATATGCATAGACATAGATTACGGTACAAAAAGGTTCTTCTTGTT

CTTGATGATGTTGATCACATAGATCAGTTAGAGGTTTTAGCTCAGAAGCGTGAATGGTTT

GGTTCTGGAAGTAGAATCATCATAACAACTAAAGACAAACACTTGCTTGTTAAGCATGAT

GTGGAAAAGATATACAAAATGAGAACATTAAGTGACGATGAAAGTCTAGAACTATTTAAA

CAATATGCTTTCAAGAAGAACCATCCTACCAAGAAATTTGAGGATCTCTCAGCTCAAGTG

ATAAAGTATACTGCTGGACTCCCCTTGGCTCTGAAGGTCCTGGGCAGTTTCTTGTATGGA

AGAGATTTGGCTGAATGGAGAAGTGAAGTGGAACGATTGAAACAAATCCCGGAAGATGAA

ATTTTGAGGAAACTCGAACCAAGTTTCACTGGACTCAAAAGTATCGATCAAAAGATATTC

TTAGACATTGCGTGTTTCTTTACAGGGAAAAAGAAAGATTCAGTGACTAGAGTTCTTGAG

AGTTTTAATTTTAGCCCTATTATTGGCATAAAAGTTCTTATGGAGAAATCTTTGATTACT

ATTTCAGAAGGTAGGATTTTAATGCACCAATTGATACAAGAAATGGGATGGCACATTGTT

CGTCGAGAAGCTTTCGATTATCCAAGAAGATATAGTAGGTTATGGAAGTCTGAAGATATT
[truncated: 2,082,507 more chars]
