# Supplementary material for: A role for small RNA in regulating innate immunity during plant growth
Source: PLoS Pathog. 2018 Jan 2;14(1):e1006756. doi: 10.1371/journal.ppat.1006756 (PMC5766230; doi:10.1371/journal.ppat.1006756)
Supplement: S2 Data — (DOCX) [file ppat.1006756.s012.docx]

Supplemental data 2 (a) tobacco sRNA and NLR target alignments

DysRNA_arrange_TPM 02390238_21 AAATGTTCTTCGAGTATCTTC

target id: NTK326_mRNA_79633_cds

74 5'-GAGGTGAAGATACTCGAAGGACATTTACAGA-3' 104 cleavage site: 89

||||||||||||||o||||||

3'-CTTCTATGAGCTTCTTGTAAA-5'

DysRNA_arrange_TPM 02367577_21 AAATGTACTTCGAGTATCTTC

target id: NTK326_mRNA_79633_cds

74 5'-GAGGTGAAGATACTCGAAGGACATTTACAGA-3' 104 cleavage site: 89

|||||||||||||| ||||||

3'-CTTCTATGAGCTTCATGTAAA-5'

DysRNA_arrange_TPM 02390667_21 AAATGTTCTTCGATTATCTTC

target id: NTK326_mRNA_79633_cds

74 5'-GAGGTGAAGATACTCGAAGGACATTTACAGA-3' 104 cleavage site: 89

||||||| ||||||o||||||

3'-CTTCTATTAGCTTCTTGTAAA-5'

DysRNA_arrange_TPM 22153796_21 GAATGTTCTTCGAGTATCTTC

target id: NTK326_mRNA_79633_cds

74 5'-GAGGTGAAGATACTCGAAGGACATTTACAGA-3' 104 cleavage site: 89

||||||||||||||o|||||o

3'-CTTCTATGAGCTTCTTGTAAG-5'

DysRNA_arrange_TPM 02392110_21 AAATGTTTTTCGAGTATCTTC

target id: NTK326_mRNA_79633_cds

74 5'-GAGGTGAAGATACTCGAAGGACATTTACAGA-3' 104 cleavage site: 89

|||||||||||||oo||||||

3'-CTTCTATGAGCTTTTTGTAAA-5'

DysRNA_arrange_TPM 02374763_21 AAATGTGCTTCGAGTATCTTC

target id: NTK326_mRNA_79633_cds

74 5'-GAGGTGAAGATACTCGAAGGACATTTACAGA-3' 104 cleavage site: 89

|||||||||||||| ||||||

3'-CTTCTATGAGCTTCGTGTAAA-5'

DysRNA_arrange_TPM 06471153_20 AATGTTCTTCGAGTATCTTC

target id: NTK326_mRNA_79633_cds

74 5'-GAGGTGAAGATACTCGAAGGACATTTACAG-3' 103 cleavage site: 88

||||||||||||||o|||||

3'-CTTCTATGAGCTTCTTGTAA-5'

DysRNA_arrange_TPM 34632662_21 TAATTTGGACGAGATTCGGTC

target id: NTTN90_mRNA_35268_cds

521 5'-TTGTTGACCGAATCTCGTCCAAATTATGCAA-3' 551 cleavage site: 536

|||||||||||||||||||||

3'-CTGGCTTAGAGCAGGTTTAAT-5'

DysRNA_arrange_TPM 43206041_21 TTTGAGATTGGCCGCTGCAGT

target id: NTTN90_mRNA_35268_cds

449 5'-CTTTAACTGCAGCGGCCAATCTCAAAGGCTG-3' 479 cleavage site: 464

|||||||||||||||||||||

3'-TGACGTCGCCGGTTAGAGTTT-5'

DysRNA_arrange_TPM 34633256_21 TAATTTGGACGAGATTTGGTC

target id: NTTN90_mRNA_35268_cds

521 5'-TTGTTGACCGAATCTCGTCCAAATTATGCAA-3' 551 cleavage site: 536

||||o||||||||||||||||

3'-CTGGTTTAGAGCAGGTTTAAT-5'

DysRNA_arrange_TPM 40864778_21 TTATGGATTCAAATGTAGCTG

target id: NTTN90_mRNA_35268_cds

1785 5'-ATTGCCATCTACATTTGAACCCA-AAATGCT-3' 1814 cleavage site: 1799

|| ||||||||||| ||| ||

3'-GTCGATGTAAACTTAGGTATT-5'

DysRNA_arrange_TPM 34632839_21 TAATTTGGACGAGATTTGATC

target id: NTTN90_mRNA_35268_cds

521 5'-TTGTTGACCGAATCTCGTCCAAATTATGCAA-3' 551 cleavage site: 536

|| |o||||||||||||||||

3'-CTAGTTTAGAGCAGGTTTAAT-5'

DysRNA_arrange_TPM 19766731_21 ATTATGGATTCAAATGTAGCT

target id: NTTN90_mRNA_35268_cds

1786 5'-TTGCCATCTACATTTGAACCCAAAATGCTTG-3' 1816 cleavage site: 1801

||||||||||| ||| |||

3'-TCGATGTAAACTTAGGTATTA-5'

DysRNA_arrange_TPM 35198903_22 TACAGGTGACTTGTAAATGTTT

target id: NTTN90_mRNA_35268_cds

87 5'-TCGGAAAACATTTACGAGTCACCTGTACGAAA-3' 118 cleavage site: 103

||||||||||o|||||||||||

3'-TTTGTAAATGTTCAGTGGACAT-5'

DysRNA_arrange_TPM 41151740_21 TTGAGATTGGCCGCTGCAGTT

target id: NTTN90_mRNA_35268_cds

448 5'-GCTTTAACTGCAGCGGCCAATCTCAAAGGCT-3' 478 cleavage site: 463

|||||||||||||||||||||

3'-TTGACGTCGCCGGTTAGAGTT-5'

DysRNA_arrange_TPM 34632315_22 TAATTTGGACGAGATTCGGTCA

target id: NTTN90_mRNA_35268_cds

520 5'-ATTGTTGACCGAATCTCGTCCAAATTATGCAA-3' 551 cleavage site: 536

||||||||||||||||||||||

3'-ACTGGCTTAGAGCAGGTTTAAT-5'

DysRNA_arrange_TPM 43963689_22 TTTTAGCCAGAGTTGTTTTCCC

target id: NTK326_mRNA_99997_cds

680 5'-GCATAGGGAAAACAACTCTGGC-AAAAGCTAT-3' 710 cleavage site: 695

||||||||||||||||| ||||

3'-CCCTTTTGTTGAGACCGATTTT-5'

DysRNA_arrange_TPM 42993390_21 TTTAGCCAGAGTTGTTTTCCC

target id: NTK326_mRNA_99997_cds

680 5'-GCATAGGGAAAACAACTCTGGC-AAAAGCTA-3' 709 cleavage site: 694

||||||||||||||||| |||

3'-CCCTTTTGTTGAGACCGATTT-5'

DysRNA_arrange_TPM 43964061_21 TTTTAGCCAGAGTTGTTTTCC

target id: NTK326_mRNA_99997_cds

681 5'-CATAGGGAAAACAACTCTGGC-AAAAGCTAT-3' 710 cleavage site: 695

|||||||||||||||| ||||

3'-CCTTTTGTTGAGACCGATTTT-5'

DysRNA_arrange_TPM 40391668_20 TTAATATTTCAGCTCGAACT

target id: NTTN90_mRNA_46474_cds

1342 5'-CATGAAG-TT-A-CTGAAATATTAAATGCA-3' 1368 cleavage site: 1353

|| |o | ||||||||||||

3'-TCAAGCTCGACTTTATAATT-5'

DysRNA_arrange_TPM 39183736_22 TCCACTGAGATTCAGCCCTTTG

target id: NTTN90_mRNA_56562_cds

2608 5'-TCTTTGAAAGAGTTGGATCTCAGTAGAAATAA-3' 2639 cleavage site: 2624

|||| |o||o|||||||| ||

3'-GTTTCCCGACTTAGAGTCACCT-5'

DysRNA_arrange_TPM 39184277_21 TCCACTGAGATTCAGCCCTTT

target id: NTTN90_mRNA_56562_cds

2609 5'-CTTTGAAAGAGTTGGATCTCAGTAGAAATAA-3' 2639 cleavage site: 2624

|||| |o||o|||||||| ||

3'-TTTCCCGACTTAGAGTCACCT-5'

DysRNA_arrange_TPM 34632662_21 TAATTTGGACGAGATTCGGTC

target id: NTK326_mRNA_39598_cds

518 5'-TTGTTGATCAAATCTCGTCCAAATTATGCAA-3' 548 cleavage site: 533

||o| ||||||||||||||||

3'-CTGGCTTAGAGCAGGTTTAAT-5'

DysRNA_arrange_TPM 43206041_21 TTTGAGATTGGCCGCTGCAGT

target id: NTK326_mRNA_39598_cds

446 5'-CTTTAACTGCAGCGGCCAATCTCAAAGGTTG-3' 476 cleavage site: 461

|||||||||||||||||||||

3'-TGACGTCGCCGGTTAGAGTTT-5'

DysRNA_arrange_TPM 34633256_21 TAATTTGGACGAGATTTGGTC

target id: NTK326_mRNA_39598_cds

518 5'-TTGTTGATCAAATCTCGTCCAAATTATGCAA-3' 548 cleavage site: 533

||o||||||||||||||||||

3'-CTGGTTTAGAGCAGGTTTAAT-5'

DysRNA_arrange_TPM 34632839_21 TAATTTGGACGAGATTTGATC

target id: NTK326_mRNA_39598_cds

518 5'-TTGTTGATCAAATCTCGTCCAAATTATGCAA-3' 548 cleavage site: 533

|||||||||||||||||||||

3'-CTAGTTTAGAGCAGGTTTAAT-5'

DysRNA_arrange_TPM 35198903_22 TACAGGTGACTTGTAAATGTTT

target id: NTK326_mRNA_39598_cds

84 5'-TCGGAAAACATTTACGAGTCACCTGTACGAAA-3' 115 cleavage site: 100

||||||||||o|||||||||||

3'-TTTGTAAATGTTCAGTGGACAT-5'

DysRNA_arrange_TPM 41151740_21 TTGAGATTGGCCGCTGCAGTT

target id: NTK326_mRNA_39598_cds

445 5'-GCTTTAACTGCAGCGGCCAATCTCAAAGGTT-3' 475 cleavage site: 460

|||||||||||||||||||||

3'-TTGACGTCGCCGGTTAGAGTT-5'

DysRNA_arrange_TPM 34632315_22 TAATTTGGACGAGATTCGGTCA

target id: NTK326_mRNA_39598_cds

517 5'-ATTGTTGATCAAATCTCGTCCAAATTATGCAA-3' 548 cleavage site: 533

|||o| ||||||||||||||||

3'-ACTGGCTTAGAGCAGGTTTAAT-5'

DysRNA_arrange_TPM 34632662_21 TAATTTGGACGAGATTCGGTC

target id: NTBX_mRNA_29733_cds

518 5'-TTGTTGATCAAATCTCGTCCAAATTATGCAA-3' 548 cleavage site: 533

||o| ||||||||||||||||

3'-CTGGCTTAGAGCAGGTTTAAT-5'

DysRNA_arrange_TPM 43206041_21 TTTGAGATTGGCCGCTGCAGT

target id: NTBX_mRNA_29733_cds

446 5'-CTTTAACTGCAGCGGCCAATCTCAAAGGTTG-3' 476 cleavage site: 461

|||||||||||||||||||||

3'-TGACGTCGCCGGTTAGAGTTT-5'

DysRNA_arrange_TPM 34633256_21 TAATTTGGACGAGATTTGGTC

target id: NTBX_mRNA_29733_cds

518 5'-TTGTTGATCAAATCTCGTCCAAATTATGCAA-3' 548 cleavage site: 533

||o||||||||||||||||||

3'-CTGGTTTAGAGCAGGTTTAAT-5'

DysRNA_arrange_TPM 34632839_21 TAATTTGGACGAGATTTGATC

target id: NTBX_mRNA_29733_cds

518 5'-TTGTTGATCAAATCTCGTCCAAATTATGCAA-3' 548 cleavage site: 533

|||||||||||||||||||||

3'-CTAGTTTAGAGCAGGTTTAAT-5'

DysRNA_arrange_TPM 35198903_22 TACAGGTGACTTGTAAATGTTT

target id: NTBX_mRNA_29733_cds

84 5'-TCGGAAAACATTTACGAGTCACCTGTACGAAA-3' 115 cleavage site: 100

||||||||||o|||||||||||

3'-TTTGTAAATGTTCAGTGGACAT-5'

DysRNA_arrange_TPM 41151740_21 TTGAGATTGGCCGCTGCAGTT

target id: NTBX_mRNA_29733_cds

445 5'-GCTTTAACTGCAGCGGCCAATCTCAAAGGTT-3' 475 cleavage site: 460

|||||||||||||||||||||

3'-TTGACGTCGCCGGTTAGAGTT-5'

DysRNA_arrange_TPM 34632315_22 TAATTTGGACGAGATTCGGTCA

target id: NTBX_mRNA_29733_cds

517 5'-ATTGTTGATCAAATCTCGTCCAAATTATGCAA-3' 548 cleavage site: 533

|||o| ||||||||||||||||

3'-ACTGGCTTAGAGCAGGTTTAAT-5'

DysRNA_arrange_TPM 27046016_20 GTGAAATTCTTGGATCGTCT

target id: NTTN90_mRNA_51079_cds

1834 5'-GAGAGTGAT-ATCCAAGAATTTCGATTGAA-3' 1862 cleavage site: 1847

||o |||||||||||||o

3'-TCTGCTAGGTTCTTAAAGTG-5'

DysRNA_arrange_TPM 43963689_22 TTTTAGCCAGAGTTGTTTTCCC

target id: NTTN90_mRNA_42249_cds

680 5'-GCATAGGGAAAACAACTCTGGC-AAAAGCTAT-3' 710 cleavage site: 695

||||||||||||||||| ||||

3'-CCCTTTTGTTGAGACCGATTTT-5'

DysRNA_arrange_TPM 44325453_23 TTTTTGATCCTTCGATGTCGGCT

target id: NTTN90_mRNA_42249_cds

196 5'-GAGAAAG-C-A-ATTGAAGGATCAAGAATTTCC-3' 225 cleavage site: 210

|| | | ||o||||||||||o||

3'-TCGGCTGTAGCTTCCTAGTTTTT-5'

DysRNA_arrange_TPM 20072557_21 ATTGGTCAAGGAAGTTTTTCT

target id: NTTN90_mRNA_42249_cds

796 5'-CTACAAGAGAAACTTCTTCGACAAATTCTCA-3' 826 cleavage site: 811

|||o|||||||o| ||| |||

3'-TCTTTTTGAAGGAACTGGTTA-5'

DysRNA_arrange_TPM 44061720_22 TTTTGATCCTTCGATGTCGGCT

target id: NTTN90_mRNA_42249_cds

196 5'-GAGAAAG-C-A-ATTGAAGGATCAAGAATTTC-3' 224 cleavage site: 209

|| | | ||o||||||||||o|

3'-TCGGCTGTAGCTTCCTAGTTTT-5'

DysRNA_arrange_TPM 41297271_20 TTGATCCTTCGATGTCGGCT

target id: NTTN90_mRNA_42249_cds

196 5'-GAGAAAG-C-A-ATTGAAGGATCAAGAATT-3' 222 cleavage site: 207

|| | | ||o||||||||||

3'-TCGGCTGTAGCTTCCTAGTT-5'

DysRNA_arrange_TPM 42993390_21 TTTAGCCAGAGTTGTTTTCCC

target id: NTTN90_mRNA_42249_cds

680 5'-GCATAGGGAAAACAACTCTGGC-AAAAGCTA-3' 709 cleavage site: 694

||||||||||||||||| |||

3'-CCCTTTTGTTGAGACCGATTT-5'

DysRNA_arrange_TPM 43964061_21 TTTTAGCCAGAGTTGTTTTCC

target id: NTTN90_mRNA_42249_cds

681 5'-CATAGGGAAAACAACTCTGGC-AAAAGCTAT-3' 710 cleavage site: 695

|||||||||||||||| ||||

3'-CCTTTTGTTGAGACCGATTTT-5'

DysRNA_arrange_TPM 40391668_20 TTAATATTTCAGCTCGAACT

target id: NTTN90_mRNA_42249_cds

1369 5'-CATGAAG-TT-A-CTGAAATATTAAATGCA-3' 1395 cleavage site: 1380

|| |o | ||||||||||||

3'-TCAAGCTCGACTTTATAATT-5'

DysRNA_arrange_TPM 32487580_22 CCTTTCGTGCTCAAGTTCTCTC

target id: NTTN90_mRNA_42249_cds

1641 5'-AAAGGGAGTGAACTTGAGCACCAAAGCATTTG-3' 1672 cleavage site: 1657

||| |||||||||||| ||||

3'-CTCTCTTGAACTCGTGCTTTCC-5'

DysRNA_arrange_TPM 40586237_20 TTAGCTGTATCAGAACACCT

target id: NTBX_mRNA_13089_cds

3814 5'-TTGATGGGGGTTCCTGATACAGATGACGATC-3' 3844 cleavage site: 3829

o|| ||| ||||||||| |o|

3'-TCCACAA-GACTATGTCGATT-5'

DysRNA_arrange_TPM 43963689_22 TTTTAGCCAGAGTTGTTTTCCC

target id: NTTN90_mRNA_89783_cds

680 5'-GCATAGGGAAAACAACTCTGGC-AAAAGCTAT-3' 710 cleavage site: 695

||||||||||||||||| ||||

3'-CCCTTTTGTTGAGACCGATTTT-5'

DysRNA_arrange_TPM 42993390_21 TTTAGCCAGAGTTGTTTTCCC

target id: NTTN90_mRNA_89783_cds

680 5'-GCATAGGGAAAACAACTCTGGC-AAAAGCTA-3' 709 cleavage site: 694

||||||||||||||||| |||

3'-CCCTTTTGTTGAGACCGATTT-5'

DysRNA_arrange_TPM 43964061_21 TTTTAGCCAGAGTTGTTTTCC

target id: NTTN90_mRNA_89783_cds

681 5'-CATAGGGAAAACAACTCTGGC-AAAAGCTAT-3' 710 cleavage site: 695

|||||||||||||||| ||||

3'-CCTTTTGTTGAGACCGATTTT-5'

DysRNA_arrange_TPM 36082679_22 TATTGTGGTGTCGGATCCTCCT

target id: NTBX_mRNA_99245_cds

3425 5'-GCATAAGGAGGACCCGATATAACAATAGTGAA-3' 3456 cleavage site: 3441

||||||| ||||o|o ||||||

3'-TCCTCCTAGGCTGTGGTGTTAT-5'

DysRNA_arrange_TPM 35978105_20 TATCTGATATGTGGGCCATC

target id: NTTN90_mRNA_90522_cds

3445 5'-GTCTTTAT-GCCTTCATATCAGATGGCGGC-3' 3473 cleavage site: 3458

|| |||o ||||||||||o

3'-CTACCGGGTGTATAGTCTAT-5'

DysRNA_arrange_TPM 03780453_24 AAGCGAGGCAATGAAACAGTAACT

target id: NTTN90_mRNA_90522_cds

3261 5'-TGATCAGATTAATGTTTCATTGCCTTGTTGGGGTA-3' 3295 cleavage site: 3280

|| ||| |||||||||||||o|o|

3'-TC-AATGACAAAGTAACGGAGCGAA-5'

DysRNA_arrange_TPM 29446078_21 CACTAGAAGATGCTGCAGCGC

target id: NTTN90_mRNA_90522_cds

3352 5'-GACCAGTCCT-CAGCATCTACTAGTGACGCA-3' 3381 cleavage site: 3366

|o || |||||||| ||||||

3'-CGCGACGTCGTAGAAGATCAC-5'

DysRNA_arrange_TPM 40586237_20 TTAGCTGTATCAGAACACCT

target id: NTTN90_mRNA_13879_cds

3814 5'-TTGATGGGGGTTCCTGATACAGATGACGATC-3' 3844 cleavage site: 3829

o|| ||| ||||||||| |o|

3'-TCCACAA-GACTATGTCGATT-5'

DysRNA_arrange_TPM 40498063_21 TTAGATACTGAAACAACAGGT

target id: NTK326_mRNA_109102_cds

2200 5'-CCATCATCTATT-TTTCAGTATC-AAACTCA-3' 2228 cleavage site: 2213

|o|| || |||||||||| ||

3'-TGGACAACAAAGTCATAGATT-5'

DysRNA_arrange_TPM 43963689_22 TTTTAGCCAGAGTTGTTTTCCC

target id: NTBX_mRNA_99861_cds

680 5'-GCATAGGGAAAACAACTCTGGC-AAAAGCTAT-3' 710 cleavage site: 695

||||||||||||||||| ||||

3'-CCCTTTTGTTGAGACCGATTTT-5'

DysRNA_arrange_TPM 42993390_21 TTTAGCCAGAGTTGTTTTCCC

target id: NTBX_mRNA_99861_cds

680 5'-GCATAGGGAAAACAACTCTGGC-AAAAGCTA-3' 709 cleavage site: 694

||||||||||||||||| |||

3'-CCCTTTTGTTGAGACCGATTT-5'

DysRNA_arrange_TPM 43964061_21 TTTTAGCCAGAGTTGTTTTCC

target id: NTBX_mRNA_99861_cds

681 5'-CATAGGGAAAACAACTCTGGC-AAAAGCTAT-3' 710 cleavage site: 695

|||||||||||||||| ||||

3'-CCTTTTGTTGAGACCGATTTT-5'

DysRNA_arrange_TPM 43963689_22 TTTTAGCCAGAGTTGTTTTCCC

target id: NTBX_mRNA_95736_cds

671 5'-GCATAGGGAAAACAACTCTGGC-AAAAGCTAT-3' 701 cleavage site: 686

||||||||||||||||| ||||

3'-CCCTTTTGTTGAGACCGATTTT-5'

DysRNA_arrange_TPM 42993390_21 TTTAGCCAGAGTTGTTTTCCC

target id: NTBX_mRNA_95736_cds

671 5'-GCATAGGGAAAACAACTCTGGC-AAAAGCTA-3' 700 cleavage site: 685

||||||||||||||||| |||

3'-CCCTTTTGTTGAGACCGATTT-5'

DysRNA_arrange_TPM 43964061_21 TTTTAGCCAGAGTTGTTTTCC

target id: NTBX_mRNA_95736_cds

672 5'-CATAGGGAAAACAACTCTGGC-AAAAGCTAT-3' 701 cleavage site: 686

|||||||||||||||| ||||

3'-CCTTTTGTTGAGACCGATTTT-5'

DysRNA_arrange_TPM 40391668_20 TTAATATTTCAGCTCGAACT

target id: NTBX_mRNA_95736_cds

1360 5'-CATGAAG-TT-A-CTGAAATATTAAATGCA-3' 1386 cleavage site: 1371

|| |o | ||||||||||||

3'-TCAAGCTCGACTTTATAATT-5'

DysRNA_arrange_TPM 34632662_21 TAATTTGGACGAGATTCGGTC

target id: NTBX_mRNA_42672_cds

521 5'-TTGTTGACCGAATCTCGTCCAAATTATGCAA-3' 551 cleavage site: 536

|||||||||||||||||||||

3'-CTGGCTTAGAGCAGGTTTAAT-5'

DysRNA_arrange_TPM 43206041_21 TTTGAGATTGGCCGCTGCAGT

target id: NTBX_mRNA_42672_cds

449 5'-CTTTAACTGCAGCGGCCAATCTCAAAGGCTG-3' 479 cleavage site: 464

|||||||||||||||||||||

3'-TGACGTCGCCGGTTAGAGTTT-5'

DysRNA_arrange_TPM 34633256_21 TAATTTGGACGAGATTTGGTC

target id: NTBX_mRNA_42672_cds

521 5'-TTGTTGACCGAATCTCGTCCAAATTATGCAA-3' 551 cleavage site: 536

||||o||||||||||||||||

3'-CTGGTTTAGAGCAGGTTTAAT-5'

DysRNA_arrange_TPM 35716976_20 TATATCGAGTCCTGTTAATT

target id: NTBX_mRNA_42672_cds

3319 5'-ACTTGGATAAGGAGGACCCGATATAACAAT-3' 3348 cleavage site: 3333

o|| |o ||||| |||||||

3'-TTAATTGTCCTGAGCTATAT-5'

DysRNA_arrange_TPM 34632839_21 TAATTTGGACGAGATTTGATC

target id: NTBX_mRNA_42672_cds

521 5'-TTGTTGACCGAATCTCGTCCAAATTATGCAA-3' 551 cleavage site: 536

|| |o||||||||||||||||

3'-CTAGTTTAGAGCAGGTTTAAT-5'

DysRNA_arrange_TPM 35198903_22 TACAGGTGACTTGTAAATGTTT

target id: NTBX_mRNA_42672_cds

87 5'-TCGGAAAACATTTACGAGTCACCTGTACGAAA-3' 118 cleavage site: 103

||||||||||o|||||||||||

3'-TTTGTAAATGTTCAGTGGACAT-5'

DysRNA_arrange_TPM 41151740_21 TTGAGATTGGCCGCTGCAGTT

target id: NTBX_mRNA_42672_cds

448 5'-GCTTTAACTGCAGCGGCCAATCTCAAAGGCT-3' 478 cleavage site: 463

|||||||||||||||||||||

3'-TTGACGTCGCCGGTTAGAGTT-5'

DysRNA_arrange_TPM 34632315_22 TAATTTGGACGAGATTCGGTCA

target id: NTBX_mRNA_42672_cds

520 5'-ATTGTTGACCGAATCTCGTCCAAATTATGCAA-3' 551 cleavage site: 536

||||||||||||||||||||||

3'-ACTGGCTTAGAGCAGGTTTAAT-5'

DysRNA_arrange_TPM 34632662_21 TAATTTGGACGAGATTCGGTC

target id: NTTN90_mRNA_68293_cds

518 5'-TTGTTGATCAAATCTCGTCCAAATTATGCAA-3' 548 cleavage site: 533

||o| ||||||||||||||||

3'-CTGGCTTAGAGCAGGTTTAAT-5'

DysRNA_arrange_TPM 43206041_21 TTTGAGATTGGCCGCTGCAGT

target id: NTTN90_mRNA_68293_cds

446 5'-CTTTAACTGCAGCGGCCAATCTCAAAGGTTG-3' 476 cleavage site: 461

|||||||||||||||||||||

3'-TGACGTCGCCGGTTAGAGTTT-5'

DysRNA_arrange_TPM 34633256_21 TAATTTGGACGAGATTTGGTC

target id: NTTN90_mRNA_68293_cds

518 5'-TTGTTGATCAAATCTCGTCCAAATTATGCAA-3' 548 cleavage site: 533

||o||||||||||||||||||

3'-CTGGTTTAGAGCAGGTTTAAT-5'

DysRNA_arrange_TPM 34632839_21 TAATTTGGACGAGATTTGATC

target id: NTTN90_mRNA_68293_cds

518 5'-TTGTTGATCAAATCTCGTCCAAATTATGCAA-3' 548 cleavage site: 533

|||||||||||||||||||||

3'-CTAGTTTAGAGCAGGTTTAAT-5'

DysRNA_arrange_TPM 35198903_22 TACAGGTGACTTGTAAATGTTT

target id: NTTN90_mRNA_68293_cds

84 5'-TCGGAAAACATTTACGAGTCACCTGTACGAAA-3' 115 cleavage site: 100

||||||||||o|||||||||||

3'-TTTGTAAATGTTCAGTGGACAT-5'

DysRNA_arrange_TPM 41151740_21 TTGAGATTGGCCGCTGCAGTT

target id: NTTN90_mRNA_68293_cds

445 5'-GCTTTAACTGCAGCGGCCAATCTCAAAGGTT-3' 475 cleavage site: 460

|||||||||||||||||||||

3'-TTGACGTCGCCGGTTAGAGTT-5'

DysRNA_arrange_TPM 34632315_22 TAATTTGGACGAGATTCGGTCA

target id: NTTN90_mRNA_68293_cds

517 5'-ATTGTTGATCAAATCTCGTCCAAATTATGCAA-3' 548 cleavage site: 533

|||o| ||||||||||||||||

3'-ACTGGCTTAGAGCAGGTTTAAT-5'

DysRNA_arrange_TPM 43963689_22 TTTTAGCCAGAGTTGTTTTCCC

target id: NTBX_mRNA_49496_cds

680 5'-GCATAGGGAAAACAACTCTGGC-AAAAGCTAT-3' 710 cleavage site: 695

||||||||||||||||| ||||

3'-CCCTTTTGTTGAGACCGATTTT-5'

DysRNA_arrange_TPM 42993390_21 TTTAGCCAGAGTTGTTTTCCC

target id: NTBX_mRNA_49496_cds

680 5'-GCATAGGGAAAACAACTCTGGC-AAAAGCTA-3' 709 cleavage site: 694

||||||||||||||||| |||

3'-CCCTTTTGTTGAGACCGATTT-5'

DysRNA_arrange_TPM 43964061_21 TTTTAGCCAGAGTTGTTTTCC

target id: NTBX_mRNA_49496_cds

681 5'-CATAGGGAAAACAACTCTGGC-AAAAGCTAT-3' 710 cleavage site: 695

|||||||||||||||| ||||

3'-CCTTTTGTTGAGACCGATTTT-5'

DysRNA_arrange_TPM 27046016_20 GTGAAATTCTTGGATCGTCT

target id: NTBX_mRNA_49496_cds

1897 5'-GAGAGTGAT-ATCCAAGAATTTCGATTGAA-3' 1925 cleavage site: 1910

||o |||||||||||||o

3'-TCTGCTAGGTTCTTAAAGTG-5'

DysRNA_arrange_TPM 33334126_21 CTCCGCCCTTCTGTTTTTCTC

target id: NTTN90_mRNA_98067_cds

376 5'-GTAGTGA-GAAAACAGAA-GG-GGAGTTATC-3' 403 cleavage site: 388

|| o||||||||| || ||||

3'-CTCTTTTTGTCTTCCCGCCTC-5'

DysRNA_arrange_TPM 30920174_21 CGCCCTTCTGTTTTTCTCGCT

target id: NTTN90_mRNA_98067_cds

373 5'-TCAGTAGTGA-GAAAACAGAAGGGGAGTTAT-3' 402 cleavage site: 387

||o|| o||||||||||||

3'-TCGCTCTTTTTGTCTTCCCGC-5'

DysRNA_arrange_TPM 34174695_22 TAAGAACTTGCATGATGGCACC

target id: NTK326_mRNA_90605_cds

1355 5'-AAAAAGGTGCCATCATGCAAGTTCTTAAGAGT-3' 1386 cleavage site: 1371

||||||||||||||||||||||

3'-CCACGGTAGTACGTTCAAGAAT-5'

DysRNA_arrange_TPM 36089404_21 TATTGTTGATCACCCTTTCCT

target id: NTK326_mRNA_90605_cds

1557 5'-TTTTGAAGAAATGATGATCAACAATACGGGT-3' 1587 cleavage site: 1572

|||| | ||||||||||||

3'-TCCTTTCCCACTAGTTGTTAT-5'

DysRNA_arrange_TPM 34632662_21 TAATTTGGACGAGATTCGGTC

target id: NTTN90_mRNA_35271_cds

521 5'-TTGTTGACCGAATCTCGTCCAAATTATGCAA-3' 551 cleavage site: 536

|||||||||||||||||||||

3'-CTGGCTTAGAGCAGGTTTAAT-5'

DysRNA_arrange_TPM 43206041_21 TTTGAGATTGGCCGCTGCAGT

target id: NTTN90_mRNA_35271_cds

449 5'-CTTTAACTGCAGCGGCCAATCTCAAAGGCTG-3' 479 cleavage site: 464

|||||||||||||||||||||

3'-TGACGTCGCCGGTTAGAGTTT-5'

DysRNA_arrange_TPM 34633256_21 TAATTTGGACGAGATTTGGTC

target id: NTTN90_mRNA_35271_cds

521 5'-TTGTTGACCGAATCTCGTCCAAATTATGCAA-3' 551 cleavage site: 536

||||o||||||||||||||||

3'-CTGGTTTAGAGCAGGTTTAAT-5'

DysRNA_arrange_TPM 34632839_21 TAATTTGGACGAGATTTGATC

target id: NTTN90_mRNA_35271_cds

521 5'-TTGTTGACCGAATCTCGTCCAAATTATGCAA-3' 551 cleavage site: 536

|| |o||||||||||||||||

3'-CTAGTTTAGAGCAGGTTTAAT-5'

DysRNA_arrange_TPM 35198903_22 TACAGGTGACTTGTAAATGTTT

target id: NTTN90_mRNA_35271_cds

87 5'-TCGGAAAACATTTACGAGTCACCTGTACGAAA-3' 118 cleavage site: 103

||||||||||o|||||||||||

3'-TTTGTAAATGTTCAGTGGACAT-5'

DysRNA_arrange_TPM 41151740_21 TTGAGATTGGCCGCTGCAGTT

target id: NTTN90_mRNA_35271_cds

448 5'-GCTTTAACTGCAGCGGCCAATCTCAAAGGCT-3' 478 cleavage site: 463

|||||||||||||||||||||

3'-TTGACGTCGCCGGTTAGAGTT-5'

DysRNA_arrange_TPM 34632315_22 TAATTTGGACGAGATTCGGTCA

target id: NTTN90_mRNA_35271_cds

520 5'-ATTGTTGACCGAATCTCGTCCAAATTATGCAA-3' 551 cleavage site: 536

||||||||||||||||||||||

3'-ACTGGCTTAGAGCAGGTTTAAT-5'

DysRNA_arrange_TPM 43963689_22 TTTTAGCCAGAGTTGTTTTCCC

target id: NTTN90_mRNA_42253_cds

680 5'-GCATAGGGAAAACAACTCTGGC-AAAAGCTAT-3' 710 cleavage site: 695

||||||||||||||||| ||||

3'-CCCTTTTGTTGAGACCGATTTT-5'

DysRNA_arrange_TPM 20072557_21 ATTGGTCAAGGAAGTTTTTCT

target id: NTTN90_mRNA_42253_cds

796 5'-CTACAAGAGAAACTTCTTCGACAAATTCTCA-3' 826 cleavage site: 811

|||o|||||||o| ||| |||

3'-TCTTTTTGAAGGAACTGGTTA-5'

DysRNA_arrange_TPM 42993390_21 TTTAGCCAGAGTTGTTTTCCC

target id: NTTN90_mRNA_42253_cds

680 5'-GCATAGGGAAAACAACTCTGGC-AAAAGCTA-3' 709 cleavage site: 694

||||||||||||||||| |||

3'-CCCTTTTGTTGAGACCGATTT-5'

DysRNA_arrange_TPM 43964061_21 TTTTAGCCAGAGTTGTTTTCC

target id: NTTN90_mRNA_42253_cds

681 5'-CATAGGGAAAACAACTCTGGC-AAAAGCTAT-3' 710 cleavage site: 695

|||||||||||||||| ||||

3'-CCTTTTGTTGAGACCGATTTT-5'

DysRNA_arrange_TPM 32487580_22 CCTTTCGTGCTCAAGTTCTCTC

target id: NTTN90_mRNA_42253_cds

1641 5'-AAAGGGAGTGAACTTGAGCACCAAAGCATTTG-3' 1672 cleavage site: 1657

||| |||||||||||| ||||

3'-CTCTCTTGAACTCGTGCTTTCC-5'

DysRNA_arrange_TPM 40391668_20 TTAATATTTCAGCTCGAACT

target id: NTTN90_mRNA_42253_cds

1369 5'-CATGAAG-TT-A-CTGAAATATTAAATGCA-3' 1395 cleavage site: 1380

|| |o | ||||||||||||

3'-TCAAGCTCGACTTTATAATT-5'

DysRNA_arrange_TPM 43963689_22 TTTTAGCCAGAGTTGTTTTCCC

target id: NTTN90_mRNA_86966_cds

671 5'-GCATAGGGAAAACAACTCTGGC-AAAAGCTAT-3' 701 cleavage site: 686

||||||||||||||||| ||||

3'-CCCTTTTGTTGAGACCGATTTT-5'

DysRNA_arrange_TPM 42993390_21 TTTAGCCAGAGTTGTTTTCCC

target id: NTTN90_mRNA_86966_cds

671 5'-GCATAGGGAAAACAACTCTGGC-AAAAGCTA-3' 700 cleavage site: 685

||||||||||||||||| |||

3'-CCCTTTTGTTGAGACCGATTT-5'

DysRNA_arrange_TPM 43964061_21 TTTTAGCCAGAGTTGTTTTCC

target id: NTTN90_mRNA_86966_cds

672 5'-CATAGGGAAAACAACTCTGGC-AAAAGCTAT-3' 701 cleavage site: 686

|||||||||||||||| ||||

3'-CCTTTTGTTGAGACCGATTTT-5'

DysRNA_arrange_TPM 40391668_20 TTAATATTTCAGCTCGAACT

target id: NTTN90_mRNA_86966_cds

1360 5'-CATGAAG-TT-A-CTGAAATATTAAATGCA-3' 1386 cleavage site: 1371

|| |o | ||||||||||||

3'-TCAAGCTCGACTTTATAATT-5'

DysRNA_arrange_TPM 36654165_22 TGACTGCACATTGAGACTCTTC

target id: NTTN90_mRNA_69999_cds

200 5'-CTATCGAAGAGTCTCAATGTGCAGTCATCATT-3' 231 cleavage site: 216

||||||||||||||||||||||

3'-CTTCTCAGAGTTACACGTCAGT-5'

DysRNA_arrange_TPM 36564529_22 TGACAGCACATTGAGACTCTTC

target id: NTTN90_mRNA_69999_cds

200 5'-CTATCGAAGAGTCTCAATGTGCAGTCATCATT-3' 231 cleavage site: 216

||||||||||||||||| ||||

3'-CTTCTCAGAGTTACACGACAGT-5'

DysRNA_arrange_TPM 43206041_21 TTTGAGATTGGCCGCTGCAGT

target id: NTTN90_mRNA_69999_cds

446 5'-CTTTAACTGCAGCGGCCAATCTCAAAGGCTG-3' 476 cleavage site: 461

|||||||||||||||||||||

3'-TGACGTCGCCGGTTAGAGTTT-5'

DysRNA_arrange_TPM 35198903_22 TACAGGTGACTTGTAAATGTTT

target id: NTTN90_mRNA_69999_cds

84 5'-TCGGAAAACATTTACGAGTCACCTGTACGAAA-3' 115 cleavage site: 100

||||||||||o|||||||||||

3'-TTTGTAAATGTTCAGTGGACAT-5'

DysRNA_arrange_TPM 41151740_21 TTGAGATTGGCCGCTGCAGTT

target id: NTTN90_mRNA_69999_cds

445 5'-GCTTTAACTGCAGCGGCCAATCTCAAAGGCT-3' 475 cleavage site: 460

|||||||||||||||||||||

3'-TTGACGTCGCCGGTTAGAGTT-5'

DysRNA_arrange_TPM 34632662_21 TAATTTGGACGAGATTCGGTC

target id: NTK326_mRNA_39595_cds

518 5'-TTGTTGATCAAATCTCGTCCAAATTATGCAA-3' 548 cleavage site: 533

||o| ||||||||||||||||

3'-CTGGCTTAGAGCAGGTTTAAT-5'

DysRNA_arrange_TPM 43206041_21 TTTGAGATTGGCCGCTGCAGT

target id: NTK326_mRNA_39595_cds

446 5'-CTTTAACTGCAGCGGCCAATCTCAAAGGTTG-3' 476 cleavage site: 461

|||||||||||||||||||||

3'-TGACGTCGCCGGTTAGAGTTT-5'

DysRNA_arrange_TPM 34633256_21 TAATTTGGACGAGATTTGGTC

target id: NTK326_mRNA_39595_cds

518 5'-TTGTTGATCAAATCTCGTCCAAATTATGCAA-3' 548 cleavage site: 533

||o||||||||||||||||||

3'-CTGGTTTAGAGCAGGTTTAAT-5'

DysRNA_arrange_TPM 34632839_21 TAATTTGGACGAGATTTGATC

target id: NTK326_mRNA_39595_cds

518 5'-TTGTTGATCAAATCTCGTCCAAATTATGCAA-3' 548 cleavage site: 533

|||||||||||||||||||||

3'-CTAGTTTAGAGCAGGTTTAAT-5'

DysRNA_arrange_TPM 35198903_22 TACAGGTGACTTGTAAATGTTT

target id: NTK326_mRNA_39595_cds

84 5'-TCGGAAAACATTTACGAGTCACCTGTACGAAA-3' 115 cleavage site: 100

||||||||||o|||||||||||

3'-TTTGTAAATGTTCAGTGGACAT-5'

DysRNA_arrange_TPM 41151740_21 TTGAGATTGGCCGCTGCAGTT

target id: NTK326_mRNA_39595_cds

445 5'-GCTTTAACTGCAGCGGCCAATCTCAAAGGTT-3' 475 cleavage site: 460

|||||||||||||||||||||

3'-TTGACGTCGCCGGTTAGAGTT-5'

DysRNA_arrange_TPM 34632315_22 TAATTTGGACGAGATTCGGTCA

target id: NTK326_mRNA_39595_cds

517 5'-ATTGTTGATCAAATCTCGTCCAAATTATGCAA-3' 548 cleavage site: 533

|||o| ||||||||||||||||

3'-ACTGGCTTAGAGCAGGTTTAAT-5'

DysRNA_arrange_TPM 40498063_21 TTAGATACTGAAACAACAGGT

target id: NTBX_mRNA_108279_cds

2200 5'-CCATCATCTATT-TTTCAGTATC-AAACTCA-3' 2228 cleavage site: 2213

|o|| || |||||||||| ||

3'-TGGACAACAAAGTCATAGATT-5'

DysRNA_arrange_TPM 34632662_21 TAATTTGGACGAGATTCGGTC

target id: NTK326_mRNA_43111_cds

521 5'-TTGTTGACCGAATCTCGTCCAAATTATGCAA-3' 551 cleavage site: 536

|||||||||||||||||||||

3'-CTGGCTTAGAGCAGGTTTAAT-5'

DysRNA_arrange_TPM 43206041_21 TTTGAGATTGGCCGCTGCAGT

target id: NTK326_mRNA_43111_cds

449 5'-CTTTAACTGCAGCGGCCAATCTCAAAGGCTG-3' 479 cleavage site: 464

|||||||||||||||||||||

3'-TGACGTCGCCGGTTAGAGTTT-5'

DysRNA_arrange_TPM 34633256_21 TAATTTGGACGAGATTTGGTC

target id: NTK326_mRNA_43111_cds

521 5'-TTGTTGACCGAATCTCGTCCAAATTATGCAA-3' 551 cleavage site: 536

||||o||||||||||||||||

3'-CTGGTTTAGAGCAGGTTTAAT-5'

DysRNA_arrange_TPM 35716976_20 TATATCGAGTCCTGTTAATT

target id: NTK326_mRNA_43111_cds

3319 5'-ACTTGGATAAGGAGGACCCGATATAACAAT-3' 3348 cleavage site: 3333

o|| |o ||||| |||||||

3'-TTAATTGTCCTGAGCTATAT-5'

DysRNA_arrange_TPM 34632839_21 TAATTTGGACGAGATTTGATC

target id: NTK326_mRNA_43111_cds

521 5'-TTGTTGACCGAATCTCGTCCAAATTATGCAA-3' 551 cleavage site: 536

|| |o||||||||||||||||

3'-CTAGTTTAGAGCAGGTTTAAT-5'

DysRNA_arrange_TPM 35198903_22 TACAGGTGACTTGTAAATGTTT

target id: NTK326_mRNA_43111_cds

87 5'-TCGGAAAACATTTACGAGTCACCTGTACGAAA-3' 118 cleavage site: 103

||||||||||o|||||||||||

3'-TTTGTAAATGTTCAGTGGACAT-5'

DysRNA_arrange_TPM 41151740_21 TTGAGATTGGCCGCTGCAGTT

target id: NTK326_mRNA_43111_cds

448 5'-GCTTTAACTGCAGCGGCCAATCTCAAAGGCT-3' 478 cleavage site: 463

|||||||||||||||||||||

3'-TTGACGTCGCCGGTTAGAGTT-5'

DysRNA_arrange_TPM 34632315_22 TAATTTGGACGAGATTCGGTCA

target id: NTK326_mRNA_43111_cds

520 5'-ATTGTTGACCGAATCTCGTCCAAATTATGCAA-3' 551 cleavage site: 536

||||||||||||||||||||||

3'-ACTGGCTTAGAGCAGGTTTAAT-5'

DysRNA_arrange_TPM 39288440_22 TCCGGGAAATGAAAATAGCTGC

target id: NTTN90_mRNA_90508_cds

3000 5'-TATACACAGC-ATTTTCA-TTCCTGGAGGCAA-3' 3029 cleavage site: 3014

|||| ||||||| ||||o|||

3'-CGTCGATAAAAGTAAAGGGCCT-5'

DysRNA_arrange_TPM 43963689_22 TTTTAGCCAGAGTTGTTTTCCC

target id: NTTN90_mRNA_72762_cds

680 5'-GCATAGGGAAAACAACTCTGGC-AAAAGCTAT-3' 710 cleavage site: 695

||||||||||||||||| ||||

3'-CCCTTTTGTTGAGACCGATTTT-5'

DysRNA_arrange_TPM 42993390_21 TTTAGCCAGAGTTGTTTTCCC

target id: NTTN90_mRNA_72762_cds

680 5'-GCATAGGGAAAACAACTCTGGC-AAAAGCTA-3' 709 cleavage site: 694

||||||||||||||||| |||

3'-CCCTTTTGTTGAGACCGATTT-5'

DysRNA_arrange_TPM 43964061_21 TTTTAGCCAGAGTTGTTTTCC

target id: NTTN90_mRNA_72762_cds

681 5'-CATAGGGAAAACAACTCTGGC-AAAAGCTAT-3' 710 cleavage site: 695

|||||||||||||||| ||||

3'-CCTTTTGTTGAGACCGATTTT-5'

DysRNA_arrange_TPM 40391668_20 TTAATATTTCAGCTCGAACT

target id: NTTN90_mRNA_72762_cds

1369 5'-CATGAAG-TT-A-CTGAAATATTAAATGCA-3' 1395 cleavage site: 1380

|| |o | ||||||||||||

3'-TCAAGCTCGACTTTATAATT-5'

DysRNA_arrange_TPM 43963689_22 TTTTAGCCAGAGTTGTTTTCCC

target id: NTBX_mRNA_95914_cds

671 5'-GCATAGGGAAAACAACTCTGGC-AAAAGCTAT-3' 701 cleavage site: 686

||||||||||||||||| ||||

3'-CCCTTTTGTTGAGACCGATTTT-5'

DysRNA_arrange_TPM 42993390_21 TTTAGCCAGAGTTGTTTTCCC

target id: NTBX_mRNA_95914_cds

671 5'-GCATAGGGAAAACAACTCTGGC-AAAAGCTA-3' 700 cleavage site: 685

||||||||||||||||| |||

3'-CCCTTTTGTTGAGACCGATTT-5'

DysRNA_arrange_TPM 43964061_21 TTTTAGCCAGAGTTGTTTTCC

target id: NTBX_mRNA_95914_cds

672 5'-CATAGGGAAAACAACTCTGGC-AAAAGCTAT-3' 701 cleavage site: 686

|||||||||||||||| ||||

3'-CCTTTTGTTGAGACCGATTTT-5'

DysRNA_arrange_TPM 40391668_20 TTAATATTTCAGCTCGAACT

target id: NTBX_mRNA_95914_cds

1360 5'-CATGAAG-TT-A-CTGAAATATTAAATGCA-3' 1386 cleavage site: 1371

|| |o | ||||||||||||

3'-TCAAGCTCGACTTTATAATT-5'

DysRNA_arrange_TPM 43499236_21 TTTGTCATGATAAATAAAGTC

target id: NTTN90_mRNA_42140_cds

950 5'-CCAAGGACTTTATTTATCATGACAAATTGTT-3' 980 cleavage site: 965

|||||||||||||||||||||

3'-CTGAAATAAATAGTACTGTTT-5'

DysRNA_arrange_TPM 43498503_21 TTTGTCATGATGAATAAAGTC

target id: NTTN90_mRNA_42140_cds

950 5'-CCAAGGACTTTATTTATCATGACAAATTGTT-3' 980 cleavage site: 965

|||||||||o|||||||||||

3'-CTGAAATAAGTAGTACTGTTT-5'

DysRNA_arrange_TPM 39918426_21 TCTCGTTGAAATCTAGCTACC

target id: NTTN90_mRNA_42140_cds

2548 5'-AGCTTGGT-GATCGTTTTCAACGAGAGAGGT-3' 2577 cleavage site: 2562

||| | | | |||||||||||

3'-CCATCGATCTAAAGTTGCTCT-5'

DysRNA_arrange_TPM 41905826_21 TTGTCTCACGTCATTCCATGC

target id: NTBX_mRNA_24678_cds

611 5'-GTAAAGCATGGGATGACGTGAGACTATGCTT-3' 641 cleavage site: 626

||||||o||||||||||||

3'-CGTACCTTACTGCACTCTGTT-5'

DysRNA_arrange_TPM 37756134_21 TGTCTCACGTCATTCCATGCA

target id: NTBX_mRNA_24678_cds

610 5'-AGTAAAGCATGGGATGACGTGAGACTATGCT-3' 640 cleavage site: 625

||||||o||||||||||||

3'-ACGTACCTTACTGCACTCTGT-5'

DysRNA_arrange_TPM 38706727_21 TCGACTCCCATTGTTTTGACT

target id: NTBX_mRNA_24678_cds

641 5'-TTCCAAGTGAAAACAATGGGAGTCGAATACT-3' 671 cleavage site: 656

||| |||||||||||||||||

3'-TCAGTTTTGTTACCCTCAGCT-5'

DysRNA_arrange_TPM 30234614_21 CGACTCCCATTGTTTTGACTT

target id: NTBX_mRNA_24678_cds

640 5'-TTTCCAAGTGAAAACAATGGGAGTCGAATAC-3' 670 cleavage site: 655

|||| ||||||||||||||||

3'-TTCAGTTTTGTTACCCTCAGC-5'

DysRNA_arrange_TPM 32948576_20 CTGGGAAGTCCTCGTGTTGC

target id: NTTN90_mRNA_102492_cds

3412 5'-CGGCTACAACATGTGGACTTCTCAGGTGTC-3' 3441 cleavage site: 3426

|||||o| |||||||o|||

3'-CGTTGTGCTCCTGAAGGGTC-5'

DysRNA_arrange_TPM 38030093_21 TCAACATCATCAGACATATGG

target id: NTTN90_mRNA_124334_cds

2241 5'-TTTGCCTATATGTCTGAAGAGGTTGACTTTA-3' 2271 cleavage site: 2256

|o|||||||||| || |||||

3'-GGTATACAGACTACTACAACT-5'

DysRNA_arrange_TPM 32948576_20 CTGGGAAGTCCTCGTGTTGC

target id: NTTN90_mRNA_102488_cds

3412 5'-CGGCTACAACATGTGGACTTCTCAGGTGTC-3' 3441 cleavage site: 3426

|||||o| |||||||o|||

3'-CGTTGTGCTCCTGAAGGGTC-5'

DysRNA_arrange_TPM 41911182_21 TTGTCTTTCCGATACCTCCCA

target id: NTK326_mRNA_115279_cds

621 5'-TGGAATGGCAGGTGTTGGCAAGACAACACTT-3' 651 cleavage site: 636

||| ||||o|o|| |||||||

3'-ACCCTCCATAGCCTTTCTGTT-5'

DysRNA_arrange_TPM 22042883_24 GAACTGTAGAGTTTGATCCTGGCT

target id: NTTN90_mRNA_46764_cds

66 5'-AAGGGAG-CA-GATCAAACTCTGTAGTTACTTTT-3' 97 cleavage site: 82

|| || |||||||||||oo||||

3'-TCGGTCCTAGTTTGAGATGTCAAG-5'

DysRNA_arrange_TPM 22043376_20 GAACTGTAGAGTTTGATCCT

target id: NTTN90_mRNA_46764_cds

68 5'-GGGAGCAGATCAAACTCTGTAGTTACTTTT-3' 97 cleavage site: 82

|||||||||||oo||||

3'-TCCTAGTTTGAGATGTCAAG-5'

DysRNA_arrange_TPM 22746892_21 GAGGTAGTGACAATAAATAAC

target id: NTBX_mRNA_91223_cds

2744 5'-TACGTCTTATTGATTGTCACTCTCTTACCTC-3' 2774 cleavage site: 2759

||||| ||||||||| o||o

3'-CAATAAATAACAGTGATGGAG-5'

DysRNA_arrange_TPM 02841428_21 AAGAACTTTGAAGAGAGAGTT

target id: NTBX_mRNA_91223_cds

3577 5'-CGCCTAAC-CTCACTTCAAAG-TCTACGGAT-3' 3605 cleavage site: 3590

||| ||| |||||||| |||

3'-TTGAGAGAGAAGTTTCAAGAA-5'

DysRNA_arrange_TPM 02842108_20 AAGAACTTTGAAGAGAGAGT

target id: NTBX_mRNA_91223_cds

3578 5'-GCCTAAC-CTCACTTCAAAG-TCTACGGAT-3' 3605 cleavage site: 3590

|| ||| |||||||| |||

3'-TGAGAGAGAAGTTTCAAGAA-5'

DysRNA_arrange_TPM 10032960_20 AGGTAGTGACAATAAATAAC

target id: NTBX_mRNA_91223_cds

2744 5'-TACGTCTTATTGATTGTCACTCTCTTACCT-3' 2773 cleavage site: 2758

||||| ||||||||| o||

3'-CAATAAATAACAGTGATGGA-5'

DysRNA_arrange_TPM 41905826_21 TTGTCTCACGTCATTCCATGC

target id: NTBX_mRNA_24683_cds

797 5'-GTAAAGCATGGGATGATGTGAGACAATGCTT-3' 827 cleavage site: 812

||||||o||||o|||||||||

3'-CGTACCTTACTGCACTCTGTT-5'

DysRNA_arrange_TPM 37756134_21 TGTCTCACGTCATTCCATGCA

target id: NTBX_mRNA_24683_cds

796 5'-AGTAAAGCATGGGATGATGTGAGACAATGCT-3' 826 cleavage site: 811

||||||o||||o||||||||

3'-ACGTACCTTACTGCACTCTGT-5'

DysRNA_arrange_TPM 32948576_20 CTGGGAAGTCCTCGTGTTGC

target id: NTTN90_mRNA_102491_cds

3412 5'-CGGCTACAACATGTGGACTTCTCAGGTGTC-3' 3441 cleavage site: 3426

|||||o| |||||||o|||

3'-CGTTGTGCTCCTGAAGGGTC-5'

DysRNA_arrange_TPM 31384081_21 CCACGATCCACTGAGATTCAG

target id: NTBX_mRNA_132524_cds

1643 5'-GAGTTCTGAATCTGAGTGGAAC-TGGTATTA-3' 1672 cleavage site: 1657

|||||||| |||||| | |||

3'-GACTTAGAGTCACCTAGCACC-5'

DysRNA_arrange_TPM 31383804_22 CCACGATCCACTGAGATTCAGC

target id: NTBX_mRNA_132524_cds

1642 5'-AGAGTTCTGAATCTGAGTGGAAC-TGGTATTA-3' 1672 cleavage site: 1657

|||||||| |||||| | |||

3'-CGACTTAGAGTCACCTAGCACC-5'

DysRNA_arrange_TPM 32948576_20 CTGGGAAGTCCTCGTGTTGC

target id: NTK326_mRNA_117088_cds

3412 5'-CGGCTACAACATGTGGACTTCTCAGGTGTC-3' 3441 cleavage site: 3426

|||||o| |||||||o|||

3'-CGTTGTGCTCCTGAAGGGTC-5'

DysRNA_arrange_TPM 40742547_22 TTACCTGAACCTGGCATACCAA

target id: NTBX_mRNA_20748_cds

1863 5'-GATCACTGGTATGCCGGGTTCGGGTAAAACTA-3' 1894 cleavage site: 1879

|||||||||o|||||o|||||

3'-AACCATACGGTCCAAGTCCATT-5'

DysRNA_arrange_TPM 40741736_21 TTACCTGAACCTGGCATACCA

target id: NTBX_mRNA_20748_cds

1864 5'-ATCACTGGTATGCCGGGTTCGGGTAAAACTA-3' 1894 cleavage site: 1879

|||||||||o|||||o|||||

3'-ACCATACGGTCCAAGTCCATT-5'

DysRNA_arrange_TPM 40740236_21 TTACCTGAACCTGGCATACCT

target id: NTBX_mRNA_20748_cds

1864 5'-ATCACTGGTATGCCGGGTTCGGGTAAAACTA-3' 1894 cleavage site: 1879

||||||||o|||||o|||||

3'-TCCATACGGTCCAAGTCCATT-5'

DysRNA_arrange_TPM 40741593_20 TTACCTGAACCTGGCATACC

target id: NTBX_mRNA_20748_cds

1865 5'-TCACTGGTATGCCGGGTTCGGGTAAAACTA-3' 1894 cleavage site: 1879

||||||||o|||||o|||||

3'-CCATACGGTCCAAGTCCATT-5'

DysRNA_arrange_TPM 40742348_23 TTACCTGAACCTGGCATACCAAA

target id: NTBX_mRNA_20748_cds

1862 5'-CGATCACTGGTATGCCGGGTTCGGGTAAAACTA-3' 1894 cleavage site: 1879

|||||||||o|||||o|||||

3'-AAACCATACGGTCCAAGTCCATT-5'

DysRNA_arrange_TPM 40740693_23 TTACCTGAACCTGGCATACCAAT

target id: NTBX_mRNA_20748_cds

1862 5'-CGATCACTGGTATGCCGGGTTCGGGTAAAACTA-3' 1894 cleavage site: 1879

|||||||||o|||||o|||||

3'-TAACCATACGGTCCAAGTCCATT-5'

DysRNA_arrange_TPM 35450798_21 TACCTGAACCTGGCATACCAA

target id: NTBX_mRNA_20748_cds

1863 5'-GATCACTGGTATGCCGGGTTCGGGTAAAACT-3' 1893 cleavage site: 1878

|||||||||o|||||o||||

3'-AACCATACGGTCCAAGTCCAT-5'

DysRNA_arrange_TPM 40740134_22 TTACCTGAACCTGGCATACCAT

target id: NTBX_mRNA_20748_cds

1863 5'-GATCACTGGTATGCCGGGTTCGGGTAAAACTA-3' 1894 cleavage site: 1879

|||||||||o|||||o|||||

3'-TACCATACGGTCCAAGTCCATT-5'

DysRNA_arrange_TPM 40741540_22 TTACCTGAACCTGGCATACCTT

target id: NTBX_mRNA_20748_cds

1863 5'-GATCACTGGTATGCCGGGTTCGGGTAAAACTA-3' 1894 cleavage site: 1879

||||||||o|||||o|||||

3'-TTCCATACGGTCCAAGTCCATT-5'

DysRNA_arrange_TPM 40742481_23 TTACCTGAACCTGGCATACCATT

target id: NTBX_mRNA_20748_cds

1862 5'-CGATCACTGGTATGCCGGGTTCGGGTAAAACTA-3' 1894 cleavage site: 1879

|||||||||o|||||o|||||

3'-TTACCATACGGTCCAAGTCCATT-5'

DysRNA_arrange_TPM 40741489_20 TTACCTGAACCTGGCATACT

target id: NTBX_mRNA_20748_cds

1865 5'-TCACTGGTATGCCGGGTTCGGGTAAAACTA-3' 1894 cleavage site: 1879

o|||||||o|||||o|||||

3'-TCATACGGTCCAAGTCCATT-5'

DysRNA_arrange_TPM 40742536_21 TTACCTGAACCTGGCATACCG

target id: NTBX_mRNA_20748_cds

1864 5'-ATCACTGGTATGCCGGGTTCGGGTAAAACTA-3' 1894 cleavage site: 1879

o||||||||o|||||o|||||

3'-GCCATACGGTCCAAGTCCATT-5'

DysRNA_arrange_TPM 40742033_21 TTACCTGAACCTGGCATACCC

target id: NTBX_mRNA_20748_cds

1864 5'-ATCACTGGTATGCCGGGTTCGGGTAAAACTA-3' 1894 cleavage site: 1879

||||||||o|||||o|||||

3'-CCCATACGGTCCAAGTCCATT-5'

DysRNA_arrange_TPM 40739408_23 TTACCTGAACCTGGCATACCAAG

target id: NTBX_mRNA_20748_cds

1862 5'-CGATCACTGGTATGCCGGGTTCGGGTAAAACTA-3' 1894 cleavage site: 1879

|||||||||o|||||o|||||

3'-GAACCATACGGTCCAAGTCCATT-5'

DysRNA_arrange_TPM 43050316_22 TTTACCTGAACCTGGCATACCA

target id: NTBX_mRNA_20748_cds

1864 5'-ATCACTGGTATGCCGGGTTCGGGTAAAACTAC-3' 1895 cleavage site: 1880

|||||||||o|||||o||||||

3'-ACCATACGGTCCAAGTCCATTT-5'

DysRNA_arrange_TPM 40741174_22 TTACCTGAACCTGGCATACCAG

target id: NTBX_mRNA_20748_cds

1863 5'-GATCACTGGTATGCCGGGTTCGGGTAAAACTA-3' 1894 cleavage site: 1879

||||||||||o|||||o|||||

3'-GACCATACGGTCCAAGTCCATT-5'

DysRNA_arrange_TPM 43050143_24 TTTACCTGAACCTGGCATACCAAC

target id: NTBX_mRNA_20748_cds

1862 5'-CGATCACTGGTATGCCGGGTTCGGGTAAAACTAC-3' 1895 cleavage site: 1880

|||||||||o|||||o||||||

3'-CAACCATACGGTCCAAGTCCATTT-5'

DysRNA_arrange_TPM 13424659_24 ACGTGCCCAGAGATCTTTATAGAA

target id: NTBX_mRNA_20748_cds

1529 5'-AAGAATTGTATAAAGATCTCTGGGCACGTACTTT-3' 1562 cleavage site: 1547

|| |||||||||||||||||||||

3'-AAGATATTTCTAGAGACCCGTGCA-5'

DysRNA_arrange_TPM 35450403_20 TACCTGAACCTGGCATACCA

target id: NTBX_mRNA_20748_cds

1864 5'-ATCACTGGTATGCCGGGTTCGGGTAAAACT-3' 1893 cleavage site: 1878

|||||||||o|||||o||||

3'-ACCATACGGTCCAAGTCCAT-5'

DysRNA_arrange_TPM 40739753_22 TTACCTGAACCTGGCATACCTA

target id: NTBX_mRNA_20748_cds

1863 5'-GATCACTGGTATGCCGGGTTCGGGTAAAACTA-3' 1894 cleavage site: 1879

||||||||o|||||o|||||

3'-ATCCATACGGTCCAAGTCCATT-5'

DysRNA_arrange_TPM 40742347_21 TTACCTGAACCTGGCATACTT

target id: NTBX_mRNA_20748_cds

1864 5'-ATCACTGGTATGCCGGGTTCGGGTAAAACTA-3' 1894 cleavage site: 1879

o|||||||o|||||o|||||

3'-TTCATACGGTCCAAGTCCATT-5'

DysRNA_arrange_TPM 13424647_24 ACGTGCCCAGAGATCTTTATACAA

target id: NTBX_mRNA_20748_cds

1529 5'-AAGAATTGTATAAAGATCTCTGGGCACGTACTTT-3' 1562 cleavage site: 1547

||||||||||||||||||||||||

3'-AACATATTTCTAGAGACCCGTGCA-5'

DysRNA_arrange_TPM 40739960_22 TTACCTGAGCCTGGCATACCAA

target id: NTBX_mRNA_20748_cds

1863 5'-GATCACTGGTATGCCGGGTTCGGGTAAAACTA-3' 1894 cleavage site: 1879

|||||||||o||o||o|||||

3'-AACCATACGGTCCGAGTCCATT-5'

DysRNA_arrange_TPM 40741291_23 TTACCTGAACCTGGCATACCATC

target id: NTBX_mRNA_20748_cds

1862 5'-CGATCACTGGTATGCCGGGTTCGGGTAAAACTA-3' 1894 cleavage site: 1879

|||||||||o|||||o|||||

3'-CTACCATACGGTCCAAGTCCATT-5'

DysRNA_arrange_TPM 40741578_22 TTACCTGAACCAGGCATACCAA

target id: NTBX_mRNA_20748_cds

1863 5'-GATCACTGGTATGCCGGGTTCGGGTAAAACTA-3' 1894 cleavage site: 1879

||||||||| |||||o|||||

3'-AACCATACGGACCAAGTCCATT-5'

DysRNA_arrange_TPM 40739574_21 TTACCTGAACCTGGCATACTA

target id: NTBX_mRNA_20748_cds

1864 5'-ATCACTGGTATGCCGGGTTCGGGTAAAACTA-3' 1894 cleavage site: 1879

|o|||||||o|||||o|||||

3'-ATCATACGGTCCAAGTCCATT-5'

DysRNA_arrange_TPM 40665598_22 TTACATGAACCTGGCATACCAA

target id: NTBX_mRNA_20748_cds

1863 5'-GATCACTGGTATGCCGGGTTCGGGTAAAACTA-3' 1894 cleavage site: 1879

|||||||||o|||||o ||||

3'-AACCATACGGTCCAAGTACATT-5'

DysRNA_arrange_TPM 43050825_21 TTTACCTGAACCTGGCATACC

target id: NTBX_mRNA_20748_cds

1865 5'-TCACTGGTATGCCGGGTTCGGGTAAAACTAC-3' 1895 cleavage site: 1880

||||||||o|||||o||||||

3'-CCATACGGTCCAAGTCCATTT-5'

DysRNA_arrange_TPM 40741435_21 TTACCTGAACCTGACATACCA

target id: NTBX_mRNA_20748_cds

1864 5'-ATCACTGGTATGCCGGGTTCGGGTAAAACTA-3' 1894 cleavage site: 1879

||||||| |o|||||o|||||

3'-ACCATACAGTCCAAGTCCATT-5'

DysRNA_arrange_TPM 40742312_23 TTACCTGAACCTGGCATACCAAC

target id: NTBX_mRNA_20748_cds

1862 5'-CGATCACTGGTATGCCGGGTTCGGGTAAAACTA-3' 1894 cleavage site: 1879

|||||||||o|||||o|||||

3'-CAACCATACGGTCCAAGTCCATT-5'

DysRNA_arrange_TPM 40739464_22 TTACCTGAACCTGGCATACCAC

target id: NTBX_mRNA_20748_cds

1863 5'-GATCACTGGTATGCCGGGTTCGGGTAAAACTA-3' 1894 cleavage site: 1879

|||||||||o|||||o|||||

3'-CACCATACGGTCCAAGTCCATT-5'

DysRNA_arrange_TPM 40740425_22 TTACCTGAACCTGACATACCAA

target id: NTBX_mRNA_20748_cds

1863 5'-GATCACTGGTATGCCGGGTTCGGGTAAAACTA-3' 1894 cleavage site: 1879

||||||| |o|||||o|||||

3'-AACCATACAGTCCAAGTCCATT-5'

DysRNA_arrange_TPM 35450164_20 TACCTGAACCTGGCATACCT

target id: NTBX_mRNA_20748_cds

1864 5'-ATCACTGGTATGCCGGGTTCGGGTAAAACT-3' 1893 cleavage site: 1878

||||||||o|||||o||||

3'-TCCATACGGTCCAAGTCCAT-5'

DysRNA_arrange_TPM 43050821_23 TTTACCTGAACCTGGCATACCAA

target id: NTBX_mRNA_20748_cds

1863 5'-GATCACTGGTATGCCGGGTTCGGGTAAAACTAC-3' 1895 cleavage site: 1880

|||||||||o|||||o||||||

3'-AACCATACGGTCCAAGTCCATTT-5'

DysRNA_arrange_TPM 40741495_22 TTACCTGAACCTGGCATACCTC

target id: NTBX_mRNA_20748_cds

1863 5'-GATCACTGGTATGCCGGGTTCGGGTAAAACTA-3' 1894 cleavage site: 1879

||||||||o|||||o|||||

3'-CTCCATACGGTCCAAGTCCATT-5'

DysRNA_arrange_TPM 43983812_21 TTTTACCTGAACCTGGCATAC

target id: NTBX_mRNA_20748_cds

1866 5'-CACTGGTATGCCGGGTTCGGGTAAAACTACT-3' 1896 cleavage site: 1881

|||||||o|||||o|||||||

3'-CATACGGTCCAAGTCCATTTT-5'

DysRNA_arrange_TPM 40739986_22 TTACCTGAACCTAGCATACCAA

target id: NTBX_mRNA_20748_cds

1863 5'-GATCACTGGTATGCCGGGTTCGGGTAAAACTA-3' 1894 cleavage site: 1879

|||||||| o|||||o|||||

3'-AACCATACGATCCAAGTCCATT-5'

DysRNA_arrange_TPM 40741138_22 TTACCTGAACCTGGCATACTTT

target id: NTBX_mRNA_20748_cds

1863 5'-GATCACTGGTATGCCGGGTTCGGGTAAAACTA-3' 1894 cleavage site: 1879

o|||||||o|||||o|||||

3'-TTTCATACGGTCCAAGTCCATT-5'

DysRNA_arrange_TPM 40739646_22 TTACCTGAACCGGGCATACCAA

target id: NTBX_mRNA_20748_cds

1863 5'-GATCACTGGTATGCCGGGTTCGGGTAAAACTA-3' 1894 cleavage site: 1879

||||||||| |||||o|||||

3'-AACCATACGGGCCAAGTCCATT-5'

DysRNA_arrange_TPM 40740203_21 TTACCTGAACCTGGCATACTC

target id: NTBX_mRNA_20748_cds

1864 5'-ATCACTGGTATGCCGGGTTCGGGTAAAACTA-3' 1894 cleavage site: 1879

o|||||||o|||||o|||||

3'-CTCATACGGTCCAAGTCCATT-5'

DysRNA_arrange_TPM 40740924_20 TTACCTGAACCTGGCATACA

target id: NTBX_mRNA_20748_cds

1865 5'-TCACTGGTATGCCGGGTTCGGGTAAAACTA-3' 1894 cleavage site: 1879

|||||||o|||||o|||||

3'-ACATACGGTCCAAGTCCATT-5'

DysRNA_arrange_TPM 40738063_22 TTACCTAAACCTGGCATACCAA

target id: NTBX_mRNA_20748_cds

1863 5'-GATCACTGGTATGCCGGGTTCGGGTAAAACTA-3' 1894 cleavage site: 1879

|||||||||o|||| o|||||

3'-AACCATACGGTCCAAATCCATT-5'

DysRNA_arrange_TPM 40745342_22 TTACCTCAACCTGGCATACCAA

target id: NTBX_mRNA_20748_cds

1863 5'-GATCACTGGTATGCCGGGTTCGGGTAAAACTA-3' 1894 cleavage site: 1879

|||||||||o|||| o|||||

3'-AACCATACGGTCCAACTCCATT-5'

DysRNA_arrange_TPM 40747677_21 TTACCTTAACCTGGCATACCA

target id: NTBX_mRNA_20748_cds

1864 5'-ATCACTGGTATGCCGGGTTCGGGTAAAACTA-3' 1894 cleavage site: 1879

|||||||||o|||| o|||||

3'-ACCATACGGTCCAATTCCATT-5'

DysRNA_arrange_TPM 40737853_21 TTACCTAAACCTGGCATACCA

target id: NTBX_mRNA_20748_cds

1864 5'-ATCACTGGTATGCCGGGTTCGGGTAAAACTA-3' 1894 cleavage site: 1879

|||||||||o|||| o|||||

3'-ACCATACGGTCCAAATCCATT-5'

DysRNA_arrange_TPM 26914223_21 GTACCTGAACCTGGCATACCT

target id: NTBX_mRNA_20748_cds

1864 5'-ATCACTGGTATGCCGGGTTCGGGTAAAACTA-3' 1894 cleavage site: 1879

||||||||o|||||o||||

3'-TCCATACGGTCCAAGTCCATG-5'

DysRNA_arrange_TPM 26914450_20 GTACCTGAACCTGGCATACC

target id: NTBX_mRNA_20748_cds

1865 5'-TCACTGGTATGCCGGGTTCGGGTAAAACTA-3' 1894 cleavage site: 1879

||||||||o|||||o||||

3'-CCATACGGTCCAAGTCCATG-5'

DysRNA_arrange_TPM 26914545_21 GTACCTGAACCTGGCATACCA

target id: NTBX_mRNA_20748_cds

1864 5'-ATCACTGGTATGCCGGGTTCGGGTAAAACTA-3' 1894 cleavage site: 1879

|||||||||o|||||o||||

3'-ACCATACGGTCCAAGTCCATG-5'

DysRNA_arrange_TPM 40741202_22 TTACCTGAACCTGGCATACTAA

target id: NTBX_mRNA_20748_cds

1863 5'-GATCACTGGTATGCCGGGTTCGGGTAAAACTA-3' 1894 cleavage site: 1879

|o|||||||o|||||o|||||

3'-AATCATACGGTCCAAGTCCATT-5'

DysRNA_arrange_TPM 16439463_22 ATACCTGAACCTGGCATACCAA

target id: NTBX_mRNA_20748_cds

1863 5'-GATCACTGGTATGCCGGGTTCGGGTAAAACTA-3' 1894 cleavage site: 1879

|||||||||o|||||o||||

3'-AACCATACGGTCCAAGTCCATA-5'

DysRNA_arrange_TPM 35451289_22 TACCTGAACCTGGCATACCAAA

target id: NTBX_mRNA_20748_cds

1862 5'-CGATCACTGGTATGCCGGGTTCGGGTAAAACT-3' 1893 cleavage site: 1878

|||||||||o|||||o||||

3'-AAACCATACGGTCCAAGTCCAT-5'

DysRNA_arrange_TPM 40739499_22 TTACCTGAACCTGTCATACCAA

target id: NTBX_mRNA_20748_cds

1863 5'-GATCACTGGTATGCCGGGTTCGGGTAAAACTA-3' 1894 cleavage site: 1879

||||||| |o|||||o|||||

3'-AACCATACTGTCCAAGTCCATT-5'

DysRNA_arrange_TPM 35450589_22 TACCTGAACCTGGCATACCAAT

target id: NTBX_mRNA_20748_cds

1862 5'-CGATCACTGGTATGCCGGGTTCGGGTAAAACT-3' 1893 cleavage site: 1878

|||||||||o|||||o||||

3'-TAACCATACGGTCCAAGTCCAT-5'

DysRNA_arrange_TPM 40717233_22 TTACCGGAACCTGGCATACCAA

target id: NTBX_mRNA_20748_cds

1863 5'-GATCACTGGTATGCCGGGTTCGGGTAAAACTA-3' 1894 cleavage site: 1879

|||||||||o||||| |||||

3'-AACCATACGGTCCAAGGCCATT-5'

DysRNA_arrange_TPM 40747954_22 TTACCTTAACCTGGCATACCAA

target id: NTBX_mRNA_20748_cds

1863 5'-GATCACTGGTATGCCGGGTTCGGGTAAAACTA-3' 1894 cleavage site: 1879

|||||||||o|||| o|||||

3'-AACCATACGGTCCAATTCCATT-5'

DysRNA_arrange_TPM 26914581_22 GTACCTGAACCTGGCATACCAA

target id: NTBX_mRNA_20748_cds

1863 5'-GATCACTGGTATGCCGGGTTCGGGTAAAACTA-3' 1894 cleavage site: 1879

|||||||||o|||||o||||

3'-AACCATACGGTCCAAGTCCATG-5'

DysRNA_arrange_TPM 40742285_22 TTACCTGAACCTGGCATACTTC

target id: NTBX_mRNA_20748_cds

1863 5'-GATCACTGGTATGCCGGGTTCGGGTAAAACTA-3' 1894 cleavage site: 1879

o|||||||o|||||o|||||

3'-CTTCATACGGTCCAAGTCCATT-5'

DysRNA_arrange_TPM 40727231_22 TTACCCGAACCTGGCATACCAA

target id: NTBX_mRNA_20748_cds

1863 5'-GATCACTGGTATGCCGGGTTCGGGTAAAACTA-3' 1894 cleavage site: 1879

|||||||||o|||||||||||

3'-AACCATACGGTCCAAGCCCATT-5'

DysRNA_arrange_TPM 40741969_22 TTACCTGAACCTTGCATACCAA

target id: NTBX_mRNA_20748_cds

1863 5'-GATCACTGGTATGCCGGGTTCGGGTAAAACTA-3' 1894 cleavage site: 1879

|||||||| o|||||o|||||

3'-AACCATACGTTCCAAGTCCATT-5'

DysRNA_arrange_TPM 40741702_22 TTACCTGAACCCGGCATACCAA

target id: NTBX_mRNA_20748_cds

1863 5'-GATCACTGGTATGCCGGGTTCGGGTAAAACTA-3' 1894 cleavage site: 1879

|||||||||||||||o|||||

3'-AACCATACGGCCCAAGTCCATT-5'

DysRNA_arrange_TPM 40741040_22 TTACCTGATCCTGGCATACCAA

target id: NTBX_mRNA_20748_cds

1863 5'-GATCACTGGTATGCCGGGTTCGGGTAAAACTA-3' 1894 cleavage site: 1879

|||||||||o|| ||o|||||

3'-AACCATACGGTCCTAGTCCATT-5'

DysRNA_arrange_TPM 40739525_22 TTACCTGAATCTGGCATACCAA

target id: NTBX_mRNA_20748_cds

1863 5'-GATCACTGGTATGCCGGGTTCGGGTAAAACTA-3' 1894 cleavage site: 1879

|||||||||o|o|||o|||||

3'-AACCATACGGTCTAAGTCCATT-5'

DysRNA_arrange_TPM 40745543_21 TTACCTCAACCTGGCATACCA

target id: NTBX_mRNA_20748_cds

1864 5'-ATCACTGGTATGCCGGGTTCGGGTAAAACTA-3' 1894 cleavage site: 1879

|||||||||o|||| o|||||

3'-ACCATACGGTCCAACTCCATT-5'

DysRNA_arrange_TPM 40785222_22 TTACTTGAACCTGGCATACCAA

target id: NTBX_mRNA_20748_cds

1863 5'-GATCACTGGTATGCCGGGTTCGGGTAAAACTA-3' 1894 cleavage site: 1879

|||||||||o|||||oo||||

3'-AACCATACGGTCCAAGTTCATT-5'

DysRNA_arrange_TPM 40740904_22 TTACCTGAACTTGGCATACCAA

target id: NTBX_mRNA_20748_cds

1863 5'-GATCACTGGTATGCCGGGTTCGGGTAAAACTA-3' 1894 cleavage site: 1879

|||||||||oo||||o|||||

3'-AACCATACGGTTCAAGTCCATT-5'

DysRNA_arrange_TPM 40741094_23 TTACCTGAACCTGGCATACCACT

target id: NTBX_mRNA_20748_cds

1862 5'-CGATCACTGGTATGCCGGGTTCGGGTAAAACTA-3' 1894 cleavage site: 1879

|||||||||o|||||o|||||

3'-TCACCATACGGTCCAAGTCCATT-5'

DysRNA_arrange_TPM 40932757_22 TTATCTGAACCTGGCATACCAA

target id: NTBX_mRNA_20748_cds

1863 5'-GATCACTGGTATGCCGGGTTCGGGTAAAACTA-3' 1894 cleavage site: 1879

|||||||||o|||||o|o|||

3'-AACCATACGGTCCAAGTCTATT-5'

DysRNA_arrange_TPM 40740779_22 TTACCTGAACCTGGCATACCCT

target id: NTBX_mRNA_20748_cds

1863 5'-GATCACTGGTATGCCGGGTTCGGGTAAAACTA-3' 1894 cleavage site: 1879

||||||||o|||||o|||||

3'-TCCCATACGGTCCAAGTCCATT-5'

DysRNA_arrange_TPM 31384081_21 CCACGATCCACTGAGATTCAG

target id: NTBX_mRNA_132526_cds

1436 5'-GAGTTCTGAATCTGAGTGGAAC-TGGTATTA-3' 1465 cleavage site: 1450

|||||||| |||||| | |||

3'-GACTTAGAGTCACCTAGCACC-5'

DysRNA_arrange_TPM 31383804_22 CCACGATCCACTGAGATTCAGC

target id: NTBX_mRNA_132526_cds

1435 5'-AGAGTTCTGAATCTGAGTGGAAC-TGGTATTA-3' 1465 cleavage site: 1450

|||||||| |||||| | |||

3'-CGACTTAGAGTCACCTAGCACC-5'

DysRNA_arrange_TPM 41911182_21 TTGTCTTTCCGATACCTCCCA

target id: NTTN90_mRNA_103113_cds

621 5'-TGGAATGGCAGGTGTTGGCAAGACAACACTT-3' 651 cleavage site: 636

||| ||||o|o|| |||||||

3'-ACCCTCCATAGCCTTTCTGTT-5'

DysRNA_arrange_TPM 37472283_20 TGCTTGAAATTGTCGGGAGG

target id: NTBX_mRNA_97358_cds

674 5'-ATGTCCCTCAAGTCAATTTCAAGCTAGCTC-3' 703 cleavage site: 688

|||| | |||||||||||

3'-GGAGGGCTGTTAAAGTTCGT-5'

DysRNA_arrange_TPM 26571156_22 GCTTGAAATTGTCGGGAGGGAA

target id: NTBX_mRNA_97358_cds

671 5'-TTGATGTCCCTCAAGTCAATTTCAAGCTAGCT-3' 702 cleavage site: 687

|||||| | |||||||||||

3'-AAGGGAGGGCTGTTAAAGTTCG-5'

DysRNA_arrange_TPM 26571173_21 GCTTGAAATTGTCGGGAGGGA

target id: NTBX_mRNA_97358_cds

672 5'-TGATGTCCCTCAAGTCAATTTCAAGCTAGCT-3' 702 cleavage site: 687

|||||| | |||||||||||

3'-AGGGAGGGCTGTTAAAGTTCG-5'

DysRNA_arrange_TPM 37472140_21 TGCTTGAAATTGTCGGGAGGG

target id: NTBX_mRNA_97358_cds

673 5'-GATGTCCCTCAAGTCAATTTCAAGCTAGCTC-3' 703 cleavage site: 688

||||| | |||||||||||

3'-GGGAGGGCTGTTAAAGTTCGT-5'

DysRNA_arrange_TPM 43499236_21 TTTGTCATGATAAATAAAGTC

target id: NTBX_mRNA_97358_cds

950 5'-CCAAGGACTTTATTTATCATGACAAATTGTT-3' 980 cleavage site: 965

|||||||||||||||||||||

3'-CTGAAATAAATAGTACTGTTT-5'

DysRNA_arrange_TPM 41815455_20 TTGTGAAATGACTTGAGAGG

target id: NTBX_mRNA_97358_cds

672 5'-TGATGTCCCTCAAGTCAATTTCA-AGCTAGC-3' 701 cleavage site: 686

o| |||||||| |||||| |o

3'-GGAGAGTTCAG-TAAAGTGTT-5'

DysRNA_arrange_TPM 37472075_22 TGCTTGAAATTGTCGGGAGGGA

target id: NTBX_mRNA_97358_cds

672 5'-TGATGTCCCTCAAGTCAATTTCAAGCTAGCTC-3' 703 cleavage site: 688

|||||| | |||||||||||

3'-AGGGAGGGCTGTTAAAGTTCGT-5'

DysRNA_arrange_TPM 43498503_21 TTTGTCATGATGAATAAAGTC

target id: NTBX_mRNA_97358_cds

950 5'-CCAAGGACTTTATTTATCATGACAAATTGTT-3' 980 cleavage site: 965

|||||||||o|||||||||||

3'-CTGAAATAAGTAGTACTGTTT-5'

DysRNA_arrange_TPM 26571723_20 GCTTGAAATTGTCGGGAGGG

target id: NTBX_mRNA_97358_cds

673 5'-GATGTCCCTCAAGTCAATTTCAAGCTAGCT-3' 702 cleavage site: 687

||||| | |||||||||||

3'-GGGAGGGCTGTTAAAGTTCG-5'

DysRNA_arrange_TPM 22746892_21 GAGGTAGTGACAATAAATAAC

target id: NTTN90_mRNA_98286_cds

2846 5'-TACGTCTTATTGATTGTCACTCTCTTACCTC-3' 2876 cleavage site: 2861

||||| ||||||||| o||o

3'-CAATAAATAACAGTGATGGAG-5'

DysRNA_arrange_TPM 02841428_21 AAGAACTTTGAAGAGAGAGTT

target id: NTTN90_mRNA_98286_cds

3679 5'-CGCCTAAC-CTCACTTCAAAG-TCTACGGAT-3' 3707 cleavage site: 3692

||| ||| |||||||| |||

3'-TTGAGAGAGAAGTTTCAAGAA-5'

DysRNA_arrange_TPM 02842108_20 AAGAACTTTGAAGAGAGAGT

target id: NTTN90_mRNA_98286_cds

3680 5'-GCCTAAC-CTCACTTCAAAG-TCTACGGAT-3' 3707 cleavage site: 3692

|| ||| |||||||| |||

3'-TGAGAGAGAAGTTTCAAGAA-5'

DysRNA_arrange_TPM 10032960_20 AGGTAGTGACAATAAATAAC

target id: NTTN90_mRNA_98286_cds

2846 5'-TACGTCTTATTGATTGTCACTCTCTTACCT-3' 2875 cleavage site: 2860

||||| ||||||||| o||

3'-CAATAAATAACAGTGATGGA-5'

DysRNA_arrange_TPM 38030093_21 TCAACATCATCAGACATATGG

target id: NTK326_mRNA_108678_cds

2223 5'-TTTGCCTGTATGTCTGAAGAGGTTGACTTTA-3' 2253 cleavage site: 2238

|oo||||||||| || |||||

3'-GGTATACAGACTACTACAACT-5'

DysRNA_arrange_TPM 37756134_21 TGTCTCACGTCATTCCATGCA

target id: NTTN90_mRNA_15578_cds

610 5'-AGTAAAGCATGGGATGACGTGAGACTATGCT-3' 640 cleavage site: 625

||||||o||||||||||||

3'-ACGTACCTTACTGCACTCTGT-5'

DysRNA_arrange_TPM 38706727_21 TCGACTCCCATTGTTTTGACT

target id: NTTN90_mRNA_15578_cds

641 5'-TTCCAAGTGAAAACAATGGGAGTCGAATACT-3' 671 cleavage site: 656

||| |||||||||||||||||

3'-TCAGTTTTGTTACCCTCAGCT-5'

DysRNA_arrange_TPM 41905826_21 TTGTCTCACGTCATTCCATGC

target id: NTTN90_mRNA_15578_cds

611 5'-GTAAAGCATGGGATGACGTGAGACTATGCTT-3' 641 cleavage site: 626

||||||o||||||||||||

3'-CGTACCTTACTGCACTCTGTT-5'

DysRNA_arrange_TPM 30234614_21 CGACTCCCATTGTTTTGACTT

target id: NTTN90_mRNA_15578_cds

640 5'-TTTCCAAGTGAAAACAATGGGAGTCGAATAC-3' 670 cleavage site: 655

|||| ||||||||||||||||

3'-TTCAGTTTTGTTACCCTCAGC-5'

DysRNA_arrange_TPM 38030093_21 TCAACATCATCAGACATATGG

target id: NTK326_mRNA_108677_cds

2223 5'-TTTGCCTGTATGTCTGAAGAGGTTGACTTTA-3' 2253 cleavage site: 2238

|oo||||||||| || |||||

3'-GGTATACAGACTACTACAACT-5'

DysRNA_arrange_TPM 37756134_21 TGTCTCACGTCATTCCATGCA

target id: NTTN90_mRNA_15580_cds

796 5'-AGTAAAGCATGGGATGATGTGAGACAATGCT-3' 826 cleavage site: 811

||||||o||||o||||||||

3'-ACGTACCTTACTGCACTCTGT-5'

DysRNA_arrange_TPM 41905826_21 TTGTCTCACGTCATTCCATGC

target id: NTTN90_mRNA_15580_cds

797 5'-GTAAAGCATGGGATGATGTGAGACAATGCTT-3' 827 cleavage site: 812

||||||o||||o|||||||||

3'-CGTACCTTACTGCACTCTGTT-5'

DysRNA_arrange_TPM 37756134_21 TGTCTCACGTCATTCCATGCA

target id: NTBX_mRNA_24682_cds

796 5'-AGTAAAGCATGGGATGATGTGAGACAATGCT-3' 826 cleavage site: 811

||||||o||||o||||||||

3'-ACGTACCTTACTGCACTCTGT-5'

DysRNA_arrange_TPM 41905826_21 TTGTCTCACGTCATTCCATGC

target id: NTBX_mRNA_24682_cds

797 5'-GTAAAGCATGGGATGATGTGAGACAATGCTT-3' 827 cleavage site: 812

||||||o||||o|||||||||

3'-CGTACCTTACTGCACTCTGTT-5'

DysRNA_arrange_TPM 24549066_21 GGGGATGTATCTCAAATGGTA

target id: NTTN90_mRNA_42816_cds

1736 5'-AGCTATATCTTTTGAGATACATCGCTTTATCA-3' 1767 cleavage site: 1752

||o| ||||||||||||| |oo

3'-ATGGTAAACTCTATGTAG-GGG-5'

DysRNA_arrange_TPM 02221656_22 AAATATAGATTTGGTTAAGGCT

target id: NTTN90_mRNA_98694_cds

2123 5'-TTGAGAGCCTTCAACCAAATTCTG-ATTTGAGCA-3' 2155 cleavage site: 2140

|||||| ||||||| |||o ||||

3'-TCGGAA-TTGGTTT-AGATATAAA-5'

DysRNA_arrange_TPM 43499236_21 TTTGTCATGATAAATAAAGTC

target id: NTTN90_mRNA_42144_cds

950 5'-CCAAGGACTTTATTTATCATGACAAATTGTT-3' 980 cleavage site: 965

|||||||||||||||||||||

3'-CTGAAATAAATAGTACTGTTT-5'

DysRNA_arrange_TPM 43498503_21 TTTGTCATGATGAATAAAGTC

target id: NTTN90_mRNA_42144_cds

950 5'-CCAAGGACTTTATTTATCATGACAAATTGTT-3' 980 cleavage site: 965

|||||||||o|||||||||||

3'-CTGAAATAAGTAGTACTGTTT-5'

DysRNA_arrange_TPM 39918426_21 TCTCGTTGAAATCTAGCTACC

target id: NTTN90_mRNA_42144_cds

2548 5'-AGCTTGGT-GATCGTTTTCAACGAGAGAGGT-3' 2577 cleavage site: 2562

||| | | | |||||||||||

3'-CCATCGATCTAAAGTTGCTCT-5'

DysRNA_arrange_TPM 22746892_21 GAGGTAGTGACAATAAATAAC

target id: NTBX_mRNA_91224_cds

2846 5'-TACGTCTTATTGATTGTCACTCTCTTACCTC-3' 2876 cleavage site: 2861

||||| ||||||||| o||o

3'-CAATAAATAACAGTGATGGAG-5'

DysRNA_arrange_TPM 02841428_21 AAGAACTTTGAAGAGAGAGTT

target id: NTBX_mRNA_91224_cds

3679 5'-CGCCTAAC-CTCACTTCAAAG-TCTACGGAT-3' 3707 cleavage site: 3692

||| ||| |||||||| |||

3'-TTGAGAGAGAAGTTTCAAGAA-5'

DysRNA_arrange_TPM 02842108_20 AAGAACTTTGAAGAGAGAGT

target id: NTBX_mRNA_91224_cds

3680 5'-GCCTAAC-CTCACTTCAAAG-TCTACGGAT-3' 3707 cleavage site: 3692

|| ||| |||||||| |||

3'-TGAGAGAGAAGTTTCAAGAA-5'

DysRNA_arrange_TPM 10032960_20 AGGTAGTGACAATAAATAAC

target id: NTBX_mRNA_91224_cds

2846 5'-TACGTCTTATTGATTGTCACTCTCTTACCT-3' 2875 cleavage site: 2860

||||| ||||||||| o||

3'-CAATAAATAACAGTGATGGA-5'

DysRNA_arrange_TPM 22746892_21 GAGGTAGTGACAATAAATAAC

target id: NTTN90_mRNA_98279_cds

2591 5'-TACGTCTTATTGATTGTCACTCTCTTACCTC-3' 2621 cleavage site: 2606

||||| ||||||||| o||o

3'-CAATAAATAACAGTGATGGAG-5'

DysRNA_arrange_TPM 02841428_21 AAGAACTTTGAAGAGAGAGTT

target id: NTTN90_mRNA_98279_cds

3424 5'-CGCCTAAC-CTCACTTCAAAG-TCTACGGAT-3' 3452 cleavage site: 3437

||| ||| |||||||| |||

3'-TTGAGAGAGAAGTTTCAAGAA-5'

DysRNA_arrange_TPM 02842108_20 AAGAACTTTGAAGAGAGAGT

target id: NTTN90_mRNA_98279_cds

3425 5'-GCCTAAC-CTCACTTCAAAG-TCTACGGAT-3' 3452 cleavage site: 3437

|| ||| |||||||| |||

3'-TGAGAGAGAAGTTTCAAGAA-5'

DysRNA_arrange_TPM 10032960_20 AGGTAGTGACAATAAATAAC

target id: NTTN90_mRNA_98279_cds

2591 5'-TACGTCTTATTGATTGTCACTCTCTTACCT-3' 2620 cleavage site: 2605

||||| ||||||||| o||

3'-CAATAAATAACAGTGATGGA-5'

DysRNA_arrange_TPM 37472283_20 TGCTTGAAATTGTCGGGAGG

target id: NTK326_mRNA_97821_cds

674 5'-ATGTCCCTCAAGTCAATTTCAAGCTAGCTC-3' 703 cleavage site: 688

|||| | |||||||||||

3'-GGAGGGCTGTTAAAGTTCGT-5'

DysRNA_arrange_TPM 26571156_22 GCTTGAAATTGTCGGGAGGGAA

target id: NTK326_mRNA_97821_cds

671 5'-TTGATGTCCCTCAAGTCAATTTCAAGCTAGCT-3' 702 cleavage site: 687

|||||| | |||||||||||

3'-AAGGGAGGGCTGTTAAAGTTCG-5'

DysRNA_arrange_TPM 26571173_21 GCTTGAAATTGTCGGGAGGGA

target id: NTK326_mRNA_97821_cds

672 5'-TGATGTCCCTCAAGTCAATTTCAAGCTAGCT-3' 702 cleavage site: 687

|||||| | |||||||||||

3'-AGGGAGGGCTGTTAAAGTTCG-5'

DysRNA_arrange_TPM 37472140_21 TGCTTGAAATTGTCGGGAGGG

target id: NTK326_mRNA_97821_cds

673 5'-GATGTCCCTCAAGTCAATTTCAAGCTAGCTC-3' 703 cleavage site: 688

||||| | |||||||||||

3'-GGGAGGGCTGTTAAAGTTCGT-5'

DysRNA_arrange_TPM 43499236_21 TTTGTCATGATAAATAAAGTC

target id: NTK326_mRNA_97821_cds

950 5'-CCAAGGACTTTATTTATCATGACAAATTGTT-3' 980 cleavage site: 965

|||||||||||||||||||||

3'-CTGAAATAAATAGTACTGTTT-5'

DysRNA_arrange_TPM 41815455_20 TTGTGAAATGACTTGAGAGG

target id: NTK326_mRNA_97821_cds

672 5'-TGATGTCCCTCAAGTCAATTTCA-AGCTAGC-3' 701 cleavage site: 686

o| |||||||| |||||| |o

3'-GGAGAGTTCAG-TAAAGTGTT-5'

DysRNA_arrange_TPM 37472075_22 TGCTTGAAATTGTCGGGAGGGA

target id: NTK326_mRNA_97821_cds

672 5'-TGATGTCCCTCAAGTCAATTTCAAGCTAGCTC-3' 703 cleavage site: 688

|||||| | |||||||||||

3'-AGGGAGGGCTGTTAAAGTTCGT-5'

DysRNA_arrange_TPM 43498503_21 TTTGTCATGATGAATAAAGTC

target id: NTK326_mRNA_97821_cds

950 5'-CCAAGGACTTTATTTATCATGACAAATTGTT-3' 980 cleavage site: 965

|||||||||o|||||||||||

3'-CTGAAATAAGTAGTACTGTTT-5'

DysRNA_arrange_TPM 26571723_20 GCTTGAAATTGTCGGGAGGG

target id: NTK326_mRNA_97821_cds

673 5'-GATGTCCCTCAAGTCAATTTCAAGCTAGCT-3' 702 cleavage site: 687

||||| | |||||||||||

3'-GGGAGGGCTGTTAAAGTTCG-5'

DysRNA_arrange_TPM 43499236_21 TTTGTCATGATAAATAAAGTC

target id: NTBX_mRNA_49338_cds

917 5'-CCAAGGACTTTATTTATCATGACAAATTGTT-3' 947 cleavage site: 932

|||||||||||||||||||||

3'-CTGAAATAAATAGTACTGTTT-5'

DysRNA_arrange_TPM 43498503_21 TTTGTCATGATGAATAAAGTC

target id: NTBX_mRNA_49338_cds

917 5'-CCAAGGACTTTATTTATCATGACAAATTGTT-3' 947 cleavage site: 932

|||||||||o|||||||||||

3'-CTGAAATAAGTAGTACTGTTT-5'

DysRNA_arrange_TPM 39918426_21 TCTCGTTGAAATCTAGCTACC

target id: NTBX_mRNA_49338_cds

2458 5'-AGCTTGGT-GATCGTTTTCAACGAGAGAGGT-3' 2487 cleavage site: 2472

||| | | | |||||||||||

3'-CCATCGATCTAAAGTTGCTCT-5'

DysRNA_arrange_TPM 37472283_20 TGCTTGAAATTGTCGGGAGG

target id: NTTN90_mRNA_15567_cds

674 5'-ATGTCCCTCAAGTCAATTTCAAGCTAGCTC-3' 703 cleavage site: 688

|||| | |||||||||||

3'-GGAGGGCTGTTAAAGTTCGT-5'

DysRNA_arrange_TPM 26571156_22 GCTTGAAATTGTCGGGAGGGAA

target id: NTTN90_mRNA_15567_cds

671 5'-TTGATGTCCCTCAAGTCAATTTCAAGCTAGCT-3' 702 cleavage site: 687

|||||| | |||||||||||

3'-AAGGGAGGGCTGTTAAAGTTCG-5'

DysRNA_arrange_TPM 26571173_21 GCTTGAAATTGTCGGGAGGGA

target id: NTTN90_mRNA_15567_cds

672 5'-TGATGTCCCTCAAGTCAATTTCAAGCTAGCT-3' 702 cleavage site: 687

|||||| | |||||||||||

3'-AGGGAGGGCTGTTAAAGTTCG-5'

DysRNA_arrange_TPM 37472140_21 TGCTTGAAATTGTCGGGAGGG

target id: NTTN90_mRNA_15567_cds

673 5'-GATGTCCCTCAAGTCAATTTCAAGCTAGCTC-3' 703 cleavage site: 688

||||| | |||||||||||

3'-GGGAGGGCTGTTAAAGTTCGT-5'

DysRNA_arrange_TPM 43499236_21 TTTGTCATGATAAATAAAGTC

target id: NTTN90_mRNA_15567_cds

950 5'-CCAAGGACTTTATTTATCATGACAAATTGTT-3' 980 cleavage site: 965

|||||||||||||||||||||

3'-CTGAAATAAATAGTACTGTTT-5'

DysRNA_arrange_TPM 41815455_20 TTGTGAAATGACTTGAGAGG

target id: NTTN90_mRNA_15567_cds

672 5'-TGATGTCCCTCAAGTCAATTTCA-AGCTAGC-3' 701 cleavage site: 686

o| |||||||| |||||| |o

3'-GGAGAGTTCAG-TAAAGTGTT-5'

DysRNA_arrange_TPM 37472075_22 TGCTTGAAATTGTCGGGAGGGA

target id: NTTN90_mRNA_15567_cds

672 5'-TGATGTCCCTCAAGTCAATTTCAAGCTAGCTC-3' 703 cleavage site: 688

|||||| | |||||||||||

3'-AGGGAGGGCTGTTAAAGTTCGT-5'

DysRNA_arrange_TPM 43498503_21 TTTGTCATGATGAATAAAGTC

target id: NTTN90_mRNA_15567_cds

950 5'-CCAAGGACTTTATTTATCATGACAAATTGTT-3' 980 cleavage site: 965

|||||||||o|||||||||||

3'-CTGAAATAAGTAGTACTGTTT-5'

DysRNA_arrange_TPM 26571723_20 GCTTGAAATTGTCGGGAGGG

target id: NTTN90_mRNA_15567_cds

673 5'-GATGTCCCTCAAGTCAATTTCAAGCTAGCT-3' 702 cleavage site: 687

||||| | |||||||||||

3'-GGGAGGGCTGTTAAAGTTCG-5'

DysRNA_arrange_TPM 41911182_21 TTGTCTTTCCGATACCTCCCA

target id: NTTN90_mRNA_103115_cds

732 5'-TGGAATGGCAGGTGTTGGCAAGACAACACTT-3' 762 cleavage site: 747

||| ||||o|o|| |||||||

3'-ACCCTCCATAGCCTTTCTGTT-5'

DysRNA_arrange_TPM 37756134_21 TGTCTCACGTCATTCCATGCA

target id: NTTN90_mRNA_109401_cds

769 5'-AGTGAAGCATGGGATGACGTGAGACTATGCT-3' 799 cleavage site: 784

||||||o||||||||||||

3'-ACGTACCTTACTGCACTCTGT-5'

DysRNA_arrange_TPM 41905826_21 TTGTCTCACGTCATTCCATGC

target id: NTTN90_mRNA_109401_cds

770 5'-GTGAAGCATGGGATGACGTGAGACTATGCTT-3' 800 cleavage site: 785

||||||o||||||||||||

3'-CGTACCTTACTGCACTCTGTT-5'

DysRNA_arrange_TPM 38030093_21 TCAACATCATCAGACATATGG

target id: NTTN90_mRNA_98323_cds

2223 5'-TTTGCCTGTATGTCTGAAGAGGTTGACTTTA-3' 2253 cleavage site: 2238

|oo||||||||| || |||||

3'-GGTATACAGACTACTACAACT-5'

DysRNA_arrange_TPM 40123240_21 TCTTCTTTGTAGCCTTTCATA

target id: NTTN90_mRNA_64613_cds

3467 5'-GAGTTTGTGATAGCCTATCAAAGAAGATGGAC-3' 3498 cleavage site: 3483

|o||| || ||| |||||||||

3'-ATACTTTCCGAT-GTTTCTTCT-5'

DysRNA_arrange_TPM 41935941_21 TTGTTGGTAGCTTGATGTATG

target id: NTTN90_mRNA_21859_cds

2333 5'-TCGGACATATCAGTAAGCTACCAG-AACACCA-3' 2363 cleavage site: 2348

|||| || o|||||||||o ||

3'-GTAT-GTAGTTCGATGGTTGTT-5'

DysRNA_arrange_TPM 38030093_21 TCAACATCATCAGACATATGG

target id: NTK326_mRNA_140205_cds

2223 5'-TTTGCCTATATGTCTGAAGAGGTTGACTTTA-3' 2253 cleavage site: 2238

|o|||||||||| || |||||

3'-GGTATACAGACTACTACAACT-5'

DysRNA_arrange_TPM 02221656_22 AAATATAGATTTGGTTAAGGCT

target id: NTBX_mRNA_113576_cds

2024 5'-TTGAGAGCCTTCAACCAAATTCTG-ATTTGAGCA-3' 2056 cleavage site: 2041

|||||| ||||||| |||o ||||

3'-TCGGAA-TTGGTTT-AGATATAAA-5'

DysRNA_arrange_TPM 38030093_21 TCAACATCATCAGACATATGG

target id: NTTN90_mRNA_98324_cds

2223 5'-TTTGCCTGTATGTCTGAAGAGGTTGACTTTA-3' 2253 cleavage site: 2238

|oo||||||||| || |||||

3'-GGTATACAGACTACTACAACT-5'

DysRNA_arrange_TPM 43499236_21 TTTGTCATGATAAATAAAGTC

target id: NTTN90_mRNA_42145_cds

917 5'-CCAAGGACTTTATTTATCATGACAAATTGTT-3' 947 cleavage site: 932

|||||||||||||||||||||

3'-CTGAAATAAATAGTACTGTTT-5'

DysRNA_arrange_TPM 43498503_21 TTTGTCATGATGAATAAAGTC

target id: NTTN90_mRNA_42145_cds

917 5'-CCAAGGACTTTATTTATCATGACAAATTGTT-3' 947 cleavage site: 932

|||||||||o|||||||||||

3'-CTGAAATAAGTAGTACTGTTT-5'

DysRNA_arrange_TPM 39918426_21 TCTCGTTGAAATCTAGCTACC

target id: NTTN90_mRNA_42145_cds

2515 5'-AGCTTGGT-GATCGTTTTCAACGAGAGAGGT-3' 2544 cleavage site: 2529

||| | | | |||||||||||

3'-CCATCGATCTAAAGTTGCTCT-5'

DysRNA_arrange_TPM 32948576_20 CTGGGAAGTCCTCGTGTTGC

target id: NTTN90_mRNA_102494_cds

3082 5'-CGGCTACAACATGTGGACTTCTCAGGTGTC-3' 3111 cleavage site: 3096

|||||o| |||||||o|||

3'-CGTTGTGCTCCTGAAGGGTC-5'

DysRNA_arrange_TPM 41911182_21 TTGTCTTTCCGATACCTCCCA

target id: NTK326_mRNA_115281_cds

732 5'-TGGAATGGCAGGTGTTGGCAAGACAACACTT-3' 762 cleavage site: 747

||| ||||o|o|| |||||||

3'-ACCCTCCATAGCCTTTCTGTT-5'

DysRNA_arrange_TPM 22746892_21 GAGGTAGTGACAATAAATAAC

target id: NTTN90_mRNA_98280_cds

2693 5'-TACGTCTTATTGATTGTCACTCTCTTACCTC-3' 2723 cleavage site: 2708

||||| ||||||||| o||o

3'-CAATAAATAACAGTGATGGAG-5'

DysRNA_arrange_TPM 02841428_21 AAGAACTTTGAAGAGAGAGTT

target id: NTTN90_mRNA_98280_cds

3526 5'-CGCCTAAC-CTCACTTCAAAG-TCTACGGAT-3' 3554 cleavage site: 3539

||| ||| |||||||| |||

3'-TTGAGAGAGAAGTTTCAAGAA-5'

DysRNA_arrange_TPM 02842108_20 AAGAACTTTGAAGAGAGAGT

target id: NTTN90_mRNA_98280_cds

3527 5'-GCCTAAC-CTCACTTCAAAG-TCTACGGAT-3' 3554 cleavage site: 3539

|| ||| |||||||| |||

3'-TGAGAGAGAAGTTTCAAGAA-5'

DysRNA_arrange_TPM 10032960_20 AGGTAGTGACAATAAATAAC

target id: NTTN90_mRNA_98280_cds

2693 5'-TACGTCTTATTGATTGTCACTCTCTTACCT-3' 2722 cleavage site: 2707

||||| ||||||||| o||

3'-CAATAAATAACAGTGATGGA-5'

DysRNA_arrange_TPM 22746892_21 GAGGTAGTGACAATAAATAAC

target id: NTK326_mRNA_85299_cds

2846 5'-TACGTCTTATTGATTGTCACTCTCTTACCTC-3' 2876 cleavage site: 2861

||||| ||||||||| o||o

3'-CAATAAATAACAGTGATGGAG-5'

DysRNA_arrange_TPM 02841428_21 AAGAACTTTGAAGAGAGAGTT

target id: NTK326_mRNA_85299_cds

3679 5'-CGCCTAAC-CTCACTTCAAAG-TCTACGGAT-3' 3707 cleavage site: 3692

||| ||| |||||||| |||

3'-TTGAGAGAGAAGTTTCAAGAA-5'

DysRNA_arrange_TPM 02842108_20 AAGAACTTTGAAGAGAGAGT

target id: NTK326_mRNA_85299_cds

3680 5'-GCCTAAC-CTCACTTCAAAG-TCTACGGAT-3' 3707 cleavage site: 3692

|| ||| |||||||| |||

3'-TGAGAGAGAAGTTTCAAGAA-5'

DysRNA_arrange_TPM 10032960_20 AGGTAGTGACAATAAATAAC

target id: NTK326_mRNA_85299_cds

2846 5'-TACGTCTTATTGATTGTCACTCTCTTACCT-3' 2875 cleavage site: 2860

||||| ||||||||| o||

3'-CAATAAATAACAGTGATGGA-5'

DysRNA_arrange_TPM 38706727_21 TCGACTCCCATTGTTTTGACT

target id: NTK326_mRNA_61519_cds

818 5'-TTCCAAGTGAAAACAATGGGAGTCGAATACT-3' 848 cleavage site: 833

||| |||||||||||||||||

3'-TCAGTTTTGTTACCCTCAGCT-5'

DysRNA_arrange_TPM 30234614_21 CGACTCCCATTGTTTTGACTT

target id: NTK326_mRNA_61519_cds

817 5'-TTTCCAAGTGAAAACAATGGGAGTCGAATAC-3' 847 cleavage site: 832

|||| ||||||||||||||||

3'-TTCAGTTTTGTTACCCTCAGC-5'

DysRNA_arrange_TPM 02767294_21 AAGAAGATGATTATGGAGGGC

target id: NTK326_mRNA_72001_cds

2319 5'-TCCAAGTGCTCCATCATCATCTTCTTCAGAT-3' 2349 cleavage site: 2334

|o |||||| |||||||||||

3'-CGGGAGGTATTAGTAGAAGAA-5'

DysRNA_arrange_TPM 02768834_22 AAGAAGATGATTATGGAGGGCA

target id: NTK326_mRNA_72001_cds

2318 5'-GTCCAAGTGCTCCATCATCATCTTCTTCAGAT-3' 2349 cleavage site: 2334

|o |||||| |||||||||||

3'-ACGGGAGGTATTAGTAGAAGAA-5'

DysRNA_arrange_TPM 32195924_21 CCTAAGATTGTATGGACGTGG

target id: NTK326_mRNA_3237_cds

766 5'-GAAACCCA-G-CCAGACATTCTTAGGGGATA-3' 794 cleavage site: 779

||| | ||| ||| |||||||

3'-GGTGCAGGTATGTTAGAATCC-5'

DysRNA_arrange_TPM 15928038_22 ATAGAATAATTATGAATGTGCT

target id: NTTN90_mRNA_117651_cds

2280 5'-ATCATGTCGCATTCATAATTA-TCTATATCAT-3' 2310 cleavage site: 2295

|o|||||||||||| |||||

3'-TCGTGTAAGTATTAATAAGATA-5'

DysRNA_arrange_TPM 43514857_22 TTTGTCTTTCCGATACCTCCCA

target id: NTTN90_mRNA_82332_cds

516 5'-AGGGATGGGTGGCATCGGTAAGACAACTTTAG-3' 547 cleavage site: 532

|||| || ||||| |||||||

3'-ACCCTCCATAGCCTTTCTGTTT-5'

DysRNA_arrange_TPM 43514186_22 TTTGTCTTTCCGATGCCTCCCA

target id: NTTN90_mRNA_82332_cds

516 5'-AGGGATGGGTGGCATCGGTAAGACAACTTTAG-3' 547 cleavage site: 532

|||| |||||||| |||||||

3'-ACCCTCCGTAGCCTTTCTGTTT-5'

DysRNA_arrange_TPM 41911182_21 TTGTCTTTCCGATACCTCCCA

target id: NTTN90_mRNA_82332_cds

516 5'-AGGGATGGGTGGCATCGGTAAGACAACTTTA-3' 546 cleavage site: 531

|||| || ||||| |||||||

3'-ACCCTCCATAGCCTTTCTGTT-5'

DysRNA_arrange_TPM 43513291_22 TTTGTCTTTCCGATGCCTCCCT

target id: NTTN90_mRNA_82332_cds

516 5'-AGGGATGGGTGGCATCGGTAAGACAACTTTAG-3' 547 cleavage site: 532

||| |||||||| |||||||

3'-TCCCTCCGTAGCCTTTCTGTTT-5'

DysRNA_arrange_TPM 20802129_24 ATTTACACTTTGTAGGCCCGAGTA

target id: NTTN90_mRNA_37812_cds

1513 5'-AAAACTACTTTGGCATACAAAGTGTATAGTGATAA-3' 1547 cleavage site: 1532

||||o ||| ||||||||||| |o|

3'-ATGAGCCCGGATGTTTCACAT-TTA-5'

DysRNA_arrange_TPM 21202783_24 ATTTTACACTTTGTAGGCCCGAGT

target id: NTTN90_mRNA_37812_cds

1514 5'-AAACTACTTTGGCATACAAAGTGTATAGTGATAA-3' 1547 cleavage site: 1532

|||o ||| ||||||||||| |o|

3'-TGAGCCCGGATGTTTCACATTTTA-5'

DysRNA_arrange_TPM 20608921_24 ATTCTAGATCTTCTTTCACCAGTC

target id: NTTN90_mRNA_37812_cds

1095 5'-AATTGGACTGGTGAAAGAAGATCTAGAATTCATA-3' 1128 cleavage site: 1113

||||||||||||||||||||||||

3'-CTGACCACTTTCTTCTAGATCTTA-5'

DysRNA_arrange_TPM 40506628_21 TTAGATCTTCTTTCACCAGCT

target id: NTTN90_mRNA_37812_cds

1094 5'-AAATTGGACTGGTGAAAGAAGATCTAGAATTC-3' 1125 cleavage site: 1110

o| ||||||||||||||||||o

3'-TC-GACCACTTTCTTCTAGATT-5'

DysRNA_arrange_TPM 43983812_21 TTTTACCTGAACCTGGCATAC

target id: NTTN90_mRNA_63333_cds

1878 5'-CACTGGCATGCCGGGTTCGGGTAAAACTACT-3' 1908 cleavage site: 1893

|||||o|||||o|||||||

3'-CATACGGTCCAAGTCCATTTT-5'

DysRNA_arrange_TPM 40741688_22 TTACCTGAACCTGGCATGCCAA

target id: NTTN90_mRNA_63333_cds

1875 5'-GATCACTGGCATGCCGGGTTCGGGTAAAACTA-3' 1906 cleavage site: 1891

|||||||||o|||||o|||||

3'-AACCGTACGGTCCAAGTCCATT-5'

DysRNA_arrange_TPM 40727231_22 TTACCCGAACCTGGCATACCAA

target id: NTTN90_mRNA_63333_cds

1875 5'-GATCACTGGCATGCCGGGTTCGGGTAAAACTA-3' 1906 cleavage site: 1891

||| |||||o|||||||||||

3'-AACCATACGGTCCAAGCCCATT-5'

DysRNA_arrange_TPM 40741702_22 TTACCTGAACCCGGCATACCAA

target id: NTTN90_mRNA_63333_cds

1875 5'-GATCACTGGCATGCCGGGTTCGGGTAAAACTA-3' 1906 cleavage site: 1891

||| |||||||||||o|||||

3'-AACCATACGGCCCAAGTCCATT-5'

DysRNA_arrange_TPM 07119696_21 AATTGTCACTTGATTGTTGCA

target id: NTBX_mRNA_49494_cds

1850 5'-AATGGTGCAGGAATCAAGTAACAGCTTCATCT-3' 1881 cleavage site: 1866

||||o |||||||| |||o ||

3'-ACGTTGTTAGTTCACTGTT-AA-5'

DysRNA_arrange_TPM 40391668_20 TTAATATTTCAGCTCGAACT

target id: NTBX_mRNA_49494_cds

448 5'-CATGAAG-TT-A-CTGAAATATTAAATGCA-3' 474 cleavage site: 459

|| |o | ||||||||||||

3'-TCAAGCTCGACTTTATAATT-5'

DysRNA_arrange_TPM 32487580_22 CCTTTCGTGCTCAAGTTCTCTC

target id: NTBX_mRNA_49494_cds

720 5'-AAAGGGAGTGAACTTGAGCACCAAAGCATTTG-3' 751 cleavage site: 736

||| |||||||||||| ||||

3'-CTCTCTTGAACTCGTGCTTTCC-5'

DysRNA_arrange_TPM 38561019_24 TCGAAGAAGATGATGAATAGTAGT

target id: NTBX_mRNA_83517_cds

1871 5'-CAAGTGCT-CTA-TCATCATCTTCTTCAGATCTGA-3' 1903 cleavage site: 1888

o|| ||| |||||||||||||| ||

3'-TGATGATAAGTAGTAGAAGAAG-CT-5'

DysRNA_arrange_TPM 02767294_21 AAGAAGATGATTATGGAGGGC

target id: NTBX_mRNA_83517_cds

1869 5'-TTCAAGTGCTCTATCATCATCTTCTTCAGAT-3' 1899 cleavage site: 1884

|o |||o|| |||||||||||

3'-CGGGAGGTATTAGTAGAAGAA-5'

DysRNA_arrange_TPM 24021801_23 GGAGAAGATGATGAATAGTAATT

target id: NTBX_mRNA_83517_cds

1867 5'-AGTTCAAGTGCTCTATCATCATCTTCTTCAGATC-3' 1900 cleavage site: 1885

|| |o|| | ||||||||||||o|

3'-TTAATGATA-AGTAGTAGAAGAGG-5'

DysRNA_arrange_TPM 02768834_22 AAGAAGATGATTATGGAGGGCA

target id: NTBX_mRNA_83517_cds

1868 5'-GTTCAAGTGCTCTATCATCATCTTCTTCAGAT-3' 1899 cleavage site: 1884

|o |||o|| |||||||||||

3'-ACGGGAGGTATTAGTAGAAGAA-5'

DysRNA_arrange_TPM 41343934_21 TTGGAGAAGATGATGAATAGT

target id: NTBX_mRNA_83517_cds

1873 5'-AGTGCTCTA-TCATCATCTTCTTCAGATCTG-3' 1902 cleavage site: 1887

||| ||||||||||||o||o

3'-TGATAAGTAGTAGAAGAGGTT-5'

DysRNA_arrange_TPM 24023543_24 GGAGAAGATGATGAATAGTAATTG

target id: NTBX_mRNA_83517_cds

1866 5'-AAGTTCAAGTGCTCTATCATCATCTTCTTCAGATC-3' 1900 cleavage site: 1885

||| |o|| | ||||||||||||o|

3'-GTTAATGATA-AGTAGTAGAAGAGG-5'

DysRNA_arrange_TPM 27959210_20 GTTGTTTTGAGTTTGAGGCC

target id: NTBX_mRNA_122265_cds

1250 5'-CTGATTGCATTAAACTCAAAA-AATTGCCA-3' 1278 cleavage site: 1263

|| |o|||||||||| ||o

3'-CCGGAGTTTGAGTTTTGTTG-5'

DysRNA_arrange_TPM 27959210_20 GTTGTTTTGAGTTTGAGGCC

target id: NTK326_mRNA_95708_cds

1394 5'-TTGATTGCATTAAACTCAAAA-AATTGCCA-3' 1422 cleavage site: 1407

|| |o|||||||||| ||o

3'-CCGGAGTTTGAGTTTTGTTG-5'

DysRNA_arrange_TPM 40969969_23 TTATTGCTTGAGAATACACGTAG

target id: NTTN90_mRNA_16577_cds

519 5'-ATGGGTTAC-TGT-TTCTCAAGCATATGATGCAA-3' 550 cleavage site: 535

o||| ||| |||||||||| ||o|

3'-GATGCACATAAGAGTTCGT-TATT-5'

DysRNA_arrange_TPM 36076634_22 TATTGCTTGAGAATACACGTAG

target id: NTTN90_mRNA_16577_cds

519 5'-ATGGGTTAC-TGT-TTCTCAAGCATATGATGCA-3' 549 cleavage site: 534

o||| ||| |||||||||| ||o

3'-GATGCACATAAGAGTTCGT-TAT-5'

DysRNA_arrange_TPM 38561019_24 TCGAAGAAGATGATGAATAGTAGT

target id: NTTN90_mRNA_63309_cds

2867 5'-CAAGTGCT-CTA-TCATCATCTTCTTCAGATCTGA-3' 2899 cleavage site: 2884

o|| ||| |||||||||||||| ||

3'-TGATGATAAGTAGTAGAAGAAG-CT-5'

DysRNA_arrange_TPM 02767294_21 AAGAAGATGATTATGGAGGGC

target id: NTTN90_mRNA_63309_cds

2865 5'-TTCAAGTGCTCTATCATCATCTTCTTCAGAT-3' 2895 cleavage site: 2880

|o |||o|| |||||||||||

3'-CGGGAGGTATTAGTAGAAGAA-5'

DysRNA_arrange_TPM 24021801_23 GGAGAAGATGATGAATAGTAATT

target id: NTTN90_mRNA_63309_cds

2863 5'-AGTTCAAGTGCTCTATCATCATCTTCTTCAGATC-3' 2896 cleavage site: 2881

|| |o|| | ||||||||||||o|

3'-TTAATGATA-AGTAGTAGAAGAGG-5'

DysRNA_arrange_TPM 02768834_22 AAGAAGATGATTATGGAGGGCA

target id: NTTN90_mRNA_63309_cds

2864 5'-GTTCAAGTGCTCTATCATCATCTTCTTCAGAT-3' 2895 cleavage site: 2880

|o |||o|| |||||||||||

3'-ACGGGAGGTATTAGTAGAAGAA-5'

DysRNA_arrange_TPM 41343934_21 TTGGAGAAGATGATGAATAGT

target id: NTTN90_mRNA_63309_cds

2869 5'-AGTGCTCTA-TCATCATCTTCTTCAGATCTG-3' 2898 cleavage site: 2883

||| ||||||||||||o||o

3'-TGATAAGTAGTAGAAGAGGTT-5'

DysRNA_arrange_TPM 24023543_24 GGAGAAGATGATGAATAGTAATTG

target id: NTTN90_mRNA_63309_cds

2862 5'-AAGTTCAAGTGCTCTATCATCATCTTCTTCAGATC-3' 2896 cleavage site: 2881

||| |o|| | ||||||||||||o|

3'-GTTAATGATA-AGTAGTAGAAGAGG-5'

DysRNA_arrange_TPM 43514857_22 TTTGTCTTTCCGATACCTCCCA

target id: NTTN90_mRNA_82331_cds

516 5'-AGGGATGGGTGGCATCGGTAAGACAACTTTAG-3' 547 cleavage site: 532

|||| || ||||| |||||||

3'-ACCCTCCATAGCCTTTCTGTTT-5'

DysRNA_arrange_TPM 43514186_22 TTTGTCTTTCCGATGCCTCCCA

target id: NTTN90_mRNA_82331_cds

516 5'-AGGGATGGGTGGCATCGGTAAGACAACTTTAG-3' 547 cleavage site: 532

|||| |||||||| |||||||

3'-ACCCTCCGTAGCCTTTCTGTTT-5'

DysRNA_arrange_TPM 41911182_21 TTGTCTTTCCGATACCTCCCA

target id: NTTN90_mRNA_82331_cds

516 5'-AGGGATGGGTGGCATCGGTAAGACAACTTTA-3' 546 cleavage site: 531

|||| || ||||| |||||||

3'-ACCCTCCATAGCCTTTCTGTT-5'

DysRNA_arrange_TPM 43513291_22 TTTGTCTTTCCGATGCCTCCCT

target id: NTTN90_mRNA_82331_cds

516 5'-AGGGATGGGTGGCATCGGTAAGACAACTTTAG-3' 547 cleavage site: 532

||| |||||||| |||||||

3'-TCCCTCCGTAGCCTTTCTGTTT-5'

DysRNA_arrange_TPM 15928038_22 ATAGAATAATTATGAATGTGCT

target id: NTBX_mRNA_124800_cds

2178 5'-ATCATGTCGCATTCATAATTA-TCTATATCAT-3' 2208 cleavage site: 2193

|o|||||||||||| |||||

3'-TCGTGTAAGTATTAATAAGATA-5'

DysRNA_arrange_TPM 40742547_22 TTACCTGAACCTGGCATACCAA

target id: NTTN90_mRNA_37813_cds

1449 5'-GATCACTGGTACGCCAGGTTCGGGTAAAACTA-3' 1480 cleavage site: 1465

||||| |||||||||o|||||

3'-AACCATACGGTCCAAGTCCATT-5'

DysRNA_arrange_TPM 40741736_21 TTACCTGAACCTGGCATACCA

target id: NTTN90_mRNA_37813_cds

1450 5'-ATCACTGGTACGCCAGGTTCGGGTAAAACTA-3' 1480 cleavage site: 1465

||||| |||||||||o|||||

3'-ACCATACGGTCCAAGTCCATT-5'

DysRNA_arrange_TPM 40740236_21 TTACCTGAACCTGGCATACCT

target id: NTTN90_mRNA_37813_cds

1450 5'-ATCACTGGTACGCCAGGTTCGGGTAAAACTA-3' 1480 cleavage site: 1465

|||| |||||||||o|||||

3'-TCCATACGGTCCAAGTCCATT-5'

DysRNA_arrange_TPM 40741593_20 TTACCTGAACCTGGCATACC

target id: NTTN90_mRNA_37813_cds

1451 5'-TCACTGGTACGCCAGGTTCGGGTAAAACTA-3' 1480 cleavage site: 1465

|||| |||||||||o|||||

3'-CCATACGGTCCAAGTCCATT-5'

DysRNA_arrange_TPM 40742348_23 TTACCTGAACCTGGCATACCAAA

target id: NTTN90_mRNA_37813_cds

1448 5'-CGATCACTGGTACGCCAGGTTCGGGTAAAACTA-3' 1480 cleavage site: 1465

||||| |||||||||o|||||

3'-AAACCATACGGTCCAAGTCCATT-5'

DysRNA_arrange_TPM 40740693_23 TTACCTGAACCTGGCATACCAAT

target id: NTTN90_mRNA_37813_cds

1448 5'-CGATCACTGGTACGCCAGGTTCGGGTAAAACTA-3' 1480 cleavage site: 1465

||||| |||||||||o|||||

3'-TAACCATACGGTCCAAGTCCATT-5'

DysRNA_arrange_TPM 35450798_21 TACCTGAACCTGGCATACCAA

target id: NTTN90_mRNA_37813_cds

1449 5'-GATCACTGGTACGCCAGGTTCGGGTAAAACT-3' 1479 cleavage site: 1464

||||| |||||||||o||||

3'-AACCATACGGTCCAAGTCCAT-5'

DysRNA_arrange_TPM 40740134_22 TTACCTGAACCTGGCATACCAT

target id: NTTN90_mRNA_37813_cds

1449 5'-GATCACTGGTACGCCAGGTTCGGGTAAAACTA-3' 1480 cleavage site: 1465

||||| |||||||||o|||||

3'-TACCATACGGTCCAAGTCCATT-5'

DysRNA_arrange_TPM 40741540_22 TTACCTGAACCTGGCATACCTT

target id: NTTN90_mRNA_37813_cds

1449 5'-GATCACTGGTACGCCAGGTTCGGGTAAAACTA-3' 1480 cleavage site: 1465

|||| |||||||||o|||||

3'-TTCCATACGGTCCAAGTCCATT-5'

DysRNA_arrange_TPM 40742481_23 TTACCTGAACCTGGCATACCATT

target id: NTTN90_mRNA_37813_cds

1448 5'-CGATCACTGGTACGCCAGGTTCGGGTAAAACTA-3' 1480 cleavage site: 1465

||||| |||||||||o|||||

3'-TTACCATACGGTCCAAGTCCATT-5'

DysRNA_arrange_TPM 40741489_20 TTACCTGAACCTGGCATACT

target id: NTTN90_mRNA_37813_cds

1451 5'-TCACTGGTACGCCAGGTTCGGGTAAAACTA-3' 1480 cleavage site: 1465

o||| |||||||||o|||||

3'-TCATACGGTCCAAGTCCATT-5'

DysRNA_arrange_TPM 40742536_21 TTACCTGAACCTGGCATACCG

target id: NTTN90_mRNA_37813_cds

1450 5'-ATCACTGGTACGCCAGGTTCGGGTAAAACTA-3' 1480 cleavage site: 1465

o|||| |||||||||o|||||

3'-GCCATACGGTCCAAGTCCATT-5'

DysRNA_arrange_TPM 40742033_21 TTACCTGAACCTGGCATACCC

target id: NTTN90_mRNA_37813_cds

1450 5'-ATCACTGGTACGCCAGGTTCGGGTAAAACTA-3' 1480 cleavage site: 1465

|||| |||||||||o|||||

3'-CCCATACGGTCCAAGTCCATT-5'

DysRNA_arrange_TPM 40739408_23 TTACCTGAACCTGGCATACCAAG

target id: NTTN90_mRNA_37813_cds

1448 5'-CGATCACTGGTACGCCAGGTTCGGGTAAAACTA-3' 1480 cleavage site: 1465

||||| |||||||||o|||||

3'-GAACCATACGGTCCAAGTCCATT-5'

DysRNA_arrange_TPM 43050316_22 TTTACCTGAACCTGGCATACCA

target id: NTTN90_mRNA_37813_cds

1450 5'-ATCACTGGTACGCCAGGTTCGGGTAAAACTAC-3' 1481 cleavage site: 1466

||||| |||||||||o||||||

3'-ACCATACGGTCCAAGTCCATTT-5'

DysRNA_arrange_TPM 40741174_22 TTACCTGAACCTGGCATACCAG

target id: NTTN90_mRNA_37813_cds

1449 5'-GATCACTGGTACGCCAGGTTCGGGTAAAACTA-3' 1480 cleavage site: 1465

|||||| |||||||||o|||||

3'-GACCATACGGTCCAAGTCCATT-5'

DysRNA_arrange_TPM 43050143_24 TTTACCTGAACCTGGCATACCAAC

target id: NTTN90_mRNA_37813_cds

1448 5'-CGATCACTGGTACGCCAGGTTCGGGTAAAACTAC-3' 1481 cleavage site: 1466

||||| |||||||||o||||||

3'-CAACCATACGGTCCAAGTCCATTT-5'

DysRNA_arrange_TPM 35450403_20 TACCTGAACCTGGCATACCA

target id: NTTN90_mRNA_37813_cds

1450 5'-ATCACTGGTACGCCAGGTTCGGGTAAAACT-3' 1479 cleavage site: 1464

||||| |||||||||o||||

3'-ACCATACGGTCCAAGTCCAT-5'

DysRNA_arrange_TPM 40739753_22 TTACCTGAACCTGGCATACCTA

target id: NTTN90_mRNA_37813_cds

1449 5'-GATCACTGGTACGCCAGGTTCGGGTAAAACTA-3' 1480 cleavage site: 1465

|||| |||||||||o|||||

3'-ATCCATACGGTCCAAGTCCATT-5'

DysRNA_arrange_TPM 40742347_21 TTACCTGAACCTGGCATACTT

target id: NTTN90_mRNA_37813_cds

1450 5'-ATCACTGGTACGCCAGGTTCGGGTAAAACTA-3' 1480 cleavage site: 1465

o||| |||||||||o|||||

3'-TTCATACGGTCCAAGTCCATT-5'

DysRNA_arrange_TPM 40741291_23 TTACCTGAACCTGGCATACCATC

target id: NTTN90_mRNA_37813_cds

1448 5'-CGATCACTGGTACGCCAGGTTCGGGTAAAACTA-3' 1480 cleavage site: 1465

||||| |||||||||o|||||

3'-CTACCATACGGTCCAAGTCCATT-5'

DysRNA_arrange_TPM 40739574_21 TTACCTGAACCTGGCATACTA

target id: NTTN90_mRNA_37813_cds

1450 5'-ATCACTGGTACGCCAGGTTCGGGTAAAACTA-3' 1480 cleavage site: 1465

|o||| |||||||||o|||||

3'-ATCATACGGTCCAAGTCCATT-5'

DysRNA_arrange_TPM 40742607_22 TTACCTGAACCTGGTATACCAA

target id: NTTN90_mRNA_37813_cds

1449 5'-GATCACTGGTACGCCAGGTTCGGGTAAAACTA-3' 1480 cleavage site: 1465

||||| o||||||||o|||||

3'-AACCATATGGTCCAAGTCCATT-5'

DysRNA_arrange_TPM 40665598_22 TTACATGAACCTGGCATACCAA

target id: NTTN90_mRNA_37813_cds

1449 5'-GATCACTGGTACGCCAGGTTCGGGTAAAACTA-3' 1480 cleavage site: 1465

||||| |||||||||o ||||

3'-AACCATACGGTCCAAGTACATT-5'

DysRNA_arrange_TPM 43050825_21 TTTACCTGAACCTGGCATACC

target id: NTTN90_mRNA_37813_cds

1451 5'-TCACTGGTACGCCAGGTTCGGGTAAAACTAC-3' 1481 cleavage site: 1466

|||| |||||||||o||||||

3'-CCATACGGTCCAAGTCCATTT-5'

DysRNA_arrange_TPM 40741435_21 TTACCTGAACCTGACATACCA

target id: NTTN90_mRNA_37813_cds

1450 5'-ATCACTGGTACGCCAGGTTCGGGTAAAACTA-3' 1480 cleavage site: 1465

||||| | |||||||o|||||

3'-ACCATACAGTCCAAGTCCATT-5'

DysRNA_arrange_TPM 40742312_23 TTACCTGAACCTGGCATACCAAC

target id: NTTN90_mRNA_37813_cds

1448 5'-CGATCACTGGTACGCCAGGTTCGGGTAAAACTA-3' 1480 cleavage site: 1465

||||| |||||||||o|||||

3'-CAACCATACGGTCCAAGTCCATT-5'

DysRNA_arrange_TPM 40739464_22 TTACCTGAACCTGGCATACCAC

target id: NTTN90_mRNA_37813_cds

1449 5'-GATCACTGGTACGCCAGGTTCGGGTAAAACTA-3' 1480 cleavage site: 1465

||||| |||||||||o|||||

3'-CACCATACGGTCCAAGTCCATT-5'

DysRNA_arrange_TPM 40740425_22 TTACCTGAACCTGACATACCAA

target id: NTTN90_mRNA_37813_cds

1449 5'-GATCACTGGTACGCCAGGTTCGGGTAAAACTA-3' 1480 cleavage site: 1465

||||| | |||||||o|||||

3'-AACCATACAGTCCAAGTCCATT-5'

DysRNA_arrange_TPM 35450164_20 TACCTGAACCTGGCATACCT

target id: NTTN90_mRNA_37813_cds

1450 5'-ATCACTGGTACGCCAGGTTCGGGTAAAACT-3' 1479 cleavage site: 1464

|||| |||||||||o||||

3'-TCCATACGGTCCAAGTCCAT-5'

DysRNA_arrange_TPM 43050821_23 TTTACCTGAACCTGGCATACCAA

target id: NTTN90_mRNA_37813_cds

1449 5'-GATCACTGGTACGCCAGGTTCGGGTAAAACTAC-3' 1481 cleavage site: 1466

||||| |||||||||o||||||

3'-AACCATACGGTCCAAGTCCATTT-5'

DysRNA_arrange_TPM 40740942_22 TTACCTGAACCTGGCATAACAA

target id: NTTN90_mRNA_37813_cds

1449 5'-GATCACTGGTACGCCAGGTTCGGGTAAAACTA-3' 1480 cleavage site: 1465

|| || |||||||||o|||||

3'-AACAATACGGTCCAAGTCCATT-5'

DysRNA_arrange_TPM 40741495_22 TTACCTGAACCTGGCATACCTC

target id: NTTN90_mRNA_37813_cds

1449 5'-GATCACTGGTACGCCAGGTTCGGGTAAAACTA-3' 1480 cleavage site: 1465

|||| |||||||||o|||||

3'-CTCCATACGGTCCAAGTCCATT-5'

DysRNA_arrange_TPM 43983812_21 TTTTACCTGAACCTGGCATAC

target id: NTTN90_mRNA_37813_cds

1452 5'-CACTGGTACGCCAGGTTCGGGTAAAACTACT-3' 1482 cleavage site: 1467

||| |||||||||o|||||||

3'-CATACGGTCCAAGTCCATTTT-5'

DysRNA_arrange_TPM 40741138_22 TTACCTGAACCTGGCATACTTT

target id: NTTN90_mRNA_37813_cds

1449 5'-GATCACTGGTACGCCAGGTTCGGGTAAAACTA-3' 1480 cleavage site: 1465

o||| |||||||||o|||||

3'-TTTCATACGGTCCAAGTCCATT-5'

DysRNA_arrange_TPM 40740203_21 TTACCTGAACCTGGCATACTC

target id: NTTN90_mRNA_37813_cds

1450 5'-ATCACTGGTACGCCAGGTTCGGGTAAAACTA-3' 1480 cleavage site: 1465

o||| |||||||||o|||||

3'-CTCATACGGTCCAAGTCCATT-5'

DysRNA_arrange_TPM 40740924_20 TTACCTGAACCTGGCATACA

target id: NTTN90_mRNA_37813_cds

1451 5'-TCACTGGTACGCCAGGTTCGGGTAAAACTA-3' 1480 cleavage site: 1465

||| |||||||||o|||||

3'-ACATACGGTCCAAGTCCATT-5'

DysRNA_arrange_TPM 40742510_22 TTACCTGAACCTGGCATTCCAA

target id: NTTN90_mRNA_37813_cds

1449 5'-GATCACTGGTACGCCAGGTTCGGGTAAAACTA-3' 1480 cleavage site: 1465

||| | |||||||||o|||||

3'-AACCTTACGGTCCAAGTCCATT-5'

DysRNA_arrange_TPM 40740451_21 TTACCTGAACCTGGCATATCA

target id: NTTN90_mRNA_37813_cds

1450 5'-ATCACTGGTACGCCAGGTTCGGGTAAAACTA-3' 1480 cleavage site: 1465

||o|| |||||||||o|||||

3'-ACTATACGGTCCAAGTCCATT-5'

DysRNA_arrange_TPM 40738063_22 TTACCTAAACCTGGCATACCAA

target id: NTTN90_mRNA_37813_cds

1449 5'-GATCACTGGTACGCCAGGTTCGGGTAAAACTA-3' 1480 cleavage site: 1465

||||| |||||||| o|||||

3'-AACCATACGGTCCAAATCCATT-5'

DysRNA_arrange_TPM 34428539_21 TAACCTGAACCTGGCATACCA

target id: NTTN90_mRNA_37813_cds

1450 5'-ATCACTGGTACGCCAGGTTCGGGTAAAACTA-3' 1480 cleavage site: 1465

||||| |||||||||o|||

3'-ACCATACGGTCCAAGTCCAAT-5'

DysRNA_arrange_TPM 40745342_22 TTACCTCAACCTGGCATACCAA

target id: NTTN90_mRNA_37813_cds

1449 5'-GATCACTGGTACGCCAGGTTCGGGTAAAACTA-3' 1480 cleavage site: 1465

||||| |||||||| o|||||

3'-AACCATACGGTCCAACTCCATT-5'

DysRNA_arrange_TPM 40747677_21 TTACCTTAACCTGGCATACCA

target id: NTTN90_mRNA_37813_cds

1450 5'-ATCACTGGTACGCCAGGTTCGGGTAAAACTA-3' 1480 cleavage site: 1465

||||| |||||||| o|||||

3'-ACCATACGGTCCAATTCCATT-5'

DysRNA_arrange_TPM 40737853_21 TTACCTAAACCTGGCATACCA

target id: NTTN90_mRNA_37813_cds

1450 5'-ATCACTGGTACGCCAGGTTCGGGTAAAACTA-3' 1480 cleavage site: 1465

||||| |||||||| o|||||

3'-ACCATACGGTCCAAATCCATT-5'

DysRNA_arrange_TPM 40739905_22 TTACCTGAACCTGGCATATCAA

target id: NTTN90_mRNA_37813_cds

1449 5'-GATCACTGGTACGCCAGGTTCGGGTAAAACTA-3' 1480 cleavage site: 1465

||o|| |||||||||o|||||

3'-AACTATACGGTCCAAGTCCATT-5'

DysRNA_arrange_TPM 40740398_22 TTACCTGAACCTGGCATAGCAA

target id: NTTN90_mRNA_37813_cds

1449 5'-GATCACTGGTACGCCAGGTTCGGGTAAAACTA-3' 1480 cleavage site: 1465

|| || |||||||||o|||||

3'-AACGATACGGTCCAAGTCCATT-5'

DysRNA_arrange_TPM 26914223_21 GTACCTGAACCTGGCATACCT

target id: NTTN90_mRNA_37813_cds

1450 5'-ATCACTGGTACGCCAGGTTCGGGTAAAACTA-3' 1480 cleavage site: 1465

|||| |||||||||o||||

3'-TCCATACGGTCCAAGTCCATG-5'

DysRNA_arrange_TPM 26914450_20 GTACCTGAACCTGGCATACC

target id: NTTN90_mRNA_37813_cds

1451 5'-TCACTGGTACGCCAGGTTCGGGTAAAACTA-3' 1480 cleavage site: 1465

|||| |||||||||o||||

3'-CCATACGGTCCAAGTCCATG-5'

DysRNA_arrange_TPM 40741207_20 TTACCTGAACCTGGCATTTC

target id: NTTN90_mRNA_37813_cds

1451 5'-TCACTGGTACGCCAGGTTCGGGTAAAACTA-3' 1480 cleavage site: 1465

|o | |||||||||o|||||

3'-CTTTACGGTCCAAGTCCATT-5'

DysRNA_arrange_TPM 26914545_21 GTACCTGAACCTGGCATACCA

target id: NTTN90_mRNA_37813_cds

1450 5'-ATCACTGGTACGCCAGGTTCGGGTAAAACTA-3' 1480 cleavage site: 1465

||||| |||||||||o||||

3'-ACCATACGGTCCAAGTCCATG-5'

DysRNA_arrange_TPM 40741304_22 TTACCTGAACCTGGCATCCCAA

target id: NTTN90_mRNA_37813_cds

1449 5'-GATCACTGGTACGCCAGGTTCGGGTAAAACTA-3' 1480 cleavage site: 1465

||| | |||||||||o|||||

3'-AACCCTACGGTCCAAGTCCATT-5'

DysRNA_arrange_TPM 40741202_22 TTACCTGAACCTGGCATACTAA

target id: NTTN90_mRNA_37813_cds

1449 5'-GATCACTGGTACGCCAGGTTCGGGTAAAACTA-3' 1480 cleavage site: 1465

|o||| |||||||||o|||||

3'-AATCATACGGTCCAAGTCCATT-5'

DysRNA_arrange_TPM 16439463_22 ATACCTGAACCTGGCATACCAA

target id: NTTN90_mRNA_37813_cds

1449 5'-GATCACTGGTACGCCAGGTTCGGGTAAAACTA-3' 1480 cleavage site: 1465

||||| |||||||||o||||

3'-AACCATACGGTCCAAGTCCATA-5'

DysRNA_arrange_TPM 35451289_22 TACCTGAACCTGGCATACCAAA

target id: NTTN90_mRNA_37813_cds

1448 5'-CGATCACTGGTACGCCAGGTTCGGGTAAAACT-3' 1479 cleavage site: 1464

||||| |||||||||o||||

3'-AAACCATACGGTCCAAGTCCAT-5'

DysRNA_arrange_TPM 40741342_22 TTACCTGAACCTGGCACACCAA

target id: NTTN90_mRNA_37813_cds

1449 5'-GATCACTGGTACGCCAGGTTCGGGTAAAACTA-3' 1480 cleavage site: 1465

|||| |||||||||o|||||

3'-AACCACACGGTCCAAGTCCATT-5'

DysRNA_arrange_TPM 40739499_22 TTACCTGAACCTGTCATACCAA

target id: NTTN90_mRNA_37813_cds

1449 5'-GATCACTGGTACGCCAGGTTCGGGTAAAACTA-3' 1480 cleavage site: 1465

||||| | |||||||o|||||

3'-AACCATACTGTCCAAGTCCATT-5'

DysRNA_arrange_TPM 35450589_22 TACCTGAACCTGGCATACCAAT

target id: NTTN90_mRNA_37813_cds

1448 5'-CGATCACTGGTACGCCAGGTTCGGGTAAAACT-3' 1479 cleavage site: 1464

||||| |||||||||o||||

3'-TAACCATACGGTCCAAGTCCAT-5'

DysRNA_arrange_TPM 40741351_22 TTACCTGAACCTGGCGTACCAA

target id: NTTN90_mRNA_37813_cds

1449 5'-GATCACTGGTACGCCAGGTTCGGGTAAAACTA-3' 1480 cleavage site: 1465

|||||||||||||||o|||||

3'-AACCATGCGGTCCAAGTCCATT-5'

DysRNA_arrange_TPM 40717233_22 TTACCGGAACCTGGCATACCAA

target id: NTTN90_mRNA_37813_cds

1449 5'-GATCACTGGTACGCCAGGTTCGGGTAAAACTA-3' 1480 cleavage site: 1465

||||| ||||||||| |||||

3'-AACCATACGGTCCAAGGCCATT-5'

DysRNA_arrange_TPM 40747954_22 TTACCTTAACCTGGCATACCAA

target id: NTTN90_mRNA_37813_cds

1449 5'-GATCACTGGTACGCCAGGTTCGGGTAAAACTA-3' 1480 cleavage site: 1465

||||| |||||||| o|||||

3'-AACCATACGGTCCAATTCCATT-5'

DysRNA_arrange_TPM 36635486_21 TGACCTGAACCTGGCATACCA

target id: NTTN90_mRNA_37813_cds

1450 5'-ATCACTGGTACGCCAGGTTCGGGTAAAACTA-3' 1480 cleavage site: 1465

||||| |||||||||o|||

3'-ACCATACGGTCCAAGTCCAGT-5'

DysRNA_arrange_TPM 40741331_21 TTACCTGAACCTGGCATCCCT

target id: NTTN90_mRNA_37813_cds

1450 5'-ATCACTGGTACGCCAGGTTCGGGTAAAACTA-3' 1480 cleavage site: 1465

|| | |||||||||o|||||

3'-TCCCTACGGTCCAAGTCCATT-5'

DysRNA_arrange_TPM 26914581_22 GTACCTGAACCTGGCATACCAA

target id: NTTN90_mRNA_37813_cds

1449 5'-GATCACTGGTACGCCAGGTTCGGGTAAAACTA-3' 1480 cleavage site: 1465

||||| |||||||||o||||

3'-AACCATACGGTCCAAGTCCATG-5'

DysRNA_arrange_TPM 40742285_22 TTACCTGAACCTGGCATACTTC

target id: NTTN90_mRNA_37813_cds

1449 5'-GATCACTGGTACGCCAGGTTCGGGTAAAACTA-3' 1480 cleavage site: 1465

o||| |||||||||o|||||

3'-CTTCATACGGTCCAAGTCCATT-5'

DysRNA_arrange_TPM 40741688_22 TTACCTGAACCTGGCATGCCAA

target id: NTTN90_mRNA_37813_cds

1449 5'-GATCACTGGTACGCCAGGTTCGGGTAAAACTA-3' 1480 cleavage site: 1465

|||o| |||||||||o|||||

3'-AACCGTACGGTCCAAGTCCATT-5'

DysRNA_arrange_TPM 40727231_22 TTACCCGAACCTGGCATACCAA

target id: NTTN90_mRNA_37813_cds

1449 5'-GATCACTGGTACGCCAGGTTCGGGTAAAACTA-3' 1480 cleavage site: 1465

||||| |||||||||||||||

3'-AACCATACGGTCCAAGCCCATT-5'

DysRNA_arrange_TPM 40745543_21 TTACCTCAACCTGGCATACCA

target id: NTTN90_mRNA_37813_cds

1450 5'-ATCACTGGTACGCCAGGTTCGGGTAAAACTA-3' 1480 cleavage site: 1465

||||| |||||||| o|||||

3'-ACCATACGGTCCAACTCCATT-5'

DysRNA_arrange_TPM 40785222_22 TTACTTGAACCTGGCATACCAA

target id: NTTN90_mRNA_37813_cds

1449 5'-GATCACTGGTACGCCAGGTTCGGGTAAAACTA-3' 1480 cleavage site: 1465

||||| |||||||||oo||||

3'-AACCATACGGTCCAAGTTCATT-5'

DysRNA_arrange_TPM 40741094_23 TTACCTGAACCTGGCATACCACT

target id: NTTN90_mRNA_37813_cds

1448 5'-CGATCACTGGTACGCCAGGTTCGGGTAAAACTA-3' 1480 cleavage site: 1465

||||| |||||||||o|||||

3'-TCACCATACGGTCCAAGTCCATT-5'

DysRNA_arrange_TPM 40739650_22 TTACCTGAACCTGGCTTACCAA

target id: NTTN90_mRNA_37813_cds

1449 5'-GATCACTGGTACGCCAGGTTCGGGTAAAACTA-3' 1480 cleavage site: 1465

||||| |||||||||o|||||

3'-AACCATTCGGTCCAAGTCCATT-5'

DysRNA_arrange_TPM 40932757_22 TTATCTGAACCTGGCATACCAA

target id: NTTN90_mRNA_37813_cds

1449 5'-GATCACTGGTACGCCAGGTTCGGGTAAAACTA-3' 1480 cleavage site: 1465

||||| |||||||||o|o|||

3'-AACCATACGGTCCAAGTCTATT-5'

DysRNA_arrange_TPM 40740779_22 TTACCTGAACCTGGCATACCCT

target id: NTTN90_mRNA_37813_cds

1449 5'-GATCACTGGTACGCCAGGTTCGGGTAAAACTA-3' 1480 cleavage site: 1465

|||| |||||||||o|||||

3'-TCCCATACGGTCCAAGTCCATT-5'

DysRNA_arrange_TPM 27959210_20 GTTGTTTTGAGTTTGAGGCC

target id: NTTN90_mRNA_81051_cds

1349 5'-TTGATTGCATTAAACTCAAAA-AATTGCCA-3' 1377 cleavage site: 1362

|| |o|||||||||| ||o

3'-CCGGAGTTTGAGTTTTGTTG-5'

DysRNA_arrange_TPM 43983812_21 TTTTACCTGAACCTGGCATAC

target id: NTTN90_mRNA_63335_cds

1926 5'-CACTGGCATGCCGGGTTCGGGTAAAACTACT-3' 1956 cleavage site: 1941

|||||o|||||o|||||||

3'-CATACGGTCCAAGTCCATTTT-5'

DysRNA_arrange_TPM 40741688_22 TTACCTGAACCTGGCATGCCAA

target id: NTTN90_mRNA_63335_cds

1923 5'-GATCACTGGCATGCCGGGTTCGGGTAAAACTA-3' 1954 cleavage site: 1939

|||||||||o|||||o|||||

3'-AACCGTACGGTCCAAGTCCATT-5'

DysRNA_arrange_TPM 40727231_22 TTACCCGAACCTGGCATACCAA

target id: NTTN90_mRNA_63335_cds

1923 5'-GATCACTGGCATGCCGGGTTCGGGTAAAACTA-3' 1954 cleavage site: 1939

||| |||||o|||||||||||

3'-AACCATACGGTCCAAGCCCATT-5'

DysRNA_arrange_TPM 40741702_22 TTACCTGAACCCGGCATACCAA

target id: NTTN90_mRNA_63335_cds

1923 5'-GATCACTGGCATGCCGGGTTCGGGTAAAACTA-3' 1954 cleavage site: 1939

||| |||||||||||o|||||

3'-AACCATACGGCCCAAGTCCATT-5'

DysRNA_arrange_TPM 37959495_22 TCAAATCCAATAGTAGGTACCA

target id: NTTN90_mRNA_59866_cds

2370 5'-ATTGTTGGAATCT-TTATCGGATTTGAGATTG-3' 2400 cleavage site: 2385

||| |o|| o||| ||||||||

3'-ACCATGGATGATAACCTAAACT-5'

DysRNA_arrange_TPM 37959584_22 TCAAATCCGATAAGGGGCTCCA

target id: NTTN90_mRNA_59866_cds

2370 5'-ATTGTTGGA-ATCTTTATCGGATTTGAGATTG-3' 2400 cleavage site: 2385

|||| o|o|||||||||||||

3'-ACCTCGGGGAATAGCCTAAACT-5'

DysRNA_arrange_TPM 28460746_21 CAAATCCGATAAGGGGCTCCA

target id: NTTN90_mRNA_59866_cds

2370 5'-ATTGTTGGA-ATCTTTATCGGATTTGAGATT-3' 2399 cleavage site: 2384

|||| o|o||||||||||||

3'-ACCTCGGGGAATAGCCTAAAC-5'

DysRNA_arrange_TPM 02453693_20 AAATCCGATAAGGGGCTCCA

target id: NTTN90_mRNA_59866_cds

2370 5'-ATTGTTGGA-ATCTTTATCGGATTTGAGAT-3' 2398 cleavage site: 2383

|||| o|o|||||||||||

3'-ACCTCGGGGAATAGCCTAAA-5'

DysRNA_arrange_TPM 43983812_21 TTTTACCTGAACCTGGCATAC

target id: NTK326_mRNA_53814_cds

1380 5'-CACTGGCATGCCGGGTTCGGGTAAAACTACT-3' 1410 cleavage site: 1395

|||||o|||||o|||||||

3'-CATACGGTCCAAGTCCATTTT-5'

DysRNA_arrange_TPM 40741688_22 TTACCTGAACCTGGCATGCCAA

target id: NTK326_mRNA_53814_cds

1377 5'-GATCACTGGCATGCCGGGTTCGGGTAAAACTA-3' 1408 cleavage site: 1393

|||||||||o|||||o|||||

3'-AACCGTACGGTCCAAGTCCATT-5'

DysRNA_arrange_TPM 40727231_22 TTACCCGAACCTGGCATACCAA

target id: NTK326_mRNA_53814_cds

1377 5'-GATCACTGGCATGCCGGGTTCGGGTAAAACTA-3' 1408 cleavage site: 1393

||| |||||o|||||||||||

3'-AACCATACGGTCCAAGCCCATT-5'

DysRNA_arrange_TPM 40741702_22 TTACCTGAACCCGGCATACCAA

target id: NTK326_mRNA_53814_cds

1377 5'-GATCACTGGCATGCCGGGTTCGGGTAAAACTA-3' 1408 cleavage site: 1393

||| |||||||||||o|||||

3'-AACCATACGGCCCAAGTCCATT-5'

DysRNA_arrange_TPM 20608921_24 ATTCTAGATCTTCTTTCACCAGTC

target id: NTTN90_mRNA_37809_cds

1122 5'-AATTGGACTGGTGAAAGAAGATCTAGAATTCATA-3' 1155 cleavage site: 1140

||||||||||||||||||||||||

3'-CTGACCACTTTCTTCTAGATCTTA-5'

DysRNA_arrange_TPM 40506628_21 TTAGATCTTCTTTCACCAGCT

target id: NTTN90_mRNA_37809_cds

1121 5'-AAATTGGACTGGTGAAAGAAGATCTAGAATTC-3' 1152 cleavage site: 1137

o| ||||||||||||||||||o

3'-TC-GACCACTTTCTTCTAGATT-5'

DysRNA_arrange_TPM 27959210_20 GTTGTTTTGAGTTTGAGGCC

target id: NTBX_mRNA_122255_cds

1247 5'-TTAATTGCATTAAACTCAAAA-AATTGCCA-3' 1275 cleavage site: 1260

|| |o|||||||||| ||o

3'-CCGGAGTTTGAGTTTTGTTG-5'

DysRNA_arrange_TPM 27959210_20 GTTGTTTTGAGTTTGAGGCC

target id: NTK326_mRNA_121252_cds

1097 5'-TTAATTGCATTAAACTCAAAA-AATTGCCA-3' 1125 cleavage site: 1110

|| |o|||||||||| ||o

3'-CCGGAGTTTGAGTTTTGTTG-5'

DysRNA_arrange_TPM 27959210_20 GTTGTTTTGAGTTTGAGGCC

target id: NTTN90_mRNA_134534_cds

1413 5'-TTAATTGCATTAAACTCAAAA-AATTGCCA-3' 1441 cleavage site: 1426

|| |o|||||||||| ||o

3'-CCGGAGTTTGAGTTTTGTTG-5'

DysRNA_arrange_TPM 43319357_21 TTTGGATTGAAGGGAGCTCTA

target id: NTTN90_mRNA_87634_cds

112 5'-TCTGGTACAG-TTTCTTCAATCCAAGCTGTA-3' 141 cleavage site: 126

|| || |oo|||||||||||o

3'-ATCTCGAGGGAAGTTAGGTTT-5'

DysRNA_arrange_TPM 41411013_20 TTGGATTGAAGGGAGCTCTA

target id: NTTN90_mRNA_87634_cds

112 5'-TCTGGTACAG-TTTCTTCAATCCAAGCTGT-3' 140 cleavage site: 125

|| || |oo|||||||||||

3'-ATCTCGAGGGAAGTTAGGTT-5'

DysRNA_arrange_TPM 43319585_22 TTTGGATTGAAGGGAGCTCTAT

target id: NTTN90_mRNA_87634_cds

111 5'-TTCTGGTACAG-TTTCTTCAATCCAAGCTGTA-3' 141 cleavage site: 126

o|| || |oo|||||||||||o

3'-TATCTCGAGGGAAGTTAGGTTT-5'

DysRNA_arrange_TPM 43316465_21 TTTGGATTGAAGGGAGCTCTG

target id: NTTN90_mRNA_87634_cds

112 5'-TCTGGTACAG-TTTCTTCAATCCAAGCTGTA-3' 141 cleavage site: 126

o| || |oo|||||||||||o

3'-GTCTCGAGGGAAGTTAGGTTT-5'

DysRNA_arrange_TPM 43316124_21 TTTGGATTGAAGGGGGCTCTA

target id: NTTN90_mRNA_87634_cds

112 5'-TCTGGTACAG-TTTCTTCAATCCAAGCTGTA-3' 141 cleavage site: 126

|| || ooo|||||||||||o

3'-ATCTCGGGGGAAGTTAGGTTT-5'

DysRNA_arrange_TPM 43315582_22 TTTGGATTGAAGGGAGCTCTAC

target id: NTTN90_mRNA_87634_cds

111 5'-TTCTGGTACAG-TTTCTTCAATCCAAGCTGTA-3' 141 cleavage site: 126

||| || |oo|||||||||||o

3'-CATCTCGAGGGAAGTTAGGTTT-5'

DysRNA_arrange_TPM 43321659_21 TTTGGATTGAAGGAAGCTCTA

target id: NTTN90_mRNA_87634_cds

112 5'-TCTGGTACAG-TTTCTTCAATCCAAGCTGTA-3' 141 cleavage site: 126

|| || ||o|||||||||||o

3'-ATCTCGAAGGAAGTTAGGTTT-5'

DysRNA_arrange_TPM 43317025_21 TTTGGATTGAAGGTAGCTCTA

target id: NTTN90_mRNA_87634_cds

112 5'-TCTGGTACAGTT-TCTTCAATCCAAGCTGTA-3' 141 cleavage site: 126

|| ||o| o|||||||||||o

3'-ATCTCGATGGAAGTTAGGTTT-5'

DysRNA_arrange_TPM 43320131_21 TTTGGATTGAAGTGAGCTCTA

target id: NTTN90_mRNA_87634_cds

112 5'-TCTGGTACAGTTT-CTTCAATCCAAGCTGTA-3' 141 cleavage site: 126

|| ||o|o |||||||||||o

3'-ATCTCGAGTGAAGTTAGGTTT-5'

DysRNA_arrange_TPM 43320956_21 TTTGGATTGAAGGGAGCTGTA

target id: NTTN90_mRNA_87634_cds

112 5'-TCTGGTACAG-TTTCTTCAATCCAAGCTGTA-3' 141 cleavage site: 126

||||| |oo|||||||||||o

3'-ATGTCGAGGGAAGTTAGGTTT-5'

DysRNA_arrange_TPM 43317682_21 TTTGGATTGAAGGGAGCTATA

target id: NTTN90_mRNA_87634_cds

112 5'-TCTGGTACAG-TTTCTTCAATCCAAGCTGTA-3' 141 cleavage site: 126

|| || |oo|||||||||||o

3'-ATATCGAGGGAAGTTAGGTTT-5'

DysRNA_arrange_TPM 20002187_21 ATTGGATTGAAGGGAGCTCTA

target id: NTTN90_mRNA_87634_cds

112 5'-TCTGGTACAG-TTTCTTCAATCCAAGCTGTA-3' 141 cleavage site: 126

|| || |oo|||||||||||

3'-ATCTCGAGGGAAGTTAGGTTA-5'

DysRNA_arrange_TPM 43317086_21 TTTGGATTGAAGGGCGCTCTA

target id: NTTN90_mRNA_87634_cds

112 5'-TCTGGTACAGT-TTCTTCAATCCAAGCTGTA-3' 141 cleavage site: 126

|| ||o oo|||||||||||o

3'-ATCTCGCGGGAAGTTAGGTTT-5'

DysRNA_arrange_TPM 33578164_21 CTTGGATTGAAGGGAGCTCTA

target id: NTTN90_mRNA_87634_cds

112 5'-TCTGGTACAG-TTTCTTCAATCCAAGCTGTA-3' 141 cleavage site: 126

|| || |oo||||||||||||

3'-ATCTCGAGGGAAGTTAGGTTC-5'

DysRNA_arrange_TPM 43316328_21 TTTGGATTGAAGGGAGCTTTA

target id: NTTN90_mRNA_87634_cds

112 5'-TCTGGTACAG-TTTCTTCAATCCAAGCTGTA-3' 141 cleavage site: 126

|| || |oo|||||||||||o

3'-ATTTCGAGGGAAGTTAGGTTT-5'

DysRNA_arrange_TPM 43315028_21 TTTGGATTGAAGGGTGCTCTA

target id: NTTN90_mRNA_87634_cds

112 5'-TCTGGTACAGT-TTCTTCAATCCAAGCTGTA-3' 141 cleavage site: 126

|| ||o oo|||||||||||o

3'-ATCTCGTGGGAAGTTAGGTTT-5'

DysRNA_arrange_TPM 42289602_21 TTCGGATTGAAGGGAGCTCTA

target id: NTTN90_mRNA_87634_cds

112 5'-TCTGGTACAG-TTTCTTCAATCC-AAGCTGT-3' 140 cleavage site: 125

|| || |oo||||||||| ||

3'-ATCTCGAGGGAAGTTAGGCTT-5'

DysRNA_arrange_TPM 43317643_23 TTTGGATTGAAGGGAGCTCTACT

target id: NTTN90_mRNA_87634_cds

110 5'-TTTCTGGTACAG-TTTCTTCAATCCAAGCTGTA-3' 141 cleavage site: 126

o||| || |oo|||||||||||o

3'-TCATCTCGAGGGAAGTTAGGTTT-5'

DysRNA_arrange_TPM 43317291_21 TTTGGATTGAAGAGAGCTCTA

target id: NTTN90_mRNA_87634_cds

112 5'-TCTGGTACAG-TTTCTTCAATCCAAGCTGTA-3' 141 cleavage site: 126

|| || |o||||||||||||o

3'-ATCTCGAGAGAAGTTAGGTTT-5'

DysRNA_arrange_TPM 43317729_20 TTTGGATTGAAGGAGCTCTA

target id: NTTN90_mRNA_87634_cds

112 5'-TCTGGTACAGTTTCTTCAATCCAAGCTGTA-3' 141 cleavage site: 126

|| ||o|o|||||||||||o

3'-ATCTCGAGGAAGTTAGGTTT-5'

DysRNA_arrange_TPM 43316738_21 TTTGGATTGAAGGGAACTCTA

target id: NTTN90_mRNA_87634_cds

112 5'-TCTGGTACAG-TTTCTTCAATCCAAGCTGTA-3' 141 cleavage site: 126

|| || |oo|||||||||||o

3'-ATCTCAAGGGAAGTTAGGTTT-5'

DysRNA_arrange_TPM 40528105_21 TTAGGATTGAAGGGAGCTCTA

target id: NTTN90_mRNA_87634_cds

112 5'-TCTGGTACAG-TTTCTTCAATCC-AAGCTGT-3' 140 cleavage site: 125

|| || |oo||||||||| ||

3'-ATCTCGAGGGAAGTTAGGATT-5'

DysRNA_arrange_TPM 27871038_21 GTTGGATTGAAGGGAGCTCTA

target id: NTTN90_mRNA_87634_cds

112 5'-TCTGGTACAG-TTTCTTCAATCCAAGCTGTA-3' 141 cleavage site: 126

|| || |oo|||||||||||

3'-ATCTCGAGGGAAGTTAGGTTG-5'

DysRNA_arrange_TPM 43321070_21 TTTGGATTGAAGCGAGCTCTA

target id: NTTN90_mRNA_87634_cds

112 5'-TCTGGTACAGTTT-CTTCAATCCAAGCTGTA-3' 141 cleavage site: 126

|| ||o|o |||||||||||o

3'-ATCTCGAGCGAAGTTAGGTTT-5'

DysRNA_arrange_TPM 43983812_21 TTTTACCTGAACCTGGCATAC

target id: NTBX_mRNA_72663_cds

1488 5'-CACTGGCATGCCGGGTTCGGGTAAAACTACT-3' 1518 cleavage site: 1503

|||||o|||||o|||||||

3'-CATACGGTCCAAGTCCATTTT-5'

DysRNA_arrange_TPM 40741688_22 TTACCTGAACCTGGCATGCCAA

target id: NTBX_mRNA_72663_cds

1485 5'-GATCACTGGCATGCCGGGTTCGGGTAAAACTA-3' 1516 cleavage site: 1501

|||||||||o|||||o|||||

3'-AACCGTACGGTCCAAGTCCATT-5'

DysRNA_arrange_TPM 40727231_22 TTACCCGAACCTGGCATACCAA

target id: NTBX_mRNA_72663_cds

1485 5'-GATCACTGGCATGCCGGGTTCGGGTAAAACTA-3' 1516 cleavage site: 1501

||| |||||o|||||||||||

3'-AACCATACGGTCCAAGCCCATT-5'

DysRNA_arrange_TPM 40741702_22 TTACCTGAACCCGGCATACCAA

target id: NTBX_mRNA_72663_cds

1485 5'-GATCACTGGCATGCCGGGTTCGGGTAAAACTA-3' 1516 cleavage site: 1501

||| |||||||||||o|||||

3'-AACCATACGGCCCAAGTCCATT-5'

DysRNA_arrange_TPM 43983812_21 TTTTACCTGAACCTGGCATAC

target id: NTK326_mRNA_53821_cds

1167 5'-CACTGGCATGCCGGGTTCGGGTAAAACTACT-3' 1197 cleavage site: 1182

|||||o|||||o|||||||

3'-CATACGGTCCAAGTCCATTTT-5'

DysRNA_arrange_TPM 40741688_22 TTACCTGAACCTGGCATGCCAA

target id: NTK326_mRNA_53821_cds

1164 5'-GATCACTGGCATGCCGGGTTCGGGTAAAACTA-3' 1195 cleavage site: 1180

|||||||||o|||||o|||||

3'-AACCGTACGGTCCAAGTCCATT-5'

DysRNA_arrange_TPM 40727231_22 TTACCCGAACCTGGCATACCAA

target id: NTK326_mRNA_53821_cds

1164 5'-GATCACTGGCATGCCGGGTTCGGGTAAAACTA-3' 1195 cleavage site: 1180

||| |||||o|||||||||||

3'-AACCATACGGTCCAAGCCCATT-5'

DysRNA_arrange_TPM 40741702_22 TTACCTGAACCCGGCATACCAA

target id: NTK326_mRNA_53821_cds

1164 5'-GATCACTGGCATGCCGGGTTCGGGTAAAACTA-3' 1195 cleavage site: 1180

||| |||||||||||o|||||

3'-AACCATACGGCCCAAGTCCATT-5'

DysRNA_arrange_TPM 43983812_21 TTTTACCTGAACCTGGCATAC

target id: NTBX_mRNA_20754_cds

1884 5'-CACTGGCATGCCGGGTTCGGGTAAAACTACT-3' 1914 cleavage site: 1899

|||||o|||||o|||||||

3'-CATACGGTCCAAGTCCATTTT-5'

DysRNA_arrange_TPM 40741688_22 TTACCTGAACCTGGCATGCCAA

target id: NTBX_mRNA_20754_cds

1881 5'-GATCACTGGCATGCCGGGTTCGGGTAAAACTA-3' 1912 cleavage site: 1897

|||||||||o|||||o|||||

3'-AACCGTACGGTCCAAGTCCATT-5'

DysRNA_arrange_TPM 40727231_22 TTACCCGAACCTGGCATACCAA

target id: NTBX_mRNA_20754_cds

1881 5'-GATCACTGGCATGCCGGGTTCGGGTAAAACTA-3' 1912 cleavage site: 1897

||| |||||o|||||||||||

3'-AACCATACGGTCCAAGCCCATT-5'

DysRNA_arrange_TPM 40741702_22 TTACCTGAACCCGGCATACCAA

target id: NTBX_mRNA_20754_cds

1881 5'-GATCACTGGCATGCCGGGTTCGGGTAAAACTA-3' 1912 cleavage site: 1897

||| |||||||||||o|||||

3'-AACCATACGGCCCAAGTCCATT-5'

DysRNA_arrange_TPM 02767294_21 AAGAAGATGATTATGGAGGGC

target id: NTK326_mRNA_72003_cds

3471 5'-TCCAAGTGCTCCATCATCATCTTCTTCAGAT-3' 3501 cleavage site: 3486

|o |||||| |||||||||||

3'-CGGGAGGTATTAGTAGAAGAA-5'

DysRNA_arrange_TPM 02768834_22 AAGAAGATGATTATGGAGGGCA

target id: NTK326_mRNA_72003_cds

3470 5'-GTCCAAGTGCTCCATCATCATCTTCTTCAGAT-3' 3501 cleavage site: 3486

|o |||||| |||||||||||

3'-ACGGGAGGTATTAGTAGAAGAA-5'

DysRNA_arrange_TPM 40391668_20 TTAATATTTCAGCTCGAACT

target id: NTTN90_mRNA_46479_cds

715 5'-TGTGAAG-TT-A-CTGAAATATTAAATGCA-3' 741 cleavage site: 726

|| |o | ||||||||||||

3'-TCAAGCTCGACTTTATAATT-5'

DysRNA_arrange_TPM 20608921_24 ATTCTAGATCTTCTTTCACCAGTC

target id: NTTN90_mRNA_37810_cds

1095 5'-AATTGGACTGGTGAAAGAAGATCTAGAATTCATA-3' 1128 cleavage site: 1113

||||||||||||||||||||||||

3'-CTGACCACTTTCTTCTAGATCTTA-5'

DysRNA_arrange_TPM 40506628_21 TTAGATCTTCTTTCACCAGCT

target id: NTTN90_mRNA_37810_cds

1094 5'-AAATTGGACTGGTGAAAGAAGATCTAGAATTC-3' 1125 cleavage site: 1110

o| ||||||||||||||||||o

3'-TC-GACCACTTTCTTCTAGATT-5'

DysRNA_arrange_TPM 27046016_20 GTGAAATTCTTGGATCGTCT

target id: NTTN90_mRNA_42243_cds

1522 5'-GAGAGTGAT-ATCCAAGAATTTCGATTGAA-3' 1550 cleavage site: 1535

||o |||||||||||||o

3'-TCTGCTAGGTTCTTAAAGTG-5'

DysRNA_arrange_TPM 28228704_20 GTTTCCCGGAACACCCCCCA

target id: NTBX_mRNA_69716_cds

457 5'-GGAGGTGGAGGGTGTTTAGGGAAAGCTGTG-3' 486 cleavage site: 471

||| |||||||o ||||||

3'-ACCCCCCACAAGGCCCTTTG-5'

DysRNA_arrange_TPM 42440292_21 TTCCACAGCTTTCTTTAACTG

target id: NTBX_mRNA_69716_cds

464 5'-GAGGGTGTTTAGGGAAAGCTGT-GAAAAGAA-3' 493 cleavage site: 478

oo |||oo||||||||| |||

3'-GTCAATTTCTTTCGACACCTT-5'

DysRNA_arrange_TPM 42441010_21 TTCCACAGCTTTCTTGAAATG

target id: NTBX_mRNA_69716_cds

464 5'-GAGGGTGTTTAGGGAAAGCTGT-GAAAAGAA-3' 493 cleavage site: 478

oo||| oo||||||||| |||

3'-GTAAAGTTCTTTCGACACCTT-5'

DysRNA_arrange_TPM 19415850_21 ATCTCCTCTACTGGCTCTCCT

target id: NTTN90_mRNA_54536_cds

1587 5'-TGATAAGGAGA--CAGTAGAAGAGAGAAAGA-3' 1615 cleavage site: 1600

|||||| ||||||| ||||

3'-TCCTCTCGGTCATCTCCTCTA-5'

DysRNA_arrange_TPM 02940567_21 AAGAGAGTTGGGAATTATTGC

target id: NTTN90_mRNA_54536_cds

1442 5'-CAATGGCAATCTTCTTTCAACTCTCTTCACAA-3' 1473 cleavage site: 1458

||||| | |oo||||||||||

3'-CGTTATTA-AGGGTTGAGAGAA-5'

DysRNA_arrange_TPM 43499236_21 TTTGTCATGATAAATAAAGTC

target id: NTBX_mRNA_97357_cds

5 5'-CCAAGGACTTTATTTATCATGACAAATTGTT-3' 35 cleavage site: 20

|||||||||||||||||||||

3'-CTGAAATAAATAGTACTGTTT-5'

DysRNA_arrange_TPM 43498503_21 TTTGTCATGATGAATAAAGTC

target id: NTBX_mRNA_97357_cds

5 5'-CCAAGGACTTTATTTATCATGACAAATTGTT-3' 35 cleavage site: 20

|||||||||o|||||||||||

3'-CTGAAATAAGTAGTACTGTTT-5'

DysRNA_arrange_TPM 07119696_21 AATTGTCACTTGATTGTTGCA

target id: NTBX_mRNA_49495_cds

1832 5'-AATGGTGCAGGAATCAAGTAACAGCTTCATCT-3' 1863 cleavage site: 1848

||||o |||||||| |||o ||

3'-ACGTTGTTAGTTCACTGTT-AA-5'

DysRNA_arrange_TPM 27046016_20 GTGAAATTCTTGGATCGTCT

target id: NTBX_mRNA_49495_cds

898 5'-GAGAGTGAT-ATCCAAGAATTTCGATTGAA-3' 926 cleavage site: 911

||o |||||||||||||o

3'-TCTGCTAGGTTCTTAAAGTG-5'

DysRNA_arrange_TPM 15928038_22 ATAGAATAATTATGAATGTGCT

target id: NTBX_mRNA_124795_cds

2307 5'-ATCATGTCGCATTCATAATTA-TCTATATCAT-3' 2337 cleavage site: 2322

|o|||||||||||| |||||

3'-TCGTGTAAGTATTAATAAGATA-5'

DysRNA_arrange_TPM 02767294_21 AAGAAGATGATTATGGAGGGC

target id: NTTN90_mRNA_63311_cds

2931 5'-TTCAAGTGCTCTATCATCATCTTCTTCAGAT-3' 2961 cleavage site: 2946

|o |||o|| |||||||||||

3'-CGGGAGGTATTAGTAGAAGAA-5'

DysRNA_arrange_TPM 38561019_24 TCGAAGAAGATGATGAATAGTAGT

target id: NTTN90_mRNA_63311_cds

2933 5'-CAAGTGCT-CTA-TCATCATCTTCTTCAGATCTGA-3' 2965 cleavage site: 2950

o|| ||| |||||||||||||| ||

3'-TGATGATAAGTAGTAGAAGAAG-CT-5'

DysRNA_arrange_TPM 24021801_23 GGAGAAGATGATGAATAGTAATT

target id: NTTN90_mRNA_63311_cds

2929 5'-AGTTCAAGTGCTCTATCATCATCTTCTTCAGATC-3' 2962 cleavage site: 2947

|| |o|| | ||||||||||||o|

3'-TTAATGATA-AGTAGTAGAAGAGG-5'

DysRNA_arrange_TPM 41343934_21 TTGGAGAAGATGATGAATAGT

target id: NTTN90_mRNA_63311_cds

2935 5'-AGTGCTCTA-TCATCATCTTCTTCAGATCTG-3' 2964 cleavage site: 2949

||| ||||||||||||o||o

3'-TGATAAGTAGTAGAAGAGGTT-5'

DysRNA_arrange_TPM 24023543_24 GGAGAAGATGATGAATAGTAATTG

target id: NTTN90_mRNA_63311_cds

2928 5'-AAGTTCAAGTGCTCTATCATCATCTTCTTCAGATC-3' 2962 cleavage site: 2947

||| |o|| | ||||||||||||o|

3'-GTTAATGATA-AGTAGTAGAAGAGG-5'

DysRNA_arrange_TPM 02768834_22 AAGAAGATGATTATGGAGGGCA

target id: NTTN90_mRNA_63311_cds

2930 5'-GTTCAAGTGCTCTATCATCATCTTCTTCAGAT-3' 2961 cleavage site: 2946

|o |||o|| |||||||||||

3'-ACGGGAGGTATTAGTAGAAGAA-5'

DysRNA_arrange_TPM 27959210_20 GTTGTTTTGAGTTTGAGGCC

target id: NTK326_mRNA_121253_cds

1247 5'-TTAATTGCATTAAACTCAAAA-AATTGCCA-3' 1275 cleavage site: 1260

|| |o|||||||||| ||o

3'-CCGGAGTTTGAGTTTTGTTG-5'

DysRNA_arrange_TPM 08965277_24 AGATTTAGGCAAATGGAAAGAACT

target id: NTBX_mRNA_125078_cds

1928 5'-AATTGAGTTCATTTCATTTGCC-AAATCCTCCAA-3' 1960 cleavage site: 1945

||||| ||o|||||||| |||||

3'-TCAAGAAAGGTAAACGGATTTAGA-5'

DysRNA_arrange_TPM 43880920_20 TTTCTCTTTTTATTTATCGT

target id: NTBX_mRNA_125078_cds

2599 5'-ATTCAAC-ATGAACAAAAAGAGAACCTCGG-3' 2627 cleavage site: 2612

|| ||o|| ||||||||||

3'-TGCTATTTATTTTTCTCTTT-5'

DysRNA_arrange_TPM 08965277_24 AGATTTAGGCAAATGGAAAGAACT

target id: NTK326_mRNA_123992_cds

1868 5'-AATTGAGTTCATTTCATTTGCC-AAATCCTCCAA-3' 1900 cleavage site: 1885

||||| ||o|||||||| |||||

3'-TCAAGAAAGGTAAACGGATTTAGA-5'

DysRNA_arrange_TPM 43880920_20 TTTCTCTTTTTATTTATCGT

target id: NTK326_mRNA_123992_cds

2539 5'-ATTCAAC-ATGAACAAAAAGAGAACCTCGG-3' 2567 cleavage site: 2552

|| ||o|| ||||||||||

3'-TGCTATTTATTTTTCTCTTT-5'

DysRNA_arrange_TPM 27959210_20 GTTGTTTTGAGTTTGAGGCC

target id: NTBX_mRNA_91409_cds

1100 5'-TTGATTGCATTAAACTCAAAA-AATTGCCA-3' 1128 cleavage site: 1113

|| |o|||||||||| ||o

3'-CCGGAGTTTGAGTTTTGTTG-5'

DysRNA_arrange_TPM 08965277_24 AGATTTAGGCAAATGGAAAGAACT

target id: NTK326_mRNA_123993_cds

1868 5'-AATTGAGTTCATTTCATTTGCC-AAATCCTCCAA-3' 1900 cleavage site: 1885

||||| ||o|||||||| |||||

3'-TCAAGAAAGGTAAACGGATTTAGA-5'

DysRNA_arrange_TPM 43983812_21 TTTTACCTGAACCTGGCATAC

target id: NTBX_mRNA_20755_cds

1932 5'-CACTGGCATGCCGGGTTCGGGTAAAACTACT-3' 1962 cleavage site: 1947

|||||o|||||o|||||||

3'-CATACGGTCCAAGTCCATTTT-5'

DysRNA_arrange_TPM 40741688_22 TTACCTGAACCTGGCATGCCAA

target id: NTBX_mRNA_20755_cds

1929 5'-GATCACTGGCATGCCGGGTTCGGGTAAAACTA-3' 1960 cleavage site: 1945

|||||||||o|||||o|||||

3'-AACCGTACGGTCCAAGTCCATT-5'

DysRNA_arrange_TPM 40727231_22 TTACCCGAACCTGGCATACCAA

target id: NTBX_mRNA_20755_cds

1929 5'-GATCACTGGCATGCCGGGTTCGGGTAAAACTA-3' 1960 cleavage site: 1945

||| |||||o|||||||||||

3'-AACCATACGGTCCAAGCCCATT-5'

DysRNA_arrange_TPM 40741702_22 TTACCTGAACCCGGCATACCAA

target id: NTBX_mRNA_20755_cds

1929 5'-GATCACTGGCATGCCGGGTTCGGGTAAAACTA-3' 1960 cleavage site: 1945

||| |||||||||||o|||||

3'-AACCATACGGCCCAAGTCCATT-5'

DysRNA_arrange_TPM 27046016_20 GTGAAATTCTTGGATCGTCT

target id: NTTN90_mRNA_42242_cds

1522 5'-GAGAGTGAT-ATCCAAGAATTTCGATTGAA-3' 1550 cleavage site: 1535

||o |||||||||||||o

3'-TCTGCTAGGTTCTTAAAGTG-5'

DysRNA_arrange_TPM 15928038_22 ATAGAATAATTATGAATGTGCT

target id: NTTN90_mRNA_117647_cds

2307 5'-ATCATGTCGCATTCATAATTA-TCTATATCAT-3' 2337 cleavage site: 2322

|o|||||||||||| |||||

3'-TCGTGTAAGTATTAATAAGATA-5'

DysRNA_arrange_TPM 28228704_20 GTTTCCCGGAACACCCCCCA

target id: NTK326_mRNA_69163_cds

457 5'-GGAGGTGGAGGGTGTTTAGGGAAAGCTGTG-3' 486 cleavage site: 471

||| |||||||o ||||||

3'-ACCCCCCACAAGGCCCTTTG-5'

DysRNA_arrange_TPM 42440292_21 TTCCACAGCTTTCTTTAACTG

target id: NTK326_mRNA_69163_cds

464 5'-GAGGGTGTTTAGGGAAAGCTGT-GAAAAGAA-3' 493 cleavage site: 478

oo |||oo||||||||| |||

3'-GTCAATTTCTTTCGACACCTT-5'

DysRNA_arrange_TPM 42441010_21 TTCCACAGCTTTCTTGAAATG

target id: NTK326_mRNA_69163_cds

464 5'-GAGGGTGTTTAGGGAAAGCTGT-GAAAAGAA-3' 493 cleavage site: 478

oo||| oo||||||||| |||

3'-GTAAAGTTCTTTCGACACCTT-5'

DysRNA_arrange_TPM 02767294_21 AAGAAGATGATTATGGAGGGC

target id: NTTN90_mRNA_6839_cds

2430 5'-TTCAAGTGCTCCATCATCATCTTCTTCAGAT-3' 2460 cleavage site: 2445

|o |||||| |||||||||||

3'-CGGGAGGTATTAGTAGAAGAA-5'

DysRNA_arrange_TPM 02768834_22 AAGAAGATGATTATGGAGGGCA

target id: NTTN90_mRNA_6839_cds

2429 5'-GTTCAAGTGCTCCATCATCATCTTCTTCAGAT-3' 2460 cleavage site: 2445

|o |||||| |||||||||||

3'-ACGGGAGGTATTAGTAGAAGAA-5'

DysRNA_arrange_TPM 43983812_21 TTTTACCTGAACCTGGCATAC

target id: NTTN90_mRNA_63334_cds

1488 5'-CACTGGCATGCCGGGTTCGGGTAAAACTACT-3' 1518 cleavage site: 1503

|||||o|||||o|||||||

3'-CATACGGTCCAAGTCCATTTT-5'

DysRNA_arrange_TPM 40741688_22 TTACCTGAACCTGGCATGCCAA

target id: NTTN90_mRNA_63334_cds

1485 5'-GATCACTGGCATGCCGGGTTCGGGTAAAACTA-3' 1516 cleavage site: 1501

|||||||||o|||||o|||||

3'-AACCGTACGGTCCAAGTCCATT-5'

DysRNA_arrange_TPM 40727231_22 TTACCCGAACCTGGCATACCAA

target id: NTTN90_mRNA_63334_cds

1485 5'-GATCACTGGCATGCCGGGTTCGGGTAAAACTA-3' 1516 cleavage site: 1501

||| |||||o|||||||||||

3'-AACCATACGGTCCAAGCCCATT-5'

DysRNA_arrange_TPM 40741702_22 TTACCTGAACCCGGCATACCAA

target id: NTTN90_mRNA_63334_cds

1485 5'-GATCACTGGCATGCCGGGTTCGGGTAAAACTA-3' 1516 cleavage site: 1501

||| |||||||||||o|||||

3'-AACCATACGGCCCAAGTCCATT-5'

DysRNA_arrange_TPM 28228704_20 GTTTCCCGGAACACCCCCCA

target id: NTTN90_mRNA_60680_cds

457 5'-GGAGGTGGAGGGTGTTTAGGGAAAGCTGTG-3' 486 cleavage site: 471

||| |||||||o ||||||

3'-ACCCCCCACAAGGCCCTTTG-5'

DysRNA_arrange_TPM 42440292_21 TTCCACAGCTTTCTTTAACTG

target id: NTTN90_mRNA_60680_cds

464 5'-GAGGGTGTTTAGGGAAAGCTGT-GAAAAGAA-3' 493 cleavage site: 478

oo |||oo||||||||| |||

3'-GTCAATTTCTTTCGACACCTT-5'

DysRNA_arrange_TPM 42441010_21 TTCCACAGCTTTCTTGAAATG

target id: NTTN90_mRNA_60680_cds

464 5'-GAGGGTGTTTAGGGAAAGCTGT-GAAAAGAA-3' 493 cleavage site: 478

oo||| oo||||||||| |||

3'-GTAAAGTTCTTTCGACACCTT-5'

DysRNA_arrange_TPM 19415850_21 ATCTCCTCTACTGGCTCTCCT

target id: NTTN90_mRNA_54533_cds

639 5'-TGATAAGGAGA--CAGTAGAAGAGAGAAAGA-3' 667 cleavage site: 652

|||||| ||||||| ||||

3'-TCCTCTCGGTCATCTCCTCTA-5'

DysRNA_arrange_TPM 02940567_21 AAGAGAGTTGGGAATTATTGC

target id: NTTN90_mRNA_54533_cds

494 5'-CAATGGCAATCTTCTTTCAACTCTCTTCACAA-3' 525 cleavage site: 510

||||| | |oo||||||||||

3'-CGTTATTA-AGGGTTGAGAGAA-5'

DysRNA_arrange_TPM 02767294_21 AAGAAGATGATTATGGAGGGC

target id: NTBX_mRNA_81028_cds

1659 5'-TTCAAGTGCTCCATCATCATCTTCTTCAGAT-3' 1689 cleavage site: 1674

|o |||||| |||||||||||

3'-CGGGAGGTATTAGTAGAAGAA-5'

DysRNA_arrange_TPM 02768834_22 AAGAAGATGATTATGGAGGGCA

target id: NTBX_mRNA_81028_cds

1658 5'-GTTCAAGTGCTCCATCATCATCTTCTTCAGAT-3' 1689 cleavage site: 1674

|o |||||| |||||||||||

3'-ACGGGAGGTATTAGTAGAAGAA-5'

DysRNA_arrange_TPM 08965277_24 AGATTTAGGCAAATGGAAAGAACT

target id: NTTN90_mRNA_109400_cds

1868 5'-AATTGAGTTCATTTCATTTGCC-AAATCCTCCAA-3' 1900 cleavage site: 1885

||||| ||o|||||||| |||||

3'-TCAAGAAAGGTAAACGGATTTAGA-5'

DysRNA_arrange_TPM 43880920_20 TTTCTCTTTTTATTTATCGT

target id: NTTN90_mRNA_109400_cds

2539 5'-ATTCAAC-ATGAACAAAAAGAGAACCTCGG-3' 2567 cleavage site: 2552

|| ||o|| ||||||||||

3'-TGCTATTTATTTTTCTCTTT-5'

DysRNA_arrange_TPM 43319357_21 TTTGGATTGAAGGGAGCTCTA

target id: NTK326_mRNA_95709_cds

112 5'-TCTGGTACAG-TTTCTTCAATCCAAGCTGTA-3' 141 cleavage site: 126

|| || |oo|||||||||||o

3'-ATCTCGAGGGAAGTTAGGTTT-5'

DysRNA_arrange_TPM 41411013_20 TTGGATTGAAGGGAGCTCTA

target id: NTK326_mRNA_95709_cds

112 5'-TCTGGTACAG-TTTCTTCAATCCAAGCTGT-3' 140 cleavage site: 125

|| || |oo|||||||||||

3'-ATCTCGAGGGAAGTTAGGTT-5'

DysRNA_arrange_TPM 43319585_22 TTTGGATTGAAGGGAGCTCTAT

target id: NTK326_mRNA_95709_cds

111 5'-TTCTGGTACAG-TTTCTTCAATCCAAGCTGTA-3' 141 cleavage site: 126

o|| || |oo|||||||||||o

3'-TATCTCGAGGGAAGTTAGGTTT-5'

DysRNA_arrange_TPM 43316465_21 TTTGGATTGAAGGGAGCTCTG

target id: NTK326_mRNA_95709_cds

112 5'-TCTGGTACAG-TTTCTTCAATCCAAGCTGTA-3' 141 cleavage site: 126

o| || |oo|||||||||||o

3'-GTCTCGAGGGAAGTTAGGTTT-5'

DysRNA_arrange_TPM 43316124_21 TTTGGATTGAAGGGGGCTCTA

target id: NTK326_mRNA_95709_cds

112 5'-TCTGGTACAG-TTTCTTCAATCCAAGCTGTA-3' 141 cleavage site: 126

|| || ooo|||||||||||o

3'-ATCTCGGGGGAAGTTAGGTTT-5'

DysRNA_arrange_TPM 43315582_22 TTTGGATTGAAGGGAGCTCTAC

target id: NTK326_mRNA_95709_cds

111 5'-TTCTGGTACAG-TTTCTTCAATCCAAGCTGTA-3' 141 cleavage site: 126

||| || |oo|||||||||||o

3'-CATCTCGAGGGAAGTTAGGTTT-5'

DysRNA_arrange_TPM 43321659_21 TTTGGATTGAAGGAAGCTCTA

target id: NTK326_mRNA_95709_cds

112 5'-TCTGGTACAG-TTTCTTCAATCCAAGCTGTA-3' 141 cleavage site: 126

|| || ||o|||||||||||o

3'-ATCTCGAAGGAAGTTAGGTTT-5'

DysRNA_arrange_TPM 43317025_21 TTTGGATTGAAGGTAGCTCTA

target id: NTK326_mRNA_95709_cds

112 5'-TCTGGTACAGTT-TCTTCAATCCAAGCTGTA-3' 141 cleavage site: 126

|| ||o| o|||||||||||o

3'-ATCTCGATGGAAGTTAGGTTT-5'

DysRNA_arrange_TPM 43320131_21 TTTGGATTGAAGTGAGCTCTA

target id: NTK326_mRNA_95709_cds

112 5'-TCTGGTACAGTTT-CTTCAATCCAAGCTGTA-3' 141 cleavage site: 126

|| ||o|o |||||||||||o

3'-ATCTCGAGTGAAGTTAGGTTT-5'

DysRNA_arrange_TPM 43320956_21 TTTGGATTGAAGGGAGCTGTA

target id: NTK326_mRNA_95709_cds

112 5'-TCTGGTACAG-TTTCTTCAATCCAAGCTGTA-3' 141 cleavage site: 126

||||| |oo|||||||||||o

3'-ATGTCGAGGGAAGTTAGGTTT-5'

DysRNA_arrange_TPM 43317682_21 TTTGGATTGAAGGGAGCTATA

target id: NTK326_mRNA_95709_cds

112 5'-TCTGGTACAG-TTTCTTCAATCCAAGCTGTA-3' 141 cleavage site: 126

|| || |oo|||||||||||o

3'-ATATCGAGGGAAGTTAGGTTT-5'

DysRNA_arrange_TPM 20002187_21 ATTGGATTGAAGGGAGCTCTA

target id: NTK326_mRNA_95709_cds

112 5'-TCTGGTACAG-TTTCTTCAATCCAAGCTGTA-3' 141 cleavage site: 126

|| || |oo|||||||||||

3'-ATCTCGAGGGAAGTTAGGTTA-5'

DysRNA_arrange_TPM 43317086_21 TTTGGATTGAAGGGCGCTCTA

target id: NTK326_mRNA_95709_cds

112 5'-TCTGGTACAGT-TTCTTCAATCCAAGCTGTA-3' 141 cleavage site: 126

|| ||o oo|||||||||||o

3'-ATCTCGCGGGAAGTTAGGTTT-5'

DysRNA_arrange_TPM 33578164_21 CTTGGATTGAAGGGAGCTCTA

target id: NTK326_mRNA_95709_cds

112 5'-TCTGGTACAG-TTTCTTCAATCCAAGCTGTA-3' 141 cleavage site: 126

|| || |oo||||||||||||

3'-ATCTCGAGGGAAGTTAGGTTC-5'

DysRNA_arrange_TPM 43316328_21 TTTGGATTGAAGGGAGCTTTA

target id: NTK326_mRNA_95709_cds

112 5'-TCTGGTACAG-TTTCTTCAATCCAAGCTGTA-3' 141 cleavage site: 126

|| || |oo|||||||||||o

3'-ATTTCGAGGGAAGTTAGGTTT-5'

DysRNA_arrange_TPM 43315028_21 TTTGGATTGAAGGGTGCTCTA

target id: NTK326_mRNA_95709_cds

112 5'-TCTGGTACAGT-TTCTTCAATCCAAGCTGTA-3' 141 cleavage site: 126

|| ||o oo|||||||||||o

3'-ATCTCGTGGGAAGTTAGGTTT-5'

DysRNA_arrange_TPM 42289602_21 TTCGGATTGAAGGGAGCTCTA

target id: NTK326_mRNA_95709_cds

112 5'-TCTGGTACAG-TTTCTTCAATCC-AAGCTGT-3' 140 cleavage site: 125

|| || |oo||||||||| ||

3'-ATCTCGAGGGAAGTTAGGCTT-5'

DysRNA_arrange_TPM 43317643_23 TTTGGATTGAAGGGAGCTCTACT

target id: NTK326_mRNA_95709_cds

110 5'-TTTCTGGTACAG-TTTCTTCAATCCAAGCTGTA-3' 141 cleavage site: 126

o||| || |oo|||||||||||o

3'-TCATCTCGAGGGAAGTTAGGTTT-5'

DysRNA_arrange_TPM 43317291_21 TTTGGATTGAAGAGAGCTCTA

target id: NTK326_mRNA_95709_cds

112 5'-TCTGGTACAG-TTTCTTCAATCCAAGCTGTA-3' 141 cleavage site: 126

|| || |o||||||||||||o

3'-ATCTCGAGAGAAGTTAGGTTT-5'

DysRNA_arrange_TPM 43317729_20 TTTGGATTGAAGGAGCTCTA

target id: NTK326_mRNA_95709_cds

112 5'-TCTGGTACAGTTTCTTCAATCCAAGCTGTA-3' 141 cleavage site: 126

|| ||o|o|||||||||||o

3'-ATCTCGAGGAAGTTAGGTTT-5'

DysRNA_arrange_TPM 43316738_21 TTTGGATTGAAGGGAACTCTA

target id: NTK326_mRNA_95709_cds

112 5'-TCTGGTACAG-TTTCTTCAATCCAAGCTGTA-3' 141 cleavage site: 126

|| || |oo|||||||||||o

3'-ATCTCAAGGGAAGTTAGGTTT-5'

DysRNA_arrange_TPM 40528105_21 TTAGGATTGAAGGGAGCTCTA

target id: NTK326_mRNA_95709_cds

112 5'-TCTGGTACAG-TTTCTTCAATCC-AAGCTGT-3' 140 cleavage site: 125

|| || |oo||||||||| ||

3'-ATCTCGAGGGAAGTTAGGATT-5'

DysRNA_arrange_TPM 27871038_21 GTTGGATTGAAGGGAGCTCTA

target id: NTK326_mRNA_95709_cds

112 5'-TCTGGTACAG-TTTCTTCAATCCAAGCTGTA-3' 141 cleavage site: 126

|| || |oo|||||||||||

3'-ATCTCGAGGGAAGTTAGGTTG-5'

DysRNA_arrange_TPM 43321070_21 TTTGGATTGAAGCGAGCTCTA

target id: NTK326_mRNA_95709_cds

112 5'-TCTGGTACAGTTT-CTTCAATCCAAGCTGTA-3' 141 cleavage site: 126

|| ||o|o |||||||||||o

3'-ATCTCGAGCGAAGTTAGGTTT-5'

DysRNA_arrange_TPM 27959210_20 GTTGTTTTGAGTTTGAGGCC

target id: NTK326_mRNA_95709_cds

1916 5'-TTGATTGCATTAAACTCAAAA-AATTGCCA-3' 1944 cleavage site: 1929

|| |o|||||||||| ||o

3'-CCGGAGTTTGAGTTTTGTTG-5'

DysRNA_arrange_TPM 02767294_21 AAGAAGATGATTATGGAGGGC

target id: NTTN90_mRNA_63305_cds

3012 5'-TTCAAGTGCTCTATCATCATCTTCTTCAGAT-3' 3042 cleavage site: 3027

|o |||o|| |||||||||||

3'-CGGGAGGTATTAGTAGAAGAA-5'

DysRNA_arrange_TPM 38561019_24 TCGAAGAAGATGATGAATAGTAGT

target id: NTTN90_mRNA_63305_cds

3014 5'-CAAGTGCT-CTA-TCATCATCTTCTTCAGATCTGA-3' 3046 cleavage site: 3031

o|| ||| |||||||||||||| ||

3'-TGATGATAAGTAGTAGAAGAAG-CT-5'

DysRNA_arrange_TPM 24021801_23 GGAGAAGATGATGAATAGTAATT

target id: NTTN90_mRNA_63305_cds

3010 5'-AGTTCAAGTGCTCTATCATCATCTTCTTCAGATC-3' 3043 cleavage site: 3028

|| |o|| | ||||||||||||o|

3'-TTAATGATA-AGTAGTAGAAGAGG-5'

DysRNA_arrange_TPM 41343934_21 TTGGAGAAGATGATGAATAGT

target id: NTTN90_mRNA_63305_cds

3016 5'-AGTGCTCTA-TCATCATCTTCTTCAGATCTG-3' 3045 cleavage site: 3030

||| ||||||||||||o||o

3'-TGATAAGTAGTAGAAGAGGTT-5'

DysRNA_arrange_TPM 24023543_24 GGAGAAGATGATGAATAGTAATTG

target id: NTTN90_mRNA_63305_cds

3009 5'-AAGTTCAAGTGCTCTATCATCATCTTCTTCAGATC-3' 3043 cleavage site: 3028

||| |o|| | ||||||||||||o|

3'-GTTAATGATA-AGTAGTAGAAGAGG-5'

DysRNA_arrange_TPM 02768834_22 AAGAAGATGATTATGGAGGGCA

target id: NTTN90_mRNA_63305_cds

3011 5'-GTTCAAGTGCTCTATCATCATCTTCTTCAGAT-3' 3042 cleavage site: 3027

|o |||o|| |||||||||||

3'-ACGGGAGGTATTAGTAGAAGAA-5'

DysRNA_arrange_TPM 02767294_21 AAGAAGATGATTATGGAGGGC

target id: NTTN90_mRNA_63326_cds

2841 5'-TTCAAGTGCTCCATCATCATCTTCTTCAGAT-3' 2871 cleavage site: 2856

|o |||||| |||||||||||

3'-CGGGAGGTATTAGTAGAAGAA-5'

DysRNA_arrange_TPM 31162881_20 CGTTGCTGCGATGATTCATG

target id: NTTN90_mRNA_63326_cds

3513 5'-TGAACTA-GAAATCATCGCAGTAACTTTTCA-3' 3542 cleavage site: 3527

o| | |||||||||||o|||

3'-GTAC-TTAGTAGCGTCGTTGC-5'

DysRNA_arrange_TPM 27913008_20 GTTGCTGCGATGATTCATGA

target id: NTTN90_mRNA_63326_cds

3512 5'-ATGAACTA-GAAATCATCGCAGTAACTTTTC-3' 3541 cleavage site: 3526

o| | |||||||||||o|||

3'-AGTAC-TTAGTAGCGTCGTTG-5'

DysRNA_arrange_TPM 02768834_22 AAGAAGATGATTATGGAGGGCA

target id: NTTN90_mRNA_63326_cds

2840 5'-GTTCAAGTGCTCCATCATCATCTTCTTCAGAT-3' 2871 cleavage site: 2856

|o |||||| |||||||||||

3'-ACGGGAGGTATTAGTAGAAGAA-5'

DysRNA_arrange_TPM 15928038_22 ATAGAATAATTATGAATGTGCT

target id: NTTN90_mRNA_117652_cds

2178 5'-ATCATGTCGCATTCATAATTA-TCTATATCAT-3' 2208 cleavage site: 2193

|o|||||||||||| |||||

3'-TCGTGTAAGTATTAATAAGATA-5'

DysRNA_arrange_TPM 43319357_21 TTTGGATTGAAGGGAGCTCTA

target id: NTBX_mRNA_122258_cds

112 5'-TCTGGTACAG-TTTCTTCAATCCAAGCTGTA-3' 141 cleavage site: 126

|| || |oo|||||||||||o

3'-ATCTCGAGGGAAGTTAGGTTT-5'

DysRNA_arrange_TPM 41411013_20 TTGGATTGAAGGGAGCTCTA

target id: NTBX_mRNA_122258_cds

112 5'-TCTGGTACAG-TTTCTTCAATCCAAGCTGT-3' 140 cleavage site: 125

|| || |oo|||||||||||

3'-ATCTCGAGGGAAGTTAGGTT-5'

DysRNA_arrange_TPM 43319585_22 TTTGGATTGAAGGGAGCTCTAT

target id: NTBX_mRNA_122258_cds

111 5'-TTCTGGTACAG-TTTCTTCAATCCAAGCTGTA-3' 141 cleavage site: 126

o|| || |oo|||||||||||o

3'-TATCTCGAGGGAAGTTAGGTTT-5'

DysRNA_arrange_TPM 43316465_21 TTTGGATTGAAGGGAGCTCTG

target id: NTBX_mRNA_122258_cds

112 5'-TCTGGTACAG-TTTCTTCAATCCAAGCTGTA-3' 141 cleavage site: 126

o| || |oo|||||||||||o

3'-GTCTCGAGGGAAGTTAGGTTT-5'

DysRNA_arrange_TPM 43316124_21 TTTGGATTGAAGGGGGCTCTA

target id: NTBX_mRNA_122258_cds

112 5'-TCTGGTACAG-TTTCTTCAATCCAAGCTGTA-3' 141 cleavage site: 126

|| || ooo|||||||||||o

3'-ATCTCGGGGGAAGTTAGGTTT-5'

DysRNA_arrange_TPM 43315582_22 TTTGGATTGAAGGGAGCTCTAC

target id: NTBX_mRNA_122258_cds

111 5'-TTCTGGTACAG-TTTCTTCAATCCAAGCTGTA-3' 141 cleavage site: 126

||| || |oo|||||||||||o

3'-CATCTCGAGGGAAGTTAGGTTT-5'

DysRNA_arrange_TPM 43321659_21 TTTGGATTGAAGGAAGCTCTA

target id: NTBX_mRNA_122258_cds

112 5'-TCTGGTACAG-TTTCTTCAATCCAAGCTGTA-3' 141 cleavage site: 126

|| || ||o|||||||||||o

3'-ATCTCGAAGGAAGTTAGGTTT-5'

DysRNA_arrange_TPM 43317025_21 TTTGGATTGAAGGTAGCTCTA

target id: NTBX_mRNA_122258_cds

112 5'-TCTGGTACAGTT-TCTTCAATCCAAGCTGTA-3' 141 cleavage site: 126

|| ||o| o|||||||||||o

3'-ATCTCGATGGAAGTTAGGTTT-5'

DysRNA_arrange_TPM 43320131_21 TTTGGATTGAAGTGAGCTCTA

target id: NTBX_mRNA_122258_cds

112 5'-TCTGGTACAGTTT-CTTCAATCCAAGCTGTA-3' 141 cleavage site: 126

|| ||o|o |||||||||||o

3'-ATCTCGAGTGAAGTTAGGTTT-5'

DysRNA_arrange_TPM 43320956_21 TTTGGATTGAAGGGAGCTGTA

target id: NTBX_mRNA_122258_cds

112 5'-TCTGGTACAG-TTTCTTCAATCCAAGCTGTA-3' 141 cleavage site: 126

||||| |oo|||||||||||o

3'-ATGTCGAGGGAAGTTAGGTTT-5'

DysRNA_arrange_TPM 43317682_21 TTTGGATTGAAGGGAGCTATA

target id: NTBX_mRNA_122258_cds

112 5'-TCTGGTACAG-TTTCTTCAATCCAAGCTGTA-3' 141 cleavage site: 126

|| || |oo|||||||||||o

3'-ATATCGAGGGAAGTTAGGTTT-5'

DysRNA_arrange_TPM 20002187_21 ATTGGATTGAAGGGAGCTCTA

target id: NTBX_mRNA_122258_cds

112 5'-TCTGGTACAG-TTTCTTCAATCCAAGCTGTA-3' 141 cleavage site: 126

|| || |oo|||||||||||

3'-ATCTCGAGGGAAGTTAGGTTA-5'

DysRNA_arrange_TPM 43317086_21 TTTGGATTGAAGGGCGCTCTA

target id: NTBX_mRNA_122258_cds

112 5'-TCTGGTACAGT-TTCTTCAATCCAAGCTGTA-3' 141 cleavage site: 126

|| ||o oo|||||||||||o

3'-ATCTCGCGGGAAGTTAGGTTT-5'

DysRNA_arrange_TPM 33578164_21 CTTGGATTGAAGGGAGCTCTA

target id: NTBX_mRNA_122258_cds

112 5'-TCTGGTACAG-TTTCTTCAATCCAAGCTGTA-3' 141 cleavage site: 126

|| || |oo||||||||||||

3'-ATCTCGAGGGAAGTTAGGTTC-5'

DysRNA_arrange_TPM 43316328_21 TTTGGATTGAAGGGAGCTTTA

target id: NTBX_mRNA_122258_cds

112 5'-TCTGGTACAG-TTTCTTCAATCCAAGCTGTA-3' 141 cleavage site: 126

|| || |oo|||||||||||o

3'-ATTTCGAGGGAAGTTAGGTTT-5'

DysRNA_arrange_TPM 43315028_21 TTTGGATTGAAGGGTGCTCTA

target id: NTBX_mRNA_122258_cds

112 5'-TCTGGTACAGT-TTCTTCAATCCAAGCTGTA-3' 141 cleavage site: 126

|| ||o oo|||||||||||o

3'-ATCTCGTGGGAAGTTAGGTTT-5'

DysRNA_arrange_TPM 42289602_21 TTCGGATTGAAGGGAGCTCTA

target id: NTBX_mRNA_122258_cds

112 5'-TCTGGTACAG-TTTCTTCAATCC-AAGCTGT-3' 140 cleavage site: 125

|| || |oo||||||||| ||

3'-ATCTCGAGGGAAGTTAGGCTT-5'

DysRNA_arrange_TPM 43317643_23 TTTGGATTGAAGGGAGCTCTACT

target id: NTBX_mRNA_122258_cds

110 5'-TTTCTGGTACAG-TTTCTTCAATCCAAGCTGTA-3' 141 cleavage site: 126

o||| || |oo|||||||||||o

3'-TCATCTCGAGGGAAGTTAGGTTT-5'

DysRNA_arrange_TPM 43317291_21 TTTGGATTGAAGAGAGCTCTA

target id: NTBX_mRNA_122258_cds

112 5'-TCTGGTACAG-TTTCTTCAATCCAAGCTGTA-3' 141 cleavage site: 126

|| || |o||||||||||||o

3'-ATCTCGAGAGAAGTTAGGTTT-5'

DysRNA_arrange_TPM 43317729_20 TTTGGATTGAAGGAGCTCTA

target id: NTBX_mRNA_122258_cds

112 5'-TCTGGTACAGTTTCTTCAATCCAAGCTGTA-3' 141 cleavage site: 126

|| ||o|o|||||||||||o

3'-ATCTCGAGGAAGTTAGGTTT-5'

DysRNA_arrange_TPM 43316738_21 TTTGGATTGAAGGGAACTCTA

target id: NTBX_mRNA_122258_cds

112 5'-TCTGGTACAG-TTTCTTCAATCCAAGCTGTA-3' 141 cleavage site: 126

|| || |oo|||||||||||o

3'-ATCTCAAGGGAAGTTAGGTTT-5'

DysRNA_arrange_TPM 40528105_21 TTAGGATTGAAGGGAGCTCTA

target id: NTBX_mRNA_122258_cds

112 5'-TCTGGTACAG-TTTCTTCAATCC-AAGCTGT-3' 140 cleavage site: 125

|| || |oo||||||||| ||

3'-ATCTCGAGGGAAGTTAGGATT-5'

DysRNA_arrange_TPM 27871038_21 GTTGGATTGAAGGGAGCTCTA

target id: NTBX_mRNA_122258_cds

112 5'-TCTGGTACAG-TTTCTTCAATCCAAGCTGTA-3' 141 cleavage site: 126

|| || |oo|||||||||||

3'-ATCTCGAGGGAAGTTAGGTTG-5'

DysRNA_arrange_TPM 43321070_21 TTTGGATTGAAGCGAGCTCTA

target id: NTBX_mRNA_122258_cds

112 5'-TCTGGTACAGTTT-CTTCAATCCAAGCTGTA-3' 141 cleavage site: 126

|| ||o|o |||||||||||o

3'-ATCTCGAGCGAAGTTAGGTTT-5'

DysRNA_arrange_TPM 27046016_20 GTGAAATTCTTGGATCGTCT

target id: NTTN90_mRNA_42244_cds

1183 5'-GAGAGTGAT-ATCCAAGAATTTCGATTGAA-3' 1211 cleavage site: 1196

||o |||||||||||||o

3'-TCTGCTAGGTTCTTAAAGTG-5'

DysRNA_arrange_TPM 15928038_22 ATAGAATAATTATGAATGTGCT

target id: NTBX_mRNA_124794_cds

2532 5'-ATCATGTCGCATTCATAATTA-TCTATATCAT-3' 2562 cleavage site: 2547

|o|||||||||||| |||||

3'-TCGTGTAAGTATTAATAAGATA-5'

DysRNA_arrange_TPM 02767294_21 AAGAAGATGATTATGGAGGGC

target id: NTK326_mRNA_72004_cds

3177 5'-TCCAAGTGCTCCATCATCATCTTCTTCAGAT-3' 3207 cleavage site: 3192

|o |||||| |||||||||||

3'-CGGGAGGTATTAGTAGAAGAA-5'

DysRNA_arrange_TPM 02768834_22 AAGAAGATGATTATGGAGGGCA

target id: NTK326_mRNA_72004_cds

3176 5'-GTCCAAGTGCTCCATCATCATCTTCTTCAGAT-3' 3207 cleavage site: 3192

|o |||||| |||||||||||

3'-ACGGGAGGTATTAGTAGAAGAA-5'

DysRNA_arrange_TPM 02767294_21 AAGAAGATGATTATGGAGGGC

target id: NTK326_mRNA_72002_cds

2529 5'-TCCAAGTGCTCCATCATCATCTTCTTCAGAT-3' 2559 cleavage site: 2544

|o |||||| |||||||||||

3'-CGGGAGGTATTAGTAGAAGAA-5'

DysRNA_arrange_TPM 02768834_22 AAGAAGATGATTATGGAGGGCA

target id: NTK326_mRNA_72002_cds

2528 5'-GTCCAAGTGCTCCATCATCATCTTCTTCAGAT-3' 2559 cleavage site: 2544

|o |||||| |||||||||||

3'-ACGGGAGGTATTAGTAGAAGAA-5'

DysRNA_arrange_TPM 43963689_22 TTTTAGCCAGAGTTGTTTTCCC

target id: NTK326_mRNA_99998_cds

26 5'-GCATAGGGAAAACAACTCTGGC-AAAAGCTAT-3' 56 cleavage site: 41

||||||||||||||||| ||||

3'-CCCTTTTGTTGAGACCGATTTT-5'

DysRNA_arrange_TPM 42993390_21 TTTAGCCAGAGTTGTTTTCCC

target id: NTK326_mRNA_99998_cds

26 5'-GCATAGGGAAAACAACTCTGGC-AAAAGCTA-3' 55 cleavage site: 40

||||||||||||||||| |||

3'-CCCTTTTGTTGAGACCGATTT-5'

DysRNA_arrange_TPM 43964061_21 TTTTAGCCAGAGTTGTTTTCC

target id: NTK326_mRNA_99998_cds

27 5'-CATAGGGAAAACAACTCTGGC-AAAAGCTAT-3' 56 cleavage site: 41

|||||||||||||||| ||||

3'-CCTTTTGTTGAGACCGATTTT-5'

DysRNA_arrange_TPM 43499236_21 TTTGTCATGATAAATAAAGTC

target id: NTK326_mRNA_49586_cds

5 5'-CCAAGGACTTTATTTATCATGACAAATTGTT-3' 35 cleavage site: 20

|||||||||||||||||||||

3'-CTGAAATAAATAGTACTGTTT-5'

DysRNA_arrange_TPM 43498503_21 TTTGTCATGATGAATAAAGTC

target id: NTK326_mRNA_49586_cds

5 5'-CCAAGGACTTTATTTATCATGACAAATTGTT-3' 35 cleavage site: 20

|||||||||o|||||||||||

3'-CTGAAATAAGTAGTACTGTTT-5'

DysRNA_arrange_TPM 39918426_21 TCTCGTTGAAATCTAGCTACC

target id: NTK326_mRNA_49586_cds

1546 5'-AGCTTGGT-GATCGTTTTCAACGAGAGAGGT-3' 1575 cleavage site: 1560

||| | | | |||||||||||

3'-CCATCGATCTAAAGTTGCTCT-5'

DysRNA_arrange_TPM 43963689_22 TTTTAGCCAGAGTTGTTTTCCC

target id: NTTN90_mRNA_54399_cds

152 5'-GCATAGGAAAAACAACTCTGGC-AAAAGCTAT-3' 182 cleavage site: 167

|| |||||||||||||| ||||

3'-CCCTTTTGTTGAGACCGATTTT-5'

DysRNA_arrange_TPM 40391668_20 TTAATATTTCAGCTCGAACT

target id: NTTN90_mRNA_54399_cds

841 5'-CGTGTAG-TT-A-CTGAAATATTAAATGCA-3' 867 cleavage site: 852

|| |o | ||||||||||||

3'-TCAAGCTCGACTTTATAATT-5'

DysRNA_arrange_TPM 42993390_21 TTTAGCCAGAGTTGTTTTCCC

target id: NTTN90_mRNA_54399_cds

152 5'-GCATAGGAAAAACAACTCTGGC-AAAAGCTA-3' 181 cleavage site: 166

|| |||||||||||||| |||

3'-CCCTTTTGTTGAGACCGATTT-5'

DysRNA_arrange_TPM 43964061_21 TTTTAGCCAGAGTTGTTTTCC

target id: NTTN90_mRNA_54399_cds

152 5'-GCATAGGAAAAACAACTCTGGC-AAAAGCTAT-3' 182 cleavage site: 167

|| |||||||||||||| ||||

3'-CC-TTTTGTTGAGACCGATTTT-5'

DysRNA_arrange_TPM 43319357_21 TTTGGATTGAAGGGAGCTCTA

target id: NTTN90_mRNA_106750_cds

112 5'-TCTGGTACAG-TTTCTTCAATCCAAGCTGTA-3' 141 cleavage site: 126

|| || |oo|||||||||||o

3'-ATCTCGAGGGAAGTTAGGTTT-5'

DysRNA_arrange_TPM 41411013_20 TTGGATTGAAGGGAGCTCTA

target id: NTTN90_mRNA_106750_cds

112 5'-TCTGGTACAG-TTTCTTCAATCCAAGCTGT-3' 140 cleavage site: 125

|| || |oo|||||||||||

3'-ATCTCGAGGGAAGTTAGGTT-5'

DysRNA_arrange_TPM 43319585_22 TTTGGATTGAAGGGAGCTCTAT

target id: NTTN90_mRNA_106750_cds

111 5'-TTCTGGTACAG-TTTCTTCAATCCAAGCTGTA-3' 141 cleavage site: 126

o|| || |oo|||||||||||o

3'-TATCTCGAGGGAAGTTAGGTTT-5'

DysRNA_arrange_TPM 43316465_21 TTTGGATTGAAGGGAGCTCTG

target id: NTTN90_mRNA_106750_cds

112 5'-TCTGGTACAG-TTTCTTCAATCCAAGCTGTA-3' 141 cleavage site: 126

o| || |oo|||||||||||o

3'-GTCTCGAGGGAAGTTAGGTTT-5'

DysRNA_arrange_TPM 43316124_21 TTTGGATTGAAGGGGGCTCTA

target id: NTTN90_mRNA_106750_cds

112 5'-TCTGGTACAG-TTTCTTCAATCCAAGCTGTA-3' 141 cleavage site: 126

|| || ooo|||||||||||o

3'-ATCTCGGGGGAAGTTAGGTTT-5'

DysRNA_arrange_TPM 43315582_22 TTTGGATTGAAGGGAGCTCTAC

target id: NTTN90_mRNA_106750_cds

111 5'-TTCTGGTACAG-TTTCTTCAATCCAAGCTGTA-3' 141 cleavage site: 126

||| || |oo|||||||||||o

3'-CATCTCGAGGGAAGTTAGGTTT-5'

DysRNA_arrange_TPM 43321659_21 TTTGGATTGAAGGAAGCTCTA

target id: NTTN90_mRNA_106750_cds

112 5'-TCTGGTACAG-TTTCTTCAATCCAAGCTGTA-3' 141 cleavage site: 126

|| || ||o|||||||||||o

3'-ATCTCGAAGGAAGTTAGGTTT-5'

DysRNA_arrange_TPM 43317025_21 TTTGGATTGAAGGTAGCTCTA

target id: NTTN90_mRNA_106750_cds

112 5'-TCTGGTACAGTT-TCTTCAATCCAAGCTGTA-3' 141 cleavage site: 126

|| ||o| o|||||||||||o

3'-ATCTCGATGGAAGTTAGGTTT-5'

DysRNA_arrange_TPM 43320131_21 TTTGGATTGAAGTGAGCTCTA

target id: NTTN90_mRNA_106750_cds

112 5'-TCTGGTACAGTTT-CTTCAATCCAAGCTGTA-3' 141 cleavage site: 126

|| ||o|o |||||||||||o

3'-ATCTCGAGTGAAGTTAGGTTT-5'

DysRNA_arrange_TPM 43320956_21 TTTGGATTGAAGGGAGCTGTA

target id: NTTN90_mRNA_106750_cds

112 5'-TCTGGTACAG-TTTCTTCAATCCAAGCTGTA-3' 141 cleavage site: 126

||||| |oo|||||||||||o

3'-ATGTCGAGGGAAGTTAGGTTT-5'

DysRNA_arrange_TPM 43317682_21 TTTGGATTGAAGGGAGCTATA

target id: NTTN90_mRNA_106750_cds

112 5'-TCTGGTACAG-TTTCTTCAATCCAAGCTGTA-3' 141 cleavage site: 126

|| || |oo|||||||||||o

3'-ATATCGAGGGAAGTTAGGTTT-5'

DysRNA_arrange_TPM 20002187_21 ATTGGATTGAAGGGAGCTCTA

target id: NTTN90_mRNA_106750_cds

112 5'-TCTGGTACAG-TTTCTTCAATCCAAGCTGTA-3' 141 cleavage site: 126

|| || |oo|||||||||||

3'-ATCTCGAGGGAAGTTAGGTTA-5'

DysRNA_arrange_TPM 43317086_21 TTTGGATTGAAGGGCGCTCTA

target id: NTTN90_mRNA_106750_cds

112 5'-TCTGGTACAGT-TTCTTCAATCCAAGCTGTA-3' 141 cleavage site: 126

|| ||o oo|||||||||||o

3'-ATCTCGCGGGAAGTTAGGTTT-5'

DysRNA_arrange_TPM 33578164_21 CTTGGATTGAAGGGAGCTCTA

target id: NTTN90_mRNA_106750_cds

112 5'-TCTGGTACAG-TTTCTTCAATCCAAGCTGTA-3' 141 cleavage site: 126

|| || |oo||||||||||||

3'-ATCTCGAGGGAAGTTAGGTTC-5'

DysRNA_arrange_TPM 43316328_21 TTTGGATTGAAGGGAGCTTTA

target id: NTTN90_mRNA_106750_cds

112 5'-TCTGGTACAG-TTTCTTCAATCCAAGCTGTA-3' 141 cleavage site: 126

|| || |oo|||||||||||o

3'-ATTTCGAGGGAAGTTAGGTTT-5'

DysRNA_arrange_TPM 43315028_21 TTTGGATTGAAGGGTGCTCTA

target id: NTTN90_mRNA_106750_cds

112 5'-TCTGGTACAGT-TTCTTCAATCCAAGCTGTA-3' 141 cleavage site: 126

|| ||o oo|||||||||||o

3'-ATCTCGTGGGAAGTTAGGTTT-5'

DysRNA_arrange_TPM 42289602_21 TTCGGATTGAAGGGAGCTCTA

target id: NTTN90_mRNA_106750_cds

112 5'-TCTGGTACAG-TTTCTTCAATCC-AAGCTGT-3' 140 cleavage site: 125

|| || |oo||||||||| ||

3'-ATCTCGAGGGAAGTTAGGCTT-5'

DysRNA_arrange_TPM 43317643_23 TTTGGATTGAAGGGAGCTCTACT

target id: NTTN90_mRNA_106750_cds

110 5'-TTTCTGGTACAG-TTTCTTCAATCCAAGCTGTA-3' 141 cleavage site: 126

o||| || |oo|||||||||||o

3'-TCATCTCGAGGGAAGTTAGGTTT-5'

DysRNA_arrange_TPM 43317291_21 TTTGGATTGAAGAGAGCTCTA

target id: NTTN90_mRNA_106750_cds

112 5'-TCTGGTACAG-TTTCTTCAATCCAAGCTGTA-3' 141 cleavage site: 126

|| || |o||||||||||||o

3'-ATCTCGAGAGAAGTTAGGTTT-5'

DysRNA_arrange_TPM 43317729_20 TTTGGATTGAAGGAGCTCTA

target id: NTTN90_mRNA_106750_cds

112 5'-TCTGGTACAGTTTCTTCAATCCAAGCTGTA-3' 141 cleavage site: 126

|| ||o|o|||||||||||o

3'-ATCTCGAGGAAGTTAGGTTT-5'

DysRNA_arrange_TPM 43316738_21 TTTGGATTGAAGGGAACTCTA

target id: NTTN90_mRNA_106750_cds

112 5'-TCTGGTACAG-TTTCTTCAATCCAAGCTGTA-3' 141 cleavage site: 126

|| || |oo|||||||||||o

3'-ATCTCAAGGGAAGTTAGGTTT-5'

DysRNA_arrange_TPM 40528105_21 TTAGGATTGAAGGGAGCTCTA

target id: NTTN90_mRNA_106750_cds

112 5'-TCTGGTACAG-TTTCTTCAATCC-AAGCTGT-3' 140 cleavage site: 125

|| || |oo||||||||| ||

3'-ATCTCGAGGGAAGTTAGGATT-5'

DysRNA_arrange_TPM 27871038_21 GTTGGATTGAAGGGAGCTCTA

target id: NTTN90_mRNA_106750_cds

112 5'-TCTGGTACAG-TTTCTTCAATCCAAGCTGTA-3' 141 cleavage site: 126

|| || |oo|||||||||||

3'-ATCTCGAGGGAAGTTAGGTTG-5'

DysRNA_arrange_TPM 43321070_21 TTTGGATTGAAGCGAGCTCTA

target id: NTTN90_mRNA_106750_cds

112 5'-TCTGGTACAGTTT-CTTCAATCCAAGCTGTA-3' 141 cleavage site: 126

|| ||o|o |||||||||||o

3'-ATCTCGAGCGAAGTTAGGTTT-5'

DysRNA_arrange_TPM 27959210_20 GTTGTTTTGAGTTTGAGGCC

target id: NTTN90_mRNA_106747_cds

1247 5'-TTAATTGCATTAAACTCAAAA-AATTGCCA-3' 1275 cleavage site: 1260

|| |o|||||||||| ||o

3'-CCGGAGTTTGAGTTTTGTTG-5'

DysRNA_arrange_TPM 02767294_21 AAGAAGATGATTATGGAGGGC

target id: NTTN90_mRNA_63328_cds

3567 5'-TTCAAGTGCTCCATCATCATCTTCTTCAGAT-3' 3597 cleavage site: 3582

|o |||||| |||||||||||

3'-CGGGAGGTATTAGTAGAAGAA-5'

DysRNA_arrange_TPM 31162881_20 CGTTGCTGCGATGATTCATG

target id: NTTN90_mRNA_63328_cds

4239 5'-TGAACTA-GAAATCATCGCAGTAACTTTTCA-3' 4268 cleavage site: 4253

o| | |||||||||||o|||

3'-GTAC-TTAGTAGCGTCGTTGC-5'

DysRNA_arrange_TPM 27913008_20 GTTGCTGCGATGATTCATGA

target id: NTTN90_mRNA_63328_cds

4238 5'-ATGAACTA-GAAATCATCGCAGTAACTTTTC-3' 4267 cleavage site: 4252

o| | |||||||||||o|||

3'-AGTAC-TTAGTAGCGTCGTTG-5'

DysRNA_arrange_TPM 02768834_22 AAGAAGATGATTATGGAGGGCA

target id: NTTN90_mRNA_63328_cds

3566 5'-GTTCAAGTGCTCCATCATCATCTTCTTCAGAT-3' 3597 cleavage site: 3582

|o |||||| |||||||||||

3'-ACGGGAGGTATTAGTAGAAGAA-5'

DysRNA_arrange_TPM 27959210_20 GTTGTTTTGAGTTTGAGGCC

target id: NTTN90_mRNA_106745_cds

1097 5'-TTAATTGCATTAAACTCAAAA-AATTGCCA-3' 1125 cleavage site: 1110

|| |o|||||||||| ||o

3'-CCGGAGTTTGAGTTTTGTTG-5'

Supplemental data 2 (b) tomato sRNA and NLR target alignment

D51_TPM.filter 26469771_21 TTTCCAATTCCACCCATTCCC

target id: Solyc01g008800.1.1

675 5'-GATCTGGGGCATGGGGGGAATTGGTAAGACGAC-3' 707 cleavage site: 692

|||o ||||| |||||||| ||o

3'-CCCT-TACCCACCTTAACC-TTT-5'

D51_TPM.filter 15303725_21 GTTGCTGGCTTCACTGTGTGA

target id: Solyc01g008800.1.1

3879 5'-CTCCGTCAC-CAATGAAGCCAGTTGCTCCTC-3' 3908 cleavage site: 3893

|||| || |||||||||o o|

3'-AGTGTGTCACTTCGGTCGTTG-5'

D51_TPM.filter 24955707_21 TTGCCATGTTTCTCAAACACG

target id: Solyc01g008800.1.1

3950 5'-TAGCCCGTCTCTTCAGAAACATTGCAACTCTT-3' 3981 cleavage site: 3966

||| | || |||||||| ||||

3'-GCACA-AACTCTTTGTACCGTT-5'

D51_TPM.filter 26469519_22 TTTCCAATTCCACCCATTCCCA

target id: Solyc01g008800.1.1

674 5'-GGATCTGGGGCATGGGGGGAATTGGTAAGACGAC-3' 707 cleavage site: 692

||||o ||||| |||||||| ||o

3'-ACCCT-TACCCACCTTAACC-TTT-5'

D51_TPM.filter 20736031_21 TATTTCTGGAAAGATTCTTCT

target id: Solyc11g011090.1.1

7171 5'-AGTTTAGAA-AA-TTTTCCAGAAATCACCGG-3' 7199 cleavage site: 7184

|||| || o|||||||||||

3'-TCTTCTTAGAAAGGTCTTTAT-5'

D51_TPM.filter 26295007_21 TTTGTCGTCTAAAAACTAGAG

target id: Solyc11g011090.1.1

6400 5'-GATTGCTCTAG--TTTAGA-GA-AAATTCCG-3' 6426 cleavage site: 6411

|||||| |||||| || |||

3'-GAGATCAAAAATCTGCTGTTT-5'

D51_TPM.filter 25755064_21 TTTAAATGACGTGGAAACTCT

target id: Solyc11g011080.1.1

2935 5'-CTGCCAGAATTTCCACGGCAATTAGACACAA-3' 2965 cleavage site: 2950

||| |||||||| || |||o|

3'-TCTCAAAGGTGCAGTAAATTT-5'

D51_TPM.filter 25490144_21 TTCCATGATCTTCTGAGCTTC

target id: Solyc11g011080.1.1

482 5'-TGAATGAA-CTAGTGAAGATCATGGAATGCAA-3' 512 cleavage site: 497

||| || o |||||||||||||

3'-CTTCGAGT-CTTCTAGTACCTT-5'

D51_TPM.filter 16350356_22 CATCTGTTCTACTTTCATTGGT

target id: Solyc11g011080.1.1

120 5'-TGGGAATGAATTGAAGATAGAACAGATGAATCT-3' 152 cleavage site: 137

|o || ||||o |||||||||||

3'-TGGTT-ACTTTCATCTTGTCTAC-5'

D51_TPM.filter 25585965_21 TTCTACTTCCATTACCAAACC

target id: Solyc11g011350.1.1

1089 5'-TGGTTGGTTTGGTAATGGCAGCAGAATTGTT-3' 1119 cleavage site: 1104

||||||||||||| || ||||

3'-CCAAACCATTACCTTCATCTT-5'

D51_TPM.filter 25585868_22 TTCTACTTCCATTACCAAACCA

target id: Solyc11g011350.1.1

1088 5'-TTGGTTGGTTTGGTAATGGCAGCAGAATTGTT-3' 1119 cleavage site: 1104

|||||||||||||| || ||||

3'-ACCAAACCATTACCTTCATCTT-5'

D51_TPM.filter 23336413_21 TCTACTTCCATTACCAAACCA

target id: Solyc11g011350.1.1

1088 5'-TTGGTTGGTTTGGTAATGGCAGCAGAATTGT-3' 1118 cleavage site: 1103

|||||||||||||| || |||

3'-ACCAAACCATTACCTTCATCT-5'

D51_TPM.filter 25585907_21 TTCTACTTCCATTACCAAACT

target id: Solyc11g011350.1.1

1089 5'-TGGTTGGTTTGGTAATGGCAGCAGAATTGTT-3' 1119 cleavage site: 1104

o|||||||||||| || ||||

3'-TCAAACCATTACCTTCATCTT-5'

D51_TPM.filter 23721561_22 TCTTTCCTACTCCTCCCATACC

target id: Solyc05g006630.2.1

679 5'-TGTACGGAATGGGAGGAGTGGGCAAGACCACT-3' 710 cleavage site: 695

|| |||||||||||o|| ||||

3'-CCATACCCTCCTCATCCTTTCT-5'

D51_TPM.filter 23721573_22 TCTTTCCTACTCCTCCCATACT

target id: Solyc05g006630.2.1

679 5'-TGTACGGAATGGGAGGAGTGGGCAAGACCACT-3' 710 cleavage site: 695

o| |||||||||||o|| ||||

3'-TCATACCCTCCTCATCCTTTCT-5'

D51_TPM.filter 03891831_24 AGAAAGACTGTTTCTGATAGACGG

target id: Solyc05g006630.2.1

4173 5'-GCGGATGGTCTATCAGAAACAATCTCTCTACCTT-3' 4206 cleavage site: 4191

|||||||||||||| ||| |||

3'-GGCAGATAGTCTTTGTCAGAAAGA-5'

D51_TPM.filter 04105884_24 AGAGAGGTTGTTTCTGATAGACGT

target id: Solyc05g006630.2.1

4172 5'-TGCGGATGGTCTATCAGAAACAATCTCTCTACCTT-3' 4206 cleavage site: 4191

|o |||||||||||||||o||||||

3'-TG-CAGATAGTCTTTGTTGGAGAGA-5'

D51_TPM.filter 23720788_20 TCTTTCCTACTCCTCCCATA

target id: Solyc05g006630.2.1

681 5'-TACGGAATGGGAGGAGTGGGCAAGACCACT-3' 710 cleavage site: 695

|||||||||||o|| ||||

3'-ATACCCTCCTCATCCTTTCT-5'

D51_TPM.filter 08114450_24 ATAGAGTGGTTGTTTCTGAAAGAC

target id: Solyc05g006630.2.1

4175 5'-GGATGGTCTATCAGAAACAATCTCTCTACCTTCT-3' 4208 cleavage site: 4193

|||| ||||||||||o| |||||

3'-CAGAAAGTCTTTGTTGGTGAGATA-5'

D51_TPM.filter 04118657_24 AGAGACGTTGTTTCTGATAGAACG

target id: Solyc05g006630.2.1

4173 5'-GCGGATGGTCTATCAGAAACAATCTCTCTACCTT-3' 4206 cleavage site: 4191

o| ||||||||||||||o |||||

3'-GCAAGATAGTCTTTGTTGCAGAGA-5'

D51_TPM.filter 04102043_23 AGAGAGATTGTTTCTGATAGACC

target id: Solyc05g006630.2.1

4174 5'-CGGATGGTCTATCAGAAACAATCTCTCTACCTT-3' 4206 cleavage site: 4191

|||||||||||||||||||||||

3'-CCAGATAGTCTTTGTTAGAGAGA-5'

D51_TPM.filter 04105614_24 AGAGAGGTTGTTTCTGATAGACGC

target id: Solyc05g006630.2.1

4173 5'-GCGGATGGTCTATCAGAAACAATCTCTCTACCTT-3' 4206 cleavage site: 4191

|||||||||||||||o||||||

3'-CGCAGATAGTCTTTGTTGGAGAGA-5'

D51_TPM.filter 04150134_24 AGAGATTGTTTCTAGAGTAGAACA

target id: Solyc05g006630.2.1

4173 5'-GCGGATGGTCTA-TC-AGAAACAATCTCTCTACC-3' 4204 cleavage site: 4189

|| |||| || |||||||||||||

3'-ACAAGATGAGATCTTTGTTAGAGA-5'

D51_TPM.filter 03895766_24 AGAAAGGATTGTTTCTGATAGACC

target id: Solyc05g006630.2.1

4174 5'-CGGATGGTCTATCAGAAACAA-TCTCTCTACCTT-3' 4206 cleavage site: 4191

|||||||||||||||| o|| |||

3'-CCAGATAGTCTTTGTTAGGAAAGA-5'

D51_TPM.filter 04194430_24 AGAGGTTGTTTCTGATAGACGTTC

target id: Solyc05g006630.2.1

4170 5'-AATGCGGATGGTCTATCAGAAACAATCTCTCTACC-3' 4204 cleavage site: 4189

|o|o |||||||||||||||o||||

3'-CTTG-CAGATAGTCTTTGTTGGAGA-5'

D51_TPM.filter 04119374_24 AGAGACGTTGTTTCTGATAGAACA

target id: Solyc05g006630.2.1

4173 5'-GCGGATGGTCTATCAGAAACAATCTCTCTACCTT-3' 4206 cleavage site: 4191

|| ||||||||||||||o |||||

3'-ACAAGATAGTCTTTGTTGCAGAGA-5'

D51_TPM.filter 03891018_24 AGAAAGACTGTTTCTGATAGACGA

target id: Solyc05g006630.2.1

4173 5'-GCGGATGGTCTATCAGAAACAATCTCTCTACCTT-3' 4206 cleavage site: 4191

|||||||||||||| ||| |||

3'-AGCAGATAGTCTTTGTCAGAAAGA-5'

D51_TPM.filter 04128258_24 AGAGACTGTTTCCGATAGACGTTC

target id: Solyc05g006630.2.1

4170 5'-AATGCGGATGGTCTATCAGAAACAATCTCTCTACC-3' 4204 cleavage site: 4189

|o|o ||||||| |||||| |||||

3'-CTTG-CAGATAGCCTTTGTCAGAGA-5'

D51_TPM.filter 14624692_22 GTATTTTGAGCATGTAGACATG

target id: Solyc05g006630.2.1

3404 5'-TTTGGTATGTTTACATGCTGCAAACTGCTCCGC-3' 3436 cleavage site: 3421

o||||o|||||||| |||| |o|

3'-GTACAGATGTACGA-GTTTTATG-5'

D51_TPM.filter 04100338_24 AGAGAGACTGTTTCCGAAAGACCT

target id: Solyc05g006630.2.1

4173 5'-GCGGATGGTCTATCAGAAACAATCTCTCTACCTT-3' 4206 cleavage site: 4191

||||| || |||||| |||||||

3'-TCCAGAAAGCCTTTGTCAGAGAGA-5'

D51_TPM.filter 19690207_24 TAGAAAGATTGTTTCCGATAAGAC

target id: Solyc05g006630.2.1

4175 5'-GGATGGTC-TATCAGAAACAATCTCTCTACCTTC-3' 4207 cleavage site: 4192

||| |||| |||||||||| ||||

3'-CAGAATAGCCTTTGTTAGAAAGAT-5'

D51_TPM.filter 03893284_24 AGAAAGACTGTTTCTGATAGACGT

target id: Solyc05g006630.2.1

4172 5'-TGCGGATGGTCTATCAGAAACAATCTCTCTACCTT-3' 4206 cleavage site: 4191

|o |||||||||||||| ||| |||

3'-TG-CAGATAGTCTTTGTCAGAAAGA-5'

D51_TPM.filter 03895968_24 AGAAAGGATTGTTTCTGATAGACT

target id: Solyc05g006630.2.1

4174 5'-CGGATGGTCTATCAGAAACAA-TCTCTCTACCTT-3' 4206 cleavage site: 4191

o||||||||||||||| o|| |||

3'-TCAGATAGTCTTTGTTAGGAAAGA-5'

D51_TPM.filter 03894391_24 AGAAAGATTGTTTCCGATAAGACC

target id: Solyc05g006630.2.1

4174 5'-CGGATGGTC-TATCAGAAACAATCTCTCTACCTT-3' 4206 cleavage site: 4191

|||| |||| |||||||||| |||

3'-CCAGAATAGCCTTTGTTAGAAAGA-5'

D51_TPM.filter 23721641_22 TCTTTCCTACTCCTCCCATGCC

target id: Solyc05g006630.2.1

679 5'-TGTACGGAATGGGAGGAGTGGGCAAGACCACT-3' 710 cleavage site: 695

|| |||||||||||o|| ||||

3'-CCGTACCCTCCTCATCCTTTCT-5'

D51_TPM.filter 12134699_24 GAGAGATTGTTTCTGAAAGACTAT

target id: Solyc05g006630.2.1

4172 5'-TGCGGATGGTCTATCAGAAACAATCTCTCTACCT-3' 4205 cleavage site: 4190

||o|||| ||||||||||||||||

3'-TATCAGAAAGTCTTTGTTAGAGAG-5'

D51_TPM.filter 12134986_24 GAGAGATTGTTTCTGATAGACTTC

target id: Solyc05g006630.2.1

4171 5'-ATGCGGATGGTCTATCAGAAACAATCTCTCTACCT-3' 4205 cleavage site: 4190

|| o|||||||||||||||||||||

3'-CT-TCAGATAGTCTTTGTTAGAGAG-5'

D51_TPM.filter 04101898_24 AGAGAGATTGTTTCTGATAGACCC

target id: Solyc05g006630.2.1

4173 5'-GCGGATGGTCTATCAGAAACAATCTCTCTACCTT-3' 4206 cleavage site: 4191

|||||||||||||||||||||||

3'-CCCAGATAGTCTTTGTTAGAGAGA-5'

D51_TPM.filter 04415961_24 AGACTGTTTCTGATAGACCTCGGC

target id: Solyc05g006630.2.1

4168 5'-TAAATGCGGATGGTCTATCAGAAACAATCTCTCTA-3' 4202 cleavage site: 4187

|| || ||||||||||||||| |||

3'-CGGCT-CCAGATAGTCTTTGTCAGA-5'

D51_TPM.filter 23720544_22 TCTTTCCTGCTCCTCCCATACC

target id: Solyc05g006630.2.1

679 5'-TGTACGGAATGGGAGGAGTGGGCAAGACCACT-3' 710 cleavage site: 695

|| ||||||||||oo|| ||||

3'-CCATACCCTCCTCGTCCTTTCT-5'

D51_TPM.filter 23720488_22 TCTTTCCTACTCCTCCCGTACC

target id: Solyc05g006630.2.1

679 5'-TGTACGGAATGGGAGGAGTGGGCAAGACCACT-3' 710 cleavage site: 695

|| |o|||||||||o|| ||||

3'-CCATGCCCTCCTCATCCTTTCT-5'

D51_TPM.filter 12143148_24 GAGAGGTTGTTTCTGATAGACGTT

target id: Solyc05g006630.2.1

4171 5'-ATGCGGATGGTCTATCAGAAACAATCTCTCTACCT-3' 4205 cleavage site: 4190

o|o |||||||||||||||o|||||

3'-TTG-CAGATAGTCTTTGTTGGAGAG-5'

D51_TPM.filter 03902786_24 AGAAAGTTTGTTTCTGATAGACGT

target id: Solyc05g006630.2.1

4172 5'-TGCGGATGGTCTATCAGAAACAATCTCTCTACCTT-3' 4206 cleavage site: 4191

|o ||||||||||||||| || |||

3'-TG-CAGATAGTCTTTGTTTGAAAGA-5'

D51_TPM.filter 23720639_22 TCTTTCCTACTCCTCCCATATC

target id: Solyc05g006630.2.1

679 5'-TGTACGGAATGGGAGGAGTGGGCAAGACCACT-3' 710 cleavage site: 695

|o |||||||||||o|| ||||

3'-CTATACCCTCCTCATCCTTTCT-5'

D51_TPM.filter 23720411_22 TCTTTCCCACTCCTCCCATACC

target id: Solyc05g006630.2.1

679 5'-TGTACGGAATGGGAGGAGTGGGCAAGACCACT-3' 710 cleavage site: 695

|| |||||||||||||| ||||

3'-CCATACCCTCCTCACCCTTTCT-5'

D51_TPM.filter 12134806_24 GAGAGATTGTTTCTGATAGACCAT

target id: Solyc05g006630.2.1

4172 5'-TGCGGATGGTCTATCAGAAACAATCTCTCTACCT-3' 4205 cleavage site: 4190

||||||||||||||||||||||||

3'-TACCAGATAGTCTTTGTTAGAGAG-5'

D51_TPM.filter 23721096_21 TCTTTCCTACTCCTCCCATAC

target id: Solyc05g006630.2.1

680 5'-GTACGGAATGGGAGGAGTGGGCAAGACCACT-3' 710 cleavage site: 695

|||||||||||o|| ||||

3'-CATACCCTCCTCATCCTTTCT-5'

D51_TPM.filter 04551713_24 AGATGTTGTTTCTGATAGACCCGT

target id: Solyc05g006630.2.1

4172 5'-TGCGGAT-GGTCTATCAGAAACAATCTCTCTACC-3' 4204 cleavage site: 4189

|o ||||||||||||||||o |||

3'-TGCCCAGATAGTCTTTGTTGTAGA-5'

D51_TPM.filter 11762965_24 GAAAGACTGTTTCTGAAAGACTAT

target id: Solyc05g006630.2.1

4172 5'-TGCGGATGGTCTATCAGAAACAATCTCTCTACCT-3' 4205 cleavage site: 4190

||o|||| ||||||||| ||| ||

3'-TATCAGAAAGTCTTTGTCAGAAAG-5'

D51_TPM.filter 04150041_24 AGAGATTGTTTCTGATAGACCATC

target id: Solyc05g006630.2.1

4171 5'-ATGCGGATGGTCTATCAGAAACAATCTCTCTACC-3' 4204 cleavage site: 4189

||||||||||||||||||||||||

3'-CTACCAGATAGTCTTTGTTAGAGA-5'

D51_TPM.filter 13022104_24 GGAGAGGTTGTTTCTGATAGACGT

target id: Solyc05g006630.2.1

4172 5'-TGCGGATGGTCTATCAGAAACAATCTCTCTACCTT-3' 4206 cleavage site: 4191

|o |||||||||||||||o|||||o

3'-TG-CAGATAGTCTTTGTTGGAGAGG-5'

D51_TPM.filter 04550849_24 AGATGTTGTTTCTGATAGACCCTC

target id: Solyc05g006630.2.1

4171 5'-ATGCGGATGGTCTATCAGAAACAATCTCTCTACC-3' 4204 cleavage site: 4189

|| ||||||||||||||||o |||

3'-CTCCCAGATAGTCTTTGTTGTAGA-5'

D51_TPM.filter 23583218_22 TCTCTCCTACTCCTCCCATACC

target id: Solyc05g006630.2.1

679 5'-TGTACGGAATGGGAGGAGTGGGCA-AGACCACT-3' 710 cleavage site: 695

|| |||||||||||o|| | |||

3'-CCATACCCTCCTCATCC-TCTCT-5'

D51_TPM.filter 01550132_23 AAGACTGTTTCTGAAAGACTATC

target id: Solyc05g006630.2.1

4171 5'-ATGCGGATGGTCTATCAGAAACAATCTCTCTAC-3' 4203 cleavage site: 4188

|||o|||| ||||||||| |||

3'-CTATCAGAAAGTCTTTGTCAGAA-5'

D51_TPM.filter 03895569_24 AGAAAGATTGTTTCCGATAAGACT

target id: Solyc05g006630.2.1

4174 5'-CGGATGGTC-TATCAGAAACAATCTCTCTACCTT-3' 4206 cleavage site: 4191

o||| |||| |||||||||| |||

3'-TCAGAATAGCCTTTGTTAGAAAGA-5'

D51_TPM.filter 23681971_22 TCTTCCCTACTCCTCCCATACC

target id: Solyc05g006630.2.1

679 5'-TGTACGGAATGGGAGGAGTGGGCAAGACCACT-3' 710 cleavage site: 695

|| |||||||||||o|| ||||

3'-CCATACCCTCCTCATCCCTTCT-5'

D51_TPM.filter 03892961_24 AGAAAGACTGTTTTTGATAGACCT

target id: Solyc05g006630.2.1

4173 5'-GCGGATGGTCTATCAGAAACAATCTCTCTACCTT-3' 4206 cleavage site: 4191

|||||||||o||||| ||| |||

3'-TCCAGATAGTTTTTGTCAGAAAGA-5'

D51_TPM.filter 04150597_24 AGAGATTGATTCTGATAGATTAAG

target id: Solyc05g006630.2.1

4171 5'-ATGCGGATGGTCTATCAGAAACAATCTCTCTACC-3' 4204 cleavage site: 4189

|oo|||||||||| ||||||||

3'-GAATTAGATAGTCTTAGTTAGAGA-5'

D51_TPM.filter 04128232_23 AGAGACTGTTTCCGATAGACGTT

target id: Solyc05g006630.2.1

4171 5'-ATGCGGATGGTCTATCAGAAACAATCTCTCTACC-3' 4204 cleavage site: 4189

o|o ||||||| |||||| |||||

3'-TTG-CAGATAGCCTTTGTCAGAGA-5'

D51_TPM.filter 04099040_24 AGAGAGACTGTTTTCGATAGATCC

target id: Solyc05g006630.2.1

4173 5'-GCGGATGGTCTATCAGAAACAATCTCTCTACCTT-3' 4206 cleavage site: 4191

|o|||||| o||||| |||||||

3'-CCTAGATAGCTTTTGTCAGAGAGA-5'

D51_TPM.filter 04102608_23 AGAGAGATTGTTTCTGATAGATC

target id: Solyc05g006630.2.1

4174 5'-CGGATGGTCTATCAGAAACAATCTCTCTACCTT-3' 4206 cleavage site: 4191

|o|||||||||||||||||||||

3'-CTAGATAGTCTTTGTTAGAGAGA-5'

D51_TPM.filter 04127744_24 AGAGACTGTTTCTGATAAACCTTT

target id: Solyc05g006630.2.1

4170 5'-AATGCGGATGGTCTATCAGAAACAATCTCTCTACC-3' 4204 cleavage site: 4189

oo| ||| ||||||||||| |||||

3'-TTT-CCAAATAGTCTTTGTCAGAGA-5'

D51_TPM.filter 04148791_24 AGAGATTGTTTCTGATAGACTTCA

target id: Solyc05g006630.2.1

4170 5'-AATGCGGATGGTCTATCAGAAACAATCTCTCTACC-3' 4204 cleavage site: 4189

|| o||||||||||||||||||||

3'-ACT-TCAGATAGTCTTTGTTAGAGA-5'

D51_TPM.filter 04101122_24 AGAGAGATTGTTTCTGATAGACCA

target id: Solyc05g006630.2.1

4173 5'-GCGGATGGTCTATCAGAAACAATCTCTCTACCTT-3' 4206 cleavage site: 4191

||||||||||||||||||||||||

3'-ACCAGATAGTCTTTGTTAGAGAGA-5'

D51_TPM.filter 04194591_23 AGAGGTTGTTTCTGATAGACGTT

target id: Solyc05g006630.2.1

4171 5'-ATGCGGATGGTCTATCAGAAACAATCTCTCTACC-3' 4204 cleavage site: 4189

o|o |||||||||||||||o||||

3'-TTG-CAGATAGTCTTTGTTGGAGA-5'

D51_TPM.filter 03895727_23 AGAAAGATTGTTTCTGATAGACT

target id: Solyc05g006630.2.1

4174 5'-CGGATGGTCTATCAGAAACAATCTCTCTACCTT-3' 4206 cleavage site: 4191

o|||||||||||||||||| |||

3'-TCAGATAGTCTTTGTTAGAAAGA-5'

D51_TPM.filter 14438775_24 GTAGAGAGATTGTTTCTGATAGAT

target id: Solyc05g006630.2.1

4175 5'-GGATGGTCTATCAGAAACAATCTCTCTACCTTCT-3' 4208 cleavage site: 4193

o|||||||||||||||||||||||

3'-TAGATAGTCTTTGTTAGAGAGATG-5'

D51_TPM.filter 04100957_24 AGAGAGACTGTTTCCAATAGACCC

target id: Solyc05g006630.2.1

4173 5'-GCGGATGGTCTATCAGAAACAATCTCTCTACCTT-3' 4206 cleavage site: 4191

||||||| |||||| |||||||

3'-CCCAGATAACCTTTGTCAGAGAGA-5'

D51_TPM.filter 04149726_24 AGAGATTGTTTCTGATAGACCCTC

target id: Solyc05g006630.2.1

4171 5'-ATGCGGATGGTCTATCAGAAACAATCTCTCTACC-3' 4204 cleavage site: 4189

|| |||||||||||||||||||||

3'-CTCCCAGATAGTCTTTGTTAGAGA-5'

D51_TPM.filter 03894700_24 AGAAAGATTGTTTCTGATAGACTC

target id: Solyc05g006630.2.1

4173 5'-GCGGATGGTCTATCAGAAACAATCTCTCTACCTT-3' 4206 cleavage site: 4191

o|||||||||||||||||| |||

3'-CTCAGATAGTCTTTGTTAGAAAGA-5'

D51_TPM.filter 04101801_24 AGAGAGATTGTTTCTGATAGATTC

target id: Solyc05g006630.2.1

4173 5'-GCGGATGGTCTATCAGAAACAATCTCTCTACCTT-3' 4206 cleavage site: 4191

oo|||||||||||||||||||||

3'-CTTAGATAGTCTTTGTTAGAGAGA-5'

D51_TPM.filter 04902030_24 AGGATTGTTTCTGATAGACCTTCA

target id: Solyc05g006630.2.1

4169 5'-AAATGCGGATGGTCTATCAGAAACAATCTCTCTAC-3' 4203 cleavage site: 4188

|o| ||||||||||||||||||o

3'-ACTT-CCAGATAGTCTTTGTTAGGA-5'

D51_TPM.filter 04105464_24 AGAGAGGTTGTTTCTGAACAGACA

target id: Solyc05g006630.2.1

4174 5'-CGGATGGTCT-ATCAGAAACAATCTCTCTACCTT-3' 4206 cleavage site: 4191

|||| ||||||||||o||||||

3'-ACAGACAAGTCTTTGTTGGAGAGA-5'

D51_TPM.filter 01728719_24 AAGGATTGTTTCTGATAGATTATC

target id: Solyc05g006630.2.1

4171 5'-ATGCGGATGGTCTATCAGAAACAATCTCTCTACC-3' 4204 cleavage site: 4189

|||oo||||||||||||||||o

3'-CTATTAGATAGTCTTTGTTAGGAA-5'

D51_TPM.filter 12100626_24 GAGAAAGATTGTTTCTGATAGACT

target id: Solyc05g006630.2.1

4174 5'-CGGATGGTCTATCAGAAACAATCTCTCTACCTTC-3' 4207 cleavage site: 4192

o|||||||||||||||||| |||

3'-TCAGATAGTCTTTGTTAGAAAGAG-5'

D51_TPM.filter 23720275_22 TCTTTCCCACTCCTCCCATACT

target id: Solyc05g006630.2.1

679 5'-TGTACGGAATGGGAGGAGTGGGCAAGACCACT-3' 710 cleavage site: 695

o| |||||||||||||| ||||

3'-TCATACCCTCCTCACCCTTTCT-5'

D51_TPM.filter 23720810_22 TCTTTCCTACTCCTCCCGTACT

target id: Solyc05g006630.2.1

679 5'-TGTACGGAATGGGAGGAGTGGGCAAGACCACT-3' 710 cleavage site: 695

o| |o|||||||||o|| ||||

3'-TCATGCCCTCCTCATCCTTTCT-5'

D51_TPM.filter 04099907_23 AGAGAGACTGTTTCCGATAAACT

target id: Solyc05g006630.2.1

4174 5'-CGGATGGTCTATCAGAAACAATCTCTCTACCTT-3' 4206 cleavage site: 4191

o|| |||| |||||| |||||||

3'-TCAAATAGCCTTTGTCAGAGAGA-5'

D51_TPM.filter 12131357_24 GAGAGACTGTTTCTGAAAGACTTT

target id: Solyc05g006630.2.1

4171 5'-ATGCGGATGGTCTATCAGAAACAATCTCTCTACCT-3' 4205 cleavage site: 4190

o| o|||| ||||||||| ||||||

3'-TT-TCAGAAAGTCTTTGTCAGAGAG-5'

D51_TPM.filter 04101483_23 AGAGAGATTGTTTCTGAAAGACT

target id: Solyc05g006630.2.1

4174 5'-CGGATGGTCTATCAGAAACAATCTCTCTACCTT-3' 4206 cleavage site: 4191

o|||| |||||||||||||||||

3'-TCAGAAAGTCTTTGTTAGAGAGA-5'

D51_TPM.filter 12134584_24 GAGAGATTGTTTCTGATAGACCCT

target id: Solyc05g006630.2.1

4172 5'-TGCGGATGGTCTATCAGAAACAATCTCTCTACCT-3' 4205 cleavage site: 4190

||||||||||||||||||||||

3'-TCCCAGATAGTCTTTGTTAGAGAG-5'

D51_TPM.filter 03892022_23 AGAAAGACTGTTTCTGAAAGACT

target id: Solyc05g006630.2.1

4174 5'-CGGATGGTCTATCAGAAACAATCTCTCTACCTT-3' 4206 cleavage site: 4191

o|||| ||||||||| ||| |||

3'-TCAGAAAGTCTTTGTCAGAAAGA-5'

D51_TPM.filter 13550532_24 GGTGAGGTTGTTTCTGATAGACGT

target id: Solyc05g006630.2.1

4172 5'-TGCGGATGGTCTATCAGAAACAATCTC-TCTACCT-3' 4205 cleavage site: 4190

|o |||||||||||||||o||| o|

3'-TG-CAGATAGTCTTTGTTGGAGTGG-5'

D51_TPM.filter 03894949_24 AGAAAGATTGTTTCTGATAGAACC

target id: Solyc05g006630.2.1

4174 5'-CGGATGG-TCTATCAGAAACAATCTCTCTACCTT-3' 4206 cleavage site: 4191

|| ||||||||||||||||| |||

3'-CCAAGATAGTCTTTGTTAGAAAGA-5'

D51_TPM.filter 03891198_24 AGAAAGACTGTTTTCGATAGACCA

target id: Solyc05g006630.2.1

4173 5'-GCGGATGGTCTATCAGAAACAATCTCTCTACCTT-3' 4206 cleavage site: 4191

||||||||| o||||| ||| |||

3'-ACCAGATAGCTTTTGTCAGAAAGA-5'

D51_TPM.filter 04150186_24 AGAGATTGTTTCTGATAGACCATT

target id: Solyc05g006630.2.1

4171 5'-ATGCGGATGGTCTATCAGAAACAATCTCTCTACC-3' 4204 cleavage site: 4189

o|||||||||||||||||||||||

3'-TTACCAGATAGTCTTTGTTAGAGA-5'

D51_TPM.filter 12134589_24 GAGAGATTGTTTCTGATAGATTCT

target id: Solyc05g006630.2.1

4172 5'-TGCGGATGGTCTATCAGAAACAATCTCTCTACCT-3' 4205 cleavage site: 4190

oo||||||||||||||||||||

3'-TCTTAGATAGTCTTTGTTAGAGAG-5'

D51_TPM.filter 12286999_21 GAGGTTGTTTCTGATAGACGT

target id: Solyc05g006630.2.1

4172 5'-TGCGGATGGTCTATCAGAAACAATCTCTCTAC-3' 4203 cleavage site: 4188

|o |||||||||||||||o|||

3'-TG-CAGATAGTCTTTGTTGGAG-5'

D51_TPM.filter 04419739_23 AGACTGTTTCTGATAGATCCTCA

target id: Solyc05g006630.2.1

4170 5'-AATGCGGATGGTCTATCAGAAACAATCTCTCTA-3' 4202 cleavage site: 4187

|| |o||||||||||||| |||

3'-ACTCCTAGATAGTCTTTGTCAGA-5'

D51_TPM.filter 04099698_24 AGAGAGACTGTTTCTGATAAACCT

target id: Solyc05g006630.2.1

4173 5'-GCGGATGGTCTATCAGAAACAATCTCTCTACCTT-3' 4206 cleavage site: 4191

||| ||||||||||| |||||||

3'-TCCAAATAGTCTTTGTCAGAGAGA-5'

D51_TPM.filter 03892421_24 AGAAAGACTGTTTCTGACAGACCT

target id: Solyc05g006630.2.1

4173 5'-GCGGATGGTCTATCAGAAACAATCTCTCTACCTT-3' 4206 cleavage site: 4191

||||| ||||||||| ||| |||

3'-TCCAGACAGTCTTTGTCAGAAAGA-5'

D51_TPM.filter 01547968_24 AAGACTGTTTTTGATAGACCTTCC

target id: Solyc05g006630.2.1

4170 5'-AATGCGGATGGTCTATCAGAAACAATCTCTCTAC-3' 4203 cleavage site: 4188

||| |||||||||o||||| |||

3'-CCTTCCAGATAGTTTTTGTCAGAA-5'

D51_TPM.filter 13033486_24 GGAGATTGTTTCTGATAGACCATC

target id: Solyc05g006630.2.1

4171 5'-ATGCGGATGGTCTATCAGAAACAATCTCTCTACC-3' 4204 cleavage site: 4189

|||||||||||||||||||||||o

3'-CTACCAGATAGTCTTTGTTAGAGG-5'

D51_TPM.filter 04101414_23 AGAGAGATTGTTTCTGATAGACA

target id: Solyc05g006630.2.1

4174 5'-CGGATGGTCTATCAGAAACAATCTCTCTACCTT-3' 4206 cleavage site: 4191

||||||||||||||||||||||

3'-ACAGATAGTCTTTGTTAGAGAGA-5'

D51_TPM.filter 04344329_24 AGACGTTGTTTCTGACAGACTATT

target id: Solyc05g006630.2.1

4171 5'-ATGCGGATGGTCTATCAGAAACAATCTCTCTACC-3' 4204 cleavage site: 4189

o||o|||| ||||||||||o |||

3'-TTATCAGACAGTCTTTGTTGCAGA-5'

D51_TPM.filter 04105355_24 AGAGAGGTTGTTTCTGATAGACGA

target id: Solyc05g006630.2.1

4173 5'-GCGGATGGTCTATCAGAAACAATCTCTCTACCTT-3' 4206 cleavage site: 4191

|||||||||||||||o||||||

3'-AGCAGATAGTCTTTGTTGGAGAGA-5'

D51_TPM.filter 04102163_24 AGAGAGATTGTTTCTGATAGACCT

target id: Solyc05g006630.2.1

4173 5'-GCGGATGGTCTATCAGAAACAATCTCTCTACCTT-3' 4206 cleavage site: 4191

|||||||||||||||||||||||

3'-TCCAGATAGTCTTTGTTAGAGAGA-5'

D51_TPM.filter 19722314_24 TAGAGAGATTGTTTCTGATAGACC

target id: Solyc05g006630.2.1

4174 5'-CGGATGGTCTATCAGAAACAATCTCTCTACCTTC-3' 4207 cleavage site: 4192

||||||||||||||||||||||||

3'-CCAGATAGTCTTTGTTAGAGAGAT-5'

D51_TPM.filter 01550992_24 AAGACTGTTTTTGATAGACCTTCT

target id: Solyc05g006630.2.1

4170 5'-AATGCGGATGGTCTATCAGAAACAATCTCTCTAC-3' 4203 cleavage site: 4188

o|| |||||||||o||||| |||

3'-TCTTCCAGATAGTTTTTGTCAGAA-5'

D51_TPM.filter 04100262_24 AGAGAGACTGTTTCCGATAGACCC

target id: Solyc05g006630.2.1

4173 5'-GCGGATGGTCTATCAGAAACAATCTCTCTACCTT-3' 4206 cleavage site: 4191

|||||||| |||||| |||||||

3'-CCCAGATAGCCTTTGTCAGAGAGA-5'

D51_TPM.filter 04415284_24 AGACTGTTTTTGATAGACCTTCCA

target id: Solyc05g006630.2.1

4167 5'-ATAAATGCGGATGGTCTATCAGAAACAATCTCTCTA-3' 4202 cleavage site: 4187

|| |o| |||||||||o||||| |||

3'-AC-CTT-CCAGATAGTTTTTGTCAGA-5'

D51_TPM.filter 04415020_24 AGACTGTTTCTGATAGACCTCGGT

target id: Solyc05g006630.2.1

4168 5'-TAAATGCGGATGGTCTATCAGAAACAATCTCTCTA-3' 4202 cleavage site: 4187

o| || ||||||||||||||| |||

3'-TGGCT-CCAGATAGTCTTTGTCAGA-5'

D51_TPM.filter 04716074_24 AGGAGATTGTTTCTGAAAGACATT

target id: Solyc05g006630.2.1

4171 5'-ATGCGGATGGTCTATCAGAAACAATCTCTCTACCT-3' 4205 cleavage site: 4190

o|| |||| ||||||||||||||o

3'-TTA-CAGAAAGTCTTTGTTAGAGGA-5'

D51_TPM.filter 23682147_22 TCTTCCCTACTCCTCCCATACT

target id: Solyc05g006630.2.1

679 5'-TGTACGGAATGGGAGGAGTGGGCAAGACCACT-3' 710 cleavage site: 695

o| |||||||||||o|| ||||

3'-TCATACCCTCCTCATCCCTTCT-5'

D51_TPM.filter 04149350_24 AGAGATTGTTTCTGATAGACCCTT

target id: Solyc05g006630.2.1

4171 5'-ATGCGGATGGTCTATCAGAAACAATCTCTCTACC-3' 4204 cleavage site: 4189

o| |||||||||||||||||||||

3'-TTCCCAGATAGTCTTTGTTAGAGA-5'

D51_TPM.filter 04099751_23 AGAGAGACTGTTTCTGAAAGATT

target id: Solyc05g006630.2.1

4174 5'-CGGATGGTCTATCAGAAACAATCTCTCTACCTT-3' 4206 cleavage site: 4191

oo||| ||||||||| |||||||

3'-TTAGAAAGTCTTTGTCAGAGAGA-5'

D51_TPM.filter 04194100_24 AGAGGTTGTTTCTGATAGATCATC

target id: Solyc05g006630.2.1

4171 5'-ATGCGGATGGTCTATCAGAAACAATCTCTCTACC-3' 4204 cleavage site: 4189

||||o||||||||||||||o||||

3'-CTACTAGATAGTCTTTGTTGGAGA-5'

D51_TPM.filter 04101818_23 AGAGAGATTGTTTCTGATAGACT

target id: Solyc05g006630.2.1

4174 5'-CGGATGGTCTATCAGAAACAATCTCTCTACCTT-3' 4206 cleavage site: 4191

o||||||||||||||||||||||

3'-TCAGATAGTCTTTGTTAGAGAGA-5'

D51_TPM.filter 04082495_24 AGAGAAACTGTTACTGATAGATCA

target id: Solyc05g006630.2.1

4173 5'-GCGGATGGTCTATCAGAAACAATCTCTCTACCTT-3' 4206 cleavage site: 4191

||o|||||||| |||| | |||||

3'-ACTAGATAGTCATTGTCAAAGAGA-5'

D51_TPM.filter 04148894_24 AGAGATTGTTTCGGAAAGGCCATC

target id: Solyc05g006630.2.1

4171 5'-ATGCGGATGGTCTATCAGAAACAATCTCTCTACC-3' 4204 cleavage site: 4189

|||||o|| || ||||||||||||

3'-CTACCGGAAAGGCTTTGTTAGAGA-5'

D51_TPM.filter 04105307_23 AGAGAGGTTGTTTCTGATAGACA

target id: Solyc05g006630.2.1

4174 5'-CGGATGGTCTATCAGAAACAATCTCTCTACCTT-3' 4206 cleavage site: 4191

|||||||||||||||o||||||

3'-ACAGATAGTCTTTGTTGGAGAGA-5'

D51_TPM.filter 04101813_22 AGAGAGATTGTTTCTGATAGAC

target id: Solyc05g006630.2.1

4175 5'-GGATGGTCTATCAGAAACAATCTCTCTACCTT-3' 4206 cleavage site: 4191

||||||||||||||||||||||

3'-CAGATAGTCTTTGTTAGAGAGA-5'

D51_TPM.filter 23720471_22 TCTTTCCTACTCCTCCCATGCT

target id: Solyc05g006630.2.1

679 5'-TGTACGGAATGGGAGGAGTGGGCAAGACCACT-3' 710 cleavage site: 695

o| |||||||||||o|| ||||

3'-TCGTACCCTCCTCATCCTTTCT-5'

D51_TPM.filter 01449341_24 AAGAGATTGTTTTTGATAGACCCT

target id: Solyc05g006630.2.1

4172 5'-TGCGGATGGTCTATCAGAAACAATCTCTCTACCT-3' 4205 cleavage site: 4190

|||||||||o|||||||||||

3'-TCCCAGATAGTTTTTGTTAGAGAA-5'

D51_TPM.filter 04127623_24 AGAGACTGTTTCTGATAGACCCTC

target id: Solyc05g006630.2.1

4171 5'-ATGCGGATGGTCTATCAGAAACAATCTCTCTACC-3' 4204 cleavage site: 4189

|| ||||||||||||||| |||||

3'-CTCCCAGATAGTCTTTGTCAGAGA-5'

D51_TPM.filter 14438774_24 GTAGAGAGATTGTTTCTGATAGAC

target id: Solyc05g006630.2.1

4175 5'-GGATGGTCTATCAGAAACAATCTCTCTACCTTCT-3' 4208 cleavage site: 4193

||||||||||||||||||||||||

3'-CAGATAGTCTTTGTTAGAGAGATG-5'

D51_TPM.filter 23734664_22 TCTTTTCTACTCCTCCCATACC

target id: Solyc05g006630.2.1

679 5'-TGTACGGAATGGGAGGAGTGGGCAAGACCACT-3' 710 cleavage site: 695

|| |||||||||||o|o ||||

3'-CCATACCCTCCTCATCTTTTCT-5'

D51_TPM.filter 23721242_22 TCTTTCCTACTCCTCCTATACC

target id: Solyc05g006630.2.1

679 5'-TGTACGGAATGGGAGGAGTGGGCAAGACCACT-3' 710 cleavage site: 695

|| ||o||||||||o|| ||||

3'-CCATATCCTCCTCATCCTTTCT-5'

D51_TPM.filter 23481048_22 TCTGTCCTACTCCTCCCATACC

target id: Solyc05g006630.2.1

679 5'-TGTACGGAATGGGAGGAGTGGGCA-AGACCACT-3' 710 cleavage site: 695

|| |||||||||||o|| | |||

3'-CCATACCCTCCTCATCC-TGTCT-5'

D51_TPM.filter 14438772_24 GTAGAGAGGTTGTTTCTGATAGAC

target id: Solyc05g006630.2.1

4175 5'-GGATGGTCTATCAGAAACAATCTCTCTACCTTCT-3' 4208 cleavage site: 4193

|||||||||||||||o||||||||

3'-CAGATAGTCTTTGTTGGAGAGATG-5'

D51_TPM.filter 04140284_23 AGAGATGTTGTTTCTGTTAGACA

target id: Solyc05g006630.2.1

4174 5'-CGGATGGTCTATCAGAAACAATCTCTCTACCTT-3' 4206 cleavage site: 4191

||||| |||||||||o |||||

3'-ACAGATTGTCTTTGTTGTAGAGA-5'

D51_TPM.filter 04099295_24 AGAGAGACTGTTTTCGATAGACCT

target id: Solyc05g006630.2.1

4173 5'-GCGGATGGTCTATCAGAAACAATCTCTCTACCTT-3' 4206 cleavage site: 4191

|||||||| o||||| |||||||

3'-TCCAGATAGCTTTTGTCAGAGAGA-5'

D51_TPM.filter 04723617_24 AGGAGGTTGTTTCTGATAGATCTT

target id: Solyc05g006630.2.1

4171 5'-ATGCGGATGGTCTATCAGAAACAATCTCTCTACCT-3' 4205 cleavage site: 4190

o| |o||||||||||||||o|||o

3'-TT-CTAGATAGTCTTTGTTGGAGGA-5'

D51_TPM.filter 00661060_24 AAAGATTGTTTCTGATAGACTCTC

target id: Solyc05g006630.2.1

4171 5'-ATGCGGATGGTCTATCAGAAACAATCTCTCTACC-3' 4204 cleavage site: 4189

|| o||||||||||||||||||

3'-CTCTCAGATAGTCTTTGTTAGAAA-5'

D51_TPM.filter 08115503_24 ATAGAGTGGTTGTTTCTGAAAGAT

target id: Solyc05g006630.2.1

4175 5'-GGATGGTCTATCAGAAACAATCTCTCTACCTTCT-3' 4208 cleavage site: 4193

o||| ||||||||||o| |||||

3'-TAGAAAGTCTTTGTTGGTGAGATA-5'

D51_TPM.filter 23721541_23 TCTTTCCTACTCCTCCCATACCT

target id: Solyc05g006630.2.1

678 5'-TTGTACGGAATGGGAGGAGTGGGCAAGACCACT-3' 710 cleavage site: 695

|| |||||||||||o|| ||||

3'-TCCATACCCTCCTCATCCTTTCT-5'

D51_TPM.filter 04104922_24 AGAGAGGTTGTTTCTGATAGACAT

target id: Solyc05g006630.2.1

4172 5'-TGCGGATGGTCTATCAGAAACAATCTCTCTACCTT-3' 4206 cleavage site: 4191

|| |||||||||||||||o||||||

3'-TA-CAGATAGTCTTTGTTGGAGAGA-5'

D51_TPM.filter 23720962_23 TCTTTCCTACTCCTCCCATACCA

target id: Solyc05g006630.2.1

678 5'-TTGTACGGAATGGGAGGAGTGGGCAAGACCACT-3' 710 cleavage site: 695

|| |||||||||||o|| ||||

3'-ACCATACCCTCCTCATCCTTTCT-5'

D51_TPM.filter 04105339_24 AGAGAGGTTGTTTCTGATAGACCT

target id: Solyc05g006630.2.1

4173 5'-GCGGATGGTCTATCAGAAACAATCTCTCTACCTT-3' 4206 cleavage site: 4191

||||||||||||||||o||||||

3'-TCCAGATAGTCTTTGTTGGAGAGA-5'

D51_TPM.filter 04148858_24 AGAGATTGTTTCTGATAAGATGTC

target id: Solyc05g006630.2.1

4171 5'-ATGCGGATGGTC-TATCAGAAACAATCTCTCTACC-3' 4204 cleavage site: 4189

||o o|| |||||||||||||||||

3'-CTG-TAGAATAGTCTTTGTTAGAGA-5'

D51_TPM.filter 01448641_24 AAGAGATTGTTTCTCAGAGACTAT

target id: Solyc05g006630.2.1

4172 5'-TGCGGATGGTCTATCAGAAACAATCTCTCTACCT-3' 4205 cleavage site: 4190

||o|||| | |||||||||||||

3'-TATCAGAGACTCTTTGTTAGAGAA-5'

D51_TPM.filter 23721693_22 TCTTTCCTACTCCTCTCATACC

target id: Solyc05g006630.2.1

679 5'-TGTACGGAATGGGAGGAGTGGGCAAGACCACT-3' 710 cleavage site: 695

|| |||o|||||||o|| ||||

3'-CCATACTCTCCTCATCCTTTCT-5'

D51_TPM.filter 19690230_23 TAGAAAGACTGTTTTTGATAGAC

target id: Solyc05g006630.2.1

4175 5'-GGATGGTCTATCAGAAACAATCTCTCTACCTTC-3' 4207 cleavage site: 4192

||||||||o||||| ||| ||||

3'-CAGATAGTTTTTGTCAGAAAGAT-5'

D51_TPM.filter 04149702_24 AGAGATTGTTTCTGATAGATCTTT

target id: Solyc05g006630.2.1

4170 5'-AATGCGGATGGTCTATCAGAAACAATCTCTCTACC-3' 4204 cleavage site: 4189

oo| |o|||||||||||||||||||

3'-TTT-CTAGATAGTCTTTGTTAGAGA-5'

D51_TPM.filter 03895761_24 AGAAAGGATTGTTTCTGATAGACA

target id: Solyc05g006630.2.1

4174 5'-CGGATGGTCTATCAGAAACAA-TCTCTCTACCTT-3' 4206 cleavage site: 4191

||||||||||||||| o|| |||

3'-ACAGATAGTCTTTGTTAGGAAAGA-5'

D51_TPM.filter 01547094_23 AAGACTGTTTTTGATAGACCTTC

target id: Solyc05g006630.2.1

4170 5'-AATGCGGATGGTCTATCAGAAACAATCTCTCTAC-3' 4203 cleavage site: 4188

|o| |||||||||o||||| |||

3'-CTT-CCAGATAGTTTTTGTCAGAA-5'

D51_TPM.filter 04102331_24 AGAGAGATTGTTTCTGATAGACTT

target id: Solyc05g006630.2.1

4173 5'-GCGGATGGTCTATCAGAAACAATCTCTCTACCTT-3' 4206 cleavage site: 4191

o||||||||||||||||||||||

3'-TTCAGATAGTCTTTGTTAGAGAGA-5'

D51_TPM.filter 04100183_23 AGAGAGACTGTTTTTGATAGATC

target id: Solyc05g006630.2.1

4174 5'-CGGATGGTCTATCAGAAACAATCTCTCTACCTT-3' 4206 cleavage site: 4191

|o|||||||o||||| |||||||

3'-CTAGATAGTTTTTGTCAGAGAGA-5'

D51_TPM.filter 01731232_24 AAGGATTGTTTCTGATAGACCTTC

target id: Solyc05g006630.2.1

4170 5'-AATGCGGATGGTCTATCAGAAACAATCTCTCTACC-3' 4204 cleavage site: 4189

|o| ||||||||||||||||||o

3'-CTT-CCAGATAGTCTTTGTTAGGAA-5'

D51_TPM.filter 04100848_23 AGAGAGACTGTTTTCGATAGATC

target id: Solyc05g006630.2.1

4174 5'-CGGATGGTCTATCAGAAACAATCTCTCTACCTT-3' 4206 cleavage site: 4191

|o|||||| o||||| |||||||

3'-CTAGATAGCTTTTGTCAGAGAGA-5'

D51_TPM.filter 12131742_24 GAGAGACTGTTTCTGATAGACCCT

target id: Solyc05g006630.2.1

4172 5'-TGCGGATGGTCTATCAGAAACAATCTCTCTACCT-3' 4205 cleavage site: 4190

||||||||||||||| ||||||

3'-TCCCAGATAGTCTTTGTCAGAGAG-5'

D51_TPM.filter 04102083_23 AGAGAGATTGTTTCTGATAGATT

target id: Solyc05g006630.2.1

4174 5'-CGGATGGTCTATCAGAAACAATCTCTCTACCTT-3' 4206 cleavage site: 4191

oo|||||||||||||||||||||

3'-TTAGATAGTCTTTGTTAGAGAGA-5'

D51_TPM.filter 04105706_23 AGAGAGGTTGTTTCTGATAGATC

target id: Solyc05g006630.2.1

4174 5'-CGGATGGTCTATCAGAAACAATCTCTCTACCTT-3' 4206 cleavage site: 4191

|o||||||||||||||o||||||

3'-CTAGATAGTCTTTGTTGGAGAGA-5'

D51_TPM.filter 04105654_24 AGAGAGGTTGTTTCTGATAGACGG

target id: Solyc05g006630.2.1

4173 5'-GCGGATGGTCTATCAGAAACAATCTCTCTACCTT-3' 4206 cleavage site: 4191

|||||||||||||||o||||||

3'-GGCAGATAGTCTTTGTTGGAGAGA-5'

D51_TPM.filter 12134681_24 GAGAGATTGTTTCTGATAGACTTT

target id: Solyc05g006630.2.1

4171 5'-ATGCGGATGGTCTATCAGAAACAATCTCTCTACCT-3' 4205 cleavage site: 4190

o| o|||||||||||||||||||||

3'-TT-TCAGATAGTCTTTGTTAGAGAG-5'

D51_TPM.filter 15467463_24 GTTTCTGATAGACCCTCGACCATT

target id: Solyc05g006630.2.1

4165 5'-TTATAAATGCG--GATGGTCTATCAGAAACAATCT-3' 4197 cleavage site: 4182

|||| | || ||||||||||||||

3'-TTAC-CAGCTCCCAGATAGTCTTTG-5'

D51_TPM.filter 03892387_24 AGAAAGACTGTTTCTGACAGACCC

target id: Solyc05g006630.2.1

4173 5'-GCGGATGGTCTATCAGAAACAATCTCTCTACCTT-3' 4206 cleavage site: 4191

||||| ||||||||| ||| |||

3'-CCCAGACAGTCTTTGTCAGAAAGA-5'

D51_TPM.filter 23721661_22 TCTTTCCTATTCCTCCCATACC

target id: Solyc05g006630.2.1

679 5'-TGTACGGAATGGGAGGAGTGGGCAAGACCACT-3' 710 cleavage site: 695

|| |||||||||o|o|| ||||

3'-CCATACCCTCCTTATCCTTTCT-5'

D51_TPM.filter 13676454_20 GGTTGTTCGATGATGTTGTT

target id: Solyc09g007710.2.1

3325 5'-TATTTCACAACATCATCGGCAC-ACCTTTCC-3' 3354 cleavage site: 3339

||||||||||||o || |||

3'-TTGTTGTAGTAGCT-TGTTGG-5'

D51_TPM.filter 01442869_24 AAGAGACTTCTTGAAAAACTAAGA

target id: Solyc05g006620.2.1

4415 5'-AACTTTCTTAGTTTTGTTAAGGAGATTTTTTGTGCT-3' 4450 cleavage site: 4435

|||||||||| |o|||o|| |o|o||

3'-AGAATCAAAA-AGTTCTTC-AGAGAA-5'

D51_TPM.filter 23811530_20 TTAAATGATCTCTTGAATAG

target id: Solyc05g006620.2.1

4458 5'-ACCTTTTCTTCAAGAGA-T-TTTAAACCTC-3' 4485 cleavage site: 4470

o| ||||||||| o |||||

3'-GATAAGTTCTCTAGTAAATT-5'

D51_TPM.filter 08131702_21 ATAGACTGTTCATATTCAATA

target id: Solyc05g006620.2.1

4489 5'-ACCGCGGTTGAATATGAAC-GTCGTGTGTTTG-3' 4519 cleavage site: 4504

o|||||||||||| ||| |o|

3'-ATAACTTATACTTGTCAG-ATA-5'

D51_TPM.filter 21609381_23 TGGTGGAATGATTGAGATCTCTT

target id: Solyc05g007850.1.1

2875 5'-TTTCTAA-ATATCTC-ATCATTCCAACATAACA-3' 2905 cleavage site: 2890

|| | ||||| ||||||||| ||

3'-TTCTCTAGAGTTAGTAAGGTGGT-5'

D51_TPM.filter 21889278_21 TGTAGCAATAGATTCTTTGAT

target id: Solyc05g007850.1.1

238 5'-ATTTTCTCAAAGAA-TTA-TGCTACATCTAG-3' 266 cleavage site: 251

|||||||| o|| |||||||

3'-TAGTTTCTTAGATAACGATGT-5'

D51_TPM.filter 00871983_22 AAACGATATGAAGCTTTGATTA

target id: Solyc01g014840.2.1

2554 5'-CTTCGTGAGCTAAGCTTCTTATCGTTTTCTCG-3' 2585 cleavage site: 2570

|o| | ||||||| ||||||||

3'-ATTAGTTTCGAAGTATAGCAAA-5'

D51_TPM.filter 25095444_24 TTGTGTCTCCAATTCTGTAATGGG

target id: Solyc01g014840.2.1

3906 5'-AGTATCCCA--AC--AATTGGAGACACAATGCAC-3' 3935 cleavage site: 3920

|||| || ||||||||||||||

3'-GGGTAATGTCTTAACCTCTGTGTT-5'

D51_TPM.filter 07886306_21 ATAAGCAACAATCTCTGTTCA

target id: Solyc01g102850.1.1

2998 5'-AAAAGTGAACAGAGATTGTTGACATTGTATGTG-3' 3030 cleavage site: 3015

|||||||||||||||| | ||o|

3'-ACTTGTCTCTAACAAC-G-AATA-5'

D51_TPM.filter 19421447_22 TAAGCAACAATCTCTGTTCATA

target id: Solyc01g102850.1.1

2996 5'-ACAAAAGTGAACAGAGATTGTTGACATTGTATGT-3' 3029 cleavage site: 3014

o|||||||||||||||| | ||o

3'-ATACTTGTCTCTAACAAC-G-AAT-5'

D51_TPM.filter 26816211_21 TTTTGTTGGTGAACCTTTTTG

target id: Solyc01g102850.1.1

2976 5'-GGTTCCAAGCTGGTTCAGCAACAAAAGTGAA-3' 3006 cleavage site: 2991

|||o |||||| ||||||||

3'-GTTTTTCCAAGTGGTTGTTTT-5'

D51_TPM.filter 07886425_20 ATAAGCAACAATCTCTGTTC

target id: Solyc01g102850.1.1

2999 5'-AAAGTGAACAGAGATTGTTGACATTGTATGTG-3' 3030 cleavage site: 3015

||||||||||||||| | ||o|

3'-CTTGTCTCTAACAAC-G-AATA-5'

D51_TPM.filter 19421557_21 TAAGCAACAATCTCTGTTCAT

target id: Solyc01g102850.1.1

2997 5'-CAAAAGTGAACAGAGATTGTTGACATTGTATGT-3' 3029 cleavage site: 3014

o|||||||||||||||| | ||o

3'-TACTTGTCTCTAACAAC-G-AAT-5'

D51_TPM.filter 01861783_23 AAGCAACAATCTCTGTTCATATT

target id: Solyc01g102850.1.1

2994 5'-CAACAAAAGTGAACAGAGATTGTTGACATTGTATG-3' 3028 cleavage site: 3013

|| o|||||||||||||||| | ||

3'-TTATACTTGTCTCTAACAAC-G-AA-5'

D51_TPM.filter 07886270_21 ATAAGCAACAATCTCTGTTCG

target id: Solyc01g102850.1.1

2998 5'-AAAAGTGAACAGAGATTGTTGACATTGTATGTG-3' 3030 cleavage site: 3015

o||||||||||||||| | ||o|

3'-GCTTGTCTCTAACAAC-G-AATA-5'

D51_TPM.filter 07886623_21 ATAAGCAACAATCTCTGTTCT

target id: Solyc01g102850.1.1

2998 5'-AAAAGTGAACAGAGATTGTTGACATTGTATGTG-3' 3030 cleavage site: 3015

||||||||||||||| | ||o|

3'-TCTTGTCTCTAACAAC-G-AATA-5'

D51_TPM.filter 19421763_22 TAAGCAACAATCTCTGTTCATG

target id: Solyc01g102850.1.1

2996 5'-ACAAAAGTGAACAGAGATTGTTGACATTGTATGT-3' 3029 cleavage site: 3014

o|||||||||||||||| | ||o

3'-GTACTTGTCTCTAACAAC-G-AAT-5'

D51_TPM.filter 20923589_22 TGAGAACTTTGAAGGCCGAAGA

target id: Solyc04g007490.2.1

2613 5'-TTTCATCTTTGGCAACTTCAAATTTCTCAGGGTG-3' 2646 cleavage site: 2631

||||o||| ||||||| ||||||

3'-AGAAGCCG--GAAGTTTCAAGAGT-5'

D51_TPM.filter 22378270_23 TCAGACTGTCTTTTATTAATATA

target id: Solyc04g007490.2.1

1655 5'-TCACTGATATTAGTAAAAGA-GGTGCTGATATAT-3' 1687 cleavage site: 1672

||||||o||||||| o|| ||||

3'-ATATAATTATTTTCTGTCA-GACT-5'

D51_TPM.filter 20923365_21 TGAGAACTTTGAAGGCCGAAG

target id: Solyc04g007490.2.1

2614 5'-TTCATCTTTGGCAACTTCAAATTTCTCAGGGTG-3' 2646 cleavage site: 2631

|||o||| ||||||| ||||||

3'-GAAGCCG--GAAGTTTCAAGAGT-5'

D51_TPM.filter 20922957_20 TGAGAACTTTGAAGGCCGAA

target id: Solyc04g007490.2.1

2615 5'-TCATCTTTGGCAACTTCAAATTTCTCAGGGTG-3' 2646 cleavage site: 2631

||o||| ||||||| ||||||

3'-AAGCCG--GAAGTTTCAAGAGT-5'

D51_TPM.filter 12117058_21 GAGAACTTTGAAGGCCGAAGA

target id: Solyc04g007490.2.1

2613 5'-TTTCATCTTTGGCAACTTCAAATTTCTCAGGGT-3' 2645 cleavage site: 2630

||||o||| ||||||| |||||

3'-AGAAGCCG--GAAGTTTCAAGAG-5'

D51_TPM.filter 12116877_20 GAGAACTTTGAAGGCCGAAG

target id: Solyc04g007490.2.1

2614 5'-TTCATCTTTGGCAACTTCAAATTTCTCAGGGT-3' 2645 cleavage site: 2630

|||o||| ||||||| |||||

3'-GAAGCCG--GAAGTTTCAAGAG-5'

D51_TPM.filter 26409180_21 TTTCGAGTCCTGAAATCCATC

target id: Solyc04g007490.2.1

3416 5'-TCCCTGATGGTTTTCAGGAC-CTAAATTGTT-3' 3445 cleavage site: 3430

||||| ||||||||| | |||

3'-CTACCTAAAGTCCTGAGCTTT-5'

D51_TPM.filter 07221053_24 ACTAATGCTGACTATATCTGAATT

target id: Solyc04g007490.2.1

2829 5'-GAGTAAGTTGAGATAT-GTTAGCATTAGTGATGG-3' 2861 cleavage site: 2846

|o|| |||||| ||o|||||||||

3'-TTAAGTCTATATCAGTCGTAATCA-5'

D51_TPM.filter 25569464_21 TTCTAACCTTGTATCGCTTGT

target id: Solyc05g054010.2.1

2997 5'-GTTAAATGAG-GATTTAAGGTTAGAAAATTT-3' 3026 cleavage site: 3011

|oo|| ||| o||||||||||

3'-TGTTCGCTATGTTCCAATCTT-5'

D51_TPM.filter 03712288_24 AATTCTACGTCTAGCTCTGATACT

target id: Solyc05g054010.2.1

3688 5'-GGAGAAG-A-CA-AGCTTGACGTAGAATTCTACG-3' 3718 cleavage site: 3703

|| | || |||| |||||||||||

3'-TCATAGTCTCGATCTGCATCTTAA-5'

D51_TPM.filter 12426895_23 GACACTTTTAGAATTTTGAGATT

target id: Solyc05g054010.2.1

3383 5'-TCCAGAATCTC-AAATCTCTAAAATTGTCTTGTC-3' 3415 cleavage site: 3400

|||||| |||| ||||||| ||||

3'-TTAGAGTTTTA-AGATTTTCACAG-5'

D51_TPM.filter 08974426_24 ATGAATTTTGAATAATTGAAGTGT

target id: Solyc05g054010.2.1

2433 5'-TTTCGATGATTCTATT-TTCAAAATTCATGATCT-3' 2465 cleavage site: 2450

|oo ||| ||| ||||||||||||

3'-TGTGAAGTTAATAAGTTTTAAGTA-5'

D51_TPM.filter 05470250_24 AGCTGAGATCTCTTCATTCTTAAG

target id: Solyc06g008450.2.1

1424 5'-TCAAACTTATCAAAGAAGAGATCTCTGCTTTAGA-3' 1457 cleavage site: 1442

|||| || ||||||||||| |||

3'-GAATTCTTACTTCTCTAGAGTCGA-5'

D51_TPM.filter 22274495_20 TCAAGAGTTTGTAGACGAGG

target id: Solyc04g007060.2.1

2026 5'-AATCTTCTTCTCTACAAACTCTTTCTATGG-3' 2055 cleavage site: 2040

o||o |||||||||||||

3'-GGAGCAGATGTTTGAGAACT-5'

D51_TPM.filter 22922690_24 TCGTGATCTAATGACATTTGTTCT

target id: Solyc04g007060.2.1

861 5'-GTGTGGGAAGCAAATGTCATT-GATCACGTAAAGA-3' 894 cleavage site: 879

o| |o||||||||||| |||||||

3'-TC-TTGTTTACAGTAATCTAGTGCT-5'

D51_TPM.filter 01724746_22 AAGGATCTGACATGCATCTGTC

target id: Solyc04g007060.2.1

1650 5'-CCATCTGCAGA-ACATGTCAGATCTTTCTATT-3' 1680 cleavage site: 1665

o|||| |||||||||||o||

3'-CTGTCTACGTACAGTCTAGGAA-5'

D51_TPM.filter 00227355_22 AAAAGACACAACTTTCTGGATA

target id: Solyc04g007060.2.1

2952 5'-GTATGTACTCCCTGGAGTTGTGTCTTTTTCGAG-3' 2984 cleavage site: 2969

|| ||| oo|||||||||||||

3'-AT-AGGTCTTTCAACACAGAAAA-5'

D51_TPM.filter 05604292_24 AGTAGCTACTCCATTTATTTTAGT

target id: Solyc04g007060.2.1

2933 5'-AATACGCT-AAATAAATGGAGTATG-TACTCCCTG-3' 2965 cleavage site: 2950

o|| |||||||||||||| | ||||

3'-TGATTTTATTTACCTCAT-CGATGA-5'

D51_TPM.filter 11722156_21 GAAAAGAACTTTGAAGAGAGA

target id: Solyc04g007060.2.1

1769 5'-TGTTGAATCTC-TCAAATTTCTTTTCTCCAA-3' 1798 cleavage site: 1783

|||| ||||| ||||||||

3'-AGAGAGAAGTTTCAAGAAAAG-5'

D51_TPM.filter 04746779_21 AGGACAACTACAGAAAGTGGA

target id: Solyc04g007060.2.1

1104 5'-GGAGTACCACTTATTGTAGTTGTCATTGCAGG-3' 1135 cleavage site: 1120

|||||| o|||||||||| o|

3'-AGGTGAAAGACATCAACAG-GA-5'

D51_TPM.filter 11721523_20 GAAAAGAACTTTGAAGAGAG

target id: Solyc04g007060.2.1

1770 5'-GTTGAATCTC-TCAAATTTCTTTTCTCCAA-3' 1798 cleavage site: 1783

|||| ||||| ||||||||

3'-GAGAGAAGTTTCAAGAAAAG-5'

D51_TPM.filter 23251003_21 TCCTTGACATTGTTGTATGAT

target id: Solyc04g011990.1.1

1004 5'-AGGAAATAGTAGAAAAATGTCAAGGACTACC-3' 1034 cleavage site: 1019

|| o|| || |||||||||||

3'-TAGTATGTTGTTACAGTTCCT-5'

D51_TPM.filter 26744759_22 TTTTGATGTGGGTGTAGTTATT

target id: Solyc11g071420.1.1

2256 5'-ACTTGAGGAGCTACGCCCACA-TAAAAACATA-3' 2286 cleavage site: 2271

|o |o||||o|||||| o||||

3'-TTATTGATGTGGGTGTAGTTTT-5'

D51_TPM.filter 10671292_21 ATTATTTTCCTCAAACAAGGA

target id: Solyc12g044180.1.1

1214 5'-TTTGTTCTTTATTTGAGGAAGATAGTGACAT-3' 1244 cleavage site: 1229

||o|| |||||||||o|||o|

3'-AGGAACAAACTCCTTTTATTA-5'

D51_TPM.filter 25866430_21 TTTAGCAAGAGTTGTTTTACC

target id: Solyc05g005330.2.1

1748 5'-GATTGGGTAAGACAACTCTCGCTAACAAGCT-3' 1778 cleavage site: 1763

|||||o|||||||| |||||

3'-CCATTTTGTTGAGAACGATTT-5'

D51_TPM.filter 26653861_22 TTTTAGCAAGAGTTGTTTTACC

target id: Solyc05g005330.2.1

1748 5'-GATTGGGTAAGACAACTCTCGCTAACAAGCTAT-3' 1780 cleavage site: 1765

|||||o|||||||| ||||| ||

3'-CCATTTTGTTGAGAACGATT-TT-5'

D51_TPM.filter 22272625_21 TCAAGAATTTGTAGACGAGGT

target id: Solyc05g005330.2.1

3481 5'-CATTTCCCTCACCT-CAAATTCTTGAAACTG-3' 3510 cleavage site: 3495

|||| || |||||||||||

3'-TGGAGCAGATGTTTAAGAACT-5'

D51_TPM.filter 25866155_21 TTTAGCAAGAGTTGTTTTACT

target id: Solyc05g005330.2.1

1748 5'-GATTGGGTAAGACAACTCTCGCTAACAAGCT-3' 1778 cleavage site: 1763

o||||o|||||||| |||||

3'-TCATTTTGTTGAGAACGATTT-5'

D51_TPM.filter 26654022_22 TTTTAGCAAGAGTTGTTTTACT

target id: Solyc05g005330.2.1

1748 5'-GATTGGGTAAGACAACTCTCGCTAACAAGCTAT-3' 1780 cleavage site: 1765

o||||o|||||||| ||||| ||

3'-TCATTTTGTTGAGAACGATT-TT-5'

D51_TPM.filter 22272871_20 TCAAGAATTTGTAGACGAGG

target id: Solyc05g005330.2.1

3482 5'-ATTTCCCTCACCT-CAAATTCTTGAAACTG-3' 3510 cleavage site: 3495

|||| || |||||||||||

3'-GGAGCAGATGTTTAAGAACT-5'

D51_TPM.filter 24840868_21 TTGGCATCATAGAAGCATGGC

target id: Solyc05g005330.2.1

2014 5'-CTAATGCCATGCTTCTATGATGCCAATAATG-3' 2044 cleavage site: 2029

|||||||||||||||||||||

3'-CGGTACGAAGATACTACGGTT-5'

D51_TPM.filter 22272968_21 TCAAGAATTTGTAGACGAGGC

target id: Solyc05g005330.2.1

3481 5'-CATTTCCCTCACCT-CAAATTCTTGAAACTG-3' 3510 cleavage site: 3495

|||| || |||||||||||

3'-CGGAGCAGATGTTTAAGAACT-5'

D51_TPM.filter 22850645_20 TCGGTCTGCATGCTCCATGT

target id: Solyc05g005330.2.1

3177 5'-TTCTTGTGTGGAGGATGCAGACAGGATCTT-3' 3206 cleavage site: 3191

ooo||||| |||||||| |o

3'-TGTACCTCGTACGTCTGGCT-5'

D51_TPM.filter 26653812_23 TTTTAGCAAGAGTTGTTTTACCT

target id: Solyc05g005330.2.1

1747 5'-GGATTGGGTAAGACAACTCTCGCTAACAAGCTAT-3' 1780 cleavage site: 1765

o|||||o|||||||| ||||| ||

3'-TCCATTTTGTTGAGAACGATT-TT-5'

D51_TPM.filter 23394319_21 TCTGACTTAGCCTGGATATGT

target id: Solyc05g005330.2.1

2326 5'-GGTCCACATATCCAGGCTAAGTCAGAGGATA-3' 2356 cleavage site: 2341

|||||||||||||||||||||

3'-TGTATAGGTCCGATTCAGTCT-5'

D51_TPM.filter 19300725_24 TAAACTTTAAATCTTGCATCCACT

target id: Solyc05g005330.2.1

2880 5'-GTTCCTGTGGGTGCTAGATTTAGAATTTACTGTA-3' 2913 cleavage site: 2898

||||o||| |||||||o| ||||

3'-TCACCTACGTTCTAAATTTCAAAT-5'

D51_TPM.filter 18737068_21 CTCTTCTGCAACTTTGACTGC

target id: Solyc05g005330.2.1

2951 5'-ATGTTGCTGTTAAAGTTGCTGAAGATTCTTC-3' 2981 cleavage site: 2966

|| ||o|||||||| |||||

3'-CGTCAGTTTCAACGTCTTCTC-5'

D51_TPM.filter 23600566_21 TCTTAACACTCCGAAGAATGG

target id: Solyc05g005330.2.1

1863 5'-ACTAACCATTCTTCGGAGTGTTAAGAAGGAT-3' 1893 cleavage site: 1878

|||||||||||||||||||||

3'-GGTAAGAAGCCTCACAATTCT-5'

D51_TPM.filter 22273044_21 TCAAGAATTTGTAGACGAGGA

target id: Solyc05g005330.2.1

3481 5'-CATTTCCCTCACCT-CAAATTCTTGAAACTG-3' 3510 cleavage site: 3495

|||| || |||||||||||

3'-AGGAGCAGATGTTTAAGAACT-5'

D51_TPM.filter 24841039_21 TTGGCATCATAGAAGCATGGT

target id: Solyc05g005330.2.1

2014 5'-CTAATGCCATGCTTCTATGATGCCAATAATG-3' 2044 cleavage site: 2029

o||||||||||||||||||||

3'-TGGTACGAAGATACTACGGTT-5'

D51_TPM.filter 26654357_22 TTTTAGCAAGAGTTGTTTTACA

target id: Solyc05g005330.2.1

1748 5'-GATTGGGTAAGACAACTCTCGCTAACAAGCTAT-3' 1780 cleavage site: 1765

||||o|||||||| ||||| ||

3'-ACATTTTGTTGAGAACGATT-TT-5'

D51_TPM.filter 18062356_21 CTAATTCATTCTCTGGTAGTT

target id: Solyc05g005330.2.1

1905 5'-TGATAAACTACCAGAGAATGAATTAGCAGAT-3' 1935 cleavage site: 1920

|||||||||||||||||||||

3'-TTGATGGTCTCTTACTTAATC-5'

D51_TPM.filter 25866253_21 TTTAGCAAGAGTTGTTTTACA

target id: Solyc05g005330.2.1

1748 5'-GATTGGGTAAGACAACTCTCGCTAACAAGCT-3' 1778 cleavage site: 1763

||||o|||||||| |||||

3'-ACATTTTGTTGAGAACGATTT-5'

D51_TPM.filter 25866391_22 TTTAGCAAGAGTTGTTTTACCT

target id: Solyc05g005330.2.1

1747 5'-GGATTGGGTAAGACAACTCTCGCTAACAAGCT-3' 1778 cleavage site: 1763

o|||||o|||||||| |||||

3'-TCCATTTTGTTGAGAACGATTT-5'

D51_TPM.filter 21236840_20 TGATTGTTGCAACTTTGATT

target id: Solyc05g005330.2.1

3070 5'-AAGATGGTC-AAGTTGCAACATTTGCATGT-3' 3098 cleavage site: 3083

oo|| ||||||||||| |oo

3'-TTAGTTTCAACGTTGTTAGT-5'

D51_TPM.filter 25866557_21 TTTAGCAAGAGTTGTTTTACG

target id: Solyc05g005330.2.1

1748 5'-GATTGGGTAAGACAACTCTCGCTAACAAGCT-3' 1778 cleavage site: 1763

||||o|||||||| |||||

3'-GCATTTTGTTGAGAACGATTT-5'

D51_TPM.filter 25866736_20 TTTAGCAAGAGTTGTTTTAC

target id: Solyc05g005330.2.1

1749 5'-ATTGGGTAAGACAACTCTCGCTAACAAGCT-3' 1778 cleavage site: 1763

||||o|||||||| |||||

3'-CATTTTGTTGAGAACGATTT-5'

D51_TPM.filter 26654347_23 TTTTAGCAAGAGTTGTTTTACCA

target id: Solyc05g005330.2.1

1747 5'-GGATTGGGTAAGACAACTCTCGCTAACAAGCTAT-3' 1780 cleavage site: 1765

|||||o|||||||| ||||| ||

3'-ACCATTTTGTTGAGAACGATT-TT-5'

D51_TPM.filter 25866163_22 TTTAGCAAGAGTTGTTTTACCC

target id: Solyc05g005330.2.1

1747 5'-GGATTGGGTAAGACAACTCTCGCTAACAAGCT-3' 1778 cleavage site: 1763

||||||o|||||||| |||||

3'-CCCATTTTGTTGAGAACGATTT-5'

D51_TPM.filter 26654102_21 TTTTAGCAAGAGTTGTTTTAC

target id: Solyc05g005330.2.1

1749 5'-ATTGGGTAAGACAACTCTCGCTAACAAGCTAT-3' 1780 cleavage site: 1765

||||o|||||||| ||||| ||

3'-CATTTTGTTGAGAACGATT-TT-5'

D51_TPM.filter 22273205_20 TCAAGAATTTGTAGACGAGA

target id: Solyc05g005330.2.1

3482 5'-ATTTCCCTCACCT-CAAATTCTTGAAACTG-3' 3510 cleavage site: 3495

||| || |||||||||||

3'-AGAGCAGATGTTTAAGAACT-5'

D51_TPM.filter 26653916_22 TTTTAGCAAGAGTTGTTTTACG

target id: Solyc05g005330.2.1

1748 5'-GATTGGGTAAGACAACTCTCGCTAACAAGCTAT-3' 1780 cleavage site: 1765

||||o|||||||| ||||| ||

3'-GCATTTTGTTGAGAACGATT-TT-5'

D51_TPM.filter 25866348_22 TTTAGCAAGAGTTGTTTTACCA

target id: Solyc05g005330.2.1

1747 5'-GGATTGGGTAAGACAACTCTCGCTAACAAGCT-3' 1778 cleavage site: 1763

|||||o|||||||| |||||

3'-ACCATTTTGTTGAGAACGATTT-5'

D51_TPM.filter 22272516_21 TCAAGAATTTGTAGACGAGGG

target id: Solyc05g005330.2.1

3481 5'-CATTTCCCTCACCT-CAAATTCTTGAAACTG-3' 3510 cleavage site: 3495

||||| || |||||||||||

3'-GGGAGCAGATGTTTAAGAACT-5'

D51_TPM.filter 10284897_22 ATCTCTGTATGATGTTCTTGTC

target id: Solyc05g005330.2.1

2479 5'-GGTTTGGTAAGAAC-TCATACAGAGAAGCTTT-3' 2509 cleavage site: 2494

|oo|||||| |||||||||||

3'-CTGTTCTTGTAGTATGTCTCTA-5'

D51_TPM.filter 26653934_23 TTTTAGCAAGAGTTGTTTTACCC

target id: Solyc05g005330.2.1

1747 5'-GGATTGGGTAAGACAACTCTCGCTAACAAGCTAT-3' 1780 cleavage site: 1765

||||||o|||||||| ||||| ||

3'-CCCATTTTGTTGAGAACGATT-TT-5'

D51_TPM.filter 25425552_21 TTCGGTAAAACTCCTTTGAGA

target id: Solyc05g005330.2.1

3793 5'-ATCAGTTTTTAAGGAGTTTTATC-AGCTGAA-3' 3822 cleavage site: 3807

|o|o |||||||||||o| |o

3'-AGAGTTTCCTCAAAATGGCTT-5'

D51_TPM.filter 24280214_20 TTATAGTTTGTTTGATGGTA

target id: Solyc06g062440.2.1

1879 5'-CCCTATTCCATCAAACAAGCTACAAAATCT-3' 1908 cleavage site: 1893

|||||||||||o||| ||

3'-ATGGTAGTTTGTTTGATATT-5'

D51_TPM.filter 24280482_22 TTATAGTTTGTTTGATGGTATC

target id: Solyc06g062440.2.1

1877 5'-TTCCCTATTCCATCAAACAAGCTACAAAATCT-3' 1908 cleavage site: 1893

|| |||||||||||o||| ||

3'-CTATGGTAGTTTGTTTGATATT-5'

D51_TPM.filter 11609576_21 ATTTTGTAGATTTGTAGCTTC

target id: Solyc06g062440.2.1

1889 5'-TCAAACAAGCTACAAAATCTACAGGAACTCTC-3' 1920 cleavage site: 1905

||||||| |||||||||oo|

3'-CTTCGATG-TTTAGATGTTTTA-5'

D51_TPM.filter 14630656_21 GTGAAAGAGACATAACTTTGG

target id: Solyc06g062440.2.1

2605 5'-TCATTCTGCAAGTTAAGTCTCTTTTATGTTCC-3' 2636 cleavage site: 2621

|oo |||||| ||||||||o|o

3'-GGT-TTCAATACAGAGAAAGTG-5'

D51_TPM.filter 26793551_21 TTTTGTAGATTTGTAGCTTCA

target id: Solyc06g062440.2.1

1888 5'-ATCAAACAAGCTACAAAATCTACAGGAACTCT-3' 1919 cleavage site: 1904

||||||| |||||||||oo|

3'-ACTTCGATG-TTTAGATGTTTT-5'

D51_TPM.filter 20334398_20 TATAGTTTGTTTGATGGTAT

target id: Solyc06g062440.2.1

1878 5'-TCCCTATTCCATCAAACAAGCTACAAAATC-3' 1907 cleavage site: 1892

|| |||||||||||o|||

3'-TATGGTAGTTTGTTTGATAT-5'

D51_TPM.filter 22272625_21 TCAAGAATTTGTAGACGAGGT

target id: Solyc05g012740.1.1

3331 5'-CAGTTCCCTCACCT-CAAATTCTTGAAACTG-3' 3360 cleavage site: 3345

|||| || |||||||||||

3'-TGGAGCAGATGTTTAAGAACT-5'

D51_TPM.filter 22272871_20 TCAAGAATTTGTAGACGAGG

target id: Solyc05g012740.1.1

3332 5'-AGTTCCCTCACCT-CAAATTCTTGAAACTG-3' 3360 cleavage site: 3345

|||| || |||||||||||

3'-GGAGCAGATGTTTAAGAACT-5'

D51_TPM.filter 22272968_21 TCAAGAATTTGTAGACGAGGC

target id: Solyc05g012740.1.1

3331 5'-CAGTTCCCTCACCT-CAAATTCTTGAAACTG-3' 3360 cleavage site: 3345

|||| || |||||||||||

3'-CGGAGCAGATGTTTAAGAACT-5'

D51_TPM.filter 22273044_21 TCAAGAATTTGTAGACGAGGA

target id: Solyc05g012740.1.1

3331 5'-CAGTTCCCTCACCT-CAAATTCTTGAAACTG-3' 3360 cleavage site: 3345

|||| || |||||||||||

3'-AGGAGCAGATGTTTAAGAACT-5'

D51_TPM.filter 22273205_20 TCAAGAATTTGTAGACGAGA

target id: Solyc05g012740.1.1

3332 5'-AGTTCCCTCACCT-CAAATTCTTGAAACTG-3' 3360 cleavage site: 3345

||| || |||||||||||

3'-AGAGCAGATGTTTAAGAACT-5'

D51_TPM.filter 22272516_21 TCAAGAATTTGTAGACGAGGG

target id: Solyc05g012740.1.1

3331 5'-CAGTTCCCTCACCT-CAAATTCTTGAAACTG-3' 3360 cleavage site: 3345

||||| || |||||||||||

3'-GGGAGCAGATGTTTAAGAACT-5'

D51_TPM.filter 23646402_22 TCTTGCCTACACCGCCCATGCC

target id: Solyc02g036270.2.1

509 5'-TTTGTGGTATGGGTGGTGTAGGTAAGACGACA-3' 540 cleavage site: 525

||o|||||o||||||||o||||

3'-CCGTACCCGCCACATCCGTTCT-5'

D51_TPM.filter 23646461_22 TCTTGCCTACACCGCCCATGCT

target id: Solyc02g036270.2.1

509 5'-TTTGTGGTATGGGTGGTGTAGGTAAGACGACA-3' 540 cleavage site: 525

o|o|||||o||||||||o||||

3'-TCGTACCCGCCACATCCGTTCT-5'

D51_TPM.filter 23646169_22 TCTTGCCTACACCGCCCATGCA

target id: Solyc02g036270.2.1

509 5'-TTTGTGGTATGGGTGGTGTAGGTAAGACGACA-3' 540 cleavage site: 525

|o|||||o||||||||o||||

3'-ACGTACCCGCCACATCCGTTCT-5'

D51_TPM.filter 16288140_21 CATGTGATTGTATTGTTCGTA

target id: Solyc02g036270.2.1

1484 5'-CCTCTTACGAGCCATACAGTCACATGTCAAT-3' 1514 cleavage site: 1499

|||||o| |||||o|||||||

3'-ATGCTTGTTATGTTAGTGTAC-5'

D51_TPM.filter 25283883_21 TTCACAAACATCTCGGAGTCG

target id: Solyc02g036270.2.1

836 5'-CAACGCGACTCCGAGATGTTTGTGAAACAAT-3' 866 cleavage site: 851

|||||||||||||||||||||

3'-GCTGAGGCTCTACAAACACTT-5'

D51_TPM.filter 22451103_21 TCACAAACATCTCGGAGTCGC

target id: Solyc02g036270.2.1

835 5'-ACAACGCGACTCCGAGATGTTTGTGAAACAA-3' 865 cleavage site: 850

|||||||||||||||||||||

3'-CGCTGAGGCTCTACAAACACT-5'

D51_TPM.filter 16288287_20 CATGTGATTGTATTGTTCGT

target id: Solyc02g036270.2.1

1485 5'-CTCTTACGAGCCATACAGTCACATGTCAAT-3' 1514 cleavage site: 1499

||||o| |||||o|||||||

3'-TGCTTGTTATGTTAGTGTAC-5'

D51_TPM.filter 22146615_21 TGTTGATTTTTGTGTGTAGTT

target id: Solyc02g036270.2.1

1074 5'-CCTTAAACAAT-TACAAAAATCCACACCAAA-3' 1103 cleavage site: 1088

||| |o o||||||||| |||

3'-TTGATGTGTGTTTTTAGTTGT-5'

D51_TPM.filter 25906981_21 TTTACGCTTTAGCGCTCCTGC

target id: Solyc02g036270.2.1

1025 5'-CAGTTGCAGGAGCGCTAAAGCGTAAAAGCAA-3' 1055 cleavage site: 1040

|||||||||||||||||||||

3'-CGTCCTCGCGATTTCGCATTT-5'

D51_TPM.filter 24188044_21 TTACGCTTTAGCGCTCCTGCA

target id: Solyc02g036270.2.1

1024 5'-ACAGTTGCAGGAGCGCTAAAGCGTAAAAGCA-3' 1054 cleavage site: 1039

|||||||||||||||||||||

3'-ACGTCCTCGCGATTTCGCATT-5'

D51_TPM.filter 22451027_21 TCACAAACATCTCGGAGTCGT

target id: Solyc02g036270.2.1

835 5'-ACAACGCGACTCCGAGATGTTTGTGAAACAA-3' 865 cleavage site: 850

o||||||||||||||||||||

3'-TGCTGAGGCTCTACAAACACT-5'

D51_TPM.filter 26994070_21 TTTTTGTAATTGTTTAAGGGC

target id: Solyc02g036270.2.1

1067 5'-AGGATGCCCTTAAACAATTACAAAAATCCAC-3' 1097 cleavage site: 1082

|||||||||||||||||||||

3'-CGGGAATTTGTTAATGTTTTT-5'

D51_TPM.filter 26791466_20 TTTTGTAATTGTTTAAGGGC

target id: Solyc02g036270.2.1

1067 5'-AGGATGCCCTTAAACAATTACAAAAATCCA-3' 1096 cleavage site: 1081

||||||||||||||||||||

3'-CGGGAATTTGTTAATGTTTT-5'

D51_TPM.filter 23218350_22 TCCTGCCTACACCGCCCATGCC

target id: Solyc02g036270.2.1

509 5'-TTTGTGGTATGGGTGGTGTAGGTAAGACGACA-3' 540 cleavage site: 525

||o|||||o||||||||o| ||

3'-CCGTACCCGCCACATCCGTCCT-5'

D51_TPM.filter 16017999_21 CACAAACATCTCGGAGTCGCG

target id: Solyc02g036270.2.1

834 5'-GACAACGCGACTCCGAGATGTTTGTGAAACA-3' 864 cleavage site: 849

|||||||||||||||||||||

3'-GCGCTGAGGCTCTACAAACAC-5'

D51_TPM.filter 09830638_21 ATCATCCTGTAATTTGAACGG

target id: Solyc02g036270.2.1

1592 5'-AAGAGCCGTTCAAATTACAGGATGATTTTTT-3' 1622 cleavage site: 1607

|||||||||||||||||||||

3'-GGCAAGTTTAATGTCCTACTA-5'

D51_TPM.filter 21451263_22 TGGGATATTTTTTGGTGTGGAT

target id: Solyc02g036270.2.1

1087 5'-CAAAAATCCACACCAAAAAATATCCCAGGAGT-3' 1118 cleavage site: 1103

||||||||||||||||||||||

3'-TAGGTGTGGTTTTTTATAGGGT-5'

D51_TPM.filter 23646688_21 TCTTGCCTACACCGCCCATGC

target id: Solyc02g036270.2.1

510 5'-TTGTGGTATGGGTGGTGTAGGTAAGACGACA-3' 540 cleavage site: 525

|o|||||o||||||||o||||

3'-CGTACCCGCCACATCCGTTCT-5'

D51_TPM.filter 26668496_21 TTTTACGCTTTAGCGCTCCTG

target id: Solyc02g036270.2.1

1026 5'-AGTTGCAGGAGCGCTAAAGCGTAAAAGCAAG-3' 1056 cleavage site: 1041

|||||||||||||||||||||

3'-GTCCTCGCGATTTCGCATTTT-5'

D51_TPM.filter 23646640_22 TCTTGCCTACACCGCCCATGTC

target id: Solyc02g036270.2.1

509 5'-TTTGTGGTATGGGTGGTGTAGGTAAGACGACA-3' 540 cleavage site: 525

|oo|||||o||||||||o||||

3'-CTGTACCCGCCACATCCGTTCT-5'

D51_TPM.filter 23646467_20 TCTTGCCTACACCGCCCATG

target id: Solyc02g036270.2.1

511 5'-TGTGGTATGGGTGGTGTAGGTAAGACGACA-3' 540 cleavage site: 525

o|||||o||||||||o||||

3'-GTACCCGCCACATCCGTTCT-5'

D51_TPM.filter 22578698_21 TCATCCTGTAATTTGAACGGC

target id: Solyc02g036270.2.1

1591 5'-GAAGAGCCGTTCAAATTACAGGATGATTTTT-3' 1621 cleavage site: 1606

|||||||||||||||||||||

3'-CGGCAAGTTTAATGTCCTACT-5'

D51_TPM.filter 25284143_21 TTCACAAACATCTCGGAGTCA

target id: Solyc02g036270.2.1

836 5'-CAACGCGACTCCGAGATGTTTGTGAAACAAT-3' 866 cleavage site: 851

||||||||||||||||||||

3'-ACTGAGGCTCTACAAACACTT-5'

D51_TPM.filter 21450901_21 TGGGATATTTTTTGGTGTGGA

target id: Solyc02g036270.2.1

1088 5'-AAAAATCCACACCAAAAAATATCCCAGGAGT-3' 1118 cleavage site: 1103

|||||||||||||||||||||

3'-AGGTGTGGTTTTTTATAGGGT-5'

D51_TPM.filter 23540290_22 TCTCGCCTACACCGCCCATGCC

target id: Solyc02g036270.2.1

509 5'-TTTGTGGTATGGGTGGTGTAGGTAAGACGACA-3' 540 cleavage site: 525

||o|||||o||||||||o |||

3'-CCGTACCCGCCACATCCGCTCT-5'

D51_TPM.filter 26791535_21 TTTTGTAATTGTTTAAGGGCA

target id: Solyc02g036270.2.1

1066 5'-GAGGATGCCCTTAAACAATTACAAAAATCCA-3' 1096 cleavage site: 1081

|||||||||||||||||||||

3'-ACGGGAATTTGTTAATGTTTT-5'

D51_TPM.filter 25907065_21 TTTACGCTTTAGCGCTCCTGT

target id: Solyc02g036270.2.1

1025 5'-CAGTTGCAGGAGCGCTAAAGCGTAAAAGCAA-3' 1055 cleavage site: 1040

o||||||||||||||||||||

3'-TGTCCTCGCGATTTCGCATTT-5'

D51_TPM.filter 23646606_22 TCTTGCCTACACCGCCCATGAC

target id: Solyc02g036270.2.1

509 5'-TTTGTGGTATGGGTGGTGTAGGTAAGACGACA-3' 540 cleavage site: 525

o|||||o||||||||o||||

3'-CAGTACCCGCCACATCCGTTCT-5'

D51_TPM.filter 23646244_22 TCTTGCCTACACCGCCCACGCC

target id: Solyc02g036270.2.1

509 5'-TTTGTGGTATGGGTGGTGTAGGTAAGACGACA-3' 540 cleavage site: 525

||o ||||o||||||||o||||

3'-CCGCACCCGCCACATCCGTTCT-5'

D51_TPM.filter 21182359_21 TGATGGTTGTGATTGCTACAA

target id: Solyc02g036270.2.1

793 5'-CCTAGTTGTAGCAATCACAACCATCAGTGCA-3' 823 cleavage site: 808

|||||||||||||||||||||

3'-AACATCGTTAGTGTTGGTAGT-5'

D51_TPM.filter 16810111_23 CGGATTTCTGTAATTATTTTAAG

target id: Solyc02g036270.2.1

1070 5'-ATGCCCTT-AAACAATTACAAAAATCCACACCA-3' 1101 cleavage site: 1086

||| ||| ||||||| ||||||

3'-GAATTTTATTAATGTCTTTAGGC-5'

D51_TPM.filter 16288438_20 CATGTGATTGTATTGTTCGG

target id: Solyc02g036270.2.1

1485 5'-CTCTTACGAGCCATACAGTCACATGTCAAT-3' 1514 cleavage site: 1499

|||o| |||||o|||||||

3'-GGCTTGTTATGTTAGTGTAC-5'

D51_TPM.filter 06515870_20 ACATCTATAGTAGAATCTCT

target id: Solyc02g036270.2.1

1793 5'-TTATTAGAGATTCTACTATAGATGTGCTTC-3' 1822 cleavage site: 1807

||||||||||||||||||||

3'-TCTCTAAGATGATATCTACA-5'

D51_TPM.filter 26668585_21 TTTTACGCTTTAGCGCTCCTA

target id: Solyc02g036270.2.1

1026 5'-AGTTGCAGGAGCGCTAAAGCGTAAAAGCAAG-3' 1056 cleavage site: 1041

||||||||||||||||||||

3'-ATCCTCGCGATTTCGCATTTT-5'

D51_TPM.filter 16288141_20 CATGTGATTGTATTGTTCGC

target id: Solyc02g036270.2.1

1485 5'-CTCTTACGAGCCATACAGTCACATGTCAAT-3' 1514 cleavage site: 1499

|||o| |||||o|||||||

3'-CGCTTGTTATGTTAGTGTAC-5'

D51_TPM.filter 16288446_21 CATGTGATTGTATTGTTCGTG

target id: Solyc02g036270.2.1

1484 5'-CCTCTTACGAGCCATACAGTCACATGTCAAT-3' 1514 cleavage site: 1499

o||||o| |||||o|||||||

3'-GTGCTTGTTATGTTAGTGTAC-5'

D51_TPM.filter 23646300_22 TCTTGCCTACACCGCCCGTGCC

target id: Solyc02g036270.2.1

509 5'-TTTGTGGTATGGGTGGTGTAGGTAAGACGACA-3' 540 cleavage site: 525

||o|o|||o||||||||o||||

3'-CCGTGCCCGCCACATCCGTTCT-5'

D51_TPM.filter 16287866_21 CATGTGACTGTATGGCTCGTA

target id: Solyc02g036270.2.1

1484 5'-CCTCTTACGAGCCATACAGTCACATGTCAAT-3' 1514 cleavage site: 1499

|||||||||||||||||||||

3'-ATGCTCGGTATGTCAGTGTAC-5'

D51_TPM.filter 23219160_21 TCCTGTAATTTGAACGGCTCT

target id: Solyc02g036270.2.1

1588 5'-TTAGAAGAGCCGTTCAAATTACAGGATGATT-3' 1618 cleavage site: 1603

|||||||||||||||||||||

3'-TCTCGGCAAGTTTAATGTCCT-5'

D51_TPM.filter 26994308_21 TTTTTGTAATTGTTTAAGGGT

target id: Solyc02g036270.2.1

1067 5'-AGGATGCCCTTAAACAATTACAAAAATCCAC-3' 1097 cleavage site: 1082

o||||||||||||||||||||

3'-TGGGAATTTGTTAATGTTTTT-5'

D51_TPM.filter 06104606_21 ACAAACATGTCGGAAACGCGT

target id: Solyc02g036270.2.1

833 5'-TGACAACGCGACTCCGAGATGTTTGTGAAAC-3' 863 cleavage site: 848

||||| ||||| ||||||||

3'-TGCGCAAAGGCTGTACAAACA-5'

D51_TPM.filter 23646419_22 TCTTGCCTACACCGCCCATGCG

target id: Solyc02g036270.2.1

509 5'-TTTGTGGTATGGGTGGTGTAGGTAAGACGACA-3' 540 cleavage site: 525

|o|||||o||||||||o||||

3'-GCGTACCCGCCACATCCGTTCT-5'

D51_TPM.filter 26238477_21 TTTGTAATTGTTTAAGGGCAT

target id: Solyc02g036270.2.1

1065 5'-GGAGGATGCCCTTAAACAATTACAAAAATCC-3' 1095 cleavage site: 1080

|||||||||||||||||||||

3'-TACGGGAATTTGTTAATGTTT-5'

D51_TPM.filter 17943644_22 CCTTGCCTACACCGCCCATGCT

target id: Solyc02g036270.2.1

509 5'-TTTGTGGTATGGGTGGTGTAGGTAAGACGACA-3' 540 cleavage site: 525

o|o|||||o||||||||o|||

3'-TCGTACCCGCCACATCCGTTCC-5'

D51_TPM.filter 17943647_22 CCTTGCCTACACCGCCCATGCC

target id: Solyc02g036270.2.1

509 5'-TTTGTGGTATGGGTGGTGTAGGTAAGACGACA-3' 540 cleavage site: 525

||o|||||o||||||||o|||

3'-CCGTACCCGCCACATCCGTTCC-5'

D51_TPM.filter 16018140_21 CACAAACATCTCGGAGTCGCA

target id: Solyc02g036270.2.1

834 5'-GACAACGCGACTCCGAGATGTTTGTGAAACA-3' 864 cleavage site: 849

||||||||||||||||||||

3'-ACGCTGAGGCTCTACAAACAC-5'

D51_TPM.filter 23646712_23 TCTTGCCTACACCGCCCATGCCA

target id: Solyc02g036270.2.1

508 5'-ATTTGTGGTATGGGTGGTGTAGGTAAGACGACA-3' 540 cleavage site: 525

|||o|||||o||||||||o||||

3'-ACCGTACCCGCCACATCCGTTCT-5'

D51_TPM.filter 06515505_21 ACATCTATAGTAGAATCTCTA

target id: Solyc02g036270.2.1

1792 5'-ATTATTAGAGATTCTACTATAGATGTGCTTC-3' 1822 cleavage site: 1807

|||||||||||||||||||||

3'-ATCTCTAAGATGATATCTACA-5'

D51_TPM.filter 21109233_21 TGACTGTATGGCTCGTAAGAG

target id: Solyc02g036270.2.1

1480 5'-AGAACCTCTTACGAGCCATACAGTCACATGT-3' 1510 cleavage site: 1495

|||||||||||||||||||||

3'-GAGAATGCTCGGTATGTCAGT-5'

D51_TPM.filter 24188068_20 TTACGCTTTAGCGCTCCTGC

target id: Solyc02g036270.2.1

1025 5'-CAGTTGCAGGAGCGCTAAAGCGTAAAAGCA-3' 1054 cleavage site: 1039

||||||||||||||||||||

3'-CGTCCTCGCGATTTCGCATT-5'

D51_TPM.filter 22578697_21 TCATCCTGTAATTTGAACGGT

target id: Solyc02g036270.2.1

1591 5'-GAAGAGCCGTTCAAATTACAGGATGATTTTT-3' 1621 cleavage site: 1606

o||||||||||||||||||||

3'-TGGCAAGTTTAATGTCCTACT-5'

D51_TPM.filter 23646515_22 TCTTGCCTACACCGCCCATCCT

target id: Solyc02g036270.2.1

509 5'-TTTGTGGTATGGGTGGTGTAGGTAAGACGACA-3' 540 cleavage site: 525

o| |||||o||||||||o||||

3'-TCCTACCCGCCACATCCGTTCT-5'

D51_TPM.filter 06187027_21 ACAACGATCTTTTCTGAATAG

target id: Solyc05g008070.2.1

949 5'-AAAAGCTATTCAGAAAAGATCGTTGTCCTCC-3' 979 cleavage site: 964

|||||||||||||||||||||

3'-GATAAGTCTTTTCTAGCAACA-5'

D51_TPM.filter 22612814_21 TCATTTCTTGATTGTTGACTC

target id: Solyc05g008070.2.1

674 5'-TAGTGGAGTCAACAATCAAGAAATGAGCAAT-3' 704 cleavage site: 689

|||||||||||||||||||||

3'-CTCAGTTGTTAGTTCTTTACT-5'

D51_TPM.filter 06675206_21 ACGATCTTTTCTGAATAGCTT

target id: Solyc05g008070.2.1

946 5'-CCAAAAAGCTATTCAGAAAAGATCGTTGTCC-3' 976 cleavage site: 961

|||||||||||||||||||||

3'-TTCGATAAGTCTTTTCTAGCA-5'

D51_TPM.filter 25866430_21 TTTAGCAAGAGTTGTTTTACC

target id: Solyc05g008070.2.1

538 5'-GTATAGGTAAGACAACTCTTGCTAGAAAAGT-3' 568 cleavage site: 553

|||||o|||||||||||||o|

3'-CCATTTTGTTGAGAACGATTT-5'

D51_TPM.filter 26653861_22 TTTTAGCAAGAGTTGTTTTACC

target id: Solyc05g008070.2.1

538 5'-GTATAGGTAAGACAACTCTTGCTAGAAAAGTT-3' 569 cleavage site: 554

|||||o|||||||||||||o||

3'-CCATTTTGTTGAGAACGATTTT-5'

D51_TPM.filter 06186295_21 ACAACGATCTTTTCTGAATAA

target id: Solyc05g008070.2.1

949 5'-AAAAGCTATTCAGAAAAGATCGTTGTCCTCC-3' 979 cleavage site: 964

||||||||||||||||||||

3'-AATAAGTCTTTTCTAGCAACA-5'

D51_TPM.filter 21792301_21 TGCCTTAATGAACTGCTCAGC

target id: Solyc05g008070.2.1

1264 5'-GGATAGCTGAGCAGTTCATTAAGGCAACAAG-3' 1294 cleavage site: 1279

|||||||||||||||||||||

3'-CGACTCGTCAAGTAATTCCGT-5'

D51_TPM.filter 21054313_21 TGACGATTAGAAATCTCCTAC

target id: Solyc05g008070.2.1

737 5'-CAAGGGTAGGAGATTTCTAATCGTCATAGAT-3' 767 cleavage site: 752

|||||||||||||||||||||

3'-CATCCTCTAAAGATTAGCAGT-5'

D51_TPM.filter 25866155_21 TTTAGCAAGAGTTGTTTTACT

target id: Solyc05g008070.2.1

538 5'-GTATAGGTAAGACAACTCTTGCTAGAAAAGT-3' 568 cleavage site: 553

o||||o|||||||||||||o|

3'-TCATTTTGTTGAGAACGATTT-5'

D51_TPM.filter 22612900_21 TCATTTCTTGATTGTTGACTT

target id: Solyc05g008070.2.1

674 5'-TAGTGGAGTCAACAATCAAGAAATGAGCAAT-3' 704 cleavage site: 689

o||||||||||||||||||||

3'-TTCAGTTGTTAGTTCTTTACT-5'

D51_TPM.filter 26654022_22 TTTTAGCAAGAGTTGTTTTACT

target id: Solyc05g008070.2.1

538 5'-GTATAGGTAAGACAACTCTTGCTAGAAAAGTT-3' 569 cleavage site: 554

o||||o|||||||||||||o||

3'-TCATTTTGTTGAGAACGATTTT-5'

D51_TPM.filter 06673952_21 ACGATCTTTTCTGAATAGCTC

target id: Solyc05g008070.2.1

946 5'-CCAAAAAGCTATTCAGAAAAGATCGTTGTCC-3' 976 cleavage site: 961

||||||||||||||||||||

3'-CTCGATAAGTCTTTTCTAGCA-5'

D51_TPM.filter 24963827_22 TTGCCTTAATGAACTGCTCAGC

target id: Solyc05g008070.2.1

1264 5'-GGATAGCTGAGCAGTTCATTAAGGCAACAAGC-3' 1295 cleavage site: 1280

||||||||||||||||||||||

3'-CGACTCGTCAAGTAATTCCGTT-5'

D51_TPM.filter 06673767_20 ACGATCTTTTCTGAATAGCT

target id: Solyc05g008070.2.1

947 5'-CAAAAAGCTATTCAGAAAAGATCGTTGTCC-3' 976 cleavage site: 961

||||||||||||||||||||

3'-TCGATAAGTCTTTTCTAGCA-5'

D51_TPM.filter 06673453_22 ACGATCTTTTCTGAATAGCTTT

target id: Solyc05g008070.2.1

945 5'-ACCAAAAAGCTATTCAGAAAAGATCGTTGTCC-3' 976 cleavage site: 961

||||||||||||||||||||||

3'-TTTCGATAAGTCTTTTCTAGCA-5'

D51_TPM.filter 16681326_21 CGATCTTTTCTGAATAGCTTT

target id: Solyc05g008070.2.1

945 5'-ACCAAAAAGCTATTCAGAAAAGATCGTTGTC-3' 975 cleavage site: 960

|||||||||||||||||||||

3'-TTTCGATAAGTCTTTTCTAGC-5'

D51_TPM.filter 04747075_21 AGGACAACGATCTTTTCTGAA

target id: Solyc05g008070.2.1

952 5'-AGCTATTCAGAAAAGATCGTTGTCCTCCACA-3' 982 cleavage site: 967

|||||||||||||||||||||

3'-AAGTCTTTTCTAGCAACAGGA-5'

D51_TPM.filter 22612566_21 TCATTTCTTGATTGTTGACTA

target id: Solyc05g008070.2.1

674 5'-TAGTGGAGTCAACAATCAAGAAATGAGCAAT-3' 704 cleavage site: 689

||||||||||||||||||||

3'-ATCAGTTGTTAGTTCTTTACT-5'

D51_TPM.filter 26653812_23 TTTTAGCAAGAGTTGTTTTACCT

target id: Solyc05g008070.2.1

537 5'-GGTATAGGTAAGACAACTCTTGCTAGAAAAGTT-3' 569 cleavage site: 554

||||||o|||||||||||||o||

3'-TCCATTTTGTTGAGAACGATTTT-5'

D51_TPM.filter 06674117_21 ACGATCTTTTCTGAATAGCTA

target id: Solyc05g008070.2.1

946 5'-CCAAAAAGCTATTCAGAAAAGATCGTTGTCC-3' 976 cleavage site: 961

||||||||||||||||||||

3'-ATCGATAAGTCTTTTCTAGCA-5'

D51_TPM.filter 15702558_21 CAACGATCTTTTCTGAATAGC

target id: Solyc05g008070.2.1

948 5'-AAAAAGCTATTCAGAAAAGATCGTTGTCCTC-3' 978 cleavage site: 963

|||||||||||||||||||||

3'-CGATAAGTCTTTTCTAGCAAC-5'

D51_TPM.filter 06299020_21 ACAGCGATCTTTTCTGAATAG

target id: Solyc05g008070.2.1

949 5'-AAAAGCTATTCAGAAAAGATCGTTGTCCTCC-3' 979 cleavage site: 964

|||||||||||||||||o|||

3'-GATAAGTCTTTTCTAGCGACA-5'

D51_TPM.filter 06674680_21 ACGATCTTTTCTGAATAGCGT

target id: Solyc05g008070.2.1

946 5'-CCAAAAAGCTATTCAGAAAAGATCGTTGTCC-3' 976 cleavage site: 961

|||||||||||||||||||

3'-TGCGATAAGTCTTTTCTAGCA-5'

D51_TPM.filter 19348773_21 TAAGAAGATCATGAATTTTGC

target id: Solyc05g008070.2.1

1394 5'-AACTTGCAAAATTCATGATCTTCTTAGGCAG-3' 1424 cleavage site: 1409

|||||||||||||||||||||

3'-CGTTTTAAGTACTAGAAGAAT-5'

D51_TPM.filter 04746133_24 AGGACAACGATCTTTTCTGAATAG

target id: Solyc05g008070.2.1

949 5'-AAAAGCTATTCAGAAAAGATCGTTGTCCTCCACA-3' 982 cleavage site: 967

||||||||||||||||||||||||

3'-GATAAGTCTTTTCTAGCAACAGGA-5'

D51_TPM.filter 06186245_21 ACAACGATCTTCTCTGAATAG

target id: Solyc05g008070.2.1

949 5'-AAAAGCTATTCAGAAAAGATCGTTGTCCTCC-3' 979 cleavage site: 964

||||||||| |||||||||||

3'-GATAAGTCTCTTCTAGCAACA-5'

D51_TPM.filter 06186470_21 ACAACGATCTTTTCTGAGTAG

target id: Solyc05g008070.2.1

949 5'-AAAAGCTATTCAGAAAAGATCGTTGTCCTCC-3' 979 cleavage site: 964

|||o|||||||||||||||||

3'-GATGAGTCTTTTCTAGCAACA-5'

D51_TPM.filter 26654357_22 TTTTAGCAAGAGTTGTTTTACA

target id: Solyc05g008070.2.1

538 5'-GTATAGGTAAGACAACTCTTGCTAGAAAAGTT-3' 569 cleavage site: 554

||||o|||||||||||||o||

3'-ACATTTTGTTGAGAACGATTTT-5'

D51_TPM.filter 21792344_21 TGCCTTAATGAACTGCTCAGT

target id: Solyc05g008070.2.1

1264 5'-GGATAGCTGAGCAGTTCATTAAGGCAACAAG-3' 1294 cleavage site: 1279

o||||||||||||||||||||

3'-TGACTCGTCAAGTAATTCCGT-5'

D51_TPM.filter 06186540_20 ACAACGATCTTTTCTGAATA

target id: Solyc05g008070.2.1

950 5'-AAAGCTATTCAGAAAAGATCGTTGTCCTCC-3' 979 cleavage site: 964

||||||||||||||||||||

3'-ATAAGTCTTTTCTAGCAACA-5'

D51_TPM.filter 25866253_21 TTTAGCAAGAGTTGTTTTACA

target id: Solyc05g008070.2.1

538 5'-GTATAGGTAAGACAACTCTTGCTAGAAAAGT-3' 568 cleavage site: 553

||||o|||||||||||||o|

3'-ACATTTTGTTGAGAACGATTT-5'

D51_TPM.filter 06623130_21 ACGACGATCTTTTCTGAATAG

target id: Solyc05g008070.2.1

949 5'-AAAAGCTATTCAGAAAAGATCGTTGTCCTCC-3' 979 cleavage site: 964

||||||||||||||||||o||

3'-GATAAGTCTTTTCTAGCAGCA-5'

D51_TPM.filter 25866391_22 TTTAGCAAGAGTTGTTTTACCT

target id: Solyc05g008070.2.1

537 5'-GGTATAGGTAAGACAACTCTTGCTAGAAAAGT-3' 568 cleavage site: 553

||||||o|||||||||||||o|

3'-TCCATTTTGTTGAGAACGATTT-5'

D51_TPM.filter 18929669_21 CTTGTTGCCTTAATGAACTGC

target id: Solyc05g008070.2.1

1269 5'-GCTGAGCAGTTCATTAAGGCAACAAGCGATA-3' 1299 cleavage site: 1284

|||||||||||||||||||||

3'-CGTCAAGTAATTCCGTTGTTC-5'

D51_TPM.filter 19521143_21 TAACCATCAATTCATCATTGT

target id: Solyc05g008070.2.1

695 5'-AATGAGCAATGATGAATTGATGGTTATTGTG-3' 725 cleavage site: 710

o||||||||||||||||||||

3'-TGTTACTACTTAACTACCAAT-5'

D51_TPM.filter 22612283_20 TCATTTCTTGATTGTTGACT

target id: Solyc05g008070.2.1

675 5'-AGTGGAGTCAACAATCAAGAAATGAGCAAT-3' 704 cleavage site: 689

||||||||||||||||||||

3'-TCAGTTGTTAGTTCTTTACT-5'

D51_TPM.filter 23985396_21 TTAGAAAAGACACCTCGCGAG

target id: Solyc05g008070.2.1

893 5'-TCCTCCTCGCGAGGTGTCTTTTCTAAGTTTT-3' 923 cleavage site: 908

|||||||||||||||||||||

3'-GAGCGCTCCACAGAAAAGATT-5'

D51_TPM.filter 26653543_24 TTTTAGCAAGAGTTGTTTTACTTT

target id: Solyc05g008070.2.1

536 5'-CGGTATAGGTAAGACAACTCTTGCTAGAAAAGTT-3' 569 cleavage site: 554

|o||||o|||||||||||||o||

3'-TTTCATTTTGTTGAGAACGATTTT-5'

D51_TPM.filter 25866686_23 TTTAGCAAGAGTTGTTTTACTTT

target id: Solyc05g008070.2.1

536 5'-CGGTATAGGTAAGACAACTCTTGCTAGAAAAGT-3' 568 cleavage site: 553

|o||||o|||||||||||||o|

3'-TTTCATTTTGTTGAGAACGATTT-5'

D51_TPM.filter 24963894_22 TTGCCTTAATGAACTGCTCAGT

target id: Solyc05g008070.2.1

1264 5'-GGATAGCTGAGCAGTTCATTAAGGCAACAAGC-3' 1295 cleavage site: 1280

o|||||||||||||||||||||

3'-TGACTCGTCAAGTAATTCCGTT-5'

D51_TPM.filter 06186440_22 ACAACGATCTTTTCTGAATAGC

target id: Solyc05g008070.2.1

948 5'-AAAAAGCTATTCAGAAAAGATCGTTGTCCTCC-3' 979 cleavage site: 964

||||||||||||||||||||||

3'-CGATAAGTCTTTTCTAGCAACA-5'

D51_TPM.filter 08383950_24 ATACGTCGACGTTTCTATCTTGCT

target id: Solyc05g008070.2.1

1057 5'-TTCTTAGCAAGAT--AGACG-TGACGTATGATAA-3' 1087 cleavage site: 1072

|||||||| |o||| o|||||||

3'-TCGTTCTATCTTTGCAGCTGCATA-5'

D51_TPM.filter 26362070_21 TTTCAGGTACTAGATAAGATC

target id: Solyc05g008070.2.1

1616 5'-TGGTAGATCTTATCTAGTACCTGAAATGAAC-3' 1646 cleavage site: 1631

|||||||||||||||||||||

3'-CTAGAATAGATCATGGACTTT-5'

D51_TPM.filter 23651578_21 TCTTGTGAGTAAGTAGAAACA

target id: Solyc05g008070.2.1

1476 5'-GATCTTGTTTCTACTTACTCACAAGACATAA-3' 1506 cleavage site: 1491

|||||||||||||||||||||

3'-ACAAAGATGAATGAGTGTTCT-5'

D51_TPM.filter 09051143_20 ATGACGATTAGAAATCTCCT

target id: Solyc05g008070.2.1

739 5'-AGGGTAGGAGATTTCTAATCGTCATAGATG-3' 768 cleavage site: 753

||||||||||||||||||||

3'-TCCTCTAAAGATTAGCAGTA-5'

D51_TPM.filter 06186423_21 ACAACGATCTTTTCTGGATAG

target id: Solyc05g008070.2.1

949 5'-AAAAGCTATTCAGAAAAGATCGTTGTCCTCC-3' 979 cleavage site: 964

||||o||||||||||||||||

3'-GATAGGTCTTTTCTAGCAACA-5'

D51_TPM.filter 06673968_21 ACGATCTTTTCTGAATAGCTG

target id: Solyc05g008070.2.1

946 5'-CCAAAAAGCTATTCAGAAAAGATCGTTGTCC-3' 976 cleavage site: 961

||||||||||||||||||||

3'-GTCGATAAGTCTTTTCTAGCA-5'

D51_TPM.filter 21054411_21 TGACGATTAGAAATCTCCTAT

target id: Solyc05g008070.2.1

737 5'-CAAGGGTAGGAGATTTCTAATCGTCATAGAT-3' 767 cleavage site: 752

o||||||||||||||||||||

3'-TATCCTCTAAAGATTAGCAGT-5'

D51_TPM.filter 22144770_22 TGTTGATCTTCCATACATTGAA

target id: Solyc05g008070.2.1

1908 5'-AATCCTTTTA-GTATTGGAGATCAACAAACTC-3' 1938 cleavage site: 1923

||o | |||| |o|||||||||

3'-AAGTTACATACCTTCTAGTTGT-5'

D51_TPM.filter 06674072_21 ACGATCTTTTCTGAATAGCCT

target id: Solyc05g008070.2.1

946 5'-CCAAAAAGCTATTCAGAAAAGATCGTTGTCC-3' 976 cleavage site: 961

|||||||||||||||||||

3'-TCCGATAAGTCTTTTCTAGCA-5'

D51_TPM.filter 19521300_21 TAACCATCAATTCATCATTGC

target id: Solyc05g008070.2.1

695 5'-AATGAGCAATGATGAATTGATGGTTATTGTG-3' 725 cleavage site: 710

|||||||||||||||||||||

3'-CGTTACTACTTAACTACCAAT-5'

D51_TPM.filter 11529788_21 ATTTCTTGATTGTTGACTCCA

target id: Solyc05g008070.2.1

672 5'-ATTAGTGGAGTCAACAATCAAGAAATGAGCA-3' 702 cleavage site: 687

|||||||||||||||||||||

3'-ACCTCAGTTGTTAGTTCTTTA-5'

D51_TPM.filter 15702362_22 CAACGATCTTTTCTGAATAGCT

target id: Solyc05g008070.2.1

947 5'-CAAAAAGCTATTCAGAAAAGATCGTTGTCCTC-3' 978 cleavage site: 963

||||||||||||||||||||||

3'-TCGATAAGTCTTTTCTAGCAAC-5'

D51_TPM.filter 12402716_24 GACAACGATCTTTTCTGAATAGCT

target id: Solyc05g008070.2.1

947 5'-CAAAAAGCTATTCAGAAAAGATCGTTGTCCTCCA-3' 980 cleavage site: 965

||||||||||||||||||||||||

3'-TCGATAAGTCTTTTCTAGCAACAG-5'

D51_TPM.filter 06186298_21 ACAACGATCTTTTCTGAATAT

target id: Solyc05g008070.2.1

949 5'-AAAAGCTATTCAGAAAAGATCGTTGTCCTCC-3' 979 cleavage site: 964

||||||||||||||||||||

3'-TATAAGTCTTTTCTAGCAACA-5'

D51_TPM.filter 22047720_21 TGTGTCTATTTGTTCAACTTC

target id: Solyc05g008070.2.1

1879 5'-ATGTTGATA-TTGAACAAATGGATATGCCTAA-3' 1909 cleavage site: 1894

|| | ||||||||||o||o|oo

3'-CT-TCAACTTGTTTATCTGTGT-5'

D51_TPM.filter 06186518_24 ACAACGATCTTTTCTGAATAGCTT

target id: Solyc05g008070.2.1

946 5'-CCAAAAAGCTATTCAGAAAAGATCGTTGTCCTCC-3' 979 cleavage site: 964

||||||||||||||||||||||||

3'-TTCGATAAGTCTTTTCTAGCAACA-5'

D51_TPM.filter 06187126_23 ACAACGATCTTTTCTGAATAGCT

target id: Solyc05g008070.2.1

947 5'-CAAAAAGCTATTCAGAAAAGATCGTTGTCCTCC-3' 979 cleavage site: 964

|||||||||||||||||||||||

3'-TCGATAAGTCTTTTCTAGCAACA-5'

D51_TPM.filter 23400199_21 TCTGATGCTGTTATACTTAGC

target id: Solyc05g008070.2.1

2106 5'-GAAAAGCTAAGTATAACAGCATCAGATGACA-3' 2136 cleavage site: 2121

|||||||||||||||||||||

3'-CGATTCATATTGTCGTAGTCT-5'

D51_TPM.filter 22191575_21 TGTTTGAGACGAGTGGTTAGT

target id: Solyc05g008070.2.1

837 5'-ATTATACTAACCACTCGTCTCAAACATGTTG-3' 867 cleavage site: 852

|||||||||||||||||||||

3'-TGATTGGTGAGCAGAGTTTGT-5'

D51_TPM.filter 24451788_21 TTATTTCTTGATTGTTGACTC

target id: Solyc05g008070.2.1

674 5'-TAGTGGAGTCAACAATCAAGAAATGAGCAAT-3' 704 cleavage site: 689

|||||||||||||||||||o|

3'-CTCAGTTGTTAGTTCTTTATT-5'

D51_TPM.filter 06186408_21 ACAACGATCTTTTCTAAATAG

target id: Solyc05g008070.2.1

949 5'-AAAAGCTATTCAGAAAAGATCGTTGTCCTCC-3' 979 cleavage site: 964

||||| |||||||||||||||

3'-GATAAATCTTTTCTAGCAACA-5'

D51_TPM.filter 14996699_20 GTGTCTATTTGTTCAACTTT

target id: Solyc05g008070.2.1

1879 5'-ATGTTGATA-TTGAACAAATGGATATGCCTA-3' 1908 cleavage site: 1893

o| | ||||||||||o||o|o

3'-TT-TCAACTTGTTTATCTGTG-5'

D51_TPM.filter 25269398_21 TTCAGGTACTAGATAAGATCT

target id: Solyc05g008070.2.1

1615 5'-GTGGTAGATCTTATCTAGTACCTGAAATGAA-3' 1645 cleavage site: 1630

|||||||||||||||||||||

3'-TCTAGAATAGATCATGGACTT-5'

D51_TPM.filter 12219929_21 GAGGACAACGATCTTTTCTGA

target id: Solyc05g008070.2.1

953 5'-GCTATTCAGAAAAGATCGTTGTCCTCCACAA-3' 983 cleavage site: 968

|||||||||||||||||||||

3'-AGTCTTTTCTAGCAACAGGAG-5'

D51_TPM.filter 22307430_21 TCAACGATCTTTTCTGAATAG

target id: Solyc05g008070.2.1

949 5'-AAAAGCTATTCAGAAAAGATCGTTGTCCTCC-3' 979 cleavage site: 964

||||||||||||||||||||

3'-GATAAGTCTTTTCTAGCAACT-5'

D51_TPM.filter 25866557_21 TTTAGCAAGAGTTGTTTTACG

target id: Solyc05g008070.2.1

538 5'-GTATAGGTAAGACAACTCTTGCTAGAAAAGT-3' 568 cleavage site: 553

||||o|||||||||||||o|

3'-GCATTTTGTTGAGAACGATTT-5'

D51_TPM.filter 18573702_22 CTCATTTCTTGATTGTTGACTC

target id: Solyc05g008070.2.1

674 5'-TAGTGGAGTCAACAATCAAGAAATGAGCAATG-3' 705 cleavage site: 690

||||||||||||||||||||||

3'-CTCAGTTGTTAGTTCTTTACTC-5'

D51_TPM.filter 26654347_23 TTTTAGCAAGAGTTGTTTTACCA

target id: Solyc05g008070.2.1

537 5'-GGTATAGGTAAGACAACTCTTGCTAGAAAAGTT-3' 569 cleavage site: 554

|||||o|||||||||||||o||

3'-ACCATTTTGTTGAGAACGATTTT-5'

D51_TPM.filter 22047067_21 TGTGTCTATTTGTTCAATTTT

target id: Solyc05g008070.2.1

1879 5'-ATGTTGATATTGAACAAATGGATATGCCTAA-3' 1909 cleavage site: 1894

o| |||||||||||o||o|oo

3'-TTTTAACTTGTTTATCTGTGT-5'

D51_TPM.filter 25866163_22 TTTAGCAAGAGTTGTTTTACCC

target id: Solyc05g008070.2.1

537 5'-GGTATAGGTAAGACAACTCTTGCTAGAAAAGT-3' 568 cleavage site: 553

|||||o|||||||||||||o|

3'-CCCATTTTGTTGAGAACGATTT-5'

D51_TPM.filter 19059194_22 CTTTAGCAAGAGTTGTTTTACC

target id: Solyc05g008070.2.1

538 5'-GTATAGGTAAGACAACTCTTGCTAGAAAAGTT-3' 569 cleavage site: 554

|||||o|||||||||||||o|

3'-CCATTTTGTTGAGAACGATTTC-5'

D51_TPM.filter 26654102_21 TTTTAGCAAGAGTTGTTTTAC

target id: Solyc05g008070.2.1

539 5'-TATAGGTAAGACAACTCTTGCTAGAAAAGTT-3' 569 cleavage site: 554

||||o|||||||||||||o||

3'-CATTTTGTTGAGAACGATTTT-5'

D51_TPM.filter 22047447_21 TGTGTCTATTTGTTCAACTGT

target id: Solyc05g008070.2.1

1880 5'-TGTTGATA-TTGAACAAATGGATATGCCTAA-3' 1909 cleavage site: 1894

|o| ||||||||||o||o|oo

3'-TGTCAACTTGTTTATCTGTGT-5'

D51_TPM.filter 25374791_21 TTCGATACACGAATCTCCATA

target id: Solyc05g008070.2.1

1224 5'-GAAGATATGGAGATTCGTGTATCGAAGATGA-3' 1254 cleavage site: 1239

|||||||||||||||||||||

3'-ATACCTCTAAGCACATAGCTT-5'

D51_TPM.filter 25866348_22 TTTAGCAAGAGTTGTTTTACCA

target id: Solyc05g008070.2.1

537 5'-GGTATAGGTAAGACAACTCTTGCTAGAAAAGT-3' 568 cleavage site: 553

|||||o|||||||||||||o|

3'-ACCATTTTGTTGAGAACGATTT-5'

D51_TPM.filter 17260698_21 CGTGTCTATTTGTTCAACTTT

target id: Solyc05g008070.2.1

1879 5'-ATGTTGATA-TTGAACAAATGGATATGCCTAA-3' 1909 cleavage site: 1894

o| | ||||||||||o||o|o|

3'-TT-TCAACTTGTTTATCTGTGC-5'

D51_TPM.filter 21054734_21 TGACGATTAGAAATCTCCTAA

target id: Solyc05g008070.2.1

737 5'-CAAGGGTAGGAGATTTCTAATCGTCATAGAT-3' 767 cleavage site: 752

||||||||||||||||||||

3'-AATCCTCTAAAGATTAGCAGT-5'

D51_TPM.filter 24978863_24 TTGCTCATTTCTTGATTGTTGACT

target id: Solyc05g008070.2.1

675 5'-AGTGGAGTCAACAATCAAGAAATGAGCAATGATG-3' 708 cleavage site: 693

||||||||||||||||||||||||

3'-TCAGTTGTTAGTTCTTTACTCGTT-5'

D51_TPM.filter 16681520_21 CGATCTTTTCTGAATAGCTTC

target id: Solyc05g008070.2.1

945 5'-ACCAAAAAGCTATTCAGAAAAGATCGTTGTC-3' 975 cleavage site: 960

||||||||||||||||||||

3'-CTTCGATAAGTCTTTTCTAGC-5'

D51_TPM.filter 19348359_21 TAAGAAGATCATGAATTTTGT

target id: Solyc05g008070.2.1

1394 5'-AACTTGCAAAATTCATGATCTTCTTAGGCAG-3' 1424 cleavage site: 1409

o||||||||||||||||||||

3'-TGTTTTAAGTACTAGAAGAAT-5'

D51_TPM.filter 10598242_22 ATTATATTGTTCACTTATTGGT

target id: Solyc05g008070.2.1

613 5'-GGGTCACC-ATATCTGAACAATATAATAAGAG-3' 643 cleavage site: 628

||| ||| |||||||||||||

3'-TGGTTATTCACTTGTTATATTA-5'

D51_TPM.filter 25866736_20 TTTAGCAAGAGTTGTTTTAC

target id: Solyc05g008070.2.1

539 5'-TATAGGTAAGACAACTCTTGCTAGAAAAGT-3' 568 cleavage site: 553

||||o|||||||||||||o|

3'-CATTTTGTTGAGAACGATTT-5'

D51_TPM.filter 25048327_21 TTGTGAGTAAGTAGAAACAAG

target id: Solyc05g008070.2.1

1474 5'-GTGATCTTGTTTCTACTTACTCACAAGACAT-3' 1504 cleavage site: 1489

|||||||||||||||||||||

3'-GAACAAAGATGAATGAGTGTT-5'

D51_TPM.filter 12220148_22 GAGGACAACGATCTTTTCTGAA

target id: Solyc05g008070.2.1

952 5'-AGCTATTCAGAAAAGATCGTTGTCCTCCACAA-3' 983 cleavage site: 968

||||||||||||||||||||||

3'-AAGTCTTTTCTAGCAACAGGAG-5'

D51_TPM.filter 03562038_22 AATTATATTGTTCACTTATTGG

target id: Solyc05g008070.2.1

614 5'-GGTCACC-ATATCTGAACAATATAATAAGAGA-3' 644 cleavage site: 629

|| ||| |||||||||||||

3'-GGTTATTCACTTGTTATATTAA-5'

D51_TPM.filter 24301904_22 TTATATTGTTCAGATATGGTGA

target id: Solyc05g008070.2.1

611 5'-ATGGGTCACCATATCTGAACAATATAATAAGA-3' 642 cleavage site: 627

||||||||||||||||||||||

3'-AGTGGTATAGACTTGTTATATT-5'

D51_TPM.filter 24978911_21 TTGCTCATTTCTTGATTGTTG

target id: Solyc05g008070.2.1

678 5'-GGAGTCAACAATCAAGAAATGAGCAATGATG-3' 708 cleavage site: 693

|||||||||||||||||||||

3'-GTTGTTAGTTCTTTACTCGTT-5'

D51_TPM.filter 20412523_21 TATGACGATTAGAAATCTCCT

target id: Solyc05g008070.2.1

739 5'-AGGGTAGGAGATTTCTAATCGTCATAGATGA-3' 769 cleavage site: 754

|||||||||||||||||||||

3'-TCCTCTAAAGATTAGCAGTAT-5'

D51_TPM.filter 26653916_22 TTTTAGCAAGAGTTGTTTTACG

target id: Solyc05g008070.2.1

538 5'-GTATAGGTAAGACAACTCTTGCTAGAAAAGTT-3' 569 cleavage site: 554

||||o|||||||||||||o||

3'-GCATTTTGTTGAGAACGATTTT-5'

D51_TPM.filter 26362371_22 TTTCAGGTACTAGATAAGATCT

target id: Solyc05g008070.2.1

1615 5'-GTGGTAGATCTTATCTAGTACCTGAAATGAAC-3' 1646 cleavage site: 1631

||||||||||||||||||||||

3'-TCTAGAATAGATCATGGACTTT-5'

D51_TPM.filter 22612400_21 TCATTTCTTGATTGTTGACGC

target id: Solyc05g008070.2.1

674 5'-TAGTGGAGTCAACAATCAAGAAATGAGCAAT-3' 704 cleavage site: 689

|||||||||||||||||||

3'-CGCAGTTGTTAGTTCTTTACT-5'

D51_TPM.filter 06186628_21 ACAACGATCTTTTCTGAATAC

target id: Solyc05g008070.2.1

949 5'-AAAAGCTATTCAGAAAAGATCGTTGTCCTCC-3' 979 cleavage site: 964

||||||||||||||||||||

3'-CATAAGTCTTTTCTAGCAACA-5'

D51_TPM.filter 26653934_23 TTTTAGCAAGAGTTGTTTTACCC

target id: Solyc05g008070.2.1

537 5'-GGTATAGGTAAGACAACTCTTGCTAGAAAAGTT-3' 569 cleavage site: 554

|||||o|||||||||||||o||

3'-CCCATTTTGTTGAGAACGATTTT-5'

D51_TPM.filter 14979410_22 GTGTGTCTATTTGTTCAACTTC

target id: Solyc05g008070.2.1

1879 5'-ATGTTGATA-TTGAACAAATGGATATGCCTAAT-3' 1910 cleavage site: 1895

|| | ||||||||||o||o|oo|

3'-CT-TCAACTTGTTTATCTGTGTG-5'

D51_TPM.filter 25166580_21 TTGTTGCCTTAATGAACTGCT

target id: Solyc05g008070.2.1

1268 5'-AGCTGAGCAGTTCATTAAGGCAACAAGCGAT-3' 1298 cleavage site: 1283

|||||||||||||||||||||

3'-TCGTCAAGTAATTCCGTTGTT-5'

D51_TPM.filter 05684092_20 AGTGAACTGTAGTTGTGAAA

target id: Solyc04g009240.1.1

2351 5'-ACAGCTTCTGTCAACTACAGTTC-CTTCAAC-3' 2380 cleavage site: 2365

|| oo |||||||||||| ||

3'-AAAGT-GTTGATGTCAAGTGA-5'

D51_TPM.filter 10902796_21 ATTGTGTTGTACTCTTGAGAG

target id: Solyc04g009240.1.1

655 5'-TGTGTTTCTCAAGAGTACAACACAATGGATC-3' 685 cleavage site: 670

o||||||||||||||||||||

3'-GAGAGTTCTCATGTTGTGTTA-5'

D51_TPM.filter 23617531_22 TCTTATGAATTCTAGGTCTTCT

target id: Solyc05g053990.2.1

685 5'-GTGAAACAAGACCTCGAAATCATAAGATCGTT-3' 716 cleavage site: 701

||||||| ||| ||||||||

3'-TCTTCTGGATCTTAAGTATTCT-5'

D51_TPM.filter 23617480_21 TCTTATGAATTCTAGGTCTTC

target id: Solyc05g053990.2.1

686 5'-TGAAACAAGACCTCGAAATCATAAGATCGTT-3' 716 cleavage site: 701

||||||| ||| ||||||||

3'-CTTCTGGATCTTAAGTATTCT-5'

D51_TPM.filter 23617435_22 TCTTATGAATTCTAGGTCTTCA

target id: Solyc05g053990.2.1

685 5'-GTGAAACAAGACCTCGAAATCATAAGATCGTT-3' 716 cleavage site: 701

||||||| ||| ||||||||

3'-ACTTCTGGATCTTAAGTATTCT-5'

D51_TPM.filter 23617345_22 TCTTATGAATTCTAGGTCTTCC

target id: Solyc05g053990.2.1

685 5'-GTGAAACAAGACCTCGAAATCATAAGATCGTT-3' 716 cleavage site: 701

||||||| ||| ||||||||

3'-CCTTCTGGATCTTAAGTATTCT-5'

D51_TPM.filter 23617392_21 TCTTATGAATTCTAGGTCTTT

target id: Solyc05g053990.2.1

686 5'-TGAAACAAGACCTCGAAATCATAAGATCGTT-3' 716 cleavage site: 701

||||||| ||| ||||||||

3'-TTTCTGGATCTTAAGTATTCT-5'

D51_TPM.filter 08974426_24 ATGAATTTTGAATAATTGAAGTGT

target id: Solyc05g053990.2.1

1890 5'-TTTCGATGATTCTATT-TTCAAAATTCATGATCT-3' 1922 cleavage site: 1907

|oo ||| ||| ||||||||||||

3'-TGTGAAGTTAATAAGTTTTAAGTA-5'

D51_TPM.filter 25776990_20 TTTAAGTATGAGTTTGGACT

target id: Solyc11g006640.1.1

1346 5'-GCCTAAG-TGAAACTCATAC-TGAAAATGT-3' 1373 cleavage site: 1358

|| o |||||||||| |o||

3'-TCAGGTTTGAGTATGAATTT-5'

D51_TPM.filter 20554586_23 TATCCGATCAAAATTAAACTGTT

target id: Solyc11g006640.1.1

1259 5'-TTGATAGAAGTCTAATTTTGA-TGGATAGCTTA-3' 1290 cleavage site: 1275

|o ||| ||||||||| o|||||

3'-TTGTCAAATTAAAACTAGCCTAT-5'

D51_TPM.filter 20554474_24 TATCCGATCAAAATTAAACTGTTT

target id: Solyc11g006640.1.1

1259 5'-TTGATAGA-AGTCTAATTTTGA-TGGATAGCTTA-3' 1290 cleavage site: 1275

|o| ||| ||||||||| o|||||

3'-TTTGTCAAATTAAAACTAGCCTAT-5'

D51_TPM.filter 25374791_21 TTCGATACACGAATCTCCATA

target id: Solyc11g006640.1.1

712 5'-GAACTAATGGAGA-TTGTGTATAGAGGTCTG-3' 741 cleavage site: 726

||||||| |o|||||| ||o

3'-ATACCTCTAAGCACATAGCTT-5'

D51_TPM.filter 24662617_23 TTGATATAGGGACTTCATATTCT

target id: Solyc11g006640.1.1

2097 5'-AATCAAGAA-ATTAAGTCCTAATATCAAGAAAT-3' 2128 cleavage site: 2113

|||| || ||||||o |||||||

3'-TCTTATACTTCAGGGATATAGTT-5'

D51_TPM.filter 22162669_21 TGTTGTCAGTAGATTTCTCGA

target id: Solyc11g006640.1.1

1703 5'-ATAAATTGAGAAATCTACAGACCATAATTCT-3' 1733 cleavage site: 1718

|o||||||||||| ||| |o|

3'-AGCTCTTTAGATGACTGTTGT-5'

D51_TPM.filter 24330061_20 TTATGGTCTTGGATATTTCT

target id: Solyc11g006640.1.1

1704 5'-TAAATTGAGAAATCTACAGACCATAATTCTT-3' 1734 cleavage site: 1719

||o| |||o| |||||||||

3'-TCTTTATAGGT-TCTGGTATT-5'

D51_TPM.filter 20269440_22 TACTTCTTCTATTGTATCTGGT

target id: Solyc04g005550.1.1

3411 5'-AGCCTGCCA-AT-CAATAGAAGAAGTGATCAC-3' 3440 cleavage site: 3425

o||| || |||||||||||||o

3'-TGGTCTATGTTATCTTCTTCAT-5'

D51_TPM.filter 22129026_21 TGTTACTTTGCACTGATGGTT

target id: Solyc04g005550.1.1

836 5'-ACCACAAACATCGGTGCAAAGTGACATTCAC-3' 866 cleavage site: 851

|| ||||o|||||||||o|||

3'-TTGGTAGTCACGTTTCATTGT-5'

D51_TPM.filter 22987009_21 TCCAGCTGCATACTGAGTAGT

target id: Solyc04g005550.1.1

3759 5'-CGATGGCTAC-CAATATGAAGCTGGAGATGT-3' 3788 cleavage site: 3773

o|||| || |||| |||||||

3'-TGATGAGTCATACGTCGACCT-5'

D51_TPM.filter 22405750_24 TCAGGGCACCTATCTAATCTAATT

target id: Solyc04g005550.1.1

3580 5'-AGAGTAGTATGGATTA-ATAGTTGCCCTGAAATGG-3' 3613 cleavage site: 3598

|o| |o||||| |||| ||||||||

3'-TTA-ATCTAATCTATCCACGGGACT-5'

D51_TPM.filter 16707448_21 CGGAAACGCGTTGTGAATGTC

target id: Solyc04g005550.1.1

853 5'-AAAGTGACATTCACAACGCGTTTCCGACATG-3' 883 cleavage site: 868

|||||||||||||||||||||

3'-CTGTAAGTGTTGCGCAAAGGC-5'

D51_TPM.filter 10554185_21 ATTACCGACTTTCTGTCTGAA

target id: Solyc04g005550.1.1

950 5'-ACCTTTTCAGGCAGAAAGCCGGTAACTCAGT-3' 980 cleavage site: 965

|||||o||||||| ||||||

3'-AAGTCTGTCTTTCAGCCATTA-5'

D51_TPM.filter 20738537_22 TATTTCTTCTATTGTATCTGGT

target id: Solyc04g005550.1.1

3411 5'-AGCCTGCCA-AT-CAATAGAAGAAGTGATCAC-3' 3440 cleavage site: 3425

o||| || |||||||||||o|o

3'-TGGTCTATGTTATCTTCTTTAT-5'

D51_TPM.filter 02410133_21 AACGCGTTGTGAATGTCACTT

target id: Solyc04g005550.1.1

849 5'-GTGCAAAGTGACATTCACAACGCGTTTCCGA-3' 879 cleavage site: 864

|||||||||||||||||||||

3'-TTCACTGTAAGTGTTGCGCAA-5'

D51_TPM.filter 03547390_22 AATTACCGGCTTTCTGCCTGAA

target id: Solyc04g005550.1.1

950 5'-ACCTTTTCAGGCAGAAAGCCGGTAACTCAGTT-3' 981 cleavage site: 966

||||||||||||||||||||

3'-AAGTCCGTCTTTCGGCCATTAA-5'

D51_TPM.filter 24207800_21 TTACCGGCTTTCTGCCTGAAA

target id: Solyc04g005550.1.1

949 5'-AACCTTTTCAGGCAGAAAGCCGGTAACTCAG-3' 979 cleavage site: 964

|||||||||||||||||||||

3'-AAAGTCCGTCTTTCGGCCATT-5'

D51_TPM.filter 16707484_21 CGGAAACGCGTTGTGAATGTT

target id: Solyc04g005550.1.1

853 5'-AAAGTGACATTCACAACGCGTTTCCGACATG-3' 883 cleavage site: 868

o||||||||||||||||||||

3'-TTGTAAGTGTTGCGCAAAGGC-5'

D51_TPM.filter 10554422_21 ATTACCGGCTTTCTGCCTGAA

target id: Solyc04g005550.1.1

950 5'-ACCTTTTCAGGCAGAAAGCCGGTAACTCAGT-3' 980 cleavage site: 965

||||||||||||||||||||

3'-AAGTCCGTCTTTCGGCCATTA-5'

D51_TPM.filter 17072469_21 CGCGTTGTCAATGTTACTTTG

target id: Solyc04g005550.1.1

847 5'-CGGTGCAAAGTGACATTCACAACGCGTTTCC-3' 877 cleavage site: 862

||||||o||||| ||||||||

3'-GTTTCATTGTAACTGTTGCGC-5'

D51_TPM.filter 24206278_21 TTACCGACTTTCTGTCTGAAA

target id: Solyc04g005550.1.1

949 5'-AACCTTTTCAGGCAGAAAGCCGGTAACTCAG-3' 979 cleavage site: 964

||||||o||||||| ||||||

3'-AAAGTCTGTCTTTCAGCCATT-5'

D51_TPM.filter 06217752_21 ACAATGATCTTACTTCCTATA

target id: Solyc11g064770.1.1

682 5'-GGAGATTTGGGAAGTAAGATCATTGTAACTA-3' 712 cleavage site: 697

|o|||||||||||||||||

3'-ATATCCTTCATTCTAGTAACA-5'

D51_TPM.filter 10902796_21 ATTGTGTTGTACTCTTGAGAG

target id: Solyc04g009150.1.1

655 5'-TGTGTTTCTCAAGAGTACAACACAATGGATC-3' 685 cleavage site: 670

o||||||||||||||||||||

3'-GAGAGTTCTCATGTTGTGTTA-5'

D51_TPM.filter 10823624_21 ATTGGTGAAATATTCTAGAGT

target id: Solyc09g092310.1.1

2020 5'-GTTGAAC-CGAGATTATTTCACCGGTTGATC-3' 2049 cleavage site: 2034

|| | ||| |||||||||oo|

3'-TGAGATCTTATAAAGTGGTTA-5'

D51_TPM.filter 24787600_21 TTGGATCTTGGAATATTTCTC

target id: Solyc01g086810.2.1

1861 5'-AATGTGA-AAGTACTTCCAAGATCCATTGGAA-3' 1891 cleavage site: 1876

|| ||o|| ||||||||||||

3'-CTCTTTAT-AAGGTTCTAGGTT-5'

D51_TPM.filter 03155661_24 AATGGATCTTGTGAAACTTTCACC

target id: Solyc01g086810.2.1

1858 5'-ACCAATGTGAAAGTACTTC-CAAGATCCATTGGAAG-3' 1892 cleavage site: 1877

|||||||| ||| |||||||||||

3'-CCACTTTCA--AAGTGTTCTAGGTAA-5'

D51_TPM.filter 25527754_21 TTCCGTTCTTTTAGTCCTTTG

target id: Solyc01g086810.2.1

1398 5'-ATTTGTAAA--ACAAAAAGAACGGAGGACAG-3' 1426 cleavage site: 1411

o||| || |||||||||||o

3'-GTTTCCTGATTTTCTTGCCTT-5'

D51_TPM.filter 08974426_24 ATGAATTTTGAATAATTGAAGTGT

target id: Solyc05g050430.1.1

2382 5'-TTTCGATGATTCTATT-TTCAAAATTCATGATCT-3' 2414 cleavage site: 2399

|oo ||| ||| ||||||||||||

3'-TGTGAAGTTAATAAGTTTTAAGTA-5'

D51_TPM.filter 20685825_20 TATTCCATGAGACTGTTTTT

target id: Solyc12g094660.1.1

2779 5'-AGGAAAAAAACAAGTCTCAGGGAATTGATCA-3' 2809 cleavage site: 2794

|||||| ||||||| |||||

3'-TTTTTG-TCAGAGTACCTTAT-5'

D51_TPM.filter 20686125_21 TATTCCATGAGACTGTTTTTG

target id: Solyc12g094660.1.1

2778 5'-GAGGAAAAAAACAAGTCTCAGGGAATTGATCA-3' 2809 cleavage site: 2794

|||||| ||||||| |||||

3'-GTTTTTG-TCAGAGTACCTTAT-5'

D51_TPM.filter 25488995_21 TTCCATGAGACTGTTTTTGTT

target id: Solyc12g094660.1.1

2776 5'-CTGAGGAAAAAAACAAGTCTCA-GGGAATTGA-3' 2806 cleavage site: 2791

o| |||||| ||||||| ||o|

3'-TTGTTTTTG-TCAGAGTACCTT-5'

D51_TPM.filter 25489540_21 TTCCATGAGACTGTTTTTTTT

target id: Solyc12g094660.1.1

2776 5'-CTGAGGAAAAAAACAAGTCTCA-GGGAATTGA-3' 2806 cleavage site: 2791

o|||||||| ||||||| ||o|

3'-TTTTTTTTG-TCAGAGTACCTT-5'

D51_TPM.filter 20686002_23 TATTCCATGAGACTGTTTTTTTT

target id: Solyc12g094660.1.1

2776 5'-CTGAGGAAAAAAACAAGTCTCAGGGAATTGATCA-3' 2809 cleavage site: 2794

o|||||||| ||||||| |||||

3'-TTTTTTTTG-TCAGAGTACCTTAT-5'

D51_TPM.filter 20686090_21 TATTCCATGAGACTGTTTTTT

target id: Solyc12g094660.1.1

2778 5'-GAGGAAAAAAACAAGTCTCAGGGAATTGATCA-3' 2809 cleavage site: 2794

||||||| ||||||| |||||

3'-TTTTTTG-TCAGAGTACCTTAT-5'

D51_TPM.filter 20685962_22 TATTCCATGAGACTGTTTTTTT

target id: Solyc12g094660.1.1

2777 5'-TGAGGAAAAAAACAAGTCTCAGGGAATTGATCA-3' 2809 cleavage site: 2794

|||||||| ||||||| |||||

3'-TTTTTTTG-TCAGAGTACCTTAT-5'

D51_TPM.filter 25490119_20 TTCCATGAGACTGTTTTTTT

target id: Solyc12g094660.1.1

2777 5'-TGAGGAAAAAAACAAGTCTCA-GGGAATTGA-3' 2806 cleavage site: 2791

|||||||| ||||||| ||o|

3'-TTTTTTTG-TCAGAGTACCTT-5'

D51_TPM.filter 25490165_22 TTCCATGAGACTGTTTTTTTTT

target id: Solyc12g094660.1.1

2775 5'-ACTGAGGAAAAAAACAAGTCTCA-GGGAATTGA-3' 2806 cleavage site: 2791

oo|||||||| ||||||| ||o|

3'-TTTTTTTTTG-TCAGAGTACCTT-5'

D51_TPM.filter 25489031_22 TTCCATGAGACTGTTTTTGTTC

target id: Solyc12g094660.1.1

2775 5'-ACTGAGGAAAAAAACAAGTCTCA-GGGAATTGA-3' 2806 cleavage site: 2791

|o| |||||| ||||||| ||o|

3'-CTTGTTTTTG-TCAGAGTACCTT-5'

D51_TPM.filter 20686722_21 TATTCCATGAGACTGTTTTTC

target id: Solyc12g094660.1.1

2778 5'-GAGGAAAAAAACAAGTCTCAGGGAATTGATCA-3' 2809 cleavage site: 2794

|||||| ||||||| |||||

3'-CTTTTTG-TCAGAGTACCTTAT-5'

D51_TPM.filter 20686987_23 TATTCCATGAGACTGTTTTTTTC

target id: Solyc12g094660.1.1

2776 5'-CTGAGGAAAAAAACAAGTCTCAGGGAATTGATCA-3' 2809 cleavage site: 2794

||||||||| ||||||| |||||

3'-CTTTTTTTG-TCAGAGTACCTTAT-5'

D51_TPM.filter 25489643_21 TTCCATGAGACTGTTTTTTTC

target id: Solyc12g094660.1.1

2776 5'-CTGAGGAAAAAAACAAGTCTCA-GGGAATTGA-3' 2806 cleavage site: 2791

||||||||| ||||||| ||o|

3'-CTTTTTTTG-TCAGAGTACCTT-5'

D51_TPM.filter 20686860_24 TATTCCATGAGACTGTTTTTTTTT

target id: Solyc12g094660.1.1

2775 5'-ACTGAGGAAAAAAACAAGTCTCAGGGAATTGATCA-3' 2809 cleavage site: 2794

oo|||||||| ||||||| |||||

3'-TTTTTTTTTG-TCAGAGTACCTTAT-5'

D51_TPM.filter 20685760_21 TATTCCATGAGACTGTTTTTA

target id: Solyc12g094660.1.1

2778 5'-GAGGAAAAAAACAAGTCTCAGGGAATTGATCA-3' 2809 cleavage site: 2794

|||||| ||||||| |||||

3'-ATTTTTG-TCAGAGTACCTTAT-5'

D51_TPM.filter 25489283_23 TTCCATGAGACTGTTTTTTTTTT

target id: Solyc12g094660.1.1

2774 5'-TACTGAGGAAAAAAACAAGTCTCA-GGGAATTGA-3' 2806 cleavage site: 2791

|oo|||||||| ||||||| ||o|

3'-TTTTTTTTTTG-TCAGAGTACCTT-5'

D51_TPM.filter 25490151_21 TTCCATGAGACTGTTTTTTCT

target id: Solyc12g094660.1.1

2775 5'-ACTGAGGAAAAAAACAAGTCTCA-GGGAATTGA-3' 2806 cleavage site: 2791

o| ||||||| ||||||| ||o|

3'-TC-TTTTTTG-TCAGAGTACCTT-5'

D51_TPM.filter 25490326_20 TTCCATGAGACTGTTTTTTC

target id: Solyc12g094660.1.1

2777 5'-TGAGGAAAAAAACAAGTCTCA-GGGAATTGA-3' 2806 cleavage site: 2791

||||||| ||||||| ||o|

3'-CTTTTTTG-TCAGAGTACCTT-5'

D51_TPM.filter 25490580_21 TTCCATGAGACTGTTTTTGTC

target id: Solyc12g094660.1.1

2776 5'-CTGAGGAAAAAAACAAGTCTCA-GGGAATTGA-3' 2806 cleavage site: 2791

|| |||||| ||||||| ||o|

3'-CTGTTTTTG-TCAGAGTACCTT-5'

D51_TPM.filter 25490156_22 TTCCATGAGACTGTTTTTTTTC

target id: Solyc12g094660.1.1

2775 5'-ACTGAGGAAAAAAACAAGTCTCA-GGGAATTGA-3' 2806 cleavage site: 2791

|o|||||||| ||||||| ||o|

3'-CTTTTTTTTG-TCAGAGTACCTT-5'

D51_TPM.filter 20685809_22 TATTCCATGAGACTGTTTTTTG

target id: Solyc12g094660.1.1

2777 5'-TGAGGAAAAAAACAAGTCTCAGGGAATTGATCA-3' 2809 cleavage site: 2794

||||||| ||||||| |||||

3'-GTTTTTTG-TCAGAGTACCTTAT-5'

D51_TPM.filter 20686690_22 TATTCCATGAGACTGTTTTTTC

target id: Solyc12g094660.1.1

2777 5'-TGAGGAAAAAAACAAGTCTCAGGGAATTGATCA-3' 2809 cleavage site: 2794

||||||| ||||||| |||||

3'-CTTTTTTG-TCAGAGTACCTTAT-5'

D51_TPM.filter 03701049_22 AATTCCATGAGACTGTTTTTGG

target id: Solyc12g094660.1.1

2777 5'-TGAGGAAAAAAACAAGTCTCAGGGAATTGATCA-3' 2809 cleavage site: 2794

|||||| ||||||| ||||||

3'-GGTTTTTG-TCAGAGTACCTTAA-5'

D51_TPM.filter 20685862_24 TATTCCATGAGACTGTTTTTTTTC

target id: Solyc12g094660.1.1

2775 5'-ACTGAGGAAAAAAACAAGTCTCAGGGAATTGATCA-3' 2809 cleavage site: 2794

|o|||||||| ||||||| |||||

3'-CTTTTTTTTG-TCAGAGTACCTTAT-5'

D51_TPM.filter 20686721_23 TATTCCATGAGACTGTTTTTGTC

target id: Solyc12g094660.1.1

2776 5'-CTGAGGAAAAAAACAAGTCTCAGGGAATTGATCA-3' 2809 cleavage site: 2794

|| |||||| ||||||| |||||

3'-CTGTTTTTG-TCAGAGTACCTTAT-5'

D51_TPM.filter 26017789_21 TTTGAAAATGGACCAACAATG

target id: Solyc10g085460.1.1

974 5'-ATGATCATTGTTTGTCCATTTTCAAACAAAG-3' 1004 cleavage site: 989

||||||| |||||||||||||

3'-GTAACAACCAGGTAAAAGTTT-5'

D51_TPM.filter 26323898_21 TTTGTTTGAAAATGGACCAAC

target id: Solyc10g085460.1.1

978 5'-TCATTGTTTGTCCATTTTCAAACAAAGAGCA-3' 1008 cleavage site: 993

||| |||||||||||||||||

3'-CAACCAGGTAAAAGTTTGTTT-5'

D51_TPM.filter 20771087_21 TGAAAATGGACCAACAATGAT

target id: Solyc10g085460.1.1

972 5'-AGATGATCATTGTTTGTCCATTTTCAAACAA-3' 1002 cleavage site: 987

||||||||| |||||||||||

3'-TAGTAACAACCAGGTAAAAGT-5'

D51_TPM.filter 00758108_24 AAAGTATCGAACTGGAGTAAGATT

target id: Solyc10g085460.1.1

1523 5'-GTGAAAATCTTTCTCAAGTTCGATACTTTGGATG-3' 1556 cleavage site: 1541

|||||| ||| |||||||||||||

3'-TTAGAATGAGGTCAAGCTATGAAA-5'

D51_TPM.filter 04526962_21 AGATGGACTTGAGAAAGGGTT

target id: Solyc10g085460.1.1

1522 5'-GGTGAAAATCTTTCTCAAGTTCGATACTTTGGA-3' 1554 cleavage site: 1539

|| o|||||||||||o| || ||

3'-TTGGGAAAGAGTTCAGG-TA-GA-5'

D51_TPM.filter 26324645_21 TTTGTTTGAAAATGGACCAAT

target id: Solyc10g085460.1.1

978 5'-TCATTGTTTGTCCATTTTCAAACAAAGAGCA-3' 1008 cleavage site: 993

o|| |||||||||||||||||

3'-TAACCAGGTAAAAGTTTGTTT-5'

D51_TPM.filter 24468620_21 TTGAAAATGGACCAACAATGA

target id: Solyc10g085460.1.1

973 5'-GATGATCATTGTTTGTCCATTTTCAAACAAA-3' 1003 cleavage site: 988

|||||||| ||||||||||||

3'-AGTAACAACCAGGTAAAAGTT-5'

D51_TPM.filter 02000669_24 AAGTATCGAACTGGAGTAAGATTC

target id: Solyc10g085460.1.1

1522 5'-GGTGAAAATCTTTCTCAAGTTCGATACTTTGGAT-3' 1555 cleavage site: 1540

|||||| ||| ||||||||||||

3'-CTTAGAATGAGGTCAAGCTATGAA-5'

D51_TPM.filter 01962304_24 AAGTAACTGTTCAACGAAGCAATT

target id: Solyc10g085460.1.1

3475 5'-GGCAACCTTGCTTCTTTGGAACAGTTACTTATTTT-3' 3509 cleavage site: 3494

||||||| || ||||||||||||

3'-TTAACGAAGCAA-CTTGTCAATGAA-5'

D51_TPM.filter 08685594_24 ATATGTCATCTTTTGAATTTCAAG

target id: Solyc04g015210.2.1

2368 5'-CCACCCTTGAGA--TAAAAGAGGACATATGGAAA-3' 2399 cleavage site: 2384

|||||o| o|||||| |||||||

3'-GAACTTTAAGTTTTCTACTGTATA-5'

D51_TPM.filter 18756713_21 CTTAAGGATGTTAGTCTAGGC

target id: Solyc11g069620.1.1

1705 5'-ATACTGCCAAGACTGACATCCTTAAGGGCAT-3' 1735 cleavage site: 1720

||| |||||o|||||||||||

3'-CGGATCTGATTGTAGGAATTC-5'

D51_TPM.filter 06217752_21 ACAATGATCTTACTTCCTATA

target id: Solyc11g069620.1.1

922 5'-GGAGGTATGGGAAGTAAGATCATTGTAACTA-3' 952 cleavage site: 937

|||o|||||||||||||||||

3'-ATATCCTTCATTCTAGTAACA-5'

D51_TPM.filter 24156334_21 TTACAATGATCTTACTTCCTA

target id: Solyc11g069620.1.1

924 5'-AGGTATGGGAAGTAAGATCATTGTAACTACA-3' 954 cleavage site: 939

|o|||||||||||||||||||

3'-ATCCTTCATTCTAGTAACATT-5'

D51_TPM.filter 23852157_21 TTAAGGATGTTAGTCTAGGCA

target id: Solyc11g069620.1.1

1704 5'-CATACTGCCAAGACTGACATCCTTAAGGGCA-3' 1734 cleavage site: 1719

|||| |||||o||||||||||

3'-ACGGATCTGATTGTAGGAATT-5'

D51_TPM.filter 23852925_20 TTAAGGATGTTAGTCTAGGC

target id: Solyc11g069620.1.1

1705 5'-ATACTGCCAAGACTGACATCCTTAAGGGCA-3' 1734 cleavage site: 1719

||| |||||o||||||||||

3'-CGGATCTGATTGTAGGAATT-5'

D51_TPM.filter 18756878_21 CTTAAGGATGTTAGTCTAGGT

target id: Solyc11g069620.1.1

1705 5'-ATACTGCCAAGACTGACATCCTTAAGGGCAT-3' 1735 cleavage site: 1720

o|| |||||o|||||||||||

3'-TGGATCTGATTGTAGGAATTC-5'

D51_TPM.filter 21734213_21 TGCGGGAAGATCATTGTAGCT

target id: Solyc11g069620.1.1

1250 5'-TGTTGAGCTACAATGATCTTCCTGCACAATT-3' 1280 cleavage site: 1265

|||||||||||||||||o|||

3'-TCGATGTTACTAGAAGGGCGT-5'

D51_TPM.filter 21688701_21 TGCAGGAAGATCATTGTAGCT

target id: Solyc11g069620.1.1

1250 5'-TGTTGAGCTACAATGATCTTCCTGCACAATT-3' 1280 cleavage site: 1265

|||||||||||||||||||||

3'-TCGATGTTACTAGAAGGACGT-5'

D51_TPM.filter 20044818_21 TACAATGATCTTACTTCCTAT

target id: Solyc11g069620.1.1

923 5'-GAGGTATGGGAAGTAAGATCATTGTAACTAC-3' 953 cleavage site: 938

||o||||||||||||||||||

3'-TATCCTTCATTCTAGTAACAT-5'

D51_TPM.filter 01607288_21 AAGATCATTGTAGCTCAACAT

target id: Solyc11g069620.1.1

1244 5'-CATTGATGTTGAGCTACAATGATCTTCCTGC-3' 1274 cleavage site: 1259

|||||||||||||||||||||

3'-TACAACTCGATGTTACTAGAA-5'

D51_TPM.filter 01607370_21 AAGATCATCGTAGCTCAACAT

target id: Solyc11g069620.1.1

1244 5'-CATTGATGTTGAGCTACAATGATCTTCCTGC-3' 1274 cleavage site: 1259

|||||||||||| ||||||||

3'-TACAACTCGATGCTACTAGAA-5'

D51_TPM.filter 24156686_22 TTACAATGATCTTACTTCCTAT

target id: Solyc11g069620.1.1

923 5'-GAGGTATGGGAAGTAAGATCATTGTAACTACA-3' 954 cleavage site: 939

||o|||||||||||||||||||

3'-TATCCTTCATTCTAGTAACATT-5'

D51_TPM.filter 02685629_24 AACTTATCGATTGATGTCAACTGC

target id: Solyc05g012910.2.1

3992 5'-GTGCTGCA-TTGACATCAGTCGATAAGCTGAAGC-3' 4024 cleavage site: 4009

||| |||||||||o||||||||

3'-CGTCAACTGTAGTTAGCTATTCAA-5'

D51_TPM.filter 07559125_24 ACTTATCGATTGATGTCAACTGCT

target id: Solyc05g012910.2.1

3991 5'-GGTGCTGCA-TTGACATCAGTCGATAAGCTGAAG-3' 4023 cleavage site: 4008

||| |||||||||o||||||||

3'-TCGTCAACTGTAGTTAGCTATTCA-5'

D51_TPM.filter 23251003_21 TCCTTGACATTGTTGTATGAT

target id: Solyc04g012010.2.1

1024 5'-AGGAAATAGTAGAAAAATGTCAAGGACTACC-3' 1054 cleavage site: 1039

|| o|| || |||||||||||

3'-TAGTATGTTGTTACAGTTCCT-5'

D51_TPM.filter 10365346_24 ATCTTTTTTGACTGTGTATAAATA

target id: Solyc07g005770.2.1

2571 5'-TAAGTTACTTGTACATAGTCAAAGAAGACATAAC-3' 2604 cleavage site: 2589

|| ||o||||o|||||||o||||

3'-ATAAATATGTGTCAGTTTTTTCTA-5'

D51_TPM.filter 09830638_21 ATCATCCTGTAATTTGAACGG

target id: Solyc12g016220.1.1

1739 5'-AAGAGTCATTCAAGTTACAGGCTGATTTTTC-3' 1769 cleavage site: 1754

o| |||||o||||||| ||||

3'-GGCAAGTTTAATGTCCTACTA-5'

D51_TPM.filter 22578698_21 TCATCCTGTAATTTGAACGGC

target id: Solyc12g016220.1.1

1738 5'-AAAGAGTCATTCAAGTTACAGGCTGATTTTT-3' 1768 cleavage site: 1753

|o| |||||o||||||| |||

3'-CGGCAAGTTTAATGTCCTACT-5'

D51_TPM.filter 23219160_21 TCCTGTAATTTGAACGGCTCT

target id: Solyc12g016220.1.1

1735 5'-GTTAAAGAGTCATTCAAGTTACAGGCTGATT-3' 1765 cleavage site: 1750

||||o| |||||o|||||||

3'-TCTCGGCAAGTTTAATGTCCT-5'

D51_TPM.filter 22578697_21 TCATCCTGTAATTTGAACGGT

target id: Solyc12g016220.1.1

1738 5'-AAAGAGTCATTCAAGTTACAGGCTGATTTTT-3' 1768 cleavage site: 1753

oo| |||||o||||||| |||

3'-TGGCAAGTTTAATGTCCTACT-5'

D51_TPM.filter 20900898_21 TGAATCCTTCGGCTATCCATA

target id: Solyc11g069020.1.1

1305 5'-AAGGTTATGGATTGCTGAAGGATTTATAAAG-3' 1335 cleavage site: 1320

||||||| ||o||||||||o|

3'-ATACCTATCGGCTTCCTAAGT-5'

D51_TPM.filter 25866430_21 TTTAGCAAGAGTTGTTTTACC

target id: Solyc11g069020.1.1

566 5'-GTATAGGTAAAACCACTCTTGCTAAGAGAAT-3' 596 cleavage site: 581

|||||||| |||||||||||o

3'-CCATTTTGTTGAGAACGATTT-5'

D51_TPM.filter 26653861_22 TTTTAGCAAGAGTTGTTTTACC

target id: Solyc11g069020.1.1

566 5'-GTATAGGTAAAACCACTCTTGCTAAGAGAATT-3' 597 cleavage site: 582

|||||||| |||||||||||o|

3'-CCATTTTGTTGAGAACGATTTT-5'

D51_TPM.filter 25866155_21 TTTAGCAAGAGTTGTTTTACT

target id: Solyc11g069020.1.1

566 5'-GTATAGGTAAAACCACTCTTGCTAAGAGAAT-3' 596 cleavage site: 581

o||||||| |||||||||||o

3'-TCATTTTGTTGAGAACGATTT-5'

D51_TPM.filter 26654022_22 TTTTAGCAAGAGTTGTTTTACT

target id: Solyc11g069020.1.1

566 5'-GTATAGGTAAAACCACTCTTGCTAAGAGAATT-3' 597 cleavage site: 582

o||||||| |||||||||||o|

3'-TCATTTTGTTGAGAACGATTTT-5'

D51_TPM.filter 20900987_22 TGAATCCTTCGGCTATCCATAA

target id: Solyc11g069020.1.1

1304 5'-TAAGGTTATGGATTGCTGAAGGATTTATAAAG-3' 1335 cleavage site: 1320

|||||||| ||o||||||||o|

3'-AATACCTATCGGCTTCCTAAGT-5'

D51_TPM.filter 20900999_20 TGAATCCTTCGGCTATCCAT

target id: Solyc11g069020.1.1

1306 5'-AGGTTATGGATTGCTGAAGGATTTATAAAG-3' 1335 cleavage site: 1320

|||||| ||o||||||||o|

3'-TACCTATCGGCTTCCTAAGT-5'

D51_TPM.filter 26653812_23 TTTTAGCAAGAGTTGTTTTACCT

target id: Solyc11g069020.1.1

565 5'-GGTATAGGTAAAACCACTCTTGCTAAGAGAATT-3' 597 cleavage site: 582

||||||||| |||||||||||o|

3'-TCCATTTTGTTGAGAACGATTTT-5'

D51_TPM.filter 26654357_22 TTTTAGCAAGAGTTGTTTTACA

target id: Solyc11g069020.1.1

566 5'-GTATAGGTAAAACCACTCTTGCTAAGAGAATT-3' 597 cleavage site: 582

||||||| |||||||||||o|

3'-ACATTTTGTTGAGAACGATTTT-5'

D51_TPM.filter 25866253_21 TTTAGCAAGAGTTGTTTTACA

target id: Solyc11g069020.1.1

566 5'-GTATAGGTAAAACCACTCTTGCTAAGAGAAT-3' 596 cleavage site: 581

||||||| |||||||||||o

3'-ACATTTTGTTGAGAACGATTT-5'

D51_TPM.filter 25866391_22 TTTAGCAAGAGTTGTTTTACCT

target id: Solyc11g069020.1.1

565 5'-GGTATAGGTAAAACCACTCTTGCTAAGAGAAT-3' 596 cleavage site: 581

||||||||| |||||||||||o

3'-TCCATTTTGTTGAGAACGATTT-5'

D51_TPM.filter 20900927_23 TGAATCCTTCGGCTATCCATAAT

target id: Solyc11g069020.1.1

1303 5'-ATAAGGTTATGGATTGCTGAAGGATTTATAAAG-3' 1335 cleavage site: 1320

o|||||||| ||o||||||||o|

3'-TAATACCTATCGGCTTCCTAAGT-5'

D51_TPM.filter 26653543_24 TTTTAGCAAGAGTTGTTTTACTTT

target id: Solyc11g069020.1.1

564 5'-GGGTATAGGTAAAACCACTCTTGCTAAGAGAATT-3' 597 cleavage site: 582

|o||||||| |||||||||||o|

3'-TTTCATTTTGTTGAGAACGATTTT-5'

D51_TPM.filter 25866686_23 TTTAGCAAGAGTTGTTTTACTTT

target id: Solyc11g069020.1.1

564 5'-GGGTATAGGTAAAACCACTCTTGCTAAGAGAAT-3' 596 cleavage site: 581

|o||||||| |||||||||||o

3'-TTTCATTTTGTTGAGAACGATTT-5'

D51_TPM.filter 20900852_21 TGAATCCTTCGGCTATCCATT

target id: Solyc11g069020.1.1

1305 5'-AAGGTTATGGATTGCTGAAGGATTTATAAAG-3' 1335 cleavage site: 1320

|||||| ||o||||||||o|

3'-TTACCTATCGGCTTCCTAAGT-5'

D51_TPM.filter 20901206_20 TGAATCCTTCGGCTATCCAC

target id: Solyc11g069020.1.1

1306 5'-AGGTTATGGATTGCTGAAGGATTTATAAAG-3' 1335 cleavage site: 1320

||||| ||o||||||||o|

3'-CACCTATCGGCTTCCTAAGT-5'

D51_TPM.filter 25866557_21 TTTAGCAAGAGTTGTTTTACG

target id: Solyc11g069020.1.1

566 5'-GTATAGGTAAAACCACTCTTGCTAAGAGAAT-3' 596 cleavage site: 581

||||||| |||||||||||o

3'-GCATTTTGTTGAGAACGATTT-5'

D51_TPM.filter 26654347_23 TTTTAGCAAGAGTTGTTTTACCA

target id: Solyc11g069020.1.1

565 5'-GGTATAGGTAAAACCACTCTTGCTAAGAGAATT-3' 597 cleavage site: 582

|||||||| |||||||||||o|

3'-ACCATTTTGTTGAGAACGATTTT-5'

D51_TPM.filter 25866163_22 TTTAGCAAGAGTTGTTTTACCC

target id: Solyc11g069020.1.1

565 5'-GGTATAGGTAAAACCACTCTTGCTAAGAGAAT-3' 596 cleavage site: 581

|||||||| |||||||||||o

3'-CCCATTTTGTTGAGAACGATTT-5'

D51_TPM.filter 19059194_22 CTTTAGCAAGAGTTGTTTTACC

target id: Solyc11g069020.1.1

566 5'-GTATAGGTAAAACCACTCTTGCTAAGAGAATT-3' 597 cleavage site: 582

|||||||| |||||||||||o

3'-CCATTTTGTTGAGAACGATTTC-5'

D51_TPM.filter 26654102_21 TTTTAGCAAGAGTTGTTTTAC

target id: Solyc11g069020.1.1

567 5'-TATAGGTAAAACCACTCTTGCTAAGAGAATT-3' 597 cleavage site: 582

||||||| |||||||||||o|

3'-CATTTTGTTGAGAACGATTTT-5'

D51_TPM.filter 25866348_22 TTTAGCAAGAGTTGTTTTACCA

target id: Solyc11g069020.1.1

565 5'-GGTATAGGTAAAACCACTCTTGCTAAGAGAAT-3' 596 cleavage site: 581

|||||||| |||||||||||o

3'-ACCATTTTGTTGAGAACGATTT-5'

D51_TPM.filter 20900824_21 TGAATCCTTCGGCTATCCATG

target id: Solyc11g069020.1.1

1305 5'-AAGGTTATGGATTGCTGAAGGATTTATAAAG-3' 1335 cleavage site: 1320

o|||||| ||o||||||||o|

3'-GTACCTATCGGCTTCCTAAGT-5'

D51_TPM.filter 20901072_21 TGAATCCTTCGGCTATCCACA

target id: Solyc11g069020.1.1

1305 5'-AAGGTTATGGATTGCTGAAGGATTTATAAAG-3' 1335 cleavage site: 1320

||||| ||o||||||||o|

3'-ACACCTATCGGCTTCCTAAGT-5'

D51_TPM.filter 11370259_24 ATTTGATCTTGTGAATATTCTATA

target id: Solyc11g069020.1.1

2588 5'-GACTCGCTAGAATGATTCAACAAGATCAAATTGACT-3' 2623 cleavage site: 2608

|||||| |||| ||||||||||||

3'-ATATCTTA-TAAG-TGTTCTAGTTTA-5'

D51_TPM.filter 25866736_20 TTTAGCAAGAGTTGTTTTAC

target id: Solyc11g069020.1.1

567 5'-TATAGGTAAAACCACTCTTGCTAAGAGAAT-3' 596 cleavage site: 581

||||||| |||||||||||o

3'-CATTTTGTTGAGAACGATTT-5'

D51_TPM.filter 26653916_22 TTTTAGCAAGAGTTGTTTTACG

target id: Solyc11g069020.1.1

566 5'-GTATAGGTAAAACCACTCTTGCTAAGAGAATT-3' 597 cleavage site: 582

||||||| |||||||||||o|

3'-GCATTTTGTTGAGAACGATTTT-5'

D51_TPM.filter 02562802_22 AACTAGATCTTGTGGAATCATA

target id: Solyc11g069020.1.1

2593 5'-GCTAGAATGATTCAACAAGATCAAATTGACTC-3' 2624 cleavage site: 2609

||||||| |||||||| | ||

3'-ATACTAAGGTGTTCTAGATCAA-5'

D51_TPM.filter 26653934_23 TTTTAGCAAGAGTTGTTTTACCC

target id: Solyc11g069020.1.1

565 5'-GGTATAGGTAAAACCACTCTTGCTAAGAGAATT-3' 597 cleavage site: 582

|||||||| |||||||||||o|

3'-CCCATTTTGTTGAGAACGATTTT-5'

D51_TPM.filter 20900836_21 TGAATCCTTCGGCTACCCATA

target id: Solyc11g069020.1.1

1305 5'-AAGGTTATGGATTGCTGAAGGATTTATAAAG-3' 1335 cleavage site: 1320

||||| | ||o||||||||o|

3'-ATACCCATCGGCTTCCTAAGT-5'

D51_TPM.filter 05291770_22 AGCAACTAATTCATGATCTGGT

target id: Solyc02g084890.1.1

875 5'-TAACTACAAGA-AATGAGTTAGTTGCTCGCAA-3' 905 cleavage site: 890

|| ||| ||||o|||||||||

3'-TGGTCTAGTACTTAATCAACGA-5'

D51_TPM.filter 05709005_22 AGTGACTAACTCATGATCTGGT

target id: Solyc02g084890.1.1

875 5'-TAACTACAAGA-AATGAGTTAGTTGCTCGCAA-3' 905 cleavage site: 890

|| ||| ||||||||||oo||

3'-TGGTCTAGTACTCAATCAGTGA-5'

D51_TPM.filter 05517686_20 AGCTTGTCAATGACTGTACA

target id: Solyc04g009130.2.1

528 5'-GATGTTGTACAGACATT-GCTAGCTCAACT-3' 556 cleavage site: 541

||||||| |||| o| ||||

3'-ACATGTCAGTAACTGTTCGA-5'

D51_TPM.filter 25866430_21 TTTAGCAAGAGTTGTTTTACC

target id: Solyc10g008230.1.1

506 5'-GAATTGGCAAAACGACTCTTGCTAGAAAAGT-3' 536 cleavage site: 521

|| |||||o||||||||||o|

3'-CCATTTTGTTGAGAACGATTT-5'

D51_TPM.filter 26653861_22 TTTTAGCAAGAGTTGTTTTACC

target id: Solyc10g008230.1.1

506 5'-GAATTGGCAAAACGACTCTTGCTAGAAAAGTT-3' 537 cleavage site: 522

|| |||||o||||||||||o||

3'-CCATTTTGTTGAGAACGATTTT-5'

D51_TPM.filter 25866155_21 TTTAGCAAGAGTTGTTTTACT

target id: Solyc10g008230.1.1

506 5'-GAATTGGCAAAACGACTCTTGCTAGAAAAGT-3' 536 cleavage site: 521

o| |||||o||||||||||o|

3'-TCATTTTGTTGAGAACGATTT-5'

D51_TPM.filter 26654022_22 TTTTAGCAAGAGTTGTTTTACT

target id: Solyc10g008230.1.1

506 5'-GAATTGGCAAAACGACTCTTGCTAGAAAAGTT-3' 537 cleavage site: 522

o| |||||o||||||||||o||

3'-TCATTTTGTTGAGAACGATTTT-5'

D51_TPM.filter 26653812_23 TTTTAGCAAGAGTTGTTTTACCT

target id: Solyc10g008230.1.1

505 5'-GGAATTGGCAAAACGACTCTTGCTAGAAAAGTT-3' 537 cleavage site: 522

|| |||||o||||||||||o||

3'-TCCATTTTGTTGAGAACGATTTT-5'

D51_TPM.filter 25866391_22 TTTAGCAAGAGTTGTTTTACCT

target id: Solyc10g008230.1.1

505 5'-GGAATTGGCAAAACGACTCTTGCTAGAAAAGT-3' 536 cleavage site: 521

|| |||||o||||||||||o|

3'-TCCATTTTGTTGAGAACGATTT-5'

D51_TPM.filter 26654347_23 TTTTAGCAAGAGTTGTTTTACCA

target id: Solyc10g008230.1.1

505 5'-GGAATTGGCAAAACGACTCTTGCTAGAAAAGTT-3' 537 cleavage site: 522

||| |||||o||||||||||o||

3'-ACCATTTTGTTGAGAACGATTTT-5'

D51_TPM.filter 25866163_22 TTTAGCAAGAGTTGTTTTACCC

target id: Solyc10g008230.1.1

505 5'-GGAATTGGCAAAACGACTCTTGCTAGAAAAGT-3' 536 cleavage site: 521

|| |||||o||||||||||o|

3'-CCCATTTTGTTGAGAACGATTT-5'

D51_TPM.filter 19059194_22 CTTTAGCAAGAGTTGTTTTACC

target id: Solyc10g008230.1.1

506 5'-GAATTGGCAAAACGACTCTTGCTAGAAAAGTT-3' 537 cleavage site: 522

|| |||||o||||||||||o|

3'-CCATTTTGTTGAGAACGATTTC-5'

D51_TPM.filter 26654102_21 TTTTAGCAAGAGTTGTTTTAC

target id: Solyc10g008230.1.1

507 5'-AATTGGCAAAACGACTCTTGCTAGAAAAGTT-3' 537 cleavage site: 522

|||||o||||||||||o||

3'-CATTTTGTTGAGAACGATTTT-5'

D51_TPM.filter 25866348_22 TTTAGCAAGAGTTGTTTTACCA

target id: Solyc10g008230.1.1

505 5'-GGAATTGGCAAAACGACTCTTGCTAGAAAAGT-3' 536 cleavage site: 521

||| |||||o||||||||||o|

3'-ACCATTTTGTTGAGAACGATTT-5'

D51_TPM.filter 25866736_20 TTTAGCAAGAGTTGTTTTAC

target id: Solyc10g008230.1.1

507 5'-AATTGGCAAAACGACTCTTGCTAGAAAAGT-3' 536 cleavage site: 521

|||||o||||||||||o|

3'-CATTTTGTTGAGAACGATTT-5'

D51_TPM.filter 26653934_23 TTTTAGCAAGAGTTGTTTTACCC

target id: Solyc10g008230.1.1

505 5'-GGAATTGGCAAAACGACTCTTGCTAGAAAAGTT-3' 537 cleavage site: 522

|| |||||o||||||||||o||

3'-CCCATTTTGTTGAGAACGATTTT-5'

D51_TPM.filter 24998006_21 TTGTAAGCGGCTTTAGCAAGA

target id: Solyc11g068360.1.1

643 5'-ACAACACTAGCTAAAGCGGTTTACAACGATG-3' 673 cleavage site: 658

|| |||||||| |o||||||

3'-AGAACGATTTCGGCGAATGTT-5'

D51_TPM.filter 24997995_21 TTGTAAGCGGCTTTAGCAAGT

target id: Solyc11g068360.1.1

643 5'-ACAACACTAGCTAAAGCGGTTTACAACGATG-3' 673 cleavage site: 658

||| |||||||| |o||||||

3'-TGAACGATTTCGGCGAATGTT-5'

D51_TPM.filter 21859117_20 TGTAAGCGGCTTTAGCAAGA

target id: Solyc11g068360.1.1

643 5'-ACAACACTAGCTAAAGCGGTTTACAACGAT-3' 672 cleavage site: 657

|| |||||||| |o|||||

3'-AGAACGATTTCGGCGAATGT-5'

D51_TPM.filter 25984397_22 TTTATCCCATATTTGTATTTTT

target id: Solyc11g068360.1.1

1121 5'-TTTTAAGAAGTG-AAATATGGGATCAGAAAAGT-3' 1152 cleavage site: 1137

|o||o|o ||||||||||| |o|

3'-TTTTTATGTTTATACCCTA-TTT-5'

D51_TPM.filter 26699166_22 TTTTATCCCATATTTGTATTTT

target id: Solyc11g068360.1.1

1122 5'-TTTAAGAAGTG-AAATATGGGATCAGAAAAGTT-3' 1153 cleavage site: 1138

o||o|o ||||||||||| |o||

3'-TTTTATGTTTATACCCTA-TTTT-5'

D51_TPM.filter 24997956_21 TTGTAAGCGGCTTTAGCAAGG

target id: Solyc11g068360.1.1

643 5'-ACAACACTAGCTAAAGCGGTTTACAACGATG-3' 673 cleavage site: 658

|| |||||||| |o||||||

3'-GGAACGATTTCGGCGAATGTT-5'

D51_TPM.filter 11510382_24 ATTTCTATTTTCTACGTCACATGT

target id: Solyc11g068360.1.1

973 5'-AAACGACAT-TCAC-TAGAAAATAGGGATCATCC-3' 1004 cleavage site: 989

|||| | || ||||||||||oo||

3'-TGTACACTGCATCTTTTATCTTTA-5'

D51_TPM.filter 21859136_20 TGTAAGCGGCTTTAGCAAGG

target id: Solyc11g068360.1.1

643 5'-ACAACACTAGCTAAAGCGGTTTACAACGAT-3' 672 cleavage site: 657

|| |||||||| |o|||||

3'-GGAACGATTTCGGCGAATGT-5'

D51_TPM.filter 21859201_20 TGTAAGCGGCTTTAGCAAGT

target id: Solyc11g068360.1.1

643 5'-ACAACACTAGCTAAAGCGGTTTACAACGAT-3' 672 cleavage site: 657

||| |||||||| |o|||||

3'-TGAACGATTTCGGCGAATGT-5'

D51_TPM.filter 21859116_20 TGTAAGCGGCTTTAGCAAGC

target id: Solyc11g068360.1.1

643 5'-ACAACACTAGCTAAAGCGGTTTACAACGAT-3' 672 cleavage site: 657

|| |||||||| |o|||||

3'-CGAACGATTTCGGCGAATGT-5'

D51_TPM.filter 03260061_24 AATGTCTGGTATTTTTCATGAAGT

target id: Solyc01g090430.2.1

2961 5'-ATGTAACGTTCA--AGAAATATCAGACATTGTTTC-3' 2993 cleavage site: 2978

|| |||| |o|||||o||||||||

3'-TG-AAGTACTTTTTATGGTCTGTAA-5'

D51_TPM.filter 21584478_21 TGGTAGAAATTGAAAGATGCT

target id: Solyc01g090430.2.1

1803 5'-CAAGGAGT-TCTTTCAA-TTGTATCATCTGA-3' 1831 cleavage site: 1816

||o |||||||| || ||o||

3'-TCGTAGAAAGTTAAAGATGGT-5'

D51_TPM.filter 15129408_22 GTCTGTAGATCAAAATTGCGAG

target id: Solyc01g090430.2.1

2697 5'-ATTCCCTCCTGATTCTGATGTACAGACAGCTT-3' 2728 cleavage site: 2713

||| oo||| |||| |||||||

3'-GAGCGTTAAAACTAGATGTCTG-5'

D51_TPM.filter 06660851_24 ACGATGTCTGATATTACTTTGAAG

target id: Solyc01g090430.2.1

2963 5'-GTAACGTTC-AAGAAATATCAGACATTGTTTCTT-3' 2995 cleavage site: 2980

||| ||| ||||||||||||o||

3'-GAAGTTTCATTATAGTCTGTAGCA-5'

D51_TPM.filter 08974426_24 ATGAATTTTGAATAATTGAAGTGT

target id: Solyc05g054020.1.1

2463 5'-TTTCGATGATTCTATTA-TCAAAATTCATGATCT-3' 2495 cleavage site: 2480

|oo ||| |||| |||||||||||

3'-TGTGAAGTTAATAAGTTTTAAGTA-5'

D51_TPM.filter 05238206_24 AGGTCTGTCAGAAACACTCTCTGT

target id: Solyc04g007070.2.1

1387 5'-AGGAGATAGCAGAGTTTTATTTGACAGACCTTCTCA-3' 1422 cleavage site: 1407

|o|| ||||| || |o||||||||||

3'-TGTC-TCTCACAA-AGACTGTCTGGA-5'

D51_TPM.filter 02817047_21 AATAATAGAATAGGACGTGTG

target id: Solyc04g007070.2.1

2840 5'-GTTTGCATCTTTCCTATTCTATTATCTTTGT-3' 2870 cleavage site: 2855

||o o ||||||||||||||

3'-GTGTGCAGGATAAGATAATAA-5'

D51_TPM.filter 15616407_21 CAAGAGTTTGTAGACGAGGTG

target id: Solyc04g007070.2.1

2003 5'-TAAACCTTCTTGTCTACAAACTCTTTCTATG-3' 2033 cleavage site: 2018

o||o||||||||||||||

3'-GTGGAGCAGATGTTTGAGAAC-5'

D51_TPM.filter 02817804_20 AATAATAGAATAGGACGTGT

target id: Solyc04g007070.2.1

2841 5'-TTTGCATCTTTCCTATTCTATTATCTTTGT-3' 2870 cleavage site: 2855

|o o ||||||||||||||

3'-TGTGCAGGATAAGATAATAA-5'

D51_TPM.filter 22274775_21 TCAAGAGTTTGTAGACGAGGT

target id: Solyc04g007070.2.1

2004 5'-AAACCTTCTTGTCTACAAACTCTTTCTATGG-3' 2034 cleavage site: 2019

o||o||||||||||||||

3'-TGGAGCAGATGTTTGAGAACT-5'

D51_TPM.filter 08001492_20 ATAATAGAATAGGACGTGTG

target id: Solyc04g007070.2.1

2840 5'-GTTTGCATCTTTCCTATTCTATTATCTTTG-3' 2869 cleavage site: 2854

||o o ||||||||||||||

3'-GTGTGCAGGATAAGATAATA-5'

D51_TPM.filter 20413660_23 TATGACGTGTCTTTGATTTTATT

target id: Solyc04g007070.2.1

397 5'-TACATGATCAAATCAAAGACAT-TCGTAACAAC-3' 428 cleavage site: 413

o|| ||||||||||||o ||o||

3'-TTATTTTAGTTTCTGTGCAGTAT-5'

D51_TPM.filter 22274495_20 TCAAGAGTTTGTAGACGAGG

target id: Solyc04g007070.2.1

2005 5'-AACCTTCTTGTCTACAAACTCTTTCTATGG-3' 2034 cleavage site: 2019

o||o||||||||||||||

3'-GGAGCAGATGTTTGAGAACT-5'

D51_TPM.filter 05056565_24 AGGCAAAAACAGAATTACAGAATA

target id: Solyc04g007070.2.1

2897 5'-TTTCCTATTCTGTAATCTTTGTATTTGCTTTGGTA-3' 2931 cleavage site: 2916

||||||||||| |o||| |||||o|

3'-ATAAGACATTA-AGACAAAAACGGA-5'

D51_TPM.filter 01724746_22 AAGGATCTGACATGCATCTGTC

target id: Solyc04g007070.2.1

1629 5'-CCTTTTGCAGA-ACATGTCAGATCTTTCTATT-3' 1659 cleavage site: 1644

o|||| |||||||||||o||

3'-CTGTCTACGTACAGTCTAGGAA-5'

D51_TPM.filter 11141430_24 ATTCTGAACCTTTCAACCACTAAG

target id: Solyc04g007070.2.1

2403 5'-CAAGTCTT-GTGGATTGAAAGG-TCAGAATTAGAA-3' 2435 cleavage site: 2420

||| |||| |||||||| |||||||

3'-GAATCACC-AACTTTCCAAGTCTTA-5'

D51_TPM.filter 23660461_21 TCTTCAAACTTTGGACGTTCA

target id: Solyc04g007070.2.1

2325 5'-TCTCTTGAG-GTCCTAAAGTTTAAAGAAAATG-3' 2355 cleavage site: 2340

|||o || |o||||||| ||||

3'-ACTTGCA-GGTTTCAAACTTCT-5'

D51_TPM.filter 12256083_24 GAGGCAAAAACAGAATTACAGAAT

target id: Solyc04g007070.2.1

2898 5'-TTCCTATTCTGTAATCTTTGTATTTGCTTTGGTAT-3' 2932 cleavage site: 2917

|||||||||| |o||| |||||o|o

3'-TAAGACATTA-AGACAAAAACGGAG-5'

D51_TPM.filter 10823624_21 ATTGGTGAAATATTCTAGAGT

target id: Solyc09g092280.1.1

2002 5'-GTTGAAC-CGAGATTATTTCACCGGTTGATC-3' 2031 cleavage site: 2016

|| | ||| |||||||||oo|

3'-TGAGATCTTATAAAGTGGTTA-5'

D51_TPM.filter 25866430_21 TTTAGCAAGAGTTGTTTTACC

target id: Solyc11g020100.1.1

530 5'-GTATCGGTAAGACAACTCTTGCCAAAAAAGT-3' 560 cleavage site: 545

|||||o||||||||||| |||

3'-CCATTTTGTTGAGAACGATTT-5'

D51_TPM.filter 26653861_22 TTTTAGCAAGAGTTGTTTTACC

target id: Solyc11g020100.1.1

530 5'-GTATCGGTAAGACAACTCTTGCCAAAAAAGTT-3' 561 cleavage site: 546

|||||o||||||||||| ||||

3'-CCATTTTGTTGAGAACGATTTT-5'

D51_TPM.filter 25694497_22 TTCTTGGCTAGAGTTGTATTGC

target id: Solyc11g020100.1.1

531 5'-TATCGGTAAGACAACTCTTGCCAA-AAAAGTT-3' 561 cleavage site: 546

|o|| |||||||| ||||| ||

3'-CGTTATGTTGAGATCGGTTCTT-5'

D51_TPM.filter 25866155_21 TTTAGCAAGAGTTGTTTTACT

target id: Solyc11g020100.1.1

530 5'-GTATCGGTAAGACAACTCTTGCCAAAAAAGT-3' 560 cleavage site: 545

o||||o||||||||||| |||

3'-TCATTTTGTTGAGAACGATTT-5'

D51_TPM.filter 26654022_22 TTTTAGCAAGAGTTGTTTTACT

target id: Solyc11g020100.1.1

530 5'-GTATCGGTAAGACAACTCTTGCCAAAAAAGTT-3' 561 cleavage site: 546

o||||o||||||||||| ||||

3'-TCATTTTGTTGAGAACGATTTT-5'

D51_TPM.filter 25694767_22 TTCTTGGCTAGAGTTGTATTGT

target id: Solyc11g020100.1.1

531 5'-TATCGGTAAGACAACTCTTGCCAA-AAAAGTT-3' 561 cleavage site: 546

oo|| |||||||| ||||| ||

3'-TGTTATGTTGAGATCGGTTCTT-5'

D51_TPM.filter 02344324_22 AACGATATGAATTTTGGCTAAC

target id: Solyc11g020100.1.1

1380 5'-GAGAAGTT-G-CAAAATTCATGATC-TTCTTCG-3' 1409 cleavage site: 1394

||| | |||||||||| ||| ||

3'-CAATCGGTTTTAAGTA-TAGCAA-5'

D51_TPM.filter 20804880_21 TGAAATCTTTAGAATATCTAG

target id: Solyc11g020100.1.1

603 5'-ATGGGTTA-CTATCTCTGAAGATTTCAACCAG-3' 633 cleavage site: 618

o|| ||| |||o|||||||||

3'-GATCTATA-AGATTTCTAAAGT-5'

D51_TPM.filter 21647675_21 TGGTTAATAGAATTCGGCTTT

target id: Solyc11g020100.1.1

813 5'-GAATAAAAGCCGAATTCTATTAACCACTCGT-3' 843 cleavage site: 828

|||||||||||||||||||||

3'-TTTCGGCTTAAGATAATTGGT-5'

D51_TPM.filter 26653812_23 TTTTAGCAAGAGTTGTTTTACCT

target id: Solyc11g020100.1.1

529 5'-GGTATCGGTAAGACAACTCTTGCCAAAAAAGTT-3' 561 cleavage site: 546

|||||o||||||||||| ||||

3'-TCCATTTTGTTGAGAACGATTTT-5'

D51_TPM.filter 19348773_21 TAAGAAGATCATGAATTTTGC

target id: Solyc11g020100.1.1

1383 5'-AAGTTGCAAAATTCATGATCTTCTTCGCCAA-3' 1413 cleavage site: 1398

||||||||||||||||||||

3'-CGTTTTAAGTACTAGAAGAAT-5'

D51_TPM.filter 26654357_22 TTTTAGCAAGAGTTGTTTTACA

target id: Solyc11g020100.1.1

530 5'-GTATCGGTAAGACAACTCTTGCCAAAAAAGTT-3' 561 cleavage site: 546

||||o||||||||||| ||||

3'-ACATTTTGTTGAGAACGATTTT-5'

D51_TPM.filter 25694504_20 TTCTTGGCTAGAGTTGTATT

target id: Solyc11g020100.1.1

533 5'-TCGGTAAGACAACTCTTGCCAA-AAAAGTT-3' 561 cleavage site: 546

|| |||||||| ||||| ||

3'-TTATGTTGAGATCGGTTCTT-5'

D51_TPM.filter 25866253_21 TTTAGCAAGAGTTGTTTTACA

target id: Solyc11g020100.1.1

530 5'-GTATCGGTAAGACAACTCTTGCCAAAAAAGT-3' 560 cleavage site: 545

||||o||||||||||| |||

3'-ACATTTTGTTGAGAACGATTT-5'

D51_TPM.filter 25866391_22 TTTAGCAAGAGTTGTTTTACCT

target id: Solyc11g020100.1.1

529 5'-GGTATCGGTAAGACAACTCTTGCCAAAAAAGT-3' 560 cleavage site: 545

|||||o||||||||||| |||

3'-TCCATTTTGTTGAGAACGATTT-5'

D51_TPM.filter 26653543_24 TTTTAGCAAGAGTTGTTTTACTTT

target id: Solyc11g020100.1.1

528 5'-CGGTATCGGTAAGACAACTCTTGCCAAAAAAGTT-3' 561 cleavage site: 546

o||||o||||||||||| ||||

3'-TTTCATTTTGTTGAGAACGATTTT-5'

D51_TPM.filter 20006612_21 TAGTTAATAGAATTCGGCTTT

target id: Solyc11g020100.1.1

813 5'-GAATAAAAGCCGAATTCTATTAACCACTCGT-3' 843 cleavage site: 828

|||||||||||||||||||

3'-TTTCGGCTTAAGATAATTGAT-5'

D51_TPM.filter 25866686_23 TTTAGCAAGAGTTGTTTTACTTT

target id: Solyc11g020100.1.1

528 5'-CGGTATCGGTAAGACAACTCTTGCCAAAAAAGT-3' 560 cleavage site: 545

o||||o||||||||||| |||

3'-TTTCATTTTGTTGAGAACGATTT-5'

D51_TPM.filter 15582627_21 CAAATAGAGAAAACAGAACGT

target id: Solyc11g020100.1.1

1176 5'-TTTGAGCG-CTTGTTTTCTCTATGTTGGAGGT-3' 1206 cleavage site: 1191

o|| o|||||||||||| |||

3'-TGCAAGACAAAAGAGATA-AAC-5'

D51_TPM.filter 24483281_22 TTGAAATCTTTAGAATATCTAG

target id: Solyc11g020100.1.1

603 5'-ATGGGTTA-CTATCTCTGAAGATTTCAACCAGA-3' 634 cleavage site: 619

o|| ||| |||o||||||||||

3'-GATCTATA-AGATTTCTAAAGTT-5'

D51_TPM.filter 05613524_21 AGTAGTTAATAGAATTCGGCT

target id: Solyc11g020100.1.1

815 5'-ATAAAAGCCGAATTCTATTAACCACTCGTCT-3' 845 cleavage site: 830

||||||||||||||||| |||

3'-TCGGCTTAAGATAATTGATGA-5'

D51_TPM.filter 05767456_21 AGTGGTTAATAGAATTCGGCT

target id: Solyc11g020100.1.1

815 5'-ATAAAAGCCGAATTCTATTAACCACTCGTCT-3' 845 cleavage site: 830

|||||||||||||||||||||

3'-TCGGCTTAAGATAATTGGTGA-5'

D51_TPM.filter 05613335_21 AGTAGTTAATAGAATTCGGCC

target id: Solyc11g020100.1.1

815 5'-ATAAAAGCCGAATTCTATTAACCACTCGTCT-3' 845 cleavage site: 830

|||||||||||||||| |||

3'-CCGGCTTAAGATAATTGATGA-5'

D51_TPM.filter 25866557_21 TTTAGCAAGAGTTGTTTTACG

target id: Solyc11g020100.1.1

530 5'-GTATCGGTAAGACAACTCTTGCCAAAAAAGT-3' 560 cleavage site: 545

||||o||||||||||| |||

3'-GCATTTTGTTGAGAACGATTT-5'

D51_TPM.filter 26654347_23 TTTTAGCAAGAGTTGTTTTACCA

target id: Solyc11g020100.1.1

529 5'-GGTATCGGTAAGACAACTCTTGCCAAAAAAGTT-3' 561 cleavage site: 546

|||||o||||||||||| ||||

3'-ACCATTTTGTTGAGAACGATTTT-5'

D51_TPM.filter 25866163_22 TTTAGCAAGAGTTGTTTTACCC

target id: Solyc11g020100.1.1

529 5'-GGTATCGGTAAGACAACTCTTGCCAAAAAAGT-3' 560 cleavage site: 545

|||||o||||||||||| |||

3'-CCCATTTTGTTGAGAACGATTT-5'

D51_TPM.filter 19059194_22 CTTTAGCAAGAGTTGTTTTACC

target id: Solyc11g020100.1.1

530 5'-GTATCGGTAAGACAACTCTTGCCAAAAAAGTT-3' 561 cleavage site: 546

|||||o||||||||||| |||

3'-CCATTTTGTTGAGAACGATTTC-5'

D51_TPM.filter 26654102_21 TTTTAGCAAGAGTTGTTTTAC

target id: Solyc11g020100.1.1

531 5'-TATCGGTAAGACAACTCTTGCCAAAAAAGTT-3' 561 cleavage site: 546

||||o||||||||||| ||||

3'-CATTTTGTTGAGAACGATTTT-5'

D51_TPM.filter 25866348_22 TTTAGCAAGAGTTGTTTTACCA

target id: Solyc11g020100.1.1

529 5'-GGTATCGGTAAGACAACTCTTGCCAAAAAAGT-3' 560 cleavage site: 545

|||||o||||||||||| |||

3'-ACCATTTTGTTGAGAACGATTT-5'

D51_TPM.filter 25039268_21 TTGTATTCTTCAGAGATAGTG

target id: Solyc11g020100.1.1

604 5'-TGGGTTACTATCTCTGAAGATTTCAACCAGA-3' 634 cleavage site: 619

o|||||||||||||| | |||

3'-GTGATAGAGACTTCTTATGTT-5'

D51_TPM.filter 19348359_21 TAAGAAGATCATGAATTTTGT

target id: Solyc11g020100.1.1

1383 5'-AAGTTGCAAAATTCATGATCTTCTTCGCCAA-3' 1413 cleavage site: 1398

o|||||||||||||||||||

3'-TGTTTTAAGTACTAGAAGAAT-5'

D51_TPM.filter 25866736_20 TTTAGCAAGAGTTGTTTTAC

target id: Solyc11g020100.1.1

32 5'-ATACATTAGAACAACTCTT-C-AAACCTAA-3' 59 cleavage site: 44

||o|||||||||| | |||

3'-CATTTTGTTGAGAACGATTT-5'

D51_TPM.filter 26653916_22 TTTTAGCAAGAGTTGTTTTACG

target id: Solyc11g020100.1.1

530 5'-GTATCGGTAAGACAACTCTTGCCAAAAAAGTT-3' 561 cleavage site: 546

||||o||||||||||| ||||

3'-GCATTTTGTTGAGAACGATTTT-5'

D51_TPM.filter 12335067_21 GAGTAGTTAATAGAATTCGGC

target id: Solyc11g020100.1.1

816 5'-TAAAAGCCGAATTCTATTAACCACTCGTCTC-3' 846 cleavage site: 831

|||||||||||||||| ||||

3'-CGGCTTAAGATAATTGATGAG-5'

D51_TPM.filter 26653934_23 TTTTAGCAAGAGTTGTTTTACCC

target id: Solyc11g020100.1.1

529 5'-GGTATCGGTAAGACAACTCTTGCCAAAAAAGTT-3' 561 cleavage site: 546

|||||o||||||||||| ||||

3'-CCCATTTTGTTGAGAACGATTTT-5'

D51_TPM.filter 24396922_22 TTATTAACAAGATCGTCCTGGA

target id: Solyc05g013280.2.1

4161 5'-AGGTTTCTTGGACGATCTTATTGGTAGGAATC-3' 4192 cleavage site: 4177

||o |||||||||| ||oo||o

3'-AGGTCCTGCTAGAACAATTATT-5'

D51_TPM.filter 21044149_21 TGACATCAGGGAGAGAAAGTA

target id: Solyc05g013280.2.1

2739 5'-TTACACACTATATCTCTCTGATGTCAAGCAA-3' 2769 cleavage site: 2754

||| | ||||o|||||||||

3'-ATGAAAGAGAGGGACTACAGT-5'

D51_TPM.filter 24606163_21 TTGACATCAGGGAGAGAAAGT

target id: Solyc05g013280.2.1

2740 5'-TACACACTATATCTCTCTGATGTCAAGCAAT-3' 2770 cleavage site: 2755

||| | ||||o||||||||||

3'-TGAAAGAGAGGGACTACAGTT-5'

D51_TPM.filter 23721561_22 TCTTTCCTACTCCTCCCATACC

target id: Solyc04g005540.2.1

633 5'-TATGTGGTATGGGTGGAGTTGGTAAGACAACA-3' 664 cleavage site: 649

|||||||| ||||| || ||||

3'-CCATACCCTCCTCATCCTTTCT-5'

D51_TPM.filter 26575701_21 TTTCTCTTGTAGCTTGACTAG

target id: Solyc04g005540.2.1

170 5'-TATTTCTA--CTA-CTACAAGAGAAACATGA-3' 197 cleavage site: 182

||| | | ||||||||||||

3'-GATCAGTTCGATGTTCTCTTT-5'

D51_TPM.filter 23721573_22 TCTTTCCTACTCCTCCCATACT

target id: Solyc04g005540.2.1

633 5'-TATGTGGTATGGGTGGAGTTGGTAAGACAACA-3' 664 cleavage site: 649

o||||||| ||||| || ||||

3'-TCATACCCTCCTCATCCTTTCT-5'

D51_TPM.filter 23720559_22 TCTTTCCTACTCCTCCCATACA

target id: Solyc04g005540.2.1

633 5'-TATGTGGTATGGGTGGAGTTGGTAAGACAACA-3' 664 cleavage site: 649

||||||| ||||| || ||||

3'-ACATACCCTCCTCATCCTTTCT-5'

D51_TPM.filter 18113632_20 CTAGTGTCGTCTTACCTACT

target id: Solyc04g005540.2.1

644 5'-GGTGGAGTTGGTAAGACAACACTGACTGAG-3' 673 cleavage site: 658

||| |||||||| |||||o

3'-TCATCCATTCTGCTGTGATC-5'

D51_TPM.filter 07624441_21 ACTTCCATGATCTTCTGAGCT

target id: Solyc04g005540.2.1

992 5'-ATGGGAGCTCAGAAGATCATGGAAGTTGGAA-3' 1022 cleavage site: 1007

|||||||||||||||||||||

3'-TCGAGTCTTCTAGTACCTTCA-5'

D51_TPM.filter 26469238_21 TTTCCAATTCCACCCATTCCT

target id: Solyc04g005540.2.1

632 5'-ATATGTGGTATGGGTGGAGTTGGTAAGACAAC-3' 663 cleavage site: 648

|| |||||||||o|||| ||o

3'-TCCTTACCCACCTTAACC-TTT-5'

D51_TPM.filter 04919682_24 AGGATTTTAGTAAGTGAGATGAGT

target id: Solyc04g005540.2.1

2800 5'-CAACAACTC-TATCACTGACTCAAATCCTCTTTT-3' 2832 cleavage site: 2817

|||| | ||||| ||| |||||||

3'-TGAGTAGAGTGAATGATTTTAGGA-5'

D51_TPM.filter 25489903_21 TTCCATGATCTTCTGAGCTCC

target id: Solyc04g005540.2.1

990 5'-CAATGGGAGCTCAGAAGATCATGGAAGTTGG-3' 1020 cleavage site: 1005

|||||||||||||||||||||

3'-CCTCGAGTCTTCTAGTACCTT-5'

D51_TPM.filter 18113699_21 CTAGTGTCGTCTTACCTACTT

target id: Solyc04g005540.2.1

643 5'-GGGTGGAGTTGGTAAGACAACACTGACTGAG-3' 673 cleavage site: 658

o||| |||||||| |||||o

3'-TTCATCCATTCTGCTGTGATC-5'

D51_TPM.filter 23720745_22 TCTTTCCTACTCCCCCCATACC

target id: Solyc04g005540.2.1

633 5'-TATGTGGTATGGGTGGAGTTGGTAAGACAACA-3' 664 cleavage site: 649

|||||||| ||||| || ||||

3'-CCATACCCCCCTCATCCTTTCT-5'

D51_TPM.filter 09169183_24 ATGATTTGAGTCGGTAGATAGAGT

target id: Solyc04g005540.2.1

2800 5'-CAACAACTCTATC-ACTGACTCAAATCCTCTTTT-3' 2832 cleavage site: 2817

|||||||| ||o||||||||||

3'-TGAGATAGATGGCTGAGTTTAGTA-5'

D51_TPM.filter 07629883_21 ACTTCTATGATCTTTCGAGCC

target id: Solyc04g005540.2.1

992 5'-ATGGGAGCTCAGAAGATCATGGAAGTTGGAA-3' 1022 cleavage site: 1007

|||| o||||||||o|||||

3'-CCGAGCTTTCTAGTATCTTCA-5'

D51_TPM.filter 19000618_21 CTTCCATGATCTTCTGAGCTT

target id: Solyc04g005540.2.1

991 5'-AATGGGAGCTCAGAAGATCATGGAAGTTGGA-3' 1021 cleavage site: 1006

o||||||||||||||||||||

3'-TTCGAGTCTTCTAGTACCTTC-5'

D51_TPM.filter 15676271_20 CAAGTGTTGTCTTACCTACT

target id: Solyc04g005540.2.1

644 5'-GGTGGAGTTGGTAAGACAACACTGACTGAG-3' 673 cleavage site: 658

||| ||||||||||||||

3'-TCATCCATTCTGTTGTGAAC-5'

D51_TPM.filter 19000730_21 CTTCCATGATCTTCTGAGCTC

target id: Solyc04g005540.2.1

991 5'-AATGGGAGCTCAGAAGATCATGGAAGTTGGA-3' 1021 cleavage site: 1006

|||||||||||||||||||||

3'-CTCGAGTCTTCTAGTACCTTC-5'

D51_TPM.filter 19439255_21 TAAGTAATTCTTCAGGACAGA

target id: Solyc04g005540.2.1

1351 5'-TAATATCTGTCCTGAAGAATTACTTAGATAT-3' 1381 cleavage site: 1366

|||||||||||||||||||||

3'-AGACAGGACTTCTTAATGAAT-5'

D51_TPM.filter 18113819_21 CTAGTGTCGTCTTACCTACTC

target id: Solyc04g005540.2.1

643 5'-GGGTGGAGTTGGTAAGACAACACTGACTGAG-3' 673 cleavage site: 658

|||| |||||||| |||||o

3'-CTCATCCATTCTGCTGTGATC-5'

D51_TPM.filter 23636187_21 TCTTGGAGCTCTCTTGAAGTT

target id: Solyc04g005540.2.1

2378 5'-GATTCAAC--CGGAGAGGGCTCCAAGAACGTG-3' 2407 cleavage site: 2392

||| | o||||o|||||||||

3'-TTGAAG-TTCTCTCGAGGTTCT-5'

D51_TPM.filter 15546764_21 CAAAGCAAAAATAGGTACCTG

target id: Solyc04g005540.2.1

1304 5'-GAAGCTAAGTACCTCTTTTTGCTTTGTTCCT-3' 1334 cleavage site: 1319

o| |||||| |||||||||||

3'-GTCCATGGATAAAAACGAAAC-5'

D51_TPM.filter 07629765_21 ACTTCTATGATCTTTCGAGCT

target id: Solyc04g005540.2.1

992 5'-ATGGGAGCTCAGAAGATCATGGAAGTTGGAA-3' 1022 cleavage site: 1007

||||| o||||||||o|||||

3'-TCGAGCTTTCTAGTATCTTCA-5'

D51_TPM.filter 22129026_21 TGTTACTTTGCACTGATGGTT

target id: Solyc04g005540.2.1

933 5'-ACCACAAACATCGGTGCAAAGTGACATTCAC-3' 963 cleavage site: 948

|| ||||o|||||||||o|||

3'-TTGGTAGTCACGTTTCATTGT-5'

D51_TPM.filter 25490266_21 TTCCATGATCTTCTGAGCTCT

target id: Solyc04g005540.2.1

990 5'-CAATGGGAGCTCAGAAGATCATGGAAGTTGG-3' 1020 cleavage site: 1005

o||||||||||||||||||||

3'-TCTCGAGTCTTCTAGTACCTT-5'

D51_TPM.filter 23721078_22 TCTTTCCTACTCCTCCCATACG

target id: Solyc04g005540.2.1

633 5'-TATGTGGTATGGGTGGAGTTGGTAAGACAACA-3' 664 cleavage site: 649

||||||| ||||| || ||||

3'-GCATACCCTCCTCATCCTTTCT-5'

D51_TPM.filter 25675546_21 TTCTCTTCTGTGAATAATTCT

target id: Solyc04g005540.2.1

3050 5'-ATGGAAGAAGTGATCACAGAAGAGGAACAAC-3' 3080 cleavage site: 3065

|||| |o |||||||||||o|

3'-TCTTAATAAGTGTCTTCTCTT-5'

D51_TPM.filter 23720411_22 TCTTTCCCACTCCTCCCATACC

target id: Solyc04g005540.2.1

633 5'-TATGTGGTATGGGTGGAGTTGGTAAGACAACA-3' 664 cleavage site: 649

|||||||| ||||| || ||||

3'-CCATACCCTCCTCACCCTTTCT-5'

D51_TPM.filter 02707603_21 AACTTCTATGATCTTTCGAGC

target id: Solyc04g005540.2.1

993 5'-TGGGAGCTCAGAAGATCATGGAAGTTGGAAC-3' 1023 cleavage site: 1008

|||| o||||||||o||||||

3'-CGAGCTTTCTAGTATCTTCAA-5'

D51_TPM.filter 07624113_21 ACTTCCATGATCTTCTGAGCC

target id: Solyc04g005540.2.1

992 5'-ATGGGAGCTCAGAAGATCATGGAAGTTGGAA-3' 1022 cleavage site: 1007

||||||||||||||||||||

3'-CCGAGTCTTCTAGTACCTTCA-5'

D51_TPM.filter 23721096_21 TCTTTCCTACTCCTCCCATAC

target id: Solyc04g005540.2.1

634 5'-ATGTGGTATGGGTGGAGTTGGTAAGACAACA-3' 664 cleavage site: 649

||||||| ||||| || ||||

3'-CATACCCTCCTCATCCTTTCT-5'

D51_TPM.filter 23721277_22 TCTTTCCTACTCCCCCCATACT

target id: Solyc04g005540.2.1

633 5'-TATGTGGTATGGGTGGAGTTGGTAAGACAACA-3' 664 cleavage site: 649

o||||||| ||||| || ||||

3'-TCATACCCCCCTCATCCTTTCT-5'

D51_TPM.filter 26469771_21 TTTCCAATTCCACCCATTCCC

target id: Solyc04g005540.2.1

632 5'-ATATGTGGTATGGGTGGAGTTGGTAAGACAAC-3' 663 cleavage site: 648

|| |||||||||o|||| ||o

3'-CCCTTACCCACCTTAACC-TTT-5'

D51_TPM.filter 15676224_20 CAAGTGTTGTCTTACCTACC

target id: Solyc04g005540.2.1

644 5'-GGTGGAGTTGGTAAGACAACACTGACTGAG-3' 673 cleavage site: 658

|| ||||||||||||||

3'-CCATCCATTCTGTTGTGAAC-5'

D51_TPM.filter 26121034_21 TTTGATTCCTGGAAAGATGTG

target id: Solyc04g005540.2.1

1389 5'-GGCTTCACATCTTTCCAGGAATCAAAAATTT-3' 1419 cleavage site: 1404

|||||||||||||||||||||

3'-GTGTAGAAAGGTCCTTAGTTT-5'

D51_TPM.filter 23002816_21 TCCATGATCTTCTGAGCTCCC

target id: Solyc04g005540.2.1

989 5'-GCAATGGGAGCTCAGAAGATCATGGAAGTTG-3' 1019 cleavage site: 1004

|||||||||||||||||||||

3'-CCCTCGAGTCTTCTAGTACCT-5'

D51_TPM.filter 19273735_21 TAAACAACCTTTCTTTTTTCG

target id: Solyc04g005540.2.1

685 5'-AATGGCGAAAAAAGAAAGGTTGTTTAAAGAT-3' 715 cleavage site: 700

|||||||||||||||||||||

3'-GCTTTTTTCTTTCCAACAAAT-5'

D51_TPM.filter 19000757_20 CTTCCATGATCTTCTGAGCT

target id: Solyc04g005540.2.1

992 5'-ATGGGAGCTCAGAAGATCATGGAAGTTGGA-3' 1021 cleavage site: 1006

||||||||||||||||||||

3'-TCGAGTCTTCTAGTACCTTC-5'

D51_TPM.filter 19674942_22 TAATTCTTCTTCTGTATTCATT

target id: Solyc04g005540.2.1

4392 5'-ACGGTAATGAAT-CTGAAGAAGATTTAAGTGA-3' 4422 cleavage site: 4407

||||||| | |||||||| |||

3'-TTACTTATGTCTTCTTCTTAAT-5'

D51_TPM.filter 23720544_22 TCTTTCCTGCTCCTCCCATACC

target id: Solyc04g005540.2.1

633 5'-TATGTGGTATGGGTGGAGTTGGTAAGACAACA-3' 664 cleavage site: 649

|||||||| ||||o || ||||

3'-CCATACCCTCCTCGTCCTTTCT-5'

D51_TPM.filter 23681971_22 TCTTCCCTACTCCTCCCATACC

target id: Solyc04g005540.2.1

633 5'-TATGTGGTATGGGTGGAGTTGGTAAGACAACA-3' 664 cleavage site: 649

|||||||| ||||| || ||||

3'-CCATACCCTCCTCATCCCTTCT-5'

D51_TPM.filter 07624096_21 ACTTCCATGATCTTCTGAGCA

target id: Solyc04g005540.2.1

992 5'-ATGGGAGCTCAGAAGATCATGGAAGTTGGAA-3' 1022 cleavage site: 1007

||||||||||||||||||||

3'-ACGAGTCTTCTAGTACCTTCA-5'

D51_TPM.filter 15546744_22 CAAAGCAAAAATAGGTACCTGG

target id: Solyc04g005540.2.1

1303 5'-TGAAGCTAAGTACCTCTTTTTGCTTTGTTCCT-3' 1334 cleavage site: 1319

|o| |||||| |||||||||||

3'-GGTCCATGGATAAAAACGAAAC-5'

D51_TPM.filter 26575101_21 TTTCTCTTGTAGCTTGGCTAG

target id: Solyc04g005540.2.1

170 5'-TATTTCTA-CT-A-CTACAAGAGAAACATGA-3' 197 cleavage site: 182

||| |o | ||||||||||||

3'-GATCGGTTCGATGTTCTCTTT-5'

D51_TPM.filter 26469466_21 TTTCCAATTCCACCCATTCCA

target id: Solyc04g005540.2.1

632 5'-ATATGTGGTATGGGTGGAGTTGGTAAGACAAC-3' 663 cleavage site: 648

||| |||||||||o|||| ||o

3'-ACCTTACCCACCTTAACC-TTT-5'

D51_TPM.filter 24417457_21 TTATTGGGGAACTTGTCACTT

target id: Solyc04g005540.2.1

2638 5'-ACGTGAAGTAATCAAGTTCCCCAATTTATATG-3' 2669 cleavage site: 2654

|||| | |||||||||||||

3'-TTCACT-GTTCAAGGGGTTATT-5'

D51_TPM.filter 23219160_21 TCCTGTAATTTGAACGGCTCT

target id: Solyc04g005540.2.1

1724 5'-GAAGAAGAACCAATCAAATTACAGGATGATT-3' 1754 cleavage site: 1739

||| || |||||||||||||

3'-TCTCGGCAAGTTTAATGTCCT-5'

D51_TPM.filter 02706291_22 AACTTCCATGATCTTCTGAGCT

target id: Solyc04g005540.2.1

992 5'-ATGGGAGCTCAGAAGATCATGGAAGTTGGAAC-3' 1023 cleavage site: 1008

||||||||||||||||||||||

3'-TCGAGTCTTCTAGTACCTTCAA-5'

D51_TPM.filter 25490144_21 TTCCATGATCTTCTGAGCTTC

target id: Solyc04g005540.2.1

990 5'-CAATGGGAGCTCAGAAGATCATGGAAGTTGG-3' 1020 cleavage site: 1005

|o|||||||||||||||||||

3'-CTTCGAGTCTTCTAGTACCTT-5'

D51_TPM.filter 23721541_23 TCTTTCCTACTCCTCCCATACCT

target id: Solyc04g005540.2.1

632 5'-ATATGTGGTATGGGTGGAGTTGGTAAGACAACA-3' 664 cleavage site: 649

|||||||| ||||| || ||||

3'-TCCATACCCTCCTCATCCTTTCT-5'

D51_TPM.filter 15676276_20 CAAGTGTTGTCTTACCTACA

target id: Solyc04g005540.2.1

644 5'-GGTGGAGTTGGTAAGACAACACTGACTGAG-3' 673 cleavage site: 658

|| ||||||||||||||

3'-ACATCCATTCTGTTGTGAAC-5'

D51_TPM.filter 20374407_21 TATATCAAGTAGAGAAGGATC

target id: Solyc04g005540.2.1

1077 5'-TCGACGATCCTTCTCTACTTGATATAGCAAA-3' 1107 cleavage site: 1092

|||||||||||||||||||||

3'-CTAGGAAGAGATGAACTATAT-5'

D51_TPM.filter 15675722_20 CAAGTGTTGTCTTACCTACG

target id: Solyc04g005540.2.1

644 5'-GGTGGAGTTGGTAAGACAACACTGACTGAG-3' 673 cleavage site: 658

|| ||||||||||||||

3'-GCATCCATTCTGTTGTGAAC-5'

D51_TPM.filter 23720962_23 TCTTTCCTACTCCTCCCATACCA

target id: Solyc04g005540.2.1

632 5'-ATATGTGGTATGGGTGGAGTTGGTAAGACAACA-3' 664 cleavage site: 649

||||||||| ||||| || ||||

3'-ACCATACCCTCCTCATCCTTTCT-5'

D51_TPM.filter 12762967_21 GATGCTGGAAAAGGTAGACTG

target id: Solyc04g005540.2.1

1811 5'-TACACCAGTCTACCTTTTCCAGCATCCATTC-3' 1841 cleavage site: 1826

|||||||||||||||||||||

3'-GTCAGATGGAAAAGGTCGTAG-5'

D51_TPM.filter 23720275_22 TCTTTCCCACTCCTCCCATACT

target id: Solyc04g005540.2.1

633 5'-TATGTGGTATGGGTGGAGTTGGTAAGACAACA-3' 664 cleavage site: 649

o||||||| ||||| || ||||

3'-TCATACCCTCCTCACCCTTTCT-5'

D51_TPM.filter 06097574_21 ACAAAGCAAAAATAGGTACCT

target id: Solyc04g005540.2.1

1305 5'-AAGCTAAGTACCTCTTTTTGCTTTGTTCCTT-3' 1335 cleavage site: 1320

|||||| ||||||||||||

3'-TCCATGGATAAAAACGAAACA-5'

D51_TPM.filter 23721121_22 TCTTTCCTACTCCACCCATACC

target id: Solyc04g005540.2.1

633 5'-TATGTGGTATGGGTGGAGTTGGTAAGACAACA-3' 664 cleavage site: 649

|||||||||||||| || ||||

3'-CCATACCCACCTCATCCTTTCT-5'

D51_TPM.filter 24601758_21 TTGACACAATTTTTTTCTGAA

target id: Solyc04g005540.2.1

1484 5'-CAAGGTTCAGAAAAAAATTGTGTCAAGATGC-3' 1514 cleavage site: 1499

|||||||||||||||||||||

3'-AAGTCTTTTTTTAACACAGTT-5'

D51_TPM.filter 02157709_21 AACAAAGCAAAAATAGGTACC

target id: Solyc04g005540.2.1

1306 5'-AGCTAAGTACCTCTTTTTGCTTTGTTCCTTG-3' 1336 cleavage site: 1321

|||||| |||||||||||||

3'-CCATGGATAAAAACGAAACAA-5'

D51_TPM.filter 27044364_21 TTTTTTAGTGCTCCTGCAACT

target id: Solyc04g005540.2.1

1148 5'-ATTACAGTTGCAGGAGCACTAAAAAAGCATA-3' 1178 cleavage site: 1163

|||||||||||||||||||||

3'-TCAACGTCCTCGTGATTTTTT-5'

D51_TPM.filter 04678105_21 AGGAACAAAGCAAAAATAGGT

target id: Solyc04g005540.2.1

1309 5'-TAAGTACCTCTTTTTGCTTTGTTCCTTGTTT-3' 1339 cleavage site: 1324

|||| ||||||||||||||||

3'-TGGATAAAAACGAAACAAGGA-5'

D51_TPM.filter 06097443_21 ACAAAGCAAAAATAGGTACCC

target id: Solyc04g005540.2.1

1305 5'-AAGCTAAGTACCTCTTTTTGCTTTGTTCCTT-3' 1335 cleavage site: 1320

|||||| ||||||||||||

3'-CCCATGGATAAAAACGAAACA-5'

D51_TPM.filter 26469519_22 TTTCCAATTCCACCCATTCCCA

target id: Solyc04g005540.2.1

630 5'-GGATATGTGGTATGGGTGGAGTTGGTAAGACAAC-3' 663 cleavage site: 648

|| || |||||||||o|||| ||o

3'-AC-CCTTACCCACCTTAACC-TTT-5'

D51_TPM.filter 23721073_22 TCTTTCCTACTCTTCCCATACC

target id: Solyc04g005540.2.1

633 5'-TATGTGGTATGGGTGGAGTTGGTAAGACAACA-3' 664 cleavage site: 649

|||||||| o|||| || ||||

3'-CCATACCCTTCTCATCCTTTCT-5'

D51_TPM.filter 23682147_22 TCTTCCCTACTCCTCCCATACT

target id: Solyc04g005540.2.1

633 5'-TATGTGGTATGGGTGGAGTTGGTAAGACAACA-3' 664 cleavage site: 649

o||||||| ||||| || ||||

3'-TCATACCCTCCTCATCCCTTCT-5'

D51_TPM.filter 15676233_21 CAAGTGTTGTCTTACCTACTA

target id: Solyc04g005540.2.1

643 5'-GGGTGGAGTTGGTAAGACAACACTGACTGAG-3' 673 cleavage site: 658

||| ||||||||||||||

3'-ATCATCCATTCTGTTGTGAAC-5'

D51_TPM.filter 15676078_21 CAAGTGTTGTCTTACCTACTT

target id: Solyc04g005540.2.1

643 5'-GGGTGGAGTTGGTAAGACAACACTGACTGAG-3' 673 cleavage site: 658

o||| ||||||||||||||

3'-TTCATCCATTCTGTTGTGAAC-5'

D51_TPM.filter 02706558_21 AACTTCCATGATCTTCTGAGC

target id: Solyc04g005540.2.1

993 5'-TGGGAGCTCAGAAGATCATGGAAGTTGGAAC-3' 1023 cleavage site: 1008

|||||||||||||||||||||

3'-CGAGTCTTCTAGTACCTTCAA-5'

D51_TPM.filter 23721661_22 TCTTTCCTATTCCTCCCATACC

target id: Solyc04g005540.2.1

633 5'-TATGTGGTATGGGTGGAGTTGGTAAGACAACA-3' 664 cleavage site: 649

|||||||| |||o| || ||||

3'-CCATACCCTCCTTATCCTTTCT-5'

D51_TPM.filter 25866430_21 TTTAGCAAGAGTTGTTTTACC

target id: Solyc10g008240.2.1

506 5'-GGATTGGCAAAACGACTCTTGCTAGAAAAGT-3' 536 cleavage site: 521

|| |||||o||||||||||o|

3'-CCATTTTGTTGAGAACGATTT-5'

D51_TPM.filter 26653861_22 TTTTAGCAAGAGTTGTTTTACC

target id: Solyc10g008240.2.1

506 5'-GGATTGGCAAAACGACTCTTGCTAGAAAAGTT-3' 537 cleavage site: 522

|| |||||o||||||||||o||

3'-CCATTTTGTTGAGAACGATTTT-5'

D51_TPM.filter 25866155_21 TTTAGCAAGAGTTGTTTTACT

target id: Solyc10g008240.2.1

506 5'-GGATTGGCAAAACGACTCTTGCTAGAAAAGT-3' 536 cleavage site: 521

o| |||||o||||||||||o|

3'-TCATTTTGTTGAGAACGATTT-5'

D51_TPM.filter 26654022_22 TTTTAGCAAGAGTTGTTTTACT

target id: Solyc10g008240.2.1

506 5'-GGATTGGCAAAACGACTCTTGCTAGAAAAGTT-3' 537 cleavage site: 522

o| |||||o||||||||||o||

3'-TCATTTTGTTGAGAACGATTTT-5'

D51_TPM.filter 26653812_23 TTTTAGCAAGAGTTGTTTTACCT

target id: Solyc10g008240.2.1

505 5'-GGGATTGGCAAAACGACTCTTGCTAGAAAAGTT-3' 537 cleavage site: 522

|| |||||o||||||||||o||

3'-TCCATTTTGTTGAGAACGATTTT-5'

D51_TPM.filter 25866391_22 TTTAGCAAGAGTTGTTTTACCT

target id: Solyc10g008240.2.1

505 5'-GGGATTGGCAAAACGACTCTTGCTAGAAAAGT-3' 536 cleavage site: 521

|| |||||o||||||||||o|

3'-TCCATTTTGTTGAGAACGATTT-5'

D51_TPM.filter 26654347_23 TTTTAGCAAGAGTTGTTTTACCA

target id: Solyc10g008240.2.1

505 5'-GGGATTGGCAAAACGACTCTTGCTAGAAAAGTT-3' 537 cleavage site: 522

||| |||||o||||||||||o||

3'-ACCATTTTGTTGAGAACGATTTT-5'

D51_TPM.filter 25866163_22 TTTAGCAAGAGTTGTTTTACCC

target id: Solyc10g008240.2.1

505 5'-GGGATTGGCAAAACGACTCTTGCTAGAAAAGT-3' 536 cleavage site: 521

|| |||||o||||||||||o|

3'-CCCATTTTGTTGAGAACGATTT-5'

D51_TPM.filter 19059194_22 CTTTAGCAAGAGTTGTTTTACC

target id: Solyc10g008240.2.1

506 5'-GGATTGGCAAAACGACTCTTGCTAGAAAAGTT-3' 537 cleavage site: 522

|| |||||o||||||||||o|

3'-CCATTTTGTTGAGAACGATTTC-5'

D51_TPM.filter 26654102_21 TTTTAGCAAGAGTTGTTTTAC

target id: Solyc10g008240.2.1

507 5'-GATTGGCAAAACGACTCTTGCTAGAAAAGTT-3' 537 cleavage site: 522

|||||o||||||||||o||

3'-CATTTTGTTGAGAACGATTTT-5'

D51_TPM.filter 25866348_22 TTTAGCAAGAGTTGTTTTACCA

target id: Solyc10g008240.2.1

505 5'-GGGATTGGCAAAACGACTCTTGCTAGAAAAGT-3' 536 cleavage site: 521

||| |||||o||||||||||o|

3'-ACCATTTTGTTGAGAACGATTT-5'

D51_TPM.filter 25866736_20 TTTAGCAAGAGTTGTTTTAC

target id: Solyc10g008240.2.1

507 5'-GATTGGCAAAACGACTCTTGCTAGAAAAGT-3' 536 cleavage site: 521

|||||o||||||||||o|

3'-CATTTTGTTGAGAACGATTT-5'

D51_TPM.filter 26653934_23 TTTTAGCAAGAGTTGTTTTACCC

target id: Solyc10g008240.2.1

505 5'-GGGATTGGCAAAACGACTCTTGCTAGAAAAGTT-3' 537 cleavage site: 522

|| |||||o||||||||||o||

3'-CCCATTTTGTTGAGAACGATTTT-5'

D51_TPM.filter 06513050_24 ACATCCTTCTATCTTAAACTCTGT

target id: Solyc12g044200.1.1

1714 5'-AACCTCCGAAGTTTATGGATAGAAGGATGTTCATC-3' 1748 cleavage site: 1733

|o |||||| o|||||||||||||

3'-TGTCTCAAAT-TCTATCTTCCTACA-5'

D51_TPM.filter 12762967_21 GATGCTGGAAAAGGTAGACTG

target id: Solyc12g044200.1.1

1579 5'-GGCTCCA-TTTGTCGTTTTCCAGCATCCATTC-3' 1609 cleavage site: 1594

|| |o|oo| ||||||||||||

3'-GTCAGATGG-AAAAGGTCGTAG-5'

D51_TPM.filter 22430539_21 TCAGTACTCCAAAGATCATCT

target id: Solyc07g053010.1.1

787 5'-GTTTTGGATGATGTATGGAGTATTGATGATG-3' 817 cleavage site: 802

o|||||| | |||||||o|||

3'-TCTACTAGAAACCTCATGACT-5'

D51_TPM.filter 22430596_21 TCAGTACTCCAAAGATCATCC

target id: Solyc07g053010.1.1

787 5'-GTTTTGGATGATGTATGGAGTATTGATGATG-3' 817 cleavage site: 802

||||||| | |||||||o|||

3'-CCTACTAGAAACCTCATGACT-5'

D51_TPM.filter 09403596_22 ATGCCTGAGAGTCTTGACATTC

target id: Solyc07g053010.1.1

1670 5'-TGGCTGAA-ATCAAGACTCTCAGG-GTCTTGG-3' 1699 cleavage site: 1684

||| |||||||||||||| o|

3'-CTTACAGTTCTGAGAGTCCGTA-5'

D51_TPM.filter 23202424_21 TCCTACTACTTCTGGTTCTGG

target id: Solyc07g049700.1.1

989 5'-GTCCTCCACAACTAGAAGTAATAGGGAAGCA-3' 1019 cleavage site: 1004

||| |||o||||||| ||||o

3'-GGTCTTGGTCTTCATCATCCT-5'

D51_TPM.filter 23202273_22 TCCTACTACTTCTGGTTCTGGG

target id: Solyc07g049700.1.1

988 5'-TGTCCTCCACAACTAGAAGTAATAGGGAAGCA-3' 1019 cleavage site: 1004

o||| |||o||||||| ||||o

3'-GGGTCTTGGTCTTCATCATCCT-5'

D51_TPM.filter 23202482_22 TCCTACTACTTCTGGTTCTGGC

target id: Solyc07g049700.1.1

988 5'-TGTCCTCCACAACTAGAAGTAATAGGGAAGCA-3' 1019 cleavage site: 1004

||| |||o||||||| ||||o

3'-CGGTCTTGGTCTTCATCATCCT-5'

D51_TPM.filter 23202385_23 TCCTACTACTTCTGGTTCTGGGG

target id: Solyc07g049700.1.1

987 5'-TTGTCCTCCACAACTAGAAGTAATAGGGAAGCA-3' 1019 cleavage site: 1004

|o||| |||o||||||| ||||o

3'-GGGGTCTTGGTCTTCATCATCCT-5'

D51_TPM.filter 23202354_21 TCCTACTACTTCTGGTTCTGA

target id: Solyc07g049700.1.1

989 5'-GTCCTCCACAACTAGAAGTAATAGGGAAGCA-3' 1019 cleavage site: 1004

|| |||o||||||| ||||o

3'-AGTCTTGGTCTTCATCATCCT-5'

D51_TPM.filter 25908487_21 TTTACGTGTCGTCACAATGAT

target id: Solyc08g007630.1.1

935 5'-GTAAGATCATTGTGACGACACGTAAAGAGAG-3' 965 cleavage site: 950

|||||||||||||||||||||

3'-TAGTAACACTGCTGTGCATTT-5'

D51_TPM.filter 11530073_21 ATTTCTTGAAGTAACCCTTTC

target id: Solyc08g007630.1.1

742 5'-ATAACGAAAGGGTTACTTCAAGAAATTGGCT-3' 772 cleavage site: 757

|||||||||||||||||||||

3'-CTTTCCCAATGAAGTTCTTTA-5'

D51_TPM.filter 23249027_21 TCCTTACGTGTAGTTACAATG

target id: Solyc08g007630.1.1

937 5'-AAGATCATTGTGACGACACGTAAAGAGAGTG-3' 967 cleavage site: 952

||||||o|| |||||||| ||

3'-GTAACATTGATGTGCATTCCT-5'

D51_TPM.filter 24194266_21 TTACGTGTCGTCACAATGATC

target id: Solyc08g007630.1.1

934 5'-AGTAAGATCATTGTGACGACACGTAAAGAGA-3' 964 cleavage site: 949

|||||||||||||||||||||

3'-CTAGTAACACTGCTGTGCATT-5'

D51_TPM.filter 20141108_20 TACGTGTAGTTACAATGATT

target id: Solyc08g007630.1.1

934 5'-AGTAAGATCATTGTGACGACACGTAAAGAG-3' 963 cleavage site: 948

o||||||||o|| |||||||

3'-TTAGTAACATTGATGTGCAT-5'

D51_TPM.filter 24193966_21 TTACGTGTAGTTACAATGATT

target id: Solyc08g007630.1.1

934 5'-AGTAAGATCATTGTGACGACACGTAAAGAGA-3' 964 cleavage site: 949

o||||||||o|| ||||||||

3'-TTAGTAACATTGATGTGCATT-5'

D51_TPM.filter 23501236_21 TCTCAAGTCATCCCACTCATT

target id: Solyc08g007630.1.1

881 5'-ACTACAATGAGTGGGATGACTTGAGAAATAT-3' 911 cleavage site: 896

|||||||||||||||||||||

3'-TTACTCACCCTACTGAACTCT-5'

D51_TPM.filter 25523143_21 TTCCGTGTAGTTACAATGATT

target id: Solyc08g007630.1.1

934 5'-AGTAAGATCATTGTGACGACACGTAAAGAGA-3' 964 cleavage site: 949

o||||||||o|| ||||| ||

3'-TTAGTAACATTGATGTGCCTT-5'

D51_TPM.filter 20140807_21 TACGTGTAGTTACAATGATCT

target id: Solyc08g007630.1.1

933 5'-AAGTAAGATCATTGTGACGACACGTAAAGAG-3' 963 cleavage site: 948

||||||||||o|| |||||||

3'-TCTAGTAACATTGATGTGCAT-5'

D51_TPM.filter 06217752_21 ACAATGATCTTACTTCCTATA

target id: Solyc08g007630.1.1

922 5'-GGAGATATAGGAAGTAAGATCATTGTGACGA-3' 952 cleavage site: 937

|||||||||||||||||||||

3'-ATATCCTTCATTCTAGTAACA-5'

D51_TPM.filter 03821356_21 AATTTCTTGAAGTAACCCTTT

target id: Solyc08g007630.1.1

743 5'-TAACGAAAGGGTTACTTCAAGAAATTGGCTC-3' 773 cleavage site: 758

|||||||||||||||||||||

3'-TTTCCCAATGAAGTTCTTTAA-5'

D51_TPM.filter 25908337_21 TTTACGTGTCGTCACAATGAC

target id: Solyc08g007630.1.1

935 5'-GTAAGATCATTGTGACGACACGTAAAGAGAG-3' 965 cleavage site: 950

||||||||||||||||||||

3'-CAGTAACACTGCTGTGCATTT-5'

D51_TPM.filter 24193570_21 TTACGTGTAGTTACAATGATC

target id: Solyc08g007630.1.1

934 5'-AGTAAGATCATTGTGACGACACGTAAAGAGA-3' 964 cleavage site: 949

|||||||||o|| ||||||||

3'-CTAGTAACATTGATGTGCATT-5'

D51_TPM.filter 16180747_22 CACTTTCTGTAGTTGTCCTTCT

target id: Solyc08g007630.1.1

867 5'-CGTGTGGAATGACAACTACA-ATGAGTGGGATG-3' 898 cleavage site: 883

o||| |||||||||| | o||||

3'-TCTTCCTGTTGATGTCT-TTCAC-5'

D51_TPM.filter 17440615_22 CCACTTTCTGTAGTTGTCCTTC

target id: Solyc08g007630.1.1

868 5'-GTGTGGAATGACAACTACA-ATGAGTGGGATGA-3' 899 cleavage site: 884

||| |||||||||| | o|||||

3'-CTTCCTGTTGATGTCT-TTCACC-5'

D51_TPM.filter 21734213_21 TGCGGGAAGATCATTGTAGCT

target id: Solyc08g007630.1.1

1250 5'-TGTTGAGCTACAATGATCTTCCCGCACATTT-3' 1280 cleavage site: 1265

|||||||||||||||||||||

3'-TCGATGTTACTAGAAGGGCGT-5'

D51_TPM.filter 25523171_21 TTCCGTGTAGTTACAATGATC

target id: Solyc08g007630.1.1

934 5'-AGTAAGATCATTGTGACGACACGTAAAGAGA-3' 964 cleavage site: 949

|||||||||o|| ||||| ||

3'-CTAGTAACATTGATGTGCCTT-5'

D51_TPM.filter 24194001_21 TTACGTGTAGTTACGATGATT

target id: Solyc08g007630.1.1

934 5'-AGTAAGATCATTGTGACGACACGTAAAGAGA-3' 964 cleavage site: 949

o|||||o||o|| ||||||||

3'-TTAGTAGCATTGATGTGCATT-5'

D51_TPM.filter 24193897_21 TTACGTGTCGTCACAATGATT

target id: Solyc08g007630.1.1

934 5'-AGTAAGATCATTGTGACGACACGTAAAGAGA-3' 964 cleavage site: 949

o||||||||||||||||||||

3'-TTAGTAACACTGCTGTGCATT-5'

D51_TPM.filter 26210487_21 TTTGCAGCGTAAAATACCAGC

target id: Solyc08g007630.1.1

1145 5'-CGCTCGCTGGCATGTTACGCTCCAAATCAGA-3' 1175 cleavage site: 1160

||||| || ||||||| ||||

3'-CGACCATAAAATGCGACGTTT-5'

D51_TPM.filter 24194358_22 TTACGTGTAGTTACAATGATCT

target id: Solyc08g007630.1.1

933 5'-AAGTAAGATCATTGTGACGACACGTAAAGAGA-3' 964 cleavage site: 949

||||||||||o|| ||||||||

3'-TCTAGTAACATTGATGTGCATT-5'

D51_TPM.filter 21688701_21 TGCAGGAAGATCATTGTAGCT

target id: Solyc08g007630.1.1

1250 5'-TGTTGAGCTACAATGATCTTCCCGCACATTT-3' 1280 cleavage site: 1265

||||||||||||||||| |||

3'-TCGATGTTACTAGAAGGACGT-5'

D51_TPM.filter 20140474_20 TACGTGTAGTTACAATGATG

target id: Solyc08g007630.1.1

934 5'-AGTAAGATCATTGTGACGACACGTAAAGAG-3' 963 cleavage site: 948

||||||||o|| |||||||

3'-GTAGTAACATTGATGTGCAT-5'

D51_TPM.filter 09531475_21 ATGTGCGGGAAGATCATTGTA

target id: Solyc08g007630.1.1

1253 5'-TGAGCTACAATGATCTTCCCGCACATTTAAA-3' 1283 cleavage site: 1268

|||||||||||||||||||||

3'-ATGTTACTAGAAGGGCGTGTA-5'

D51_TPM.filter 24189463_21 TTACGTATTGTTACAATGATT

target id: Solyc08g007630.1.1

934 5'-AGTAAGATCATTGTGACGACACGTAAAGAGA-3' 964 cleavage site: 949

o||||||||o||o| ||||||

3'-TTAGTAACATTGTTATGCATT-5'

D51_TPM.filter 25648313_21 TTCTCAAGTCATCCCACTCAT

target id: Solyc08g007630.1.1

882 5'-CTACAATGAGTGGGATGACTTGAGAAATATT-3' 912 cleavage site: 897

|||||||||||||||||||||

3'-TACTCACCCTACTGAACTCTT-5'

D51_TPM.filter 24193976_21 TTACGTGTAGTTACAATGATG

target id: Solyc08g007630.1.1

934 5'-AGTAAGATCATTGTGACGACACGTAAAGAGA-3' 964 cleavage site: 949

||||||||o|| ||||||||

3'-GTAGTAACATTGATGTGCATT-5'

D51_TPM.filter 20140227_20 TACGTGTAGTTACAATGATA

target id: Solyc08g007630.1.1

934 5'-AGTAAGATCATTGTGACGACACGTAAAGAG-3' 963 cleavage site: 948

||||||||o|| |||||||

3'-ATAGTAACATTGATGTGCAT-5'

D51_TPM.filter 20141079_21 TACGTGTAGTTACAATGATCC

target id: Solyc08g007630.1.1

933 5'-AAGTAAGATCATTGTGACGACACGTAAAGAG-3' 963 cleavage site: 948

|||||||||o|| |||||||

3'-CCTAGTAACATTGATGTGCAT-5'

D51_TPM.filter 18799966_21 CTTACGTGTCGTCACAATGAT

target id: Solyc08g007630.1.1

935 5'-GTAAGATCATTGTGACGACACGTAAAGAGAG-3' 965 cleavage site: 950

||||||||||||||||||||

3'-TAGTAACACTGCTGTGCATTC-5'

D51_TPM.filter 25909349_21 TTTACGTGTCGTCACAATGAA

target id: Solyc08g007630.1.1

935 5'-GTAAGATCATTGTGACGACACGTAAAGAGAG-3' 965 cleavage site: 950

||||||||||||||||||||

3'-AAGTAACACTGCTGTGCATTT-5'

D51_TPM.filter 24193674_21 TTACGTGTAGTTACGATGATC

target id: Solyc08g007630.1.1

934 5'-AGTAAGATCATTGTGACGACACGTAAAGAGA-3' 964 cleavage site: 949

||||||o||o|| ||||||||

3'-CTAGTAGCATTGATGTGCATT-5'

D51_TPM.filter 26219011_21 TTTGCGTGTCGTCACAATGAT

target id: Solyc08g007630.1.1

935 5'-GTAAGATCATTGTGACGACACGTAAAGAGAG-3' 965 cleavage site: 950

|||||||||||||||||o|||

3'-TAGTAACACTGCTGTGCGTTT-5'

D51_TPM.filter 20132125_20 TACGTATTGTTACAATGATT

target id: Solyc08g007630.1.1

934 5'-AGTAAGATCATTGTGACGACACGTAAAGAG-3' 963 cleavage site: 948

o||||||||o||o| |||||

3'-TTAGTAACATTGTTATGCAT-5'

D51_TPM.filter 23561099_21 TCTCCTTACGTGTAGTTACAA

target id: Solyc08g007630.1.1

939 5'-GATCATTGTGACGACACGTAAAGAGAGTGTT-3' 969 cleavage site: 954

||||o|| |||||||| ||||

3'-AACATTGATGTGCATTCCTCT-5'

D51_TPM.filter 25909254_21 TTTACGTGTCGTCACAATGAG

target id: Solyc08g007630.1.1

935 5'-GTAAGATCATTGTGACGACACGTAAAGAGAG-3' 965 cleavage site: 950

||||||||||||||||||||

3'-GAGTAACACTGCTGTGCATTT-5'

D51_TPM.filter 25908113_21 TTTACGTGTCGTCACAATGGT

target id: Solyc08g007630.1.1

935 5'-GTAAGATCATTGTGACGACACGTAAAGAGAG-3' 965 cleavage site: 950

|o|||||||||||||||||||

3'-TGGTAACACTGCTGTGCATTT-5'

D51_TPM.filter 03821873_21 AATTTCTTGAAGTAACCCTTC

target id: Solyc08g007630.1.1

743 5'-TAACGAAAGGGTTACTTCAAGAAATTGGCTC-3' 773 cleavage site: 758

||||||||||||||||||||

3'-CTTCCCAATGAAGTTCTTTAA-5'

D51_TPM.filter 23249093_21 TCCTTACGTGTAGTTACAATA

target id: Solyc08g007630.1.1

937 5'-AAGATCATTGTGACGACACGTAAAGAGAGTG-3' 967 cleavage site: 952

|||||o|| |||||||| ||

3'-ATAACATTGATGTGCATTCCT-5'

D51_TPM.filter 11530192_21 ATTTCTTGAAGTAACCCTTTT

target id: Solyc08g007630.1.1

742 5'-ATAACGAAAGGGTTACTTCAAGAAATTGGCT-3' 772 cleavage site: 757

o||||||||||||||||||||

3'-TTTTCCCAATGAAGTTCTTTA-5'

D51_TPM.filter 23248970_22 TCCTTACGTGTAGTTACAATGA

target id: Solyc08g007630.1.1

936 5'-TAAGATCATTGTGACGACACGTAAAGAGAGTG-3' 967 cleavage site: 952

|||||||o|| |||||||| ||

3'-AGTAACATTGATGTGCATTCCT-5'

D51_TPM.filter 20140343_20 TACGTGTAGTTACGATGATT

target id: Solyc08g007630.1.1

934 5'-AGTAAGATCATTGTGACGACACGTAAAGAG-3' 963 cleavage site: 948

o|||||o||o|| |||||||

3'-TTAGTAGCATTGATGTGCAT-5'

D51_TPM.filter 23501427_21 TCTCAAGTCATCCCACTCATC

target id: Solyc08g007630.1.1

881 5'-ACTACAATGAGTGGGATGACTTGAGAAATAT-3' 911 cleavage site: 896

||||||||||||||||||||

3'-CTACTCACCCTACTGAACTCT-5'

D51_TPM.filter 20140844_20 TACGTGTAGTTACAATGATC

target id: Solyc08g007630.1.1

934 5'-AGTAAGATCATTGTGACGACACGTAAAGAG-3' 963 cleavage site: 948

|||||||||o|| |||||||

3'-CTAGTAACATTGATGTGCAT-5'

D51_TPM.filter 24321395_21 TTATGGAACACCCTCTTGGTT

target id: Solyc08g007630.1.1

1708 5'-CCTCTAAGCAAGAGGGTGTTGCATAACATAC-3' 1738 cleavage site: 1723

|| |||||||||||| |||||

3'-TTGGTTCTCCCACAAGGTATT-5'

D51_TPM.filter 24193509_21 TTACGTGTAGTTACAATGATA

target id: Solyc08g007630.1.1

934 5'-AGTAAGATCATTGTGACGACACGTAAAGAGA-3' 964 cleavage site: 949

||||||||o|| ||||||||

3'-ATAGTAACATTGATGTGCATT-5'

D51_TPM.filter 01607326_22 AAGATCATGTGGAACACTTGTG

target id: Solyc02g027080.1.1

1334 5'-AAGCTGACAAGTG-TTTACAAGATCTTATCAA-3' 1364 cleavage site: 1349

||||||| |oo||| ||||||

3'-GTGTTCACAAGGTGTACTAGAA-5'

D51_TPM.filter 11357638_22 ATTTGACGGATACTGCCATGCT

target id: Solyc09g064610.2.1

1115 5'-CATCAAGCA--GCAATATCCGTCAAGTACAAG-3' 1144 cleavage site: 1129

|||| ||| ||||||||||o|

3'-TCGTACCGTCATAGGCAGTTTA-5'

D51_TPM.filter 06339668_24 ACACAGTCTTGAATGAATAGCTCA

target id: Solyc09g064610.2.1

2216 5'-CTCATGGAGTTATTCATTCAGGATTGTGAGATGC-3' 2249 cleavage site: 2234

|||o||||||||||o||o||||

3'-ACTCGATAAGTAAGTTCTGACACA-5'

D51_TPM.filter 20716693_22 TATTTGACGGATACTGCCATGC

target id: Solyc09g064610.2.1

1116 5'-ATCAAGCA--GCAATATCCGTCAAGTACAAGT-3' 1145 cleavage site: 1130

||| ||| ||||||||||o||

3'-CGTACCGTCATAGGCAGTTTAT-5'

D51_TPM.filter 20717144_22 TATTTGACGGATACTGCCATGT

target id: Solyc09g064610.2.1

1116 5'-ATCAAGCA--GCAATATCCGTCAAGTACAAGT-3' 1145 cleavage site: 1130

o|| ||| ||||||||||o||

3'-TGTACCGTCATAGGCAGTTTAT-5'

D51_TPM.filter 10920819_22 ATTGTCTTGAATATGAATTACA

target id: Solyc09g064610.2.1

1729 5'-GGGTCTGTAGATTATATTGAAGACAATGTTCA-3' 1760 cleavage site: 1745

||||o |o||||| ||||||||

3'-ACATTAAGTATAAGTTCTGTTA-5'

D51_TPM.filter 14258948_21 GCTCAGCGAGGACGGAAACCT

target id: Solyc09g064610.2.1

1766 5'-GTAGAAGGTTTCCATCCTTGC-GATGCCTTGT-3' 1796 cleavage site: 1781

|||||||| ||||o|| || ||

3'-TCCAAAGGCAGGAGCGACT-CG-5'

D51_TPM.filter 08107654_22 ATAGAGACGATATTTTTGATAG

target id: Solyc09g064610.2.1

1620 5'-TTCAGTT-TTGAAAAATGTTGTCTCTATTGATG-3' 1651 cleavage site: 1636

o| |o ||||||o|o||||||||

3'-GATAG-TTTTTATAGCAGAGATA-5'

D51_TPM.filter 24692840_21 TTGATCTGTGAATGTCTTTGC

target id: Solyc09g064610.2.1

1935 5'-TTGAGGCAAAGACAGATCACAGATGCAACGACT-3' 1967 cleavage site: 1952

|||||||| o |||||||| |||

3'-CGTTTCTG-TAAGTGTCTA-GTT-5'

D51_TPM.filter 25668830_22 TTCTCTGAGGACATAGATTCTC

target id: Solyc10g047320.1.1

158 5'-TCAACGAGAA-CGAAGTCCTCAGAGAACTTGT-3' 188 cleavage site: 173

||||| | | ||||||||||||

3'-CTCTTAGATACAGGAGTCTCTT-5'

D51_TPM.filter 11201972_22 ATTCTTTCGTATAGCTCTTGTC

target id: Solyc10g047320.1.1

325 5'-GGTGAGATCAA-AGCTATACGAAACAAAGTCAG-3' 356 cleavage site: 341

|| ||| |||||||||||| ||

3'-CT-GTTCTCGATATGCTTTCTTA-5'

D51_TPM.filter 25187438_21 TTGTTTATGTTTGATTGCTTT

target id: Solyc10g047320.1.1

2582 5'-CCAAAAAAGACAATCAAAC-TCAACAAGGGAC-3' 2612 cleavage site: 2597

|||| ||||||||| | |||||

3'-TTTC-GTTAGTTTGTATTTGTT-5'

D51_TPM.filter 01818007_24 AAGGTATTCTAGTCATTTTGGATT

target id: Solyc10g047320.1.1

2123 5'-TAAAAAATGTGAAGAGGCTAGAATACCTTGAAAA-3' 2156 cleavage site: 2141

||| oo||o |o||||||||||||

3'-TTAGGTTTTACTGATCTTATGGAA-5'

D51_TPM.filter 21221178_20 TGATTAACAGGGACAGTCGA

target id: Solyc10g047320.1.1

1934 5'-CTAAGTTGCCTGTCCCTGTTGCTCCAAAAA-3' 1963 cleavage site: 1948

|o| |||||||||||o ||

3'-AGCTGACAGGGACAATTAGT-5'

D51_TPM.filter 25668411_22 TTCTCTGAGGACATAGATTCTA

target id: Solyc10g047320.1.1

158 5'-TCAACGAGAA-CGAAGTCCTCAGAGAACTTGT-3' 188 cleavage site: 173

|||| | | ||||||||||||

3'-ATCTTAGATACAGGAGTCTCTT-5'

D51_TPM.filter 25668817_22 TTCTCTGAGGACATAGATTCTT

target id: Solyc10g047320.1.1

158 5'-TCAACGAGAA-CGAAGTCCTCAGAGAACTTGT-3' 188 cleavage site: 173

o|||| | | ||||||||||||

3'-TTCTTAGATACAGGAGTCTCTT-5'

D51_TPM.filter 26469194_22 TTTCCAATTCCACCCATTCCTA

target id: Solyc01g087200.2.1

510 5'-AATTACAGGTATGGGTGGGATTGGCAAAACGAC-3' 542 cleavage site: 527

||| ||||||||o||||| |||

3'-ATCCTTACCCACCTTAACC-TTT-5'

D51_TPM.filter 26653861_22 TTTTAGCAAGAGTTGTTTTACC

target id: Solyc01g087200.2.1

527 5'-GGATTGGCAAAACGACTCTCGCTAAAAAAGCT-3' 558 cleavage site: 543

|| |||||o||||| |||||||

3'-CCATTTTGTTGAGAACGATTTT-5'

D51_TPM.filter 26654022_22 TTTTAGCAAGAGTTGTTTTACT

target id: Solyc01g087200.2.1

527 5'-GGATTGGCAAAACGACTCTCGCTAAAAAAGCT-3' 558 cleavage site: 543

o| |||||o||||| |||||||

3'-TCATTTTGTTGAGAACGATTTT-5'

D51_TPM.filter 26469238_21 TTTCCAATTCCACCCATTCCT

target id: Solyc01g087200.2.1

511 5'-ATTACAGGTATGGGTGGGATTGGCAAAACGAC-3' 542 cleavage site: 527

||| ||||||||o||||| |||

3'-TCCTTACCCACCTTAACC-TTT-5'

D51_TPM.filter 22430539_21 TCAGTACTCCAAAGATCATCT

target id: Solyc01g087200.2.1

757 5'-GTCATTGATGATATTTGGAGTACTAAAGTTT-3' 787 cleavage site: 772

|||||| |||||||||||

3'-TCTACTAGAAACCTCATGACT-5'

D51_TPM.filter 26653812_23 TTTTAGCAAGAGTTGTTTTACCT

target id: Solyc01g087200.2.1

526 5'-GGGATTGGCAAAACGACTCTCGCTAAAAAAGCT-3' 558 cleavage site: 543

|| |||||o||||| |||||||

3'-TCCATTTTGTTGAGAACGATTTT-5'

D51_TPM.filter 26469771_21 TTTCCAATTCCACCCATTCCC

target id: Solyc01g087200.2.1

511 5'-ATTACAGGTATGGGTGGGATTGGCAAAACGAC-3' 542 cleavage site: 527

|| ||||||||o||||| |||

3'-CCCTTACCCACCTTAACC-TTT-5'

D51_TPM.filter 26469737_22 TTTCCAATTCCACCCATTCCTG

target id: Solyc01g087200.2.1

510 5'-AATTACAGGTATGGGTGGGATTGGCAAAACGAC-3' 542 cleavage site: 527

|||| ||||||||o||||| |||

3'-GTCCTTACCCACCTTAACC-TTT-5'

D51_TPM.filter 14693136_21 GTGACAATAACAATAGGGATT

target id: Solyc01g087200.2.1

2672 5'-TGCTAAGTTTC--TTGTTATTGTTACTTTAA-3' 2700 cleavage site: 2685

|o|oo| ||||||||||o||

3'-TTAGGGATAACAATAACAGTG-5'

D51_TPM.filter 26469466_21 TTTCCAATTCCACCCATTCCA

target id: Solyc01g087200.2.1

511 5'-ATTACAGGTATGGGTGGGATTGGCAAAACGAC-3' 542 cleavage site: 527

|| ||||||||o||||| |||

3'-ACCTTACCCACCTTAACC-TTT-5'

D51_TPM.filter 15973909_21 CAGTACTCCAAAGATCATCTA

target id: Solyc01g087200.2.1

756 5'-TGTCATTGATGATATTTGGAGTACTAAAGTT-3' 786 cleavage site: 771

|||||| |||||||||||

3'-ATCTACTAGAAACCTCATGAC-5'

D51_TPM.filter 26654347_23 TTTTAGCAAGAGTTGTTTTACCA

target id: Solyc01g087200.2.1

526 5'-GGGATTGGCAAAACGACTCTCGCTAAAAAAGCT-3' 558 cleavage site: 543

||| |||||o||||| |||||||

3'-ACCATTTTGTTGAGAACGATTTT-5'

D51_TPM.filter 26654102_21 TTTTAGCAAGAGTTGTTTTAC

target id: Solyc01g087200.2.1

528 5'-GATTGGCAAAACGACTCTCGCTAAAAAAGCT-3' 558 cleavage site: 543

|||||o||||| |||||||

3'-CATTTTGTTGAGAACGATTTT-5'

D51_TPM.filter 22430596_21 TCAGTACTCCAAAGATCATCC

target id: Solyc01g087200.2.1

757 5'-GTCATTGATGATATTTGGAGTACTAAAGTTT-3' 787 cleavage site: 772

|||||| |||||||||||

3'-CCTACTAGAAACCTCATGACT-5'

D51_TPM.filter 26653934_23 TTTTAGCAAGAGTTGTTTTACCC

target id: Solyc01g087200.2.1

526 5'-GGGATTGGCAAAACGACTCTCGCTAAAAAAGCT-3' 558 cleavage site: 543

|| |||||o||||| |||||||

3'-CCCATTTTGTTGAGAACGATTTT-5'

D51_TPM.filter 25866430_21 TTTAGCAAGAGTTGTTTTACC

target id: Solyc11g006520.1.1

521 5'-GTATCGGTAAGACAACTCTTGCCAAAAAAGT-3' 551 cleavage site: 536

|||||o||||||||||| |||

3'-CCATTTTGTTGAGAACGATTT-5'

D51_TPM.filter 26653861_22 TTTTAGCAAGAGTTGTTTTACC

target id: Solyc11g006520.1.1

521 5'-GTATCGGTAAGACAACTCTTGCCAAAAAAGTT-3' 552 cleavage site: 537

|||||o||||||||||| ||||

3'-CCATTTTGTTGAGAACGATTTT-5'

D51_TPM.filter 25694497_22 TTCTTGGCTAGAGTTGTATTGC

target id: Solyc11g006520.1.1

522 5'-TATCGGTAAGACAACTCTTGCCAA-AAAAGTT-3' 552 cleavage site: 537

|o|| |||||||| ||||| ||

3'-CGTTATGTTGAGATCGGTTCTT-5'

D51_TPM.filter 25866155_21 TTTAGCAAGAGTTGTTTTACT

target id: Solyc11g006520.1.1

521 5'-GTATCGGTAAGACAACTCTTGCCAAAAAAGT-3' 551 cleavage site: 536

o||||o||||||||||| |||

3'-TCATTTTGTTGAGAACGATTT-5'

D51_TPM.filter 26654022_22 TTTTAGCAAGAGTTGTTTTACT

target id: Solyc11g006520.1.1

521 5'-GTATCGGTAAGACAACTCTTGCCAAAAAAGTT-3' 552 cleavage site: 537

o||||o||||||||||| ||||

3'-TCATTTTGTTGAGAACGATTTT-5'

D51_TPM.filter 25694767_22 TTCTTGGCTAGAGTTGTATTGT

target id: Solyc11g006520.1.1

522 5'-TATCGGTAAGACAACTCTTGCCAA-AAAAGTT-3' 552 cleavage site: 537

oo|| |||||||| ||||| ||

3'-TGTTATGTTGAGATCGGTTCTT-5'

D51_TPM.filter 26653812_23 TTTTAGCAAGAGTTGTTTTACCT

target id: Solyc11g006520.1.1

520 5'-GGTATCGGTAAGACAACTCTTGCCAAAAAAGTT-3' 552 cleavage site: 537

|||||o||||||||||| ||||

3'-TCCATTTTGTTGAGAACGATTTT-5'

D51_TPM.filter 26654357_22 TTTTAGCAAGAGTTGTTTTACA

target id: Solyc11g006520.1.1

521 5'-GTATCGGTAAGACAACTCTTGCCAAAAAAGTT-3' 552 cleavage site: 537

||||o||||||||||| ||||

3'-ACATTTTGTTGAGAACGATTTT-5'

D51_TPM.filter 25694504_20 TTCTTGGCTAGAGTTGTATT

target id: Solyc11g006520.1.1

524 5'-TCGGTAAGACAACTCTTGCCAA-AAAAGTT-3' 552 cleavage site: 537

|| |||||||| ||||| ||

3'-TTATGTTGAGATCGGTTCTT-5'

D51_TPM.filter 25866253_21 TTTAGCAAGAGTTGTTTTACA

target id: Solyc11g006520.1.1

521 5'-GTATCGGTAAGACAACTCTTGCCAAAAAAGT-3' 551 cleavage site: 536

||||o||||||||||| |||

3'-ACATTTTGTTGAGAACGATTT-5'

D51_TPM.filter 25866391_22 TTTAGCAAGAGTTGTTTTACCT

target id: Solyc11g006520.1.1

520 5'-GGTATCGGTAAGACAACTCTTGCCAAAAAAGT-3' 551 cleavage site: 536

|||||o||||||||||| |||

3'-TCCATTTTGTTGAGAACGATTT-5'

D51_TPM.filter 26653543_24 TTTTAGCAAGAGTTGTTTTACTTT

target id: Solyc11g006520.1.1

519 5'-TGGTATCGGTAAGACAACTCTTGCCAAAAAAGTT-3' 552 cleavage site: 537

o||||o||||||||||| ||||

3'-TTTCATTTTGTTGAGAACGATTTT-5'

D51_TPM.filter 25866686_23 TTTAGCAAGAGTTGTTTTACTTT

target id: Solyc11g006520.1.1

519 5'-TGGTATCGGTAAGACAACTCTTGCCAAAAAAGT-3' 551 cleavage site: 536

o||||o||||||||||| |||

3'-TTTCATTTTGTTGAGAACGATTT-5'

D51_TPM.filter 25866557_21 TTTAGCAAGAGTTGTTTTACG

target id: Solyc11g006520.1.1

521 5'-GTATCGGTAAGACAACTCTTGCCAAAAAAGT-3' 551 cleavage site: 536

||||o||||||||||| |||

3'-GCATTTTGTTGAGAACGATTT-5'

D51_TPM.filter 26654347_23 TTTTAGCAAGAGTTGTTTTACCA

target id: Solyc11g006520.1.1

520 5'-GGTATCGGTAAGACAACTCTTGCCAAAAAAGTT-3' 552 cleavage site: 537

|||||o||||||||||| ||||

3'-ACCATTTTGTTGAGAACGATTTT-5'

D51_TPM.filter 06335281_24 ACACAATTTGTAGAATTTCAACTT

target id: Solyc11g006520.1.1

2213 5'-TCCGCAAATTGAAATATCTACAAATTGTTTACATA-3' 2247 cleavage site: 2232

|| ||||||| ||||||||||||

3'-TTCAACTTTA-AGATGTTTAACACA-5'

D51_TPM.filter 25866163_22 TTTAGCAAGAGTTGTTTTACCC

target id: Solyc11g006520.1.1

520 5'-GGTATCGGTAAGACAACTCTTGCCAAAAAAGT-3' 551 cleavage site: 536

|||||o||||||||||| |||

3'-CCCATTTTGTTGAGAACGATTT-5'

D51_TPM.filter 19059194_22 CTTTAGCAAGAGTTGTTTTACC

target id: Solyc11g006520.1.1

521 5'-GTATCGGTAAGACAACTCTTGCCAAAAAAGTT-3' 552 cleavage site: 537

|||||o||||||||||| |||

3'-CCATTTTGTTGAGAACGATTTC-5'

D51_TPM.filter 26654102_21 TTTTAGCAAGAGTTGTTTTAC

target id: Solyc11g006520.1.1

522 5'-TATCGGTAAGACAACTCTTGCCAAAAAAGTT-3' 552 cleavage site: 537

||||o||||||||||| ||||

3'-CATTTTGTTGAGAACGATTTT-5'

D51_TPM.filter 25374791_21 TTCGATACACGAATCTCCATA

target id: Solyc11g006520.1.1

691 5'-GAACTAATGGAGA-TTGTGTATAGAGGTCTG-3' 720 cleavage site: 705

||||||| |o|||||| ||o

3'-ATACCTCTAAGCACATAGCTT-5'

D51_TPM.filter 25866348_22 TTTAGCAAGAGTTGTTTTACCA

target id: Solyc11g006520.1.1

520 5'-GGTATCGGTAAGACAACTCTTGCCAAAAAAGT-3' 551 cleavage site: 536

|||||o||||||||||| |||

3'-ACCATTTTGTTGAGAACGATTT-5'

D51_TPM.filter 25039268_21 TTGTATTCTTCAGAGATAGTG

target id: Solyc11g006520.1.1

595 5'-TGGGTCACTATCTCTGAAGAATACAATGAGA-3' 625 cleavage site: 610

|||||||||||||||||||||

3'-GTGATAGAGACTTCTTATGTT-5'

D51_TPM.filter 25866736_20 TTTAGCAAGAGTTGTTTTAC

target id: Solyc11g006520.1.1

522 5'-TATCGGTAAGACAACTCTTGCCAAAAAAGT-3' 551 cleavage site: 536

||||o||||||||||| |||

3'-CATTTTGTTGAGAACGATTT-5'

D51_TPM.filter 06200968_24 ACAACTCGTAGATATTTTAATTTG

target id: Solyc11g006520.1.1

2212 5'-TTCCGCAAATTGAAATATCTACAAATTGTTTACA-3' 2245 cleavage site: 2230

||||||o|||||||||| | ||||

3'-GTTTAATTTTATAGATGCTCAACA-5'

D51_TPM.filter 26653916_22 TTTTAGCAAGAGTTGTTTTACG

target id: Solyc11g006520.1.1

521 5'-GTATCGGTAAGACAACTCTTGCCAAAAAAGTT-3' 552 cleavage site: 537

||||o||||||||||| ||||

3'-GCATTTTGTTGAGAACGATTTT-5'

D51_TPM.filter 26653934_23 TTTTAGCAAGAGTTGTTTTACCC

target id: Solyc11g006520.1.1

520 5'-GGTATCGGTAAGACAACTCTTGCCAAAAAAGTT-3' 552 cleavage site: 537

|||||o||||||||||| ||||

3'-CCCATTTTGTTGAGAACGATTTT-5'

D51_TPM.filter 16558487_22 CGAGTCAACGGTGATCTTACGA

target id: Solyc12g005970.1.1

168 5'-TCAAGTTG-AAGA-TATTGTTGACTCGAAGCT-3' 197 cleavage site: 182

|o| |||| o|oo|||||||||

3'-AGCATTCTAGTGGCAACTGAGC-5'

D51_TPM.filter 05535911_21 AGTAAAGATATTGTAGACTAT

target id: Solyc07g053020.1.1

2301 5'-GTTAAATGGACTACAATATCTTTGTATCGAA-3' 2331 cleavage site: 2316

||o| |||||||||||||oo

3'-TATCAGATGTTATAGAAATGA-5'

D51_TPM.filter 01755447_24 AAGGGCGATTGAATTATGTTAACT

target id: Solyc07g053020.1.1

1878 5'-AGAACAGCT-ACATAGATCAATCGCCCTTCTTCC-3' 1910 cleavage site: 1895

|| | |||||o ||||||||||||

3'-TCAATTGTATTAAGTTAGCGGGAA-5'

D51_TPM.filter 21252343_21 TGATTTCTGCCCAGTGCTCTG

target id: Solyc07g053020.1.1

2427 5'-TCTAGAAGAGACA-TGGGAAGAAATCACAAGT-3' 2457 cleavage site: 2442

|||| || |||| ||||||||

3'-GTCTC-GTGACCCGTCTTTAGT-5'

D51_TPM.filter 21252993_20 TGATTTCTGCCCAGTGCTCT

target id: Solyc07g053020.1.1

294 5'-TGGAAAGA-AGTT-GGCAGAAATCAACAGG-3' 321 cleavage site: 306

||| oo| |||||||||||

3'-TCTCGTGACCCGTCTTTAGT-5'

D51_TPM.filter 12943369_20 GATTTCTGCCCAGTGCTCTG

target id: Solyc07g053020.1.1

2427 5'-TCTAGAAGAGACA-TGGGAAGAAATCACAAG-3' 2456 cleavage site: 2441

|||| || |||| |||||||

3'-GTCTC-GTGACCCGTCTTTAG-5'

D51_TPM.filter 09512444_20 ATGTGATTTCTGCCCAGTGC

target id: Solyc07g053020.1.1

2432 5'-AAGAGACA-TGGGAAGAAATCACAAGTGCA-3' 2460 cleavage site: 2445

|| |||| ||||||||||

3'-CGTGACCCGTCTTTAGTGTA-5'

D51_TPM.filter 09512184_23 ATGTGATTTCTGCCCAGTGCTCT

target id: Solyc07g053020.1.1

2428 5'-CTAGAAGAGACA-TGGGAAGAAATCACAAGTGCA-3' 2460 cleavage site: 2445

|||| || |||| ||||||||||

3'-TCTC-GTGACCCGTCTTTAGTGTA-5'

D51_TPM.filter 09513361_21 ATGTGATTTCTGCCCAGTGCT

target id: Solyc07g053020.1.1

2430 5'-AGAAGAGACA-TGGGAAGAAATCACAAGTGCA-3' 2460 cleavage site: 2445

|| || |||| ||||||||||

3'-TC-GTGACCCGTCTTTAGTGTA-5'

D51_TPM.filter 20703731_21 TATTTAAGGATTTAGGAGATT

target id: Solyc07g053020.1.1

2284 5'-TGGATAG-CTCCTAAATTGTTAAATGGACTA-3' 2313 cleavage site: 2298

|o |||||||||o ||||||o

3'-TTAGAGGATTTAGGAATTTAT-5'

D51_TPM.filter 10902796_21 ATTGTGTTGTACTCTTGAGAG

target id: Solyc04g009250.1.1

667 5'-TGTGTTTCTCAAGAGTACAACACAATGGATC-3' 697 cleavage site: 682

o||||||||||||||||||||

3'-GAGAGTTCTCATGTTGTGTTA-5'

D51_TPM.filter 13452591_21 GGCCCATTAGCTCAGTTGGTT

target id: Solyc04g009250.1.1

1872 5'-CAGCGAAACAACTGAGCTAATAGATCTAAGA-3' 1902 cleavage site: 1887

|| ||||||||||||| | o|

3'-TTGGTTGACTCGATTACCCGG-5'

D51_TPM.filter 08160426_22 ATAGATTTCTCTGTTCATACTG

target id: Solyc03g005660.2.1

268 5'-TGGAAGAGT-TGAACAGAGCAATCAATAAAGC-3' 298 cleavage site: 283

||| ||||||||| |||| ||

3'-GTCATACTTGTCTCTTTAGATA-5'

D51_TPM.filter 20318547_22 TATAGATTTCTCTGTTCATACT

target id: Solyc03g005660.2.1

269 5'-GGAAGAGT-TGAACAGAGCAATCAATAAAGCT-3' 299 cleavage site: 284

||| ||||||||| |||| |||

3'-TCATACTTGTCTCTTTAGATAT-5'

D51_TPM.filter 08852194_20 ATATTTGATTGTTCTGTTCA

target id: Solyc03g005660.2.1

272 5'-AGAGTTGAACAGAGCAATC-AATAAAGCTG-3' 300 cleavage site: 285

||||||||o||||| ||||

3'-ACTTGTCTTGTTAGTTTATA-5'

D51_TPM.filter 11188332_22 ATTCTTGAAAGGAGAACTCAAA

target id: Solyc03g005660.2.1

1621 5'-TCAGCATTGA-TTCTTCTGTTCAAGAATTCATT-3' 1652 cleavage site: 1637

|||| ||||o|| |||||||||

3'-AAACTCAAGAGGA-AAGTTCTTA-5'

D51_TPM.filter 20900898_21 TGAATCCTTCGGCTATCCATA

target id: Solyc09g098130.1.1

2412 5'-AAGTTTATGGATTGCTGAAGGATTCGTGGAG-3' 2442 cleavage site: 2427

||||||| ||o|||||||||o

3'-ATACCTATCGGCTTCCTAAGT-5'

D51_TPM.filter 20900987_22 TGAATCCTTCGGCTATCCATAA

target id: Solyc09g098130.1.1

2411 5'-TAAGTTTATGGATTGCTGAAGGATTCGTGGAG-3' 2442 cleavage site: 2427

|||||||| ||o|||||||||o

3'-AATACCTATCGGCTTCCTAAGT-5'

D51_TPM.filter 20900999_20 TGAATCCTTCGGCTATCCAT

target id: Solyc09g098130.1.1

2413 5'-AGTTTATGGATTGCTGAAGGATTCGTGGAG-3' 2442 cleavage site: 2427

|||||| ||o|||||||||o

3'-TACCTATCGGCTTCCTAAGT-5'

D51_TPM.filter 02315843_21 AACGAACTTATTTAATTTGAC

target id: Solyc09g098130.1.1

3310 5'-TTGCCTTCAAATTTAAATAAGTTGGTACTTGA-3' 3341 cleavage site: 3326

||||| ||||||||||| ||

3'-CAGTTT-AATTTATTCAAGCAA-5'

D51_TPM.filter 20900927_23 TGAATCCTTCGGCTATCCATAAT

target id: Solyc09g098130.1.1

2410 5'-ATAAGTTTATGGATTGCTGAAGGATTCGTGGAG-3' 2442 cleavage site: 2427

|||||||| ||o|||||||||o

3'-TAATACCTATCGGCTTCCTAAGT-5'

D51_TPM.filter 20900852_21 TGAATCCTTCGGCTATCCATT

target id: Solyc09g098130.1.1

2412 5'-AAGTTTATGGATTGCTGAAGGATTCGTGGAG-3' 2442 cleavage site: 2427

|||||| ||o|||||||||o

3'-TTACCTATCGGCTTCCTAAGT-5'

D51_TPM.filter 20901206_20 TGAATCCTTCGGCTATCCAC

target id: Solyc09g098130.1.1

2413 5'-AGTTTATGGATTGCTGAAGGATTCGTGGAG-3' 2442 cleavage site: 2427

||||| ||o|||||||||o

3'-CACCTATCGGCTTCCTAAGT-5'

D51_TPM.filter 20900824_21 TGAATCCTTCGGCTATCCATG

target id: Solyc09g098130.1.1

2412 5'-AAGTTTATGGATTGCTGAAGGATTCGTGGAG-3' 2442 cleavage site: 2427

o|||||| ||o|||||||||o

3'-GTACCTATCGGCTTCCTAAGT-5'

D51_TPM.filter 20901072_21 TGAATCCTTCGGCTATCCACA

target id: Solyc09g098130.1.1

2412 5'-AAGTTTATGGATTGCTGAAGGATTCGTGGAG-3' 2442 cleavage site: 2427

||||| ||o|||||||||o

3'-ACACCTATCGGCTTCCTAAGT-5'

D51_TPM.filter 04438187_24 AGACTTATTTGAATTTTGAAAGGC

target id: Solyc09g098130.1.1

3307 5'-CAGTTGCC-TTC-AAATTTAAATAAGTTGGTACT-3' 3338 cleavage site: 3323

||| ||| |||||o||||||||o

3'-CGGAAAGTTTTAAGTTTATTCAGA-5'

D51_TPM.filter 20900836_21 TGAATCCTTCGGCTACCCATA

target id: Solyc09g098130.1.1

2412 5'-AAGTTTATGGATTGCTGAAGGATTCGTGGAG-3' 2442 cleavage site: 2427

||||| | ||o|||||||||o

3'-ATACCCATCGGCTTCCTAAGT-5'

D51_TPM.filter 15696687_21 CAACATGTTTGAGACGAGTGG

target id: Solyc03g078300.1.1

2405 5'-AACTGCCA-TC-TCTCAAACATCTTTCAGTC-3' 2433 cleavage site: 2418

||| || |||||||||| ||

3'-GGTGAGCAGAGTTTGTACAAC-5'

D51_TPM.filter 09170535_24 ATGATTTGTAGACTATTACACAGT

target id: Solyc03g078300.1.1

1850 5'-TTTGTACTTTGT-ATAATCTACAAA-CATTGTTG-3' 1881 cleavage site: 1866

||| ||| ||| |||||||| |||

3'-TGACACATTATCAGATGTTTAGTA-5'

D51_TPM.filter 26501672_22 TTTCCTATTCCACCCATGCCAA

target id: Solyc08g075630.2.1

531 5'-AATACTTGGTATGGG-GGGATTAGGAAAGACGA-3' 562 cleavage site: 547

||||o||||| ||o| |||||||

3'-AACCGTACCCACCTT-ATCCTTT-5'

D51_TPM.filter 26502462_20 TTTCCTATTCCACCCATGCC

target id: Solyc08g075630.2.1

533 5'-TACTTGGTATGGG-GGGATTAGGAAAGACGA-3' 562 cleavage site: 547

||o||||| ||o| |||||||

3'-CCGTACCCACCTT-ATCCTTT-5'

D51_TPM.filter 26501547_21 TTTCCTATTCCACCCATGCCA

target id: Solyc08g075630.2.1

532 5'-ATACTTGGTATGGG-GGGATTAGGAAAGACGA-3' 562 cleavage site: 547

|||o||||| ||o| |||||||

3'-ACCGTACCCACCTT-ATCCTTT-5'

D51_TPM.filter 26501688_20 TTTCCTATTCCACCCATGCT

target id: Solyc08g075630.2.1

533 5'-TACTTGGTATGGG-GGGATTAGGAAAGACGA-3' 562 cleavage site: 547

o|o||||| ||o| |||||||

3'-TCGTACCCACCTT-ATCCTTT-5'

D51_TPM.filter 19124997_21 CTTTCCTACTCCTCCCATACC

target id: Solyc08g075630.2.1

533 5'-TACTTGGTATGGGGGGATTAGGAAAGACGAC-3' 563 cleavage site: 548

||||||||o||| ||||||||

3'-CCATACCCTCCTCATCCTTTC-5'

D51_TPM.filter 26501726_20 TTTCCTATTCCACCCATGCA

target id: Solyc08g075630.2.1

533 5'-TACTTGGTATGGG-GGGATTAGGAAAGACGA-3' 562 cleavage site: 547

|o||||| ||o| |||||||

3'-ACGTACCCACCTT-ATCCTTT-5'

D51_TPM.filter 10902796_21 ATTGTGTTGTACTCTTGAGAG

target id: Solyc04g009260.1.1

670 5'-TGTGTTTCTCAAGAGTACAACACAATGGATC-3' 700 cleavage site: 685

o||||||||||||||||||||

3'-GAGAGTTCTCATGTTGTGTTA-5'

D51_TPM.filter 21290291_21 TGGAATACCATCATCACCGTC

target id: Solyc04g009260.1.1

254 5'-AAGGTGATGATGATGAT-GAATTTCATAGTC-3' 283 cleavage site: 268

||o| ||||||| | |||o||

3'-CTGCCACTACTACCATAAGGT-5'

D51_TPM.filter 22690482_24 TCGACTAAATTTGAATCACGCTTT

target id: Solyc05g012890.1.1

1704 5'-GTTTCAGAGCATGTATTCAAATTCAGTTGATTCGG-3' 1738 cleavage site: 1723

|o||| || ||||||||| |||o||

3'-TTTCGCAC-TAAGTTTAAATCAGCT-5'

D51_TPM.filter 05209942_24 AGGTGTCTTTTTGTGAATCCAGAA

target id: Solyc09g018220.1.1

1616 5'-TACTTTTCT-GTTTCAC-AAAAGAC-CCTTCCAT-3' 1646 cleavage site: 1631

|||| | ||||| ||||||| |||

3'-AAGACCTAAGTGTTTTTCTGTGGA-5'

D51_TPM.filter 23501236_21 TCTCAAGTCATCCCACTCATT

target id: Solyc11g071430.1.1

704 5'-ACTACAACGAGTGGGATGACTTGAGAAACAT-3' 734 cleavage site: 719

|| ||||||||||||||||||

3'-TTACTCACCCTACTGAACTCT-5'

D51_TPM.filter 25171037_22 TTGTTGTCCTATATCTTCTGGT

target id: Solyc11g071430.1.1

736 5'-TTTGTACAAGGAGATATAGGA-AGTAAGATCA-3' 766 cleavage site: 751

|| ||o|||||||||| |oo||

3'-TGGTCTTCTATATCCTGTTGTT-5'

D51_TPM.filter 21734213_21 TGCGGGAAGATCATTGTAGCT

target id: Solyc11g071430.1.1

1073 5'-TGTTGAGCTACAATGATCTTCCCGCACATTT-3' 1103 cleavage site: 1088

|||||||||||||||||||||

3'-TCGATGTTACTAGAAGGGCGT-5'

D51_TPM.filter 21688701_21 TGCAGGAAGATCATTGTAGCT

target id: Solyc11g071430.1.1

1073 5'-TGTTGAGCTACAATGATCTTCCCGCACATTT-3' 1103 cleavage site: 1088

||||||||||||||||| |||

3'-TCGATGTTACTAGAAGGACGT-5'

D51_TPM.filter 09531475_21 ATGTGCGGGAAGATCATTGTA

target id: Solyc11g071430.1.1

1076 5'-TGAGCTACAATGATCTTCCCGCACATTTAAA-3' 1106 cleavage site: 1091

|||||||||||||||||||||

3'-ATGTTACTAGAAGGGCGTGTA-5'

D51_TPM.filter 25648313_21 TTCTCAAGTCATCCCACTCAT

target id: Solyc11g071430.1.1

705 5'-CTACAACGAGTGGGATGACTTGAGAAACATT-3' 735 cleavage site: 720

|||||||||||||||||||

3'-TACTCACCCTACTGAACTCTT-5'

D51_TPM.filter 24321395_21 TTATGGAACACCCTCTTGGTT

target id: Solyc11g071430.1.1

1534 5'-CCTCTAAGCAAGAGGGTGTTGCATAACATAC-3' 1564 cleavage site: 1549

|| |||||||||||| |||||

3'-TTGGTTCTCCCACAAGGTATT-5'

D51_TPM.filter 25483274_21 TTCCACAGCTTTCTTGAACTT

target id: Solyc08g005440.2.1

2040 5'-GGTGCAAGTTCCGGGCAAGCTGTGGAAGATGA-3' 2071 cleavage site: 2056

||||| |oo| |||||||||||

3'-TTCAA-GTTCTTTCGACACCTT-5'

D51_TPM.filter 25483505_21 TTCCACAGCTTTCTTGAACTG

target id: Solyc08g005440.2.1

2040 5'-GGTGCAAGTTCCGGGCAAGCTGTGGAAGATGA-3' 2071 cleavage site: 2056

|||| |oo| |||||||||||

3'-GTCAA-GTTCTTTCGACACCTT-5'

D51_TPM.filter 25484006_20 TTCCACAGCTTTCTTGAACT

target id: Solyc08g005440.2.1

2041 5'-GTGCAAGTTCCGGGCAAGCTGTGGAAGATGA-3' 2071 cleavage site: 2056

|||| |oo| |||||||||||

3'-TCAA-GTTCTTTCGACACCTT-5'

D51_TPM.filter 25483500_22 TTCCACAGCTTTCTTGAACTTC

target id: Solyc08g005440.2.1

2039 5'-GGGTGCAAGTTCCGGGCAAGCTGTGGAAGATGA-3' 2071 cleavage site: 2056

||||| |oo| |||||||||||

3'-CTTCAA-GTTCTTTCGACACCTT-5'

D51_TPM.filter 25484129_21 TTCCACAGCTTTCTTGAACTC

target id: Solyc08g005440.2.1

2040 5'-GGTGCAAGTTCCGGGCAAGCTGTGGAAGATGA-3' 2071 cleavage site: 2056

|||| |oo| |||||||||||

3'-CTCAA-GTTCTTTCGACACCTT-5'

D51_TPM.filter 25482910_22 TTCCACAGCTTTCTTGAACTTT

target id: Solyc08g005440.2.1

2039 5'-GGGTGCAAGTTCCGGGCAAGCTGTGGAAGATGA-3' 2071 cleavage site: 2056

||||| |oo| |||||||||||

3'-TTTCAA-GTTCTTTCGACACCTT-5'

D51_TPM.filter 25482556_21 TTCCACAGCTTTCTTGAACTA

target id: Solyc08g005440.2.1

2040 5'-GGTGCAAGTTCCGGGCAAGCTGTGGAAGATGA-3' 2071 cleavage site: 2056

|||| |oo| |||||||||||

3'-ATCAA-GTTCTTTCGACACCTT-5'

D51_TPM.filter 25483836_20 TTCCACAGCTTTCTTGAACC

target id: Solyc08g005440.2.1

2041 5'-GTGCAAGTTCCGGGCAAGCTGTGGAAGATGA-3' 2071 cleavage site: 2056

||| |oo| |||||||||||

3'-CCAA-GTTCTTTCGACACCTT-5'

D51_TPM.filter 25483427_21 TTCCACAGCTTTCTTGGACTT

target id: Solyc08g005440.2.1

2040 5'-GGTGCAAGTTCCGGGCAAGCTGTGGAAGATGA-3' 2071 cleavage site: 2056

||| |||oo| |||||||||||

3'-TTC-AGGTTCTTTCGACACCTT-5'

D51_TPM.filter 25484021_21 TTCCACAGCTTTCTTGAGCTT

target id: Solyc08g005440.2.1

2040 5'-GGTGCAAGTTCCGGGCAAGCTGTGGAAGATGA-3' 2071 cleavage site: 2056

|||o| |oo| |||||||||||

3'-TTCGA-GTTCTTTCGACACCTT-5'

D51_TPM.filter 25482540_20 TTCCACAGCTTTCTTGAACA

target id: Solyc08g005440.2.1

2041 5'-GTGCAAGTTCCGGGCAAGCTGTGGAAGATGA-3' 2071 cleavage site: 2056

||| |oo| |||||||||||

3'-ACAA-GTTCTTTCGACACCTT-5'

D51_TPM.filter 25482753_21 TTCCACAGCTTCCTTGAACTT

target id: Solyc08g005440.2.1

2040 5'-GGTGCAAGTTCCGGGCAAGCTGTGGAAGATGA-3' 2071 cleavage site: 2056

||||| |oo| |||||||||||

3'-TTCAA-GTTCCTTCGACACCTT-5'

D51_TPM.filter 25483876_20 TTCCACAGCTTTCTTGAACG

target id: Solyc08g005440.2.1

2041 5'-GTGCAAGTTCCGGGCAAGCTGTGGAAGATGA-3' 2071 cleavage site: 2056

||| |oo| |||||||||||

3'-GCAA-GTTCTTTCGACACCTT-5'

D51_TPM.filter 25483020_21 TTCCACAGCTTTCTTAAACTT

target id: Solyc08g005440.2.1

2040 5'-GGTGCAAGTTCCGGGCAAGCTGTGGAAGATGA-3' 2071 cleavage site: 2056

||||| oo| |||||||||||

3'-TTCAA-ATTCTTTCGACACCTT-5'

D51_TPM.filter 25483307_21 TTCCACAGCTTTCCTGAACTT

target id: Solyc08g005440.2.1

2040 5'-GGTGCAAGTTCCGGGCAAGCTGTGGAAGATGA-3' 2071 cleavage site: 2056

||||| |o|| |||||||||||

3'-TTCAA-GTCCTTTCGACACCTT-5'

D51_TPM.filter 25483253_21 TTCCACAGCTTTCTCGAACTT

target id: Solyc08g005440.2.1

2040 5'-GGTGCAAGTTCCGGGCAAGCTGTGGAAGATGA-3' 2071 cleavage site: 2056

||||| ||o| |||||||||||

3'-TTCAA-GCTCTTTCGACACCTT-5'

D51_TPM.filter 25482974_22 TTCCACAGCTTTCTTGAACTTA

target id: Solyc08g005440.2.1

2039 5'-GGGTGCAAGTTCCGGGCAAGCTGTGGAAGATGA-3' 2071 cleavage site: 2056

||||| |oo| |||||||||||

3'-ATTCAA-GTTCTTTCGACACCTT-5'

D51_TPM.filter 25483218_21 TTCCACAGCTTTCCTGAACTG

target id: Solyc08g005440.2.1

2040 5'-GGTGCAAGTTCCGGGCAAGCTGTGGAAGATGA-3' 2071 cleavage site: 2056

|||| |o|| |||||||||||

3'-GTCAA-GTCCTTTCGACACCTT-5'

D51_TPM.filter 16602015_21 CGACCTTGATCTTCTGAGAAG

target id: Solyc08g005440.2.1

2389 5'-AGCCACTTCCCAGAAGATCAGGATCCTATCC-3' 2419 cleavage site: 2404

|||| ||||||||||o| ||

3'-GAAGAGTCTTCTAGTTCCAGC-5'

D51_TPM.filter 16601847_20 CGACCTTGATCTTCTGAGAA

target id: Solyc08g005440.2.1

2390 5'-GCCACTTCCCAGAAGATCAGGATCCTATCC-3' 2419 cleavage site: 2404

||| ||||||||||o| ||

3'-AAGAGTCTTCTAGTTCCAGC-5'

D51_TPM.filter 25483933_20 TTCCACAGCTTTCTTGGACT

target id: Solyc08g005440.2.1

2041 5'-GTGCAAGTTCCGGGCAAGCTGTGGAAGATGA-3' 2071 cleavage site: 2056

|| |||oo| |||||||||||

3'-TC-AGGTTCTTTCGACACCTT-5'

D51_TPM.filter 25483685_22 TTCCACAGCTTTCTTGAACTGC

target id: Solyc08g005440.2.1

2038 5'-GGGGTGCAAGTTCCGGGCAAGCTGTGGAAGATGA-3' 2071 cleavage site: 2056

|| |||| |oo| |||||||||||

3'-CG-TCAA-GTTCTTTCGACACCTT-5'

D51_TPM.filter 25482533_21 TTCCACAGCTTTCTTGAATTT

target id: Solyc08g005440.2.1

2040 5'-GGTGCAAGTTCCGGGCAAGCTGTGGAAGATGA-3' 2071 cleavage site: 2056

||o|| |oo| |||||||||||

3'-TTTAA-GTTCTTTCGACACCTT-5'

D51_TPM.filter 26472044_21 TTTCCACAGCTTTCTTGAACT

target id: Solyc08g005440.2.1

2041 5'-GTGCAAGTTCCGGGCAAGCTGTGGAAGATGAA-3' 2072 cleavage site: 2057

|||| |oo| |||||||||||o

3'-TCAA-GTTCTTTCGACACCTTT-5'

D51_TPM.filter 25482725_21 TTCCACAGCTTTCTTGGACTG

target id: Solyc08g005440.2.1

2040 5'-GGTGCAAGTTCCGGGCAAGCTGTGGAAGATGA-3' 2071 cleavage site: 2056

|| |||oo| |||||||||||

3'-GTC-AGGTTCTTTCGACACCTT-5'

D51_TPM.filter 25482628_21 TTCCACAGCTTTCTTGGACTC

target id: Solyc08g005440.2.1

2040 5'-GGTGCAAGTTCCGGGCAAGCTGTGGAAGATGA-3' 2071 cleavage site: 2056

|| |||oo| |||||||||||

3'-CTC-AGGTTCTTTCGACACCTT-5'

D51_TPM.filter 25483704_20 TTCCACAGCTTTCTTAAACT

target id: Solyc08g005440.2.1

2041 5'-GTGCAAGTTCCGGGCAAGCTGTGGAAGATGA-3' 2071 cleavage site: 2056

|||| oo| |||||||||||

3'-TCAA-ATTCTTTCGACACCTT-5'

D51_TPM.filter 25483197_20 TTCCACAGCTTTCTCGAACT

target id: Solyc08g005440.2.1

2041 5'-GTGCAAGTTCCGGGCAAGCTGTGGAAGATGA-3' 2071 cleavage site: 2056

|||| ||o| |||||||||||

3'-TCAA-GCTCTTTCGACACCTT-5'

D51_TPM.filter 25482922_20 TTCCACAGCTTTCTTGAGCT

target id: Solyc08g005440.2.1

2041 5'-GTGCAAGTTCCGGGCAAGCTGTGGAAGATGA-3' 2071 cleavage site: 2056

||o| |oo| |||||||||||

3'-TCGA-GTTCTTTCGACACCTT-5'

D51_TPM.filter 25482587_20 TTCCACAGCTTCCTTGAACT

target id: Solyc08g005440.2.1

2041 5'-GTGCAAGTTCCGGGCAAGCTGTGGAAGATGA-3' 2071 cleavage site: 2056

|||| |oo| |||||||||||

3'-TCAA-GTTCCTTCGACACCTT-5'

D51_TPM.filter 25482930_21 TTCCACAGCTTCCTTGAACTG

target id: Solyc08g005440.2.1

2040 5'-GGTGCAAGTTCCGGGCAAGCTGTGGAAGATGA-3' 2071 cleavage site: 2056

|||| |oo| |||||||||||

3'-GTCAA-GTTCCTTCGACACCTT-5'

D51_TPM.filter 23617531_22 TCTTATGAATTCTAGGTCTTCT

target id: Solyc05g054340.2.1

1108 5'-GTGAAAGAAGACCTGGAAATCATAAGATCGTT-3' 1139 cleavage site: 1124

|||||||||o||| ||||||||

3'-TCTTCTGGATCTTAAGTATTCT-5'

D51_TPM.filter 23617480_21 TCTTATGAATTCTAGGTCTTC

target id: Solyc05g054340.2.1

1109 5'-TGAAAGAAGACCTGGAAATCATAAGATCGTT-3' 1139 cleavage site: 1124

||||||||o||| ||||||||

3'-CTTCTGGATCTTAAGTATTCT-5'

D51_TPM.filter 25632153_21 TTCTGTAATTCTGTTTTTTGC

target id: Solyc05g054340.2.1

3561 5'-AGCGAGTAAATAAACAGAATTGTA-AATAATA-3' 3591 cleavage site: 3576

|o||| ||||||||||oo| ||

3'-CGTTT-TTTGTCTTAATGTCTT-5'

D51_TPM.filter 23617435_22 TCTTATGAATTCTAGGTCTTCA

target id: Solyc05g054340.2.1

1108 5'-GTGAAAGAAGACCTGGAAATCATAAGATCGTT-3' 1139 cleavage site: 1124

||||||||o||| ||||||||

3'-ACTTCTGGATCTTAAGTATTCT-5'

D51_TPM.filter 15710389_23 CAACGTGACTTATGATATATATA

target id: Solyc05g054340.2.1

1527 5'-CATAGTGTATA-ATCATAAGTCCATTGTTGATCAT-3' 1560 cleavage site: 1545

|o|||| ||||||||| || o||||

3'-ATATATATAGTATTCA-GT-GCAAC-5'

D51_TPM.filter 23617345_22 TCTTATGAATTCTAGGTCTTCC

target id: Solyc05g054340.2.1

1108 5'-GTGAAAGAAGACCTGGAAATCATAAGATCGTT-3' 1139 cleavage site: 1124

||||||||o||| ||||||||

3'-CCTTCTGGATCTTAAGTATTCT-5'

D51_TPM.filter 01038720_22 AAATAATTGTCTGATCTCTGTC

target id: Solyc05g054340.2.1

3659 5'-TAGGCGATTGAAATCCGGACAATTATTTCTAGC-3' 3691 cleavage site: 3676

||o || || |o|||||||||||

3'-CTGTCTCTA-GTCTGTTAATAAA-5'

D51_TPM.filter 23617392_21 TCTTATGAATTCTAGGTCTTT

target id: Solyc05g054340.2.1

1109 5'-TGAAAGAAGACCTGGAAATCATAAGATCGTT-3' 1139 cleavage site: 1124

o|||||||o||| ||||||||

3'-TTTCTGGATCTTAAGTATTCT-5'

D51_TPM.filter 09001551_20 ATGAGCAAATTCAGACTGTG

target id: Solyc02g090380.2.1

2626 5'-GAACATATCAATTTGAATTTGCTGCATAGATC-3' 2657 cleavage site: 2642

o| || |o|||||||||| |||

3'-GT-GTCAGACTTAAACGA-GTA-5'

D51_TPM.filter 05901770_23 AGTCTGTTTCTCTCTGTGAAAAC

target id: Solyc02g090380.2.1

1412 5'-AAAAAGTTCTTTACAGCAGAGAAACA-ACTACATT-3' 1445 cleavage site: 1430

||| ||o|||| ||||||||| |||

3'-CAA-AAGTGTC-TCTCTTTGTCTGA-5'

D51_TPM.filter 20407754_21 TATGAGCAAATTCAGACTGTG

target id: Solyc02g090380.2.1

2626 5'-GAACATATCAATTTGAATTTGCTGCATAGATCA-3' 2658 cleavage site: 2643

o| || |o|||||||||| ||||

3'-GT-GTCAGACTTAAACGA-GTAT-5'

D51_TPM.filter 12576523_22 GACTCTCGGCAACGGATATCTC

target id: Solyc05g009760.1.1

1841 5'-TATCTGAGATATACT-TTGCAGGGAGTCATTGG-3' 1872 cleavage site: 1857

||||||| |o |||| |o|||||

3'-CTCTATA-GGCAACGGCTCTCAG-5'

D51_TPM.filter 20994429_21 TGAGTAAGTAGAAACAAGATC

target id: Solyc05g009760.1.1

1682 5'-TTGGAGATCATGTTTCTGATTACCCAAGCTC-3' 1712 cleavage site: 1697

|||| |||||||o |||| ||

3'-CTAGAACAAAGATGAATGAGT-5'

D51_TPM.filter 12577295_23 GACTCTCGGCAACGGATATCTCA

target id: Solyc05g009760.1.1

1840 5'-TTATCTGAGATATACT-TTGCAGGGAGTCATTGG-3' 1872 cleavage site: 1857

|||||||| |o |||| |o|||||

3'-ACTCTATA-GGCAACGGCTCTCAG-5'

D51_TPM.filter 26760739_21 TTTTGGACTATTAAACAAAGT

target id: Solyc05g009760.1.1

2016 5'-GGAGGCCTTTGTTT-ATATTCCAAGACTACG-3' 2045 cleavage site: 2030

|||||||| ||| |||||o|

3'-TGAAACAAATTATCAGGTTTT-5'

D51_TPM.filter 18732768_20 CTCTTCAAACTTTGGACGTT

target id: Solyc05g009760.1.1

1985 5'-TAAAAAGTTGTCCCAAGCTTGAAGAGATCCC-3' 2015 cleavage site: 2000

|o o|||| ||| ||||||||

3'-TT-GCAGGTTTCAAACTTCTC-5'

D51_TPM.filter 23014737_21 TCCGAAAATTCTGACTCTGTC

target id: Solyc07g052790.1.1

2234 5'-TTTTTCCCAGAGTCAGAA-TTTCAGAGTTGT-3' 2263 cleavage site: 2248

||||||||||| |||| ||

3'-CTGTCTCAGTCTTAAAAGCCT-5'

D51_TPM.filter 19989762_22 TAGTGTTTAAAATAGTATGTAG

target id: Solyc07g052790.1.1

2488 5'-TGCTTGTACATACCATTTGAAACATTGTGGAA-3' 2519 cleavage site: 2504

||||||| |||| |||||o|o

3'-GATGTATGATAAAATTTGTGAT-5'

D51_TPM.filter 18205016_20 CTATCATTGTGAAGCAGAAT

target id: Solyc07g039410.2.1

917 5'-TTCAAATTC--CATCACAATGATAGATCTT-3' 944 cleavage site: 929

|||| | ||||||||||||

3'-TAAGACGAAGTGTTACTATC-5'

D51_TPM.filter 18205332_22 CTATCATTGTGAAGCAGAATTC

target id: Solyc07g039410.2.1

915 5'-TTTTCAAATTC--CATCACAATGATAGATCTT-3' 944 cleavage site: 929

||||| | ||||||||||||

3'-CTTAAGACGAAGTGTTACTATC-5'

D51_TPM.filter 20527493_21 TATCATTGTGAAGCAGAATTC

target id: Solyc07g039410.2.1

915 5'-TTTTCAAATTC--CATCACAATGATAGATCT-3' 943 cleavage site: 928

||||| | |||||||||||

3'-CTTAAGACGAAGTGTTACTAT-5'

D51_TPM.filter 20527296_20 TATCATTGTGAAGCAGAATT

target id: Solyc07g039410.2.1

916 5'-TTTCAAATTC--CATCACAATGATAGATCT-3' 943 cleavage site: 928

||||| | |||||||||||

3'-TTAAGACGAAGTGTTACTAT-5'

D51_TPM.filter 25890832_22 TTTAGTTTCTGTTCCATTTGTT

target id: Solyc01g088060.2.1

2609 5'-CTAGCTACAAATGGAAACA-AAGCTGAATACCT-3' 2640 cleavage site: 2625

|||||||| |||| ||o||o||

3'-TTGTTTACC-TTGTCTTTGATTT-5'

D51_TPM.filter 01566387_22 AAGACTTTGAATACTTTGCACA

target id: Solyc01g088060.2.1

2024 5'-GAGAGTGTCCAGAG-ATTCAAAGTCTTTCGAG-3' 2054 cleavage site: 2039

||| ||o|| ||||||||||||

3'-ACACGTTTCATAAGTTTCAGAA-5'

D51_TPM.filter 25699572_21 TTCTTGTCACTGATTCTGATG

target id: Solyc01g088060.2.1

2397 5'-TTCTCTACCAGAATCAGTGA-AA-AAGCTAA-3' 2425 cleavage site: 2410

o| |||||||||||| || ||

3'-GTAGTCTTAGTCACTGTTCTT-5'

D51_TPM.filter 25874399_24 TTTAGTAGCTAGTTCATTAAGACT

target id: Solyc01g088060.2.1

2594 5'-CAAGTAG-C-TGATGGACTAGCTAC-AAATGGAA-3' 2624 cleavage site: 2609

|| | |o|||o||||||||| |||

3'-TCAGAATTACTTGATCGATGATTT-5'

D51_TPM.filter 25393306_24 TTCGATTTATTTCAAGACTTGAAT

target id: Solyc05g009740.1.1

1963 5'-CGAGAGTTCAAAGTCTTGAAAT-ATTTGAAACTAG-3' 1996 cleavage site: 1981

o||| |||||||||||| | |o|||

3'-TAAG-TTCAGAACTTTATTTAGCTT-5'

D51_TPM.filter 26472711_24 TTTCCACGTGACATGTTTAAGAGT

target id: Solyc05g009740.1.1

260 5'-TTGATATTCTTAAACAAGTCAC-TGGTGAAAAACA-3' 293 cleavage site: 278

|o||||||||| ||||| ||| o||

3'-TGAGAATTTGTACAGTGCACC-TTT-5'

D51_TPM.filter 03125976_21 AATGATCTTGAACTCAACTCG

target id: Solyc11g069990.1.1

439 5'-TTTCTCGAGTTGAGGTCAAGATCATTATTCG-3' 469 cleavage site: 454

||||||||| |||||||||||

3'-GCTCAACTCAAGTTCTAGTAA-5'

D51_TPM.filter 03129505_21 AATGATCTTGATCTCAACTCA

target id: Solyc11g069990.1.1

439 5'-TTTCTCGAGTTGAGGTCAAGATCATTATTCG-3' 469 cleavage site: 454

||||||||o|||||||||||

3'-ACTCAACTCTAGTTCTAGTAA-5'

D51_TPM.filter 03128094_21 AATGATCTTGACCTCAACTCG

target id: Solyc11g069990.1.1

439 5'-TTTCTCGAGTTGAGGTCAAGATCATTATTCG-3' 469 cleavage site: 454

|||||||||||||||||||||

3'-GCTCAACTCCAGTTCTAGTAA-5'

D51_TPM.filter 03126219_21 AATGATCTTGAACTCAACTCA

target id: Solyc11g069990.1.1

439 5'-TTTCTCGAGTTGAGGTCAAGATCATTATTCG-3' 469 cleavage site: 454

|||||||| |||||||||||

3'-ACTCAACTCAAGTTCTAGTAA-5'

D51_TPM.filter 19603416_21 TAATGATCTTGAACTCAACTC

target id: Solyc11g069990.1.1

440 5'-TTCTCGAGTTGAGGTCAAGATCATTATTCGA-3' 470 cleavage site: 455

|||||||| ||||||||||||

3'-CTCAACTCAAGTTCTAGTAAT-5'

D51_TPM.filter 21325976_21 TGGAGTGTCTAAGTGAAAATT

target id: Solyc11g069990.1.1

1401 5'-GGTAGAATTGTCACTTAGGCACTGCAAGGAC-3' 1431 cleavage site: 1416

|||| ||||||||o|||| ||

3'-TTAAAAGTGAATCTGTGAGGT-5'

D51_TPM.filter 24745631_22 TTGGAGTGTCTAAGTGAAAATT

target id: Solyc11g069990.1.1

1401 5'-GGTAGAATTGTCACTTAGGCACTGCAAGGACT-3' 1432 cleavage site: 1417

|||| ||||||||o|||| |||

3'-TTAAAAGTGAATCTGTGAGGTT-5'

D51_TPM.filter 19603252_22 TAATGATCTTGAACTCAACTCG

target id: Solyc11g069990.1.1

439 5'-TTTCTCGAGTTGAGGTCAAGATCATTATTCGA-3' 470 cleavage site: 455

||||||||| ||||||||||||

3'-GCTCAACTCAAGTTCTAGTAAT-5'

D51_TPM.filter 26210487_21 TTTGCAGCGTAAAATACCAGC

target id: Solyc11g069990.1.1

167 5'-CTCTTGCTGGCATGTTACGCTCCAAATCAGA-3' 197 cleavage site: 182

||||| || ||||||| ||||

3'-CGACCATAAAATGCGACGTTT-5'

D51_TPM.filter 19603637_21 TAATGATCTTGATCTCAACTC

target id: Solyc11g069990.1.1

440 5'-TTCTCGAGTTGAGGTCAAGATCATTATTCGA-3' 470 cleavage site: 455

||||||||o||||||||||||

3'-CTCAACTCTAGTTCTAGTAAT-5'

D51_TPM.filter 03126376_21 AATGATCTTGATCTCAACTCG

target id: Solyc11g069990.1.1

439 5'-TTTCTCGAGTTGAGGTCAAGATCATTATTCG-3' 469 cleavage site: 454

|||||||||o|||||||||||

3'-GCTCAACTCTAGTTCTAGTAA-5'

D51_TPM.filter 24321395_21 TTATGGAACACCCTCTTGGTT

target id: Solyc11g069990.1.1

730 5'-CCACTAAGCAAGAGGGTGTTGCATAACATAC-3' 760 cleavage site: 745

|| |||||||||||| |||||

3'-TTGGTTCTCCCACAAGGTATT-5'

D51_TPM.filter 19604076_21 TAATGATCTTGACCTCAACTC

target id: Solyc11g069990.1.1

440 5'-TTCTCGAGTTGAGGTCAAGATCATTATTCGA-3' 470 cleavage site: 455

|||||||||||||||||||||

3'-CTCAACTCCAGTTCTAGTAAT-5'

D51_TPM.filter 18070827_21 CTAGAGAAACTTGGAATTCCC

target id: Solyc11g069660.1.1

778 5'-CTAGTGGGAG--CCAAGTTTCTTGTAGGTAGT-3' 807 cleavage site: 792

||||o ||||||||||o |||

3'-CCCTTAAGGTTCAAAGAG-ATC-5'

D51_TPM.filter 18756713_21 CTTAAGGATGTTAGTCTAGGC

target id: Solyc11g069660.1.1

466 5'-ATACTGCCAAGACTGACATCCTTAAGGGCAT-3' 496 cleavage site: 481

||| |||||o|||||||||||

3'-CGGATCTGATTGTAGGAATTC-5'

D51_TPM.filter 18070593_21 CTAGAGAAACTTGGAATTCCA

target id: Solyc11g069660.1.1

777 5'-GCTAGTGGGA-GCCAAGTTTCTTGTAGGTAGT-3' 807 cleavage site: 792

|||o| ||||||||||o |||

3'-ACCTTAAGGTTCAAAGAG-ATC-5'

D51_TPM.filter 23852925_20 TTAAGGATGTTAGTCTAGGC

target id: Solyc11g069660.1.1

466 5'-ATACTGCCAAGACTGACATCCTTAAGGGCA-3' 495 cleavage site: 480

||| |||||o||||||||||

3'-CGGATCTGATTGTAGGAATT-5'

D51_TPM.filter 18756878_21 CTTAAGGATGTTAGTCTAGGT

target id: Solyc11g069660.1.1

466 5'-ATACTGCCAAGACTGACATCCTTAAGGGCAT-3' 496 cleavage site: 481

o|| |||||o|||||||||||

3'-TGGATCTGATTGTAGGAATTC-5'

D51_TPM.filter 23852157_21 TTAAGGATGTTAGTCTAGGCA

target id: Solyc11g069660.1.1

465 5'-CATACTGCCAAGACTGACATCCTTAAGGGCA-3' 495 cleavage site: 480

|||| |||||o||||||||||

3'-ACGGATCTGATTGTAGGAATT-5'

D51_TPM.filter 25057979_21 TTGTGATTGAAAAAGGAGATT

target id: Solyc11g069660.1.1

2013 5'-TTGGCAGTTGCCTTGTTCTATCACAAGACTT-3' 2043 cleavage site: 2028

|o|o |||| ||| |||||||

3'-TTAGAGGAAAAAGTTAGTGTT-5'

D51_TPM.filter 24321395_21 TTATGGAACACCCTCTTGGTT

target id: Solyc11g069660.1.1

439 5'-CATATAAGCAAGAGGGTGTTGCATAACATAC-3' 469 cleavage site: 454

|| |||||||||||| |||||

3'-TTGGTTCTCCCACAAGGTATT-5'

D51_TPM.filter 18070482_21 CTAGAGAAACTTGGAACTCCT

target id: Solyc11g069660.1.1

778 5'-CTAGTGGGAG--CCAAGTTTCTTGTAGGTAGT-3' 807 cleavage site: 792

o|||| ||||||||||o |||

3'-TCCTCAAGGTTCAAAGAG-ATC-5'

D51_TPM.filter 04338850_24 AGACGTGTTAAACTATTATAGTAT

target id: Solyc08g013970.1.1

2042 5'-TTAACAT-TTGTGACAGTTTAACATGTCTGTTCA-3' 2074 cleavage site: 2059

|| o|o|o| |||||||||o||||

3'-TATGATATTATCAAATTGTGCAGA-5'

D51_TPM.filter 06513050_24 ACATCCTTCTATCTTAAACTCTGT

target id: Solyc12g044190.1.1

2869 5'-AACCTCCGAAGTTTACGGATAGAAGGATGTTCATC-3' 2903 cleavage site: 2888

|o |||||| o|||||||||||||

3'-TGTCTCAAAT-TCTATCTTCCTACA-5'

D51_TPM.filter 22990149_21 TCCACAAATCATCCCCTTCTA

target id: Solyc12g044190.1.1

573 5'-TACATTACAAGGGGATGATTTGTGGAGTCGT-3' 603 cleavage site: 588

|| ||||||||||||||||||

3'-ATCTTCCCCTACTAAACACCT-5'

D51_TPM.filter 10671292_21 ATTATTTTCCTCAAACAAGGA

target id: Solyc12g044190.1.1

1109 5'-TTTGTTCTTTGTTTGAGGAAGATAGTGACAT-3' 1139 cleavage site: 1124

||o||||||||||||o|||o|

3'-AGGAACAAACTCCTTTTATTA-5'

D51_TPM.filter 07624441_21 ACTTCCATGATCTTCTGAGCT

target id: Solyc07g044800.2.1

454 5'-ATGAAAACTCAGAAGATCATGGAGGTTGGAA-3' 484 cleavage site: 469

||||||||||||||||o||

3'-TCGAGTCTTCTAGTACCTTCA-5'

D51_TPM.filter 03461935_24 AATCTCTATGATTCTTCTGAATTT

target id: Solyc07g044800.2.1

453 5'-AATGAAAACTCAGAA-GATCATGGAGGTTGGAAC-3' 485 cleavage site: 470

||| |||||| o|||||o|||o||

3'-TTTAAGTCTTCTTAGTATCTCTAA-5'

D51_TPM.filter 19000618_21 CTTCCATGATCTTCTGAGCTT

target id: Solyc07g044800.2.1

453 5'-AATGAAAACTCAGAAGATCATGGAGGTTGGA-3' 483 cleavage site: 468

|| ||||||||||||||||o|

3'-TTCGAGTCTTCTAGTACCTTC-5'

D51_TPM.filter 19000757_20 CTTCCATGATCTTCTGAGCT

target id: Solyc07g044800.2.1

454 5'-ATGAAAACTCAGAAGATCATGGAGGTTGGA-3' 483 cleavage site: 468

||||||||||||||||o|

3'-TCGAGTCTTCTAGTACCTTC-5'

D51_TPM.filter 07624113_21 ACTTCCATGATCTTCTGAGCC

target id: Solyc07g044800.2.1

454 5'-ATGAAAACTCAGAAGATCATGGAGGTTGGAA-3' 484 cleavage site: 469

||||||||||||||||o||

3'-CCGAGTCTTCTAGTACCTTCA-5'

D51_TPM.filter 07624096_21 ACTTCCATGATCTTCTGAGCA

target id: Solyc07g044800.2.1

454 5'-ATGAAAACTCAGAAGATCATGGAGGTTGGAA-3' 484 cleavage site: 469

||||||||||||||||o||

3'-ACGAGTCTTCTAGTACCTTCA-5'

D51_TPM.filter 15741889_21 CAACTTCCATGATCTTCTGAG

target id: Solyc07g044800.2.1

456 5'-GAAAACTCAGAAGATCATGGAGGTTGGAACC-3' 486 cleavage site: 471

||||||||||||||||o||||

3'-GAGTCTTCTAGTACCTTCAAC-5'

D51_TPM.filter 25490144_21 TTCCATGATCTTCTGAGCTTC

target id: Solyc07g044800.2.1

452 5'-AAATGAAAACTCAGAAGATCATGGAGGTTGG-3' 482 cleavage site: 467

|| ||||||||||||||||o

3'-CTTCGAGTCTTCTAGTACCTT-5'

D51_TPM.filter 02706291_22 AACTTCCATGATCTTCTGAGCT

target id: Solyc07g044800.2.1

454 5'-ATGAAAACTCAGAAGATCATGGAGGTTGGAAC-3' 485 cleavage site: 470

||||||||||||||||o|||

3'-TCGAGTCTTCTAGTACCTTCAA-5'

D51_TPM.filter 02706558_21 AACTTCCATGATCTTCTGAGC

target id: Solyc07g044800.2.1

455 5'-TGAAAACTCAGAAGATCATGGAGGTTGGAAC-3' 485 cleavage site: 470

||||||||||||||||o|||

3'-CGAGTCTTCTAGTACCTTCAA-5'

D51_TPM.filter 06187027_21 ACAACGATCTTTTCTGAATAG

target id: Solyc10g051050.1.1

236 5'-AAAGACTATTCAAAAAAGATTTG-TGTCCTCC-3' 266 cleavage site: 251

||||||| |||||| |o| |||

3'-GATAAGTCTTTTCT-AGCAACA-5'

D51_TPM.filter 06186540_20 ACAACGATCTTTTCTGAATA

target id: Solyc10g051050.1.1

237 5'-AAGACTATTCAAAAAAGATTTG-TGTCCTCC-3' 266 cleavage site: 251

|||||| |||||| |o| |||

3'-ATAAGTCTTTTCT-AGCAACA-5'

D51_TPM.filter 06299020_21 ACAGCGATCTTTTCTGAATAG

target id: Solyc10g051050.1.1

236 5'-AAAGACTATTCAAAAAAGATTTG-TGTCCTCC-3' 266 cleavage site: 251

||||||| |||||| |o| |||

3'-GATAAGTCTTTTCT-AGCGACA-5'

D51_TPM.filter 25553387_21 TTCCTCAGCTTTCTTGAACTT

target id: Solyc10g051050.1.1

368 5'-ATGACAA-TTGGAAGAAGGTTGAGGAAAATCT-3' 398 cleavage site: 383

|| || |||||o|o|||||||

3'-TTCAA-GTTCTTTCGACTCCTT-5'

D51_TPM.filter 18378363_21 CTGGCATTGTTCGGAGACATG

target id: Solyc10g051050.1.1

411 5'-TGGTACG-GTATCCGAACGATGCCAATCAAT-3' 440 cleavage site: 425

|o || |||||||o||||||

3'-GTACAGAGGCTTGTTACGGTC-5'

D51_TPM.filter 21533248_20 TGGCATTGTTCGGAGACATG

target id: Solyc10g051050.1.1

411 5'-TGGTACG-GTATCCGAACGATGCCAATCAA-3' 439 cleavage site: 424

|o || |||||||o||||||

3'-GTACAGAGGCTTGTTACGGT-5'

D51_TPM.filter 04908394_23 AGGATTTAGGAGATTACTTTGTT

target id: Solyc04g079420.2.1

1449 5'-TTTCAGACAAA--AATCTCCTAAATCTAGTCAA-3' 1479 cleavage site: 1464

o||||| |||||||||||||o

3'-TTGTTTCATTAGAGGATTTAGGA-5'

D51_TPM.filter 18429739_20 CTGCCGAAGCTGTGGGATGC

target id: Solyc04g079420.2.1

2030 5'-GGTTTGT-TCCCACAGCTCTCGG-AGTTCAC-3' 2058 cleavage site: 2043

|o |||||||||| |||| ||

3'-CGTAGGGTGTCGA-AGCCGTC-5'

D51_TPM.filter 19556119_22 TAACTTGTATGGATCTCTATCA

target id: Solyc04g079420.2.1

1487 5'-CGAGCTGG-AGACATGTATACAAGTTACTATG-3' 1517 cleavage site: 1502

||o ||| || o||||||||||

3'-ACTATCTCTAGGTATGTTCAAT-5'

D51_TPM.filter 18429611_20 CTGCCGAAGCTGTGGGATGA

target id: Solyc04g079420.2.1

2028 5'-CTGGTTTGTTCCCACAGCTCTCGG-AGTTCAC-3' 2058 cleavage site: 2043

|oo |||||||||| |||| ||

3'-AGT-AGGGTGTCGA-AGCCGTC-5'

D51_TPM.filter 18429013_21 CTGCCGAAGCTGTGGGATGCA

target id: Solyc04g079420.2.1

2029 5'-TGGTTTGT-TCCCACAGCTCTCGG-AGTTCAC-3' 2058 cleavage site: 2043

||o |||||||||| |||| ||

3'-ACGTAGGGTGTCGA-AGCCGTC-5'

D51_TPM.filter 12064522_20 GAATCCCTCCTCGCCCACCA

target id: Solyc04g079420.2.1

488 5'-AGAAGTGGTGGAG-GAGGAGGGTGTTTAGGGG-3' 518 cleavage site: 503

|||||| | |||||||| o||o

3'-ACCACC-CGCTCCTCCC-TAAG-5'

D51_TPM.filter 18428999_21 CTGCCGAAGCTGTGGGATGTA

target id: Solyc04g079420.2.1

2029 5'-TGGTTTGT-TCCCACAGCTCTCGG-AGTTCAC-3' 2058 cleavage site: 2043

|oo |||||||||| |||| ||

3'-ATGTAGGGTGTCGA-AGCCGTC-5'

D51_TPM.filter 18429277_22 CTGCCGAAGCTGTGGGATGTAA

target id: Solyc04g079420.2.1

2028 5'-CTGGTTTGT-TCCCACAGCTCTCGG-AGTTCAC-3' 2058 cleavage site: 2043

||oo |||||||||| |||| ||

3'-AATGTAGGGTGTCGA-AGCCGTC-5'

D51_TPM.filter 06530231_24 ACATTATCACTTGTGTGAACCTCC

target id: Solyc04g079420.2.1

355 5'-AGTAGCGAGGTTCATGCAAGTGACAATGCAAGCT-3' 388 cleavage site: 373

||||||||oo||||||| ||||

3'-CCTCCAAGTGTGTTCACTATTACA-5'

D51_TPM.filter 18429725_20 CTGCCGAAGCTGTGGGATGT

target id: Solyc04g079420.2.1

2030 5'-GGTTTGT-TCCCACAGCTCTCGG-AGTTCAC-3' 2058 cleavage site: 2043

oo |||||||||| |||| ||

3'-TGTAGGGTGTCGA-AGCCGTC-5'

D51_TPM.filter 18429203_20 CTGCCGAAGCTGTGGGACGT

target id: Solyc04g079420.2.1

2030 5'-GGTTTGT-TCCCACAGCTCTCGG-AGTTCAC-3' 2058 cleavage site: 2043

oo |||||||||| |||| ||

3'-TGCAGGGTGTCGA-AGCCGTC-5'

D51_TPM.filter 26206894_21 TTTGCAAAACTGTGCTCAATT

target id: Solyc04g079420.2.1

547 5'-GAAATGGTTTGAGGATAGTTTTGTAAATTTAG-3' 578 cleavage site: 563

oo ||||| |o|||||||o|||

3'-TT-AACTCGTGTCAAAACGTTT-5'

D51_TPM.filter 18429386_21 CTGCCGAAGCTGTGGGATACT

target id: Solyc04g079420.2.1

2029 5'-TGGTTTGT-TCCCACAGCTCTCGG-AGTTCAC-3' 2058 cleavage site: 2043

|| |||||||||| |||| ||

3'-TCATAGGGTGTCGA-AGCCGTC-5'

D51_TPM.filter 11630625_22 ATTTTCGAACCAAAGTGATTCT

target id: Solyc04g079420.2.1

1890 5'-ACTTGAGAAGC-CTTTGGTTCGAGAAGATATC-3' 1920 cleavage site: 1905

|||| | |||||||||||o||

3'-TCTTAGTGAAACCAAGCTTTTA-5'

D51_TPM.filter 04913432_22 AGGATTTGTGGACTGGTAGATA

target id: Solyc04g079420.2.1

1927 5'-TCACTTATCTG--ACTCCACAAATCCTCTCAA-3' 1956 cleavage site: 1941

|||||o | ||||||||||||

3'-ATAGATGGTCAGGTGTTTAGGA-5'

D51_TPM.filter 17487999_20 CCGAAGCTGTGGGATGTAAA

target id: Solyc04g079420.2.1

2027 5'-CCTGGTTTGT-TCCCACAGCTCTCGGAGTTC-3' 2056 cleavage site: 2041

|||oo |||||||||| ||||

3'-AAATGTAGGGTGTCGA-AGCC-5'

D51_TPM.filter 18429391_23 CTGCCGAAGCTGTGGGATGTAAA

target id: Solyc04g079420.2.1

2027 5'-CCTGGTTTGT-TCCCACAGCTCTCGG-AGTTCAC-3' 2058 cleavage site: 2043

|||oo |||||||||| |||| ||

3'-AAATGTAGGGTGTCGA-AGCCGTC-5'

D51_TPM.filter 07665318_20 ACTTTGTGAAATGACTTGAG

target id: Solyc04g079420.2.1

2265 5'-AGTACCTTGA--CATTTCACAATGTGTTGG-3' 2292 cleavage site: 2277

||oo| |||||||||| ||

3'-GAGTTCAGTAAAGTGTTTCA-5'

D51_TPM.filter 09799974_23 ATCACTTGTGTGAACCTCCCATA

target id: Solyc04g079420.2.1

352 5'-AAGAGTA-GCGAGGTTCATGCAAGTGACAATGC-3' 383 cleavage site: 368

|| | ||||||||oo|||||||

3'-ATACCCTCCAAGTGTGTTCACTA-5'
