# Supplementary material for: A role for small RNA in regulating innate immunity during plant growth
Source: PLoS Pathog. 2018 Jan 2;14(1):e1006756. doi: 10.1371/journal.ppat.1006756 (PMC5766230; doi:10.1371/journal.ppat.1006756)
Supplement: S3 Data — (DOCX) [file ppat.1006756.s013.docx]

**Supplemental data 3. Information of vectors used in this research**

|  | Name | Vector backbone | Insertion | Experiment |
| --- | --- | --- | --- | --- |
| 1 | Npro::GUS | pH7 | N promoter, GUS | N gene expression |
| 2 | nta-MIR6019, 6020proa-2k::Exon1a | pH7Lic15.0 | nta-MIR6019, 6020proa-2k::Exon1a | nta-MIR6019, 6020 expression |
| 3 | nta-MIR6019, 6020proa-3k::Exon1a | pH7Lic15.0 | nta-MIR6019, 6020proa-3k::Exon1a |  |
| 4 | nta-MIR6019, 6020proa-5k::Exon1a | pH7Lic15.0 | nta-MIR6019, 6020proa-5k::Exon1a |  |
| 5 | nta-MIR6019, 6020prob-2k::Exon1b | pH7Lic15.0 | nta-MIR6019, 6020prob-2k::Exon1b |  |
| 6 | nta-MIR6019, 6020prob-4k::Exon1b | pH7Lic15.0 | nta-MIR6019, 6020prob-4k::Exon1b |  |
| 7 | nta-MIR6019, 6020proa-3k::GUS | pCambia1381xb | nta-MIR6019, 6020proa-3k |  |
| 8 | nta-MIR6019, 6020prob-2k::GUS | pCambia1381xb | nta-MIR6019, 6020prob-2k |  |
| 9 | sly-MIR6020 | pH7Lic2.0 | sly-MIR6020 | sly-miR6020 cleavage assay |
| 10 | AMIR6020 | pH7Lic1.0 | Artificial sly-miR6020 |  |
| 11 | MS4miR6020 | pH7Lic2.0 | sly-miR6020 binding site |  |
| 12 | MS4miR6020^E^ | pH7Lic2.0 | sly-miR6020 mutant binding site |  |

The sequence of vectors used in this research:

**>Npro::GUS (1bp - 16054bp, complementary) 16054bp**

**GGTACCCTCGAATTATCATACATGAGAATTAAGGGAGTCACGTTATGACCCCGCCGATGACGCGGGACAA**

**GCCGTTTTACGTTTGGAACTGACAGAACCGCAACGTTGAAGGAGCCACTGAGCCGCGGGTTTCTGGAGTT**

**TAATGAGCTAAGCACATACGTCAGAAACCATTATTGCGCGTTCAAAAGTCGCCTAAGGTCACTATCAGCT**

**AGCAAATATTTCTTGTCAAAAATGCTCCACTGACGTTCCATAAATTCCCCTCGGTATCCAATTAGAGTCT**

**CATATTCACTCTCAACTCGAATCCCCCCTATGAAAAAGCCTGAACTCACCGCGACGTCTGTCGAGAAGTT**

**TCTGATCGAAAAGTTCGACAGCGTCTCCGACCTGATGCAGCTCTCGGAGGGCGAAGAATCTCGTGCTTTC**

**AGCTTCGATGTAGGAGGGCGTGGATATGTCCTGCGGGTAAATAGCTGCGCCGATGGTTTCTACAAAGATC**

**GTTATGTTTATCGGCACTTTGCATCGGCCGCGCTCCCGATTCCGGAAGTGCTTGACATTGGGGAGTTCAG**

**CGAGAGCCTGACCTATTGCATCTCCCGCCGTGCACAGGGTGTCACGTTGCAAGACCTGCCTGAAACCGAA**

**CTGCCCGCTGTTCTTCAGCCGGTCGCGGAGGCTATGGATGCTATCGCTGCGGCCGATCTTAGCCAGACGA**

**GCGGGTTCGGCCCATTCGGACCGCAAGGAATCGGTCAATACACTACATGGCGTGATTTCATATGCGCGAT**

**TGCTGATCCCCATGTGTATCACTGGCAAACTGTGATGGACGACACCGTCAGTGCGTCCGTCGCGCAGGCT**

**CTCGATGAGCTGATGCTTTGGGCGAGGACTGCCCCGAAGTCCGGCACTCGTGCACGCGGATTCGGCTCCA**

**ACAATGTCCTGACGGACAATGGCCGCATAACAGCGGTCATTGACTGGAGCGAGGCGATGTTCGGGGATTC**

**CCAATACGAGGTCGCCAACATCTTCTTCTGGAGGCCGTGGTTGGCTTGTATGGAGCAGCAGACGCGCTAC**

**TTCGAGCGGAGGCATCCGGAGCTTGCAGGATCGCCAAGGCTCCGGGCGTATATGCTCCGCATTGGTCTTG**

**ACCAACTCTATCAGAGCTTGGTTGACGGCAATTTCGATGATGCAGCTTGGGCGCAGGGTCGATGCGACGC**

**AATCGTCCGATCCGGAGCCGGGACTGTCGGGCGTACACAAATCGCCCGCAGAAGCGCGGCCGTCTGGACC**

**GATGGCTGTGTAGAAGTACTCGCCGATAGTGGAAACCGACGCCCCAGCACTCGTCCGAGGGCAAAGGAAT**

**AGAGTAGATGCCGACCGAACAAGAGCTGATTTCGAGAACGCCTCAGCCAGCAACTCGCGCGAGCCTAGCA**

**AGGCAAATGCGAGAGAACGGCCTTACGCTTGGTGGCACAGTTCTCGTCCACAGTTCGCTAAGCTCGCTCG**

**GCTGGGTCGCGGGAGGGCCGGTCGCAGTGATTCAGGAATTAATTCCCTAGAGTCAAGCAGATCGTTCAAA**

**CATTTGGCAATAAAGTTTCTTAAGATTGAATCCTGTTGCCGGTCTTGCGATGATTATCATATAATTTCTG**

**TTGAATTACGTTAAGCATGTAATAATTAACATGTAATGCATGACGTTATTTATGAGATGGGTTTTTATGA**

**TTAGAGTCCCGCAATTATACATTTAATACGCGATAGAAAACAAAATATAGCGCGCAAACTAGGATAAATT**

**ATCGCGCGCGGTGTCATCTATGTTACTAGATCGACCGGCATGCAAGCTGATAATTCAATTCGGCGTTAAT**

**TCAGTACATTAAAAACGTCCGCAATGTGTTATTAAGTTGTCTAAGCGTCAATTTGTTTACACCACAATAT**

**ATCCTGCCACCAGCCAGCCAACAGCTCCCCGACCGGCAGCTCGGCACAAAATCACCACTCGATACAGGCA**

**GCCCATCAGTCCGGGACGGCGTCAGCGGGAGAGCCGTTGTAAGGCGGCAGACTTTGCTCATGTTACCGAT**

**GCTATTCGGAAGAACGGCAACTAAGCTGCCGGGTTTGAAACACGGATGATCTCGCGGAGGGTAGCATGTT**

**GATTGTAACGATGACAGAGCGTTGCTGCCTGTGATCAATTCGGGCACGAACCCAGTGGACATAAGCCTCG**

**TTCGGTTCGTAAGCTGTAATGCAAGTAGCGTAACTGCCGTCACGCAACTGGTCCAGAACCTTGACCGAAC**

**GCAGCGGTGGTAACGGCGCAGTGGCGGTTTTCATGGCTTCTTGTTATGACATGTTTTTTTGGGGTACAGT**

**CTATGCCTCGGGCATCCAAGCAGCAAGCGCGTTACGCCGTGGGTCGATGTTTGATGTTATGGAGCAGCAA**

**CGATGTTACGCAGCAGGGCAGTCGCCCTAAAACAAAGTTAAACATCATGGGGGAAGCGGTGATCGCCGAA**

**GTATCGACTCAACTATCAGAGGTAGTTGGCGTCATCGAGCGCCATCTCGAACCGACGTTGCTGGCCGTAC**

**ATTTGTACGGCTCCGCAGTGGATGGCGGCCTGAAGCCACACAGTGATATTGATTTGCTGGTTACGGTGAC**

**CGTAAGGCTTGATGAAACAACGCGGCGAGCTTTGATCAACGACCTTTTGGAAACTTCGGCTTCCCCTGGA**

**GAGAGCGAGATTCTCCGCGCTGTAGAAGTCACCATTGTTGTGCACGACGACATCATTCCGTGGCGTTATC**

**CAGCTAAGCGCGAACTGCAATTTGGAGAATGGCAGCGCAATGACATTCTTGCAGGTATCTTCGAGCCAGC**

**CACGATCGACATTGATCTGGCTATCTTGCTGACAAAAGCAAGAGAACATAGCGTTGCCTTGGTAGGTCCA**

**GCGGCGGAGGAACTCTTTGATCCGGTTCCTGAACAGGATCTATTTGAGGCGCTAAATGAAACCTTAACGC**

**TATGGAACTCGCCGCCCGACTGGGCTGGCGATGAGCGAAATGTAGTGCTTACGTTGTCCCGCATTTGGTA**

**CAGCGCAGTAACCGGCAAAATCGCGCCGAAGGATGTCGCTGCCGACTGGGCAATGGAGCGCCTGCCGGCC**

**CAGTATCAGCCCGTCATACTTGAAGCTAGACAGGCTTATCTTGGACAAGAAGAAGATCGCTTGGCCTCGC**

**GCGCAGATCAGTTGGAAGAATTTGTCCACTACGTGAAAGGCGAGATCACCAAGGTAGTCGGCAAATAATG**

**TCTAGCTAGAAATTCGTTCAAGCCGACGCCGCTTCGCCGGCGTTAACTCAAGCGATTAGATGCACTAAGC**

**ACATAATTGCTCACAGCCAAACTATCAGGTCAAGTCTGCTTTTATTATTTTTAAGCGTGCATAATAAGCC**

**CTACACAAATTGGGAGATATATCATGCATGACCAAAATCCCTTAACGTGAGTTTTCGTTCCACTGAGCGT**

**CAGACCCCGTAGAAAAGATCAAAGGATCTTCTTGAGATCCTTTTTTTCTGCGCGTAATCTGCTGCTTGCA**

**AACAAAAAAACCACCGCTACCAGCGGTGGTTTGTTTGCCGGATCAAGAGCTACCAACTCTTTTTCCGAAG**

**GTAACTGGCTTCAGCAGAGCGCAGATACCAAATACTGTCCTTCTAGTGTAGCCGTAGTTAGGCCACCACT**

**TCAAGAACTCTGTAGCACCGCCTACATACCTCGCTCTGCTAATCCTGTTACCAGTGGCTGCTGCCAGTGG**

**CGATAAGTCGTGTCTTACCGGGTTGGACTCAAGACGATAGTTACCGGATAAGGCGCAGCGGTCGGGCTGA**

**ACGGGGGGTTCGTGCACACAGCCCAGCTTGGAGCGAACGACCTACACCGAACTGAGATACCTACAGCGTG**

**AGCTATGAGAAAGCGCCACGCTTCCCGAAGGGAGAAAGGCGGACAGGTATCCGGTAAGCGGCAGGGTCGG**

**AACAGGAGAGCGCACGAGGGAGCTTCCAGGGGGAAACGCCTGGTATCTTTATAGTCCTGTCGGGTTTCGC**

**CACCTCTGACTTGAGCGTCGATTTTTGTGATGCTCGTCAGGGGGGCGGAGCCTATGGAAAAACGCCAGCA**

**ACGCGGCCTTTTTACGGTTCCTGGCCTTTTGCTGGCCTTTTGCTCACATGTTCTTTCCTGCGTTATCCCC**

**TGATTCTGTGGATAACCGTATTACCGCCTTTGAGTGAGCTGATACCGCTCGCCGCAGCCGAACGACCGAG**

**CGCAGCGAGTCAGTGAGCGAGGAAGCGGAAGAGCGCCTGATGCGGTATTTTCTCCTTACGCATCTGTGCG**

**GTATTTCACACCGCATATGGTGCACTCTCAGTACAATCTGCTCTGATGCCGCATAGTTAAGCCAGTATAC**

**ACTCCGCTATCGCTACGTGACTGGGTCATGGCTGCGCCCCGACACCCGCCAACACCCGCTGACGCGCCCT**

**GACGGGCTTGTCTGCTCCCGGCATCCGCTTACAGACAAGCTGTGACCGTCTCCGGGAGCTGCATGTGTCA**

**GAGGTTTTCACCGTCATCACCGAAACGCGCGAGGCAGGGTGCCTTGATGTGGGCGCCGGCGGTCGAGTGG**

**CGACGGCGCGGCTTGTCCGCGCCCTGGTAGATTGCCTGGCCGTAGGCCAGCCATTTTTGAGCGGCCAGCG**

**GCCGCGATAGGCCGACGCGAAGCGGCGGGGCGTAGGGAGCGCAGCGACCGAAGGGTAGGCGCTTTTTGCA**

**GCTCTTCGGCTGTGCGCTGGCCAGACAGTTATGCACAGGCCAGGCGGGTTTTAAGAGTTTTAATAAGTTT**

**TAAAGAGTTTTAGGCGGAAAAATCGCCTTTTTTCTCTTTTATATCAGTCACTTACATGTGTGACCGGTTC**

**CCAATGTACGGCTTTGGGTTCCCAATGTACGGGTTCCGGTTCCCAATGTACGGCTTTGGGTTCCCAATGT**

**ACGTGCTATCCACAGGAAAGAGACCTTTTCGACCTTTTTCCCCTGCTAGGGCAATTTGCCCTAGCATCTG**

**CTCCGTACATTAGGAACCGGCGGATGCTTCGCCCTCGATCAGGTTGCGGTAGCGCATGACTAGGATCGGG**

**CCAGCCTGCCCCGCCTCCTCCTTCAAATCGTACTCCGGCAGGTCATTTGACCCGATCAGCTTGCGCACGG**

**TGAAACAGAACTTCTTGAACTCTCCGGCGCTGCCACTGCGTTCGTAGATCGTCTTGAACAACCATCTGGC**

**TTCTGCCTTGCCTGCGGCGCGGCGTGCCAGGCGGTAGAGAAAACGGCCGATGCCGGGATCGATCAAAAAG**

**TAATCGGGGTGAACCGTCAGCACGTCCGGGTTCTTGCCTTCTGTGATCTCGCGGTACATCCAATCAGCTA**

**GCTCGATCTCGATGTACTCCGGCCGCCCGGTTTCGCTCTTTACGATCTTGTAGCGGCTAATCAAGGCTTC**

**ACCCTCGGATACCGTCACCAGGCGGCCGTTCTTGGCCTTCTTCGTACGCTGCATGGCAACGTGCGTGGTG**

**TTTAACCGAATGCAGGTTTCTACCAGGTCGTCTTTCTGCTTTCCGCCATCGGCTCGCCGGCAGAACTTGA**

**GTACGTCCGCAACGTGTGGACGGAACACGCGGCCGGGCTTGTCTCCCTTCCCTTCCCGGTATCGGTTCAT**

**GGATTCGGTTAGATGGGAAACCGCCATCAGTACCAGGTCGTAATCCCACACACTGGCCATGCCGGCCGGC**

**CCTGCGGAAACCTCTACGTGCCCGTCTGGAAGCTCGTAGCGGATCACCTCGCCAGCTCGTCGGTCACGCT**

**TCGACAGACGGAAAACGGCCACGTCCATGATGCTGCGACTATCGCGGGTGCCCACGTCATAGAGCATCGG**

**AACGAAAAAATCTGGTTGCTCGTCGCCCTTGGGCGGCTTCCTAATCGACGGCGCACCGGCTGCCGGCGGT**

**TGCCGGGATTCTTTGCGGATTCGATCAGCGGCCGCTTGCCACGATTCACCGGGGCGTGCTTCTGCCTCGA**

**TGCGTTGCCGCTGGGCGGCCTGCGCGGCCTTCAACTTCTCCACCAGGTCATCACCCAGCGCCGCGCCGAT**

**TTGTACCGGGCCGGATGGTTTGCGACCGTCACGCCGATTCCTCGGGCTTGGGGGTTCCAGTGCCATTGCA**

**GGGCCGGCAGACAACCCAGCCGCTTACGCCTGGCCAACCGCCCGTTCCTCCACACATGGGGCATTCCACG**

**GCGTCGGTGCCTGGTTGTTCTTGATTTTCCATGCCGCCTCCTTTAGCCGCTAAAATTCATCTACTCATTT**

**ATTCATTTGCTCATTTACTCTGGTAGCTGCGCGATGTATTCAGATAGCAGCTCGGTAATGGTCTTGCCTT**

**GGCGTACCGCGTACATCTTCAGCTTGGTGTGATCCTCCGCCGGCAACTGAAAGTTGACCCGCTTCATGGC**

**TGGCGTGTCTGCCAGGCTGGCCAACGTTGCAGCCTTGCTGCTGCGTGCGCTCGGACGGCCGGCACTTAGC**

**GTGTTTGTGCTTTTGCTCATTTTCTCTTTACCTCATTAACTCAAATGAGTTTTGATTTAATTTCAGCGGC**

**CAGCGCCTGGACCTCGCGGGCAGCGTCGCCCTCGGGTTCTGATTCAAGAACGGTTGTGCCGGCGGCGGCA**

**GTGCCTGGGTAGCTCACGCGCTGCGTGATACGGGACTCAAGAATGGGCAGCTCGTACCCGGCCAGCGCCT**

**CGGCAACCTCACCGCCGATGCGCGTGCCTTTGATCGCCCGCGACACGACAAAGGCCGCTTGTAGCCTTCC**

**ATCCGTGACCTCAATGCGCTGCTTAACCAGCTCCACCAGGTCGGCGGTGGCCCATATGTCGTAAGGGCTT**

**GGCTGCACCGGAATCAGCACGAAGTCGGCTGCCTTGATCGCGGACACAGCCAAGTCCGCCGCCTGGGGCG**

**CTCCGTCGATCACTACGAAGTCGCGCCGGCCGATGGCCTTCACGTCGCGGTCAATCGTCGGGCGGTCGAT**

**GCCGACAACGGTTAGCGGTTGATCTTCCCGCACGGCCGCCCAATCGCGGGCACTGCCCTGGGGATCGGAA**

**TCGACTAACAGAACATCGGCCCCGGCGAGTTGCAGGGCGCGGGCTAGATGGGTTGCGATGGTCGTCTTGC**

**CTGACCCGCCTTTCTGGTTAAGTACAGCGATAACCTTCATGCGTTCCCCTTGCGTATTTGTTTATTTACT**

**CATCGCATCATATACGCAGCGACCGCATGACGCAAGCTGTTTTACTCAAATACACATCACCTTTTTAGAC**

**GGCGGCGCTCGGTTTCTTCAGCGGCCAAGCTGGCCGGCCAGGCCGCCAGCTTGGCATCAGACAAACCGGC**

**CAGGATTTCATGCAGCCGCACGGTTGAGACGTGCGCGGGCGGCTCGAACACGTACCCGGCCGCGATCATC**

**TCCGCCTCGATCTCTTCGGTAATGAAAAACGGTTCGTCCTGGCCGTCCTGGTGCGGTTTCATGCTTGTTC**

**CTCTTGGCGTTCATTCTCGGCGGCCGCCAGGGCGTCGGCCTCGGTCAATGCGTCCTCACGGAAGGCACCG**

**CGCCGCCTGGCCTCGGTGGGCGTCACTTCCTCGCTGCGCTCAAGTGCGCGGTACAGGGTCGAGCGATGCA**

**CGCCAAGCAGTGCAGCCGCCTCTTTCACGGTGCGGCCTTCCTGGTCGATCAGCTCGCGGGCGTGCGCGAT**

**CTGTGCCGGGGTGAGGGTAGGGCGGGGGCCAAACTTCACGCCTCGGGCCTTGGCGGCCTCGCGCCCGCTC**

**CGGGTGCGGTCGATGATTAGGGAACGCTCGAACTCGGCAATGCCGGCGAACACGGTCAACACCATGCGGC**

**CGGCCGGCGTGGTGGTGTCGGCCCACGGCTCTGCCAGGCTACGCAGGCCCGCGCCGGCCTCCTGGATGCG**

**CTCGGCAATGTCCAGTAGGTCGCGGGTGCTGCGGGCCAGGCGGTCTAGCCTGGTCACTGTCACAACGTCG**

**CCAGGGCGTAGGTGGTCAAGCATCCTGGCCAGCTCCGGGCGGTCGCGCCTGGTGCCGGTGATCTTCTCGG**

**AAAACAGCTTGGTGCAGCCGGCCGCGTGCAGTTCGGCCCGTTGGTTGGTCAAGTCCTGGTCGTCGGTGCT**

**GACGCGGGCATAGCCCAGCAGGCCAGCGGCGGCGCTCTTGTTCATGGCGTAATGTCTCCGGTTCTAGTCG**

**CAAGTATTCTACTTTATGCGACTAAAACACGCGACAAGAAAACGCCAGGAAAAGGGCAGGGCGGCAGCCT**

**GTCGCGTAACTTAGGACTTGTGCGACATGTCGTTTTCAGAAGACGGCTGCACTGAACGTCAGAAGCCGAC**

**TGCACTATAGCAGCGGAGGGGTTGGATCAAAGTACTTTGATCCCGAGGGGAACCCTGTGGTTGGCATGCA**

**CATACAAATGGACGAACGGATAAACCTTTTCACGCCCTTTTAAATATCCGTTATTCTAATAAACGCTCTT**

**TTCTCTTAGGTTTACCCGCCAATATATCCTGTCAAACACTGATAGTTTAAACTGAAGGCGGGAAACGACA**

**ATCTGATCCAAGCTCAAGCTAAGCTCAGCTCGAGGATCTTATTCTAATTATATGACATTTGCAACTGTGA**

**AGGCAAGAATTTCTTACTCTATAATTTTTTAATTAAATATCTAATCTAAAATTTCTATAGTAAAATTGTG**

**ATTTTGTGCTCATATTCTCATATTTTTCAATGTCTTTGTTTTTCTTTCTTGTTTTTTATTTACTTTAGGG**

**AGGAGGGCACACAGCTCCTGAGTACAAACGTGAAGAGTGTTTTCATATGTTTAAAAGATGGATAACTCAA**

**CAACCTTTGTAAACATGTAACCCCATCGAAGATTAATTTATTAAATAGCCATTATTAAGCATCTGTCTTC**

**TTTTCTTTCCGATTTTTATGTGTGAGGGTGCAAAAATTAACTGTAAAAATAGTACGGGCTAGCCAGTTTT**

**CGGACTAATCATTCAAAATAGTCAACGTTTGTCAAGTCATTGAAAAATATCCGCTATTTTGCTGCAACAG**

**AAACCGTCCAGCATATATACTGGAGTTGGGTGCACATGTGTATGTATTTCCAGTACATTATGCTGGAACT**

**CCAACACGCGGAAAGTTCCAGCATAATATACTGGAGATTCGAGCACCTGTGTAAGAACTTCCAGAATATT**

**ATACTGGACCGATATAGTTTGCTGGAAATCCAGTATATTATGCTGGAGTTCTAGTATATTTATGCTGGAA**

**CTCCATTATATTATCCTGGAGTTCCAGTATACTTATGCTGGAACTCCAGTATAATATGCTGGAGTTTCAC**

**TATACTTATGCTGGAACTCTAGTATAATATACTGGAATATTTTCCGGATCTTGAACAATGTGTTCGTTCA**

**AATTTATCTTTACATGAAAAGTGACTAAATTTTAATTACTTTTGAAAGTGTGACTATTTTTGAATGAGCA**

**CTTGTAAATCTGGTTATTTTTGAATTTCTCCCGAATTAACTTAGTCTAACAATATCTTGTTCTGACTGGA**

**AAATTCAGTCTAATTAATTACTGCATTAACTATCTCTTCTTCTCTTTGTGAATTTTTTTTTTTTTTTTAT**

**AACAAATATGAGATAATATAAAAACCTCTAGTTCTTCTTTGAAAAACAGGTGAGATTCCAATTAAAACAA**

**AATGCCATTCTTGAACGATTTTGACAGGGCTTTTGCATCTATATCCACTTTTTGGGTCATATTTTAATTT**

**ATACCCGCTTTGCAAAAAAATTACAAGCGTATCCACTTTTCGCATAAACTTCAGGCTTACGGGTCTGGAG**

**TAGCAAAGGCAATCACACAAAGGTTCAGCATTCTAGGCTTTTTCGAAAACTTCAGCAGAATGCTGAAGTT**

**ATTTAGTTCATTTGTAAAAACTTCAGCACTAAATAAGCTGAAGTTTTGTCCTGGATTAAATAATTTTGTC**

**ATAAAGCTTTTTCAATAACTTCAGCAGAAGATGCTGAAGTTATTTAGTTCATTTATAAAAACTCCAGCAC**

**TAAATAAGCTGAAGTTTTTTCTTGAATTAATTAGTTTTGTCATAAAGCTTTTTCAAAAAACTTCAGTGCT**

**GAAGTTATTTAGTTCGTTTTTAAAATCTTCAGCAGAAGATGGTGAAGTTATTAAGTTCATTTGTAAAAAC**

**TTCAGCATCAGATAAGCTGAAGTTTTGTCCTGGATTCATTAGTTTTGCAGTAAAGCTTTTTCAAAAACTT**

**CAGCAGAAGATGCTGAAGTTATTTAGTTCATTTGTAAAAACTTTAGCACTAAATATGCTGAAGTTTTGCA**

**CAGGTATTAGAAAGGTGGCGCCTGAAATTGTAAAAATTAAGATATATATTAAATAATTTAAAAATAAAGG**

**TATAAATTAAATGAGAGCGATCAAATAAGGCGCCTGCGCAATTTTTGATGTCAATTAGGTAGCATCAAGT**

**TAATTTTGCACAATTTTTGCGTTTCTCTATTTAGATTGTTTGAAAAAATGACAACTTTAATAAATTGCCG**

**AAATAATAAAAAAATAAACAAGTTGACAGTTACCTCTTTCTCCTCCCGTACAACCTTTTCACCACCACCC**

**CTCCATGTCCATGATTTGTTGGTCCCTAAAGTTTAAATAATAATAAATAAATAAATAAAAATTGTAATTA**

**AAATTTAGAGATCAACTTTGGTCGTTAAATATATATTATTAAAATATTATACCGACCGAAGTTGGTCGGT**

**ATTTTATTTATCCTAAATATTTGGTTCTTTTAACTTAGTGACCAACGTTGGTCGCTAAATTAAAAGGACC**

**ACCAATATAGCGACCAATCCATTTTGGACGCGTTTTGGTCGGTATATTGTGATAAGCGACCAACTTTGGT**

**CGCTATTTGTGGTCTCTTTTTGCCGGATTTCTAGCAGTGTGTACACGCAAATCGAAAAGGATAAAATGAG**

**ATTTTTAAGGCTAACGAGTGCAGAATTAAATTTTAAAACGTAAGTTTAGGTCATCACATATTATGTGATT**

**TTTAAAAAAATGATCTTCATATAGAATACACACGTAACACGCTTGCCCAAAAACTATTAGAACAAAATAA**

**GTAACGGCTATTTTTAAACCTTCAATCCGTAGCAGCCCACTAATCCCTGGCTCCAATTTTCTTCAATAAT**

**AAGTTGTATGCAGAAGGAAAAAGATTGTTCCTAGAAGTTGTATGCGATACTAAACACCTTCCCCCTGTTA**

**TTTTTCTGTCTGTTTTCTTTAAAGCAACGAATCCTGTGCCTTGATTCTTTTCTTGTTTCCTGTGTTAGTT**

**ATAAGTTTCAATAATGAAAAATAATATATTATATTGGGCGTAGGATCACAAGGGATTCAAGAAGCAACAC**

**TAGTCGGGAATAGATAAAGGAACATAATCAATAATCAGCATGGAAAAGGAAGAAGTAGCGAAAATTCGGC**

**AAGAATAATCAATTTAATTAATTACAGTAGCTAATTCTTATATATTAAGTTTCTGAGAAAAGTAACATTT**

**CTTCACATTTATGGACCTACATTTGTTGTCACTTTCTATCTGCGCAAAGAAAAATAAGACCATAGTACTG**

**CTTTTGGTTAGTACAACTGTTGACAAAGAAAATTACTGGGATATTACCCTTCGTTTTCTTTGTAGCTTTA**

**TTTATCGGCTTGTACTTTTAGTTGTTCCTTGTGAACATATTACTGTTGAATTTGGTGCAGGGAGGGTGGG**

**TGGTCTTTGAAGGAATTACCTACTTCCCTTCTATTACAGTGCAAAGAAAACCCTATAACAATAATAATTC**

**TAATCAACTGGAGTAAACATTAAGATGAAGCTTCACAAAAAAATCCTACAATTTACTTTCTATTAGGAGT**

**AGTCGGTGGCGGATTTAGGATTTTGCGAATATGAGTGCACTATTACGAAGAGGCGAATCTAGGATATAAA**

**TTTTACAGGTTTAACGTTTGGTTCTTACTATTGCACCCATTACAATTTTGAAATTATAAGTTCAAAATTA**

**TTATTTTTTAATTGTAATTTTCTTATATCTATTTCCATACTCCGTACTTAAAATATTGGGATCAGTTTAA**

**CCCAATAGCATACACTGCATTATGCACTAGTTTAATATGCAAATTTTATTTAATCATATAAGATTTTTCG**

**GTGACAAATAACAAATAGGAATTTTAATATGTGAAAATTTTAAAAGAATAAATCAAAAAGAAAGAAAGAA**

**AGAAAAAGAAATGTATTTAATTAATACGCACCAAGTGATGCCTAGTTTTAGAAAAGAAAAAATAACAATA**

**AGATTGTCATAGGAAAAAGGATTGAAAGGTCGACCAGATAATTTTTTTTTTTTTTTTTTTACCAGAATGA**

**TATGTTCCACAATATATTGTACAATTTTGTCGAAACTTTATAATAACTTTCTTAACGTTAATAAATTGGG**

**AACAAGTTTACGATTAAATTTCACATGTGATCATTCAACTTTGTGTTTATTATCCAACAAAAATGAAAAA**

**TATTTTGCTAGATGAAGACTTTGTCATCCTCGGTAGAAAACTAAAATAGAAAAAGAATTCAATCAATGGA**

**GACCTTTTTCTCTTTGGAGCAATAATTCAATTCAATTGGGAAGGAATTTCCTACTCCCTTCTATTAAAGT**

**TCAAAGAAAACCCAATAATTCCTTTTATTGCATTAAGAAGAATTTTCCTACTAGTGTATATCAGTTGACT**

**AGGACACCAATAATTCTATGGAGTAGAGCCCATCTCACACAAACTTTTTCCAATAGCAATATAACTCTTA**

**TCTCTTCTAATATATATAAAAATTTGTTGAAAATATCATCTATTATTTTCTTACCACAATCACAATTTTT**

**TCACATACAGTTTCTTATTCTTTTCAGAGAATTAACGTTGAGATGGGATTACGTCCTGTAGAAACCCCAA**

**CCCGTGAAATCAAAAAACTCGACGGCCTGTGGGCATTCAGTCTGGATCGCGAAAACTGTGGAATTGATCA**

**GCGTTGGTGGGAAAGCGCGTTACAAGAAAGCCGGGCAATTGCTGTGCCAGGCAGTTTTAACGATCAGTTC**

**GCCGATGCAGATATTCGTAATTATGCGGGCAACGTCTGGTATCAGCGCGAAGTCTTTATACCGAAAGGTT**

**GGGCAGGCCAGCGTATCGTGCTGCGTTTCGATGCGGTCACTCATTACGGCAAAGTGTGGGTCAATAATCA**

**GGAAGTGATGGAGCATCAGGGCGGCTATACGCCATTTGAAGCCGATGTCACGCCGTATGTTATTGCCGGG**

**AAAAGTGTACGTATCACCGTTTGTGTGAACAACGAACTGAACTGGCAGACTATCCCGCCGGGAATGGTGA**

**TTACCGACGAAAACGGCAAGAAAAAGCAGTCTTACTTCCATGATTTCTTTAACTATGCCGGAATCCATCG**

**CAGCGTAATGCTCTACACCACGCCGAACACCTGGGTGGACGATATCACCGTGGTGACGCATGTCGCGCAA**

**GACTGTAACCACGCGTCTGTTGACTGGCAGGTGGTGGCCAATGGTGATGTCAGCGTTGAACTGCGTGATG**

**CGGATCAACAGGTGGTTGCAACTGGACAAGGCACTAGCGGGACTTTGCAAGTGGTGAATCCGCACCTCTG**

**GCAACCGGGTGAAGGTTATCTCTATGAACTGTGCGTCACAGCCAAAAGCCAGACAGAGTGTGATATCTAC**

**CCGCTTCGCGTCGGCATCCGGTCAGTGGCAGTGAAGGGCCAACAGTTCCTGATTAACCACAAACCGTTCT**

**ACTTTACTGGCTTTGGTCGTCATGAAGATGCGGACTTACGTGGCAAAGGATTCGATAACGTGCTGATGGT**

**GCACGACCACGCATTAATGGACTGGATTGGGGCCAACTCCTACCGTACCTCGCATTACCCTTACGCTGAA**

**GAGATGCTCGACTGGGCAGATGAACATGGCATCGTGGTGATTGATGAAACTGCTGCTGTCGGCTTTCAGC**

**TGTCTTTAGGCATTGGTTTCGAAGCGGGCAACAAGCCGAAAGAACTGTACAGCGAAGAGGCAGTCAACGG**

**GGAAACTCAGCAAGCGCACTTACAGGCGATTAAAGAGCTGATAGCGCGTGACAAAAACCACCCAAGCGTG**

**GTGATGTGGAGTATTGCCAACGAACCGGATACCCGTCCGCAAGGTGCACGGGAATATTTCGCGCCACTGG**

**CGGAAGCAACGCGTAAACTCGACCCGACGCGTCCGATCACCTGCGTCAATGTAATGTTCTGCGACGCTCA**

**CACCGATACCATCAGCGATCTCTTTGATGTGCTGTGCCTGAACCGTTATTACGGATGGTATGTCCAAAGC**

**GGCGATTTGGAAACGGCAGAGAAGGTACTGGAAAAAGAACTTCTGGCCTGGCAGGAGAAACTGCATCAGC**

**CGATTATCATCACCGAATACGGCGTGGATACGTTAGCCGGGCTGCACTCAATGTACACCGACATGTGGAG**

**TGAAGAGTATCAGTGTGCATGGCTGGATATGTATCACCGCGTCTTTGATCGCGTCAGCGCCGTCGTCGGT**

**GAACAGGTATGGAATTTCGCCGATTTTGCGACCTCGCAAGGCATATTGCGCGTTGGCGGTAACAAGAAAG**

**GGATCTTCACTCGCGACCGCAAACCGAAGTCGGCGGCTTTTCTGCTGCAAAAACGCTGGACTGGCATGAA**

**CTTCGGTGAAAAACCGCAGCAGGGAGGCAAACAAGCTAGCCACCACCACCACCACCACGTGGAACAAAAG**

**CTAATCTCCGAGGAAGACTTGAACGGTGAACAAAAATTAATCTCAGAAGAAGACTTGAACGGACATCACC**

**ATCACCATCACTGATGTACATATCAACAACGAGTTTTTAAAGGATTCCAACAAGTATAACTTTTTATGCT**

**CAAATCAGCTCCTTGTATTGTGGAGAAAGCTGAGTACGAGATGAAGTTGACGTCCGTTATCCTTTATGAT**

**CTCTCTGTTCTTTGTGTTAACTTGCCTACTTCATCAGATGAATAACAGAAGCCCGTTCCTCTCATTCTCA**

**ACACTGTTTGCACGTCTGTTGTTACTTGTTAAAATGGATCTTGATAAAGTAATAACATCTCTATATTACT**

**TATAAGTGGTTTTAACAAGTTCACTCTTTTGCTTTTGCAGTTCAAATGGGAACACAATGTATATTGAGAA**

**CTAGAACAATGACACTGCATATATATATATATATGTATGTATGTAATTCTCGTCTTTTGGACTAGAATAC**

**CTTGTTTCATTATGAAATGAATTAACATCTTCGCCTTTGCTGACAAGTAACCAATTACAGATGAATGAAA**

**TCACCTGATCAACATTCATTAGCTTTGTATTCTTTGACGATTTCGGTTTCATAACTCTTTCCCCTGCAGT**

**TAAAATATGTAGTTAGCCCGATTGCACCTCTAGGGCGCAGCGGAGTATTAAAAAAAAAAAGATCTTTCTC**

**ATTTGTCTAAGTCTTGGTAGTCAGAATTACGAGTTTGTATAAAGTTGGCTCAAACATCACCTTTGTATAA**

**GAAAAATACATACACACACAGTAGAAAGAAACAGATACCTTCGCAAATTTGATTGGGAGGTACTGATTTC**

**TTCTTTCAGTTGGCGATTAGCCTCTTGTGTCATCTTTGGAGCTTCTTATGATTTTTTTTTCTTAGGTAAA**

**ATTCATTTAATAATTTGTTAATCATATTACTGTTGGGCTAAACTAGCCCCGATACACTCATAACATGGTG**

**TGATATTGTTCGCTTTGGGCCAAGCCCGTATGGTTTTCCCCAAAAGGCCTCGCACCATTAAGAGATCCAT**

**ACACCTTAAATGTAGACTCACAATCTTTTCAGCTATTAATGTGGCACTTTATTCGCATACCCAACATTAT**

**GTCTACACTACAGGAATTAGAGTTGGAACAGAGTTTTAAAACTAGTCAAAGAGTTTTGGAGCTAACAAAA**

**CTATCTTGATAAATATAATACAAACAACTCGTAGTGTTCAGAGGCGGAATAACTATGTGATTACTGTAGA**

**AACTTATAAACTTTAAATTTTGGATTCGCATTTGCTTACCGTTGATTTTCTATCTCATTTATCTTGGCTG**

**GTTGTGCCATAATTAAATCCATTGGAGGGACATTGTAGGATTAGCTTACGTAAATGTGCTTGTAAATTGA**

**ATAACGTGAGCTAACATTGTTGAC**

**>nta-MIR6019, 6020proa-2k::Exon1a (1bp - 11550bp, direct) 11550bp**

**GGGTACCCTCGAATTATCATACATGAGAATTAAGGGAGTCACGTTATGACCCCGCCGATGACGCGGGACA**

**AGCCGTTTTACGTTTGGAACTGACAGAACCGCAACGTTGAAGGAGCCACTGAGCCGCGGGTTTCTGGAGT**

**TTAATGAGCTAAGCACATACGTCAGAAACCATTATTGCGCGTTCAAAAGTCGCCTAAGGTCACTATCAGC**

**TAGCAAATATTTCTTGTCAAAAATGCTCCACTGACGTTCCATAAATTCCCCTCGGTATCCAATTAGAGTC**

**TCATATTCACTCTCAACTCGAATCCCCCCTATGAAAAAGCCTGAACTCACCGCGACGTCTGTCGAGAAGT**

**TTCTGATCGAAAAGTTCGACAGCGTCTCCGACCTGATGCAGCTCTCGGAGGGCGAAGAATCTCGTGCTTT**

**CAGCTTCGATGTAGGAGGGCGTGGATATGTCCTGCGGGTAAATAGCTGCGCCGATGGTTTCTACAAAGAT**

**CGTTATGTTTATCGGCACTTTGCATCGGCCGCGCTCCCGATTCCGGAAGTGCTTGACATTGGGGAGTTCA**

**GCGAGAGCCTGACCTATTGCATCTCCCGCCGTGCACAGGGTGTCACGTTGCAAGACCTGCCTGAAACCGA**

**ACTGCCCGCTGTTCTTCAGCCGGTCGCGGAGGCTATGGATGCTATCGCTGCGGCCGATCTTAGCCAGACG**

**AGCGGGTTCGGCCCATTCGGACCGCAAGGAATCGGTCAATACACTACATGGCGTGATTTCATATGCGCGA**

**TTGCTGATCCCCATGTGTATCACTGGCAAACTGTGATGGACGACACCGTCAGTGCGTCCGTCGCGCAGGC**

**TCTCGATGAGCTGATGCTTTGGGCGAGGACTGCCCCGAAGTCCGGCACTCGTGCACGCGGATTCGGCTCC**

**AACAATGTCCTGACGGACAATGGCCGCATAACAGCGGTCATTGACTGGAGCGAGGCGATGTTCGGGGATT**

**CCCAATACGAGGTCGCCAACATCTTCTTCTGGAGGCCGTGGTTGGCTTGTATGGAGCAGCAGACGCGCTA**

**CTTCGAGCGGAGGCATCCGGAGCTTGCAGGATCGCCAAGGCTCCGGGCGTATATGCTCCGCATTGGTCTT**

**GACCAACTCTATCAGAGCTTGGTTGACGGCAATTTCGATGATGCAGCTTGGGCGCAGGGTCGATGCGACG**

**CAATCGTCCGATCCGGAGCCGGGACTGTCGGGCGTACACAAATCGCCCGCAGAAGCGCGGCCGTCTGGAC**

**CGATGGCTGTGTAGAAGTACTCGCCGATAGTGGAAACCGACGCCCCAGCACTCGTCCGAGGGCAAAGGAA**

**TAGAGTAGATGCCGACCGAACAAGAGCTGATTTCGAGAACGCCTCAGCCAGCAACTCGCGCGAGCCTAGC**

**AAGGCAAATGCGAGAGAACGGCCTTACGCTTGGTGGCACAGTTCTCGTCCACAGTTCGCTAAGCTCGCTC**

**GGCTGGGTCGCGGGAGGGCCGGTCGCAGTGATTCAGGAATTAATTCCCTAGAGTCAAGCAGATCGTTCAA**

**ACATTTGGCAATAAAGTTTCTTAAGATTGAATCCTGTTGCCGGTCTTGCGATGATTATCATATAATTTCT**

**GTTGAATTACGTTAAGCATGTAATAATTAACATGTAATGCATGACGTTATTTATGAGATGGGTTTTTATG**

**ATTAGAGTCCCGCAATTATACATTTAATACGCGATAGAAAACAAAATATAGCGCGCAAACTAGGATAAAT**

**TATCGCGCGCGGTGTCATCTATGTTACTAGATCGACCGGCATGCAAGCTGATAATTCAATTCGGCGTTAA**

**TTCAGTACATTAAAAACGTCCGCAATGTGTTATTAAGTTGTCTAAGCGTCAATTTGTTTACACCACAATA**

**TATCCTGCCACCAGCCAGCCAACAGCTCCCCGACCGGCAGCTCGGCACAAAATCACCACTCGATACAGGC**

**AGCCCATCAGTCCGGGACGGCGTCAGCGGGAGAGCCGTTGTAAGGCGGCAGACTTTGCTCATGTTACCGA**

**TGCTATTCGGAAGAACGGCAACTAAGCTGCCGGGTTTGAAACACGGATGATCTCGCGGAGGGTAGCATGT**

**TGATTGTAACGATGACAGAGCGTTGCTGCCTGTGATCAATTCGGGCACGAACCCAGTGGACATAAGCCTC**

**GTTCGGTTCGTAAGCTGTAATGCAAGTAGCGTAACTGCCGTCACGCAACTGGTCCAGAACCTTGACCGAA**

**CGCAGCGGTGGTAACGGCGCAGTGGCGGTTTTCATGGCTTCTTGTTATGACATGTTTTTTTGGGGTACAG**

**TCTATGCCTCGGGCATCCAAGCAGCAAGCGCGTTACGCCGTGGGTCGATGTTTGATGTTATGGAGCAGCA**

**ACGATGTTACGCAGCAGGGCAGTCGCCCTAAAACAAAGTTAAACATCATGGGGGAAGCGGTGATCGCCGA**

**AGTATCGACTCAACTATCAGAGGTAGTTGGCGTCATCGAGCGCCATCTCGAACCGACGTTGCTGGCCGTA**

**CATTTGTACGGCTCCGCAGTGGATGGCGGCCTGAAGCCACACAGTGATATTGATTTGCTGGTTACGGTGA**

**CCGTAAGGCTTGATGAAACAACGCGGCGAGCTTTGATCAACGACCTTTTGGAAACTTCGGCTTCCCCTGG**

**AGAGAGCGAGATTCTCCGCGCTGTAGAAGTCACCATTGTTGTGCACGACGACATCATTCCGTGGCGTTAT**

**CCAGCTAAGCGCGAACTGCAATTTGGAGAATGGCAGCGCAATGACATTCTTGCAGGTATCTTCGAGCCAG**

**CCACGATCGACATTGATCTGGCTATCTTGCTGACAAAAGCAAGAGAACATAGCGTTGCCTTGGTAGGTCC**

**AGCGGCGGAGGAACTCTTTGATCCGGTTCCTGAACAGGATCTATTTGAGGCGCTAAATGAAACCTTAACG**

**CTATGGAACTCGCCGCCCGACTGGGCTGGCGATGAGCGAAATGTAGTGCTTACGTTGTCCCGCATTTGGT**

**ACAGCGCAGTAACCGGCAAAATCGCGCCGAAGGATGTCGCTGCCGACTGGGCAATGGAGCGCCTGCCGGC**

**CCAGTATCAGCCCGTCATACTTGAAGCTAGACAGGCTTATCTTGGACAAGAAGAAGATCGCTTGGCCTCG**

**CGCGCAGATCAGTTGGAAGAATTTGTCCACTACGTGAAAGGCGAGATCACCAAGGTAGTCGGCAAATAAT**

**GTCTAGCTAGAAATTCGTTCAAGCCGACGCCGCTTCGCCGGCGTTAACTCAAGCGATTAGATGCACTAAG**

**CACATAATTGCTCACAGCCAAACTATCAGGTCAAGTCTGCTTTTATTATTTTTAAGCGTGCATAATAAGC**

**CCTACACAAATTGGGAGATATATCATGCATGACCAAAATCCCTTAACGTGAGTTTTCGTTCCACTGAGCG**

**TCAGACCCCGTAGAAAAGATCAAAGGATCTTCTTGAGATCCTTTTTTTCTGCGCGTAATCTGCTGCTTGC**

**AAACAAAAAAACCACCGCTACCAGCGGTGGTTTGTTTGCCGGATCAAGAGCTACCAACTCTTTTTCCGAA**

**GGTAACTGGCTTCAGCAGAGCGCAGATACCAAATACTGTCCTTCTAGTGTAGCCGTAGTTAGGCCACCAC**

**TTCAAGAACTCTGTAGCACCGCCTACATACCTCGCTCTGCTAATCCTGTTACCAGTGGCTGCTGCCAGTG**

**GCGATAAGTCGTGTCTTACCGGGTTGGACTCAAGACGATAGTTACCGGATAAGGCGCAGCGGTCGGGCTG**

**AACGGGGGGTTCGTGCACACAGCCCAGCTTGGAGCGAACGACCTACACCGAACTGAGATACCTACAGCGT**

**GAGCTATGAGAAAGCGCCACGCTTCCCGAAGGGAGAAAGGCGGACAGGTATCCGGTAAGCGGCAGGGTCG**

**GAACAGGAGAGCGCACGAGGGAGCTTCCAGGGGGAAACGCCTGGTATCTTTATAGTCCTGTCGGGTTTCG**

**CCACCTCTGACTTGAGCGTCGATTTTTGTGATGCTCGTCAGGGGGGCGGAGCCTATGGAAAAACGCCAGC**

**AACGCGGCCTTTTTACGGTTCCTGGCCTTTTGCTGGCCTTTTGCTCACATGTTCTTTCCTGCGTTATCCC**

**CTGATTCTGTGGATAACCGTATTACCGCCTTTGAGTGAGCTGATACCGCTCGCCGCAGCCGAACGACCGA**

**GCGCAGCGAGTCAGTGAGCGAGGAAGCGGAAGAGCGCCTGATGCGGTATTTTCTCCTTACGCATCTGTGC**

**GGTATTTCACACCGCATATGGTGCACTCTCAGTACAATCTGCTCTGATGCCGCATAGTTAAGCCAGTATA**

**CACTCCGCTATCGCTACGTGACTGGGTCATGGCTGCGCCCCGACACCCGCCAACACCCGCTGACGCGCCC**

**TGACGGGCTTGTCTGCTCCCGGCATCCGCTTACAGACAAGCTGTGACCGTCTCCGGGAGCTGCATGTGTC**

**AGAGGTTTTCACCGTCATCACCGAAACGCGCGAGGCAGGGTGCCTTGATGTGGGCGCCGGCGGTCGAGTG**

**GCGACGGCGCGGCTTGTCCGCGCCCTGGTAGATTGCCTGGCCGTAGGCCAGCCATTTTTGAGCGGCCAGC**

**GGCCGCGATAGGCCGACGCGAAGCGGCGGGGCGTAGGGAGCGCAGCGACCGAAGGGTAGGCGCTTTTTGC**

**AGCTCTTCGGCTGTGCGCTGGCCAGACAGTTATGCACAGGCCAGGCGGGTTTTAAGAGTTTTAATAAGTT**

**TTAAAGAGTTTTAGGCGGAAAAATCGCCTTTTTTCTCTTTTATATCAGTCACTTACATGTGTGACCGGTT**

**CCCAATGTACGGCTTTGGGTTCCCAATGTACGGGTTCCGGTTCCCAATGTACGGCTTTGGGTTCCCAATG**

**TACGTGCTATCCACAGGAAAGAGACCTTTTCGACCTTTTTCCCCTGCTAGGGCAATTTGCCCTAGCATCT**

**GCTCCGTACATTAGGAACCGGCGGATGCTTCGCCCTCGATCAGGTTGCGGTAGCGCATGACTAGGATCGG**

**GCCAGCCTGCCCCGCCTCCTCCTTCAAATCGTACTCCGGCAGGTCATTTGACCCGATCAGCTTGCGCACG**

**GTGAAACAGAACTTCTTGAACTCTCCGGCGCTGCCACTGCGTTCGTAGATCGTCTTGAACAACCATCTGG**

**CTTCTGCCTTGCCTGCGGCGCGGCGTGCCAGGCGGTAGAGAAAACGGCCGATGCCGGGATCGATCAAAAA**

**GTAATCGGGGTGAACCGTCAGCACGTCCGGGTTCTTGCCTTCTGTGATCTCGCGGTACATCCAATCAGCT**

**AGCTCGATCTCGATGTACTCCGGCCGCCCGGTTTCGCTCTTTACGATCTTGTAGCGGCTAATCAAGGCTT**

**CACCCTCGGATACCGTCACCAGGCGGCCGTTCTTGGCCTTCTTCGTACGCTGCATGGCAACGTGCGTGGT**

**GTTTAACCGAATGCAGGTTTCTACCAGGTCGTCTTTCTGCTTTCCGCCATCGGCTCGCCGGCAGAACTTG**

**AGTACGTCCGCAACGTGTGGACGGAACACGCGGCCGGGCTTGTCTCCCTTCCCTTCCCGGTATCGGTTCA**

**TGGATTCGGTTAGATGGGAAACCGCCATCAGTACCAGGTCGTAATCCCACACACTGGCCATGCCGGCCGG**

**CCCTGCGGAAACCTCTACGTGCCCGTCTGGAAGCTCGTAGCGGATCACCTCGCCAGCTCGTCGGTCACGC**

**TTCGACAGACGGAAAACGGCCACGTCCATGATGCTGCGACTATCGCGGGTGCCCACGTCATAGAGCATCG**

**GAACGAAAAAATCTGGTTGCTCGTCGCCCTTGGGCGGCTTCCTAATCGACGGCGCACCGGCTGCCGGCGG**

**TTGCCGGGATTCTTTGCGGATTCGATCAGCGGCCGCTTGCCACGATTCACCGGGGCGTGCTTCTGCCTCG**

**ATGCGTTGCCGCTGGGCGGCCTGCGCGGCCTTCAACTTCTCCACCAGGTCATCACCCAGCGCCGCGCCGA**

**TTTGTACCGGGCCGGATGGTTTGCGACCGTCACGCCGATTCCTCGGGCTTGGGGGTTCCAGTGCCATTGC**

**AGGGCCGGCAGACAACCCAGCCGCTTACGCCTGGCCAACCGCCCGTTCCTCCACACATGGGGCATTCCAC**

**GGCGTCGGTGCCTGGTTGTTCTTGATTTTCCATGCCGCCTCCTTTAGCCGCTAAAATTCATCTACTCATT**

**TATTCATTTGCTCATTTACTCTGGTAGCTGCGCGATGTATTCAGATAGCAGCTCGGTAATGGTCTTGCCT**

**TGGCGTACCGCGTACATCTTCAGCTTGGTGTGATCCTCCGCCGGCAACTGAAAGTTGACCCGCTTCATGG**

**CTGGCGTGTCTGCCAGGCTGGCCAACGTTGCAGCCTTGCTGCTGCGTGCGCTCGGACGGCCGGCACTTAG**

**CGTGTTTGTGCTTTTGCTCATTTTCTCTTTACCTCATTAACTCAAATGAGTTTTGATTTAATTTCAGCGG**

**CCAGCGCCTGGACCTCGCGGGCAGCGTCGCCCTCGGGTTCTGATTCAAGAACGGTTGTGCCGGCGGCGGC**

**AGTGCCTGGGTAGCTCACGCGCTGCGTGATACGGGACTCAAGAATGGGCAGCTCGTACCCGGCCAGCGCC**

**TCGGCAACCTCACCGCCGATGCGCGTGCCTTTGATCGCCCGCGACACGACAAAGGCCGCTTGTAGCCTTC**

**CATCCGTGACCTCAATGCGCTGCTTAACCAGCTCCACCAGGTCGGCGGTGGCCCATATGTCGTAAGGGCT**

**TGGCTGCACCGGAATCAGCACGAAGTCGGCTGCCTTGATCGCGGACACAGCCAAGTCCGCCGCCTGGGGC**

**GCTCCGTCGATCACTACGAAGTCGCGCCGGCCGATGGCCTTCACGTCGCGGTCAATCGTCGGGCGGTCGA**

**TGCCGACAACGGTTAGCGGTTGATCTTCCCGCACGGCCGCCCAATCGCGGGCACTGCCCTGGGGATCGGA**

**ATCGACTAACAGAACATCGGCCCCGGCGAGTTGCAGGGCGCGGGCTAGATGGGTTGCGATGGTCGTCTTG**

**CCTGACCCGCCTTTCTGGTTAAGTACAGCGATAACCTTCATGCGTTCCCCTTGCGTATTTGTTTATTTAC**

**TCATCGCATCATATACGCAGCGACCGCATGACGCAAGCTGTTTTACTCAAATACACATCACCTTTTTAGA**

**CGGCGGCGCTCGGTTTCTTCAGCGGCCAAGCTGGCCGGCCAGGCCGCCAGCTTGGCATCAGACAAACCGG**

**CCAGGATTTCATGCAGCCGCACGGTTGAGACGTGCGCGGGCGGCTCGAACACGTACCCGGCCGCGATCAT**

**CTCCGCCTCGATCTCTTCGGTAATGAAAAACGGTTCGTCCTGGCCGTCCTGGTGCGGTTTCATGCTTGTT**

**CCTCTTGGCGTTCATTCTCGGCGGCCGCCAGGGCGTCGGCCTCGGTCAATGCGTCCTCACGGAAGGCACC**

**GCGCCGCCTGGCCTCGGTGGGCGTCACTTCCTCGCTGCGCTCAAGTGCGCGGTACAGGGTCGAGCGATGC**

**ACGCCAAGCAGTGCAGCCGCCTCTTTCACGGTGCGGCCTTCCTGGTCGATCAGCTCGCGGGCGTGCGCGA**

**TCTGTGCCGGGGTGAGGGTAGGGCGGGGGCCAAACTTCACGCCTCGGGCCTTGGCGGCCTCGCGCCCGCT**

**CCGGGTGCGGTCGATGATTAGGGAACGCTCGAACTCGGCAATGCCGGCGAACACGGTCAACACCATGCGG**

**CCGGCCGGCGTGGTGGTGTCGGCCCACGGCTCTGCCAGGCTACGCAGGCCCGCGCCGGCCTCCTGGATGC**

**GCTCGGCAATGTCCAGTAGGTCGCGGGTGCTGCGGGCCAGGCGGTCTAGCCTGGTCACTGTCACAACGTC**

**GCCAGGGCGTAGGTGGTCAAGCATCCTGGCCAGCTCCGGGCGGTCGCGCCTGGTGCCGGTGATCTTCTCG**

**GAAAACAGCTTGGTGCAGCCGGCCGCGTGCAGTTCGGCCCGTTGGTTGGTCAAGTCCTGGTCGTCGGTGC**

**TGACGCGGGCATAGCCCAGCAGGCCAGCGGCGGCGCTCTTGTTCATGGCGTAATGTCTCCGGTTCTAGTC**

**GCAAGTATTCTACTTTATGCGACTAAAACACGCGACAAGAAAACGCCAGGAAAAGGGCAGGGCGGCAGCC**

**TGTCGCGTAACTTAGGACTTGTGCGACATGTCGTTTTCAGAAGACGGCTGCACTGAACGTCAGAAGCCGA**

**CTGCACTATAGCAGCGGAGGGGTTGGATCAAAGTACTTTGATCCCGAGGGGAACCCTGTGGTTGGCATGC**

**ACATACAAATGGACGAACGGATAAACCTTTTCACGCCCTTTTAAATATCCGTTATTCTAATAAACGCTCT**

**TTTCTCTTAGGTTTACCCGCCAATATATCCTGTCAAACACTGATAGTTTAAACTGAAGGCGGGAAACGAC**

**AATCTGATCCAAGCTCAAGCTAAGCTTGAGCTCTGCATGCCTGCAGGTCCCAGGGCGCCCTTGTTTTGGT**

**CAATTTTACGGGGAAGAAGGAAAATTGCAAAGTTCATTATAGAATTTTCTTTTCAGTCGTAATCGCAAAA**

**GCTCGTGTTGATATAATCCTATCTATATTTTTGTCTTTAACTGTTTGGCTAAGGTTCTTTATGATATTAA**

**TATTTTCAATTGCAATCGGCTCACTTCTTAGAGCAGTTTGATGGATGTAGTAGGTGGCAGTGACGGGTAG**

**GGAGGATTACAGTGTGCATAGTTTCTGTTGAAGTGCTTTGACTGGTGGCTCTGTTGTTGGAGCATGTATT**

**TTCCTTCAGTGATTGATCGGCTTTCTTAGAGTTGGAAGTAATGTAAATTGCCTTTGACTGGTTTTCTTAA**

**GCAGGTAATGAGGGGTTAATATTGCTTAATGGTGTTGAATCTTTTGAAAGAAAACTACACTATCCAGGTT**

**CTGTTTAAGTATTAAAATTGCAATAAAAAACAGTAGGTAAATGGATTCAAGTTGCATTTTAACCTTTTAA**

**GTGCCTGCCTCTATTTTTTTTTCCCATTCAAAATTGCTCTCGTCTCAATGTCCTAAGACATTTTGAAAGA**

**ATTGAGAATGTCTTTCTGGACAGACGATTGACACCTCAATGGAGTAAAGATATTAGGGGTATGGAACTTT**

**AATTCTAGTCCTTCTGCTTTGTTCTTAGGCAGACCCCCCTGTAGGCCTATCGACCAATGACTCGTGATCC**

**CAACTTGCGTAATGGAAAAATTGGAGTAATAGAACAATTGATTTCGTTGTGGTGGTAAAAGATATCACGT**

**ATCTTGATTGTGTACAAAGGCAACTTGGTCTCTGGCTATAAAGTTTCCAGTATTTTGCACCGCTTGTGAA**

**TTATAAGACGATCTTATACCAGAAAATGAAACATTGGACATAACTTTGGAACCCCCCCCCCACTGGCTGC**

**AAGGAAATAACGGATTGGATTTTCTAACTCTCTTTAAAGAGACTGGTTTCGAATTCACTTGCTTGCAAGC**

**TTAGGGTTAGCAGGTTGTTGACTGGGAGAGCTGGTAAAAGAATAAAATCTTCAAGAGATCATGGTTAACA**

**AGCCAGCTGAGGAATAACAATCTTCCTCAGCAATACCAAAAATCCATCCAAGGAATGAGCTTCCCCAAAG**

**TTAATTCGCGTGGACTACAAAGAAAAGATAGAGTCTCACCCGGTAACATCAGCTGAGTCAATGGCAGAAA**

**GTCTTGAAAAGAACCTTTTATTCGATGCTTCTGATTCAAGCTTATTCCTTTCCGCCTTTTGAATAAATCT**

**AGAACCTCAATTAGCAGATTCATTAGAAACCTTTCGACCAACATAATCTTTGAATTTTCCGCATATATAA**

**CCAGCAGAGCTCTCGTATGAGCAAAGTAACGCCTTCGTCCTTTTTCTCCATGTATGCTTAGGAATCACAC**

**CAATCTATCCCGAATTTGGTGGTTAAACTCTACTGGTATGAGAAGGGTCCTCCTCTCCCTTGTCAAATAA**

**AAATGACACACTATGTGGGCGGATGGAGAATAACTTTAAGGTCTACATTAAAGAAAAAAAAAGAGCAAAA**

**AGGATAAGATTTAAGTGATTTTTGGTAGTTATTTTCAATTACAATAAAGTCAAATCGTCGTACAACTGTT**

**GTTAGTCATAAAGAAATTAATCAAATATTACCTTTTTCAAATGTTTTCCTACATATTTACAATCTTCAAC**

**TTTGTGTAGAGTATAAGTTTCAGCTAATTTCTTATTTTATAAATTTACTTTTAGATATTTAGTCTCTTAA**

**AGGCTGACAATTTTCCCTGAACGAAACTTTCTCGACAAGACGACCACCATTATTAAAAATAAAAACTAGA**

**CTAGAAACACGATTAAATCAATAGGAAAATCTGAGTAGCCTCATTGGCTTGTACTTTTAGCTGTCTATTG**

**CGTACATACTAAGGTGGATTTTAGGCAAGGGAATTACAGGGTATTATAGTCATTCTCGCTCACGTCTATA**

**TATATATTCAAACCCTAGCAAAATCAATGGTACGGGCGTCGGGTCGATGAGTAAAGGAGAAGAACTTTTC**

**ACTGGAGTTGTCCCAATTCTTGTTGAATTAGATGGTGATGTTAATGGGTACAAATTTTCTGTCAGTGGAG**

**AGGGTGAAGGTGATGCAACATACGGAAAACTTACCCTTAAATTTATTTGCACTACTGGAAAACTACCTGT**

**TCCATGGCCAACACTTGTCACTACTTTCTCTTATGGTGTTCAATGCTTTTCAAGATACCCAGATCATATG**

**AAGCGGCACGACTTCTTCAAGAGCGCCATGCCTGAGGGATACGTGCAGGAGAGGACCATCTTCTTCAAGG**

**ACGACGGGAACTACAAGACACGTGCTGAAGTCAAGTTTGAGGGAGACACCCTCGTCAACAGGATCGAGCT**

**TAAGGGAATCGATTTCAAGGAGGACGGAAACATCCTCGGCCACAAGTTGGAATACAACTACAACTCCCAC**

**AACGTATACATCATGGCCGACAAGCAAAAGAACGGCATCAAAGCCAACTTCAAGACCCGCCACAACATCG**

**AAGACGGCGGCGTGCAACTCGCTGATCATTATCAACAAAATACTCCAATTGGCGATGGCCCTGTCCTTTT**

**ACCAGACAACCATTACCTGTCCACACAATCTGCCCTTTCGAAAGATCCCAACGAAAAGAGAGACCACATG**

**GTCCTTCTTGAGTTTGTAACAGCTGCTGGGATTACACATGGCATGGATGAACTATACAAACATGATGAGC**

**TTGATTACGCCGAGGCCTCTTCCCTAATGATATCCCGCGGCCATGCTAGAGTCCGCAAAAATCACCAGTC**

**TCTCTCTACAAATCTATCTCTCTCTATTTTTCTCCAGAATAATGTGTGAGTAGTTCCCAGATAAGGGAAT**

**TAGGGTTCTTATAGGGTTTCGCTCATGTGTTGAGCATATAAGAAACCCTTAGTATGTATTTGTATTTGTA**

**AAATACTTCTATCAATAAAATTTCTAATTCCTAAAACCAAAATCCAGTGACCTGCAGGCATGCGACGTCG**

**>nta-MIR6019, 6020proa-3k::Exon1a (1bp - 12475bp, direct) 12475bp**

**GGGTACCCTCGAATTATCATACATGAGAATTAAGGGAGTCACGTTATGACCCCGCCGATGACGCGGGACA**

**AGCCGTTTTACGTTTGGAACTGACAGAACCGCAACGTTGAAGGAGCCACTGAGCCGCGGGTTTCTGGAGT**

**TTAATGAGCTAAGCACATACGTCAGAAACCATTATTGCGCGTTCAAAAGTCGCCTAAGGTCACTATCAGC**

**TAGCAAATATTTCTTGTCAAAAATGCTCCACTGACGTTCCATAAATTCCCCTCGGTATCCAATTAGAGTC**

**TCATATTCACTCTCAACTCGAATCCCCCCTATGAAAAAGCCTGAACTCACCGCGACGTCTGTCGAGAAGT**

**TTCTGATCGAAAAGTTCGACAGCGTCTCCGACCTGATGCAGCTCTCGGAGGGCGAAGAATCTCGTGCTTT**

**CAGCTTCGATGTAGGAGGGCGTGGATATGTCCTGCGGGTAAATAGCTGCGCCGATGGTTTCTACAAAGAT**

**CGTTATGTTTATCGGCACTTTGCATCGGCCGCGCTCCCGATTCCGGAAGTGCTTGACATTGGGGAGTTCA**

**GCGAGAGCCTGACCTATTGCATCTCCCGCCGTGCACAGGGTGTCACGTTGCAAGACCTGCCTGAAACCGA**

**ACTGCCCGCTGTTCTTCAGCCGGTCGCGGAGGCTATGGATGCTATCGCTGCGGCCGATCTTAGCCAGACG**

**AGCGGGTTCGGCCCATTCGGACCGCAAGGAATCGGTCAATACACTACATGGCGTGATTTCATATGCGCGA**

**TTGCTGATCCCCATGTGTATCACTGGCAAACTGTGATGGACGACACCGTCAGTGCGTCCGTCGCGCAGGC**

**TCTCGATGAGCTGATGCTTTGGGCGAGGACTGCCCCGAAGTCCGGCACTCGTGCACGCGGATTCGGCTCC**

**AACAATGTCCTGACGGACAATGGCCGCATAACAGCGGTCATTGACTGGAGCGAGGCGATGTTCGGGGATT**

**CCCAATACGAGGTCGCCAACATCTTCTTCTGGAGGCCGTGGTTGGCTTGTATGGAGCAGCAGACGCGCTA**

**CTTCGAGCGGAGGCATCCGGAGCTTGCAGGATCGCCAAGGCTCCGGGCGTATATGCTCCGCATTGGTCTT**

**GACCAACTCTATCAGAGCTTGGTTGACGGCAATTTCGATGATGCAGCTTGGGCGCAGGGTCGATGCGACG**

**CAATCGTCCGATCCGGAGCCGGGACTGTCGGGCGTACACAAATCGCCCGCAGAAGCGCGGCCGTCTGGAC**

**CGATGGCTGTGTAGAAGTACTCGCCGATAGTGGAAACCGACGCCCCAGCACTCGTCCGAGGGCAAAGGAA**

**TAGAGTAGATGCCGACCGAACAAGAGCTGATTTCGAGAACGCCTCAGCCAGCAACTCGCGCGAGCCTAGC**

**AAGGCAAATGCGAGAGAACGGCCTTACGCTTGGTGGCACAGTTCTCGTCCACAGTTCGCTAAGCTCGCTC**

**GGCTGGGTCGCGGGAGGGCCGGTCGCAGTGATTCAGGAATTAATTCCCTAGAGTCAAGCAGATCGTTCAA**

**ACATTTGGCAATAAAGTTTCTTAAGATTGAATCCTGTTGCCGGTCTTGCGATGATTATCATATAATTTCT**

**GTTGAATTACGTTAAGCATGTAATAATTAACATGTAATGCATGACGTTATTTATGAGATGGGTTTTTATG**

**ATTAGAGTCCCGCAATTATACATTTAATACGCGATAGAAAACAAAATATAGCGCGCAAACTAGGATAAAT**

**TATCGCGCGCGGTGTCATCTATGTTACTAGATCGACCGGCATGCAAGCTGATAATTCAATTCGGCGTTAA**

**TTCAGTACATTAAAAACGTCCGCAATGTGTTATTAAGTTGTCTAAGCGTCAATTTGTTTACACCACAATA**

**TATCCTGCCACCAGCCAGCCAACAGCTCCCCGACCGGCAGCTCGGCACAAAATCACCACTCGATACAGGC**

**AGCCCATCAGTCCGGGACGGCGTCAGCGGGAGAGCCGTTGTAAGGCGGCAGACTTTGCTCATGTTACCGA**

**TGCTATTCGGAAGAACGGCAACTAAGCTGCCGGGTTTGAAACACGGATGATCTCGCGGAGGGTAGCATGT**

**TGATTGTAACGATGACAGAGCGTTGCTGCCTGTGATCAATTCGGGCACGAACCCAGTGGACATAAGCCTC**

**GTTCGGTTCGTAAGCTGTAATGCAAGTAGCGTAACTGCCGTCACGCAACTGGTCCAGAACCTTGACCGAA**

**CGCAGCGGTGGTAACGGCGCAGTGGCGGTTTTCATGGCTTCTTGTTATGACATGTTTTTTTGGGGTACAG**

**TCTATGCCTCGGGCATCCAAGCAGCAAGCGCGTTACGCCGTGGGTCGATGTTTGATGTTATGGAGCAGCA**

**ACGATGTTACGCAGCAGGGCAGTCGCCCTAAAACAAAGTTAAACATCATGGGGGAAGCGGTGATCGCCGA**

**AGTATCGACTCAACTATCAGAGGTAGTTGGCGTCATCGAGCGCCATCTCGAACCGACGTTGCTGGCCGTA**

**CATTTGTACGGCTCCGCAGTGGATGGCGGCCTGAAGCCACACAGTGATATTGATTTGCTGGTTACGGTGA**

**CCGTAAGGCTTGATGAAACAACGCGGCGAGCTTTGATCAACGACCTTTTGGAAACTTCGGCTTCCCCTGG**

**AGAGAGCGAGATTCTCCGCGCTGTAGAAGTCACCATTGTTGTGCACGACGACATCATTCCGTGGCGTTAT**

**CCAGCTAAGCGCGAACTGCAATTTGGAGAATGGCAGCGCAATGACATTCTTGCAGGTATCTTCGAGCCAG**

**CCACGATCGACATTGATCTGGCTATCTTGCTGACAAAAGCAAGAGAACATAGCGTTGCCTTGGTAGGTCC**

**AGCGGCGGAGGAACTCTTTGATCCGGTTCCTGAACAGGATCTATTTGAGGCGCTAAATGAAACCTTAACG**

**CTATGGAACTCGCCGCCCGACTGGGCTGGCGATGAGCGAAATGTAGTGCTTACGTTGTCCCGCATTTGGT**

**ACAGCGCAGTAACCGGCAAAATCGCGCCGAAGGATGTCGCTGCCGACTGGGCAATGGAGCGCCTGCCGGC**

**CCAGTATCAGCCCGTCATACTTGAAGCTAGACAGGCTTATCTTGGACAAGAAGAAGATCGCTTGGCCTCG**

**CGCGCAGATCAGTTGGAAGAATTTGTCCACTACGTGAAAGGCGAGATCACCAAGGTAGTCGGCAAATAAT**

**GTCTAGCTAGAAATTCGTTCAAGCCGACGCCGCTTCGCCGGCGTTAACTCAAGCGATTAGATGCACTAAG**

**CACATAATTGCTCACAGCCAAACTATCAGGTCAAGTCTGCTTTTATTATTTTTAAGCGTGCATAATAAGC**

**CCTACACAAATTGGGAGATATATCATGCATGACCAAAATCCCTTAACGTGAGTTTTCGTTCCACTGAGCG**

**TCAGACCCCGTAGAAAAGATCAAAGGATCTTCTTGAGATCCTTTTTTTCTGCGCGTAATCTGCTGCTTGC**

**AAACAAAAAAACCACCGCTACCAGCGGTGGTTTGTTTGCCGGATCAAGAGCTACCAACTCTTTTTCCGAA**

**GGTAACTGGCTTCAGCAGAGCGCAGATACCAAATACTGTCCTTCTAGTGTAGCCGTAGTTAGGCCACCAC**

**TTCAAGAACTCTGTAGCACCGCCTACATACCTCGCTCTGCTAATCCTGTTACCAGTGGCTGCTGCCAGTG**

**GCGATAAGTCGTGTCTTACCGGGTTGGACTCAAGACGATAGTTACCGGATAAGGCGCAGCGGTCGGGCTG**

**AACGGGGGGTTCGTGCACACAGCCCAGCTTGGAGCGAACGACCTACACCGAACTGAGATACCTACAGCGT**

**GAGCTATGAGAAAGCGCCACGCTTCCCGAAGGGAGAAAGGCGGACAGGTATCCGGTAAGCGGCAGGGTCG**

**GAACAGGAGAGCGCACGAGGGAGCTTCCAGGGGGAAACGCCTGGTATCTTTATAGTCCTGTCGGGTTTCG**

**CCACCTCTGACTTGAGCGTCGATTTTTGTGATGCTCGTCAGGGGGGCGGAGCCTATGGAAAAACGCCAGC**

**AACGCGGCCTTTTTACGGTTCCTGGCCTTTTGCTGGCCTTTTGCTCACATGTTCTTTCCTGCGTTATCCC**

**CTGATTCTGTGGATAACCGTATTACCGCCTTTGAGTGAGCTGATACCGCTCGCCGCAGCCGAACGACCGA**

**GCGCAGCGAGTCAGTGAGCGAGGAAGCGGAAGAGCGCCTGATGCGGTATTTTCTCCTTACGCATCTGTGC**

**GGTATTTCACACCGCATATGGTGCACTCTCAGTACAATCTGCTCTGATGCCGCATAGTTAAGCCAGTATA**

**CACTCCGCTATCGCTACGTGACTGGGTCATGGCTGCGCCCCGACACCCGCCAACACCCGCTGACGCGCCC**

**TGACGGGCTTGTCTGCTCCCGGCATCCGCTTACAGACAAGCTGTGACCGTCTCCGGGAGCTGCATGTGTC**

**AGAGGTTTTCACCGTCATCACCGAAACGCGCGAGGCAGGGTGCCTTGATGTGGGCGCCGGCGGTCGAGTG**

**GCGACGGCGCGGCTTGTCCGCGCCCTGGTAGATTGCCTGGCCGTAGGCCAGCCATTTTTGAGCGGCCAGC**

**GGCCGCGATAGGCCGACGCGAAGCGGCGGGGCGTAGGGAGCGCAGCGACCGAAGGGTAGGCGCTTTTTGC**

**AGCTCTTCGGCTGTGCGCTGGCCAGACAGTTATGCACAGGCCAGGCGGGTTTTAAGAGTTTTAATAAGTT**

**TTAAAGAGTTTTAGGCGGAAAAATCGCCTTTTTTCTCTTTTATATCAGTCACTTACATGTGTGACCGGTT**

**CCCAATGTACGGCTTTGGGTTCCCAATGTACGGGTTCCGGTTCCCAATGTACGGCTTTGGGTTCCCAATG**

**TACGTGCTATCCACAGGAAAGAGACCTTTTCGACCTTTTTCCCCTGCTAGGGCAATTTGCCCTAGCATCT**

**GCTCCGTACATTAGGAACCGGCGGATGCTTCGCCCTCGATCAGGTTGCGGTAGCGCATGACTAGGATCGG**

**GCCAGCCTGCCCCGCCTCCTCCTTCAAATCGTACTCCGGCAGGTCATTTGACCCGATCAGCTTGCGCACG**

**GTGAAACAGAACTTCTTGAACTCTCCGGCGCTGCCACTGCGTTCGTAGATCGTCTTGAACAACCATCTGG**

**CTTCTGCCTTGCCTGCGGCGCGGCGTGCCAGGCGGTAGAGAAAACGGCCGATGCCGGGATCGATCAAAAA**

**GTAATCGGGGTGAACCGTCAGCACGTCCGGGTTCTTGCCTTCTGTGATCTCGCGGTACATCCAATCAGCT**

**AGCTCGATCTCGATGTACTCCGGCCGCCCGGTTTCGCTCTTTACGATCTTGTAGCGGCTAATCAAGGCTT**

**CACCCTCGGATACCGTCACCAGGCGGCCGTTCTTGGCCTTCTTCGTACGCTGCATGGCAACGTGCGTGGT**

**GTTTAACCGAATGCAGGTTTCTACCAGGTCGTCTTTCTGCTTTCCGCCATCGGCTCGCCGGCAGAACTTG**

**AGTACGTCCGCAACGTGTGGACGGAACACGCGGCCGGGCTTGTCTCCCTTCCCTTCCCGGTATCGGTTCA**

**TGGATTCGGTTAGATGGGAAACCGCCATCAGTACCAGGTCGTAATCCCACACACTGGCCATGCCGGCCGG**

**CCCTGCGGAAACCTCTACGTGCCCGTCTGGAAGCTCGTAGCGGATCACCTCGCCAGCTCGTCGGTCACGC**

**TTCGACAGACGGAAAACGGCCACGTCCATGATGCTGCGACTATCGCGGGTGCCCACGTCATAGAGCATCG**

**GAACGAAAAAATCTGGTTGCTCGTCGCCCTTGGGCGGCTTCCTAATCGACGGCGCACCGGCTGCCGGCGG**

**TTGCCGGGATTCTTTGCGGATTCGATCAGCGGCCGCTTGCCACGATTCACCGGGGCGTGCTTCTGCCTCG**

**ATGCGTTGCCGCTGGGCGGCCTGCGCGGCCTTCAACTTCTCCACCAGGTCATCACCCAGCGCCGCGCCGA**

**TTTGTACCGGGCCGGATGGTTTGCGACCGTCACGCCGATTCCTCGGGCTTGGGGGTTCCAGTGCCATTGC**

**AGGGCCGGCAGACAACCCAGCCGCTTACGCCTGGCCAACCGCCCGTTCCTCCACACATGGGGCATTCCAC**

**GGCGTCGGTGCCTGGTTGTTCTTGATTTTCCATGCCGCCTCCTTTAGCCGCTAAAATTCATCTACTCATT**

**TATTCATTTGCTCATTTACTCTGGTAGCTGCGCGATGTATTCAGATAGCAGCTCGGTAATGGTCTTGCCT**

**TGGCGTACCGCGTACATCTTCAGCTTGGTGTGATCCTCCGCCGGCAACTGAAAGTTGACCCGCTTCATGG**

**CTGGCGTGTCTGCCAGGCTGGCCAACGTTGCAGCCTTGCTGCTGCGTGCGCTCGGACGGCCGGCACTTAG**

**CGTGTTTGTGCTTTTGCTCATTTTCTCTTTACCTCATTAACTCAAATGAGTTTTGATTTAATTTCAGCGG**

**CCAGCGCCTGGACCTCGCGGGCAGCGTCGCCCTCGGGTTCTGATTCAAGAACGGTTGTGCCGGCGGCGGC**

**AGTGCCTGGGTAGCTCACGCGCTGCGTGATACGGGACTCAAGAATGGGCAGCTCGTACCCGGCCAGCGCC**

**TCGGCAACCTCACCGCCGATGCGCGTGCCTTTGATCGCCCGCGACACGACAAAGGCCGCTTGTAGCCTTC**

**CATCCGTGACCTCAATGCGCTGCTTAACCAGCTCCACCAGGTCGGCGGTGGCCCATATGTCGTAAGGGCT**

**TGGCTGCACCGGAATCAGCACGAAGTCGGCTGCCTTGATCGCGGACACAGCCAAGTCCGCCGCCTGGGGC**

**GCTCCGTCGATCACTACGAAGTCGCGCCGGCCGATGGCCTTCACGTCGCGGTCAATCGTCGGGCGGTCGA**

**TGCCGACAACGGTTAGCGGTTGATCTTCCCGCACGGCCGCCCAATCGCGGGCACTGCCCTGGGGATCGGA**

**ATCGACTAACAGAACATCGGCCCCGGCGAGTTGCAGGGCGCGGGCTAGATGGGTTGCGATGGTCGTCTTG**

**CCTGACCCGCCTTTCTGGTTAAGTACAGCGATAACCTTCATGCGTTCCCCTTGCGTATTTGTTTATTTAC**

**TCATCGCATCATATACGCAGCGACCGCATGACGCAAGCTGTTTTACTCAAATACACATCACCTTTTTAGA**

**CGGCGGCGCTCGGTTTCTTCAGCGGCCAAGCTGGCCGGCCAGGCCGCCAGCTTGGCATCAGACAAACCGG**

**CCAGGATTTCATGCAGCCGCACGGTTGAGACGTGCGCGGGCGGCTCGAACACGTACCCGGCCGCGATCAT**

**CTCCGCCTCGATCTCTTCGGTAATGAAAAACGGTTCGTCCTGGCCGTCCTGGTGCGGTTTCATGCTTGTT**

**CCTCTTGGCGTTCATTCTCGGCGGCCGCCAGGGCGTCGGCCTCGGTCAATGCGTCCTCACGGAAGGCACC**

**GCGCCGCCTGGCCTCGGTGGGCGTCACTTCCTCGCTGCGCTCAAGTGCGCGGTACAGGGTCGAGCGATGC**

**ACGCCAAGCAGTGCAGCCGCCTCTTTCACGGTGCGGCCTTCCTGGTCGATCAGCTCGCGGGCGTGCGCGA**

**TCTGTGCCGGGGTGAGGGTAGGGCGGGGGCCAAACTTCACGCCTCGGGCCTTGGCGGCCTCGCGCCCGCT**

**CCGGGTGCGGTCGATGATTAGGGAACGCTCGAACTCGGCAATGCCGGCGAACACGGTCAACACCATGCGG**

**CCGGCCGGCGTGGTGGTGTCGGCCCACGGCTCTGCCAGGCTACGCAGGCCCGCGCCGGCCTCCTGGATGC**

**GCTCGGCAATGTCCAGTAGGTCGCGGGTGCTGCGGGCCAGGCGGTCTAGCCTGGTCACTGTCACAACGTC**

**GCCAGGGCGTAGGTGGTCAAGCATCCTGGCCAGCTCCGGGCGGTCGCGCCTGGTGCCGGTGATCTTCTCG**

**GAAAACAGCTTGGTGCAGCCGGCCGCGTGCAGTTCGGCCCGTTGGTTGGTCAAGTCCTGGTCGTCGGTGC**

**TGACGCGGGCATAGCCCAGCAGGCCAGCGGCGGCGCTCTTGTTCATGGCGTAATGTCTCCGGTTCTAGTC**

**GCAAGTATTCTACTTTATGCGACTAAAACACGCGACAAGAAAACGCCAGGAAAAGGGCAGGGCGGCAGCC**

**TGTCGCGTAACTTAGGACTTGTGCGACATGTCGTTTTCAGAAGACGGCTGCACTGAACGTCAGAAGCCGA**

**CTGCACTATAGCAGCGGAGGGGTTGGATCAAAGTACTTTGATCCCGAGGGGAACCCTGTGGTTGGCATGC**

**ACATACAAATGGACGAACGGATAAACCTTTTCACGCCCTTTTAAATATCCGTTATTCTAATAAACGCTCT**

**TTTCTCTTAGGTTTACCCGCCAATATATCCTGTCAAACACTGATAGTTTAAACTGAAGGCGGGAAACGAC**

**AATCTGATCCAAGCTCAAGCTAAGCTTGAGCTCTGCATGCCTGCAGGTCCCAGGGCGCCCTAACCAACTC**

**TACTTGCTGTTTTCTATGTTATGATAAGAGATACTTCCAATGTTAGATTAGTTATGTTAAAAGATAAGCC**

**ATGATTAAGTTGTTTTGCCTGCGAGTTTGATACTAGTGAGGATTCGTGGAGAATGTTCATGGCATATGTA**

**CCTGAACATGACCCAAAGTAGGAGATAGCCGGGTATTTCCACAGAGAAGTCTAGCACTGGCTTGCCCAAA**

**GCGTTGAACATAGAGAATTAAGGTTGTCTTACATACACACTGGTGGAATTTCATTTTCTTCCGCTTTTTA**

**GAAGGCGTAGCATTGGAACTGCTATGTAGGCTTTTGTTTACCGAGGGTTCGAATCCCTCTCTTTCCGTAC**

**CTTCGCTTAATTCACCAATTTTACTAACAACAAGGGCTCAAATATTGTTTTCCCTTCAGACTGTGTTTGA**

**CTGAACTCCCTACTTTTGCAGTATATGAAACTGAAGCCTTCCTTGAAGGTGTACAAAGGTGCCCTGCCAA**

**AGTGTTTGATGCTGCCTGAGATGCATGTTGCTACTAATCGTGGACCTGCTTCTTCCGAGTCGACTTCTTC**

**TGAATCTGATGCTTGAATATAGTGATTTCGCATGTATGGATGCACACAATTGGGTTTATCAAGAGATACA**

**TCTCTTCTTTTTGTGATCTATTTAGTTTTGGAGCATCTCTGATGCATGTTGACAAGTGGAGGGTGGCTTT**

**TGGAATTTTGAAGTTCAGAGAAGAGAAATAAGTTAGTCTGCTGCTGTCCTACTGGCTCAAAAGTAAGCTA**

**AGGTATCAAAGGTAGCGTTTTATAAGTAGAGTTTAGGAAAATGTTAGTCTGCAGTGGATAATGCTGGGCA**

**AATTGCTTCTTTTTGGAAATTTTTTTGTGCTTTTCCTTTCCAGTATCAATTGAAAAGGAAAGAGAAGGTT**

**TTACGTTGTTTTGGTCAATTTTACGGGGAAGAAGGAAAATTGCAAAGTTCATTATAGAATTTTCTTTTCA**

**GTCGTAATCGCAAAAGCTCGTGTTGATATAATCCTATCTATATTTTTGTCTTTAACTGTTTGGCTAAGGT**

**TCTTTATGATATTAATATTTTCAATTGCAATCGGCTCACTTCTTAGAGCAGTTTGATGGATGTAGTAGGT**

**GGCAGTGACGGGTAGGGAGGATTACAGTGTGCATAGTTTCTGTTGAAGTGCTTTGACTGGTGGCTCTGTT**

**GTTGGAGCATGTATTTTCCTTCAGTGATTGATCGGCTTTCTTAGAGTTGGAAGTAATGTAAATTGCCTTT**

**GACTGGTTTTCTTAAGCAGGTAATGAGGGGTTAATATTGCTTAATGGTGTTGAATCTTTTGAAAGAAAAC**

**TACACTATCCAGGTTCTGTTTAAGTATTAAAATTGCAATAAAAAACAGTAGGTAAATGGATTCAAGTTGC**

**ATTTTAACCTTTTAAGTGCCTGCCTCTATTTTTTTTTCCCATTCAAAATTGCTCTCGTCTCAATGTCCTA**

**AGACATTTTGAAAGAATTGAGAATGTCTTTCTGGACAGACGATTGACACCTCAATGGAGTAAAGATATTA**

**GGGGTATGGAACTTTAATTCTAGTCCTTCTGCTTTGTTCTTAGGCAGACCCCCCTGTAGGCCTATCGACC**

**AATGACTCGTGATCCCAACTTGCGTAATGGAAAAATTGGAGTAATAGAACAATTGATTTCGTTGTGGTGG**

**TAAAAGATATCACGTATCTTGATTGTGTACAAAGGCAACTTGGTCTCTGGCTATAAAGTTTCCAGTATTT**

**TGCACCGCTTGTGAATTATAAGACGATCTTATACCAGAAAATGAAACATTGGACATAACTTTGGAACCCC**

**CCCCCCACTGGCTGCAAGGAAATAACGGATTGGATTTTCTAACTCTCTTTAAAGAGACTGGTTTCGAATT**

**CACTTGCTTGCAAGCTTAGGGTTAGCAGGTTGTTGACTGGGAGAGCTGGTAAAAGAATAAAATCTTCAAG**

**AGATCATGGTTAACAAGCCAGCTGAGGAATAACAATCTTCCTCAGCAATACCAAAAATCCATCCAAGGAA**

**TGAGCTTCCCCAAAGTTAATTCGCGTGGACTACAAAGAAAAGATAGAGTCTCACCCGGTAACATCAGCTG**

**AGTCAATGGCAGAAAGTCTTGAAAAGAACCTTTTATTCGATGCTTCTGATTCAAGCTTATTCCTTTCCGC**

**CTTTTGAATAAATCTAGAACCTCAATTAGCAGATTCATTAGAAACCTTTCGACCAACATAATCTTTGAAT**

**TTTCCGCATATATAACCAGCAGAGCTCTCGTATGAGCAAAGTAACGCCTTCGTCCTTTTTCTCCATGTAT**

**GCTTAGGAATCACACCAATCTATCCCGAATTTGGTGGTTAAACTCTACTGGTATGAGAAGGGTCCTCCTC**

**TCCCTTGTCAAATAAAAATGACACACTATGTGGGCGGATGGAGAATAACTTTAAGGTCTACATTAAAGAA**

**AAAAAAAGAGCAAAAAGGATAAGATTTAAGTGATTTTTGGTAGTTATTTTCAATTACAATAAAGTCAAAT**

**CGTCGTACAACTGTTGTTAGTCATAAAGAAATTAATCAAATATTACCTTTTTCAAATGTTTTCCTACATA**

**TTTACAATCTTCAACTTTGTGTAGAGTATAAGTTTCAGCTAATTTCTTATTTTATAAATTTACTTTTAGA**

**TATTTAGTCTCTTAAAGGCTGACAATTTTCCCTGAACGAAACTTTCTCGACAAGACGACCACCATTATTA**

**AAAATAAAAACTAGACTAGAAACACGATTAAATCAATAGGAAAATCTGAGTAGCCTCATTGGCTTGTACT**

**TTTAGCTGTCTATTGCGTACATACTAAGGTGGATTTTAGGCAAGGGAATTACAGGGTATTATAGTCATTC**

**TCGCTCACGTCTATATATATATTCAAACCCTAGCAAAATCAATGGTACGGGCGTCGGGTCGATGAGTAAA**

**GGAGAAGAACTTTTCACTGGAGTTGTCCCAATTCTTGTTGAATTAGATGGTGATGTTAATGGGTACAAAT**

**TTTCTGTCAGTGGAGAGGGTGAAGGTGATGCAACATACGGAAAACTTACCCTTAAATTTATTTGCACTAC**

**TGGAAAACTACCTGTTCCATGGCCAACACTTGTCACTACTTTCTCTTATGGTGTTCAATGCTTTTCAAGA**

**TACCCAGATCATATGAAGCGGCACGACTTCTTCAAGAGCGCCATGCCTGAGGGATACGTGCAGGAGAGGA**

**CCATCTTCTTCAAGGACGACGGGAACTACAAGACACGTGCTGAAGTCAAGTTTGAGGGAGACACCCTCGT**

**CAACAGGATCGAGCTTAAGGGAATCGATTTCAAGGAGGACGGAAACATCCTCGGCCACAAGTTGGAATAC**

**AACTACAACTCCCACAACGTATACATCATGGCCGACAAGCAAAAGAACGGCATCAAAGCCAACTTCAAGA**

**CCCGCCACAACATCGAAGACGGCGGCGTGCAACTCGCTGATCATTATCAACAAAATACTCCAATTGGCGA**

**TGGCCCTGTCCTTTTACCAGACAACCATTACCTGTCCACACAATCTGCCCTTTCGAAAGATCCCAACGAA**

**AAGAGAGACCACATGGTCCTTCTTGAGTTTGTAACAGCTGCTGGGATTACACATGGCATGGATGAACTAT**

**ACAAACATGATGAGCTTGATTACGCCGAGGCCTCTTCCCTAATGATATCCCGCGGCCATGCTAGAGTCCG**

**CAAAAATCACCAGTCTCTCTCTACAAATCTATCTCTCTCTATTTTTCTCCAGAATAATGTGTGAGTAGTT**

**CCCAGATAAGGGAATTAGGGTTCTTATAGGGTTTCGCTCATGTGTTGAGCATATAAGAAACCCTTAGTAT**

**GTATTTGTATTTGTAAAATACTTCTATCAATAAAATTTCTAATTCCTAAAACCAAAATCCAGTGACCTGC**

**AGGCATGCGACGTCG**

**>nta-MIR6019, 6020proa-5k::Exon1a (direct) 14546bp**

**GGGTACCCTCGAATTATCATACATGAGAATTAAGGGAGTCACGTTATGACCCCGCCGATGACGCGGGACA**

**AGCCGTTTTACGTTTGGAACTGACAGAACCGCAACGTTGAAGGAGCCACTGAGCCGCGGGTTTCTGGAGT**

**TTAATGAGCTAAGCACATACGTCAGAAACCATTATTGCGCGTTCAAAAGTCGCCTAAGGTCACTATCAGC**

**TAGCAAATATTTCTTGTCAAAAATGCTCCACTGACGTTCCATAAATTCCCCTCGGTATCCAATTAGAGTC**

**TCATATTCACTCTCAACTCGAATCCCCCCTATGAAAAAGCCTGAACTCACCGCGACGTCTGTCGAGAAGT**

**TTCTGATCGAAAAGTTCGACAGCGTCTCCGACCTGATGCAGCTCTCGGAGGGCGAAGAATCTCGTGCTTT**

**CAGCTTCGATGTAGGAGGGCGTGGATATGTCCTGCGGGTAAATAGCTGCGCCGATGGTTTCTACAAAGAT**

**CGTTATGTTTATCGGCACTTTGCATCGGCCGCGCTCCCGATTCCGGAAGTGCTTGACATTGGGGAGTTCA**

**GCGAGAGCCTGACCTATTGCATCTCCCGCCGTGCACAGGGTGTCACGTTGCAAGACCTGCCTGAAACCGA**

**ACTGCCCGCTGTTCTTCAGCCGGTCGCGGAGGCTATGGATGCTATCGCTGCGGCCGATCTTAGCCAGACG**

**AGCGGGTTCGGCCCATTCGGACCGCAAGGAATCGGTCAATACACTACATGGCGTGATTTCATATGCGCGA**

**TTGCTGATCCCCATGTGTATCACTGGCAAACTGTGATGGACGACACCGTCAGTGCGTCCGTCGCGCAGGC**

**TCTCGATGAGCTGATGCTTTGGGCGAGGACTGCCCCGAAGTCCGGCACTCGTGCACGCGGATTCGGCTCC**

**AACAATGTCCTGACGGACAATGGCCGCATAACAGCGGTCATTGACTGGAGCGAGGCGATGTTCGGGGATT**

**CCCAATACGAGGTCGCCAACATCTTCTTCTGGAGGCCGTGGTTGGCTTGTATGGAGCAGCAGACGCGCTA**

**CTTCGAGCGGAGGCATCCGGAGCTTGCAGGATCGCCAAGGCTCCGGGCGTATATGCTCCGCATTGGTCTT**

**GACCAACTCTATCAGAGCTTGGTTGACGGCAATTTCGATGATGCAGCTTGGGCGCAGGGTCGATGCGACG**

**CAATCGTCCGATCCGGAGCCGGGACTGTCGGGCGTACACAAATCGCCCGCAGAAGCGCGGCCGTCTGGAC**

**CGATGGCTGTGTAGAAGTACTCGCCGATAGTGGAAACCGACGCCCCAGCACTCGTCCGAGGGCAAAGGAA**

**TAGAGTAGATGCCGACCGAACAAGAGCTGATTTCGAGAACGCCTCAGCCAGCAACTCGCGCGAGCCTAGC**

**AAGGCAAATGCGAGAGAACGGCCTTACGCTTGGTGGCACAGTTCTCGTCCACAGTTCGCTAAGCTCGCTC**

**GGCTGGGTCGCGGGAGGGCCGGTCGCAGTGATTCAGGAATTAATTCCCTAGAGTCAAGCAGATCGTTCAA**

**ACATTTGGCAATAAAGTTTCTTAAGATTGAATCCTGTTGCCGGTCTTGCGATGATTATCATATAATTTCT**

**GTTGAATTACGTTAAGCATGTAATAATTAACATGTAATGCATGACGTTATTTATGAGATGGGTTTTTATG**

**ATTAGAGTCCCGCAATTATACATTTAATACGCGATAGAAAACAAAATATAGCGCGCAAACTAGGATAAAT**

**TATCGCGCGCGGTGTCATCTATGTTACTAGATCGACCGGCATGCAAGCTGATAATTCAATTCGGCGTTAA**

**TTCAGTACATTAAAAACGTCCGCAATGTGTTATTAAGTTGTCTAAGCGTCAATTTGTTTACACCACAATA**

**TATCCTGCCACCAGCCAGCCAACAGCTCCCCGACCGGCAGCTCGGCACAAAATCACCACTCGATACAGGC**

**AGCCCATCAGTCCGGGACGGCGTCAGCGGGAGAGCCGTTGTAAGGCGGCAGACTTTGCTCATGTTACCGA**

**TGCTATTCGGAAGAACGGCAACTAAGCTGCCGGGTTTGAAACACGGATGATCTCGCGGAGGGTAGCATGT**

**TGATTGTAACGATGACAGAGCGTTGCTGCCTGTGATCAATTCGGGCACGAACCCAGTGGACATAAGCCTC**

**GTTCGGTTCGTAAGCTGTAATGCAAGTAGCGTAACTGCCGTCACGCAACTGGTCCAGAACCTTGACCGAA**

**CGCAGCGGTGGTAACGGCGCAGTGGCGGTTTTCATGGCTTCTTGTTATGACATGTTTTTTTGGGGTACAG**

**TCTATGCCTCGGGCATCCAAGCAGCAAGCGCGTTACGCCGTGGGTCGATGTTTGATGTTATGGAGCAGCA**

**ACGATGTTACGCAGCAGGGCAGTCGCCCTAAAACAAAGTTAAACATCATGGGGGAAGCGGTGATCGCCGA**

**AGTATCGACTCAACTATCAGAGGTAGTTGGCGTCATCGAGCGCCATCTCGAACCGACGTTGCTGGCCGTA**

**CATTTGTACGGCTCCGCAGTGGATGGCGGCCTGAAGCCACACAGTGATATTGATTTGCTGGTTACGGTGA**

**CCGTAAGGCTTGATGAAACAACGCGGCGAGCTTTGATCAACGACCTTTTGGAAACTTCGGCTTCCCCTGG**

**AGAGAGCGAGATTCTCCGCGCTGTAGAAGTCACCATTGTTGTGCACGACGACATCATTCCGTGGCGTTAT**

**CCAGCTAAGCGCGAACTGCAATTTGGAGAATGGCAGCGCAATGACATTCTTGCAGGTATCTTCGAGCCAG**

**CCACGATCGACATTGATCTGGCTATCTTGCTGACAAAAGCAAGAGAACATAGCGTTGCCTTGGTAGGTCC**

**AGCGGCGGAGGAACTCTTTGATCCGGTTCCTGAACAGGATCTATTTGAGGCGCTAAATGAAACCTTAACG**

**CTATGGAACTCGCCGCCCGACTGGGCTGGCGATGAGCGAAATGTAGTGCTTACGTTGTCCCGCATTTGGT**

**ACAGCGCAGTAACCGGCAAAATCGCGCCGAAGGATGTCGCTGCCGACTGGGCAATGGAGCGCCTGCCGGC**

**CCAGTATCAGCCCGTCATACTTGAAGCTAGACAGGCTTATCTTGGACAAGAAGAAGATCGCTTGGCCTCG**

**CGCGCAGATCAGTTGGAAGAATTTGTCCACTACGTGAAAGGCGAGATCACCAAGGTAGTCGGCAAATAAT**

**GTCTAGCTAGAAATTCGTTCAAGCCGACGCCGCTTCGCCGGCGTTAACTCAAGCGATTAGATGCACTAAG**

**CACATAATTGCTCACAGCCAAACTATCAGGTCAAGTCTGCTTTTATTATTTTTAAGCGTGCATAATAAGC**

**CCTACACAAATTGGGAGATATATCATGCATGACCAAAATCCCTTAACGTGAGTTTTCGTTCCACTGAGCG**

**TCAGACCCCGTAGAAAAGATCAAAGGATCTTCTTGAGATCCTTTTTTTCTGCGCGTAATCTGCTGCTTGC**

**AAACAAAAAAACCACCGCTACCAGCGGTGGTTTGTTTGCCGGATCAAGAGCTACCAACTCTTTTTCCGAA**

**GGTAACTGGCTTCAGCAGAGCGCAGATACCAAATACTGTCCTTCTAGTGTAGCCGTAGTTAGGCCACCAC**

**TTCAAGAACTCTGTAGCACCGCCTACATACCTCGCTCTGCTAATCCTGTTACCAGTGGCTGCTGCCAGTG**

**GCGATAAGTCGTGTCTTACCGGGTTGGACTCAAGACGATAGTTACCGGATAAGGCGCAGCGGTCGGGCTG**

**AACGGGGGGTTCGTGCACACAGCCCAGCTTGGAGCGAACGACCTACACCGAACTGAGATACCTACAGCGT**

**GAGCTATGAGAAAGCGCCACGCTTCCCGAAGGGAGAAAGGCGGACAGGTATCCGGTAAGCGGCAGGGTCG**

**GAACAGGAGAGCGCACGAGGGAGCTTCCAGGGGGAAACGCCTGGTATCTTTATAGTCCTGTCGGGTTTCG**

**CCACCTCTGACTTGAGCGTCGATTTTTGTGATGCTCGTCAGGGGGGCGGAGCCTATGGAAAAACGCCAGC**

**AACGCGGCCTTTTTACGGTTCCTGGCCTTTTGCTGGCCTTTTGCTCACATGTTCTTTCCTGCGTTATCCC**

**CTGATTCTGTGGATAACCGTATTACCGCCTTTGAGTGAGCTGATACCGCTCGCCGCAGCCGAACGACCGA**

**GCGCAGCGAGTCAGTGAGCGAGGAAGCGGAAGAGCGCCTGATGCGGTATTTTCTCCTTACGCATCTGTGC**

**GGTATTTCACACCGCATATGGTGCACTCTCAGTACAATCTGCTCTGATGCCGCATAGTTAAGCCAGTATA**

**CACTCCGCTATCGCTACGTGACTGGGTCATGGCTGCGCCCCGACACCCGCCAACACCCGCTGACGCGCCC**

**TGACGGGCTTGTCTGCTCCCGGCATCCGCTTACAGACAAGCTGTGACCGTCTCCGGGAGCTGCATGTGTC**

**AGAGGTTTTCACCGTCATCACCGAAACGCGCGAGGCAGGGTGCCTTGATGTGGGCGCCGGCGGTCGAGTG**

**GCGACGGCGCGGCTTGTCCGCGCCCTGGTAGATTGCCTGGCCGTAGGCCAGCCATTTTTGAGCGGCCAGC**

**GGCCGCGATAGGCCGACGCGAAGCGGCGGGGCGTAGGGAGCGCAGCGACCGAAGGGTAGGCGCTTTTTGC**

**AGCTCTTCGGCTGTGCGCTGGCCAGACAGTTATGCACAGGCCAGGCGGGTTTTAAGAGTTTTAATAAGTT**

**TTAAAGAGTTTTAGGCGGAAAAATCGCCTTTTTTCTCTTTTATATCAGTCACTTACATGTGTGACCGGTT**

**CCCAATGTACGGCTTTGGGTTCCCAATGTACGGGTTCCGGTTCCCAATGTACGGCTTTGGGTTCCCAATG**

**TACGTGCTATCCACAGGAAAGAGACCTTTTCGACCTTTTTCCCCTGCTAGGGCAATTTGCCCTAGCATCT**

**GCTCCGTACATTAGGAACCGGCGGATGCTTCGCCCTCGATCAGGTTGCGGTAGCGCATGACTAGGATCGG**

**GCCAGCCTGCCCCGCCTCCTCCTTCAAATCGTACTCCGGCAGGTCATTTGACCCGATCAGCTTGCGCACG**

**GTGAAACAGAACTTCTTGAACTCTCCGGCGCTGCCACTGCGTTCGTAGATCGTCTTGAACAACCATCTGG**

**CTTCTGCCTTGCCTGCGGCGCGGCGTGCCAGGCGGTAGAGAAAACGGCCGATGCCGGGATCGATCAAAAA**

**GTAATCGGGGTGAACCGTCAGCACGTCCGGGTTCTTGCCTTCTGTGATCTCGCGGTACATCCAATCAGCT**

**AGCTCGATCTCGATGTACTCCGGCCGCCCGGTTTCGCTCTTTACGATCTTGTAGCGGCTAATCAAGGCTT**

**CACCCTCGGATACCGTCACCAGGCGGCCGTTCTTGGCCTTCTTCGTACGCTGCATGGCAACGTGCGTGGT**

**GTTTAACCGAATGCAGGTTTCTACCAGGTCGTCTTTCTGCTTTCCGCCATCGGCTCGCCGGCAGAACTTG**

**AGTACGTCCGCAACGTGTGGACGGAACACGCGGCCGGGCTTGTCTCCCTTCCCTTCCCGGTATCGGTTCA**

**TGGATTCGGTTAGATGGGAAACCGCCATCAGTACCAGGTCGTAATCCCACACACTGGCCATGCCGGCCGG**

**CCCTGCGGAAACCTCTACGTGCCCGTCTGGAAGCTCGTAGCGGATCACCTCGCCAGCTCGTCGGTCACGC**

**TTCGACAGACGGAAAACGGCCACGTCCATGATGCTGCGACTATCGCGGGTGCCCACGTCATAGAGCATCG**

**GAACGAAAAAATCTGGTTGCTCGTCGCCCTTGGGCGGCTTCCTAATCGACGGCGCACCGGCTGCCGGCGG**

**TTGCCGGGATTCTTTGCGGATTCGATCAGCGGCCGCTTGCCACGATTCACCGGGGCGTGCTTCTGCCTCG**

**ATGCGTTGCCGCTGGGCGGCCTGCGCGGCCTTCAACTTCTCCACCAGGTCATCACCCAGCGCCGCGCCGA**

**TTTGTACCGGGCCGGATGGTTTGCGACCGTCACGCCGATTCCTCGGGCTTGGGGGTTCCAGTGCCATTGC**

**AGGGCCGGCAGACAACCCAGCCGCTTACGCCTGGCCAACCGCCCGTTCCTCCACACATGGGGCATTCCAC**

**GGCGTCGGTGCCTGGTTGTTCTTGATTTTCCATGCCGCCTCCTTTAGCCGCTAAAATTCATCTACTCATT**

**TATTCATTTGCTCATTTACTCTGGTAGCTGCGCGATGTATTCAGATAGCAGCTCGGTAATGGTCTTGCCT**

**TGGCGTACCGCGTACATCTTCAGCTTGGTGTGATCCTCCGCCGGCAACTGAAAGTTGACCCGCTTCATGG**

**CTGGCGTGTCTGCCAGGCTGGCCAACGTTGCAGCCTTGCTGCTGCGTGCGCTCGGACGGCCGGCACTTAG**

**CGTGTTTGTGCTTTTGCTCATTTTCTCTTTACCTCATTAACTCAAATGAGTTTTGATTTAATTTCAGCGG**

**CCAGCGCCTGGACCTCGCGGGCAGCGTCGCCCTCGGGTTCTGATTCAAGAACGGTTGTGCCGGCGGCGGC**

**AGTGCCTGGGTAGCTCACGCGCTGCGTGATACGGGACTCAAGAATGGGCAGCTCGTACCCGGCCAGCGCC**

**TCGGCAACCTCACCGCCGATGCGCGTGCCTTTGATCGCCCGCGACACGACAAAGGCCGCTTGTAGCCTTC**

**CATCCGTGACCTCAATGCGCTGCTTAACCAGCTCCACCAGGTCGGCGGTGGCCCATATGTCGTAAGGGCT**

**TGGCTGCACCGGAATCAGCACGAAGTCGGCTGCCTTGATCGCGGACACAGCCAAGTCCGCCGCCTGGGGC**

**GCTCCGTCGATCACTACGAAGTCGCGCCGGCCGATGGCCTTCACGTCGCGGTCAATCGTCGGGCGGTCGA**

**TGCCGACAACGGTTAGCGGTTGATCTTCCCGCACGGCCGCCCAATCGCGGGCACTGCCCTGGGGATCGGA**

**ATCGACTAACAGAACATCGGCCCCGGCGAGTTGCAGGGCGCGGGCTAGATGGGTTGCGATGGTCGTCTTG**

**CCTGACCCGCCTTTCTGGTTAAGTACAGCGATAACCTTCATGCGTTCCCCTTGCGTATTTGTTTATTTAC**

**TCATCGCATCATATACGCAGCGACCGCATGACGCAAGCTGTTTTACTCAAATACACATCACCTTTTTAGA**

**CGGCGGCGCTCGGTTTCTTCAGCGGCCAAGCTGGCCGGCCAGGCCGCCAGCTTGGCATCAGACAAACCGG**

**CCAGGATTTCATGCAGCCGCACGGTTGAGACGTGCGCGGGCGGCTCGAACACGTACCCGGCCGCGATCAT**

**CTCCGCCTCGATCTCTTCGGTAATGAAAAACGGTTCGTCCTGGCCGTCCTGGTGCGGTTTCATGCTTGTT**

**CCTCTTGGCGTTCATTCTCGGCGGCCGCCAGGGCGTCGGCCTCGGTCAATGCGTCCTCACGGAAGGCACC**

**GCGCCGCCTGGCCTCGGTGGGCGTCACTTCCTCGCTGCGCTCAAGTGCGCGGTACAGGGTCGAGCGATGC**

**ACGCCAAGCAGTGCAGCCGCCTCTTTCACGGTGCGGCCTTCCTGGTCGATCAGCTCGCGGGCGTGCGCGA**

**TCTGTGCCGGGGTGAGGGTAGGGCGGGGGCCAAACTTCACGCCTCGGGCCTTGGCGGCCTCGCGCCCGCT**

**CCGGGTGCGGTCGATGATTAGGGAACGCTCGAACTCGGCAATGCCGGCGAACACGGTCAACACCATGCGG**

**CCGGCCGGCGTGGTGGTGTCGGCCCACGGCTCTGCCAGGCTACGCAGGCCCGCGCCGGCCTCCTGGATGC**

**GCTCGGCAATGTCCAGTAGGTCGCGGGTGCTGCGGGCCAGGCGGTCTAGCCTGGTCACTGTCACAACGTC**

**GCCAGGGCGTAGGTGGTCAAGCATCCTGGCCAGCTCCGGGCGGTCGCGCCTGGTGCCGGTGATCTTCTCG**

**GAAAACAGCTTGGTGCAGCCGGCCGCGTGCAGTTCGGCCCGTTGGTTGGTCAAGTCCTGGTCGTCGGTGC**

**TGACGCGGGCATAGCCCAGCAGGCCAGCGGCGGCGCTCTTGTTCATGGCGTAATGTCTCCGGTTCTAGTC**

**GCAAGTATTCTACTTTATGCGACTAAAACACGCGACAAGAAAACGCCAGGAAAAGGGCAGGGCGGCAGCC**

**TGTCGCGTAACTTAGGACTTGTGCGACATGTCGTTTTCAGAAGACGGCTGCACTGAACGTCAGAAGCCGA**

**CTGCACTATAGCAGCGGAGGGGTTGGATCAAAGTACTTTGATCCCGAGGGGAACCCTGTGGTTGGCATGC**

**ACATACAAATGGACGAACGGATAAACCTTTTCACGCCCTTTTAAATATCCGTTATTCTAATAAACGCTCT**

**TTTCTCTTAGGTTTACCCGCCAATATATCCTGTCAAACACTGATAGTTTAAACTGAAGGCGGGAAACGAC**

**AATCTGATCCAAGCTCAAGCTAAGCTTGAGCTCTGCATGCCTGCAGGTCCCAGGGCGCCCTTGATTAACC**

**GGGGAATGCTGCTAATTCATATTGGGGACAACCAATGTTTTATAGTAAATGGTCATGAATTATGTTTAAT**

**TAAACTTGAATTTGCGCGTAAATTATGGCACTTGAACTTTTATTGTTCCCTATGTAATGTCATAACCTTG**

**TGGTTGATCACATTCATTGGTCAGGTGAATTATTTAGTCTCTTTAATAATTCTTTGGCACTTACAAAAGA**

**TAGTAGTACTAGAATTCAAATGGTTACTTGATTATGTTTGACCTGTTCATGTGACATTATCCTGTCCCAA**

**CCTCTTGCTCCCACCCTCTCAGGGAGTGTATAACCATGAATATGTAAGACTTGTTTAGAGGAGCTTGTGA**

**CTTGTGACGAGTGTGTGCTTTTGTTGGGAGCTAGTGTGCTTTCCTAGTTCTATCTCACTGGAAAAGCAAT**

**ATTAGAGAACAAACATAAAGGAATCACATCTTCAGCTAATTGCTGTTACATATATTGTGCCTTCTCTCCT**

**CTAAAGCTTAATGCACCAAGGGGTCGTTTGGTTAGAAGACAAGTTATGCATGGATTAACAATGCATGTAT**

**TAGTATTTTCAAGGAAAAGACAAATTAAATCTAAAGAATGAGGAGCTTTTTGTCAAATTTTGTTCAATCC**

**ATGTATTACTTTACCTGTATTCTTATTCTATATTCTACCCTGCATAAAATAATAAGTAGATTCCAGCATA**

**ACTTATGCGAGTATTATTTCATGTGGAAAACAAAAGAAGCAACTAAAAAACGTATATAATTGTTTGGAGT**

**TTAATGCAGTGATGATGTTTCTCTGTGCGCCTCTCTTGGCCAATGGTTCGCATTTCCTGCCGTATGTATG**

**TTTTTTGTCGTTGTAACGTACCATGATGGTTCCTTCTCTGGCATAATCATTCATCTGTACTTGGGTCCTA**

**GCGGTTCTGTAGCACTTCTTGTTACTGTGAAGGGTATTATTTCTGATGCACTGTTTACCGCTTTTTCCAC**

**AAATTGCTTGTTCACTGGCACTCAAGGAACCAAATTGGTAGCTACATTTTAGCTTTTTCTTTCTTAGAGA**

**GAGAGAGAGAGAGAGTATTGTAGCTTTTATGCATCTTTATGTAGAAAAGACAGCAGAATGTCTACTTGAA**

**AGGAAAAGGGTTGGAATCCATCTTGCTTATGAATATTGTTGAAGAATCTGTATATTTTCCATTTTAGTTG**

**TATCTTCCTCTATTTGGATATAGAATTTATGTTCAAAGGTTTGTGCTTGATGGATAGTTGATATCCATGT**

**TAATGGGAAGTAATTGTTTCCAGGTTTCCAGTTCTGCTGATAAAGCTAAGCAACCAAAAGCCACAGCTAG**

**GGCAAATGAGATGGTTGGATTAGCTAAGAGTATGCAACAACCTTTTTCCACTCAACTTGCAAATCAGCCC**

**CGTAGTGGACAAGTCAGATCGGAAGCTCCAGCTACCTCTCATGGCAAAACTCTTCTGGTTCTGAAGCCAG**

**GACGGGAAAACGGTGTCACCTCTTTATCAAAAGAAGCACTGAGTCCAGCTAACAACACTGGCGGTAGATT**

**AGCTAATTGCTCTCCTGCAGTGGCTCCTTCAGCTCCTGCTTTGACCAGTCCAACTTCGAGGGTGACTTCC**

**CTTGAATCTAAAGCTGCTACATTGTCGCTGAAAGCACGATCTACTGCAGAGAAGAGATCTTCTCTGTCTC**

**AAGCCCAAAGCAGGAGTGATTTTTTCAATCTAATGAGGAAGAAGACTTCATTAAATTCTTCTACTGTCCT**

**TCCAGACTCTGGTATGGCTTCATCTGACAGTAGTGAGCAATCTGGTATTAAAACTAAAGATGAGGATAGT**

**GCCTCATTGAGTCCTTGTGTTTCTGAAAATGGAAGTGAGAGAACCAGCAATGGAGATGCTCATGAATCTC**

**AAAACGATGTTCAAAGACATAATGGTATCGAGGAGAACAACTTGTCCATAAATGGAGCGCTATATCCAGA**

**TGAGGAAGAGGCTGCTTTCCTACGTTCTCTTGGTTGGGATGAAAATGCTGGGGAAGACGAAGGCCTGACA**

**GAGGAAGAAATTAATGCATTTTATCAGGAGGTAACCAACTCTACTTGCTGTTTTCTATGTTATGATAAGA**

**GATACTTCCAATGTTAGATTAGTTATGTTAAAAGATAAGCCATGATTAAGTTGTTTTGCCTGCGAGTTTG**

**ATACTAGTGAGGATTCGTGGAGAATGTTCATGGCATATGTACCTGAACATGACCCAAAGTAGGAGATAGC**

**CGGGTATTTCCACAGAGAAGTCTAGCACTGGCTTGCCCAAAGCGTTGAACATAGAGAATTAAGGTTGTCT**

**TACATACACACTGGTGGAATTTCATTTTCTTCCGCTTTTTAGAAGGCGTAGCATTGGAACTGCTATGTAG**

**GCTTTTGTTTACCGAGGGTTCGAATCCCTCTCTTTCCGTACCTTCGCTTAATTCACCAATTTTACTAACA**

**ACAAGGGCTCAAATATTGTTTTCCCTTCAGACTGTGTTTGACTGAACTCCCTACTTTTGCAGTATATGAA**

**ACTGAAGCCTTCCTTGAAGGTGTACAAAGGTGCCCTGCCAAAGTGTTTGATGCTGCCTGAGATGCATGTT**

**GCTACTAATCGTGGACCTGCTTCTTCCGAGTCGACTTCTTCTGAATCTGATGCTTGAATATAGTGATTTC**

**GCATGTATGGATGCACACAATTGGGTTTATCAAGAGATACATCTCTTCTTTTTGTGATCTATTTAGTTTT**

**GGAGCATCTCTGATGCATGTTGACAAGTGGAGGGTGGCTTTTGGAATTTTGAAGTTCAGAGAAGAGAAAT**

**AAGTTAGTCTGCTGCTGTCCTACTGGCTCAAAAGTAAGCTAAGGTATCAAAGGTAGCGTTTTATAAGTAG**

**AGTTTAGGAAAATGTTAGTCTGCAGTGGATAATGCTGGGCAAATTGCTTCTTTTTGGAAATTTTTTTGTG**

**CTTTTCCTTTCCAGTATCAATTGAAAAGGAAAGAGAAGGTTTTACGTTGTTTTGGTCAATTTTACGGGGA**

**AGAAGGAAAATTGCAAAGTTCATTATAGAATTTTCTTTTCAGTCGTAATCGCAAAAGCTCGTGTTGATAT**

**AATCCTATCTATATTTTTGTCTTTAACTGTTTGGCTAAGGTTCTTTATGATATTAATATTTTCAATTGCA**

**ATCGGCTCACTTCTTAGAGCAGTTTGATGGATGTAGTAGGTGGCAGTGACGGGTAGGGAGGATTACAGTG**

**TGCATAGTTTCTGTTGAAGTGCTTTGACTGGTGGCTCTGTTGTTGGAGCATGTATTTTCCTTCAGTGATT**

**GATCGGCTTTCTTAGAGTTGGAAGTAATGTAAATTGCCTTTGACTGGTTTTCTTAAGCAGGTAATGAGGG**

**GTTAATATTGCTTAATGGTGTTGAATCTTTTGAAAGAAAACTACACTATCCAGGTTCTGTTTAAGTATTA**

**AAATTGCAATAAAAAACAGTAGGTAAATGGATTCAAGTTGCATTTTAACCTTTTAAGTGCCTGCCTCTAT**

**TTTTTTTTCCCATTCAAAATTGCTCTCGTCTCAATGTCCTAAGACATTTTGAAAGAATTGAGAATGTCTT**

**TCTGGACAGACGATTGACACCTCAATGGAGTAAAGATATTAGGGGTATGGAACTTTAATTCTAGTCCTTC**

**TGCTTTGTTCTTAGGCAGACCCCCCTGTAGGCCTATCGACCAATGACTCGTGATCCCAACTTGCGTAATG**

**GAAAAATTGGAGTAATAGAACAATTGATTTCGTTGTGGTGGTAAAAGATATCACGTATCTTGATTGTGTA**

**CAAAGGCAACTTGGTCTCTGGCTATAAAGTTTCCAGTATTTTGCACCGCTTGTGAATTATAAGACGATCT**

**TATACCAGAAAATGAAACATTGGACATAACTTTGGAACCCCCCCCCCACTGGCTGCAAGGAAATAACGGA**

**TTGGATTTTCTAACTCTCTTTAAAGAGACTGGTTTCGAATTCACTTGCTTGCAAGCTTAGGGTTAGCAGG**

**TTGTTGACTGGGAGAGCTGGTAAAAGAATAAAATCTTCAAGAGATCATGGTTAACAAGCCAGCTGAGGAA**

**TAACAATCTTCCTCAGCAATACCAAAAATCCATCCAAGGAATGAGCTTCCCCAAAGTTAATTCGCGTGGA**

**CTACAAAGAAAAGATAGAGTCTCACCCGGTAACATCAGCTGAGTCAATGGCAGAAAGTCTTGAAAAGAAC**

**CTTTTATTCGATGCTTCTGATTCAAGCTTATTCCTTTCCGCCTTTTGAATAAATCTAGAACCTCAATTAG**

**CAGATTCATTAGAAACCTTTCGACCAACATAATCTTTGAATTTTCCGCATATATAACCAGCAGAGCTCTC**

**GTATGAGCAAAGTAACGCCTTCGTCCTTTTTCTCCATGTATGCTTAGGAATCACACCAATCTATCCCGAA**

**TTTGGTGGTTAAACTCTACTGGTATGAGAAGGGTCCTCCTCTCCCTTGTCAAATAAAAATGACACACTAT**

**GTGGGCGGATGGAGAATAACTTTAAGGTCTACATTAAAGAAAAAAAAAGAGCAAAAAGGATAAGATTTAA**

**GTGATTTTTGGTAGTTATTTTCAATTACAATAAAGTCAAATCGTCGTACAACTGTTGTTAGTCATAAAGA**

**AATTAATCAAATATTACCTTTTTCAAATGTTTTCCTACATATTTACAATCTTCAACTTTGTGTAGAGTAT**

**AAGTTTCAGCTAATTTCTTATTTTATAAATTTACTTTTAGATATTTAGTCTCTTAAAGGCTGACAATTTT**

**CCCTGAACGAAACTTTCTCGACAAGACGACCACCATTATTAAAAATAAAAACTAGACTAGAAACACGATT**

**AAATCAATAGGAAAATCTGAGTAGCCTCATTGGCTTGTACTTTTAGCTGTCTATTGCGTACATACTAAGG**

**TGGATTTTAGGCAAGGGAATTACAGGGTATTATAGTCATTCTCGCTCACGTCTATATATATATTCAAACC**

**CTAGCAAAATCAATGGTACGGGCGTCGGGTCGATGAGTAAAGGAGAAGAACTTTTCACTGGAGTTGTCCC**

**AATTCTTGTTGAATTAGATGGTGATGTTAATGGGTACAAATTTTCTGTCAGTGGAGAGGGTGAAGGTGAT**

**GCAACATACGGAAAACTTACCCTTAAATTTATTTGCACTACTGGAAAACTACCTGTTCCATGGCCAACAC**

**TTGTCACTACTTTCTCTTATGGTGTTCAATGCTTTTCAAGATACCCAGATCATATGAAGCGGCACGACTT**

**CTTCAAGAGCGCCATGCCTGAGGGATACGTGCAGGAGAGGACCATCTTCTTCAAGGACGACGGGAACTAC**

**AAGACACGTGCTGAAGTCAAGTTTGAGGGAGACACCCTCGTCAACAGGATCGAGCTTAAGGGAATCGATT**

**TCAAGGAGGACGGAAACATCCTCGGCCACAAGTTGGAATACAACTACAACTCCCACAACGTATACATCAT**

**GGCCGACAAGCAAAAGAACGGCATCAAAGCCAACTTCAAGACCCGCCACAACATCGAAGACGGCGGCGTG**

**CAACTCGCTGATCATTATCAACAAAATACTCCAATTGGCGATGGCCCTGTCCTTTTACCAGACAACCATT**

**ACCTGTCCACACAATCTGCCCTTTCGAAAGATCCCAACGAAAAGAGAGACCACATGGTCCTTCTTGAGTT**

**TGTAACAGCTGCTGGGATTACACATGGCATGGATGAACTATACAAACATGATGAGCTTGATTACGCCGAG**

**GCCTCTTCCCTAATGATATCCCGCGGCCATGCTAGAGTCCGCAAAAATCACCAGTCTCTCTCTACAAATC**

**TATCTCTCTCTATTTTTCTCCAGAATAATGTGTGAGTAGTTCCCAGATAAGGGAATTAGGGTTCTTATAG**

**GGTTTCGCTCATGTGTTGAGCATATAAGAAACCCTTAGTATGTATTTGTATTTGTAAAATACTTCTATCA**

**ATAAAATTTCTAATTCCTAAAACCAAAATCCAGTGACCTGCAGGCATGCGACGTCG**

**>nta-MIR6019, 6020prob-2k::Exon1b (direct) 11618bp**

**GGGTACCCTCGAATTATCATACATGAGAATTAAGGGAGTCACGTTATGACCCCGCCGATGACGCGGGACA**

**AGCCGTTTTACGTTTGGAACTGACAGAACCGCAACGTTGAAGGAGCCACTGAGCCGCGGGTTTCTGGAGT**

**TTAATGAGCTAAGCACATACGTCAGAAACCATTATTGCGCGTTCAAAAGTCGCCTAAGGTCACTATCAGC**

**TAGCAAATATTTCTTGTCAAAAATGCTCCACTGACGTTCCATAAATTCCCCTCGGTATCCAATTAGAGTC**

**TCATATTCACTCTCAACTCGAATCCCCCCTATGAAAAAGCCTGAACTCACCGCGACGTCTGTCGAGAAGT**

**TTCTGATCGAAAAGTTCGACAGCGTCTCCGACCTGATGCAGCTCTCGGAGGGCGAAGAATCTCGTGCTTT**

**CAGCTTCGATGTAGGAGGGCGTGGATATGTCCTGCGGGTAAATAGCTGCGCCGATGGTTTCTACAAAGAT**

**CGTTATGTTTATCGGCACTTTGCATCGGCCGCGCTCCCGATTCCGGAAGTGCTTGACATTGGGGAGTTCA**

**GCGAGAGCCTGACCTATTGCATCTCCCGCCGTGCACAGGGTGTCACGTTGCAAGACCTGCCTGAAACCGA**

**ACTGCCCGCTGTTCTTCAGCCGGTCGCGGAGGCTATGGATGCTATCGCTGCGGCCGATCTTAGCCAGACG**

**AGCGGGTTCGGCCCATTCGGACCGCAAGGAATCGGTCAATACACTACATGGCGTGATTTCATATGCGCGA**

**TTGCTGATCCCCATGTGTATCACTGGCAAACTGTGATGGACGACACCGTCAGTGCGTCCGTCGCGCAGGC**

**TCTCGATGAGCTGATGCTTTGGGCGAGGACTGCCCCGAAGTCCGGCACTCGTGCACGCGGATTCGGCTCC**

**AACAATGTCCTGACGGACAATGGCCGCATAACAGCGGTCATTGACTGGAGCGAGGCGATGTTCGGGGATT**

**CCCAATACGAGGTCGCCAACATCTTCTTCTGGAGGCCGTGGTTGGCTTGTATGGAGCAGCAGACGCGCTA**

**CTTCGAGCGGAGGCATCCGGAGCTTGCAGGATCGCCAAGGCTCCGGGCGTATATGCTCCGCATTGGTCTT**

**GACCAACTCTATCAGAGCTTGGTTGACGGCAATTTCGATGATGCAGCTTGGGCGCAGGGTCGATGCGACG**

**CAATCGTCCGATCCGGAGCCGGGACTGTCGGGCGTACACAAATCGCCCGCAGAAGCGCGGCCGTCTGGAC**

**CGATGGCTGTGTAGAAGTACTCGCCGATAGTGGAAACCGACGCCCCAGCACTCGTCCGAGGGCAAAGGAA**

**TAGAGTAGATGCCGACCGAACAAGAGCTGATTTCGAGAACGCCTCAGCCAGCAACTCGCGCGAGCCTAGC**

**AAGGCAAATGCGAGAGAACGGCCTTACGCTTGGTGGCACAGTTCTCGTCCACAGTTCGCTAAGCTCGCTC**

**GGCTGGGTCGCGGGAGGGCCGGTCGCAGTGATTCAGGAATTAATTCCCTAGAGTCAAGCAGATCGTTCAA**

**ACATTTGGCAATAAAGTTTCTTAAGATTGAATCCTGTTGCCGGTCTTGCGATGATTATCATATAATTTCT**

**GTTGAATTACGTTAAGCATGTAATAATTAACATGTAATGCATGACGTTATTTATGAGATGGGTTTTTATG**

**ATTAGAGTCCCGCAATTATACATTTAATACGCGATAGAAAACAAAATATAGCGCGCAAACTAGGATAAAT**

**TATCGCGCGCGGTGTCATCTATGTTACTAGATCGACCGGCATGCAAGCTGATAATTCAATTCGGCGTTAA**

**TTCAGTACATTAAAAACGTCCGCAATGTGTTATTAAGTTGTCTAAGCGTCAATTTGTTTACACCACAATA**

**TATCCTGCCACCAGCCAGCCAACAGCTCCCCGACCGGCAGCTCGGCACAAAATCACCACTCGATACAGGC**

**AGCCCATCAGTCCGGGACGGCGTCAGCGGGAGAGCCGTTGTAAGGCGGCAGACTTTGCTCATGTTACCGA**

**TGCTATTCGGAAGAACGGCAACTAAGCTGCCGGGTTTGAAACACGGATGATCTCGCGGAGGGTAGCATGT**

**TGATTGTAACGATGACAGAGCGTTGCTGCCTGTGATCAATTCGGGCACGAACCCAGTGGACATAAGCCTC**

**GTTCGGTTCGTAAGCTGTAATGCAAGTAGCGTAACTGCCGTCACGCAACTGGTCCAGAACCTTGACCGAA**

**CGCAGCGGTGGTAACGGCGCAGTGGCGGTTTTCATGGCTTCTTGTTATGACATGTTTTTTTGGGGTACAG**

**TCTATGCCTCGGGCATCCAAGCAGCAAGCGCGTTACGCCGTGGGTCGATGTTTGATGTTATGGAGCAGCA**

**ACGATGTTACGCAGCAGGGCAGTCGCCCTAAAACAAAGTTAAACATCATGGGGGAAGCGGTGATCGCCGA**

**AGTATCGACTCAACTATCAGAGGTAGTTGGCGTCATCGAGCGCCATCTCGAACCGACGTTGCTGGCCGTA**

**CATTTGTACGGCTCCGCAGTGGATGGCGGCCTGAAGCCACACAGTGATATTGATTTGCTGGTTACGGTGA**

**CCGTAAGGCTTGATGAAACAACGCGGCGAGCTTTGATCAACGACCTTTTGGAAACTTCGGCTTCCCCTGG**

**AGAGAGCGAGATTCTCCGCGCTGTAGAAGTCACCATTGTTGTGCACGACGACATCATTCCGTGGCGTTAT**

**CCAGCTAAGCGCGAACTGCAATTTGGAGAATGGCAGCGCAATGACATTCTTGCAGGTATCTTCGAGCCAG**

**CCACGATCGACATTGATCTGGCTATCTTGCTGACAAAAGCAAGAGAACATAGCGTTGCCTTGGTAGGTCC**

**AGCGGCGGAGGAACTCTTTGATCCGGTTCCTGAACAGGATCTATTTGAGGCGCTAAATGAAACCTTAACG**

**CTATGGAACTCGCCGCCCGACTGGGCTGGCGATGAGCGAAATGTAGTGCTTACGTTGTCCCGCATTTGGT**

**ACAGCGCAGTAACCGGCAAAATCGCGCCGAAGGATGTCGCTGCCGACTGGGCAATGGAGCGCCTGCCGGC**

**CCAGTATCAGCCCGTCATACTTGAAGCTAGACAGGCTTATCTTGGACAAGAAGAAGATCGCTTGGCCTCG**

**CGCGCAGATCAGTTGGAAGAATTTGTCCACTACGTGAAAGGCGAGATCACCAAGGTAGTCGGCAAATAAT**

**GTCTAGCTAGAAATTCGTTCAAGCCGACGCCGCTTCGCCGGCGTTAACTCAAGCGATTAGATGCACTAAG**

**CACATAATTGCTCACAGCCAAACTATCAGGTCAAGTCTGCTTTTATTATTTTTAAGCGTGCATAATAAGC**

**CCTACACAAATTGGGAGATATATCATGCATGACCAAAATCCCTTAACGTGAGTTTTCGTTCCACTGAGCG**

**TCAGACCCCGTAGAAAAGATCAAAGGATCTTCTTGAGATCCTTTTTTTCTGCGCGTAATCTGCTGCTTGC**

**AAACAAAAAAACCACCGCTACCAGCGGTGGTTTGTTTGCCGGATCAAGAGCTACCAACTCTTTTTCCGAA**

**GGTAACTGGCTTCAGCAGAGCGCAGATACCAAATACTGTCCTTCTAGTGTAGCCGTAGTTAGGCCACCAC**

**TTCAAGAACTCTGTAGCACCGCCTACATACCTCGCTCTGCTAATCCTGTTACCAGTGGCTGCTGCCAGTG**

**GCGATAAGTCGTGTCTTACCGGGTTGGACTCAAGACGATAGTTACCGGATAAGGCGCAGCGGTCGGGCTG**

**AACGGGGGGTTCGTGCACACAGCCCAGCTTGGAGCGAACGACCTACACCGAACTGAGATACCTACAGCGT**

**GAGCTATGAGAAAGCGCCACGCTTCCCGAAGGGAGAAAGGCGGACAGGTATCCGGTAAGCGGCAGGGTCG**

**GAACAGGAGAGCGCACGAGGGAGCTTCCAGGGGGAAACGCCTGGTATCTTTATAGTCCTGTCGGGTTTCG**

**CCACCTCTGACTTGAGCGTCGATTTTTGTGATGCTCGTCAGGGGGGCGGAGCCTATGGAAAAACGCCAGC**

**AACGCGGCCTTTTTACGGTTCCTGGCCTTTTGCTGGCCTTTTGCTCACATGTTCTTTCCTGCGTTATCCC**

**CTGATTCTGTGGATAACCGTATTACCGCCTTTGAGTGAGCTGATACCGCTCGCCGCAGCCGAACGACCGA**

**GCGCAGCGAGTCAGTGAGCGAGGAAGCGGAAGAGCGCCTGATGCGGTATTTTCTCCTTACGCATCTGTGC**

**GGTATTTCACACCGCATATGGTGCACTCTCAGTACAATCTGCTCTGATGCCGCATAGTTAAGCCAGTATA**

**CACTCCGCTATCGCTACGTGACTGGGTCATGGCTGCGCCCCGACACCCGCCAACACCCGCTGACGCGCCC**

**TGACGGGCTTGTCTGCTCCCGGCATCCGCTTACAGACAAGCTGTGACCGTCTCCGGGAGCTGCATGTGTC**

**AGAGGTTTTCACCGTCATCACCGAAACGCGCGAGGCAGGGTGCCTTGATGTGGGCGCCGGCGGTCGAGTG**

**GCGACGGCGCGGCTTGTCCGCGCCCTGGTAGATTGCCTGGCCGTAGGCCAGCCATTTTTGAGCGGCCAGC**

**GGCCGCGATAGGCCGACGCGAAGCGGCGGGGCGTAGGGAGCGCAGCGACCGAAGGGTAGGCGCTTTTTGC**

**AGCTCTTCGGCTGTGCGCTGGCCAGACAGTTATGCACAGGCCAGGCGGGTTTTAAGAGTTTTAATAAGTT**

**TTAAAGAGTTTTAGGCGGAAAAATCGCCTTTTTTCTCTTTTATATCAGTCACTTACATGTGTGACCGGTT**

**CCCAATGTACGGCTTTGGGTTCCCAATGTACGGGTTCCGGTTCCCAATGTACGGCTTTGGGTTCCCAATG**

**TACGTGCTATCCACAGGAAAGAGACCTTTTCGACCTTTTTCCCCTGCTAGGGCAATTTGCCCTAGCATCT**

**GCTCCGTACATTAGGAACCGGCGGATGCTTCGCCCTCGATCAGGTTGCGGTAGCGCATGACTAGGATCGG**

**GCCAGCCTGCCCCGCCTCCTCCTTCAAATCGTACTCCGGCAGGTCATTTGACCCGATCAGCTTGCGCACG**

**GTGAAACAGAACTTCTTGAACTCTCCGGCGCTGCCACTGCGTTCGTAGATCGTCTTGAACAACCATCTGG**

**CTTCTGCCTTGCCTGCGGCGCGGCGTGCCAGGCGGTAGAGAAAACGGCCGATGCCGGGATCGATCAAAAA**

**GTAATCGGGGTGAACCGTCAGCACGTCCGGGTTCTTGCCTTCTGTGATCTCGCGGTACATCCAATCAGCT**

**AGCTCGATCTCGATGTACTCCGGCCGCCCGGTTTCGCTCTTTACGATCTTGTAGCGGCTAATCAAGGCTT**

**CACCCTCGGATACCGTCACCAGGCGGCCGTTCTTGGCCTTCTTCGTACGCTGCATGGCAACGTGCGTGGT**

**GTTTAACCGAATGCAGGTTTCTACCAGGTCGTCTTTCTGCTTTCCGCCATCGGCTCGCCGGCAGAACTTG**

**AGTACGTCCGCAACGTGTGGACGGAACACGCGGCCGGGCTTGTCTCCCTTCCCTTCCCGGTATCGGTTCA**

**TGGATTCGGTTAGATGGGAAACCGCCATCAGTACCAGGTCGTAATCCCACACACTGGCCATGCCGGCCGG**

**CCCTGCGGAAACCTCTACGTGCCCGTCTGGAAGCTCGTAGCGGATCACCTCGCCAGCTCGTCGGTCACGC**

**TTCGACAGACGGAAAACGGCCACGTCCATGATGCTGCGACTATCGCGGGTGCCCACGTCATAGAGCATCG**

**GAACGAAAAAATCTGGTTGCTCGTCGCCCTTGGGCGGCTTCCTAATCGACGGCGCACCGGCTGCCGGCGG**

**TTGCCGGGATTCTTTGCGGATTCGATCAGCGGCCGCTTGCCACGATTCACCGGGGCGTGCTTCTGCCTCG**

**ATGCGTTGCCGCTGGGCGGCCTGCGCGGCCTTCAACTTCTCCACCAGGTCATCACCCAGCGCCGCGCCGA**

**TTTGTACCGGGCCGGATGGTTTGCGACCGTCACGCCGATTCCTCGGGCTTGGGGGTTCCAGTGCCATTGC**

**AGGGCCGGCAGACAACCCAGCCGCTTACGCCTGGCCAACCGCCCGTTCCTCCACACATGGGGCATTCCAC**

**GGCGTCGGTGCCTGGTTGTTCTTGATTTTCCATGCCGCCTCCTTTAGCCGCTAAAATTCATCTACTCATT**

**TATTCATTTGCTCATTTACTCTGGTAGCTGCGCGATGTATTCAGATAGCAGCTCGGTAATGGTCTTGCCT**

**TGGCGTACCGCGTACATCTTCAGCTTGGTGTGATCCTCCGCCGGCAACTGAAAGTTGACCCGCTTCATGG**

**CTGGCGTGTCTGCCAGGCTGGCCAACGTTGCAGCCTTGCTGCTGCGTGCGCTCGGACGGCCGGCACTTAG**

**CGTGTTTGTGCTTTTGCTCATTTTCTCTTTACCTCATTAACTCAAATGAGTTTTGATTTAATTTCAGCGG**

**CCAGCGCCTGGACCTCGCGGGCAGCGTCGCCCTCGGGTTCTGATTCAAGAACGGTTGTGCCGGCGGCGGC**

**AGTGCCTGGGTAGCTCACGCGCTGCGTGATACGGGACTCAAGAATGGGCAGCTCGTACCCGGCCAGCGCC**

**TCGGCAACCTCACCGCCGATGCGCGTGCCTTTGATCGCCCGCGACACGACAAAGGCCGCTTGTAGCCTTC**

**CATCCGTGACCTCAATGCGCTGCTTAACCAGCTCCACCAGGTCGGCGGTGGCCCATATGTCGTAAGGGCT**

**TGGCTGCACCGGAATCAGCACGAAGTCGGCTGCCTTGATCGCGGACACAGCCAAGTCCGCCGCCTGGGGC**

**GCTCCGTCGATCACTACGAAGTCGCGCCGGCCGATGGCCTTCACGTCGCGGTCAATCGTCGGGCGGTCGA**

**TGCCGACAACGGTTAGCGGTTGATCTTCCCGCACGGCCGCCCAATCGCGGGCACTGCCCTGGGGATCGGA**

**ATCGACTAACAGAACATCGGCCCCGGCGAGTTGCAGGGCGCGGGCTAGATGGGTTGCGATGGTCGTCTTG**

**CCTGACCCGCCTTTCTGGTTAAGTACAGCGATAACCTTCATGCGTTCCCCTTGCGTATTTGTTTATTTAC**

**TCATCGCATCATATACGCAGCGACCGCATGACGCAAGCTGTTTTACTCAAATACACATCACCTTTTTAGA**

**CGGCGGCGCTCGGTTTCTTCAGCGGCCAAGCTGGCCGGCCAGGCCGCCAGCTTGGCATCAGACAAACCGG**

**CCAGGATTTCATGCAGCCGCACGGTTGAGACGTGCGCGGGCGGCTCGAACACGTACCCGGCCGCGATCAT**

**CTCCGCCTCGATCTCTTCGGTAATGAAAAACGGTTCGTCCTGGCCGTCCTGGTGCGGTTTCATGCTTGTT**

**CCTCTTGGCGTTCATTCTCGGCGGCCGCCAGGGCGTCGGCCTCGGTCAATGCGTCCTCACGGAAGGCACC**

**GCGCCGCCTGGCCTCGGTGGGCGTCACTTCCTCGCTGCGCTCAAGTGCGCGGTACAGGGTCGAGCGATGC**

**ACGCCAAGCAGTGCAGCCGCCTCTTTCACGGTGCGGCCTTCCTGGTCGATCAGCTCGCGGGCGTGCGCGA**

**TCTGTGCCGGGGTGAGGGTAGGGCGGGGGCCAAACTTCACGCCTCGGGCCTTGGCGGCCTCGCGCCCGCT**

**CCGGGTGCGGTCGATGATTAGGGAACGCTCGAACTCGGCAATGCCGGCGAACACGGTCAACACCATGCGG**

**CCGGCCGGCGTGGTGGTGTCGGCCCACGGCTCTGCCAGGCTACGCAGGCCCGCGCCGGCCTCCTGGATGC**

**GCTCGGCAATGTCCAGTAGGTCGCGGGTGCTGCGGGCCAGGCGGTCTAGCCTGGTCACTGTCACAACGTC**

**GCCAGGGCGTAGGTGGTCAAGCATCCTGGCCAGCTCCGGGCGGTCGCGCCTGGTGCCGGTGATCTTCTCG**

**GAAAACAGCTTGGTGCAGCCGGCCGCGTGCAGTTCGGCCCGTTGGTTGGTCAAGTCCTGGTCGTCGGTGC**

**TGACGCGGGCATAGCCCAGCAGGCCAGCGGCGGCGCTCTTGTTCATGGCGTAATGTCTCCGGTTCTAGTC**

**GCAAGTATTCTACTTTATGCGACTAAAACACGCGACAAGAAAACGCCAGGAAAAGGGCAGGGCGGCAGCC**

**TGTCGCGTAACTTAGGACTTGTGCGACATGTCGTTTTCAGAAGACGGCTGCACTGAACGTCAGAAGCCGA**

**CTGCACTATAGCAGCGGAGGGGTTGGATCAAAGTACTTTGATCCCGAGGGGAACCCTGTGGTTGGCATGC**

**ACATACAAATGGACGAACGGATAAACCTTTTCACGCCCTTTTAAATATCCGTTATTCTAATAAACGCTCT**

**TTTCTCTTAGGTTTACCCGCCAATATATCCTGTCAAACACTGATAGTTTAAACTGAAGGCGGGAAACGAC**

**AATCTGATCCAAGCTCAAGCTAAGCTTGAGCTCTGCATGCCTGCAGGTCCCAGGGCGCCCTGTTGAATCT**

**TTTGAAAGAAAACTACACTATCCAGGTTCTGTTTAAGTATTAAAATTGCAATAAAAACAGAAGGTAAATG**

**GATTCAAGTTGCATTTTAACCTTGAAAGTGCCGGTCTCTTTTCTTTTTCCCATTCAAAATTGCTCTCCTC**

**TTCTCAATTGAGAATGTCTTTCTGGACAGACGATTGACACCTCAATGGAGTAAAGATATTAGGGGTATGG**

**AACTTTATTTCTTCTCACTCCGAGGAAGGTTCACTAGTCCTTCTGCTTTGTTATTAGGCAGACCCCTGTA**

**GGCCTATCGACCAATGACTGATGTGATCCCTACTTGCGTAATAGAAAAATGGATTTCGTTGTGGAGGTAA**

**ATGAATGATGAAATTAAGGATATCACGTAACTTGATTGTGTACAAAGGCAACTTGGTCTCTGGCTATAAA**

**GTTTCCAGTATTTTGCACCGCTTGTGAATTATAAGAGGAGGATCCCCCGCTGCAAGGAAACAACGGATCG**

**ACTTTTCTAACTCTCTTTAAAGAGTCTGTTTTCAAATTCACTTGCTTGCAAGTTTTGGGTTAGCAGGTTG**

**TTGACTGGGAGAGCTGGTAAAAGGGAACAACAATCTCCCCCAGCAATACCAAAGATCCATCCAAGGAATG**

**AGCTTCCCTTCCTGTGGACTACAAGGAAAAGATAGAATCTCGTCCGGCAACAGTAGCTGAGTCAATGACA**

**GATGCTCTTGGAAAGAACCTTTCATTCGATGCTTCTGATTCAAGCTTATTCTTTTCTACCTTTTGAATTA**

**ATCTAGAAGCTCAATTAGCAGATTCATTAGAAACCTTTCGACCAGTATAATCATTAAATTTCTCGCATAT**

**ATAACCAGCATAGCTCTTGTATGAGCGAAGTAATGCCTTCATCCTTTCTCTCCATGTATGCTTAGGAACC**

**ATACCAATCCATCCCGAACTTAGTGGTTAAACTCAACAAGTATGGGAAGGGTCCTCCTCTCCCTGGTCAA**

**AGCTCCGTGAACGAGAACTCGTTTTGTTTGTTGGCCGGCTAATGATGGTTTTCTGTCCCAATTCAACCTT**

**ACAGTCTTCGGATGACCCAAAAAAGCTAATACCTTTTTTCTCACAAGCTATTAGTACTACTTTTCACTGG**

**AATGCCACTTTGGAAGTTAAATTGAAAGCAATTATTGTTCATTTTAGAAATTTTCACATTGAGAAAAGTA**

**ACATGTCTTGATATTTTATTGCCGTATGTAAACTCAACTCCACTTTAAAATCAATCCTGGTTAAAAGTGA**

**GATAATAAAAAATGATACAAGATTACCACAACCGGGGGAGAGGGATAGTTACTTTCCTATTTGTGTCTCG**

**GAAAAAATGATGAGATAATAAAAATGACACACAATGTGGGGGATGGAGAATAATTTTGAAGTCTACATTA**

**AGGGAAAAAAAGAGAGCAAAAAGGATAAGATTTAAGTGTTTTTTGGTAGTTATTTTCAATTACAATAAAG**

**TCAAGTCCTAGTACAACTGTTGTTAGTCATAAATAAATTAATCAAATATTACATTTTCCCAATGTTTTCC**

**TACATATTTACAATCTTCAACTTATTGTGGAGTAAAGTTTCAGCTAATGGTCGGGTTCAAGTATTTTAGT**

**ATTAACCCGTTTGACATTGATGAAAAAACCAGGTGACAACATTTAATTGAATATTTATTACACCTCAGAT**

**TTGTATATACATACGCGCTATATTTTTTGCAATTGTTCCAGCTTTTGTGTTAAAGTGGAACATAAGAAAT**

**AAAATTGCAGGGCCAAAATGGAACAAACAAGATAAAGTTGGAGGGTCAAAATAGAACAAGATAGAGATAG**

**AGTGTCAAAATGAAAAATGGTGTCAAGTTGAATTGGCTGTATATGTATTTGGTCAAAATAAAAACTAGAC**

**TAGAAACACGAGTAAATCAATAGGAAAATCTGAGTCGCCTCATTGGCTTGTACATTTAGCTGTCTATTGT**

**GTACATACTAAGGTGGATTACGCAAGTGAAATACAGGGTATTATAGTCATTCTCGCTCACGTCTATATAT**

**ATATATTCAAACCCTAGCTAAATCAATGGTCGGGCGTCGGGTCGATGAGTAAAGGAGAAGAACTTTTCAC**

**TGGAGTTGTCCCAATTCTTGTTGAATTAGATGGTGATGTTAATGGGTACAAATTTTCTGTCAGTGGAGAG**

**GGTGAAGGTGATGCAACATACGGAAAACTTACCCTTAAATTTATTTGCACTACTGGAAAACTACCTGTTC**

**CATGGCCAACACTTGTCACTACTTTCTCTTATGGTGTTCAATGCTTTTCAAGATACCCAGATCATATGAA**

**GCGGCACGACTTCTTCAAGAGCGCCATGCCTGAGGGATACGTGCAGGAGAGGACCATCTTCTTCAAGGAC**

**GACGGGAACTACAAGACACGTGCTGAAGTCAAGTTTGAGGGAGACACCCTCGTCAACAGGATCGAGCTTA**

**AGGGAATCGATTTCAAGGAGGACGGAAACATCCTCGGCCACAAGTTGGAATACAACTACAACTCCCACAA**

**CGTATACATCATGGCCGACAAGCAAAAGAACGGCATCAAAGCCAACTTCAAGACCCGCCACAACATCGAA**

**GACGGCGGCGTGCAACTCGCTGATCATTATCAACAAAATACTCCAATTGGCGATGGCCCTGTCCTTTTAC**

**CAGACAACCATTACCTGTCCACACAATCTGCCCTTTCGAAAGATCCCAACGAAAAGAGAGACCACATGGT**

**CCTTCTTGAGTTTGTAACAGCTGCTGGGATTACACATGGCATGGATGAACTATACAAACATGATGAGCTT**

**GATTACGCCGAGGCCTCTTCCCTAATGATATCCCGCGGCCATGCTAGAGTCCGCAAAAATCACCAGTCTC**

**TCTCTACAAATCTATCTCTCTCTATTTTTCTCCAGAATAATGTGTGAGTAGTTCCCAGATAAGGGAATTA**

**GGGTTCTTATAGGGTTTCGCTCATGTGTTGAGCATATAAGAAACCCTTAGTATGTATTTGTATTTGTAAA**

**ATACTTCTATCAATAAAATTTCTAATTCCTAAAACCAAAATCCAGTGACCTGCAGGCATGCGACGTCG**

**>nta-MIR6019, 6020prob-4k::Exon1b (direct) 13502bp**

**GGGTACCCTCGAATTATCATACATGAGAATTAAGGGAGTCACGTTATGACCCCGCCGATGACGCGGGACA**

**AGCCGTTTTACGTTTGGAACTGACAGAACCGCAACGTTGAAGGAGCCACTGAGCCGCGGGTTTCTGGAGT**

**TTAATGAGCTAAGCACATACGTCAGAAACCATTATTGCGCGTTCAAAAGTCGCCTAAGGTCACTATCAGC**

**TAGCAAATATTTCTTGTCAAAAATGCTCCACTGACGTTCCATAAATTCCCCTCGGTATCCAATTAGAGTC**

**TCATATTCACTCTCAACTCGAATCCCCCCTATGAAAAAGCCTGAACTCACCGCGACGTCTGTCGAGAAGT**

**TTCTGATCGAAAAGTTCGACAGCGTCTCCGACCTGATGCAGCTCTCGGAGGGCGAAGAATCTCGTGCTTT**

**CAGCTTCGATGTAGGAGGGCGTGGATATGTCCTGCGGGTAAATAGCTGCGCCGATGGTTTCTACAAAGAT**

**CGTTATGTTTATCGGCACTTTGCATCGGCCGCGCTCCCGATTCCGGAAGTGCTTGACATTGGGGAGTTCA**

**GCGAGAGCCTGACCTATTGCATCTCCCGCCGTGCACAGGGTGTCACGTTGCAAGACCTGCCTGAAACCGA**

**ACTGCCCGCTGTTCTTCAGCCGGTCGCGGAGGCTATGGATGCTATCGCTGCGGCCGATCTTAGCCAGACG**

**AGCGGGTTCGGCCCATTCGGACCGCAAGGAATCGGTCAATACACTACATGGCGTGATTTCATATGCGCGA**

**TTGCTGATCCCCATGTGTATCACTGGCAAACTGTGATGGACGACACCGTCAGTGCGTCCGTCGCGCAGGC**

**TCTCGATGAGCTGATGCTTTGGGCGAGGACTGCCCCGAAGTCCGGCACTCGTGCACGCGGATTCGGCTCC**

**AACAATGTCCTGACGGACAATGGCCGCATAACAGCGGTCATTGACTGGAGCGAGGCGATGTTCGGGGATT**

**CCCAATACGAGGTCGCCAACATCTTCTTCTGGAGGCCGTGGTTGGCTTGTATGGAGCAGCAGACGCGCTA**

**CTTCGAGCGGAGGCATCCGGAGCTTGCAGGATCGCCAAGGCTCCGGGCGTATATGCTCCGCATTGGTCTT**

**GACCAACTCTATCAGAGCTTGGTTGACGGCAATTTCGATGATGCAGCTTGGGCGCAGGGTCGATGCGACG**

**CAATCGTCCGATCCGGAGCCGGGACTGTCGGGCGTACACAAATCGCCCGCAGAAGCGCGGCCGTCTGGAC**

**CGATGGCTGTGTAGAAGTACTCGCCGATAGTGGAAACCGACGCCCCAGCACTCGTCCGAGGGCAAAGGAA**

**TAGAGTAGATGCCGACCGAACAAGAGCTGATTTCGAGAACGCCTCAGCCAGCAACTCGCGCGAGCCTAGC**

**AAGGCAAATGCGAGAGAACGGCCTTACGCTTGGTGGCACAGTTCTCGTCCACAGTTCGCTAAGCTCGCTC**

**GGCTGGGTCGCGGGAGGGCCGGTCGCAGTGATTCAGGAATTAATTCCCTAGAGTCAAGCAGATCGTTCAA**

**ACATTTGGCAATAAAGTTTCTTAAGATTGAATCCTGTTGCCGGTCTTGCGATGATTATCATATAATTTCT**

**GTTGAATTACGTTAAGCATGTAATAATTAACATGTAATGCATGACGTTATTTATGAGATGGGTTTTTATG**

**ATTAGAGTCCCGCAATTATACATTTAATACGCGATAGAAAACAAAATATAGCGCGCAAACTAGGATAAAT**

**TATCGCGCGCGGTGTCATCTATGTTACTAGATCGACCGGCATGCAAGCTGATAATTCAATTCGGCGTTAA**

**TTCAGTACATTAAAAACGTCCGCAATGTGTTATTAAGTTGTCTAAGCGTCAATTTGTTTACACCACAATA**

**TATCCTGCCACCAGCCAGCCAACAGCTCCCCGACCGGCAGCTCGGCACAAAATCACCACTCGATACAGGC**

**AGCCCATCAGTCCGGGACGGCGTCAGCGGGAGAGCCGTTGTAAGGCGGCAGACTTTGCTCATGTTACCGA**

**TGCTATTCGGAAGAACGGCAACTAAGCTGCCGGGTTTGAAACACGGATGATCTCGCGGAGGGTAGCATGT**

**TGATTGTAACGATGACAGAGCGTTGCTGCCTGTGATCAATTCGGGCACGAACCCAGTGGACATAAGCCTC**

**GTTCGGTTCGTAAGCTGTAATGCAAGTAGCGTAACTGCCGTCACGCAACTGGTCCAGAACCTTGACCGAA**

**CGCAGCGGTGGTAACGGCGCAGTGGCGGTTTTCATGGCTTCTTGTTATGACATGTTTTTTTGGGGTACAG**

**TCTATGCCTCGGGCATCCAAGCAGCAAGCGCGTTACGCCGTGGGTCGATGTTTGATGTTATGGAGCAGCA**

**ACGATGTTACGCAGCAGGGCAGTCGCCCTAAAACAAAGTTAAACATCATGGGGGAAGCGGTGATCGCCGA**

**AGTATCGACTCAACTATCAGAGGTAGTTGGCGTCATCGAGCGCCATCTCGAACCGACGTTGCTGGCCGTA**

**CATTTGTACGGCTCCGCAGTGGATGGCGGCCTGAAGCCACACAGTGATATTGATTTGCTGGTTACGGTGA**

**CCGTAAGGCTTGATGAAACAACGCGGCGAGCTTTGATCAACGACCTTTTGGAAACTTCGGCTTCCCCTGG**

**AGAGAGCGAGATTCTCCGCGCTGTAGAAGTCACCATTGTTGTGCACGACGACATCATTCCGTGGCGTTAT**

**CCAGCTAAGCGCGAACTGCAATTTGGAGAATGGCAGCGCAATGACATTCTTGCAGGTATCTTCGAGCCAG**

**CCACGATCGACATTGATCTGGCTATCTTGCTGACAAAAGCAAGAGAACATAGCGTTGCCTTGGTAGGTCC**

**AGCGGCGGAGGAACTCTTTGATCCGGTTCCTGAACAGGATCTATTTGAGGCGCTAAATGAAACCTTAACG**

**CTATGGAACTCGCCGCCCGACTGGGCTGGCGATGAGCGAAATGTAGTGCTTACGTTGTCCCGCATTTGGT**

**ACAGCGCAGTAACCGGCAAAATCGCGCCGAAGGATGTCGCTGCCGACTGGGCAATGGAGCGCCTGCCGGC**

**CCAGTATCAGCCCGTCATACTTGAAGCTAGACAGGCTTATCTTGGACAAGAAGAAGATCGCTTGGCCTCG**

**CGCGCAGATCAGTTGGAAGAATTTGTCCACTACGTGAAAGGCGAGATCACCAAGGTAGTCGGCAAATAAT**

**GTCTAGCTAGAAATTCGTTCAAGCCGACGCCGCTTCGCCGGCGTTAACTCAAGCGATTAGATGCACTAAG**

**CACATAATTGCTCACAGCCAAACTATCAGGTCAAGTCTGCTTTTATTATTTTTAAGCGTGCATAATAAGC**

**CCTACACAAATTGGGAGATATATCATGCATGACCAAAATCCCTTAACGTGAGTTTTCGTTCCACTGAGCG**

**TCAGACCCCGTAGAAAAGATCAAAGGATCTTCTTGAGATCCTTTTTTTCTGCGCGTAATCTGCTGCTTGC**

**AAACAAAAAAACCACCGCTACCAGCGGTGGTTTGTTTGCCGGATCAAGAGCTACCAACTCTTTTTCCGAA**

**GGTAACTGGCTTCAGCAGAGCGCAGATACCAAATACTGTCCTTCTAGTGTAGCCGTAGTTAGGCCACCAC**

**TTCAAGAACTCTGTAGCACCGCCTACATACCTCGCTCTGCTAATCCTGTTACCAGTGGCTGCTGCCAGTG**

**GCGATAAGTCGTGTCTTACCGGGTTGGACTCAAGACGATAGTTACCGGATAAGGCGCAGCGGTCGGGCTG**

**AACGGGGGGTTCGTGCACACAGCCCAGCTTGGAGCGAACGACCTACACCGAACTGAGATACCTACAGCGT**

**GAGCTATGAGAAAGCGCCACGCTTCCCGAAGGGAGAAAGGCGGACAGGTATCCGGTAAGCGGCAGGGTCG**

**GAACAGGAGAGCGCACGAGGGAGCTTCCAGGGGGAAACGCCTGGTATCTTTATAGTCCTGTCGGGTTTCG**

**CCACCTCTGACTTGAGCGTCGATTTTTGTGATGCTCGTCAGGGGGGCGGAGCCTATGGAAAAACGCCAGC**

**AACGCGGCCTTTTTACGGTTCCTGGCCTTTTGCTGGCCTTTTGCTCACATGTTCTTTCCTGCGTTATCCC**

**CTGATTCTGTGGATAACCGTATTACCGCCTTTGAGTGAGCTGATACCGCTCGCCGCAGCCGAACGACCGA**

**GCGCAGCGAGTCAGTGAGCGAGGAAGCGGAAGAGCGCCTGATGCGGTATTTTCTCCTTACGCATCTGTGC**

**GGTATTTCACACCGCATATGGTGCACTCTCAGTACAATCTGCTCTGATGCCGCATAGTTAAGCCAGTATA**

**CACTCCGCTATCGCTACGTGACTGGGTCATGGCTGCGCCCCGACACCCGCCAACACCCGCTGACGCGCCC**

**TGACGGGCTTGTCTGCTCCCGGCATCCGCTTACAGACAAGCTGTGACCGTCTCCGGGAGCTGCATGTGTC**

**AGAGGTTTTCACCGTCATCACCGAAACGCGCGAGGCAGGGTGCCTTGATGTGGGCGCCGGCGGTCGAGTG**

**GCGACGGCGCGGCTTGTCCGCGCCCTGGTAGATTGCCTGGCCGTAGGCCAGCCATTTTTGAGCGGCCAGC**

**GGCCGCGATAGGCCGACGCGAAGCGGCGGGGCGTAGGGAGCGCAGCGACCGAAGGGTAGGCGCTTTTTGC**

**AGCTCTTCGGCTGTGCGCTGGCCAGACAGTTATGCACAGGCCAGGCGGGTTTTAAGAGTTTTAATAAGTT**

**TTAAAGAGTTTTAGGCGGAAAAATCGCCTTTTTTCTCTTTTATATCAGTCACTTACATGTGTGACCGGTT**

**CCCAATGTACGGCTTTGGGTTCCCAATGTACGGGTTCCGGTTCCCAATGTACGGCTTTGGGTTCCCAATG**

**TACGTGCTATCCACAGGAAAGAGACCTTTTCGACCTTTTTCCCCTGCTAGGGCAATTTGCCCTAGCATCT**

**GCTCCGTACATTAGGAACCGGCGGATGCTTCGCCCTCGATCAGGTTGCGGTAGCGCATGACTAGGATCGG**

**GCCAGCCTGCCCCGCCTCCTCCTTCAAATCGTACTCCGGCAGGTCATTTGACCCGATCAGCTTGCGCACG**

**GTGAAACAGAACTTCTTGAACTCTCCGGCGCTGCCACTGCGTTCGTAGATCGTCTTGAACAACCATCTGG**

**CTTCTGCCTTGCCTGCGGCGCGGCGTGCCAGGCGGTAGAGAAAACGGCCGATGCCGGGATCGATCAAAAA**

**GTAATCGGGGTGAACCGTCAGCACGTCCGGGTTCTTGCCTTCTGTGATCTCGCGGTACATCCAATCAGCT**

**AGCTCGATCTCGATGTACTCCGGCCGCCCGGTTTCGCTCTTTACGATCTTGTAGCGGCTAATCAAGGCTT**

**CACCCTCGGATACCGTCACCAGGCGGCCGTTCTTGGCCTTCTTCGTACGCTGCATGGCAACGTGCGTGGT**

**GTTTAACCGAATGCAGGTTTCTACCAGGTCGTCTTTCTGCTTTCCGCCATCGGCTCGCCGGCAGAACTTG**

**AGTACGTCCGCAACGTGTGGACGGAACACGCGGCCGGGCTTGTCTCCCTTCCCTTCCCGGTATCGGTTCA**

**TGGATTCGGTTAGATGGGAAACCGCCATCAGTACCAGGTCGTAATCCCACACACTGGCCATGCCGGCCGG**

**CCCTGCGGAAACCTCTACGTGCCCGTCTGGAAGCTCGTAGCGGATCACCTCGCCAGCTCGTCGGTCACGC**

**TTCGACAGACGGAAAACGGCCACGTCCATGATGCTGCGACTATCGCGGGTGCCCACGTCATAGAGCATCG**

**GAACGAAAAAATCTGGTTGCTCGTCGCCCTTGGGCGGCTTCCTAATCGACGGCGCACCGGCTGCCGGCGG**

**TTGCCGGGATTCTTTGCGGATTCGATCAGCGGCCGCTTGCCACGATTCACCGGGGCGTGCTTCTGCCTCG**

**ATGCGTTGCCGCTGGGCGGCCTGCGCGGCCTTCAACTTCTCCACCAGGTCATCACCCAGCGCCGCGCCGA**

**TTTGTACCGGGCCGGATGGTTTGCGACCGTCACGCCGATTCCTCGGGCTTGGGGGTTCCAGTGCCATTGC**

**AGGGCCGGCAGACAACCCAGCCGCTTACGCCTGGCCAACCGCCCGTTCCTCCACACATGGGGCATTCCAC**

**GGCGTCGGTGCCTGGTTGTTCTTGATTTTCCATGCCGCCTCCTTTAGCCGCTAAAATTCATCTACTCATT**

**TATTCATTTGCTCATTTACTCTGGTAGCTGCGCGATGTATTCAGATAGCAGCTCGGTAATGGTCTTGCCT**

**TGGCGTACCGCGTACATCTTCAGCTTGGTGTGATCCTCCGCCGGCAACTGAAAGTTGACCCGCTTCATGG**

**CTGGCGTGTCTGCCAGGCTGGCCAACGTTGCAGCCTTGCTGCTGCGTGCGCTCGGACGGCCGGCACTTAG**

**CGTGTTTGTGCTTTTGCTCATTTTCTCTTTACCTCATTAACTCAAATGAGTTTTGATTTAATTTCAGCGG**

**CCAGCGCCTGGACCTCGCGGGCAGCGTCGCCCTCGGGTTCTGATTCAAGAACGGTTGTGCCGGCGGCGGC**

**AGTGCCTGGGTAGCTCACGCGCTGCGTGATACGGGACTCAAGAATGGGCAGCTCGTACCCGGCCAGCGCC**

**TCGGCAACCTCACCGCCGATGCGCGTGCCTTTGATCGCCCGCGACACGACAAAGGCCGCTTGTAGCCTTC**

**CATCCGTGACCTCAATGCGCTGCTTAACCAGCTCCACCAGGTCGGCGGTGGCCCATATGTCGTAAGGGCT**

**TGGCTGCACCGGAATCAGCACGAAGTCGGCTGCCTTGATCGCGGACACAGCCAAGTCCGCCGCCTGGGGC**

**GCTCCGTCGATCACTACGAAGTCGCGCCGGCCGATGGCCTTCACGTCGCGGTCAATCGTCGGGCGGTCGA**

**TGCCGACAACGGTTAGCGGTTGATCTTCCCGCACGGCCGCCCAATCGCGGGCACTGCCCTGGGGATCGGA**

**ATCGACTAACAGAACATCGGCCCCGGCGAGTTGCAGGGCGCGGGCTAGATGGGTTGCGATGGTCGTCTTG**

**CCTGACCCGCCTTTCTGGTTAAGTACAGCGATAACCTTCATGCGTTCCCCTTGCGTATTTGTTTATTTAC**

**TCATCGCATCATATACGCAGCGACCGCATGACGCAAGCTGTTTTACTCAAATACACATCACCTTTTTAGA**

**CGGCGGCGCTCGGTTTCTTCAGCGGCCAAGCTGGCCGGCCAGGCCGCCAGCTTGGCATCAGACAAACCGG**

**CCAGGATTTCATGCAGCCGCACGGTTGAGACGTGCGCGGGCGGCTCGAACACGTACCCGGCCGCGATCAT**

**CTCCGCCTCGATCTCTTCGGTAATGAAAAACGGTTCGTCCTGGCCGTCCTGGTGCGGTTTCATGCTTGTT**

**CCTCTTGGCGTTCATTCTCGGCGGCCGCCAGGGCGTCGGCCTCGGTCAATGCGTCCTCACGGAAGGCACC**

**GCGCCGCCTGGCCTCGGTGGGCGTCACTTCCTCGCTGCGCTCAAGTGCGCGGTACAGGGTCGAGCGATGC**

**ACGCCAAGCAGTGCAGCCGCCTCTTTCACGGTGCGGCCTTCCTGGTCGATCAGCTCGCGGGCGTGCGCGA**

**TCTGTGCCGGGGTGAGGGTAGGGCGGGGGCCAAACTTCACGCCTCGGGCCTTGGCGGCCTCGCGCCCGCT**

**CCGGGTGCGGTCGATGATTAGGGAACGCTCGAACTCGGCAATGCCGGCGAACACGGTCAACACCATGCGG**

**CCGGCCGGCGTGGTGGTGTCGGCCCACGGCTCTGCCAGGCTACGCAGGCCCGCGCCGGCCTCCTGGATGC**

**GCTCGGCAATGTCCAGTAGGTCGCGGGTGCTGCGGGCCAGGCGGTCTAGCCTGGTCACTGTCACAACGTC**

**GCCAGGGCGTAGGTGGTCAAGCATCCTGGCCAGCTCCGGGCGGTCGCGCCTGGTGCCGGTGATCTTCTCG**

**GAAAACAGCTTGGTGCAGCCGGCCGCGTGCAGTTCGGCCCGTTGGTTGGTCAAGTCCTGGTCGTCGGTGC**

**TGACGCGGGCATAGCCCAGCAGGCCAGCGGCGGCGCTCTTGTTCATGGCGTAATGTCTCCGGTTCTAGTC**

**GCAAGTATTCTACTTTATGCGACTAAAACACGCGACAAGAAAACGCCAGGAAAAGGGCAGGGCGGCAGCC**

**TGTCGCGTAACTTAGGACTTGTGCGACATGTCGTTTTCAGAAGACGGCTGCACTGAACGTCAGAAGCCGA**

**CTGCACTATAGCAGCGGAGGGGTTGGATCAAAGTACTTTGATCCCGAGGGGAACCCTGTGGTTGGCATGC**

**ACATACAAATGGACGAACGGATAAACCTTTTCACGCCCTTTTAAATATCCGTTATTCTAATAAACGCTCT**

**TTTCTCTTAGGTTTACCCGCCAATATATCCTGTCAAACACTGATAGTTTAAACTGAAGGCGGGAAACGAC**

**AATCTGATCCAAGCTCAAGCTAAGCTTGAGCTCTGCATGCCTGCAGGTCCCAGGGCGCCCTGAAGCCAGG**

**ACGGGAAAATGGTGTCACCTCTTTATCAAAAGAAGCACTGAATCCAGCTAACAATACTGGCGGTAGATTA**

**GCTAATTGCTCTCCTGCAGTGGCTCCTTCGGCTCCTGCTTTGACCAGTCCAACTTCGAGGGTGACTTCCC**

**TTGAATCTAAAGCTGCTACATTGTCGCTGAAAGCACGATCTACTGCAGAGAAGAGATCTTCTCTGTCTCA**

**AGCCCAAAGCAGGAGTGATTTTTTCAATCTAATGAGGAAGAAGACTTCATTAAATTCTTCTACTGTCCTT**

**TCAGACTCTGGTATGGCTTCATCTGACAGTAGTGAGCAATCTGGTATTAAAACTAAAGATGAGGATAGTG**

**CCTCATTGAGTCCTTGTGTTTCTGAAAATGGAAGTGAGAGAACCAGCAATGGAGACACTCATGAATCTCA**

**GAACCATGTTCAAAGCCATAATGGTATTGAGGAGAACAACTCATCCATAAATGGAGCGCTATATCCAGAT**

**GAGGAAGAGGCTGCTTTCCTACGTTCTCTTGGTTGGGATGAAAATGCTGGGGAAGACGAAGGCCTGACAG**

**AGGAAGAAATTAATGCATTTTATCAGGAGGTAACCAACTCTACTTGCTGGTTTCTATATTATGATAAGAG**

**ATACTTCCAGTGTTAGATTAGTTATGTTAAAAGATAAGCCATGATTAAGTTGTTTTGCCTGCGAGTTTGA**

**TACTAGTGAGGTTTCGTGGAGAATGTTCATGGCATATGTACCTGAACATGACCCACAGAAAGTAAGTTTT**

**GTTCTAAAATTCTTAATAGATGCTATGTGGTTCTGGTGTTGGTCTTGTGGTCTGCCCCTGAGACGCACTT**

**TCTTTATGTAAATTTGGAGCAGAGTTGTACCATTGGCGTTATTTTTTCTTTTTTTTTGGGGGTGGGGGGC**

**TCAAAATTTAAGTCAGAAACTACACTGAAAAATAAAGCTTGTTTTCCCTTCAGACTGTGTTTGACTTAAC**

**TCCCTACTTTTGCAGTATATGAAACTGAAGCCTTCCTTGAAGGTGTACAAAGGTGCCCAGCCAAAGTGTT**

**TGATGCTGCCTGAGATGCATGTTGCTACTAATCGTGGACCTGCTTCTTCCGAGTCGACTTCTTCTGAATC**

**TGATGCTTGAATATAATTTCGCACGTATGGATGCATACAATTGGGTTTGTCAAGAGATACATCTCTTCTT**

**TTTGTGATCTATTTTGTTTTGGAGCATCTCTGATGCATGTTAACAAGTGGAGGGTGGCTTTTGGAATTTT**

**GAAGTTCAGAGAAGAGAAATAAGTTAGTCTGCTGGTGTCCTACTGGCTCAAAAGTAAGCTAAGGTATCAA**

**AGGGTAGCGTTTTATAAGTAGAGTTTAGGAAAATGTTAGTCTGCAGTGGATGATGCTGGGCAAATTGCTT**

**CTTTTTGGAAATTTTTTGTGCTTTTCCTTTCCAGTATCAATTGAAAAGGAAAGAGAAGGTTTTACGTTGT**

**TTTGGTCAATTTTACGGGGAAGAAGGAAAATTGCAAAGTTCATTATAGAATTTTCTTTTCAGTCGTAATC**

**GCAAAGCTCGTGTTGATATAATCCTATCTATATTTTTGTCTTTAACTGTTTGGCTAAGTTTCTTTTGTGA**

**TATTAATATTTTCAATTGCAATAGGCTCACTTCTTAGAGCAGTTTGATGGATGTAGTAGGTGGCAGTGAC**

**GGGTAGGGAGATGCCTTTTGGGAGATTACAGCGTGCATAGTTTCTGTTGAAGTGCTTTGACTGGTGGCTC**

**TGTTGTTGGAGCATGTATTTTCCTTCACAGTGATTGATCGGTTTTCTTAGAGTTGGAAATAATGTAAAAT**

**GCCTTTTGACTGATTTTCTTAAGCAGGTAATGAGGGGTTAATACTGCTTAATGGTGTTGAATCTTTTGAA**

**AGAAAACTACACTATCCAGGTTCTGTTTAAGTATTAAAATTGCAATAAAAACAGAAGGTAAATGGATTCA**

**AGTTGCATTTTAACCTTGAAAGTGCCGGTCTCTTTTCTTTTTCCCATTCAAAATTGCTCTCCTCTTCTCA**

**ATTGAGAATGTCTTTCTGGACAGACGATTGACACCTCAATGGAGTAAAGATATTAGGGGTATGGAACTTT**

**ATTTCTTCTCACTCCGAGGAAGGTTCACTAGTCCTTCTGCTTTGTTATTAGGCAGACCCCTGTAGGCCTA**

**TCGACCAATGACTGATGTGATCCCTACTTGCGTAATAGAAAAATGGATTTCGTTGTGGAGGTAAATGAAT**

**GATGAAATTAAGGATATCACGTAACTTGATTGTGTACAAAGGCAACTTGGTCTCTGGCTATAAAGTTTCC**

**AGTATTTTGCACCGCTTGTGAATTATAAGAGGAGGATCCCCCGCTGCAAGGAAACAACGGATCGACTTTT**

**CTAACTCTCTTTAAAGAGTCTGTTTTCAAATTCACTTGCTTGCAAGTTTTGGGTTAGCAGGTTGTTGACT**

**GGGAGAGCTGGTAAAAGGGAACAACAATCTCCCCCAGCAATACCAAAGATCCATCCAAGGAATGAGCTTC**

**CCTTCCTGTGGACTACAAGGAAAAGATAGAATCTCGTCCGGCAACAGTAGCTGAGTCAATGACAGATGCT**

**CTTGGAAAGAACCTTTCATTCGATGCTTCTGATTCAAGCTTATTCTTTTCTACCTTTTGAATTAATCTAG**

**AAGCTCAATTAGCAGATTCATTAGAAACCTTTCGACCAGTATAATCATTAAATTTCTCGCATATATAACC**

**AGCATAGCTCTTGTATGAGCGAAGTAATGCCTTCATCCTTTCTCTCCATGTATGCTTAGGAACCATACCA**

**ATCCATCCCGAACTTAGTGGTTAAACTCAACAAGTATGGGAAGGGTCCTCCTCTCCCTGGTCAAAGCTCC**

**GTGAACGAGAACTCGTTTTGTTTGTTGGCCGGCTAATGATGGTTTTCTGTCCCAATTCAACCTTACAGTC**

**TTCGGATGACCCAAAAAAGCTAATACCTTTTTTCTCACAAGCTATTAGTACTACTTTTCACTGGAATGCC**

**ACTTTGGAAGTTAAATTGAAAGCAATTATTGTTCATTTTAGAAATTTTCACATTGAGAAAAGTAACATGT**

**CTTGATATTTTATTGCCGTATGTAAACTCAACTCCACTTTAAAATCAATCCTGGTTAAAAGTGAGATAAT**

**AAAAAATGATACAAGATTACCACAACCGGGGGAGAGGGATAGTTACTTTCCTATTTGTGTCTCGGAAAAA**

**ATGATGAGATAATAAAAATGACACACAATGTGGGGGATGGAGAATAATTTTGAAGTCTACATTAAGGGAA**

**AAAAAGAGAGCAAAAAGGATAAGATTTAAGTGTTTTTTGGTAGTTATTTTCAATTACAATAAAGTCAAGT**

**CCTAGTACAACTGTTGTTAGTCATAAATAAATTAATCAAATATTACATTTTCCCAATGTTTTCCTACATA**

**TTTACAATCTTCAACTTATTGTGGAGTAAAGTTTCAGCTAATGGTCGGGTTCAAGTATTTTAGTATTAAC**

**CCGTTTGACATTGATGAAAAAACCAGGTGACAACATTTAATTGAATATTTATTACACCTCAGATTTGTAT**

**ATACATACGCGCTATATTTTTTGCAATTGTTCCAGCTTTTGTGTTAAAGTGGAACATAAGAAATAAAATT**

**GCAGGGCCAAAATGGAACAAACAAGATAAAGTTGGAGGGTCAAAATAGAACAAGATAGAGATAGAGTGTC**

**AAAATGAAAAATGGTGTCAAGTTGAATTGGCTGTATATGTATTTGGTCAAAATAAAAACTAGACTAGAAA**

**CACGAGTAAATCAATAGGAAAATCTGAGTCGCCTCATTGGCTTGTACATTTAGCTGTCTATTGTGTACAT**

**ACTAAGGTGGATTACGCAAGTGAAATACAGGGTATTATAGTCATTCTCGCTCACGTCTATATATATATAT**

**TCAAACCCTAGCTAAATCAATGGTCGGGCGTCGGGTCGATGAGTAAAGGAGAAGAACTTTTCACTGGAGT**

**TGTCCCAATTCTTGTTGAATTAGATGGTGATGTTAATGGGTACAAATTTTCTGTCAGTGGAGAGGGTGAA**

**GGTGATGCAACATACGGAAAACTTACCCTTAAATTTATTTGCACTACTGGAAAACTACCTGTTCCATGGC**

**CAACACTTGTCACTACTTTCTCTTATGGTGTTCAATGCTTTTCAAGATACCCAGATCATATGAAGCGGCA**

**CGACTTCTTCAAGAGCGCCATGCCTGAGGGATACGTGCAGGAGAGGACCATCTTCTTCAAGGACGACGGG**

**AACTACAAGACACGTGCTGAAGTCAAGTTTGAGGGAGACACCCTCGTCAACAGGATCGAGCTTAAGGGAA**

**TCGATTTCAAGGAGGACGGAAACATCCTCGGCCACAAGTTGGAATACAACTACAACTCCCACAACGTATA**

**CATCATGGCCGACAAGCAAAAGAACGGCATCAAAGCCAACTTCAAGACCCGCCACAACATCGAAGACGGC**

**GGCGTGCAACTCGCTGATCATTATCAACAAAATACTCCAATTGGCGATGGCCCTGTCCTTTTACCAGACA**

**ACCATTACCTGTCCACACAATCTGCCCTTTCGAAAGATCCCAACGAAAAGAGAGACCACATGGTCCTTCT**

**TGAGTTTGTAACAGCTGCTGGGATTACACATGGCATGGATGAACTATACAAACATGATGAGCTTGATTAC**

**GCCGAGGCCTCTTCCCTAATGATATCCCGCGGCCATGCTAGAGTCCGCAAAAATCACCAGTCTCTCTCTA**

**CAAATCTATCTCTCTCTATTTTTCTCCAGAATAATGTGTGAGTAGTTCCCAGATAAGGGAATTAGGGTTC**

**TTATAGGGTTTCGCTCATGTGTTGAGCATATAAGAAACCCTTAGTATGTATTTGTATTTGTAAAATACTT**

**CTATCAATAAAATTTCTAATTCCTAAAACCAAAATCCAGTGACCTGCAGGCATGCGACGTCG**

**>nta-MIR6019, 6020proa-3k::GUS (1bp - 13555bp, direct) 13555bp**

**TTAGAATAACGGATATTTAAAAGGGCGTGAAAAGGTTTATCCGTTCGTCCATTTGTATGTGCATGCCAAC**

**CACAGGGTTCCCCTCGGGATCAAAGTACTTTGATCCAACCCCTCCGCTGCTATAGTGCAGTCGGCTTCTG**

**ACGTTCAGTGCAGCCGTCTTCTGAAAACGACATGTCGCACAAGTCCTAAGTTACGCGACAGGCTGCCGCC**

**CTGCCCTTTTCCTGGCGTTTTCTTGTCGCGTGTTTTAGTCGCATAAAGTAGAATACTTGCGACTAGAACC**

**GGAGACATTACGCCATGAACAAGAGCGCCGCCGCTGGCCTGCTGGGCTATGCCCGCGTCAGCACCGACGA**

**CCAGGACTTGACCAACCAACGGGCCGAACTGCACGCGGCCGGCTGCACCAAGCTGTTTTCCGAGAAGATC**

**ACCGGCACCAGGCGCGACCGCCCGGAGCTGGCCAGGATGCTTGACCACCTACGCCCTGGCGACGTTGTGA**

**CAGTGACCAGGCTAGACCGCCTGGCCCGCAGCACCCGCGACCTACTGGACATTGCCGAGCGCATCCAGGA**

**GGCCGGCGCGGGCCTGCGTAGCCTGGCAGAGCCGTGGGCCGACACCACCACGCCGGCCGGCCGCATGGTG**

**TTGACCGTGTTCGCCGGCATTGCCGAGTTCGAGCGTTCCCTAATCATCGACCGCACCCGGAGCGGGCGCG**

**AGGCCGCCAAGGCCCGAGGCGTGAAGTTTGGCCCCCGCCCTACCCTCACCCCGGCACAGATCGCGCACGC**

**CCGCGAGCTGATCGACCAGGAAGGCCGCACCGTGAAAGAGGCGGCTGCACTGCTTGGCGTGCATCGCTCG**

**ACCCTGTACCGCGCACTTGAGCGCAGCGAGGAAGTGACGCCCACCGAGGCCAGGCGGCGCGGTGCCTTCC**

**GTGAGGACGCATTGACCGAGGCCGACGCCCTGGCGGCCGCCGAGAATGAACGCCAAGAGGAACAAGCATG**

**AAACCGCACCAGGACGGCCAGGACGAACCGTTTTTCATTACCGAAGAGATCGAGGCGGAGATGATCGCGG**

**CCGGGTACGTGTTCGAGCCGCCCGCGCACGTCTCAACCGTGCGGCTGCATGAAATCCTGGCCGGTTTGTC**

**TGATGCCAAGCTGGCGGCCTGGCCGGCCAGCTTGGCCGCTGAAGAAACCGAGCGCCGCCGTCTAAAAAGG**

**TGATGTGTATTTGAGTAAAACAGCTTGCGTCATGCGGTCGCTGCGTATATGATGCGATGAGTAAATAAAC**

**AAATACGCAAGGGGAACGCATGAAGGTTATCGCTGTACTTAACCAGAAAGGCGGGTCAGGCAAGACGACC**

**ATCGCAACCCATCTAGCCCGCGCCCTGCAACTCGCCGGGGCCGATGTTCTGTTAGTCGATTCCGATCCCC**

**AGGGCAGTGCCCGCGATTGGGCGGCCGTGCGGGAAGATCAACCGCTAACCGTTGTCGGCATCGACCGCCC**

**GACGATTGACCGCGACGTGAAGGCCATCGGCCGGCGCGACTTCGTAGTGATCGACGGAGCGCCCCAGGCG**

**GCGGACTTGGCTGTGTCCGCGATCAAGGCAGCCGACTTCGTGCTGATTCCGGTGCAGCCAAGCCCTTACG**

**ACATATGGGCCACCGCCGACCTGGTGGAGCTGGTTAAGCAGCGCATTGAGGTCACGGATGGAAGGCTACA**

**AGCGGCCTTTGTCGTGTCGCGGGCGATCAAAGGCACGCGCATCGGCGGTGAGGTTGCCGAGGCGCTGGCC**

**GGGTACGAGCTGCCCATTCTTGAGTCCCGTATCACGCAGCGCGTGAGCTACCCAGGCACTGCCGCCGCCG**

**GCACAACCGTTCTTGAATCAGAACCCGAGGGCGACGCTGCCCGCGAGGTCCAGGCGCTGGCCGCTGAAAT**

**TAAATCAAAACTCATTTGAGTTAATGAGGTAAAGAGAAAATGAGCAAAAGCACAAACACGCTAAGTGCCG**

**GCCGTCCGAGCGCACGCAGCAGCAAGGCTGCAACGTTGGCCAGCCTGGCAGACACGCCAGCCATGAAGCG**

**GGTCAACTTTCAGTTGCCGGCGGAGGATCACACCAAGCTGAAGATGTACGCGGTACGCCAAGGCAAGACC**

**ATTACCGAGCTGCTATCTGAATACATCGCGCAGCTACCAGAGTAAATGAGCAAATGAATAAATGAGTAGA**

**TGAATTTTAGCGGCTAAAGGAGGCGGCATGGAAAATCAAGAACAACCAGGCACCGACGCCGTGGAATGCC**

**CCATGTGTGGAGGAACGGGCGGTTGGCCAGGCGTAAGCGGCTGGGTTGTCTGCCGGCCCTGCAATGGCAC**

**TGGAACCCCCAAGCCCGAGGAATCGGCGTGAGCGGTCGCAAACCATCCGGCCCGGTACAAATCGGCGCGG**

**CGCTGGGTGATGACCTGGTGGAGAAGTTGAAGGCCGCGCAGGCCGCCCAGCGGCAACGCATCGAGGCAGA**

**AGCACGCCCCGGTGAATCGTGGCAAGCGGCCGCTGATCGAATCCGCAAAGAATCCCGGCAACCGCCGGCA**

**GCCGGTGCGCCGTCGATTAGGAAGCCGCCCAAGGGCGACGAGCAACCAGATTTTTTCGTTCCGATGCTCT**

**ATGACGTGGGCACCCGCGATAGTCGCAGCATCATGGACGTGGCCGTTTTCCGTCTGTCGAAGCGTGACCG**

**ACGAGCTGGCGAGGTGATCCGCTACGAGCTTCCAGACGGGCACGTAGAGGTTTCCGCAGGGCCGGCCGGC**

**ATGGCCAGTGTGTGGGATTACGACCTGGTACTGATGGCGGTTTCCCATCTAACCGAATCCATGAACCGAT**

**ACCGGGAAGGGAAGGGAGACAAGCCCGGCCGCGTGTTCCGTCCACACGTTGCGGACGTACTCAAGTTCTG**

**CCGGCGAGCCGATGGCGGAAAGCAGAAAGACGACCTGGTAGAAACCTGCATTCGGTTAAACACCACGCAC**

**GTTGCCATGCAGCGTACGAAGAAGGCCAAGAACGGCCGCCTGGTGACGGTATCCGAGGGTGAAGCCTTGA**

**TTAGCCGCTACAAGATCGTAAAGAGCGAAACCGGGCGGCCGGAGTACATCGAGATCGAGCTAGCTGATTG**

**GATGTACCGCGAGATCACAGAAGGCAAGAACCCGGACGTGCTGACGGTTCACCCCGATTACTTTTTGATC**

**GATCCCGGCATCGGCCGTTTTCTCTACCGCCTGGCACGCCGCGCCGCAGGCAAGGCAGAAGCCAGATGGT**

**TGTTCAAGACGATCTACGAACGCAGTGGCAGCGCCGGAGAGTTCAAGAAGTTCTGTTTCACCGTGCGCAA**

**GCTGATCGGGTCAAATGACCTGCCGGAGTACGATTTGAAGGAGGAGGCGGGGCAGGCTGGCCCGATCCTA**

**GTCATGCGCTACCGCAACCTGATCGAGGGCGAAGCATCCGCCGGTTCCTAATGTACGGAGCAGATGCTAG**

**GGCAAATTGCCCTAGCAGGGGAAAAAGGTCGAAAAGGTCTCTTTCCTGTGGATAGCACGTACATTGGGAA**

**CCCAAAGCCGTACATTGGGAACCGGAACCCGTACATTGGGAACCCAAAGCCGTACATTGGGAACCGGTCA**

**CACATGTAAGTGACTGATATAAAAGAGAAAAAAGGCGATTTTTCCGCCTAAAACTCTTTAAAACTTATTA**

**AAACTCTTAAAACCCGCCTGGCCTGTGCATAACTGTCTGGCCAGCGCACAGCCGAAGAGCTGCAAAAAGC**

**GCCTACCCTTCGGTCGCTGCGCTCCCTACGCCCCGCCGCTTCGCGTCGGCCTATCGCGGCCGCTGGCCGC**

**TCAAAAATGGCTGGCCTACGGCCAGGCAATCTACCAGGGCGCGGACAAGCCGCGCCGTCGCCACTCGACC**

**GCCGGCGCCCACATCAAGGCACCCTGCCTCGCGCGTTTCGGTGATGACGGTGAAAACCTCTGACACATGC**

**AGCTCCCGGAGACGGTCACAGCTTGTCTGTAAGCGGATGCCGGGAGCAGACAAGCCCGTCAGGGCGCGTC**

**AGCGGGTGTTGGCGGGTGTCGGGGCGCAGCCATGACCCAGTCACGTAGCGATAGCGGAGTGTATACTGGC**

**TTAACTATGCGGCATCAGAGCAGATTGTACTGAGAGTGCACCATATGCGGTGTGAAATACCGCACAGATG**

**CGTAAGGAGAAAATACCGCATCAGGCGCTCTTCCGCTTCCTCGCTCACTGACTCGCTGCGCTCGGTCGTT**

**CGGCTGCGGCGAGCGGTATCAGCTCACTCAAAGGCGGTAATACGGTTATCCACAGAATCAGGGGATAACG**

**CAGGAAAGAACATGTGAGCAAAAGGCCAGCAAAAGGCCAGGAACCGTAAAAAGGCCGCGTTGCTGGCGTT**

**TTTCCATAGGCTCCGCCCCCCTGACGAGCATCACAAAAATCGACGCTCAAGTCAGAGGTGGCGAAACCCG**

**ACAGGACTATAAAGATACCAGGCGTTTCCCCCTGGAAGCTCCCTCGTGCGCTCTCCTGTTCCGACCCTGC**

**CGCTTACCGGATACCTGTCCGCCTTTCTCCCTTCGGGAAGCGTGGCGCTTTCTCATAGCTCACGCTGTAG**

**GTATCTCAGTTCGGTGTAGGTCGTTCGCTCCAAGCTGGGCTGTGTGCACGAACCCCCCGTTCAGCCCGAC**

**CGCTGCGCCTTATCCGGTAACTATCGTCTTGAGTCCAACCCGGTAAGACACGACTTATCGCCACTGGCAG**

**CAGCCACTGGTAACAGGATTAGCAGAGCGAGGTATGTAGGCGGTGCTACAGAGTTCTTGAAGTGGTGGCC**

**TAACTACGGCTACACTAGAAGGACAGTATTTGGTATCTGCGCTCTGCTGAAGCCAGTTACCTTCGGAAAA**

**AGAGTTGGTAGCTCTTGATCCGGCAAACAAACCACCGCTGGTAGCGGTGGTTTTTTTGTTTGCAAGCAGC**

**AGATTACGCGCAGAAAAAAAGGATCTCAAGAAGATCCTTTGATCTTTTCTACGGGGTCTGACGCTCAGTG**

**GAACGAAAACTCACGTTAAGGGATTTTGGTCATGCATTCTAGGTACTAAAACAATTCATCCAGTAAAATA**

**TAATATTTTATTTTCTCCCAATCAGGCTTGATCCCCAGTAAGTCAAAAAATAGCTCGACATACTGTTCTT**

**CCCCGATATCCTCCCTGATCGACCGGACGCAGAAGGCAATGTCATACCACTTGTCCGCCCTGCCGCTTCT**

**CCCAAGATCAATAAAGCCACTTACTTTGCCATCTTTCACAAAGATGTTGCTGTCTCCCAGGTCGCCGTGG**

**GAAAAGACAAGTTCCTCTTCGGGCTTTTCCGTCTTTAAAAAATCATACAGCTCGCGCGGATCTTTAAATG**

**GAGTGTCTTCTTCCCAGTTTTCGCAATCCACATCGGCCAGATCGTTATTCAGTAAGTAATCCAATTCGGC**

**TAAGCGGCTGTCTAAGCTATTCGTATAGGGACAATCCGATATGTCGATGGAGTGAAAGAGCCTGATGCAC**

**TCCGCATACAGCTCGATAATCTTTTCAGGGCTTTGTTCATCTTCATACTCTTCCGAGCAAAGGACGCCAT**

**CGGCCTCACTCATGAGCAGATTGCTCCAGCCATCATGCCGTTCAAAGTGCAGGACCTTTGGAACAGGCAG**

**CTTTCCTTCCAGCCATAGCATCATGTCCTTTTCCCGTTCCACATCATAGGTGGTCCCTTTATACCGGCTG**

**TCCGTCATTTTTAAATATAGGTTTTCATTTTCTCCCACCAGCTTATATACCTTAGCAGGAGACATTCCTT**

**CCGTATCTTTTACGCAGCGGTATTTTTCGATCAGTTTTTTCAATTCCGGTGATATTCTCATTTTAGCCAT**

**TTATTATTTCCTTCCTCTTTTCTACAGTATTTAAAGATACCCCAAGAAGCTAATTATAACAAGACGAACT**

**CCAATTCACTGTTCCTTGCATTCTAAAACCTTAAATACCAGAAAACAGCTTTTTCAAAGTTGTTTTCAAA**

**GTTGGCGTATAACATAGTATCGACGGAGCCGATTTTGAAACCGCGGTGATCACAGGCAGCAACGCTCTGT**

**CATCGTTACAATCAACATGCTACCCTCCGCGAGATCATCCGTGTTTCAAACCCGGCAGCTTAGTTGCCGT**

**TCTTCCGAATAGCATCGGTAACATGAGCAAAGTCTGCCGCCTTACAACGGCTCTCCCGCTGACGCCGTCC**

**CGGACTGATGGGCTGCCTGTATCGAGTGGTGATTTTGTGCCGAGCTGCCGGTCGGGGAGCTGTTGGCTGG**

**CTGGTGGCAGGATATATTGTGGTGTAAACAAATTGACGCTTAGACAACTTAATAACACATTGCGGACGTT**

**TTTAATGTACTGAATTAACGCCGAATTAATTCGGGGGATCTGGATTTTAGTACTGGATTTTGGTTTTAGG**

**AATTAGAAATTTTATTGATAGAAGTATTTTACAAATACAAATACATACTAAGGGTTTCTTATATGCTCAA**

**CACATGAGCGAAACCCTATAGGAACCCTAATTCCCTTATCTGGGAACTACTCACACATTATTATGGAGAA**

**ACTCGAGCTTGTCGATCGACAGATCCGGTCGGCATCTACTCTATTTCTTTGCCCTCGGACGAGTGCTGGG**

**GCGTCGGTTTCCACTATCGGCGAGTACTTCTACACAGCCATCGGTCCAGACGGCCGCGCTTCTGCGGGCG**

**ATTTGTGTACGCCCGACAGTCCCGGCTCCGGATCGGACGATTGCGTCGCATCGACCCTGCGCCCAAGCTG**

**CATCATCGAAATTGCCGTCAACCAAGCTCTGATAGAGTTGGTCAAGACCAATGCGGAGCATATACGCCCG**

**GAGTCGTGGCGATCCTGCAAGCTCCGGATGCCTCCGCTCGAAGTAGCGCGTCTGCTGCTCCATACAAGCC**

**AACCACGGCCTCCAGAAGAAGATGTTGGCGACCTCGTATTGGGAATCCCCGAACATCGCCTCGCTCCAGT**

**CAATGACCGCTGTTATGCGGCCATTGTCCGTCAGGACATTGTTGGAGCCGAAATCCGCGTGCACGAGGTG**

**CCGGACTTCGGGGCAGTCCTCGGCCCAAAGCATCAGCTCATCGAGAGCCTGCGCGACGGACGCACTGACG**

**GTGTCGTCCATCACAGTTTGCCAGTGATACACATGGGGATCAGCAATCGCGCATATGAAATCACGCCATG**

**TAGTGTATTGACCGATTCCTTGCGGTCCGAATGGGCCGAACCCGCTCGTCTGGCTAAGATCGGCCGCAGC**

**GATCGCATCCATAGCCTCCGCGACCGGTTGTAGAACAGCGGGCAGTTCGGTTTCAGGCAGGTCTTGCAAC**

**GTGACACCCTGTGCACGGCGGGAGATGCAATAGGTCAGGCTCTCGCTAAACTCCCCAATGTCAAGCACTT**

**CCGGAATCGGGAGCGCGGCCGATGCAAAGTGCCGATAAACATAACGATCTTTGTAGAAACCATCGGCGCA**

**GCTATTTACCCGCAGGACATATCCACGCCCTCCTACATCGAAGCTGAAAGCACGAGATTCTTCGCCCTCC**

**GAGAGCTGCATCAGGTCGGAGACGCTGTCGAACTTTTCGATCAGAAACTTCTCGACAGACGTCGCGGTGA**

**GTTCAGGCTTTTTCATATCTCATTGCCCCCCGGGATCTGCGAAAGCTCGAGAGAGATAGATTTGTAGAGA**

**GAGACTGGTGATTTCAGCGTGTCCTCTCCAAATGAAATGAACTTCCTTATATAGAGGAAGGTCTTGCGAA**

**GGATAGTGGGATTGTGCGTCATCCCTTACGTCAGTGGAGATATCACATCAATCCACTTGCTTTGAAGACG**

**TGGTTGGAACGTCTTCTTTTTCCACGATGCTCCTCGTGGGTGGGGGTCCATCTTTGGGACCACTGTCGGC**

**AGAGGCATCTTGAACGATAGCCTTTCCTTTATCGCAATGATGGCATTTGTAGGTGCCACCTTCCTTTTCT**

**ACTGTCCTTTTGATGAAGTGACAGATAGCTGGGCAATGGAATCCGAGGAGGTTTCCCGATATTACCCTTT**

**GTTGAAAAGTCTCAATAGCCCTTTGGTCTTCTGAGACTGTATCTTTGATATTCTTGGAGTAGACGAGAGT**

**GTCGTGCTCCACCATGTTATCACATCAATCCACTTGCTTTGAAGACGTGGTTGGAACGTCTTCTTTTTCC**

**ACGATGCTCCTCGTGGGTGGGGGTCCATCTTTGGGACCACTGTCGGCAGAGGCATCTTGAACGATAGCCT**

**TTCCTTTATCGCAATGATGGCATTTGTAGGTGCCACCTTCCTTTTCTACTGTCCTTTTGATGAAGTGACA**

**GATAGCTGGGCAATGGAATCCGAGGAGGTTTCCCGATATTACCCTTTGTTGAAAAGTCTCAATAGCCCTT**

**TGGTCTTCTGAGACTGTATCTTTGATATTCTTGGAGTAGACGAGAGTGTCGTGCTCCACCATGTTGGGCC**

**CGGCGCGCCGAATTCCCGGGGATCCGTCGACTAACCAACTCTACTTGCTGTTTTCTATGTTATGATAAGA**

**GATACTTCCAATGTTAGATTAGTTATGTTAAAAGATAAGCCATGATTAAGTTGTTTTGCCTGCGAGTTTG**

**ATACTAGTGAGGATTCGTGGAGAATGTTCATGGCATATGTACCTGAACATGACCCAAAGTAGGAGATAGC**

**CGGGTATTTCCACAGAGAAGTCTAGCACTGGCTTGCCCAAAGCGTTGAACATAGAGAATTAAGGTTGTCT**

**TACATACACACTGGTGGAATTTCATTTTCTTCCGCTTTTTAGAAGGCGTAGCATTGGAACTGCTATGTAG**

**GCTTTTGTTTACCGAGGGTTCGAATCCCTCTCTTTCCGTACCTTCGCTTAATTCACCAATTTTACTAACA**

**ACAAGGGCTCAAATATTGTTTTCCCTTCAGACTGTGTTTGACTGAACTCCCTACTTTTGCAGTATATGAA**

**ACTGAAGCCTTCCTTGAAGGTGTACAAAGGTGCCCTGCCAAAGTGTTTGATGCTGCCTGAGATGCATGTT**

**GCTACTAATCGTGGACCTGCTTCTTCCGAGTCGACTTCTTCTGAATCTGATGCTTGAATATAGTGATTTC**

**GCATGTATGGATGCACACAATTGGGTTTATCAAGAGATACATCTCTTCTTTTTGTGATCTATTTAGTTTT**

**GGAGCATCTCTGATGCATGTTGACAAGTGGAGGGTGGCTTTTGGAATTTTGAAGTTCAGAGAAGAGAAAT**

**AAGTTAGTCTGCTGCTGTCCTACTGGCTCAAAAGTAAGCTAAGGTATCAAAGGTAGCGTTTTATAAGTAG**

**AGTTTAGGAAAATGTTAGTCTGCAGTGGATAATGCTGGGCAAATTGCTTCTTTTTGGAAATTTTTTTGTG**

**CTTTTCCTTTCCAGTATCAATTGAAAAGGAAAGAGAAGGTTTTACGTTGTTTTGGTCAATTTTACGGGGA**

**AGAAGGAAAATTGCAAAGTTCATTATAGAATTTTCTTTTCAGTCGTAATCGCAAAAGCTCGTGTTGATAT**

**AATCCTATCTATATTTTTGTCTTTAACTGTTTGGCTAAGGTTCTTTATGATATTAATATTTTCAATTGCA**

**ATCGGCTCACTTCTTAGAGCAGTTTGATGGATGTAGTAGGTGGCAGTGACGGGTAGGGAGGATTACAGTG**

**TGCATAGTTTCTGTTGAAGTGCTTTGACTGGTGGCTCTGTTGTTGGAGCATGTATTTTCCTTCAGTGATT**

**GATCGGCTTTCTTAGAGTTGGAAGTAATGTAAATTGCCTTTGACTGGTTTTCTTAAGCAGGTAATGAGGG**

**GTTAATATTGCTTAATGGTGTTGAATCTTTTGAAAGAAAACTACACTATCCAGGTTCTGTTTAAGTATTA**

**AAATTGCAATAAAAAACAGTAGGTAAATGGATTCAAGTTGCATTTTAACCTTTTAAGTGCCTGCCTCTAT**

**TTTTTTTTCCCATTCAAAATTGCTCTCGTCTCAATGTCCTAAGACATTTTGAAAGAATTGAGAATGTCTT**

**TCTGGACAGACGATTGACACCTCAATGGAGTAAAGATATTAGGGGTATGGAACTTTAATTCTAGTCCTTC**

**TGCTTTGTTCTTAGGCAGACCCCCCTGTAGGCCTATCGACCAATGACTCGTGATCCCAACTTGCGTAATG**

**GAAAAATTGGAGTAATAGAACAATTGATTTCGTTGTGGTGGTAAAAGATATCACGTATCTTGATTGTGTA**

**CAAAGGCAACTTGGTCTCTGGCTATAAAGTTTCCAGTATTTTGCACCGCTTGTGAATTATAAGACGATCT**

**TATACCAGAAAATGAAACATTGGACATAACTTTGGAACCCCCCCCCCACTGGCTGCAAGGAAATAACGGA**

**TTGGATTTTCTAACTCTCTTTAAAGAGACTGGTTTCGAATTCACTTGCTTGCAAGCTTAGGGTTAGCAGG**

**TTGTTGACTGGGAGAGCTGGTAAAAGAATAAAATCTTCAAGAGATCATGGTTAACAAGCCAGCTGAGGAA**

**TAACAATCTTCCTCAGCAATACCAAAAATCCATCCAAGGAATGAGCTTCCCCAAAGTTAATTCGCGTGGA**

**CTACAAAGAAAAGATAGAGTCTCACCCGGTAACATCAGCTGAGTCAATGGCAGAAAGTCTTGAAAAGAAC**

**CTTTTATTCGATGCTTCTGATTCAAGCTTATTCCTTTCCGCCTTTTGAATAAATCTAGAACCTCAATTAG**

**CAGATTCATTAGAAACCTTTCGACCAACATAATCTTTGAATTTTCCGCATATATAACCAGCAGAGCTCTC**

**GTATGAGCAAAGTAACGCCTTCGTCCTTTTTCTCCATGTATGCTTAGGAATCACACCAATCTATCCCGAA**

**TTTGGTGGTTAAACTCTACTGGTATGAGAAGGGTCCTCCTCTCCCTTGTCAAATAAAAATGACACACTAT**

**GTGGGCGGATGGAGAATAACTTTAAGGTCTACATTAAAGAAAAAAAAAGAGCAAAAAGGATAAGATTTAA**

**GTGATTTTTGGTAGTTATTTTCAATTACAATAAAGTCAAATCGTCGTACAACTGTTGTTAGTCATAAAGA**

**AATTAATCAAATATTACCTTTTTCAAATGTTTTCCTACATATTTACAATCTTCAACTTTGTGTAGAGTAT**

**AAGTTTCAGCTAATTTCTTATTTTATAAATTTACTTTTAGATATTTAGTCTCTTAAAGGCTGACAATTTT**

**CCCTGAACGAAACTTTCTCGACAAGACGACCACCATTATTAAAAATAAAAACTAGACTAGAAACACGATT**

**AAATCAATAGGAAAATCTGAGTAGCCTCATTGGCTTGTACTTTTAGCTGTCTATTGCGTACATACTAAGG**

**TGGATTTTAGGCAAGGGAATTACAGGGTATTATAGTCATTCTCGCTCACGTCTATATATATATTCAAACC**

**CTAGCAAAATCAATGGTATGGCAGATCTGACTAGTTTACGTCCTGTAGAAACCCCAACCCGTGAAATCAA**

**AAAACTCGACGGCCTGTGGGCATTCAGTCTGGATCGCGAAAACTGTGGAATTGATCAGCGTTGGTGGGAA**

**AGCGCGTTACAAGAAAGCCGGGCAATTGCTGTGCCAGGCAGTTTTAACGATCAGTTCGCCGATGCAGATA**

**TTCGTAATTATGCGGGCAACGTCTGGTATCAGCGCGAAGTCTTTATACCGAAAGGTTGGGCAGGCCAGCG**

**TATCGTGCTGCGTTTCGATGCGGTCACTCATTACGGCAAAGTGTGGGTCAATAATCAGGAAGTGATGGAG**

**CATCAGGGCGGCTATACGCCATTTGAAGCCGATGTCACGCCGTATGTTATTGCCGGGAAAAGTGTACGTA**

**TCACCGTTTGTGTGAACAACGAACTGAACTGGCAGACTATCCCGCCGGGAATGGTGATTACCGACGAAAA**

**CGGCAAGAAAAAGCAGTCTTACTTCCATGATTTCTTTAACTATGCCGGAATCCATCGCAGCGTAATGCTC**

**TACACCACGCCGAACACCTGGGTGGACGATATCACCGTGGTGACGCATGTCGCGCAAGACTGTAACCACG**

**CGTCTGTTGACTGGCAGGTGGTGGCCAATGGTGATGTCAGCGTTGAACTGCGTGATGCGGATCAACAGGT**

**GGTTGCAACTGGACAAGGCACTAGCGGGACTTTGCAAGTGGTGAATCCGCACCTCTGGCAACCGGGTGAA**

**GGTTATCTCTATGAACTGTGCGTCACAGCCAAAAGCCAGACAGAGTGTGATATCTACCCGCTTCGCGTCG**

**GCATCCGGTCAGTGGCAGTGAAGGGCCAACAGTTCCTGATTAACCACAAACCGTTCTACTTTACTGGCTT**

**TGGTCGTCATGAAGATGCGGACTTACGTGGCAAAGGATTCGATAACGTGCTGATGGTGCACGACCACGCA**

**TTAATGGACTGGATTGGGGCCAACTCCTACCGTACCTCGCATTACCCTTACGCTGAAGAGATGCTCGACT**

**GGGCAGATGAACATGGCATCGTGGTGATTGATGAAACTGCTGCTGTCGGCTTTCAGCTGTCTTTAGGCAT**

**TGGTTTCGAAGCGGGCAACAAGCCGAAAGAACTGTACAGCGAAGAGGCAGTCAACGGGGAAACTCAGCAA**

**GCGCACTTACAGGCGATTAAAGAGCTGATAGCGCGTGACAAAAACCACCCAAGCGTGGTGATGTGGAGTA**

**TTGCCAACGAACCGGATACCCGTCCGCAAGGTGCACGGGAATATTTCGCGCCACTGGCGGAAGCAACGCG**

**TAAACTCGACCCGACGCGTCCGATCACCTGCGTCAATGTAATGTTCTGCGACGCTCACACCGATACCATC**

**AGCGATCTCTTTGATGTGCTGTGCCTGAACCGTTATTACGGATGGTATGTCCAAAGCGGCGATTTGGAAA**

**CGGCAGAGAAGGTACTGGAAAAAGAACTTCTGGCCTGGCAGGAGAAACTGCATCAGCCGATTATCATCAC**

**CGAATACGGCGTGGATACGTTAGCCGGGCTGCACTCAATGTACACCGACATGTGGAGTGAAGAGTATCAG**

**TGTGCATGGCTGGATATGTATCACCGCGTCTTTGATCGCGTCAGCGCCGTCGTCGGTGAACAGGTATGGA**

**ATTTCGCCGATTTTGCGACCTCGCAAGGCATATTGCGCGTTGGCGGTAACAAGAAAGGGATCTTCACTCG**

**CGACCGCAAACCGAAGTCGGCGGCTTTTCTGCTGCAAAAACGCTGGACTGGCATGAACTTCGGTGAAAAA**

**CCGCAGCAGGGAGGCAAACAAGCTAGCCACCACCACCACCACCACGTGTGAATTGGTGACCAGCTCGAAT**

**TTCCCCGATCGTTCAAACATTTGGCAATAAAGTTTCTTAAGATTGAATCCTGTTGCCGGTCTTGCGATGA**

**TTATCATATAATTTCTGTTGAATTACGTTAAGCATGTAATAATTAACATGTAATGCATGACGTTATTTAT**

**GAGATGGGTTTTTATGATTAGAGTCCCGCAATTATACATTTAATACGCGATAGAAAACAAAATATAGCGC**

**GCAAACTAGGATAAATTATCGCGCGCGGTGTCATCTATGTTACTAGATCGGGAATTAAACTATCAGTGTT**

**TGACAGGATATATTGGCGGGTAAACCTAAGAGAAAAGAGCGTTTA**

**>nta-MIR6019, 6020prob-2k::GUS (1bp - 13530bp, direct) 13530bp**

**TTAGAATAACGGATATTTAAAAGGGCGTGAAAAGGTTTATCCGTTCGTCCATTTGTATGTGCATGCCAAC**

**CACAGGGTTCCCCTCGGGATCAAAGTACTTTGATCCAACCCCTCCGCTGCTATAGTGCAGTCGGCTTCTG**

**ACGTTCAGTGCAGCCGTCTTCTGAAAACGACATGTCGCACAAGTCCTAAGTTACGCGACAGGCTGCCGCC**

**CTGCCCTTTTCCTGGCGTTTTCTTGTCGCGTGTTTTAGTCGCATAAAGTAGAATACTTGCGACTAGAACC**

**GGAGACATTACGCCATGAACAAGAGCGCCGCCGCTGGCCTGCTGGGCTATGCCCGCGTCAGCACCGACGA**

**CCAGGACTTGACCAACCAACGGGCCGAACTGCACGCGGCCGGCTGCACCAAGCTGTTTTCCGAGAAGATC**

**ACCGGCACCAGGCGCGACCGCCCGGAGCTGGCCAGGATGCTTGACCACCTACGCCCTGGCGACGTTGTGA**

**CAGTGACCAGGCTAGACCGCCTGGCCCGCAGCACCCGCGACCTACTGGACATTGCCGAGCGCATCCAGGA**

**GGCCGGCGCGGGCCTGCGTAGCCTGGCAGAGCCGTGGGCCGACACCACCACGCCGGCCGGCCGCATGGTG**

**TTGACCGTGTTCGCCGGCATTGCCGAGTTCGAGCGTTCCCTAATCATCGACCGCACCCGGAGCGGGCGCG**

**AGGCCGCCAAGGCCCGAGGCGTGAAGTTTGGCCCCCGCCCTACCCTCACCCCGGCACAGATCGCGCACGC**

**CCGCGAGCTGATCGACCAGGAAGGCCGCACCGTGAAAGAGGCGGCTGCACTGCTTGGCGTGCATCGCTCG**

**ACCCTGTACCGCGCACTTGAGCGCAGCGAGGAAGTGACGCCCACCGAGGCCAGGCGGCGCGGTGCCTTCC**

**GTGAGGACGCATTGACCGAGGCCGACGCCCTGGCGGCCGCCGAGAATGAACGCCAAGAGGAACAAGCATG**

**AAACCGCACCAGGACGGCCAGGACGAACCGTTTTTCATTACCGAAGAGATCGAGGCGGAGATGATCGCGG**

**CCGGGTACGTGTTCGAGCCGCCCGCGCACGTCTCAACCGTGCGGCTGCATGAAATCCTGGCCGGTTTGTC**

**TGATGCCAAGCTGGCGGCCTGGCCGGCCAGCTTGGCCGCTGAAGAAACCGAGCGCCGCCGTCTAAAAAGG**

**TGATGTGTATTTGAGTAAAACAGCTTGCGTCATGCGGTCGCTGCGTATATGATGCGATGAGTAAATAAAC**

**AAATACGCAAGGGGAACGCATGAAGGTTATCGCTGTACTTAACCAGAAAGGCGGGTCAGGCAAGACGACC**

**ATCGCAACCCATCTAGCCCGCGCCCTGCAACTCGCCGGGGCCGATGTTCTGTTAGTCGATTCCGATCCCC**

**AGGGCAGTGCCCGCGATTGGGCGGCCGTGCGGGAAGATCAACCGCTAACCGTTGTCGGCATCGACCGCCC**

**GACGATTGACCGCGACGTGAAGGCCATCGGCCGGCGCGACTTCGTAGTGATCGACGGAGCGCCCCAGGCG**

**GCGGACTTGGCTGTGTCCGCGATCAAGGCAGCCGACTTCGTGCTGATTCCGGTGCAGCCAAGCCCTTACG**

**ACATATGGGCCACCGCCGACCTGGTGGAGCTGGTTAAGCAGCGCATTGAGGTCACGGATGGAAGGCTACA**

**AGCGGCCTTTGTCGTGTCGCGGGCGATCAAAGGCACGCGCATCGGCGGTGAGGTTGCCGAGGCGCTGGCC**

**GGGTACGAGCTGCCCATTCTTGAGTCCCGTATCACGCAGCGCGTGAGCTACCCAGGCACTGCCGCCGCCG**

**GCACAACCGTTCTTGAATCAGAACCCGAGGGCGACGCTGCCCGCGAGGTCCAGGCGCTGGCCGCTGAAAT**

**TAAATCAAAACTCATTTGAGTTAATGAGGTAAAGAGAAAATGAGCAAAAGCACAAACACGCTAAGTGCCG**

**GCCGTCCGAGCGCACGCAGCAGCAAGGCTGCAACGTTGGCCAGCCTGGCAGACACGCCAGCCATGAAGCG**

**GGTCAACTTTCAGTTGCCGGCGGAGGATCACACCAAGCTGAAGATGTACGCGGTACGCCAAGGCAAGACC**

**ATTACCGAGCTGCTATCTGAATACATCGCGCAGCTACCAGAGTAAATGAGCAAATGAATAAATGAGTAGA**

**TGAATTTTAGCGGCTAAAGGAGGCGGCATGGAAAATCAAGAACAACCAGGCACCGACGCCGTGGAATGCC**

**CCATGTGTGGAGGAACGGGCGGTTGGCCAGGCGTAAGCGGCTGGGTTGTCTGCCGGCCCTGCAATGGCAC**

**TGGAACCCCCAAGCCCGAGGAATCGGCGTGAGCGGTCGCAAACCATCCGGCCCGGTACAAATCGGCGCGG**

**CGCTGGGTGATGACCTGGTGGAGAAGTTGAAGGCCGCGCAGGCCGCCCAGCGGCAACGCATCGAGGCAGA**

**AGCACGCCCCGGTGAATCGTGGCAAGCGGCCGCTGATCGAATCCGCAAAGAATCCCGGCAACCGCCGGCA**

**GCCGGTGCGCCGTCGATTAGGAAGCCGCCCAAGGGCGACGAGCAACCAGATTTTTTCGTTCCGATGCTCT**

**ATGACGTGGGCACCCGCGATAGTCGCAGCATCATGGACGTGGCCGTTTTCCGTCTGTCGAAGCGTGACCG**

**ACGAGCTGGCGAGGTGATCCGCTACGAGCTTCCAGACGGGCACGTAGAGGTTTCCGCAGGGCCGGCCGGC**

**ATGGCCAGTGTGTGGGATTACGACCTGGTACTGATGGCGGTTTCCCATCTAACCGAATCCATGAACCGAT**

**ACCGGGAAGGGAAGGGAGACAAGCCCGGCCGCGTGTTCCGTCCACACGTTGCGGACGTACTCAAGTTCTG**

**CCGGCGAGCCGATGGCGGAAAGCAGAAAGACGACCTGGTAGAAACCTGCATTCGGTTAAACACCACGCAC**

**GTTGCCATGCAGCGTACGAAGAAGGCCAAGAACGGCCGCCTGGTGACGGTATCCGAGGGTGAAGCCTTGA**

**TTAGCCGCTACAAGATCGTAAAGAGCGAAACCGGGCGGCCGGAGTACATCGAGATCGAGCTAGCTGATTG**

**GATGTACCGCGAGATCACAGAAGGCAAGAACCCGGACGTGCTGACGGTTCACCCCGATTACTTTTTGATC**

**GATCCCGGCATCGGCCGTTTTCTCTACCGCCTGGCACGCCGCGCCGCAGGCAAGGCAGAAGCCAGATGGT**

**TGTTCAAGACGATCTACGAACGCAGTGGCAGCGCCGGAGAGTTCAAGAAGTTCTGTTTCACCGTGCGCAA**

**GCTGATCGGGTCAAATGACCTGCCGGAGTACGATTTGAAGGAGGAGGCGGGGCAGGCTGGCCCGATCCTA**

**GTCATGCGCTACCGCAACCTGATCGAGGGCGAAGCATCCGCCGGTTCCTAATGTACGGAGCAGATGCTAG**

**GGCAAATTGCCCTAGCAGGGGAAAAAGGTCGAAAAGGTCTCTTTCCTGTGGATAGCACGTACATTGGGAA**

**CCCAAAGCCGTACATTGGGAACCGGAACCCGTACATTGGGAACCCAAAGCCGTACATTGGGAACCGGTCA**

**CACATGTAAGTGACTGATATAAAAGAGAAAAAAGGCGATTTTTCCGCCTAAAACTCTTTAAAACTTATTA**

**AAACTCTTAAAACCCGCCTGGCCTGTGCATAACTGTCTGGCCAGCGCACAGCCGAAGAGCTGCAAAAAGC**

**GCCTACCCTTCGGTCGCTGCGCTCCCTACGCCCCGCCGCTTCGCGTCGGCCTATCGCGGCCGCTGGCCGC**

**TCAAAAATGGCTGGCCTACGGCCAGGCAATCTACCAGGGCGCGGACAAGCCGCGCCGTCGCCACTCGACC**

**GCCGGCGCCCACATCAAGGCACCCTGCCTCGCGCGTTTCGGTGATGACGGTGAAAACCTCTGACACATGC**

**AGCTCCCGGAGACGGTCACAGCTTGTCTGTAAGCGGATGCCGGGAGCAGACAAGCCCGTCAGGGCGCGTC**

**AGCGGGTGTTGGCGGGTGTCGGGGCGCAGCCATGACCCAGTCACGTAGCGATAGCGGAGTGTATACTGGC**

**TTAACTATGCGGCATCAGAGCAGATTGTACTGAGAGTGCACCATATGCGGTGTGAAATACCGCACAGATG**

**CGTAAGGAGAAAATACCGCATCAGGCGCTCTTCCGCTTCCTCGCTCACTGACTCGCTGCGCTCGGTCGTT**

**CGGCTGCGGCGAGCGGTATCAGCTCACTCAAAGGCGGTAATACGGTTATCCACAGAATCAGGGGATAACG**

**CAGGAAAGAACATGTGAGCAAAAGGCCAGCAAAAGGCCAGGAACCGTAAAAAGGCCGCGTTGCTGGCGTT**

**TTTCCATAGGCTCCGCCCCCCTGACGAGCATCACAAAAATCGACGCTCAAGTCAGAGGTGGCGAAACCCG**

**ACAGGACTATAAAGATACCAGGCGTTTCCCCCTGGAAGCTCCCTCGTGCGCTCTCCTGTTCCGACCCTGC**

**CGCTTACCGGATACCTGTCCGCCTTTCTCCCTTCGGGAAGCGTGGCGCTTTCTCATAGCTCACGCTGTAG**

**GTATCTCAGTTCGGTGTAGGTCGTTCGCTCCAAGCTGGGCTGTGTGCACGAACCCCCCGTTCAGCCCGAC**

**CGCTGCGCCTTATCCGGTAACTATCGTCTTGAGTCCAACCCGGTAAGACACGACTTATCGCCACTGGCAG**

**CAGCCACTGGTAACAGGATTAGCAGAGCGAGGTATGTAGGCGGTGCTACAGAGTTCTTGAAGTGGTGGCC**

**TAACTACGGCTACACTAGAAGGACAGTATTTGGTATCTGCGCTCTGCTGAAGCCAGTTACCTTCGGAAAA**

**AGAGTTGGTAGCTCTTGATCCGGCAAACAAACCACCGCTGGTAGCGGTGGTTTTTTTGTTTGCAAGCAGC**

**AGATTACGCGCAGAAAAAAAGGATCTCAAGAAGATCCTTTGATCTTTTCTACGGGGTCTGACGCTCAGTG**

**GAACGAAAACTCACGTTAAGGGATTTTGGTCATGCATTCTAGGTACTAAAACAATTCATCCAGTAAAATA**

**TAATATTTTATTTTCTCCCAATCAGGCTTGATCCCCAGTAAGTCAAAAAATAGCTCGACATACTGTTCTT**

**CCCCGATATCCTCCCTGATCGACCGGACGCAGAAGGCAATGTCATACCACTTGTCCGCCCTGCCGCTTCT**

**CCCAAGATCAATAAAGCCACTTACTTTGCCATCTTTCACAAAGATGTTGCTGTCTCCCAGGTCGCCGTGG**

**GAAAAGACAAGTTCCTCTTCGGGCTTTTCCGTCTTTAAAAAATCATACAGCTCGCGCGGATCTTTAAATG**

**GAGTGTCTTCTTCCCAGTTTTCGCAATCCACATCGGCCAGATCGTTATTCAGTAAGTAATCCAATTCGGC**

**TAAGCGGCTGTCTAAGCTATTCGTATAGGGACAATCCGATATGTCGATGGAGTGAAAGAGCCTGATGCAC**

**TCCGCATACAGCTCGATAATCTTTTCAGGGCTTTGTTCATCTTCATACTCTTCCGAGCAAAGGACGCCAT**

**CGGCCTCACTCATGAGCAGATTGCTCCAGCCATCATGCCGTTCAAAGTGCAGGACCTTTGGAACAGGCAG**

**CTTTCCTTCCAGCCATAGCATCATGTCCTTTTCCCGTTCCACATCATAGGTGGTCCCTTTATACCGGCTG**

**TCCGTCATTTTTAAATATAGGTTTTCATTTTCTCCCACCAGCTTATATACCTTAGCAGGAGACATTCCTT**

**CCGTATCTTTTACGCAGCGGTATTTTTCGATCAGTTTTTTCAATTCCGGTGATATTCTCATTTTAGCCAT**

**TTATTATTTCCTTCCTCTTTTCTACAGTATTTAAAGATACCCCAAGAAGCTAATTATAACAAGACGAACT**

**CCAATTCACTGTTCCTTGCATTCTAAAACCTTAAATACCAGAAAACAGCTTTTTCAAAGTTGTTTTCAAA**

**GTTGGCGTATAACATAGTATCGACGGAGCCGATTTTGAAACCGCGGTGATCACAGGCAGCAACGCTCTGT**

**CATCGTTACAATCAACATGCTACCCTCCGCGAGATCATCCGTGTTTCAAACCCGGCAGCTTAGTTGCCGT**

**TCTTCCGAATAGCATCGGTAACATGAGCAAAGTCTGCCGCCTTACAACGGCTCTCCCGCTGACGCCGTCC**

**CGGACTGATGGGCTGCCTGTATCGAGTGGTGATTTTGTGCCGAGCTGCCGGTCGGGGAGCTGTTGGCTGG**

**CTGGTGGCAGGATATATTGTGGTGTAAACAAATTGACGCTTAGACAACTTAATAACACATTGCGGACGTT**

**TTTAATGTACTGAATTAACGCCGAATTAATTCGGGGGATCTGGATTTTAGTACTGGATTTTGGTTTTAGG**

**AATTAGAAATTTTATTGATAGAAGTATTTTACAAATACAAATACATACTAAGGGTTTCTTATATGCTCAA**

**CACATGAGCGAAACCCTATAGGAACCCTAATTCCCTTATCTGGGAACTACTCACACATTATTATGGAGAA**

**ACTCGAGCTTGTCGATCGACAGATCCGGTCGGCATCTACTCTATTTCTTTGCCCTCGGACGAGTGCTGGG**

**GCGTCGGTTTCCACTATCGGCGAGTACTTCTACACAGCCATCGGTCCAGACGGCCGCGCTTCTGCGGGCG**

**ATTTGTGTACGCCCGACAGTCCCGGCTCCGGATCGGACGATTGCGTCGCATCGACCCTGCGCCCAAGCTG**

**CATCATCGAAATTGCCGTCAACCAAGCTCTGATAGAGTTGGTCAAGACCAATGCGGAGCATATACGCCCG**

**GAGTCGTGGCGATCCTGCAAGCTCCGGATGCCTCCGCTCGAAGTAGCGCGTCTGCTGCTCCATACAAGCC**

**AACCACGGCCTCCAGAAGAAGATGTTGGCGACCTCGTATTGGGAATCCCCGAACATCGCCTCGCTCCAGT**

**CAATGACCGCTGTTATGCGGCCATTGTCCGTCAGGACATTGTTGGAGCCGAAATCCGCGTGCACGAGGTG**

**CCGGACTTCGGGGCAGTCCTCGGCCCAAAGCATCAGCTCATCGAGAGCCTGCGCGACGGACGCACTGACG**

**GTGTCGTCCATCACAGTTTGCCAGTGATACACATGGGGATCAGCAATCGCGCATATGAAATCACGCCATG**

**TAGTGTATTGACCGATTCCTTGCGGTCCGAATGGGCCGAACCCGCTCGTCTGGCTAAGATCGGCCGCAGC**

**GATCGCATCCATAGCCTCCGCGACCGGTTGTAGAACAGCGGGCAGTTCGGTTTCAGGCAGGTCTTGCAAC**

**GTGACACCCTGTGCACGGCGGGAGATGCAATAGGTCAGGCTCTCGCTAAACTCCCCAATGTCAAGCACTT**

**CCGGAATCGGGAGCGCGGCCGATGCAAAGTGCCGATAAACATAACGATCTTTGTAGAAACCATCGGCGCA**

**GCTATTTACCCGCAGGACATATCCACGCCCTCCTACATCGAAGCTGAAAGCACGAGATTCTTCGCCCTCC**

**GAGAGCTGCATCAGGTCGGAGACGCTGTCGAACTTTTCGATCAGAAACTTCTCGACAGACGTCGCGGTGA**

**GTTCAGGCTTTTTCATATCTCATTGCCCCCCGGGATCTGCGAAAGCTCGAGAGAGATAGATTTGTAGAGA**

**GAGACTGGTGATTTCAGCGTGTCCTCTCCAAATGAAATGAACTTCCTTATATAGAGGAAGGTCTTGCGAA**

**GGATAGTGGGATTGTGCGTCATCCCTTACGTCAGTGGAGATATCACATCAATCCACTTGCTTTGAAGACG**

**TGGTTGGAACGTCTTCTTTTTCCACGATGCTCCTCGTGGGTGGGGGTCCATCTTTGGGACCACTGTCGGC**

**AGAGGCATCTTGAACGATAGCCTTTCCTTTATCGCAATGATGGCATTTGTAGGTGCCACCTTCCTTTTCT**

**ACTGTCCTTTTGATGAAGTGACAGATAGCTGGGCAATGGAATCCGAGGAGGTTTCCCGATATTACCCTTT**

**GTTGAAAAGTCTCAATAGCCCTTTGGTCTTCTGAGACTGTATCTTTGATATTCTTGGAGTAGACGAGAGT**

**GTCGTGCTCCACCATGTTATCACATCAATCCACTTGCTTTGAAGACGTGGTTGGAACGTCTTCTTTTTCC**

**ACGATGCTCCTCGTGGGTGGGGGTCCATCTTTGGGACCACTGTCGGCAGAGGCATCTTGAACGATAGCCT**

**TTCCTTTATCGCAATGATGGCATTTGTAGGTGCCACCTTCCTTTTCTACTGTCCTTTTGATGAAGTGACA**

**GATAGCTGGGCAATGGAATCCGAGGAGGTTTCCCGATATTACCCTTTGTTGAAAAGTCTCAATAGCCCTT**

**TGGTCTTCTGAGACTGTATCTTTGATATTCTTGGAGTAGACGAGAGTGTCGTGCTCCACCATGTTGGGCC**

**CGGCGCGCCGAATTCCCGGGGATCCGTCGACTGTTGAATCTTTTGAAAGAAAACTACACTATCCAGGTTC**

**TGTTTAAGTATTAAAATTGCAATAAAAACAGAAGGTAAATGGATTCAAGTTGCATTTTAACCTTGAAAGT**

**GCCGGTCTCTTTTCTTTTTCCCATTCAAAATTGCTCTCCTCTTCTCAATTGAGAATGTCTTTCTGGACAG**

**ACGATTGACACCTCAATGGAGTAAAGATATTAGGGGTATGGAACTTTATTTCTTCTCACTCCGAGGAAGG**

**TTCACTAGTCCTTCTGCTTTGTTATTAGGCAGACCCCTGTAGGCCTATCGACCAATGACTGATGTGATCC**

**CTACTTGCGTAATAGAAAAATGGATTTCGTTGTGGAGGTAAATGAATGATGAAATTAAGGATATCACGTA**

**ACTTGATTGTGTACAAAGGCAACTTGGTCTCTGGCTATAAAGTTTCCAGTATTTTGCACCGCTTGTGAAT**

**TATAAGAGGAGGATCCCCCGCTGCAAGGAAACAACGGATCGACTTTTCTAACTCTCTTTAAAGAGTCTGT**

**TTTCAAATTCACTTGCTTGCAAGTTTTGGGTTAGCAGGTTGTTGACTGGGAGAGCTGGTAAAAGGGAACA**

**ACAATCTCCCCCAGCAATACCAAAGATCCATCCAAGGAATGAGCTTCCCTTCCTGTGGACTACAAGGAAA**

**AGATAGAATCTCGTCCGGCAACAGTAGCTGAGTCAATGACAGATGCTCTTGGAAAGAACCTTTCATTCGA**

**TGCTTCTGATTCAAGCTTATTCTTTTCTACCTTTTGAATTAATCTAGAAGCTCAATTAGCAGATTCATTA**

**GAAACCTTTCGACCAGTATAATCATTAAATTTCTCGCATATATAACCAGCATAGCTCTTGTATGAGCGAA**

**GTAATGCCTTCATCCTTTCTCTCCATGTATGCTTAGGAACCATACCAATCCATCCCGAACTTAGTGGTTA**

**AACTCAACAAGTATGGGAAGGGTCCTCCTCTCCCTGGTCAAAGCTCCGTGAACGAGAACTCGTTTTGTTT**

**GTTGGCCGGCTAATGATGGTTTTCTGTCCCAATTCAACCTTACAGTCTTCGGATGACCCAAAAAAGCTAA**

**TACCTTTTTTCTCACAAGCTATTAGTACTACTTTTCACTGGAATGCCACTTTGGAAGTTAAATTGAAAGC**

**AATTATTGTTCATTTTAGAAATTTTCACATTGAGAAAAGTAACATGTCTTGATATTTTATTGCCGTATGT**

**AAACTCAACTCCACTTTAAAATCAATCCTGGTTAAAAGTGAGATAATAAAAAATGATACAAGATTACCAC**

**AACCGGGGGAGAGGGATAGTTACTTTCCTATTTGTGTCTCGGAAAAAATGATGAGATAATAAAAATGACA**

**CACAATGTGGGGGATGGAGAATAATTTTGAAGTCTACATTAAGGGAAAAAAAGAGAGCAAAAAGGATAAG**

**ATTTAAGTGTTTTTTGGTAGTTATTTTCAATTACAATAAAGTCAAGTCCTAGTACAACTGTTGTTAGTCA**

**TAAATAAATTAATCAAATATTACATTTTCCCAATGTTTTCCTACATATTTACAATCTTCAACTTATTGTG**

**GAGTAAAGTTTCAGCTAATGGTCGGGTTCAAGTATTTTAGTATTAACCCGTTTGACATTGATGAAAAAAC**

**CAGGTGACAACATTTAATTGAATATTTATTACACCTCAGATTTGTATATACATACGCGCTATATTTTTTG**

**CAATTGTTCCAGCTTTTGTGTTAAAGTGGAACATAAGAAATAAAATTGCAGGGCCAAAATGGAACAAACA**

**AGATAAAGTTGGAGGGTCAAAATAGAACAAGATAGAGATAGAGTGTCAAAATGAAAAATGGTGTCAAGTT**

**GAATTGGCTGTATATGTATTTGGTCAAAATAAAAACTAGACTAGAAACACGAGTAAATCAATAGGAAAAT**

**CTGAGTCGCCTCATTGGCTTGTACATTTAGCTGTCTATTGTGTACATACTAAGGTGGATTACGCAAGTGA**

**AATACAGGGTATTATAGTCATTCTCGCTCACGTCTATATATATATATTCAAACCCTAGCTAAATCAATGG**

**TATCGCTCAATCGCCATTTTCTAGTCTCTTATCAAATTTCCCTATGCAGCTGACTTGTAAATGTTCTTCG**

**AGTATCTTCATTCTTCCTTAATCCTAATGAAGTTTTAGAGGCGAAGATACTCGAAAAACATTTACTTGTT**

**ATTTGTATAGAATTACTATATATAGTCCGTCAGAACTCAAACACAATTTCTTCCTCCAGATCTGTGCTTA**

**CCAAATCGATAGTTTAGCTCAATCGCCGTTTCTCAGTCTCTTAAATTTCTCCTAGTTGTCAAAAGTTCGT**

**ACAGGTGACTTGTAAATGTTTTGCTGACCTTCTAATATGATATAACTGGCAAAACATTTACACGTACCTT**

**GTATGAACTTTTGATGCTAGCTTTGCTGAAACCTTTTGATGGAGAACTGCTCTAACTCAAGTGGCCAACC**

**TGAAAGGCTGTGATATTCACAGGTCGGCGAGTTTAAAAAACACATTAATTGGTATAATGTTTCCATTTTT**

**GCTTAGAACACTTCGAAATGAATTAATTAAACAAAATCAGCACTAACTCAACTGAGTTTTTGGATCTAAC**

**ATGAGGGAAAAATTTAATAATTAATTGTTATTTTGATGTTAAACAGAGACGCTCTATGCTTTAATAGGAG**

**TTAGCAAATCTCTTGGGATTCATAATGGAAGAAAAAGATCTATTAGTTGAGAATATGTTTTAGTCAAGTG**

**AGCACTTTTATTTTACATCCTATACTATTCTTGTAGTTGTTTTAAGCGTTTTTAGAAGCGTTTTAGAGAT**

**TTATACATCAATAGAAAATATAAAGAATCTTTAATACTTATTATTATTGTTTTTGTTTTGTAATGGCAGA**

**TCTGACTAGTTTACGTCCTGTAGAAACCCCAACCCGTGAAATCAAAAAACTCGACGGCCTGTGGGCATTC**

**AGTCTGGATCGCGAAAACTGTGGAATTGATCAGCGTTGGTGGGAAAGCGCGTTACAAGAAAGCCGGGCAA**

**TTGCTGTGCCAGGCAGTTTTAACGATCAGTTCGCCGATGCAGATATTCGTAATTATGCGGGCAACGTCTG**

**GTATCAGCGCGAAGTCTTTATACCGAAAGGTTGGGCAGGCCAGCGTATCGTGCTGCGTTTCGATGCGGTC**

**ACTCATTACGGCAAAGTGTGGGTCAATAATCAGGAAGTGATGGAGCATCAGGGCGGCTATACGCCATTTG**

**AAGCCGATGTCACGCCGTATGTTATTGCCGGGAAAAGTGTACGTATCACCGTTTGTGTGAACAACGAACT**

**GAACTGGCAGACTATCCCGCCGGGAATGGTGATTACCGACGAAAACGGCAAGAAAAAGCAGTCTTACTTC**

**CATGATTTCTTTAACTATGCCGGAATCCATCGCAGCGTAATGCTCTACACCACGCCGAACACCTGGGTGG**

**ACGATATCACCGTGGTGACGCATGTCGCGCAAGACTGTAACCACGCGTCTGTTGACTGGCAGGTGGTGGC**

**CAATGGTGATGTCAGCGTTGAACTGCGTGATGCGGATCAACAGGTGGTTGCAACTGGACAAGGCACTAGC**

**GGGACTTTGCAAGTGGTGAATCCGCACCTCTGGCAACCGGGTGAAGGTTATCTCTATGAACTGTGCGTCA**

**CAGCCAAAAGCCAGACAGAGTGTGATATCTACCCGCTTCGCGTCGGCATCCGGTCAGTGGCAGTGAAGGG**

**CCAACAGTTCCTGATTAACCACAAACCGTTCTACTTTACTGGCTTTGGTCGTCATGAAGATGCGGACTTA**

**CGTGGCAAAGGATTCGATAACGTGCTGATGGTGCACGACCACGCATTAATGGACTGGATTGGGGCCAACT**

**CCTACCGTACCTCGCATTACCCTTACGCTGAAGAGATGCTCGACTGGGCAGATGAACATGGCATCGTGGT**

**GATTGATGAAACTGCTGCTGTCGGCTTTCAGCTGTCTTTAGGCATTGGTTTCGAAGCGGGCAACAAGCCG**

**AAAGAACTGTACAGCGAAGAGGCAGTCAACGGGGAAACTCAGCAAGCGCACTTACAGGCGATTAAAGAGC**

**TGATAGCGCGTGACAAAAACCACCCAAGCGTGGTGATGTGGAGTATTGCCAACGAACCGGATACCCGTCC**

**GCAAGGTGCACGGGAATATTTCGCGCCACTGGCGGAAGCAACGCGTAAACTCGACCCGACGCGTCCGATC**

**ACCTGCGTCAATGTAATGTTCTGCGACGCTCACACCGATACCATCAGCGATCTCTTTGATGTGCTGTGCC**

**TGAACCGTTATTACGGATGGTATGTCCAAAGCGGCGATTTGGAAACGGCAGAGAAGGTACTGGAAAAAGA**

**ACTTCTGGCCTGGCAGGAGAAACTGCATCAGCCGATTATCATCACCGAATACGGCGTGGATACGTTAGCC**

**GGGCTGCACTCAATGTACACCGACATGTGGAGTGAAGAGTATCAGTGTGCATGGCTGGATATGTATCACC**

**GCGTCTTTGATCGCGTCAGCGCCGTCGTCGGTGAACAGGTATGGAATTTCGCCGATTTTGCGACCTCGCA**

**AGGCATATTGCGCGTTGGCGGTAACAAGAAAGGGATCTTCACTCGCGACCGCAAACCGAAGTCGGCGGCT**

**TTTCTGCTGCAAAAACGCTGGACTGGCATGAACTTCGGTGAAAAACCGCAGCAGGGAGGCAAACAAGCTA**

**GCCACCACCACCACCACCACGTGTGAATTGGTGACCAGCTCGAATTTCCCCGATCGTTCAAACATTTGGC**

**AATAAAGTTTCTTAAGATTGAATCCTGTTGCCGGTCTTGCGATGATTATCATATAATTTCTGTTGAATTA**

**CGTTAAGCATGTAATAATTAACATGTAATGCATGACGTTATTTATGAGATGGGTTTTTATGATTAGAGTC**

**CCGCAATTATACATTTAATACGCGATAGAAAACAAAATATAGCGCGCAAACTAGGATAAATTATCGCGCG**

**CGGTGTCATCTATGTTACTAGATCGGGAATTAAACTATCAGTGTTTGACAGGATATATTGGCGGGTAAAC**

**CTAAGAGAAAAGAGCGTTTA**

**>sly-MIR6020 (1bp - 10087bp, direct) 10087bp**

**GGGTACCCTCGAATTATCATACATGAGAATTAAGGGAGTCACGTTATGACCCCGCCGATGACGCGGGACA**

**AGCCGTTTTACGTTTGGAACTGACAGAACCGCAACGTTGAAGGAGCCACTGAGCCGCGGGTTTCTGGAGT**

**TTAATGAGCTAAGCACATACGTCAGAAACCATTATTGCGCGTTCAAAAGTCGCCTAAGGTCACTATCAGC**

**TAGCAAATATTTCTTGTCAAAAATGCTCCACTGACGTTCCATAAATTCCCCTCGGTATCCAATTAGAGTC**

**TCATATTCACTCTCAACTCGAATCCCCCCTATGAAAAAGCCTGAACTCACCGCGACGTCTGTCGAGAAGT**

**TTCTGATCGAAAAGTTCGACAGCGTCTCCGACCTGATGCAGCTCTCGGAGGGCGAAGAATCTCGTGCTTT**

**CAGCTTCGATGTAGGAGGGCGTGGATATGTCCTGCGGGTAAATAGCTGCGCCGATGGTTTCTACAAAGAT**

**CGTTATGTTTATCGGCACTTTGCATCGGCCGCGCTCCCGATTCCGGAAGTGCTTGACATTGGGGAGTTCA**

**GCGAGAGCCTGACCTATTGCATCTCCCGCCGTGCACAGGGTGTCACGTTGCAAGACCTGCCTGAAACCGA**

**ACTGCCCGCTGTTCTTCAGCCGGTCGCGGAGGCTATGGATGCTATCGCTGCGGCCGATCTTAGCCAGACG**

**AGCGGGTTCGGCCCATTCGGACCGCAAGGAATCGGTCAATACACTACATGGCGTGATTTCATATGCGCGA**

**TTGCTGATCCCCATGTGTATCACTGGCAAACTGTGATGGACGACACCGTCAGTGCGTCCGTCGCGCAGGC**

**TCTCGATGAGCTGATGCTTTGGGCGAGGACTGCCCCGAAGTCCGGCACTCGTGCACGCGGATTCGGCTCC**

**AACAATGTCCTGACGGACAATGGCCGCATAACAGCGGTCATTGACTGGAGCGAGGCGATGTTCGGGGATT**

**CCCAATACGAGGTCGCCAACATCTTCTTCTGGAGGCCGTGGTTGGCTTGTATGGAGCAGCAGACGCGCTA**

**CTTCGAGCGGAGGCATCCGGAGCTTGCAGGATCGCCAAGGCTCCGGGCGTATATGCTCCGCATTGGTCTT**

**GACCAACTCTATCAGAGCTTGGTTGACGGCAATTTCGATGATGCAGCTTGGGCGCAGGGTCGATGCGACG**

**CAATCGTCCGATCCGGAGCCGGGACTGTCGGGCGTACACAAATCGCCCGCAGAAGCGCGGCCGTCTGGAC**

**CGATGGCTGTGTAGAAGTACTCGCCGATAGTGGAAACCGACGCCCCAGCACTCGTCCGAGGGCAAAGGAA**

**TAGAGTAGATGCCGACCGAACAAGAGCTGATTTCGAGAACGCCTCAGCCAGCAACTCGCGCGAGCCTAGC**

**AAGGCAAATGCGAGAGAACGGCCTTACGCTTGGTGGCACAGTTCTCGTCCACAGTTCGCTAAGCTCGCTC**

**GGCTGGGTCGCGGGAGGGCCGGTCGCAGTGATTCAGGAATTAATTCCCTAGAGTCAAGCAGATCGTTCAA**

**ACATTTGGCAATAAAGTTTCTTAAGATTGAATCCTGTTGCCGGTCTTGCGATGATTATCATATAATTTCT**

**GTTGAATTACGTTAAGCATGTAATAATTAACATGTAATGCATGACGTTATTTATGAGATGGGTTTTTATG**

**ATTAGAGTCCCGCAATTATACATTTAATACGCGATAGAAAACAAAATATAGCGCGCAAACTAGGATAAAT**

**TATCGCGCGCGGTGTCATCTATGTTACTAGATCGACCGGCATGCAAGCTGATAATTCAATTCGGCGTTAA**

**TTCAGTACATTAAAAACGTCCGCAATGTGTTATTAAGTTGTCTAAGCGTCAATTTGTTTACACCACAATA**

**TATCCTGCCACCAGCCAGCCAACAGCTCCCCGACCGGCAGCTCGGCACAAAATCACCACTCGATACAGGC**

**AGCCCATCAGTCCGGGACGGCGTCAGCGGGAGAGCCGTTGTAAGGCGGCAGACTTTGCTCATGTTACCGA**

**TGCTATTCGGAAGAACGGCAACTAAGCTGCCGGGTTTGAAACACGGATGATCTCGCGGAGGGTAGCATGT**

**TGATTGTAACGATGACAGAGCGTTGCTGCCTGTGATCAATTCGGGCACGAACCCAGTGGACATAAGCCTC**

**GTTCGGTTCGTAAGCTGTAATGCAAGTAGCGTAACTGCCGTCACGCAACTGGTCCAGAACCTTGACCGAA**

**CGCAGCGGTGGTAACGGCGCAGTGGCGGTTTTCATGGCTTCTTGTTATGACATGTTTTTTTGGGGTACAG**

**TCTATGCCTCGGGCATCCAAGCAGCAAGCGCGTTACGCCGTGGGTCGATGTTTGATGTTATGGAGCAGCA**

**ACGATGTTACGCAGCAGGGCAGTCGCCCTAAAACAAAGTTAAACATCATGGGGGAAGCGGTGATCGCCGA**

**AGTATCGACTCAACTATCAGAGGTAGTTGGCGTCATCGAGCGCCATCTCGAACCGACGTTGCTGGCCGTA**

**CATTTGTACGGCTCCGCAGTGGATGGCGGCCTGAAGCCACACAGTGATATTGATTTGCTGGTTACGGTGA**

**CCGTAAGGCTTGATGAAACAACGCGGCGAGCTTTGATCAACGACCTTTTGGAAACTTCGGCTTCCCCTGG**

**AGAGAGCGAGATTCTCCGCGCTGTAGAAGTCACCATTGTTGTGCACGACGACATCATTCCGTGGCGTTAT**

**CCAGCTAAGCGCGAACTGCAATTTGGAGAATGGCAGCGCAATGACATTCTTGCAGGTATCTTCGAGCCAG**

**CCACGATCGACATTGATCTGGCTATCTTGCTGACAAAAGCAAGAGAACATAGCGTTGCCTTGGTAGGTCC**

**AGCGGCGGAGGAACTCTTTGATCCGGTTCCTGAACAGGATCTATTTGAGGCGCTAAATGAAACCTTAACG**

**CTATGGAACTCGCCGCCCGACTGGGCTGGCGATGAGCGAAATGTAGTGCTTACGTTGTCCCGCATTTGGT**

**ACAGCGCAGTAACCGGCAAAATCGCGCCGAAGGATGTCGCTGCCGACTGGGCAATGGAGCGCCTGCCGGC**

**CCAGTATCAGCCCGTCATACTTGAAGCTAGACAGGCTTATCTTGGACAAGAAGAAGATCGCTTGGCCTCG**

**CGCGCAGATCAGTTGGAAGAATTTGTCCACTACGTGAAAGGCGAGATCACCAAGGTAGTCGGCAAATAAT**

**GTCTAGCTAGAAATTCGTTCAAGCCGACGCCGCTTCGCCGGCGTTAACTCAAGCGATTAGATGCACTAAG**

**CACATAATTGCTCACAGCCAAACTATCAGGTCAAGTCTGCTTTTATTATTTTTAAGCGTGCATAATAAGC**

**CCTACACAAATTGGGAGATATATCATGCATGACCAAAATCCCTTAACGTGAGTTTTCGTTCCACTGAGCG**

**TCAGACCCCGTAGAAAAGATCAAAGGATCTTCTTGAGATCCTTTTTTTCTGCGCGTAATCTGCTGCTTGC**

**AAACAAAAAAACCACCGCTACCAGCGGTGGTTTGTTTGCCGGATCAAGAGCTACCAACTCTTTTTCCGAA**

**GGTAACTGGCTTCAGCAGAGCGCAGATACCAAATACTGTCCTTCTAGTGTAGCCGTAGTTAGGCCACCAC**

**TTCAAGAACTCTGTAGCACCGCCTACATACCTCGCTCTGCTAATCCTGTTACCAGTGGCTGCTGCCAGTG**

**GCGATAAGTCGTGTCTTACCGGGTTGGACTCAAGACGATAGTTACCGGATAAGGCGCAGCGGTCGGGCTG**

**AACGGGGGGTTCGTGCACACAGCCCAGCTTGGAGCGAACGACCTACACCGAACTGAGATACCTACAGCGT**

**GAGCTATGAGAAAGCGCCACGCTTCCCGAAGGGAGAAAGGCGGACAGGTATCCGGTAAGCGGCAGGGTCG**

**GAACAGGAGAGCGCACGAGGGAGCTTCCAGGGGGAAACGCCTGGTATCTTTATAGTCCTGTCGGGTTTCG**

**CCACCTCTGACTTGAGCGTCGATTTTTGTGATGCTCGTCAGGGGGGCGGAGCCTATGGAAAAACGCCAGC**

**AACGCGGCCTTTTTACGGTTCCTGGCCTTTTGCTGGCCTTTTGCTCACATGTTCTTTCCTGCGTTATCCC**

**CTGATTCTGTGGATAACCGTATTACCGCCTTTGAGTGAGCTGATACCGCTCGCCGCAGCCGAACGACCGA**

**GCGCAGCGAGTCAGTGAGCGAGGAAGCGGAAGAGCGCCTGATGCGGTATTTTCTCCTTACGCATCTGTGC**

**GGTATTTCACACCGCATATGGTGCACTCTCAGTACAATCTGCTCTGATGCCGCATAGTTAAGCCAGTATA**

**CACTCCGCTATCGCTACGTGACTGGGTCATGGCTGCGCCCCGACACCCGCCAACACCCGCTGACGCGCCC**

**TGACGGGCTTGTCTGCTCCCGGCATCCGCTTACAGACAAGCTGTGACCGTCTCCGGGAGCTGCATGTGTC**

**AGAGGTTTTCACCGTCATCACCGAAACGCGCGAGGCAGGGTGCCTTGATGTGGGCGCCGGCGGTCGAGTG**

**GCGACGGCGCGGCTTGTCCGCGCCCTGGTAGATTGCCTGGCCGTAGGCCAGCCATTTTTGAGCGGCCAGC**

**GGCCGCGATAGGCCGACGCGAAGCGGCGGGGCGTAGGGAGCGCAGCGACCGAAGGGTAGGCGCTTTTTGC**

**AGCTCTTCGGCTGTGCGCTGGCCAGACAGTTATGCACAGGCCAGGCGGGTTTTAAGAGTTTTAATAAGTT**

**TTAAAGAGTTTTAGGCGGAAAAATCGCCTTTTTTCTCTTTTATATCAGTCACTTACATGTGTGACCGGTT**

**CCCAATGTACGGCTTTGGGTTCCCAATGTACGGGTTCCGGTTCCCAATGTACGGCTTTGGGTTCCCAATG**

**TACGTGCTATCCACAGGAAAGAGACCTTTTCGACCTTTTTCCCCTGCTAGGGCAATTTGCCCTAGCATCT**

**GCTCCGTACATTAGGAACCGGCGGATGCTTCGCCCTCGATCAGGTTGCGGTAGCGCATGACTAGGATCGG**

**GCCAGCCTGCCCCGCCTCCTCCTTCAAATCGTACTCCGGCAGGTCATTTGACCCGATCAGCTTGCGCACG**

**GTGAAACAGAACTTCTTGAACTCTCCGGCGCTGCCACTGCGTTCGTAGATCGTCTTGAACAACCATCTGG**

**CTTCTGCCTTGCCTGCGGCGCGGCGTGCCAGGCGGTAGAGAAAACGGCCGATGCCGGGATCGATCAAAAA**

**GTAATCGGGGTGAACCGTCAGCACGTCCGGGTTCTTGCCTTCTGTGATCTCGCGGTACATCCAATCAGCT**

**AGCTCGATCTCGATGTACTCCGGCCGCCCGGTTTCGCTCTTTACGATCTTGTAGCGGCTAATCAAGGCTT**

**CACCCTCGGATACCGTCACCAGGCGGCCGTTCTTGGCCTTCTTCGTACGCTGCATGGCAACGTGCGTGGT**

**GTTTAACCGAATGCAGGTTTCTACCAGGTCGTCTTTCTGCTTTCCGCCATCGGCTCGCCGGCAGAACTTG**

**AGTACGTCCGCAACGTGTGGACGGAACACGCGGCCGGGCTTGTCTCCCTTCCCTTCCCGGTATCGGTTCA**

**TGGATTCGGTTAGATGGGAAACCGCCATCAGTACCAGGTCGTAATCCCACACACTGGCCATGCCGGCCGG**

**CCCTGCGGAAACCTCTACGTGCCCGTCTGGAAGCTCGTAGCGGATCACCTCGCCAGCTCGTCGGTCACGC**

**TTCGACAGACGGAAAACGGCCACGTCCATGATGCTGCGACTATCGCGGGTGCCCACGTCATAGAGCATCG**

**GAACGAAAAAATCTGGTTGCTCGTCGCCCTTGGGCGGCTTCCTAATCGACGGCGCACCGGCTGCCGGCGG**

**TTGCCGGGATTCTTTGCGGATTCGATCAGCGGCCGCTTGCCACGATTCACCGGGGCGTGCTTCTGCCTCG**

**ATGCGTTGCCGCTGGGCGGCCTGCGCGGCCTTCAACTTCTCCACCAGGTCATCACCCAGCGCCGCGCCGA**

**TTTGTACCGGGCCGGATGGTTTGCGACCGTCACGCCGATTCCTCGGGCTTGGGGGTTCCAGTGCCATTGC**

**AGGGCCGGCAGACAACCCAGCCGCTTACGCCTGGCCAACCGCCCGTTCCTCCACACATGGGGCATTCCAC**

**GGCGTCGGTGCCTGGTTGTTCTTGATTTTCCATGCCGCCTCCTTTAGCCGCTAAAATTCATCTACTCATT**

**TATTCATTTGCTCATTTACTCTGGTAGCTGCGCGATGTATTCAGATAGCAGCTCGGTAATGGTCTTGCCT**

**TGGCGTACCGCGTACATCTTCAGCTTGGTGTGATCCTCCGCCGGCAACTGAAAGTTGACCCGCTTCATGG**

**CTGGCGTGTCTGCCAGGCTGGCCAACGTTGCAGCCTTGCTGCTGCGTGCGCTCGGACGGCCGGCACTTAG**

**CGTGTTTGTGCTTTTGCTCATTTTCTCTTTACCTCATTAACTCAAATGAGTTTTGATTTAATTTCAGCGG**

**CCAGCGCCTGGACCTCGCGGGCAGCGTCGCCCTCGGGTTCTGATTCAAGAACGGTTGTGCCGGCGGCGGC**

**AGTGCCTGGGTAGCTCACGCGCTGCGTGATACGGGACTCAAGAATGGGCAGCTCGTACCCGGCCAGCGCC**

**TCGGCAACCTCACCGCCGATGCGCGTGCCTTTGATCGCCCGCGACACGACAAAGGCCGCTTGTAGCCTTC**

**CATCCGTGACCTCAATGCGCTGCTTAACCAGCTCCACCAGGTCGGCGGTGGCCCATATGTCGTAAGGGCT**

**TGGCTGCACCGGAATCAGCACGAAGTCGGCTGCCTTGATCGCGGACACAGCCAAGTCCGCCGCCTGGGGC**

**GCTCCGTCGATCACTACGAAGTCGCGCCGGCCGATGGCCTTCACGTCGCGGTCAATCGTCGGGCGGTCGA**

**TGCCGACAACGGTTAGCGGTTGATCTTCCCGCACGGCCGCCCAATCGCGGGCACTGCCCTGGGGATCGGA**

**ATCGACTAACAGAACATCGGCCCCGGCGAGTTGCAGGGCGCGGGCTAGATGGGTTGCGATGGTCGTCTTG**

**CCTGACCCGCCTTTCTGGTTAAGTACAGCGATAACCTTCATGCGTTCCCCTTGCGTATTTGTTTATTTAC**

**TCATCGCATCATATACGCAGCGACCGCATGACGCAAGCTGTTTTACTCAAATACACATCACCTTTTTAGA**

**CGGCGGCGCTCGGTTTCTTCAGCGGCCAAGCTGGCCGGCCAGGCCGCCAGCTTGGCATCAGACAAACCGG**

**CCAGGATTTCATGCAGCCGCACGGTTGAGACGTGCGCGGGCGGCTCGAACACGTACCCGGCCGCGATCAT**

**CTCCGCCTCGATCTCTTCGGTAATGAAAAACGGTTCGTCCTGGCCGTCCTGGTGCGGTTTCATGCTTGTT**

**CCTCTTGGCGTTCATTCTCGGCGGCCGCCAGGGCGTCGGCCTCGGTCAATGCGTCCTCACGGAAGGCACC**

**GCGCCGCCTGGCCTCGGTGGGCGTCACTTCCTCGCTGCGCTCAAGTGCGCGGTACAGGGTCGAGCGATGC**

**ACGCCAAGCAGTGCAGCCGCCTCTTTCACGGTGCGGCCTTCCTGGTCGATCAGCTCGCGGGCGTGCGCGA**

**TCTGTGCCGGGGTGAGGGTAGGGCGGGGGCCAAACTTCACGCCTCGGGCCTTGGCGGCCTCGCGCCCGCT**

**CCGGGTGCGGTCGATGATTAGGGAACGCTCGAACTCGGCAATGCCGGCGAACACGGTCAACACCATGCGG**

**CCGGCCGGCGTGGTGGTGTCGGCCCACGGCTCTGCCAGGCTACGCAGGCCCGCGCCGGCCTCCTGGATGC**

**GCTCGGCAATGTCCAGTAGGTCGCGGGTGCTGCGGGCCAGGCGGTCTAGCCTGGTCACTGTCACAACGTC**

**GCCAGGGCGTAGGTGGTCAAGCATCCTGGCCAGCTCCGGGCGGTCGCGCCTGGTGCCGGTGATCTTCTCG**

**GAAAACAGCTTGGTGCAGCCGGCCGCGTGCAGTTCGGCCCGTTGGTTGGTCAAGTCCTGGTCGTCGGTGC**

**TGACGCGGGCATAGCCCAGCAGGCCAGCGGCGGCGCTCTTGTTCATGGCGTAATGTCTCCGGTTCTAGTC**

**GCAAGTATTCTACTTTATGCGACTAAAACACGCGACAAGAAAACGCCAGGAAAAGGGCAGGGCGGCAGCC**

**TGTCGCGTAACTTAGGACTTGTGCGACATGTCGTTTTCAGAAGACGGCTGCACTGAACGTCAGAAGCCGA**

**CTGCACTATAGCAGCGGAGGGGTTGGATCAAAGTACTTTGATCCCGAGGGGAACCCTGTGGTTGGCATGC**

**ACATACAAATGGACGAACGGATAAACCTTTTCACGCCCTTTTAAATATCCGTTATTCTAATAAACGCTCT**

**TTTCTCTTAGGTTTACCCGCCAATATATCCTGTCAAACACTGATAGTTTAAACTGAAGGCGGGAAACGAC**

**AATCTGATCCAAGCTCAAGCTAAGCTTGAGCTCTGCATGCCTGCAGGTCCCCAGATTAGCCTTTTCAATT**

**TCAGAAAGAATGCTAACCCACAGATGGTTAGAGAGGCTTACGCAGCAGGTCTCATCAAGACGATCTACCC**

**GAGCAATAATCTCCAGGAAATCAAATACCTTCCCAAGAAGGTTAAAGATGCAGTCAAAAGATTCAGGACT**

**AACTGCATCAAGAACACAGAGAAAGATATATTTCTCAAGATCAGAAGTACTATTCCAGTATGGACGATTC**

**AAGGCTTGCTTCACAAACCAAGGCAAGTAATAGAGATTGGAGTCTCTAAAAAGGTAGTTCCCACTGAATC**

**AAAGGCCATGGAGTCAAAGATTCAAATAGAGGACCTAACAGAACTCGCCGTAAAGACTGGCGAACAGTTC**

**ATACAGAGTCTCTTACGACTCAATGACAAGAAGAAAATCTTCGTCAACATGGTGGAGCACGACACACTTG**

**TCTACTCCAAAAATATCAAAGATACAGTCTCAGAAGACCAAAGGGCAATTGAGACTTTTCAACAAAGGGT**

**AATATCCGGAAACCTCCTCGGATTCCATTGCCCAGCTATCTGTCACTTTATTGTGAAGATAGTGGAAAAG**

**GAAGGTGGCTCCTACAAATGCCATCATTGCGATAAAGGAAAGGCCATCGTTGAAGATGCCTCTGCCGACA**

**GTGGTCCCAAAGATGGACCCCCACCCACGAGGAGCATCGTGGAAAAAGAAGACGTTCCAACCACGTCTTC**

**AAAGCAAGTGGATTGATGTGATATCTCCACTGACGTAAGGGATGACGCACAATCCCACTATCCTTCGCAA**

**GACCCTTCCTCTATATAAGGAAGTTCATTTCATTTGGAGAGAACACGGGGGACTCTAGAGGATCCAAGGA**

**GATATAACATTACGCCGAGGCCAATTTCAAATAAGGCCCAAAGGCCATTTGTGAGCCCATAACTAGATAA**

**CCTATAAAATCTAACCAGCTGTAGTGAAAACCTAAAAGCTGTAACATTTTCTTTGAAAATGGAGTTTAAT**

**GGCGAAAATACTCGTAAAACATTTACGGATCACTTCTAGGTAGGATTGAATATCAGAGCAATATCATATA**

**TTGTAGATCTGTAAATGTTTTCCGAGTATCTTTTGCCCATAACCTTCAAAAATTATGAAGTATTTTAATA**

**CTGATATGGATCCGTCCGATGTTCAAATCCAAAGGGATTGCCAATCTGACGGGTGAGTTGAGTAAAATAA**

**AATATTTTTAATTATCAATTATTGTATCTCGTAGTAGTTTTTATATATAAATTTCATTTAATGGAATAAA**

**GGTTTATCTACAAATTGTCAGTCCTCTTCCCTAATGATATCCCGCGGCCATGCTAGAGTCCGCAAAAATC**

**ACCAGTCTCTCTCTACAAATCTATCTCTCTCTATTTTTCTCCAGAATAATGTGTGAGTAGTTCCCAGATA**

**AGGGAATTAGGGTTCTTATAGGGTTTCGCTCATGTGTTGAGCATATAAGAAACCCTTAGTATGTATTTGT**

**ATTTGTAAAATACTTCTATCAATAAAATTTCTAATTCCTAAAACCAAAATCCAGTGACCTGCAGGCATGC**

**GACGTCG**

**>AMIR6020 (1bp - 9806bp, complementary) 9806bp**

**GGTACCCTCGAATTATCATACATGAGAATTAAGGGAGTCACGTTATGACCCCCGCCGATGACGCGGGACA**

**AGCCGTTTTACGTTTGGAACTGACAGAACCGCAACGTTGAAGGAGCCACTCAGCCGCGGGTTTCTGGAGT**

**TTAATGAGCTAAGCACATACGTCAGAAACCATTATTGCGCGTTCAAAAGTCGCCTAAGGTCACTATCAGC**

**TAGCAAATATTTCTTGTCAAAAATGCTCCACTGACGTTCCATAAATTCCCCTCGGTATCCAATTAGAGTC**

**TCATATTCACTCTCAACTCGATCGAGGCATGATTGAACAAGATGGATTGCACGCAGGTTCTCCGGCCGCT**

**TGGGTGGAGAGGCTATTCGGCTATGACTGGGCACAACAGACAATCGGCTGCTCTGATGCCGCCGTGTTCC**

**GGCTGTCAGCGCAGGGGCGCCCGGTTCTTTTTGTCAAGACCGACCTGTCCGGTGCCCTGAATGAACTCCA**

**AGACGAGGCAGCGCGGCTATCGTGGCTGGCCACGACGGGCGTTCCTTGCGCAGCTGTGCTCGACGTTGTC**

**ACTGAAGCGGGAAGGGACTGGCTGCTATTGGGCGAAGTGCCGGGGCAGGATCTCCTGTCATCTCACCTTG**

**CTCCTGCCGAGAAAGTATCCATCATGGCTGATGCAATGCGGCGGCTGCATACGCTTGATCCGGCTACCTG**

**CCCATTCGACCACCAAGCGAAACATCGCATCGAGCGAGCACGTACTCGGATGGAAGCCGGTCTTGTCGAT**

**CAGGATGATCTGGACGAAGAGCATCAGGGGCTCGCGCCAGCCGAACTGTTCGCCAGGCTCAAGGCGCGGA**

**TGCCCGACGGCGAGGATCTCGTCGTGACCCACGGCGATGCCTGCTTGCCGAATATCATGGTGGAAAATGG**

**CCGCTTTTCTGGATTCATCGACTGTGGCCGGCTGGGTGTGGCGGACCGCTATCAGGACATAGCGTTGGCT**

**ACCCGTGATATTGCTGAAGAGCTTGGCGGCGAATGGGCTGACCGCTTCCTCGTGCTTTACGGTATCGCCG**

**CTCCCGATTCGCAGCGCATCGCCTTCTATCGCCTTCTTGACGAGTTCTTCTGAGCGGGACTCTGGGGTTC**

**GGACTCTAGCTAGAGTCAAGCAGATCGTTCAAACATTTGGCAATAAAGTTTCTTAAGATTGAATCCTGTT**

**GCCGGTCTTGCGATGATTATCATATAATTTCTGTTGAATTACGTTAAGCATGTAATAATTAACATGTAAT**

**GCATGACGTTATTTATGAGATGGGTTTTTATGATTAGAGTCCCGCAATTATACATTTAATACGCGATAGA**

**AAACAAAATATAGCGCGCAAACTAGGATAAATTATCGCGCGCGGTGTCATCTATGTTACTAGATCGACCG**

**GCATGCAAGCTGATAATTCAATTCGGCGTTAATTCAGTACATTAAAAACGTCCGCAATGTGTTATTAAGT**

**TGTCTAAGCGTCAATTTGTTTACACCACAATATATCCTGCCACCAGCCAGCCAACAGCTCCCCGACCGGC**

**AGCTCGGCACAAAATCACCACTCGATACAGGCAGCCCATCAGTCCGGGACGGCGTCAGCGGGAGAGCCGT**

**TGTAAGGCGGCAGACTTTGCTCATGTTACCGATGCTATTCGGAAGAACGGCAACTAAGCTGCCGGGTTTG**

**AAACACGGATGATCTCGCGGAGGGTAGCATGTTGATTGTAACGATGACAGAGCGTTGCTGCCTGTGATCA**

**ATTCGGGCACGAACCCAGTGGACATAAGCCTCGTTCGGTTCGTAAGCTGTAATGCAAGTAGCGTAACTGC**

**CGTCACGCAACTGGTCCAGAACCTTGACCGAACGCAGCGGTGGTAACGGCGCAGTGGCGGTTTTCATGGC**

**TTCTTGTTATGACATGTTTTTTTGGGGTACAGTCTATGCCTCGGGCATCCAAGCAGCAAGCGCGTTACGC**

**CGTGGGTCGATGTTTGATGTTATGGAGCAGCAACGATGTTACGCAGCAGGGCAGTCGCCCTAAAACAAAG**

**TTAAACATCATGGGGGAAGCGGTGATCGCCGAAGTATCGACTCAACTATCAGAGGTAGTTGGCGTCATCG**

**AGCGCCATCTCGAACCGACGTTGCTGGCCGTACATTTGTACGGCTCCGCAGTGGATGGCGGCCTGAAGCC**

**ACACAGTGATATTGATTTGCTGGTTACGGTGACCGTAAGGCTTGATGAAACAACGCGGCGAGCTTTGATC**

**AACGACCTTTTGGAAACTTCGGCTTCCCCTGGAGAGAGCGAGATTCTCCGCGCTGTAGAAGTCACCATTG**

**TTGTGCACGACGACATCATTCCGTGGCGTTATCCAGCTAAGCGCGAACTGCAATTTGGAGAATGGCAGCG**

**CAATGACATTCTTGCAGGTATCTTCGAGCCAGCCACGATCGACATTGATCTGGCTATCTTGCTGACAAAA**

**GCAAGAGAACATAGCGTTGCCTTGGTAGGTCCAGCGGCGGAGGAACTCTTTGATCCGGTTCCTGAACAGG**

**ATCTATTTGAGGCGCTAAATGAAACCTTAACGCTATGGAACTCGCCGCCCGACTGGGCTGGCGATGAGCG**

**AAATGTAGTGCTTACGTTGTCCCGCATTTGGTACAGCGCAGTAACCGGCAAAATCGCGCCGAAGGATGTC**

**GCTGCCGACTGGGCAATGGAGCGCCTGCCGGCCCAGTATCAGCCCGTCATACTTGAAGCTAGACAGGCTT**

**ATCTTGGACAAGAAGAAGATCGCTTGGCCTCGCGCGCAGATCAGTTGGAAGAATTTGTCCACTACGTGAA**

**AGGCGAGATCACCAAGGTAGTCGGCAAATAATGTCTAGCTAGAAATTCGTTCAAGCCGACGCCGCTTCGC**

**CGGCGTTAACTCAAGCGATTAGATGCACTAAGCACATAATTGCTCACAGCCAAACTATCAGGTCAAGTCT**

**GCTTTTATTATTTTTAAGCGTGCATAATAAGCCCTACACAAATTGGGAGATATATCATGCATGACCAAAA**

**TCCCTTAACGTGAGTTTTCGTTCCACTGAGCGTCAGACCCCGTAGAAAAGATCAAAGGATCTTCTTGAGA**

**TCCTTTTTTTCTGCGCGTAATCTGCTGCTTGCAAACAAAAAAACCACCGCTACCAGCGGTGGTTTGTTTG**

**CCGGATCAAGAGCTACCAACTCTTTTTCCGAAGGTAACTGGCTTCAGCAGAGCGCAGATACCAAATACTG**

**TCCTTCTAGTGTAGCCGTAGTTAGGCCACCACTTCAAGAACTCTGTAGCACCGCCTACATACCTCGCTCT**

**GCTAATCCTGTTACCAGTGGCTGCTGCCAGTGGCGATAAGTCGTGTCTTACCGGGTTGGACTCAAGACGA**

**TAGTTACCGGATAAGGCGCAGCGGTCGGGCTGAACGGGGGGTTCGTGCACACAGCCCAGCTTGGAGCGAA**

**CGACCTACACCGAACTGAGATACCTACAGCGTGAGCTATGAGAAAGCGCCACGCTTCCCGAAGGGAGAAA**

**GGCGGACAGGTATCCGGTAAGCGGCAGGGTCGGAACAGGAGAGCGCACGAGGGAGCTTCCAGGGGGAAAC**

**GCCTGGTATCTTTATAGTCCTGTCGGGTTTCGCCACCTCTGACTTGAGCGTCGATTTTTGTGATGCTCGT**

**CAGGGGGGCGGAGCCTATGGAAAAACGCCAGCAACGCGGCCTTTTTACGGTTCCTGGCCTTTTGCTGGCC**

**TTTTGCTCACATGTTCTTTCCTGCGTTATCCCCTGATTCTGTGGATAACCGTATTACCGCCTTTGAGTGA**

**GCTGATACCGCTCGCCGCAGCCGAACGACCGAGCGCAGCGAGTCAGTGAGCGAGGAAGCGGAAGAGCGCC**

**TGATGCGGTATTTTCTCCTTACGCATCTGTGCGGTATTTCACACCGCATATGGTGCACTCTCAGTACAAT**

**CTGCTCTGATGCCGCATAGTTAAGCCAGTATACACTCCGCTATCGCTACGTGACTGGGTCATGGCTGCGC**

**CCCGACACCCGCCAACACCCGCTGACGCGCCCTGACGGGCTTGTCTGCTCCCGGCATCCGCTTACAGACA**

**AGCTGTGACCGTCTCCGGGAGCTGCATGTGTCAGAGGTTTTCACCGTCATCACCGAAACGCGCGAGGCAG**

**GGTGCCTTGATGTGGGCGCCGGCGGTCGAGTGGCGACGGCGCGGCTTGTCCGCGCCCTGGTAGATTGCCT**

**GGCCGTAGGCCAGCCATTTTTGAGCGGCCAGCGGCCGCGATAGGCCGACGCGAAGCGGCGGGGCGTAGGG**

**AGCGCAGCGACCGAAGGGTAGGCGCTTTTTGCAGCTCTTCGGCTGTGCGCTGGCCAGACAGTTATGCACA**

**GGCCAGGCGGGTTTTAAGAGTTTTAATAAGTTTTAAAGAGTTTTAGGCGGAAAAATCGCCTTTTTTCTCT**

**TTTATATCAGTCACTTACATGTGTGACCGGTTCCCAATGTACGGCTTTGGGTTCCCAATGTACGGGTTCC**

**GGTTCCCAATGTACGGCTTTGGGTTCCCAATGTACGTGCTATCCACAGGAAAGAGACCTTTTCGACCTTT**

**TTCCCCTGCTAGGGCAATTTGCCCTAGCATCTGCTCCGTACATTAGGAACCGGCGGATGCTTCGCCCTCG**

**ATCAGGTTGCGGTAGCGCATGACTAGGATCGGGCCAGCCTGCCCCGCCTCCTCCTTCAAATCGTACTCCG**

**GCAGGTCATTTGACCCGATCAGCTTGCGCACGGTGAAACAGAACTTCTTGAACTCTCCGGCGCTGCCACT**

**GCGTTCGTAGATCGTCTTGAACAACCATCTGGCTTCTGCCTTGCCTGCGGCGCGGCGTGCCAGGCGGTAG**

**AGAAAACGGCCGATGCCGGGATCGATCAAAAAGTAATCGGGGTGAACCGTCAGCACGTCCGGGTTCTTGC**

**CTTCTGTGATCTCGCGGTACATCCAATCAGCTAGCTCGATCTCGATGTACTCCGGCCGCCCGGTTTCGCT**

**CTTTACGATCTTGTAGCGGCTAATCAAGGCTTCACCCTCGGATACCGTCACCAGGCGGCCGTTCTTGGCC**

**TTCTTCGTACGCTGCATGGCAACGTGCGTGGTGTTTAACCGAATGCAGGTTTCTACCAGGTCGTCTTTCT**

**GCTTTCCGCCATCGGCTCGCCGGCAGAACTTGAGTACGTCCGCAACGTGTGGACGGAACACGCGGCCGGG**

**CTTGTCTCCCTTCCCTTCCCGGTATCGGTTCATGGATTCGGTTAGATGGGAAACCGCCATCAGTACCAGG**

**TCGTAATCCCACACACTGGCCATGCCGGCCGGCCCTGCGGAAACCTCTACGTGCCCGTCTGGAAGCTCGT**

**AGCGGATCACCTCGCCAGCTCGTCGGTCACGCTTCGACAGACGGAAAACGGCCACGTCCATGATGCTGCG**

**ACTATCGCGGGTGCCCACGTCATAGAGCATCGGAACGAAAAAATCTGGTTGCTCGTCGCCCTTGGGCGGC**

**TTCCTAATCGACGGCGCACCGGCTGCCGGCGGTTGCCGGGATTCTTTGCGGATTCGATCAGCGGCCGCTT**

**GCCACGATTCACCGGGGCGTGCTTCTGCCTCGATGCGTTGCCGCTGGGCGGCCTGCGCGGCCTTCAACTT**

**CTCCACCAGGTCATCACCCAGCGCCGCGCCGATTTGTACCGGGCCGGATGGTTTGCGACCGTCACGCCGA**

**TTCCTCGGGCTTGGGGGTTCCAGTGCCATTGCAGGGCCGGCAGACAACCCAGCCGCTTACGCCTGGCCAA**

**CCGCCCGTTCCTCCACACATGGGGCATTCCACGGCGTCGGTGCCTGGTTGTTCTTGATTTTCCATGCCGC**

**CTCCTTTAGCCGCTAAAATTCATCTACTCATTTATTCATTTGCTCATTTACTCTGGTAGCTGCGCGATGT**

**ATTCAGATAGCAGCTCGGTAATGGTCTTGCCTTGGCGTACCGCGTACATCTTCAGCTTGGTGTGATCCTC**

**CGCCGGCAACTGAAAGTTGACCCGCTTCATGGCTGGCGTGTCTGCCAGGCTGGCCAACGTTGCAGCCTTG**

**CTGCTGCGTGCGCTCGGACGGCCGGCACTTAGCGTGTTTGTGCTTTTGCTCATTTTCTCTTTACCTCATT**

**AACTCAAATGAGTTTTGATTTAATTTCAGCGGCCAGCGCCTGGACCTCGCGGGCAGCGTCGCCCTCGGGT**

**TCTGATTCAAGAACGGTTGTGCCGGCGGCGGCAGTGCCTGGGTAGCTCACGCGCTGCGTGATACGGGACT**

**CAAGAATGGGCAGCTCGTACCCGGCCAGCGCCTCGGCAACCTCACCGCCGATGCGCGTGCCTTTGATCGC**

**CCGCGACACGACAAAGGCCGCTTGTAGCCTTCCATCCGTGACCTCAATGCGCTGCTTAACCAGCTCCACC**

**AGGTCGGCGGTGGCCCATATGTCGTAAGGGCTTGGCTGCACCGGAATCAGCACGAAGTCGGCTGCCTTGA**

**TCGCGGACACAGCCAAGTCCGCCGCCTGGGGCGCTCCGTCGATCACTACGAAGTCGCGCCGGCCGATGGC**

**CTTCACGTCGCGGTCAATCGTCGGGCGGTCGATGCCGACAACGGTTAGCGGTTGATCTTCCCGCACGGCC**

**GCCCAATCGCGGGCACTGCCCTGGGGATCGGAATCGACTAACAGAACATCGGCCCCGGCGAGTTGCAGGG**

**CGCGGGCTAGATGGGTTGCGATGGTCGTCTTGCCTGACCCGCCTTTCTGGTTAAGTACAGCGATAACCTT**

**CATGCGTTCCCCTTGCGTATTTGTTTATTTACTCATCGCATCATATACGCAGCGACCGCATGACGCAAGC**

**TGTTTTACTCAAATACACATCACCTTTTTAGACGGCGGCGCTCGGTTTCTTCAGCGGCCAAGCTGGCCGG**

**CCAGGCCGCCAGCTTGGCATCAGACAAACCGGCCAGGATTTCATGCAGCCGCACGGTTGAGACGTGCGCG**

**GGCGGCTCGAACACGTACCCGGCCGCGATCATCTCCGCCTCGATCTCTTCGGTAATGAAAAACGGTTCGT**

**CCTGGCCGTCCTGGTGCGGTTTCATGCTTGTTCCTCTTGGCGTTCATTCTCGGCGGCCGCCAGGGCGTCG**

**GCCTCGGTCAATGCGTCCTCACGGAAGGCACCGCGCCGCCTGGCCTCGGTGGGCGTCACTTCCTCGCTGC**

**GCTCAAGTGCGCGGTACAGGGTCGAGCGATGCACGCCAAGCAGTGCAGCCGCCTCTTTCACGGTGCGGCC**

**TTCCTGGTCGATCAGCTCGCGGGCGTGCGCGATCTGTGCCGGGGTGAGGGTAGGGCGGGGGCCAAACTTC**

**ACGCCTCGGGCCTTGGCGGCCTCGCGCCCGCTCCGGGTGCGGTCGATGATTAGGGAACGCTCGAACTCGG**

**CAATGCCGGCGAACACGGTCAACACCATGCGGCCGGCCGGCGTGGTGGTGTCGGCCCACGGCTCTGCCAG**

**GCTACGCAGGCCCGCGCCGGCCTCCTGGATGCGCTCGGCAATGTCCAGTAGGTCGCGGGTGCTGCGGGCC**

**AGGCGGTCTAGCCTGGTCACTGTCACAACGTCGCCAGGGCGTAGGTGGTCAAGCATCCTGGCCAGCTCCG**

**GGCGGTCGCGCCTGGTGCCGGTGATCTTCTCGGAAAACAGCTTGGTGCAGCCGGCCGCGTGCAGTTCGGC**

**CCGTTGGTTGGTCAAGTCCTGGTCGTCGGTGCTGACGCGGGCATAGCCCAGCAGGCCAGCGGCGGCGCTC**

**TTGTTCATGGCGTAATGTCTCCGGTTCTAGTCGCAAGTATTCTACTTTATGCGACTAAAACACGCGACAA**

**GAAAACGCCAGGAAAAGGGCAGGGCGGCAGCCTGTCGCGTAACTTAGGACTTGTGCGACATGTCGTTTTC**

**AGAAGACGGCTGCACTGAACGTCAGAAGCCGACTGCACTATAGCAGCGGAGGGGTTGGATCAAAGTACTT**

**TGATCCCGAGGGGAACCCTGTGGTTGGCATGCACATACAAATGGACGAACGGATAAACCTTTTCACGCCC**

**TTTTAAATATCCGTTATTCTAATAAACGCTCTTTTCTCTTAGGTTTACCCGCCAATATATCCTGTCAAAC**

**ACTGATAGTTTAAACTGAAGGCGGGAAACGACAATCTGATCCAAGCTCAAGCTAAGCTTGAGCTCTCCCA**

**TATGGTCGACTAGAGCCAAGCTGATCTCCTTTGCCCCGGAGATCACCATGGACGACTTTCTCTATCTCTA**

**CGATCTAGGAAGAAAGTTCGACGGAGAAGGTGACGATACCATGTTCACCACCGATAATGAGAAGATTAGC**

**CTCTTCAATTTCAGAAAGAATGCTGACCCACAGATGGTTAGAGAGGCCTACGCGGCAGGTCTCATCAAGA**

**CGATCTACCCGAGTAATAATCTCCAGGAGATCAAATACCTTCCCAAGAAGGTTAAAGATGCAGTCAAAAG**

**ATTCAGGACTAACTGCATCAAGAACACAGAGAAAGATATATTTCTCAAGATCAGAAGTACTATTCCAGTA**

**TGGACGATTCAAGGCTTGCTTCATAAACCAAGGCAAGTAATAGAGATTGGAGTCTCTAAGAAAGTAGTTC**

**CTACTGAATCAAAGGCCATGGAGTCAAAAATTCAGATCGAGGATCTAACAGAACTCGCCGTGAAGACTGG**

**CGAACAGTTCATACAGAGTCTTTTACGACTCAATGACAAGAAGAAAATCTTCGTCAACATGGTGGAGCAC**

**GACACTCTCGTCTACTCCAAGAATATCAAAGATACAGTCTCAGAAGACCAAAGGGCTATTGAGACTTTTC**

**AACAAAGGGTAATATCGGGAAACCTCCTCGGATTCCATTGCCCAGCTATCTGTCACTTCATCAAAAGGAC**

**AGTAGAAAAGGAAGGTGGCACCTACAAATGCCATCATTGCGATAAAGGAAAGGCTATCGTTCAAGATGCC**

**TCTGCCGACAGTGGTCCCAAAGATGGACCCCCACCCACGAGGAGCATCGTGGAAAAAGAAGACGTTCCAA**

**CCACGTCTTCAAAGCAAGTGGATTGATGTGATGATATCATCTCCACTGACGTAAGGGATGACGCACAATC**

**CCACTATCCTTCGCAAGACCCTTCCTCTATATAAGGAAGTTCATTTCATTTGGAGAGGACTCCGGTATTT**

**TTACAACAATACCACAACAAAACAAACAACAAACAACATTACAATTTACTATTCTAGTCGACCTGCAGGC**

**GGCCGCACTAGTTCCAGGGCGCCCTCACCAAACAAAACCTCACAATGAGAGAGTCCCTGTTCTAAATGTT**

**TTCCGAGTATCTTTTTCTTACCTGACCACACACGTAGATATACATTATTCTCTCATGATTAAAAAGATAT**

**CGGAAAACACTTAGATCAGTACTCTCTCGTCAACCAAAGTAAGTAATCACACATAAGAGCTTGGCGGCGA**

**ATAAACCTCCACCAACAATGGGTGAGATTCTCCATTCTTGTCACGCGCTATCAGCTCTTAACCAACAAAC**

**ACGAAATCCGTCACATTTGCTTATTAAGAGCTGATCGCGCGTGCCAAGAATGAGCACTTCACCCCAATAC**

**CATTACCCAACAGAACTAGGGCGTCGGGTCGATGATATCCCGCGGCCATGCTAGAGTCCGCAAAAATCAC**

**CAGTCTCTCTCTACAAATCTATCTCTCTCTATTTTTCTCCAGAATAATGTGTGAGTAGTTCCCAGATAAG**

**GGAATTAGGGTTCTTATAGGGTTTCGCTCATGTGTTGAGCATATAAGAAACCCTTAGTATGTATTTGTAT**

**TTGTAAAATACTTCTATCAATAAAATTTCTAATTCCTAAAACCAAAATCCAGTGACCTGCAGGCATGCGA**

**CGTCGG**

**>** MS4miR6020 **(1bp - 15359bp, direct) 15359bp**

**GGCCGCACTAGTGATATCCCGCGGCCATGGCGGCCGGGAGCATGCGACGTCGGGCCCAATTCGCCCTATA**

**GTGAGTCGTATTACAATTCACTGGCCGTCGTTTTACAACGTCGTGACTGGGAAAACCCTGGCGTTACCCA**

**ACTTAATCGCCTTGCAGCACATCCCCCTTTCGCCAGCTGGCGTAATAGCGAAGAGGCCCGCACCGATCGC**

**CCTTCCCAACAGTTGCGCAGCCTGAATGGCGAATGGAAATTGTAAACGTTAATGGGTTTCTGGAGTTTAA**

**TGAGCTAAGCACATACGTCAGAAACCATTATTGCGCGTTCAAAAGTCGCCTAAGGTCACTATCAGCTAGC**

**AAATATTTCTTGTCAAAAATGCTCCACTGACGTTCCATAAATTCCCCTCGGTATCCAATTAGAGTCTCAT**

**ATTCACTCTCAATCCAAATAATCTGCAATGGTGAGCAAGGGCGAGGAGAATAACATGGCCATCATCAAGG**

**AGTTCATGCGCTTCAAGGTGCGCATGGAGGGCTCCGTGAACGGCCACGAGTTCGAGATCGAGGGCGAGGG**

**CGAGGGCCGCCCCTACGAGGGCTTTCAGACCGCTAAGCTGAAGGTGACCAAGGGTGGCCCCCTGCCCTTC**

**GCCTGGGACATCCTGTCCCCTCAGTTCACCTACGGCTCCAAGGCCTACGTGAAGCACCCCGCCGACATCC**

**CCGACTACTTCAAGCTGTCCTTCCCCGAGGGCTTCAAGTGGGAGCGCGTGATGAACTTCGAGGACGGCGG**

**CGTGGTGACCGTGACCCAGGACTCCTCCCTGCAGGACGGCGAGTTCATCTACAAGGTGAAGCTGCGCGGC**

**ACCAACTTCCCCTCCGACGGCCCCGTAATGCAGAAGAAGACCATGGGCTGGGAGGCCTCCTCCGAGCGGA**

**TGTACCCCGAGGACGGCGCCCTGAAGGGCGAGATCAAGATGAGGCTGAAGCTGAAGGACGGCGGCCACTA**

**CACCTCCGAGGTCAAGACCACCTACAAGGCCAAGAAGCCCGTGCAGCTGCCCGGCGCCTACATCGTCGGC**

**ATCAAGTTGGACATCACCTCCCACAACGAGGACTACACCATCGTGGAACAGTACGAACGCGCCGAGGGCC**

**GCCACTCCACCGGCGGCATGGACGAGCTGTACAAGATGGCAATTACCTTATCCGCAACTTCTTTACCTAT**

**TTCCGCCCGGATCCGGGCAGGTTCTCCGGCCGCTTGGGTGGAGAGGCTATTCGGCTATGACTGGGCACAA**

**CAGACAATCGGCTGCTCTGATGCCGCCGTGTTCCGGCTGTCAGCGCAGGGGCGCCCGGTTCTTTTTGTCA**

**AGACCGACCTGTCCGGTGCCCTGAATGAACTGCAGGACGAGGCAGCGCGGCTATCGTGGCTGGCCACGAC**

**GGGCGTTCCTTGCGCAGCTGTGCTCGACGTTGTCACTGAAGCGGGAAGGGACTGGCTGCTATTGGGCGAA**

**GTGCCGGGGCAGGATCTCCTGTCATCTCACCTTGCTCCTGCCGAGAAAGTATCCATCATGGCTGATGCAA**

**TGCGGCGGCTGCATACGCTTGATCCGGCTACCTGCCCATTCGACCACCAAGCGAAACATCGCATCGAGCG**

**AGCACGTACTCGGATGGAAGCCGGTCTTGTCGATCAGGATGATCTGGACGAAGAGCATCAGGGGCTCGCG**

**CCAGCCGAACTGTTCGCCAGGCTCAAGGCGCGCATGCCCGACGGCGAGGATCTCGTCGTGACCCATGGCG**

**ATGCCTGCTTGCCGAATATCATGGTGGAAAATGGCCGCTTTTCTGGATTCATCGACTGTGGCCGGCTGGG**

**TGTGGCGGACCGCTATCAGGACATAGCGTTGGCTACCCGTGATATTGCTGAAGAGCTTGGCGGCGAATGG**

**GCTGACCGCTTCCTCGTGCTTTACGGTATCGCCGCTCCCGATTCGCAGCGCATCGCCTTCTATCGCCTTC**

**TTGACGAGTTCTTCTGAGCGGGACTCTGGGGTTCGAAATGACCGACCAAGCGACGCCCAACCTGCCATCA**

**CGAGATTTCGATTCCACCGCCGCCTTCTATGAAAGGTTGGGCTTCGGAATCGTTTTCCGGGACGCCGGCT**

**GGATGATCCTCCAGCGCGGGGATCTCATGCTGGAGTTCTTCGCCCACCCCGATCCAACACTTACGTTTGC**

**AACGTCCAAGAGCAAATAGACCACGAACGCCGGAAGGTTGCCGCAGCGTGTGGATTGCGTCTCAATTCTC**

**TCTTGCAGGAATGCAATGATGAATATGATACTGACTATGAAACTTTGAGGGAATACTGCCTAGCACCGTC**

**ACCTCATAACGTGCATCATGCATGCCCTGACAACATGGAACATCGCTATTTTTCTGAAGAATTATGCTCG**

**TTGGAGGATGTCGCGGCAATTGCAGCTATTGCCAACATCGAACTACCCCTCACGCATGCATTCATCAATA**

**TTATTCATGCGGGGAAAGGCAAGATTAATCCAACTGGCAAATCATCCAGCGTGATTGGTAACTTCAGTTC**

**CAGCGACTTGATTCGTTTTGGTGCTACCCACGTTTTCAATAAGGACGAGATGGTGGAGTAAAGAAGGAGT**

**GCGTCGAAGCAGATCGTTCAAACATTTGGCAATAAAGTTTCTTAAGATTGAATCCTGTTGCCGGTCTTGC**

**GATGATTATCATATAATTTCTGTTGAATTACGTTAAGCATGTAATAATTAACATGTAATGCATGACGTTA**

**TTTATGAGATGGGTTTTTATGATTAGAGTCCCGCAATTATACATTTAATACGCGATAGAAAACAAAATAT**

**AGCGCGCAAACTAGGATAAATTATCGCGCGCGGTGTCATCTATGTTACTAGATCGAATTAATTCCAGGCG**

**GTGAAGGGCAATCAGCTGTTGCCCGTCTCACTGGTGAAAAGAAAAACCACCCCAGTACATTAAAAACGTC**

**CGCAATGTGTTATTAAGTTGTCTAAGCGTCAATTTGTTTACACCACAATATATCCTGCCACCAGCCAGCC**

**AACAGCTCCCCGACCGGCAGCTCGGCACAAAATCACCACTCGATACAGGCAGCCCATCAGTCCGGGACGG**

**CGTCAGCGGGAGAGCCGTTGTAAGGCGGCAGACTTTGCTCATGTTACCGATGCTATTCGGAAGAACGGCA**

**ACTAAGCTGCCGGGTTTGAAACACGGATGATCTCGCGGAGGGTAGCATGTTGATTGTAACGATGACAGAG**

**CGTTGCTGCCTGTGATCAAATATCATCTCCCTCGCAGAGATCCGAATTATCAGCCTTCTTATTCATTTCT**

**CGCTTAACCGTGACAGGCTGTCGATCTTGAGAACTATGCCGACATAATAGGAAATCGCTGGATAAAGCCG**

**CTGAGGAAGCTGAGTGGCGCTATTTCTTTAGAAGTGAACGTTGACGATGTCGACGGATCTTTTCCGCTGC**

**ATAACCCTGCTTCGGGGTCATTATAGCGATTTTTTCGGTATATCCATCCTTTTTCGCACGATATACAGGA**

**TTTTGCCAAAGGGTTCGTGTAGACTTTCCTTGGTGTATCCAACGGCGTCAGCCGGGCAGGATAGGTGAAG**

**TAGGCCCACCCGCGAGCGGGTGTTCCTTCTTCACTGTCCCTTATTCGCACCTGGCGGTGCTCAACGGGAA**

**TCCTGCTCTGCGAGGCTGGCCGGCTACCGCCGGCGTAACAGATGAGGGCAAGCGGATGGCTGATGAAACC**

**AAGCCAACCAGGGGTGATGCTGCCAACTTACTGATTTAGTGTATGATGGTGTTTTTGAGGTGCTCCAGTG**

**GCTTCTGTTTCTATCAGCTGTCCCTCCTGTTCAGCTACTGACGGGGTGGTGCGTAACGGCAAAAGCACCG**

**CCGGACATCAGCGCTATCTCTGCTCTCACTGCCGTAAAACATGGCAACTGCAGTTCACTTACACCGCTTC**

**TCAACCCGGTACGCACCAGAAAATCATTGATATGGCCATGAATGGCGTTGGATGCCGGGCAACAGCCCGC**

**ATTATGGGCGTTGGCCTCAACACGATTTTACGTCACTTAAAAAACTCAGGCCGCAGTCGGTAACCTCGCG**

**CATACAGCCGGGCAGTGACGTCATCGTCTGCGCGGAAATGGACGAACAGTGGGGCTATGTCGGGGCTAAA**

**TCGCGCCAGCGCTGGCTGTTTTACGCGTATGACAGTCTCCGGAAGACGGTTGTTGCGCACGTATTCGGTG**

**AACGCACTATGGCGACGCTGGGGCGTCTTATGAGCCTGCTGTCACCCTTTGACGTGGTGATATGGATGAC**

**GGATGGCTGGCCGCTGTATGAATCCCGCCTGAAGGGAAAGCTGCACGTAATCAGCAAGCGATATACGCAG**

**CGAATTGAGCGGCATAACCTGAATCTGAGGCAGCACCTGGCACGGCTGGGACGGAAGTCGCTGTCGTTCT**

**CAAAATCGGTGGAGCTGCATGACAAAGTCATCGGGCATTATCTGAACATAAAACACTATCAATAAGTTGG**

**AGTCATTACCCAACCAGGAAGGGCAGCCCACCTATCAAGGTGTACTGCCTTCCAGACGAACGAAGAGCGA**

**TTGAGGAAAAGGCGGCGGCGGCCGGCATGAGCCTGTCGGCCTACCTGCTGGCCGTCGGCCAGGGCTACAA**

**AATCACGGGCGTCGTGGACTATGAGCACGTCCGCGAGCTGGCCCGCATCAATGGCGACCTGGGCCGCCTG**

**GGCGGCCTGCTGAAACTCTGGCTCACCGACGACCCGCGCACGGCGCGGTTCGGTGATGCCACGATCCTCG**

**CCCTGCTGGCGAAGATCGAAGAGAAGCAGGACGAGCTTGGCAAGGTCATGATGGGCGTGGTCCGCCCGAG**

**GGCAGAGCCATGACTTTTTTAGCCGCTAAAACGGCCGGGGGGTGCGCGTGATTGCCAAGCACGTCCCCAT**

**GCGCTCCATCAAGAAGAGCGACTTCGCGGAGCTGGTATTCGTGCAGGGCAAGATTCGGAATACCAAGTAC**

**GAGAAGGACGGCCAGACGGTCTACGGGACCGACTTCATTGCCGATAAGGTGGATTATCTGGACACCAAGG**

**CACCAGGCGGGTCAAATCAGGAATAAGGGCACATTGCCCCGGCGTGAGTCGGGGCAATCCCGCAAGGAGG**

**GTGAATGAATCGGACGTTTGACCGGAAGGCATACAGGCAAGAACTGATCGACGCGGGGTTTTCCGCCGAG**

**GATGCCGAAACCATCGCAAGCCGCACCGTCATGCGTGCGCCCCGCGAAACCTTCCAGTCCGTCGGCTCGA**

**TGGTCCAGCAAGCTACGGCCAAGATCGAGCGCGACAGCGTGCAACTGGCTCCCCCTGCCCTGCCCGCGCC**

**ATCGGCCGCCGTGGAGCGTTCGCGTCGTCTCGAACAGGAGGCGGCAGGTTTGGCGAAGTCGATGACCATC**

**GACACGCGAGGAACTATGACGACCAAGAAGCGAAAAACCGCCGGCGAGGACCTGGCAAAACAGGTCAGCG**

**AGGCCAAGCAGGCCGCGTTGCTGAAACACACGAAGCAGCAGATCAAGGAAATGCAGCTTTCCTTGTTCGA**

**TATTGCGCCGTGGCCGGACACGATGCGAGCGATGCCAAACGACACGGCCCGCTCTGCCCTGTTCACCACG**

**CGCAACAAGAAAATCCCGCGCGAGGCGCTGCAAAACAAGGTCATTTTCCACGTCAACAAGGACGTGAAGA**

**TCACCTACACCGGCGTCGAGCTGCGGGCCGACGATGACGAACTGGTGTGGCAGCAGGTGTTGGAGTACGC**

**GAAGCGCACCCCTATCGGCGAGCCGATCACCTTCACGTTCTACGAGCTTTGCCAGGACCTGGGCTGGTCG**

**ATCAATGGCCGGTATTACACGAAGGCCGAGGAATGCCTGTCGCGCCTACAGGCGACGGCGATGGGCTTCA**

**CGTCCGACCGCGTTGGGCACCTGGAATCGGTGTCGCTGCTGCACCGCTTCCGCGTCCTGGACCGTGGCAA**

**GAAAACGTCCCGTTGCCAGGTCCTGATCGACGAGGAAATCGTCGTGCTGTTTGCTGGCGACCACTACACG**

**AAATTCATATGGGAGAAGTACCGCAAGCTGTCGCCGACGGCCCGACGGATGTTCGACTATTTCAGCTCGC**

**ACCGGGAGCCGTACCCGCTCAAGCTGGAAACCTTCCGCCTCATGTGCGGATCGGATTCCACCCGCGTGAA**

**GAAGTGGCGCGAGCAGGTCGGCGAAGCCTGCGAAGAGTTGCGAGGCAGCGGCCTGGTGGAACACGCCTGG**

**GTCAATGATGACCTGGTGCATTGCAAACGCTAGGGCCTTGTGGGGTCAGTTCCGGCTGGGGGTTCAGCAG**

**CCAGCGCTTTACTGGCATTTCAGGAACAAGCGGGCACTGCTCGACGCACTTGCTTCGCTCAGTATCGCTC**

**GGGACGCACGGCGCGCTCTACGAACTGCCGATAAACAGAGGATTAAAATTGACAATTGTGATTAAGGCTC**

**AGATTCGACGGCTTGGAGCGGCCGACGTGCAGGATTTCCGCGAGATCCGATTGTCGGCCCTGAAGAAAGC**

**TCCAGAGATGTTCGGGTCCGTTTACGAGCACGAGGAGAAAAAGCCCATGGAGGCGTTCGCTGAACGGTTG**

**CGAGATGCCGTGGCATTCGGCGCCTACATCGACGGCGAGATCATTGGGCTGTCGGTCTTCAAACAGGAGG**

**ACGGCCCCAAGGACGCTCACAAGGCGCATCTGTCCGGCGTTTTCGTGGAGCCCGAACAGCGAGGCCGAGG**

**GGTCGCCGGTATGCTGCTGCGGGCGTTGCCGGCGGGTTTATTGCTCGTGATGATCGTCCGACAGATTCCA**

**ACGGGAATCTGGTGGATGCGCATCTTCATCCTCGGCGCACTTAATATTTCGCTATTCTGGAGCTTGTTGT**

**TTATTTCGGTCTACCGCCTGCCGGGCGGGGTCGCGGCGACGGTAGGCGCTGTGCAGCCGCTGATGGTCGT**

**GTTCATCTCTGCCGCTCTGCTAGGTAGCCCGATACGATTGATGGCGGTCCTGGGGGCTATTTGCGGAACT**

**GCGGGCGTGGCGCTGTTGGTGTTGACACCAAACGCAGCGCTAGATCCTGTCGGCGTCGCAGCGGGCCTGG**

**CGGGGGCGGTTTCCATGGCGTTCGGAACCGTGCTGACCCGCAAGTGGCAACCTCCCGTGCCTCTGCTCAC**

**CTTTACCGCCTGGCAACTGGCGGCCGGAGGACTTCTGCTCGTTCCAGTAGCTTTAGTGTTTGATCCGCCA**

**ATCCCGATGCCTACAGGAACCAATGTTCTCGGCCTGGCGTGGCTCGGCCTGATCGGAGCGGGTTTAACCT**

**ACTTCCTTTGGTTCCGGGGGATCTCGCGACTCGAACCTACAGTTGTTTCCTTACTGGGCTTTCTCAGCCG**

**GGATGGCGCTAAGAAGCTATTGCCGCCGATCTTCATATGCGGTGTGAAATACCGCACAGATGCGTAAGGA**

**GAAAATACCGCATCAGGCGCTCTTCCGCTTCCTCGCTCACTGACTCGCTGCGCTCGGTCGTTCGGCTGCG**

**GCGAGCGGTATCAGCTCACTCAAAGGCGGTAATACGGTTATCCACAGAATCAGGGGATAACGCAGGAAAG**

**AACATGTGAGCAAAAGGCCAGCAAAAGGCCAGGAACCGTAAAAAGGCCGCGTTGCTGGCGTTTTTCCATA**

**GGCTCCGCCCCCCTGACGAGCATCACAAAAATCGACGCTCAAGTCAGAGGTGGCGAAACCCGACAGGACT**

**ATAAAGATACCAGGCGTTTCCCCCTGGAAGCTCCCTCGTGCGCTCTCCTGTTCCGACCCTGCCGCTTACC**

**GGATACCTGTCCGCCTTTCTCCCTTCGGGAAGCGTGGCGCTTTCTCAATGCTCACGCTGTAGGTATCTCA**

**GTTCGGTGTAGGTCGTTCGCTCCAAGCTGGGCTGTGTGCACGAACCCCCCGTTCAGCCCGACCGCTGCGC**

**CTTATCCGGTAACTATCGTCTTGAGTCCAACCCGGTAAGACACGACTTATCGCCACTGGCAGCAGCCACT**

**GGTAACAGGATTAGCAGAGCGAGGTATGTAGGCGGTGCTACAGAGTTCTTGAAGTGGTGGCCTAACTACG**

**GCTACACTAGAAGGACAGTATTTGGTATCTGCGCTCTGCTGAAGCCAGTTACCTTCGGAAAAAGAGTTGG**

**TAGCTCTTGATCCGGCAAACAAACCACCGCTGGTAGCGGTGGTTTTTTTGTTTGCAAGCAGCAGATTACG**

**CGCAGAAAAAAAGGATATCAAGAAGATCCTTTGATCTTTTCTACGGGGTCTGACGCTCAGTGGAACGAAA**

**ACTCACGTTAAGGGATTTTGGTCATGAGATTATCAAAAAGGATCTTCACCTAGATCCTTTTAAATTAAAA**

**ATGAAGTTTTAAATCAATCTAAAGTATATATGAGTAAACTTGGTCTGACAGTTACCAATGCTTAATCAGT**

**GAGGCACCTATCTCAGCGATCTGTCTATTTCGTTCATCCATAGTTGCCTGACTCCCCGTCGTGTAGATAA**

**CTACGATACGGGAGGGCTTACCATCTGGCCCCAGTGCTGCAATGATACCGCGAGACCCACGCTCACCGGC**

**TCCAGATTTATCAGCAATAAACCAGCCAGCCGGAAGGGCCGAGCGCAGAAGTGGTCCTGCAACTTTATCC**

**GCCTCCATCCAGTCTATTAAACAAGTGGCAGCAACGGATTCGCAAACCTGTCACGCCTTTTGTGCCAAAA**

**GCCGCGCCAGGTTTGCGATCCGCTGTGCCAGGCGTTAGGCGTCATATGAAGATTTCGGTGATCCCTGAGC**

**AGGTGGCGGAAACATTGGATGCTGAGAACCATTTCATTGTTCGTGAAGTGTTCGATGTGCACCTATCCGA**

**CCAAGGCTTTGAACTATCTACCAGAAGTGTGAGCCCCTACCGGAAGGATTACATCTCGGATGATGACTCT**

**GATGAAGACTCTGCTTGCTATGGCGCATTCATCGACCAAGAGCTTGTCGGGAAGATTGAACTCAACTCAA**

**CATGGAACGATCTAGCCTCTATCGAACACATTGTTGTGTCGCACACGCACCGAGGCAAAGGAGTCGCGCA**

**CAGTCTCATCGAATTTGCGAAAAAGTGGGCACTAAGCAGACAGCTCCTTGGCATACGATTAGAGACACAA**

**ACGAACAATGTACCTGCCTGCAATTTGTACGCAAAATGTGGCTTTACTCTCGGCGGCATTGACCTGTTCA**

**CGTATAAAACTAGACCTCAAGTCTCGAACGAAACAGCGATGTACTGGTACTGGTTCTCGGGAGCACAGGA**

**TGACGCCTAACAATTCATTCAAGCCGACACCGCTTCGCGGCGCGGCTTAATTCAGGAGTTAAACATCATG**

**AGGGAAGCGGTGATCGCCGAAGTATCGACTCAACTATCAGAGGTAGTTGGCGTCATCGAGCGCCATCTCG**

**AACCGACGTTGCTGGCCGTACATTTGTACGGCTCCGCAGTGGATGGCGGCCTGAAGCCACACAGTGATAT**

**TGATTTGCTGGTTACGGTGACCGTAAGGCTTGATGAAACAACGCGGCGAGCTTTGATCAACGACCTTTTG**

**GAAACTTCGGCTTCCCCTGGAGAGAGCGAGATTCTCCGCGCTGTAGAAGTCACCATTGTTGTGCACGACG**

**ACATCATTCCGTGGCGTTATCCAGCTAAGCGCGAACTGCAATTTGGAGAATGGCAGCGCAATGACATTCT**

**TGCAGGTATCTTCGAGCCAGCCACGATCGACATTGATCTGGCTATCTTGCTGACAAAAGCAAGAGAACAT**

**AGCGTTGCCTTGGTAGGTCCAGCGGCGGAGGAACTCTTTGATCCGGTTCCTGAACAGGATCTATTTGAGG**

**CGCTAAATGAAACCTTAACGCTATGGAACTCGCCGCCCGACTGGGCTGGCGATGAGCGAAATGTAGTGCT**

**TACGTTGTCCCGCATTTGGTACAGCGCAGTAACCGGCAAAATCGCGCCGAAGGATGTCGCTGCCGACTGG**

**GCAATGGAGCGCCTGCCGGCCCAGTATCAGCCCGTCATACTTGAAGCTAGGCAGGCTTATCTTGGACAAG**

**AAGATCGCTTGGCCTCGCGCGCAGATCAGTTGGAAGAATTTGTTCACTACGTGAAAGGCGAGATCACCAA**

**GGTAGTCGGCAAATAATGTCTAACAATTCGTTCAAGCCGACGCCGCTTCGCGGCGCGGCTTAACTCAAGC**

**GTTAGAGAGCTGGGGAAGACTATGCGCGATCTGTTGAAGGTGGTTCTAAGCCTCGTACTTGCGATGGCAT**

**CGGGGCAGGCACTTGCTGACCTGCCAATTGTTTTAGTGGATGAAGCTCGTCTTCCCTATGACTACTCCCC**

**ATCCAACTACGACATTTCTCCAAGCAACTACGACAACTCCATAAGCAATTACGACAATAGTCCATCAAAT**

**TACGACAACTCTGAGAGCAACTACGATAATAGTTCATCCAATTACGACAATAGTCGCAACGGAAATCGTA**

**GGCTTATATATAGCGCAAATGGGTCTCGCACTTTCGCCGGCTACTACGTCATTGCCAACAATGGGACAAC**

**GAACTTCTTTTCCACATCTGGCAAAAGGATGTTCTACACCCCAAAAGGGGGGCGCGGCGTCTATGGCGGC**

**AAAGATGGGAGCTTCTGCGGGGCATTGGTCGTCATAAATGGCCAATTTTCGCTTGCCCTGACAGATAACG**

**GCCTGAAGATCATGTATCTAAGCAACTAGCCTGCTCTCTAATAAAATGTTAGGAGCTTGGCTGCCATTTT**

**TGGGGTGAGGCCGTTCGCGGCCGAGGGGCGCAGCCCCTGGGGGGATGGGAGGCCCGCGTTAGCGGGCCGG**

**GAGGGTTCGAGAAGGGGGGGCACCCCCCTTCGGCGTGCGCGGTCACGCGCCAGGGCGCAGCCCTGGTTAA**

**AAACAAGGTTTATAAATATTGGTTTAAAAGCAGGTTAAAAGACAGGTTAGCGGTGGCCGAAAAACGGGCG**

**GAAACCCTTGCAAATGCTGGATTTTCTGCCTGTGGACAGCCCCTCAAATGTCAATAGGTGCGCCCCTCAT**

**CTGTCAGCACTCTGCCCCTCAAGTGTCAAGGATCGCGCCCCTCATCTGTCAGTAGTCGCGCCCCTCAAGT**

**GTCAATACCGCAGGGCACTTATCCCCAGGCTTGTCCACATCATCTGTGGGAAACTCGCGTAAAATCAGGC**

**GTTTTCGCCGATTTGCGAGGCTGGCCAGCTCCACGTCGCCGGCCGAAATCGAGCCTGCCCCTCATCTGTC**

**AACGCCGCGCCGGGTGAGTCGGCCCCTCAAGTGTCAACGTCCGCCCCTCATCTGTCAGTGAGGGCCAAGT**

**TTTCCGCGAGGTATCCACAACGCCGGCGGCCGGCCGCGGTGTCTCGCACACGGCTTCGACGGCGTTTCTG**

**GCGCGTTTGCAGGGCCATAGACGGCCGCCAGCCCAGCGGCGAGGGCAACCAGCCCGGTGAGCGTCGGAAA**

**GGGTCGACATCTTGCTGCGTTCGGATATTTTCGTGGAGTTCCCGCCACAGACCCGGATTGAAGGCGAGAT**

**CCAGCAACTCGCGCCAGATCATCCTGTGACGGAACTTTGGCGCGTGATGACTGGCCAGGACGTCGGCCGA**

**AAGAGCGACAAGCAGATCACGATTTTCGACAGCGTCGGATTTGCGATCGAGGATTTTTCGGCGCTGCGCT**

**ACGTCCGCGACCGCGTTGAGGGATCAAGCCACAGCAGCCCACTCGACCTTCTAGCCGACCCAGACGAGCC**

**AAGGGATCTTTTTGGAATGCTGCTCCGTCGTCAGGCTTTCCGACGTTTGGGTGGTTGAACAGAAGTCATT**

**ATCGTACGGAATGCCAGCACTCCCGAGGGGAACCCTGTGGTTGGCATGCACATACAAATGGACGAACGGA**

**TAAACCTTTTCACGCCCTTTTAAATATCCGTTATTCTAATAAACGCTCTTTTCTCTTAGGTTTACCCGCC**

**AATATATCCTGTCAAACACTGATAGTTTAAACTGAAGGCGGGAAACGACAATCTGATCATGAGCGGAGAA**

**TTAAGGGAGTCACGTTATGACCCCCGCCGATGACGCGGGACAAGCCGTTTTACGTTTGGAACTGACAGAA**

**CCGCAACGATTGAAGGAGCCACTCAGCCCCAATACGCAAACCGCCTCTCCCCGCGCGTTGGCCGATTCAT**

**TAATGCAGCTGGCACGACAGGTTTCCCGACTGGAAAGCGGGCAGTGAGCGCAACGCAATTAATGTGAGTT**

**AGCTCACTCATTAGGCACCCCAGGCTTTACACTTTATGCTTCCGGCTCGTATGTTGTGTGGAATTGTGAG**

**CGGATAACAATTTCACACAGGAAACAGCTATGACCATGATTACGCCAAGCTATTTAGGTGACACTATAGA**

**ATACTCAAGCTATGCATCCAACGCGTTGGGAGCTCTCCCATATCGACCTGCAGGCGGCCGCTCGACGAAT**

**TAATTCCAATCCCACAAAAATCTGAGCTTAACAGCACAGTTGCTCCTCTCAGAGCAGAATCGGGTATTCA**

**ACACCCTCATATCAACTACTACGTTGTGTATAACGGTCCACATGCCGGTATATACGATGACTGGGGTTGT**

**ACAAAGGCGGCAACAAACGGCGTTCCCGGAGTTGCACACAAGAAATTTGCCACTATTACAGAGGCAAGAG**

**CAGCAGCTGACGCGTACACAACAAGTCAGCAAACAGACAGGTTGAACTTCATCCCCAAAGGAGAAGCTCA**

**ACTCAAGCCCAAGAGCTTTGCTAAGGCCCTAACAAGCCCACCAAAGCAAAAAGCCCACTGGCTCACGCTA**

**GGAACCAAAAGGCCCAGCAGTGATCCAGCCCCAAAAGAGATCTCCTTTGCCCCGGAGATTACAATGGACG**

**ATTTCCTCTATCTTTACGATCTAGGAAGGAAGTTCGAAGGTGAAGGTGACGACACTATGTTCACCACTGA**

**TAATGAGAAGGTTAGCCTCTTCAATTTCAGAAAGAATGCTGACCCACAGATGGTTAGAGAGGCCTACGCA**

**GCAGGTCTCATCAAGACGATCTACCCGAGTAACAATCTCCAGGAGATCAAATACCTTCCCAAGAAGGTTA**

**AAGATGCAGTCAAAAGATTCAGGACTAATTGCATCAAGAACACAGAGAAAGACATATTTCTCAAGATCAG**

**AAGTACTATTCCAGTATGGACGATTCAAGGCTTGCTTCATAAACCAAGGCAAGTAATAGAGATTGGAGTC**

**TCTAAAAAGGTAGTTCCTACTGAATCTAAGGCCATGCATGGAGTCTAAGATTCAAATCGAGGATCTAACA**

**GAACTCGCCGTGAAGACTGGCGAACAGTTCATACAGAGTCTTTTACGACTCAATGACAAGAAGAAAATCT**

**TCGTCAACATGGTGGAGCACGACACTCTGGTCTACTCCAAAAATGTCAAAGATACAGTCTCAGAAGACCA**

**AAGGGCTATTGAGACTTTTCAACAAAGGATAATTTCGGGAAACCTCCTCGGATTCCATTGCCCAGCTATC**

**TGTCACTTCATCGAAAGGACAGTAGAAAAGGAAGGTGGCTCCTACAAATGCCATCATTGCGATAAAGGAA**

**AGGCTATCATTCAAGATCTCTCTGCCGACAGTGGTCCCAAAGATGGACCCCCACCCACGAGGAGCATCGT**

**GGAAAAAGAAGACGTTCCAACCACGTCTTCAAAGCAAGTGGATTGATGTGACATCTCCACTGACGTAAGG**

**GATGACGCACAATCCCACTATCCTTCGCAAGACCCTTCCTCTATATAAGGAAGTTCATTTCATTTGGAGA**

**GGACACGCTCGACTATTTTTACAACAATTACCATGAAGACTAATCTTTTTCTCTTTCTCATCTTTTCACT**

**TCTCCTATCATTATCCTCGGCCGAATTCCTCGAGGCGAAGATACTCGAAAAACGTTTATCTAGAAGTAAA**

**GGAGAAGAACTTTTCACTGGAGTTGTCCCAATTCTTGTTGAATTAGATGGTGATGTTAATGGGTACAAAT**

**TTTCTGTCAGTGGAGAGGGTGAAGGTGATGCAACATACGGAAAACTTACCCTTAAATTTATTTGCACTAC**

**TGGAAAACTACCTGTTCCATGGCCAACACTTGTCACTACTTTCTCTTATGGTGTTCAATGCTTTTCAAGA**

**TACCCAGATCATATGAAGCGGCACGACTTCTTCAAGAGCGCCATGCCTGAGGGATACGTGCAGGAGAGGA**

**CCATCTTCTTCAAGGACGACGGGAACTACAAGACACGTGCTGAAGTCAAGTTTGAGGGAGACACCCTCGT**

**CAACAGGATCGAGCTTAAGGGAATCGATTTCAAGGAGGACGGAAACATCCTCGGCCACAAGTTGGAATAC**

**AACTACAACTCCCACAACGTATACATCATGGCCGACAAGCAAAAGAACGGCATCAAAGCCAACTTCAAGA**

**CCCGCCACAACATCGAAGACGGCGGCGTGCAACTCGCTGATCATTATCAACAAAATACTCCAATTGGCGA**

**TGGCCCTGTCCTTTTACCAGACAACCATTACCTGTCCACACAATCTGCCCTTTCGAAAGATCCCAACGAA**

**AAGAGAGACCACATGGTCCTTCTTGAGTTTGTAACAGCTGCTGGGATTACACATGGCATGGATGAACTAT**

**ACAAACATGATGAGCTTTGAACTAGAGTCCTGCTTTAATGAGATATGCGAGACGCCTATGATCGCATGAT**

**ATTTGCTTTCAATTCTGTTGTGCACGTTGTAAAAAACCTGAGCATGTGTAGCTCAGATCCTTACCGCCGG**

**TTTCGGTTCATTCTAATGAATATATCACCCGTTACTATCGTATTTTTATGAATAATATTCTCCGTTCAAT**

**TTACTGATTGTACCCTACTACTTATATGTACAATATTAAAATGAAAACAATATATTGTGCTGAATAGGTT**

**TATAGCGACATCTATGATAGAGCGCCACAATAACAAACAATTGCGTTTTATTATTACAAATCCAATTTTA**

**AAAAAAGCGGCAGAACCGGTCAAACCTAAAAGACTGATTACATAAATCTTATTCAAATTTCAAAAGGCCC**

**CAGGGGCTAGTATCTACGACACACCGAGCGGCGAACTAATAACGTTCACTGAAGGGAACTCCGGTTCCCC**

**GCCGGCGCGCATGGGTGAGATTCCTTGAAGTTGAGTATTGGCCGTCCGCTCTACCGAAAGTTACGGGCAC**

**CATTCAACCCGGTCCAGCACGGCGGCCGGGTAACCGACTTGCTGCCCCGAGAATTATGCAGCATTTTTTT**

**GGTGTATGTGGGCCCCAAATGAAGTGCAGGTCAAACCTTGACAGTGACGACAAATCGTTGGGCGGGTCCA**

**GGGCGAATTTTGCGACAACATGTCGAGGCTCAGCAGGACCTGCAGGCATGCAAGCTAGCTTACTAGTGAT**

**GCATATTCTATAGTGTCACCTAAATCTGC**

**>**MS4miR6020^E^ **(1bp - 15359bp, direct) 15359bp**

**GGCCGCACTAGTGATATCCCGCGGCCATGGCGGCCGGGAGCATGCGACGTCGGGCCCAATTCGCCCTATA**

**GTGAGTCGTATTACAATTCACTGGCCGTCGTTTTACAACGTCGTGACTGGGAAAACCCTGGCGTTACCCA**

**ACTTAATCGCCTTGCAGCACATCCCCCTTTCGCCAGCTGGCGTAATAGCGAAGAGGCCCGCACCGATCGC**

**CCTTCCCAACAGTTGCGCAGCCTGAATGGCGAATGGAAATTGTAAACGTTAATGGGTTTCTGGAGTTTAA**

**TGAGCTAAGCACATACGTCAGAAACCATTATTGCGCGTTCAAAAGTCGCCTAAGGTCACTATCAGCTAGC**

**AAATATTTCTTGTCAAAAATGCTCCACTGACGTTCCATAAATTCCCCTCGGTATCCAATTAGAGTCTCAT**

**ATTCACTCTCAATCCAAATAATCTGCAATGGTGAGCAAGGGCGAGGAGAATAACATGGCCATCATCAAGG**

**AGTTCATGCGCTTCAAGGTGCGCATGGAGGGCTCCGTGAACGGCCACGAGTTCGAGATCGAGGGCGAGGG**

**CGAGGGCCGCCCCTACGAGGGCTTTCAGACCGCTAAGCTGAAGGTGACCAAGGGTGGCCCCCTGCCCTTC**

**GCCTGGGACATCCTGTCCCCTCAGTTCACCTACGGCTCCAAGGCCTACGTGAAGCACCCCGCCGACATCC**

**CCGACTACTTCAAGCTGTCCTTCCCCGAGGGCTTCAAGTGGGAGCGCGTGATGAACTTCGAGGACGGCGG**

**CGTGGTGACCGTGACCCAGGACTCCTCCCTGCAGGACGGCGAGTTCATCTACAAGGTGAAGCTGCGCGGC**

**ACCAACTTCCCCTCCGACGGCCCCGTAATGCAGAAGAAGACCATGGGCTGGGAGGCCTCCTCCGAGCGGA**

**TGTACCCCGAGGACGGCGCCCTGAAGGGCGAGATCAAGATGAGGCTGAAGCTGAAGGACGGCGGCCACTA**

**CACCTCCGAGGTCAAGACCACCTACAAGGCCAAGAAGCCCGTGCAGCTGCCCGGCGCCTACATCGTCGGC**

**ATCAAGTTGGACATCACCTCCCACAACGAGGACTACACCATCGTGGAACAGTACGAACGCGCCGAGGGCC**

**GCCACTCCACCGGCGGCATGGACGAGCTGTACAAGATGGCAATTACCTTATCCGCAACTTCTTTACCTAT**

**TTCCGCCCGGATCCGGGCAGGTTCTCCGGCCGCTTGGGTGGAGAGGCTATTCGGCTATGACTGGGCACAA**

**CAGACAATCGGCTGCTCTGATGCCGCCGTGTTCCGGCTGTCAGCGCAGGGGCGCCCGGTTCTTTTTGTCA**

**AGACCGACCTGTCCGGTGCCCTGAATGAACTGCAGGACGAGGCAGCGCGGCTATCGTGGCTGGCCACGAC**

**GGGCGTTCCTTGCGCAGCTGTGCTCGACGTTGTCACTGAAGCGGGAAGGGACTGGCTGCTATTGGGCGAA**

**GTGCCGGGGCAGGATCTCCTGTCATCTCACCTTGCTCCTGCCGAGAAAGTATCCATCATGGCTGATGCAA**

**TGCGGCGGCTGCATACGCTTGATCCGGCTACCTGCCCATTCGACCACCAAGCGAAACATCGCATCGAGCG**

**AGCACGTACTCGGATGGAAGCCGGTCTTGTCGATCAGGATGATCTGGACGAAGAGCATCAGGGGCTCGCG**

**CCAGCCGAACTGTTCGCCAGGCTCAAGGCGCGCATGCCCGACGGCGAGGATCTCGTCGTGACCCATGGCG**

**ATGCCTGCTTGCCGAATATCATGGTGGAAAATGGCCGCTTTTCTGGATTCATCGACTGTGGCCGGCTGGG**

**TGTGGCGGACCGCTATCAGGACATAGCGTTGGCTACCCGTGATATTGCTGAAGAGCTTGGCGGCGAATGG**

**GCTGACCGCTTCCTCGTGCTTTACGGTATCGCCGCTCCCGATTCGCAGCGCATCGCCTTCTATCGCCTTC**

**TTGACGAGTTCTTCTGAGCGGGACTCTGGGGTTCGAAATGACCGACCAAGCGACGCCCAACCTGCCATCA**

**CGAGATTTCGATTCCACCGCCGCCTTCTATGAAAGGTTGGGCTTCGGAATCGTTTTCCGGGACGCCGGCT**

**GGATGATCCTCCAGCGCGGGGATCTCATGCTGGAGTTCTTCGCCCACCCCGATCCAACACTTACGTTTGC**

**AACGTCCAAGAGCAAATAGACCACGAACGCCGGAAGGTTGCCGCAGCGTGTGGATTGCGTCTCAATTCTC**

**TCTTGCAGGAATGCAATGATGAATATGATACTGACTATGAAACTTTGAGGGAATACTGCCTAGCACCGTC**

**ACCTCATAACGTGCATCATGCATGCCCTGACAACATGGAACATCGCTATTTTTCTGAAGAATTATGCTCG**

**TTGGAGGATGTCGCGGCAATTGCAGCTATTGCCAACATCGAACTACCCCTCACGCATGCATTCATCAATA**

**TTATTCATGCGGGGAAAGGCAAGATTAATCCAACTGGCAAATCATCCAGCGTGATTGGTAACTTCAGTTC**

**CAGCGACTTGATTCGTTTTGGTGCTACCCACGTTTTCAATAAGGACGAGATGGTGGAGTAAAGAAGGAGT**

**GCGTCGAAGCAGATCGTTCAAACATTTGGCAATAAAGTTTCTTAAGATTGAATCCTGTTGCCGGTCTTGC**

**GATGATTATCATATAATTTCTGTTGAATTACGTTAAGCATGTAATAATTAACATGTAATGCATGACGTTA**

**TTTATGAGATGGGTTTTTATGATTAGAGTCCCGCAATTATACATTTAATACGCGATAGAAAACAAAATAT**

**AGCGCGCAAACTAGGATAAATTATCGCGCGCGGTGTCATCTATGTTACTAGATCGAATTAATTCCAGGCG**

**GTGAAGGGCAATCAGCTGTTGCCCGTCTCACTGGTGAAAAGAAAAACCACCCCAGTACATTAAAAACGTC**

**CGCAATGTGTTATTAAGTTGTCTAAGCGTCAATTTGTTTACACCACAATATATCCTGCCACCAGCCAGCC**

**AACAGCTCCCCGACCGGCAGCTCGGCACAAAATCACCACTCGATACAGGCAGCCCATCAGTCCGGGACGG**

**CGTCAGCGGGAGAGCCGTTGTAAGGCGGCAGACTTTGCTCATGTTACCGATGCTATTCGGAAGAACGGCA**

**ACTAAGCTGCCGGGTTTGAAACACGGATGATCTCGCGGAGGGTAGCATGTTGATTGTAACGATGACAGAG**

**CGTTGCTGCCTGTGATCAAATATCATCTCCCTCGCAGAGATCCGAATTATCAGCCTTCTTATTCATTTCT**

**CGCTTAACCGTGACAGGCTGTCGATCTTGAGAACTATGCCGACATAATAGGAAATCGCTGGATAAAGCCG**

**CTGAGGAAGCTGAGTGGCGCTATTTCTTTAGAAGTGAACGTTGACGATGTCGACGGATCTTTTCCGCTGC**

**ATAACCCTGCTTCGGGGTCATTATAGCGATTTTTTCGGTATATCCATCCTTTTTCGCACGATATACAGGA**

**TTTTGCCAAAGGGTTCGTGTAGACTTTCCTTGGTGTATCCAACGGCGTCAGCCGGGCAGGATAGGTGAAG**

**TAGGCCCACCCGCGAGCGGGTGTTCCTTCTTCACTGTCCCTTATTCGCACCTGGCGGTGCTCAACGGGAA**

**TCCTGCTCTGCGAGGCTGGCCGGCTACCGCCGGCGTAACAGATGAGGGCAAGCGGATGGCTGATGAAACC**

**AAGCCAACCAGGGGTGATGCTGCCAACTTACTGATTTAGTGTATGATGGTGTTTTTGAGGTGCTCCAGTG**

**GCTTCTGTTTCTATCAGCTGTCCCTCCTGTTCAGCTACTGACGGGGTGGTGCGTAACGGCAAAAGCACCG**

**CCGGACATCAGCGCTATCTCTGCTCTCACTGCCGTAAAACATGGCAACTGCAGTTCACTTACACCGCTTC**

**TCAACCCGGTACGCACCAGAAAATCATTGATATGGCCATGAATGGCGTTGGATGCCGGGCAACAGCCCGC**

**ATTATGGGCGTTGGCCTCAACACGATTTTACGTCACTTAAAAAACTCAGGCCGCAGTCGGTAACCTCGCG**

**CATACAGCCGGGCAGTGACGTCATCGTCTGCGCGGAAATGGACGAACAGTGGGGCTATGTCGGGGCTAAA**

**TCGCGCCAGCGCTGGCTGTTTTACGCGTATGACAGTCTCCGGAAGACGGTTGTTGCGCACGTATTCGGTG**

**AACGCACTATGGCGACGCTGGGGCGTCTTATGAGCCTGCTGTCACCCTTTGACGTGGTGATATGGATGAC**

**GGATGGCTGGCCGCTGTATGAATCCCGCCTGAAGGGAAAGCTGCACGTAATCAGCAAGCGATATACGCAG**

**CGAATTGAGCGGCATAACCTGAATCTGAGGCAGCACCTGGCACGGCTGGGACGGAAGTCGCTGTCGTTCT**

**CAAAATCGGTGGAGCTGCATGACAAAGTCATCGGGCATTATCTGAACATAAAACACTATCAATAAGTTGG**

**AGTCATTACCCAACCAGGAAGGGCAGCCCACCTATCAAGGTGTACTGCCTTCCAGACGAACGAAGAGCGA**

**TTGAGGAAAAGGCGGCGGCGGCCGGCATGAGCCTGTCGGCCTACCTGCTGGCCGTCGGCCAGGGCTACAA**

**AATCACGGGCGTCGTGGACTATGAGCACGTCCGCGAGCTGGCCCGCATCAATGGCGACCTGGGCCGCCTG**

**GGCGGCCTGCTGAAACTCTGGCTCACCGACGACCCGCGCACGGCGCGGTTCGGTGATGCCACGATCCTCG**

**CCCTGCTGGCGAAGATCGAAGAGAAGCAGGACGAGCTTGGCAAGGTCATGATGGGCGTGGTCCGCCCGAG**

**GGCAGAGCCATGACTTTTTTAGCCGCTAAAACGGCCGGGGGGTGCGCGTGATTGCCAAGCACGTCCCCAT**

**GCGCTCCATCAAGAAGAGCGACTTCGCGGAGCTGGTATTCGTGCAGGGCAAGATTCGGAATACCAAGTAC**

**GAGAAGGACGGCCAGACGGTCTACGGGACCGACTTCATTGCCGATAAGGTGGATTATCTGGACACCAAGG**

**CACCAGGCGGGTCAAATCAGGAATAAGGGCACATTGCCCCGGCGTGAGTCGGGGCAATCCCGCAAGGAGG**

**GTGAATGAATCGGACGTTTGACCGGAAGGCATACAGGCAAGAACTGATCGACGCGGGGTTTTCCGCCGAG**

**GATGCCGAAACCATCGCAAGCCGCACCGTCATGCGTGCGCCCCGCGAAACCTTCCAGTCCGTCGGCTCGA**

**TGGTCCAGCAAGCTACGGCCAAGATCGAGCGCGACAGCGTGCAACTGGCTCCCCCTGCCCTGCCCGCGCC**

**ATCGGCCGCCGTGGAGCGTTCGCGTCGTCTCGAACAGGAGGCGGCAGGTTTGGCGAAGTCGATGACCATC**

**GACACGCGAGGAACTATGACGACCAAGAAGCGAAAAACCGCCGGCGAGGACCTGGCAAAACAGGTCAGCG**

**AGGCCAAGCAGGCCGCGTTGCTGAAACACACGAAGCAGCAGATCAAGGAAATGCAGCTTTCCTTGTTCGA**

**TATTGCGCCGTGGCCGGACACGATGCGAGCGATGCCAAACGACACGGCCCGCTCTGCCCTGTTCACCACG**

**CGCAACAAGAAAATCCCGCGCGAGGCGCTGCAAAACAAGGTCATTTTCCACGTCAACAAGGACGTGAAGA**

**TCACCTACACCGGCGTCGAGCTGCGGGCCGACGATGACGAACTGGTGTGGCAGCAGGTGTTGGAGTACGC**

**GAAGCGCACCCCTATCGGCGAGCCGATCACCTTCACGTTCTACGAGCTTTGCCAGGACCTGGGCTGGTCG**

**ATCAATGGCCGGTATTACACGAAGGCCGAGGAATGCCTGTCGCGCCTACAGGCGACGGCGATGGGCTTCA**

**CGTCCGACCGCGTTGGGCACCTGGAATCGGTGTCGCTGCTGCACCGCTTCCGCGTCCTGGACCGTGGCAA**

**GAAAACGTCCCGTTGCCAGGTCCTGATCGACGAGGAAATCGTCGTGCTGTTTGCTGGCGACCACTACACG**

**AAATTCATATGGGAGAAGTACCGCAAGCTGTCGCCGACGGCCCGACGGATGTTCGACTATTTCAGCTCGC**

**ACCGGGAGCCGTACCCGCTCAAGCTGGAAACCTTCCGCCTCATGTGCGGATCGGATTCCACCCGCGTGAA**

**GAAGTGGCGCGAGCAGGTCGGCGAAGCCTGCGAAGAGTTGCGAGGCAGCGGCCTGGTGGAACACGCCTGG**

**GTCAATGATGACCTGGTGCATTGCAAACGCTAGGGCCTTGTGGGGTCAGTTCCGGCTGGGGGTTCAGCAG**

**CCAGCGCTTTACTGGCATTTCAGGAACAAGCGGGCACTGCTCGACGCACTTGCTTCGCTCAGTATCGCTC**

**GGGACGCACGGCGCGCTCTACGAACTGCCGATAAACAGAGGATTAAAATTGACAATTGTGATTAAGGCTC**

**AGATTCGACGGCTTGGAGCGGCCGACGTGCAGGATTTCCGCGAGATCCGATTGTCGGCCCTGAAGAAAGC**

**TCCAGAGATGTTCGGGTCCGTTTACGAGCACGAGGAGAAAAAGCCCATGGAGGCGTTCGCTGAACGGTTG**

**CGAGATGCCGTGGCATTCGGCGCCTACATCGACGGCGAGATCATTGGGCTGTCGGTCTTCAAACAGGAGG**

**ACGGCCCCAAGGACGCTCACAAGGCGCATCTGTCCGGCGTTTTCGTGGAGCCCGAACAGCGAGGCCGAGG**

**GGTCGCCGGTATGCTGCTGCGGGCGTTGCCGGCGGGTTTATTGCTCGTGATGATCGTCCGACAGATTCCA**

**ACGGGAATCTGGTGGATGCGCATCTTCATCCTCGGCGCACTTAATATTTCGCTATTCTGGAGCTTGTTGT**

**TTATTTCGGTCTACCGCCTGCCGGGCGGGGTCGCGGCGACGGTAGGCGCTGTGCAGCCGCTGATGGTCGT**

**GTTCATCTCTGCCGCTCTGCTAGGTAGCCCGATACGATTGATGGCGGTCCTGGGGGCTATTTGCGGAACT**

**GCGGGCGTGGCGCTGTTGGTGTTGACACCAAACGCAGCGCTAGATCCTGTCGGCGTCGCAGCGGGCCTGG**

**CGGGGGCGGTTTCCATGGCGTTCGGAACCGTGCTGACCCGCAAGTGGCAACCTCCCGTGCCTCTGCTCAC**

**CTTTACCGCCTGGCAACTGGCGGCCGGAGGACTTCTGCTCGTTCCAGTAGCTTTAGTGTTTGATCCGCCA**

**ATCCCGATGCCTACAGGAACCAATGTTCTCGGCCTGGCGTGGCTCGGCCTGATCGGAGCGGGTTTAACCT**

**ACTTCCTTTGGTTCCGGGGGATCTCGCGACTCGAACCTACAGTTGTTTCCTTACTGGGCTTTCTCAGCCG**

**GGATGGCGCTAAGAAGCTATTGCCGCCGATCTTCATATGCGGTGTGAAATACCGCACAGATGCGTAAGGA**

**GAAAATACCGCATCAGGCGCTCTTCCGCTTCCTCGCTCACTGACTCGCTGCGCTCGGTCGTTCGGCTGCG**

**GCGAGCGGTATCAGCTCACTCAAAGGCGGTAATACGGTTATCCACAGAATCAGGGGATAACGCAGGAAAG**

**AACATGTGAGCAAAAGGCCAGCAAAAGGCCAGGAACCGTAAAAAGGCCGCGTTGCTGGCGTTTTTCCATA**

**GGCTCCGCCCCCCTGACGAGCATCACAAAAATCGACGCTCAAGTCAGAGGTGGCGAAACCCGACAGGACT**

**ATAAAGATACCAGGCGTTTCCCCCTGGAAGCTCCCTCGTGCGCTCTCCTGTTCCGACCCTGCCGCTTACC**

**GGATACCTGTCCGCCTTTCTCCCTTCGGGAAGCGTGGCGCTTTCTCAATGCTCACGCTGTAGGTATCTCA**

**GTTCGGTGTAGGTCGTTCGCTCCAAGCTGGGCTGTGTGCACGAACCCCCCGTTCAGCCCGACCGCTGCGC**

**CTTATCCGGTAACTATCGTCTTGAGTCCAACCCGGTAAGACACGACTTATCGCCACTGGCAGCAGCCACT**

**GGTAACAGGATTAGCAGAGCGAGGTATGTAGGCGGTGCTACAGAGTTCTTGAAGTGGTGGCCTAACTACG**

**GCTACACTAGAAGGACAGTATTTGGTATCTGCGCTCTGCTGAAGCCAGTTACCTTCGGAAAAAGAGTTGG**

**TAGCTCTTGATCCGGCAAACAAACCACCGCTGGTAGCGGTGGTTTTTTTGTTTGCAAGCAGCAGATTACG**

**CGCAGAAAAAAAGGATATCAAGAAGATCCTTTGATCTTTTCTACGGGGTCTGACGCTCAGTGGAACGAAA**

**ACTCACGTTAAGGGATTTTGGTCATGAGATTATCAAAAAGGATCTTCACCTAGATCCTTTTAAATTAAAA**

**ATGAAGTTTTAAATCAATCTAAAGTATATATGAGTAAACTTGGTCTGACAGTTACCAATGCTTAATCAGT**

**GAGGCACCTATCTCAGCGATCTGTCTATTTCGTTCATCCATAGTTGCCTGACTCCCCGTCGTGTAGATAA**

**CTACGATACGGGAGGGCTTACCATCTGGCCCCAGTGCTGCAATGATACCGCGAGACCCACGCTCACCGGC**

**TCCAGATTTATCAGCAATAAACCAGCCAGCCGGAAGGGCCGAGCGCAGAAGTGGTCCTGCAACTTTATCC**

**GCCTCCATCCAGTCTATTAAACAAGTGGCAGCAACGGATTCGCAAACCTGTCACGCCTTTTGTGCCAAAA**

**GCCGCGCCAGGTTTGCGATCCGCTGTGCCAGGCGTTAGGCGTCATATGAAGATTTCGGTGATCCCTGAGC**

**AGGTGGCGGAAACATTGGATGCTGAGAACCATTTCATTGTTCGTGAAGTGTTCGATGTGCACCTATCCGA**

**CCAAGGCTTTGAACTATCTACCAGAAGTGTGAGCCCCTACCGGAAGGATTACATCTCGGATGATGACTCT**

**GATGAAGACTCTGCTTGCTATGGCGCATTCATCGACCAAGAGCTTGTCGGGAAGATTGAACTCAACTCAA**

**CATGGAACGATCTAGCCTCTATCGAACACATTGTTGTGTCGCACACGCACCGAGGCAAAGGAGTCGCGCA**

**CAGTCTCATCGAATTTGCGAAAAAGTGGGCACTAAGCAGACAGCTCCTTGGCATACGATTAGAGACACAA**

**ACGAACAATGTACCTGCCTGCAATTTGTACGCAAAATGTGGCTTTACTCTCGGCGGCATTGACCTGTTCA**

**CGTATAAAACTAGACCTCAAGTCTCGAACGAAACAGCGATGTACTGGTACTGGTTCTCGGGAGCACAGGA**

**TGACGCCTAACAATTCATTCAAGCCGACACCGCTTCGCGGCGCGGCTTAATTCAGGAGTTAAACATCATG**

**AGGGAAGCGGTGATCGCCGAAGTATCGACTCAACTATCAGAGGTAGTTGGCGTCATCGAGCGCCATCTCG**

**AACCGACGTTGCTGGCCGTACATTTGTACGGCTCCGCAGTGGATGGCGGCCTGAAGCCACACAGTGATAT**

**TGATTTGCTGGTTACGGTGACCGTAAGGCTTGATGAAACAACGCGGCGAGCTTTGATCAACGACCTTTTG**

**GAAACTTCGGCTTCCCCTGGAGAGAGCGAGATTCTCCGCGCTGTAGAAGTCACCATTGTTGTGCACGACG**

**ACATCATTCCGTGGCGTTATCCAGCTAAGCGCGAACTGCAATTTGGAGAATGGCAGCGCAATGACATTCT**

**TGCAGGTATCTTCGAGCCAGCCACGATCGACATTGATCTGGCTATCTTGCTGACAAAAGCAAGAGAACAT**

**AGCGTTGCCTTGGTAGGTCCAGCGGCGGAGGAACTCTTTGATCCGGTTCCTGAACAGGATCTATTTGAGG**

**CGCTAAATGAAACCTTAACGCTATGGAACTCGCCGCCCGACTGGGCTGGCGATGAGCGAAATGTAGTGCT**

**TACGTTGTCCCGCATTTGGTACAGCGCAGTAACCGGCAAAATCGCGCCGAAGGATGTCGCTGCCGACTGG**

**GCAATGGAGCGCCTGCCGGCCCAGTATCAGCCCGTCATACTTGAAGCTAGGCAGGCTTATCTTGGACAAG**

**AAGATCGCTTGGCCTCGCGCGCAGATCAGTTGGAAGAATTTGTTCACTACGTGAAAGGCGAGATCACCAA**

**GGTAGTCGGCAAATAATGTCTAACAATTCGTTCAAGCCGACGCCGCTTCGCGGCGCGGCTTAACTCAAGC**

**GTTAGAGAGCTGGGGAAGACTATGCGCGATCTGTTGAAGGTGGTTCTAAGCCTCGTACTTGCGATGGCAT**

**CGGGGCAGGCACTTGCTGACCTGCCAATTGTTTTAGTGGATGAAGCTCGTCTTCCCTATGACTACTCCCC**

**ATCCAACTACGACATTTCTCCAAGCAACTACGACAACTCCATAAGCAATTACGACAATAGTCCATCAAAT**

**TACGACAACTCTGAGAGCAACTACGATAATAGTTCATCCAATTACGACAATAGTCGCAACGGAAATCGTA**

**GGCTTATATATAGCGCAAATGGGTCTCGCACTTTCGCCGGCTACTACGTCATTGCCAACAATGGGACAAC**

**GAACTTCTTTTCCACATCTGGCAAAAGGATGTTCTACACCCCAAAAGGGGGGCGCGGCGTCTATGGCGGC**

**AAAGATGGGAGCTTCTGCGGGGCATTGGTCGTCATAAATGGCCAATTTTCGCTTGCCCTGACAGATAACG**

**GCCTGAAGATCATGTATCTAAGCAACTAGCCTGCTCTCTAATAAAATGTTAGGAGCTTGGCTGCCATTTT**

**TGGGGTGAGGCCGTTCGCGGCCGAGGGGCGCAGCCCCTGGGGGGATGGGAGGCCCGCGTTAGCGGGCCGG**

**GAGGGTTCGAGAAGGGGGGGCACCCCCCTTCGGCGTGCGCGGTCACGCGCCAGGGCGCAGCCCTGGTTAA**

**AAACAAGGTTTATAAATATTGGTTTAAAAGCAGGTTAAAAGACAGGTTAGCGGTGGCCGAAAAACGGGCG**

**GAAACCCTTGCAAATGCTGGATTTTCTGCCTGTGGACAGCCCCTCAAATGTCAATAGGTGCGCCCCTCAT**

**CTGTCAGCACTCTGCCCCTCAAGTGTCAAGGATCGCGCCCCTCATCTGTCAGTAGTCGCGCCCCTCAAGT**

**GTCAATACCGCAGGGCACTTATCCCCAGGCTTGTCCACATCATCTGTGGGAAACTCGCGTAAAATCAGGC**

**GTTTTCGCCGATTTGCGAGGCTGGCCAGCTCCACGTCGCCGGCCGAAATCGAGCCTGCCCCTCATCTGTC**

**AACGCCGCGCCGGGTGAGTCGGCCCCTCAAGTGTCAACGTCCGCCCCTCATCTGTCAGTGAGGGCCAAGT**

**TTTCCGCGAGGTATCCACAACGCCGGCGGCCGGCCGCGGTGTCTCGCACACGGCTTCGACGGCGTTTCTG**

**GCGCGTTTGCAGGGCCATAGACGGCCGCCAGCCCAGCGGCGAGGGCAACCAGCCCGGTGAGCGTCGGAAA**

**GGGTCGACATCTTGCTGCGTTCGGATATTTTCGTGGAGTTCCCGCCACAGACCCGGATTGAAGGCGAGAT**

**CCAGCAACTCGCGCCAGATCATCCTGTGACGGAACTTTGGCGCGTGATGACTGGCCAGGACGTCGGCCGA**

**AAGAGCGACAAGCAGATCACGATTTTCGACAGCGTCGGATTTGCGATCGAGGATTTTTCGGCGCTGCGCT**

**ACGTCCGCGACCGCGTTGAGGGATCAAGCCACAGCAGCCCACTCGACCTTCTAGCCGACCCAGACGAGCC**

**AAGGGATCTTTTTGGAATGCTGCTCCGTCGTCAGGCTTTCCGACGTTTGGGTGGTTGAACAGAAGTCATT**

**ATCGTACGGAATGCCAGCACTCCCGAGGGGAACCCTGTGGTTGGCATGCACATACAAATGGACGAACGGA**

**TAAACCTTTTCACGCCCTTTTAAATATCCGTTATTCTAATAAACGCTCTTTTCTCTTAGGTTTACCCGCC**

**AATATATCCTGTCAAACACTGATAGTTTAAACTGAAGGCGGGAAACGACAATCTGATCATGAGCGGAGAA**

**TTAAGGGAGTCACGTTATGACCCCCGCCGATGACGCGGGACAAGCCGTTTTACGTTTGGAACTGACAGAA**

**CCGCAACGATTGAAGGAGCCACTCAGCCCCAATACGCAAACCGCCTCTCCCCGCGCGTTGGCCGATTCAT**

**TAATGCAGCTGGCACGACAGGTTTCCCGACTGGAAAGCGGGCAGTGAGCGCAACGCAATTAATGTGAGTT**

**AGCTCACTCATTAGGCACCCCAGGCTTTACACTTTATGCTTCCGGCTCGTATGTTGTGTGGAATTGTGAG**

**CGGATAACAATTTCACACAGGAAACAGCTATGACCATGATTACGCCAAGCTATTTAGGTGACACTATAGA**

**ATACTCAAGCTATGCATCCAACGCGTTGGGAGCTCTCCCATATCGACCTGCAGGCGGCCGCTCGACGAAT**

**TAATTCCAATCCCACAAAAATCTGAGCTTAACAGCACAGTTGCTCCTCTCAGAGCAGAATCGGGTATTCA**

**ACACCCTCATATCAACTACTACGTTGTGTATAACGGTCCACATGCCGGTATATACGATGACTGGGGTTGT**

**ACAAAGGCGGCAACAAACGGCGTTCCCGGAGTTGCACACAAGAAATTTGCCACTATTACAGAGGCAAGAG**

**CAGCAGCTGACGCGTACACAACAAGTCAGCAAACAGACAGGTTGAACTTCATCCCCAAAGGAGAAGCTCA**

**ACTCAAGCCCAAGAGCTTTGCTAAGGCCCTAACAAGCCCACCAAAGCAAAAAGCCCACTGGCTCACGCTA**

**GGAACCAAAAGGCCCAGCAGTGATCCAGCCCCAAAAGAGATCTCCTTTGCCCCGGAGATTACAATGGACG**

**ATTTCCTCTATCTTTACGATCTAGGAAGGAAGTTCGAAGGTGAAGGTGACGACACTATGTTCACCACTGA**

**TAATGAGAAGGTTAGCCTCTTCAATTTCAGAAAGAATGCTGACCCACAGATGGTTAGAGAGGCCTACGCA**

**GCAGGTCTCATCAAGACGATCTACCCGAGTAACAATCTCCAGGAGATCAAATACCTTCCCAAGAAGGTTA**

**AAGATGCAGTCAAAAGATTCAGGACTAATTGCATCAAGAACACAGAGAAAGACATATTTCTCAAGATCAG**

**AAGTACTATTCCAGTATGGACGATTCAAGGCTTGCTTCATAAACCAAGGCAAGTAATAGAGATTGGAGTC**

**TCTAAAAAGGTAGTTCCTACTGAATCTAAGGCCATGCATGGAGTCTAAGATTCAAATCGAGGATCTAACA**

**GAACTCGCCGTGAAGACTGGCGAACAGTTCATACAGAGTCTTTTACGACTCAATGACAAGAAGAAAATCT**

**TCGTCAACATGGTGGAGCACGACACTCTGGTCTACTCCAAAAATGTCAAAGATACAGTCTCAGAAGACCA**

**AAGGGCTATTGAGACTTTTCAACAAAGGATAATTTCGGGAAACCTCCTCGGATTCCATTGCCCAGCTATC**

**TGTCACTTCATCGAAAGGACAGTAGAAAAGGAAGGTGGCTCCTACAAATGCCATCATTGCGATAAAGGAA**

**AGGCTATCATTCAAGATCTCTCTGCCGACAGTGGTCCCAAAGATGGACCCCCACCCACGAGGAGCATCGT**

**GGAAAAAGAAGACGTTCCAACCACGTCTTCAAAGCAAGTGGATTGATGTGACATCTCCACTGACGTAAGG**

**GATGACGCACAATCCCACTATCCTTCGCAAGACCCTTCCTCTATATAAGGAAGTTCATTTCATTTGGAGA**

**GGACACGCTCGACTATTTTTACAACAATTACCATGAAGACTAATCTTTTTCTCTTTCTCATCTTTTCACT**

**TCTCCTATCATTATCCTCGGCCGAATTCCTCGAGGCAAAGATACTCGGAAAACATTTATCTAGAAGTAAA**

**GGAGAAGAACTTTTCACTGGAGTTGTCCCAATTCTTGTTGAATTAGATGGTGATGTTAATGGGTACAAAT**

**TTTCTGTCAGTGGAGAGGGTGAAGGTGATGCAACATACGGAAAACTTACCCTTAAATTTATTTGCACTAC**

**TGGAAAACTACCTGTTCCATGGCCAACACTTGTCACTACTTTCTCTTATGGTGTTCAATGCTTTTCAAGA**

**TACCCAGATCATATGAAGCGGCACGACTTCTTCAAGAGCGCCATGCCTGAGGGATACGTGCAGGAGAGGA**

**CCATCTTCTTCAAGGACGACGGGAACTACAAGACACGTGCTGAAGTCAAGTTTGAGGGAGACACCCTCGT**

**CAACAGGATCGAGCTTAAGGGAATCGATTTCAAGGAGGACGGAAACATCCTCGGCCACAAGTTGGAATAC**

**AACTACAACTCCCACAACGTATACATCATGGCCGACAAGCAAAAGAACGGCATCAAAGCCAACTTCAAGA**

**CCCGCCACAACATCGAAGACGGCGGCGTGCAACTCGCTGATCATTATCAACAAAATACTCCAATTGGCGA**

**TGGCCCTGTCCTTTTACCAGACAACCATTACCTGTCCACACAATCTGCCCTTTCGAAAGATCCCAACGAA**

**AAGAGAGACCACATGGTCCTTCTTGAGTTTGTAACAGCTGCTGGGATTACACATGGCATGGATGAACTAT**

**ACAAACATGATGAGCTTTGAACTAGAGTCCTGCTTTAATGAGATATGCGAGACGCCTATGATCGCATGAT**

**ATTTGCTTTCAATTCTGTTGTGCACGTTGTAAAAAACCTGAGCATGTGTAGCTCAGATCCTTACCGCCGG**

**TTTCGGTTCATTCTAATGAATATATCACCCGTTACTATCGTATTTTTATGAATAATATTCTCCGTTCAAT**

**TTACTGATTGTACCCTACTACTTATATGTACAATATTAAAATGAAAACAATATATTGTGCTGAATAGGTT**

**TATAGCGACATCTATGATAGAGCGCCACAATAACAAACAATTGCGTTTTATTATTACAAATCCAATTTTA**

**AAAAAAGCGGCAGAACCGGTCAAACCTAAAAGACTGATTACATAAATCTTATTCAAATTTCAAAAGGCCC**

**CAGGGGCTAGTATCTACGACACACCGAGCGGCGAACTAATAACGTTCACTGAAGGGAACTCCGGTTCCCC**

**GCCGGCGCGCATGGGTGAGATTCCTTGAAGTTGAGTATTGGCCGTCCGCTCTACCGAAAGTTACGGGCAC**

**CATTCAACCCGGTCCAGCACGGCGGCCGGGTAACCGACTTGCTGCCCCGAGAATTATGCAGCATTTTTTT**

**GGTGTATGTGGGCCCCAAATGAAGTGCAGGTCAAACCTTGACAGTGACGACAAATCGTTGGGCGGGTCCA**

**GGGCGAATTTTGCGACAACATGTCGAGGCTCAGCAGGACCTGCAGGCATGCAAGCTAGCTTACTAGTGAT**

**GCATATTCTATAGTGTCACCTAAATCTGC**

sly-miR6020::N-CFP^T1T2^ TAS match

3'-TTTCTATGAGCCTTTTGTAAAT-5'

o|||||||||| |||||o||||

48 AGTTTTAGAGGCGAAGATACTCGA*AAAACGTTTACAAGTCACTTA* **TACATGGTGAGCAAGGGCGAG**

TCAAAATCTCCGCTTCTATGAG CTTTTTGCAAATGTTCAGTGA ATATGTACCACTCGTTCCCGC

114 GAGCTGTTCACCGGGGTGGTG CCCATCCTGGTCGAGCTGGAC GGCGACGTAAACGGCCACAAG

TCCTCGACAAGTGGCCCCACC ACGGGTAGGACCAGCTCGACC **TGCCGCTGCATTTGCCGGTGT**

177 **TTCAGCGTGTCCGGCGAGGGC** GAGGGCGATGCCACCTACGGC AAGCTGACCCTGAAGTTCATC

TCAAGTCGCACAGGCCGCTCC CGCTCCCGCTACGGTGGATGC **CGTTCGACTGGGACTTCAAGT**

240 TGCACCACCGGCAAGCTGCCC GTGCCCTGGCCCACCCTCGTG ACCACCTTCGGCTACGGCCTG

AGACGTGGTGGCCGTTCGACG GGCACGGGACCGGGTGGGAGC ACTGGTGGAAGCCGATGCCGG

303 ATGTGCTTCGCCCGCTACCCC GACCACATGAAGCAGCACGAC TTCTTCAAGTCCGCCATGCCC

ACTACACGAAGCGGGCGATGG GGCTGGTGTACTTCGTCGTGC TGAAGAAGTTCAGGCGGTACG

366 GAAGGCTACGTCCAGGAGCGC ACCATCTTCTTCAAGGACGAC GGCAACTACAAGACCCGCGCC

GGCTTCCGATGCAGGTCCTCG CGTGGTAGAAGAAGTTCCTGC TGCCGTTGATGTTCTGGGCGC

429 GAGGTGAAGTTCGAGGGCGAC

GGCTCCACTTCAAGCTCCCGC TG

sly-miR6020::MS4miR6020 TAS match

3'-TTTCTATGAGCCTTTTGTAAAT-5'

o|||||||||| |||||o||||

48 TTATCCTCGGCCGAATTCCTCGAGGCGAAGATACTCG*AAAAACGTTTATCTAGAAGTAA* AGGAGAAGAACTTTTCACTGG

AATAGGAGCCGGCTTAAGGAGCTCCGTTTCTATGAG CCTTTTGTAAATAGATCTTCA TTTCCTCTTCTTGAAAAGTGA

128 AGTTGTCCCAATTCTTGTTGA ATTAGATGGTGATGTTAATGG GTACAAATTTTCTGTCAGTGG

CCTCAACAGGGTTAAGAACAA CTTAATCTACCACTACAATTA CCCATGTTTAAAAGACAGTCA

191 AGAGGGTGAAGGTGATGCAAC ATACGGAAAACTTACCCTTAA ATTTATTTGCACTACTGGAAA

**CCTCTCCCACTTCCACTACGT** TGTATGCCTTTTGAATGGGAA **TTTAAATAAACGTGATGACCT**

254 ACTACCTGTTCCATGGCCAAC TGGTGTTCAATGCTTTTCAAG ATACCCAGATCATATGAAGCG

**TTTGATGGACAAGGTACCGGT** **ATACCACAAGTTACGAAAAGT** TCTATGGGTCTAGTATACTTC

317 TGTGAACAGTGATGAAAGAGA GCACGACTTCTTCAAGAGCGC GCCGTGCTGAAGAAGTTCTCG

ACTTGTCACTACTTTCTCTTA CATGCCTGAGGGATACGTGCA CGGTACGGACTCCCTATGCAC

380 GGAGAGGACCATCTTCTTCAA GGACGACGGGAACTACAAGAC ACGTGCTGAAGTCAAGTTTGA GGGAG

GTCCTCTCCTGGTAGAAGAAG TTCCTGCTGCCCTTGATGTTC TGTGCACGACTTCAGTTCAAA CTCCCTC

sly-miR6020::MS4miR6020^E^ TAS mutant to perfect match

3'-TTTCTATGAGCCTTTTGTAAAT-5'

||||||||||||||||||||||

48  TTATCCTCGGCCGAATTCCTCGAGGCAAAGATACTCGG*AAAACATTTATCTAGAAGTAA* AGGAGAAGAACTTTTCACTGG

AATAGGAGCCGGCTTAAGGAGCTCCGTTTCTATGAG CCTTTTGTAAATAGATCTTCA TTTCCTCTTCTTGAAAAGTGA

128 AGTTGTCCCAATTCTTGTTGA ATTAGATGGTGATGTTAATGG GTACAAATTTTCTGTCAGTGG

CCTCAACAGGGTTAAGAACAA CTTAATCTACCACTACAATTA CCCATGTTTAAAAGACAGTCA

191 AGAGGGTGAAGGTGATGCAAC ATACGGAAAACTTACCCTTAA ATTTATTTGCACTACTGGAAA

**CCTCTCCCACTTCCACTACGT**  TGTATGCCTTTTGAATGGGAA **TTTAAATAAACGTGATGACCT**

254 ACTACCTGTTCCATGGCCAAC ACTTGTCACTACTTTCTCTTA TGGTGTTCAATGCTTTTCAAG

**TTTGATGGACAAGGTACCGGT** TGTGAACAGTGATGAAAGAGA **ATACCACAAGTTACGAAAAGT**

317 ATACCCAGATCATATGAAGCG GCACGACTTCTTCAAGAGCGC CATGCCTGAGGGATACGTGCA

TCTATGGGTCTAGTATACTTC GCCGTGCTGAAGAAGTTCTCG CGGTACGGACTCCCTATGCAC

380 GGAGAGGACCATCTTCTTCAA GGACGACGGGAACTACAAGAC ACGTGCTGAAGTCAAGTTTGA GGGAG

GTCCTCTCCTGGTAGAAGAAG TTCCTGCTGCCCTTGATGTTC TGTGCACGACTTCAGTTCAAA CTCCCTC
